# Supplementary figures and images for: Selective role of the DNA helicase Mcm5 in BMP retrograde signaling during Drosophila neuronal differentiation
Source: PLoS Genet. 2022 Jun 23;18(6):e1010255. doi: 10.1371/journal.pgen.1010255 (PMC9258838; doi:10.1371/journal.pgen.1010255)

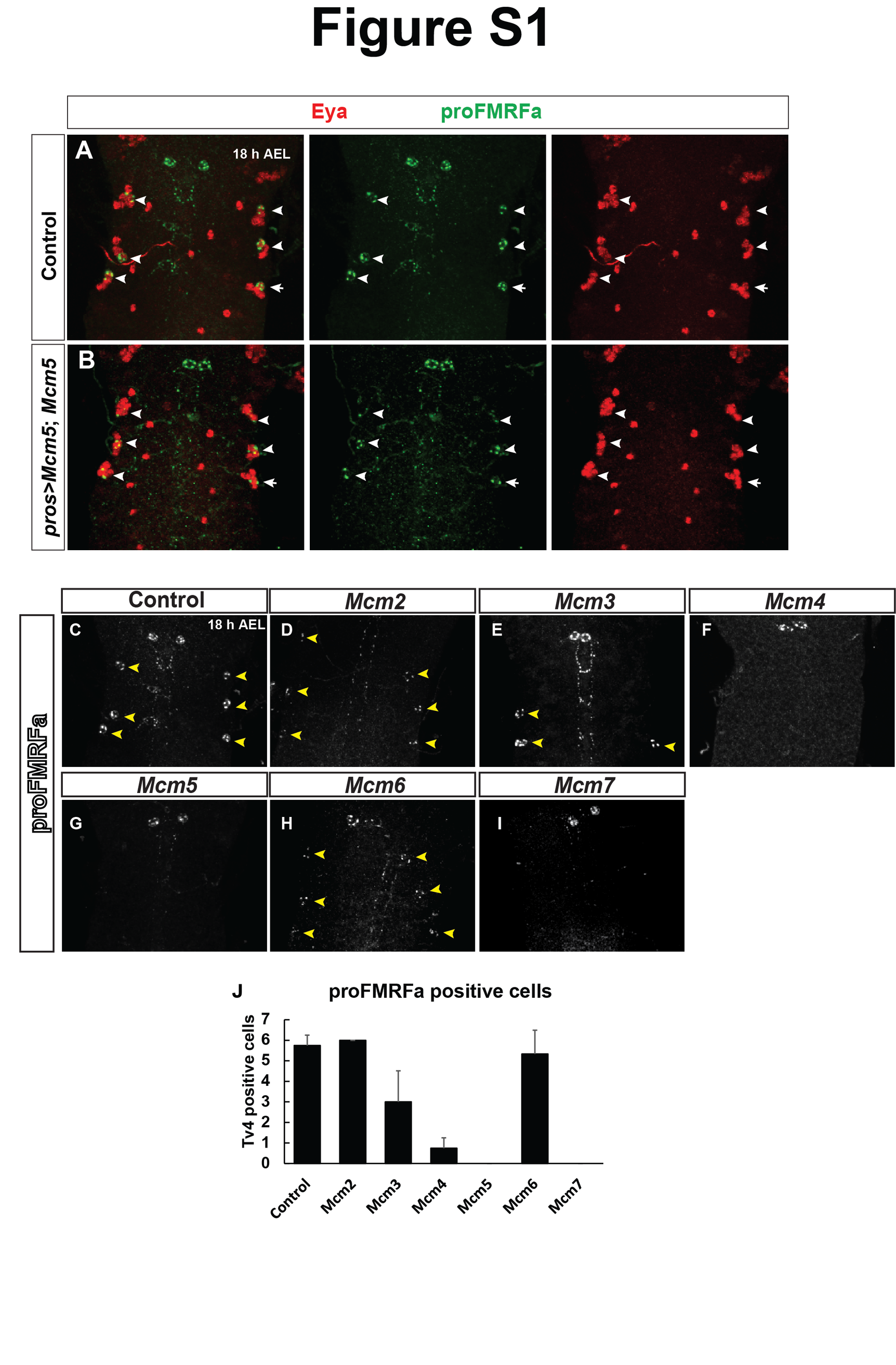

Supplement: S1 Fig — FMRFa expression analysis in Mcm2-7 Complex gene related mutants. Immunostaining for proFMRFa and Eya in Control (A) and Mcm5, prospero>UAS-Mcm5 (B) at stage 18h AEL. Immunostaining for proFMRFa in Control, and mutants for the Mcm2-7 complex components Mcm2 (D), Mcm3 (E), Mcm4 (F), Mcm5 (G), Mcm6 (H), and Mcm7 (I) and the respective quantification of Tv4 FMRFa positive cells (J) Merge and individual antibody images are shown in each panel. Genotypes: (A, C) w1118. (B) prospero-Gal4/ UAS-Mcm5; Mcm5exc222, (D) Mcm2MI0634 (E) Mcm3smu (F) dpa1 (G) Mcm5exc222 (H) Mcm7 f03462. (TIF) [file pgen.1010255.s001.tif]

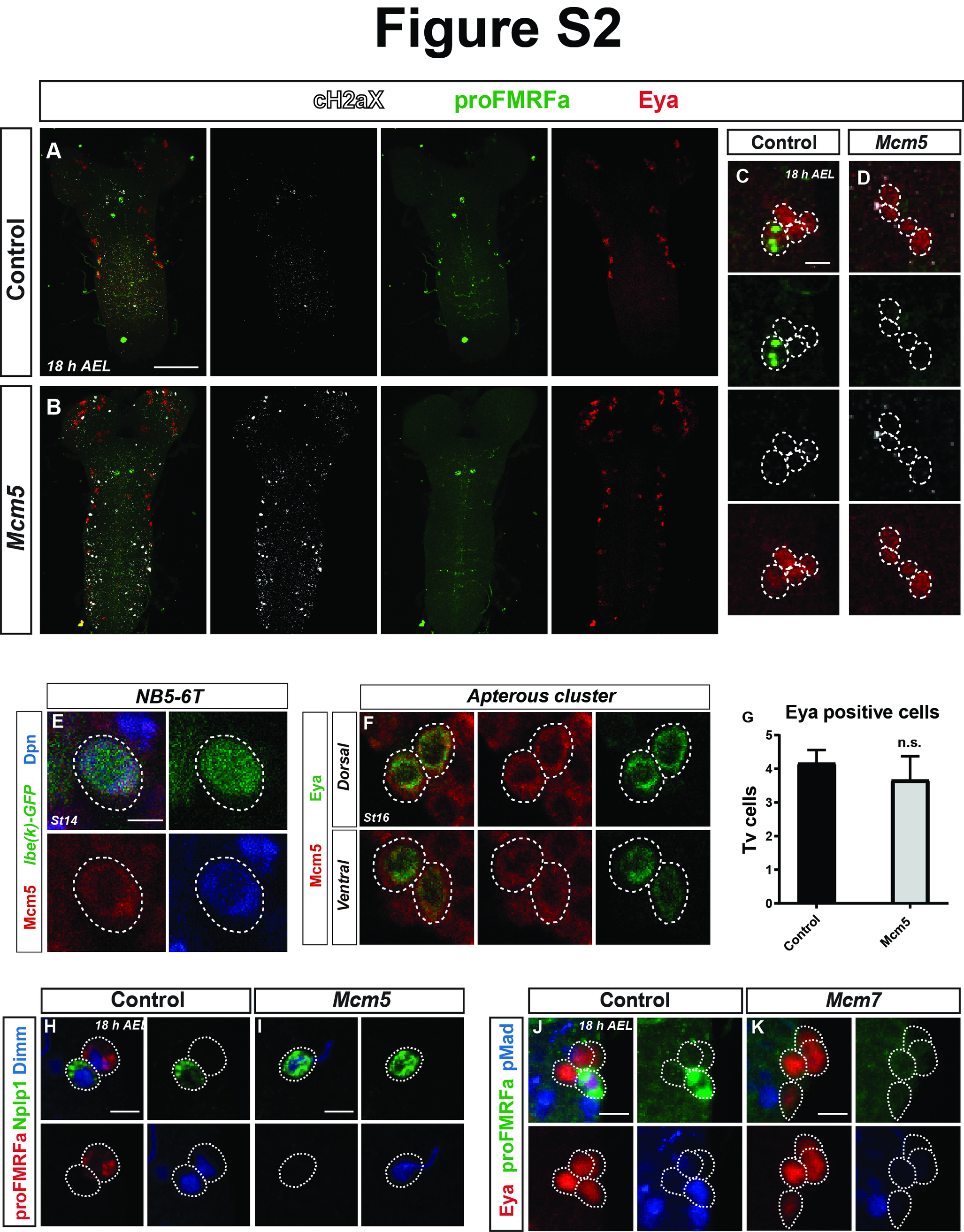

Supplement: S2 Fig — Mcm5 expression in NB5-6 Lineage. Nplp1 expression and quantification of Eya expressing cells in control and Mcm5 mutants. Dimm and pMad expression in Mcm7 mutants. Immunostaining for cH2aX, Eya and proFMRFa in Control (A) and Mcm5 mutants (B) at stage 18h AEL (scale bar 50 μm). Detail of immunostaining for cH2aX, Eya and proFMRFa in the apterous cluster cells of Control (C) and Mcm5 (D) mutants (scale bar 5 μm). (E) Expression of Mcm5 protein detected by immunostaining in the NB 5–6, identified by the reporter line Lbe(k)-GFP, and Deadpan immunostaining (Dpn). (F) Expression of Mcm5 protein detected by immunostaining in the Ap cluster cells, identified by Eya. (G) Quantification of Eya expressing cells within thoracic segments T2 and T3 in control and Mcm5 mutants (U-Mann-Whitney test; n≥3 CNS per genotype, n≥ 9 hemisegments per genotype, n.s = non-significant). (H-I) Immunostaining for proFMRFa, Nplp1 and Dimm in Control (H) and Mcm5 mutant (I), at stage 18h AEL (scale bar 5 μm). (J-K) Immunostaining for proFMRFa, Eya and pMad in Control (J) and Mcm7 mutant (K), at stage 18h AEL (scale bar 5 μm). Merge and individual antibody images are shown in each panel. Genotypes: (A, C, E, F, H, J) w1118. (B, D, I) Mcm5exc222/Mcm5exc222 (K) Mcm7 f03462/Mcm7 f03462. Mcm3smu (F) dpa1 (G) Mcm5exc222 (H) Mcm7 f03462. (TIF) [file pgen.1010255.s002.tif]

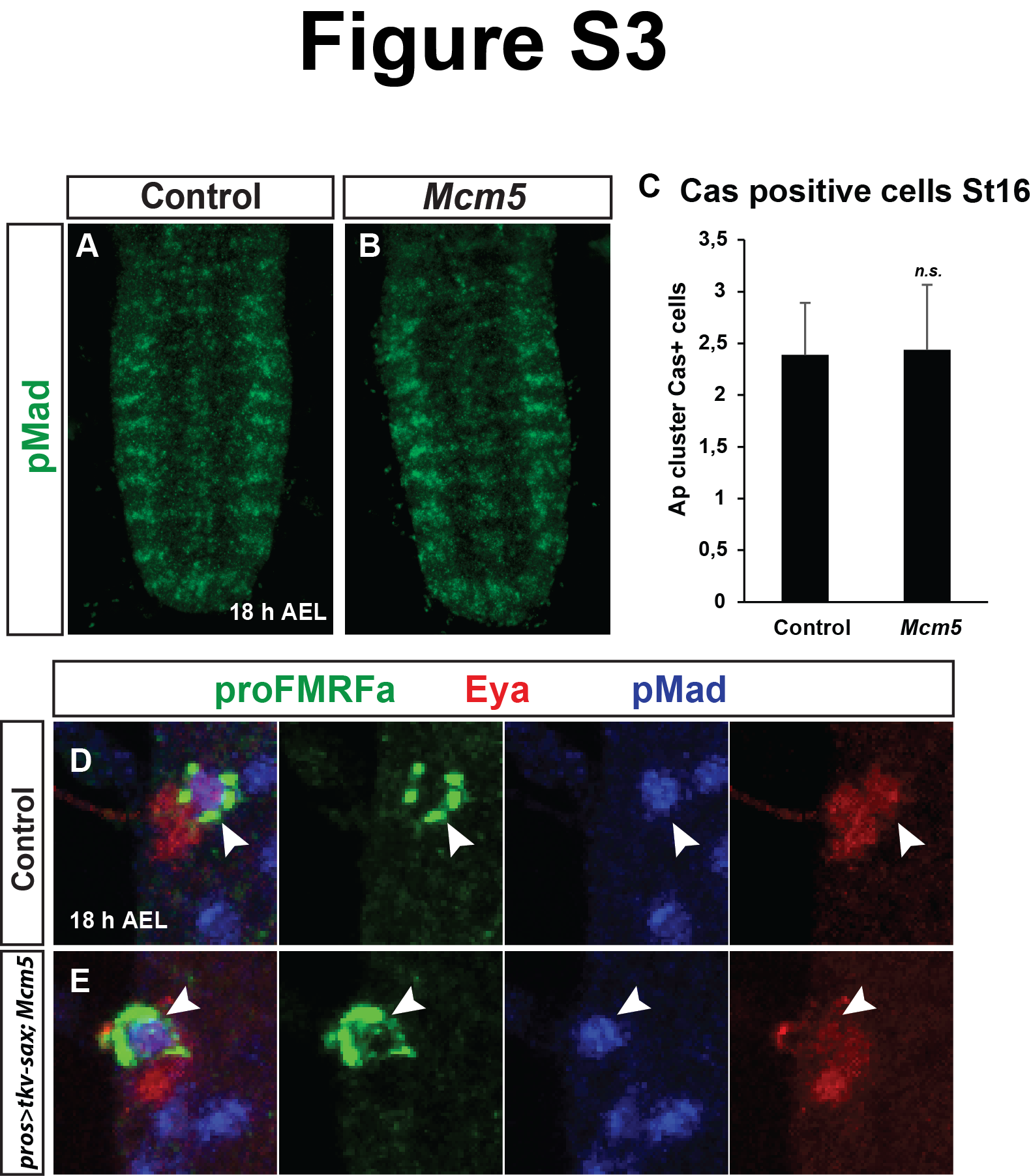

Supplement: S3 Fig — Immunostaining for p-Mad in Control (A) and Mcm5 mutants (B) at stage 18h AEL. (C) Quantification of Cas positive Ap cluster cells in control and mcm5 mutant (U-Mann-Whitney test; n≥3 CNS per genotype, n≥ 18 hemisegments per genotype, n.s = non-significant). Immunostaining for proFMRFa, Eya and pMad in Control (D) and expression of wild type forms of the type I BMP receptors (sax and tkv) in Mcm5 mutants (E), at stage 18h AEL. Genotypes: (A,D) w1118 (B) Mcm5exc222/Mcm5exc222, (E) pros-Gal4/UAS-sax, UAS-tkv; Mcm5exc222. (TIF) [file pgen.1010255.s003.tif]
