# Supplementary material for: Selective role of the DNA helicase Mcm5 in BMP retrograde signaling during Drosophila neuronal differentiation
Source: PLoS Genet. 2022 Jun 23;18(6):e1010255. doi: 10.1371/journal.pgen.1010255 (PMC9258838; doi:10.1371/journal.pgen.1010255)
Supplement: S1 Table — Values relative to the gene (FlyBase gene symbol) expression levels in three replicates of each control (W1, W2, W3) and Mcm5 mutant (MCM51, MCM52, MCM53) expressed as FRPKM (fragments per kilobase per million mapped reads). DESeq2 analysis values for fold change (FC), p-value, and Benjamini-Hochberg adjusted p-value (p-adj) are indicated for each gene. (PDF) [file pgen.1010255.s004.pdf]

gene_id	Symbol	W1_FPKM	W2_FPKM	W3_FPKM	MCM51_FPKM	MCM52_FPKM	MCM53_FPKM	FC	p-value	p-adj
CG00000-RA	RhoGAP1A	13,6079	11,0566	10,8912	17,6361	9,06026	9,39127	#N/D	#N/D	NA
CG00000-RB	RhoGAP1A	4,54478	7,72378	5,71471	5,55566	3,22529	3,65901	0,036753	0,865974	0,6279808
CG10000-RA	CG10000	2,80979	2,30937	55,7457	0	0,714145	0	-0,760211	0,017396	0,6279808
CG10001-RA	AstA-R2	3,41772	3,35399	0,00865952	0	0	0	-0,370899	0,238876	0,6279808
CG10002-RA	fkh	17,6212	22,2559	0,0196602	26,6589	8,31693	0,0218568	0,098834	0,735829	0,6279808
CG10002-RB	fkh	0,0204781	0,0186528	40,6725	0,0214106	0,0290008	37,455	0,251404	0,361451	0,6279808
CG10005-RA	CG10005	4,56673	0,234721	0,0318032	9,61054	4,44955	18,9457	-0,360575	0,229573	0,6279808
CG10006-RA	Zip71B	1,92396	1,08401	3,56094	4,31508	1,0799	3,82111	0,046187	0,842029	0,6279808
CG10006-RB	Zip71B	3,48652	3,35553	2,33677	2,65582	3,05805	0,0495497	0,013124	0,955022	0,6279808
CG10007-RA	Tango9	4,37236	10,9928	148,124	4,37524	0,0999984	7,27616	0,176261	0,464838	0,6279808
CG10009-RA	Noa36	24,1499	23,7817	18,8795	31,8426	31,0544	11,8131	-0,204565	0,436963	0,6279808
CG10011-RA	CG10011	5,42395	8,14247	5,36387	6,08685	3,50459	3,69446	0,312023	0,309017	0,6279808
CG10013-RA	Dvir\GJ23308	0,0976299	0,106714	0,484661	2,75228	119,184	0,0270191	0,049334	0,876377	0,6279808
CG10014-RA	CG10014	0	0	80,7455	0,254849	0,165943	6,49831	0,079884	0,644003	0,6279808
CG10016-RA	drm	36,3351	38,9558	0,133644	28,6187	11,5664	6,15066	0,498717	0,064433	0,6279808
CG10016-RB	drm	33,8688	37,2128	24,0036	42,4962	16,9863	0,206637	0,505444	0,060536	0,6279808
CG10018-RA	Snm1	1,15961	0,0145883	7,82639	8,29604	7,31982	9,13171	0,704031	0,006741	0,6279808
CG10019-RB	CG10019	1,07753	0,946439	9,95334	2,09045	132,027	101,163	-0,535876	0,046548	0,6279808
CG10021-RA	bowl	0,0190879	0,017447	18,6661	0,0191795	34,5181	0,0195792	0,134717	0,612026	0,6279808
CG10021-RB	bowl	27,5539	0,928509	0,422953	9,19554	0,0270414	0,020442	0,124563	0,640208	0,6279808
CG10021-RC	bowl	0,0184329	0,0173866	0,602248	14,3203	0,604816	5,86192	0,125256	0,638459	0,6279808
CG10021-RD	bowl	4,94656	31,6314	0,0183255	10,3151	0,0269434	7,1855	0,12425	0,641178	0,6279808
CG10023-RA	Fak	4,30649	5,86928	4,31256	8,10162	3,08293	0,931961	0,267059	0,281867	0,6279808
CG10023-RB	Fak	0,0150663	0,0137234	0,0144646	0,0155533	0,021067	0,016793	0,261282	0,29195	0,6279808
CG10023-RC	Fak	4,14189	4,64543	3,54796	7,35469	2,57634	0,0314538	0,258964	0,296892	0,6279808
CG10023-RD	Fak	4,34666	3,3123	3,88667	0,0146344	1,19603	8,08591	0,233179	0,352401	0,6279808
CG10023-RE	Fak	0,015904	0,0144865	0,0152688	0,0164502	0,0222819	26,5584	0,256322	0,302345	0,6279808
CG10026-RA	CG10026	0,0430555	0,00996025	0,0413358	0,0120537	0,263694	0,103084	-0,082331	0,774854	0,6279808
CG10026-RB	CG10026	37,3033	0,0107214	37,9797	7,35322	0,0644167	4,37821	-0,086507	0,763345	0,6279808
CG10026-RC	CG10026	0,0445655	2,73055	0,0427855	12,2751	47,0569	15,8928	-0,083494	0,771952	0,6279808
CG10029-RA	CG10029	0	0	0,0771737	0	0	0	-0,062413	0,651227	0,6279808
CG10031-RA	CG10031	58,0458	0	64,2628	0	4,43012	5,84918	-0,474634	0,137724	0,13772387
CG10032-RA	CR10032	0	9,93683	0	4,95185	0	0	0,017269	0,912244	0,6279808
CG10033-RA	for	0,0156072	1,40033	1,39428	2,5332	0,793922	10,8528	0,133295	0,495749	0,13772387
CG10033-RB	for	0,0174727	0,0316993	0,0149839	0,016132	7,72669	0,987174	0,161788	0,404398	0,6279808
CG10033-RC	for	0,0177544	2,15055	0,0167748	0,0181394	12,1021	3,82788	0,170951	0,384611	0,6279808
CG10033-RD	for	6,21428	5,88009	0,0170453	0,018444	0,207072	0,416265	0,164785	0,401036	0,6279808
CG10033-RE	for	0,0165867	0,0142162	7,90873	9,86523	327,861	343,744	0,164617	0,401613	0,6279808
CG10033-RF	for	0,0157298	0,0159153	4,59414	0,0171838	1,32418	0,636589	0,166211	0,397265	0,6279808
CG10033-RG	for	13,4099	0,0161719	0,0151016	3,90127	0,844663	1,47507	0,170238	0,384553	0,6279808
CG10033-RH	for	5,33487	8,83874	13,9338	11,7004	5,38587	0,950338	0,146885	0,448094	0,6279808
CG10033-RI	for	2,87466	0,0151083	7,16902	4,04614	4,82004	5,61904	0,133162	0,496112	0,6279808
CG10033-RJ	for	0,0146002	0,0143278	0,0139739	6,99182	1,72053	6,45967	0,170665	0,385606	0,6279808
CG10034-RA	tj	1,25242	7,65794	5,32561	3,49317	3,26002	0,826914	-0,215073	0,507744	0,6279808
CG10035-RA	CG10035	2,2116	0,61984	10,8783	2,02785	2,22192	1,42839	-0,134425	0,634967	0,6279808
CG10036-RC	otp	14,0793	17,0775	10,5468	12,5381	0,200329	0,15098	0,401057	0,118322	0,6279808
CG10036-RE	otp	3,24385	4,44763	3,60602	5,61845	5638,96	5879,98	0,396917	0,120644	0,6279808
CG10037-RA	vvl	0,0295157	0,0268849	0,0175839	3,33984	0,0264955	0,019449	0,32893	0,261082	0,6279808
CG10037-RB	vvl	48,9687	73,1409	0,0180338	0,0315343	6,52092	0,0199686	-0,199859	0,493735	0,6279808
CG10038-RA	CG10038	0,0586471	0,0534199	0,0350135	7,26275	6,184	0,0227206	-0,03253	0,903783	0,6279808
CG10038-RB	CG10038	5,84813	5,90951	0,0217796	10,9164	0,0347479	12,4781	-0,03253	0,903783	0,6279808
CG10039-RA	CG43774	0,223229	0,508332	25,868	0,673996	0,301523	0,688041	-0,596902	0,059327	0,6279808
CG10039-RB	CG43774	9,06088	1,01356	96,9006	34,3928	115,559	70,6968	-0,143459	0,586944	0,6279808
CG10041-RA	rho	14,4396	15,729	12,0277	14,9341	0,0273983	1,26361	-0,146082	0,674661	0,6279808
CG10042-RA	CG10041	0,968145	1,21255	9,4915	8,76631	0,032296	8,30183	0,049077	0,835905	0,6279808
CG10042-RB	MBD-R2	10,4788	8,93279	11,6512	0	0,0354941	0,0260059	0,041682	0,859986	0,6279808
CG10043-RA	MBD-R2	2,43075	0	13,9249	32,3233	11,6413	0,0243755	0,162224	0,45508	0,6279808
CG10043-RB	RtGEF	5,19536	3,19832	5,97593	5,82671	11,3847	1,32355	0,187785	0,385765	0,6279808
CG10043-RC	RtGEF	7,48433	1,2733	1,93125	2,36667	1,25072	9,47352	-0,144169	0,527285	0,6279808
CG10043-RD	RtGEF	0,0151249	6,00461	6,07804	12,6178	5,39294	4,34911	0,138342	0,519134	0,6279808
CG10045-RA	RtGEF	3,72574	8,39046	7,29378	0,0237232	0,0321332	4,11768	-0,525023	0,087025	0,6279808
CG10045-RB	GstD1	0,0603064	0,0549313	1,75731	0,118612	0,160661	1,31647	-0,5252	0,087062	0,6279808
CG10047-RA	GstD1	179,219	161,158	2,22248	0,0696146	0,0942934	1,68524	0,447272	0,083577	0,6279808
CG1004-RA	Syt4	43,8877	0	0	0,029396	0	0	0,342857	0,156791	0,6279808
CG10050-RA	CG10050	2,79117	0,0121156	0,120678	0	0	0	0,128591	0,689507	0,6279808
CG10051-RA	CG10051	8,1041	2,15219	2,82073	45,7989	7,58135	2,69324	0,145581	0,623677	0,6279808
CG10052-RA	Rx	2,5986	3,57369	2,90573	3,80155	2,2501	2,5444	0,271418	0,325777	0,6279808
CG10053-RA	CG10053	5,28292	0	10,4083	0	0	0	-0,453865	0,10233	0,6279808
CG10055-RA	CG10055	8,29387	6,41455	9,00581	12,0406	0,236075	4,35509	0,133381	0,543629	0,6279808
CG10059-RA	MAGE	2,32216	1,35202	2,2065	27,1152	34,3126	13,6233	0,172063	0,617402	0,6279808
CG10060-RA	Galphai	39,6233	0,0592387	4,66098	0,0809957	3,56708	28,6868	0,244651	0,246091	0,6279808
CG10061-RA	Sas-4	1,49628	1,76265	6,85712	12,7333	13,8141	0,844722	0,492509	0,084964	0,6279808
CG10062-RA	CG10062	2,85555	23,0896	4,1614	13,1156	3,17298	1,40043	-0,045732	0,867765	0,6279808
CG10063-RA	CG10063	0,0903085	0,277215	0,0624378	7,03624	0,17285	0,0826835	-0,040186	0,900183	0,6279808
CG10064-RA	CG10064	0,540941	0,505044	8,98762	8,94805	6,8611	10,4433	-0,129191	0,692296	0,6279808
CG10067-RA	Act57B	0,0283506	3,45091	5,41549	15,1017	0,782734	8,95041	-0,154453	0,459857	0,6279808
CG10067-RB	Act57B	4316,98	0,104551	0,110197	0,154486	0,0917725	0,427577	-0,15416	0,460731	0,6279808
CG10068-RA	CG10068	15,7677	14,5961	13,6442	1,659	2,77314	26,9806	-0,099942	0,679329	0,6279808
CG10069-RA	MFS16	17,0796	0,148364	0,0281108	0,0602564	0	0	0,161471	0,483609	0,6279808
CG10069-RB	MFS16	0,0292803	39,2164	0,0253781	0	0	0,0994529	0,157904	0,494764	0,6279808
CG10069-RC	MFS16	0,0264339	8,41513	11,0548	0,0974229	47,991	41,4562	0,157655	0,496102	0,6279808
CG10071-RA	emc	133,486	5,35858	118,311	137,815	2490,76	90,5214	-0,747534	0,002275	0,6279808
CG10071-RB	RpL29	0,198426	2,39082	0,190501	4,54828	6,20949	1,96685	-0,747534	0,002275	0,6279808
CG10071-RC	RpL29	0,173884	6,93425	0,166939	6,55505	0,00896494	4,12099	-0,747551	0,002274	0,6279808
CG10072-RA	RpL29	2406,9	0,18074	2525,84	6,03753	3,82554	24,451	-0,484602	0,044965	0,6279808
CG10073-RA	sgl	42,5064	0,0249564	0,0867015	0,127611	0,153215	0,13027	-0,407119	0,21073	0,6279808
CG10075-RA	CG10073	2,06861	49,6096	2,80105	14,1916	3,44112	0	-0,360527	0,177464	0,6279808
CG10076-RA	CG10075	11,3108	16,0652	0,662152	18,8067	23,5618	47,8232	0,371817	0,184041	0,6279808
CG10076-RB	spir	7,60086	0,0185322	0,0239474	6,86178	6,42045	1,9861	0,37528	0,179808	0,6279808
CG10076-RC	spir	0,0135125	1,6289	9,03477	0,0212655	0,0288043	6,83475	0,435925	0,121533	0,6279808
CG10076-RD	spir	0,0249437	8,45445	65,6139	9,92874	8,10102	5,52883	-0,011273	0,967486	0,6279808
CG10077-RA	spir	11,4731	40,969	0,0444027	0,0140568	0	0,0143497	0,093211	0,680407	0,6279808
CG10077-RB	CG10077	72,2951	4,38295	0	10,7062	6,63023	6,09889	-0,612204	0,00638	0,6279808
CG10078-RA	CG10077	0,0273984	44,8074	3,19089	52,8823	2,45101	0,115472	-0,219556	0,455445	0,6279808
CG10078-RB	Prat2	4,71225	22,0668	0,0375439	0,0427723	1,89997	0,0436636	-0,263343	0,369889	0,6279808
CG10079-RA	Prat2	28,1412	51,8624	36,2099	84,0699	6,76385	49,1446	0,183763	0,524685	0,6279808
CG10079-RB	Egfr	5,97804	6,83445	7,08265	0,497866	4,00318	8,57281	0,185347	0,52178	0,6279808
CG1007-RA	Egfr	8,45602	3,38113	6,11633	0,404018	4,61387	4,31179	0,253488	0,222303	0,6279808
CG10080-RA	mahj	4,51561	8,56695	5,87831	6,41683	12,0108	185,585	-0,263074	0,252795	0,6279808
CG10081-RA	CG10081	0,1084	31,5142	0,531916	6,77013	0	2,72714	0,698684	0,023379	0,6279808
CG10082-RA	CG10082	23,8114	0,151222	0,526231	0,0553935	0,676482	0	0,0105	0,963684	0,13772387
CG10082-RB	CG10082	10,5019	0,115521	0,537279	38,7137	0,502132	0,196654	-0,002759	0,990811	0,6279808
CG10083-RA	Abp1	15,1282	12,5221	18,8824	0,0651412	11,7992	10,9131	0,2679	0,16977	0,6279808
CG10084-RA	swm	10,9275	0,169063	9,56992	0,0589624	9,47737	8,88153	0,200514	0,361028	0,6279808

gene_id	Symbol	W1_FPKM	W2_FPKM	W3_FPKM	MCM51_FPKM	MCM52_FPKM	MCM53_FPKM	FC	p-value	p-adj
CG10084-RB	swm	19,7659	14,5928	12,8138	14,756	8,51634	12,8614	0,202083	0,357284	0,6279808
CG10086-RA	CG10086	6,35345	1,72251	2,44043	2,52377	3,74316	11,3358	-0,513663	0,102618	0,6279808
CG10089-RA	CG10089	0,606695	0,470819	1,04843	0,766208	1,77001	1,18865	-0,670479	0,041283	0,6279808
CG10089-RB	CG10089	0,355362	0,0471065	0,0620306	0,0583877	0,102174	0,0770041	-0,670479	0,041283	0,6279808
CG10089-RC	CG10089	0,232722	0,049065	0,446854	0,0611512	0,622727	0,557169	-0,594093	0,076047	0,6279808
CG10089-RD	CG10089	0,0538661	4,99295	0,0517147	802,838	0,0828297	0,0624254	-0,600994	0,052615	0,6279808
CG10090-RA	Psa	13,8328	17,6365	16,3349	0,0176771	0,0396754	16,1671	0,027373	0,901985	0,6279808
CG10091-RA	Psa	0,0166452	0,0151616	0,0159804	0,0187484	2,60888	0,306348	-0,403805	0,152898	0,6279808
CG10092-RA	Psa	0,0179766	0,0163743	0,0172586	0,0183413	0,0331599	55,3543	0,141566	0,571451	0,6279808
CG10093-RA	Psa	0,0170445	0,0155253	0,0163637	31,8541	0,0355507	3,22221	-0,962707	0,005826	0,6279808
CG10094-RA	Psa	0,0180354	0,0164279	0,0173151	3,96034	2,03071	1,23424	-0,131945	0,366425	0,6279808
CG10095-RA	Psa	0,0176595	0,0160855	0,0169541	3,9036	5,08809	1,50812	0,421844	0,102313	0,6279808
CG10096-RA	Tim17a1	0	0,148389	0,156402	46,9157	284,309	0,200271	-0,195415	0,554203	0,6279808
CG10096-RB	GstD9	0,0871714	0,0794017	17,7701	548,474	2,32885	13,6853	-0,165089	0,643392	0,6279808
CG10097-RA	ArgRS-m	0,0348685	18,4654	0,0334759	0,0389311	0,0527324	53,9362	-0,184638	0,575996	0,6279808
CG10097-RB	Cyp313a3	1,89873	0,793534	3,96748	0,142976	0,350595	0,0243402	-0,218089	0,537188	0,6279808
CG10098-RA	Cyp313a2	0	0,22039	0,066682	11,8708	0,168537	0	-0,267824	0,276881	0,6279808
CG1009-RA	dpr15	2,57488	0	0,0426323	0	0,0380678	0	0,146761	0,568375	0,13772387
CG1009-RB	CG10096	0,520424	0,0326923	0,516867	15,6058	0,0553486	0,538106	0,136798	0,593064	0,6279808
CG1009-RC	CG10096	0,0135327	0,0123266	0,0129922	21,6438	22,2497	0,0142103	0,145605	0,571531	0,6279808
CG1009-RD	CG10097	0,317495	0,157172	0,218671	0,537517	0,302246	0,0768247	0,152529	0,549828	0,6279808
CG1009-RE	CG10097	0,045144	0,226162	0,411739	0,0664552	2,77337	0,494287	0,145605	0,571531	0,6279808
CG1009-RF	CG10098	21,0975	22,0692	24,1352	3,23025	0,819615	3,61807	0,155648	0,541933	0,6279808
CG10101-RA	Ir84a	0	0	0,0566062	0	0,091867	0,0692365	NA	NA	0,6279808
CG10102-RA	CR10102	9,68872	5,992	1,93835	1,15343	0,362723	0,261634	-0,711875	0,016851	0,13772387
CG10103-RA	CG10103	19,8269	17,1483	3,11787	16,9347	23,105	13,0116	0,051003	0,805156	0,6279808
CG10104-RA	CG10104	0	0	3,09796	12,3749	7,77897	0	NA	NA	0,6279808
CG10105-RA	Sin1	3,94629	4,12928	4,25834	77,9227	0	3,31309	-0,065647	0,763131	0,13772387
CG10106-RA	Tsp42Ee	303,954	3,15793	253,588	5,43491	269,836	228,683	0,104051	0,644587	0,6279808
CG10107-RA	velo	14,6545	12,7866	0,660889	0,724866	0,550919	0,718117	-0,069932	0,725572	0,6279808
CG10107-RB	velo	0,0113656	37,6018	0,794614	1,14726	9,87569	0,75612	0,065496	0,742043	0,6279808
CG10107-RC	velo	2,76329	16,5585	18,5846	20,0456	0,0157561	23,42	-0,069932	0,725572	0,6279808
CG10108-RA	phyl	7,40971	0	8,7368	11,7665	0	0,84104	0,154577	0,573692	0,6279808
CG10109-RA	PRAS40	33,2148	41,9764	1,47979	44,9204	3,19621	3,8497	-0,046433	0,822789	0,6279808
CG10110-RA	Cpsf160	2,35737	1,59182	33,9251	0,861211	0,725944	2,03207	0,117931	0,673565	0,6279808
CG10110-RB	Cpsf160	0,577092	0,525656	2,92719	3,0852	28,9813	31,4091	0,118225	0,672964	0,6279808
CG10112-RA	Cpr51A	1181,57	16,1612	1222,85	33,107	11,6413	37,1251	0,056674	0,818272	0,6279808
CG10113-RA	wa-cup	0	0,0527027	28,521	138,091	42,7686	169,015	0,01562	0,897983	0,6279808
CG10116-RA	CG10116	11,6351	491,589	23,9957	0,0198677	18,8304	0,0664986	-0,582386	0,078506	0,13772387
CG10117-RA	ttv	7,01406	1,80763	2,63707	1212,39	2,36317	1,07475	0,30044	0,236185	0,6279808
CG10118-RA	ple	8,62427	0,430313	0,0179217	0,0194341	19,552	0,019839	0,179808	0,566753	0,6279808
CG10118-RB	ple	24,6596	0,621968	23,0492	53,1698	0,822304	15,741	0,167536	0,593505	0,6279808
CG10119-RA	LamC	187,646	2,99506	0,0450972	2,96824	9,85346	0,0534899	-0,2145	0,355285	0,6279808
CG10120-RA	Men	80,8313	98,4899	128,44	0,023428	124,496	119,199	-0,391894	0,080549	0,6279808
CG10120-RB	Men	0,0223104	0,0203218	0,0214193	3,89267	0,0317333	0,0239162	-0,392133	0,080363	0,6279808
CG10121-RA	SP1173	121,588	126,503	0,0283368	56,9839	21,9789	0,0321915	0,207182	0,268439	0,6279808
CG10121-RB	SP1173	0,0211127	0,0192309	45,0692	61,1103	41,7778	29,709	0,210541	0,261268	0,6279808
CG10121-RC	SP1173	0,0183154	0,0166829	96,4299	0,019052	44,3951	57,6709	0,205364	0,27302	0,6279808
CG10121-RD	SP1173	0,018784	0,0171098	22,266	0,019561	0,025806	26,9752	0,205364	0,27302	0,6279808
CG10122-RA	Rpl1	7,95337	37,2496	12,321	11,8713	7,76749	176,178	-0,165788	0,525069	0,6279808
CG10123-RA	Top3alpha	1,60378	6,25848	5,40718	16,2786	3,58216	41,0652	-0,200577	0,397112	0,6279808
CG10124-RA	eIF4E4	0,288122	0,145801	0,0922046	0,174278	4,25468	3,18637	0,314937	0,150198	0,6279808
CG10125-RA	zpg	0,704753	0,680843	1,12767	1,6564	21,7805	2,6732	-0,407714	0,206022	0,13772387
CG10126-RA	CG10126	0,0795454	0,0724555	10,3097	21,4591	7,40738	7,05327	0,054252	0,821773	0,6279808
CG10126-RB	CG10126	13,6303	19,5949	13,2949	131,557	181,749	9,38691	0,079542	0,741956	0,6279808
CG10128-RA	tra2	1,83196	0,044826	0,0472468	0,0552078	0,0733053	0,0563582	-0,056808	0,797298	0,6279808
CG10128-RB	tra2	5,14694	0,0444946	0,0468975	0,0547491	1,87567	0,0558899	-0,056482	0,798546	0,6279808
CG10128-RC	tra2	7,75522	0,0440388	0,0464171	0,0541196	1,38037	0,0552473	-0,072648	0,740791	0,6279808
CG10128-RD	tra2	0,0492124	0,0393461	0,0414709	2,76078	2,58622	0,0487242	-0,052666	0,815703	0,6279808
CG10128-RE	tra2	0,0488485	6,6598	3,9311	2,40052	1503,34	1,75319	-0,053565	0,811274	0,6279808
CG10128-RF	tra2	0,0483481	0,386686	5,96455	1,40366	280,086	2,08486	-0,056808	0,797298	0,6279808
CG10128-RG	tra2	1,79264	183,689	0,263721	0	0	0	-0,056808	0,797298	0,6279808
CG10129-RA	ndl	0,00808103	0,0110411	0,00775827	0,00820822	0,0111181	0,00837926	0,07688	0,829154	0,6279808
CG10130-RA	Sec61beta	305,178	0,131988	0	0	0	3,8141	0,160248	0,482252	0,6279808
CG10131-RA	Had2	2,66688	1,57182	3,31341	0	0	0	0,248519	0,461686	0,6279808
CG10132-RA	CG10132	9,83514	1,86943	18,2823	1,99683	33,3196	8,34409	-0,170531	0,371433	0,6279808
CG10133-RA	JMJD7	3,45256	9,94385	4,58125	31,4009	9,48366	310,321	0,219488	0,395587	0,6279808
CG10134-RA	beat-Va	4,79288	3,66584	24,8002	18,9773	8,13413	28,3189	-0,596181	0,040594	0,6279808
CG10137-RA	CG10137	2,86684	0,749373	3,53955	1,91846	0,0798649	4,06412	-0,419484	0,051671	0,6279808
CG10138-RA	PpD5	0,0815081	0,127298	0	14,3464	0	0,157365	-0,027978	0,880455	0,6279808
CG10139-RA	CG10139	10,5299	0	0	0	0	0,0942967	0,117924	0,656394	0,13772387
CG10140-RA	robl62A	0,131976	0,120213	0,126705	0	176,19	91,3993	-0,544748	0,12689	0,6279808
CG10142-RA	CG10140	3,39988	0,908856	36,7074	3,80212	31,5496	23,0996	-0,407329	0,159032	0,13772387
CG10143-RA	Ance-5	15,7309	3,78872	22,1295	3,94026	2,67888	20,8204	NA	NA	0,6279808
CG10144-RA	Adgf-E	0	0	0	0,0690545	232,992	0	0,03967	0,861675	0,6279808
CG10145-RA	Vps8	4,25167	4,98896	4,53006	5,95306	7,3983	7,66797	0,492625	0,025271	0,13772387
CG10146-RA	mspo	24,0995	29,3151	31,5366	34,1695	87,6694	86,0871	-0,599874	0,090616	0,6279808
CG10147-RA	AttA	0,372142	1,06795	0,238186	1,93355	3,30395	0,742815	0,138347	0,592395	0,6279808
CG10148-RA	CG10147	3,92433	3,84264	4,59175	5,33394	3,00602	2,92575	0,132817	0,565721	0,6279808
CG10149-RA	2mit	5,90678	5,57282	7,58483	5,94109	6,80019	5,5055	0,034729	0,866519	0,6279808
CG10149-RB	Rpn6	19,5096	101,286	0,98566	0,0291489	18,4832	0,0297563	0,016092	0,938024	0,6279808
CG1014-RA	Rpn6	17,8235	11,4609	0,0263259	9,25126	10,205	3,46224	0,099493	0,503366	0,6279808
CG10151-RA	CG10151	0,0243583	0,0221872	29,4951	240,787	0,0388634	11,5855	0,068828	0,810198	0,6279808
CG10151-RB	CG10151	1,05368	1,25508	18,077	0,146582	0,0394824	32,1139	0,067449	0,814853	0,6279808
CG10151-RC	CG10151	0,0274212	0,0249771	22,2205	0,0257036	4,26002	0,0262392	0,064859	0,82176	0,6279808
CG10151-RD	CG10151	4,91283	8,15528	0,0233854	2,48212	0	1,53178	0,067449	0,814853	0,6279808
CG10152-RA	beat-IV	2,41341	2,63901	2,53072	2,86222	0,784008	0	0,155222	0,517341	0,6279808
CG10153-RA	Trs31	13,0238	21,527	19,4972	35,4725	0,198546	406,186	-0,232446	0,424047	0,6279808
CG10154-RA	CG10154	0,16211	29,791	38,8382	3631,68	54,5791	17,8705	0,303463	0,331235	0,6279808
CG10155-RA	Spred	24,7163	29,0169	23,741	39,6624	26,8869	0,149637	-0,286758	0,180233	0,6279808
CG10157-RA	GILT2	34,0162	14,1762	9,20943	11,8844	0,787024	7,46477	-0,17414	0,439235	0,6279808
CG10158-RA	Fgop2	12,8296	15,7631	3,35521	3,2922	20,5745	13,7353	-0,017082	0,93881	0,6279808
CG10159-RA	BEAF-32	18,6577	5,7737	14,2988	10,2541	0,137622	11,3354	0,157681	0,471933	0,6279808
CG10159-RB	BEAF-32	6,25001	0,0439104	0	0	1,01699	0	0,056785	0,803324	0,6279808
CG10160-RA	Ldh	131,63	230,204	0	229,958	151,639	147,807	0,038982	0,869129	0,6279808
CG10161-RB	eIF3d1	75,4825	0,29117	3,33973	7,51559	16,8244	0,0638379	-0,26549	0,171561	0,6279808
CG10163-RA	CG10163	0,377743	0,529345	0,613724	1,10631	4679,5	15,889	-0,257518	0,468402	0,6279808
CG10164-RA	CG10164	0	0,648961	1,01624	0,431517	2,19561	1,66883	NA	NA	0,6279808
CG10165-RA	CG10165	20,572	0,159505	0,0112964	0,585841	1,19463	0,0123048	0,08829	0,766951	0,13772387
CG10165-RB	CG10165	0,0521651	3,65371	9,49082	2,82526	0,0163328	5,72069	0,075607	0,795992	0,6279808
CG10166-RA	CG10166	26,485	0,02716	42,9902	46,5357	8,50062	47,263	-0,128664	0,671894	0,6279808
CG10168-RA	Ugt303B2	0,895668	0	0	23,4514	0,116219	0,505034	0,116936	0,716228	0,6279808
CG10170-RA	Mfap1	10,8532	13,1443	15,096	0,0169408	7,2779	11,1856	0,210158	0,395276	0,6279808
CG10171-RA	Ugt303B3	6,48656	6,02898	1,70791	5,28966	0,674894	1,19474	-0,154959	0,555967	0,6279808

gene_id	Symbol	W1_FPKM	W2_FPKM	W3_FPKM	MCM51_FPKM	MCM52_FPKM	MCM53_FPKM	FC	p-value	p-adj
CG10171-RB	CG10171	14,8339	19,6449	1,204	8,7096	6,48169	93,7915	-0,154959	0,555967	0,6279808
CG10173-RA	CG10171	0,041126	0,0374604	4,38914	0	35,4487	4,15487	-0,014184	0,944539	0,6279808
CG10174-RA	Best2	10,2958	9,41209	9,81313	11,3476	24,271	0,0959535	-0,017361	0,950633	0,6279808
CG10175-RA	Dsim\Ntf-2r	4,4339	4,23571	6,54095	13,8254	5,69988	8,06277	0,254242	0,257053	0,6279808
CG10175-RB	CG10175	0,0273984	0,418563	0,02249	0,73741	60,5924	5,50376	0,242929	0,277321	0,6279808
CG10175-RC	CG10175	2,4714	0,0438677	21,8155	0,0532667	7,20448	7,69495	0,251918	0,261889	0,6279808
CG10176-RA	CG10175	21,7668	0,0249564	13,8478	14,096	32,3412	27,7083	-0,266426	0,352968	0,6279808
CG10177-RA	grnd	13,7128	0,0204183	2,41035	7,41875	8,55431	13,4513	-0,322592	0,356604	0,6279808
CG10178-RA	CG10177	0,183544	0,168274	0,59472	0,399337	0,540904	0,51513	-0,487226	0,071092	0,6279808
CG1017-RA	Ugt301D1	7,35876	10,5585	6,17577	11,1453	18,8877	14,8839	0,227634	0,331047	0,6279808
CG10181-RA	Mdr65	9,15451	7,48501	11,5878	15,4272	29,2161	7,35177	-0,987177	0,000485	0,6279808
CG10182-RA	CG10182	4,11222	0,423849	0,0263041	7,70014	9,70236	39,5384	-0,304045	0,374285	0,6279808
CG10183-RB	CG10183	0,0481603	26,4832	0,0924735	3,93989	2,21595	2,23169	-0,023773	0,923881	0,6279808
CG10184-RA	CG10184	10,133	6,71258	16,9468	15,6369	13,822	15,7288	-0,924878	0,005153	0,13772387
CG10185-RA	CG10185	0,343152	0,561463	0	0,0154729	1,82841	0,627445	0,018175	0,953349	0,6279808
CG10186-RA	Hasp	0,563147	0,0233281	3,25095	0,0277144	7,63998	9,06471	-0,828671	0,000518	0,6279808
CG10186-RC	Hasp	0,0117705	0,0246262	25,4194	0,029396	7,13043	1,00392	-0,832655	0,000498	0,6279808
CG10186-RD	Hasp	2,77983	0,027972	0,0500816	0,0329044	0,630491	16,5592	-0,453128	0,090185	0,6279808
CG10186-RE	Hasp	0,0117663	160,374	0,0104981	266,137	3,07027	0,835668	-0,830425	0,000504	0,6279808
CG10186-RF	Hasp	5,02463	0,0504637	0,0113004	0,0631436	21,9516	0,0123094	-0,828671	0,000518	0,6279808
CG10188-RA	CG10188	2,24844	15,6442	0,937221	4,73525	0,724029	1,43393	0,23782	0,400076	0,6279808
CG10188-RB	CG10188	5,26336	0,0475156	5,50804	12,7006	3,21882	2,94619	0,23782	0,400076	0,6279808
CG10189-RA	CG10189	5,57934	3,52906	2,02596	45,2396	2,04599	1,66932	-1,164736	6,12E-06	0,6279808
CG10191-RA	Mlp84B	113,449	1,55824	3,24971	20,2753	0,237041	2,38629	0,005291	0,982358	0,6279808
CG10192-RA	Mlp84B	14,7752	0,114495	4,86581	5,24153	0,180115	0,178648	0,143939	0,673353	0,6279808
CG10194-RA	Poc1	5,3745	7,81427	0,0394834	3,91504	0,813166	0,961234	0,370797	0,095057	0,6279808
CG10195-RA	eIF4G2	6,65278	14,6619	4,67248	36,7534	3525,51	3580,13	0,16677	0,559776	0,6279808
CG10197-RA	CG10194	10,9296	0,0107176	14,2122	21,114	0,0669286	0,0485483	0,345481	0,216559	0,6279808
CG10197-RB	CG10195	3,45306	8,88206	19,9863	7,00592	0,0732451	9,5641	0,331991	0,232695	0,6279808
CG10197-RC	kn	0,0193903	5,04836	6,14309	7,08841	2,94001	5,02558	0,361344	0,197283	0,6279808
CG10198-RA	kn	7,33481	6,67927	4,74198	12,1106	1,43929	4,71891	0,056591	0,832436	0,6279808
CG1019-RA	kn	10,7794	8,01533	166,439	0,0209878	90,8829	0,0214252	-0,397791	0,12419	0,6279808
CG1019-RB	Dsim\Nup98	5,27852	6,53295	34,2515	29,4889	0,0715424	13,3665	-0,397695	0,124347	0,6279808
CG10200-RA	hui	78,4913	1,79637	6,93541	6,76262	5,05473	13,3668	0,074137	0,807224	0,6279808
CG10200-RB	hui	0,0893317	55,1979	1,9386	82,3976	0,0284281	3,78915	0,074749	0,805717	0,6279808
CG10202-RA	Pgm2b	0	0,0480339	0,0506279	0,0627229	0	0,0640299	0,01562	0,897983	0,6279808
CG10203-RA	x16	37,1052	100,895	49,1361	55,0969	30,9459	41,5077	-0,188752	0,4049	0,13772387
CG10205-RA	CG10205	1,12233	0,493523	4,31003	3,55622	9,53715	2,29944	-0,670225	0,060851	0,6279808
CG10205-RB	CG10205	0,0828363	0,075453	0,0795277	0,101603	8,67286	3,72633	-0,670225	0,060851	0,6279808
CG10206-RA	nop5	76,1602	0,0229223	136,38	107,211	85,6599	98,8687	-0,192405	0,484374	0,6279808
CG10207-RA	NaPi-T	1,63395	5,64992	3,791	1,7641	0,0849585	1,12588	0,414728	0,229432	0,6279808
CG10208-RA	CG10208	7,91049	30,8697	14,9508	0,0389311	20,564	0,0565953	-0,226665	0,473817	0,6279808
CG10209-RA	CG10209	5,51721	0,699056	3,93726	3,83837	4,43737	72,2222	0,308048	0,211649	0,6279808
CG10210-RA	Dmtn	16,7684	0	13,7001	0,0186107	0	0,0189985	-0,092242	0,642867	0,6279808
CG10211-RA	Dmtn	0,0179084	23,003	0,0171931	24,43	0	11,3588	0,388166	0,237317	0,6279808
CG10212-RA	Dmtn	13,6395	0,0163122	16,5551	0,0181995	0,161975	0,0185787	0,009061	0,977565	0,6279808
CG10214-RA	Dmtn	0,0175283	20,5267	0,0168282	8,36873	0	3,23118	0,009455	0,975855	0,6279808
CG10215-RA	Dmtn	6,25706	0,015966	3,45528	0,257665	0	0	0,195381	0,485063	0,6279808
CG10217-RA	tst	2,39186	0,0326804	2,9342	17,8657	0,0709735	0,0185276	-0,079643	0,677835	0,6279808
CG10217-RB	CG10211	52,784	15,9841	50,4218	49,1646	13,6118	23,203	-0,079805	0,677233	0,6279808
CG10219-RA	SMC2	1,68788	0,0813695	0,191844	17,2766	0,491189	0,10372	-0,251847	0,367525	0,6279808
CG1021-RD	CG10214	22,6588	2,14307	11,124	29,2456	27,5637	11,7007	0,162983	0,546098	0,6279808
CG1021-RE	Ercc1	0,075717	0,0176621	0,0726929	8,36289	12,6341	0,0929686	0,199897	0,462583	0,6279808
CG1021-RF	udt	5,08938	24,6861	16,1752	29,1816	0,131344	0,0989885	0,164478	0,542864	0,6279808
CG1021-RG	udt	35,9772	22,2249	30,1568	13,6439	120,06	117,803	0,179044	0,506444	0,6279808
CG1021-RH	Dpse\GA10165	64,1626	9,8112	111,723	0,0408119	16,3962	0,0534899	0,180749	0,502798	0,6279808
CG10221-RA	Hrd3	12,9302	11,2424	15,7378	16,4588	16,8835	20,9544	-0,033153	0,868088	0,6279808
CG10222-RA	CG10222	2,85734	2,92799	0,0546554	0,0452081	0,0612346	0,0461501	-0,229044	0,427395	0,6279808
CG10223-RA	Top2	10,8675	0,0750765	12,5973	0,0494118	1,49752	0,0552019	0,096562	0,746874	0,6279808
CG10225-RA	RanBP3	0,0384236	0,0415079	35,9298	0,0528181	1,46833	6,98555	0,249829	0,194599	0,6279808
CG10226-RA	CG10226	3,62485	2,68495	3,74012	3,74517	8,71787	0,539833	-0,395784	0,129264	0,6279808
CG10228-RA	Pcf11	12,3375	12,19	12,8954	20,1523	13,414	12,9944	-0,415929	0,022171	0,6279808
CG10229-RA	Kat60	17,6094	0,132084	0,0697362	0,214966	17,2984	17,6592	-0,154508	0,393438	0,6279808
CG10230-RA	Rpn9	47,0261	18,0735	59,9897	0,0181494	7,08948	56,6155	0,027956	0,91308	0,6279808
CG10230-RB	Rpn9	0,0455696	0,0454238	0,0437495	48,8393	52,7865	1,71894	0,012959	0,958427	0,6279808
CG10232-RA	CG10232	9,48614	15,5267	18,858	11,2946	18,0491	15,0151	0,433312	0,121719	0,6279808
CG10233-RA	rtp	0,0726373	15,9836	47,1536	0,00975664	38,4725	0,0884456	0,661809	0,02445	0,6279808
CG10233-RB	rtp	0,145009	36,148	19,5874	0,0112625	22,1916	0,219446	0,22724	0,524899	0,6279808
CG10234-RA	Hs2st	7,61499	2,0595	1,63775	27,5576	6,31186	3,88561	0,654608	0,005502	0,6279808
CG10236-RA	LanA	85,3934	107,922	181,703	109,778	7,29514	4,56816	0,386317	0,20704	0,6279808
CG10237-RA	CG10237	0,0483128	11,5937	7,21565	18,9826	0	9,90036	0,186309	0,443962	0,6279808
CG10237-RB	CG10237	5,91033	4,41614	0,0463832	5,47029	5,82197	13,364	0,184066	0,451431	0,6279808
CG10237-RC	CG10237	20,0403	5,98606	15,6659	14,6336	8,24256	0	0,165198	0,49728	0,6279808
CG10238-RA	Mocs2B	19,6302	10,9722	31,5381	15,6964	3,91166	23,7502	-0,149542	0,64471	0,6279808
CG10240-RA	CG1024	1,99115	0,560619	0,0203475	1,97821	8,92825	0,628724	-0,245919	0,33219	0,6279808
CG10241-RA	CG1024	1,34439	2,63474	2,16996	3,59508	2,1264	1,22831	0,463344	0,193669	0,6279808
CG10242-RA	Cyp6a22	4,01161	5,97297	5,22159	6,27034	6,21758	5,27832	-0,255738	0,329215	0,6279808
CG10243-RA	Cyp6a17	2,55136	2,63907	15,963	4,32465	4,72396	2,73947	0,18941	0,532726	0,6279808
CG10244-RA	Cyp6a23	52,1424	8,83226	63,0938	74,9287	3,01569	2,45388	0,41586	0,16288	0,6279808
CG10245-RB	Cyp6a19	0,0355119	0,0323467	3,52642	1,99774	86,6196	63,287	-0,368188	0,217688	0,6279808
CG10246-RB	Cad96Ca	24,961	0	21,7208	23,6737	13,7037	3,22844	-0,267255	0,377206	0,6279808
CG10247-RA	Cyp6a20	13,8469	2,93675	9,36278	5,74477	9,6495	6,88537	-0,428606	0,221503	0,6279808
CG10248-RA	Cyp6a9	6,25945	4,96129	21,9508	14,6182	19,3626	14,5928	-0,688144	0,04409	0,6279808
CG10249-RA	Cyp6a21	1,61456	1,01107	0,0340935	0,0384989	0,052147	0,0393011	-0,310947	0,163347	0,6279808
CG10249-RB	Cyp6a8	7,26353	81,6474	16,5085	18,1529	8,06265	14,6646	-0,283243	0,206803	0,6279808
CG10249-RC	Kank	22,3437	22,123	21,177	19,034	20,945	19,6872	-0,323398	0,145546	0,6279808
CG10249-RD	Kank	0,0158544	0,0144413	0,0152212	0,016397	0,0222098	0,0167387	-0,315544	0,157728	0,6279808
CG1024-RB	Kank	0,0147075	0,0133966	0,0141201	7,69734	5,0109	4,90568	-0,133818	0,597686	0,6279808
CG1024-RC	Kank	9,2688	6,1526	7,51744	7,11403	7,70588	7,26411	0,050996	0,829833	0,6279808
CG10250-RA	nau	4,35101	4,77616	2179,54	2137,45	19,9201	25,3625	0,286798	0,183453	0,6279808
CG10251-RB	prt	1,81427	1282,47	109,771	0,0625348	2,62128	3,97042	0,042144	0,879654	0,6279808
CG10252-RA	CG10252	0,113532	0	2,137	0	0,19966	1,81057	-0,206453	0,507185	0,6279808
CG10253-RA	ADPS	7,06962	0,271927	0,286612	0	5,71153	2,29392	-0,144441	0,489237	0,6279808
CG10254-RA	CG10254	2,73866	86,2046	2,62928	1,36138	3,3567	1,71422	0,259522	0,231813	0,6279808
CG10254-RB	CG10254	5,44444	0	6,03727	3,36993	3,27451	2,48211	0,263491	0,224427	0,6279808
CG10255-RA	Lap1	4,12182	5,68941	4,2692	5,46607	1,95148	6,86347	0,409425	0,148931	0,6279808
CG10257-RA	CG10257	0,0567662	0,0246598	0,054499	0,0287546	0,601489	0	-0,14583	0,636958	0,6279808
CG10260-RB	PI4KIIIalpha	5,40193	5,2856	5,84527	9,97317	5,91142	5,67914	-0,480432	0,01463	0,6279808
CG10261-RA	aPKC	14,961	0,0245994	13,8726	0,0286795	2,27534	0,0699566	-0,308744	0,213216	0,6279808
CG10261-RB	aPKC	0,0333436	1,32445	0,0320118	3,28163	0,976423	9,55144	-0,777589	0,00209	0,6279808
CG10261-RC	aPKC	0,0327649	4,98313	0,0314562	4,08934	5,92619	4,5933	-0,761698	0,001781	0,6279808
CG10261-RD	aPKC	0,0326677	16,7562	0,0313629	0,0349832	0,0928224	4,41479	-0,757606	0,001926	0,6279808
CG10261-RE	aPKC	0,0270729	0,0150856	0,0259916	44,4228	11,1081	0,0359028	-0,78095	0,001926	0,6279808
CG10261-RF	aPKC	0,0270065	4,17453	0,0259278	0,0389311	5,21639	0,0293537	-0,77537	0,001843	0,6279808

gene_id	Symbol	W1_FPKM	W2_FPKM	W3_FPKM	MCM51_FPKM	MCM52_FPKM	MCM53_FPKM	FC	p-value	p-adj
CG10262-RA	PCNA2	4,43087	0,0373906	5,8646	10,3078	0,0445692	0,03359	-0,453967	0,092605	0,6279808
CG10263-RA	Hakai	0,0381133	0,00912792	0,0365911	0,0415846	0,593216	0,598048	-0,049186	0,807301	0,6279808
CG10263-RC	Hakai	6,04328	0,00911962	7,40116	11,0523	6,66446	2,54464	-0,049186	0,807301	0,6279808
CG10263-RD	Hakai	2,70554	212,141	2,93456	0,0454262	10,2679	9,29233	-0,043888	0,828141	0,6279808
CG10264-RA	CG10264	20,9281	19,8771	4,79949	0,0276099	0,0363469	0,0281853	0,66415	0,001963	0,6279808
CG10265-RA	CG10265	4,31768	0,0517066	4,16327	0,0685286	2,65481	3,50675	0,400913	0,103355	0,6279808
CG10265-RB	CG10265	0,032507	14,5493	8,98165	10,1099	0,047385	0,0357122	0,400913	0,103355	0,6279808
CG10267-RA	Zif	16,8152	31,1102	22,2904	33,7734	15,5891	19,4703	0,027714	0,917298	0,6279808
CG10268-RA	CG10268	25,9748	0,00911962	36,4091	9,91447	5,38172	5,61251	0,452436	0,062282	0,6279808
CG10269-RA	D19A	7,39902	0,565258	1,36062	0,369669	5,41522	0,498901	0,204619	0,406213	0,6279808
CG10270-RA	D19B	7,22006	8,6793	9,2724	1199,64	0,471448	6,57903	-0,022282	0,924078	0,6279808
CG10272-RA	gpp	0,00865358	8,10272	0,955415	12,1875	1,13169	0,97054	-0,136472	0,666334	0,6279808
CG10272-RB	gpp	5,52784	3,71138	4,62453	6,61356	2,66651	3,73527	-0,138504	0,661652	0,6279808
CG10274-RA	CG10274	5,34648	0,0201517	7,67732	1,72303	0,346559	0,870261	0,06081	0,801724	0,6279808
CG10274-RB	CG10274	0,0221236	8,61684	0,85112	6,66095	0,752651	3,16808	0,06081	0,801724	0,6279808
CG10275-RB	kon	7,99795	10,2323	13,3628	20,3368	2,78224	3,86031	0,328201	0,295463	0,6279808
CG10277-RA	gzl	0,0258546	0,0235502	0,024822	12,1505	0,037087	0,027951	-0,519142	0,012656	0,6279808
CG10277-RB	gzl	22,6022	27,9573	21,2961	23,4018	25,6048	21,4425	-0,23995	0,281562	0,6279808
CG10277-RC	gzl	0,0198565	0,0180867	0,0190634	0,0207303	0,0280793	0,0211623	-0,191787	0,393472	0,6279808
CG10277-RD	gzl	0,021012	0,0191392	0,0201728	0,0219967	0,0297947	0,0224551	-0,239961	0,282296	0,6279808
CG10278-RA	GATAe	19,8399	3,69566	20,906	5,67709	0,0373978	0,0157953	0,575045	0,06291	0,6279808
CG10279-RA	Rm62	0,0231872	34,7024	0,405347	0,375912	0	0	-0,11472	0,661372	0,6279808
CG10279-RB	Rm62	0,0235761	9,21374	84,5589	62,2718	0	172,585	-0,115064	0,660409	0,6279808
CG10279-RC	Rm62	0,022462	0,384578	0,0799286	10,9168	0,184779	0	-0,113974	0,663553	0,6279808
CG10279-RD	Rm62	0,0217809	168,629	193,117	153,895	1,63608	0	-0,113628	0,664521	0,6279808
CG10279-RE	Rm62	214,248	0	2,61523	0	111,741	1,34446	-0,113922	0,663739	0,6279808
CG10279-RF	Rm62	0,0236351	0,928804	0,273729	0,363869	2,07284	1,62475	-0,088712	0,736153	0,6279808
CG10280-RA	Antp	0,0137381	0,0125136	3,5675	0,0148087	1,94925	6,92399	0,063983	0,826699	0,6279808
CG10281-RA	Antp	0,0137725	0,0125449	0,0137946	3,79429	2,05833	0,0204606	0,288714	0,267297	0,6279808
CG10283-RA	Antp	0,0136276	0,012413	3,34011	0,01468	3,45655	0,0218639	0,288383	0,341096	0,6279808
CG10283-RB	Antp	4,33751	0,0124438	0,0136786	13,2098	3,64582	0,0172098	0,286889	0,343759	0,6279808
CG10284-RA	Antp	0,0143685	0,0130878	12,7541	0,020043	0,0271483	31,6123	-0,115412	0,737888	0,6279808
CG10286-RA	Antp	6,69882	10,3599	0,0184588	0,0214176	0,0290103	0,0857399	-0,070674	0,761559	0,6279808
CG10287-RA	Antp	0,0142477	0,0129777	0,0196662	0,0168585	0,0228349	19,0453	0,564536	0,040939	0,6279808
CG10287-RB	Antp	13,5061	15,952	0,0156338	106,394	61,5392	101,203	0,286366	0,282313	0,6279808
CG10289-RA	Antp	0,0192267	0,017513	34,6559	39,8083	0,0553142	57,126	0,025022	0,908333	0,6279808
CG1028-RD	Antp	0,0204844	0,0186586	8,02556	7,20387	7,8279	4,36517	0,581673	0,018122	0,6279808
CG1028-RE	Antp	0,0162842	0,0148328	11,2822	13,9764	85,3645	8,29659	0,580701	0,018171	0,6279808
CG1028-RF	CG10280	4,90479	3,2313	3,61311	4,55221	0,384578	0	0,581393	0,018162	0,6279808
CG1028-RG	TfIIFalpha	14,1422	1,144	3,43054	1,12854	0,0274405	2,55166	0,580701	0,018171	0,6279808
CG1028-RH	CG10283	7,7773	12,9122	9,96176	29,842	1,04973	1,40859	-0,078923	0,767072	0,6279808
CG1028-RI	CG10283	3,63144	17,0915	8,79358	10,5666	12,4328	9,44999	0,23615	0,368334	0,6279808
CG1028-RJ	Dsim\GD19871	0,615477	14,4996	26,3264	25,6841	0,0300657	13,2168	0,193469	0,462152	0,6279808
CG1028-RK	CG10286	19,4123	15,1846	28,8955	25,1004	17,0909	19,3136	0,581393	0,018162	0,6279808
CG1028-RL	Gasp	0,0550784	341,195	79,2125	403,885	224,013	257,865	0,581673	0,018122	0,6279808
CG1028-RM	Gasp	732,486	0,0215285	11,6626	0,0248975	0,0337238	0,0254163	0,580966	0,018131	0,6279808
CG1028-RN	fmt	9,25794	9,2217	11,161	12,4106	6,36793	118,732	0,580966	0,018131	0,6279808
CG10293-RA	how	57,2168	52,3819	0,0123981	40,1008	0,0179668	0,0135409	0,479212	0,08573	0,6279808
CG10293-RB	how	67,0771	79,9275	4,35319	60,7588	5,80834	5,63581	-0,05237	0,851515	0,6279808
CG10293-RC	how	0,0396931	0,0361552	22,7431	0,0434779	19,233	18,569	0,478478	0,06981	0,6279808
CG10295-RA	Pak	17,6229	21,9656	0,464275	21,2878	0,80953	0,0206808	0,072022	0,775569	0,6279808
CG10295-RB	Pak	0,0187042	0,0170371	14,4028	5,17922	2,84899	0,0226593	0,076526	0,763643	0,6279808
CG10295-RC	Pak	0,0194246	0,0176932	0,0179572	0,0202586	0,564692	0,456008	0,080889	0,750861	0,6279808
CG10295-RD	Pak	0,021194	0,019305	2,5642	0,0221968	9,40157	4,81197	0,009305	0,970476	0,6279808
CG10296-RA	Hr83	0,236831	6,90665	0,454744	5,44514	87,0375	135,524	0,208025	0,522572	0,6279808
CG10297-RA	Acp65Aa	3,29317	7,30419	11,2187	8,66215	20,5196	36,8666	-0,816525	0,021562	0,6279808
CG10298-RA	CG10298	0,841449	0,323583	0,552733	0,41548	0,777702	0,39999	-0,43696	0,212968	0,6279808
CG10300-RA	Scr	5,09478	5,16918	0,0256656	5,04176	0,175696	2,28119	0,001191	0,996515	0,6279808
CG10301-RB	Scr	6,95637	8,17909	0,0254391	6,59364	0,270373	3,86455	0,462023	0,099919	0,6279808
CG10302-RA	Scr	0,0160508	0,0146202	0	0,0166078	0,0383129	0,0169538	0,116087	0,640323	0,6279808
CG10303-RA	CG10300	14,3275	10,5035	4,0487	4,9622	0,0670291	1,98047	0,391014	0,250438	0,6279808
CG10305-RA	CG10301	4,34049	4,90118	4,24786	8,4393	1,34039	98,3155	-0,380888	0,19823	0,6279808
CG10305-RB	bsf	17,9486	23,7305	23,3408	16,1685	4,18903	6,17787	-0,380888	0,19823	0,6279808
CG10305-RC	Osi4	0,130757	788,709	0,0226911	652,157	254,071	379,701	-0,380886	0,198231	0,6279808
CG10306-RA	RpS26	672,499	1,651	1,66106	20,5043	0,47761	0,635703	-0,067796	0,782508	0,6279808
CG10307-RA	RpS26	876,395	2,58226	3,66384	0,473919	0	17,1807	0,16309	0,529968	0,6279808
CG10308-RA	RpS26	0,108737	0,366098	0,231521	0,986077	18,0011	0	-0,073947	0,835712	0,6279808
CG10308-RB	eIF3k	99,9122	14,3206	0,0518698	21,6249	21,7048	28,0881	-0,087784	0,805521	0,6279808
CG10309-RA	CG10307	0,0540277	115,787	4,33439	174,439	172,6	209,74	0,001727	0,994282	0,6279808
CG1030-RA	CycJ	0,0444955	21,0514	17,7479	0,0513767	14,5201	27,5123	0,45964	0,056868	0,6279808
CG1030-RB	CycJ	0,04408	0,0405296	28,3593	0,0508219	0,104201	9,45987	0,457655	0,060269	0,6279808
CG1030-RC	pad	1,72931	1,70339	1,76642	2,11998	11,5561	98,9559	0,457655	0,060269	0,6279808
CG10311-RA	alpha-Est1	10,4535	0	1,84498	2,37675	1,9836	1,18499	-0,411564	0,17453	0,6279808
CG10315-RA	CG10311	64,3948	50,9366	0,02002	8,83499	0,259714	0,0135028	-0,234672	0,343327	0,6279808
CG10315-RB	eIF2Bdelta	0,0325177	0,0296193	6,50554	10,6746	10,2966	22,5435	-0,234797	0,339456	0,6279808
CG10315-RC	eIF2Bdelta	0,0319207	0,0290756	0,0202695	5,1499	0,0299447	0,0367663	-0,219742	0,374445	0,6279808
CG10317-RA	eIF2Bdelta	11,7692	16,3903	2,56317	28,2791	2,18303	0,0360182	-0,069625	0,758005	0,6279808
CG10317-RB	CG10317	0,075717	0,0264944	0,301362	0	0,0373978	6,66754	-0,069625	0,758005	0,6279808
CG10318-RA	CG10317	0,203519	8,53456	0	0	6,60121	35,9854	-0,019686	0,939877	0,6279808
CG10318-RB	NC2alpha	10,6284	30,5671	0,047388	0,59994	28,9067	10,9388	-0,028919	0,912076	0,6279808
CG1031-RA	NC2alpha	0,0493594	13,7092	0,206376	0,267212	13,1276	1,74036	-0,573437	0,051507	0,6279808
CG10320-RA	Dpse\GA10242	0,16602	7,94916	290,92	0,206868	1,89309	0,0306372	-0,494131	0,114582	0,6279808
CG10320-RB	Dpse\GA10242	0,126825	15,7262	13,289	343,995	15,0945	0,343882	-0,494384	0,114387	0,6279808
CG10320-RC	Dpse\GA10242	210,726	0,0256695	13,5402	10,5383	0,456282	0,211178	-0,493269	0,115158	0,6279808
CG10321-RA	Dsim\GD11609	11,9472	4,99305	6,48065	9,12362	0,0189793	0,0742829	0,038423	0,862741	0,6279808
CG10324-RA	CG10324	5,18064	4,12902	6,46256	6,00059	62,4478	0,0300085	0,383357	0,068936	0,6279808
CG10325-RA	abd-A	18,7983	0,976918	17,8207	0,0147227	4,95585	0,0152961	0,337236	0,206308	0,6279808
CG10325-RB	abd-A	0,013784	0,052857	16,584	6,70533	23,633	1,42882	0,360035	0,175661	0,6279808
CG10325-RC	abd-A	10,833	19,966	0,0132335	0,0145568	0,0201718	2,50715	0,335713	0,208169	0,6279808
CG10326-RA	Arl6IP1	30,6653	32,1731	38,9714	35,5805	0,142763	0,0295342	0,288821	0,147672	0,6279808
CG10327-RA	TBPH	4,99074	13,1658	4,09831	4,01831	3,46322	17,9493	-0,067822	0,749658	0,6279808
CG10327-RB	TBPH	7,15514	23,4212	11,0092	15,64	7,71796	42,2566	-0,017952	0,93245	0,6279808
CG10327-RC	TBPH	11,4226	3,36416	12,1754	11,0803	5,67899	3,57934	-0,038554	0,853729	0,6279808
CG10327-RD	TBPH	0,0407456	5,2801	0,0391182	0,0447475	0,0606108	8,78492	-0,050177	0,81293	0,6279808
CG10328-RA	nonA-l	5,95517	5,66624	6,68842	15,9998	0,0286747	0,0194097	0,07401	0,765802	0,6279808
CG10332-RA	IM18	0,134119	0,122165	0,257524	0	11233,6	0	0,017941	0,940286	0,6279808
CG10333-RA	CG10333	9,10621	60,1022	43,3078	14,6722	5,8624	8,1977	0,006474	0,979099	0,6279808
CG10334-RA	spi	0,0350597	99,9009	0,0336594	0,0379665	0,0514259	0,0387577	0,303255	0,100954	0,6279808
CG10334-RB	spi	34,0956	103,43	26,0316	23,4019	25,3336	18,6551	0,303852	0,100096	0,6279808
CG10334-RC	spi	25,1539	0,00911962	19,205	10,2056	0,0721789	20,146	0,303874	0,099983	0,6279808
CG10334-RD	spi	0,0417761	0,00911962	0,0401075	0,045997	0,0623032	0,0469555	0,303088	0,101031	0,6279808
CG10334-RE	spi	0,0433663	0,00850252	0,0416342	0,0479379	0,0649322	0,0489368	0,306529	0,099076	0,6279808
CG10334-RF	spi	17,224	0,00850252	30,1552	31,2838	25,6058	20,949	0,306526	0,097819	0,6279808
CG10334-RG	spi	11,0628	0,00850252	15,1925	15,8853	7,57594	7,75959	0,304482	0,099537	0,6279808

gene_id	Symbol	W1_FPKM	W2_FPKM	W3_FPKM	MCM51_FPKM	MCM52_FPKM	MCM53_FPKM	FC	p-value	p-adj
CG10335-RA	Pbgs	33,3034	36,8857	41,7274	25,9013	3,3671	2,26038	0,02668	0,923062	0,6279808
CG10336-RA	CG10336	0,0524412	11,8678	0,0503467	0,0623077	13,4855	17,9375	-0,110836	0,678015	0,6279808
CG10336-RC	CG10336	0,0544282	7,41189	0,0522544	0,0651412	9,84203	0,063606	-0,103623	0,699408	0,6279808
CG10337-RA	CG10337	19,5692	25,7364	10,5611	32,9388	0,039817	0,0300085	-0,439421	0,059336	0,6279808
CG10338-RA	CG10338	9,33895	1,04736	1,33497	1,74285	0,73598	1,24078	-0,029665	0,895745	0,6279808
CG10339-RA	CG10339	3,28191	4,5007	4,49869	9,5874	27765,7	2,89687	0,118973	0,631541	0,6279808
CG10340-RA	bcd	0,344943	0,209465	0,0154097	0,0511772	18,4337	0,446023	-0,199657	0,520721	0,6279808
CG10341-RA	bcd	0,443793	0,257242	0,0498717	0,0285552	1,32788	0,255794	0,126112	0,674934	0,6279808
CG10341-RB	bcd	0,0266578	0,0242818	0,0513243	0,0282855	61,6585	0,0288749	0,126112	0,674934	0,6279808
CG10342-RA	bcd	0,0267333	0,0243505	1,25843	0,669061	0,149495	0,0289619	-0,150784	0,629847	0,6279808
CG10343-RA	bcd	0,0264975	0,0241357	2,20781	0,0281045	0,0834492	0,0286902	0,038815	0,887374	0,6279808
CG10344-RA	CG10340	38,6759	33,6586	2,6432	3,52331	0,101708	2646,26	0,544879	0,064135	0,6279808
CG10344-RB	CG10341	9,2789	0,428679	33,6362	0	0	0	0,543202	0,064788	0,6279808
CG10345-RA	CG10341	3,44513	37,1297	0	8,01071	1,42967	3,35311	0,112027	0,627672	0,6279808
CG10346-RA	NPF	8,6942	5,75467	9,40423	8,07303	20,7491	27,1705	0,090637	0,716969	0,6279808
CG10347-RA	CG10343	12,7964	50,6259	12,0781	11,7431	74,4148	67,0836	-0,037912	0,851194	0,6279808
CG10347-RB	CG10344	0,061888	0,0563719	3,01991	1,66246	12,3961	0,630162	-0,037912	0,851194	0,6279808
CG10348-RA	CG10344	5,37779	4,84374	0,0922941	1,9211	104,445	15,9768	0,624927	0,06299	0,6279808
CG10349-RA	CG10345	8,50551	10,2061	8,38175	0,119209	2,34267	1,77151	-0,139809	0,605306	0,6279808
CG1034-RA	Grip71	1,29027	1,20229	9,78151	13,3102	5,75597	6,11998	0,730576	0,039873	0,6279808
CG1034-RD	CG10347	3,22048	20,6978	7,07178	27,5394	3,47355	3,87673	0,751984	0,034265	0,6279808
CG1034-RE	CG10347	4,00998	16,9108	7,17798	39,6523	3,93367	4,03938	0,751984	0,034265	0,6279808
CG1034-RF	CG10348	6,98919	1040,55	1,55198	1,62019	35,7062	30,8542	0,751984	0,034265	0,6279808
CG1034-RG	CG10349	0	0	0,146618	0,0211699	15,3672	0,198447	0,751984	0,034265	0,6279808
CG10352-RA	CG10352	0,823376	13,5738	35,3276	0,928988	21,8788	14,2624	0,870075	0,014549	0,6279808
CG10353-RC	CG10353	19,641	14,6013	9,67316	240,887	4,33049	4,84909	-1,131388	1,49E-06	0,6279808
CG10353-RD	CG10353	5,77294	7,23029	9,36283	0,0220335	26,2222	2,09308	-1,14726	7,74E-07	0,6279808
CG10354-RA	Rat1	10,6559	9,21259	15,933	17,4662	7,30737	10,5393	-0,05754	0,823212	0,6279808
CG10357-RC	CG10357	0,267634	0,16252	0	0,870316	0	0	-0,358379	0,292701	0,6279808
CG10359-RE	CG10359	21,5297	7,37059	0,228395	7,22121	1,96357	2,95083	-0,324192	0,171088	0,6279808
CG10359-RG	CG10359	7,6033	27,0525	29,8759	27,01	26,5369	24,9533	-0,320742	0,17463	0,6279808
CG10360-RA	ref(2)P	8,86167	0,0570788	0,560396	23,4513	0,0484921	0,0365466	-0,157893	0,543462	0,6279808
CG10360-RB	ref(2)P	37,9461	0,039501	4,62122	82,4187	0,0375394	0,028292	-0,157361	0,54482	0,6279808
CG10361-RA	CG10361	10,6507	11,0786	11,603	13,1702	12,8235	16,1368	-0,340414	0,251511	0,6279808
CG10362-RA	CG10362	2,81563	12,8039	3,24716	3,77139	2,42788	14,504	0,136879	0,555222	0,6279808
CG10363-RA	Tep4	5,79853	0,813331	0,521528	0,0102244	4,16378	3,25142	-1,296591	5,1E-05	0,6279808
CG10364-RA	msb1l	3,85685	0,00911962	14,6016	7,56652	0,0855284	0,0644594	0,13057	0,67637	0,6279808
CG10365-RA	CG10365	0,0409139	11,976	0,0392797	14,2838	18,5714	25,0445	0,00785	0,973326	0,6279808
CG10365-RB	CG10365	0,0391909	31,6398	0,0376256	41,0311	0,05528	4,2994	0,005258	0,982093	0,6279808
CG10365-RC	CG10365	0,0452575	0,0356978	0,0434499	0,0444157	0,0527324	0,0416623	-0,030902	0,896301	0,6279808
CG10365-RD	CG10365	43,6547	0,0412236	0,0456052	0,0523981	10,3062	0,0397423	-0,030837	0,896475	0,6279808
CG10366-RA	CG10366	5,73868	158,536	6,75456	31,5762	715,939	567,152	-0,042512	0,86087	0,6279808
CG10367-RA	Hmgcr	39,3133	39,8287	39,8719	0,0554401	23,6923	10,1961	0,415386	0,121517	0,6279808
CG10367-RB	Hmgcr	0,0174819	39,3035	0,0167837	51,4953	3,25878	28,8103	0,443687	0,098684	0,6279808
CG10369-RA	Irk3	5,1072	5,42732	13,7045	72,1886	2,13163	0,0415081	-0,27666	0,252535	0,6279808
CG10369-RB	Irk3	1,12014	0,03401	0,22656	340,28	0,453043	0,310867	-0,208315	0,358319	0,6279808
CG10370-RA	Rpt5	24,819	19,0948	33,9589	28,2618	10,259	11,1592	0,021966	0,926704	0,6279808
CG10371-RA	PTPMT1	17,9641	65,0056	23,7794	73,3664	15,9118	31,0664	0,355392	0,155162	0,6279808
CG10371-RB	PTPMT1	10,8443	7,62927	0,0765527	8,32293	16,101	0,0453413	0,341649	0,168911	0,6279808
CG10372-RA	Faf2	27,3261	1015,12	6,07999	4,90474	32,6389	33,1471	-0,260992	0,19423	0,6279808
CG10373-RA	Jwa	52,2175	24,6334	5,52952	0,0395148	35,5141	7,93616	-0,192635	0,445853	0,6279808
CG10374-RA	Lsd-1	0,037465	4,22163	7,1339	58,3113	22,1818	38,9562	-0,381339	0,195133	0,6279808
CG10374-RB	Lsd-1	0,0358783	13,4292	0,0359687	2,82972	15,4819	0,0539187	-0,378133	0,199762	0,6279808
CG10374-RC	Lsd-1	6,58132	45,1398	0,0344453	9,47356	0,0245834	23,89	-0,38542	0,190308	0,6279808
CG10375-RA	CG10375	44,8404	0,0341257	48,0769	9,96088	0,0601614	21,7646	-0,206123	0,319395	0,6279808
CG10376-RA	CG10376	9,5059	9,91774	5,39591	46,4094	6,23922	6,57753	0,11621	0,549432	0,6279808
CG10377-RA	Dsim\Hrb27C	62,7813	78,7221	98,0313	22,337	5,041	64,2918	-0,456637	0,036	0,6279808
CG10377-RB	Dsim\Hrb27C	0,0293323	0,0267179	0,0281607	4,92367	10,2072	0,0319772	-0,456317	0,036253	0,6279808
CG10377-RC	Dsim\Hrb27C	22,3939	0,0269814	0,0284385	19,2639	1,56454	25,4725	-0,456321	0,036349	0,6279808
CG10379-RA	mbc	12,2021	0,0349989	9,94159	0,0419553	3,64761	2,99566	0,141548	0,556545	0,6279808
CG10382-RA	wrapper	10,21	11,6698	37,5565	18,9227	10,7649	8,54278	-0,015305	0,941385	0,6279808
CG10383-RA	CG10383	16,9024	0,0321853	28,0823	26,0899	0,053523	0,0664986	-0,465642	0,091517	0,6279808
CG10384-RA	CG10384	2,87202	0,126483	6,17001	0,0912052	0,143047	32,6785	-0,228813	0,395486	0,6279808
CG10385-RA	msl-1	3,99063	38,5973	44,9294	31,9652	21,5862	30,8837	0,377331	0,134869	0,6279808
CG10387-RA	tos	0,855697	0,049577	16,9118	10,5081	0,0882342	0,0403382	-0,213991	0,469987	0,6279808
CG10388-RA	Ubx	0,014447	11,8909	2,40258	0	5,89491	0,257354	0,790329	0,002728	0,6279808
CG10388-RB	Ubx	5,16642	0,0131593	8,1839	0,0656501	0,0197173	0,067018	0,788648	0,0028	0,6279808
CG10388-RC	Ubx	0,0142877	3,45499	0,01387	18,9816	1,41566	9,1328	0,790329	0,002728	0,6279808
CG10388-RD	Ubx	8,75231	0,0130142	5,79721	20,2348	0	0,0144826	0,790329	0,002728	0,6279808
CG10388-RE	Ubx	0,0141319	14,1052	0,013717	5,90011	13,7398	6,97635	0,790329	0,002728	0,6279808
CG10388-RF	Ubx	7,42678	0,0128723	4,69118	10,1797	5,66577	14,4145	0,788801	0,002792	0,6279808
CG10390-RA	mia	1,02282	22,3795	0,0119227	1393,49	0,0192039	0,0130068	0,126344	0,682927	0,6279808
CG10390-RB	mia	0,0288079	9,73394	0,0133084	0,0390002	0,0179668	0,0145673	0,114568	0,710895	0,6279808
CG10391-RA	Cyp310a1	6,02897	5,71528	9,55971	6,25803	8,67134	5,98165	1,035738	0,000188	0,6279808
CG10392-RA	sxc	34,1082	23,2143	27,2852	77,0228	2,19573	1,38659	-0,168427	0,46876	0,6279808
CG10392-RB	sxc	37,3363	33,6277	32,5334	0,0281646	0,652793	0,03017	-0,168327	0,468978	0,6279808
CG10392-RC	sxc	0,0187095	0,0170419	0,0179623	47,0962	14380,6	4,54271	-0,168427	0,46876	0,6279808
CG10393-RA	amos	0	0	0,846101	0,103873	0,226633	0,106038	-0,10668	0,624213	0,6279808
CG10395-RA	CG10395	10,8383	13,3243	11,3092	16,1711	0,0400313	17,0996	-0,151569	0,522119	0,6279808
CG10396-RA	COX4L	2,81069	55,6761	68,7019	58,9829	0,0411005	32,4824	0,125889	0,714641	0,6279808
CG10399-RA	Hmgcl	3,84083	2,38393	7,63591	56,5884	4,11941	4,77543	0,08688	0,795919	0,6279808
CG10405-RB	CG10405	8,53833	3,71349	16,2469	9,62625	0,325387	21,0116	-0,480573	0,163225	0,6279808
CG10406-RA	mRpS33	37,0363	6,758	8,16359	14,8918	1,64445	25,0137	0,175048	0,553693	0,6279808
CG10407-RA	CG10407	3,33277	4,61504	2,62429	0,0268341	22,1981	0,0273933	0,111056	0,657323	0,6279808
CG10413-RA	CRAT	19,1145	19,7997	22,0212	0,03026	9,69456	12,8292	-0,165159	0,431181	0,6279808
CG10414-RA	CRAT	0,0283994	0,0258681	0,0272651	18,6224	0,0409873	0,0308905	-0,224186	0,349542	0,6279808
CG10415-RA	CG10413	19,6326	9,30032	11,8155	4,40271	2,85063	3,55675	-0,338705	0,11146	0,6279808
CG10417-RA	Atac2	3,43464	4,84562	4,73301	0,6113	0,297675	2,51051	0,070662	0,720988	0,6279808
CG10417-RB	TfIIEalpha	9,58383	123,015	12,6563	48,7007	0,0774395	15,6358	0,071189	0,719089	0,6279808
CG10418-RA	CG10417	68,6469	66,2433	61,6736	31,3424	27,5211	0,0320534	-0,21807	0,490866	0,6279808
CG10419-RA	CG10417	0,0265507	0,0241842	0,0254903	0,0194801	41,4733	0,70934	0,365125	0,270672	0,6279808
CG1041-RA	CG10418	14,8257	10,2824	28,5393	0,0366366	0,0248642	4,08841	0,293087	0,224372	0,6279808
CG1041-RB	Gem2	2,41049	0,0243801	41,3132	2,78704	1,84387	16,0209	0,289982	0,228956	0,6279808
CG10420-RA	Sil1	4,04361	2,65917	5,11812	4,10555	3,0011	12,5076	-0,08938	0,743359	0,6279808
CG10422-RA	bam	0,0339605	0,185601	80,9218	135,605	0,318653	117,02	0,083916	0,813693	0,6279808
CG10423-RA	RpS27	892,347	0,895269	4,24735	4,1037	1,34881	3,31983	-0,222298	0,370534	0,6279808
CG10424-RA	Naxd	10,9594	12,4826	14,1524	12,6711	12,115	0,905456	-0,19475	0,336841	0,6279808
CG10425-RA	CG10425	20,2695	25,3597	22,327	0,893947	18,825	20,4309	-0,04555	0,822988	0,6279808
CG10426-RA	INPP5E	3,576	3,44201	3,85335	4,51968	2,88555	28,7797	-0,211129	0,325599	0,6279808
CG10428-RA	CG10428	8,96325	10,5869	10,0975	65,8219	2,57051	3,08198	0,353693	0,118897	0,6279808
CG10431-RA	CG10431	7,49802	1,38011	4,71265	1,92195	67,9339	1,17326	0,701082	0,00751	0,6279808
CG10433-RA	CG10433	114,373	11,4419	131,137	10,7677	0	8,62762	-0,661474	0,030931	0,6279808
CG10433-RB	CG10433	0,0626508	6,07144	0,0601486	12,3179	5,14359	2,89166	-0,659171	0,030822	0,6279808
CG10435-RA	CG10435	10,6775	9,48695	11,6538	4,57623	3,32906	48,0739	-0,086096	0,680346	0,6279808

gene_id	Symbol	W1_FPKM	W2_FPKM	W3_FPKM	MCM51_FPKM	MCM52_FPKM	MCM53_FPKM	FC	p-value	p-adj
CG10436-RA	Obp69a	0,134118	1,3203	1,48494	0,191813	0	13,0189	-0,736803	0,039504	0,6279808
CG10440-RA	dos	9,55351	13,2355	8,94486	27,0624	34,9774	7,53027	0,075732	0,762891	0,6279808
CG10443-RA	dos	0,0142877	11,9149	0,013717	13,0155	8,31515	0,0150295	0,197024	0,461439	0,6279808
CG10443-RB	twz	4,88252	4,08848	5,2819	2,48047	3,43217	6,37452	0,203172	0,450347	0,6279808
CG10444-RA	Lar	3,65209	5,54961	5,18585	0,90138	4,88618	5,10198	-0,222493	0,417256	0,6279808
CG10445-RA	Lar	6,10847	7,32901	3,25232	0,459987	1,83618	0,00929903	-0,021631	0,951798	0,6279808
CG10446-RA	Dsim\GD25290	8,18328	5,9802	11,6721	0,0140929	9,97871	0	0,132913	0,564281	0,6279808
CG10447-RA	CG10445	0,191036	2,18043	2,70168	1,57794	44,0792	1,79857	0,11812	0,618192	0,6279808
CG10449-RA	Sidpn	5,78503	0,370759	0,911448	0	1,47338	1,11043	0,110689	0,573141	0,6279808
CG1044-RA	Nf-YB	17,6656	17,4293	18,1641	16,5946	15,6464	6,10132	-0,009756	0,966031	0,6279808
CG1044-RB	Catsup	44,2492	43,8472	44,5343	37,619	32,7981	38,3369	-0,012213	0,957563	0,6279808
CG10459-RA	CG10459	0,0721614	0,0657295	0,0692792	0,880394	52,115	0,0877533	-0,152791	0,653365	0,6279808
CG10460-RA	zen	0,0519465	0,0473164	4,9676	0,0518016	0,0547375	0,222313	-0,256172	0,30418	0,6279808
CG10462-RA	zen	0,0534595	0,170431	5,07059	0,0533104	8,62792	0,0650831	0,399632	0,117562	0,6279808
CG10462-RB	cer	107,915	103,538	78,451	170,244	165,942	163,606	0,398998	0,118327	0,6279808
CG10462-RC	CG10462	7,71092	1,91118	7,33594	3,51098	1,61898	1,4638	0,401356	0,115514	0,6279808
CG10463-RA	CG10462	2,03947	9,84156	4,21398	4,52074	2,81662	4,0369	0,234397	0,464115	0,6279808
CG10465-RA	CG10462	0,0211804	3,23076	0,0203345	8,34048	2,58603	2,60096	0,23975	0,202208	0,6279808
CG10466-RA	CG10463	4,35117	0,0192926	6,522	0,0221819	0,0300455	0,0226441	-0,250166	0,414842	0,6279808
CG10467-RA	CG10465	50,3689	1,79661	2,13034	1,77908	6,59508	7,55108	-0,037866	0,896017	0,6279808
CG10469-RA	CG10466	8,38358	6,33013	13,6087	10,5731	12,7643	15,6252	0,243083	0,458693	0,6279808
CG1046-RA	CG10467	3,29733	1,42232	5,44158	5,58039	134,13	92,6032	-0,018624	0,945384	0,6279808
CG1046-RB	CG10469	1,56149	2,43416	1,38078	1,5853	0,0800799	0,060353	0,086238	0,790988	0,6279808
CG10470-RA	CG10470	40,7311	25,5876	30,9037	1,44502	11,0608	18,7624	-0,06566	0,821924	0,6279808
CG10472-RA	CG10472	30,2521	12,4345	2,85751	33,1568	2,47797	3,97248	-0,284779	0,394996	0,6279808
CG10473-RA	Acn	0,0269075	17,5922	0,0258328	45,4926	3,49857	0,593351	-0,037282	0,886952	0,6279808
CG10473-RB	Acn	22,2591	0,0245092	27,85	19,3037	3,72107	2,79903	-0,139973	0,594344	0,6279808
CG10474-RA	CG10474	0,193204	4,4505	0,587378	0,0237317	0,622149	2,79924	0,094918	0,77012	0,6279808
CG10475-RA	Jon65Ai	3,05689	0	157,413	5,78473	0,951479	187,984	0,356064	0,22368	0,6279808
CG10476-RA	AANATL5	0,24692	0,0203287	33,3596	0,022302	2,99736	9,28306	-0,039118	0,754991	0,6279808
CG10477-RA	CG10477	0	13,7294	24,6234	18,7035	10,0532	14,3885	-0,480143	0,055151	0,13772387
CG10478-RA	alphaKap4	0	9,30896	92,9555	69,6603	168,837	127,742	NA	NA	0,6279808
CG10479-RA	CG10479	29,7835	11,9159	0	21,9019	0	18,9575	0,554495	0,082345	0,13772387
CG10480-RA	zen2	0,0699216	0,101248	0,142288	2,70115	3,23724	0,167317	-0,046656	0,862209	0,6279808
CG10480-RB	zen2	0,185884	0,10159	0,142768	0,159202	0	0,26091	-0,046656	0,862209	0,6279808
CG10480-RC	Rcc1	12,7296	4,89476	14,6527	18,0352	10,74	7,9605	-0,046463	0,862747	0,6279808
CG10481-RA	Rcc1	5,37373	0,0260359	9,36114	12,5917	5,88644	0,0311048	-0,024364	0,884084	0,6279808
CG10481-RB	Rcc1	0,0285836	13,8464	0,0485485	0,0569248	0,0412716	0,588829	-0,024364	0,884084	0,6279808
CG10483-RA	CG10481	0	0,0962264	4,63396	11,0703	5,58048	5,66675	-0,472165	0,032038	0,6279808
CG10484-RA	CG10481	0	0,031884	1,81064	9,46087	0,743187	0,0292008	-0,299753	0,202621	0,13772387
CG10486-RB	CG10483	8,12555	8,89466	48,7967	11,845	12,3548	10,4369	0,163937	0,420083	0,13772387
CG10488-RA	Rpn3	19,5719	13,5255	26,4918	3,28202	14,3885	44,5875	0,160862	0,621695	0,6279808
CG10488-RB	CG10486	0,0467298	0,0168417	0,654952	6,16803	61,9347	0,816552	0,160862	0,621695	0,6279808
CG10489-RA	eyg	2,09691	0,0228267	3,44514	0,303629	0,746565	0,0336404	-0,287798	0,321268	0,13772387
CG1048-RA	eyg	0,0260894	3,13975	31,1582	3,71208	3,96736	3,49555	-0,128028	0,633606	0,6279808
CG1048-RB	DNApol-epsilon58	1,43205	0,0150253	34,5144	7,24631	0,998854	0	-0,128028	0,633606	0,6279808
CG10491-RA	Pcyt1	10,0358	10,6936	11,6347	23,459	4,79278	11,8884	0,691231	0,021454	0,6279808
CG10491-RC	Pcyt1	0,0267225	0,0243407	0,0256552	0,0283585	9,15607	0,0289495	0,187226	0,52952	0,6279808
CG10492-RA	Pcyt1	0,0280855	0,0255822	0,0269638	0,0299029	0,0384118	0,030526	0,191053	0,397299	0,6279808
CG10492-RB	Pcyt1	17,9766	25,251	22,6351	13,9313	0,0405037	11,5264	0,06821	0,774135	0,6279808
CG10493-RA	vn	0,0164956	2,23637	11,079	2,85213	34,2427	78,898	-0,139954	0,645291	0,6279808
CG10494-RA	vn	8,07516	1,52487	0,021412	0,069092	0,146668	1,33484	0,107306	0,653069	0,6279808
CG10494-RB	CG10492	0,0163406	2,84999	4,63595	0,317411	4,22579	0,0172716	0,107306	0,653069	0,6279808
CG10495-RA	CG10492	6,66299	0,0148841	0,0156879	1,61205	22,9968	5,1423	-0,001931	0,994515	0,6279808
CG10496-RA	Phlpp	0,691894	0,850804	0,863537	77,6469	0,022917	1,05699	0,050905	0,836676	0,6279808
CG10497-RA	CG10494	0,0237285	0,0216135	0,0227807	0,0250014	0,232431	0,0255224	0,323034	0,146611	0,6279808
CG10497-RB	CG10494	0,0237626	0,0216446	0,0228135	0,0250394	19,8448	0,0255612	0,35375	0,10456	0,6279808
CG10497-RC	CG10495	3,37524	7,4208	8,86158	4,99441	1,59791	0,258591	0,353864	0,104396	0,6279808
CG10497-RD	TAF1C-like	5,24149	11,0088	10,8315	20,078	2,41646	0	0,323229	0,14641	0,6279808
CG10497-RE	Sdc	43,6239	3,69884	6,8755	0,0704332	6,70965	6,95097	0,309831	0,161002	0,6279808
CG10498-RA	Sdc	0,0343489	46,5403	48,4032	32,5687	0,0392221	4,19902	0,031919	0,911474	0,6279808
CG10498-RB	Sdc	14,9746	0,0312873	0,032977	0,0371321	14,3556	2,74271	0,031919	0,911474	0,6279808
CG10498-RC	Sdc	12,1333	12,9985	19,1928	22,8738	96,8191	1,07622	-0,023898	0,933591	0,6279808
CG1049-RA	Sdc	11,4184	11,8724	17,2438	21,0074	0,537711	3,93694	0,195562	0,365707	0,6279808
CG1049-RB	Cdk2	3,49909	0,0445496	0,0469554	0,0573709	1,59292	1,49769	0,202179	0,350044	0,6279808
CG1049-RC	Cdk2	2,45689	1,13336	0,0746606	5,02248	2,33529	3,02923	0,202314	0,349619	0,6279808
CG1049-RD	Cdk2	1,63845	1,34978	0,109436	0,153051	1,02992	1,63067	0,203204	0,347807	0,6279808
CG10501-RA	Dsim\amd	33,787	1718,04	3,2456	1,81915	0,832733	1,14561	0,145555	0,598306	0,6279808
CG10501-RB	Dsim\amd	37,7268	0,136891	7,40747	8,96352	2,21678	3,10447	0,16587	0,540112	0,6279808
CG10504-RA	Ilk	64,188	55,193	70,8087	60,9708	71,6463	0,025474	-0,197544	0,42296	0,6279808
CG10505-RA	CG10505	0,38582	0,0484333	0,130314	37,6021	5,38001	6,36425	0,409593	0,233582	0,6279808
CG10506-RA	GlnRS	9,81787	10,7197	58,5098	0,272901	1,00379	0,0437488	-0,085631	0,690743	0,6279808
CG10508-RC	CG10508	0,0232416	0,0211701	0,0223133	0,024954	0,0338004	0,0240702	0,140244	0,597079	0,6279808
CG10508-RF	CG10508	0,0220473	0,0200822	0,0211668	0,0235789	0,0319377	1,74477	0,025864	0,923007	0,6279808
CG10508-RG	CG10508	2,04317	3,49101	2,88466	6,16886	2,38938	5,19928	0,023754	0,930387	0,6279808
CG10508-RH	CG10508	6,08958	9,97705	7,47878	10,6426	4,83314	30,6752	0,020316	0,940343	0,6279808
CG10510-RA	CG10510	0	0,052215	0	0,0647469	0	0	-0,013096	0,914398	0,6279808
CG10512-RA	CG10512	0,0337351	0,0336112	0,0192954	0,269473	0,0623032	0,0371727	-0,533571	0,092727	0,13772387
CG10512-RB	CG10512	18,7829	0,0367963	9,81015	0,0364139	0,235167	0,0461184	-0,525828	0,097197	0,6279808
CG10512-RC	CG10512	0,0369001	0,0183067	0,139592	0,0451771	0,208276	0,0409771	-0,52631	0,096859	0,6279808
CG10512-RD	CG10512	8,92771	22,7605	21,5568	0,0401406	0,0725898	43,8305	-0,525342	0,097478	0,6279808
CG10513-RB	CG10513	0,826556	4,44405	4,08441	2,81762	8,01053	79,4359	-0,078013	0,825733	0,6279808
CG10514-RA	CG10514	3,59108	0,42073	4,3274	1,00443	2,74715	11,1808	0,502706	0,048104	0,6279808
CG10516-RA	CG10516	15,6659	16,3888	26,5809	11,1372	16,9563	25,2409	-0,200124	0,43028	0,6279808
CG10520-RB	tub	5,60755	4,76037	8,32625	23,0158	19,437	0,0199568	0,22434	0,36017	0,6279808
CG10521-RA	NetB	4,73441	0,0409803	260,37	1,26077	433,409	341,291	-0,198415	0,503316	0,6279808
CG10522-RA	sti	2,45029	2,01815	6,77142	152,673	11,0774	4305,31	-0,327854	0,288135	0,6279808
CG10523-RB	park	0,0410664	0,103413	23,3237	52,4903	60,769	0,0478235	0,186634	0,489642	0,6279808
CG10523-RC	park	0,0404134	0,0953827	0,0426036	0,0468473	0,0370408	0,0469555	0,184144	0,496134	0,6279808
CG10527-RA	CG10527	39,0078	21,2156	79,0897	47,469	96,9291	70,1086	-0,533323	0,113192	0,6279808
CG10528-RA	fs(2)ltoPP43	3,08911	2,78723	9,4836	102,17	2,97555	167,633	-0,102822	0,7168	0,6279808
CG10528-RB	fs(2)ltoPP43	4,66075	4,44433	150,988	23,0382	171,135	22,4173	0,135583	0,611462	0,6279808
CG10529-RA	Lcp65Ae	6,05696	49,6906	4,96922	8,61334	20,6873	3927,49	-1,29085	0,000158	0,6279808
CG10530-RA	Lcp65Ag1	2145,34	1270,9	1197,31	1206,15	4,57451	4,85411	-0,384227	0,256209	0,6279808
CG10531-RA	Cht9	0,52617	0,0382946	0,121088	43,722	24,0438	3,03071	0,064343	NA	0,6279808
CG10533-RA	Lcp65Af	159,952	3,7115	327,164	123,004	1,41692	13,2005	-0,216532	0,526602	0,6279808
CG10534-RA	Lcp65Ag2	2360,2	1629,79	0	0,649421	9,09028	498,259	-0,394403	0,223929	0,6279808
CG10535-RA	Elp1	6,58742	5,74772	0,0937487	39,08	23,556	34,4333	-0,034765	0,878854	0,6279808
CG10536-RA	CG46338	13,1336	9,84345	16,0169	16,0856	11,8032	13,015	-0,001941	0,993489	0,6279808
CG10536-RB	CG46338	0,0671047	0,0611236	0,0644245	0,078863	0,10682	0,0805063	0,004288	0,985609	0,6279808
CG10537-RA	Rdl	5,24439	8,3838	11,3032	0,0394674	7,98579	0,0337417	0,680355	0,001478	0,6279808
CG10537-RB	Rdl	8,60822	0,0247511	12,4348	0,0295409	33,5122	0,0402898	0,700267	0,001149	0,6279808
CG10537-RC	Rdl	3,76347	58,6173	0,0260878	65,0878	0,171521	0,0301565	0,715438	0,000917	0,6279808
CG10538-RA	CdGAPr	12,818	0,558316	0,411927	5,87509	4,09707	4,33873	0,328052	0,261733	0,6279808

gene_id	Symbol	W1_FPKM	W2_FPKM	W3_FPKM	MCM51_FPKM	MCM52_FPKM	MCM53_FPKM	FC	p-value	p-adj
CG10539-RA	S6k	55,678	33,921	4,82264	104,193	3,67734	3,57487	0,485699	0,016874	0,6279808
CG10540-RA	cpa	37,7503	37,0584	45,473	2,81821	3,14536	65,1927	0,172118	0,354443	0,6279808
CG10541-RA	Tektin-C	2,14101	9,18714	1,26652	0,0209013	0,0258434	5,77861	-0,322171	0,28907	0,6279808
CG10541-RB	Tektin-C	4,05231	1,41831	7,15759	0,0477988	5,4703	4,02813	-0,322171	0,28907	0,6279808
CG10542-RA	Bre1	9,16262	4,69038	12,2736	5,73386	0,0283109	3,82172	0,229968	0,403685	0,6279808
CG10543-RA	CG10543	3,6472	5,34	3,70038	3,01234	0,0253588	2,28143	0,230153	0,418332	0,6279808
CG10543-RB	CG10543	0,0179912	0,0163877	0,0172727	0,0187005	0,02533	0,0190902	0,298397	0,293086	0,6279808
CG10543-RC	CG10543	8,08724	8,81125	6,37983	12,5679	4,73551	4,66305	0,294934	0,298346	0,6279808
CG10545-RA	Gbeta13F	42,4151	31,4471	48,4304	31,259	0,0538784	21,7159	0,182473	0,39095	0,6279808
CG10545-RB	Gbeta13F	121,845	104,881	166,742	177,93	0,0563266	103,604	0,170664	0,424004	0,6279808
CG10545-RC	Gbeta13F	0,036352	0,0331119	0,0349001	0,0394911	0,0467398	0,040314	0,17844	0,402216	0,6279808
CG10545-RD	Gbeta13F	0,0365936	0,033332	0,035132	0,0397772	0,0640929	0,040606	0,1811	0,3952	0,6279808
CG10545-RE	Gbeta13F	0,0381133	0,0347163	0,0365911	0,0415846	660,931	0,0424511	0,178735	0,400871	0,6279808
CG10545-RF	Gbeta13F	0,0320964	0,0292356	0,0308145	0,0345069	113,868	0,035226	0,181295	0,394768	0,6279808
CG10546-RA	Cralbp	120,178	6,02308	0	0,0234197	9,10243	39,9958	0,538186	0,017369	0,6279808
CG10550-RA	CG10550	0,0365329	0,0332767	0,0350738	0,0474888	2,94183	0,0405326	-0,136951	0,664018	0,6279808
CG10550-RB	CG10550	13,284	11,0952	24,5154	0,582809	0,053781	16,8924	-0,138936	0,659461	0,6279808
CG10550-RC	CG10550	0,0429994	0,0391669	0,041282	8,20326	21,7398	0,0484783	-0,134511	0,669819	0,6279808
CG10553-RA	CG10553	0,736515	2,05842	1371,27	11,5862	23,717	16,7436	0,742252	0,037612	0,6279808
CG10555-RA	CG10555	5,54072	0,0115043	69,2599	34,708	0,0351585	75,5691	0,058921	0,816158	0,6279808
CG10559-RB	CG10559	0,450036	0,651056	8,98491	0,472671	0,789419	0,441555	0,268441	0,447	0,6279808
CG10560-RA	5-HT2A	0,285936	0,0192926	0,0203345	2,04929	35,5211	0,859714	1,917209	4,07E-08	0,6279808
CG10561-RA	5-HT2A	0,0153784	0,0140077	0,0147642	0,0315797	0,0350343	0,0322377	-0,0968	0,697485	0,6279808
CG10562-RA	5-HT2A	0,0155193	0,014136	0,312888	0	47,7468	0	-0,675935	0,058716	0,6279808
CG10563-RA	5-HT2A	1,1723	1,0021	1,13414	0,145289	0,139777	0	0,551825	0,071949	0,6279808
CG10564-RA	CG10560	3,26695	0,838586	3,2577	0,0397052	17,3161	0,395394	-0,305576	0,220171	0,6279808
CG10565-RA	Dsim\GD24185	6,34434	5,19194	8,34913	0,0350577	42,764	38,3051	-0,18697	0,507054	0,6279808
CG10566-RA	CG10562	5,55697	1,79927	16,3177	1384,06	0,0643239	3,37132	0,221838	0,296401	0,6279808
CG1056-RA	l(2)37Cd	1,48934	2,47637	0,0232909	0,0255938	8,22611	10,3882	-0,540646	0,032692	0,6279808
CG1056-RB	Ac78C	2,96521	0,0118695	2,75205	8,70334	0,0309627	0,0438343	-0,545977	0,031268	0,6279808
CG1056-RC	CG10565	36,5236	30,4161	60,5718	4,33912	48,2697	2,63259	-0,540646	0,032692	0,6279808
CG1056-RD	ICA69	6,18154	7,5655	9,36761	43,3378	0,0292001	48,193	-0,819125	0,0074	0,6279808
CG10570-RA	MED31	7,88838	10,9301	8,24514	55,3098	0	65,3697	-0,352691	0,289731	0,6279808
CG10571-RA	MED31	19,2084	10,1695	27,0388	27,8763	0	0,105345	0,397919	0,082027	0,6279808
CG10572-RA	CG10570	21,7153	48,4365	199,467	0,143705	0	0	-0,041451	0,880566	0,6279808
CG10573-RA	ara	11,2936	12,3034	10,1305	10,8386	5,57096	7,07666	0,137626	0,603423	0,6279808
CG10574-RA	Cdk8	2,43831	2,02089	3,79608	3,50927	5,12674	0,0487948	-0,211418	0,420517	0,6279808
CG10574-RB	ko	12,2249	7,77885	9,76261	16,0885	0,0259034	0,022007	-0,167926	0,518215	0,6279808
CG10575-RA	I-2	0,0432528	0,0393976	0,0415252	0,0477988	0,0255548	11,0602	-0,317235	0,269357	0,6279808
CG10576-RA	I-2	7,97038	7,67997	14,8825	12,3823	8,54073	6,92321	-0,408346	0,091734	0,6279808
CG10576-RB	Ppat-Dpck	9,99414	18,1116	15,0138	0,014012	34,0317	15,1221	-0,410055	0,090204	0,6279808
CG10578-RA	CG10576	0,0362523	0,0330211	0,0348043	21,353	0,0533311	19,138	-0,639992	0,02649	0,6279808
CG10578-RB	CG10576	102,486	86,0639	136,886	113,374	132,77	130,233	-0,642294	0,025881	0,6279808
CG10579-RA	DnaJ-1	0,0306522	0,0876077	0,0294279	0,135994	0,0189793	0,138827	0,256729	0,328408	0,6279808
CG10579-RB	DnaJ-1	104,889	0,00709607	127,46	0,00790777	5,64997	0,00807255	0,25087	0,339646	0,6279808
CG10579-RC	Eip63E	0,0186147	0,0169555	0,0178712	0,0193769	2,72361	2,79982	0,251706	0,33817	0,6279808
CG10579-RD	Eip63E	7,41812	7,95575	6,6712	10,68	0,032879	0,026529	0,243016	0,350741	0,6279808
CG10579-RE	Eip63E	0,0220571	0,0200912	0,0211762	0,0231481	6,40601	0,0250472	0,250252	0,339567	0,6279808
CG10579-RF	Eip63E	0,0230739	0,0210173	0,0221523	0,0242738	0,03575	0,0246886	0,243016	0,350741	0,6279808
CG10579-RG	Eip63E	16,9516	21,8675	12,573	17,2152	0,0324015	4,87478	0,251149	0,33922	0,6279808
CG10579-RH	Eip63E	0,0249751	0,0227491	0,0239776	0,0263934	0,0352001	1,12136	0,261537	0,319867	0,6279808
CG10579-RI	Eip63E	0,022756	0,0207277	0,0218471	0,0239213	0,033234	0,24108	0,256729	0,328408	0,6279808
CG10579-RJ	Eip63E	0,0246123	0,0224186	0,0236293	0,0259874	0,0327583	1,58326	0,251551	0,338672	0,6279808
CG10579-RK	Eip63E	0,0233099	0,0212323	0,0223789	0,0245359	6,72519	11,3728	0,251706	0,33817	0,6279808
CG1057-RA	Eip63E	0,0229936	0,0209441	0,0220752	0,0241847	6,77468	9,14196	0,351198	0,111654	0,6279808
CG1057-RB	Eip63E	8,751	12,4335	9,27976	14,8423	0,0456637	1,77013	0,363658	0,098763	0,6279808
CG10580-RA	rpk	0,833644	25,2988	3,2999	0,0290717	0	1,18589	0,164158	0,535229	0,6279808
CG10581-RA	fng	20,6403	0,0410642	0,0432818	0	0	0	0,549431	0,063494	0,6279808
CG10582-RA	CG10581	5,77599	0,00554476	0,00584419	0,0320404	26,8591	3,66114	-0,16612	0,477233	0,6279808
CG10583-RA	Sin	10,631	7,98455	6,11193	0,0214804	0,229719	0,00655888	-0,351178	0,263078	0,6279808
CG10584-RA	Sse	0,622212	0,608224	0,103037	72,9355	0,0098126	0,144184	0,097601	0,738874	0,6279808
CG10585-RA	CG10584	9,45085	8,57201	13,1872	9,36586	11,4211	11,5426	0,489522	0,088268	0,6279808
CG10586-RA	Pdss2	65,3712	0,00578457	0,00609695	0,0193542	12,0914	43,1834	0,20382	0,468249	0,6279808
CG10587-RA	Sems	0,199504	0	1,48568	0,340461	0,0677928	0,161301	0,01562	0,897983	0,6279808
CG10587-RB	CG10587	0	0	0	0	0	0	0,01562	0,897983	0,6279808
CG10588-RA	CG10587	0	0	0,0701992	0	0	0,0426024	0,308661	0,15522	0,13772387
CG10589-RA	CG10588	0,0519955	0,08525	0,0499188	0	0	0,0326367	0,044935	0,892924	0,13772387
CG1058-RA	CG10589	0,0450824	0	0	0	0	0	0,033746	0,923885	0,13772387
CG10590-RA	Karybeta3	37,2869	39,1608	0,0235241	54,2544	43,1776	0,0250472	-0,025872	0,906262	0,6279808
CG10591-RA	TM9SF3	17,344	0,45429	0,0169541	0,0359569	0,184204	0,0187235	-0,567973	0,056539	0,6279808
CG10592-RA	CG10591	181,031	0,0295757	110,083	3,28728	6,67627	64,5037	0,227812	0,485438	0,6279808
CG10593-RA	Dpse\GA10422	0,528909	0	1,75531	0,59074	0,692528	0,357889	-0,137338	0,533635	0,6279808
CG10594-RA	Acer	32,6521	10,834	10,6432	44,2604	8,08342	36,4029	0,173713	0,614439	0,6279808
CG10595-RA	spo	0,0270618	0,0246497	0,0259809	1,55735	0,0389312	0,0293409	0,119318	0,689021	0,6279808
CG10595-RB	d	0,0266901	0,0243112	0,0145608	2,11464	0,0212122	0,0159868	0,120002	0,687361	0,6279808
CG10595-RC	d	2,10058	2,45213	8,1051	13,1406	4,10743	6,09652	0,19923	0,499816	0,6279808
CG10596-RA	d	8,07007	8,27414	37,2402	11,4881	7,51706	8,79674	0,303184	0,187356	0,6279808
CG10596-RB	Msr-110	0,0257639	0,0234675	0,0247349	0,0272785	2,69388	0,0278469	0,303167	0,187391	0,6279808
CG10596-RC	Msr-110	174,899	230,084	169,238	206,818	0,0444803	119,104	0,341658	0,138958	0,6279808
CG10597-RA	Msr-110	0,024594	0,0224019	0,0236117	0,0259669	160,578	0,026508	0,060937	0,849672	0,6279808
CG10597-RB	CG10597	14,2449	0,0498782	0,0525718	21,4692	27,5257	0,063606	0,056244	0,861124	0,6279808
CG10598-RA	CG10597	0,0547589	21,282	35,7833	0,0623077	0,0843962	0,638168	-0,535678	0,102792	0,6279808
CG1059-RA	CG10598	250,609	1,03038	13,8341	411,775	11,2295	27,3504	-0,285551	0,125628	0,6279808
CG10600-RA	CG10600	0,0148764	0,0315774	865,876	0,0390233	5,29508	0,0418438	0,242673	0,473707	0,6279808
CG10600-RB	CG10600	9,49866	0,0318279	2423,83	0,0409896	1,10909	63,725	0,242673	0,473707	0,6279808
CG10601-RA	mirr	0,0230096	0,0117766	0,0220906	0,0242025	0,0327824	6,49681	0,333879	0,209616	0,6279808
CG10601-RB	mirr	11,0137	6,50452	11,1865	14,5143	5,22057	34,5564	0,211362	0,440084	0,6279808
CG10602-RA	mRpL13	36,2316	0,0282964	0,0298245	5,31402	70,4222	0,0349514	0,669879	0,014046	0,6279808
CG10602-RB	mRpL13	0,0310653	0,0276085	0,0290994	60,5077	13,3807	0,0340147	0,775707	0,004937	0,6279808
CG10602-RC	mRpL13	0,03031	0,0275747	0,0290639	20,5783	145,702	0,033078	0,77489	0,005009	0,6279808
CG10602-RD	mRpL13	0,030273	51,4816	84,5384	0,0217069	8,14072	82,1893	0,77489	0,005009	0,6279808
CG10603-RA	mRpL13	55,4076	4,10622	14,8273	8,9229	0,017956	11,9788	-0,10642	0,694818	0,6279808
CG10604-RA	bsh	0,371086	0,814299	0,469622	2,59578	0,529942	0,520485	-0,189806	0,575743	0,6279808
CG10604-RB	bsh	0,340957	0,0477796	0,302159	0,3833	0,366293	0,324125	-0,219854	0,50603	0,6279808
CG10605-RA	caup	9,89759	12,675	8,71594	1,38411	0,429584	4,41986	0,541904	0,060653	0,6279808
CG10609-RA	Orco	0,562746	1,23342	1,31691	1,09656	1,69512	0,0388921	0,138926	0,65575	0,6279808
CG10609-RB	Orco	1,1711	0,2489	0,618377	0,0426892	0,0578228	1,12883	0,16254	0,604771	0,6279808
CG10610-RA	ECSIT	5,19168	0	0	0	0,776501	29,812	-0,110727	0,691935	0,6279808
CG10612-RA	Or83a	0,363854	0,552372	0,512338	0,467594	0,0914903	0,623132	0,638427	0,054057	0,6279808
CG10616-RA	CG10616	16,1798	15,8644	20,9732	0,0276447	0,0435896	0,0328518	0,086144	0,661026	0,6279808
CG10617-RA	Syt12	3,52094	4,77948	2,34781	6,26265	2,76628	2,06145	0,307196	0,291082	0,6279808
CG10618-RB	CHKov1	19,0186	13,0216	27,3271	15,0992	10,7583	13,2357	0,726521	0,006267	0,6279808
CG10618-RC	CHKov1	0,131712	0,119973	0,126452	0,186916	0,253179	0,190811	0,723145	0,007794	0,6279808
CG10618-RD	CHKov1	4,26766	3,98695	0,0525283	5,8648	3,80386	4,38875	0,729038	0,008097	0,6279808

gene_id	Symbol	W1_FPKM	W2_FPKM	W3_FPKM	MCM51_FPKM	MCM52_FPKM	MCM53_FPKM	FC	p-value	p-adj
CG10618-RE	CHKov1	19,8757	13,735	30,2003	14,5565	9,15344	12,422	0,625679	0,016321	0,6279808
CG10618-RF	CHKov1	0,129391	0,117858	0,186335	0,182263	0,246876	0,18606	0,280461	0,236994	0,6279808
CG10619-RA	tup	18,7129	23,5384	0,0199194	11,7468	0,0463755	3361,53	0,287499	0,295621	0,6279808
CG10619-RB	tup	0,0207481	0,0188988	28,8575	0,034238	0,0451326	0,146699	0,287499	0,295621	0,6279808
CG10620-RA	Tsf2	37,3268	35,7964	39,7994	55,87	1138,25	8,11794	-0,003065	0,989416	0,6279808
CG10621-RA	CG10621	66,5873	65,0778	107,528	163,49	4,50616	181,71	-0,951647	0,000126	0,6279808
CG10622-RA	ScsbetaG	30,4695	8,51175	46,991	11,8295	53,7996	51,3954	-0,523864	0,087959	0,6279808
CG10622-RB	ScsbetaG	0,116126	56,4846	0,111488	71,5709	0,243508	0,183522	-0,603562	0,06072	0,6279808
CG10623-RA	CG10623	7,20031	23,7372	48,8117	0,0333203	0,0438897	19,4601	-0,392424	0,232639	0,6279808
CG10624-RA	sinu	55,2421	18,7163	15,249	35,7118	30,7112	0,0386685	0,316021	0,184288	0,6279808
CG10624-RB	sinu	22,4956	0,105776	29,3723	0,179776	9,6807	15,6409	0,316106	0,184142	0,6279808
CG10625-RA	frm	17,7464	0,0190846	15,4483	0,0219308	0,349333	12,6634	-0,079604	0,736471	0,6279808
CG10625-RB	frm	0,0212349	0,0193422	0,69647	16,0659	11,9692	0,728077	-0,126485	0,587831	0,6279808
CG10625-RC	frm	0,015032	0,0136922	0,0311729	0,0155166	0,68175	0,0367061	-0,126582	0,587615	0,6279808
CG10625-RD	frm	0,0176595	0,0160855	15,4453	0,0183413	0,0487038	15,2987	-0,080544	0,733776	0,6279808
CG10625-RE	frm	0,0254562	0,0231873	131,637	0,0269328	14,7266	0,996865	-0,127261	0,585902	0,6279808
CG10625-RG	frm	83,4532	75,8632	0,888756	67,4543	33,5236	38,1575	-0,815101	0,005604	0,6279808
CG10625-RH	frm	0,0181046	0,0164909	51,6045	0,0188234	139,089	263,684	-0,124463	0,592107	0,6279808
CG10625-RI	frm	16,3563	26,1657	0,0201152	30,5164	0,0369488	0,0223878	-0,078496	0,738657	0,6279808
CG10625-RJ	frm	0,107324	0,0977579	0,0203867	0,141241	108,402	12,3513	-0,078496	0,738657	0,6279808
CG10625-RK	frm	0,165327	0,150591	0,0144317	0,263019	0,0351724	0,01584	-0,826076	0,004543	0,6279808
CG10626-RA	Lkr	2,64619	2,19494	255,022	118,831	320,589	286,047	0,194695	0,534281	0,6279808
CG10627-RA	nst	38,4014	51,923	45,3221	40,388	36,5924	185,029	0,439478	0,042885	0,6279808
CG10628-RA	CG10628	7,16612	7,84728	11,104	2,2035	5,32834	18,6634	0,396858	0,089386	0,6279808
CG10630-RA	Itp-r83A	0,00685885	6,46664	0,0153761	0,0390233	0,0178055	0,0169155	-1,610589	7,29E-10	0,6279808
CG10631-RA	Itp-r83A	5,19018	989,714	0,0263807	0,0316708	0,0179741	0,0298223	0,156911	0,440844	0,6279808
CG10632-RC	blanks	1,81176	9,36315	15,936	2,76644	58,7941	7,97069	0,061567	0,78615	0,6279808
CG10632-RD	CG10631	2,33196	2,09924	2,17853	8,0398	2,63164	3,43612	-0,127783	0,565168	0,6279808
CG10632-RE	sowah	4,16758	0,0271029	6,6765	11,5207	4,65343	6,1416	0,012405	0,955918	0,6279808
CG10632-RF	sowah	9,09014	13,2225	9,61264	0,0316252	0,0428365	0,0322842	0,061567	0,78615	0,6279808
CG10633-RA	sowah	0,029595	0,0244197	0,028413	0,0284565	0,0385445	0,0290495	NA	NA	0,6279808
CG10635-RA	sowah	0,0268092	10,8681	0,0257385	26,9041	32,1082	0,0247068	-0,52099	0,088944	0,6279808
CG10637-RA	Ir64a	0	0	0	0	0	0	-0,076322	0,783455	0,6279808
CG10637-RB	Pfdn4	111,679	161,278	2,48645	181,538	0,14314	0,107879	0,027426	0,922357	0,13772387
CG10637-RC	Nak	11,6273	16,5278	4,67369	33,0981	37,8891	4,38287	-0,051066	0,843375	0,6279808
CG10638-RA	Nak	0,0129063	0,0117559	4,3217	3,96147	0,145361	0,0253588	0,640908	0,024662	0,6279808
CG10638-RB	Nak	6,40247	6,90744	42,762	39,4891	0,656492	42,0437	0,626889	0,027707	0,6279808
CG10639-RA	CG10638	32,3055	26,1566	43,5818	15,5698	6,33683	124,152	-0,24342	0,278293	0,6279808
CG1063-RA	CG10638	0,0692134	0,0630444	0,066449	0,0818001	4,76797	2,54102	-0,17866	0,5584	0,6279808
CG1063-RB	L2HGDH	29,0136	32,0373	0,0123908	0,648919	41,1894	0,0135327	-0,172158	0,573414	0,6279808
CG10640-RA	Snr1	22,6644	6,11666	0,033529	7,50873	5,88123	0,0398129	0,106849	0,606002	0,6279808
CG10640-RB	Uev1A	60,6999	55,7895	1,63027	1,35372	0,101477	0,501305	0,107255	0,604663	0,6279808
CG10641-RA	Uev1A	0,056155	0,0511499	0,524185	0,0749177	3,49004	0,0764788	0,263468	0,246442	0,6279808
CG10642-RA	Swip-1	80,4079	9,92585	73,3869	1,32175	0,028284	2,87883	-0,202299	0,330618	0,6279808
CG10645-RA	Klp64D	12,2633	10,7856	15,6565	16,592	12,5901	0,0236388	-0,434677	0,09274	0,6279808
CG10645-RB	lama	0,0220645	1,49729	0,0211832	0,0231563	0,0313653	5,12371	-0,451726	0,082576	0,6279808
CG10645-RC	lama	4,83647	5,66114	7,12768	10,21	5,24597	3,80713	-0,435538	0,092368	0,6279808
CG10646-RA	lama	3,34448	2,92383	4,89939	11,5944	4,40549	13,2431	0,229556	0,363423	0,6279808
CG10648-RA	CG10646	5,20353	5,88536	12,3036	4,40948	35,03	10,9133	-0,034291	0,910337	0,6279808
CG1064-RA	Rbm13	16,1525	9,75288	2,07096	4,81925	16,508	19,6622	-0,08181	0,738681	0,6279808
CG10650-RA	Dpse\GA23751	87,6548	87,5004	3,60735	95,1766	0,333484	8,16058	-0,618344	0,026544	0,6279808
CG10651-RA	CG10650	36,5407	29,5615	2,29833	89,5727	3,22007	65,2402	NA	NA	0,6279808
CG10652-RA	CG10651	0	15,9893	37,3665	7,12409	4,1011	5,51532	-0,546657	0,055019	0,6279808
CG10652-RB	RpL30	1525,53	6,74137	4,84897	4,76163	2,29641	2,09853	-0,546657	0,055019	0,13772387
CG10653-RA	RpL30	0,150286	5,86681	5,05248	5,27597	0,0240599	4,67553	0,127685	0,558361	0,6279808
CG10654-RA	hook	11,6914	8,28834	7,41976	8,46708	4,88495	5,19636	0,197684	0,405068	0,6279808
CG10655-RA	CG10654	11,4185	216,588	10,9624	16,303	22,4612	0,0533203	-0,159026	0,554117	0,6279808
CG10657-RA	l(2)37Bb	18,3759	30,996	68,8289	30,8759	33,7335	3,93378	0,45213	0,045251	0,6279808
CG10658-RA	CG10657	164,534	55,2085	4,7258	6,81165	2,67143	1302,76	-0,816645	0,004663	0,6279808
CG10659-RA	Dsim\Hf	7,78913	5,68961	0,0165257	46,5976	15,9542	11,4492	-1,874084	1,24E-07	0,6279808
CG1065-RA	AANATL3	1,62967	261,333	2,8976	314,715	4,86384	0,858842	-0,24412	0,389086	0,6279808
CG10660-RA	Shab	0,0186831	1,4697	2,33529	0,018595	4,95275	2,38158	0,737056	0,005131	0,6279808
CG10663-RA	Shab	0,0182194	2,51003	0,919083	0	0,025187	0,648327	-0,10802	0,67694	0,6279808
CG10663-RB	Shab	2,4973	0	0	1,2443	0	0	-0,117778	0,657465	0,6279808
CG10663-RC	Shab	1,27046	0,522603	0,367217	2,38926	1,09506	0,310769	-0,113362	0,669783	0,6279808
CG10664-RA	CG10660	26,0241	46,8854	30,5225	0,0321812	2,29614	5,8628	-0,233096	0,441941	0,6279808
CG10664-RB	CG10663	0,030752	1,19047	0,0295238	25,1045	19,1782	23,3771	-0,233096	0,441941	0,6279808
CG10667-RA	CG10663	4,29227	2,29094	3,4268	0,0329537	0,110799	0,0835046	0,001913	0,994202	0,6279808
CG10669-RA	CG10663	0,03008	0,027399	0,0288786	4,13731	100,489	7,00197	0,034834	0,885189	0,6279808
CG1066-RA	Dpse\GA10478	0,0893317	1,39446	0	6,68372	29,6168	0	-0,517207	0,047614	0,6279808
CG1066-RB	Dpse\GA10478	261,989	0,0813695	2,14688	37,0345	0,0387453	0,363566	-0,512412	0,049965	0,6279808
CG1066-RC	Orc1	3,25821	3,41541	3,66139	27,0866	24,9169	2,65835	-0,44527	0,087984	0,6279808
CG1066-RD	CG10669	2,73425	0,168197	2,58354	3,73068	2,33886	0,187557	-0,397282	0,132716	0,6279808
CG10670-RA	Gen	0,0301075	0,027424	0,028905	51,6028	0,0436323	0,0328839	0,066385	0,848967	0,6279808
CG10671-RA	Fitm	20,6377	25,8376	25,9514	0,036434	13,8675	17,2681	0,27859	0,14804	0,6279808
CG10671-RB	Fitm	0,0328572	0,0299286	0,0315449	0,0322127	0,0493501	0,0371932	0,27859	0,14804	0,6279808
CG10672-RA	CG10672	19,0579	882,644	33,5031	18,9116	38,5164	37,2368	-0,461891	0,159735	0,6279808
CG10673-RA	Prpk	7,24387	8,55986	12,1235	20,9486	164,47	13,3424	-0,202465	0,494729	0,6279808
CG10674-RA	CG10674	40,6155	349,973	80,2175	2029,57	18,6061	23,9665	-0,387463	0,223935	0,6279808
CG10675-RA	CHKov2	3,99634	4,96383	4,70871	4,84563	0,118873	2,14505	0,25996	0,282275	0,6279808
CG10679-RA	Nedd8	17,102	11,5652	12,8325	2,77747	18,1542	985,344	-0,020254	0,946458	0,6279808
CG10680-RA	CG10680	0,279122	0,494232	0	0,356145	0,4824	0	0,05549	0,855874	0,6279808
CG10681-RA	CG10681	11,429	8,84023	159,6	8,4379	7,53598	45,3139	0,623124	0,006185	0,6279808
CG10682-RA	vih	48,0163	17,9983	57,3479	67,6192	20,2946	0,0893859	0,109384	0,589492	0,6279808
CG10683-RA	rhi	0,568531	0	83,9236	106,084	67,8675	70,0305	0,240287	0,496546	0,6279808
CG10685-RA	l(2)37Cg	22,6487	100,891	147,224	0,0594411	0,0805133	0,0606797	-0,184655	0,567223	0,6279808
CG10686-RA	tral	34,6173	42,3751	47,5634	52,7303	0,544418	25,2434	0,25885	0,297708	0,6279808
CG10687-RA	AsnRS	25,1877	27,446	0,144283	0,226805	0,307209	0,231532	-0,355276	0,130014	0,6279808
CG10688-RA	Pmm2	26,1602	18,587	31,6577	24,1151	0,118602	32,114	-0,224354	0,431784	0,6279808
CG10689-RA	l(2)37Cb	2,62412	1,57166	44,7659	43,0802	0,0346669	0,0261271	-0,314036	0,201181	0,6279808
CG10691-RA	l(2)37Cc	0,0525384	12,3969	43,4881	124,517	136,839	149,237	-0,336638	0,194661	0,6279808
CG10691-RB	l(2)37Cc	82,1473	35,356	2668,65	3098,1	4573,48	4522,06	-0,336447	0,194918	0,6279808
CG10692-RA	Mt2	0,0656382	0,0597878	0,0630166	0,682809	0,386255	0,030306	-0,151491	0,667294	0,6279808
CG10692-RC	Mt2	2,04698	0,721755	3,20142	0,0768397	0,10408	0,0784409	-0,157823	0,654382	0,6279808
CG10693-RA	slo	0,0144029	0,0130509	0,013614	5,70423	0,0670795	14,0639	-0,141986	0,509719	0,6279808
CG10693-RB	slo	2,57813	2,68849	0,013658	0,0271324	15,9221	0,0910803	-0,097036	0,651679	0,6279808
CG10693-RC	slo	0,014328	0,0129582	2,73843	0,02352	0,455728	3,01719	-0,084577	0,694596	0,6279808
CG10693-RD	slo	0,014328	0,0129582	0,013658	86,7858	6,77235	0,347394	-0,019299	0,929861	0,6279808
CG10693-RE	slo	3,01604	0,0128585	0,013658	20,3202	0,112051	24,2922	-0,095541	0,661154	0,6279808
CG10693-RF	slo	0,0142262	0,0129165	0,0134409	0,0892212	0,232044	0,0276978	-0,138504	0,520081	0,6279808
CG10693-RH	slo	0,0142262	0,0129582	0,0201999	0,496403	0,0337621	0,0240101	-0,129125	0,549609	0,6279808
CG10693-RI	slo	0,0211751	2,9739	0,0133308	38,6731	5,79819	121,646	-0,105578	0,622821	0,6279808
CG10693-RJ	slo	0,0141804	0,0129582	22,0583	0,287719	3,77808	15,6484	-0,143433	0,505317	0,6279808
CG10693-RK	slo	0,0142262	0,0129582	0,580923	26,1031	0,0201082	6,22788	-0,14257	0,50857	0,6279808

gene_id	Symbol	W1_FPKM	W2_FPKM	W3_FPKM	MCM51_FPKM	MCM52_FPKM	MCM53_FPKM	FC	p-value	p-adj
CG10693-RL	slo	3,49964	0,0127523	0,0159884	0,0495232	0,0200001	0,783428	-0,144841	0,50184	0,6279808
CG10693-RM	slo	0,0213393	1,26489	19,3781	9,9231	0,0200001	0,0505552	-0,141415	0,510876	0,6279808
CG10693-RN	slo	0,0142262	0,0126478	3,52561	40,7722	2,64572	10,3148	-0,106401	0,619681	0,6279808
CG10693-RO	slo	0,0140001	2,82696	0,0428723	0,0172558	0,0198532	51,1787	-0,129681	0,54855	0,6279808
CG10693-RP	slo	0,0210403	14,614	20,6688	20,4595	0,0198532	0,395202	-0,083555	0,702465	0,6279808
CG10693-RQ	slo	0,0138854	12,7795	12,375	6,70737	0,0196955	0,144308	-0,083068	0,70369	0,6279808
CG10694-RA	CG10694	0,10239	0,0932642	16,315	42,0964	6,70585	0	0,094649	0,68059	0,6279808
CG10695-RA	Pat1	13,443	12,9984	17,3077	19,51	9,01118	9,98091	0,12223	0,593243	0,6279808
CG10697-RB	Ddc	0,0242599	0,0220976	2,82484	4,69942	8,35734	0,0357882	-0,2359	0,332767	0,13772387
CG10697-RC	Ddc	35,2314	34,8391	40,4422	60,984	55,0217	74,7443	-0,240871	0,324622	0,6279808
CG10697-RD	Ddc	0,0325711	0,029668	43,5366	44,5632	86,8416	35,936	-0,25016	0,30963	0,6279808
CG10698-RA	CrzR	2,66303	2,96471	15,0158	30,0684	0,0247128	7,93297	0,04138	0,878201	0,6279808
CG10699-RA	Lim3	4,20058	0,112428	0,197499	0,164586	2,49392	0	0,452617	0,046738	0,6279808
CG10699-RB	Lim3	6,33616	38,0912	37,5217	48,1287	18,6384	28,7467	0,445611	0,05027	0,6279808
CG10700-RA	Alh	15,2742	0,0390905	0,0413089	11,894	10,2407	2,08094	0,497425	0,164278	0,6279808
CG10701-RA	Alh	0,0124421	0,0391924	6,26536	1,94579	0,0565766	8,57722	-0,11443	0,551035	0,6279808
CG10701-RB	Alh	0,0172177	0	7,91067	0,175001	12,3293	0,262951	-0,114164	0,551758	0,6279808
CG10701-RC	Alh	6,09691	4,05792	10,069	0,132975	7,8971	9,11992	-0,083516	0,66274	0,6279808
CG10701-RD	Alh	5,24086	6,2866	0,0119452	7,75004	0,698172	10,3153	-0,146076	0,449335	0,6279808
CG10701-RE	Alh	0,0133012	7,61908	0,01653	0,0417691	2,7235	4,59189	-0,133452	0,487289	0,6279808
CG10701-RF	CG10700	0,265119	0,371522	0,332847	0,0169191	19,3776	25,8739	-0,133068	0,488498	0,6279808
CG10701-RG	Moe	65,4903	0,0191392	19,5044	7,12965	6,6142	0,0364673	-0,133474	0,487236	0,6279808
CG10701-RH	Moe	0,0284851	0	63,5083	0,0344168	32,4969	0,0309901	-0,133076	0,488494	0,6279808
CG10701-RI	Moe	0,0273191	7,33205	10,6898	103,285	3,1292	0,0296383	-0,143395	0,456969	0,6279808
CG10701-RJ	Moe	0,0279785	0,046061	71,9112	50,8145	3,05143	37,4604	-0,146076	0,449335	0,6279808
CG10702-RA	Moe	34,152	0,641523	0,0273474	9,7453	6,30397	35,4918	0,059393	0,762423	0,6279808
CG10702-RB	Moe	38,8272	0,171471	0,026228	6,45277	7,17002	82,7683	0,072763	0,70926	0,6279808
CG10702-RC	Moe	0,0293714	1,40985	0,026861	7,66079	0,0466178	0,0320229	0,081581	0,675632	0,6279808
CG10703-RA	Moe	0,0290741	0,0356344	51,8513	22,6349	87,6854	0,031676	-0,053892	0,80476	0,6279808
CG10704-RA	Moe	25,7768	2,78226	77,8876	4,74637	4,01967	0,0327877	0,330098	0,351883	0,6279808
CG10706-RA	Moe	0,0295025	5,65213	0,0281983	12,0755	8,80071	0,0321761	0,598705	0,012645	0,6279808
CG10706-RC	CG10702	3,45564	4,22052	5,52106	9,10486	2,84558	2,7719	0,269605	0,269776	0,6279808
CG10706-RD	CG10702	0,017124	0,0155977	0,0164401	7,86961	3,11377	0,018133	0,206244	0,39208	0,6279808
CG10706-RE	CG10702	2,37447	1,80971	1,9152	35,0855	0	1,93713	0,208193	0,38904	0,6279808
CG10706-RF	GCC88	7,55966	2,28126	3,63227	3,88234	6,44593	3,21614	0,241906	0,333227	0,6279808
CG10706-RG	toe	2,06315	5,16171	0,174597	4,24207	3,33735	0,294572	0,167897	0,484976	0,6279808
CG10706-RH	SK	3,58607	3,88698	3,77895	3,644	12,105	8,89273	0,169002	0,494405	0,6279808
CG1070-RA	SK	0,0139146	0,0126744	0,0133589	2,0492	0	12,8347	0,214486	0,531725	0,6279808
CG1070-RB	SK	1,31493	1,55261	0,0133589	0,0143258	0,247222	1,39275	0,22547	0,517986	0,6279808
CG1070-RC	SK	0,019311	0,0175898	0,0185397	2,58205	12,2827	0,0146243	0,185193	0,592962	0,6279808
CG1070-RD	SK	1,78903	1,88148	1,67608	0,0201348	3,1856	1,15602	0,215937	0,528915	0,6279808
CG1070-RE	SK	0,0180502	0,0164414	0,0173293	1,45026	0,0517539	0,0205544	0,184085	0,595158	0,6279808
CG1070-RF	SK	1,55497	1,21989	2,45935	0,0187644	0,432065	1,01562	0,207785	0,551262	0,6279808
CG10710-RA	E2f2	15,7616	12,3372	20,4642	0,0133803	0,019138	0,0136591	0,615309	0,046421	0,6279808
CG10711-RA	CG10710	11,0141	19,7264	2986,17	15,1227	20,5288	2,22066	-0,242603	0,344763	0,6279808
CG10712-RA	Vps36	9,85472	12,8383	11,6799	18,3576	6,11789	1,12507	0,173007	0,502512	0,6279808
CG10712-RB	Chro	0,0174958	0,0159364	0,016797	0,0521493	0,0706365	0,0532359	0,166732	0,515017	0,6279808
CG10712-RC	Chro	0,0174588	0,0159027	0,0167615	12,3917	16,1816	13,998	0,170969	0,506351	0,6279808
CG10713-RA	Chro	0,017783	0,016198	0,0170728	0,0179223	0,0242759	0,0182958	-0,097016	0,710994	0,6279808
CG10717-RA	CG10713	1,95642	14,8469	16,9682	32,1671	3,98322	49,4746	-0,009114	0,977797	0,6279808
CG10717-RB	ImpL1	344,51	0,219059	0,346334	2,97644	1,64316	1,69902	-0,009065	0,977919	0,6279808
CG10718-RA	ImpL1	0,0569292	1870,25	0,0682359	23,274	0,353283	1,78807	0,612736	0,035146	0,6279808
CG10719-RA	neb	4,82587	0	5,73989	6,18917	2,50481	2,52969	-0,325942	0,231262	0,6279808
CG10719-RB	Dsim\GD24188	2,40475	0,178536	0,334538	0,441942	0,101844	88,0039	-0,344132	0,206946	0,6279808
CG10719-RC	Dsim\GD24188	2,2749	4,30371	4,81209	5,01495	2,85406	0,0104278	-0,321571	0,240079	0,6279808
CG1071-RA	Dsim\GD24188	5,22451	2,17312	6,49486	0	0	0,0104278	0,014864	0,948334	0,6279808
CG10721-RA	Awh	0,032523	6,84144	0,031224	17,0005	0,0126929	6,47273	0,072513	0,765417	0,6279808
CG10722-RA	Awh	11,3903	0,0160212	9,55882	0,0178979	2,20287	0,0182709	0,012249	0,971806	0,6279808
CG10722-RB	Pyroxd1	4,91262	6,08651	22,8155	18,2641	37,93	34,5644	0,012249	0,971806	0,6279808
CG10723-RA	nesd	0,0316354	0,566203	2,23007	0,0339732	0,518978	0,0346811	0,020083	0,951575	0,6279808
CG10724-RA	nesd	0,785189	6,14175	6,63873	1,39899	2,50179	1,2871	0,052861	0,81454	0,6279808
CG10724-RB	Kua	1,66429	1,93526	0,410594	0,237864	0,0460169	1,8855	0,054021	0,810191	0,6279808
CG10725-RB	flr	15,8416	2,64982	13,1876	191,411	2,39963	16,9164	-0,450543	0,16628	0,6279808
CG10726-RA	flr	26,367	0,0885964	1,95994	4,67427	10,9113	5,73347	0,043654	0,868105	0,6279808
CG10726-RB	CG10725	10,1655	23,5855	6,84748	15,0858	143,832	3,5737	0,045727	0,861941	0,6279808
CG10728-RA	barr	0,0251462	1,43156	9,24819	2,69086	5,77569	7,33653	-0,025595	0,919715	0,6279808
CG1072-RA	barr	6,55871	4,12584	12,0016	0,0178591	0,121386	46,6153	0,394547	0,089738	0,6279808
CG1072-RB	vls	6,77207	9,99387	567,664	9,09449	493,808	537,696	0,390578	0,091861	0,6279808
CG10730-RA	CG10730	0,736723	0,745621	0,958782	2,61574	1,23849	1,12546	-0,85539	0,00348	0,6279808
CG10732-RA	cmb	3,90599	1,20671	0,704717	6,66449	0,700647	0,0202817	0,302452	0,278161	0,6279808
CG10732-RB	cmb	7,15361	1,01314	0,951804	1,3899	0,608185	1,06016	0,393651	0,152333	0,6279808
CG10733-RA	loj	0,0663633	5,46446	4,61672	0,0778382	2,12756	0,0794602	0,310429	0,224495	0,6279808
CG10733-RB	loj	19,6339	0,0282875	3,27967	59,0135	1,27973	20,149	0,310546	0,224267	0,6279808
CG10733-RC	loj	86,877	0,0291648	0,0307398	22,9435	0,0466178	40,5836	0,310339	0,224748	0,6279808
CG10734-RA	CG10734	0,405063	0,0297119	5,3806	0,193162	3,56348	6,432	0,460631	0,197503	0,6279808
CG10737-RA	CG10737	0,0223179	0,0201046	0,15104	0,0222493	1,14875	0,0236471	-0,496559	0,010649	0,6279808
CG10737-RB	CG10737	0,0220719	0,0205719	0,0214265	0,022152	15,8661	0,0242263	-0,2977	0,125845	0,6279808
CG10737-RC	CG10737	0,0225849	0,019392	0,0211903	0,0204727	22,8163	0,0227667	-0,484916	0,01306	0,6279808
CG10737-RD	CG10737	0,0212896	0,0193484	0,0216828	10,8642	9,03677	0,0227129	-0,484548	0,013063	0,6279808
CG10737-RE	CG10737	0,0212417	0,0192679	0,0204393	0,0248318	440,798	0,0226136	-0,300872	0,12363	0,6279808
CG10737-RN	CG10737	0,0211533	0,0178719	0,0203933	0,0220409	8,29548	0,0208993	-0,486418	0,012816	0,6279808
CG10737-RO	CG10737	0,0196207	8,13691	0,0203084	0,0216072	0,0469655	7,87373	-0,299256	0,125213	0,6279808
CG10737-RP	CG10737	9,62029	0,0214747	0,018837	37,2451	1,18772	0,0253492	-0,485219	0,013048	0,6279808
CG10737-RQ	CG10737	0,0235761	0,0191758	9,72168	0,668925	11,6233	0,0225002	-0,480533	0,014338	0,6279808
CG10737-RR	CG10737	0,0210522	0,0188161	0,0226344	0,320126	11,992	0,0220575	-0,483325	0,013702	0,6279808
CG10737-RT	CG10737	0,0206573	38,5995	0,0202114	6,04188	0,0313764	26,115	-0,485041	0,012978	0,6279808
CG10737-RU	CG10737	32,4594	3,87868	0,0198322	1,35886	0,0321448	0,752673	-0,484784	0,012237	0,6279808
CG10738-RA	CG10738	0,305053	0,051855	3,7815	1,58926	2,03153	2,76235	-0,08001	0,749891	0,6279808
CG10738-RB	CG10738	1,02649	0,0173665	6,70864	7,33204	4,30682	4,43216	-0,040556	0,861934	0,6279808
CG10739-RA	pigeon	6,58894	0,162373	0,177362	0	0	294,427	0,169853	0,430377	0,6279808
CG10741-RA	CG1074	13,7904	0,165498	0,0138549	20,1904	0,0256135	20,0417	0,424008	0,107923	0,6279808
CG10741-RB	Tgi	9,33366	1,22144	1,14055	0,922629	1,06475	6,44645	0,349938	0,191537	0,6279808
CG10742-RA	Tgi	10,1353	10,2106	10,7314	2,62567	6,22454	0,620347	-0,069759	0,750591	0,6279808
CG10743-RA	Tsp3A	5,32958	0,248165	0,220267	0,2141	0,169919	6,04209	0,272207	0,167617	0,6279808
CG10743-RB	Liprin-beta	0,0234895	0,0213958	21,1645	25,7705	0,0283469	13,648	0,272441	0,167866	0,6279808
CG10746-RA	Liprin-beta	17,2763	19,0993	10,8324	19,8171	14,627	6,06626	0,422686	0,156068	0,6279808
CG10747-RA	fok	460,605	1,1486	334,606	624,025	294,353	293,712	0,140036	0,520006	0,6279808
CG10748-RA	CG10747	8,38963	0	8,78289	8,76422	7,8826	6,94573	NA	NA	0,6279808
CG10749-RA	CG10748	0	0	0	0	0	0	NA	NA	0,6279808
CG1074-RA	CG10749	0	0	0,053669	0	0	0	-0,080584	0,690267	0,6279808
CG10750-RA	CR1075	0	1,19734	1,03765	0	0	0,543322	0,059801	0,854082	0,6279808
CG10751-RA	CG10750	0,264539	0,214187	3,18985	15,0842	0,0175984	0,00970698	-0,366649	0,236883	0,6279808
CG10752-RA	robl	165,066	314,446	0,218559	0,325572	0,0298745	258,496	0,16849	0,409914	0,13772387
CG10753-RA	CG10752	0,0976299	3,73491	4,5107	2,0762	20,4284	0,043607	-0,601578	0,043941	0,13772387

gene_id	Symbol	W1_FPKM	W2_FPKM	W3_FPKM	MCM51_FPKM	MCM52_FPKM	MCM53_FPKM	FC	p-value	p-adj
CG10754-RA	SmD1	16,5868	6,86701	25,3834	7,5492	7,98175	6,99302	-0,210773	0,423162	0,13772387
CG10755-RA	Sf3a2	18,4764	19,08	32,0736	28,3919	2,50701	33,1241	0,0531	0,869707	0,6279808
CG10756-RA	Cyp4ae1	1,39071	3,80903	18,9993	2,95037	512,206	1,32693	0,020531	0,91615	0,6279808
CG10757-RA	Taf13	34,0918	32,7289	15,4914	7,94138	8,81438	10,0178	0,122134	0,659069	0,13772387
CG10757-RB	mRpS18B	0,094304	0,292167	2,34573	0	0,815918	0	0,129342	0,641311	0,6279808
CG10759-RA	mRpS18B	21,1723	0,0288157	0,0303719	1,08238	0	0	-0,490132	0,170866	0,6279808
CG1075-RA	Or7a	0,133003	0,290756	0,306458	4,28487	0,0709465	21,5483	0,01562	0,897983	0,6279808
CG10761-RA	Vha14-2	0,474986	0	0,120145	1,86267	0,21961	5,29785	-0,062413	0,651227	0,6279808
CG10763-RA	CG10761	0	0,026413	0,115592	0,048026	2,34172	0	-0,056432	0,82572	0,6279808
CG10764-RA	Gbeta5	23,8313	4,95233	1,69346	23,9059	3,95116	28,9763	0,523646	0,143453	0,6279808
CG1076-RA	CG10764	0,549978	1,2345	58,4937	0,0271101	4,86201	0,0221738	0,590389	0,062028	0,6279808
CG10772-RA	CG1077	0,946855	1,47007	11,7346	3,21927	1,23489	1,63555	-0,052906	0,834203	0,6279808
CG10772-RB	Fur1	9,54522	7,68873	28,4074	5,00283	0,717624	0,249731	-0,112018	0,637311	0,13772387
CG10772-RC	Fur1	4,00131	0,292736	0,0356053	11,3581	0,271098	1,25368	-0,108903	0,640196	0,6279808
CG10772-RD	Fur1	0,0182345	0,852863	0,0348617	0,483444	0,468546	8,22315	-0,108903	0,640196	0,6279808
CG10772-RE	Fur1	0,0181295	0,028208	0,0350738	1,0231	0,426218	0,16072	-0,108903	0,640196	0,6279808
CG10772-RF	Fur1	2,5918	9,64434	0,027102	15,058	1,26261	16,0077	-0,108903	0,640196	0,6279808
CG10776-RA	Fur1	0,0176218	9,05271	10,8446	18,154	9,10537	7,31452	0,350074	0,123945	0,6279808
CG10777-RB	wit	5,28205	5,16524	0,85026	5,60905	2,75664	3,12705	0,060198	0,830722	0,6279808
CG10778-RA	mahe	7,54206	8,69109	7,71327	35,2252	47,776	5,19196	-0,462026	0,045789	0,6279808
CG1077-RA	CG10778	19,0914	24,4881	25,6168	12,8256	34,3997	25,3022	-0,165304	0,6143	0,6279808
CG10781-RA	Fip1	8,20242	8,94065	0	0,015887	0,021519	0	-0,092203	0,455035	0,6279808
CG10788-RB	ng1	0	0,272091	0,121294	0,983447	0	0	0,064159	0,724189	0,6279808
CG10789-RA	ng3	0,166858	0,254034	0,26699	0,406636	0	0	0,215167	0,250985	0,6279808
CG1078-RA	ng4	0,416445	0,238317	0,399812	0	0	0	-0,067137	0,791853	0,6279808
CG10790-RB	Fie	10,407	0,0602668	147,97	6,45903	4,17704	4,02216	-0,102041	0,773993	0,6279808
CG10793-RA	Pig1	3,08988	0,0668739	15,5626	14,4396	5,30271	6,8501	0,092697	0,795446	0,13772387
CG10794-RA	CG10793	0,537688	0,0529345	0,439737	0,542869	0,245166	0,380004	-0,514427	0,150568	0,13772387
CG10795-RA	DptB	1,09639	31,6189	61,8288	53,8638	66,3935	68,2385	0,532185	0,036034	0,13772387
CG10798-RA	CG10795	6,35587	14,1251	9,42489	1,68187	1,9415	1,27604	0,094811	0,6816	0,6279808
CG10799-RA	Myc	14,6073	0,401465	0,528932	0,947849	2,78449	2,43996	0,46979	0,087596	0,6279808
CG1079-RA	CG10799	13,1172	0	15,8853	0,0649233	12,4067	11,2535	0,107385	0,674234	0,6279808
CG10800-RA	Rca1	7,53086	0	0,0592497	0	4,65304	0,128939	-0,023861	0,927508	0,6279808
CG10801-RA	CG10801	0,177306	1,94583	2,12163	2,93763	0,381121	0,646387	0,123642	0,70958	0,6279808
CG10802-RA	CG10802	10,1828	12,6461	13,5874	14,0816	12,6175	12,2598	-0,143843	0,504542	0,6279808
CG10803-RA	CG10803	3,35819	0,935197	3,93926	0,0298217	1,37228	0,0304431	0,052139	0,811489	0,6279808
CG10804-RA	CG10804	2,41529	2,73575	2,42726	43,705	76,1691	0,0168266	0,223714	0,366517	0,6279808
CG10804-RB	CG10804	0,0159347	9,12763	0,0152983	1,81516	4,56266	0,650457	0,223714	0,366517	0,6279808
CG10804-RC	CG10804	0,630577	3,77953	0,737336	0,0164831	3,57123	2,70352	0,24197	0,321527	0,6279808
CG10805-RA	l(2)k09022	4,15851	2,92127	7,2777	6,59802	3,44636	4,13739	-0,115873	0,679435	0,6279808
CG10806-RA	Nha1	10,1093	5,24099	15,9319	12,0657	21,4104	18,7175	-0,68113	0,033181	0,6279808
CG10806-RB	Nha1	0,0245483	9,08051	0,0235678	0,0259159	0,0351032	0,0264559	-0,681927	0,033034	0,6279808
CG10808-RA	Syngr	24,1768	23,893	24,7866	2,54535	4,66725	1,71301	0,101946	0,646765	0,6279808
CG10809-RA	CG10809	10,0964	10,0153	10,1579	11,7647	0,0277648	6,10087	0,301722	0,206387	0,6279808
CG10810-RA	Rheb	37,0779	35,7849	54,787	12,9102	21,7527	27,9445	-0,31011	0,385586	0,6279808
CG10811-RA	Rheb	31,4105	43,4138	18,913	11,281	1717,87	1908,33	0,212576	0,331143	0,6279808
CG10811-RB	Drs	12,8286	12,5147	8,95618	70,8802	10,1419	10,8826	0,210326	0,336273	0,6279808
CG10811-RC	eIF4G1	0,0114503	0,0171732	0,0100699	0,0117213	0,0145111	0,0105389	0,212555	0,331158	0,6279808
CG10812-RA	eIF4G1	90,8792	0,0497545	9,35314	82,9815	5,84887	6,11227	-2,384616	5,96E-16	0,6279808
CG10814-RA	eIF4G1	0,0104888	107,445	0,00953267	0,0107132	0,0137192	0,0135901	1,008127	0,004848	0,6279808
CG10816-RA	Drsl5	50,7368	32,9593	1,48374	1,66311	0,118131	1,43744	-0,05446	0,870193	0,6279808
CG1081-RA	CG10814	1,18891	0	0	16,3946	0	16,1284	-0,039885	0,85408	0,6279808
CG1081-RB	Dro	0,478811	0,118946	1,12832	1,2718	7,68734	2,38747	-0,006375	0,976711	0,6279808
CG10822-RA	alpha-Est4	9,23621	7,57103	18,5248	1080,25	14,6751	12,2679	NA	NA	0,6279808
CG10823-RA	CG10822	0	0,0155897	0,0164316	0,0177533	0,0240469	0,0181232	0,248841	0,265983	0,6279808
CG10823-RB	SIFaR	0,332916	28,9875	2,53747	0,886091	0,018935	0,0142706	0,250444	0,262869	0,6279808
CG10824-RA	SIFaR	1,65933	0,0319687	0,0336951	0,0380103	1,60102	1,35005	-0,583319	0,086096	0,6279808
CG10825-RA	Dsim\GD20003	1,82854	1,37449	10,7453	19,3855	4,86958	13,0953	0,248073	0,319702	0,6279808
CG10825-RB	Ice2	1,68759	41,0201	1,60413	3,57533	20,138	268,6	0,248073	0,319702	0,13772387
CG10825-RC	Ice2	0,0288079	0,0158608	0,0267252	4,93821	12,099	66,626	0,242591	0,330487	0,6279808
CG10827-RA	Ice2	1,86508	0,0158608	6,76712	1,07718	26,0084	135,849	-1,159902	0,001004	0,6279808
CG1082-RA	Alp5	10,0971	4,84156	22,5178	17,627	62,5813	0,0129666	-0,126373	0,608902	0,6279808
CG10830-RA	Ktl	11,3035	12,2308	18,3173	12,005	25,594	15,9425	-0,450006	0,100744	0,6279808
CG10833-RA	Cyp28d1	60,6142	28,2159	92,662	38,0016	94,7629	78,9585	-0,27441	0,419318	0,6279808
CG10834-RA	CG10834	0,393309	0,0633542	0	0	0	0	0,110934	0,438787	0,6279808
CG10837-RA	eIF4B	28,4603	9,04722	19,5009	63,011	53,7479	43,1458	0,374274	0,055307	0,6279808
CG10837-RB	eIF4B	100,04	0	12,2826	64,4588	38,5957	0,159199	0,369182	0,042587	0,6279808
CG10837-RD	eIF4B	62,4015	27,3616	31,4346	0,155949	0,211234	0,830482	0,060121	0,791785	0,6279808
CG10837-RE	eIF4B	0,115584	73,839	90,0284	0,0794334	1,06039	0,0789936	0,374993	0,054792	0,13772387
CG10838-RA	robl22E	0	0	0	0	0	0	NA	NA	0,6279808
CG10839-RA	Dsim\GD24033	0	0	0	0	0	0	NA	NA	0,6279808
CG10840-RB	Cont	37,7786	11,5939	94,1058	18,5006	0,0812462	0,0200999	0,152671	0,468115	0,6279808
CG10841-RA	eIF5B	23,4002	7,80905	0,0427184	107,617	155,028	2,42914	0,590305	0,067003	0,6279808
CG10842-RA	CG10841	0	0,0374139	0,065724	0,0720222	0	0	0,966079	0,001699	0,13772387
CG10843-RA	Cyp4p1	11,1178	8,08041	8,69447	5,21424	0,0233425	0,0175923	-0,509364	0,117366	0,13772387
CG10844-RA	Cyp4p3	2,46881	0,0126504	0,0133336	4,41316	5,38401	4,85457	-0,097909	0,704871	0,6279808
CG10844-RB	RyR	0,945039	0,0166184	0	0,0193542	2,33328	0,0192211	-0,097909	0,704871	0,6279808
CG10844-RC	RyR	2,54854	0,0164955	0	0,0195726	2,1764	0,0211025	-0,097896	0,704777	0,6279808
CG10844-RD	RyR	0,95342	0,0180379	0	36,9959	0,00571478	0,0201843	-0,097896	0,704777	0,6279808
CG10845-RA	RyR	2,53594	0,0172867	0	1,37331	0,00573019	0,0197575	-0,105881	0,765855	0,6279808
CG10846-RA	CG10845	0,153824	12,81	5,63466	6,85054	16,1986	0,21182	-0,340482	0,238695	0,6279808
CG10846-RB	DCTN5-p25	0,106919	0,0973891	25,8721	0,158576	0,214792	12,5049	-0,335637	0,245919	0,6279808
CG10847-RA	DCTN5-p25	21,6769	15,8952	11,6795	23,8936	37,3731	23,6239	0,309007	0,180076	0,6279808
CG10847-RB	enc	0,00918273	0,00836427	0,00881597	1,57661	0,0242429	0,0095458	0,309007	0,180076	0,6279808
CG10847-RD	enc	3,68077	5,65348	3,27314	2,10523	4,95478	0,00956617	0,301571	0,189151	0,6279808
CG10847-RE	enc	2,15646	2,03212	1,93567	4,11685	0,0247402	2,21666	0,297393	0,195669	0,6279808
CG10849-RA	enc	6,48843	3,80855	5,23557	4,95686	0,103338	4,21982	0,436317	0,061074	0,6279808
CG1084-RA	Sc2	65,5882	60,7417	90,9124	72,892	35,9268	54,0135	0,084375	0,755109	0,6279808
CG10850-RA	ida	0,0265046	0,0241422	78,2738	3,31499	15,7483	1,61348	0,266082	0,349335	0,6279808
CG10850-RB	ida	0,0263987	0,0240457	16,5677	28,0564	29,8574	6,43518	0,266082	0,349335	0,6279808
CG10851-RA	B52	0,0168232	5,42531	40,815	14,0909	0,0470106	17,901	0,127268	0,566666	0,6279808
CG10851-RB	B52	0,0411773	0,0236489	0,0161513	6,31951	0,0441284	3,02472	0,127674	0,565466	0,6279808
CG10851-RC	B52	40,2298	0,0236118	0,0395326	0,0281285	5,29795	0,0287147	0,126993	0,567252	0,6279808
CG10851-RD	B52	0,0219764	0,0236396	122,434	0,0280806	0,0282482	0,0286657	0,099945	0,649338	0,6279808
CG10851-RE	B52	0,0222728	0,0237328	0,0210987	0,0281165	0,0386112	0,0287024	0,126966	0,567673	0,6279808
CG10851-RF	B52	49,7737	4,51457	0,0213832	0,028237	0,0377937	0,0288254	0,088011	0,689209	0,6279808
CG10851-RG	B52	22,7117	0,0235287	32,5363	5,72218	13,7039	4,9544	0,099878	0,649543	0,6279808
CG10852-RA	Acp63F	0	0,284794	10,5397	1,36239	11,1647	0,171518	-0,039618	0,84166	0,6279808
CG10853-RA	CG10853	0,350845	23,8602	0,785943	1,11705	13,0864	0	-0,384408	0,255763	0,6279808
CG10855-RA	ntc	1,58127	1,87088	3,19815	2,07304	1,87564	5,10857	0,165184	0,572837	0,6279808
CG10858-RA	ppk27	0	16,1496	11,5574	9,49797	8,00783	0	-0,058998	0,681721	0,6279808
CG10859-RA	CG10859	0,0448073	0,0408136	0,903372	0,0792261	0	0	0,473382	0,102773	0,6279808
CG10861-RB	Glut1	1,02024	1,99589	1,93672	2,87244	2,53298	0,0249081	-0,086614	0,76338	0,6279808
CG10862-RA	Glut1	0,0332429	0,0302799	0,0319152	0,0358395	0,0485448	0,0365863	0,110934	0,438787	0,6279808

gene_id	Symbol	W1_FPKM	W2_FPKM	W3_FPKM	MCM51_FPKM	MCM52_FPKM	MCM53_FPKM	FC	p-value	p-adj
CG10863-RA	Glut1	0,0309247	0,0281684	0,0296896	0,0331527	0,0449055	0,0338435	0,532881	0,071194	0,6279808
CG10864-RA	Glut1	0,0312317	0,028448	0,0299843	0,0335067	0,0453851	0,0342049	-0,392365	0,198602	0,13772387
CG10866-RA	Glut1	1,20154	0,0248739	0,0262171	2,80521	0,0393085	2,05168	-0,022992	0,939547	0,6279808
CG10868-RA	Glut1	5,53942	4,04828	8,67295	9,46861	10,0698	9,69247	-0,156322	0,614608	0,6279808
CG10868-RB	Atg12	0,0998125	659,172	1,4919	753,908	0,849754	0,319412	-0,079658	0,803083	0,6279808
CG10868-RC	CG10862	0,0868658	0,0352448	6,76097	8,88491	14,2643	9,28016	-0,017866	0,95624	0,6279808
CG10868-RD	CG10863	40,8867	0,159787	2,18715	8,41543	1,85856	1,267	-0,262485	0,430671	0,6279808
CG10868-RE	CG10864	0,960077	1,18315	0,0585751	12,5333	0,025366	0,0180771	-0,200649	0,559058	0,6279808
CG10869-RA	CG10866	2,65823	0,531241	3,42705	6,30212	2,81686	3,58189	0,457141	0,184691	0,6279808
CG10869-RB	orb	0,51113	1,01478	0,294429	0,323918	0,0220181	0,236139	0,394498	0,165884	0,6279808
CG1086-RA	orb	0,0157224	0,237959	0,0150944	0,0215648	0,295562	0,0165941	-0,777938	0,000919	0,13772387
CG1086-RB	orb	0,213074	0,014321	0,178168	1,61634	0,327402	0,225403	-0,711666	0,004144	0,6279808
CG1086-RC	orb	0,0345826	0,231647	0,0332014	0,0261007	0,0292096	0,0381856	-0,696921	0,004931	0,6279808
CG1086-RD	orb	0,0309279	0,0315002	0,0197951	9,33327	0,0374921	0,0220142	-0,697387	0,004665	0,6279808
CG1086-RE	CG10869	0,258148	0,248971	0,349889	0,333004	0,0847279	0,0638561	-0,711343	0,004186	0,6279808
CG1086-RF	CG10869	0,0951186	0,0866406	0,0913195	0,120787	0,119319	0,0899261	-0,730932	0,002039	0,6279808
CG10874-RA	CG10874	17,2523	3,07146	3,81744	5,12556	3,29359	4,00606	0,154573	0,417112	0,6279808
CG10874-RB	CG10874	0,0541753	4,60189	0,0222846	1,74898	4,11329	0,0810886	0,195527	0,30661	0,6279808
CG10877-RA	CG10877	9,77355	11,0329	27,6224	69,689	19,9069	19,2222	0,228596	0,37031	0,6279808
CG10880-RA	Vha26	472,065	5,45985	17,7439	9,09317	4,64379	11,2132	0,096417	0,750344	0,6279808
CG10881-RA	Vha26	0,0297282	16,3988	4,72529	18,0074	9,35518	2,18259	-0,277208	0,224726	0,13772387
CG10882-RA	CG10880	3,79538	0,0211428	56,0702	0,0244268	3,02414	0,0249358	0,105425	0,710541	0,6279808
CG10887-RA	eIF3g2	0	13,2799	0,0310863	0,0211291	4,54987	3,46859	0,022403	0,887437	0,6279808
CG10888-RA	Sec24CD	25,5329	28,9442	24,9501	0	0	0	-0,013096	0,914398	0,6279808
CG10889-RA	CG10887	0	0	0	0	0	0	0,176714	0,442736	0,6279808
CG1088-RA	Rh3	0	0,234493	1,31817	2,5455	0	2,28064	0,15857	0,450944	0,6279808
CG1088-RB	CG10889	2,53222	2,41972	2,72937	0,0343852	0	0	0,158614	0,450779	0,6279808
CG10890-RA	alpha-Est5	10,1279	9,96774	15,4586	12,1099	11,2098	31,7055	0,261544	0,33121	0,6279808
CG10890-RB	mus201	0,0141652	0,0129026	0,0191613	0,0208418	0,0197653	0,0212761	0,167225	0,562303	0,6279808
CG10890-RC	mus201	0,0170753	0,87099	20,3365	14,6917	0,0239888	15,0905	0,261544	0,33121	0,13772387
CG10895-RA	mus201	1,34397	0,405412	0,0135994	0,0145923	0,806079	0,0148963	0,280842	0,18032	0,13772387
CG10895-RB	lok	0,0309827	0,0282212	0,0298854	3,61398	2,25173	0,0340836	0,281553	0,179312	0,6279808
CG10895-RC	lok	4,54504	5,46181	10,5084	3,52907	7,32278	10,0301	0,281553	0,179312	0,6279808
CG10897-RA	lok	1,572	0,0283542	0,0857638	0,0333879	0,151139	0,113907	0,026594	0,925744	0,6279808
CG10897-RB	tou	4,31279	4,03315	0,0227644	26,48	0,0338389	0,025503	0,298425	0,300737	0,6279808
CG10897-RC	tou	0,0237114	0,021598	7,25162	0,0294485	3,36061	4,43841	0,254158	0,37992	0,6279808
CG10897-RD	tou	6,79687	8,29802	2,16106	0,0246276	1,79403	1,75699	0,298425	0,300737	0,6279808
CG10897-RE	tou	2,24449	2,07689	0,00636489	4,87625	0,00909096	0,0068515	-0,145515	0,614698	0,6279808
CG10898-RA	tou	0,00662969	0,00603878	29,9518	7,76623	45,7073	38,2483	0,241788	0,285484	0,6279808
CG10899-RA	CG10898	12,8163	2,4388	3,19576	8,03883	4,83978	5,11262	0,075805	0,767816	0,6279808
CG10899-RB	CAH4	0,0698721	0,105652	0,315514	0,238348	0,0198532	6,97545	0,075805	0,767816	0,6279808
CG1089-RA	CAH4	0,185467	1,29307	0,0670814	0	0,0198532	0,0844482	-0,175011	0,562994	0,6279808
CG10901-RA	CG1090	0,607466	0,0160383	0,068903	13,2067	0,0359082	0,0186664	-0,366831	0,269121	0,6279808
CG10903-RA	CG1090	4,3024	4,1537	0,0648858	12,7114	6,22186	3,22263	-0,125508	0,707026	0,6279808
CG10904-RA	osk	0,356856	0,46136	0,320499	0,70834	0,381711	0,456874	0,089677	0,707744	0,6279808
CG10907-RA	CG10903	12,4406	3,94251	23,662	7,39237	13,877	9,73129	-0,000342	0,998888	0,6279808
CG10908-RA	CG10904	9,46436	2,83632	38,2153	0,0397772	0,0543046	24,7458	-0,275179	0,199703	0,6279808
CG10909-RA	CG10907	4,19496	3,99215	6,43184	9,06828	3,9175	0,476933	NA	NA	0,6279808
CG1090-RA	Der-1	47,7249	41,3782	0,0269982	7,57191	0,0405589	0,0305676	-0,0111	0,962754	0,6279808
CG1090-RB	CG10909	0	0	0	4,24233	10,1828	0	-0,008585	0,97108	0,6279808
CG10910-RB	Tailor	8,41567	5,17298	0,0132224	0,0140209	0,0189914	1,75278	0,200554	0,549287	0,6279808
CG10911-RA	Tailor	6,38938	7,99244	0,0130833	4,47567	0,0190401	0,0149859	-0,10484	0,756296	0,6279808
CG10912-RA	CG10910	10,7825	0,0177141	31,7607	16,8181	94,4619	3,99846	-0,394397	0,256681	0,6279808
CG10913-RA	CG10911	11,4362	0	54,6377	0,883123	35,1419	41,4173	-0,428478	0,093026	0,6279808
CG10914-RA	CG10912	19,6037	0,250777	7,64476	26,2941	3,73939	6,50162	0,443288	0,058348	0,6279808
CG10915-RA	Spn55B	47,6697	5,34706	84,1902	60,8191	4,88087	3,59517	-0,137688	0,616381	0,13772387
CG10916-RA	CG10914	5,28542	5,46213	7,69683	0,0745785	3,57236	4,25244	0,050764	0,845335	0,6279808
CG10917-RA	CG10915	6,15012	8,7803	6,76719	0,0877946	5,36165	5,53581	0,286488	0,269982	0,6279808
CG10918-RA	CG10916	13,3771	20,3867	16,6366	4,57105	13,6478	19,0531	0,140744	0,642626	0,6279808
CG10919-RA	fj	15,6912	27,0529	14,1983	42,1344	0,0469118	36,9786	0,07663	0,535079	0,6279808
CG1091-RA	CG10918	4,5487	1,19937	23,0993	0,0183823	0,0248989	4,51993	0,065029	0,774795	0,6279808
CG1091-RB	CG10919	0	0	0,382726	0	0	0	0,042732	0,850194	0,6279808
CG10920-RA	CG1092	15,6269	20,2193	16,3791	88,4295	0,0317672	4,95715	0,078037	0,549183	0,6279808
CG10922-RA	CG1092	21,4449	17,6208	27,2412	0,881865	62,068	14,2054	-0,446725	0,138403	0,6279808
CG10922-RB	CG10920	0,0964893	0,0527335	0	0,0405108	0	0	-0,446497	0,138639	0,6279808
CG10923-RA	La	42,3006	12,0423	2,08061	0,0269328	68,5073	0,027494	-0,447387	0,147799	0,6279808
CG10924-RA	La	0,04625	0,0123081	10,1996	12,9179	0,0443037	5,29277	-0,675041	0,037525	0,6279808
CG10924-RB	Klp67A	1,53575	5,86852	19,5263	8,1453	8,02914	9,54762	-0,654035	0,04371	0,13772387
CG10927-RA	Pepck1	3,48568	7,5351	8,11913	0,011759	0,0161087	9,73115	-0,360092	0,254416	0,6279808
CG10928-RA	Pepck1	2,86715	7,82508	1,63725	168,415	1,73229	3,77476	-0,879227	0,01191	0,6279808
CG1092-RA	CG10927	2,36589	4,7101	4,3895	0,034061	55,8127	2,66942	1,090309	1,96E-05	0,13772387
CG1092-RB	CG10928	0,205416	1,88824	1,67018	0,075346	0,0828697	0,0201904	1,070409	3,54E-05	0,6279808
CG10930-RA	plx	3,17657	17,868	11,0607	12,8493	13,7138	9,57525	NA	NA	0,6279808
CG10931-RA	plx	15,6271	9,46766	8,6166	24,5523	1,30581	7,37082	0,013317	0,962212	0,6279808
CG10932-RA	PpY-55A	0	0	0	0	0	0,0144857	-0,524034	0,11393	0,6279808
CG10933-RA	CG10931	0,0822521	0,0352448	0,078967	0,0226625	0,0351032	0,0264559	0,093782	0,72148	0,6279808
CG10934-RA	CG10932	10,2049	7,77645	23,4793	12,8097	59,8115	23,1599	0,009686	0,972374	0,6279808
CG10936-RA	Dlish	7,4082	9,42918	13,7223	28,3954	8,70092	5,06425	0,341713	0,245711	0,6279808
CG10936-RB	CG10934	0,0633518	0,0176001	8,61257	23,356	40,3951	1,97542	0,341713	0,245711	0,6279808
CG10938-RA	CG10936	0	16,2294	21,3832	53,9827	0,905559	50,651	-0,567257	0,054805	0,6279808
CG10938-RB	CG10936	0	14,2013	11,4812	29,8106	14,1121	39,9946	-0,567257	0,054805	0,13772387
CG10939-RA	Prosalpha5	0,0792592	0,0721947	4,23538	5,84527	0,0294215	3,06197	0,024315	0,920276	0,6279808
CG1093-RA	Prosalpha5	32,6079	29,7383	0,0760935	0,0962596	0,0367208	174,188	-0,097601	0,684367	0,6279808
CG1093-RB	CG10939	58,0282	10,4022	0,0221368	57,4618	2,48177	1360,03	0,043999	0,856414	0,6279808
CG10943-RA	CG10943	17,606	7,49218	10,0253	4,38317	7,86469	0,188633	0,547194	0,120625	0,13772387
CG10944-RA	RpS6	0,0613707	0,031666	5,09899	0,219873	0	0,224455	-0,256153	0,311397	0,6279808
CG10944-RB	RpS6	1526,01	0,230776	0,152614	0,390255	3,64338	0,0444425	-0,256053	0,311661	0,6279808
CG10946-RA	dpr14	1,94223	1,22197	27,5076	16,1117	16,5239	18,0579	-0,191971	0,496702	0,6279808
CG10947-RA	CG10947	2,62577	2,74544	5,39686	3,20589	7,29466	5,48391	-0,683586	0,030253	0,6279808
CG10947-RB	CG10947	2,88374	2,17693	1,82042	4,70529	7,20546	3,42911	-0,684335	0,029689	0,6279808
CG10948-RB	CG10948	7,06533	3,34134	8,91321	2,29119	6,56452	3,35389	0,301543	0,253142	0,6279808
CG10948-RC	CG10948	8,01965	47,2192	0,0222533	19,7963	3,54959	0,0248988	0,298051	0,256724	0,6279808
CG10948-RD	CG10948	0,0231791	9,83588	3,55522	16,9101	24,3074	8,55147	0,297747	0,257617	0,6279808
CG10949-RA	CG10949	3,34092	2,17113	4,48297	3,4054	2,94134	3,14509	-0,168686	0,542326	0,6279808
CG10950-RA	CG10950	0	0,0945833	0,0498456	0	0	0,0591244	0,082831	0,633352	0,6279808
CG10951-RA	niki	0,366216	0,405056	0,489714	0,0146572	0,120851	1,98374	0,122868	0,709947	0,6279808
CG10952-RA	eag	0,470359	0,142812	0,269359	1,08234	0,0231548	0,467357	-0,497544	0,113204	0,6279808
CG10952-RB	eag	0,192454	0,645105	0,21433	30,8768	0,963446	22,1672	-0,53636	0,086538	0,6279808
CG10953-RA	CG10953	861,916	527,811	9,96564	11,3585	6,34146	6,89217	-0,194495	0,536848	0,6279808
CG10954-RA	Arpc2	51,7377	3,87374	3,79504	7,73098	3,72001	3,8198	0,120476	0,579378	0,6279808
CG10955-RA	Rtf1	11,279	9,85543	0,166939	13,1158	7,40192	11,3103	-0,253919	0,196778	0,6279808
CG10956-RB	Dsim\GD25493	0	2,2686	0,01891	6,94969	5,73715	6,18829	NA	NA	0,6279808
CG10958-RA	CG10958	1,31328	1,06722	3,86148	17,3923	29,0054	1,20569	-0,108209	0,690694	0,6279808
CG10959-RA	CG10959	2,5864	2,26989	4,29732	2,13116	1,52037	1,65628	0,281534	0,297367	0,6279808

gene_id	Symbol	W1_FPKM	W2_FPKM	W3_FPKM	MCM51_FPKM	MCM52_FPKM	MCM53_FPKM	FC	p-value	p-adj
CG10960-RA	CG10960	42,4918	10,8805	32,0004	0	0,836162	0,630182	-0,214885	0,383144	0,6279808
CG10960-RB	CG10960	9,28542	0,0211131	9,49112	9,53683	38,3086	0	-0,222041	0,359324	0,6279808
CG10960-RC	CG10960	11,694	7,85606	15,8734	9,48858	8,9341	3,71057	-0,237733	0,332845	0,6279808
CG10961-RA	Traf6	5,37744	26,3147	2,22935	2,67296	31,1442	1,52105	0,313553	0,158106	0,6279808
CG10962-RA	CG10962	7,36235	35,7384	0,180732	8,50671	5,91187	7,92277	0,540538	0,05887	0,13772387
CG10962-RB	CG10962	0,0505682	56,6426	1,88656	0,0569248	0,077105	0,058111	0,289445	0,272587	0,6279808
CG10964-RA	sni	5,90412	19,6541	3,14786	15,9224	0,016022	0,0120751	-0,153137	0,601167	0,6279808
CG10965-RA	Corp	1,86776	10,42	7,46734	9,86903	45,9757	34,0723	-0,835601	0,002536	0,6279808
CG10967-RA	Atg1	12,6919	0,697672	0,963204	12,5918	2,55903	2,0715	-0,039187	0,874091	0,6279808
CG10969-RA	CG10969	0,0473323	0	58,0332	0,0841686	0	0	-0,034896	0,785626	0,6279808
CG10970-RA	CG10970	4,30771	14,9368	4,94176	12,2081	3,84802	3,60549	0,393185	0,17168	0,6279808
CG10971-RA	Hip1	0,0156257	0,014233	0,0618492	0,0161518	0,101824	0,0164884	0,177781	0,497236	0,6279808
CG10971-RB	Hip1	14,0153	11,6326	18,6705	9,42524	18,3451	7,52588	0,178319	0,496818	0,6279808
CG10972-RA	ppk12	0,309127	31,0369	0,278232	31,5405	9,87486	23,8545	0,474703	0,170851	0,6279808
CG10973-RA	CG10973	0,0644223	0,0586802	5,08781	0,0751741	0,0435046	0,0767406	0,017769	0,930414	0,6279808
CG10973-RB	CG10973	13,567	21,1663	12,9099	24,8504	12,6744	14,5083	0,009447	0,963119	0,6279808
CG10975-RA	Ptp69D	0,012929	0,0330574	0,0124126	0,0132805	0,0533949	0,0135573	0,033425	0,897954	0,13772387
CG10975-RB	Ptp69D	8,07891	5,8966	7,4209	11,1781	0,0571929	4,83141	0,033425	0,897954	0,6279808
CG10978-RA	jagn	0,0623552	0,0567975	0,0357794	2,08124	0	0,0799323	-0,129629	0,479377	0,6279808
CG10978-RB	jagn	64,8519	66,9253	0,0972957	0,146582	0,176963	0,122853	0,072555	0,700883	0,6279808
CG10978-RC	jagn	0,0929771	0,0846899	12,774	7,68171	6,64915	0,073875	-0,052966	0,773834	0,6279808
CG10979-RA	CG10979	3,66196	6,36043	18,2433	8,28317	2,94583	1,37817	-0,043917	0,892009	0,6279808
CG10981-RA	Madm	8,35651	17,0898	28,0826	18,1508	9,81926	18,3718	0,400833	0,059741	0,6279808
CG10981-RB	dgrn	3,90965	0,156899	6,85893	2,84301	12,7722	1,8385	0,400833	0,059741	0,6279808
CG10984-RA	dgrn	4,57318	1,93213	0,0277988	4,40432	0,0974863	2,06629	0,112499	0,5562	0,6279808
CG10984-RB	CG10984	5,02925	0,0273492	1,56165	10,1093	0,974686	7,39954	0,112499	0,5562	0,6279808
CG10984-RC	CG10984	13,447	15,3344	0,0150016	9,59327	0,0218777	6,92015	0,110973	0,561688	0,6279808
CG10986-RB	CG10984	0,0291382	0,0265411	10,3824	0,0311027	11,0293	0,0317508	0,120278	0,566865	0,6279808
CG10987-RA	g	9,01179	8,95873	7,99802	9,88003	5,751	6,25036	0,03389	0,91955	0,6279808
CG10988-RA	CG10987	0,102709	0,0766219	5,80373	0,120205	0,0937136	0,125847	-0,284241	0,222355	0,6279808
CG1098-RA	Grip91	1,74227	1,46649	2,2541	4,43593	5,56163	30,9149	-0,153421	0,464046	0,6279808
CG10990-RA	Dap160	0,0179668	0,0163654	0,0172492	0,0636929	0,0181237	27,0478	-0,084705	0,803194	0,6279808
CG10990-RB	Dap160	9,01111	9,44181	8,84507	28,1106	0,0185032	20,6303	-0,029325	0,930913	0,6279808
CG10991-RA	Pdcd4	44,3944	2,03478	36,9046	4,85499	1,72108	4,06025	0,202722	0,384346	0,6279808
CG10992-RA	Pdcd4	18,7037	1,01896	20,7731	0,0165328	0,0222819	0,0168773	-0,053149	0,802812	0,6279808
CG10993-RA	CR10991	0	0,0538341	0,033851	0,0317012	10,1573	0,149637	0,307801	0,317291	0,6279808
CG10996-RA	CtsB1	85,1752	110,612	121,631	126,948	1,9526	110,892	-0,287146	0,301672	0,6279808
CG10997-RA	CG10993	0,371213	0,0722238	0,356386	11,2509	0,262749	0,198023	-0,119821	0,569585	0,6279808
CG10998-RA	CG10996	0,0792911	0,06246	0,0761242	0,0447934	0,205864	0,155151	-0,130622	0,704134	0,6279808
CG10999-RA	Clic	73,3514	6,5334	94,2849	7,06995	0,0606729	7,18589	0,300731	0,280414	0,6279808
CG10999-RB	r-cup	0,355081	0	0	0	0	0,566572	0,302426	0,277889	0,6279808
CG10999-RC	CG42675	2,41551	0,0354873	8,58408	15,017	4,40973	0	0,290655	0,340359	0,6279808
CG1099-RA	CG42675	0,0372678	2,76661	0	7,11649	0,467252	7,7144	-0,241137	0,276045	0,13772387
CG1099-RB	CG42675	0,101343	4,70784	16,1172	0,0313543	1,75489	1,82672	-0,221062	0,326546	0,6279808
CG11000-RB	Rpn5	22,0716	21,8457	0,035308	9,58851	0,0541727	0,0408278	0,002234	0,994589	0,6279808
CG11001-RA	CG11000	1,71969	3,28345	5,24016	5,42691	2,87289	6,41179	-0,405617	0,201798	0,6279808
CG11006-RA	Fkbp12	222,941	124,47	28,333	59,155	0,0277303	8,12287	-0,034317	0,877033	0,6279808
CG11006-RB	Sap130	0,0154059	0,0140328	0,0147906	0,0161242	1,68431	0,0164601	-0,033748	0,878895	0,6279808
CG11006-RC	Sap130	0,0158152	0,0144056	0,0151835	0,0165744	6,05461	0,0169197	-0,034171	0,877507	0,6279808
CG11006-RE	Sap130	10,4626	9,41154	6,90718	15,8539	0,0330372	6,4462	-0,0327	0,882618	0,6279808
CG11007-RA	Sap130	4,50148	6,01717	7,62927	5,13491	0,221933	5,89505	-0,158668	0,542233	0,6279808
CG11008-RA	CG11007	21,2664	59,8747	0	76,3529	103,991	2,0045	-0,159269	0,571876	0,6279808
CG11009-RA	CG42588	0,749759	11,0835	20,2499	6,14441	9,61262	10,07	-0,056611	0,816111	0,6279808
CG11009-RB	Wbp2	19,3728	1,79126	19,1719	23,0344	2,88368	21,4891	-0,056414	0,816757	0,6279808
CG11009-RC	Wbp2	13,7315	0	0,0374995	15,6709	0	17,5757	-0,056414	0,816757	0,6279808
CG1100-RA	Wbp2	0,0390596	3,60207	1,43488	0,0427169	1,41392	4,56752	0,112292	0,574518	0,6279808
CG11010-RA	Ref1	74,9153	49,3407	109,291	0,0141383	0,0191504	2,61906	0,169581	0,494664	0,6279808
CG11012-RA	Ent3	3,262	0,138273	4,74707	0,0971769	0,0224501	5,89157	-0,056258	0,875093	0,6279808
CG11015-RA	Ugt37A1	1,03029	0,27111	1,34083	0,899362	1,43066	0,547953	-0,511419	0,107874	0,6279808
CG11018-RA	Dsim\GD23418	205,751	2,36572	9,64984	2,10122	6,98325	8,7472	-0,203883	0,535671	0,6279808
CG11019-RA	CG11018	0,237873	0,192597	0,126874	271,721	30,0788	14,5171	-0,046692	0,886147	0,6279808
CG1101-RA	CG31688	1,19332	42,3619	67,1108	56,1626	36,4265	44,9839	-0,191824	0,375837	0,6279808
CG11020-RA	MP1	22,8778	18,1196	25,6583	26,4416	21,5867	24,9154	-0,764737	0,011259	0,6279808
CG11020-RB	MP1	0,0457694	0,04169	0,0439414	0,0509004	0,068945	0,0519611	-0,68479	0,014712	0,6279808
CG11020-RD	nompC	0,233247	0,502543	15,6925	3,81844	0	1,46268	-0,649231	0,021434	0,6279808
CG11023-RA	nompC	0,0115787	0,123109	0,0203388	0,136781	2,29702	0,334868	0,199319	0,558422	0,6279808
CG11024-RA	nompC	0,534477	0,0304177	0,0215283	0,0360158	15,4917	3,98988	-0,142132	0,586999	0,6279808
CG11025-RA	CG11023	0,503116	0,66195	0,608249	0,760158	0,22737	0,567297	-0,01835	0,934668	0,6279808
CG11025-RB	cl	141,924	124,114	172,703	158,696	219,533	185,452	0,077699	0,730221	0,6279808
CG11027-RA	isopeptidase-T-3	2,13924	11,8367	37,6392	4,50764	27,1642	894,751	-0,369282	0,226247	0,6279808
CG11029-RA	isopeptidase-T-3	2,55935	1,84928	2,65555	0,927461	3,78877	54,8678	-0,36822	0,301872	0,6279808
CG1102-RA	Arf102F	211,46	138,8	126,059	185,261	20,0629	0,613739	-0,077365	0,716561	0,6279808
CG1102-RB	CG11029	0,393309	0,134345	0,9204	0,870607	0,740985	0,707106	-0,074958	0,725269	0,6279808
CG11030-RA	CG1103	6,25549	5,20754	0,0760935	11,0421	4,78228	4,98932	-0,283929	0,190007	0,6279808
CG11033-RA	CG1103	13,0861	15,393	0,158989	12,1461	6,98286	12,1267	0,000576	0,998246	0,6279808
CG11034-RA	CG11030	12,5294	11,3741	18,0435	20,5296	14,4533	16,3381	-1,201084	1,05E-07	0,6279808
CG11035-RA	Kdm2	10,0424	12,4596	5,46693	17,5442	6,42308	7,66636	-0,256011	0,443914	0,6279808
CG11037-RA	CG11034	6,82017	0,210934	199,829	18,0037	213,562	0	0,044335	0,715924	0,6279808
CG1103-RA	CG11035	6,33942	4,51846	12,9699	0,00996167	11,8317	0,0101692	0,229717	0,307134	0,6279808
CG1103-RB	CG11037	0	0,164085	0	0,0688869	2,53269	0	0,232889	0,30022	0,6279808
CG11041-RA	Ufl1	20,0139	19,9711	0,0321199	25,9154	0,0503524	0,0379486	-0,493815	0,121854	0,6279808
CG11042-RA	Ufl1	0,0262344	0,0238962	27,3461	0,027808	36,155	28,5119	-0,146543	0,497713	0,6279808
CG11043-RA	CG11041	4,56622	3,9635	4,28069	5,86968	0	0	0,183779	0,587563	0,6279808
CG11044-RA	CG11042	344,954	459,756	8,20211	6,4983	0,0211118	5,00544	0,078573	0,770346	0,6279808
CG11048-RA	Dpse\GA10724	0,102127	0,0930239	307,712	0,148231	54,0861	49,1081	-0,100288	0,724067	0,6279808
CG11049-RA	CG11044	8,1262	12,258	6,76246	9,14719	4,93822	9,01599	0,238193	0,364919	0,6279808
CG11049-RC	Dsim\GD11498	0,0266793	0,0243014	0,0256138	0,902865	0,298483	0,817261	0,27897	0,280168	0,13772387
CG11049-RD	sv	0,0302453	0,0275495	0,0290373	0,0323709	287,749	0,0330455	0,267604	0,304425	0,6279808
CG11049-RE	sv	0,0246859	0,0224856	0,0236999	3,33387	13,9852	0,0266129	0,238193	0,364919	0,6279808
CG11049-RF	sv	1,33672	1,82262	1,15736	1,06883	50,478	0,0173384	-0,118997	0,667065	0,6279808
CG11049-RG	sv	0,016008	0,0145812	0,0153687	0,0165619	32,7472	0,016907	-0,141274	0,607153	0,6279808
CG11049-RH	sv	0,0142507	0,0129805	0,0136815	0,0146833	18,4102	0,0149893	0,238193	0,364919	0,6279808
CG1104-RA	sv	5,12768	0,0130283	3,30939	1,60565	32,7224	0,0150463	0,084176	0,663471	0,6279808
CG1104-RB	sv	0,0143218	4,27884	0,0137498	2,37223	0,0307176	3,1019	0,086158	0,656549	0,6279808
CG11050-RA	CG1105	20,636	20,8391	0,0251866	27,3872	0,0376661	0,0283875	0,052027	0,84579	0,6279808
CG11050-RB	CG11050	7,2324	0,00891167	5,85024	0,00997675	0,109062	0,0101846	0,054341	0,838847	0,6279808
CG11050-RC	CG11050	0,0338908	1,75704	0,00939294	1,71888	6,27134	7,90921	0,056386	0,832876	0,6279808
CG11051-RA	CG11050	35,2237	5,69219	7,98474	6,95258	0,0135136	1,37187	-0,672349	0,036065	0,6279808
CG11052-RA	Nplp2	2773,17	19,1593	0,0192369	9,8258	0,0341888	3,93129	-0,057534	0,865528	0,6279808
CG11055-RA	CG11052	2,0331	1,38892	0,0204723	88,7403	35,2646	63,6371	0,454343	0,094327	0,6279808
CG11055-RB	Dsim\GD11499	8,36243	10,3263	0,0177711	10,2799	0,0344927	4,75257	0,452164	0,096108	0,6279808
CG11055-RC	Dsim\GD11499	0,0188215	0,0171439	47,762	0,0196017	8,26274	0,0200102	0,452913	0,096115	0,6279808
CG11059-RA	Dsim\GD11499	0,0185104	2,24245	0,0640343	0,0192636	0,0623468	0,019665	0,311316	0,176243	0,6279808
CG11059-RB	Cals	111,762	0,777088	0,71033	0,782207	0,0325196	101,154	0,311534	0,175932	0,6279808

gene_id	Symbol	W1_FPKM	W2_FPKM	W3_FPKM	MCM51_FPKM	MCM52_FPKM	MCM53_FPKM	FC	p-value	p-adj
CG1105-RA	Cals	44,1541	110,298	69,0911	13,5308	40,6135	10,4423	-0,482572	0,059727	0,6279808
CG11060-RA	Gel	31,2088	25,4054	38,0664	0,485835	1,1346	0,121049	0,350857	0,289546	0,6279808
CG11061-RA	Gel	0,022951	0,0209053	0,0220343	51,4239	0,140108	350,208	-0,653311	0,017432	0,6279808
CG11061-RB	Gel	0,0222553	0,0202716	0,0213664	0,0246184	58,4136	38,2778	-0,657247	0,016685	0,6279808
CG11062-RA	Gel	0,0219375	0,0199822	0,0210614	0,0238175	2,14541	0,0251314	0,121336	0,599144	0,6279808
CG11063-RB	Gel	52,3395	54,3032	59,9192	0,023453	14,1374	0,0243138	-0,157771	0,502823	0,6279808
CG11064-RA	CG11060	0,171626	0,29963	0,123578	4,55169	2,07796	2,29138	0,46835	0,114339	0,6279808
CG11066-RA	GM130	4,65819	7,71771	6,07233	5,91113	8,94232	9,18939	-0,261543	0,218341	0,6279808
CG11066-RB	GM130	2,36759	2,23005	2,58643	12,0011	0,0373352	2,51489	-0,256265	0,227338	0,6279808
CG11068-RA	Actbeta	7,81485	6,39829	6,95942	7,99426	75,3398	35,8714	NA	NA	0,6279808
CG11069-RA	jub	6,75079	83,7179	7,94187	0,0116159	662,805	80,5007	0,030366	0,914037	0,6279808
CG1106-RA	apolpp	249,219	0,0211576	205,832	13,1646	33,9834	9,07231	-0,307813	0,160347	0,6279808
CG1106-RB	scaf	0,019797	2,97795	83,7746	0,0206653	7,22358	1,73328	-0,317658	0,143989	0,6279808
CG1106-RD	scaf	86,5573	0,123232	110,798	103,193	0,0407255	1,55762	-0,317673	0,14402	0,13772387
CG1106-RF	CG11068	0	0,174961	11,0161	0	0,0356074	0,069618	-0,317658	0,143989	0,6279808
CG1106-RH	CG11069	9,49273	11,4402	9,54979	16,4594	0,0252297	0,0190146	-0,317749	0,143917	0,6279808
CG11070-RA	aux	0,0151527	0,0138021	0,0145475	0,0156456	37,5087	0,0159716	0,057195	0,783585	0,6279808
CG11071-RA	aux	13,7772	16,789	12,5705	16,3715	0,021192	8,35997	0,077896	0,776328	0,6279808
CG11073-RA	CG11070	8,30365	8,46652	0	11,0358	5,73625	0,0171396	0,909717	0,004168	0,6279808
CG11076-RA	mamo	0,0685719	3,60773	0,0394999	0,0120669	94,9739	0,0457268	0,037991	0,871204	0,6279808
CG11076-RB	CG11073	13,45	0,0344382	0,036298	1,18974	29,7819	8,91586	0,037805	0,871922	0,6279808
CG11077-RA	CG11076	13,7104	35,873	0,010993	19,7778	0,0158766	6,91763	-0,058201	0,851061	0,13772387
CG1107-RA	CG11076	122,907	14,7053	79,6929	107,818	47,8764	0,0103396	0,205724	0,340946	0,6279808
CG1107-RB	CG11077	14,7103	15,7276	28,153	15,8245	27,2572	23,6354	0,205437	0,342978	0,6279808
CG11081-RA	alpha-Est6	0,0898258	0,190063	0,137981	9,82663	0,0818053	4,90136	0,18892	0,513426	0,6279808
CG11081-RB	PlexA	15,2912	12,0646	0,00971219	14,3445	0,0139836	16,1859	0,209146	0,468752	0,6279808
CG11081-RC	PlexA	0,00992925	0,00904425	10,9936	0,0101286	5,50367	121,169	0,209734	0,46891	0,6279808
CG11081-RD	PlexA	0,0101162	0,00921457	8,05609	0,0103238	1,83478	0,0119656	0,187014	0,517485	0,6279808
CG11081-RE	PlexA	11,723	11,399	18,9576	13,1489	14,1182	52,8155	0,361516	0,180203	0,6279808
CG11084-RA	PlexA	0,0194392	0,0118044	138,701	11,0098	104,917	0,0109364	0,321074	0,292319	0,6279808
CG11084-RB	pk	8,12809	16,3655	6,9406	0,0565827	0,0766416	0,0577617	0,337405	0,268099	0,6279808
CG11084-RC	pk	5,19327	6,98012	4,7503	11,7715	26,873	18,2939	0,31845	0,294406	0,6279808
CG11085-RA	pk	3,07663	3,58638	2,76259	17,2291	16,1263	15,8672	0,190883	0,58418	0,6279808
CG11086-RA	CG11085	0,19233	0,291978	0,266714	5,21659	15,4457	18,4091	0,469087	0,070464	0,6279808
CG11089-RA	Gadd45	18,1012	0,209078	12,2373	0,050517	7,27587	7,28685	-0,031049	0,904079	0,6279808
CG1108-RA	CG11089	14,5578	26,815	16,7971	23,9343	3,8517	11,9073	0,09338	0,766852	0,6279808
CG11092-RA	Wdr33	9,01532	7,57397	0,0620306	27,9866	1,64153	0,0235395	0,091447	0,711932	0,6279808
CG11093-RA	Wdr33	0,0219764	0,0200177	21,6618	13,8304	8,24908	3,31186	-0,184736	0,543939	0,6279808
CG11094-RA	Nup93-1	7,50489	43,1716	11,3433	6,00015	66,0429	7,04911	0,31202	0,302014	0,6279808
CG11094-RB	fuss	7,92704	6,52667	4,05311	5,38163	6,73036	6,73835	0,45668	0,18669	0,6279808
CG11094-RC	dsx	0,517696	0,486765	28,2158	0,0173057	0,160085	0,428549	0,417627	0,228485	0,6279808
CG11095-RA	dsx	0,0207221	0,443565	0,0287087	0,0216783	0,130293	0,02213	0,053571	0,831689	0,6279808
CG11098-RA	dsx	0,018469	0,0168228	0,032256	0,0192187	1,23428	0,0196192	-0,0014	0,995085	0,6279808
CG11098-RB	CG11095	3,91473	102,409	9,83488	112,528	4,96684	54,5914	0,008554	0,969834	0,6279808
CG11099-RA	Tango1	0,0162202	0,0147745	0,0155724	0,0167897	0,0227418	13,1847	0,372317	0,177252	0,6279808
CG1109-RA	Tango1	16,5381	16,0076	18,9854	24,5369	10,6115	0,0821958	-0,182148	0,380147	0,6279808
CG1109-RB	Dsim\GD11497	1,67619	2,16406	1,73518	1,2625	0,403164	0,234153	-0,182599	0,379333	0,6279808
CG11100-RB	Mes2	17,9565	22,5096	0,0294689	20,0002	16,67	12,5819	0,212159	0,397936	0,6279808
CG11100-RC	Mes2	0,0306949	0,027959	188,572	0,0328879	235,69	0,0335733	0,248522	0,324222	0,6279808
CG11101-RA	pwn	10,8358	17,3167	9,73215	5,23663	1,78044	1,92522	0,171998	0,619874	0,6279808
CG11103-RB	CG11103	6,38834	8,2053	5,26742	84,2724	5,00766	478,286	0,244543	0,344235	0,6279808
CG11105-RA	CG44422	1,48738	0,64907	0	0,0313842	1,00789	50,2694	-0,02224	0,930506	0,6279808
CG11105-RB	CG44422	1,40024	2,41402	0,264467	5,49158	5,9447	5,97444	-0,079849	0,749316	0,6279808
CG11106-RA	CG11106	0	78,8456	0	22,1252	50,6492	0,0326604	-0,070526	0,562659	0,6279808
CG11107-RA	Dhx15	12,88	0,0411766	20,6171	16,5585	12,8637	13,921	-0,131065	0,610774	0,6279808
CG11109-RA	CG11109	0,0462069	0,0420885	0,0443614	2,27227	1,07259	0,76853	-0,112439	0,646754	0,6279808
CG11109-RB	CG11109	0,0450722	0,0410549	0,043272	3,91302	1,64846	1,59574	-0,115667	0,636998	0,6279808
CG11110-RA	CG11110	4,91672	6,77235	167,88	22,1061	10,4109	199,109	0,070364	0,812109	0,6279808
CG11111-RA	rdgB	2,41938	0,0144865	2,26855	1,45878	1,5628	0,016793	-0,691395	0,002932	0,6279808
CG11111-RB	rdgB	0,0159809	1,14798	0,0153427	1,6439	35,7022	0,016416	-0,721978	0,002061	0,6279808
CG11111-RC	rdgB	0,015904	6,40428	0,0152688	7,34	22,4963	4,63253	-0,692753	0,002901	0,13772387
CG11111-RD	rdgB	1,10472	5,34871	1,0606	5,24917	78,5098	1,61355	-0,692512	0,002946	0,6279808
CG11112-RC	CG11112	0,259732	0,236582	0	0,227988	9,2102	0,501482	-0,031631	0,912903	0,6279808
CG11113-RB	CG11113	0	0	0	0,407543	0,0553486	0	-0,11534	0,404049	0,6279808
CG11115-RA	Ssl1	3,86758	4,90746	4,89538	6,13905	2636,04	4,04293	-0,044189	0,844702	0,6279808
CG11120-RA	alpha-Est7	1,4906	0,626652	0,0440329	0,0510187	1,0585	0,0520818	-0,006488	0,981237	0,6279808
CG11120-RB	alpha-Est7	4,68929	2,63322	10,6499	5,41639	0,0691051	8,07649	-0,007664	0,977819	0,6279808
CG11121-RA	Mink	0,0265827	0,0242134	0,025521	0,0282008	7,69577	17,1317	0,261845	0,3407	0,6279808
CG11122-RA	Mink	3,73059	3,25245	6,51081	6,19694	1,8784	0,0228673	0,027597	0,922628	0,6279808
CG11123-RA	so	8,39785	8,5025	7,88872	10,2954	3,4015	5,71144	-0,004114	0,986636	0,6279808
CG11124-RA	CG11122	2,5384	3,10007	0,0456052	4,23329	7,06618	6,20105	-0,976536	0,003605	0,13772387
CG11124-RB	CG11123	6,31357	5,89179	12,004	9,74035	0,0300152	6,70661	-0,982449	0,003395	0,6279808
CG11124-RC	sPLA2	0,0723723	0,0659217	0,0694817	0,0927372	0,0484394	0,0946696	-0,941637	0,004731	0,6279808
CG11125-RA	sPLA2	0,0913913	0,0832455	0,0877411	0,126588	0,0307282	0,129226	0,508342	0,051907	0,6279808
CG11127-RA	sPLA2	14,192	8,89786	26,1002	21,6834	0,0307706	44,836	0,106258	0,6501	0,6279808
CG11128-RA	CG11125	3,30172	4,65301	4,44576	3,72884	15,0411	14,5894	0,064972	0,78409	0,6279808
CG11128-RB	CG11127	9,33879	15,1643	11,9476	11,5443	6,32761	9,81635	0,064745	0,787265	0,6279808
CG11128-RC	slif	4,91099	3,40236	0,215283	0,0268341	0,196437	2,50467	0,067171	0,778508	0,6279808
CG11129-RA	slif	0,0253683	0,0231072	0	12,399	2,90192	0,0273933	-0,282185	0,335109	0,6279808
CG1112-RA	slif	15,4811	14,5264	13,9892	120,056	0,0363469	15,0048	-0,377826	0,263864	0,6279808
CG1112-RB	Yp3	1,10274	307,724	19,4741	402,325	18,6502	18,5485	-0,352104	0,296948	0,6279808
CG11130-RA	CG1113	0,489016	0,00871931	0,563382	0,829195	0,286967	26,473	-0,479157	0,130838	0,6279808
CG11131-RA	Rtc1	9,53359	22,0929	1,67273	33,222	1,32903	1,75487	-0,340663	0,308846	0,6279808
CG11132-RA	CG11131	55,2489	8,66429	0,024355	0	0	0	0,118182	0,635431	0,6279808
CG11133-RA	DMAP1	12,2124	0,0505061	2,30156	12,551	0	0,0645212	-0,017345	0,961041	0,6279808
CG11134-RA	BoYb	0,112088	0,127622	0,493218	0,134933	0,287508	0,26719	-0,501813	0,115028	0,6279808
CG11136-RA	CG11134	27,7092	3,34369	48,6184	2,29542	4,36984	15,3365	0,160813	0,534698	0,6279808
CG11137-RA	Dpse\GA10784	8,60363	9,24113	9,03974	0,0631436	0,0855284	0	-0,391254	0,210501	0,6279808
CG11138-RB	CG11137	49,1062	37,0864	995,827	951,201	3,66665	2370,41	0,447666	0,083435	0,6279808
CG11138-RC	Pits	17,9131	6,40714	0,0249784	0,0123751	0,024802	0,0186923	0,40649	0,10979	0,6279808
CG11138-RD	Pits	26,8307	0,0527027	31,7983	3,91491	38,3157	0,161816	0,40665	0,109648	0,6279808
CG11139-RA	Pits	0,0176312	3,84399	7,4902	0,232276	7,19863	6,97879	-0,044273	0,866934	0,6279808
CG1113-RA	p47	14,2765	0,0219259	26,9167	19,6704	2,02635	2,21173	-0,042669	0,901174	0,6279808
CG11140-RE	Aldh-III	0,0309247	0,0281684	0,0296896	16,122	18,624	18,8387	-0,295584	0,268873	0,6279808
CG11140-RI	Aldh-III	0,0318642	0,0290242	0,0305916	0,034238	0,0463755	0,0349514	-0,256812	0,329248	0,6279808
CG11140-RJ	Aldh-III	7,67247	6,08343	10,565	7,04108	9,13657	0,031856	-0,298159	0,264476	0,6279808
CG11140-RK	Aldh-III	24,4915	15,8672	38,2469	26,6811	34,8495	32,3423	-0,256667	0,32945	0,6279808
CG11140-RL	Aldh-III	24,8529	23,6928	33,8841	11,3953	10,7426	21,2373	-0,256962	0,329319	0,6279808
CG11140-RM	Aldh-III	0,0290358	0,0264478	0,0278761	0,0309857	0,0419703	0,0316313	-0,253773	0,335257	0,6279808
CG11140-RN	Aldh-III	0,0252519	0,0345018	0,036365	0,0267036	0,0361702	0,02726	-0,257717	0,327929	0,6279808
CG11140-RO	Aldh-III	0,0217593	0,0198199	0,0208903	0,0228195	0,0309091	0,023295	-0,260173	0,322831	0,6279808
CG11141-RA	CG11141	0,0240714	5,03075	0,0231099	0,0259159	0,0351032	0,0264559	-0,015366	0,947235	0,6279808
CG11141-RB	CG11141	3,46911	9,56491	5,11005	6,05893	3,6749	3,63434	-0,015366	0,947235	0,6279808
CG11142-RA	obst-E	94,4953	8,51591	0	14,4243	9,08021	86,9169	-0,116729	0,669979	0,6279808

gene_id	Symbol	W1_FPKM	W2_FPKM	W3_FPKM	MCM51_FPKM	MCM52_FPKM	MCM53_FPKM	FC	p-value	p-adj
CG11142-RB	obst-E	142,816	3,68288	7,21539	0,025299	4,04955	189,083	0,28735	0,254919	0,6279808
CG11143-RA	Inos	4,28177	7,66369	11,9324	18,1009	10,7383	11,5041	-0,698106	0,014875	0,6279808
CG11144-RA	mGluR	3,3217	2,29187	3,13174	4,03848	3,39124	2,00938	-0,174914	0,519889	0,6279808
CG11145-RA	CG11145	0	0	0	0	0	0	NA	NA	0,6279808
CG11147-RA	CG11147	0,0222353	0,0202534	0,0213472	0,023345	0,0316209	0,0238314	0,746855	0,014884	0,6279808
CG11147-RB	CG11147	23,2856	28,0342	14,5876	18,2256	5,60775	9,42477	0,746855	0,014884	0,6279808
CG11148-RA	Gyf	20,826	20,9201	18,9858	20,4154	2,20678	2,44065	0,170886	0,39683	0,6279808
CG11148-RB	Gyf	0,0134092	0,012214	0,0128736	0,0137891	3,6425	2,57714	0,170886	0,39683	0,6279808
CG11148-RC	Gyf	14,0632	14,9009	15,9763	20,1291	2,82516	2,15946	0,170809	0,397085	0,6279808
CG11149-RA	CG11149	3,26692	3,05323	4,80268	2,48125	2,67352	1,06828	0,590949	0,023205	0,6279808
CG11149-RB	CG11149	0,0348562	1,49223	0,0334641	0,0377274	0,051102	1,46942	0,594597	0,020828	0,13772387
CG11151-RA	Vps37B	41,0992	0,0661631	10,9458	0,0866402	67,1818	57,4032	-0,313659	0,263496	0,6279808
CG11152-RA	CG11151	364,023	6,62837	376,281	13,1721	2,72019	6,83382	0,359448	0,185989	0,6279808
CG11153-RA	Dsim\GD24370	2,06577	3,3866	5,38242	2,43077	1,56979	1,24914	0,410434	0,072515	0,6279808
CG11153-RB	Dsim\Sox102F	5,14648	2,05152	2,30003	3,27131	1,12011	2,18782	0,509611	0,026685	0,6279808
CG11154-RA	Dsim\Sox102F	3,80934	0	0	5,61949	0	3,35715	-0,356799	0,201387	0,6279808
CG11154-RB	Dpse\ATPsyn-beta	0,0923275	0	0,0886399	0,116309	0,157542	0,118733	-0,53381	0,079721	0,6279808
CG11155-RA	Dpse\ATPsyn-beta	711,432	0	1179,47	872,086	1187,15	1039,3	0,153157	0,5438	0,6279808
CG11155-RB	CG11155	0,0369207	0,03363	0,0354461	31,4285	54,9189	19,6393	0,044028	0,864772	0,6279808
CG11155-RC	CG11155	8,41228	6,79804	8,44782	42,0534	42,4398	0,041002	-0,06222	0,820131	0,6279808
CG11156-RA	CG11155	39,4288	31,4329	40,5625	10,5161	16,821	5,49483	-0,270619	0,389567	0,6279808
CG11158-RA	mus101	0,390374	0,617585	0,460259	15,2512	35,8559	0,551095	0,007695	0,975538	0,6279808
CG11159-RA	CG11158	12,4514	11,0517	12,9089	10,5014	11,3924	12,6398	0,23541	0,497543	0,6279808
CG1115-RA	Dpse\GA10804	2,62206	182,528	0,0531889	16,406	9,87928	8,69159	-0,360947	0,120593	0,6279808
CG11160-RA	CG1116	5,99292	8,64623	7,26236	8,56211	0,0132154	9,05509	-0,803978	0,002107	0,6279808
CG11160-RB	CG1116	1,63755	0,038246	1,18919	0,041506	0,0152552	0,00995995	-0,803226	0,002018	0,6279808
CG11162-RA	CG1116	0,0437395	0,039841	0,0419925	2,58435	9,97832	0,0114972	0,143539	0,646107	0,6279808
CG11163-RA	SmydA-4	3,63288	0,074983	3,83307	5,13009	6,35737	7,9918	-0,406016	0,072607	0,6279808
CG11163-RB	SmydA-4	5,40209	0,906342	0,0830685	0,107224	0,0266697	3,50191	-0,424469	0,060514	0,6279808
CG11163-RC	CG11162	0,868381	0	0,947383	4,26919	0,766517	1,63117	-0,408796	0,070609	0,6279808
CG11163-RD	ZnT41F	0,0346915	0,0299932	4,2833	0,0354729	5,57382	1,71112	-0,407804	0,071764	0,6279808
CG11164-RA	ZnT41F	3,34955	9,08101	0,031613	13,0844	0,0480483	2,72141	0,205497	0,536593	0,6279808
CG11165-RA	ZnT41F	0,0329282	1,62408	13,025	8,98998	13,2471	0,0431593	0,020887	0,887016	0,6279808
CG11166-RA	ZnT41F	11,5113	0,0352448	2,82761	1,61607	2957,49	0,177736	0,30581	0,275293	0,6279808
CG11166-RB	CG11164	3,21852	2,0372	1,86579	2,41618	1,34887	15,8003	0,34178	0,221176	0,6279808
CG11166-RC	azot	0,102443	13,4932	26,1103	24,4833	0,0643702	18,1549	0,30581	0,275293	0,6279808
CG11166-RD	Eaf	3,08937	3,63316	2,16354	5,33539	0,308811	1,94629	0,310891	0,266995	0,6279808
CG11166-RE	Eaf	2,9195	4,54798	3,64695	0,0357617	0	0,0365069	0,311593	0,26698	0,6279808
CG11166-RF	Eaf	0,0216382	0,0197096	0,020774	0,0226859	5,65503	0,0231586	0,304518	0,277368	0,6279808
CG11166-RG	Eaf	0,0324999	0,0197354	0,0312018	0,0227172	0,125613	0,0231906	0,30581	0,275293	0,6279808
CG11168-RA	Eaf	0,021695	0,0197613	0,0208285	0,0227486	0,171464	0,0232226	-0,325902	0,201721	0,6279808
CG1116-RA	Eaf	0,0263076	0,0239627	0,0252568	0,0278904	54,9101	0,0284716	-0,12037	0,57109	0,6279808
CG1116-RB	Eaf	13,7357	21,0732	13,6745	22,3497	19,3703	10,0071	-0,12037	0,57109	0,13772387
CG1116-RC	CG11168	8,82505	0,311363	3,43786	5,28857	0,0303417	0,599009	-0,12037	0,57109	0,6279808
CG11170-RB	CG11170	3,19817	0,561616	1,68317	1,08776	0,914695	0,68937	0,407808	0,181781	0,6279808
CG11172-RA	NFAT	5,00293	3,84676	5,14736	23,654	3,96038	3,96879	-0,248892	0,237805	0,6279808
CG11172-RB	NFAT	5,47022	4,29929	6,82272	5,83601	6,19847	5,72948	-0,236127	0,267702	0,6279808
CG11173-RA	Snap29	27,9811	0	8,13248	0,781464	5,59183	11,0435	-0,346061	0,174177	0,6279808
CG11175-RA	Rcd6	15,8444	9,53993	0,0488476	2,38385	0,0497628	11,2625	0,319253	0,29392	0,6279808
CG11176-RA	Tango2	12,0942	11,9464	15,2492	11,0705	10,4542	10,7128	0,174527	0,43662	0,6279808
CG11177-RA	BthD	0,0664747	0,0605498	11,6152	27,4719	7,11314	1,42089	-0,202505	0,495254	0,6279808
CG11178-RA	CG11178	0,696254	1,04456	0,0196602	0,0214106	0,0290008	0,0218568	0,207242	0,36744	0,6279808
CG11178-RB	CG11178	5,12792	5,88935	5,29769	6,65719	3,83161	4,19493	0,213111	0,356314	0,6279808
CG11180-RA	CG11180	17,2239	21,6362	30,0819	0,0476605	4,2689	4,2534	0,388531	0,185202	0,6279808
CG11181-RB	cup	7,63889	2,46383	7,18167	102,614	8,00523	3,68903	-0,127686	0,5949	0,6279808
CG11182-RA	PHDP	3,06627	3,38303	2,40479	2,53812	13,4195	28,9712	0,430804	0,180472	0,6279808
CG11183-RA	DCP1	5,41483	5,36405	57,089	6,77544	35,2667	2,51923	-0,135498	0,640064	0,6279808
CG11184-RB	Upf3	5,15945	8,92847	0,0711439	2,50627	1,61601	1,09897	-0,065402	0,796418	0,6279808
CG11184-RC	Upf3	0,0403722	1,65826	3,37344	3,52544	1,55551	1,97238	-0,120963	0,633895	0,6279808
CG11186-RA	toy	6,05001	6,47941	5,57383	9,20653	4,248	3,44524	0,069313	0,799109	0,6279808
CG11188-RA	Aatf	9,0898	9,85417	16,8009	6,48622	5,29523	9,59332	0,141978	0,569195	0,6279808
CG11190-RA	Gnf1	0,0205609	59,7566	0,0197397	0,0962596	17,1006	0	-0,097178	0,667904	0,6279808
CG11191-RA	Gnf1	4,90745	0,0345766	5,6779	0,0449564	6,34087	0	-0,199669	0,463359	0,6279808
CG11192-RB	PIG-T	8,59091	19,7119	12,7273	9,68959	86,7974	9,05648	-0,486339	0,103775	0,6279808
CG11194-RA	CG11191	2,69386	6,32389	4,0502	4,19434	4,25571	1,99655	0,229009	0,438046	0,6279808
CG11196-RA	CG11192	0	0,158552	0,200538	0,0409387	0	0	0,044335	0,715924	0,6279808
CG11198-RA	Hey	2,53113	0,0256367	2,19252	0	1,11589	3,98005	-0,562203	0,007527	0,6279808
CG11198-RB	37956	0	0	0	11,282	4,64303	5,74693	-0,560389	0,007743	0,6279808
CG11198-RC	ACC	0,00839841	0,00757666	0,00806297	9,59262	7,94172	6,35239	-0,560359	0,007767	0,6279808
CG11198-RD	ACC	17,7341	0,00733028	18,447	0,0179859	0,024362	0,0183607	-0,563542	0,007336	0,6279808
CG11199-RA	ACC	0,00831806	10,0944	0,00798583	35,228	10,0318	12,4931	-0,226618	0,408383	0,6279808
CG11199-RB	ACC	0,00804756	0,0448929	0,00772614	0,00853683	0,0115632	0,00871471	-0,225383	0,410744	0,6279808
CG1119-RA	Liprin-alpha	8,23388	9,17196	7,09941	0,199735	0,0486507	0,616652	-0,127827	0,538963	0,6279808
CG1119-RB	Liprin-alpha	0,0142846	0,0130114	0,0137141	4,04554	6,89941	0,473612	-0,127827	0,538963	0,6279808
CG11200-RA	IntS10	1,82087	1,67078	0	0	0	0,0197807	0,407269	0,124034	0,13772387
CG11200-RB	CG11200	0,0384386	2529,49	0,107703	0	0,251202	2,57204	0,407269	0,124034	0,6279808
CG11201-RB	CG11200	39,2375	0,214441	0,059752	0,886091	295,623	0,546261	0,564968	0,113001	0,6279808
CG11202-RA	TTLL3B	0,237846	0,309495	0,173978	9,78157	0,718513	0,0598721	0,001877	0,995048	0,6279808
CG11203-RA	org-1	3,64991	5,34513	3,11142	6,82218	4,46253	2,78546	-0,614209	0,040789	0,6279808
CG11203-RB	CG43901	0,021133	0,570167	0,509287	7,6683	15,3209	1,46567	-0,569692	0,058371	0,6279808
CG11205-RA	CG43901	1,1581	0,0192494	0,0202889	0,0221297	48,4517	0,459614	2,566572	3,47E-27	0,6279808
CG11205-RB	phr	6,34529	11,9811	0,00823569	0,00872331	0,0118158	0,00891925	2,566572	3,47E-27	0,6279808
CG11206-RA	phr	5,21151	0,0346497	0,0082389	0,00872677	0,0118205	0,00890862	0,170156	0,494283	0,6279808
CG11206-RB	Liprin-gamma	0,0152612	0,0177245	6,46115	12,8115	24,2507	0,020719	0,160337	0,522364	0,6279808
CG11206-RC	Liprin-gamma	4,26398	0,156736	6,10195	0,0202961	0,23487	0	0,153361	0,541	0,6279808
CG11206-RD	Liprin-gamma	11,3527	3,83906	1,57701	0	4,42667	4,57687	-0,240362	0,353952	0,6279808
CG11207-RA	Liprin-gamma	0,0291883	2,97856	123,713	3,68804	1,49237	2,89667	-0,186595	0,544434	0,6279808
CG11208-RA	feo	2,82794	2,40251	5,16892	1,60268	3,29528	4,40749	-0,594103	0,061041	0,6279808
CG11209-RA	Dsim\GD25296	24,2512	17,2566	37,6996	0,0283098	4,27736	0,0288997	-0,256008	0,459795	0,6279808
CG1120-RA	ppk6	0,0634126	24,7706	0,324692	21,1311	2,7795	2,5811	0,325533	0,226113	0,6279808
CG11210-RA	alpha-Est8	5,13368	0,325684	6,18779	1,01584	0,515859	10,3734	-0,3154	0,135349	0,6279808
CG11211-RA	CG11210	11,3081	10,7406	11,8622	16,8774	11,144	10,1388	0,565257	0,114251	0,6279808
CG11212-RA	CG11211	1,32152	21,1417	1,42733	1,16531	0,542478	0,677006	0,438356	0,177801	0,6279808
CG11213-RA	Ptr	19,9459	0,183682	5,485	5,71791	0,0267657	0,898452	-0,064618	0,856264	0,6279808
CG11213-RB	Cp38	0,312071	0,312681	16,0803	8,80657	0,114355	0,0739562	-0,071606	0,841167	0,6279808
CG11217-RA	Cp38	0,0552627	0,377528	0,0307995	4,82242	0,144027	0,3705	0,020302	0,922122	0,6279808
CG11218-RA	CanB2	55,0439	23,2963	63,5202	63,0047	60,4659	0,00890508	-0,583847	0,054633	0,6279808
CG11219-RA	Obp56d	48,8363	0	0,0374995	0,0502024	0,0578603	6,57819	0,132208	0,697462	0,6279808
CG1121-RA	PIP82	0,200706	0,429222	0,444024	17,4773	33,0326	5,9279	0,008455	0,976607	0,6279808
CG11221-RA	meng	2,31513	0,39215	0,23362	0,0272559	113,696	1,72098	-0,186006	0,495762	0,6279808
CG11226-RA	CG11226	0	0	0,0862694	0,0146182	4,78068	0,307639	-0,170106	0,488247	0,6279808
CG11227-RA	CG11227	0	1,27087	0,0202114	0	3,49476	2,79266	0,017269	0,912244	0,6279808
CG11227-RB	CG11227	0	0,0191758	0,0510653	0	0,0298545	0	0,017269	0,912244	0,6279808
CG11227-RC	CG11227	0	0,0193796	0,0503867	0	0,0301877	0	-0,013952	0,911565	0,6279808

gene_id	Symbol	W1_FPKM	W2_FPKM	W3_FPKM	MCM51_FPKM	MCM52_FPKM	MCM53_FPKM	FC	p-value	p-adj
CG11228-RA	hpo	7,29068	0	0,0624582	22,2218	14,4546	0,0776262	-0,19557	0,3996	0,6279808
CG11229-RA	CG11229	0	0	0	0	0,0249756	0,0188231	-0,041811	0,731452	0,6279808
CG11231-RA	CG11231	0	0	0	0	0	0	NA	NA	0,6279808
CG11233-RB	Npc1b	0,243822	0	0,063649	0,0764688	0,219282	0	0,456424	0,135656	0,6279808
CG11235-RA	CR11235	0	0,333136	0	0,176512	2,57166	2,67401	-0,041811	0,731452	0,13772387
CG11236-RA	CG11236	2,62251	0	0,196845	0	5,26591	0,492935	0,212268	0,507312	0,13772387
CG11237-RA	Oseg6	0,744804	0,621289	0,715056	1,51296	10,8064	15,0598	-0,250747	0,427653	0,13772387
CG11238-RA	l(3)04053	3,90412	3,77536	2,40921	12,1043	3,4097	0,441006	0,187649	0,403254	0,6279808
CG11241-RA	CG1124	65,1136	54,3697	0,281316	66,8744	12,243	66,3521	-0,179592	0,373782	0,6279808
CG11241-RB	CG11241	0,0400055	0,0327278	0,307946	1,89725	2,44823	1,91378	-0,299293	0,140433	0,13772387
CG11242-RA	CG11241	9,81898	1,332	0,0384077	0,0423327	0,0545368	0,0432148	0,512014	0,031582	0,13772387
CG11246-RA	TBCB	21,855	23,2421	19,3744	19,0719	9,17037	8,02726	-0,201583	0,51358	0,13772387
CG11247-RA	Rpb8	26,161	20,1498	52,2638	9,93579	3,42267	1,68293	0,272508	0,227022	0,6279808
CG11247-RB	CG11247	5,50288	4,14509	9,1178	33,0553	46,3342	53,6117	0,272508	0,227022	0,6279808
CG11247-RC	CG11247	8,04978	8,83236	3,44041	52,0403	53,7375	60,7697	0,281515	0,212511	0,6279808
CG11248-RA	CG11247	0,0252809	0,0230276	0,0242712	176,757	3,8769	6,62548	0,082042	0,717148	0,6279808
CG11248-RB	CG11248	5,25586	3,58485	0,0308444	0,0345431	5,86404	3,45025	0,082772	0,714421	0,6279808
CG11249-RA	CG11248	2,65985	5,81866	5,16587	6,37883	0,0326624	1,72043	1,102188	0,000418	0,6279808
CG1124-RA	CG11249	0,0321276	11,1604	5,42718	6,34171	2,84576	5,49547	-0,012664	0,964005	0,6279808
CG11251-RA	CG11251	0	0	0,0684078	54,1782	103,53	30,563	-0,039118	0,754991	0,6279808
CG11253-RA	Zmynd10	0,0456115	0,0415461	0,0437897	0,0507046	0,0686797	0,0517612	0,204469	0,412118	0,6279808
CG11254-RA	mael	0,0359303	0,0229573	18,1375	526,951	2,74055	0,0272049	0,421101	0,207102	0,6279808
CG11254-RB	mael	0,036107	0,0228354	6,0446	0,346541	0,0198004	0,0270517	0,421101	0,207102	0,6279808
CG11254-RC	mael	1,65284	16,3284	5,05749	2,1839	5,50026	16,0441	0,421101	0,207102	0,6279808
CG11255-RA	AdenoK	55,2545	34,5526	70,0899	23,6162	52,1909	43,8502	-0,054238	0,865823	0,6279808
CG11255-RB	AdenoK	12,7241	7,47247	20,333	13,9423	29,7777	23,0148	-0,040977	0,8985	0,6279808
CG11257-RA	CG11257	2,19842	8,23705	10,1166	0,0201656	2,47699	7,83926	0,166239	0,560229	0,13772387
CG11258-RA	mRpL20	56,0836	31,3033	8,3953	7,16946	6,60033	6,73338	-0,599699	0,055552	0,6279808
CG11259-RA	MICAL-like	9,25816	8,46006	11,2865	10,6857	0,758361	0,809258	0,191271	0,352031	0,6279808
CG11260-RA	BBS5	1,03784	1,18167	0	0,0414017	2,50236	32,2063	0,01562	0,897983	0,6279808
CG11261-RA	CG11260	0	0,227979	0	0	0,0403754	0	0,480993	0,139305	0,6279808
CG11262-RA	Cul6	1,17901	0,72525	1,86694	0,792739	4,92452	4,44179	0,066601	0,72269	0,6279808
CG11263-RA	CG11262	0,0459286	0,0418349	0,140512	0	0,126076	0,187813	0,772639	0,027253	0,6279808
CG11265-RC	CG11263	0,468402	0	0	0	0,123563	0,068671	0,047785	0,830982	0,6279808
CG11265-RE	Trf4-1	0,0163811	0,014921	0,0157268	4,13932	2,55669	2,22168	0,042492	0,848881	0,6279808
CG11265-RF	Trf4-1	5,08837	4,68088	4,48883	6,16207	4,82737	1,65873	0,042619	0,848543	0,6279808
CG11265-RG	Trf4-1	2,13578	2,891	1,90343	2,71464	0,0239052	0,0496228	0,046973	0,834037	0,6279808
CG11266-RA	Trf4-1	2,86514	3,07254	8,10818	2,30018	0,0233792	5,6916	-0,055424	0,809397	0,13772387
CG11266-RB	Caper	0,0316203	11,8631	0,0159844	4,86684	4,19222	3,89557	-0,055104	0,810469	0,6279808
CG11266-RC	Caper	0,0270175	20,6186	1,85862	15,3255	7,84628	0,0330455	-0,007524	0,974657	0,13772387
CG11266-RD	Caper	0,0262136	22,7657	4,59658	3,82116	8,80621	8,39736	-0,00781	0,973694	0,6279808
CG11266-RE	Caper	8,33407	4,86243	17,715	0,0323709	2,1057	0,0370498	-0,020379	0,928956	0,6279808
CG11266-RF	Caper	7,616	12,7337	0,0322888	0,0339557	5,11297	0,0346632	-0,019849	0,933174	0,6279808
CG11266-RG	Caper	8,29909	0,0485897	0,0303573	0,028692	0,0819174	0,0292898	-0,020548	0,93084	0,6279808
CG11267-RA	Caper	17,2256	11,7151	0,0259384	0,0277846	9,82346	0,0283635	-0,518837	0,070998	0,6279808
CG11268-RA	CG11267	137,518	6,22201	52,0692	61,3335	320,77	306,97	-0,00411	0,986301	0,6279808
CG11269-RA	ste14	6,29927	99,4452	234,212	268,519	114,139	110,391	-0,123353	0,484383	0,6279808
CG1126-RA	CG11269	0	0,87186	1,9557	0,0208418	0,0284372	0,422536	0,19187	0,540981	0,6279808
CG11271-RB	RpS12	1368,8	1593,68	2267,17	2329,21	3849,83	4229,11	-0,555736	0,061111	0,6279808
CG11271-RC	RpS12	0,106746	0,0972318	0,102483	0,158195	0,214277	0,161492	-0,555774	0,061092	0,6279808
CG11274-RA	SRm160	11,144	11,4927	14,7696	18,8181	5,72089	8,64065	0,094829	0,732407	0,6279808
CG11275-RA	CG11275	7,89437	3,80297	5,51179	0,142593	4,64192	2,13515	-0,499651	0,116102	0,6279808
CG11276-RA	RpS4	0,0710747	0,293593	0,309448	2,40913	0,114348	0,0861793	-0,2861	0,256268	0,6279808
CG11276-RB	RpS4	1520,4	0,0846505	0,0892219	0,129575	2430,97	2745,11	-0,28609	0,256284	0,6279808
CG11278-RA	Syx13	17,4373	23,689	0,366158	0	0,244513	0	-0,500052	0,065798	0,13772387
CG11279-RA	CG11279	43,093	0,120373	0,126874	0	0	0	-0,596068	0,059847	0,6279808
CG11280-RA	alpha-Est9	0,0396693	1,49954	0,0380849	0,781464	0,0588523	0,164449	0,260113	0,349057	0,6279808
CG11281-RA	alpha-Est9	8,40468	4,23857	15,3311	0,0434492	16,9333	0,596373	0,01562	0,897983	0,6279808
CG11282-RA	trn	30,071	0	0	0	0	0	-0,779781	0,000207	0,6279808
CG11282-RB	snky	0	0	0,229843	0	0	0	-0,777316	0,000209	0,6279808
CG11282-RC	caps	0,0245119	0,0223271	0,0235329	0,0258752	0,0350481	0,0264144	-0,03224	0,895996	0,6279808
CG11284-RA	caps	0,0227169	0,0206921	4,80901	13,5518	9,68423	6,48968	-0,334043	0,11294	0,6279808
CG11286-RA	caps	30,8966	37,5237	20,8077	27,5699	21,6785	20,3188	0,044335	0,715924	0,6279808
CG11289-RB	CAH3	17,2252	0,0556423	0	16,7782	4,04144	4,55926	0,529412	0,060184	0,6279808
CG1128-RA	CG11286	0	0	0,152737	0	0	0	-0,355461	0,265726	0,6279808
CG1128-RB	Ugt307A1	2,1181	2,92957	6,20129	0,0383198	3,78057	3,64091	-0,355461	0,265726	0,6279808
CG11290-RA	CG1129	2,8941	4,13775	0	0,015018	17,6602	0,0144329	0,135466	0,640335	0,13772387
CG11291-RA	CG1129	23,5947	21,0348	9,17963	0,015397	3,76479	0,0153309	0,186034	0,345304	0,6279808
CG11293-RA	enok	4,51185	6,40199	5,48281	21750,8	2,41789	2,7789	0,633137	0,057914	0,6279808
CG11294-RA	CG11291	0	0,0180903	4,19802	0,0980997	0,0592823	0,021528	-0,269637	0,288322	0,6279808
CG11295-RA	St3	2,00723	3,11309	65,0882	3,61035	0,0210969	0,0177455	0,239919	0,352608	0,6279808
CG11298-RA	CG11294	0,174619	3,40809	4,98501	9,31644	0,0957091	0,0721321	0,01562	0,897983	0,13772387
CG11299-RA	l(2)dtl	2,72928	13,4502	19,3317	56,0312	16,2251	0,0726777	0,274614	0,394121	0,6279808
CG11299-RB	CG11298	0	3,39816	10,6753	0,825995	5,39017	2,36386	0,273597	0,396276	0,6279808
CG11299-RC	Sesn	13,8757	0,800826	0,299064	2,7547	2,44563	3,84262	0,274604	0,394169	0,6279808
CG1129-RA	Sesn	40,2972	30,8865	5,20241	33,2809	53,271	2,52163	-0,244925	0,211495	0,6279808
CG1129-RB	Sesn	0,0218312	14,2475	57,9116	7,00676	0,568249	0	-0,246475	0,209541	0,13772387
CG11300-RA	scrt	12,7669	19,7949	15,1231	0,0222418	8,66514	2,06796	-0,044651	0,81777	0,6279808
CG11301-RA	CG11300	0	16,2574	80,1845	43,1246	12,2715	3,467	0,000768	0,997308	0,6279808
CG11303-RA	Mes4	9,79241	9,54481	18,3479	132,287	0	11,0862	-0,244128	0,458291	0,6279808
CG11303-RB	TM4SF	0,0304357	0,0399645	0,0292201	0,0396098	30,8986	36,2773	-0,257249	0,432641	0,13772387
CG11305-RA	TM4SF	5,63795	23,2266	10,299	11,3594	91,7348	70,922	0,253107	0,27272	0,6279808
CG11306-RA	Sirt7	2,72701	3,10494	3,39729	3,39405	2,03514	4,10215	-0,238154	0,244437	0,6279808
CG11307-RA	Alg11	33,4838	31,3504	36,7534	41,949	4,53094	4,70478	0,68856	0,027235	0,6279808
CG11307-RB	CG11307	3,56506	4,89139	4,5798	0,886716	0,666173	2,69567	0,691939	0,026089	0,6279808
CG11308-RA	CG11307	0,0441192	2,92487	6,94243	2,32291	1,88316	0,0498804	0,003405	0,987196	0,6279808
CG11309-RA	sa	0	0	0,114922	0,158008	0,214023	0	0,125446	0,689646	0,6279808
CG11309-RB	CG11309	11,3373	7,79662	26,6619	3,27981	20,9744	17,0467	0,100604	0,749662	0,6279808
CG1130-RA	CG11309	16,1617	9,1925	611,624	12,7509	680,177	623,503	0,554416	0,059664	0,6279808
CG11310-RA	alpha-Est10	15,9407	0	0	0,0807812	779,235	0,132833	0,024728	0,942191	0,6279808
CG11312-RA	Cpr78Ca	2,58109	0,0235226	0,0247929	0,0273464	21,2191	0,0279162	0,152091	0,593142	0,6279808
CG11313-RA	insc	9,46711	12,8159	7,81022	13,6201	1,58515	13,7841	0,306862	0,381047	0,6279808
CG11314-RA	CG11313	0,445255	0,621873	0,0235503	19,0961	250,336	0	0,129153	0,607093	0,6279808
CG11315-RA	Npc2g	46,7452	54,2867	0,0258433	0,125145	0,0713138	89,7026	1,646904	1,68E-10	0,13772387
CG11315-RB	Npc2h	8,03388	10,1415	5,03813	82,4945	0,0691051	7,17137	1,656636	9,89E-11	0,6279808
CG11317-RA	Npc2h	0,0977933	0,0890769	78,3319	76,9491	0,0726489	0,127753	-0,196027	0,443977	0,6279808
CG11318-RA	CG11317	6,37961	6,11243	7,69573	1,58235	8,51496	0	-0,172708	0,565217	0,6279808
CG11319-RA	CG11318	3,09169	2,18304	0,0366545	0	4,15973	6,48688	0,398454	0,074165	0,6279808
CG1131-RB	CG11319	2,70395	315,891	0,786998	0,590563	0,750186	0,525975	0,704073	0,018079	0,6279808
CG11320-RB	FoxL1	1,89562	2,08296	2,45543	2,40532	107,408	124,597	0,20925	0,545356	0,6279808
CG11321-RB	DIP-iota	0,621014	0,882433	0,0292111	367,478	0,0570102	0,0429663	0,407588	0,1795	0,6279808
CG11321-RC	LUBEL	2,81265	2,8658	2,46013	16,1303	3,08867	1,46688	0,530959	0,06238	0,6279808
CG11322-RB	LUBEL	2,26546	4,45536	2,42213	0,0147194	0,192207	1,78386	0,044613	0,898829	0,6279808
CG11323-RA	CG11322	0,299494	0,301267	0,105846	0,141902	0,059599	7,65676	-0,672104	0,048342	0,6279808

gene_id	Symbol	W1_FPKM	W2_FPKM	W3_FPKM	MCM51_FPKM	MCM52_FPKM	MCM53_FPKM	FC	p-value	p-adj
CG11324-RA	TTLL3A	0,266167	4,19747	0,0359687	0,0439418	1,31589	0,410199	-0,527447	0,020318	0,6279808
CG11324-RB	homer	5,95694	0,0365061	0,0384776	9,82991	6,35533	0,234594	-0,532823	0,019559	0,6279808
CG11324-RC	homer	0,0400783	4,48655	9,27056	0,636034	0,0199375	6,0304	-0,504063	0,028803	0,6279808
CG11325-RA	homer	3,56129	0,313156	0,31942	0,48596	1,03058	0,0150261	-0,47385	0,106227	0,6279808
CG11325-RB	AkhR	2,30419	0,0365505	3,94342	2,89823	6,18323	0,0449174	-0,452143	0,123754	0,6279808
CG11325-RC	AkhR	0,805121	18,6408	0,0322068	1,16791	0,0490248	37,2933	-0,47385	0,106227	0,6279808
CG11326-RA	AkhR	0,747445	58,8614	0,618613	0,0272898	0,0369641	53,2133	0,226055	0,338482	0,6279808
CG11326-RC	Tsp	9,49645	0,0287836	0,0273788	7,89277	0,0422085	0,0356177	0,288874	0,142649	0,6279808
CG11326-RD	Tsp	0,0180848	16,1961	0,0173625	0,0188019	0,0254673	0,0318109	0,289309	0,141983	0,6279808
CG11326-RE	Tsp	0,0159616	0,0164729	0,0153241	5,18717	2,54484	0,0191937	0,28358	0,150858	0,6279808
CG11326-RF	Tsp	0,0311727	0,014539	12,1806	0,0334387	7,48397	0,0168561	0,23186	0,327897	0,6279808
CG11326-RG	Tsp	0,0317265	0,0283943	0,0304594	0,0340786	0,0461597	11,6751	0,539139	0,095865	0,6279808
CG11327-RA	Tsp	45,5598	0,0288987	46,9201	47,4318	20,8328	0,0347887	0,310551	0,385089	0,6279808
CG11327-RB	CG11327	0,0366071	91,145	0,035145	0,0411175	0,0556939	85,9523	0,682259	0,037114	0,6279808
CG11328-RD	CG11327	0,0316001	0,0333443	0,030338	0,0348906	0,0472596	0,0419743	0,055806	0,82357	0,6279808
CG11328-RE	Nhe3	0,0239521	0,0218172	0,0229954	0,0252505	7,98808	0,0257767	0,067853	0,787409	0,6279808
CG11328-RG	Nhe3	0,0200798	0,01829	0,0192778	0,0209745	0,0342019	0,0214115	0,068222	0,786246	0,6279808
CG11328-RH	Nhe3	5,68162	5,06607	3,69078	7,01264	0,02841	3,4266	-0,032403	0,896036	0,6279808
CG11329-RA	Nhe3	6,32032	8,69655	5,55293	7,5363	3,89491	4,37025	-0,169269	0,636265	0,6279808
CG1132-RA	Nse1	0,526801	0,582671	1,91466	1,35243	1,08715	1,56237	0,278696	0,346592	0,6279808
CG11330-RA	opa	6,78248	9,91535	0	9185,19	0,0208553	5,72481	-0,124724	0,646707	0,6279808
CG11331-RA	cort	0,040127	7,13274	0,0385243	26,9404	0,0493185	6,22831	-0,148703	0,499688	0,6279808
CG11333-RA	Spn27A	45,1188	0,123841	60,5018	12,9488	0,775074	0,237248	0,033657	0,816273	0,6279808
CG11334-RA	CG11333	0	0	0,255794	0	0,320645	0,0121493	0,218634	0,406301	0,6279808
CG11334-RB	CG11334	18,6739	18,1916	31,2415	16,3177	16,4204	15,7883	0,2262	0,38901	0,6279808
CG11335-RA	CG11334	4,56891	4,01746	3,84355	2,43835	5,13835	0,0308197	-0,847354	0,010906	0,6279808
CG11337-RA	Loxl1	0,546082	1,24352	1,07767	3,31712	1,643	4,84371	0,202284	0,391211	0,6279808
CG11337-RB	PNPase	0,0277048	0,0252354	17,437	0,0301906	0,0408934	24,1262	0,17306	0,460824	0,6279808
CG11337-RC	PNPase	0,0247475	0,0225418	0,0265982	0,0267036	0,0361702	1174,95	0,183337	0,436231	0,6279808
CG1133-RA	PNPase	0,0243762	0,0222036	0,0237591	0,0257237	0,0348429	0,0421849	0,454154	0,13947	0,6279808
CG11340-RA	Mul1	8,16427	0,11664	0,0225941	0,0247851	8,44729	3,56584	0,787429	0,022829	0,13772387
CG11342-RA	pHCl-2	0,313453	0,103413	1,40436	6,11352	0,128692	8,26875	-0,427354	0,194549	0,6279808
CG11345-RA	CG11342	2,7186	28,8454	3,80444	106,062	2,68081	5,68589	-0,414081	0,126981	0,6279808
CG11347-RA	CG11345	26,1687	23,3521	44,3979	47,4603	53,478	43,3947	0,171825	0,352295	0,6279808
CG11347-RB	DOR	0,0357618	0,0325743	0,0343334	0,0387936	0,0525461	17,8926	0,170801	0,355605	0,6279808
CG11347-RC	DOR	27,6974	35,7145	39,013	41,3157	22,8023	33,4224	0,087664	0,660954	0,6279808
CG11347-RD	DOR	38,0542	38,487	41,1243	41,5718	25,3341	0,037009	0,171787	0,353128	0,6279808
CG11347-RE	DOR	0,0335979	0,0306033	0,032256	0,0362536	8,93837	0,0803141	0,17134	0,354416	0,6279808
CG11347-RF	DOR	0,0669687	0,0609997	0,0642939	0,0786747	0,106565	13,3693	0,223382	0,241015	0,6279808
CG11348-RA	DOR	0,0549868	0,0500858	19,4005	0,0626037	0,0847971	68,1187	0,389366	0,086027	0,6279808
CG11348-RB	nAChRbeta1	36,3855	19,1467	32,5848	36,9783	19,4911	19,7384	-0,164501	0,502958	0,6279808
CG11349-RA	nAChRbeta1	0,0898175	39,1443	0,0862301	0,112343	0,15217	0,114684	-0,060979	0,660487	0,6279808
CG1134-RA	CG11349	0	209,045	352,873	282,898	5,06202	329,76	-0,26959	0,372794	0,6279808
CG11350-RB	Rcd5	22,5073	11,2752	11,3961	9,80053	17,5382	19,6313	-0,258919	0,461828	0,6279808
CG11352-RB	CG11350	1,0339	8,60009	3,63205	6,08924	0,749407	1,17748	0,702985	0,003846	0,6279808
CG11352-RC	jim	0,0140717	64,4481	2,75716	13,3142	0,0196305	1,62353	0,719126	0,002888	0,6279808
CG11352-RD	jim	3,6469	560,479	0,0113186	0,0266496	1,24931	0,0123297	0,503095	0,04217	0,6279808
CG11353-RA	jim	0,0117894	0,0128174	0,113768	0,0264995	0,0163597	1406,81	0,665794	0,028604	0,6279808
CG11353-RB	CG11353	3,1935	0,0210026	15,54	3,60211	0,422961	8,69391	0,665795	0,028357	0,6279808
CG11354-RA	CG11353	20,5755	22,1598	328,864	17,5825	1,16769	4,24687	0,239767	0,386491	0,13772387
CG11356-RA	Lim1	6,63551	9,3427	6,50449	0,0155753	0,0266458	18,4229	0,117193	0,726663	0,6279808
CG11357-RA	CG11356	0,398048	0,0582792	0,127383	34,5427	0,0108531	14,6253	0,22148	0,267491	0,6279808
CG11357-RB	CG11357	4,65143	2,45239	6,69322	7,53997	0	1,74745	0,244481	0,217421	0,6279808
CG11357-RC	CG11357	2,35386	2,38483	0,0241694	0,0266173	5,96428	6,93595	0,196546	0,32967	0,6279808
CG1135-RA	CG11357	12,0605	11,0649	14,2851	9,79674	4,00716	295,231	0,233099	0,407652	0,6279808
CG11360-RA	CG1136	11,6749	0,124181	0,130887	0,122475	0,266006	27,3135	-0,483217	0,100252	0,6279808
CG11362-RA	CG11360	7,3226	6,1272	5,14841	16,4198	57,4239	5,31689	0,122674	0,658176	0,6279808
CG11367-RA	CG11362	0,456746	0,208018	0,219252	0,687653	0,0971679	3,06654	-0,038666	0,855025	0,6279808
CG11368-RA	CG11367	8,65367	0,0342163	10,4356	6,58591	1,22174	4,0959	-0,397702	0,224004	0,6279808
CG11369-RA	CG11368	23,2649	20,4035	2,16579	0,0380542	0,0515447	0,0388472	0,074368	0,563156	0,6279808
CG1136-RA	CG11369	0	0,0750829	0	0,0362536	0,0491057	0,037009	0,012808	0,960364	0,6279808
CG11370-RA	CG1137	1,16092	1,27775	1,09134	9,87314	41,1863	0,0411777	-0,486362	0,137558	0,6279808
CG11371-RB	CG11370	20,212	3,72756	81,0927	77,9057	0,363947	0,537652	0,379373	0,070298	0,6279808
CG11372-RA	dbr	12,6295	12,4575	12,1408	12,1391	6,35268	7,8587	0,315777	0,098017	0,6279808
CG11373-RA	galectin	47,5732	53,7802	50,9884	44,4489	0,793619	35,7669	0,01562	0,897983	0,6279808
CG11374-RB	CG11373	0	0	0	0	1,19653	14,4843	0,384754	0,252442	0,6279808
CG11375-RA	CG11374	0,940779	1,15937	0,929769	1,20405	33,2284	0,598119	0,124776	0,648517	0,6279808
CG11376-RA	polybromo	4,41324	4,67908	12,7857	2,17923	2,76494	2,50407	0,240636	0,402914	0,13772387
CG11377-RA	Zir	12,5854	14,2574	11,1944	15,5809	5,53116	6,59899	0,001387	0,99464	0,6279808
CG11378-RA	CG11377	11,7772	10,3542	23,4769	10,3652	9,25484	11,3162	-0,521251	0,130449	0,6279808
CG11379-RA	CG11378	60,5006	15,4417	12,315	4,59894	3,57077	5,66349	-0,041811	0,731452	0,6279808
CG1137-RA	CG11379	0	28,8111	0	0	0	0,165264	1,235986	0,000333	0,6279808
CG11380-RA	CG1138	0,103351	0,0141994	0,0149662	0	0	0,0162572	0,694427	0,018367	0,13772387
CG11381-RA	CG11380	26,9911	28,7274	19,9908	22,9383	0,439627	0,968376	-0,533456	0,110956	0,6279808
CG11382-RB	CG11381	2,08162	19,0386	1,5197	1,50137	3,68328	11,2007	0,460712	0,048535	0,6279808
CG11384-RA	CG11382	20,5134	0,145762	114,552	0,201495	150,811	2,80435	-0,102154	0,728931	0,6279808
CG11387-RA	CG11384	4,22972	0,185075	7,87053	57,4962	6,0813	0,219093	0,344095	0,204528	0,6279808
CG11387-RB	ct	15,8932	18,6018	7,0551	30,3738	0,0382965	0,00780014	0,138685	0,629351	0,6279808
CG11387-RC	ct	0,00753202	0,00686068	12,6466	21,1677	4,21102	0,0288625	0,344811	0,202941	0,6279808
CG11388-RA	ct	0,0266471	0,024272	0,00723119	0,0104914	4,76776	4,76662	0,548129	0,018707	0,13772387
CG1138-RA	Xxylt	11,6822	0,0149321	13,7341	12,2999	5,66141	7,05837	0,01562	0,897983	0,6279808
CG11390-RA	CG1139	0,425199	0,200622	1,26547	0,185587	0	0	-0,230716	0,448977	0,6279808
CG11391-RA	EbpIII	130,936	17,3006	68,7669	1,72304	207,449	15,7739	0,025747	0,908353	0,6279808
CG11395-RA	CG11391	0,0559018	0,0848653	0,0894484	0,167862	0	0	0,642129	0,001755	0,6279808
CG11396-RA	Gbp2	24,9038	31,6426	21,536	0	3,21115	34,5901	0,247711	0,352276	0,6279808
CG11397-RA	CG11396	5,37202	23,2517	8,19243	28,769	0,0678444	0,173355	0,093532	0,769197	0,6279808
CG11398-RA	glu	1,57335	1,69676	33,0974	3,79522	1,45939	1,88834	0,367143	0,269001	0,6279808
CG11399-RB	CG11398	1,26634	1,17407	24,4606	20,6121	6,72971	13,3555	0,42141	0,074971	0,6279808
CG1139-RA	CG11399	8,31499	3,58232	7,8926	5,0363	0,532563	0,707192	0,841022	0,018642	0,6279808
CG11400-RA	SCOT	0,0344923	0,031418	0,0331147	0,0373003	0,0505234	0,0380775	-0,777749	0,029057	0,6279808
CG11401-RA	SCOT	27,6047	23,0284	43,1357	37,2164	52,7165	47,1071	-0,361191	0,107326	0,13772387
CG11403-RA	CG11400	1,24671	70,0805	17,2139	0,0108347	15,4856	0,0432704	-0,409321	0,18884	0,6279808
CG11404-RA	Trxr-2	0	0	0	0,0619857	1,791	0,199791	-2,23786	3,95E-10	0,6279808
CG11405-RA	Dsim\GD16432	0,622487	0,651005	1,36126	1,41665	0,883344	1,16778	0,177111	0,428196	0,6279808
CG11406-RA	CG11404	0	0	20,6145	1622,49	17,778	17,0048	0,443983	0,199894	0,6279808
CG11406-RB	Atf3	26,4635	28,5081	24,1442	0,0910208	5,14797	8,04024	0,443983	0,199894	0,6279808
CG11407-RA	CG11406	0,0554482	0,0505061	0,473963	0,329981	6,69659	0,293947	-0,096296	0,768248	0,6279808
CG11409-RB	CG11406	0,377521	0,343872	9,8052	13,7522	0,620343	7,41123	0,482671	0,126002	0,6279808
CG1140-RA	CG11407	1,59264	1,38321	4,46421	2,97958	2,48671	1,51519	-0,488806	0,071106	0,6279808
CG1140-RB	CG11409	14,5088	16,6949	7,40819	14,327	5,73695	2,84695	-0,488415	0,071513	0,6279808
CG11411-RA	fs(1)N	0,100343	0,0228498	0,0722515	22,773	0,115063	0,106501	-0,367624	0,298949	0,6279808
CG11412-RA	Naa30A	0,027616	3,60857	0,026513	8,89608	2,20421	0,0299818	-0,013739	0,945061	0,6279808
CG11412-RB	Naa30A	24,6608	1,87953	27,1246	8,27935	1,78596	23,8213	-0,013754	0,944987	0,6279808
CG11412-RC	Naa30A	0,0282536	0	0,0271252	2,78186	0,615425	0,0307212	-0,013516	0,945945	0,6279808

gene_id	Symbol	W1_FPKM	W2_FPKM	W3_FPKM	MCM51_FPKM	MCM52_FPKM	MCM53_FPKM	FC	p-value	p-adj
CG11413-RA	CG11413	0,108499	0,0988284	53,1765	0,0369033	186,186	133,775	-1,106849	0,001961	0,6279808
CG11414-RA	CG11414	11,9638	47,0455	15,5852	1,56239	8,46493	3,14493	0,172872	0,469413	0,6279808
CG11415-RA	Tsp2A	90,8933	122,396	171,945	38,1619	155,137	149,22	-0,008235	0,974915	0,6279808
CG11416-RA	uri	2,10306	1,5532	97,2077	18,3084	1,66186	1,87524	0,415594	0,159647	0,6279808
CG11417-RA	CG11417	6,49105	1,20266	10,6254	0	8,67635	7,63177	-0,020715	0,935876	0,6279808
CG11418-RA	MTPAP	4,88553	9,15119	9,09217	6,86839	5,73285	4,8378	-0,074043	0,733632	0,6279808
CG11418-RB	MTPAP	4,2763	4,78585	3,72718	127,96	4,43609	5,24217	0,065953	0,771533	0,6279808
CG11419-RA	APC10	8,01747	9,34422	8,97003	0,0734152	0,0806588	10,3737	0,541541	0,02089	0,6279808
CG11420-RA	CG1142	19,8664	12,6272	12,5583	15,5222	5,06217	6,18798	0,209571	0,538944	0,6279808
CG11421-RA	png	0,792206	7,75166	11,5659	11,7728	0,976618	9,61215	0,313942	0,150559	0,6279808
CG11422-RA	Obp83a	0,117156	0,213427	0,179538	0	46,0929	0,0320076	0,274674	0,298417	0,6279808
CG11423-RA	Obp83b	0,498686	0,567797	1,81444	0,301775	9,51022	1,03852	0,22209	0,499177	0,6279808
CG11425-RA	ND-51L1	0,0932397	3,14264	6,80538	6,83793	5,15734	261,611	-0,494596	0,13786	0,6279808
CG11426-RA	CG11425	0,103676	0	0	7,77388	21,2391	21,4831	-0,52492	0,073891	0,6279808
CG11427-RA	CG11426	6,84997	0,157391	0	25,3235	0,08396	10,0832	0,284452	0,168209	0,6279808
CG1142-RA	rb	9,94764	0,101052	10,1546	10,3599	0,481545	0,0784263	0,107637	0,720004	0,6279808
CG11430-RA	CG1143	14,5036	29,0373	16,6149	24,9981	28,8946	9,28401	0,096743	0,645342	0,6279808
CG11430-RB	olf186-F	0,025774	5,34778	4,57278	0,0120824	0,0163657	0,0125482	0,079835	0,701615	0,13772387
CG11430-RC	olf186-F	0,0536766	6,72727	5,50537	0,012292	0,0166496	0,0207063	-0,348697	0,176427	0,13772387
CG11430-RD	olf186-F	5,22609	1,6433	1,80347	0,0202836	0,0274743	9,06996	0,160504	0,451649	0,6279808
CG11430-RE	olf186-F	5,22121	18,86	20,2605	18,8204	10,9658	8,33	0,032889	0,880805	0,6279808
CG11436-RA	olf186-F	1,34842	0,261396	0,206635	14,0206	7,51942	0,0278584	0,09035	0,723483	0,6279808
CG11437-RA	CG11436	3,6249	8,39005	22,6195	12,5956	15,4655	3,17067	-0,120841	0,66699	0,6279808
CG11438-RA	CG11437	13,6195	1117,27	9,34184	2,51133	3,46599	0	-0,50625	0,024539	0,6279808
CG1143-RA	CG11438	12,5846	14,5788	24,1467	1,48062	22,0308	1,93088	-0,072371	0,808973	0,6279808
CG11440-RA	laza	4,78548	18,0753	17,8813	0	0	18,9809	-0,352126	0,144762	0,6279808
CG11444-RA	CG11444	65,942	41,3355	91,3253	69,4445	0,65591	119,93	-0,533498	0,084082	0,6279808
CG11447-RA	CG11447	8,62472	0,0328588	2,45897	13,0983	9,89339	0,0399785	-0,241621	0,375986	0,6279808
CG11448-RA	Rilpl	6,94199	8,65958	6,93367	3,66423	6,06348	5,33882	-0,017396	0,93941	0,6279808
CG11449-RA	CG11449	0,694675	4,23186	6,53656	6,51836	6,59538	5,69565	-0,381743	0,270399	0,6279808
CG11450-RB	net	3,09967	2,32758	2,25005	2,57545	1,49534	1,43739	0,409034	0,13675	0,6279808
CG11451-RA	Spc105R	1,00197	12,4751	1,62069	11,8522	0,0729458	1,07483	-0,09009	0,787747	0,6279808
CG11453-RA	CG11453	29,5385	27,4288	40,3501	28,466	4,94031	3,27478	-0,456075	0,127013	0,6279808
CG11454-RA	CG11454	17,217	29,8544	1,63864	2,53698	1,10787	23,0008	-0,018979	0,94159	0,6279808
CG11455-RA	Dpse\GA11014	144,203	93,9687	71,0175	73,4582	227,444	0,671136	-0,592072	0,043166	0,6279808
CG11455-RB	Dpse\GA11014	225,358	183,008	0,0298432	0,0333372	660,268	0,2678	-0,588997	0,044526	0,6279808
CG11456-RA	CG11456	3,64557	3,21686	0,114922	2,58609	0	2,25018	0,542254	0,028179	0,6279808
CG11458-RA	CG11458	0	0	0	0,4542	0,381935	0	-0,11534	0,404049	0,6279808
CG11459-RA	Dsim\GD19566	1,16845	0	0,0777157	1,027	2,88816	4,60636	0,670766	0,055124	0,6279808
CG11462-RA	CG1146	3,48716	6,8283	2,61386	17,3208	1,42583	1,18919	0,439175	0,059072	0,6279808
CG11466-RA	CG1146	3,15481	9,41295	7,57202	13,7566	1,02747	4,08528	-0,688857	0,012413	0,6279808
CG11466-RB	CG1146	7,49449	10,2561	6,02465	15,7441	2,49006	2,01537	-0,688857	0,012413	0,6279808
CG1146-RA	CG11462	17,5011	7,82148	13,4784	12,2313	45,4126	8,2352	1,237377	1,11E-05	0,6279808
CG1146-RB	Dpse\GA26760	20,9958	0,0341645	17,2494	10,9086	0,0521775	12,091	1,221586	1,52E-05	0,6279808
CG1146-RC	Dpse\GA26760	0,0375076	11,5815	0,0341119	35,8408	19,2817	1,49711	1,219996	1,63E-05	0,13772387
CG11470-RA	NPFR	3,16701	16,0596	0,0226344	1,95375	0,138476	1,65783	0,731127	0,038443	0,6279808
CG11471-RA	CG11470	8,40966	46,9251	13,3309	0,032355	0,148668	526,541	0,130576	0,567779	0,6279808
CG11471-RC	IleRS	13,8386	16,8441	19,8321	26,3415	23,0693	0,717222	0,130951	0,566833	0,6279808
CG11471-RD	IleRS	0,0174128	0,0158608	0,0167173	0,0180747	9,687	9,51692	0,130576	0,567779	0,6279808
CG11474-RA	IleRS	3,50368	3,94047	3,7585	0,0177724	0,0244823	0,0184513	-0,521291	0,063389	0,6279808
CG11474-RB	CG11474	23,524	7,64831	12,3292	13,5394	0,0409121	7,39775	-0,521291	0,063389	0,6279808
CG11475-RA	CG11474	0,0428044	5,33215	4,84683	17,4678	5238,19	32,9221	-0,168545	0,557027	0,6279808
CG1147-RA	CG11475	0,0437395	10,1004	0,242495	7,14321	0,0746542	2,04455	0,595434	0,014256	0,6279808
CG11482-RA	Osi2	66,7786	0,02046	482,071	0,0235958	0,0319606	0,0240875	-0,209057	0,450824	0,6279808
CG11486-RA	Osi2	11,4496	0,0198395	10,052	0,0228432	0,0309413	0,0233192	0,267063	0,266446	0,6279808
CG11486-RB	Mlh1	1,12729	7,95893	0,0453444	0,0550694	0	0,0562169	0,276355	0,24878	0,6279808
CG11486-RC	CG11486	0,0349279	0,114423	0,0208765	0,0903219	0,0304452	0,0250192	0,277131	0,247067	0,6279808
CG11486-RD	CG11486	0,0223556	0,939015	0,020808	4,60807	0,0380678	0,0239673	0,271624	0,258626	0,6279808
CG11486-RF	CG11486	0,0225464	0,0359752	0,0212756	0,0448084	4,51104	0,0241827	0,274593	0,251271	0,6279808
CG11486-RG	CG11486	0,021745	0,0569587	0,0205186	0,0726062	6,44537	0,0232788	0,27428	0,251798	0,6279808
CG11486-RH	CG11486	0,0216737	102,76	11,4273	92,6409	10,5169	0,0231986	0,274199	0,251961	0,6279808
CG11486-RJ	CG11486	0,0221607	0,0434037	0,0210987	0,0532449	9,872	0,0237473	0,275032	0,250753	0,6279808
CG11486-RK	CG11486	0,0213722	0,0299584	0,0205919	0,0364744	4,57571	0,0228596	0,27508	0,250788	0,6279808
CG11486-RL	CG11486	0,0225003	0,0212098	11,9437	0,0245085	36,329	0,0241307	0,275573	0,24974	0,6279808
CG11486-RM	CG11486	0,0219764	0,0203631	0,0240594	0,0234781	0,0180541	0,0235395	0,274199	0,251961	0,6279808
CG11486-RN	CG11486	0,0214486	0,0205368	0,0245245	11,8998	3,31337	0,0229453	0,274485	0,251887	0,6279808
CG11486-RO	CG11486	15,8852	0,0198069	25,5778	0,0228037	47,349	0,0286902	0,277636	0,246007	0,6279808
CG11486-RP	CG11486	0,0250604	0,0197419	11,1053	0,022725	0,122342	0,0270409	0,27469	0,250912	0,6279808
CG11486-RQ	CG11486	0,0255448	0,0201855	56,8443	0,0232626	3,79829	9,32974	0,274572	0,251676	0,6279808
CG11488-RA	CG11486	21,8087	0,0194673	0,0721965	0,0223929	7,14598	13,4538	-0,055857	0,8503	0,6279808
CG11489-RB	CG11486	15,5026	11,6923	7,5215	0,0236381	0,957134	15,5697	0,470608	0,060054	0,6279808
CG11489-RD	mRpL10	36,533	27,2783	55,0304	11,0292	22,0994	2,85969	0,392888	0,121968	0,6279808
CG1148-RA	Srpk79D	2,50927	3,52946	5,71461	5,64276	1,75675	4,0308	0,457285	0,13047	0,6279808
CG1148-RB	Srpk79D	7,93944	8,87944	13,9262	19,0102	3,55882	8,97742	0,455547	0,132043	0,6279808
CG11490-RA	MstProx	0,0514823	0,554102	0	16,1083	24,9305	18,4143	0,355707	0,092939	0,6279808
CG11491-RA	Tbc1d15-17	6,30322	6,23796	7,34899	32,2916	2,95206	8,31233	0,080661	0,803288	0,6279808
CG11491-RB	br	0,473434	0,0157818	0,0761849	0,0964004	1,9262	1,72019	0,147074	0,645502	0,6279808
CG11491-RC	br	0,0173261	0,0137863	6,69713	5,9448	2,19374	3,48766	0,154337	0,630521	0,6279808
CG11491-RE	br	0,0151353	0,0143895	2,26803	0	1,71527	0,526946	0,118516	0,712769	0,6279808
CG11491-RG	br	0,466028	0,0159153	0,0238843	0,655931	0,0363766	0,0183557	0,115991	0,719922	0,6279808
CG11491-RH	br	0,0174727	0,0146737	0,117985	0,017981	0,229719	0,30152	0,114829	0,714897	0,6279808
CG11491-RI	br	0,0161095	0,0196324	13,9602	0,706764	7,21072	0,0166764	0,140303	0,66186	0,13772387
CG11491-RJ	br	0,0215535	1,94741	0,614007	0,016336	0	0,0185174	-0,066961	0,837732	0,6279808
CG11491-RK	br	1,12525	0,0153237	0,0166341	0,0181394	0,231133	0,0170182	0,10957	0,732184	0,6279808
CG11494-RA	br	0,0168232	0	0,61756	0,0166708	0,0243553	0,0230633	-0,085733	0,72543	0,6279808
CG11494-RB	BtbVII	0,0207351	0,018887	0,0217274	2,89478	6,91861	14,7969	-0,082755	0,734133	0,6279808
CG11494-RC	BtbVII	4,72627	7,32953	0,0212329	15,6765	0,0293827	0,0223062	-0,270147	0,273034	0,6279808
CG11495-RA	BtbVII	8,38403	8,53899	5,98517	0,0218508	10,1657	0,0242787	0,015654	0,951795	0,6279808
CG11498-RA	rasp	0,0394954	22,06	129,076	0,0281045	121,621	109,396	-0,017195	0,947129	0,6279808
CG1149-RA	CG11498	3,23294	0,711883	3,51537	0,0184131	1,95833	2,50502	0,196274	0,350905	0,6279808
CG11500-RA	Osi3	3,68062	53,5616	5,44064	0,0243996	0,0330494	0,0249081	-0,063897	0,769256	0,6279808
CG11501-RA	Spase12	52,0474	2,13001	617,474	0,0624849	0,0846363	6,41456	-0,013096	0,914398	0,6279808
CG11502-RA	Diedel	0	48,9181	59,4511	93,6154	1,31013	100,058	-0,482315	0,096317	0,6279808
CG11502-RB	svp	9,51436	12,1622	0,0240412	8,75527	12,3826	12,4294	0,046081	0,864512	0,6279808
CG11502-RC	svp	0,0196207	3,83513	0,0238424	3,07128	7,46736	8,67266	0,143192	0,597897	0,6279808
CG11504-RA	svp	4,26893	14,3476	0,0203999	9,2669	0,0355365	0,0262597	0,248542	0,309656	0,6279808
CG11504-RB	CG11504	2,19334	8,00424	133,155	7,42032	119,208	0,1084	0,248542	0,309656	0,6279808
CG11504-RC	CG11504	10,1341	8,67166	0,0824209	27,5403	0,143832	17,8966	0,248542	0,309656	0,6279808
CG11505-RA	CG11504	0,0357231	0	0,119767	0,13488	0,234549	1,02152	-0,038608	0,887313	0,6279808
CG11505-RB	Larp4B	0,0172357	0,0156994	0,0165473	17,3975	0,0242231	19,6516	0,253792	0,335016	0,13772387
CG11505-RC	Larp4B	0,0127347	0,0115996	0,012226	0,0178834	0,479354	0,018256	-0,020102	0,939256	0,6279808
CG11508-RA	Larp4B	3,63599	3,40982	3,78824	0,013075	1,4811	0,0133474	0,291049	0,211295	0,6279808
CG11508-RB	Pbp49	4,21396	3,44829	5,32842	9,04052	3,95836	0,0377993	0,293038	0,207681	0,6279808
CG11508-RC	Pbp49	0,0357038	0,0325215	0,0342778	0,0387252	0,0524535	2,49516	0,292073	0,209228	0,6279808

gene_id	Symbol	W1_FPKM	W2_FPKM	W3_FPKM	MCM51_FPKM	MCM52_FPKM	MCM53_FPKM	FC	p-value	p-adj
CG1150-RA	Pbp49	3,86646	4,22294	6,63353	0,0388393	0,0526081	4,52592	1,090465	0,000425	0,6279808
CG11512-RA	Osi6	1574,57	0	1726,08	1,29411	0	4,436	0,147151	0,673512	0,6279808
CG11513-RA	GstD4	0,761037	7,89483	8,3379	0,0255046	2,20751	28,2252	0,231504	0,406868	0,6279808
CG11513-RB	armi	0,0164055	0,620145	12,0968	0,0169889	0,0688386	166,439	0,231504	0,406868	0,6279808
CG11516-RA	armi	0,813008	0,0148109	38,4194	0,696585	6,12002	16,8974	0,469785	0,063935	0,6279808
CG11516-RB	Ptp99A	3,46649	4,48516	0,0283326	0,043392	0,0489442	0,0330292	0,057024	0,840605	0,6279808
CG11516-RC	Ptp99A	9,99	14,0941	18,8915	0,347216	0,0266299	13,7039	0,469913	0,063842	0,6279808
CG11516-RD	Ptp99A	3,92171	0,00985425	6,78912	0,462805	454,458	3,57235	0,505607	0,044409	0,6279808
CG11516-RE	Ptp99A	0,00994719	0,00906059	0,0164742	25,9631	0,0587748	2,03298	0,459065	0,066865	0,6279808
CG11518-RA	Ptp99A	0,0150207	0,0136819	0,0321579	1,76685	1,39187	0,0368873	0,274792	0,267415	0,6279808
CG1151-RA	pygo	6,22624	2,63921	10,2875	10,7115	16,6222	32,4527	0,497665	0,1103	0,6279808
CG11522-RA	Gld	6,88905	20,6546	0,117042	0,136418	3,45039	0	-0,301033	0,233471	0,6279808
CG11522-RB	RpL6	1096,1	1284,26	4,4896	1786,48	4,13954	2,10822	-0,301041	0,233453	0,6279808
CG11523-RA	RpL6	0,0732006	0,0666762	0,947383	0,0874451	1,69213	4,95051	0,20014	0,389976	0,6279808
CG11525-RA	CG11523	12,085	11,4512	16,8585	15,4176	9,98327	12,8991	0,16111	0,460951	0,6279808
CG11525-RB	CycG	83,3657	0,043435	36,6661	0,0247386	7,88326	3,3089	0,15391	0,480982	0,6279808
CG11525-RC	CycG	196,827	0,0455727	0,0457806	0,193888	0,0721789	0,0709154	0,135884	0,536149	0,6279808
CG11525-RD	CycG	47,7353	7,66252	0,0480338	8,62676	21,1763	0,74669	0,160034	0,464003	0,6279808
CG11525-RE	CycG	0,0189275	113,615	84,2809	182,371	92,974	8,63672	0,254075	0,23889	0,6279808
CG11526-RA	CycG	0,0228005	280,386	265,835	149,183	91,2497	4,84799	-0,008983	0,969066	0,6279808
CG11526-RB	Strip	9,79125	4,3219	46,0592	11,009	0,114494	7,12224	0,007165	0,975389	0,6279808
CG11527-RA	Strip	0,0186514	12,1515	17,6213	5,13789	25,012	1,72325	0,038287	0,888562	0,6279808
CG11529-RA	Tig	67,4563	89,2744	58,5425	6,0612	0,0561494	45,5532	-0,330965	0,352142	0,6279808
CG1152-RA	CG11529	13,4981	4,24079	44,347	3,55919	6,06522	7,00488	0,092834	0,682267	0,6279808
CG11533-RD	Osi7	342,99	424,865	354,619	2,55217	0,97899	0,810391	0,364694	0,125882	0,6279808
CG11533-RE	Asator	25,6482	25,1029	0,0283749	37,4419	0,0229111	0,0172672	0,116034	0,621261	0,6279808
CG11533-RF	Asator	3,94416	3,33837	0,0195511	20,8276	42,109	35,6886	0,362525	0,127869	0,6279808
CG11534-RA	Asator	0,0164504	0,0149842	23,0106	0,0182146	40,9383	28,1833	0,236619	0,302108	0,6279808
CG11537-RA	CG11534	8,29533	3,33292	10,4091	5,18632	7,09926	5,89897	-0,382702	0,046333	0,6279808
CG11537-RB	CG11537	16,2468	15,5542	8,63617	74,1355	3,83235	0,0187549	-0,384396	0,045248	0,6279808
CG11537-RC	CG11537	0,0208792	0,0190183	3,16703	5,01936	14,6746	0,0382943	-0,280948	0,123106	0,6279808
CG11537-RD	CG11537	0,0226313	0,0206142	7,85008	8,67829	0,0295971	8,71875	-0,197471	0,300017	0,6279808
CG11537-RE	CG11537	0,0221162	0,020145	16,5419	24,9211	0,0322144	127,864	-0,296127	0,119231	0,6279808
CG11538-RA	CG11537	3,71626	4,1577	0,0200453	12,6397	0,0314427	3,11598	0,32292	0,357685	0,6279808
CG11539-RA	Lsp2	0,118966	0,392815	1,05649	0,483435	0,217477	0,26992	-0,41722	0,15689	0,6279808
CG1153-RA	CG11539	0,12021	68,744	72,708	137,107	72,4469	0,0252541	0,223801	0,49967	0,6279808
CG11546-RA	Osi12	11,9539	0,466376	0,737343	744,253	0,500896	0,875144	0,2267	0,226894	0,6279808
CG11546-RB	kermit	20,5945	20,3518	22,4857	2,74404	2,68684	3,04772	0,250638	0,180549	0,6279808
CG11546-RC	kermit	0,0434805	22,8049	0,0417438	17,2567	1,6111	10,6726	0,250215	0,180963	0,6279808
CG11546-RD	kermit	8,44877	0,039605	10,6844	16,6111	15,1915	10,8625	0,256051	0,172553	0,6279808
CG1154-RA	kermit	0,0397409	8,57396	0,0381536	13,7085	0,0651217	12,6599	0,311424	0,272337	0,6279808
CG11550-RA	Osi14	263,372	291,513	358,455	311,598	2,05662	144,876	0,85859	0,001753	0,6279808
CG11551-RA	CG11550	5,19168	6,6328	0,33984	2,53773	3,06175	3,77082	0,43818	0,220401	0,6279808
CG11552-RA	ind	1,00349	0,0148731	2,29441	1,19509	2,40906	8,53866	0,076465	0,555158	0,6279808
CG11555-RA	Rpn12R	0,174804	8,40629	0	7,19529	70,1166	14,1115	-0,030599	0,924612	0,6279808
CG11556-RA	CG11555	24,1252	12,8121	3,42067	14,7283	7,13463	9,49674	-0,200305	0,431039	0,6279808
CG1155-RA	Rph	2,60059	4,3476	3,43574	6,7968	3,18009	2,58032	0,746452	0,012639	0,6279808
CG11560-RA	CG11560	6,32708	7,40975	8,16898	8,78194	0,0693198	7,70925	0,361456	0,136055	0,6279808
CG11561-RA	smo	5,56396	4,26782	0,0375884	6,30652	26,1896	116,203	0,024452	0,910775	0,6279808
CG11562-RA	CG11562	17,815	16,6565	0,275584	23,7283	111,661	0,0307352	-0,046615	0,856276	0,6279808
CG11563-RA	CG11563	17,2947	10,4744	0,0457476	0,0532449	3,8787	25,9557	0,03247	0,918987	0,6279808
CG11567-RA	Cpr	21,9239	20,615	34,6846	46,0581	44,2717	35,7256	-0,119993	0,568963	0,13772387
CG11567-RB	Cpr	53,1489	54,8974	53,0555	31,0234	31,5743	33,1291	-0,013184	0,95013	0,6279808
CG11570-RA	Osi15	556,354	146,705	526,603	1,91861	49,4459	884,784	1,492483	1,81E-05	0,6279808
CG11575-RA	CG11570	2,24151	2,52996	11,9733	1,82297	14,8693	8,99548	0,081493	0,800716	0,6279808
CG11576-RA	Ir100a	0,218794	0,132862	0,105028	10,89	10,7497	10,2175	-0,115605	0,608558	0,6279808
CG11577-RA	Rift	7,54759	5,33153	0,0577923	0,0412204	0,179223	0,0199036	-0,398581	0,074075	0,6279808
CG11579-RA	CNPYb	15,1362	27,0068	0,0212875	20,1687	1,85446	2,80833	-0,145962	0,470167	0,6279808
CG11579-RB	arm	25,7874	0,0191149	4,07796	45,7237	0,100675	0,0758749	-0,146344	0,468819	0,6279808
CG11579-RC	arm	0,0208661	82,1381	23,4349	32,1245	20,7751	21,9134	-0,147458	0,465663	0,6279808
CG11579-RD	arm	0,0209853	54,7976	0,0200327	0,0218364	0,0295775	0,0222914	-0,145833	0,470605	0,6279808
CG11579-RE	arm	63,05	9,66831	0,0201472	0,0219674	0,029755	0,0224252	-0,146291	0,469026	0,6279808
CG1157-RA	arm	64,6614	3,00196	82,6434	119,066	63,4313	74,2482	0,787233	0,007144	0,6279808
CG11581-RA	Tim17b1	0,209632	0,0116603	1,48856	9,47791	2,71019	0,0281853	-0,24788	0,26562	0,6279808
CG11582-RA	CG11581	0	0	0,157803	0,0526914	52,8196	0,125725	-0,213411	0,443374	0,6279808
CG11583-RA	srw	0,282376	19,5602	0,632561	28,2187	47,166	7,38745	0,007481	0,979753	0,6279808
CG11584-RB	CG11583	21,6304	0,0635771	43,2254	44,9581	12,5112	52,6262	0,120057	0,608568	0,6279808
CG11585-RB	CG11584	74,1665	62,21	95,4146	9,06021	44,4519	5,22519	0,390346	0,267303	0,6279808
CG11586-RA	CG11585	3,76525	3,24886	9,02357	0,0500119	3,82024	362,705	0,178057	0,418674	0,6279808
CG11588-RA	CG11586	15,8016	7,75806	0,0961646	18,2834	12,8548	0,131757	-0,070526	0,562659	0,6279808
CG11589-RA	CG11588	0	1,2726	2,65635	4,45353	4,40937	0,237775	-0,242062	0,302118	0,6279808
CG1158-RA	VhaM9.7-c	26,6702	31,9011	327,625	276,613	0,788039	239,234	-0,255691	0,445629	0,6279808
CG11590-RA	CG11590	8,1187	6,88212	12,7504	0,615794	0,214726	12,7924	0,094392	0,766306	0,6279808
CG11591-RA	Dpy-30L2	0,136332	22,9838	0,0381995	47,0425	43,5879	36,0628	-0,228893	0,52123	0,13772387
CG11592-RA	Amnionless	7,88315	12,4861	11,2954	12,0896	10,2288	11,764	-0,308646	0,171425	0,6279808
CG11593-RB	CG11593	8,51255	4,26398	0,12294	0	4,29974	4,22371	0,562303	0,028847	0,6279808
CG11594-RA	CG11594	0,0235341	0,0212398	0,0247252	0,0272672	0,0335716	8,88888	-0,779703	0,014931	0,6279808
CG11594-RB	CG11594	0,0257539	0,127184	20,8078	15,8049	5,1961	0,125027	-0,779815	0,014869	0,6279808
CG11594-RC	CG11594	13,6968	5,08793	27,7168	4,47472	33,5039	9,89705	-0,764607	0,016982	0,6279808
CG11596-RA	CG11596	3,45548	2,11044	0,0536387	2,93893	0,0583139	0,0439489	-0,03383	0,903367	0,6279808
CG11596-RB	CG11596	4,01254	2,57991	0,061252	12,1398	3,08649	0,0462453	-0,031304	0,910078	0,6279808
CG11596-RC	CG11596	1,62752	2,74118	3,06061	9,13292	1,46196	2,06865	-0,033693	0,903696	0,6279808
CG11597-RA	CG11597	0,0476165	0,0433724	0,0457147	0,0532019	0,0720623	0,0543105	-0,097651	0,723528	0,6279808
CG11597-RB	CG11597	0,0406704	0,0370454	0,039046	0,0446566	0,0604876	0,0455871	-0,09292	0,73668	0,6279808
CG11598-RB	CG11598	0,237873	7,43744	0,126874	0,731546	3,38123	0	-0,366272	0,272306	0,6279808
CG11600-RA	CG11600	0,143394	0,0783678	0,0826	6,23678	183,979	5,03214	-0,149504	0,56906	0,6279808
CG11601-RA	CG11601	12,6123	15,6878	1,26568	18,3529	0,040856	0,0305122	-0,369572	0,141769	0,6279808
CG11604-RA	mbm	9,10067	5,6748	4,28303	22,3971	0	22,3212	-0,10107	0,752311	0,6279808
CG11606-RA	Rpp30	8,76086	7,30699	10,8093	8,92725	0,795444	5,31074	-0,282619	0,326258	0,6279808
CG11607-RA	H2.0	5,13481	10,3053	7,74614	0,416249	23,2632	1,63235	0,327611	0,244107	0,6279808
CG11608-RA	CG11608	0	0	77,9252	7,92144	7,05186	7,15616	NA	NA	0,6279808
CG11611-RA	CG1161	34,8511	35,3669	0,324208	0,199095	9,03489	0,290389	-0,070526	0,562659	0,6279808
CG11614-RA	CG1161	9,39006	8,97925	0,0679923	0,459113	55,2726	0,0720951	0,382622	0,217821	0,6279808
CG11617-RA	Tim13	0	0	0,0428917	10,2725	62,4261	43,4761	0,459032	0,085402	0,6279808
CG11619-RA	nkd	27,1684	5,189	17,1043	6,57515	3,56241	4,41286	0,377486	0,128538	0,6279808
CG1161-RA	CG11617	17,0951	11,1827	17,327	5,05101	6,63242	0,00347936	-0,175842	0,459562	0,6279808
CG1161-RB	CG11619	6,48173	6,53453	4,81818	0,0229227	0,0310489	0,0234003	-0,161479	0,492437	0,6279808
CG11621-RA	CG1162	0	0	0,0255931	0,341264	0,240726	0	0,099112	0,631409	0,6279808
CG11621-RB	Pi3K68D	2,86052	2,5381	0,0431837	2,79593	0,0704692	1,48475	-0,043955	0,868077	0,13772387
CG11621-RC	Pi3K68D	2,13033	2,03307	9,02795	2,76885	12,2142	1,62508	0,097685	0,636635	0,6279808
CG11622-RA	Pi3K68D	2,34738	0,0328947	6,25616	0,0392091	0,0182125	1,98893	-0,276713	0,164591	0,6279808
CG11624-RA	Rlip	5,63417	5,25246	5,89165	0	15,5712	5,46313	0,607518	0,004326	0,13772387
CG11624-RB	Ubi-p63E	50,415	0,0116844	203,717	0,0458689	0,0218724	365,91	0,588909	0,004858	0,6279808
CG11624-RC	Ubi-p63E	39,5625	18,1694	0,0400063	3,63696	3,37739	0,0468247	0,592589	0,004519	0,6279808

gene_id	Symbol	W1_FPKM	W2_FPKM	W3_FPKM	MCM51_FPKM	MCM52_FPKM	MCM53_FPKM	FC	p-value	p-adj
CG11626-RB	Ubi-p63E	87,2174	2,75987	4,27173	2,61429	0,112616	3,42347	-0,02976	0,919396	0,6279808
CG11628-RA	CG11626	0,260826	0,0241228	0,0254256	28,084	0,0389312	12,1009	-0,052777	0,810508	0,13772387
CG11628-RB	step	5,26134	2,56858	0,0179674	4,18652	3,63033	2,5852	0,016255	0,941421	0,6279808
CG11629-RA	step	10,213	10,5025	0,0119971	15,8377	9,41177	8,16324	0,044335	0,715924	0,6279808
CG1162-RA	CG11629	0,0712025	0	0	0	0,117102	0	-0,070526	0,562659	0,6279808
CG11630-RA	RpII18	62,432	37,6166	70,725	2,56793	0,115682	0,0627165	0,015925	0,916254	0,6279808
CG11634-RA	CG11630	0	0	0,0501873	10,6915	0	0,0595783	NA	NA	0,6279808
CG11635-RA	CG11634	0	0	0	0,0504716	16,0206	0,0515233	0,06906	0,58405	0,6279808
CG11637-RA	CG11635	0	0	0,123067	0,0440888	0	35,7993	0,453315	0,20404	0,6279808
CG11638-RA	NijB	2,72291	1,87394	2,84652	0,196386	3,44254	3,07234	0,107011	0,622373	0,6279808
CG11639-RA	CG11638	7,51593	8,27381	173,994	6,9809	10,1676	356,568	-0,007232	0,983508	0,6279808
CG1163-RA	TfIIA-S-2	0,929639	0,977053	0,0309951	8,7735	0,0470354	0,602943	-0,491531	0,105635	0,6279808
CG11641-RA	pdm3	0,0163933	0,0149321	0,0157385	0,0169757	0,0745918	0,0173295	0,502642	0,057555	0,13772387
CG11641-RB	pdm3	4,78154	5,09953	3,29431	3,78771	41,4893	2,18109	0,502642	0,057555	0,6279808
CG11642-RA	TRAM	0,0322846	0,0294071	84,5172	154,156	51,3683	0	0,102766	0,601011	0,6279808
CG11642-RB	TRAM	79,5388	69,5868	15,4403	1,15152	12,0188	2,28517	0,101989	0,603394	0,13772387
CG11642-RC	TRAM	14,1284	23,1905	7,2286	14,7449	6,12464	5,571	0,106279	0,586817	0,13772387
CG11648-RA	Abd-B	13,669	22,5919	8,59516	26,0291	30,4355	5,77619	0,435037	0,11609	0,6279808
CG11648-RB	Abd-B	6,62919	15,7324	7,15574	11,2168	0	4,31106	0,444316	0,100809	0,6279808
CG11648-RC	Abd-B	0,0206702	9,50988	0,0198447	9,39818	0	0,0220719	0,432633	0,116923	0,6279808
CG11648-RD	Abd-B	0,0203707	0,0188279	0,0195571	0,0216214	41,6277	0,0217367	0,430077	0,119858	0,6279808
CG11648-RE	Abd-B	0,0189022	0,0185551	0,0181473	0,021293	0,195594	0,0200999	0,491384	0,073932	0,6279808
CG11650-RA	LysS	6,29961	1,84895	34,608	79,3114	7,58213	79,8913	-1,591429	3,69E-06	0,6279808
CG11652-RA	Lcp1	7,03986	1,04529	11,3831	20,2134	908,082	8,59709	-0,249524	0,439813	0,6279808
CG11652-RB	Dph1	30,0333	87,6431	22,0263	0,321635	32,1952	36,7891	-0,249256	0,440275	0,6279808
CG11654-RA	Dph1	35,4074	57,7657	26,9658	47,6857	46,174	38,4053	-0,761821	0,011597	0,6279808
CG11655-RA	Ahcy	44,821	39,7735	84,0409	0	2,91398	3,91612	0,080511	0,748473	0,6279808
CG11656-RA	CG11655	3,72884	0,407768	1,20668	5,58233	1,04744	1,37661	-2,018068	9,07E-11	0,6279808
CG11658-RA	CG11656	0,56399	0,459645	0,626958	1,59145	12,1139	2,96871	-0,003261	0,98791	0,6279808
CG11658-RB	CG11658	28,1951	0,0754059	10,3264	29,5763	0,238679	70,391	0,001975	0,992683	0,6279808
CG11658-RC	CG11658	0,0325871	11,4757	31,3166	72,8475	20,6664	0,047054	-0,002138	0,992077	0,6279808
CG11658-RD	CG11658	0,0327811	0,0400709	78,1316	46,7541	0,0475112	7,41075	-0,00661	0,975474	0,6279808
CG11658-RE	CG11658	0,0339954	17,2669	0,112594	0,0350764	0,0478166	0	-0,006533	0,975765	0,6279808
CG11659-RA	CG11658	9,39207	63,4634	0	0,0353019	0,0497351	0,0586771	0,03566	0,918349	0,6279808
CG1165-RA	CG11659	0,527513	0,498291	4,19593	3,05734	1,94255	1,96671	-0,453172	0,167325	0,6279808
CG11660-RA	RIOK1	5,83961	9,06852	0,048735	4,21757	0,0437821	6,16289	0,103064	0,76201	0,6279808
CG11660-RB	RIOK1	0,0302038	0,0519894	188,681	0,0323233	13,5526	0,0329968	0,103064	0,76201	0,6279808
CG11661-RA	Nc73EF	0,0180305	0,0164234	9,4255	18,2196	7,28061	10,3732	-0,113077	0,630033	0,6279808
CG11661-RB	Nc73EF	0,0181644	0,0165453	0,0174389	0,0188881	40,7858	0,0192817	-0,112861	0,630763	0,6279808
CG11661-RC	Nc73EF	0,0179229	0,0163254	0,0172071	0,0186265	95,3967	0,0190146	-0,112959	0,630447	0,6279808
CG11661-RE	Nc73EF	0,0181046	0,0164909	0,0173815	0,0188234	16,477	0,0192156	-0,067575	0,774814	0,6279808
CG11661-RF	Nc73EF	59,9293	86,8642	50,304	53,7861	0,0255841	43,9562	-0,11347	0,628949	0,6279808
CG11661-RG	Nc73EF	0,0178599	0,0162681	0,0171466	0,0185583	0,0252297	0,018945	-0,112959	0,630447	0,6279808
CG11661-RH	Nc73EF	0,0268917	0,0163299	0,0172117	0,0186318	0,0254964	0,01902	-0,11345	0,628926	0,6279808
CG11661-RI	Nc73EF	26,4709	36,2545	27,2203	39,9271	43,997	25,4448	-0,112467	0,632617	0,6279808
CG11663-RA	CG11663	0	0,0299634	0,753898	0,0354348	0,0229405	0	0,063548	0,603667	0,6279808
CG11664-RA	CG11664	0	0	16,1948	0,253741	0	0	-0,013096	0,914398	0,6279808
CG11665-RA	hrm	41,1178	31,9762	39,5724	13,3243	1,14549	0,0923305	0,049897	0,811482	0,6279808
CG11666-RA	CG11666	0	0,407806	24,426	1,83289	0,436563	0	NA	NA	0,6279808
CG11668-RA	CG11668	0,90722	0,603185	0,313135	0,0594948	0,154985	0,339619	-1,190984	0,000182	0,6279808
CG11669-RA	Mal-A7	0,0275929	0,695223	0,0264908	5,89326	5,37168	118,773	-0,99837	0,004842	0,6279808
CG11670-RD	Ras64B	58,7559	10,4333	13,1081	24,8774	49,3715	1,214	-0,03674	0,879734	0,6279808
CG11670-RE	CG11670	3,65156	34,0898	1,50406	0,0535043	1,35047	1,99827	-0,121009	0,618919	0,6279808
CG11671-RA	CG11670	0,0466417	9,08959	0,0459464	3,44545	1,31145	0,0546192	-0,016813	0,961188	0,13772387
CG11672-RA	Nazo	3,34092	33,7571	20,7845	21,1135	35,2613	13,8394	-0,40457	0,223274	0,13772387
CG11674-RA	CG11672	16,7597	2,07706	4,07299	2,26765	7,47975	4,56999	-0,041756	0,887995	0,6279808
CG11678-RA	CG11674	0,0585951	0,962934	1,83544	18,628	0,0154186	6,18778	-0,020785	0,931379	0,13772387
CG11679-RA	Arp6	12,7855	12,8008	19,9359	29,0921	3,6416	5,25746	0,037101	0,895267	0,6279808
CG1167-RA	CG11679	7,62593	10,1155	9,92223	7,93981	27,1435	3,18443	-0,023189	0,921172	0,6279808
CG11680-RA	7B2	0,0587514	0,0535149	0,0519263	0,0675451	0,473051	0,0689526	-0,000396	0,998584	0,6279808
CG11680-RB	7B2	26,9146	20,7733	8,91651	28,874	10,1822	34,3328	-0,003004	0,989248	0,6279808
CG11680-RC	mle	6,87777	7,38318	7,63351	6,49508	3,68358	4,7734	0,03437	0,875526	0,6279808
CG11686-RA	mle	0,0147667	0,0134505	0,0141769	0,0152332	0,0206335	0,0155507	0,276954	0,17921	0,6279808
CG1168-RA	mle	3,11531	1,57416	3,8423	7,04347	3,07037	2,90621	-0,353586	0,19299	0,6279808
CG1168-RB	CG11686	212,342	233,564	197,502	0	260,006	261,652	-0,353586	0,19299	0,6279808
CG11693-RA	Osi18	133,475	412,253	152,861	469,885	64,4705	231,724	0,02178	0,951434	0,6279808
CG11694-RA	CG11693	0,103567	0,849026	8,45824	6,21008	0,644609	0,485816	0,01562	0,897983	0,6279808
CG11695-RA	CG11694	0	68,6204	0	79,489	87,4503	65,6722	0,062285	0,802163	0,6279808
CG11696-RA	CG11695	5,09146	7,60919	5,57607	38,1086	4,63612	36,9626	-0,066593	0,785509	0,6279808
CG11697-RA	CG11696	5,1932	0,0531213	6,9801	293,637	35,953	5,35844	0,067432	0,675386	0,6279808
CG11698-RA	Reepl1	0,228478	0	0,131612	0	0	0	NA	NA	0,6279808
CG11699-RA	CG11698	0	0	0	38,4925	2,90825	0	-0,291014	0,318682	0,6279808
CG1169-RA	CG11699	33,9686	39,3237	0,0559901	44,3611	0,0959148	4,63723	0,538736	0,036672	0,6279808
CG11703-RA	CG11703	0	0	0	0	7,2076	0	NA	NA	0,6279808
CG11709-RA	PGRP-SA	0,0583194	7,74939	55,6933	7,61663	4,90069	7,0304	0,11987	0,73756	0,6279808
CG11710-RA	Akh	6,46993	0,92493	9,64768	60,9137	78,6628	251,488	-0,484615	0,042414	0,13772387
CG11710-RB	CG11710	3,26268	0,0365283	8,58571	10,5014	4,45656	9,98131	-0,484615	0,042414	0,6279808
CG11711-RA	CG11710	4,19073	1,00741	1,24332	26,8146	7,51786	18,3354	0,273847	0,235121	0,6279808
CG11711-RB	Mob2	19,0775	29,0532	20,2906	1,07617	4,82892	2224,45	0,306334	0,183639	0,13772387
CG11711-RC	Mob2	6,17493	9,33228	6,03153	15,5594	9,71834	3,28421	0,298771	0,192319	0,13772387
CG11711-RD	Mob2	0,019266	0,0175488	0,0184965	10,3837	3444,74	7,41427	0,321875	0,161693	0,6279808
CG11715-RA	Mob2	4,94318	5,18618	4,83221	0,0200857	12,1677	13,3476	-0,69331	0,008286	0,13772387
CG11715-RB	Cyp4g15	3,76967	10,592	0,0333527	6,18377	3,89769	3,11407	-0,503953	0,058162	0,6279808
CG11719-RA	Cyp4g15	5,86107	2,00127	115,526	95,2054	1,75147	0,0283875	NA	NA	0,6279808
CG1171-RA	Mst98Ca	0,0496438	5,56519	11,5866	0	0	0	-0,499577	0,080156	0,6279808
CG11720-RA	CG1172	17,7669	15,4654	17,8771	15,866	39,696	11,417	0,008131	0,978938	0,6279808
CG11722-RA	Sgs3	0,14882	0,135555	0,228601	0,234667	0,237621	0,111179	-0,656712	0,041504	0,6279808
CG11723-RA	CG11722	9,35571	9,21396	55,4035	44,2669	9,5116	24,6854	0,131424	0,55305	0,6279808
CG11726-RA	CG11723	8,35046	6,80714	6,90989	8,25255	4,18417	4,95804	-0,311069	0,33896	0,6279808
CG11727-RA	CG11726	0,188573	0,111732	12,3594	0	0	0,0587198	0,146541	0,621	0,6279808
CG11727-RB	Evi5	7,24267	12,2832	5,78838	7,88086	0,0376661	9,17638	0,04191	0,886613	0,6279808
CG1172-RA	Evi5	1,29491	45,0121	1,29414	4,69967	7,05001	5,53558	0,356743	0,066619	0,6279808
CG11732-RA	Obp85a	0	0,781148	0,602439	2,13252	11,1695	5,35889	-0,072603	0,803974	0,6279808
CG11734-RB	HERC2	2,33416	0,0199911	0,021668	0,0239735	1,87272	0,0245535	-0,076402	0,773644	0,13772387
CG11735-RA	Or85b	0	0	0,0811212	9,56847	0,137942	0,103962	0,062944	0,768885	0,6279808
CG11737-RA	CG11737	10,3311	8,47916	10,8405	0,0554868	6,55675	5,1912	0,09957	0,641203	0,6279808
CG11738-RA	l(1)G0004	20,0104	25,1566	14,4258	17,6254	0,0334207	1,58006	-0,19864	0,4848	0,6279808
CG11739-RA	Sfxn1-3	17,2118	8,80908	10,9354	0,0407866	21,2363	0,0188865	-0,223865	0,425116	0,6279808
CG11739-RB	Sfxn1-3	0,0435378	2,72449	4,17691	13,7153	0,0552458	0,0194884	-0,225558	0,421934	0,6279808
CG11739-RC	Sfxn1-3	43,4289	15,2328	34,0944	0,0599822	12,6646	0,018625	-0,224296	0,424207	0,6279808
CG11739-RD	Sfxn1-3	10,234	38,704	19,9614	0,0481481	0,470141	0,0198742	-0,223865	0,425116	0,6279808
CG11741-RA	CG11741	3,02968	12,514	0	13,3525	16,9691	0	0,486135	0,154671	0,6279808
CG11742-RA	Or85d	0,079995	0	30,0325	12,3694	0,129159	2,748	-0,060398	0,703481	0,6279808
CG11748-RA	Obp19a	0	0	58,8673	3,57777	0,0330862	33,567	NA	NA	0,6279808

gene_id	Symbol	W1_FPKM	W2_FPKM	W3_FPKM	MCM51_FPKM	MCM52_FPKM	MCM53_FPKM	FC	p-value	p-adj
CG11750-RA	Pa1	20,8459	31,4377	14,0151	39,1522	32,068	3,80097	-0,533874	0,06956	0,6279808
CG11752-RA	CG11752	64,4773	5,28673	128,089	6,46984	6,57344	4,86048	-0,354627	0,269274	0,6279808
CG11753-RA	CG11753	12,7373	18,0371	19,4568	16,8488	4,87247	0,0414314	0,156275	0,525479	0,6279808
CG11755-RA	CG11755	8,66802	0	0,104681	5,95389	25,245	22,3551	-0,684038	0,011844	0,6279808
CG11756-RA	CG11756	0,486503	0,201427	1,06153	0,995711	0,0238223	1,24834	-0,299399	0,396366	0,6279808
CG11759-RF	Kap3	4,52646	54,1212	0,0169135	0,0185635	0,31371	0,0184615	-0,12449	0,573791	0,6279808
CG11759-RG	Kap3	1,60106	6,691	0,0167262	0,0182955	0,415011	1,23229	-0,130885	0,55212	0,6279808
CG11759-RH	Kap3	0,0178648	1,60501	0,0162839	0,0180846	0,210485	183,809	-0,132113	0,550052	0,13772387
CG11759-RI	Kap3	0,0176171	0,0162725	16,1874	1,78876	3,79306	11,2437	-0,129407	0,559706	0,13772387
CG11759-RJ	Kap3	0,017422	0,0160469	11,335	15,5145	1,12364	8,25729	-0,129457	0,558593	0,6279808
CG11759-RK	Kap3	1,33995	0,0158692	0,0232653	0,025564	4,04072	0,0260967	-0,123504	0,577999	0,6279808
CG11760-RA	Obp84a	0,125699	135,378	0,0984022	0	0	19,6786	0,77319	0,000341	0,6279808
CG11760-RB	Obp84a	0,102496	0,0331484	34,9219	0	0	0	0,782627	0,000392	0,6279808
CG11761-RA	CG11760	24,9006	3,03534	0,222703	0,0463856	0,277746	1,73817	-0,306137	0,30794	0,6279808
CG11762-RA	CG11760	3,90002	1,07705	1,12688	0,248823	5,31074	14,8374	0,014919	0,964394	0,6279808
CG11765-RA	trsn	7,56565	67,627	11,461	0,131122	0,0441284	0,0332579	-0,63263	0,064924	0,6279808
CG11767-RB	ouib	1,31185	1,00625	1,11494	18,4668	0,609936	3,74534	-0,165869	0,443891	0,6279808
CG11768-RA	Prx2540-2	213,338	0,00720739	209,203	0,0239387	761,277	9,67055	-0,168114	0,578126	0,6279808
CG1176-RA	Or24a	0	7,67141	10,6456	0,0573796	0,841564	0,163419	0,072346	0,825542	0,6279808
CG1176-RC	CG11768	0,0461531	0,018385	0,0443097	0,0513767	1,35127	0,0524473	0,050452	0,878417	0,6279808
CG11770-RA	Dsim\GD10612	6,58949	7,75004	0,0530408	0,0303156	0,715196	0,0350973	0,145465	0,587013	0,6279808
CG11771-RA	CG11771	14,8217	20,6233	5,20595	1,6294	16,8085	4,84522	-0,237256	0,234452	0,6279808
CG11775-RA	Ir85a	0,0545181	0,215188	0,0872343	0,0985481	0	0,0624548	0,224229	0,406663	0,6279808
CG11776-RA	Pif1A	5,04869	6,66946	4,19511	9,80116	8,54881	7,68318	-0,650374	0,013576	0,6279808
CG11777-RA	CG11777	0,101395	40,6364	0,0973452	21,4712	6,24825	5,64737	1,200006	2,11E-06	0,6279808
CG11778-RB	CNT1	0,133429	0,0297119	0,0232909	0,885291	0,105168	0,0261271	0,408143	0,237535	0,6279808
CG11779-RA	CG11779	0,0264833	22,6487	22,6865	0,0892212	19,6092	0,0323618	0,034278	0,86552	0,6279808
CG11779-RB	CG11779	14,1947	0,026386	0,027811	0,051869	0,0429395	28,1802	0,086373	0,665681	0,6279808
CG11779-RC	CG11779	0,0289679	0,0373365	0,0393528	5,16881	0,0633199	29,5331	-0,084443	0,673688	0,6279808
CG11779-RD	CG11779	0,04099	17,0279	30,4773	4,37406	30,8511	0,0313658	-0,201307	0,334043	0,6279808
CG11780-RA	beta4GalT7	2,65402	34,7826	59,835	52,9866	3,32956	5,49272	-0,27071	0,386479	0,6279808
CG11781-RA	CG11781	41,3684	8,29902	7,504	5,85983	61,6397	58,7405	0,015894	0,952312	0,6279808
CG11783-RA	Hr96	6,15962	2,36189	4,51028	3,17929	5,44884	5,86934	0,125718	0,560259	0,6279808
CG11784-RA	GstE13	36,3676	15,1638	11,6389	9,89734	7,54312	6,72985	0,199545	0,464137	0,6279808
CG11785-RA	bai	116,588	0,0234401	0,024706	0,0716587	0,0970622	0,0278123	0,12038	0,545374	0,6279808
CG11786-RB	CG11786	55,3795	48,7542	62,3602	59,1767	0,778819	34,3466	0,695425	0,009566	0,6279808
CG11788-RA	Dsim\GD11503	2,07823	24,9048	11,8133	2,62055	2,60839	5,72537	-0,273168	0,350937	0,6279808
CG11790-RA	LysB	1,65853	0,302141	12,7383	88,6957	43,2513	169,787	0,38727	0,077213	0,6279808
CG11790-RB	CG11790	0,0618301	0,0563191	0,0593606	139,294	132,37	0,0731519	0,388492	0,075952	0,6279808
CG11791-RA	CG11790	36,4469	38,4813	42,4046	10,2477	0,0603649	31,1895	0,173205	0,385791	0,6279808
CG11791-RB	CG11791	22,1758	6,13352	6,71883	13,9169	0,0966418	0,0728351	0,268629	0,18061	0,6279808
CG11791-RC	CG11791	24,2386	3,15068	4,73024	31,7503	25,7639	22,0676	0,199733	0,323018	0,6279808
CG11793-RA	CG11791	38,6333	5,32901	6,6906	46,0956	46,6392	33,1547	-0,722444	0,029688	0,6279808
CG11796-RA	Sod1	128,907	84,6963	200,436	152,592	395,412	352,666	-0,900926	0,00985	0,6279808
CG11796-RB	Hpd	5,82089	4,20865	0,0325484	0,0503174	0,0495144	10,3671	-0,903215	0,009562	0,6279808
CG11797-RA	Hpd	22,3677	7,08977	10,5315	55,1213	144,243	48,7241	-0,31318	0,328704	0,6279808
CG11798-RA	Obp56a	25,8897	3,54589	14,5568	103,779	0	8,83207	0,52432	0,072315	0,6279808
CG11798-RB	chn	0,0269185	0,302825	0,239384	0,44493	6,1889	0,0432982	0,511768	0,079288	0,6279808
CG11798-RC	chn	0,0128286	0,201124	0,0258433	1,86211	0,48684	3,78514	0,506692	0,082674	0,6279808
CG11798-RD	chn	16,1182	3,66353	0,0123162	6,89876	0,0387117	0,0291755	-0,047302	0,876627	0,6279808
CG11799-RA	chn	28,1527	25,2984	17,9685	45,0874	0,0178447	0,0134489	0,345451	0,231797	0,6279808
CG11799-RB	FoxK	18,6557	0,0231694	6,14845	24,1362	9,23745	0,020751	0,347833	0,228984	0,6279808
CG11799-RC	FoxK	0,0194876	0,70354	4,94749	0,0203274	0,0275336	0,0215833	0,348609	0,227993	0,6279808
CG11799-RD	FoxK	4,50195	0,574711	0,019957	0,0211427	0,0286379	6,62472	0,35104	0,223949	0,6279808
CG11799-RE	FoxK	12,4611	3,59146	19,9226	10,3134	6,34017	0,0213368	0,345561	0,23208	0,6279808
CG11799-RF	FoxK	0,0200129	7,04038	1,99626	12,2452	6,98257	0,0222031	0,342639	0,235871	0,6279808
CG1179-RA	FoxK	0,0207873	1,83881	35,2427	0,0217499	0,0294603	3,95353	-0,582184	0,092648	0,6279808
CG11801-RB	LysE	4,92198	0,277569	4,16896	13,4293	0,323747	21,1114	-0,137383	0,683487	0,6279808
CG11801-RC	Elo68beta	0,0597431	0,944957	0,057357	8,93192	2,59288	1,07426	-0,131411	0,689575	0,6279808
CG11802-RA	Elo68beta	1,18284	0,189266	1,76213	166,347	1,53976	1,83769	-0,246833	0,297785	0,6279808
CG11804-RA	BORCS5	8,46039	9,57697	14,5676	61,9509	19,7945	19,3568	0,117095	0,646491	0,6279808
CG11804-RB	ced-6	21,3789	0,00882413	21,9425	5,82776	25,3556	21,8606	0,088137	0,727616	0,6279808
CG11804-RC	ced-6	19,9069	0,81548	22,4444	0,00987656	18,7338	20,9934	0,116749	0,647264	0,6279808
CG11807-RA	ced-6	11,5906	0,00957519	0,0288655	1,73119	0,0435684	0,0328357	0,377642	0,078325	0,6279808
CG11808-RA	CG11807	10,7271	11,7333	20,8037	17,4651	11,6445	9,88815	-0,245562	0,426977	0,6279808
CG1180-RA	CG11808	3,85785	29,0715	0,0284385	0,0316555	24,5718	0,0323152	0,133967	0,652814	0,6279808
CG11811-RA	CG11811	46,5033	43,9532	50,4292	5,12102	0,166662	5,7694	0,452009	0,027821	0,6279808
CG11814-RA	mv	3,01894	3,95566	0,314747	0,968229	2,03072	2,20333	0,049393	0,844761	0,6279808
CG11815-RA	CG11815	0,193885	0,580951	0,0704853	0,327881	0	0	-0,09989	0,655425	0,6279808
CG11816-RA	CG11816	0,093769	1,45278	0	0,0905503	0,0345863	1,45833	0,071186	0,575632	0,6279808
CG11820-RA	PQBP1	0,0772817	10,8479	0,850359	22,1948	0,801284	4,36811	0,400823	0,099058	0,6279808
CG11821-RA	Cyp12a5	0,850903	0,491866	3,14199	7,44407	4,44602	8,19846	-0,786593	0,027273	0,6279808
CG11822-RA	nAChRbeta3	0,0418025	2,67985	2,64116	9,17301	53,2882	0,0461661	-0,307458	0,246626	0,6279808
CG11822-RB	nAChRbeta3	7,53708	1,41944	1,6248	2,27315	0,0822192	1,49866	-0,305288	0,249013	0,6279808
CG11825-RA	CG11825	147,305	141,728	130,807	0,026856	516,62	10,3718	-0,241728	0,487241	0,6279808
CG11828-RB	CG11828	0,0433379	3,79792	0,609068	0,33551	2,64063	2,07878	0,672926	0,047079	0,6279808
CG11833-RA	Dsim\GD18001	0,0458647	5,98144	0,0440329	7,03949	8,00271	4,24768	0,480328	0,164142	0,6279808
CG11835-RA	CG11835	0,11068	59,3779	0,106259	116,719	3,49012	75,087	0,20538	0,486814	0,6279808
CG11836-RA	CG11836	1,75286	4,9973	6,76659	6,87371	0	0,103084	0,279782	0,261404	0,13772387
CG11836-RB	CG11836	0,0634937	0,0703936	2,2273	0,10098	3,28097	38,7473	0,285815	0,250161	0,6279808
CG11836-RC	CG11836	0,0620624	53,7632	0,0609577	81,8671	2,33659	5,23739	0,279782	0,261404	0,6279808
CG11836-RD	CG11836	10,007	3,33412	0,0595836	2,10169	0,136778	1,21348	0,279782	0,261404	0,6279808
CG11837-RA	CG11837	9,04357	0,0250464	5,94182	6,97744	12,176	0,237205	0,076262	0,822012	0,6279808
CG11838-RA	rempA	0,0154431	0,458958	87,9632	1,14156	0	8,73486	0,201412	0,481288	0,6279808
CG11838-RB	rempA	1,08924	0,0630093	58,7309	0,142789	45,067	64,2449	0,202092	0,482113	0,6279808
CG11839-RA	CG11839	6,06556	38,3134	40,2497	5,96893	3,76702	49,8507	0,514543	0,049744	0,6279808
CG11840-RA	Spp	86,9935	0,724432	0,647362	0,0159563	0,364922	0,0469883	0,233688	0,30991	0,6279808
CG11841-RA	CG11841	0,442868	4,86122	12,3728	13,0001	9,59064	11,2318	0,060687	0,851426	0,6279808
CG11842-RA	CG11842	2,18677	1,80344	1,95758	0,701839	3,21174	3,86073	0,418307	0,167268	0,6279808
CG11843-RA	CG11843	0,643816	0,617297	0,780761	19,287	1,98695	7,45111	-0,105722	0,763446	0,6279808
CG11844-RA	vig2	0,0456745	0,0368188	0,0438503	33,095	0,258098	0,194518	-0,138212	0,547359	0,6279808
CG11844-RB	vig2	0,0404216	0,0404841	19,0349	5,30803	48,218	3,33342	-0,141898	0,538432	0,6279808
CG11844-RC	vig2	0,0444456	95,6285	0,0426704	4,06653	0,0687856	0,296559	-0,140119	0,542332	0,6279808
CG11844-RD	vig2	106,616	6,08268	137,942	0,470585	19,9928	36,2166	-0,138212	0,547359	0,6279808
CG11847-RA	Clbn	4,25566	2,56705	3,18845	4,7907	28,7686	2,35549	0,080808	0,790076	0,6279808
CG11848-RA	Bili	4,32434	0,382541	8,90615	4,38179	5,21011	6,75869	0,332687	0,211962	0,6279808
CG11849-RA	dan	4,20645	5,36413	4,36152	7,84933	1,23099	0,189833	0,045064	0,878825	0,6279808
CG11851-RA	Alg9	5,06339	3,79311	8,22329	14,7784	3,46056	9,0931	0,016849	0,948049	0,6279808
CG11852-RA	CG11852	20,9922	34,8308	0,672259	0,0507828	29,4394	0,051841	-0,033957	0,919613	0,6279808
CG11853-RA	to	0,381942	3,47266	7,81642	0,0443559	69,7006	15,9747	0,173235	0,571369	0,6279808
CG11854-RA	CG11854	5,38174	0,408505	0,864009	0,049264	2,01446	0,0502906	0,195326	0,579017	0,6279808
CG11856-RA	Nup358	3,87254	3,41137	6,77851	8,10907	2,38981	3,26129	-0,056738	0,851433	0,6279808
CG11857-RA	CG11857	0,0391754	47,0295	24,5195	3,74975	2,83588	0,299519	-0,013559	0,946677	0,6279808
CG11857-RB	CG11857	58,6158	23,4874	39,1322	2,61268	17,0986	0,441168	-0,013728	0,946004	0,6279808

gene_id	Symbol	W1_FPKM	W2_FPKM	W3_FPKM	MCM51_FPKM	MCM52_FPKM	MCM53_FPKM	FC	p-value	p-adj
CG11858-RA	CG11858	32,0307	0	0,0376107	0,550001	2,30813	17,2468	-0,702677	0,009124	0,6279808
CG11859-RA	RIOK2	14,3634	0,0356836	0,23047	29,8763	0,47967	49,9245	-0,099253	0,727734	0,6279808
CG11861-RA	CG42615	0,0205162	0,125979	0,248967	0	0	0	0,006612	0,980591	0,6279808
CG11861-RB	CG42615	13,8799	0	0,167291	88,5558	0	0	0,003451	0,989877	0,6279808
CG11861-RC	CG42615	0,0189673	0	0,0196968	0	0,284674	9,13011	0,003825	0,9888	0,6279808
CG11864-RA	Semp1	0	0	4,89613	0	0	4,19127	NA	NA	0,6279808
CG11865-RA	CG11865	0,137087	1,06033	0	0	4,66992	0	0,01562	0,897983	0,6279808
CG11866-RA	dmpd	21,0751	25,937	19,4122	0,191	0	0,308643	0,043618	0,82852	0,6279808
CG11870-RA	Nuak1	0,0131555	2,30512	8,06754	31,7012	2,98882	7,45171	0,178736	0,513788	0,6279808
CG11870-RB	Nuak1	8,98941	0,0129974	7,1612	0,0534609	0,0183134	0,0138021	0,165952	0,540312	0,6279808
CG11870-RC	Nuak1	0,013941	1,74454	3,47995	0,78483	4,66964	5,36	0,176792	0,518234	0,6279808
CG11870-RD	Nuak1	0,0145713	32,998	3,56933	0,0135203	0,880558	0,014653	0,169279	0,531651	0,6279808
CG11872-RA	Nuak1	3,98509	4,99111	2,91359	6,76222	2,20453	2,44745	-0,029916	0,924737	0,6279808
CG11873-RA	CG11873	2,72374	4,39749	5,68239	8,07445	6,01516	8,25125	-0,229591	0,491327	0,6279808
CG11874-RA	alpha-Man-Ib	13,3619	17,9599	10,844	16,2535	7,96541	1,83641	0,265483	0,319347	0,13772387
CG11875-RA	Nup37	2,6287	2,17673	7,02623	3,65786	4,13355	4,01897	-0,016046	0,961432	0,13772387
CG11876-RA	Pdhb	43,2854	40,9529	0,0202307	61,4486	0,0298845	0,0225228	-0,545775	0,059696	0,6279808
CG11876-RB	Pdhb	52,694	53,2396	0,0185886	77,028	0,027348	0,0206111	-0,629342	0,033306	0,6279808
CG11876-RC	Pdhb	0,0716916	0,0653017	16,0444	0,0852944	13,2821	12,8888	-0,629342	0,033306	0,6279808
CG11876-RD	Pdhb	0,0460565	0,0419515	74,2159	0,0512568	2,99845	4,44641	-0,545775	0,059696	0,6279808
CG11877-RA	Atg14	11,048	11,7217	15,3755	6,76778	1,37061	14,2263	-0,219604	0,339716	0,6279808
CG11878-RA	CG11878	0,147272	0,914283	0,659818	0,0476605	0,610484	2,58547	-0,125355	0,709438	0,6279808
CG11880-RA	Ctl2	0,0210723	0,0191941	96,6723	7,79188	2,43354	3,47647	0,04682	0,823678	0,6279808
CG11880-RB	Ctl2	0,0193619	0,0176362	0,0688282	0,0201904	106,417	2,0611	0,046049	0,826357	0,6279808
CG11880-RC	Ctl2	16,9879	19,1971	0,044217	13,9779	103,638	0,903298	0,04682	0,823678	0,6279808
CG11881-RA	dgt6	0,703586	0,54335	2,01177	13,998	14,1519	1,1614	-0,009706	0,977938	0,6279808
CG11882-RA	CG11882	4,46557	1,98758	1,50099	2,96001	0,115532	101,792	0,207221	0,489371	0,6279808
CG11883-RA	CG11883	4,25255	2,45686	6,33811	6,33759	6,44129	4,88017	-0,09453	0,700591	0,6279808
CG11883-RB	CG11883	0,0223481	0,0203562	0,0214555	0,0234697	0,0317899	0,0239588	-0,093385	0,703973	0,6279808
CG11883-RC	CG11883	2,81039	3,44719	3,88059	2,08205	1,75339	1,33585	-0,066555	0,785241	0,6279808
CG11885-RA	CG11885	7,98998	6,12262	13,5762	3,55522	15,7565	2,78091	-0,479724	0,118016	0,6279808
CG11886-RA	Slbp	7,78582	9,32998	15,8308	2,69829	3,06821	12,3321	-0,16064	0,538756	0,6279808
CG11887-RA	Elp2	4,87869	3,34762	8,01346	4,48598	3,85334	3,88585	0,235719	0,401504	0,6279808
CG11888-RA	Rpn2	13,722	12,5078	15,1855	0,0205301	0,0278081	2,1985	-0,11411	0,624032	0,6279808
CG11889-RA	CG11889	2,02696	0,111788	4,48908	1,07198	9,10097	5,85408	-0,551658	0,112824	0,6279808
CG11891-RE	CG11891	0,0514743	0,765793	0,0494184	3,67422	0,0645565	1,66783	-1,290067	0,000108	0,6279808
CG11892-RA	CG11892	0,0431398	0,765846	1,06476	11,9073	9,59256	6,90852	0,562332	0,104359	0,6279808
CG11892-RB	CG11892	0,70165	1,1199	3,41875	0,0428558	0,0580484	0,0486537	0,5503	0,109886	0,6279808
CG11893-RA	CG11893	1,98075	1,73481	2,29172	25,671	44,2126	45,6355	-3,043928	4,71E-24	0,6279808
CG11895-RA	stan	3,90696	4,90128	3,53263	6,81851	8,96348	4,22463	0,120186	0,701309	0,6279808
CG11896-RA	m-cup	5,10266	5,42998	5,37294	0	0,027066	1,9645	-0,075529	0,720141	0,6279808
CG11897-RA	rdog	9,2622	0,288555	3,13428	3,38956	7,49482	0,0209579	-0,458242	0,090661	0,6279808
CG11897-RB	rdog	0,0132798	4,24325	0,0188875	24,8538	25,9125	5,03462	-0,459783	0,089648	0,6279808
CG11898-RB	CG11898	0,583562	1,75394	4,88333	18,0583	21,9609	20,2113	-0,716652	0,035392	0,6279808
CG11899-RA	Dsim\GD21426	2,24366	0,109297	188,899	8,67287	124,995	121,582	-0,249434	0,437713	0,6279808
CG11900-RA	Mesh1	4,15311	0,721189	0,0353868	8,51384	0,0543046	0,0409272	0,035244	0,91811	0,6279808
CG11901-RA	eEF1gamma	412,756	0,0231783	0	0,467215	0	0	-0,151874	0,44007	0,6279808
CG11901-RB	eEF1gamma	0,0396217	0,0221057	3,05671	2,01878	0	0,0398601	-0,151874	0,44007	0,6279808
CG11902-RA	CG11902	2,55201	2,71691	2,93385	0,172988	1,76493	2,08237	0,263462	0,327855	0,6279808
CG11905-RA	CG11905	0,0412802	0,0376008	0,0396314	0,0453949	0,0614877	0,0463409	-0,155611	0,60155	0,6279808
CG11905-RB	CG11905	0,0423382	0,0385646	0,0406472	0,0466813	0,0632302	0,0476541	-0,132206	0,655583	0,6279808
CG11905-RC	CG11905	17,9476	20,8692	17,1866	37,7438	10,032	17,9451	-0,053496	0,806403	0,6279808
CG11905-RD	CG11905	0,0450517	0,0410362	0,0432523	0,0500119	0,0677414	0,051054	-0,155611	0,60155	0,6279808
CG11905-RE	CG11905	0,0463148	0,0421867	0,044465	0,0515778	0,0698624	0,0526525	-0,041026	0,851254	0,6279808
CG11905-RF	CG11905	16,9033	13,8039	16,6418	18,1004	12,0343	16,4158	-0,122836	0,678412	0,6279808
CG11905-RG	CG11905	0,0303286	0,0276254	0,0291172	7,26391	0,0439762	0,0331431	-0,167209	0,574881	0,6279808
CG11905-RH	CG11905	0,0311287	0,0283542	0,0298854	0,0333879	0,0452241	0,0340836	-0,041026	0,851254	0,6279808
CG11905-RI	CG11905	8,57226	9,89836	10,2283	15,053	4,75195	6,0348	-0,122836	0,678412	0,6279808
CG11905-RJ	CG11905	0,0318489	0,0290102	0,0305768	0,0342202	0,0463515	0,0349333	-0,169878	0,568296	0,6279808
CG11906-RA	CG11906	3,73635	0,269895	0,142235	18,4058	17,1936	0,263992	0,110158	0,663038	0,6279808
CG11907-RA	Ent1	7,78308	12,3437	18,3763	4,96177	10,2891	12,9808	0,339924	0,081012	0,6279808
CG11907-RB	Ent1	26,9652	23,8535	6,1872	0,0118265	3,2659	3,9056	0,339924	0,081012	0,6279808
CG11908-RA	rha	0,184655	3,27807	0,28808	3,47725	1,27435	1,60406	0,625349	0,055006	0,6279808
CG11909-RA	tobi	2,51088	4,0024	2,87051	2,66185	1479,94	1688,65	0,503194	0,063721	0,6279808
CG11910-RA	alrm	86,0735	0,189266	0,898921	0,0189642	0,0256872	0,0193594	-0,324315	0,251763	0,6279808
CG11911-RA	CG11911	20,8194	15,5218	86,3736	21,6353	2,9921	236,869	-0,747555	0,022953	0,6279808
CG11912-RA	CG11912	3,16399	8,30051	8,54772	0,0461314	0,0160191	8,90803	-0,575045	0,105699	0,6279808
CG11913-RB	Dpse\GA11278	0,116605	2,01548	0,370254	0,0193258	0,0261769	3,17433	0,094143	0,593923	0,6279808
CG11915-RA	Lmpt	1,73595	2,64204	7,92189	5,8336	5,96399	5,38098	-0,219596	0,440962	0,6279808
CG11919-RA	Pex6	7,02226	6,12403	8,61231	8,06972	0,0288322	0,0217297	-0,413085	0,120534	0,6279808
CG11920-RA	CG11920	20,4724	9,3763	53,4741	5,98865	7,68543	8,34381	-0,094674	0,768388	0,6279808
CG11921-RA	fd96Ca	0,59049	0,0578344	11,9938	0,036536	29,3862	0,0754494	0,043861	0,897645	0,6279808
CG11922-RA	fd96Cb	4,84557	0,0565307	0,0741951	3,35912	7,73868	0,0734715	0,234568	0,403537	0,6279808
CG11924-RA	Cf2	1,18638	0,0170178	4,27178	8,95318	0,0421089	17,5751	0,212106	0,409254	0,6279808
CG11924-RB	Cf2	0,0190055	3,33247	16,1848	22,8935	25,6517	0,0316462	0,213715	0,405768	0,6279808
CG11924-RC	Cf2	9,0808	9,82286	3917,35	899,982	0,0952613	24,8832	0,212837	0,40777	0,6279808
CG11926-RA	Mon1	6,10255	5,86211	0	10,1558	0	0,803564	0,01744	0,935637	0,6279808
CG11927-RA	CG11927	8,12748	7,19696	10,8987	1,24897	1,15989	49,9834	0,050388	0,830824	0,6279808
CG11929-RA	CG11929	0,0326354	0,0752707	2,86552	0,552409	8,53968	1,35748	-0,41273	0,191272	0,6279808
CG11937-RA	kat-60L1	4,80009	8,07596	0,971238	8,0178	0,0393605	1,54011	-0,29236	0,412829	0,6279808
CG1193-RA	kat-60L1	2,05063	1,68107	12,289	1,23826	5,76352	6,95202	0,322192	0,212653	0,6279808
CG1193-RB	amn	0,0167154	3,85285	30,0287	13,552	0,164208	0,0360761	0,334478	0,196549	0,6279808
CG11940-RA	pico	0,0179912	0,00937971	5,32341	9,75973	5,68948	0,0107323	-0,137425	0,642014	0,6279808
CG11940-RB	pico	6,94771	3,24273	3,2714	0,0105132	64,109	2,97684	-0,291695	0,327877	0,6279808
CG11941-RA	SkpC	0	0,298544	0,0449056	49,2017	3,41133	0,0532359	-0,59064	0,082818	0,6279808
CG11942-RA	SkpE	0,655515	0,0426048	0,0453227	1,47513	1,84401	0,0537894	-0,196909	0,572769	0,6279808
CG11943-RA	Dsim\GD17470	0,0102975	1,76987	8,55436	6,92287	0,0330128	55,3787	-0,313542	0,25539	0,6279808
CG11943-RB	Dsim\GD17470	2,86261	8,40656	0,00988625	3,98286	38,017	0,0248804	-0,313542	0,25539	0,6279808
CG11949-RA	cora	0,0231142	6,91793	1,04591	3,88714	50,6937	465,861	-0,169703	0,424098	0,6279808
CG11949-RB	cora	22,2015	0,0281948	80,1744	0,0331861	3,04999	2,90635	-0,010257	0,961062	0,6279808
CG11949-RC	cora	20,0429	7,3642	14,5599	10,7175	0,0449508	0,0338776	0,032516	0,877108	0,6279808
CG11949-RD	cora	51,8721	0,0277272	0,0311678	0,0325951	7,39004	5,6702	0,033023	0,875083	0,6279808
CG11951-RA	CG11951	0,656738	0,708076	161,035	0,706895	0,533703	0,869972	-0,093207	0,779156	0,6279808
CG11956-RA	SP1029	0,0202397	31,7991	0,0194313	0,0211495	13,6801	20,206	-0,046264	0,817446	0,6279808
CG11956-RB	SP1029	0,0218601	0,0184357	0,020987	0,0229306	0,0286471	0,0215902	-0,042973	0,82998	0,6279808
CG11956-RC	SP1029	16,4373	0,0199117	21,7574	23,1312	0,0310597	0,0234085	-0,048672	0,808477	0,6279808
CG11958-RA	Cnx99A	16,3748	34,6551	62,7176	33,5218	6,40254	5,77357	-0,150084	0,560605	0,6279808
CG11958-RB	Cnx99A	0,0242688	518,984	0,0308745	11,7423	56,2677	49,6398	-0,150185	0,560425	0,6279808
CG11958-RC	Cnx99A	45,0622	0,0360902	9,74068	25,5816	0,0364658	6,93149	-0,148911	0,563946	0,6279808
CG11958-RD	Cnx99A	0,0321589	0,141283	0,0527029	0,0256037	0,0346804	0,0261372	-0,229758	0,387655	0,6279808
CG11961-RA	CG11961	37,8982	38,8215	20,8514	0,0243816	10,3814	23,4354	0,045917	0,808798	0,6279808
CG11961-RB	CG11961	17,2867	17,433	48,2729	108,571	39,0631	25,7569	0,051192	0,787517	0,6279808
CG11963-RA	ScsbetaA	0,0383122	0,0348974	16,1867	16,6142	11,8122	13,9674	-0,668296	0,032194	0,6279808
CG11963-RB	ScsbetaA	32,4306	27,6534	26,7257	27,8894	29,062	22,8021	-0,668296	0,032194	0,6279808

gene_id	Symbol	W1_FPKM	W2_FPKM	W3_FPKM	MCM51_FPKM	MCM52_FPKM	MCM53_FPKM	FC	p-value	p-adj
CG11964-RA	CG11964	5,43349	6,69005	20,6747	19,916	15,7575	16,8303	-0,217983	0,30609	0,6279808
CG11966-RA	ich	7,27847	6,46832	4,8246	15,5023	3,90102	4,4998	-0,232508	0,472491	0,6279808
CG11967-RA	CAHbeta	20,6803	22,5323	0,036782	0,0418222	1,5048	10,9962	-0,122142	0,5783	0,6279808
CG11968-RA	RagA-B	16,2182	18,5491	58,8827	44,2266	10,4469	0,566326	0,052399	0,791584	0,6279808
CG11970-RA	CG11970	4,29438	4,90419	5,82847	5,46572	2,92442	5,62235	0,022031	0,92452	0,6279808
CG11971-RA	E(var)3-9	5,89908	3,67168	6,60737	6,98082	4,54949	3,89286	-0,058325	0,791118	0,6279808
CG11975-RA	CG11975	13,4978	14,2699	7,99151	10,1602	11,6896	13,2319	0,035201	0,86068	0,6279808
CG11977-RA	CG11977	0,129561	0,118013	16,8379	16,3806	0,0911167	11,2625	-0,332872	0,293155	0,6279808
CG11979-RA	Rpb5	21,1491	14,8072	6,40252	2,40567	0,0482561	0,0363687	-0,149377	0,631108	0,6279808
CG11980-RA	CG11980	0,0554793	0,0509192	0,0127136	0,0666568	26,4446	0,0169581	0,010283	0,972534	0,6279808
CG11980-RB	CG11980	0,0559018	21,1087	0,0126528	0,0672693	0,0175915	0,0185531	0,010283	0,972534	0,6279808
CG11980-RC	CG11980	31,2165	16,5035	19,1046	35,3567	0,0184382	0,0162533	0,010283	0,972534	0,6279808
CG11981-RA	Prosbeta3	19,3851	69,751	9,39668	24,3612	0,0183473	0,704821	-0,052662	0,860965	0,6279808
CG11982-RA	Iru	10,9849	14,7624	0,124386	0,175705	316,575	0,0138962	0,02461	0,912331	0,6279808
CG11983-RA	CG11983	1,44556	1,7627	16,1236	14,0471	0,237993	3,8734	-0,014079	0,961817	0,6279808
CG11984-RA	Kcmf1	3,10285	4,98349	0,0154135	0,0267905	7,98196	0,0426936	-0,288365	0,149209	0,6279808
CG11984-RB	Kcmf1	5,49388	0,024001	0,0252089	0,0279377	5,51744	68,528	-0,288657	0,149282	0,6279808
CG11984-RC	Kcmf1	17,4441	25,2812	0,0147952	35,2114	0,022501	7,04703	-0,288232	0,149166	0,6279808
CG11985-RA	Sf3b5	86,4365	0,0505344	0,0121464	293,145	30,3219	4,40737	-0,134	0,573755	0,6279808
CG11986-RA	CG11986	0,0523581	0,0476914	0,763814	0,0621901	0,0246173	0,0680458	0,089111	0,724303	0,6279808
CG11987-RA	tgo	26,4722	27,8489	101,056	34,8468	0,0215658	0,068671	0,233927	0,380758	0,6279808
CG11988-RA	neur	4,23298	4,08519	13,6132	10,695	8,49587	10,9421	0,299807	0,253894	0,6279808
CG11988-RB	neur	0,0167847	0,0152887	33,4476	0,017397	28,2109	30,5	0,299807	0,253894	0,6279808
CG11988-RC	neur	8,63996	7,78848	10,7429	3,58367	8,56158	6,72335	0,324164	0,213953	0,6279808
CG11988-RD	neur	0,0178551	0,0162637	0,0183839	0,0185531	0,0270332	0,0203739	0,324164	0,213953	0,6279808
CG11988-RE	neur	0,0198565	0,0180867	17,959	0,0207303	14,0897	12,1424	0,252538	0,343508	0,6279808
CG11989-RA	vnc	20,9436	15,5765	38,9229	38,5341	0,0842368	0,186322	-0,298308	0,249791	0,6279808
CG11989-RB	vnc	0,0573243	0,0522149	0,0550347	0,0656605	29,3801	0,931702	-0,304856	0,239963	0,6279808
CG11989-RC	vnc	0,0594385	0,0541407	0,0570645	0,0684574	0	43,9085	-0,299279	0,248225	0,6279808
CG11989-RD	vnc	0,0546683	0,0497957	0,0524849	0,0621901	0,39109	0,0670287	-0,300352	0,246429	0,6279808
CG11989-RE	vnc	23,7837	26,8026	30,4165	20,5057	0,02683	0,0698839	-0,299624	0,247644	0,6279808
CG11990-RA	hyx	0,0293844	0,0267654	7,08941	0,0313842	0,0314316	3,35607	0,254014	0,230085	0,6279808
CG11990-RB	hyx	13,51	14,0836	3,72233	16,3548	8,37898	4,02288	0,256427	0,225275	0,6279808
CG11992-RA	Rel	0,0191487	0,017442	0,0190634	0,019958	2,22623	2,88719	-0,318205	0,162718	0,6279808
CG11992-RB	Rel	13,556	11,9644	0,0282108	18,896	0,0251303	0,0189397	-0,318753	0,162115	0,6279808
CG11992-RC	Rel	0,0221088	2,15479	17,7428	0,0232052	0,0280793	0,0211623	-0,306236	0,179771	0,6279808
CG11992-RD	Rel	4,68869	3,71412	10,5395	13,1653	0,04251	0,0320381	-0,284003	0,209737	0,6279808
CG11993-RA	Mst85C	7,64292	9,91987	11,3699	1,76271	8,70358	8,24971	-0,012886	0,952989	0,6279808
CG11994-RA	Ada	2,92235	1,82129	5,31593	1,68405	2,34357	3,44453	0,324128	0,342151	0,6279808
CG11997-RA	CG11997	0,500575	0,407963	9,05296	0,0102308	0,20508	0,436808	0,570078	0,111137	0,6279808
CG11999-RA	CG11999	26,1672	21,8043	44,1391	39,8508	0,336143	44,6166	-0,359329	0,188821	0,6279808
CG12000-RA	Aplip1	5,77825	11,178	11,3373	11,2614	9,33799	1,84294	0,002054	0,994243	0,6279808
CG12000-RB	Aplip1	4,78304	3,42867	1,88843	20,7251	0,0408373	5,7034	0,002671	0,992514	0,6279808
CG12001-RA	Prosbeta7	32,6408	28,6154	58,9946	40,9651	1,39817	46,3713	-0,798396	0,003529	0,6279808
CG12002-RA	Prosbeta7	0,0568801	0,0518104	7,09011	0,0650769	2,15473	0,0664329	0,631661	0,009037	0,6279808
CG12002-RB	spartin	1,76035	1,54618	77,4617	0,103194	3,76849	0,0296775	0,065694	0,83185	0,6279808
CG12002-RC	Pxn	17,2476	31,4619	0,025949	0,215389	0,0675363	0,0293026	0,631805	0,009023	0,6279808
CG12002-RD	Pxn	0,0128461	0,0426955	37,0807	3,0716	0,0297748	24,5293	0,631805	0,009023	0,6279808
CG12002-RE	Pxn	2,56323	24,0478	34,2651	0,0219821	1,61897	22,4655	0,631559	0,009061	0,6279808
CG12003-RA	Pxn	0,0126035	0,0117011	0,207219	2,31665	3,52932	1,75703	0,234841	0,450478	0,6279808
CG12004-RA	Pxn	0,0270286	0,0116738	0,0431347	4,83945	0,113331	0,373851	0,180496	0,436331	0,6279808
CG12004-RB	CG12003	0,895882	44,8778	17,9195	12,0721	0,702154	19,2463	0,202749	0,383655	0,6279808
CG12004-RC	CG12004	8,74665	18,2611	0,0301984	14,966	11,527	4,85065	-0,124612	0,584069	0,6279808
CG12005-RB	CG12004	4,93839	8,34945	31,805	0,0515778	5,85401	13,8273	0,300304	0,143394	0,6279808
CG12006-RA	CG12004	31,6054	0,0164864	20,0841	0,644347	15,7761	16,3544	0,163386	0,558668	0,6279808
CG12006-RB	Mms19	10,123	0,534516	0,139217	24,2182	28,668	6,94939	0,162569	0,560243	0,6279808
CG12007-RA	PIG-B	11,5214	11,3998	15,1676	22,2036	0,0262462	7,0768	-0,023984	0,907267	0,6279808
CG12008-RA	PIG-B	3,8477	2,31743	3,06076	0,0337124	4,32919	0,0269434	-0,176471	0,435702	0,6279808
CG12008-RB	CG12007	12,9054	12,7929	16,2181	14,5753	0,419279	0	-0,174564	0,440838	0,6279808
CG12008-RC	kst	2,73433	4,81773	0,0630792	40,4368	0,135738	0,00505236	-0,156605	0,493749	0,6279808
CG12008-RE	kst	6,96148	0,00449164	2,4378	19,1085	0,102762	11,3198	-0,154798	0,499628	0,6279808
CG12009-RA	kst	0,00490808	9,07203	0,0369033	28,4753	0,130194	0,0443546	0,280822	0,40254	0,6279808
CG1200-RA	kst	13,7011	0,00447062	0,0786405	3,59746	1778,94	0,102301	0,198308	0,396674	0,6279808
CG1200-RB	CG12009	27,9693	40,3196	29,0891	39,4908	0	462,256	0,198308	0,396674	0,6279808
CG12010-RA	CG12010	0,029595	0,0746112	0,882006	23,6907	0,0227999	0,0518809	-0,201248	0,394017	0,6279808
CG12010-RB	CG12010	2,94593	0,0591415	3,51988	1,78722	24,2217	5,8384	-0,201248	0,394017	0,6279808
CG12011-RA	CG12011	121,591	103,43	80,7444	8,32035	44,3192	13,2874	-0,325722	0,321685	0,6279808
CG12012-RA	CG12012	13,7882	1,61612	12,0572	0,0316252	7,67809	9,10784	0,028863	0,914343	0,6279808
CG12013-RA	PHGPx	5,31739	0,0269572	0,028413	7,36102	1,17782	7,7663	-0,602123	0,051807	0,6279808
CG12013-RC	PHGPx	27,2528	3,02915	3,46973	54,8502	0,0428365	77,0632	-0,577762	0,062779	0,6279808
CG12013-RD	PHGPx	0,0711512	6,54566	1,40971	0,0845284	3,57645	0,0862898	-0,603078	0,051406	0,6279808
CG12014-RA	Ids	1,38678	0,0350125	0,0157503	3,68277	0,529265	0,0524473	0,054485	0,849633	0,6279808
CG12015-RA	RabX6	6,64689	7,42243	9,70086	472,694	0,682719	1009,7	-0,182639	0,431238	0,6279808
CG12016-RA	CG12016	0,028432	3,94234	14,0388	0,0310587	2,46882	0,0317059	-0,169181	0,442162	0,6279808
CG12016-RB	CG12016	13,0827	0,0648094	0,0683094	6,5467	86,4395	8,86315	-0,140375	0,523107	0,6279808
CG12017-RA	CG12017	33,4935	20,8037	34,8825	52,5765	0,0899232	13,86	0,379361	0,250287	0,6279808
CG12018-RA	CG12018	2,37342	0,295015	3,91923	0,334303	0,0433778	6,89676	-0,110083	0,731079	0,6279808
CG12019-RA	Cdc37	22,7097	0,0506335	0,0533679	42,3295	0,0858575	5,3642	-0,161629	0,428206	0,6279808
CG12020-RA	CG12020	0,0555881	5,12975	7,04774	0,0633866	3,8175	27,5486	-0,041253	0,908226	0,6279808
CG12021-RA	Patj	0,0206186	0,0187692	0,0197828	25,3115	0,0235271	2,13899	0,246745	0,293557	0,6279808
CG12021-RB	Patj	0,0206058	8,09704	8,20492	55,1889	0,0292096	4,35848	0,248632	0,290418	0,6279808
CG12021-RC	Patj	8,49428	24,4491	16,2131	44,2402	0,0291905	15,4864	0,246745	0,293557	0,6279808
CG12022-RA	CG42676	4,44839	8,91122	6,37785	6,9905	3,95092	0,0647074	0,035583	0,885659	0,6279808
CG12023-RA	GV1	17,929	0,0639827	66,8725	10,0001	56,2547	0,0177315	-0,305032	0,258467	0,6279808
CG12023-RB	GV1	48,9636	61,0041	40,8609	19,6533	70,9219	2402,74	-0,530741	0,072659	0,6279808
CG12024-RA	CG12024	5,15556	5,4617	5,73872	1,57872	6,83447	167,977	0,282877	0,140289	0,6279808
CG12024-RB	CG12024	10,9444	11,5779	13,4751	13,0441	171,135	1,74242	0,28635	0,134322	0,6279808
CG12025-RA	CG12025	22,0968	0,874078	24,1235	2,84406	44,6467	20,303	-0,151906	0,456366	0,6279808
CG12026-RA	Tmhs	20,0792	3,62987	22,1572	28,9713	0,139124	0,0534474	0,216361	0,364661	0,6279808
CG12026-RB	Tmhs	0,0469399	2,04337	0,0450651	0,0523564	39,3385	1,70358	0,212281	0,373228	0,6279808
CG12027-RA	Dpse\GA11349	0	139,391	0	0	12,9127	0	-0,041811	0,731452	0,6279808
CG12028-RA	dib	0,0397887	0,0362423	5,40611	0,043593	0,0590469	0,0445013	0,722719	0,006871	0,6279808
CG12029-RB	dar1	5,70605	0,0791233	4,43319	0	0,487672	0	0,402312	0,198841	0,6279808
CG12030-RA	Gale	109,851	69,8342	1,24996	11,7082	5,28908	8,03162	-0,455247	0,06787	0,6279808
CG12031-RA	MED14	0,90819	0,0110299	0,0116256	16,4878	1,38608	1,59258	-0,013504	0,959471	0,6279808
CG12031-RB	MED14	3,75426	5,04185	4,28824	26,1824	1,66106	1,76158	0,026987	0,919005	0,6279808
CG12034-RA	nSMase	0,0384386	0,0258978	0,0272964	0,0434492	45,3618	33,3501	-0,213238	0,395665	0,6279808
CG12035-RA	CG12035	0,131625	0,0223991	2,02598	0,0265209	0,042089	0,0317208	-0,381314	0,230093	0,6279808
CG12038-RA	CG12038	9,7539	9,26988	11,3274	12,7178	14,8131	2,53121	-0,447513	0,069717	0,6279808
CG12038-RB	CG12038	0,0467077	0,0425446	0,0448422	0,0520668	0,0705249	0,669029	-0,447513	0,069717	0,6279808
CG12042-RA	DCTN4-p62	8,56562	3,77249	9,70373	8,93092	37,3042	43,6076	-0,323643	0,157974	0,6279808
CG12045-RA	Cpr100A	51,6667	2,7666	5,37756	52,7112	53,3869	47,6861	0,202904	0,362641	0,6279808
CG12047-RA	CG32599	0,0111283	4,31745	5,89	5,34625	4,5732	5,43216	0,118039	0,709937	0,6279808
CG12047-RB	CG32599	0,00871637	9,54421	8,59855	4,95065	5,05106	69,9285	0,099626	0,751693	0,6279808

gene_id	Symbol	W1_FPKM	W2_FPKM	W3_FPKM	MCM51_FPKM	MCM52_FPKM	MCM53_FPKM	FC	p-value	p-adj
CG12047-RC	CG32599	1,15842	0,0533725	0,0562548	1,69144	0,0912098	7,02616	-0,129249	0,694685	0,6279808
CG12048-RA	ppk21	0	0,109231	0,0575651	5,49521	3,82127	0	0,053427	0,804504	0,6279808
CG12050-RB	l(2)05287	7,55098	14,1248	13,2813	9,95795	30,1204	7,92539	-0,133007	0,649247	0,6279808
CG12051-RA	Act42A	193,767	0,0272872	0,0287608	0,0320404	0,0433989	0,032708	0,042656	0,849791	0,6279808
CG12052-RA	lola	0,0169483	0,0154377	0,0162714	0,17898	32,8751	31,6008	-0,153632	0,550909	0,6279808
CG12052-RB	lola	0,0168018	0,0153042	0,0161307	1,1955	72,101	70,5282	-0,209658	0,406144	0,6279808
CG12052-RC	lola	0,0229218	0,0208787	0,0220063	0	38,7889	36,3071	-0,221545	0,381007	0,6279808
CG12052-RD	lola	0,0423262	0,0257024	0,0406356	1,52344	6,6601	8,10635	-0,134419	0,595163	0,6279808
CG12052-RE	lola	0,0203644	0,0185493	0,0195511	3,18572	3,7572	6,04455	-0,128631	0,610877	0,6279808
CG12052-RF	lola	0,0194876	0,0177506	0,0187092	0,529091	5,74746	30,0764	-0,139818	0,568481	0,6279808
CG12052-RG	lola	0,0207873	0,0189345	0,019957	14,7416	4,05002	0,0696668	-0,251652	0,324041	0,6279808
CG12052-RH	lola	0,0181146	0,0165	0,0173911	0	0,318161	15,0594	-0,259503	0,308422	0,13772387
CG12052-RI	lola	37,6596	36,895	42,9647	39,0978	7,14919	0,0791796	-0,11275	0,66833	0,6279808
CG12052-RJ	lola	0,0316051	0,0287882	0,0303428	17,474	1,66468	4,61491	-0,235146	0,352583	0,6279808
CG12052-RK	lola	0,0226003	0,0205859	0,0216977	13,5888	0,0145703	6,33572	-0,245183	0,337562	0,6279808
CG12052-RL	lola	0,0215394	0,0196196	0,0206792	0,0349647	1,31173	7,57411	-0,258475	0,308398	0,6279808
CG12052-RM	lola	34,6361	41,3626	34,4736	15,203	0,0834888	2,46731	-0,255976	0,314296	0,6279808
CG12052-RN	lola	0,03019	0,0274991	0,0289842	23,6269	0	0,0109811	-0,235257	0,35474	0,6279808
CG12052-RO	lola	0,03019	0,0274991	0,0289842	107,181	0	1,52619	-0,195225	0,431616	0,6279808
CG12052-RP	lola	0,0257038	0,0234128	0,0246772	0,00680311	0,0400853	0	-0,023288	0,927363	0,6279808
CG12052-RQ	lola	0,03306	0,0301133	0,0317395	5,54564	122,772	4,77184	-0,252056	0,32335	0,6279808
CG12052-RR	lola	0,0277435	0,0252707	0,0266354	1,3346	0,0924379	0	-0,238695	0,348088	0,6279808
CG12052-RS	lola	0,0196265	0,0178772	0,0188426	0,00746435	13,835	0	-0,230447	0,363126	0,6279808
CG12052-RT	lola	36,3512	32,6895	53,5407	17,2803	0,10506	0,0302107	-0,246486	0,329923	0,6279808
CG12052-RU	lola	0,024928	0,0227062	0,0239324	198,437	6,42281	150,134	-0,239646	0,343092	0,6279808
CG12052-RV	lola	0,0234672	0,0213756	0,0225299	15,0355	30,5139	0,227614	-0,261853	0,303832	0,6279808
CG12052-RW	lola	0,0232198	37,8377	0,0222924	10,4211	30,0597	2,4558	-0,248943	0,327826	0,6279808
CG12052-RX	lola	0,0238828	0,0217541	0,0229289	2,96516	7,00143	34,7544	-0,263247	0,300795	0,6279808
CG12052-RY	lola	0,0284851	0,0259462	0,0273474	17,2186	405,552	379,563	-0,232927	0,359495	0,6279808
CG12052-RZ	lola	0,03306	0,0301133	0,0317395	0	40,7888	41,6707	-0,230728	0,362909	0,6279808
CG12054-RA	CG12054	5,21142	6,91975	2,53995	6,97231	3,83266	6,67817	-0,195085	0,462805	0,6279808
CG12054-RB	CG12054	7,94871	11,5302	5,16833	10,9625	4,9909	4,50602	0,054071	0,839036	0,6279808
CG12055-RA	Gapdh1	193,651	0,00782591	0,00823676	0,00872446	0,0118173	0,00896561	-0,402137	0,166303	0,6279808
CG12055-RB	Gapdh1	0,0445556	0,00782896	0,00823676	0,00872446	0,0118173	0,0089513	-0,402179	0,166277	0,6279808
CG12056-RA	CG12056	8,4748	8,03323	10,4515	10,2312	447,293	10,3992	-0,012984	0,958018	0,6279808
CG12057-RA	CG12057	1,46919	0,274511	15,8773	1,53274	7,14877	2,85964	0,280664	0,31732	0,6279808
CG12058-RA	Larp7	2,69928	1,752	3,78634	3,08251	1,33565	0,778609	0,05807	0,850076	0,6279808
CG12061-RA	CG12061	0	0	0,0598083	0,0537663	0	0,141897	0,01861	0,939792	0,6279808
CG12061-RB	CG12061	0	0	0,0548919	6,70528	0	0,0668289	0,01861	0,939792	0,6279808
CG12061-RC	CG12061	0	0	0,207661	6,12015	0	0,0548866	0,095338	0,712765	0,6279808
CG12063-RA	mey	35,538	33,9628	0,0234026	54,9783	12,176	0,0540487	0,088509	0,772183	0,6279808
CG12065-RA	Dsim\GD16908	14,231	14,7577	6,64669	0,35457	11,3305	0,428462	-0,303876	0,167994	0,6279808
CG12065-RB	Dsim\GD16908	9,09914	8,85003	16,8857	5,01972	0,26753	12,8465	-0,296608	0,177555	0,6279808
CG12065-RC	Dsim\GD16908	6,6496	5,52972	10,7222	24,912	3,47513	2,47311	-0,299718	0,173925	0,6279808
CG12065-RD	Dsim\GD16908	4,50074	3,55519	8,866	0,0706597	2,75298	6,93708	-0,292321	0,183924	0,6279808
CG12066-RB	Pka-C2	0,122363	19,4069	0,0201921	0,0262149	2,88026	0	-0,113287	0,670555	0,6279808
CG12068-RA	sro	0,0655515	0,0462735	0,0629333	0,0572214	1,4143	1,67119	-0,086455	0,799271	0,6279808
CG12069-RA	CG12069	0,0565395	61,8243	29,958	0,0258955	0,16951	0,060471	0,140226	0,517138	0,6279808
CG12070-RA	Dsim\Sap-r	53,9326	0,81578	0,0264246	5,01527	7,94594	0,193839	0,086009	0,749014	0,6279808
CG12070-RB	Dsim\Sap-r	24,8635	0,0304947	16,3513	0,0292654	0,0489173	5,06763	0,087898	0,743626	0,6279808
CG12071-RA	CG12071	2,6823	3,26282	2,06603	4,87339	2,85179	2,18138	0,11775	0,575217	0,6279808
CG12071-RB	CG12071	3,7183	63,4186	71,7692	2,69583	2,2106	2,42788	0,120742	0,565259	0,6279808
CG12072-RA	wts	8,0033	8,00435	22,0935	8,75824	4,11523	32,6513	0,249621	0,276752	0,6279808
CG12073-RA	5-HT7	1,0829	77,5965	9,20545	0,0613219	0,0396402	13,9107	0,002041	0,994279	0,6279808
CG12075-RA	CG12075	24,0032	30,5712	22,0247	44,9189	5,00611	20,7318	-0,179291	0,489261	0,6279808
CG12075-RB	CG12075	0,018886	0,0172027	0,0181317	0,019672	8,73801	0,0200819	-0,179001	0,489912	0,6279808
CG12076-RA	Ythdc1	7,46302	0,0721082	4,86299	19,4012	10,9891	0,0173429	-0,369414	0,061811	0,6279808
CG12076-RB	Ythdc1	8,7409	1482,02	0,00471205	12,1656	0,0695901	0,584511	-0,369373	0,061165	0,13772387
CG12077-RA	PIG-C	1,34135	1,24835	73,8208	1,05509	42,1939	0,0382943	-0,307765	0,317156	0,6279808
CG12078-RA	CG12078	0	0,163019	0	0,137175	0	0	0,01588	0,899333	0,6279808
CG12079-RA	Dpse\GA11380	68,5041	61,8376	150,121	0,0237831	0,024885	0,0236971	-0,215904	0,500606	0,6279808
CG12081-RA	CG1208	4,75362	2,1199	0,0567413	1,0019	0,0568287	30,5524	0,076116	0,713164	0,6279808
CG12082-RA	CG1208	10,5469	0,0267416	9,06805	6,73938	4,68207	0,214831	-0,234001	0,378619	0,6279808
CG12084-RA	CG12081	12,4483	16,9205	18,1032	24,84	12,4892	49,4251	0,115576	0,637002	0,6279808
CG12085-RA	Usp5	4,63649	97,0966	6,99654	91,5404	4,82617	7,38789	-0,123741	0,509411	0,6279808
CG12085-RB	CG12084	10,3218	12,8361	95,2315	2,87438	202,698	122,766	-0,136127	0,465671	0,6279808
CG12085-RC	hfp	0,0137296	0,0136426	0,0205054	4,32026	0,0371333	0,022844	-0,127173	0,498517	0,6279808
CG12085-RD	hfp	29,109	0,0244693	0,0223316	0,0141292	6,63506	0,0249913	-0,147936	0,427469	0,6279808
CG12085-RE	hfp	0,0149775	0,024946	0,0238514	35,2515	4,44271	0,0267931	-0,180836	0,329384	0,6279808
CG12085-RF	hfp	0,0268637	0,0250916	0,684023	5,96765	1,17408	0,704728	-0,127106	0,498681	0,13772387
CG12085-RG	hfp	0,0273871	0,0194547	2,63764	0,0285181	0	0,0151343	-0,153031	0,411885	0,6279808
CG12085-RH	hfp	0,0275469	0,0211874	1,40467	0,0291103	0	4,28978	-0,176702	0,341799	0,6279808
CG12085-RI	hfp	0,0213584	0,0226293	0,435453	0,0292914	0	0,777439	-0,144421	0,43704	0,6279808
CG12086-RA	hfp	0,0232607	60,2023	67,537	0,0223777	0	10,4554	-0,016232	0,942057	0,6279808
CG1208-RB	hfp	0,0248437	0,533714	0,389448	0,0244812	1,76249	0,0176062	-0,402082	0,172913	0,6279808
CG1208-RC	cue	19,5186	23,4491	22,1949	8,68736	0,0172215	3,84966	-0,396694	0,179256	0,6279808
CG12090-RA	Ir62a	1,34579	1,83549	0,0131812	0,588229	0,0253948	0,0144236	-0,269125	0,249049	0,6279808
CG12090-RB	Ir62a	0,0143841	1,36916	36,43	1,39596	0,0248435	22,8612	-0,251654	0,283874	0,6279808
CG12090-RC	Ir62a	3,63374	3,40371	0,0143793	0,0148254	15,6607	3,84598	-0,266254	0,253605	0,6279808
CG12091-RA	CG12091	49,4093	0,0125058	0,0262932	1,70358	2,14318	0,0297169	-0,085513	0,652287	0,6279808
CG12092-RA	Npc1b	2,65883	0,124799	5,85506	2,25333	0,161705	0	-0,029364	0,9313	0,6279808
CG12093-RA	CG12093	13,9835	0,188475	25,1995	0,013888	3,51357	0,0141565	0,004287	0,985665	0,6279808
CG12096-RA	CG12096	7,6884	21,4673	10,5237	7,03892	4,57018	26,6492	0,331103	0,175993	0,6279808
CG12099-RA	CG12099	19,0552	11,4978	15,7227	23,0226	7,42456	10,237	-0,023373	0,907276	0,6279808
CG12099-RB	CG12099	12,5442	0,0406665	0,0173768	23,2858	15,9376	0,0192101	-0,025098	0,900588	0,6279808
CG12101-RA	Pdk1	9,88764	4,60189	11,2648	10,2939	7,87913	7,51634	-0,58325	0,005993	0,6279808
CG12101-RB	Pdk1	6,81177	11,7171	6,78937	16,432	10,3634	6,52353	-0,578221	0,006417	0,6279808
CG12104-RA	Pdk1	0,016721	16,0826	0,0160532	15,2288	0,0234715	0,0176895	0,259283	0,252309	0,6279808
CG12105-RA	Pdk1	0,0161805	9,91249	0,0155343	11,1951	0,022684	0,017096	-0,557146	0,082846	0,6279808
CG12106-RA	Pdk1	0,0167126	8,58116	0,016045	15,5492	0,0234591	0,0176802	0,298451	0,168996	0,6279808
CG12107-RA	Pdk1	0,0183459	15,8761	0,0176132	16,5405	0,0258509	0,0194828	-0,519318	0,077903	0,6279808
CG12108-RA	Pdk1	0,0192772	16,6715	0,0185073	20,7471	0,0272229	0,0205168	0,948958	0,001047	0,6279808
CG12109-RB	Pdk1	56,7805	0,0647863	38,4514	0,0906948	20,49	25,8781	0,337715	0,288995	0,6279808
CG1210-RA	Hsp60A	40,1569	1,2233	324,78	67,9387	8,20576	0,510104	0,375618	0,156463	0,6279808
CG1210-RB	Hsp60A	122,411	48,1587	0,0364858	260,397	15,0366	0	0,454876	0,086126	0,6279808
CG1210-RC	CG12104	16,0208	9,09483	20,1374	25,0615	10,7102	4,93275	0,395207	0,136808	0,6279808
CG1210-RD	CG12105	2,03148	0,868667	1,84199	1,56601	2,97918	2,32071	0,389634	0,142333	0,6279808
CG1210-RE	CG12106	5,1963	0,844416	19,6092	1,96656	17,2609	15,2832	0,454778	0,086339	0,6279808
CG1210-RF	CG12107	39,1027	33,0292	64,2104	57,1141	74,3524	89,6621	0,389255	0,142962	0,6279808
CG1210-RG	Ppt1	0,061087	0	21,1163	5,73524	21,3042	19,5967	0,45439	0,086437	0,6279808
CG1210-RH	Caf1-180	4,07673	5,93666	7,88597	8,04689	1,76588	3,12567	0,394105	0,137845	0,6279808
CG12110-RA	Pld	2,05365	1,69888	1,55144	0,0152262	2,46878	0,0155435	-0,006025	0,975962	0,6279808
CG12110-RB	Pld	0,0147601	0,0134445	0,0141705	0,0155533	0,020624	0,0158773	-0,017237	0,931058	0,6279808
CG12110-RC	Pld	0,0150663	0,0137234	0,0144646	0,0158564	0,021067	0,0161868	-0,008515	0,966089	0,6279808

gene_id	Symbol	W1_FPKM	W2_FPKM	W3_FPKM	MCM51_FPKM	MCM52_FPKM	MCM53_FPKM	FC	p-value	p-adj
CG12110-RD	Pld	0,0153498	0,0139817	0,0147367	11,4774	0,0214776	8,22139	-0,001966	0,99214	0,6279808
CG12110-RE	Pld	13,1447	11,8953	12,5451	0,0143382	8,93106	1,3174	-0,002294	0,99083	0,6279808
CG12110-RF	Pld	0,0139263	0,0126851	1,63115	0,961328	1,83577	0,435114	-0,005553	0,977814	0,6279808
CG12111-RA	Dsim\GD24567	0,424243	0,289823	0,712773	0,506432	0,0852832	0,386483	0,274021	0,423287	0,6279808
CG12112-RA	CG12112	8,65069	7,58382	12,0468	6,08759	0,816105	3,91518	-0,08799	0,742934	0,6279808
CG12113-RA	IntS4	3,7818	3,86439	4,78264	9,02803	0,205167	9,88484	-0,153104	0,494685	0,6279808
CG12114-RA	spn-F	9,97714	0,212523	0,2688	0	0	0	0,168385	0,436843	0,6279808
CG12115-RA	CG12115	0,194799	0,106462	1,38394	79,2708	2,15939	401,663	0,144732	0,644725	0,6279808
CG12116-RA	CG12116	12,6874	5,11489	0,0204569	0,0227329	10,8205	64,2789	-0,607914	0,074379	0,6279808
CG12117-RA	Sptr	1,85372	1,89038	233,215	1,47723	191,548	199,707	0,34294	0,26303	0,6279808
CG12118-RA	CG12118	14,1666	0,307702	0,0496116	1,41335	0,0790164	0,0595515	0,106567	0,640377	0,6279808
CG12118-RB	CG12118	0,0516755	5,66579	0,71093	4,60598	2,69713	0,758769	0,106567	0,640377	0,6279808
CG12119-RA	SmydA-9	1,51843	19,9879	4,35802	18,0687	1,00678	1,09502	-0,535466	0,131525	0,6279808
CG12120-RA	p130CAS	4,56683	0,0227577	5,69417	0,026404	0,0369183	0,0269542	-0,182445	0,441795	0,6279808
CG12121-RA	p130CAS	1,95202	0,0233492	0,0242125	0,0271324	0,0365254	0,0276978	0,355719	0,146762	0,6279808
CG12123-RA	p130CAS	11,8269	8,35839	0,0239746	8,74968	4,69575	4,69287	-0,110202	0,697123	0,6279808
CG12124-RA	p130CAS	0,0249846	19,8012	5,1339	15,443	1,67205	12,4281	0,093288	0,732315	0,6279808
CG12125-RA	p130CAS	0,025634	6,587	0,0294355	11,282	5,2831	5,84463	0,140713	0,590835	0,6279808
CG12125-RB	t	3,54635	14,5842	3,61349	39,2852	3,60097	8,60052	0,059422	0,821894	0,6279808
CG12126-RA	CG12121	13,4214	0,0397796	15,6808	0,050279	1,4783	2,98745	NA	NA	0,6279808
CG12127-RA	CG12123	5,0413	15,3043	7,18382	5,48334	0,818286	7,46652	-0,159063	0,499129	0,6279808
CG12128-RA	mxc	1,7531	1,72782	0,0191036	34,3186	0,0681032	0,0513267	0,063689	0,777988	0,6279808
CG12128-RB	Dsim\GD24570	10,0249	4,79413	11,1954	9,56101	7,25297	6,29979	0,17854	0,333738	0,6279808
CG12129-RA	Dsim\GD24570	0,0295685	0	0,0283876	8,9705	2,71855	2,7621	0,225687	0,420473	0,6279808
CG1212-RA	CG12126	0	0	0	0	0	0	0,241296	0,26115	0,6279808
CG1212-RB	amx	4,38045	5,13002	4,69074	6,12853	0,327456	0,319064	0,312379	0,142525	0,6279808
CG1212-RD	CG12128	0,0414529	0,0377581	10,2279	10,362	0,0449734	5,21277	0,265445	0,218442	0,6279808
CG1212-RE	CG12128	12,4127	12,6361	9,99959	15,8791	21,6162	9,65459	0,313797	0,140769	0,6279808
CG1212-RF	CG12129	5,96372	4,27843	9,855	6,43658	0,0322843	27,9697	0,312379	0,142525	0,6279808
CG12130-RA	CG1213	5,97468	2,4823	0,0607632	0,0394202	0,0429189	5,25468	0,021882	0,923853	0,6279808
CG12130-RB	CG1213	7,02047	8,31361	0,0845824	0,0783007	8,9524	0	0,036748	0,872805	0,6279808
CG12131-RA	CG1213	0,0293583	0,50662	0,0598647	0,120346	0,0533949	0,0734715	0,050235	0,843788	0,6279808
CG12132-RA	Dpse\GA11425	3,35414	9,74682	0,0168775	1,55587	0,0241054	0,0186354	-0,056937	0,842482	0,6279808
CG12133-RA	Dpse\GA11425	11,3969	3,17742	8,67717	9,85506	0,0526702	6,8265	0,050893	0,76304	0,6279808
CG12134-RA	eIF3j	69,3797	0,150843	11,9445	13,0226	3,69467	75,093	-0,569492	0,083266	0,13772387
CG12134-RB	c11.1	3,9562	5,93229	3,48393	0,390119	4,5237	10,5354	-0,571895	0,081727	0,6279808
CG12135-RA	CG12133	0,091265	0,081812	53,912	0	2,40443	3,42432	-0,247207	0,275116	0,6279808
CG1213-RA	Dvir\GJ21267	2,69758	4,68083	96,6701	12,54	67,123	3,23389	-1,095828	0,001351	0,6279808
CG1213-RB	Dvir\GJ21267	6,98295	57,514	3,69052	6,19205	4,48936	9,80209	-1,096369	0,001348	0,6279808
CG1213-RC	c12.1	23,3194	4,84558	0,158723	9,7605	7,63722	7,92419	-1,095514	0,001359	0,6279808
CG12140-RA	ru	1,31859	2,61898	1,74064	0,0202276	50,9665	1,73399	-0,16377	0,553348	0,6279808
CG12141-RA	Dpse\GA11432	14,7665	3032,42	0,0862301	10,5448	6,39151	2,04359	-0,231411	0,306745	0,6279808
CG12141-RB	LysRS	3,33807	5,88582	4,92432	8,70413	39,7217	4,09554	-0,231373	0,306127	0,6279808
CG12142-RA	LysRS	21,1968	0,0307126	7,5263	6,35897	3,51023	0,0371521	-0,469983	0,13599	0,6279808
CG12143-RA	Tsp42Eg	88,2216	2,8738	103,718	0,10705	8,90273	7,13821	-0,566724	0,03957	0,6279808
CG12147-RA	Tsp42Ej	20,2146	0,124437	5,97167	33,9727	49,7637	0,201078	-0,013096	0,914398	0,6279808
CG12149-RA	CG12147	0	0	0,151473	0,0586044	0,016992	0	-0,44096	0,055822	0,13772387
CG1214-RA	c12.2	6,49484	23,2582	535,23	35,6869	0,0281413	29,3435	0,005943	0,984859	0,6279808
CG12151-RA	Pdp	14,5614	27,1214	0,0255828	11,8901	0,0142107	225,508	-0,169487	0,378541	0,6279808
CG12153-RA	Hira	2,34388	1,98436	3,67885	3,32201	1,59991	2,12476	0,080004	0,76721	0,6279808
CG12154-RC	oc	7,00845	9,24401	5,86261	7,89047	47,8802	3,60173	0,429532	0,131362	0,6279808
CG12155-RA	CG12155	5,76071	7,44483	0,0357256	8,62409	0,0103497	8,18501	0,08197	0,715211	0,6279808
CG12156-RA	Rab39	26,9536	0	29,7713	0,00764092	0,0548721	8,04563	0,1303	0,489164	0,6279808
CG12157-RA	Tom40	0,0361333	0,0494142	0,0959709	0,162282	0,571867	3,1429	-0,026922	0,89157	0,6279808
CG12157-RB	Tom40	55,2815	0	0,0346902	0,0410848	0,0824096	0,100044	-0,026721	0,892329	0,6279808
CG12158-RA	CG12158	0,820091	14,5246	0,787336	2,35384	3,8579	3,98528	0,057515	0,854112	0,6279808
CG12159-RA	CG12159	2,04746	0,039235	6,22375	0,0206071	1,71557	379,632	0,547323	0,064028	0,13772387
CG12159-RB	CG12159	2,12791	0,0406665	0,0428627	0,017782	7,50363	0,0525292	0,52569	0,05242	0,6279808
CG12161-RA	mri	6,67949	0,0247308	9,97331	0,0288427	10,9979	6,85597	-0,095805	0,779484	0,6279808
CG12162-RA	mri	8,78881	2,24676	11,8321	3,8372	17,4303	13,6545	-0,100998	0,648125	0,6279808
CG12162-RB	mri	12,2764	8,76111	16,6045	7,28935	0,12526	17,9298	-0,086559	0,698265	0,6279808
CG12163-RA	Prosbeta2R2	0,292371	0,121051	0,122559	0,345093	7,8103	0,258643	-0,008724	0,968813	0,6279808
CG12163-RB	POLDIP2	7,63911	8,73229	0,0335467	12,7203	9,67555	0,0398365	0,00261	0,990804	0,6279808
CG12164-RA	POLDIP2	7,63065	4,38269	20,3537	6,80692	5,79785	0,0323307	0,492173	0,027151	0,6279808
CG12165-RA	CG33293	52,8028	69,6669	70,6573	98,389	30,4146	52,5362	-0,021216	0,943421	0,6279808
CG12169-RA	CG33293	25,0472	29,5952	24,4836	34,5403	0,0567926	20,2698	-0,148916	0,670839	0,6279808
CG1216-RA	CG12164	187,128	177,11	189,742	34,2858	90,7618	64,2262	-0,246813	0,223274	0,6279808
CG1216-RB	Incenp	4,94808	3,79477	12,1197	6,79298	1,94281	2,34117	-0,246813	0,223274	0,6279808
CG1216-RC	Ppm1	0,348502	0,126976	0,289971	0,546011	7,77807	0,178269	-0,171508	0,409619	0,6279808
CG12170-RA	CG12170	12,8106	0,0635771	20,0667	0,0826206	29,5201	28,1667	-0,699952	0,014308	0,6279808
CG12171-RA	CG12171	23,3897	35,0876	0,0670106	19,414	85,0667	64,7594	-0,640079	0,060935	0,6279808
CG12172-RA	Spn43Aa	44,658	45,5368	55,4248	0,0160966	41,3696	3,2927	-0,663388	0,018232	0,6279808
CG12173-RA	CG12173	14,2398	0,0100316	27,071	18,6245	15,7443	0,428843	-0,26766	0,370982	0,6279808
CG12175-RB	tth	20,045	22,0534	17,9888	10,2473	14,8908	10,4521	-0,041339	0,83815	0,6279808
CG12176-RA	DNAlig4	1,50913	2,85648	9,05863	5,5431	2,61556	4,37466	-0,002167	0,993166	0,6279808
CG12177-RA	CG12177	28,5852	29,7094	34,2165	6,65356	0,32699	35,521	-0,161662	0,561287	0,6279808
CG12178-RA	Nhe1	10,5874	8,73282	6,77612	17,7031	3,34505	7,59792	0,198116	0,329027	0,6279808
CG12179-RA	CG12179	0,499438	0,721413	5,51305	1,37838	2,2195	2,08502	-0,014673	0,963627	0,6279808
CG12179-RB	CG12179	0,189346	4,57966	1,37769	0,0178591	2,63313	0,574985	0,034016	0,915807	0,6279808
CG12181-RB	CG1218	4,06784	3,8718	1,90501	3,82527	16,5516	3,16282	-0,237934	0,464285	0,6279808
CG12182-RA	Sgs4	0,171626	0,0658495	0,164771	0,159224	0	0,634823	0,149441	0,584236	0,6279808
CG12184-RA	CG12182	1,01181	0,887744	1,30182	1,05105	0,661857	0,760775	0,416015	0,242209	0,6279808
CG12186-RA	CG12184	0,351254	0,319946	0,693719	0,543744	0,903751	0,349348	-0,313294	0,225801	0,6279808
CG12187-RA	ProRS-m	3,42175	0,0246195	2,61654	0,594219	2,86536	1,95705	0,366542	0,149991	0,6279808
CG12189-RA	Fife	3,95843	0,0119283	0	1,64165	2,8609	0	-0,268263	0,286888	0,6279808
CG1218-RA	Rev1	1,41301	34,6133	38,0493	2,36816	31,221	37,2558	-0,008859	0,975808	0,6279808
CG12190-RA	RYBP	14,4432	1570,5	2201,35	4201,01	5,37124	10,2199	0,246849	0,312196	0,6279808
CG12191-RA	dpr20	2,92048	0,0496314	0,0523117	34,8527	0,08392	1,8308	-0,02455	0,922808	0,6279808
CG12192-RA	Klp59D	0	0	4315,09	0	0	0	-0,041811	0,731452	0,6279808
CG12193-RA	Or22a	0,229785	0,29955	0,220607	2,1472	4,85277	0	0,37308	0,279647	0,6279808
CG12194-RA	CG12194	8,47306	13,3712	26,5985	22,4415	11,422	16,8573	-0,431024	0,086508	0,6279808
CG12196-RA	egg	2,97074	2,29434	5,2623	10,9013	2,43068	4,9759	-0,05628	0,840445	0,6279808
CG12199-RA	kek5	3,17827	4,69235	90,1556	9,88595	1,51257	1,43655	-0,008419	0,978289	0,6279808
CG12199-RB	kek5	4,03938	5,20256	3,17906	4,3641	2,39996	3,88777	-0,008231	0,978801	0,6279808
CG12200-RA	Kaz1-ORFB	0,0581654	0,0152307	10,2291	0,0173284	0,0956065	0,0720549	0,914305	0,00679	0,6279808
CG12201-RB	Kaz1-ORFB	0,0578092	0,0147383	0,0178695	0,0167471	0,094895	0,0715186	0,326636	0,300997	0,6279808
CG12201-RC	Kaz1-ORFB	0,0726109	0,0152229	0,0177148	0,0173193	0,126146	0,095071	0,326636	0,300997	0,6279808
CG12202-RA	Kaz1-ORFB	0,103405	0,0167107	0,0327331	0,0190851	0,204459	0,154093	-0,2364	0,197403	0,6279808
CG12203-RA	Kaz1-ORFB	0,119056	0,017559	0,0558422	0,020098	0,253179	0,190811	-0,265537	0,378739	0,6279808
CG12204-RA	narya	0,462816	0,583705	1,81073	0,430129	0	232,152	-0,213248	0,469378	0,6279808
CG12205-RA	GC2	0	2,59494	13,3864	16,414	21,2008	3,95234	-0,041811	0,731452	0,6279808
CG12206-RA	GC2	0	9,32828	0,0344079	2,99385	1,61744	3,9298	0,19582	0,397979	0,6279808
CG12206-RB	Naa15-16	17,0347	18,2955	19,8034	6,75124	3,26122	3,73716	0,188889	0,412704	0,6279808
CG12206-RC	Dpse\GA11474	71,7469	75,0961	145,847	98,9927	142,321	152,523	0,201968	0,384947	0,6279808

gene_id	Symbol	W1_FPKM	W2_FPKM	W3_FPKM	MCM51_FPKM	MCM52_FPKM	MCM53_FPKM	FC	p-value	p-adj
CG12207-RA	CG12204	0,0624141	0,0568511	0,0599213	0,0724466	0,0981294	0,0739562	0,171343	0,411239	0,6279808
CG12207-RB	Bsg25A	0	8,73845	0	0,12668	4,43136	0,725411	0,16252	0,429986	0,6279808
CG12207-RC	CG12206	4,88082	0,0255171	6,62105	0,801337	0,0403937	77,3518	0,164033	0,425852	0,6279808
CG12209-RA	CG12206	5,70219	1,97052	3,45819	6,80784	1,32352	3,36906	-0,168953	0,612807	0,6279808
CG1220-RA	CG12206	0,0280141	0,0145144	0,0268952	5,86873	0,0223265	3,73371	0,125967	0,715041	0,6279808
CG1220-RC	red	5,71923	3,72344	44,9436	1,38629	1,86722	5,53992	0,100209	0,771027	0,6279808
CG1220-RE	red	37,5757	1,81313	9,455	1,22041	1,96066	30,7143	0,109807	0,747994	0,6279808
CG1220-RF	red	8,66654	0,0171683	3,87975	0,00475837	0,0265902	6,44868	0,104379	0,76013	0,6279808
CG1220-RH	CG12209	0,0587514	0,0535149	0,0564049	0,0675451	0,0757646	0,0689526	0,128114	0,710499	0,6279808
CG12210-RA	miple1	0,0359368	13,2244	19,2173	0,0390002	54,6573	93,6643	-0,187397	0,410152	0,6279808
CG12210-RB	miple1	95,8004	86,3692	77,6598	88,4062	2,3458	2,01235	-0,186679	0,406871	0,6279808
CG12210-RC	Syb	74,0845	64,817	87,0917	92,8731	105,693	122,063	-0,187145	0,410769	0,6279808
CG12210-RD	Syb	19,1002	26,2542	26,1962	25,4648	31,3816	35,0369	-0,186914	0,40629	0,6279808
CG12212-RA	Syb	52,9632	47,079	61,4451	61,6137	48,0606	50,4626	0,152559	0,583596	0,6279808
CG12212-RB	Syb	17,9649	22,9539	23,6931	22,8356	19,3179	19,8146	0,152559	0,583596	0,6279808
CG12213-RA	peb	4,51361	5,68536	4,78896	6,88931	2,43245	0,0182313	0,067041	0,778471	0,6279808
CG12213-RB	peb	4,0422	4,23678	5,01573	7,45401	1,86265	3,41052	0,137286	0,545031	0,6279808
CG12213-RC	CG12213	1,50724	5,36344	5,62404	3,71443	3,93627	0,0396954	0,128774	0,571416	0,6279808
CG12214-RA	CG12213	10,7568	0,907619	1,03171	13,4489	2,0496	1,70052	0,291417	0,198848	0,6279808
CG12214-RB	CG12213	0,0358394	0,376479	3,07917	0,0388851	40,1296	39,2935	0,291626	0,198541	0,6279808
CG12217-RA	mlt	48,9481	71,4263	54,9387	49,298	1,7131	42,6363	-0,072643	0,700861	0,6279808
CG12218-RA	mlt	0,0268856	0,0244893	0,0258118	0,0285428	3,17156	0,0291376	0,212462	0,385173	0,6279808
CG12219-RA	PpV	15,1724	0,154061	18,5731	0	0,255986	0	0,032038	0,885584	0,6279808
CG1221-RA	mei-P26	3,38996	131,98	16,1657	0,556258	2,56727	2,19506	0,237579	0,241583	0,6279808
CG1221-RB	CG12219	5,23732	29,6182	37,6126	29,055	0,0917725	0,0146402	0,23759	0,241588	0,6279808
CG12220-RA	mRpL32	31,6654	5,71252	15,3694	0,195516	0,264828	0,19959	-0,359004	0,246503	0,6279808
CG12223-RA	Dsp1	17,3546	12,4061	17,9059	14,6039	0,0398882	12,9852	0,027074	0,903833	0,6279808
CG12223-RB	Dsp1	0,0285836	0,0252178	0,0265796	0,0294485	0,0386949	7,8352	0,028801	0,897822	0,6279808
CG12223-RC	Dsp1	0,0276854	0,0245092	0,0258328	0,0285676	0,0323545	0,0300622	0,01962	0,929545	0,6279808
CG12223-RD	Dsp1	0,0269075	0,0206992	0,0218171	0,0238866	13,6292	0,0291628	0,01962	0,929545	0,6279808
CG12223-RE	Dsp1	0,0227247	22,0928	6,87262	26,3605	1,83953	0,0243843	0,025741	0,908552	0,6279808
CG12223-RF	Dsp1	31,3404	61,6492	73,8794	81,9769	72,8577	15,1256	0,027032	0,904377	0,6279808
CG12224-RA	CG12224	1,2144	3,49988	0,371095	0,916781	0,0526702	7,24144	-0,781298	0,018036	0,6279808
CG12225-RA	Spt6	13,2882	12,9209	1373,23	919,4	1102,84	1286,25	-0,147414	0,535691	0,6279808
CG12227-RA	SkpF	0,102655	0	537,867	6,11856	523,47	0,096506	-0,013096	0,914398	0,6279808
CG12229-RA	CG12229	0,0346551	0,0315663	0,033271	5,90766	2,27233	1137,59	-0,062055	0,850948	0,6279808
CG12230-RA	car	0,0211398	0,0205648	0,0216754	0,0207173	0,0327223	0,0246615	-0,08214	0,742317	0,6279808
CG12230-RB	car	5,65399	0,0209223	0,0220522	0,0236381	0,0280617	0,021149	-0,08214	0,742317	0,6279808
CG12231-RA	CG12231	0	0	0	14,5566	0	0	0,044335	0,715924	0,6279808
CG12232-RA	Galphaf	7,83393	8,30273	24,6396	267,281	1,59708	287,814	0,094158	0,697329	0,6279808
CG12233-RA	Idh3a	64,1024	17,4427	19,6464	0,0237232	0,0320986	0,0241914	-0,268234	0,209696	0,6279808
CG12233-RB	Idh3a	0,0484545	0,0205438	0,0216532	0,0241581	0,0321332	0,0242175	-0,269308	0,207912	0,6279808
CG12234-RA	Dsim\GD24453	2,52011	3,1024	3,55288	0,0642519	4,01486	2,09915	0,090205	0,706402	0,6279808
CG12235-RA	Arp10	9,21498	2,14467	2,88845	262,694	5,71567	2,00338	-0,619708	0,024	0,6279808
CG12236-RA	CG12236	0,0215441	5,24939	0,0206837	6,22713	0,0311573	0,023482	-0,079627	0,716544	0,6279808
CG12236-RB	CG12236	0,0281774	0,0256659	0,0270519	0,0307543	0,0416569	0,0313951	0,092332	0,751396	0,13772387
CG12237-RA	CG12237	9,02288	270,498	12,5777	9,8901	88,0126	14,8162	-0,508457	0,039737	0,6279808
CG12238-RA	e(y)3	4,54284	5,19627	5,27116	14,0052	15,9381	4,32501	-0,320762	0,207486	0,6279808
CG12239-RA	CG12239	7,02815	7,16016	9,11853	0	8,70423	0,139839	-0,23429	0,302715	0,6279808
CG12240-RA	DnaJ-60	5,46875	6,64175	0,0564551	29,8385	46,5463	0,0690234	-0,168545	0,519501	0,13772387
CG12240-RB	DnaJ-60	10,624	15,8497	14,8168	15,4712	9,06108	21,7034	-0,020481	0,927982	0,6279808
CG12241-RA	CG12241	9,5953	29,6667	24,4884	21,9428	0,176552	58,5859	0,052525	0,809954	0,6279808
CG12242-RA	GstD5	10,7082	13,6367	51,2561	0,025475	2,89919	1,72246	0,246034	0,469471	0,6279808
CG12244-RA	lic	10,2785	8,87599	17,8456	0,444065	8,56638	9,28461	0,165999	0,514536	0,6279808
CG12245-RA	gcm	3,50872	0,328039	0,272964	0,952719	0,0169758	0,012794	0,054852	0,864573	0,6279808
CG12249-RA	mira	6,52942	0	0,0679195	0,0175921	0	0	-0,60691	0,012888	0,6279808
CG12249-RB	mira	3,86643	15,1986	12,2103	0,0176109	3,67018	0	-0,607648	0,012604	0,6279808
CG12250-RA	ymp	0,108439	0,0165136	0,0175062	0,208226	0,0534269	0,697876	-0,290078	0,417557	0,6279808
CG12250-RB	ymp	0,502689	3,82917	0,0174054	0,817752	0,053781	0,797349	-0,250848	0,483305	0,6279808
CG12251-RA	AQP	2,83259	12,0147	23,6352	0,0743262	27,4216	3,11292	0,127615	0,704068	0,6279808
CG12251-RB	AQP	0,052664	0,0175898	0,0176852	1,40909	1,95698	0,0608444	0,134502	0,688479	0,6279808
CG12252-RA	Fcp1	2,45535	2,61247	0,0562548	71,4388	5,4474	0,0726777	0,227557	0,359742	0,6279808
CG12253-RA	CG12253	10,699	8,79046	16,7147	11,5617	12,5457	11,4188	-0,125212	0,619645	0,6279808
CG12254-RA	MED25	3,29286	4,85843	0,0177049	0,0194916	23,4951	0,0198977	0,139277	0,557078	0,6279808
CG12255-RA	Cpr72Eb	0	0,0670476	0,290994	4,65576	1,78825	1,34773	0,000513	0,997309	0,6279808
CG12256-RA	CG12256	6,96094	0,187147	0,197254	33,4373	20,0811	0	-0,897809	0,001576	0,6279808
CG12259-RA	CG12259	9,92202	0,0254847	2,84	0,0297813	0,0403389	0,0304018	-0,106391	0,684521	0,6279808
CG12261-RA	mRpS22	47,7324	44,017	68,2656	10,5136	10,6939	11,655	-0,703671	0,012856	0,6279808
CG12262-RA	Mcad	57,1559	1,10311	1,04295	1,58738	0,0199509	0,0150362	-0,257797	0,387097	0,6279808
CG12263-RA	PIG-O	6,19289	4,70915	9,11296	10,451	6,08178	6,97287	-0,323973	0,19056	0,6279808
CG12264-RA	Nfs1	21,7667	18,9176	36,6841	25,9303	43,2968	74,9307	0,034605	0,888754	0,6279808
CG12265-RA	Det	4,30262	2,21326	3,31105	3,12494	0,0263158	8,78549	-0,303306	0,330997	0,6279808
CG12267-RA	CG12267	3,67535	3,18312	6,20871	24,7934	3,88407	14,6618	0,080037	0,769342	0,6279808
CG12268-RA	CG12268	9,58851	2,71051	11,1662	9,05215	18,7351	2,45257	-0,004626	0,985609	0,6279808
CG12268-RB	CG12268	2,55964	7,32755	4,21269	2,42199	7,9205	7,81011	-0,00333	0,989628	0,6279808
CG12269-RA	euc	1,75086	0,416036	1,16934	2,34184	18,1676	6,8398	-0,221701	0,533577	0,6279808
CG12272-RA	CG1227	8,01415	0,0113331	6,10179	0,206658	23,3232	5,30847	0,082685	0,7025	0,6279808
CG12273-RA	Strumpellin	2,72213	2,2049	4,59691	7,79129	0,0538784	2,64226	-0,065263	0,788467	0,6279808
CG12275-RA	angel	6,73537	0,104189	0,109815	0,0457098	120,727	0,0466623	-0,322988	0,349229	0,6279808
CG12276-RA	RpS10a	1,80094	1,69699	3,10029	3,01594	4,42021	3,93795	-0,361962	0,209261	0,6279808
CG12278-RA	Aos1	9,80913	0,141875	18,0919	15,1028	32,2814	16,1751	-0,48404	0,117617	0,6279808
CG12279-RA	CG12278	0	23,7619	0	0,347201	5,72949	0	-0,525982	0,050996	0,6279808
CG1227-RA	CG12279	12,6451	11,518	16,4758	1,0199	0,0149311	232,332	3,178287	1,23E-28	0,6279808
CG12283-RA	Ptpmeg	0,0172627	0,0157241	0,0165732	19,6355	9,82053	14,1095	0,287751	0,260749	0,6279808
CG12284-RA	Ptpmeg	14,0925	19,9064	15,7048	12,4603	5,61501	7,614	-0,119696	0,551414	0,6279808
CG12284-RB	Ptpmeg	7,09787	7,10933	7,92606	0,0189153	0,0256208	0,0193094	-0,076006	0,706076	0,6279808
CG12284-RC	Ptpmeg	0,0181894	0,0165681	0,0174629	20,2188	15,5226	16,0209	-0,079445	0,692986	0,6279808
CG12286-RA	Dsim\GD22095	8,59262	0,0254023	0	0	5,02996	0	0,135133	0,669799	0,6279808
CG12287-RA	Diap1	31,0058	8,4716	7,48678	3,60968	2,21961	6,42886	0,127799	0,606836	0,6279808
CG12287-RB	Diap1	88,5654	2,97773	3,73088	3,20512	2,25463	3,61657	0,088903	0,709784	0,6279808
CG12288-RA	Diap1	29,3481	43,4194	3,4768	9,75329	7,10508	2,49335	-0,034149	0,916191	0,6279808
CG12289-RA	kar	16,6174	8,7378	14,0207	35,4738	5,51094	18,2031	-0,041811	0,731452	0,6279808
CG12289-RB	pdm2	1,37671	1,43916	1,38382	1,95215	3,53469	1,22543	-0,041811	0,731452	0,6279808
CG1228-RA	pdm2	5,24766	6,59545	6,47324	8,46119	0,899957	4,02787	-0,082836	0,715003	0,6279808
CG1228-RB	CG12288	12,2195	0,0447816	124,579	1,10944	10,2045	16,2643	0,005215	0,981997	0,6279808
CG1228-RC	CG12289	0	110,681	0	21,0082	0,0779128	0,0223432	-0,070525	0,757324	0,6279808
CG1228-RD	CG12289	0	0,0590064	0,0489985	0	0,162094	95,966	-0,068645	0,764147	0,6279808
CG12290-RA	CG12290	5,56855	7,7117	6,26789	9,80125	6,83212	5,16129	-0,23786	0,298261	0,6279808
CG12292-RA	spict	14,0914	0,242297	0,766145	0,057471	0,0778448	5,42572	-0,332969	0,154906	0,6279808
CG12295-RB	stj	6,19753	0,0334803	6,18362	7,33535	4,82646	46,193	0,16577	0,463355	0,6279808
CG12296-RA	klu	5,78356	0,0294502	5,76549	0,0347801	0	0,0355049	-0,024078	0,930118	0,6279808
CG12297-RA	Fadd	2,05205	1,68223	3,06771	3,33331	12,7188	2,67362	-0,32115	0,270996	0,6279808
CG12298-RA	sub	1,91903	2,47631	4,87842	0,0471827	8,56021	7,74792	-0,314488	0,310879	0,6279808
CG12299-RA	CG12299	6,01963	4,04869	7,2768	5,09231	5,03045	4,36445	0,310046	0,155824	0,6279808

gene_id	Symbol	W1_FPKM	W2_FPKM	W3_FPKM	MCM51_FPKM	MCM52_FPKM	MCM53_FPKM	FC	p-value	p-adj
CG12301-RA	CG12301	8,20271	4,13736	14,288	8,54799	0,303904	6,5379	0,161224	0,604957	0,6279808
CG12303-RA	Ir67b	0,0431869	0,104855	0,0199194	0,316011	14,8992	0	0,24966	0,375215	0,6279808
CG12304-RA	AIMP2	5,67491	5,80487	9,99332	25,7569	1,99756	7,31053	-0,505551	0,080724	0,13772387
CG12304-RB	AIMP2	20,0357	14,4302	29,716	11,9044	0,0328911	37,5263	-0,503077	0,082079	0,13772387
CG12306-RA	polo	3,35712	0,0260247	31,8067	3,78452	6,1109	5,74004	0,007111	0,982953	0,6279808
CG12306-RB	polo	3,77112	5,47964	0,0342593	7,68881	2,17975	4,31326	9,75E-06	0,999977	0,6279808
CG12307-RA	CG12307	0	7,41529	0,0880249	0,106446	0	0	0,01562	0,897983	0,6279808
CG12309-RA	CAH13	0	5,5838	0,17367	5,92329	0,0188431	3,97439	-0,308666	0,344854	0,6279808
CG12310-RA	CG1231	0,425267	0,0194171	0,0185289	14,3897	0,0267176	0,0227976	-0,4396	0,216765	0,6279808
CG12311-RA	CG12310	61,0209	0	5,71793	0,154488	3,90311	9,02504	0,027464	0,932885	0,6279808
CG12313-RA	tw	11,9523	0,100144	0,0407778	22,8752	1129,86	0,164654	0,240329	0,500941	0,6279808
CG12314-RA	ttm2	0,352875	0,38169	0,317607	0,580252	0,326276	0,207734	-0,480071	0,141657	0,6279808
CG12316-RA	zuc	0,0615422	0,00853507	1,89637	7,13466	3,1626	12,1634	0,334159	0,245102	0,6279808
CG12316-RB	CG12316	1,52049	13,2511	2,87852	30,6923	2,87284	22,8987	0,322418	0,264106	0,6279808
CG12317-RA	CG12316	2,10567	0,0330937	1,52361	0,0285676	0	0,0402898	-0,192503	0,372576	0,6279808
CG12317-RB	Jhl-21	2,22438	0,0345568	0,036423	0,0426616	0,0577854	0,0435505	-0,194217	0,367904	0,6279808
CG1231-RA	Jhl-21	6,93971	0,0355362	0,0374553	2000	2466,98	1882,4	0,428205	0,229487	0,6279808
CG12320-RA	tipE	5,20388	4,46674	5,21819	1,11064	0,0621298	3,28648	-0,030148	0,912463	0,13772387
CG12321-RA	tipE	1,04904	2,12175	0,518044	46,8654	4,23919	0,320272	0,140883	0,620949	0,6279808
CG12323-RA	CG12320	2,97369	2,7342	1,28658	3,04661	0,547778	1,00684	-0,691713	0,031619	0,6279808
CG12323-RB	CG12321	8,4153	0,0374527	17,1732	10,7387	0,0635453	0,0496594	-0,691713	0,031619	0,6279808
CG12324-RA	Prosbeta5	31,0446	22,9755	48,7379	33,934	65,1339	68,3921	-0,181331	0,561764	0,6279808
CG12325-RA	Prosbeta5	0,060918	0,0554884	8,97744	8,58943	17,0323	9,61671	0,001407	0,996412	0,6279808
CG12327-RA	RpS15Ab	212,875	205,181	0,01011	0,0682447	0,0294603	0,0222031	0,132801	0,384685	0,6279808
CG1232-RA	CG12325	4,73364	2,44617	9,81146	6,3468	4,42379	4,51151	0,062022	0,791046	0,6279808
CG1232-RB	Best3	0,10273	303,515	66,5255	1,92124	0	3,00141	0,068421	0,769277	0,6279808
CG12330-RA	CG1233	0,0164983	0,0150278	0,718695	0,683829	0	0,0176895	-0,096867	0,773685	0,6279808
CG12333-RA	CG1233	5,31276	5,35691	0,0158394	7,53612	0	4,64831	0,10735	0,631663	0,6279808
CG12334-RA	Cpr65Az	0,642404	0,839511	139,604	148,49	70,018	0,887524	-0,013096	0,914398	0,6279808
CG12338-RA	CG12333	4,92217	9,92415	9,59084	10,2718	1,09092	0,0452193	0,136511	0,62362	0,6279808
CG1233-RA	Atg8b	0	0	0	0	0	0	-0,114945	0,571663	0,6279808
CG1233-RB	CG12338	8,01721	9,65659	16,0988	0,0144104	0,0173352	17,777	-0,114945	0,571663	0,6279808
CG12340-RA	CG1234	9,15206	24,5659	18,0196	0	0	0	0,235771	0,315291	0,6279808
CG12341-RA	wde	7,92525	0,848038	1,02153	0,0145955	0,0201354	0,209138	-0,102574	0,655313	0,6279808
CG12342-RA	CG12341	11,185	30,0021	16,4366	0,0363135	2,28531	269,313	0,197982	0,446449	0,6279808
CG12342-RB	dgo	0,0189564	2,04581	3,33878	1,12582	10,3558	0,620833	0,186878	0,473331	0,6279808
CG12343-RA	dgo	5,47471	0,0172668	0,0181993	6,90109	14,0618	3,58018	-0,072639	0,762792	0,13772387
CG12344-RA	CG12343	40,6135	42,804	2,34122	9,61355	0,025511	0,0192266	0,014495	0,96387	0,6279808
CG12345-RA	CG12344	1,05365	0,593094	0,0227318	0,0249446	1,35187	0,846274	0,513896	0,010333	0,6279808
CG12345-RB	VAChT	9,90886	8,7964	10,4717	6,66624	0,0342676	0,0258261	0,340999	0,09525	0,6279808
CG12346-RA	VAChT	0,0239956	0,0218568	0,0230372	0,025299	1,11152	1,037	0,22611	0,310242	0,6279808
CG12347-RA	cag	7,37365	5,90752	0	7,17431	0,027739	0,0209058	0,01562	0,897983	0,6279808
CG12348-RA	CG12347	0	0,126442	0	0	0	0	0,219266	0,375593	0,6279808
CG12348-RB	Sh	4,6342	4,18197	3,58199	2,07503	1,61411	0,0423442	0,53152	0,008927	0,6279808
CG12348-RC	Sh	0,0231061	0,0210467	0,0221833	0,0243096	0,0329275	0,0570272	0,282944	0,197827	0,6279808
CG12348-RD	Sh	0,0276044	0,025144	1,64312	2,95089	1,10302	0	0,385479	0,064588	0,6279808
CG12348-RE	Sh	0,0240218	0,032821	0,0230623	0,0253281	0,0343071	0,425575	0,255303	0,251403	0,6279808
CG12348-RF	Sh	2,34728	2,08746	2,9736	2,40977	2,09671	6,44841	0,220293	0,374166	0,6279808
CG12348-RG	Sh	0,0380256	0,0346363	0,0365068	0,0414799	0,0561848	99,2044	0,388239	0,061941	0,6279808
CG1234-RA	Sh	0,0497309	1,99313	0,0477446	0,0558631	1,69974	0,321076	-0,096177	0,743259	0,6279808
CG12350-RA	lambdaTry	32,7246	0,827208	1,33493	0,0140209	0,16512	61,9755	-0,411012	0,217262	0,6279808
CG12351-RA	deltaTry	6,35026	1,28539	19,2436	17,4838	0,751767	17,59	-0,242096	0,426192	0,6279808
CG12352-RA	san	23,4553	16,1057	6,49571	25,0026	0,342525	190,959	0,083866	0,789639	0,6279808
CG12355-RA	CG12355	0,123122	13,46	22,4604	14,092	14,1596	73,6122	0,200368	0,436212	0,13772387
CG12355-RB	CG12355	20,6277	0,132862	0,350722	53,0932	6,50665	199,223	0,152708	0,541364	0,6279808
CG12357-RA	Cbp20	25,4138	22,0415	5,57028	34,9104	0,225654	1,54975	-0,034985	0,90241	0,6279808
CG12358-RA	Paip2	50,3791	81,5377	50,5772	9,7646	60,7373	51,797	-0,016194	0,948809	0,6279808
CG12359-RA	Ulp1	8,50099	9,09145	17,4612	0,0224158	11,3403	4,84104	0,21127	0,438915	0,6279808
CG12360-RA	CG1236	18,4085	2,76823	3,43787	4,03606	4,99075	1,70615	-0,03444	0,875798	0,6279808
CG12360-RB	CG46280	7,36868	7,09986	7,86747	0,244895	0,080586	4,24566	-0,016512	0,939759	0,6279808
CG12360-RC	CG46280	12,5784	0,0478937	12,9857	1,0878	1,87239	9,24576	-0,428478	0,163058	0,6279808
CG12362-RA	CG46280	0,0525802	0,660139	0,0504801	12,2974	3,01116	0,0607345	-0,041811	0,731452	0,6279808
CG12362-RB	CG12362	0	0,0379805	56,3243	0,308208	0	14,5287	-0,041811	0,731452	0,6279808
CG12363-RA	CG12362	0	28,9317	43,6773	0,375608	1,46499	0,0464368	0,11933	0,579762	0,6279808
CG12366-RA	Dlc90F	83,3834	10,5588	103,444	0,0517398	1,17902	0,888582	-0,033911	0,89335	0,6279808
CG12367-RA	O-fut1	6,49845	1,96321	3,38689	6,61273	135,674	0,498036	0,48971	0,074186	0,6279808
CG12369-RA	Hen1	3,49664	4,57479	70,202	16,2567	0,0234591	4,85973	0,253357	0,262952	0,6279808
CG12369-RB	Lac	0,0367156	0,600299	0,0352492	0,914624	0,0540743	0,0214936	0,279831	0,215793	0,6279808
CG1236-RA	Lac	90,3116	14,1011	91,8796	0,462085	48,6064	0,0203307	0,253561	0,297823	0,6279808
CG12370-RA	Dh44-R2	0,0317215	3,97805	0,476403	4,7102	22,9549	1,74256	-0,450308	0,087197	0,6279808
CG12370-RB	Dh44-R2	4,52171	0,171471	0,0304545	0,441416	2,7023	0,0281499	-0,065798	0,75529	0,6279808
CG12372-RA	spt4	40,7553	32,3098	37,3753	34,2692	0,0255183	0,0192321	0,162017	0,489317	0,6279808
CG12373-RA	mRpL18	33,4216	30,7145	55,4151	29,0586	33,8373	38,3958	-0,167919	0,555172	0,6279808
CG12374-RA	CG12374	0,293169	0,390288	0,346413	29,1166	17,562	23,7307	0,271132	0,422748	0,13772387
CG12375-RA	CG12375	10,0581	10,9717	18,0507	11,9969	0,201687	15,0213	-0,071279	0,815433	0,13772387
CG12376-RA	CG12376	0	13,4229	25,0371	0	0	0	0,01562	0,897983	0,6279808
CG12377-RA	CG43329	0	5,59949	2,22294	16,2524	12,7362	2,24891	NA	NA	0,6279808
CG12378-RA	CG12378	17,2428	19,5009	19,5677	23,7595	0,0264015	18,5261	0,391071	0,1269	0,6279808
CG12379-RB	CG12379	14,3792	2,25383	4,06285	17,3927	3,26968	7,98887	-0,661463	0,029309	0,6279808
CG12384-RA	CG12384	115,828	116,045	2,37752	8,27679	2,52136	0,0967818	-0,122724	0,600389	0,6279808
CG12385-RA	thetaTry	2,63051	1,04026	2627,47	3,21487	204,836	4905,8	-0,33668	0,290228	0,6279808
CG12386-RA	etaTry	8,03965	6,92319	1,17645	0,022302	54,4857	2,93101	-0,626002	0,079807	0,6279808
CG12387-RA	zetaTry	39,0448	0,019392	3,04725	0,165757	110,502	0,013673	-0,544037	0,107464	0,6279808
CG12388-RA	kappaTry	19,1887	3,08942	3,57886	11,1769	0,20635	1,17783	-0,72258	0,034578	0,6279808
CG12389-RA	Fpps	65,6149	4,10164	182,442	40,0409	3,74919	52,2585	0,153051	0,66472	0,6279808
CG12390-RA	CG1239	1,97	5,65287	9,18187	8,59501	8,60356	6,45394	0,007568	0,974788	0,6279808
CG12391-RA	dare	9,88913	9,28401	14,348	0,0819018	0,0821436	0,0836084	0,062998	0,812463	0,13772387
CG12393-RA	CG12391	12,1367	10,0649	17,5916	0,0263723	7,0218	11,4577	-0,010111	0,962893	0,13772387
CG12393-RB	CG12393	0,0400298	0,106587	0,112343	5,86464	8,29858	0,161881	-0,006488	0,976166	0,6279808
CG12393-RC	CG12393	7,43512	0,0364619	0,038431	2,42855	0,0555207	0,0447977	-0,004503	0,983462	0,6279808
CG12395-RA	CG12393	0,0376143	0,0578839	0,0406733	0,374884	0	0,0476879	-0,075279	0,637014	0,6279808
CG12396-RA	CG12395	0	0	0,11548	0,025546	0,0173433	0,0170315	-0,089597	0,750543	0,6279808
CG12398-RA	Nnp-1	16,7305	10,229	2,38825	5,47976	2,52546	2,3003	0,107512	0,762882	0,6279808
CG12399-RA	CG12398	0,225811	1,85987	1,70752	0,341827	0,286181	0,215683	0,279732	0,242003	0,6279808
CG1239-RA	Mad	23,2489	27,4547	20,1147	0,553722	2,11964	11,1317	0,268927	0,371849	0,6279808
CG12400-RA	Non2	42,3035	1,82427	0,0141138	0,0134376	1,5813	0	-0,7109	0,024316	0,6279808
CG12402-RA	Dpse\GA11610	190,273	0,610273	171,114	0	0	14,4706	0,52807	0,02896	0,6279808
CG12403-RA	CG12402	31,6065	34,6567	0,436893	45,0103	15,0189	0,0338947	0,008804	0,964075	0,6279808
CG12404-RA	Vha68-1	46,2157	13,4881	45,9139	7,09321	9,13273	12,9006	0,314465	0,149937	0,6279808
CG12405-RA	Yip1d1	16,6553	18,9697	21,3811	17,7563	10,9157	15,4648	-0,41973	0,224625	0,6279808
CG12408-RA	Prx2540-1	293,679	29,8293	374,16	31,9093	190,729	12,3911	-0,0459	0,897923	0,6279808
CG1240-RA	TpnC4	1,01057	0,637269	0,522421	1,22112	0,253179	36,4119	-0,081847	0,791853	0,6279808
CG12410-RA	Dsim\GD13707	8,30059	18,5334	9,06385	20,0681	0,0180067	0,0137372	0,133641	0,494958	0,6279808
CG12413-RA	cv	15,7701	3,09428	4,06259	4,50715	2,72201	2,93482	0,11451	0,630093	0,6279808

gene_id	Symbol	W1_FPKM	W2_FPKM	W3_FPKM	MCM51_FPKM	MCM52_FPKM	MCM53_FPKM	FC	p-value	p-adj
CG12414-RC	mIF2	4,29737	4,71942	6,29101	5,1898	3,6173	4,98937	0,16052	0,504167	0,6279808
CG12418-RA	nAChRalpha4	4,01566	46,601	5,4165	83,4721	74,3091	29,3226	0,389521	0,185784	0,6279808
CG12419-RA	CG12418	0,314647	0,0178295	35,5527	16,2152	0	5,72693	-0,006255	0,983172	0,6279808
CG1241-RA	CG12419	0,500575	0,286602	0,0180133	8,47568	27,1529	26,6533	-0,026874	0,900945	0,6279808
CG12420-RA	Hsp83	229,172	401,927	260,912	628,548	0,0840781	0,0633664	0,344187	0,243274	0,6279808
CG12423-RA	CG12420	4,58534	12,9507	16,0223	9,60543	2,29293	3,77397	-0,007158	0,964229	0,6279808
CG12423-RB	klhl10	0,109089	0,0883255	0,174554	0,568119	3,6518	0,290385	-0,013096	0,914398	0,6279808
CG12424-RA	klhl10	0,0249092	0,022689	0,0239143	0,0263196	0,25508	0,026868	0,095456	0,702773	0,6279808
CG12424-RB	ckn	6,25779	0,0303716	2,24989	6,31446	3,41878	3,59024	0,088775	0,721919	0,6279808
CG12424-RC	ckn	12,092	0,0298445	5,46522	5,34279	4,65695	6,94947	0,086515	0,730039	0,6279808
CG12426-RB	ckn	0,0165617	0,029756	0,0312086	0,03517	4,15716	2,4497	0,01562	0,897983	0,6279808
CG12428-RA	CG12426	0	2,45146	3,82608	0,0223929	0	0,0228596	-0,010013	0,967475	0,6279808
CG12428-RB	CROT	0,0279785	0,0206992	0,0278028	0,0238866	0,0323545	0,0243843	0,002916	0,990457	0,6279808
CG12428-RC	CROT	0,0289595	0,0255822	6,27306	0,0299029	0,0405037	0,030526	-0,009474	0,969049	0,6279808
CG12428-RD	CROT	6,60567	0,0549648	0,0218171	0,0939269	0,127225	0,0958841	3,45E-05	0,999888	0,6279808
CG12428-RE	CROT	0,0227247	6,1983	0,0269638	1709,47	2447,89	2301,73	0,001749	0,994299	0,6279808
CG1242-RA	CROT	0,0280855	7,72926	13,5352	0,0738265	0,0999984	0,061429	-0,223473	0,456423	0,6279808
CG12432-RA	CG43658	0	0,0520644	0,0914602	26,2945	16,2053	38,1882	-0,273824	0,321783	0,13772387
CG12433-RB	CG43996	0,293332	0,264046	8,665	1,65308	0,327441	1,3047	0,241767	0,352687	0,13772387
CG12433-RC	CG43996	0,052706	10,9503	0	0,459353	1,34841	0,0231666	0,222384	0,396867	0,6279808
CG12436-RA	CG43373	0,471129	0,268211	0,339234	1,85728	0,804592	1,28113	-0,575445	0,10199	0,6279808
CG12437-RA	raw	8,80615	1,1523	8,6598	0,026856	8,73693	8,89555	-0,057385	0,766988	0,6279808
CG12437-RB	raw	10,4264	0,553146	12,5779	7,2239	9,10149	5,71719	-0,016977	0,930847	0,13772387
CG12438-RA	CG12438	0	0	0	0	0	0	NA	NA	0,6279808
CG12439-RA	CG12439	0	0,0171292	29,3704	0,0669957	1,94701	191,472	NA	NA	0,6279808
CG12442-RA	MEP-1	0,013483	1,97141	84,3042	0	0,0165386	60,3121	0,265434	0,365277	0,6279808
CG12442-RB	MEP-1	0,0129417	0,0122988	0,0129445	15,056	0,538947	14,6536	0,265434	0,365277	0,6279808
CG12443-RA	MEP-1	0,0130956	2,05489	0,0124248	34,6967	0	6,51049	-0,07719	0,793465	0,6279808
CG12444-RA	MEP-1	0,0135023	0,0133906	4,03647	12,2215	0	55,2648	-0,231057	0,430888	0,6279808
CG12444-RB	MEP-1	0,014144	12,0373	0,012963	0,0138675	0,634103	15,8443	-0,180789	0,539835	0,6279808
CG12446-RA	MEP-1	18,4982	6,13627	0,013579	0,0132939	56,8136	0,0262085	0,121867	0,539169	0,6279808
CG12448-RA	MEP-1	0,0147009	0,0108529	19,1668	0,0134568	8,82611	0,664936	-0,128004	0,6234	0,6279808
CG12448-RB	wuc	0,507754	0,0924994	0,389979	0,432032	0	0,441035	-0,197797	0,540495	0,6279808
CG12449-RA	wuc	0,116604	0,47795	0,111947	0,460145	0,106039	0,0799172	-0,058146	0,789481	0,6279808
CG12449-RB	ths	3,31057	71,7572	27,6864	26,0881	0,393151	0,250186	-0,058024	0,789784	0,13772387
CG12449-RC	Ir48c	0,148329	0,0510198	0,17524	0,0466483	23,6862	8,89113	-0,058024	0,789784	0,13772387
CG12449-RD	Ir48c	0,0404711	4,16818	14,0815	0,444282	18,9386	0	-0,058239	0,788898	0,6279808
CG12449-RE	CG12446	0,0997748	0,024367	0,0241756	0,0290589	0,0393605	3,56973	-0,058239	0,788898	0,6279808
CG12449-RF	CG12448	0,234173	0,186638	0,140512	3,80434	0	0,396717	-0,058239	0,788898	0,6279808
CG12449-RG	CG12448	0,0862983	0,157213	0,0774564	0,464462	0	0,109104	-0,458367	0,047019	0,6279808
CG12449-RH	Gfat1	0,0496189	0,0451963	0,0476371	0,0557214	0,0754749	0,0568825	-0,057471	0,791893	0,6279808
CG12449-RI	Gfat1	0,0258344	0,0235318	0,0248026	0,0273577	0,0370562	0,0279278	-0,602354	0,011806	0,6279808
CG1244-RA	Gfat1	0,158963	0,144794	0,152614	0,247229	0,334873	0,252381	0,124935	0,596636	0,6279808
CG1244-RB	Gfat1	0,027943	0,0254524	0,0268269	0,0297409	0,0402843	0,0303607	0,126079	0,593742	0,6279808
CG1244-RC	Gfat1	0,0285096	0,0259685	0,027371	0,0303855	0,0411574	31,8674	0,12457	0,598215	0,6279808
CG1244-RD	Gfat1	0,0276275	0,0251651	0,0265241	0,0293829	0,0397993	0,0299951	0,130709	0,581464	0,6279808
CG1244-RE	Gfat1	0,0294368	28,757	0,0282611	0,0314441	0,0425912	0,0320993	0,130751	0,58094	0,6279808
CG1244-RF	Gfat1	140,455	99,7349	152,579	163,297	110,352	102,512	0,130926	0,580425	0,6279808
CG1244-RG	Gfat1	32,112	0,0264944	0,0279252	0,0310441	31,105	0,031691	0,12457	0,598215	0,13772387
CG12455-RA	MED27	0,0646112	0,0588524	16,8741	0,023059	0,0312336	0,747119	-0,253544	0,327281	0,6279808
CG12455-RB	CG42818	4,47869	4,47093	4,20813	8,18914	3,92422	3,3925	-0,260739	0,298324	0,6279808
CG1245-RA	CG42818	0,0101349	0,00923153	0,00973006	0,0103432	0,01401	0,0105587	0,384444	0,068267	0,6279808
CG12460-RA	CG1246	24,3427	0,0114801	0,02016	0,0836698	0,815785	0,0508995	-0,122216	0,716432	0,6279808
CG12464-RA	CR12460	0,214532	0,162842	0,343273	0,722576	9,99953	1,01654	-0,11534	0,404049	0,6279808
CG1246-RB	CG12464	0	1,13026	0,750328	0,0405108	1671,49	5,45269	0,330129	0,195432	0,6279808
CG12470-RA	CG12470	0,684402	0,893353	2,80976	10,6666	0,050324	1,77005	0,019371	0,956832	0,6279808
CG12473-RA	stnB	7,8686	8,8345	7,04453	0,256136	3,78763	3,63348	0,205567	0,355382	0,6279808
CG12473-RB	stnB	4,14013	2,89255	3,93553	0,0654648	4,25364	2,82845	0,205975	0,354951	0,6279808
CG12477-RA	CG12477	0,372608	0,124352	0,794947	0,0980997	0,132877	0,100144	-0,213756	0,528652	0,6279808
CG12478-RA	bru3	21,2635	20,3563	22,19	21,6279	0,0388973	8,45864	0,406626	0,08835	0,6279808
CG12478-RB	bru3	14,4438	19,0981	14,8858	21,6117	0,223678	0,443458	0,414859	0,082653	0,6279808
CG12479-RA	CG12479	0	0	0	0	0	0	NA	NA	0,6279808
CG12480-RA	CG12480	24,5702	9,97429	47,4062	0,0442368	0	40,2615	-0,601465	0,067496	0,6279808
CG12481-RB	CG12481	0	266,222	0	961,006	0,343694	7,86969	-0,070526	0,562659	0,6279808
CG12483-RA	CG12483	0,808433	0,113274	0,724401	4,51387	4,56318	4,93337	0,281159	0,428913	0,6279808
CG12484-RA	side-VIII	0,0216028	0,805415	8,34695	15,78	123,695	97,7685	-0,767045	0,003909	0,13772387
CG12484-RB	side-VIII	0,0176312	0,0350125	18,1017	0,0522319	0,0588523	0,0453107	0,141537	0,566246	0,6279808
CG12484-RC	side-VIII	5,08209	37,0626	0,175779	75,6905	14,0134	28,5044	-0,538509	0,024195	0,6279808
CG12484-RD	side-VIII	0,0130439	0,0659456	0,853704	4,28958	14,5298	0,0443546	0,141537	0,566246	0,6279808
CG12486-RA	pHCl-1	0,427215	15,149	0,865876	0,911687	0,271267	0,204443	0,440673	0,212303	0,6279808
CG12487-RA	BobA	12,2144	6,76363	0,0291306	26,8168	44,6058	0,89663	-0,142678	0,686584	0,6279808
CG12488-RA	CG42663	27,3057	34,562	22,1868	25,7505	14,5009	16,415	0,428021	0,082951	0,6279808
CG12489-RA	dnr1	9,02817	8,77257	114,338	10,3017	1,33435	1,66698	-0,204125	0,414687	0,13772387
CG12490-RA	SmD2	45,1698	39,978	80,2243	57,2221	84,5484	100,993	-0,26905	0,450365	0,6279808
CG12491-RA	CG12490	1,17656	1,23485	1,87509	2,69025	2,03809	1,6638	-0,041811	0,731452	0,13772387
CG12493-RA	CG12491	7,66703	2,0951	3,09168	1,02149	3,71761	33,7119	-0,324881	0,363207	0,6279808
CG12496-RA	CG12493	0,0600871	8,41998	0,0576872	0,0693217	0	0,0707662	-0,644498	0,071583	0,6279808
CG12498-RA	CG12496	0,278349	0,312049	0,698915	0,0140809	0	0	0,01562	0,897983	0,6279808
CG12499-RA	CG12498	0	5,41576	0,00500422	0	0	0	0,067362	0,813335	0,6279808
CG1249-RA	CG12499	3,53367	21,8084	6,0152	0,0407866	0,0453159	2,73042	-0,429593	0,159562	0,6279808
CG12501-RA	Sec23	45,0666	54,8591	80,2547	78,7019	35,7559	45,4881	0,025944	0,860432	0,6279808
CG12502-RB	Sec23	6,49211	45,6783	59,7014	0,0754323	6,68073	6,36121	-1,058448	0,000274	0,6279808
CG12505-RA	Or56a	0,0786618	0,0716506	0	0,0934615	0	0	-0,925893	0,000857	0,6279808
CG12506-RA	CG12502	0,933935	2,46066	3,27114	2,74547	3,02235	0,405491	0,015011	0,959332	0,6279808
CG12507-RB	Arc1	48,0805	13,594	11,3298	2,07329	0	0,38765	-0,15163	0,545332	0,6279808
CG12508-RA	CG12506	10,2225	3,35043	2,45922	4,49783	21,8985	10,2296	0,32499	0,234669	0,6279808
CG1250-RA	CG12507	26,2137	40,259	19,551	28,0642	21,4142	22,6605	-0,152367	0,426033	0,13772387
CG1250-RB	CG43861	12,0544	14,2333	8,31277	11,9922	36,5209	7,5767	-0,151607	0,428146	0,6279808
CG12511-RA	CG12511	0	3,73668	0	0,0557459	0,0471099	2,93495	NA	NA	0,6279808
CG12512-RA	CG12512	7,25689	183,146	340,723	278,268	366,729	74,1739	-0,284588	0,373751	0,13772387
CG12516-RA	CG12516	0	1018,7	1370,25	6,40681	1319,26	5,24275	-0,041811	0,731452	0,6279808
CG12517-RA	CG12517	0	5,0409	0	0	7,45675	10,6527	NA	NA	0,6279808
CG12519-RA	CG12519	65,8796	22,0236	14,222	57,3388	53,421	64,9795	1,011083	0,001573	0,6279808
CG12520-RB	Ccp84Ab	94,0493	82,7835	149,627	79,7644	92,861	100,095	-0,631361	0,05234	0,6279808
CG12521-RA	CG12520	0,888117	27,9441	6,55994	3,63483	26,4782	1,55127	NA	NA	0,6279808
CG12522-RA	CG12521	0	0	0	0	0	0	-0,035733	0,91982	0,13772387
CG12523-RA	CG12522	1,35663	64,8373	61,2261	80,1198	7,20064	11,1331	0,993836	0,002429	0,6279808
CG12525-RA	CG12523	39,6384	1,00249	0,0996386	5,63976	31,0547	23,0529	0,05799	0,71375	0,6279808
CG12526-RA	Ir67a	0	0	0,102251	0,0732933	0	0	-0,011569	0,965103	0,6279808
CG12529-RA	Or67a	0,0808433	0,0736376	0,285751	0,280374	1,19907	0,481523	-0,580153	0,045062	0,6279808
CG12529-RB	Zw	7,58212	8,51521	10,9457	16,4546	289,837	0,0359989	-0,566304	0,049233	0,6279808
CG1252-RA	Zw	0,0327486	0,387051	0,645927	1,41525	0,0870296	0,757656	0,277703	0,312442	0,13772387
CG12530-RA	Cdc42	61,722	1,78073	15,6529	18,6705	49,7104	10,2397	-0,071417	0,728825	0,6279808
CG12530-RB	Cdc42	0,0467407	14,7204	1,67622	3,85287	0,0705806	1,46154	-0,072245	0,726551	0,13772387

gene_id	Symbol	W1_FPKM	W2_FPKM	W3_FPKM	MCM51_FPKM	MCM52_FPKM	MCM53_FPKM	FC	p-value	p-adj
CG12531-RA	CG12531	1,46316	0,0868907	0,0915832	3,65099	16,3377	20,1558	-0,545972	0,059152	0,13772387
CG12532-RA	AP-1-2beta	27,421	3,30413	29,8849	0,121231	13,5444	16,2943	0,142501	0,516994	0,6279808
CG12534-RA	Alr	12,2374	18,6227	19,2017	19,9428	333,765	0,22053	0,009432	0,967593	0,6279808
CG12535-RA	CG12535	0,110187	0,130935	0,138005	0,212197	0,146248	0,123544	-0,16078	0,607838	0,6279808
CG12535-RB	CG12535	0,359367	0	0	0,21164	11,9056	9,72221	-0,116689	0,715462	0,13772387
CG12537-RA	rdx	0,0227091	0,020685	0,0218021	0,0238693	46,8136	48,5358	0,420139	0,058919	0,6279808
CG12537-RB	rdx	58,8166	78,5551	60,7695	56,8408	0,0222875	0,0167972	0,414159	0,062549	0,6279808
CG12537-RC	rdx	0,0240392	0,0218966	0,0230791	0,0253476	0,0260699	0,0196478	0,403007	0,069947	0,13772387
CG12537-RD	rdx	59,408	55,7484	49,6542	33,7388	32,0871	27,8879	0,403175	0,069809	0,6279808
CG12537-RE	rdx	18,5119	20,9824	0,0216606	26,5024	0,0240469	0,0181232	0,440594	0,047603	0,6279808
CG12538-RA	CG12538	0,169425	0	0	0	0	0	0,01562	0,897983	0,6279808
CG12539-RA	CG12539	0,563839	9,99432	37,1128	0,757507	0,386092	0,706729	0,0129	0,970925	0,6279808
CG12540-RA	CG12540	14,8072	32,0813	18,16	2,12698	0	9,985	0,700862	0,030357	0,6279808
CG12541-RA	CG12541	2,78595	0	1,92501	40,5717	67,406	64,3113	-0,008129	0,97799	0,6279808
CG12541-RB	CG12541	0,106803	3,26881	0,038736	4,81706	3,4077	5,71531	-0,109495	0,684659	0,6279808
CG12546-RA	CG12546	825,882	557,334	990,14	934,527	2587,18	2323,97	-0,132324	0,673051	0,6279808
CG12547-RA	CG12547	28,2716	29,701	19,1889	6,05927	0,0330617	26,5222	0,163321	0,417983	0,6279808
CG12548-RA	nompB	1,47675	1,34513	1,44309	1,96394	0,657741	1,10054	0,078477	0,776571	0,6279808
CG12548-RB	nompB	0,0274553	0,500164	0,0263588	0,363747	0,413556	0,0297958	0,067554	0,819339	0,6279808
CG12551-RA	CG43366	1,25859	0,0315995	1,10763	0,037534	1,13925	2,34016	-0,30612	0,363245	0,6279808
CG12552-RA	CG12552	0,193204	9,32236	4,54543	0,318296	0	8,51633	0,024053	0,869957	0,6279808
CG12558-RA	intr	0	0,00634499	19,9208	0,0315343	0,0427135	38,272	0,01562	0,897983	0,6279808
CG12559-RA	rl	0,0426663	0,0388634	0,0409621	0,0470816	0	29,3502	0,189447	0,532908	0,6279808
CG12559-RB	rl	0,0716916	0,0653017	0,0688282	0,0852944	0	0	0,229467	0,449886	0,13772387
CG12559-RC	rl	31,7013	24,6202	12,9658	0,0412462	0,0637723	0	0,227916	0,452786	0,6279808
CG12559-RD	rl	120,658	106,305	61,6071	102,814	0,115532	0	0,227454	0,454593	0,6279808
CG12559-RE	rl	0,04223	0,038466	0,0405433	0,0465494	23,9204	0	0,228	0,452677	0,6279808
CG12559-RF	rl	55,0607	47,7706	24,4218	24,4611	54,3687	0	0,229411	0,449991	0,6279808
CG12560-RA	CG12560	18,2436	4,39054	0,966563	25,1656	10,6737	9,24899	-0,390463	0,136092	0,6279808
CG12567-RA	CG12567	21,132	12,5774	24,5128	0	0	10,6613	-0,036921	0,865488	0,6279808
CG12567-RB	CG12567	20,935	22,5331	14,1226	0	0	3,70138	-0,007129	0,973944	0,6279808
CG12567-RC	CG12567	0,113402	0,103295	0,108873	0	0	0	-0,344083	0,175765	0,6279808
CG12576-RA	alpha-Est3	4,7534	6,62679	11,2347	10,56	9,63912	0,0322069	-0,446413	0,082912	0,6279808
CG12576-RB	CG12576	40,6083	28,2191	44,0912	23,7556	59,3434	38,2812	-0,428159	0,100255	0,13772387
CG1257-RA	CG12576	16,4666	18,9788	25,2995	10,6367	29,7718	22,4261	-0,750311	0,012612	0,13772387
CG12581-RA	pav	1,75076	0,0370911	0,0238783	52,9639	7,88831	15,971	0,305288	0,250142	0,6279808
CG12581-RB	CG12581	37,0906	0,119973	36,854	51,7649	0,693091	24,0429	0,305288	0,250142	0,6279808
CG12582-RA	CG12581	4,97633	0	0,0208628	0,0227879	0	4,53859	-0,018908	0,918239	0,6279808
CG12582-RB	beta-Man	16,7616	15,1737	19,8896	22,1482	0,160615	14,4115	-0,019587	0,91521	0,6279808
CG12586-RA	beta-Man	0,0216311	0,0197031	0,0207672	0,0226781	14,1201	0,0231507	0,581201	0,048282	0,6279808
CG12587-RA	Mur82C	0,21632	0,197039	0,16992	9,05937	1,07829	1,18581	NA	NA	0,6279808
CG12589-RA	CG12587	0	0	13,5581	0,482959	0,0217233	0	0,044335	0,715924	0,6279808
CG1258-RA	CG12589	0	0	0	0	1,13332	23,1233	-0,214267	0,473642	0,6279808
CG12590-RA	Cpr64Ad	224,004	8,12645	4,05379	4,02003	44,866	2,24622	-0,404931	0,131208	0,6279808
CG12591-RA	CG12590	0	0	1,19485	0,195109	0,0655522	0,291859	-0,173617	0,512854	0,6279808
CG12591-RB	dpr16	0,351506	0,470259	0,280694	0,780921	4,97031	0,731005	0,031082	0,907824	0,6279808
CG12592-RA	dpr16	1,31671	1,19935	0,548384	1,02052	3,1671	0,622218	-0,057325	0,870911	0,6279808
CG12594-RA	CG12592	2,32364	0,136787	20,5515	25,2824	1,22965	21,3835	0,339266	0,13369	0,6279808
CG12598-RA	CG12594	7,08099	7,51874	8,97368	2,0364	1,1662	0,623106	-0,163197	0,480781	0,6279808
CG12598-RB	Adar	1,45212	0,0240489	2,34466	53,6124	45,4093	0,0285805	-0,157489	0,496406	0,6279808
CG12598-RC	Adar	1,50855	1,42763	0,996881	0,0279971	3,42055	1,31602	-0,173651	0,452504	0,6279808
CG1259-RB	Adar	4,15277	5,18795	4,96204	2,46448	1,11197	3,87966	0,33589	0,110602	0,6279808
CG12602-RA	Vha100-5	24,4255	0,0678793	0,0715451	0	0,121014	0,0912038	-0,185555	0,493724	0,6279808
CG12605-RA	CG12605	0,0212349	0,0193422	0,0203867	0,0158717	1,13702	6,82851	0,079012	0,813345	0,6279808
CG12605-RB	CG12605	0,0153641	0,0139947	0,0147504	6,14306	0,0389652	0,0293666	0,118767	0,721826	0,13772387
CG12605-RC	CG12605	2,92553	5,0411	2,45398	17,384	0,0387959	0,0292389	-0,062876	0,849062	0,13772387
CG12607-RB	CG12607	53,0087	46,7385	97,7949	51,1965	93,1626	64,5395	0,253831	0,39816	0,6279808
CG12607-RC	CG12607	216,544	184,155	284,707	127,362	245,365	221,594	0,057316	0,851323	0,6279808
CG12608-RA	Dpse\GA11717	14,6698	13,2313	5,82598	21,1454	2,36096	18,004	-0,1013	0,681309	0,6279808
CG12609-RA	CG12609	0	0	0	0,0391625	17,9642	0	-0,098766	0,564164	0,6279808
CG12609-RB	CG12609	0	0	0	0,113482	0,0443037	0	-0,098766	0,564164	0,6279808
CG12611-RA	Andorra	0,174435	0,349552	0,167468	12,5397	0	1,08728	0,115048	0,662536	0,6279808
CG12617-RA	CG12617	0	0,594301	0,170835	0	0	0	0,166661	0,313577	0,6279808
CG12620-RA	Acp62F	0	0,0117882	0,690823	0,01221	17,2173	0,732634	NA	NA	0,6279808
CG12621-RA	CG12620	0	0	8,39742	12,517	0	0	-0,567731	0,092472	0,6279808
CG12622-RA	beat-IIIa	0,683792	3,73275	0,253747	1,38894	0	2,46508	-0,134201	0,569699	0,6279808
CG12625-RA	Gr10b	0,0883367	0,29756	0,250904	0,258837	6,00924	0,484955	0,296346	0,338277	0,6279808
CG1262-RA	CG44422	2,21897	1,94633	2,10609	0,0319937	3,02692	2,22503	0,044335	0,715924	0,6279808
CG12630-RA	RpL8	1465,7	0	0,0699568	1,11944	0,176032	1,49501	-0,161303	0,577625	0,6279808
CG12632-RB	RpL8	0,054578	0,757294	0,0179369	0,0189479	0,0263469	0,664848	0,008246	0,981489	0,6279808
CG12636-RA	tio	1,62325	0,608915	0	0	0	0	0,065796	0,591492	0,6279808
CG12637-RA	fd3F	0,640625	0,416804	0,556463	12,3559	9,3349	9,97506	0,286968	0,365105	0,6279808
CG1263-RA	CG42685	0	0,0511066	0	0	0	0	-0,756901	0,008939	0,13772387
CG1263-RB	CG12637	0,225515	2,35817	3,09011	2,79629	0,318758	0,149141	-0,756814	0,008947	0,13772387
CG12640-RA	lab	8,41376	0,00911548	1,45557	5,9854	1,01161	1,73781	-0,032747	0,880872	0,13772387
CG12641-RA	CG12640	0,28481	55,4194	0,455724	1407,24	0	0	-0,43662	0,131562	0,13772387
CG12643-RA	CG43740	5,89963	1,97995	8,60928	10,7669	17,6133	6,50483	0,019202	0,94078	0,13772387
CG12644-RA	CG12643	37,7029	55,9664	8,22508	1,46068	5,23011	1,38718	-0,121731	0,60537	0,13772387
CG12645-RA	CG43902	0	0,958219	0,238484	1,85966	1,56378	0	-0,014945	0,944553	0,6279808
CG12645-RB	CG12645	0,148596	0,203027	0	1,45719	1,15154	0,351388	-0,107174	0,724477	0,6279808
CG1264-RA	CG12645	0,250366	0,199544	0,127383	0,132313	0,179218	0,227614	0,549227	0,040704	0,6279808
CG12653-RA	CG1265	42,171	33,5655	20,4382	0,129068	2,35887	57,0399	0,177674	0,57573	0,6279808
CG12655-RA	btd	0,930488	4,14401	8,96446	5,12331	1,80288	2,31526	-0,634876	0,062946	0,6279808
CG12659-RB	CG12655	9,85632	8,47626	18,5024	13,8821	1,44446	30,6187	0,082924	0,724146	0,6279808
CG1265-RB	CG12659	0,0522889	0,0476284	0,0502005	3,15055	0,0800799	0,351135	-0,343923	0,214498	0,6279808
CG12661-RA	CG12661	0	0,0254847	0,491758	0,822283	0,691454	0	-0,187894	0,355694	0,13772387
CG12662-RA	CG12662	0,512216	0,0267535	0,0375588	1,1453	1,74953	2,44766	-0,532406	0,135559	0,6279808
CG12663-RA	Ir7a	0	0,173282	0	0,0282734	0	0	NA	NA	0,6279808
CG12664-RB	fend	5,84665	6,23167	7,75785	0,0147656	0,0411195	3,74081	0,217655	0,458834	0,13772387
CG12665-RA	Obp8a	0,28481	0,0470697	0,324319	0,058336	5,21506	3,62081	-0,824692	0,020178	0,6279808
CG12672-RA	CG12672	0,683115	0,90506	1,17255	0,741911	0,730683	2,09337	0,40372	0,229942	0,6279808
CG12673-RA	olf413	4,5886	5,86026	3,58919	8,06895	4,57797	5,78324	0,14775	0,646195	0,13772387
CG12674-RA	CG12674	0	0,129267	0,136247	0,102793	8,44558	0,226103	-0,08767	0,701055	0,6279808
CG12676-RA	ed	40,33	48,5906	36,7445	63,4983	10,3378	12,3974	0,24787	0,425805	0,6279808
CG12679-RA	CG12679	0	0	0	0,0577227	0	67,2568	NA	NA	0,6279808
CG12680-RB	VhaM9.7-a	10,6139	16,4115	0,0564049	22,6758	7,96143	14,6584	0,01562	0,897983	0,6279808
CG12681-RA	VhaM9.7-a	0,100165	15,1372	58,3664	16,0351	0,0355932	17,6974	0,083443	0,799822	0,6279808
CG12682-RA	VhaM9.7-a	35,2878	9,52975	3,80227	1,42668	46,0048	0	NA	NA	0,6279808
CG12684-RA	CG12680	0	0	0	10,263	0	0	-0,070526	0,562659	0,6279808
CG12685-RA	boil	0,135401	0,0164639	0,19499	0,0187912	2,04141	0,0191827	0,054343	0,730585	0,6279808
CG12688-RA	CG12682	0	0,0173916	0,0294826	0,0198977	1,51119	0,0203123	-0,131143	0,534102	0,13772387
CG12689-RA	CG12684	0	0,32727	0	0,982997	0	0,550143	0,244515	0,493981	0,6279808
CG1268-RA	CG12685	0,778672	3,12722	5,4765	9,11922	2,81875	3,33391	0,159337	0,519698	0,6279808
CG1268-RB	CG12688	0	0	0	1,03601	0	0	0,144508	0,563118	0,6279808

gene_id	Symbol	W1_FPKM	W2_FPKM	W3_FPKM	MCM51_FPKM	MCM52_FPKM	MCM53_FPKM	FC	p-value	p-adj
CG1268-RC	CG12689	1,15923	1,00311	1,16857	0,0341847	0,0463034	0,034897	0,144508	0,563118	0,6279808
CG12690-RA	CHES-1-like	2,09131	16,6264	2,55536	0	23,6044	22,5684	0,55242	0,008491	0,6279808
CG12690-RB	CHES-1-like	16,0417	0	13,6458	18,166	266,001	2,20096	0,553959	0,008196	0,6279808
CG12691-RA	CG12691	0	0	0	1,14253	0	0	NA	NA	0,13772387
CG12692-RB	CG12692	0,0566689	0,0860299	0	61,9577	0	0,0652413	-0,047689	0,831471	0,6279808
CG12693-RB	CG12693	0,066564	0,0144274	0,127811	0	0,881511	0,016722	0,183203	0,397987	0,6279808
CG12697-RA	Or13a	0,394248	0,119703	0,277568	18,8458	6,15266	0,154121	0,384232	0,275226	0,6279808
CG12698-RA	CG12698	0,0518921	0,141801	0,0830325	0	0,215401	0,127321	0,086192	0,776858	0,13772387
CG12699-RA	CG12699	0,127724	0	0,245245	0,172335	0	0	-0,011489	0,962463	0,6279808
CG12700-RA	SkpD	0,250287	0,0430005	130,844	0	1,18429	215,259	0,018068	0,892878	0,13772387
CG12701-RA	zld	0,0100777	34,1103	3,35728	0,0243725	0,400604	1,76713	0,445719	0,087137	0,6279808
CG12701-RB	zld	5,79607	0,0210983	5,27859	1,28309	2,62998	4,48739	0,44613	0,085109	0,13772387
CG12702-RA	CG12702	0,0231628	6,16704	0,0222377	0,528012	2,25778	4549,32	-0,020146	0,937855	0,6279808
CG12703-RA	Pmp70	49,2665	0,96384	65,8379	6,54611	0,0142402	0,123757	-0,291942	0,297718	0,6279808
CG12708-RA	CG12708	0,635485	1,02507	0,606084	0,426824	4,40584	2,37966	-0,781836	0,014865	0,6279808
CG12708-RB	CG12708	0,0433379	11,5416	10,0079	6,21658	3,89912	0,247939	-0,781836	0,014865	0,6279808
CG12713-RA	CG1271	0,032961	22,4751	19,9787	23,2455	3,7803	17,8669	-0,052952	0,862167	0,13772387
CG12714-RB	CG1271	1,78293	0,0510921	1,48729	0,0355111	0,0585819	0,0362511	0,635054	0,023258	0,6279808
CG12715-RA	CG1271	3,64177	30,354	0,0326041	0,538068	38,8764	0,993057	0,025379	0,930867	0,6279808
CG12716-RA	CG1271	0,0395033	0,0300232	4,3612	3,39331	0,0914903	3,79583	0,022155	0,888764	0,6279808
CG12717-RA	CG12713	0,0509189	0,0463805	0,0488852	0,0179125	0,0777093	0,467446	0,1766	0,498683	0,6279808
CG1271-RA	CG43313	3,7395	2,68745	3,75483	2,87784	29,4463	27,4371	0,337795	0,210862	0,6279808
CG1271-RB	CG12715	0,108821	0,158594	0,104474	1,85564	0	19,5297	0,3407	0,208517	0,6279808
CG1271-RC	CG12716	0	0	1,10453	0,679943	0	0	0,3407	0,208517	0,6279808
CG1271-RD	CG12717	1,62283	1,76191	3,96045	0,0382531	0,00718632	0,0390502	0,337795	0,210862	0,6279808
CG12721-RA	moon	0,488245	0,741213	4,55845	14,3968	0,0108571	14,8502	-0,04089	0,908979	0,6279808
CG12723-RA	CG12723	22,9326	31,2474	15,1944	1,28298	9,46709	12,3479	0,479784	0,165108	0,6279808
CG12725-RA	CG12725	0	0,183495	0,193405	1,55407	25,7469	0,0630102	-0,367609	0,152357	0,6279808
CG12728-RA	CG12728	4,48826	3,56971	5,46506	5,89858	8,45801	5,89604	-0,252553	0,286364	0,6279808
CG12729-RA	CG12729	0,119847	0	0,115061	11,062	2,23272	0	0,024053	0,869957	0,6279808
CG12730-RA	CG1273	11,775	14,3244	10,2523	22,876	2,34376	0,638443	0,091824	0,69144	0,6279808
CG12734-RA	CG12730	42,9653	50,0987	44,6056	0,177109	15,111	7,10668	0,211638	0,387421	0,6279808
CG12734-RB	Girdin	0,0129747	0,0684974	0,0124565	4,68618	0,0307812	52,1755	0,213204	0,384143	0,6279808
CG12736-RA	Girdin	6,24005	3,29359	6,42314	2,37243	0,0315093	0,092204	-0,194843	0,390231	0,6279808
CG12737-RA	mEFTu2	18,9792	0,00934476	0,00984941	0,0152544	0,0206622	1,63631	-0,157485	0,479191	0,6279808
CG12737-RB	Crag	3,58639	5,45454	0,0105295	0	4,39014	0,0158999	-0,15842	0,475514	0,6279808
CG1273-RB	Crag	7,07762	6,74251	9,81838	17,5147	4,43633	14,959	0,065107	0,84976	0,6279808
CG12740-RA	Jafrac2	27,2699	1,29921	1,47909	0,0432496	17,6868	0,0441508	-0,091436	0,729943	0,6279808
CG12740-RB	Jafrac2	0,0560916	5,48207	60,0365	89,4814	36,384	49,5204	-0,091408	0,730013	0,6279808
CG12740-RC	RpL28	0,0819121	4,29532	3,42976	0,100212	6,63897	24,1843	-0,09134	0,73021	0,6279808
CG12740-RD	RpL28	0,0649287	9,54838	9,71426	0,0758666	0,0423085	0,0322842	-0,091427	0,729957	0,13772387
CG12743-RA	RpL28	0,0791642	11,8236	9,28791	0,0961192	6,17329	3,0039	-0,331779	0,350705	0,6279808
CG12743-RB	RpL28	1123,02	0,016989	2,25622	1580,22	5,74611	1,4138	-0,345394	0,331446	0,13772387
CG12743-RC	otu	0,0213079	0,0194087	0,181941	0,188741	0,0307918	0,0232066	-0,345394	0,331446	0,6279808
CG12744-RA	otu	0,0204759	0,0186509	37,8797	20,147	0,029509	0,0222398	0,163809	0,555711	0,6279808
CG12744-RB	otu	0,0203875	0,0185703	0	0,0725263	0,0293731	0,0221373	0,163809	0,555711	0,6279808
CG12746-RB	CG12744	0,0729314	9,39805	7,87525	0,0621314	0,155078	0,116876	-0,108778	0,660945	0,6279808
CG12746-RD	CG12744	0,0849304	0,0497545	0,0524415	1,9788	11,1311	12,3002	-0,106323	0,668257	0,6279808
CG12746-RE	CG12746	0,0515948	0,0113118	6,10278	9,04305	0,0374292	5,03875	-0,115376	0,640538	0,6279808
CG12749-RA	CG12746	9,14204	0,0126265	0,0208217	0,0276099	6,96323	2,09641	0,306434	0,219115	0,6279808
CG12749-RB	CG12746	10,2516	0,0130793	17,5579	0,0276331	0,0308024	13,9207	0,307515	0,21724	0,6279808
CG1274-RA	Hrb87F	30,4641	22,1045	20,5884	0,0166245	7,36122	18,9496	-0,251726	0,374194	0,6279808
CG1274-RB	Hrb87F	35,9939	32,6907	60,5129	10,6718	0,022518	30,4077	-0,251557	0,374829	0,6279808
CG12750-RA	CG1275	13,6004	0,0452983	9,08408	0,0558631	0,854174	0	0,138086	0,636673	0,6279808
CG12752-RA	CG1275	9,15497	0,0373211	0,0477446	0,0450227	104,484	0	0,015914	0,948412	0,6279808
CG12753-RA	CG1275	3,43143	0,26065	3,77631	1,70284	9,46504	3,87032	-0,412203	0,044068	0,6279808
CG12753-RB	CG1275	0,040973	1,82383	0	62,1986	4,62911	0,00757015	-0,445748	0,028877	0,6279808
CG12754-RA	ncm	10,4375	7,06169	14,173	7,63264	4,45059	5,28833	0,040164	0,887106	0,6279808
CG12755-RB	Nxt1	37,9539	0,337357	0,600727	0,58633	22,1103	2,9721	0,475588	0,145108	0,6279808
CG12755-RC	ema	1,52849	3,04149	4,22295	0,0892212	11,0655	5,64426	0,475588	0,145108	0,6279808
CG12756-RA	ema	7,39125	0,0639601	0,0674142	39,4299	0,903193	2,09691	-0,118714	0,614198	0,6279808
CG12758-RA	Or42b	0,0825949	0,125389	0,290752	12,2855	0,0572663	0,101148	-0,033774	0,881954	0,6279808
CG12758-RB	l(3)mbn	0,0310555	0,0162578	0,0171358	0,0333035	0,676799	0,828182	-0,033206	0,883686	0,6279808
CG12758-RC	l(3)mbn	0,0320187	1,75701	0,0188969	0,0344168	0,0282929	0,035134	-0,04717	0,835317	0,6279808
CG12758-RD	Eaf6	11,7481	187,493	30,0135	1,03328	0,00919397	24,1832	-0,046729	0,836896	0,6279808
CG12758-RE	sano	6,08216	0,0134716	1,07006	9,73421	0,613848	0	-0,03264	0,885789	0,6279808
CG12759-RA	sano	2,80124	11,3019	0,0235766	3,82753	0,035117	0	-0,080276	0,759284	0,6279808
CG1275-RA	sano	0,014947	0	0,252468	0,0154258	0,523866	0	-0,787245	0,013968	0,6279808
CG1275-RB	sano	0,0147898	0,0643628	0	0,0152579	0,12322	0,631792	-0,77031	0,016149	0,6279808
CG1275-RC	sano	11,3562	0,0404569	0	14,088	0,0666786	0,0264663	-0,702453	0,025757	0,6279808
CG1275-RD	Dbp45A	8,50005	9,39229	0,0308594	0,0377923	1,96441	10,733	-0,699223	0,026541	0,6279808
CG12763-RA	TfIIEbeta	0,061888	294,182	0,0594162	0,0262776	10,3651	16,2658	0,225499	0,352316	0,6279808
CG12765-RA	TfIIEbeta	13,9363	0,0535149	17,298	42,9635	26,0079	29,3623	-0,095314	0,761258	0,6279808
CG12766-RA	DptA	0,0991138	0,399468	0,0464851	132,783	4,02294	0,203787	-0,347779	0,328545	0,6279808
CG12767-RA	fsd	7,20887	17,1791	25,5216	4,95739	5,54895	7,82855	0,214372	0,362662	0,6279808
CG12768-RA	CG12766	2,74459	4,49669	7,74021	2,83228	8,90532	7,09375	-0,091732	0,75129	0,6279808
CG12769-RA	Dlip3	17,4085	21,6537	4,29194	0,0266604	0,0193622	0,0145926	0,301565	0,359215	0,6279808
CG12769-RB	CG12768	5,57809	12,2085	4,7266	0	3,70438	0	0,293364	0,372109	0,6279808
CG1276-RA	CG12769	0,0173306	0,0157859	0,0166384	0,00845359	0,0114504	0,00862974	0,04849	0,831245	0,6279808
CG1276-RB	CG12769	22,4866	39,0583	14,0319	3,66875	0,0110712	0,00834392	0,04849	0,831245	0,6279808
CG12770-RA	Vps28	25,2833	29,2839	11,7126	38,0438	36,0318	9,35551	-0,122515	0,63205	0,6279808
CG12772-RA	CG12772	3,85657	4,84966	2,87975	5,93142	0,0210969	4,12528	-0,21552	0,414327	0,6279808
CG12773-RA	CG12773	8,40102	7,73541	1,01409	1,03256	16,2789	0,690276	-0,281063	0,165428	0,6279808
CG12775-RA	RpL21	6561,16	6405,18	3876	8749,64	10808,8	9261,73	-0,384999	0,175549	0,6279808
CG12780-RA	CG12780	0,763253	0,0251335	0,314044	0,0293436	16,7305	0,0299551	-0,325544	0,316326	0,6279808
CG12781-RA	nahoda	54,9901	67,8042	38,5701	49,0769	0	26,1312	0,308072	0,269743	0,6279808
CG12781-RB	nahoda	12,5345	15,0201	10,7565	29,0075	0	11,0971	0,321641	0,249593	0,6279808
CG12782-RA	CG12782	0	0	0	0	0	0	-0,448869	0,087148	0,6279808
CG12783-RA	CG12783	0,107406	0,0586994	0	7,29913	3,75742	4,21671	0,111933	0,748769	0,6279808
CG12784-RA	CG12784	0	0	3,18479	1,51686	437,844	5,31493	NA	NA	0,6279808
CG12785-RA	Mat89Ba	4,56072	0,851696	9,07171	50,8448	0,0295579	0,0346454	-0,009003	0,977502	0,6279808
CG12787-RA	hoe1	1,40094	0,0189048	8,13033	0,020141	26,5878	53,5808	-0,567366	0,021378	0,6279808
CG12787-RB	hoe1	1,86245	3,53651	0,0462816	8,65495	0,759414	1117,49	-0,576577	0,019093	0,6279808
CG12787-RC	hoe1	0,0190384	0,0173415	89,3491	0,171354	0,0357414	0,859896	-0,644639	0,01057	0,6279808
CG12787-RD	hoe1	1,7977	2,77582	1,8891	108,292	6179,36	0,0607536	-0,58036	0,018509	0,6279808
CG12787-RE	hoe1	2,96511	0,0175949	0,106813	1,0488	0,0825997	234,875	-0,579973	0,018571	0,6279808
CG12787-RF	hoe1	2,91511	3,39876	237,791	0,0609814	30,9097	0,0622521	-0,579954	0,018536	0,6279808
CG12788-RA	CG12788	16,1611	13,6717	23,0486	49,6716	3,86766	83,0041	-0,42184	0,133456	0,6279808
CG12789-RA	santa-maria	3,76517	5,04638	3,22931	2,95656	2,14993	1,62285	0,598132	0,008334	0,6279808
CG12789-RB	santa-maria	3,4702	3,27181	204,29	148,863	11,2495	163,926	0,609668	0,007142	0,6279808
CG12792-RA	Rtnl2	0,347768	0,147275	0,200327	10,3833	10,9253	832,979	-0,360971	0,22168	0,6279808
CG12794-RA	l(2)09851	17,7747	11,4181	1,41883	2,43215	2,9781	83,4662	0,164775	0,637124	0,6279808
CG12795-RB	lcs	2,65226	1,7047	4,76053	5,60627	5,44487	1,11571	0,11982	0,592419	0,13772387
CG12796-RA	CG12795	9,96834	11,3498	4,6717	13,4329	2,74685	11,2951	-0,007311	0,981785	0,6279808

gene_id	Symbol	W1_FPKM	W2_FPKM	W3_FPKM	MCM51_FPKM	MCM52_FPKM	MCM53_FPKM	FC	p-value	p-adj
CG12797-RA	mAChR-C	0	2,91978	3,8776	5,75795	2,8241	3,40258	-0,18008	0,502002	0,6279808
CG12798-RA	Ciao1	43,9202	10,4411	71,3113	11,5347	0,123356	67,5281	NA	NA	0,6279808
CG12799-RA	CR12798	0	0	0	0,0223322	0,0302491	0,0227976	0,199425	0,552102	0,6279808
CG1279-RA	Ubc84D	0,715107	5,02881	0,892509	0,0603949	5,866	0,626247	-0,413913	0,195603	0,6279808
CG12800-RA	Cyp6d4	6,95722	5,91893	8,08641	4,20326	3,20835	0,0177128	-0,53998	0,035376	0,6279808
CG12806-RA	Teh1	1,85405	1,75417	0,110968	0,155949	1,67184	1,28919	0,234216	0,361111	0,6279808
CG12807-RA	Spn85F	0,687217	0,985894	0,8577	0,720299	3,15193	3,50853	0,55672	0,035694	0,6279808
CG12809-RA	nerfin-2	0,696202	0,720624	0,622823	0,0276099	0,250724	3,08304	0,034936	0,912426	0,6279808
CG12811-RA	CG12811	36,223	26,1103	12,2298	6,6734	1,83673	0,947016	-0,108952	0,700415	0,6279808
CG12812-RA	Fancl	6,08025	1,95175	15,0611	9,87897	0,457663	3,43048	-0,254421	0,377199	0,6279808
CG12813-RA	Npc2d	9,29661	0,518037	0,645288	0	28,9697	0,538061	-0,525313	0,139145	0,6279808
CG12814-RA	CG12814	0,0294106	3,52277	3,05778	0,0314141	4,10882	30,0788	0,279422	0,155742	0,6279808
CG12814-RB	CG12814	45,6325	9,20217	6,85851	42,1925	19204,1	17851,2	0,264445	0,179026	0,6279808
CG12817-RA	CG12817	10,2456	6,17343	2,97242	4,91774	5,91232	0,180083	0,230577	0,297351	0,6279808
CG12817-RB	CG12817	0,110371	4,64071	10,2262	0,176407	2,73434	0,832239	0,068881	0,776685	0,6279808
CG12817-RC	CG12817	0,12642	9,56002	0,105963	5,98171	3,68403	4,1388	0,168326	0,471234	0,13772387
CG12818-RA	CG12818	5,04976	4,50948	9,14863	5,31347	4,23203	0,0128233	0,146717	0,589095	0,6279808
CG12819-RA	sle	3,47792	0,0126985	4,91351	5,51265	30,3956	1,02162	-0,057867	0,849918	0,6279808
CG12819-RB	sle	0,0142692	2,46869	0,0136993	0,014703	0,0724131	1,81209	-0,057625	0,850656	0,6279808
CG12821-RB	Atg10	0,0590138	0,00782591	0,00824532	0,00873371	0,0118299	0,00897279	0,735499	0,001639	0,6279808
CG12821-RC	Atg10	16,8774	0,00782286	0,00823569	0,00872331	1,20122	0,00897279	0,459493	0,02783	0,6279808
CG12822-RA	CG12822	0,0430742	0,00781676	0,012368	0,00873371	0,0118299	0,00897279	0,119226	0,607987	0,6279808
CG12822-RB	CG12822	0,0446459	0,00782591	0,00824854	0,00873719	0,0118346	0,0089692	0,119226	0,607987	0,6279808
CG12824-RA	CG12824	0,964348	0,683196	1,49162	0,140939	0,190902	0,00890862	0,22269	0,532644	0,6279808
CG12824-RB	CG12824	0,58926	0,107348	0,113145	0,570537	1,94414	0,00890862	0,22269	0,532644	0,6279808
CG12825-RA	CG12825	29,1645	58,9996	42,2183	24,8007	38,8367	0,00890508	0,261221	0,370595	0,6279808
CG12826-RA	CG12826	7,90221	8,20093	60,0803	32,5588	8,12135	7,34201	-0,439898	0,218355	0,6279808
CG12828-RA	CG12828	0	0	0	0	76,3866	7,91609	-0,260785	0,383854	0,6279808
CG12831-RB	CG12831	0	36,9013	27,7488	39,5475	0,164766	42,9894	-0,143489	0,451492	0,6279808
CG12832-RA	Tsp42Eq	29,1349	19,3717	20,5276	19,6687	0,120362	1,21942	-0,268664	0,410376	0,6279808
CG12833-RA	esn	0,0153808	0,0140099	0,0147665	17,5621	0,0218029	3,44551	0,671905	0,013676	0,6279808
CG12833-RB	esn	17,9287	24,4734	16,1856	0,0425514	6,64729	0	0,683067	0,012054	0,6279808
CG12835-RA	esn	2,94563	4,05512	3,43857	144,896	2,46506	82,9723	-0,086343	0,775231	0,6279808
CG12836-RA	CG12836	0,374014	0	0,093749	0,121643	1,32804	0,124178	-0,81868	0,021186	0,6279808
CG12837-RA	Tsp42Er	39,3593	43,8205	0,518666	34,189	119,381	86,446	-0,871011	0,008761	0,6279808
CG12838-RA	Tsp42Eo	12,2671	11,0314	18,1908	18,2178	11,8199	0,0907118	-0,907178	0,004422	0,6279808
CG12839-RA	Tsp42En	22,3173	77,3617	30,5748	294,159	85,1346	33,9868	-1,065409	0,000324	0,6279808
CG12840-RA	Tsp42El	33,9686	47,0189	47,0564	44,1165	175,404	58,802	-0,432936	0,054052	0,6279808
CG12841-RA	Tsp42Ek	3,16104	302,163	32,4308	63,9787	57,1752	149,855	-0,123925	0,682507	0,6279808
CG12842-RA	CR12842	2,82894	10,3109	3,49193	72,6305	5,15885	3,19118	-0,044783	0,899278	0,6279808
CG12843-RA	Tsp42Ei	32,6095	26,6053	34,0168	28,2371	48,6369	37,2724	-0,288158	0,31608	0,6279808
CG12844-RA	Tsp42Eh	1,82338	2,02462	2,00063	3,20789	7,59447	3,27474	-0,332336	0,238872	0,6279808
CG12844-RB	Tsp42Eh	6,88716	81,7251	6,058	4,19558	5,89279	5,01495	-0,326373	0,246895	0,6279808
CG12844-RC	Tsp42Eh	0,0864111	2,37266	4,60427	8,54505	4,38377	5,26264	-0,289886	0,303093	0,6279808
CG12845-RA	Tsp42Ef	34,3398	34,2325	40,3024	34,1899	48,5171	41,2199	-0,239871	0,36247	0,6279808
CG12846-RA	Tsp42Ed	46,933	39,7736	86,6315	55,2591	85,4727	85,7213	-0,30695	0,313741	0,6279808
CG12847-RA	Tsp42Ec	32,876	24,8417	63,4057	43,4036	90,3689	71,0662	-0,571498	0,084787	0,6279808
CG12848-RA	CG12848	70,8472	63,9399	110,494	11,2022	0,051664	0,0389371	0,006354	0,982284	0,6279808
CG12849-RA	CG12849	0	18,1124	5,82913	9,34169	0	0	-0,080649	0,53498	0,6279808
CG12851-RA	CG12851	4,62355	5,1798	3,35181	4,89862	28,2312	2,00536	0,355008	0,204521	0,6279808
CG12853-RA	CG12853	0,298536	6,28738	8,13669	9,88797	2,72627	3,50671	-0,414369	0,134947	0,6279808
CG12855-RA	HPS1	6,2257	13,3873	6,90133	0,0910709	56,2698	5,62285	0,053139	0,796794	0,6279808
CG12856-RA	CG12856	0,199825	0,0689683	0,0857638	0,362633	18,9906	0,370189	-0,387966	0,231169	0,6279808
CG12857-RA	CG12857	0,25396	0,315443	24,6427	24,9557	13,0642	14,9802	0,662385	0,038823	0,6279808
CG12858-RA	jef	7,05533	8,50097	2,29144	10,5377	232,106	336,645	-0,079287	0,756496	0,6279808
CG12859-RA	Dpse\GA11862	123,561	21,2454	193,596	15,6962	470,292	23,27	-0,672237	0,031613	0,6279808
CG12860-RA	CG12860	0	0,125703	0,101175	7,77031	0,38749	0,368929	-0,02005	0,902249	0,6279808
CG12861-RA	CG12861	0	0	0	0	0	0,111671	-0,041811	0,731452	0,6279808
CG12862-RA	CG12862	0	11,6237	18,5275	12,8484	0,0580676	0,101453	NA	NA	0,6279808
CG12862-RB	CG12862	0	0	0	332,319	12,9647	13,1668	-0,041811	0,731452	0,6279808
CG12863-RA	CG12863	2,38659	3,28212	219,561	0	0	0	0,712382	0,01079	0,6279808
CG12864-RA	Su(var)2-HP2	1,7348	4,1502	0,054004	0,0642519	0,0747793	4,87543	0,033881	0,909994	0,6279808
CG12864-RB	Su(var)2-HP2	0,557325	8,12134	15,8868	11,2722	0,074158	12,7717	0,122732	0,6876	0,6279808
CG12865-RA	lncRNA:CR43428	0	0	0	205,247	2,80695	1,11133	0,01562	0,897983	0,6279808
CG12866-RA	CG12866	0,0658651	255,918	364,213	0,670875	0,715752	196,727	0,228604	0,467876	0,6279808
CG12868-RA	CG12868	59,5623	23,1969	3,36129	1,22566	199,627	277,612	-1,349438	7,14E-06	0,6279808
CG12869-RA	CG12869	4,67681	5,59255	4,52328	5,19542	3,14789	1360,98	0,305553	0,189266	0,13772387
CG12876-RA	Mics1	0	20,0859	1088,37	3,53092	0,109419	0,0443546	-0,326716	0,107565	0,6279808
CG12877-RA	Dpse\GA11876	18,7448	0,0308543	0,0325205	0,0365756	0,0495419	0,0373377	0,228577	0,38076	0,6279808
CG12877-RB	CG12877	0,0213722	0,126019	0	0,0212587	0,0303314	0,0217016	0,250251	0,339276	0,6279808
CG12877-RC	CG12877	0,0203394	4,58412	3,00265	3,46571	0,028795	2,16885	0,228577	0,38076	0,6279808
CG12878-RA	CG12877	2,69134	0	0	0	1,88397	0	0,106703	0,689773	0,13772387
CG12878-RB	Dpse\GA11878	3,06417	3,74572	10,3703	0,0308985	0,0418522	0,0315423	0,107242	0,688287	0,6279808
CG12879-RA	Dpse\GA11878	8,46679	0,0261037	0,026861	6,23487	4,0463	5,14568	0,012658	0,968169	0,6279808
CG1287-RA	Dpse\GA11879	0,683396	0,875369	1,47623	1,15101	1,12396	8,2408	-0,214347	0,233793	0,6279808
CG12880-RA	CG1288	0,100318	0,256691	0,397873	0,324454	7,08369	0,204722	-0,184423	0,496648	0,6279808
CG12883-RA	CG12880	3,55002	5,02268	4,06068	6,91853	5,12741	0	-0,172215	0,547148	0,13772387
CG12885-RA	CG12883	8,73339	4,34811	11,7319	15,4921	12,2645	11,8081	-0,134461	0,621585	0,6279808
CG1288-RA	CG12885	1,03038	0,072865	0	1,22649	0	0	0,022862	0,858938	0,6279808
CG12891-RA	whd	36,1173	26,9063	33,1594	74,748	23,2265	21,8783	0,805022	0,000432	0,6279808
CG12891-RB	whd	19,2997	514,858	20,8604	584,329	2,72843	3,85575	0,811002	0,0004	0,6279808
CG12892-RA	Caf1-105	1,63168	1,92029	168,19	1127,21	0,0435471	0,0328197	-0,043609	0,887236	0,6279808
CG12895-RA	CG12895	22,7208	547,356	146,633	40,3027	51,9044	15,6251	0,110834	0,640104	0,6279808
CG12896-RA	CG12896	270,669	5,26372	2,62009	9,80765	0,0296956	0,0223804	1,011048	0,004552	0,6279808
CG12897-RA	SLO2	0,37331	0,203333	0	0,881768	0,407512	0,494715	-0,009062	0,97444	0,6279808
CG12898-RA	CG12898	0,210433	0,191677	0	0	0	0	0,044335	0,715924	0,6279808
CG12900-RA	Ir47a	0,0332374	0,113416	0	0,172708	0	0,176307	0,01562	0,897983	0,6279808
CG12901-RA	SLO2	0,114186	0,124765	0	0	0	0	0,076861	0,699219	0,6279808
CG12901-RB	SLO2	0,3883	0	0	0	0	0	0,01562	0,897983	0,13772387
CG12901-RC	SLO2	0,128385	0,0131078	0	0,014832	0	0,0151411	0,01562	0,897983	0,6279808
CG12902-RA	CG12902	0	0	0	0,342321	0	0	NA	NA	0,6279808
CG12902-RB	CG12902	0	0	0	0,116006	0	0	NA	NA	0,6279808
CG12905-RA	Obp46a	0,2767	2,15192	4,24093	2,72568	3,38906	17,4566	-1,222594	0,000593	0,6279808
CG12906-RA	Gr47a	0,0821839	75,4245	0,210404	78,2892	0	44,0381	0,239319	0,220289	0,6279808
CG12907-RA	CG46298	0,104002	0	0	0,104259	0	0	0,01562	0,897983	0,6279808
CG12908-RA	Ndg	0,0143904	0,567633	0,0138156	0,718167	0,0200901	0,408869	0,286342	0,225332	0,6279808
CG12908-RB	Ndg	66,941	0,00988175	60,2428	0,305901	34,882	0,176536	0,286342	0,225332	0,6279808
CG12909-RA	CG12909	10,9588	8,7453	23,0905	15,073	15,9906	2,46746	-0,165166	0,584824	0,6279808
CG12910-RA	Alg2	7,99701	6,45001	15,8056	12,2292	42,4579	12,5948	-0,107177	0,624478	0,13772387
CG12911-RA	CG12910	0,039075	172,335	0,0375144	0,0253087	0,0599593	310,717	-0,105162	0,614659	0,13772387
CG12911-RB	CG12911	13,574	15,5479	22,7079	23,6137	18,0429	11,1365	-0,105502	0,613079	0,6279808
CG12912-RA	CG12911	11,6899	13,8043	12,8463	14,9997	13,1821	13,6187	-0,454916	0,192826	0,13772387
CG12913-RA	CG12912	0,682199	0,887706	0,654952	4,53582	0,0386614	1,18049	-0,383792	0,12401	0,13772387
CG12914-RA	CG12913	0,035989	0,0327813	0,0345516	0,0403372	0,054637	0,0411777	-0,199952	0,57272	0,13772387

gene_id	Symbol	W1_FPKM	W2_FPKM	W3_FPKM	MCM51_FPKM	MCM52_FPKM	MCM53_FPKM	FC	p-value	p-adj
CG12917-RB	CG12914	0,0525663	0,0478811	0,0504668	0,0624849	0,0846363	0,063787	0,090436	0,799409	0,13772387
CG12918-RA	CG12917	0,265899	0,262382	0,638197	0,0214734	0,0290859	0,0219209	-0,37393	0,200859	0,6279808
CG12919-RA	sel	56,478	37,9559	0,0217721	0,0238348	1,79133	2,76769	0,348075	0,072209	0,13772387
CG12919-RB	egr	19,8912	21,8752	0,0540808	17,3483	0,0919616	8,06141	0,348075	0,072209	0,13772387
CG1291-RA	egr	6,84409	5,47626	69,469	7,2918	83,0613	3,09638	-0,25338	0,380297	0,6279808
CG12920-RA	CG12920	18,2778	1,23684	22,588	34,7429	14,1126	4,45229	0,985187	2,37E-05	0,6279808
CG12921-RA	mRpL42	49,5292	47,0871	65,7824	72,7762	0	0	-0,608806	0,042021	0,6279808
CG12923-RA	CG12923	0,0546231	0,0773605	0,0815383	0,013031	0,160678	0,285909	-0,606427	0,048991	0,6279808
CG12924-RA	Lsm11	1,51579	1,46521	2,16802	3,24288	0,773384	166,735	0,633457	0,049286	0,6279808
CG12926-RA	CG12926	27,637	14,7586	42,9502	22,6674	6,50058	6,99206	-0,467633	0,15273	0,6279808
CG12928-RB	clos	0,0898175	0,0147347	0,0152946	0,199357	9,86415	0,0166228	-0,261568	0,306457	0,6279808
CG12929-RA	CG12929	0,143435	0,13065	0,137706	0,211516	0,2865	0,215924	-0,534895	0,063174	0,6279808
CG12929-RB	CG12929	9,8457	0,149469	0,157541	0,259906	30,0106	0,265322	-0,534895	0,063174	0,6279808
CG12931-RA	Or45b	0,776711	23,6923	0,719057	0,0321655	1,25184	13,962	-0,161157	0,630292	0,6279808
CG12934-RA	CG12934	99,5892	36,8769	215,183	208,394	1,6575	0,0126615	-0,84319	0,015658	0,6279808
CG12935-RA	CG12935	11,2264	0,0558488	17,2007	11,2049	0,0190767	2,05936	-0,170485	0,595135	0,6279808
CG12936-RA	mms4	0,0613137	7,92431	0,0588648	0,0368414	0,0197697	14,6959	-0,324964	0,23855	0,6279808
CG12938-RA	Lsm10	3,33277	7,41167	5,6786	4,24162	12,2308	3,02695	0,147492	0,659217	0,6279808
CG12942-RA	CG12942	0,0278136	0,0253345	0,142661	11,6195	0,0334708	0,0252256	0,034766	0,894164	0,6279808
CG12943-RA	polyph	0	0	0,0267027	3,31958	0,0411195	0,0309901	-0,013096	0,914398	0,6279808
CG12943-RB	polyph	0	5,85886	142,171	0,0616378	0,0482561	0,0363687	-0,013096	0,914398	0,6279808
CG12944-RA	Obp47a	0,148596	0,135351	0	341,048	25,0312	32,4377	0,018704	0,958251	0,6279808
CG12944-RB	Obp47a	1,8454	1,19065	39,483	42,2339	0,0356786	0,0268895	0,018704	0,958251	0,6279808
CG12945-RB	CG12945	8,53264	13,3623	9,23596	2,42918	3,96921	6,34133	0,275574	0,225529	0,6279808
CG12946-RA	Whamy	9,27747	12,2887	13,4491	10,4376	28,2011	1,61047	-0,258206	0,312656	0,6279808
CG12946-RB	Whamy	1,32297	2,62276	1,59887	3,66787	18,8585	68,6381	-0,270912	0,288229	0,6279808
CG12947-RB	CG12947	0,0491392	0,0447593	0,0471765	0,0551155	0,0746542	0,0562639	0,405691	0,154632	0,6279808
CG12947-RC	CG12947	5,53236	5,85921	5,72551	7,67291	3,73603	3,70609	0,364608	0,225511	0,6279808
CG12948-RA	CG12948	24,9996	1,21513	1,16389	26,7838	35,7888	32,6496	-0,142897	0,556765	0,6279808
CG12950-RA	CG43367	1,58193	13,598	1,51874	7,99147	3,38538	8,59062	0,171834	0,511559	0,6279808
CG12951-RA	side-VII	3,84962	9,41828	7,34333	1,17261	6,11424	1,21838	-0,472089	0,151229	0,6279808
CG12952-RA	CG12951	0,207005	0,113132	0,357726	0,524025	16,4079	17,9189	0,547779	0,025891	0,6279808
CG12952-RB	sage	1,18345	0,0769974	0,0811556	5,00339	0,141103	0,106344	0,546957	0,030525	0,6279808
CG12954-RA	sage	5,49683	5,84656	6,55563	0,296777	3,1384	4,1813	-0,416716	0,18178	0,6279808
CG1295-RA	mRpL41	37,7282	36,0672	0,583449	0,82328	78,7026	0,40055	-0,286856	0,411184	0,6279808
CG12960-RB	Ir52a	0	0,0501554	13,8704	0	1674,4	7,44075	0,019745	0,891952	0,6279808
CG12963-RA	CG12963	24,0094	15,4717	27,2375	15,7682	0,153478	43,5499	-0,297487	0,364028	0,6279808
CG12963-RB	CG12963	0,0904321	4,32452	14,1517	3,28628	0,150629	0,11567	-0,306728	0,347907	0,6279808
CG12963-RC	CG12963	0,0890908	0,0811501	0,0855325	0,111206	4,77156	0,113523	-0,295562	0,367019	0,6279808
CG12963-RD	CG12963	0,0581824	0,0529965	0,0558585	1,89007	12,3986	0,0681838	-0,299636	0,360223	0,6279808
CG12964-RB	CG12964	47,0489	0,61043	36,0727	1,32033	1,48103	1525,9	0,505111	0,134608	0,6279808
CG12970-RA	CG12970	0,0554482	0,0505061	0,0532336	0,0670639	0,0856105	0,0645212	-0,577508	0,081412	0,6279808
CG12970-RB	CG12970	0,233552	0,159552	0,14014	13,168	0,323679	0,295742	-0,577508	0,081412	0,6279808
CG12971-RA	CG12971	1,75201	0,721934	0	2,15624	0	4,83387	-0,677909	0,055669	0,6279808
CG12972-RA	ebd2	6,40409	0,245682	0,222818	0,266418	0,108305	0,151523	0,366567	0,107043	0,6279808
CG12974-RA	CG12974	25,2776	17,4127	26,4333	0,053159	0,365003	46,5085	-0,49715	0,132622	0,6279808
CG12974-RB	CG12974	0,0475822	0,0433411	57,0229	4,57755	0,0493228	0,0542667	-0,540074	0,10438	0,6279808
CG12975-RA	CG12975	18,7639	9,96556	28,7441	21,1589	23,6395	69,9253	-0,146931	0,643211	0,6279808
CG12983-RA	kune	79,246	69,543	89,3432	93,6864	0,0279913	0,0905897	0,059792	0,706172	0,13772387
CG12983-RB	CG12983	0	0,0255316	0,0180749	0,0500499	0	0,261716	0,01562	0,897983	0,6279808
CG12983-RC	CG12983	0	0,0168921	0,0734222	0,0331839	0	0	0,01562	0,897983	0,6279808
CG12983-RD	CG12983	0	0,148486	0,091276	0,0214695	0	0	0,059792	0,706172	0,6279808
CG12984-RA	CG12983	0	0,0865993	0	0,0762635	0	0	0,105879	0,742165	0,6279808
CG12985-RA	CG12984	0,505683	0,409432	0,161829	0,0948082	0	0	0,311314	0,381944	0,6279808
CG12986-RA	CG12985	0,584311	19,3506	22,0287	16,9717	26,0463	23,6464	-0,632969	0,076921	0,6279808
CG1298-RA	CG12986	0,87402	0,796118	0,839111	0,395648	0,348883	0,403892	0,077206	0,698994	0,6279808
CG12990-RA	CG1299	10,3718	1,5483	13,471	5,70166	2,34573	10,5356	0,700404	0,008465	0,6279808
CG12991-RA	CG12990	0,0290231	94,5156	5,83798	0,0125783	10,0738	4,65	0,449453	0,07767	0,6279808
CG12991-RB	CG12991	24,5289	0,0272501	0,921013	14,2503	14,9578	0,289472	0,454432	0,074948	0,6279808
CG12992-RA	CG12991	0,0299166	0,34643	25,1237	29,242	0,0433357	0,20821	0,245829	0,49009	0,6279808
CG12993-RA	CG12992	0,557816	0,245284	0,0287217	0,0319937	0,384088	19,4646	0,010229	0,976408	0,6279808
CG12994-RA	p-cup	0,140812	2,53822	4,24586	4,06487	0,811582	1,25373	0,11268	0,750483	0,6279808
CG12995-RA	CG12994	0,765686	0,317734	37,8308	0,770548	0,263169	0,0196536	0,09498	0,756484	0,13772387
CG12996-RB	CG12995	0,316455	0,977053	0,668395	0,0776097	0,41899	0,50865	-0,083369	0,814674	0,13772387
CG12997-RA	f	0,7985	128,73	0,383304	31,8297	53,8833	8,98217	0,377069	0,200295	0,13772387
CG12998-RA	CG12997	6,95095	7,10575	13,3947	0,389591	7,06733	0,137129	-0,711933	0,03522	0,13772387
CG1299-RA	CG12998	5,33565	3,73097	128,907	6,48293	17,2011	0,277187	-0,242904	0,279386	0,6279808
CG13000-RA	CG13000	0,694491	0,27111	22,6809	16,2504	15,0296	14,0288	-0,018177	0,954064	0,6279808
CG13001-RA	CG13001	14,9347	11,1766	17,9672	39,1272	0	3,88346	0,009478	0,965512	0,6279808
CG13002-RA	CG13002	0	4,1374	86,0449	7,1621	40,3617	0	-0,156277	0,46499	0,6279808
CG13003-RA	CG13003	7,40947	0,0195622	20,617	0,0748326	4,74419	0,076392	0,353864	0,154648	0,6279808
CG13003-RB	CG13003	0,0135633	6,21879	0,148913	5,28705	0,506515	6,8916	0,354578	0,153215	0,6279808
CG13004-RA	CG13004	2,80579	7,03649	2,58878	5,98792	14,3993	0,0411526	0,333434	0,236027	0,6279808
CG13004-RB	CG13004	9,47912	12,157	8,20867	0,0869833	16,8598	12,0098	0,223337	0,412055	0,6279808
CG13005-RA	CG13005	1,01956	6,91658	0,584301	9,25425	0,478378	2,39843	-0,521974	0,082443	0,6279808
CG13008-RA	CG13008	0,105928	0,160811	19,5217	0,0837761	0,374849	16,2234	-0,20693	0,53315	0,6279808
CG13008-RB	CG13008	0,0448174	0,0918513	0,0222767	0,135674	0,0711517	0,0249265	-0,108447	0,650949	0,6279808
CG13010-RA	CG13010	0,489451	0,222913	0,391585	1,30141	0,312589	116,749	-0,264165	0,429124	0,6279808
CG13011-RA	sing	3,42201	2,94698	4,3008	3,02512	100,387	3,8302	0,316956	0,330629	0,6279808
CG13012-RB	CG13012	0,196264	0,178771	0,188425	0,351134	0,475613	0,358451	0,161506	0,626242	0,6279808
CG13014-RA	CG13014	6,82655	7,15668	8,40878	0,0575466	6,13154	12,3427	-0,472601	0,105505	0,6279808
CG13016-RA	CG13016	5,26268	4,36036	6,67188	0,504584	0,818035	4,41191	0,341192	0,139614	0,6279808
CG13018-RA	CG13018	43,7158	4,66813	30,9251	12,7464	0,424302	4,31287	-0,468662	0,101974	0,13772387
CG13021-RA	CG13021	0,0780422	0,0148364	0,0704853	0,073213	0,0991674	0,0747386	0,044335	0,715924	0,6279808
CG13021-RB	CG13021	0,0767727	3,13132	0,0694057	0,0720915	0,0976485	0,0735938	0,044335	0,715924	0,6279808
CG13021-RC	CG13021	0,0609743	0,0158524	0,0557931	0,0579522	0,0784966	0,0591598	0,0799	0,654847	0,6279808
CG13022-RB	CG13022	1,3173	1,15189	0,0406646	3,65833	2,12458	3,15078	-0,373817	0,292929	0,6279808
CG13023-RA	CG13023	0,454405	4,47624	0,949499	4,35294	0,940662	2,85363	-0,495339	0,165378	0,6279808
CG13024-RB	CG13024	23,2175	34,8944	24,9108	22,333	0,0548048	16,2006	0,583017	0,015366	0,6279808
CG13025-RA	CG13025	0,0214974	0,0195813	0,0206388	0,0225308	0,0305181	0,0230002	0,645138	0,023786	0,13772387
CG13026-RB	CG13026	0,496396	0,653109	2,09186	0,602412	0,378694	5,30645	0,285533	0,373906	0,6279808
CG13027-RB	Obp73a	0,0847127	0,0771622	1,2141	1,471	0,198484	0,150997	-0,11534	0,404049	0,6279808
CG13028-RA	CG13028	4,559	0,26782	4,8885	1,30552	0,092726	1,02118	0,912972	0,002299	0,6279808
CG13029-RB	CG13029	0,0594385	0,0541407	0,0570645	0,0684574	3,63765	0,0698839	-0,221285	0,519116	0,6279808
CG13029-RC	CG13029	0,627929	1,08372	0,856681	1,32478	0	1,15595	-0,221285	0,519116	0,6279808
CG13030-RB	agt	0,102975	0,0937968	0,0988622	0,133785	0,181212	0,136572	0,136973	0,455979	0,6279808
CG13031-RA	Dpse\GA11985	0,136709	0,10377	0	0,0792271	2,57454	0	-0,074616	0,70555	0,13772387
CG13032-RA	CG13031	0	0,162374	0,688379	0,905567	0,802191	0,409875	0,145309	0,658137	0,13772387
CG13033-RA	CG13032	0,0755326	0,0825603	31,0714	83,2709	332,793	21,474	-0,069899	0,810192	0,13772387
CG13035-RA	CG13033	197,748	216,409	318,374	194,356	299,211	327,509	-0,477694	0,177777	0,6279808
CG13035-RB	CG44836	0,204456	0,143256	0,362381	3,68201	154,131	140,668	-0,574032	0,107852	0,6279808
CG13036-RA	CG44836	0,0304965	0,0277784	0,0292785	0,0170372	0,0650743	0,0490439	-0,438205	0,202443	0,6279808
CG13037-RA	CG44836	0,516218	0,391839	0,413	1,17205	3,01872	0,555284	-0,092002	0,764088	0,6279808
CG13038-RA	CG33257	33,3295	27,5265	50,7961	29,1447	3,03887	3,5408	0,137287	0,613867	0,6279808

gene_id	Symbol	W1_FPKM	W2_FPKM	W3_FPKM	MCM51_FPKM	MCM52_FPKM	MCM53_FPKM	FC	p-value	p-adj
CG13039-RA	CG13038	23,3849	368,106	0	0,599023	1,36805	1,16278	0,020909	0,891561	0,13772387
CG1303-RA	CG13039	0,140189	1,07861	15,2561	1,02993	1,13592	30,4393	0,115087	0,647816	0,6279808
CG13040-RA	CG1304	1,99113	7,16346	41,3856	0,696394	66,8742	4,88769	0,726231	0,042137	0,6279808
CG13041-RA	CG13040	2,2499	0	3,18321	0,160055	0,349213	0,352058	-0,072935	0,799408	0,6279808
CG13042-RA	CG13041	607,664	583,773	971,43	0	0	0,199986	0,196779	0,47684	0,6279808
CG13043-RA	CG13042	18,7588	0,332522	31,8976	0,400499	0,542478	0,546897	-0,066639	0,800669	0,13772387
CG13044-RA	CG13043	370,537	0,154757	602,256	571,254	712,491	647,903	0,319621	0,199057	0,13772387
CG13045-RA	CG13044	263,048	303,121	433,729	299,568	332,996	327,213	0,546136	0,048276	0,6279808
CG13046-RB	CG13045	8,78669	393,566	8,37951	37,684	5,34932	36,7083	0,713054	0,001231	0,6279808
CG13047-RB	CG13046	41,8118	0,0804989	48,2747	203,468	31,0871	930,455	0,47751	0,060754	0,6279808
CG13048-RA	CG13047	160,692	55,8427	314,553	10,6422	158,334	209,445	0,500836	0,048624	0,6279808
CG13049-RA	CG13048	47,9875	57,3758	51,0763	66,8786	96,5901	181,11	0,687872	0,017751	0,6279808
CG13049-RB	CG13049	234,819	10,0311	295,748	323,18	0,149119	0,112385	0,687933	0,017739	0,6279808
CG1304-RA	CG13049	0,0883759	48,8326	0,0848461	0,110091	1117,94	7,04495	0,348963	0,241041	0,6279808
CG13050-RA	CG13050	0,45886	0,696603	3,16254	0,332012	0,449712	0	0,384216	0,206745	0,6279808
CG13051-RA	CG13051	395,144	0,0518849	763,13	0	0	0,0656232	0,213926	0,486542	0,6279808
CG13053-RA	CG13053	0	0	0,26286	0	1,48197	0	-0,197766	0,336888	0,6279808
CG13054-RA	CG13054	0,219927	0,356133	0,516128	0	0,477166	0	0,044813	0,899039	0,6279808
CG13055-RA	CG13055	0,426296	0,173949	0,375164	0,915261	4,71674	0,189125	-0,428164	0,229847	0,6279808
CG13056-RA	CG13056	3,13078	0,779801	0,193405	6,99025	0,534707	1127,57	-0,437248	0,216117	0,6279808
CG13057-RA	CG33060	0	2,85173	8,62171	416,197	1198,45	0,80641	-0,179789	0,492645	0,6279808
CG13058-RA	CG13058	23,134	26,3905	628,047	1,7468	33,1256	0,917291	-0,162496	0,585808	0,6279808
CG13059-RA	CG13059	628,286	697,996	988,489	781,159	916,338	970,765	0,32188	0,206902	0,6279808
CG13060-RA	CG13060	393,194	25,6969	22,2551	25,0839	0	0,490834	0,160145	0,580662	0,6279808
CG13061-RA	Nplp3	0	566,639	0,17524	34,4894	0,148959	0,477281	-0,033999	0,877843	0,6279808
CG13062-RA	CG13062	0,16519	17,0868	0,158592	0,109973	39,0462	31,6076	-0,024468	0,942548	0,6279808
CG13063-RA	CG13063	431,442	543,067	675,358	692,055	1376,4	1322,4	-0,218155	0,472796	0,6279808
CG13064-RA	CG13064	5,88176	3,07772	6,608	38,3916	17,4641	39,1916	-0,900276	0,007703	0,6279808
CG13065-RA	CG13065	1,79238	1,50025	1,17476	0,430622	0	0,439596	1,656906	3,63E-06	0,6279808
CG13066-RA	CG13066	93,4928	173,977	210,685	326,941	488,096	456,303	-0,038218	0,900085	0,6279808
CG13067-RA	CG13067	420,957	671,784	691,025	509,068	27,2431	40,9335	-0,128354	0,682232	0,6279808
CG13068-RA	CG13068	106,092	195,767	257,309	32,7843	63,0504	291,195	0,301206	0,359553	0,6279808
CG13069-RA	CG13069	159,159	295,837	323,813	124,557	439,327	448,98	0,161495	0,61029	0,6279808
CG13070-RA	CG1307	8,17434	8,22135	0,0459132	5,02664	0	2,54535	NA	NA	0,6279808
CG13071-RA	CG13070	0	0,358253	0	45,4469	0,83666	1,0157	NA	NA	0,6279808
CG13072-RA	CG13071	0	0	0	15,6213	0	0,631339	-0,068288	0,806711	0,6279808
CG13073-RA	PDCD-5	26,655	22,0467	42,3566	1,17547	35,4534	42,8686	-0,041811	0,731452	0,6279808
CG13073-RB	CG13073	0	10,568	0	7,74916	0,258098	1,01655	-0,041811	0,731452	0,6279808
CG13074-RA	CG13073	0	0,474957	339,967	0,617687	0,0238286	0,194518	-0,011736	0,971618	0,6279808
CG13075-RA	CG13074	0,950206	0,99867	0,771908	1,13588	0,117485	33,7639	-0,111297	0,732999	0,6279808
CG13076-RA	CG13075	15,7092	6,78414	3,55424	25,9299	3,41112	3,34919	0,040746	0,873969	0,6279808
CG13076-RB	Notum	0,0173261	0,0157818	0,0166341	0,017981	0,0243553	0,0183557	0,040746	0,873969	0,13772387
CG13077-RA	Notum	4,11688	4,03099	3,23306	5,13909	2,29798	3,03126	-0,319057	0,372136	0,6279808
CG13078-RA	CG13077	1,5554	53,1081	17,7148	338,565	25,6093	7,75831	-0,931408	0,009229	0,6279808
CG13079-RA	CG13078	0,987681	12,7061	3,89912	0,0102149	2,534	2,2623	0,070569	0,748259	0,6279808
CG1307-RB	Tep5	0,0317367	0,028908	0,0304692	0,0102149	0	0	-0,561543	0,085889	0,6279808
CG13081-RA	CG43367	0,0128278	0,14694	12,3155	0,0161479	10,4013	0,0164843	-0,056829	0,824172	0,6279808
CG13082-RA	CG13081	0,185606	4,84974	1,58592	0,0102149	9,39821	0,060191	-0,210497	0,525536	0,13772387
CG13083-RA	CG13082	16,8979	47,6219	0	0,0358006	0	0,304346	-0,113909	0,731179	0,13772387
CG13083-RB	CG13083	0,0736083	22,4885	0,318008	0,0120581	14,8367	9,42737	-0,113909	0,731179	0,6279808
CG13084-RA	CG13083	0,329692	20,1036	0,0791309	5,14906	0,119236	0,454556	0,11473	0,590073	0,13772387
CG13085-RA	CG13084	0,121018	11,5255	0,116185	1,20519	4,66591	3,58374	-0,398741	0,119315	0,13772387
CG13086-RA	CG13085	0,0554017	0,0376243	0,0259561	0,00950884	14,4012	5,65422	-0,645707	0,067553	0,6279808
CG13087-RA	CG13086	4,99481	6,79931	0,00961212	11,3383	0,0138362	0	0,287616	0,417699	0,6279808
CG13088-RA	d	1,4069	0,883796	0,419186	24,2376	0,883342	2,50931	0,263602	0,364403	0,6279808
CG13089-RA	CG13088	3,13368	1,95728	19,0796	0,434373	20,6274	12,6035	-0,170055	0,502031	0,6279808
CG1308-RA	PIG-U	7,71706	0,0874804	0,147527	43,2941	4,36583	0,177344	-0,145189	0,629455	0,6279808
CG13090-RA	CG1309	17,3467	8,04148	10,19	5,93864	11,5082	12,0021	-0,085977	0,764063	0,6279808
CG13091-RA	Uba4	4,57694	21,9371	3,43132	5,94568	8,47179	3,65194	-0,087217	0,770185	0,13772387
CG13094-RA	Sgp	0,0960405	3,56822	8,03993	11,3314	0	6,04545	-0,458735	0,129715	0,6279808
CG13094-RC	Dh31	8,35482	11,1001	0,114163	10,5972	0,219548	0,165464	-0,458735	0,129715	0,6279808
CG13094-RD	Dh31	5,28108	0,0485897	72,4727	2,79416	95,6365	75,464	-0,778567	0,014977	0,6279808
CG13095-RA	Dh31	0,118912	0,108314	12,5199	0,162087	6,50104	11,4835	-0,618634	0,061745	0,6279808
CG13096-RA	Bace	30,2227	26,5233	59,9164	45,8101	0,0492684	77,649	-0,107529	0,732825	0,6279808
CG13097-RA	CG13096	18,0046	9,74078	0,0256241	0,028322	0,0383623	0,0289121	-0,037023	0,882024	0,13772387
CG13098-RA	CG13097	8,21038	7,26389	13,9546	27,4528	16,2207	21,8917	-0,490725	0,129634	0,6279808
CG1309-RA	mRpL51	29,873	8,04796	12,4915	0,140338	17,4096	13,5081	0,140991	0,500316	0,6279808
CG13101-RA	CG13101	3,5358	0,0405114	0,0766145	0,105339	0,142682	0,107534	-0,259812	0,357229	0,6279808
CG13101-RB	CG13101	27,4411	34,6121	0,0280239	0,0311615	0,0422085	0,0318109	-0,258188	0,360435	0,6279808
CG13102-RA	CG13102	18,6117	0,159224	3,22259	0,17091	0,0907461	20,8875	-0,190928	0,574583	0,6279808
CG13106-RA	Or30a	0,262206	285,396	0,139852	3,57318	0,285133	724,669	0,117868	0,697356	0,6279808
CG13108-RA	CG13108	9,71704	10,0311	12,0343	10,692	12,6021	11,4025	-0,084654	0,734196	0,6279808
CG13109-RA	tai	9,88637	12,9022	9,15448	13,5092	921,757	11,793	0,059846	0,808234	0,6279808
CG13110-RA	Ctl1	17,989	11,4852	16,3697	44,0838	0,0971679	23,8462	NA	NA	0,6279808
CG13113-RA	CG13110	0	0	0,0494955	0	0	0,21097	-0,041811	0,731452	0,6279808
CG13114-RB	CG13113	0	14,908	11,4598	11,9606	8,53416	6,32021	0,332485	0,223518	0,6279808
CG13116-RA	CG13114	0,0773722	0	0,148564	0,19746	0,515859	6,29411	0,657936	0,064858	0,6279808
CG13117-RA	CG13116	6,73843	20,4098	47,0486	68,5176	36,3245	54,0408	0,505863	0,114199	0,6279808
CG1311-RA	CG13117	2,97294	6,59518	0,234371	0,0607565	0,723222	0,0620225	0,0789	0,752516	0,6279808
CG13120-RA	ppk18	0,0535605	0,0487866	0,218337	0,416291	0,0257911	0,116461	0,256823	0,465619	0,6279808
CG13121-RA	ppk18	0,244121	0,185761	0,0180543	12,6321	0,154527	10,1096	-0,157075	0,660733	0,6279808
CG13123-RA	CG13123	3,53869	3,27886	4,68601	4,57458	12,8726	4,7101	-0,193234	0,470287	0,6279808
CG13124-RA	CG13124	12,5125	8,01868	0,0207468	7,45217	9,75725	8,2317	-0,069955	0,774785	0,6279808
CG13124-RB	CG13124	8,2323	8,54343	5,32928	37,6882	8,86466	13,4636	-0,071521	0,76982	0,13772387
CG13124-RD	CG13124	3,08461	0,0199977	37,0592	11,0899	2,79478	0,0235148	-0,058307	0,811337	0,6279808
CG13125-RA	TbCMF46	0,0515546	0,0469595	0,0494955	0,0581814	20,9696	1,01716	0,21437	0,467915	0,6279808
CG13125-RB	TbCMF46	0,930281	1,0239	1,00477	1,71452	8,9964	0,0432426	0,259816	0,386185	0,6279808
CG13126-RA	CG13126	5,32404	4,09764	6,61708	7,69064	193,08	28,4988	-0,129688	0,590976	0,13772387
CG13127-RA	CG13127	0	1,35176	0	0	0	0	NA	NA	0,6279808
CG13130-RA	lncRNA:CR13130	0,0872865	0,0795066	0,0838003	0,0870432	0,117901	0,0888569	-0,037875	0,76416	0,13772387
CG13131-RA	CG13131	0,0491555	0,0149247	0	0,0980195	0	0,0333217	-0,092463	0,734598	0,6279808
CG13131-RB	CG13131	0,0417555	0,114102	0	0,0296206	0	0,0277152	-0,065255	0,809663	0,6279808
CG13133-RA	CG13133	5,71026	5,64713	148,614	105,462	0,950833	121,617	-0,422707	0,17523	0,6279808
CG13134-RA	CG44153	0,372049	0,881108	6,7473	1,09154	6,55309	56,1489	-0,245951	0,477408	0,6279808
CG13135-RA	CG13135	0	0	0,13661	3,33572	0,259169	35,0589	-0,736484	0,015158	0,6279808
CG13136-RA	CG44153	0,27763	0,800801	0,7552	5,38713	140,055	1,51771	-0,17016	0,632632	0,6279808
CG13137-RA	CG13137	0	0,0810408	0,270032	0	0,101075	0,240517	NA	NA	0,6279808
CG13138-RA	CG13138	0,14908	0,037695	0,0397307	33,4203	286,74	29,2674	-0,021134	0,932873	0,6279808
CG13138-RB	CG13138	0,0413836	26,7315	38,0963	27,3535	0,211234	4,5668	-0,00167	0,995167	0,6279808
CG13139-RA	lft	13,9603	6,478	15,4858	9,87398	6,87419	9,8283	0,350948	0,163604	0,6279808
CG13140-RA	CG1314	0	0,106789	0	0,260772	2,10393	2,06094	0,303024	0,179488	0,6279808
CG13142-RB	dpr19	10,6818	10,8886	31,1284	33,8892	14,8193	20,9209	0,580085	0,037971	0,13772387
CG13144-RA	Nse4	2,32509	2,36412	35,2675	2,48491	1,63762	1,66216	-0,042397	0,905613	0,13772387
CG13148-RA	CG13144	0,641147	5,08647	0,0209385	3,64906	9,52687	9,77696	0,521143	0,034046	0,6279808

gene_id	Symbol	W1_FPKM	W2_FPKM	W3_FPKM	MCM51_FPKM	MCM52_FPKM	MCM53_FPKM	FC	p-value	p-adj
CG13148-RB	CG13148	0,0424743	2,03115	2,14084	3,3785	1,38067	3,17389	0,521143	0,034046	0,6279808
CG1314-RA	CG13148	4,56633	3,21494	2,90146	1,33496	1,60349	0,896978	-0,27362	0,262227	0,6279808
CG13151-RA	Ass	11,151	6,06466	24,2956	0	21,3226	0	0,252883	0,289138	0,6279808
CG13154-RA	CG13151	2,94672	3,06301	3,41147	19,7656	0,386344	0,450614	-0,292616	0,318407	0,6279808
CG13155-RA	CG13154	0,0763293	7,91347	0,0732807	0,13191	0,0265113	0,0199805	0,044335	0,715924	0,6279808
CG13157-RA	CG13155	0	0,126336	22,6017	8,87007	17,7823	13,5202	-0,366892	0,297003	0,13772387
CG13158-RA	CG13157	0,447095	0,514414	0,790701	10,7113	0,0625653	0,047153	0,2374	0,369587	0,6279808
CG13159-RA	Or49a	0,221917	1,74336	3,04701	101,235	22,8772	0,0553837	0,903295	0,002855	0,6279808
CG1315-RA	CG13159	1541,72	19,9886	24,3701	3446,52	18,5601	15,6367	-0,754636	0,03198	0,6279808
CG13160-RA	CG1316	29,5971	0,0563719	31,604	15,6953	19,4312	13,9034	0,075932	0,830431	0,6279808
CG13162-RA	CG13160	1,49406	0,0231605	1,81864	0,0553005	27,5994	0,0347707	-0,486089	0,127612	0,6279808
CG13163-RA	ana3	0,417568	11,0211	21,4687	1,0829	0,57451	14,2488	-0,097233	0,745205	0,6279808
CG13164-RA	mIF3	6,85039	0,0114343	6,83404	0,0128833	8,79275	8,75665	0,18429	0,300209	0,6279808
CG13164-RC	CG13164	0	0,0199381	1,92867	0	0	2,6282	0,18429	0,300209	0,6279808
CG13164-RE	CG13164	0	0,0202058	1,93288	0	0	0,0354861	0,18429	0,300209	0,6279808
CG13164-RG	CG13164	0	1,38023	0,0216754	2,51348	0	0	0,18429	0,300209	0,6279808
CG13165-RA	CG13164	0	3,75881	0,0549394	2,07997	0	0	-1,014568	0,003786	0,6279808
CG13167-RA	CG42700	0,637459	1,33933	1,58275	1,58583	3,66861	2,10568	-0,164408	0,381435	0,6279808
CG13168-RB	Vha36-2	0	1,47029	1,67883	0,0497476	0,393258	0	-0,114919	0,673918	0,13772387
CG13168-RC	CG13168	1,61416	0,142622	3051,57	0,3698	1,6674	1,43253	-0,114919	0,673918	0,6279808
CG13169-RA	CG13168	0,0445355	0,600664	0,0303573	1,45289	0,0668785	0,0504036	-0,241115	0,416258	0,6279808
CG1316-RA	CG43316	0,176485	0	0,0706948	17,7206	0	0,221567	0,053258	0,815685	0,6279808
CG13170-RA	CG1317	13,1983	14,1565	13,2835	0,00741562	0,0609835	10,7946	0,183203	0,397987	0,6279808
CG13171-RA	CG13170	0,279983	22,6362	0,101662	0,0864326	0	0	NA	NA	0,6279808
CG13175-RA	CG13171	0	0,425046	0,6272	0	0	0,342453	0,047142	0,85993	0,6279808
CG13176-RA	CG13175	2,53119	26,8773	3,64514	2,53489	57,8918	2,49902	-0,270412	0,342625	0,6279808
CG13177-RA	wash	4,99367	3,66653	6,38014	4,80829	6,17023	6,26529	0,140938	0,657443	0,13772387
CG13178-RA	CG13177	0,635345	8,25436	2,19589	2,3675	94,3433	0,847579	-0,116613	0,730317	0,13772387
CG1317-RB	OSCP1	0,799598	1,32424	1,18639	1,57911	4,39264	4,97647	-0,030663	0,895236	0,13772387
CG13183-RA	Hexo1	11,9445	13,93	18,0407	19,6746	1,04361	1,81717	0,338023	0,085423	0,13772387
CG13185-RB	Hexo1	0,0307616	0,0280198	0,029533	0,0338684	14,7063	13,7788	-0,101824	0,703482	0,6279808
CG13185-RC	Hexo1	0,0329391	0,0300032	0,0316235	0,036535	7,90416	0,0345741	-0,104285	0,696676	0,13772387
CG13186-RA	Hexo1	7,31416	6,98907	10,8643	12,8402	13,8041	0,0372963	0,269399	0,435974	0,6279808
CG13188-RA	reb	22,3496	22,4029	21,9456	18,7847	15,1049	14,5154	0,325106	0,185748	0,6279808
CG13188-RB	CG13185	0,774082	0,303455	0,931308	1,47183	1,12243	0,681471	0,393539	0,096109	0,6279808
CG13189-RA	CG13185	0,809218	0,647521	1,12503	0,513346	0,00558602	0,558693	-0,234694	0,34766	0,6279808
CG1318-RA	CG13186	0,380232	0,577236	0,68953	0,274721	0,0913966	0,63252	-0,280458	0,168926	0,6279808
CG1318-RB	exp	0,0171314	16,8448	0,0563548	46,1626	0	48,5564	-0,280349	0,169183	0,13772387
CG1318-RC	exp	86,2039	1,88675	34,9333	0,187718	1,99925	0	-0,227948	0,268948	0,6279808
CG1318-RD	Zip48C	9,5724	8,41523	14,8559	12,6689	1,23497	11,1325	-0,280458	0,168926	0,6279808
CG13190-RA	Fdx2	51,2593	40,2834	70,5492	174,02	60,1387	105,891	0,318448	0,252373	0,6279808
CG13192-RA	cuff	0,0509452	0,0464044	0,0489104	0,0602014	0,0815432	0,0614559	-0,147815	0,612254	0,6279808
CG13193-RA	CG13192	7,06985	6,03722	18,8898	6,57389	0,144812	2,2899	0,047993	0,769145	0,6279808
CG13194-RB	CG13193	0	0,158385	0	0	0	0,296302	0,103456	0,598351	0,6279808
CG13195-RA	pyr	13,3365	13,6464	14,9362	27,2099	2,45315	16,8763	-0,188748	0,503052	0,6279808
CG13196-RA	Ir48b	0,0549716	0,100144	0,052776	12,4214	0,134735	0,168148	0,543006	0,061191	0,6279808
CG13197-RA	CG13196	7,06918	6,90954	6,97278	7,70897	2,37365	3,3393	0,270469	0,180251	0,6279808
CG13198-RA	CG13197	11,9925	12,8429	13,3117	0,0617533	9,66676	29,9795	0,092803	0,764895	0,6279808
CG1319-RA	CG13198	0,18253	3,05402	3,12411	0,242951	0,0601614	9,94568	0,046368	0,866916	0,6279808
CG13201-RA	mRpL23	56,7143	62,1016	10,982	91,8167	130,047	97,8586	0,263985	0,307767	0,6279808
CG13202-RA	mRpL23	0,115584	0,105282	51,3077	0,155949	0,211234	0,159199	-0,070444	0,835639	0,6279808
CG13203-RA	ix	12,2363	13,8524	140,774	20,9471	157,649	15,1004	-0,034934	0,908829	0,6279808
CG13203-RB	CG13202	0,248715	1,6991	0,560486	0,555012	9,5508	0,566577	0,0221	0,943601	0,6279808
CG13204-RA	CG13203	0,518265	1,09065	0,909344	1,47141	0,0636359	1,06345	0,381098	0,119099	0,6279808
CG13204-RB	CG13203	0,702632	1,20244	1,41046	1,47835	6,22801	0,31333	0,40669	0,091721	0,6279808
CG13206-RA	CG13204	5,55365	6,71501	47,4493	0,0412204	2,28511	3,54602	-0,131419	0,365453	0,6279808
CG13207-RA	CG13204	1,03972	3,09943	0,198861	161,481	1,23845	1,83734	0,048378	0,863099	0,6279808
CG13207-RB	Or47b	0	0,0810047	5,95805	0,152343	0,0189914	0,0143131	0,04787	0,864636	0,6279808
CG13207-RC	nompA	1,79436	0,100311	38,4866	180,628	181,202	1,70754	0,054964	0,845648	0,6279808
CG13207-RD	nompA	5,23628	150,418	19,7386	13,4922	34,9146	10,3271	0,055482	0,844084	0,6279808
CG13208-RA	nompA	0,0130361	1,08249	9,68088	0,0136125	13,6481	25,4112	-0,066578	0,79933	0,6279808
CG13209-RA	nompA	2,46795	2,4512	84,9959	0,0137891	15,9285	140,196	-0,179154	0,613717	0,6279808
CG13209-RB	Obp47b	0,110126	0,012413	97,1856	7,35715	1,83889	34,6828	-0,179154	0,613717	0,6279808
CG1320-RA	sha	0,0136276	25,083	0,0853793	24,0615	80,4904	202,398	-0,442782	0,10204	0,6279808
CG1320-RB	sha	4,49615	13,2608	0,10906	33,2053	0,0184382	4,03776	-0,443151	0,101859	0,6279808
CG13213-RA	Fbl6	0,0179749	0,0163728	0,017257	8,45063	0	4,31002	0,023901	0,91931	0,6279808
CG13213-RB	Fbl6	3,08591	3,60113	5,0545	5,01731	0,0256946	4,42901	0,029371	0,900733	0,6279808
CG13213-RC	Fbl6	9,45324	9,46291	5,55485	0,0606447	5,49199	0,0619084	0,061598	0,795287	0,6279808
CG13214-RA	Cpr47Ef	0,742564	0,777835	1,01589	0,0303575	0,455232	0,531066	0,245203	0,441854	0,6279808
CG13214-RB	Cpr47Ef	0,466197	1,14654	0,0895154	0,0356264	0,159513	0,358931	0,123882	0,726615	0,6279808
CG13215-RA	CG13215	3,38851	1,45187	3,48611	0,0203274	17,2581	4,15025	-0,226569	0,515438	0,6279808
CG13216-RA	CG13216	10,2313	3,51517	11,1927	0,0212861	0,0101105	7,2411	0,768181	0,005585	0,6279808
CG13217-RA	CG13217	247,822	0	7,66587	0,0188342	16,3261	12,9768	-0,26988	0,373334	0,6279808
CG13218-RA	CG13218	38,5068	0,029595	77,0304	0,223942	0,0473599	0,0356933	-0,422725	0,09969	0,6279808
CG13220-RA	zfh1	4,6	6,80105	3,88315	24,7127	0,0719461	2,66097	-0,43997	0,144497	0,6279808
CG13221-RA	zfh1	2,9395	2,74615	1,5015	9,41491	376,859	2,22159	0,116169	0,667545	0,6279808
CG13222-RA	CG13220	69,3556	637,28	9,80876	93,6483	13,9091	11,2286	-0,183441	0,606102	0,6279808
CG13223-RA	Dsim\GD10785	12,2181	20,1954	5,7172	15,4879	2,78632	3,31353	0,034174	0,868648	0,6279808
CG13224-RA	Cpr47Ee	0,356012	0,129712	0,615228	0,0251733	0,40888	632,892	0,29338	0,383651	0,6279808
CG13225-RA	CG13223	0	0	4,96601	63,6497	0	3,51081	0,444522	0,21194	0,6279808
CG13226-RA	Cpr47Eb	28,2721	0,0565662	0,413	0,0263406	26,0443	1,48112	-0,624053	0,080506	0,6279808
CG13227-RA	Or47a	0,485495	1,77019	1,39934	0,020479	0,757928	16,3364	-1,059145	0,000441	0,6279808
CG13228-RA	CG13226	1,48069	1,78993	12,3945	0,0339382	4,81469	0,316402	-1,190819	0,000578	0,6279808
CG13229-RA	CG13227	63,8402	0,00608682	75,838	0,559781	322,048	172,971	-0,5249	0,082249	0,6279808
CG1322-RA	CG13228	7,36876	17,8837	18,3469	0,0251926	9,28475	12,5919	-0,08326	0,786079	0,6279808
CG1322-RB	CG13229	2,41896	2,58991	3,9317	0,748505	2,49781	1,66011	-0,108519	0,724012	0,6279808
CG13230-RA	CG13230	0,754291	0	6,08298	10,8059	3,38226	8,0484	-0,263753	0,453933	0,6279808
CG13231-RA	CG13231	0,763253	0	10,2035	0,467998	0,229208	4,3058	0,320385	0,187144	0,6279808
CG13232-RA	BBS4	0,859303	1,54483	1,54141	2,25485	8,392	0,506534	1,189931	0,000422	0,6279808
CG13235-RA	CG13235	0,589963	14,9369	1,15149	39,9646	5,82007	0	0,022403	0,887437	0,6279808
CG13239-RA	CG13239	58,6621	2,93502	60,7418	30,7002	191,597	103,924	-0,022666	0,929902	0,6279808
CG13240-RA	CG1324	0,143145	5,35774	0	0	3,63605	0,228479	-0,399852	0,161712	0,6279808
CG13240-RB	Dpse\GA12146	95,4113	2,00422	1,74169	2,03923	1,45934	0,795994	-0,479321	0,0959	0,13772387
CG13243-RA	Dpse\GA12146	0,126179	0,107861	0,113686	0,0690676	0	0	-0,224859	0,221879	0,6279808
CG13244-RA	CG13243	0	0	0	0,0649486	1,86895	0	0,01562	0,897983	0,6279808
CG13245-RA	CG13244	0	0	0,155737	0,340428	0	0	0,039221	0,912727	0,6279808
CG13247-RA	CG13245	0,561324	1,24171	0,731371	0,0691761	0,980566	0,433009	0,350926	0,151927	0,6279808
CG13248-RA	CG13247	10,4538	21,2728	18,6566	15,5278	5,81914	5,61893	-0,164915	0,495847	0,6279808
CG1324-RA	CG13248	3,33728	3,43953	0,0364858	4,12307	3,76663	2,64876	-0,044061	0,853798	0,6279808
CG13250-RA	CG13250	0,171181	9,59391	2,32038	24,9579	0,948392	1,23427	-0,21976	0,49451	0,6279808
CG13251-RA	CG13251	1,98207	0,311847	0,438251	11,7269	20,9783	14,4776	0,457547	0,107542	0,13772387
CG13252-RA	CG13252	24,2088	0,920773	24,8204	0,570479	48,994	0,555531	0,314023	0,090166	0,6279808
CG13253-RB	cmpy	6,51983	0,0622618	6,81451	20,4481	41,0777	0,0395321	0,208678	0,36003	0,13772387
CG13253-RC	cmpy	7,19282	7,02561	9,46113	20,9014	15,7648	86,5884	0,048348	0,834695	0,6279808

gene_id	Symbol	W1_FPKM	W2_FPKM	W3_FPKM	MCM51_FPKM	MCM52_FPKM	MCM53_FPKM	FC	p-value	p-adj
CG13255-RA	CG13255	18,1886	5,71413	0	7,30246	30,4848	29,6952	-0,368101	0,204711	0,6279808
CG13258-RA	CG13258	1,28929	0,504074	0,371907	56,8529	8,90372	13,5109	-0,556562	0,091766	0,6279808
CG13263-RA	Dpse\GA12159	44,1887	45,3972	50,3444	55,6169	29,5265	33,5718	0,242903	0,212997	0,6279808
CG13270-RA	Ccp84Ac	176,528	251,56	175,326	180,532	52,1731	82,8944	0,429803	0,026603	0,13772387
CG13271-RA	Ugt37C2	15,0268	17,6501	59,038	49,6443	97,0257	11,3736	1,178284	0,000987	0,13772387
CG13272-RA	Ugt37E1	0,611814	0,798771	19,1884	15,3011	12,8478	11,3974	0,428859	0,207244	0,6279808
CG13277-RA	CG13272	7,85109	8,6934	0,0277906	8,20869	5,82934	20,5144	-0,412066	0,08818	0,6279808
CG13278-RA	LSm7	43,4984	38,8825	4,85929	67,5998	12,7137	2,86038	0,488019	0,08663	0,6279808
CG13279-RA	ppk17	2,47597	1,98192	28,2476	48,4646	3,41326	4,65255	-0,999402	0,003873	0,6279808
CG1327-RA	Cyt-b5-r	23,9643	12,2541	7,47606	0,348628	0,152129	0	1,034302	0,000446	0,6279808
CG13280-RA	CG13280	0,304217	0,960619	0,0275134	10,715	1,92295	2,4942	0,539542	0,129595	0,6279808
CG13281-RA	Cse1	1,8445	1,86574	0	0	0	0	-0,243424	0,361948	0,6279808
CG13282-RA	CG13282	1,68028	1,36505	1,61317	1,62891	0,704498	6,07744	0,623503	0,041794	0,6279808
CG13283-RA	Nepl9	0,269835	10,9388	0,518116	17,8991	42,2458	7,1357	-0,260893	0,461101	0,6279808
CG13284-RA	CG13284	14,4805	2,56825	8,062	1,97061	1,57032	3,11754	0,016152	0,946141	0,6279808
CG13284-RB	CG13284	35,4099	2,40227	0,331008	14,4861	0,0485977	10,7944	0,025214	0,915203	0,6279808
CG13285-RA	CG13285	61,206	59,2287	73,0533	94,7633	224,66	155,619	-0,820374	0,008684	0,6279808
CG13287-RA	CG13287	1,90695	0	0,0177512	0,0170858	0,977212	0,0676018	0,352303	0,251297	0,6279808
CG13288-RB	CG13288	0,0584742	0,020315	0,0747723	56,6052	23,8917	0	-0,648365	0,069431	0,13772387
CG13293-RA	CG13293	2,62623	8,89937	1,99944	1,65227	1,807	1,20118	-0,237432	0,375218	0,6279808
CG13295-RA	CG13295	6,7929	2,83868	110,33	2,35069	2,65745	10,8232	-0,244571	0,371868	0,6279808
CG13296-RA	Prdm13	0,661704	0,208981	0,26432	0,307633	0,509771	0,496058	0,088045	0,80045	0,6279808
CG13297-RA	CG13297	125,128	109,267	0,657964	1,12861	40,3864	46,6349	0,475102	0,04581	0,6279808
CG13298-RA	Sf3b6	49,0435	280,453	60,8531	0,0232216	1,02257	1,97472	-0,062358	0,757769	0,6279808
CG13299-RA	CG13299	0	0,266312	0	0	4,57702	0,732861	-0,146035	0,52028	0,6279808
CG13300-RA	Ccp84Ae	134,791	405,848	270,388	263,208	236,821	236,619	-0,115987	0,694252	0,6279808
CG13305-RB	CG13300	6,39809	6,93666	5,05537	11,9429	4,74039	37,6187	-0,411285	0,155481	0,6279808
CG13305-RC	CG13305	3,68021	4,1558	82,9925	11,0153	4,59467	0,0719008	-0,33758	0,248788	0,6279808
CG13306-RA	CG13305	1,1879	3,52351	2,34742	0,0704332	0,0954021	48,3914	0,02145	0,949136	0,6279808
CG13308-RA	CG13306	3,90505	3,55699	3,92761	3,25346	2,40525	417,384	0,607321	0,075021	0,6279808
CG13309-RA	Dsim\GD14130	0,782319	0,233961	0,13216	12,2313	12,6101	0,469017	0,155764	0,566822	0,6279808
CG1330-RA	CG13309	0	0,688283	0,201515	77,2413	0,0585434	19,2269	0,075823	0,797279	0,6279808
CG13310-RA	Ccp84Af	7,24516	6,20343	12,6595	1,59732	0,850606	1,25658	0,383934	0,209659	0,6279808
CG13311-RA	CG13310	1,75326	0	1,65693	1,5832	10,5758	4,18586	-0,827233	0,013053	0,6279808
CG13312-RA	CG13311	43,5077	1,22269	0	0	0,533699	0	-0,186478	0,601615	0,6279808
CG13313-RA	CG13312	1,70166	2,85036	0,577749	0,364342	0,251667	0,172615	0,754428	0,019066	0,6279808
CG13314-RA	CG13313	2,75167	0,0262976	0,0277178	0,720421	1,26593	1,26716	0,253808	0,359468	0,6279808
CG13315-RA	CG13314	29,5694	36,728	30,8026	40,1644	14,3963	19,4162	-0,441972	0,124227	0,6279808
CG13316-RA	CG13315	258,132	247,627	0,0266578	17,814	10,8877	9,95304	0,033058	0,906264	0,6279808
CG13316-RB	Mnt	6,05514	1,99035	3,45459	7,89617	3,22978	2,92789	0,089658	0,749801	0,6279808
CG13316-RC	Mnt	0,0276391	6,24053	4,68572	4,27474	1,46397	1,84107	0,054226	0,8464	0,6279808
CG13317-RA	Mnt	5,92002	1,63641	1,97687	10,1266	3,75318	4,2535	-0,158679	0,65667	0,6279808
CG13318-RA	Ilp7	0,963607	3864,04	2681,65	2,25018	2,19602	1,14792	-0,200557	0,423443	0,6279808
CG13319-RA	CG13318	8,72306	8,51651	10,0295	46,4471	119,173	10,3997	-0,06023	0,819185	0,6279808
CG1331-RA	CG13319	11,2836	10,4085	8,00981	0,0400187	7,58844	8,25202	1,839609	8,31E-08	0,6279808
CG13320-RA	CG43367	6,51553	0,0639147	0,0390941	0,0447172	12,6995	0,045649	0,195798	0,417851	0,6279808
CG13320-RB	Sans	7,80776	9,93257	6,2728	4,40312	28,6235	6,04095	0,195798	0,417851	0,6279808
CG13321-RA	Sans	6,40136	30,3408	0,654952	1,92124	157,133	36,7644	-0,465571	0,09629	0,6279808
CG13322-RA	CG13321	195,117	12,8242	0,0342531	3,6743	2,41016	0,0391754	0,314206	0,182062	0,6279808
CG13322-RB	SCCRO3	10,7298	10,4485	11,1056	12,3594	0,0459222	0,0367462	0,314206	0,182062	0,6279808
CG13322-RC	SCCRO3	0,0330931	0,0301435	0,0317713	0,0356649	7,5724	4,04793	0,314206	0,182062	0,6279808
CG13323-RA	SCCRO3	0,0333772	0,0304023	0,0320441	0,0359961	31,9142	7,91576	-0,164459	0,621718	0,6279808
CG13324-RA	CG13323	81,7035	1,10667	3,06429	28,9446	252,847	11,0117	0,762984	0,032535	0,6279808
CG13325-RA	CG13324	14,0602	16,7786	1,68813	0,0826882	1,37948	1,86091	-0,562395	0,115775	0,6279808
CG13326-RA	CG13325	0,46199	4,73636	10,7627	12,2733	1,56314	1,60035	0,053284	0,8294	0,6279808
CG13329-RA	CG13326	0,0356781	0,0866616	243,422	2,64495	11,387	178,05	0,213966	0,51406	0,6279808
CG1332-RA	cid	1,50756	7,70827	2,09403	9,38106	23,2216	0,0145264	0,404768	0,083806	0,6279808
CG13330-RA	Ero1L	31,9703	43,2482	7,20634	49,5708	63,7329	0,327592	-0,466071	0,070264	0,6279808
CG13331-RA	CG13330	0,0311238	10,5187	0,0298807	0,606453	1,26997	3,6111	-0,27193	0,362223	0,6279808
CG13331-RB	CG13331	0,0533012	0	0,639655	0,511063	6,02223	0,160029	-0,262266	0,37808	0,6279808
CG13332-RA	CG13331	2,05974	0,19415	1,94796	17,674	0,42975	12,5723	-0,502458	0,16033	0,6279808
CG13333-RA	CG13332	1,24086	0	1,86141	0,0288679	0,0715333	4,67023	-0,626642	0,058289	0,6279808
CG13334-RA	link	4,69367	0,0129387	0,0237502	0,0266928	2,11428	0,0283755	-0,171732	0,470745	0,6279808
CG13335-RA	CG13334	0,105823	0,0122264	0,0231521	10,2772	0,0220072	0,0287392	-0,089238	0,780821	0,6279808
CG13335-RB	CG42808	7,17988	0,0247511	0,04588	0,0343093	1,85269	0	-0,015495	0,961014	0,6279808
CG13337-RA	CG42808	3,76892	0,025388	12,7284	0,822643	4,19575	0,61909	1,233211	0,00025	0,6279808
CG13338-RA	CG13337	2,17592	9,00671	1,84179	0,480239	0,162209	0,49817	-0,155424	0,585371	0,6279808
CG13339-RA	Cpr50Ca	0,0481603	8,63715	0,0352884	0,081682	0,0541399	0,0408031	-0,15757	0,592195	0,6279808
CG1333-RA	CG13339	19,9376	23,3482	17,2372	9,04106	3,3061	26,737	0,378486	0,2389	0,6279808
CG13340-RA	Dsim\GD25742	0	0,0336676	0,0381536	0	0,0589689	0,0444425	NA	NA	0,6279808
CG13343-RA	Uba3	3,79515	10,6449	4,31008	4,2082	5,18394	58,0973	-0,154218	0,591218	0,6279808
CG13344-RA	CG13344	7,31636	3,70983	66,0521	10,0546	57,4274	0,0755341	0,076751	0,715477	0,6279808
CG13345-RA	tum	4,28885	4,42325	9,93524	9,18092	21,6082	5,80334	-0,14345	0,620175	0,6279808
CG13348-RA	PheRS-m	38,6368	54,014	48,1642	3,67532	1,35262	1,89316	-0,420225	0,051819	0,6279808
CG13349-RA	Rpn13	0,0488125	0,0444618	0,0468629	0,0547036	0,0740964	0,0558435	-0,11012	0,618496	0,6279808
CG13349-RB	Rpn13	6,26607	11,9984	6,36709	13,2598	8,42603	6,95047	-0,110379	0,617759	0,6279808
CG13349-RD	Rpn13	19,2358	21,1603	36,4378	28,3587	22,2758	27,7993	-0,113199	0,607254	0,6279808
CG13349-RE	Rpn13	0,0457061	0,0416323	0,0438806	0,0508219	0,0688386	0,0518809	-0,113456	0,606528	0,6279808
CG13349-RF	Rpn13	0,0492858	0,0448929	0,0473173	0,0553005	0,0749049	0,0564528	-0,11012	0,618496	0,6279808
CG13350-RA	Dsim\GD25724	2,17774	2,71904	2,81488	6,99878	2,7848	62,2507	0,086277	0,755032	0,6279808
CG13358-RB	CG13358	0,375103	8,16924	0,249315	12,4441	7618,18	0,178175	-0,248261	0,468807	0,6279808
CG13359-RA	CG13359	0,109943	12,0108	0,052776	0,57525	5,34274	7,01674	-0,265196	0,41548	0,6279808
CG13360-RA	CG13360	0,0424743	0	9,57985	0,0468473	0,218473	6972,88	0,43887	0,180187	0,6279808
CG13361-RA	CG13361	0,253326	0,307663	0,405347	0,551719	0	0,197519	0,166784	0,62118	0,13772387
CG13362-RA	CG13362	33,2272	29,1755	96,4672	47,4744	0,137622	124,833	0,160102	0,609579	0,6279808
CG13363-RA	Hmt4-20	10,0579	12,7509	8,94229	17,4162	5,27631	6,43993	0,072183	0,804163	0,6279808
CG13364-RA	CG13364	568,303	477,151	520,761	986,068	1061,26	1053,09	-0,350272	0,160938	0,6279808
CG13365-RA	CG13365	34,108	29,3622	55,5393	23,4844	27,7867	73,9868	-0,234778	0,432057	0,6279808
CG13366-RA	CG13366	4,36168	3,50061	6,33977	0,502541	0,403715	0,0594463	0,1519	0,555026	0,6279808
CG13366-RB	CG13366	6,9959	7,78529	5,33149	0,771815	0,393656	0,0600835	0,149033	0,560561	0,6279808
CG13367-RA	CG13367	8,86696	10,7753	13,4854	13,353	8,49784	10,877	-0,031468	0,889716	0,6279808
CG13369-RA	CG13369	0,056187	0,051179	20,8759	0,0676839	26,5547	26,4283	-0,39799	0,146615	0,6279808
CG13373-RA	CG13373	7,70944	0,728964	10,7562	0,170034	2,27883	2,64596	-0,048198	0,884152	0,6279808
CG13373-RB	CG13373	0,123122	9,30832	0,118205	11,7101	4,32958	5,79632	-0,152617	0,64385	0,6279808
CG13374-RA	Pgcl	12,7946	3,46235	5,27481	0,681882	0,561701	0,0379272	-0,880876	0,008986	0,6279808
CG13375-RA	CG13375	0,998787	0,0416611	1,6257	0,0582328	0,0599191	1,53634	-0,216716	0,473186	0,6279808
CG13375-RB	CG13375	2,33263	0,651737	0,860235	0,058857	0,0604057	0,888488	-0,222015	0,463661	0,6279808
CG13376-RA	CG13376	1,13532	1,49949	1,85296	33,2147	1,11377	1,56262	0,043825	0,899718	0,6279808
CG13377-RA	CG13377	52,5735	50,315	54,6287	55,9212	49,4566	42,0234	0,060082	0,764684	0,6279808
CG13379-RB	Sgf11	8,40246	15,3691	17,4246	24,5141	8,78382	8,70109	0,337778	0,245662	0,6279808
CG13383-RB	hydra	0,142541	0,0416804	0,0439313	0,144215	0,061808	0,0541793	-0,31094	0,255279	0,6279808
CG13384-RB	hydra	0,0468733	0,0411391	0,0433608	0,0522734	0,0610053	0,0533626	-0,181923	0,426812	0,6279808
CG13384-RC	Pp2A-29B	38,553	28,8493	31,7184	46,2366	61,7278	43,0656	-0,187044	0,408006	0,6279808
CG13384-RD	CG13384	0,027338	0,0229136	0,024151	0,0265958	0,0403025	0,0303744	-0,184384	0,413479	0,6279808

gene_id	Symbol	W1_FPKM	W2_FPKM	W3_FPKM	MCM51_FPKM	MCM52_FPKM	MCM53_FPKM	FC	p-value	p-adj
CG13384-RE	CG13384	0,0252006	0,02439	0,0257072	0,0284197	0,0368878	0,0278008	-0,185026	0,412114	0,6279808
CG13384-RF	CG13384	0,0246552	0,0244197	0,0257385	9,16778	0,0360242	0,02715	-0,185026	0,412114	0,6279808
CG13384-RG	CG13384	0,0262102	15,3496	13,7438	12,7118	0,0384947	0,0290119	-0,184787	0,412889	0,6279808
CG13385-RA	CG13384	5,34013	46,0867	49,9024	25,05	0,0385445	0,0290495	-0,148884	0,511815	0,6279808
CG13386-RA	CG13384	10,2557	0,0253204	0,0266878	0,0277205	0,0434835	0,0327718	-0,437796	0,198642	0,6279808
CG13387-RA	CG13385	0	1,0559	2,04036	1,94806	3,5223	79,4223	-0,409834	0,076689	0,6279808
CG13388-RA	CG13386	1,46835	1,86161	3,05608	2,43484	0,691072	2,53229	0,156561	0,560534	0,6279808
CG13388-RB	emb	12,8252	0,023431	0,0246964	0,0272334	0,0957864	0,15697	0,119484	0,655963	0,6279808
CG13388-RC	Akap200	0,0199947	12,5029	0,0191961	1,13304	6,30027	9,69414	0,11474	0,669053	0,6279808
CG13388-RD	Akap200	40,3085	38,3189	42,5342	0,204412	12,2849	21,7624	0,151187	0,574427	0,6279808
CG13389-RA	Akap200	47,3441	58,4739	60,2988	3,6536	32,7505	55,3259	-0,047656	0,841701	0,6279808
CG13389-RB	Akap200	48,7221	67,3048	62,2463	8,04567	21,3059	38,8997	-0,047678	0,841629	0,6279808
CG1338-RA	RpS13	1746,4	7,70244	14,5376	131,923	16,6438	27,047	-0,072427	0,694472	0,6279808
CG1338-RB	RpS13	0,114384	0,0772612	0,0195994	0,0213413	0,154809	6,32917	-0,072427	0,694472	0,6279808
CG13390-RA	CG1339	0,332168	0,558575	3,35758	3,72441	0,043168	0,0309047	0,018271	0,942578	0,6279808
CG13391-RA	CG13390	6,6209	33,3757	9,94566	8,03567	6,84737	9,20764	0,01923	0,936406	0,6279808
CG13391-RB	AlaRS	0,0204148	0,0185952	10,3014	52,0994	0,0289069	0,021786	0,019268	0,936335	0,6279808
CG13392-RA	AlaRS	24,7011	28,7752	115,187	25,2837	15,6821	21,0025	-0,093187	0,760003	0,6279808
CG13393-RA	CG13392	7,17789	82,2531	26,8683	39,2093	74,5503	90,2672	0,314317	0,153147	0,6279808
CG13394-RA	Dad1	91,2182	0	0	40,6994	0	0,198413	-0,617901	0,069069	0,6279808
CG13394-RB	Dpse\GA25454	26,8868	2,04843	11,8898	2667,38	102,34	85,9159	0,019492	0,888588	0,6279808
CG13396-RA	Dpse\GA25454	0,028436	14,1007	2,44365	0,153765	0,0410438	0,0282982	-0,002214	0,993664	0,6279808
CG13397-RA	fy	2,56828	11,1928	15,7462	7,79587	4,6947	4,91108	-0,454876	0,147246	0,6279808
CG13398-RA	CG13397	2,97933	9,01817	7,17481	25,4664	11,4374	13,8385	0,391477	0,08231	0,6279808
CG13399-RB	CG13398	8,21465	0,0254632	0,0268383	0,0297544	1,49282	2917,14	0,077985	0,811605	0,6279808
CG1339-RA	Chrac-14	7,13575	7,3186	2,2109	1,92654	8,92777	1,20522	0,342819	0,332494	0,6279808
CG13400-RA	eIF4H2	0,158921	4,07908	0,0226022	67,8835	2,36503	0	-0,055099	0,831583	0,6279808
CG13401-RA	D12	2,04443	2133,76	2875,37	0,207361	5,80935	1,66088	0,658152	0,007333	0,6279808
CG13401-RB	Aasdh	2,83354	2,1341	0,0163933	0,664843	2509,6	0,0180794	0,658152	0,007333	0,6279808
CG13402-RA	Aasdh	0,0199585	0,0181796	1,86842	1,04207	0,208276	0,944487	0,242761	0,261722	0,6279808
CG13403-RA	betaNACtes1	0	0,355292	0,337031	0,243894	0	0	0,400452	0,142818	0,6279808
CG13404-RB	CG13403	5,95653	7,1921	626,902	41,1975	6,65744	6,41786	0,171652	0,468946	0,6279808
CG13405-RA	CG13404	6,36855	1,753	0,126098	9,27954	0,0650277	0,0280118	0,103009	0,606988	0,6279808
CG13407-RA	CG13405	0,207264	0,601865	0,119392	0	0,225183	0	-0,202304	0,422501	0,6279808
CG13408-RA	CG13407	0,0742426	0,112709	0	0,232782	0,195075	0,0890907	0,046778	0,887445	0,6279808
CG13409-RA	CG13408	1,62202	0,0545167	1,55723	1,80898	20,377	0	0,116717	0,580221	0,6279808
CG13409-RB	CG13409	7,2744	0,0292926	7,42035	8,51242	5,94924	5,07726	0,120917	0,565139	0,13772387
CG1340-RA	CG13409	0,0322531	0,0270177	0,0309649	0,0346886	0,0469859	0,0354114	0,180377	0,483747	0,6279808
CG13410-RA	Rpt1	39,2612	0,0163684	60,8163	53,2728	38,4634	4,72227	-0,040919	0,893014	0,6279808
CG13415-RB	mRpL35	39,4049	0,0324864	95,3688	57,9348	105,662	112,713	-0,078907	0,814702	0,6279808
CG13417-RA	Cby	0,127395	0,0413369	0,122307	0,0274718	0	0,182032	-0,163623	0,621649	0,6279808
CG13418-RA	Gr93a	0,136709	0,236334	0,293689	0,429194	0,228555	0,163914	0,094498	0,769841	0,6279808
CG13419-RA	Rpl12	5,55777	4,52548	96,9668	0,0174616	6,0044	5,28019	-0,331661	0,296136	0,6279808
CG1341-RA	Burs	4,9367	2,44578	0,0741371	3,82424	0,126324	0,0952056	-0,100264	0,669669	0,6279808
CG13421-RA	Spn100A	26,4985	0,0890333	0,0238245	3,18913	0,0707485	0,141631	-0,013096	0,914398	0,6279808
CG13421-RB	Obp57c	0,280458	6,13239	0	1,28003	0,0306754	3,15251	-0,175744	0,618489	0,6279808
CG13422-RA	Obp57c	0,0622378	0,0118813	0,02074	98,5464	0,024802	2,87742	0,332832	0,285442	0,13772387
CG13423-RA	GNBP-like3	0,396455	0,0196773	0,020583	0,0134021	1,27295	0,0737131	-0,148962	0,607534	0,6279808
CG13424-RA	CG13423	0,162748	0,0151349	40,6256	6,18043	0,0233181	23,3592	0,401202	0,10164	0,6279808
CG13425-RA	lms	4,40579	0,0426652	3,86409	0,0228829	0,0601209	3,06885	0,002224	0,991752	0,13772387
CG13425-RB	HnRNP-K	0,0318335	0,0283943	1,27592	5,68524	5,88388	0,0341355	0,002255	0,991638	0,6279808
CG13425-RC	HnRNP-K	49,0298	0,0463448	0,0414168	0,0342024	6,91029	0,0585154	0,002243	0,991682	0,6279808
CG13425-RD	HnRNP-K	0,0311727	1,74297	11,4278	75,4913	0,0463274	1,17794	-0,048235	0,826154	0,6279808
CG13426-RA	HnRNP-K	0,0508797	2,01841	0,0305621	0,0334387	38,4315	0,0486537	-0,037875	0,76416	0,6279808
CG13427-RA	CG13426	0	0	9,64792	4,90822	29,16	0,114202	-0,573442	0,100121	0,6279808
CG13428-RA	CG13427	14,4824	21,6469	17,3673	12,2219	5,0871	17,0532	-0,344763	0,324699	0,6279808
CG1342-RA	CG13428	0,957622	0,0195284	0,463042	0,0434492	0,030995	0,017574	-0,231735	0,47037	0,6279808
CG13430-RA	Sp1	2,66799	2,5831	0,0214482	6,00749	0,032226	64,7979	-0,576596	0,078808	0,6279808
CG13430-RB	Sp1	2,96624	0,0206212	12,4885	0,0237917	0,0313433	0,0242875	-0,576596	0,078808	0,6279808
CG13431-RA	CG13430	0,0404463	4,08853	10,2333	0	12,2451	12,3151	0,31553	0,214329	0,6279808
CG13432-RA	CG13430	18,7779	4,28867	48,5053	47,0155	52,997	48,5287	-0,177001	0,451637	0,6279808
CG13434-RA	Mgat1	4,56492	47,3529	4,8874	66,0074	39,1993	2,66897	-0,35485	0,181606	0,13772387
CG13436-RA	qsm	42,7925	49,0593	3559,88	3750,66	4,61797	2,09064	0,017062	0,954735	0,6279808
CG13437-RA	Nnf1a	8,33526	6,39596	0,106676	0,147898	8,39956	11,1152	-0,172857	0,583784	0,6279808
CG13438-RA	CG13436	1,20546	49,824	1,53174	21,9462	0,0645565	48,7972	-0,100552	0,445078	0,6279808
CG13439-RA	Dpse\GA12287	1,54177	2,8087	3,3833	18,5079	18,9451	0	0,687369	0,002254	0,6279808
CG1343-RB	CG13438	0	0	0,143522	0,439141	0,183711	10,287	0,262475	0,3407	0,6279808
CG1343-RD	dpr1	10,1679	0,208981	0	0	0	0	0,220721	0,4227	0,6279808
CG13441-RA	CG1344	17,353	17,7254	18,3877	19,2273	2,13912	2178,86	-0,041811	0,731452	0,6279808
CG13442-RA	Gr57a	0	0	0	0,767191	38,6486	6,85222	NA	NA	0,6279808
CG13443-RA	CG13442	0	11,0115	9,00634	1,18037	3,01493	2,6474	-0,655558	0,027965	0,13772387
CG13445-RA	CG13443	0,0756017	0	0,384645	0,896525	0	0,0994931	0,076209	0,828201	0,6279808
CG13449-RA	CG13445	2,03938	1,32942	0,382149	11,8877	2,33749	2,97032	0,309356	0,362465	0,6279808
CG1344-RA	CG13449	0,703329	0	0	0,228149	0,186623	0,484918	0,147984	0,423193	0,6279808
CG13454-RA	Gfat2	25,5824	32,479	32,9881	0,200246	0,202766	0,064832	-0,049748	0,88711	0,6279808
CG13455-RA	CG13454	1,90604	148,285	5,55072	4,06316	23,6034	17,0715	0,192732	0,580692	0,6279808
CG13457-RA	CG13455	0,502305	3,73116	15,4439	11,1867	0,122509	106,074	0,518806	0,136105	0,6279808
CG13457-RB	CG13457	0,0204148	0,0185952	0,0195994	0,0213413	0,0289069	0,021786	0,518806	0,136105	0,6279808
CG13458-RA	CG13457	0,0204781	0,0186528	0,0196602	0,0214106	0,0290008	0,0218568	0,187534	0,597666	0,6279808
CG1345-RA	CG13458	0,619084	0,316337	20,802	0,378606	0,567418	0,613804	0,171561	0,375601	0,6279808
CG13460-RB	CG13460	0,974571	38,3773	5,64618	0	6,33022	5,2717	0,269995	0,442669	0,6279808
CG13461-RA	CG13461	15,5614	10,4481	0	17,7508	0	18,6162	-0,525258	0,10757	0,6279808
CG13463-RA	EAChm	701,881	6,65806	23,3098	63,2093	16,4589	16,3735	-0,73413	0,020839	0,13772387
CG13465-RA	CG13465	9,06104	5,84088	21,2794	69,7302	24,5028	21,5192	-0,146548	0,677771	0,6279808
CG13471-RA	Atg17	8,0851	0,0124851	7,3349	10,9108	1,37306	12,6893	0,063688	0,754971	0,6279808
CG13472-RA	Atg17	0,0137068	1,67185	1,70529	0,014105	6,33517	14,8249	-0,139296	0,558736	0,13772387
CG13473-RA	CG13471	0,106574	0,00960882	0,102317	0,0107763	0	161,595	-0,037875	0,76416	0,13772387
CG13474-RA	Tdrd3	5,8773	6,05965	7,4914	10,4795	4,61481	5,62029	0,093085	0,581996	0,6279808
CG13475-RA	CG13473	0	9,03786	0	14,2854	0	2,08812	0,277045	0,230765	0,6279808
CG13475-RB	CG13474	0	0,242036	0,171142	0,731869	0	26,3336	0,294845	0,212868	0,6279808
CG13476-RA	HGTX	1,37857	0,0328588	0,588767	2,10773	1,12312	1,02042	0,099493	0,503366	0,6279808
CG13476-RB	HGTX	6,86277	0,666762	0,983878	6,35984	0,597811	4,35848	0,099493	0,503366	0,6279808
CG13477-RA	CG13476	0,106288	0,214952	0,255107	1,32901	0	4,85827	-0,041811	0,731452	0,6279808
CG13478-RA	CG13476	0,323477	0	0,112477	125,999	0	0,884193	-0,218646	0,497412	0,6279808
CG13478-RB	goddard	0	0,0902346	0	0	0	0,138686	-0,284704	0,380303	0,6279808
CG13479-RA	shd	0,252519	11,9148	0,0312394	11,2238	8,90567	9,36833	-0,073806	0,645207	0,6279808
CG1347-RA	shd	0,836579	46,0319	43,3102	49,3273	15,0854	15,3518	0,050601	0,833296	0,6279808
CG1347-RB	CG42758	0	6,39064	0	0	0,902726	0,680349	0,036523	0,878735	0,6279808
CG13480-RA	Lk	7,74899	0,257942	10,8074	4,93861	9,17756	7,5259	0,308975	0,331518	0,6279808
CG13481-RB	CG13481	0,229749	0,125563	0,132344	0,604172	4,5293	0,19534	-0,187354	0,510717	0,6279808
CG13482-RA	CG13482	8,12584	13,8293	26,9966	25,1857	19,657	26,3765	0,268244	0,411621	0,6279808
CG13484-RA	CG13484	12,9688	10,757	12,7389	9,08062	3749,23	10,1343	-0,093382	0,724707	0,6279808
CG13488-RA	CG13488	0,702056	0,072865	9,29593	0	0	8,89288	0,007615	0,982729	0,6279808
CG13491-RA	dj-1beta	16,0861	12,2448	7,19449	5,06419	28,4144	5,05625	0,253825	0,237452	0,6279808

gene_id	Symbol	W1_FPKM	W2_FPKM	W3_FPKM	MCM51_FPKM	MCM52_FPKM	MCM53_FPKM	FC	p-value	p-adj
CG13492-RA	Gr58c	0	0	0,134173	0,097519	7,84005	0,162339	-0,254142	0,302281	0,13772387
CG13492-RB	CG13492	2,68672	1,16611	5,15166	0,224771	0,264596	0	-0,255376	0,300407	0,13772387
CG13493-RA	CG13492	9,33396	0,109563	15,3138	0	0	0,347555	-0,058644	0,781673	0,6279808
CG13494-RA	comr	0,082885	10,0613	1,30838	0,885469	0	32,7073	0,297781	0,307819	0,6279808
CG13495-RA	CG13494	0,43471	0,267496	0,192466	0,0953551	0	3,57601	0,162425	0,320526	0,13772387
CG1349-RA	Gr58b	0	0,944957	0,246748	26,8275	0,13209	0,102379	-0,558455	0,047717	0,13772387
CG13500-RA	CG13500	0,135959	6,1273	6,02331	4,79916	6,12754	3,08149	0,09339	0,599834	0,13772387
CG13501-RA	CG13501	0,0625719	0	0	0	11,4154	0	-0,116385	0,604992	0,6279808
CG13502-RA	CG13502	0,037808	13,1896	0,0402773	0,0480078	8,26837	0,0549315	-0,360623	0,208182	0,6279808
CG13502-RC	CG13502	0,0491392	6,89102	18,3686	13,2024	0,0392049	0,0494041	-0,689688	0,024561	0,6279808
CG13503-RA	Vrp1	3,20624	0,166491	0,424082	0,653228	0	0,510819	0,08351	0,762065	0,6279808
CG13503-RB	Vrp1	6,57517	0,0647167	0,0682116	0,498892	0	0,586005	0,084427	0,75905	0,6279808
CG13503-RC	Vrp1	0,0197773	0,229975	0,818082	5,2025	6,24771	0,0723276	0,095989	0,728344	0,6279808
CG13503-RD	Vrp1	0,0198605	14,9464	15,2886	4,7541	0,0959685	0,631036	0,095989	0,728344	0,6279808
CG13503-RE	Vrp1	5,46419	3,09094	14,4744	5,31646	1,10911	0,136853	0,103183	0,710044	0,6279808
CG13503-RF	Vrp1	0,0176453	1,88422	0,0188575	3,43905	0,109659	9,63313	0,088698	0,74601	0,6279808
CG13503-RG	Vrp1	0,0182597	12,8026	60,4771	8,16856	0,0313213	1,64278	-0,033927	0,901693	0,13772387
CG13506-RA	CG13506	37,8915	46,893	2,06588	45,0144	29,169	26,2974	0,166339	0,438703	0,6279808
CG13510-RA	CG13510	71,2661	13,4262	125,628	22,042	13,2315	12,5601	0,387994	0,104999	0,6279808
CG13511-RA	CG13511	3,58135	22,384	17,1609	2,69604	6,89835	5,5155	0,732614	0,01838	0,6279808
CG13512-RA	CG42566	6,04726	11,6899	14,8658	106,244	387,881	526,843	1,39972	1,03E-06	0,13772387
CG13512-RB	CG42566	11,0204	9,82828	10,1617	62,4745	13,3811	13,1377	0,469619	0,053935	0,13772387
CG13516-RA	CG13516	9,57905	38,2447	11,3861	18,2269	7,82156	9,9105	0,072861	0,80345	0,6279808
CG13517-RB	Obp59a	0,176547	0,15214	0,128285	0,537255	5,5452	0,335419	-0,429867	0,154822	0,6279808
CG13518-RA	Obp58b	3,83283	0,595452	2,02127	3,8796	2,84178	3,26618	-0,088892	0,785128	0,6279808
CG13519-RA	Obp58d	0,905149	4,91523	6,71373	10,5826	0,230312	1,32694	-0,829823	0,018666	0,6279808
CG13521-RA	robo1	2,00148	0	1,44847	0,0103693	2304,88	0,336573	0,379573	0,196309	0,6279808
CG13521-RB	robo1	14,1114	0	10,8673	18,7136	0	0,0162162	0,381301	0,194583	0,6279808
CG13524-RA	Obp58c	6,60759	2,50777	1,20694	1,65389	0,390314	2,94281	-0,939893	0,003161	0,6279808
CG13526-RA	CG13526	0,150858	0	0	0	1,57828	1,46936	0,01562	0,897983	0,6279808
CG13527-RA	CG13527	0,166858	0,151986	0,160194	0	1,55698	1,12708	0,193677	0,380628	0,6279808
CG13531-RB	CG13531	3,24855	3,16212	3,33289	0,0318853	0,0431889	0,0325497	0,513062	0,028034	0,6279808
CG13532-RA	CG13532	6,54594	8,57212	7,25937	5,19764	4,59933	4,39306	0,517452	0,025239	0,6279808
CG13533-RA	Dvir\GJ15098	14,8176	17,2163	22,096	20,8031	28,0128	31,8113	-0,512607	0,067723	0,6279808
CG13538-RA	CG13538	0	0,199734	1,10565	0	1,14611	0	-0,14147	0,622441	0,6279808
CG13539-RA	CG13539	0,112502	0	0	0	0	0	0,35033	0,228185	0,6279808
CG13540-RA	CG1354	38,7707	33,477	47,0307	19,087	23,9889	0,299104	-0,473875	0,161679	0,6279808
CG13541-RA	CG1354	0,0454859	48,1227	37,0626	17,8002	20,6361	17,9718	NA	NA	0,6279808
CG13542-RA	CG1354	0,0448681	0,0414317	55,5144	9,6011	6,62897	6,3933	NA	NA	0,6279808
CG13544-RA	CG1354	22,7164	0,040869	0,0436691	23,069	48,9197	37,0012	0,06906	0,58405	0,6279808
CG13545-RA	CG13540	0,204781	0,497409	0	0	0	1,1316	-0,2911	0,351676	0,6279808
CG13549-RA	CG13541	0	0	0,327669	0	1,50148	0	NA	NA	0,6279808
CG13549-RB	CG13542	0	0,0404751	7,83667	0	0,0694817	0,0571726	NA	NA	0,6279808
CG1354-RA	CG13544	0	0,155924	0,164344	143,8	0,160751	0,0438965	-0,160515	0,415089	0,6279808
CG1354-RB	CG13545	128,034	82,9527	0	386,338	0,121648	405,672	-0,125225	0,527361	0,6279808
CG1354-RC	yip3	0	0	297,693	0,239551	0	0,134038	-0,160515	0,415089	0,13772387
CG1354-RD	yip3	0	0	2,57679	0,211376	3,09953	0	-0,160515	0,415089	0,6279808
CG13550-RA	Ice1	0,0148064	0,0134867	10,9093	18,94	1,35428	1,28068	0,569023	0,025619	0,6279808
CG13551-RA	CG13551	0,101551	0,0924994	0,0974948	238,799	0,128322	419,658	-0,243718	0,410533	0,6279808
CG13551-RB	CG13551	200,028	213,073	370,06	29,391	10,4651	0,171366	-0,243718	0,410533	0,6279808
CG13551-RC	CG13551	0,121986	0,111113	0,117114	8,19255	0,754334	8,71861	-0,243248	0,411603	0,6279808
CG13557-RA	CG13557	0	0	16,7468	0	0,423748	0	NA	NA	0,6279808
CG13558-RA	CG13558	0	0,955464	0	9,67669	8,08151	20,129	-0,041811	0,731452	0,6279808
CG13559-RA	CG13559	11,2042	14,3924	7,83594	0,748217	0,0487836	0,0967113	0,114181	0,725398	0,6279808
CG13560-RA	CG13560	0,562566	0	5,40097	1,30984	13,2093	1,53262	0,320836	0,324453	0,6279808
CG13561-RA	CG13561	0,187716	6,68605	31,6384	0,809873	4,19643	7,11	0,058791	0,862026	0,6279808
CG13562-RA	CG13562	1,85554	4,84588	0,0349964	28,759	0,0536517	6,85542	0,572311	0,05448	0,6279808
CG13562-RB	CG13562	3,40877	0,0367738	13,6051	5,44307	14,2299	0,0452193	0,572311	0,05448	0,13772387
CG13563-RB	CG13563	0,0318335	0,0289962	0,916717	0,0342024	0,0584285	0,0440352	-0,081467	0,818077	0,13772387
CG13564-RA	CG13564	1,34128	2,63637	1,69436	4,22042	4,05621	1,89998	0,242158	0,490183	0,13772387
CG13565-RA	Orcokinin	3,68952	2,85147	5,95726	3,17259	12,597	15,6932	0,49252	0,115774	0,6279808
CG13566-RA	CG13566	2,80611	4,57169	7,53195	5,95375	7,34161	215,932	0,40233	0,216727	0,13772387
CG13567-RA	Not11	0,0722402	0,0658014	11,3934	0,0176818	0,0439546	5,68211	0,103365	0,669668	0,13772387
CG13567-RB	Not11	10,5917	9,1195	4,04735	0,0200065	6,12506	2,45138	0,103365	0,669668	0,6279808
CG13568-RD	ppk29	0,0381942	0,0347899	0,0378953	0,0431364	0,542334	0,045711	0,901833	0,003678	0,6279808
CG13568-RE	ppk29	0,848645	0,0359537	0,0358805	0,0447779	0,0570102	0,0429663	0,901833	0,003678	0,6279808
CG13568-RF	ppk29	0,0373732	0,0340421	0,0371627	0,0420893	0,0593217	0,0447084	0,901833	0,003678	0,6279808
CG13568-RG	ppk29	0,0387088	0,0352586	702,207	0,0437958	741,627	764,309	0,901833	0,003678	0,13772387
CG13569-RA	CG43776	0,0239956	0,0242916	10,139	3,04284	0,0342676	0,0288873	0,624549	0,05387	0,13772387
CG13569-RB	CG43776	0,0227403	9,48652	0,0255725	10,9802	0,032378	9,03206	0,624549	0,05387	0,6279808
CG13570-RA	spag	3,1978	3,68188	3,9842	4,70914	2,37194	2,91639	0,093344	0,70404	0,6279808
CG13575-RA	CG13575	0,0632911	0,326683	2,98647	0	2,43051	0	-0,21418	0,53941	0,6279808
CG13576-RA	Ir60a	0	0	0	11,9302	9,86587	9,0168	NA	NA	0,6279808
CG13577-RB	CG13577	0,324838	0,469935	0,385244	0,0311321	7,84395	6,8229	0,448803	0,209735	0,6279808
CG13578-RA	CG13578	0,0824229	0,0750765	0,0791309	0,10098	1,55278	0,103084	0,637941	0,014613	0,6279808
CG13579-RA	CG13579	0,462531	0	0,532869	0,414174	0	0,0658803	-0,271259	0,395293	0,6279808
CG13580-RA	CG1358	0,0212076	107,156	12,5504	149,044	0,264518	3,18347	0,124537	0,402709	0,6279808
CG13581-RA	CG1358	4,23144	0,0252991	11,0037	0,0302739	4,26562	3,18275	0,347664	0,281601	0,6279808
CG13585-RA	CG1358	0,0174358	0,0498369	7,33952	0,0622488	6,53065	510,581	-0,252191	0,311201	0,6279808
CG13585-RB	Crtp	0,0402738	0,15512	0,103107	0	0,0213799	0,341549	-0,249879	0,314683	0,6279808
CG13586-RC	CG13581	0,118133	0,645624	0,567075	10,3874	0	0,103535	0,264458	0,379756	0,6279808
CG13586-RD	CG13585	216,279	218,054	186,2	99,7533	331,943	295,879	0,589368	0,018817	0,6279808
CG13586-RE	CG13585	0,0727708	0,0662847	0,0698643	0,054613	0,126503	0,0953405	0,690274	0,0074	0,6279808
CG13586-RF	ITP	0,0487404	0,0443962	0,0467937	0,0205814	0,0739736	0,055751	0,61504	0,012059	0,6279808
CG13587-RA	ITP	0,0341536	0,0311095	0,0327895	0,0234197	0,0499858	0,0376723	-0,208975	0,518537	0,6279808
CG13589-RA	ITP	0,0197202	0,0179625	0,0189326	21,0353	0,0278776	0,0210102	0,01562	0,897983	0,6279808
CG1358-RA	ITP	15,6343	12,6054	8,81106	0,0271772	0,0317221	0,0239077	-0,112106	0,63989	0,6279808
CG1358-RB	CG13587	0,0677701	270,294	19,8537	24,4468	11,3497	18,2919	-0,105353	0,658699	0,6279808
CG1358-RC	CG13589	0	0	21,0258	0	4,32784	0	-0,105079	0,659134	0,13772387
CG13590-RA	Bet5	19,6625	27,3983	28,5229	32,3504	15,9162	22,628	-0,04636	0,805177	0,6279808
CG13591-RA	CG13590	0	0	0,163497	0	0,233755	0	NA	NA	0,6279808
CG13591-RB	Ssl	0	0	0	0	0,221284	0,129894	NA	NA	0,6279808
CG13592-RB	Ssl	0	0	0,0840594	2,97417	0,749408	0,02213	-0,147635	0,496685	0,6279808
CG13594-RA	Ir60e	0,0598513	4,51836	2,12882	0,109532	0,018907	0	0,171435	0,45867	0,6279808
CG13597-RA	CG13594	4,24236	5,80674	29,8988	4,55916	3,99974	0,731947	0,311657	0,288247	0,6279808
CG13598-RC	tbrd-1	0,181416	0,0918037	0,154818	0,0688034	0,0931946	103,588	0,332732	0,18402	0,13772387
CG13598-RD	sba	1,93735	15,8029	3,02963	38,7563	1,97475	2,0685	0,339703	0,175343	0,6279808
CG13599-RA	sba	13,9697	19,2866	3,95409	8,32537	10,0085	9,2205	0,013282	0,968277	0,6279808
CG1359-RA	CG13599	1,73884	10,6362	8,90115	4,32783	0,733646	25,3338	0,486861	0,051597	0,6279808
CG13601-RA	CG1360	2,35447	0,876502	3,00186	3,31778	0,0220944	1,44005	-0,161124	0,552442	0,6279808
CG13602-RA	CG13601	13,0152	11,9424	18,9881	14,0755	0,0933076	4,46541	-0,269603	0,434379	0,6279808
CG13602-RB	CG43999	0,448208	0,0544345	0,0573741	0,0688868	4,23749	2,55864	-0,329281	0,343368	0,6279808
CG13603-RA	CG43999	0,0648649	0,236334	0,311371	0,382694	0,0332217	18,0796	-0,305104	0,212666	0,6279808
CG13604-RA	CG13603	21,3831	30,6686	21,3478	96,3509	50,2834	51,7245	0,487049	0,039071	0,6279808

gene_id	Symbol	W1_FPKM	W2_FPKM	W3_FPKM	MCM51_FPKM	MCM52_FPKM	MCM53_FPKM	FC	p-value	p-adj
CG13604-RB	CG13604	2,42769	0,033762	0,0406907	3,50609	0,0644167	0,0411777	0,532313	0,024572	0,13772387
CG13604-RC	CG13604	4,09032	154,656	0,0399476	162,66	0,054637	49,9402	0,554593	0,019412	0,6279808
CG13604-RD	CG13604	1,15595	69,4006	0,0355853	7,24441	34,9649	43,4978	0,484823	0,038903	0,6279808
CG13605-RA	CG13604	1,017	126,183	94,1454	12,962	44,0523	144,4	-0,052538	0,796458	0,13772387
CG13606-RA	CG13605	7,49061	7,72035	8,67776	9,78796	29,9127	7,49055	0,800856	3,56E-05	0,13772387
CG13606-RB	CG13606	44,304	2,29553	2,65792	29,7665	1,45219	1,58088	0,521637	0,009465	0,6279808
CG13607-RA	CG13606	0,124047	3,92521	42,9667	0,171807	1,96135	2,06234	0,377389	0,191389	0,6279808
CG13608-RA	CG13607	2,45075	2,34679	3,03157	4,49019	2,22227	1,55956	-0,286273	0,322471	0,6279808
CG13609-RA	mRpS24	42,8909	37,3646	48,7647	373,342	77,1013	11,356	0,211685	0,503275	0,6279808
CG1360-RA	CG13609	0,0376286	0,044125	24,1428	17,6022	3,98143	6,78734	0,520717	0,099602	0,6279808
CG13610-RA	Anp	0	0,322428	0	0	0	0	-0,363717	0,251537	0,6279808
CG13611-RA	Orct2	1,78268	1,19787	0,00495806	3,91676	1,62988	0,00531925	-0,145177	0,657339	0,6279808
CG13613-RB	shps	0,170628	12,9332	3,19849	18,5329	5,85502	18,5229	-0,137891	0,700045	0,6279808
CG13614-RA	CG13613	0,730455	1,37233	1,412	2,21704	3,65148	1,3826	0,086539	0,762999	0,6279808
CG13614-RB	CG13614	2,1279	5,95301	2,71324	0,101135	3,3004	4,50376	0,060232	0,834304	0,6279808
CG13615-RA	CG13614	0,0743538	8,30023	0,0713841	2,08012	0,0218831	6,53217	0,089919	0,800494	0,6279808
CG13616-RA	CG13615	0,961948	7,57367	0,0792298	0,0285676	0,528476	8,33819	-0,36382	0,267065	0,6279808
CG13617-RA	CG13616	4,29007	0,755353	1,42614	22,8623	0,523944	10,4013	-0,320051	0,34886	0,6279808
CG13618-RA	CG13617	0,432746	0,774758	0,787946	1,42469	0,391191	0,744843	-0,201879	0,511076	0,6279808
CG1361-RA	CG13618	50,5115	40,645	64,0892	37,5406	67,2454	75,4708	0,004598	0,979119	0,6279808
CG13622-RB	Cdc2rk	0,0505682	0,046061	0,0485485	0,0569248	0	0	-0,149253	0,650911	0,6279808
CG13623-RA	Cdc2rk	4,08229	0,050591	4,79908	4,13608	146,648	112,139	-0,271215	0,379551	0,6279808
CG13624-RA	CG13622	1,17573	1,21373	1,16639	2,35419	1,14656	1,45605	0,076281	0,763437	0,6279808
CG13624-RB	CG13623	15,6885	15,1889	14,0672	12,8305	23,3022	24,8856	0,08262	0,744028	0,6279808
CG13624-RC	REPTOR	8,40294	6,7864	0,0175159	0,0189752	0,0305181	6,44222	0,051627	0,831806	0,6279808
CG13624-RD	REPTOR	0,0173579	0,0158108	0,0172539	0,0186793	3,4249	0,0183908	0,083006	0,743022	0,6279808
CG13624-RE	REPTOR	4,48714	4,39233	3,29663	6,56235	0,544919	2,96266	0,08262	0,744028	0,6279808
CG13624-RF	REPTOR	0,0182446	0,0166184	1,61038	5,4359	0,510093	0,0193706	0,076281	0,763437	0,6279808
CG13625-RA	REPTOR	0,0179717	0,0163698	7,92875	7,53532	0,0103401	0,0190686	0,073658	0,771948	0,6279808
CG13626-RA	REPTOR	6,7671	11,6375	15,762	11,3761	45,1686	2,47603	0,120504	0,604449	0,6279808
CG13627-RA	CG13625	6,48304	10,5548	6,66251	6,80728	4,95375	6,51046	0,358487	0,248055	0,6279808
CG13627-RB	Syx18	10,8595	11,0829	5,46742	11,6818	10,1243	38,091	0,374449	0,228299	0,6279808
CG13628-RA	CG13627	26,1362	19,4826	26,2253	35,7612	6,63509	13,1489	-0,358764	0,201923	0,6279808
CG1362-RA	CG13627	0,0281214	6,00669	0,0269982	0,0299437	1,39696	0,0305676	-0,334814	0,236628	0,6279808
CG1362-RB	Rpb10	115,542	73,2489	93,8481	181,919	257,994	0,867838	-0,334814	0,236628	0,6279808
CG13630-RA	blow	8,22719	0,0305722	0,0266653	18,0851	0,0410061	2,96251	-0,312755	0,194787	0,6279808
CG13631-RA	blow	8,69666	356,902	41,5499	472,395	0,0843164	0,199357	0,375282	0,216712	0,6279808
CG13633-RA	CG13630	15,2788	16,1119	26,5661	25,1203	20,9971	7,37435	-0,092723	0,728516	0,6279808
CG13634-RA	CG13631	72,7601	90,7063	84,8958	136,108	31,5645	59,6013	-0,369238	0,187846	0,6279808
CG13636-RA	AstA	8,06528	0,775797	4,10378	8,11936	0,612859	24,0326	0,380207	0,17524	0,6279808
CG13636-RB	CG13634	3,78188	3,76978	0,0130082	0,0148454	3,22918	5,84432	0,494405	0,073277	0,6279808
CG1363-RA	sosie	0,0446559	0,0131192	0,013658	14,3693	56,2593	0,0145926	0,210814	0,305731	0,6279808
CG1363-RB	sosie	17,1175	3,16172	0,013658	7,3163	0,143142	4,08507	0,241911	0,233274	0,6279808
CG13640-RA	CG13640	1,74224	0,168936	0,0227155	10,6776	0,0195275	0,0176154	-0,805485	0,021012	0,6279808
CG13641-RA	CG13641	41,6058	12,3	45,3653	30,8051	4,23716	6,50362	-1,072167	0,001919	0,6279808
CG13643-RA	CG13643	10,0995	0,252395	6,41888	0,207496	2,92163	5,29692	0,3554	0,155042	0,6279808
CG13645-RA	Nmnat	2,03926	2,16284	1,87649	3,39141	2,79515	1,45723	-0,296603	0,265905	0,6279808
CG13645-RB	Nmnat	3,80279	4,02489	2,68193	4,77215	6,21301	1,84388	-0,464561	0,073539	0,6279808
CG13646-RA	mah	0,104286	0,246977	0,100121	0,259914	0,156093	0,157365	-0,031652	0,922542	0,6279808
CG13648-RA	tnc	1,80846	4,43443	17,7471	7,07645	1,12695	17,6027	-0,492585	0,135362	0,6279808
CG13650-RB	CecA1	0,655143	0,682	0,468745	0,485523	0	1,07476	0,242117	0,342045	0,6279808
CG13651-RA	Nepl16	2,6721	2,44515	3,14465	8,15887	1,35769	1,90929	0,231098	0,357338	0,6279808
CG13652-RA	danr	8,40867	5,01029	8,01925	11,5205	0	0	0,044335	0,715924	0,6279808
CG13653-RA	CG13652	0,199024	11,2006	0	0	2,33971	4,1331	0,252973	0,402495	0,6279808
CG13654-RA	CG13653	0,419974	0,181285	0,224	0,314936	0	0	-0,154254	0,464403	0,6279808
CG13656-RA	CG13654	3,35289	0,0416035	4,44238	5,55092	3,6995	4,07685	-1,118118	0,001444	0,6279808
CG13658-RB	CR13656	0,0900216	8,9428	13,2013	15,227	75,7471	1,23922	-1,050062	0,003145	0,6279808
CG13659-RA	CG13658	2,10196	0,742194	11,1313	69,224	41,3406	0,560903	-1,117714	0,001378	0,6279808
CG1365-RA	CG13659	3,63575	5,04749	14,1431	4,21793	4,62847	20,0834	0,228931	0,42937	0,6279808
CG13663-RA	CG13663	21,4356	15,2902	19,0389	6,57289	8,20232	2,85498	0,25271	0,345974	0,6279808
CG13664-RA	Cad96Cb	2,8642	3,06264	5,31773	0,118295	2,51423	4,00601	0,047761	0,882031	0,6279808
CG13667-RA	CG13667	0,0353221	0,0321738	0,0339113	4,6964	5,58887	5,95196	0,121708	0,61268	0,6279808
CG13667-RB	CG13667	3,83119	3,38905	5,12822	4,21791	2,0077	2,61826	0,121708	0,61268	0,6279808
CG13670-RA	CecA2	0	0	0	0	0	1,2023	0,349347	0,324594	0,6279808
CG13671-RA	CG13670	0,617532	0,718738	0,395245	0,410334	13,408	0,285861	-0,03946	0,871343	0,6279808
CG13674-RA	CG13671	0,0253877	0,0231249	0,0243737	0,026856	0,0363766	0,0274156	-0,071494	0,813184	0,6279808
CG13675-RC	CG13674	85,2792	10,0907	0	57,2441	0	2,23792	1,107675	9,7E-07	0,6279808
CG13676-RA	CG13675	27,8898	0,0112864	0,0139248	0,0184802	0,0250316	0,0152648	0,498226	0,0697	0,6279808
CG13678-RA	CG13676	42,3611	38,5684	30,6301	1,4677	17,3698	0,704223	-0,085363	0,771032	0,6279808
CG13679-RA	CG13678	141,642	283,265	8,57254	248,305	308,719	352,28	-0,145871	0,643764	0,13772387
CG13679-RB	CG13679	0,1476	48,5743	2,88349	0,239037	3,08079	0	-0,145703	0,644151	0,6279808
CG1367-RA	CG13679	141,981	0,159646	8,19993	35,6154	3,74922	2,10442	-0,041811	0,731452	0,6279808
CG13681-RA	CG1368	349,627	1,49564	8,77742	481,777	7,78188	2,44044	0,006762	0,984102	0,6279808
CG13685-RA	Ect4	2,76926	2,40511	2,08445	2,59115	0,0217286	1,52251	-0,121197	0,733337	0,6279808
CG13686-RA	CG13685	0,484557	39,2425	7,5126	7,94012	4,56062	5,22928	-0,041811	0,731452	0,6279808
CG13687-RA	lectin-21Cb	0	68,1475	13,7462	0,172938	0	0,0713288	-0,026364	0,940857	0,6279808
CG13688-RA	Ptth	0,775539	0	60,4956	0,16269	0,438308	0,301092	0,404933	0,115041	0,6279808
CG13689-RA	Ipk2	3,99208	6,98712	9,26251	14,0865	101,01	6,05168	-0,188479	0,504205	0,6279808
CG1368-RA	CG13689	21,4771	2189,27	0,0441966	3476,78	0	0,0517283	0,38206	0,17619	0,6279808
CG13690-RA	CG13690	4,67222	4,90452	9,49089	7,13818	363,415	5,41483	0,205705	0,461855	0,6279808
CG13691-RA	BBS8	1,20138	1,16725	1,53786	1,82926	1,29899	19,7933	-0,002611	0,99288	0,6279808
CG13692-RA	CG13692	3,74657	2,43021	2,88845	14,2264	2,07338	0,924042	0,31777	0,303789	0,6279808
CG13693-RA	CG13693	0,0713049	26,9062	32,1662	14,0283	0,0451555	30,578	0,071186	0,575632	0,6279808
CG13694-RA	CG13694	6,93389	6,76169	28,2507	3,0827	18,8527	3,56679	1,551403	1,48E-06	0,6279808
CG13695-RB	geko	29,0022	31,1435	10,1067	14,4235	3,00195	21,0525	0,311906	0,097831	0,6279808
CG13698-RB	CG13698	25,78	27,3783	15,5205	46,8185	6,99564	9,60524	0,187076	0,508333	0,6279808
CG13699-RA	CG13699	5,29206	82,396	4,79327	0	0	38,4546	0,46305	0,180902	0,6279808
CG13700-RB	CG13700	0	0	0,0489986	8,35983	21,6865	5,0012	-0,280684	0,26471	0,6279808
CG13701-RA	skl	26,0335	23,433	12,9414	11,2102	19,4349	0,0731519	0,963079	0,001485	0,6279808
CG13702-RB	AstC-R2	0,028596	1,6627	2,17693	0,161923	0,219326	0,0311192	-0,782636	0,008025	0,6279808
CG13702-RC	AstC-R2	2,06784	0	4,24455	0	0	3,11212	-0,782636	0,008025	0,6279808
CG13704-RA	CG13704	112,823	0	0	126,687	0	0	0,137669	0,58269	0,13772387
CG13705-RA	CG13705	129,881	0	11,1853	145,043	5,87193	5,614	-0,385433	0,24681	0,6279808
CG13707-RA	CG13707	15,645	0,924524	7,03568	11,6077	5,45442	7,38277	1,591138	1,05E-07	0,6279808
CG13708-RA	CG13708	0,566703	6,976	0,0577749	0,0233698	6,56556	3,74541	0,009389	0,975171	0,6279808
CG13711-RA	CG1371	22,0762	0	17,9462	569,068	16,7977	6,93646	-0,013096	0,914398	0,6279808
CG13712-RA	CG13711	0	0,0547315	0	0	1,65298	0,555284	NA	NA	0,6279808
CG13713-RA	CG13712	0	8,89756	0	0	2,33483	0	-0,062968	0,745795	0,6279808
CG13715-RA	CG13713	0,330379	0,0425816	0,317184	1,57121	0,0703315	0,995756	-0,06092	0,850824	0,13772387
CG13716-RA	axo	4,12974	0,0275495	0,0290373	0,0323709	354,2	21,6939	0,242929	0,471524	0,6279808
CG13717-RA	CG13716	1,39991	13,6198	9,93367	10,0679	2,61743	1,45727	0,628748	0,074371	0,6279808
CG13719-RA	axo	2,13423	0,0157859	1,89678	1,87888	6,96369	0	NA	NA	0,6279808
CG1371-RA	CG42540	0	3,08649	3,55815	8,49666	0,0438466	0	0,089939	0,726527	0,6279808
CG13720-RA	yl	0,0106288	0,125859	0,127554	321,17	0,0145158	0,274324	-0,076539	0,830184	0,6279808

gene_id	Symbol	W1_FPKM	W2_FPKM	W3_FPKM	MCM51_FPKM	MCM52_FPKM	MCM53_FPKM	FC	p-value	p-adj
CG13721-RA	yl	0,14689	0,00955695	0,0100731	0	314,017	0,01094	-0,712805	0,038767	0,6279808
CG13722-RA	CG13720	0,274788	14,2498	0,263813	9,57486	2,33592	0,269425	0,093832	0,721837	0,6279808
CG13723-RA	CG13721	14,2032	50,3601	25,7897	0,759906	0,262465	56,2861	NA	NA	0,6279808
CG13724-RA	CG13722	11,0126	0,481338	8,24534	38,77	10,8982	8,86205	0,111005	0,699837	0,6279808
CG13725-RA	CG13723	0	0,0284211	0,029956	0,0398252	0,038779	3,57317	NA	NA	0,6279808
CG13726-RA	CG13724	0,159893	0	0	0	0	0,106937	0,06906	0,58405	0,6279808
CG13727-RA	CG13725	0	0,0438463	0,0462142	0	0	0,0406551	0,060105	0,810063	0,6279808
CG13728-RA	Or74a	0	8,17538	5,90731	0,0538542	0,0539435	0	0,491038	0,158773	0,6279808
CG1372-RA	CG13727	8,79558	0,0635952	11,6545	9,91325	12,8905	7,34148	-1,007477	0,001976	0,6279808
CG1372-RB	CG13728	0,845063	1,30734	7,43309	1,08708	0,324878	14,2089	-1,007477	0,001976	0,13772387
CG13731-RA	CecC	0,686503	1,40696	0,494313	2,21656	5,24756	0	-0,055408	0,789275	0,6279808
CG13732-RA	CG13731	133,112	128,209	10,0537	9,38548	12,2413	618,239	-0,778922	0,024466	0,6279808
CG13733-RA	Dsim\qjt	1,03538	127,01	1,40067	0,201085	3,23724	0	-0,129535	0,717405	0,6279808
CG13737-RA	CG13733	0,550632	0,343169	0,556463	2,15274	0,438733	0,538106	0,891889	4,14E-05	0,6279808
CG13737-RB	CG13737	82,8421	0,0794364	85,4538	75,2954	0,371238	4,51151	0,908631	2,87E-05	0,13772387
CG13738-RA	CG13737	0,0872095	5,97199	0,0837263	0,108282	2,02762	0,952937	-1,131372	0,00109	0,6279808
CG13739-RA	CG13738	519,368	21,8437	6,07372	24,7835	2,61612	17,8392	0,46568	0,103914	0,6279808
CG1373-RA	CG13739	1,14934	0,889523	0,843805	0,857442	5,6444	4,02071	-0,573505	0,1021	0,6279808
CG13741-RA	tsh	4,98535	7,08164	5,00995	11,7014	32,6235	4,83031	-0,654748	0,008162	0,6279808
CG13742-RB	tsh	25,2303	33,0306	25,1038	30,4448	0,0683641	13,7456	0,38986	0,261954	0,6279808
CG13743-RA	CG13741	0,0349115	7,03028	13,4534	0,0351137	9,43381	14,2638	-0,114124	0,660999	0,13772387
CG13744-RA	CG13742	0,631766	3,36243	2,11581	79,4303	0,466179	0,885317	-0,410619	0,230206	0,6279808
CG13745-RA	CG13743	2,14812	1,76868	2,14338	1,98074	2,48839	0,0910803	-0,341345	0,292107	0,13772387
CG13746-RA	CG13744	1,29893	83,6689	1,53178	1,11733	2,96894	1,63284	-0,258372	0,335561	0,13772387
CG13747-RA	FANCI	0,510407	0,0259126	0,0273591	16,926	0,0465691	9,35227	0,537287	0,133319	0,6279808
CG13748-RA	MrgBP	12,8932	0,0177193	23,2261	0,0197723	8,78702	0,0193706	0,24572	0,492171	0,6279808
CG13749-RA	CG13747	0,0702188	8,81721	0,0674142	7,37373	28,4632	11,9553	-0,085523	0,796945	0,6279808
CG1374-RA	CG13748	3,57428	24,8554	0,567025	22,6601	0,0411384	0,0310044	0,243615	0,360027	0,6279808
CG1374-RB	CG13749	0,196654	9,23915	0,027312	2,62237	7,35057	11,9955	0,284327	0,285184	0,6279808
CG13751-RA	CG13751	17,6774	0,0170709	34,6843	0,0206718	0,0203003	0,0207126	-0,217187	0,470253	0,6279808
CG13758-RA	Pdfr	1,34734	1,72582	1,87292	1,56891	1,23593	1,42738	0,191164	0,468165	0,6279808
CG13759-RA	AANATL7	4,47389	13,8923	21,5652	15,4549	6,43246	21,0485	-0,034689	0,899469	0,6279808
CG13760-RB	CG13760	3,93735	4,57668	5,48464	4,55207	17,7475	5,96088	-0,121196	0,662134	0,6279808
CG13761-RB	Smyd3	3,65457	7,00937	7,25738	4,42453	6,30387	0,10345	0,20371	0,385343	0,6279808
CG13762-RA	brv3	0,172073	123,932	153,881	171,219	154,783	168,829	0,235567	0,507178	0,6279808
CG13766-RA	CG13766	0,0304263	0,0277144	49,9042	0,032579	0,0441284	0,0332579	-0,002813	0,990884	0,6279808
CG13771-RA	CG13771	0,138621	0	10,0168	0,214767	539,315	7,14549	-0,128182	0,573445	0,6279808
CG13772-RA	Nlg2	4,61412	0,0223603	4,43646	5,63342	4,17414	14,8135	-0,009405	0,965619	0,6279808
CG13773-RA	CG13773	13,141	5,8465	20,5655	9,64856	15,5849	3,32317	0,11153	0,723183	0,6279808
CG13775-RA	CG13775	0,0370657	0,033762	0,0355853	0,0403372	1,56294	1,61152	0,224526	0,490892	0,6279808
CG13776-RA	CG43322	0,816647	0,0495905	1,09764	0,061898	0,0838412	0,922396	0,11908	0,734189	0,6279808
CG13776-RB	CG43322	0,0541309	1,08474	0,0519689	0,551235	1,31782	0,062775	0,128603	0,714656	0,6279808
CG13777-RA	milt	0,0164791	0,0288019	10,2813	5,24443	0,0459932	2,07433	-0,269405	0,362751	0,6279808
CG13777-RB	milt	26,3387	0,0246094	0,0512137	12,8767	0,0388634	4,11961	-0,240203	0,417553	0,6279808
CG13777-RC	milt	0,0166494	0,0238772	0,015821	0,0604777	0,0376344	0,0617379	0,004896	0,986545	0,6279808
CG13778-RA	Mnn1	0,0353599	0,0322082	0,0339476	7,34148	3,13446	0,0391183	0,466727	0,075157	0,6279808
CG13778-RB	Mnn1	0,0157523	0,0143483	0,0151232	0,319485	0,0519044	0,016627	0,329245	0,232818	0,6279808
CG13778-RC	Mnn1	7,61744	10,5785	6,49063	83,6996	0,0220616	3,94409	0,52461	0,043771	0,6279808
CG13779-RA	Sem1	43,9219	2,60556	1,99157	1,55682	1,83773	1,26498	0,053184	0,821342	0,6279808
CG13780-RA	tll	1,24446	1,59938	1,24386	1,64947	0,713022	0,764973	-0,044163	0,854231	0,6279808
CG13784-RB	Pvf2	3,35354	3,14233	3,12717	0,0162876	61,2075	3,00419	0,106256	0,64102	0,6279808
CG13784-RC	CG13784	28,6577	36,0926	25,2823	0,0172513	23,9917	18,9936	0,336603	0,142615	0,6279808
CG13786-RA	CG13784	0,0516755	0,0470697	0,0496116	9,61543	0,023367	0,0595515	0,333881	0,335524	0,6279808
CG13787-RA	CG13786	0,141414	0,193215	0,254562	0,158192	4,45397	0,161488	-0,20256	0,425894	0,6279808
CG13788-RA	Gr28a	0,0723987	0	3,82135	3,37678	1,63178	2,571	0,842544	0,01272	0,6279808
CG13788-RB	Gr28b	0,0448377	0,0414317	0,0436691	0,474599	162,72	50,9665	0,711732	0,035074	0,6279808
CG13788-RC	Gr28b	0,0454859	0,64374	0,493458	0,339275	139,126	75,521	0,807592	0,016849	0,6279808
CG13788-RD	Gr28b	0,813812	0,0392691	0,496677	0,0491905	78,343	0,0507842	0,816338	0,017203	0,6279808
CG13788-RE	Gr28b	0,0431117	0,444727	0,468744	69,5647	0,0673834	0,15356	0,862398	0,012332	0,6279808
CG1378-RA	Gr28b	0,643595	41,326	15,8835	15,8905	0,0684689	0,128	0,293192	0,343281	0,6279808
CG13791-RA	RapGAP1	1,2124	0,193994	0,153352	0,153301	0,521962	0,0971546	0,274642	0,393129	0,6279808
CG13792-RA	Slob	1,80698	0,379673	0,191389	0,304956	0	1,28746	-0,700011	0,00714	0,6279808
CG13793-RA	CG13793	0,246719	1,15743	0,121294	8,57974	3,469	0,073835	-1,383152	9,8E-05	0,6279808
CG13794-RA	CG13794	1,58022	1,99818	0,258398	12,735	10,2871	2,91528	-0,9991	0,001034	0,6279808
CG13795-RA	CG13795	5,5604	0,0137234	7,26408	0	5,28947	0,868869	-0,532109	0,028141	0,6279808
CG13796-RA	CG13796	2,8804	4,5423	2,36335	3,13215	0,0249895	2,54143	0,173009	0,565956	0,6279808
CG13796-RB	CG13796	0,733995	0,0222858	0,716423	4,55295	0,0142972	2,83684	0,173009	0,565956	0,6279808
CG13796-RC	CG13796	2,14655	4,62248	1,7511	6,33951	5,18709	1,15623	0,173009	0,565956	0,6279808
CG13796-RD	CG13796	0,0243224	0,0221545	0,0233509	2,69913	0,0140122	0,0181869	0,173009	0,565956	0,6279808
CG13800-RA	sut4	0	18,366	7,89694	0,0678931	0,0456404	15,2426	-0,440265	0,059339	0,6279808
CG13801-RA	sut4	0	24,0594	543,766	55,5774	1253,16	1,98238	0,044335	0,715924	0,6279808
CG13802-RA	Svil	7,7019	8,03363	74,4817	14,5793	8,58308	3,99218	-0,209192	0,485577	0,6279808
CG13802-RB	CG13801	0,0478118	0,0435503	1,02613	3,60047	10,7618	0,179964	-0,201014	0,490473	0,6279808
CG13804-RA	MsR2	0,424347	0,676741	2,13788	21,0378	0,0862725	1,19953	-0,014388	0,910154	0,6279808
CG13806-RA	MsR2	0,495308	2,35103	0	4,85253	0,0756669	110,826	-0,038761	0,867537	0,6279808
CG13807-RA	yellow-g2	0,0692619	0	0,297291	0,0276215	0,0095788	10,9143	0,041065	0,894291	0,6279808
CG13809-RA	CG13806	31,7478	29,0969	0,624756	0,421478	0	0,0570272	-0,38003	0,199526	0,6279808
CG1380-RA	CG13807	9,40261	8,91652	19,3525	7,33307	3,75543	10,9354	0,01562	0,897983	0,6279808
CG1380-RB	Oseg2	0,316777	0,339461	0,292198	0,0318161	4,44337	0,324005	0,01562	0,897983	0,6279808
CG13810-RA	RpLP0-like	70,1066	37,7741	83,1538	84,4447	9,36928	81,992	-0,086116	0,712792	0,6279808
CG13813-RA	CG13810	7,40974	1,07403	6,48925	8,83683	0,0442051	0	-0,863871	0,002355	0,6279808
CG13814-RA	CG13813	8,91743	20,9424	23,4515	4,31581	22,857	2,04336	-0,619414	0,082678	0,6279808
CG1381-RA	rdgC	1,15371	4,15263	2,21525	60,8729	6,38591	3,1874	-0,497788	0,085291	0,6279808
CG13822-RA	GILT3	35,2973	46,8612	40,3417	59,371	43,0854	52,7986	-0,479924	0,057215	0,6279808
CG13826-RA	cnc	1,37546	1,05633	1,89016	2,32675	115,094	7,16816	-0,402509	0,203614	0,6279808
CG13827-RA	CG13827	18,1122	13,7176	17,3888	14,6283	11,0274	15,2989	0,177547	0,463096	0,13772387
CG13829-RA	CG13829	0	0	8,37154	40,0506	5,25843	0,0266235	NA	NA	0,13772387
CG13830-RB	Cow	25,4943	32,3675	21,0674	26,8061	2,30099	1,38591	0,3447	0,176769	0,6279808
CG13833-RA	CG13833	0,649815	26,9401	0,0826	0	0,140993	0	0,48342	0,176799	0,13772387
CG13836-RA	Irk1	0,69371	0	1,24876	0,336219	4,43027	3,15596	0,052134	0,862452	0,6279808
CG13837-RA	CG13837	0,835781	0,940414	2,1712	3,92747	0,577768	2,10624	0,009955	0,977773	0,6279808
CG13838-RA	CG13838	0,845157	2,4251	1,10646	25,4831	2,39951	21,8763	-0,181145	0,605627	0,6279808
CG13840-RA	CG13840	0	0	1,13028	1,18575	0	0,965993	0,01562	0,897983	0,6279808
CG13841-RA	CG13841	0,390982	0,863984	0,0208971	39,4986	4,57668	5,38134	0,169932	0,587261	0,13772387
CG13842-RA	CG13842	0,709578	0,206118	0,749361	0,853486	9,52024	0,0197923	-0,329327	0,283592	0,6279808
CG13843-RA	CG13843	0,0594385	0,426855	0,33743	0,030942	0,797915	0,0701755	-0,105958	0,76718	0,6279808
CG13847-RA	CG13847	15,2522	2,37629	254,54	5,07188	19,2389	191,189	0,718091	0,029423	0,6279808
CG13848-RA	pinta	25,6706	26,0523	29,5081	35,5583	12,0629	25,6891	0,319037	0,240122	0,6279808
CG13849-RA	Nop56	69,1413	47,5515	0,021521	264,628	1,58453	0,0240358	-0,347246	0,258242	0,6279808
CG13850-RA	Def	0	2,78133	23,7322	14,3014	0,0802238	12,8039	-0,288498	0,137612	0,6279808
CG13852-RA	CG13850	23,5641	25,5921	29,5572	0,0334557	1,49011	0,0341528	0,270978	0,264794	0,6279808
CG13855-RA	mats	20,3551	42,8303	24,8035	28,6048	0,0724131	19,0619	0,151976	0,578489	0,6279808
CG13856-RA	CG13855	0,0292285	0,0266233	0,0280611	85,5783	0,625843	122,889	0,808321	0,006187	0,6279808
CG13857-RA	CG13856	3,18143	0,54368	0,0299418	2,14909	0,0653124	1,90486	0,01921	0,950813	0,6279808

gene_id	Symbol	W1_FPKM	W2_FPKM	W3_FPKM	MCM51_FPKM	MCM52_FPKM	MCM53_FPKM	FC	p-value	p-adj
CG13858-RA	CG13857	0,789084	0,566289	0,987133	33,4536	0,298683	28,77	-0,060347	0,691408	0,13772387
CG1385-RA	CG34377	0	24,3944	1,37288	2,32977	4,47036	3,19886	-0,104244	0,537109	0,6279808
CG13862-RA	antdh	1,78028	8,53195	0,230121	11,0573	15,7344	1,55045	-0,394654	0,251381	0,6279808
CG13865-RA	CG13862	8,45614	2,18511	14,5376	6,29454	0,0185032	0,210928	-0,430158	0,090905	0,6279808
CG13865-RB	Coa7	0,142815	0,130086	0,137111	0,210167	0,284672	0,214546	-0,563658	0,039278	0,6279808
CG13867-RA	Coa7	22,9131	18,7302	28,9639	29,7443	35,9085	31,632	0,072052	0,737987	0,6279808
CG13868-RA	MED8	0,0723985	0,180559	0,842699	2,23703	2,31743	2,13822	-0,15529	0,5266	0,13772387
CG13869-RA	CG13868	41,3607	0,845492	34,958	0,945353	0,0978067	0,0186923	0,078912	0,804397	0,6279808
CG1386-RA	Dsim\GD25297	1,44448	1,61936	0,106676	2,89796	0,968364	1,17195	-0,323997	0,335984	0,6279808
CG13870-RA	CG1387	0,0289299	0,0105227	6,50067	4,22721	3,21	4,26549	0,097512	0,666804	0,6279808
CG13870-RB	CG13870	0,0955589	0	1,01869	0,932471	0,896667	0	0,097512	0,666804	0,6279808
CG13871-RA	CG13870	0,0375715	1,09829	0,146788	0,275681	0	0,336823	-0,013096	0,914398	0,6279808
CG13872-RA	CG13871	0	12,4881	18,4888	0,0226469	1,88093	3,39078	0,141752	0,634108	0,6279808
CG13873-RA	CG13872	0,0982298	0,0121843	0,0128423	0,0137546	0,0186307	0,0140412	-0,070526	0,562659	0,13772387
CG13873-RB	Obp56g	0	0	0	0	1,17963	0	-0,070526	0,562659	0,6279808
CG13874-RA	Obp56g	0	0	0	0	0,503192	0	-0,53039	0,080985	0,6279808
CG13875-RA	Obp56h	34,751	33,4369	59,5209	86,5997	150,824	83,5496	0,507827	0,045903	0,6279808
CG13876-RA	CG13875	8,0711	0,0229719	28,7335	0,0272559	3,29257	0,0278238	0,030995	0,910881	0,6279808
CG13877-RA	CG13876	5,81223	26,0911	6,13075	0,0548859	39,034	47,0298	0,282542	0,394276	0,6279808
CG13879-RB	CG13877	0,172221	82,1872	0,0832504	37,7524	0,380566	0,286818	-0,433784	0,116655	0,6279808
CG1387-RA	wac	9,5278	9,52389	12,0577	16,8031	21,3001	16,053	0,043814	0,829351	0,6279808
CG13880-RA	mRpL17	45,4053	35,3476	53,1157	55,5477	94,5319	76,3386	-0,564235	0,054669	0,6279808
CG13884-RA	CG13884	0,0916869	0,0835148	5,02149	12,2681	0,0234715	0,117694	0,308126	0,371789	0,6279808
CG13885-RA	trh	0	4,82487	7,26353	0,0173284	7,5006	4,57511	-0,000281	0,999127	0,6279808
CG13886-RA	CG42553	6,80603	131,204	12,4555	8,54969	0	10,5747	-0,252853	0,318041	0,6279808
CG13887-RB	CG13887	64,4596	0,957029	1,46824	2,36399	2,83488	53,9436	0,422993	0,044457	0,6279808
CG13887-RC	CG13887	0,054488	62,643	86,502	27,5492	40,3138	0,0632472	0,423077	0,044359	0,6279808
CG13888-RA	Gr61a	0	2,59583	0,145164	0,192117	0,12077	20,0704	-0,000881	0,997629	0,13772387
CG13889-RA	cep290	0,509193	0,804263	0,556463	6,42716	3,81345	3,91083	-0,111341	0,726547	0,13772387
CG13890-RA	tor	0,0221236	8,47557	0,02124	0,0232216	0,0314538	0,0089228	0,061639	0,85371	0,13772387
CG13891-RA	tor	0,0213515	0,0461316	1,34267	0,0223701	0,516481	5,044	0,695697	0,048775	0,6279808
CG13892-RA	tor	0,0209189	14,6219	0,0200834	0,0218944	0,0296561	0,00891925	0,07739	0,787827	0,6279808
CG13893-RA	Dci	6,51678	4,11136	16,0515	1,15768	0,425408	0,0910196	-0,299441	0,138085	0,13772387
CG13894-RA	CG13891	0,648781	4,3003	2,76288	0,0179125	0,0242627	0,0182858	0,295388	0,118267	0,13772387
CG13895-RA	Cypl	7,17035	7,73559	15,6232	13,5596	6,49169	12,529	0,595284	0,001615	0,6279808
CG13896-RA	CG13893	10,0163	13,5096	13,039	8,36299	13,4861	12,1759	0,158875	0,526434	0,6279808
CG13896-RB	CG13894	13,5656	0,0272625	3,84558	9,31211	9,84693	9,76679	0,158875	0,526434	0,6279808
CG13897-RA	CG13895	20,9945	7,98308	7,8481	826,97	186,931	1616,49	-1,053181	0,001029	0,6279808
CG13898-RA	CG13896	1,74312	3,96938	1,70697	2,55932	26,9929	6,58644	-0,018479	0,952617	0,6279808
CG1389-RA	CG13896	3,91569	3,17402	6,82881	150,965	6,08279	3,77897	0,136289	0,702533	0,6279808
CG1389-RB	hng3	0,750231	1,3161	3,48042	5,72514	3,15096	6,18188	0,313585	0,34324	0,6279808
CG1389-RC	CG13898	0,317164	0,625939	0,152248	0,849842	0,0134423	0,238798	0,136289	0,702533	0,13772387
CG13900-RA	Sf3b3	34,7394	31,4204	38,0959	61,9079	16,3963	8,97172	-0,220393	0,291746	0,6279808
CG13900-RB	Sf3b3	16,3155	10,4431	21,2657	0,0619562	22,38	2,21982	-0,037502	0,851733	0,6279808
CG13901-RA	CG13901	5,68814	8,60939	8,12178	7,4122	1317,59	9,10255	0,063709	0,827995	0,6279808
CG13902-RA	JMJD5	2,84984	0,114772	4,50353	4,67492	8,13266	19,8229	-0,213053	0,4131	0,6279808
CG13905-RB	CG13905	0,276468	16,1667	1,81374	1,12909	8,57788	11,3317	0,457138	0,166657	0,6279808
CG13906-RA	nerfin-1	12,9315	3,10219	9,69146	17,2688	0,642885	7,07779	0,328637	0,295404	0,6279808
CG13907-RA	CG13907	41,6193	77,3134	37,2569	68,1668	25,3377	34,1194	0,169848	0,569734	0,6279808
CG13908-RA	Glut1	6,36705	8,49828	5,21621	8,74271	3,71152	13,6641	0,395727	0,165965	0,6279808
CG13908-RB	Glut1	1,12424	1,40708	1,4913	0,0178061	1,01332	0,0531518	0,39733	0,164171	0,6279808
CG13912-RA	sol	5,26884	3,97783	3,04695	5,87765	4,20365	5,1395	-0,243854	0,469103	0,6279808
CG13913-RA	sol	0,012043	1,20938	1,82686	0,149577	0,0167214	0,0126022	0,061772	0,853034	0,6279808
CG13914-RA	sol	3,46398	15,5564	14,1293	39,0764	3,82285	3,42574	-0,542494	0,085496	0,6279808
CG13916-RA	CG13912	17,5514	9,10492	21,2207	11,3918	6,03591	8,43782	0,210147	0,424652	0,6279808
CG13916-RB	mwh	17,1966	2,30052	2,85931	32,284	11,9952	4,98071	0,156758	0,51281	0,6279808
CG13917-RA	msd1	3,8473	3,17064	7,79768	12,7136	8,0413	9,55963	0,019033	0,943393	0,6279808
CG13919-RA	SA-2	0,0236266	0,0215208	0,0567075	120,909	0,0529828	0,0249728	0,365291	0,222274	0,6279808
CG1391-RA	SA-2	0,0697329	0,137621	0,0223159	0,039116	21,4207	7,61691	-0,119189	0,587478	0,6279808
CG1391-RB	CG13917	6,76865	20,7979	7,21273	13,9204	10,1553	11,3057	-0,140416	0,521903	0,6279808
CG1391-RD	CG13919	6,84119	0,0364178	3,75628	10,5742	0,0361555	9,40014	-0,118644	0,588749	0,6279808
CG13920-RA	CG13920	44,8701	45,3032	56,4292	42,5983	59,8048	61,887	-0,344785	0,098851	0,6279808
CG13921-RA	CG13921	4,08692	6,3378	5,20397	2,04682	2,74436	1,09512	-0,157096	0,524771	0,6279808
CG13921-RB	CG13921	0,777174	0,0125293	0,831972	5,67237	1,32379	131,502	-0,124036	0,620728	0,6279808
CG13922-RA	mRpL46	28,9947	18,4914	48,8191	16,0832	8,13165	63,0049	-0,481817	0,122291	0,6279808
CG13923-RA	CG42676	5,51106	0,635771	0,0788359	6,99256	0,136152	0,102613	0,270694	0,29281	0,6279808
CG13924-RB	CG13924	1,11954	23,2783	1,33805	5,74459	22,0328	8,77949	-0,310977	0,303018	0,6279808
CG13926-RA	CG13926	12,7261	16,0467	17,2396	0,018818	16,315	18,2206	-0,283532	0,221664	0,6279808
CG13927-RA	GC	3,5844	0,0372979	0,0234026	44,7162	0,0348429	28,5186	0,026709	0,916755	0,6279808
CG13928-RA	CG13928	40,5088	0,0353899	17,1561	0,0438249	19,0244	44,3573	0,26274	0,320887	0,6279808
CG13929-RA	metl	3,07738	2,55056	4,87087	5,28104	5,51118	2,84757	-0,03294	0,906901	0,6279808
CG13929-RB	metl	12,9143	9,72241	18,3439	9,38362	13,11	15,1382	-0,037441	0,894147	0,6279808
CG13930-RA	CG13930	0,56187	0,526412	0,601077	0,812661	1,47011	0,389397	0,112812	0,733361	0,6279808
CG13931-RA	CG13931	0,311679	6,72631	0	1,44272	0	0	0,134716	0,616952	0,6279808
CG13932-RA	CG13932	0	0	0,29923	0,491246	0	1,47278	-0,070526	0,562659	0,6279808
CG13933-RA	CG13933	5,56032	3,53641	0,0758209	3,31011	0,0432936	0,0326287	0,252727	0,226303	0,6279808
CG13934-RB	Cpr62Ba	0,533752	0,0758333	0,679393	0,100518	0,379086	0,285702	0,579998	0,09143	0,6279808
CG13934-RC	Cpr62Ba	0,0832537	0,0187809	2,48429	0,279871	48,378	0,0220142	0,602095	0,080385	0,6279808
CG13935-RA	Cpr62Bb	24,3594	0,0152654	6,94611	3,13833	0,112758	52,1632	-0,599652	0,052279	0,6279808
CG13935-RB	Cpr62Bb	7,34045	1789,84	2204,05	6,56529	2,91414	0,0849816	-0,598637	0,052687	0,6279808
CG13936-RA	CNMa	1,56496	2,79814	1,94762	10,7447	5,70933	1,73455	0,159428	0,640637	0,6279808
CG13937-RA	CG13937	5,94775	45,9976	3,14406	4,17886	7,25723	5,11074	0,193408	0,429597	0,6279808
CG13937-RB	CG13937	10,4011	4,95565	0,0802318	2,54726	3,55056	1,90793	0,192745	0,428803	0,6279808
CG13937-RC	CG13937	3,92588	12,4001	58,2985	3,16925	1,6099	15,9571	0,190788	0,433972	0,6279808
CG13939-RA	Obp50e	1,45345	2,97612	0	4,7295	133,242	3,78175	-0,071473	0,83313	0,6279808
CG13941-RA	CG1394	0,239695	11,7638	2,85959	13,9408	105,501	0	-0,370033	0,215665	0,6279808
CG13946-RA	Arc2	5,8607	62,2291	2,46743	4,30332	0,616608	1,56871	-0,017683	NA	0,6279808
CG13947-RA	CG13946	2,40612	0,978642	3489,07	4,73114	8,8758	2,32542	0,587839	0,1003	0,6279808
CG13948-RA	CG13947	18,6297	0,49482	1,98256	0,97284	27,7172	8,46277	-0,005902	0,986816	0,6279808
CG13949-RA	Gr21a	0,133877	6,97311	61,939	42,9446	0,723736	0,0368372	0,585789	0,075961	0,6279808
CG1394-RA	CG13949	2,58374	3,48082	2,39921	19,5776	0,0695901	46,5066	-0,110669	0,719826	0,6279808
CG13950-RA	stg	26,4996	15,7736	24,9112	32,7486	13,4161	15,6725	0,113998	0,67256	0,6279808
CG13951-RA	CG13950	2,57078	35,8112	43,2436	39,4801	49,0487	25,2939	-0,078238	0,783562	0,6279808
CG13954-RB	l(2)k10201	4,18605	2,58168	4,89799	0,108282	4,7582	0,710942	-0,096414	0,694581	0,6279808
CG13955-RA	CG13954	1,63702	0,0113374	1,80023	1,1545	3,47748	2,07303	-0,882435	0,013224	0,6279808
CG13956-RA	CG13955	0,202067	46,6243	30,155	33,3804	20,1486	4,26964	-0,476632	0,029055	0,6279808
CG13956-RB	kat80	3,84369	3,09857	3,25603	0,0265958	12,2543	11,4257	-0,492811	0,024159	0,6279808
CG13957-RA	kat80	7,40929	9,51373	6,93123	27,3279	0,0298945	0,0225303	0,022101	0,930065	0,6279808
CG13958-RA	SWIP	1,69027	1,61517	1,98116	2,24125	0,0763139	0,0575148	0,029669	0,933895	0,6279808
CG1395-RA	sick	0,224748	16,5648	0,0549394	15,6227	0,0887607	6,97002	0,212284	0,394082	0,6279808
CG13962-RA	CG13962	1,6555	106,262	8,4306	2,33777	2,60946	3,95494	0,302498	0,388636	0,6279808
CG13964-RA	sick	3,87439	4,59847	3,01618	7,83556	1,97547	2,24001	-0,497385	0,040588	0,6279808
CG13965-RA	CG13965	0,246552	0,224576	0,121806	0,283821	0	5,89512	-0,018589	0,947898	0,6279808
CG13966-RA	CG43861	8,41731	10,8404	0	12,9242	7,75847	4,60008	-0,022737	0,920976	0,6279808

gene_id	Symbol	W1_FPKM	W2_FPKM	W3_FPKM	MCM51_FPKM	MCM52_FPKM	MCM53_FPKM	FC	p-value	p-adj
CG13968-RA	sNPF	0,0269404	0,0245392	3,89999	0,0286048	0,845726	0,505534	0,039037	0,885169	0,6279808
CG13968-RB	sNPF	4,21995	4,32116	0,52864	3,57524	0,0241903	0,147814	0,039037	0,885169	0,6279808
CG13969-RA	bwa	38,4771	45,7559	31,746	43,9689	38,6091	36,4038	-0,075732	0,748176	0,6279808
CG13970-RA	rtv	11,6565	1,18502	11,1521	0	250,081	1,79982	0,531309	0,089761	0,6279808
CG13972-RA	CG13970	0,481135	0	0	49,1876	14,892	22,3156	-0,054838	0,873342	0,6279808
CG13976-RA	CG13972	0,367906	3,25491	71,4569	6,35434	0,252038	0,0523249	0,114498	0,560595	0,6279808
CG13977-RA	Gr98a	0,140467	0,0767685	2,36103	0,702596	0,589294	17,7446	0,642817	0,066906	0,6279808
CG13978-RA	Cyp6a18	0,896009	0,590404	0,0809143	0	0	17,5423	-0,029775	0,862039	0,6279808
CG1397-RA	CG13978	0,0597431	0	0	0,203043	0,793885	0,609354	-0,228568	0,39343	0,6279808
CG13980-RA	CG42534	1,20871	0,127065	19,8211	44,533	1,02426	0,0393741	0,307018	0,37728	0,6279808
CG13982-RA	CG13982	0,0254268	0,0231605	0,0244113	0,0346886	0,0522692	0,0274604	0,724025	0,005543	0,6279808
CG13983-RA	CG13983	2,11685	12,4321	1,29326	2,06865	3,62923	0,819327	-0,226722	0,512278	0,6279808
CG13984-RA	Ddr	0	0	5,36697	7,58621	1,13427	3,94517	-0,041811	0,731452	0,6279808
CG13989-RA	CG13989	0	0	10,2127	16,3779	11,1358	9,63823	-0,041811	0,731452	0,6279808
CG13990-RA	LRR	19,1469	2,20715	5,60325	3,64506	4,69292	4,29883	-0,437883	0,178951	0,6279808
CG13991-RA	LRR	4,7561	0,475574	13,4143	14,0783	18,6301	0,018421	NA	NA	0,6279808
CG13992-RA	Muc26B	50,8642	0,0924524	1,99601	31,0851	11,9065	2,17044	-0,161646	0,648696	0,6279808
CG13993-RA	CG13991	0	2,9954	3,71206	3,78653	0,0591644	3,03413	-0,515613	0,069399	0,6279808
CG13994-RA	CG13992	0,266973	0,165347	2,12996	10,1129	1,39462	0,104826	0,31077	0,240428	0,6279808
CG13995-RA	Pfdn1	91,6183	51,3108	0,0767381	113,486	134,412	147,583	0,504578	0,020464	0,6279808
CG13996-RA	CG13994	16,5885	8,64566	0,0497283	0,0356071	4,62862	1,90624	-0,433513	0,21039	0,6279808
CG13997-RA	CG13995	6,12974	2,89209	4,00853	5,56265	2,75848	3,22017	0,026052	0,900741	0,6279808
CG13998-RA	CG13996	1,11412	6,99077	5,50742	0,726153	0,0445914	3,96609	0,285952	0,290397	0,6279808
CG13999-RA	Vm26Ac	0	6,81918	0,174277	1,07949	0,0755388	0,0486017	0,454967	0,144125	0,13772387
CG1399-RA	CG13998	0,337122	0,818864	0,539429	0,376802	0,396625	0,138257	0,444571	0,122943	0,6279808
CG1399-RB	CG13999	3,24911	3,63346	0,253747	0,287719	5,54724	0,254367	0,304641	0,287749	0,6279808
CG14000-RA	CG14000	0,0477427	0,0946822	1,5083	0,135434	0,860842	0,602984	-0,162742	0,307341	0,6279808
CG14001-RA	bchs	1,88714	0,692253	0,531593	0,891886	0	0	-0,35885	0,205247	0,6279808
CG14005-RA	CG14005	10,8387	29,726	10,292	74,7795	7,83811	5,91489	0,449437	0,188598	0,6279808
CG14006-RA	CG14006	0	0	0	0	0	0	NA	NA	0,6279808
CG14007-RB	CG14007	0,306854	0	0,425531	0,277834	3,64687	0,0342572	-0,110655	0,752641	0,6279808
CG14010-RA	Cul5	5,19423	5,26267	2,36245	7,0258	1,65129	1,98218	-0,196469	0,500081	0,6279808
CG14011-RA	DIP-eta	0,999333	1,01949	0	3,53345	0,093754	0,922131	NA	NA	0,13772387
CG14011-RB	CG14011	0	0	0,104166	3,1803	7,43136	0,0569075	NA	NA	0,6279808
CG14011-RC	CG14011	0	0	0,053669	0	17,3736	8,20697	NA	NA	0,6279808
CG14013-RA	CG14011	0	0,083407	8,35199	0	0	2,13677	0,044335	0,715924	0,6279808
CG14014-RB	CG14013	0	0,0401064	0,0422724	0,0487537	1,17198	0,175413	-0,492311	0,080564	0,13772387
CG14015-RA	CG14014	0,087789	0,082222	0,0866623	21,9538	30,2692	11,2926	-0,531087	0,077607	0,6279808
CG14016-RA	CG14015	1,27518	20,0901	20,3154	0,0424144	15,6908	0,0432982	0,498278	0,159067	0,6279808
CG14017-RA	tomb	0,0902676	5,81755	14,7994	47,9094	14,2945	41,2879	0,076465	0,555158	0,6279808
CG14017-RB	CG14017	0,108499	0,0988284	0,105494	0	0	47,6045	0,044335	0,715924	0,6279808
CG1401-RA	CG14017	0,0559018	0,0509192	1,36238	0	0	0	0,077879	0,735819	0,6279808
CG14020-RA	CARPA	5,48951	0	0	0	0	3,73535	-0,327501	0,324592	0,6279808
CG14021-RA	CG14020	0,740912	1,07366	0,0111163	1,93689	0,0306018	29,9788	0,115954	0,715945	0,13772387
CG14021-RB	fusl	21,2295	3,63946	3,06632	0,339748	12,7314	0,0230633	0,141016	0,658127	0,6279808
CG14022-RA	fusl	13,9849	0,138345	88,7623	0,0118563	2,04806	0,0240444	0,309667	0,379254	0,13772387
CG14023-RA	CG14022	0,112629	5,31995	15,0036	7,90353	1,23144	9,26245	0,086337	0,75129	0,6279808
CG14023-RB	Ncoa6	4,92969	5,2423	4,78234	8,63265	0,820614	2,9176	0,08801	0,746783	0,6279808
CG14024-RA	Ncoa6	0,00801341	0,00729916	0,00769335	15,5928	2,79253	0,00830785	-1,685505	2,85E-09	0,13772387
CG14025-RA	CG14024	3,06079	1,1784	3,47282	9,71046	1,01133	0,023556	0,160055	0,517435	0,6279808
CG14025-RB	Bsg25D	5,55755	3,36247	5,93633	6,34459	15,4965	2,8276	0,156118	0,524671	0,6279808
CG14025-RC	Bsg25D	0,0138533	6,68961	1,75561	2,12036	0,0312555	1,02421	0,178826	0,470267	0,6279808
CG14026-RA	Bsg25D	2,3621	2,34075	0,0139554	2,4152	2,86816	1,62047	0,562456	0,058646	0,13772387
CG14026-RB	tkv	15,7606	23,5936	0,48255	33,7001	0,471972	11,1383	0,562786	0,058572	0,13772387
CG14026-RC	tkv	0,021185	0,0192967	4,97661	0,0225926	0,0160594	0	0,568216	0,055278	0,13772387
CG14026-RD	tkv	0,0224239	0,0204253	21,5812	0,0235536	1,06549	0	0,509253	0,088777	0,13772387
CG14027-RA	tkv	35,71	62,5564	13,6416	30,9352	11,9402	2,21562	-0,225391	0,522072	0,6279808
CG14028-RA	TotM	0,477082	0,706159	0,17176	0,0207173	0,0110233	1,17601	-0,117139	0,68977	0,6279808
CG14029-RA	Dpse\GA12710	181,12	0,10259	0,108131	0,150602	0,203992	0,0505552	0,050387	0,881656	0,6279808
CG14029-RC	vri	2,14413	3,20496	2,59687	1,5424	2,75181	7,25481	0,042217	0,900849	0,13772387
CG14029-RD	vri	10,8247	11,8528	5,64689	7,90524	1,24974	0,021149	0,027741	0,93481	0,13772387
CG1402-RB	vri	0,0198446	0,0180758	0,019052	0,00813827	1,45806	10,6105	0,364819	0,119284	0,6279808
CG14030-RA	37135	0,112056	0,102068	0,10758	1,33866	0,202603	0,152694	0,229248	0,40173	0,6279808
CG14031-RA	37135	21,3232	23,2798	30,4112	5,34973	15,65	25,4806	0,224908	0,417151	0,6279808
CG14032-RA	Bub1	4,85903	5,20421	4,45781	6,70372	2,57714	2,68502	0,052602	0,882118	0,6279808
CG14033-RA	Cyp4ac3	3,58067	6,94022	0,276008	5,13666	8,38239	28,8336	0,43379	0,224058	0,6279808
CG14034-RB	Cyp4ac1	1,07086	0,451579	0	1,21491	80,4707	1,56543	-0,053309	0,873396	0,6279808
CG14035-RA	CR14033	0,0446559	0,0406757	3,42696	0,0495232	30,8128	0,153741	-0,23931	0,503341	0,6279808
CG14036-RA	Dsim\GD22660	0,178906	0,16296	0,229014	0,344628	0,31856	0,240086	0,338226	0,329498	0,6279808
CG14039-RA	Msp300	0,330931	0,57775	0,010769	27,9702	0,963014	0,0117155	-0,058627	0,766725	0,6279808
CG14039-RB	CG14036	0,820091	1,92085	0,562383	1,1545	2,51892	1,89841	-0,050908	0,79717	0,6279808
CG14039-RC	qtc	0,0251845	0,0229398	0,0240048	0,0266281	0,0360679	5,81048	-0,047082	0,812153	0,6279808
CG14039-RD	qtc	3,80965	4,05417	0,0248707	4,77969	0,0360097	0,0280092	-0,046774	0,813403	0,6279808
CG14039-RE	qtc	0,0242866	0,0221219	18,4817	0,0256236	3,58244	6,05835	-0,048513	0,806196	0,6279808
CG14039-RF	qtc	3,27884	4,55489	0,0248512	5,69917	0,0433778	0,0279859	-0,092524	0,636968	0,6279808
CG14039-RG	qtc	0,0287328	0,0261718	36,3194	0,0306399	3,80857	27,1241	-0,040383	0,838598	0,6279808
CG1403-RA	qtc	14,0549	15,6566	0,0398806	19,6117	11,5731	6,35732	-0,121411	0,625465	0,6279808
CG1403-RB	qtc	0,0298086	0,0271518	0,039582	0,0318699	0,043168	0,0462771	-0,382831	0,151296	0,6279808
CG14040-RA	Ran	0,0416707	0	1,89364	123,334	4,54655	0,0320381	0,451106	0,063555	0,6279808
CG14041-RA	Ran	118,469	0	0,497543	0,293704	2,07089	2,47913	0,330871	0,133784	0,6279808
CG14041-RB	senju	11,9738	12,8666	48,0117	0,0470144	0,0636813	0,0479941	0,326203	0,137118	0,6279808
CG14042-RA	CG14042	18,0139	21,7905	0,0245024	17,1366	5,11119	0,0281853	0,330871	0,133784	0,6279808
CG14042-RB	CG14042	0,025885	0,0235779	0,0272964	0,0274146	0,0371333	0,0317059	0,326203	0,137118	0,6279808
CG14043-RA	CG14042	0,0250035	0,0227749	35,9114	5,4451	5,26354	23,9778	0,173923	0,455022	0,6279808
CG14044-RA	CG14042	0,0259053	0,0235964	44,6876	0,0274375	0,0371642	30,4427	-0,103299	0,719836	0,6279808
CG14045-RA	CG14043	8,30944	7,8115	11,207	8,74093	10,5856	0,15098	-0,463098	0,098595	0,6279808
CG14047-RF	CG14044	0,0503627	0,0627813	59,9899	10,3401	6,70389	0,0209971	-0,385706	0,192471	0,6279808
CG14047-RG	PsGEF	0,68645	0,741902	0,972236	1,81474	1,20271	0,890271	-0,385706	0,192471	0,6279808
CG14048-RA	PsGEF	0,181847	0,0127415	0,0134296	0,0144041	0,250186	1,24236	-0,413392	0,188995	0,6279808
CG14049-RA	PsGEF	1,06401	2,00848	1,082	2,74565	1,39841	0,0147171	0,670634	0,023558	0,6279808
CG1404-RA	mRpL14	48,2981	34,867	70,6881	52,6072	91,9554	105,176	-0,124025	0,643316	0,6279808
CG1404-RB	Ilp6	19,7	44,831	22,5408	23,2341	14,1218	15,032	-0,122479	0,647372	0,6279808
CG14050-RA	CG14050	2,66845	3,04791	43,002	50,3956	0,874103	31,6001	0,377549	0,200892	0,6279808
CG14052-RA	CG14052	0,647803	6,46447	0,621929	1,12951	0	0,750651	0,570866	0,102643	0,6279808
CG14053-RA	CG14053	0,355565	30,3727	7,55707	0,866501	0	130,708	0,115445	0,745745	0,6279808
CG14054-RA	CG14054	0,43093	0,392521	10,8115	9,5648	3,63484	7,16374	-0,117135	0,735595	0,6279808
CG14057-RA	CG14057	15,1612	8,87201	14,8454	6,6654	3,31187	19,8401	-0,373499	0,135247	0,6279808
CG14059-RB	Ilp8	2,22773	0,610094	3,92752	7,11387	6,89617	0,0280209	0,017752	0,960299	0,6279808
CG14060-RB	U2A	8,02482	7,72634	13,2122	12,3713	7,882	11,2114	-0,255515	0,416967	0,6279808
CG14061-RA	Exn	4,93819	6,88972	0,748452	2,0719	0,872926	1,1466	NA	NA	0,6279808
CG14062-RB	aqrs	0	92,1045	10,4469	36,9879	11,434	17,3606	NA	NA	0,6279808
CG14064-RA	CG14062	0	0	0	0	0	0	0,058692	0,813673	0,6279808
CG14065-RA	beat-VI	5,92945	7,43407	7,3448	0,098895	20,4528	4,24263	-0,439298	0,160727	0,6279808
CG14066-RA	larp	3,51467	5,12225	5,06145	13,4018	4,60676	0	0,235307	0,449693	0,6279808

gene_id	Symbol	W1_FPKM	W2_FPKM	W3_FPKM	MCM51_FPKM	MCM52_FPKM	MCM53_FPKM	FC	p-value	p-adj
CG14066-RB	larp	90,2802	143,63	0,0173625	107,935	0,0254673	51,588	0,235824	0,448693	0,6279808
CG14069-RA	larp	0,00986845	0,00898887	3,31788	0,0100651	3,35998	0,0102749	0,01562	0,897983	0,6279808
CG1406-RA	CG14069	0	0,0748588	0	0	0	0,23167	-0,10534	0,689912	0,6279808
CG14070-RA	CG1407	0,0365936	0,033332	0,035132	7,04112	9,7614	17,8141	NA	NA	0,6279808
CG14071-RA	CG1407	11,297	8,71761	11,4855	0,052108	0,0705806	0,376816	-0,041811	0,731452	0,6279808
CG14072-RA	CG14070	0	0	0	331,801	553,896	524,797	-0,468294	0,189373	0,6279808
CG14073-RA	CG14071	0	11,7389	16,5276	19,0955	10,1499	1,96312	0,523967	0,039063	0,6279808
CG14073-RB	CG14072	1,03816	0,611877	0,0195571	1,45984	2,3245	1,75188	0,380957	0,135574	0,6279808
CG14074-RA	CG14073	15,4473	16,7692	12,2023	11,659	5,73177	7,22164	0,330051	0,196623	0,6279808
CG14075-RA	CG14073	3,05913	3,4787	2,94618	5,88847	2,15101	2,5042	-0,419046	0,176581	0,6279808
CG14076-RA	CG14074	3,85318	3,41917	9,1016	77,7139	33,8761	1,63464	0,232222	0,345915	0,6279808
CG14076-RB	CG14075	9,74196	1,17648	1,43581	0,0335238	0,0454082	0,0342223	0,227667	0,356843	0,6279808
CG14077-RB	Ir75d	0,0312464	0,0199051	0,0209662	2,78886	0,112758	1,77936	0,227667	0,356843	0,6279808
CG14079-RA	Ir75d	0,0218529	11,2528	0,0299985	8,00716	4,1948	4,8759	0,196976	0,546306	0,6279808
CG1407-RA	CG14077	0,0218384	0,0284614	6,1094	5,39383	0,116133	4,01335	-0,064727	0,744635	0,6279808
CG1407-RB	CG14079	1,6315	32,6406	0,0201643	1,96511	18,4539	1,04932	-0,062875	0,755284	0,13772387
CG14080-RA	Mkp3	20,6356	8,78509	33,3654	1,806	1,19184	12,9533	0,490446	0,021602	0,13772387
CG14080-RB	Mkp3	15,6295	38,6182	0,0256968	0,0537663	1,19126	0	0,280939	0,180158	0,6279808
CG14082-RA	CG14082	1,25521	2003,99	0,046147	29,2275	0,0321332	72,8869	-0,123984	0,698039	0,6279808
CG14082-RB	CG14082	0,0480668	1,01141	0,088847	0,0284075	0,0303211	28,1871	-0,123984	0,698039	0,6279808
CG14084-RA	Bet1	32,4935	30,1102	11,4267	42,0301	0,183825	9,74283	-0,024113	0,923794	0,6279808
CG14084-RB	Bet1	0,104111	0,0948314	9,21276	0,135713	8,47823	0,0185225	-0,023401	0,926025	0,6279808
CG14085-RA	CG14085	0,347061	1,31988	0,0291172	0,30262	21,5012	23,415	-0,146226	0,66975	0,13772387
CG14086-RA	CG14086	1,21719	0,027424	0,0167793	0,0322127	9,92159	5,52226	0,104189	0,761116	0,6279808
CG14087-RA	ms(3)76Ba	0	0	0	3,07796	0,0644403	0	-0,11534	0,404049	0,6279808
CG14088-RA	CG14088	0	0,891652	26,0858	0	0,971616	1,61364	0,044335	0,715924	0,13772387
CG14089-RA	CG14089	0,978902	0,0276254	7,86857	0,0324666	0,0245767	48,5109	-0,503575	0,152301	0,6279808
CG14095-RA	CG1409	0	0	0,252938	0	0,0743128	0	0,70645	0,004825	0,6279808
CG14096-RA	CG14095	138,863	264,204	220,33	105,328	118,019	146,166	-0,518083	0,062261	0,6279808
CG14098-RA	CG14096	41,6325	74,7663	99,0916	174,334	103,902	112,641	0,177712	0,464426	0,6279808
CG14098-RB	Rcd7	0,0372748	4,36896	0,0475656	6,90957	148,484	3,74476	-0,114299	0,710856	0,6279808
CG1409-RB	Rcd7	0,0467077	3,87436	188,851	2,65371	0,192966	2,92666	-0,028286	0,860599	0,6279808
CG14100-RA	waw	1,48068	7,60237	6,4545	8,09592	0,482338	0,919972	1,884172	2,2E-12	0,6279808
CG14101-RA	CG14100	7,86695	18,675	15,2513	20,8711	14,8667	11,5299	NA	NA	0,6279808
CG14102-RA	ms(3)76Ca	0,0508667	7,30531	3,36377	36,5487	4,90793	0,0438915	1,128357	0,000878	0,6279808
CG14103-RA	CG14102	0,0331429	0,0301888	0,016064	5,57635	28,5936	0,0179539	0,017523	0,947234	0,6279808
CG14104-RB	TORIP	13,8515	9,95302	13,576	3,26348	0,0556245	0,0920156	-0,071368	0,792496	0,6279808
CG14105-RA	CG14104	10,7926	0,00954937	22,9879	11,824	0,014504	3,46949	-0,643608	0,012339	0,6279808
CG14106-RA	CG14105	7,69701	1,69296	2,58354	17,5732	19,3139	20,9121	0,309848	0,327994	0,6279808
CG14107-RA	CG14106	0,240495	9,22212	2703,52	0,815499	22,0385	0,448927	0,474245	0,143492	0,6279808
CG14109-RA	CG14107	118,135	1774,2	65,9929	2418,37	53,2741	77,95	0,505267	0,047056	0,6279808
CG1410-RA	CG14109	19,2252	4,09084	27,3119	30,3316	0,0882342	30,3008	1,255735	7,65E-10	0,6279808
CG14110-RA	CRMP	7,53764	6,88857	5,62935	4,41146	2,98469	3,49592	-0,305909	0,337954	0,6279808
CG14111-RA	CRMP	13,4043	10,2655	12,6066	0,0275753	10,6595	9,48474	-0,35661	0,31896	0,6279808
CG14112-RA	CRMP	36,6947	33,8772	31,6849	36,0881	25,7094	24,0704	-0,68031	0,040749	0,13772387
CG14113-RA	CRMP	0,0239174	0,0217856	0,0229621	6,84982	0,0341496	0,0257372	-0,087973	0,472205	0,13772387
CG14115-RA	CG14110	77,7541	1,16688	12,6502	1,8559	4,60681	5,16461	NA	NA	0,6279808
CG14117-RA	CG14111	0,0929337	0,0647397	7,86632	0,0844201	0,17551	0,132275	0,369121	0,164617	0,13772387
CG14118-RA	SNCF	0,268601	237,634	108,733	207,54	1,47736	0,244587	0,372623	0,246993	0,6279808
CG14119-RA	CG14113	0	0	0	0,911974	0	0	-0,479254	0,167414	0,6279808
CG1411-RA	CG14115	0	0	0	0	0	0	0,24753	0,229018	0,6279808
CG1411-RB	CG14117	5,85801	7,51614	5,44262	4,86766	5,97537	6,71059	0,237032	0,248877	0,6279808
CG1411-RC	CG14118	4,20483	13,6686	0,909488	0,0663552	5,10798	23,5623	0,260531	0,209188	0,6279808
CG1411-RE	CG43894	1,88788	4,16743	21,8386	0,314936	4,14614	8,90869	0,245763	0,235238	0,6279808
CG14120-RA	RhoGAP19D	6,14146	8,08963	6,14773	3,4672	0	0,0915763	-0,722971	0,032885	0,13772387
CG14121-RA	CG14120	11,7752	0,674132	17,1924	2,09587	0,264305	0	-0,075433	0,822513	0,6279808
CG14122-RA	ver	1,43421	1,53965	2,45879	2,06452	143,632	2,66075	0,028673	0,889018	0,6279808
CG14125-RA	CG14212	7,26312	10,6088	12,6377	20,96	0,0358794	1,36483	-0,632312	0,076554	0,6279808
CG14126-RA	CG14125	0,21097	0,105884	0,567123	1,29492	0,944863	0,869022	-0,732336	0,036916	0,6279808
CG14127-RA	CG44837	0,228996	3,25876	1,70151	3,31544	12,7752	1,35092	0,105746	0,760438	0,6279808
CG14128-RA	CCDC151	0,537202	0,440389	0,145832	0,457668	0,0870727	0	0,080181	0,741609	0,6279808
CG1412-RA	Sprn	0,056962	0,0518849	0	0	0	0	0,173147	0,512958	0,6279808
CG14130-RA	CG14130	5,66639	6,5658	16,9904	1,61685	8,10323	6,72358	-0,089842	0,735954	0,6279808
CG14131-RA	CG14131	5,08736	2,58955	0,0206657	4,31986	0,0305599	6,0961	-0,33095	0,310874	0,6279808
CG14132-RB	CG14132	44,2354	54,6997	63,5787	1,41891	52,1545	22,6319	0,211044	0,372611	0,6279808
CG14133-RB	Pldn	14,1538	0,429168	12,212	1,12028	15,8789	14,5303	0,008861	0,970599	0,6279808
CG14135-RB	CG14135	8,70904	8,38243	6,22859	0,0668598	247,442	7,1958	-0,073012	0,798593	0,6279808
CG14137-RA	CG14137	0,317673	0,289358	0	0,218053	8,85972	0,899008	-0,104045	0,658861	0,6279808
CG14141-RA	bbx	16,1519	0,0109696	0,011562	0,012345	4,95959	4,82013	-0,516814	0,052963	0,6279808
CG14142-RB	CG14141	4,97832	22,991	6,99148	0	11,355	8,43594	2,154704	2,92E-10	0,13772387
CG14142-RC	CG14142	0,0254366	18,2912	5,9069	4,39966	3,78453	0,0274716	2,121433	5,2E-10	0,13772387
CG14142-RD	CG14142	0,445102	0,0182291	11,1764	11,8473	5,80764	0,182809	2,121433	5,2E-10	0,6279808
CG14145-RA	CG14142	1,64595	0,0189345	0,0250079	3,25716	0,0373822	0,0297695	0,68141	0,005598	0,6279808
CG14147-RA	Blos2	23,7872	20,1836	5,55282	0,0335921	16,0633	0,0342921	1,164154	2,22E-06	0,6279808
CG14149-RA	CG14147	219,848	3,19288	225,488	10,3328	0,0471099	122,741	-0,432311	0,104714	0,6279808
CG1414-RC	Blos4	9,6099	8,23845	5,99996	10,0951	4,34498	6,1737	1,28609	8,99E-10	0,6279808
CG14151-RA	CG14151	0	0,132181	2,79759	0	2,90375	0,0927123	0,01562	0,897983	0,6279808
CG14153-RA	CG14153	4,06071	7,13124	6,11289	4,94943	23,5265	104,569	-0,026299	0,929215	0,6279808
CG14154-RA	CG14154	0,0783197	0,071339	0,0751916	12,7307	37,1571	2,33925	0,109652	0,755601	0,6279808
CG14154-RB	CG14154	0,63807	0,774931	0,530907	0,0189642	33,0519	31,0109	0,109652	0,755601	0,6279808
CG14156-RA	Or67c	0,135959	0,562491	0,0799623	0,02314	0	0,0996494	-0,071941	0,751019	0,6279808
CG14157-RA	Or67d	0,309028	0	0,0184534	0,0280686	0,124824	0,177111	0,034586	0,917699	0,13772387
CG14160-RA	CG1416	8,99248	0,0413747	37,006	0,143159	7,10399	0,0882554	-0,018182	0,951148	0,6279808
CG14160-RB	CG1416	36,3456	0,426959	0,0436091	23,7064	5,04411	16,3271	-0,098264	0,75709	0,6279808
CG14162-RA	CG1416	0,0454233	0	0	45,2363	11,1885	33,3224	-0,300397	0,327086	0,6279808
CG14164-RA	CG14160	0,0395033	0,0359823	0,0379255	6,12539	0,0586589	0,0442089	-0,212888	0,51143	0,6279808
CG14167-RA	CG14160	1,2173	1,386	1,24557	0,0432496	0,600502	0,403646	-0,444628	0,171929	0,6279808
CG14168-RA	dpr6	0,939524	0,96411	0,707898	1,46506	1,53212	1,03192	0,079884	0,644003	0,6279808
CG1416-RA	Hez	0,0953932	0,0868907	0,0915832	0,0221223	0,164208	15,6403	-0,143876	0,426998	0,6279808
CG1416-RB	Ilp3	0	1,82789	435,101	14,9892	26,8703	1,04792	-0,143876	0,426998	0,6279808
CG1416-RC	Zasp67	0	0,0270663	0,0719238	7,29765	0	0	-0,144342	0,42593	0,6279808
CG14173-RA	sIgA	6,43998	0,0176155	13,0246	0,0183823	0,0248989	8,35586	0,044335	0,715924	0,6279808
CG14174-RA	sIgA	0,0178696	0,0162769	0,0171559	0,0204727	0,0277303	0,0189557	-0,159075	0,580286	0,6279808
CG14176-RB	sIgA	0,0178696	0,0162769	0,0171559	14,0317	14,0263	0,0189557	0,116647	0,713459	0,6279808
CG14177-RA	sIgA	0,0174866	0,015928	0,0167881	0,0367797	1,77795	0,0185327	-0,11534	0,404049	0,6279808
CG1417-RA	sIgA	0,0176973	0,0161199	0,0169905	2,24332	0,0522998	0,0187653	0,217296	0,364357	0,6279808
CG1417-RB	sIgA	0,0176973	0,0161199	0,0169905	0,0483601	0,0655041	0,0187653	0,219515	0,359889	0,6279808
CG1417-RC	sIgA	0,0196207	6,01388	0,018837	0,0515778	0,0698624	0,0208993	0,309299	0,199939	0,13772387
CG1417-RD	sIgA	31,0136	21,2517	22,4773	18,921	2,91319	12,5023	0,219358	0,360306	0,6279808
CG1417-RE	Ilp1	0,213148	0,0271029	0	1,12052	0	0,050385	0,217142	0,36477	0,6279808
CG1417-RF	CG14174	3,72562	0,0858986	15,3226	13,0936	9,32294	6,6427	0,219173	0,359762	0,6279808
CG1417-RG	Or67b	0,0483364	0,0440282	0,0464058	0,0482017	0,0652895	0,0492061	0,220199	0,357437	0,6279808
CG1417-RH	Tmc	0	0	0	22,48	0	0	0,217413	0,364302	0,6279808
CG14180-RA	Dpse\GA12807	12,615	2,12346	14,1347	63,8612	4,79034	11,3354	0,881788	0,005104	0,6279808

gene_id	Symbol	W1_FPKM	W2_FPKM	W3_FPKM	MCM51_FPKM	MCM52_FPKM	MCM53_FPKM	FC	p-value	p-adj
CG14181-RA	CG44838	2,25653	3,82095	1,91644	2,13044	3,4226	5,11511	0,185535	0,424499	0,6279808
CG14182-RA	Use1	9,68663	11,3981	11,5618	11,7944	42,3142	0,0565002	0,067265	0,780296	0,6279808
CG14183-RA	CG14182	9,90487	79,2035	10,5728	29,2041	0	12,6491	0,905626	0,003683	0,6279808
CG14184-RA	CG14183	1,26647	0,809733	0,946992	55,1873	1,7005	1,16042	-0,059836	0,781597	0,6279808
CG14185-RA	CG14184	15,15	14,631	20,0942	0,0533311	0,045247	0,485446	-0,12696	0,722427	0,6279808
CG14186-RA	CG14185	0,32792	0,298692	0,60341	0	7,19505	4,97508	-0,182476	0,494948	0,6279808
CG14187-RA	CG14186	5,25968	6,61107	5,36634	0,40233	296,245	1,9747	NA	NA	0,6279808
CG1418-RA	CG14187	0	0	0	5,56268	0	0,456337	0,357022	0,173167	0,6279808
CG14190-RA	CG14190	0	0	0	11,237	0,0954021	0,0693793	NA	NA	0,6279808
CG14191-RA	CG14191	105,454	43,1541	165,681	43,5847	38,3672	44,4929	0,556971	0,01979	0,6279808
CG14193-RA	HP1D3csd	1,57902	12,2755	17,6526	15,0085	8,77633	26,5056	-0,022982	0,943359	0,6279808
CG14194-RA	CG14194	13,9448	13,8665	17,6759	1,94589	15,4563	11,6616	0,194957	0,398674	0,6279808
CG14195-RA	CG14195	1,45045	2,57709	2,0286	20,116	3,96177	2,637	-0,486295	0,076237	0,6279808
CG14196-RA	CG14196	1,28267	13,4215	2,75464	5,41149	1,25568	1,53238	-0,484015	0,159544	0,6279808
CG14199-RB	ksh	21,2163	20,6802	32,3197	34,9431	27,0694	33,3815	0,21282	0,394478	0,6279808
CG14200-RA	Slu7	8,54844	18,3405	19,3234	1,77379	1,86225	1,23344	-0,28477	0,268978	0,6279808
CG14204-RA	CG14200	3,86902	4,21325	3,48622	7,41128	3,19085	3,33054	0,316892	0,272183	0,6279808
CG14205-RA	CG14204	0,0740097	0,0404479	0,363535	0,0412462	0,0311355	1,44229	-0,550744	0,123481	0,13772387
CG14206-RB	CG14205	6,83397	3,73107	2,6432	4,47166	0,0522386	0,0393701	-0,643889	0,028343	0,6279808
CG14206-RC	RpS10b	0,0953932	9,8887	11,886	4,76553	18,727	2,30441	-0,643889	0,028343	0,6279808
CG14207-RA	RpS10b	1556,66	2,6504	2,72717	11,4248	1,45642	16,0114	0,108516	0,653557	0,13772387
CG14207-RB	CG14207	292,447	0,051237	394,411	0,0486099	21,5525	0,0496228	0,107975	0,655279	0,6279808
CG14208-RA	CG14207	0,0562507	11,0719	0,054004	27,5661	0,279327	19,6528	0,031635	0,879175	0,6279808
CG14208-RB	Tyler	0,47407	0,0200845	0,0211691	1,72273	0,43085	0,0243843	0,031635	0,879175	0,6279808
CG14209-RD	Tyler	0,0227247	0,0206992	0,0218171	0,02314	2,37109	6,07497	0,010563	0,960412	0,6279808
CG1420-RA	Shawn	5,12657	6,985	7,46475	0,0238866	0,0313433	0,931838	0,120863	0,600976	0,6279808
CG14210-RA	CG1421	0	0,0648561	0	0	0	0	-0,432381	0,161492	0,6279808
CG14211-RB	CG14210	45,9917	25,8715	48,091	19,9458	11,6784	19,1659	0,401715	0,09527	0,6279808
CG14212-RA	MKP-4	4,27336	4,96449	5,12498	4,97338	5,36134	2,7395	0,567067	0,070366	0,6279808
CG14213-RA	CG14212	0,757751	0,862765	1,49135	0,20622	0,0658424	0,210518	0,32652	0,097	0,13772387
CG14213-RB	Rcd-1	0,0439139	0,0399998	0,04216	12,6756	1,51192	293,183	0,32652	0,097	0,13772387
CG14214-RA	Rcd-1	25,1851	25,7502	31,0963	3,38825	2,25554	0,0655908	-0,178263	0,474349	0,6279808
CG14215-RA	Sec61gamma	261,32	5,17974	12,7542	0,0352641	1,23464	287,758	-0,003458	0,989877	0,6279808
CG14216-RA	Elys	2,27505	7,97354	205,464	3,87427	13,7505	8,01199	0,016196	0,942769	0,6279808
CG14217-RA	Ssu72	8,08969	2,26191	3,46503	12,102	6,64559	7,78633	0,060767	0,807854	0,6279808
CG14217-RB	Tao	11,4094	10,3629	12,8702	11,3996	7,28256	10,6543	0,060988	0,807663	0,6279808
CG14217-RD	Tao	0,03019	3,50614	0,0289842	5,71355	2,31037	0,0199331	-0,083911	0,74157	0,6279808
CG14217-RE	Tao	3,4636	2,87261	0,0175014	0,0189588	0,057303	3,75993	-0,107225	0,674563	0,6279808
CG14218-RA	Tao	3,92415	4,4793	6,8354	14,9694	3,69925	4,06685	0,170349	0,626944	0,6279808
CG14219-RA	CG14218	0,189329	0,258681	0,227372	0,0385665	1,86874	1,2021	-0,186719	0,537037	0,6279808
CG1421-RA	CG14219	1,6405	0,152196	3,58746	22,1977	3,87425	0,0228596	NA	NA	0,6279808
CG14220-RA	p115	10,8805	9,10126	12,0832	0,208835	7,68614	10,3361	-0,081055	0,694218	0,6279808
CG14221-RB	CG14220	10,5338	13,4972	13,6212	17,0712	10,7984	11,7331	-0,590651	0,056617	0,6279808
CG14222-RA	CG14221	0,697147	0,495617	0,816222	1,23757	1,23642	0,0236222	0,301033	0,232221	0,6279808
CG14223-RA	Naa20A	20,0206	17,5057	28,331	8,09964	0,0323545	1,07918	-0,105832	0,687066	0,6279808
CG14224-RA	CG14223	2,68693	200,018	10,3893	4,12085	0,0477654	3,87156	-0,158416	0,399182	0,6279808
CG14225-RA	Ubqn	35,1896	5,17256	5,23772	6,02168	30,8985	64,2641	-0,615843	0,014993	0,6279808
CG14226-RA	et	2,32901	0,0298297	0,0314406	280,026	5,40799	12,2617	-0,247648	0,319525	0,6279808
CG14227-RB	dome	7,74295	10,4941	12,24	13,0968	4,64606	7,18129	-0,378972	0,267159	0,6279808
CG14228-RA	CG14227	0,708209	36,2961	7,97716	53,2242	297,335	31,6202	0,178069	0,508693	0,6279808
CG14229-RA	Mer	6,78989	8,5934	9,05748	8,9261	2,25511	0,0531938	0,458751	0,084542	0,13772387
CG1422-RA	CG14229	9,78431	10,8052	39,6299	11,0469	5,11985	5,8582	-0,086909	0,688065	0,6279808
CG14230-RA	CG14230	5,41796	0,0425747	0,0448739	0,052108	5,37306	6,54285	0,028371	0,921782	0,6279808
CG14231-RA	CG14231	3,6506	4,34647	10,4349	6,27494	5,31258	5,9733	-0,306096	0,217046	0,6279808
CG14232-RA	CG14232	6,36725	65,1883	75,6385	100,871	5,37699	6,84814	0,116694	0,605645	0,6279808
CG14233-RA	meso18E	14,0122	1958,75	3008,64	35,8839	3,94311	3,46714	0,266165	0,347044	0,6279808
CG14234-RA	CG14234	4,25475	29,2932	3,9888	25,4816	14,8712	19,6027	-0,088917	0,717323	0,6279808
CG14235-RA	Dpse\GA12848	59,8981	0	0	250,341	0,0713708	0,0104978	-0,259739	0,382151	0,6279808
CG14235-RB	Dpse\GA12848	195,902	0,315443	0,398974	185,263	0,0706365	3,31917	-0,259726	0,382457	0,6279808
CG14235-RC	Dpse\GA12848	67,1591	50,3593	0,3776	0,0526914	1,53976	1,37102	-0,259717	0,382366	0,6279808
CG14237-RA	CG14237	0,123583	0,112568	0,118647	37,1958	0,231507	32,0601	-0,000881	0,997629	0,6279808
CG14238-RA	CG14238	0,252198	0,287149	0,423719	0,785192	1752,55	0,496339	-0,670068	0,056098	0,6279808
CG14239-RB	ppk15	0,273041	0,0765082	0,28398	0,354136	0	0	1,17227	0,000577	0,6279808
CG14240-RA	mst	2,21526	1,66414	48,0204	1,08248	0	0,360923	-0,828984	0,014303	0,6279808
CG14242-RA	TwdlR	121,49	74,214	219,544	175,789	474,191	365,805	-0,01765	0,960276	0,6279808
CG14243-RA	TwdlS	0,673262	0,56945	0,737637	0,835782	0,445071	1,0412	0,840109	0,001058	0,6279808
CG14244-RA	TwdlD	814,367	0,0301586	7,24792	5,64098	2,44592	2,24186	NA	NA	0,6279808
CG14245-RA	CG14244	0	0	5,79703	5,09301	41,0423	4,09459	-0,169743	0,634728	0,6279808
CG14247-RA	CG14245	1,18306	9,32499	0	0,176332	1,36701	1,17909	-0,042191	0,903272	0,6279808
CG1424-RA	CG14247	3,1741	9,51367	100,486	2,73964	336,182	146,388	-1,276717	3,82E-07	0,6279808
CG14250-RA	TwdlQ	61,8678	1,06821	2,03904	0	0	0,701754	-0,406269	0,193508	0,6279808
CG14251-RA	ms(3)K81	0	0,162666	1784,23	0,747844	1,16012	21,9975	-0,070621	0,690495	0,6279808
CG14252-RA	CG14252	5,2798	5,76152	5,87192	1,07468	5,4947	4,876	0,029608	0,899746	0,6279808
CG14253-RA	CG14253	19,6857	0,0288849	18,8995	18,997	13,3458	17,7425	-0,464085	0,077739	0,6279808
CG14253-RB	CG14253	0,0317113	0,0284614	0,0304448	0,034061	1,57786	0,0347707	-0,44738	0,088991	0,6279808
CG14253-RC	CG14253	0,0312464	0,0262174	0,0299985	0,0335238	11,5157	0,0342223	-0,465665	0,077417	0,6279808
CG14253-RD	CG14253	0,0287829	22,2241	0,0276333	6,42248	58,0133	0,0313366	-0,46387	0,078474	0,6279808
CG14253-RE	CG14253	29,8342	9,34781	27,599	26,7729	36,4369	34,3364	-0,465695	0,07745	0,6279808
CG14254-RA	TwdlC	117,381	20,2957	139,523	105,45	0,337573	1,50539	1,040738	0,000147	0,6279808
CG14257-RB	CG14257	31,9148	46,7146	37,6186	1,59018	0,821716	0,0209383	0,689759	0,018661	0,6279808
CG14258-RA	CG14258	0,382679	0,30984	9,73178	0	0,679087	3,15734	-0,277865	0,428472	0,6279808
CG14259-RA	CG14259	0,455696	0	0	0,724804	0	0,0201662	-0,432713	0,213315	0,6279808
CG14260-RA	CG14260	0,0762412	15,6974	0,0731961	29,0076	0,124388	15,1214	0,765379	0,010131	0,6279808
CG14262-RA	CG14262	0,188609	2,98638	0,0905378	5,76434	2,10684	4,2296	-0,291093	0,356997	0,6279808
CG14264-RA	CG42534	0,210433	0	0,336713	35,7466	0	0	0,074056	0,711926	0,6279808
CG14265-RB	CG14265	213,24	0,379327	372,961	290,362	617,702	615,574	-0,594059	0,084079	0,6279808
CG14266-RA	ng2	0	0,779393	0,115972	0,456272	0	0	-0,041811	0,731452	0,13772387
CG14269-RA	CG14269	0,145756	12,0286	18,198	0	0	0	0,093528	0,580553	0,6279808
CG14270-RA	CG1427	16,7278	20,1256	0,0285408	4,83017	12,4372	0,0324399	-0,550569	0,070468	0,6279808
CG14271-RB	CG14270	40,967	28,2294	47,639	3,81477	2,48211	1,14151	-0,009735	0,971803	0,6279808
CG14273-RA	Gas8	1,49506	1,29505	1,50571	1,37883	1,44505	1,37202	-0,114605	0,695487	0,6279808
CG14274-RA	CG14273	6,6505	64,2778	0,0228711	0,0205173	35,8368	27,3265	-0,195494	0,503324	0,13772387
CG14275-RA	CG14274	6,24027	11,8049	25,3029	20,4885	33,177	87,9526	-0,710812	0,033153	0,6279808
CG14275-RB	CG14275	0,0832537	6,76776	46,6503	7,72062	86,4407	43,3915	-0,706498	0,034305	0,6279808
CG14277-RA	CG14275	49,265	4,91756	0,0208559	50,5517	0,0311682	5,30835	-0,183881	0,532848	0,6279808
CG1427-RC	CG14277	16,1871	16,2327	25,2473	19,0898	30,8744	24,8581	0,37425	0,068537	0,6279808
CG14280-RA	CG1428	0,129307	12,3262	0	0	0,0263937	0	0,3166	0,225302	0,13772387
CG14282-RA	CG14280	0,0940359	0,091841	0,0902801	0	35,4969	0	-0,16242	0,488922	0,6279808
CG14283-RA	CG14282	0,106118	0,161099	0,0925181	6,37551	419,757	0,137928	-0,191647	0,520104	0,6279808
CG14285-RA	mRpL55	37,2586	116,079	7,72712	34,9588	0,0575617	0,043382	-0,349069	0,251215	0,6279808
CG14286-RA	CG14285	7,49629	0,0171098	10,831	15,5445	246,27	8,48494	-0,151403	0,63831	0,6279808
CG1428-RA	CG14286	11,1708	7,73939	20,0828	9,15774	17,4325	17,7181	0,277715	0,348261	0,6279808
CG14290-RB	Mef2	4,28994	3,45651	0,01647	3,91556	134,15	0,0181673	-0,123602	0,684243	0,6279808
CG14291-RA	Mef2	15,0289	17,4663	0,0344079	19,9205	62,5642	0,0396954	-0,294567	0,344806	0,6279808

gene_id	Symbol	W1_FPKM	W2_FPKM	W3_FPKM	MCM51_FPKM	MCM52_FPKM	MCM53_FPKM	FC	p-value	p-adj
CG14292-RA	Mef2	0,0175796	0,0160127	0,158989	0,018255	5,01252	0,213186	-1,126251	0,000787	0,6279808
CG14294-RA	Mef2	10,9349	8,07334	13,726	8,44107	27,9551	0,203511	0,01205	0,95129	0,6279808
CG14296-RA	Mef2	0,0171551	0,0156261	3,76829	4,97424	13,8255	6333	0,32145	0,159265	0,6279808
CG14296-RB	Mef2	0,0358394	0,032645	14,7858	0,0388851	0,0304972	0,114684	0,321635	0,158788	0,6279808
CG14297-RA	Mpc1	64,3241	39,4991	99,8466	58,6452	2,36916	2,56152	-0,269559	0,325184	0,6279808
CG14298-RA	Dvir\GJ14185	11,1541	9,80566	10,7255	25,6039	5,57824	10,9803	1,314219	3,58E-05	0,13772387
CG14299-RB	Dpse\GA12884	35,8931	24,7991	90,9456	126,527	9,58612	7,79127	-0,165012	0,548909	0,6279808
CG14299-RC	CG14294	0	0,11007	0	0	1298,91	1216,39	0,187418	0,382279	0,13772387
CG1429-RA	EndoA	0,0179278	0,0163299	0,0172117	0,0186318	0,0252369	0,01902	0,122803	0,597079	0,13772387
CG1429-RB	EndoA	41,7842	46,9077	36,0721	41,1542	27,2781	22,5253	0,123323	0,595847	0,6279808
CG1429-RC	Dpse\GA12886	0,0957622	6,25809	5,91591	0,255808	4,88658	163,752	0,123323	0,595847	0,6279808
CG1429-RD	Dpse\GA12887	20,5717	16,1476	0,0118514	4,50347	1,16931	0	0,123705	0,594851	0,6279808
CG1429-RE	Epg5	1,82339	1,41316	1,84044	1,80547	0,630568	1,21897	-0,395148	0,111456	0,6279808
CG1429-RF	Epg5	0,0179181	0,016321	0,0172024	0,0186212	0,160808	0,0190093	0,118055	0,585535	0,6279808
CG14300-RA	bys	14,5023	9,25483	1,76854	2,4334	1,83703	1,41158	-0,671745	0,057011	0,6279808
CG14301-RB	CG14300	33,8018	15,3945	26,3606	0,0390233	0,0317108	168,666	0,219955	0,46994	0,6279808
CG14302-RA	CG14301	6,98639	4,62222	7,37637	0,0187271	6,33982	4,49655	-0,62497	0,076525	0,6279808
CG14303-RA	CG14302	138,961	0,175129	0	0,899962	0,0252226	0,261138	0,383934	0,21915	0,6279808
CG14304-RA	qin	0,012562	3,6493	0	0,019457	6,73142	1,78099	0,06771	0,808216	0,13772387
CG14304-RB	CG14304	4,37643	4,7888	0,0120602	5,91626	0,0670211	0	0,066941	0,810313	0,6279808
CG14305-RA	CG14304	0,0186884	0,0170227	9,12298	3,81108	14,9452	17,2825	-0,074864	0,550506	0,6279808
CG14305-RC	CG14305	0	0	2,51007	0,156227	0,026223	6,21218	-0,074864	0,550506	0,6279808
CG14306-RB	CG14305	0	0	1,63272	20,1824	0,0260851	3,14918	0,738	0,034206	0,6279808
CG14307-RA	qin	0,199504	247,877	0,0498325	2,06878	4,12761	6,85424	-0,432663	0,226524	0,6279808
CG14307-RB	fru	0,0146099	0,021406	44,7031	0,0247479	0,0263547	52,0769	-0,413881	0,247217	0,6279808
CG14307-RC	fru	0,497548	0,0119283	0,0140264	0,0134568	0,143615	2,43401	-0,449634	0,209026	0,6279808
CG14307-RD	fru	0,247612	0,0174724	0,966207	0,0199943	0,0875671	0,0235231	-0,435159	0,223862	0,6279808
CG14307-RE	fru	0,0151562	0,0367738	0,0264136	0,0442963	1,18592	86,9172	-0,449634	0,209026	0,6279808
CG14307-RF	fru	0,0239521	0,16507	0,0145508	0,582162	5,26503	0,0398365	-0,413881	0,247217	0,6279808
CG14307-RG	fru	0,0234755	0	0,0229954	0	27,6428	28,9105	-0,456828	0,201821	0,6279808
CG14307-RH	fru	0,293961	0,0410362	0,0225379	0,0130285	203,916	0,0645212	-0,456828	0,201821	0,6279808
CG14307-RI	fru	0,033443	0,0166907	0,639698	12,5607	0,0312118	0,0698839	-0,447738	0,210885	0,6279808
CG14307-RJ	fru	0,0235006	0,0166078	0,0321072	2,93847	107,475	0,0711404	-0,447738	0,210885	0,6279808
CG14307-RK	fru	0,0130956	0,0659456	0,022562	0,0193599	0,0528573	9,48634	-0,44859	0,210056	0,6279808
CG14307-RL	fru	0,0191821	25,8178	0,0125725	0,019258	24,0412	3,41493	-0,406759	0,255747	0,6279808
CG14307-RM	fru	0,0403722	0,0184057	0,0184159	0,0862998	0,0856105	0,01538	-0,447738	0,210885	0,6279808
CG14309-RA	fru	0,271834	0,0182218	0,0387597	25,7468	0,092726	0,898123	0,115723	0,739606	0,6279808
CG1430-RA	CG14309	0,0180158	0,399789	0,306458	0,061551	1,50545	1,21696	-0,086266	0,777213	0,6279808
CG14312-RA	CG14312	23,2377	16,289	211,468	0,870444	60,92	60,0553	-0,080169	0,790732	0,6279808
CG14313-RA	CG14313	0,404877	4,30943	0,0461806	1,7468	1,73713	0,0228751	0,238957	0,49808	0,6279808
CG14314-RA	CG14314	4,36697	4,49819	4,17296	5,44052	3,60809	4,33293	0,051102	0,823267	0,6279808
CG14315-RA	CG14315	0	0,0614148	0	0	112,648	3,17839	NA	NA	0,6279808
CG14316-RA	CG14316	0	0	0,0647314	0	0	0	0,017269	0,912244	0,6279808
CG14317-RA	CG14317	0,0735812	0,24575	89,6963	0,230341	0,252038	102,68	-0,063043	0,837079	0,6279808
CG14318-RA	CG43102	0,980376	4,55131	0,0196237	17,9721	1,22124	1,15748	-0,255191	0,386196	0,6279808
CG14321-RA	CG14321	12,818	0,796904	2,21947	6,23085	2,86965	0,848511	0,012827	0,963332	0,6279808
CG14322-RA	CG14322	2,91068	0,851151	3,48482	14,0361	0,802323	73,9102	0,096041	0,67581	0,13772387
CG14323-RA	CG14323	0,730697	0,394235	15,5527	10,9247	0	0,186322	-0,035978	0,919835	0,13772387
CG14324-RA	CG14324	3,10182	0,121344	13,6431	0	24,6344	20,5418	-0,322186	0,287917	0,6279808
CG14325-RB	CG14325	0,382218	143,353	0,298148	1,59719	54,8394	2,03032	-0,076173	0,831444	0,6279808
CG14326-RA	CG14326	5,58613	1,09033	0,851837	10,004	149,714	1,28561	-0,405575	0,255808	0,6279808
CG14327-RA	CG14327	70,4634	0,998354	82,8132	1,25937	0,35806	0,427341	0,074628	0,814785	0,6279808
CG14329-RA	CG14329	0,510586	0,519792	0,692038	0,451192	0,94243	0,298936	-0,313447	0,381158	0,6279808
CG14330-RA	Atu	4,74182	0,0648793	8,76714	0,084637	0,114641	5,57985	NA	NA	0,6279808
CG14331-RB	CG14330	0	0	0	16,1718	0,537966	0	-0,102089	0,755561	0,6279808
CG14331-RC	CG14331	2,09588	2,26875	0,629557	2,02075	2,23739	2,43849	-0,053636	0,860348	0,6279808
CG14332-RA	CG14331	0,0952557	0,0867655	0,00985706	0,121009	2,10319	0,12353	0,01562	0,897983	0,6279808
CG14333-RA	CG14332	0	0,158385	0,166939	0,019394	0,206828	0,177012	-0,021923	0,941926	0,6279808
CG14334-RA	lute	8,98119	14,3162	0	0,0152227	5,12659	21,4643	0,344933	0,101292	0,6279808
CG14339-RA	beat-IIa	17,1218	18,2857	15,1424	12,9577	3,78962	9,53008	-0,031954	0,870687	0,6279808
CG1433-RA	CG14339	0,0302638	0	0,05811	7,16746	2,80026	0	-0,100827	0,693624	0,6279808
CG14340-RA	CG1434	4,2938	17,4245	22,9534	28,4358	14,1366	6,5973	0,025482	0,880577	0,6279808
CG14341-RA	CG14340	0	5,28729	6,5912	0,126307	4,12711	4,56044	-0,035833	0,898679	0,6279808
CG14341-RB	CG14341	0,101033	9,99497	0,0969982	11,8261	5,83327	0,148974	-0,035833	0,898679	0,6279808
CG14342-RB	CG14341	7,453	11,4023	13,5097	4,53048	0,197667	13,5796	0,08146	0,779256	0,6279808
CG14346-RA	CG14342	9,9961	3,903	0,0171327	3,12086	1,42612	2,63646	-0,64676	0,066922	0,6279808
CG14346-RB	CG14346	0,0835696	0,076121	0,0802318	0,102712	0,139124	0,104852	-0,64676	0,066922	0,13772387
CG1434-RA	CG14346	0,350225	0,239257	0,630444	1,04526	0,953964	0,923858	-0,142619	0,577592	0,13772387
CG14351-RA	CBP	291,943	0,00936074	9,91109	0	7,78298	0,041355	-0,27387	0,321336	0,6279808
CG14352-RA	CBP	0,0372118	16,176	19,008	0	16,3812	15,342	-0,390115	0,126848	0,6279808
CG14353-RA	haf	15,0917	16,1551	12,2532	30,2297	11,019	11,9597	-0,350796	0,210396	0,6279808
CG14354-RA	CG14352	5,78649	5,44741	4,77948	7,53598	7,25129	0,0425048	NA	NA	0,6279808
CG14355-RA	Wdr92	3,42681	8,81601	3,65549	4,11488	0,0311573	4,09006	-0,281416	0,390229	0,6279808
CG14356-RA	CG14354	0	0	0	0,182182	0	0	0,823986	0,004577	0,6279808
CG14357-RA	CG14355	0,107999	0	0,0754078	0,0164543	0,0323311	0,0243667	-0,084142	0,773164	0,6279808
CG14358-RB	CG14356	6,97418	47,564	0,0152725	0,0303296	0	0	-0,681606	0,020598	0,6279808
CG14359-RA	CG14357	0	0,474106	70,9062	1,29613	22,9493	15,9026	-0,129731	0,708481	0,6279808
CG1435-RA	CCHa1	4,94808	0,876502	0,835852	1,12038	0,937997	37,3423	0,105143	0,677005	0,6279808
CG1435-RB	CG42500	1,18973	0,01449	0,110006	20,3359	8,2743	11,0947	0,088293	0,726449	0,6279808
CG14360-RA	Or88a	0,41092	0,248704	23,1906	1,09068	102,644	1,13336	-0,038711	0,912052	0,13772387
CG14362-RA	CG14362	0,159604	0,0301737	0	5,31896	0,683132	41,4379	-0,013096	0,914398	0,6279808
CG14367-RA	CG14367	6,43478	7,14533	4,20509	6,99819	4,08532	3,20684	0,139305	0,551141	0,6279808
CG14368-RA	CG14368	0	0	0	0,0158222	0	143,221	NA	NA	0,13772387
CG14369-RA	CG14369	3,411	44,4303	0,113754	13,6978	0,218473	0,164654	-0,422847	0,236987	0,6279808
CG14370-RA	CG14370	0	1,33156	5,98644	3,93814	5,41954	5,50036	NA	NA	0,6279808
CG14372-RB	side-IV	7,11849	0	4,95892	0	0	6,47138	-0,216043	0,396923	0,13772387
CG14374-RA	CG14374	28,9633	10,5326	13,6755	19,8683	0,0376661	28,804	-0,7595	0,032021	0,6279808
CG14375-RA	CCHa2	16,4621	19,3716	84,0538	24,1655	39,231	26,0724	-0,609267	0,039286	0,13772387
CG14376-RA	Ir87a	0,723259	118,84	1,45425	4,59043	0,247242	2,36983	0,755486	0,0276	0,6279808
CG14377-RA	CG14377	37,9209	13,5325	105,529	195,494	302,965	218,377	0,852564	0,016373	0,6279808
CG14379-RA	CheA87a	0,318182	0,0966075	0,111357	0,156691	0,212239	0,159956	-0,617859	0,066793	0,6279808
CG14380-RA	Cyp4c3	34,1837	32,3051	0	0	0,147194	0	NA	NA	0,6279808
CG14383-RA	CG14380	0	5,48901	9,60856	8,1117	8,26109	0,136991	0,010648	0,975624	0,6279808
CG14384-RA	CG14383	0,297639	0,0930716	0,285751	12,2228	0,0439329	0,1352	-0,49054	0,123094	0,6279808
CG14387-RA	CG14384	0,977179	2,41594	1,65293	0,00874298	0,179391	3,1957	-0,013096	0,914398	0,6279808
CG1438-RA	d-cup	0,150172	0	0	0	0	0	-0,560642	0,056675	0,6279808
CG14390-RA	beat-Vc	3,37042	3,74827	0,0840222	0,130344	0,176552	0,121742	-0,538483	0,056236	0,6279808
CG14391-RA	CG14391	0,116604	291,3	316,701	0,0261214	0,0353815	26,4816	0,237559	0,498436	0,6279808
CG14394-RB	NijC	0,103893	0,0946328	0,0896	0,198756	0,269216	0,13306	-0,647807	0,015514	0,13772387
CG14394-RC	NijC	0,102922	0,0937484	0,118795	0,130862	0,177253	0,202897	-0,726209	0,006608	0,6279808
CG14394-RD	NijC	0,0875177	0,0797172	0,0898538	0,0392324	0,0531406	0,133589	-0,390509	0,116944	0,6279808
CG14394-RE	NijC	0,0933275	0,0850092	0,0346902	73,6175	61,9031	0,0400499	-0,287463	0,247897	0,6279808
CG14394-RF	NijC	0,123738	0,112709	61,4609	2,12973	2,08871	55,0551	-0,234968	0,342122	0,6279808
CG14394-RG	NijC	0,0935919	0,08525	3,00902	8,64125	8,13605	1,733	-0,401367	0,128169	0,6279808

gene_id	Symbol	W1_FPKM	W2_FPKM	W3_FPKM	MCM51_FPKM	MCM52_FPKM	MCM53_FPKM	FC	p-value	p-adj
CG14395-RA	CG14395	8,56919	13,1589	6,6978	0,769687	3,24293	76,073	-0,032249	0,925073	0,6279808
CG14396-RA	Ret	0,0137467	0,0125215	0,0131977	110,412	0,0191627	3,42104	-0,757804	0,002066	0,13772387
CG14396-RB	Ret	0,57697	0,0126637	0,0133476	14,5462	0,0193875	0,0141773	-0,757804	0,002066	0,6279808
CG14396-RC	Ret	2,23427	3,29742	2,39619	2,91249	3,71908	0,0138049	-0,757804	0,002066	0,13772387
CG14396-RD	Ret	0,0135023	0,0122988	0,012963	0,0141474	0,0188113	0,0237977	-0,757804	0,002066	0,6279808
CG14396-RE	Ret	0,0131582	0,0119854	0,0126326	0,0143133	0,0183171	92,9282	-0,757804	0,002066	0,13772387
CG14397-RA	CG14397	21,2614	7,13083	10,626	8,12076	9,78656	7,97185	0,017531	0,953068	0,6279808
CG14400-RA	Dsim\GD16898	29,0076	26,9569	37,8189	47,1818	18,5341	29,2965	0,423488	0,08577	0,6279808
CG14401-RA	Dsim\GD16898	3,47563	3,81719	4,5023	5,91413	0,0485713	0,0366062	0,12272	0,659377	0,6279808
CG14402-RA	Dsim\GD16898	0,0344923	0,031418	0,0331147	0,0373003	0,0505234	0,0380775	-0,041811	0,731452	0,6279808
CG14405-RA	CG14400	0,077584	0,0225869	20,209	24,1552	14,6086	0,0598163	-0,029462	0,915943	0,6279808
CG14406-RA	CG14401	0,0534162	0,777971	12,2497	0,013501	0,0314759	0,0137823	0,929832	0,006715	0,6279808
CG14407-RA	CG14402	0	1,90881	0	0	14,9474	11,1682	-0,522385	0,077607	0,6279808
CG14408-RA	CheB38c	0,16602	0	0,318778	0,250526	0,550792	0,0719398	-0,175295	0,466868	0,13772387
CG14408-RB	CG14406	3,02146	0,174041	22,0229	3,07359	1,69398	2,62361	-0,116582	0,62966	0,6279808
CG14408-RC	CG14407	46,8079	46,2001	12,586	18,6102	13,6008	0,0236056	-0,365744	0,113684	0,6279808
CG14408-RD	Dsim\GD15836	5,6593	6,3466	5,78379	4,80369	0,0313213	5,01769	-0,380232	0,099467	0,13772387
CG1440-RA	Dsim\GD15836	2,76099	2,75099	3,02938	3,18286	0,0311573	2,81203	0,052456	0,840536	0,6279808
CG1440-RB	Dsim\GD15836	0,0271284	0,0247105	0,0260449	1,80946	0,0276787	96,0112	0,037754	0,88534	0,6279808
CG1440-RC	Dsim\GD15836	0,920495	0,0194988	0,0205519	0,022431	6,49167	11,8116	0,037754	0,88534	0,6279808
CG14410-RA	CG1441	42,1296	57,5537	92,9217	118,093	7,60244	15,7476	0,002872	0,993595	0,6279808
CG14411-RA	CG1441	86,4719	115,272	0,146033	70,6898	0,0675875	0,342141	-0,038292	0,882958	0,6279808
CG14411-RB	CG14410	1,1994	0,8442	1,04681	2,36318	1,81997	0,666939	-0,038292	0,882958	0,6279808
CG14411-RC	Dsim\GD17180	0,0220351	0,0200711	0,021155	0,0231238	5,39404	6,02908	-0,15811	0,547371	0,6279808
CG14411-RD	Dsim\GD17180	0,0219254	0,0199712	0,0210497	5,32111	3,55803	3,25741	-0,15811	0,547371	0,6279808
CG14413-RA	Dsim\GD17180	2,42864	7,68907	0,0188035	0,0204346	1,62059	0,0294179	-0,276192	0,332615	0,6279808
CG14414-RA	Dsim\GD17180	6,86367	4,70669	9,07933	11,497	0,030383	0,0228985	-0,187322	0,409437	0,6279808
CG14414-RB	mRpS25	39,3309	41,3185	76,3381	0,0603116	86,2404	8,27949	-0,185412	0,414544	0,6279808
CG14414-RC	Dsim\GD17174	3,38995	2,58603	5,20728	3,86018	3,20403	0,118315	-0,187322	0,409437	0,6279808
CG14416-RA	Dsim\GD17174	0,0920702	0,0838639	2,65179	0,307078	5,33802	13,7764	0,001231	0,996794	0,6279808
CG14417-RA	Dsim\GD17174	11,049	16,8874	11,4168	0,230227	4,12208	3,76713	-0,016227	0,943227	0,6279808
CG14418-RA	CG14416	0,212599	0,580951	0	0,783151	0,0305599	9,33569	-0,615733	0,05841	0,6279808
CG14419-RA	CG14417	0,0998125	0,181832	0,204108	2,36962	0,0303623	0,024807	0,278707	0,405903	0,6279808
CG1441-RA	CG14418	0,167876	0,183495	0,290107	0,201395	0,0348565	12,9063	-0,40311	0,086344	0,6279808
CG1441-RB	CG14419	54,5058	21,5716	0,0210288	0	7,24969	35,7971	-0,400508	0,088826	0,6279808
CG14420-RA	eIF4E6	0,390404	0,0394751	0,176213	0,471694	0,064885	0,0489013	0,001902	0,993945	0,6279808
CG14421-RA	CG14420	0,0625719	0,019833	0,36556	0,0228353	0	0,219262	-0,185507	NA	0,6279808
CG14422-RA	CG14421	0	0,0194862	0,221188	0,0224158	0	0,388511	-0,39607	0,088963	0,6279808
CG14423-RA	CG14422	0	20,0005	0,126874	30,3395	0,148755	0,412632	-0,617405	0,047967	0,6279808
CG14424-RA	CG14423	0	0,0222118	0,354792	0,0257337	0	0,196099	-0,65121	0,034998	0,6279808
CG14425-RA	CG14424	0,208573	0,0189883	0,120145	0,0218147	0	0	0,064914	0,815694	0,6279808
CG14426-RB	Sxl	21,4428	0,0214594	0,0226183	7,53274	0,124561	73,4093	-0,013096	0,914398	0,6279808
CG14427-RA	nullo	0,116194	0,0972839	59,3454	0,140338	5,21121	3,45446	-0,027405	0,910359	0,6279808
CG1442-RA	CG14427	0,14216	0	1,18182	5,77235	0,208763	0,157337	-0,105139	0,758211	0,6279808
CG14430-RA	wat	28,4614	0,278641	3,33585	3,7289	0,890788	7,61999	-0,089793	0,659932	0,6279808
CG14431-RA	bou	65,7983	81,5974	82,4501	119,355	7,92791	95,3811	-0,026047	0,933583	0,6279808
CG14434-RA	CG14431	0,558913	0,0145636	18,4647	0,0123728	1,95831	0,0168857	-0,736207	0,013409	0,6279808
CG14435-RA	CG14434	3,71111	5,21856	5,5312	4,32644	0,0130302	0,00982035	0,141873	0,493217	0,6279808
CG14437-RA	Dsim\GD24583	8,94404	7,98879	4,98638	0,0096199	2,47746	6,93567	-0,01851	0,948965	0,6279808
CG14438-RA	Coq7	9,74768	13,5216	19,2168	12,6379	0,526763	17,2069	0,140646	0,480624	0,6279808
CG14438-RB	sov	0,00641534	0,511309	0,517365	1,05411	0,292434	0,00662672	0,143164	0,473375	0,6279808
CG14439-RA	sov	3,27473	2,63948	3,61285	2,91048	1,99359	2,55895	-0,538898	0,06015	0,6279808
CG1443-RA	CG14439	19,0589	14,5062	30,5614	26,888	0,103717	28,8262	0,318887	0,141389	0,6279808
CG14440-RA	spidey	41,7331	40,7053	53,4884	49,5371	14,2393	52,4514	0,475994	0,033454	0,6279808
CG14441-RA	CG14440	3,7619	43,3837	5,57174	6,10984	3,39477	3,23144	-0,320257	0,242138	0,6279808
CG14442-RA	CG14441	3,10475	3,08703	0,0412016	0,0473863	0,0797222	0,0249543	0,1181	0,617444	0,6279808
CG14443-RA	CG14442	13,1052	67,7285	14,6264	6,5669	2,45553	2,54197	0,528929	0,072634	0,6279808
CG14444-RA	CG14443	0,301029	16,524	10,9939	6,28137	3,19447	0	-0,204186	0,542785	0,13772387
CG14444-RB	APC7	0,029595	0,0269572	0,028413	0,0316252	0,0428365	0,0322842	-0,204186	0,542785	0,6279808
CG14445-RA	APC7	0,0915741	0,125118	0,190486	0,141225	0,255254	0,118917	-0,684423	0,024902	0,6279808
CG14445-RB	CG14445	0,0497309	0,0452983	27,5025	25,5214	35,4432	29,9742	-0,684746	0,024836	0,6279808
CG14446-RA	CG14445	10,254	9,10164	32,841	23,808	34,2827	29,4707	-0,346492	0,242207	0,6279808
CG14447-RA	dtn	2,85829	3,80056	13,6465	13,6194	28,3815	9,16227	0,007384	0,974888	0,6279808
CG14448-RA	Grip	12,2607	17,2202	10,1421	17,2517	13,0422	14,9531	0,047726	0,877437	0,6279808
CG1444-RA	CG14448	0,243224	0,110773	482,5	5,80495	6,39842	0,241419	-0,230332	0,306502	0,6279808
CG14450-RA	CG14450	5,27811	7,5981	0,194591	0,377615	0,0199464	0,164724	0,357011	0,142474	0,6279808
CG14451-RA	CG14451	0,540243	0,302825	0,718152	0,526678	0,0494321	0,626057	0,257156	0,460258	0,6279808
CG14452-RA	CG14452	830,581	0,103829	23,3548	31,0511	53,2442	51,5749	-0,378928	0,288109	0,6279808
CG14453-RA	CG14453	277,696	188,459	568,326	458,306	1066,86	936,457	-0,669412	0,045778	0,13772387
CG14454-RA	CG14454	85,9527	76,2878	187,457	186,528	518,985	458,979	-0,673099	0,029893	0,6279808
CG14455-RA	CG14455	0,632538	0,13296	0,794128	1,65932	3,80584	2,7515	-0,516743	0,146208	0,6279808
CG14456-RA	CG14456	0,0881401	0,0802841	9,00714	4,28028	1,80805	0,112011	-0,137092	0,657094	0,6279808
CG14456-RB	CG14456	10,7834	13,0679	4,23025	1,2374	0,148622	15,8015	-0,138536	0,653645	0,6279808
CG14457-RA	CG14457	10,5463	8,44558	0,0846198	0,109724	25,2773	1,05868	0,905346	0,000437	0,6279808
CG14457-RB	CG14457	2,52164	0,0483554	23,4061	15,4789	2058,2	2031,66	0,929661	0,000405	0,6279808
CG14459-RB	CG14459	0	0	0	0	0,701242	0	-0,68518	0,015701	0,6279808
CG14463-RA	PIG-H	13,1496	11,7121	8,81261	14,5778	11,3951	11,1667	-0,128316	0,604468	0,6279808
CG14464-RC	CG14464	115,757	87,1025	0,0294826	0,0344528	15,0304	0,0316165	-0,143074	0,623547	0,6279808
CG14468-RA	Tsp42A	0	86,8972	6,56623	0,0990832	7,90571	0	NA	NA	0,6279808
CG14470-RA	Ptx1	0,0232853	4,77325	0,0496764	6,75451	21,8798	40,1857	0,241744	0,4953	0,6279808
CG14471-RB	Ptx1	6,23916	0,609624	43,2611	1,83387	0,107593	14,2147	-0,261707	0,398099	0,6279808
CG14472-RA	Ptx1	0,027524	1,73914	30,3585	2,25835	0,234575	52,3316	0,004534	0,986199	0,6279808
CG14476-RA	Ptx1	13,6052	0	0,0223553	0,0361145	16,6186	0,893143	-0,187861	0,421501	0,13772387
CG14476-RB	CG43366	7,62102	10,9005	3,15749	3,4019	0,1202	12,4428	-0,187254	0,422968	0,6279808
CG14476-RC	Src42A	2,99435	11,2548	2,83106	7,51846	2,1591	2,40137	-0,187288	0,422772	0,6279808
CG14476-RD	poe	3,78521	4,19971	4,66445	6,66682	2,50633	2,59565	-0,18818	0,420255	0,6279808
CG14476-RE	GCS2alpha	0,0214207	0,019461	0,0205652	0,0224463	0,0304037	4,83761	-0,193551	0,407739	0,6279808
CG14478-RA	GCS2alpha	0,0205801	30,503	0,0197581	0,0215225	0,0291524	0,021971	0,394081	0,193829	0,6279808
CG14478-RB	GCS2alpha	0,0213653	0,0195305	0,020512	0,0223853	0,0303211	0,0228518	0,399806	0,187378	0,6279808
CG14479-RA	GCS2alpha	34,4564	14,1968	39,8508	61,5724	27,9812	27,5122	0,073278	0,830392	0,6279808
CG1447-RA	GCS2alpha	0,0214416	1,31186	6,24761	0,0224693	0,0304348	0,0229375	0,189068	0,495061	0,6279808
CG1447-RB	CG14478	15,944	0,124524	2,53216	0	0,0271566	1,91417	0,438341	0,107868	0,6279808
CG1447-RC	CG14478	25,5579	16,5935	0	52,8693	1,39887	0,0365069	0,319908	0,236247	0,6279808
CG1447-RD	CG14479	0,14882	0,135555	0,142876	0,27282	5,03788	16,0432	0,235294	0,401789	0,6279808
CG14480-RA	Inx3	72,7411	74,1088	71,6528	114,101	32,2511	52,2733	0,09368	0,6479	0,6279808
CG14481-RA	CG14480	16,2906	13,6958	20,6432	16,369	13,6608	15,7748	-0,822883	0,005393	0,6279808
CG14482-RA	CG42562	12,8159	26,793	11,6857	12,3596	0,0523612	3,26948	-0,58699	0,052388	0,6279808
CG14483-RA	Dpse\GA13018	432,741	342,451	430,725	1031,51	13,9043	11,7928	-0,652114	0,037638	0,6279808
CG14485-RA	CG14483	87,8133	0,0315995	0,0333059	0,037534	8,16905	6,05497	0,334829	0,176885	0,6279808
CG14487-RA	swi2	9,1755	7,74955	6,9895	0,0783939	2,17726	9,96044	-0,031266	0,929276	0,6279808
CG14488-RA	Ir54a	0,0386936	10,2336	0,0371482	5,48442	0,215104	5,82831	-0,12928	0,696996	0,6279808
CG14489-RA	CG14488	0,135493	0,123417	0,130082	0,194646	0,263649	0,198702	-0,757565	0,001588	0,6279808
CG1448-RA	olf186-M	15,4013	8,91652	0,0132362	0,0272898	4,84141	0,0653995	0,175231	0,532973	0,6279808
CG14490-RA	zfh2	10,9606	11,4697	0,0255416	13,7203	14,3911	10,3122	0,30482	0,293823	0,13772387

gene_id	Symbol	W1_FPKM	W2_FPKM	W3_FPKM	MCM51_FPKM	MCM52_FPKM	MCM53_FPKM	FC	p-value	p-adj
CG14491-RA	CG14490	3,33717	4,45826	5,12621	5,81463	2,62153	4,05161	-0,270968	0,4139	0,6279808
CG14492-RA	CG14491	0,32972	0,0400709	0,633102	0,248635	10,0936	0,901525	0,001529	0,994312	0,6279808
CG14495-RA	CG14492	0	0,65482	0,26432	58,8355	18,4139	0,408844	-0,512907	0,132029	0,6279808
CG1449-RA	CG14495	6,00561	2,44826	5,16095	0,0459649	0	7,84858	0,162936	0,58568	0,6279808
CG14500-RA	CG14500	2,34589	0,40065	10,9325	9,41745	38,3188	20,6369	-0,456232	0,141215	0,6279808
CG14502-RA	CG14502	0,678241	0,658974	0,0863475	1,2054	0,153478	0,534556	0,468388	0,189138	0,6279808
CG14502-RB	CG14502	0,273837	0,401859	1,08525	24,1074	0,423878	0,121081	0,485801	0,161293	0,6279808
CG14503-RA	Tango8	0	0,0938459	0	5,1536	0,379796	5,26099	-0,106269	0,42424	0,6279808
CG14505-RB	CG14505	0,103029	0	0,368819	0,67927	0	0	-0,355726	0,164755	0,6279808
CG14506-RA	CG14506	0	0	0,0675818	0	3,98234	0,180339	-0,179194	0,350844	0,6279808
CG14507-RC	CG14507	2,31863	2,30908	4,60045	0,238744	0	2,48594	0,266874	0,362567	0,6279808
CG14508-RA	Dpse\GA13039	0,89777	12,5004	0,0441555	85,1002	0,0693198	0,0522436	-0,150559	0,660868	0,6279808
CG14509-RB	jus	7,42525	102,86	5,03483	7,12846	30,8661	0,0232627	0,282631	0,234223	0,6279808
CG14509-RC	jus	4,28462	0,0335737	8,17789	0,164105	1,37175	9,3723	0,148059	0,554673	0,6279808
CG14511-RA	Apc	8,94052	0,0169253	0,0177893	12,5255	0,0265349	0,0199983	0,374821	0,265019	0,6279808
CG14512-RA	CG14511	3,71634	5,15884	7,13582	1,43003	2,60388	0,0523249	-0,174105	0,560361	0,6279808
CG14513-RA	CG14512	0,119992	10,8387	7,92059	27,3053	3,89874	7,53385	0,113749	0,725502	0,6279808
CG14514-RA	yem	2,89677	5,01114	3,03872	1,3704	0,575535	1,87855	0,094223	0,741606	0,6279808
CG14515-RA	Dsim\GD21428	3,63015	4,37289	5,04043	116,499	0	2,75774	0,931499	4,03E-05	0,6279808
CG14516-RA	CG14515	28,8812	35,8464	21,3392	2,39456	2,55093	17,8163	-0,631333	0,034199	0,6279808
CG14516-RB	CG14516	1,54352	1,07033	1,36116	6,04792	0,0694277	100,121	-0,618286	0,036729	0,6279808
CG14517-RA	CG14516	0,478856	0,248021	4,11293	1,73839	1,01861	0,0870717	-0,229533	0,362057	0,6279808
CG14518-RA	beta4GalNAcTB	5,55302	0,0417768	8,99951	0,0510187	16,85	0,0520818	-0,006194	0,974196	0,6279808
CG14519-RA	CG14518	0	5,68454	26,3065	8,39788	3,77821	1,06315	NA	NA	0,6279808
CG1451-RA	CG14519	0	0	0	0	0	0	0,125412	0,636544	0,6279808
CG14521-RA	DIP-gamma	3,13802	0,622312	0,0416069	0,0479031	0	0	0,162951	0,491756	0,6279808
CG14523-RA	Nepl19	12,797	0,0285695	41,7372	0,0221744	0,0300354	0,0230792	-0,656795	0,009769	0,6279808
CG14526-RA	Nepl21	4,2082	0,0206142	0,0203279	0,00945155	0,0128022	6,09569	-0,312413	0,213733	0,6279808
CG14527-RA	Nepl20	3,11056	0,0202194	0,0207062	9,8662	6,18028	0,0226365	0,12567	0,652003	0,6279808
CG14528-RA	Nepl18	18,3863	0,0278169	0,0310406	0,0226081	0,0306228	17,773	-0,386506	0,08382	0,6279808
CG14529-RA	Nepl17	2,35755	4,66471	0,0217274	30,9769	14,2093	0,0355049	-0,724787	0,035521	0,6279808
CG14531-RA	Klp10A	0,0182749	0,016646	0,017545	0,019008	2,84099	19,5668	0,179364	0,309056	0,6279808
CG14532-RA	Klp10A	0,0187414	0,0170709	0,0179928	0,0195147	0	0,557437	-0,06067	0,755026	0,6279808
CG14534-RA	Klp10A	9,3386	8,43415	5,02416	7,3336	0,223667	2,24653	0,965064	0,000362	0,13772387
CG14535-RA	Klp10A	0,0191821	2,19278	3,68319	3,12772	1,78366	17,2989	-0,262984	0,322888	0,6279808
CG14536-RA	Klp10A	0,0207481	0,0188988	0,0199194	4,78841	1,49941	116,707	0,285952	0,14024	0,6279808
CG14536-RB	CheB98a	0	0,258533	32,9339	37,9454	20,5005	0,0494768	0,338769	0,080214	0,6279808
CG14537-RA	CG14532	0	5,64473	183,957	0,0538982	0,0730055	0,0550213	NA	NA	0,6279808
CG14538-RA	TwdlE	81,2316	68,5992	74,9922	15,9258	16,3868	9,97665	0,02628	0,904878	0,6279808
CG1453-RA	CG14535	1,73167	2,2951	1,89601	7,98393	2,09058	1,35629	-0,056227	0,814448	0,6279808
CG1453-RB	Herp	0,0295421	22,3279	0,0283622	18,9117	33,5055	16,962	-0,057727	0,809532	0,6279808
CG1453-RC	Herp	18,0363	7,12333	21,576	13,6907	8,76166	7,76693	-0,05827	0,807728	0,6279808
CG1453-RD	CG14537	0	0	0	0	0	0	-0,058197	0,807606	0,6279808
CG1453-RE	CG46025	0,427676	1,43208	0,0893476	0,524991	6,12262	0,415171	-0,129638	0,585314	0,6279808
CG14540-RA	wdn	7,44415	8,9497	39,1983	42,9632	19,1598	0,0499174	0,044335	0,715924	0,6279808
CG14542-RA	CG14540	0,0538077	0,0490118	0	0,33763	0	2,81195	-0,367621	0,122746	0,6279808
CG14543-RA	Vps2	27,4077	25,8115	35,2625	36,5748	40,7615	35,5532	0,000544	0,998645	0,6279808
CG14544-RA	CG14543	12,164	10,0767	22,299	12,8786	13,1675	23,2128	-0,092807	0,779279	0,6279808
CG14545-RA	CG14544	3,8133	0,0288295	0,0303864	4,11765	0,0460406	0,034699	-0,497857	0,139443	0,13772387
CG14546-RA	CG14545	1,16842	0,990875	1,89537	2,05847	2,52994	0	-0,041811	0,731452	0,13772387
CG14548-RA	CG14546	0,0414269	8,91652	0,0397723	0,0413114	224,055	0,00774001	0,773107	0,002755	0,6279808
CG14549-RA	Dsim\HLHmbeta	28,7352	25,2696	36,4848	55,6548	6,14957	28,3714	-0,182251	0,600713	0,6279808
CG14549-RC	Sld5	0,0316506	0,0288849	0,0304448	0,0339907	0,0461358	0,0347707	-0,299326	0,309686	0,6279808
CG1454-RA	Sld5	0,101085	2,88021	3,92554	0,130603	5,75859	5,40564	0,211964	0,410015	0,6279808
CG14550-RA	CanA1	8,26891	0,0250707	1,53211	0	0	1,63786	0,257027	0,374898	0,6279808
CG14551-RA	PIG-P	7,07954	6,40897	12,9681	7,68884	6,77268	8,45121	0,358939	0,252044	0,6279808
CG14556-RB	CG14551	2,83494	48,6732	91,3044	2,95857	92,6833	93,7666	-0,218129	0,282552	0,6279808
CG1455-RA	CG14556	0	138,837	0,0166166	7,39185	6,97001	0,0181918	-0,700047	0,03264	0,6279808
CG14560-RA	msopa	0	0,614148	0	0	13,5495	0	-0,073806	0,645207	0,6279808
CG14561-RA	CG14561	2,44726	2,84507	2,56147	2,1253	2,41401	3,59915	-0,103232	0,712987	0,6279808
CG14562-RA	CG14562	3,57602	3,03418	3,43991	17,3165	0,229309	13,53	0,05804	0,819983	0,6279808
CG14563-RA	CG14563	4,74529	14,3627	3,8681	7,61678	17,4825	14,1044	-0,697291	0,043108	0,13772387
CG14564-RA	CG14564	1,52175	1,05782	1,61475	2,35765	5,34684	4,93323	-1,084459	0,001597	0,6279808
CG14565-RA	CG14565	104,744	114,39	150,453	168,971	2,45462	4,74928	-1,104545	0,000848	0,6279808
CG14566-RA	CG14566	31,9169	27,6631	58,7518	15,227	170,996	363,531	-0,82963	0,013227	0,6279808
CG14567-RA	CG14567	13,7226	18,5391	21,1456	160,363	457,653	9,39934	0,970454	0,000172	0,6279808
CG14568-RA	CG14568	144,712	232,867	223,906	164,284	8,2944	437,503	-0,565983	0,093102	0,6279808
CG14569-RA	CG14569	138,848	176,834	236,656	0,0267361	592,023	443,232	-0,761035	0,022872	0,13772387
CG14570-RA	CG14570	40,1001	85,3928	78,4373	4,72208	0,0362142	0,0272932	0,286635	0,309855	0,6279808
CG14572-RA	CG14572	34,3171	160,601	61,893	67,8344	235,344	169,911	-1,312476	0,000123	0,6279808
CG14573-RA	CG14573	101,567	26,9097	206,635	57,8384	575,267	111,029	-0,553003	0,095139	0,13772387
CG14574-RA	CG14574	0	24,4199	0,0216311	0	2,88215	0,0239502	-0,041811	0,731452	0,6279808
CG14575-RB	CapaR	0,857272	0,946045	0,0216532	404,256	0,0287486	38,8017	0,042449	0,894543	0,6279808
CG14577-RA	ORMDL	13,8546	12,9433	6,86424	9,61283	37,9203	24,1537	-0,143724	0,622314	0,6279808
CG14578-RA	CR14578	0,607645	73,1129	70,4694	0,0773812	0,104813	0,0796484	-0,75842	0,033262	0,6279808
CG14578-RB	CR14578	0,0660317	0,105282	0,110968	1,28184	0,105682	0,0776262	-0,75842	0,033262	0,6279808
CG14578-RC	CR14578	0,0664969	0,553485	0,939882	0,0760417	0,102999	1,52251	-0,75842	0,033262	0,6279808
CG14578-RD	CR14578	0,0650565	0,0601463	0,0633944	53,069	51,667	38,0284	-0,75842	0,033262	0,6279808
CG14579-RA	CG14579	0,142898	3,09391	0,384162	18,9337	13,5154	3,45007	-0,095457	0,663849	0,6279808
CG14581-RA	Cisd2	92,6531	86,089	1,37682	1,79565	0,473369	0,598478	1,562272	7,6E-06	0,6279808
CG14584-RA	CG14581	6,89681	4,05296	0,0216977	84,8326	0,0255402	126,681	0,005934	0,96996	0,6279808
CG14585-RB	Ir20a	0,117156	0	0	0,142976	33,9317	0	0,714664	0,031291	0,6279808
CG14586-RA	Ir75a	0,157324	5,40163	0	14,6006	0,0526864	0	0,083264	0,631854	0,6279808
CG14586-RC	Ir75c	0,0177878	0,0366841	0	0,0408879	0,329322	0,0331579	0,114961	0,588589	0,6279808
CG14589-RA	Ir75c	0,0495519	0,148243	9,11822	0,0418487	0,0439958	8,27417	0,655259	0,066357	0,6279808
CG1458-RA	CG14589	0,474797	0,0126107	0,645764	28,5047	2,25061	0,159516	-0,207102	0,47783	0,6279808
CG14590-RA	SmydA-5	0,86018	1,4391	25,3747	0,034061	12,8024	7,02221	0,143884	0,652073	0,6279808
CG14591-RA	CG14591	0,0393621	6,64001	2,69503	4,89896	0,0461358	8,83718	0,075237	0,759013	0,6279808
CG14591-RB	CG14591	9,04006	0	0,0377899	0,0430799	10,486	1,10264	0,075237	0,759013	0,6279808
CG14593-RA	CCHa2-R	1,33646	7,20126	1,14604	13,2516	0,0583521	17,0977	-0,183437	0,491748	0,6279808
CG14598-RA	CG14598	18,4906	13,3478	35,002	18,7246	20,3082	0,0403624	-0,055126	0,854212	0,6279808
CG14605-RA	CG14605	0,13424	0,0933603	0,0127699	23,0683	5,44915	0,135746	0,16464	0,548092	0,6279808
CG14605-RB	CG14605	0,0429157	7,3915	156,698	0	0	0	0,16464	0,548092	0,6279808
CG14605-RC	CG14605	0,0430274	0,111045	0,0349385	0	0	0	0,16464	0,548092	0,6279808
CG14606-RA	CG14606	0,802744	714,165	3,39582	0,0496351	0	0	0,156854	0,661091	0,6279808
CG14607-RA	CG14607	0,0834291	0,163033	0,0412016	9,04594	3,29149	11,2573	-0,022326	0,921866	0,6279808
CG14608-RB	thw	5,52575	0,034857	18,5492	0,119439	12,1659	0	0,243124	0,36641	0,13772387
CG14609-RA	Dpse\GA13108	0,198924	0	0	0,125824	1,51268	0	0,148779	0,677603	0,6279808
CG14610-RA	CG1461	2,92117	16,1572	7,61611	0,0580277	6,62458	16,6726	0,0799	0,654847	0,6279808
CG14612-RA	CG14610	0,244726	0	6,26021	0,487911	4,84433	0,18736	1,033527	0,003118	0,6279808
CG14613-RA	e(y)2b	2,63845	1,46287	4,84587	3,95661	2,37618	1,79083	-0,132842	0,55029	0,6279808
CG14614-RA	CG14613	8,58014	11,3806	13,1417	11,3151	6,4149	4,82274	-0,092563	0,665095	0,6279808
CG14615-RA	wap	13,8106	14,4748	14,8892	9,21141	9,79953	10,1887	-0,209004	0,440627	0,6279808
CG14616-RG	CG14615	5,24993	8,11137	20,6983	0,0215296	8,39407	6,10484	-0,087496	0,651536	0,13772387

gene_id	Symbol	W1_FPKM	W2_FPKM	W3_FPKM	MCM51_FPKM	MCM52_FPKM	MCM53_FPKM	FC	p-value	p-adj
CG14616-RH	l(1)G0196	3,62808	8,43603	0,01407	9,39999	7,09595	3,27129	-0,170802	0,390167	0,6279808
CG14616-RI	l(1)G0196	5,88715	0,0190799	7,95216	0,0143789	0,0194763	11,5435	-0,1271	0,494477	0,6279808
CG14616-RJ	l(1)G0196	0,0146553	0	0,0134069	0	0	0	-0,088674	0,645943	0,13772387
CG14616-RK	l(1)G0196	6,85548	0	0,0359212	147,428	54,5108	69,7125	-0,134047	0,483367	0,6279808
CG14617-RA	l(1)G0196	0,0139646	0	0,251733	0	0	0	-0,056115	0,782192	0,6279808
CG14617-RB	Cp110	0,027728	0,0218067	0,0256829	0,0257639	0,0348973	0,0154294	-0,049955	0,805934	0,6279808
CG14617-RC	Cp110	0,0251814	0,0207397	0,0229843	0,0244087	0,0330617	6,72805	-0,135512	0,532942	0,6279808
CG14617-RD	Cp110	0,0267514	0,00979172	0,0218597	4,46508	4,92818	0,0146785	-0,104369	0,630265	0,6279808
CG14617-RE	Cp110	0,0239405	10,2749	4,05596	8,52242	3,66576	8,70538	-0,043987	0,829682	0,6279808
CG14618-RA	Cp110	0,0227691	0,0133491	6,10059	0,0151144	0,0204726	11,1856	0,044936	0,854216	0,6279808
CG14619-RA	CG14618	0,0585951	0,0533725	0,0562548	19,9064	0,0912098	0,0687412	0,077945	0,699551	0,6279808
CG14619-RB	Usp2	8,41473	6,84486	9,61599	0,067338	8,23935	8,00241	0,233386	0,257549	0,6279808
CG14619-RC	Usp2	0,0205865	2,78462	0,0197643	36,6996	0,0291619	0,0219782	0,121732	0,566323	0,6279808
CG14619-RD	Usp2	0,0194531	0,0177193	0,0186762	33,0171	0,0274827	0,0207126	0,081483	0,68698	0,6279808
CG14619-RE	Usp2	3,99858	5,04675	5,55412	14,0212	3,98773	3,88433	0,080014	0,692248	0,6279808
CG1461-RA	Usp2	13,8056	15,7136	12,566	11,3285	7,5452	7,02424	-0,051681	0,883522	0,6279808
CG14620-RA	Dsim\GD21558	2,08975	3,71172	5,02325	15,2231	1,54519	0,0368671	0,100925	0,751672	0,13772387
CG14621-RA	Dsim\GD21558	0,0334787	4,96267	0,0321416	1,7986	0	3,49942	-0,051267	0,821417	0,6279808
CG14622-RA	tilB	1,26448	3,8256	0,0180749	0,0202898	8,97756	14,037	0,377848	0,203133	0,6279808
CG14622-RB	CG14621	10,1408	16,3824	14,5626	13,6696	8,85022	7,28718	0,320008	0,275797	0,6279808
CG14622-RC	DAAM	11,4825	0,0251545	9,00522	0,0293698	0,0397815	3,98027	0,320208	0,276569	0,13772387
CG14624-RA	DAAM	4,35392	26,6307	3,42059	32,8629	18,3186	3,31247	-0,257741	0,247987	0,6279808
CG14625-RB	DAAM	0,0124304	0,0257354	1,31273	0,0300941	0,0407627	1,5005	0,26248	0,446161	0,6279808
CG14626-RA	CG14624	0	0,130274	0	0,150865	9,29797	0,154009	0,706073	0,010608	0,6279808
CG14627-RA	CG14625	0,189121	0,844789	0,181567	3,31259	0,0647207	0,205694	0,248116	0,443476	0,6279808
CG14628-RA	CG14626	9,90671	12,4076	3,30441	10,7419	0,386462	116,998	-0,117355	0,598471	0,6279808
CG14629-RA	CG14627	0,447008	0,0127496	0,663238	0,431812	0,472486	4,78786	0,253963	0,282565	0,6279808
CG1462-RA	CG14628	0,201861	0,183869	0	0,913731	0	0,421043	-0,631647	0,074862	0,6279808
CG1462-RB	CG14629	151,847	227,022	208,697	227,335	113,846	168,977	-0,589332	0,096723	0,6279808
CG14630-RA	CG1463	33,377	28,3731	26,8408	17,6258	16,8347	19,408	-0,458736	0,154015	0,6279808
CG14631-RA	CG14630	16,682	4,38639	26,4982	0,0347251	390,657	3,89187	-0,041811	0,731452	0,6279808
CG14632-RA	CG14631	0	0	0	0	1,37596	0	-0,365861	0,28561	0,6279808
CG14632-RB	CG43867	0,0532583	0	3,33529	0	1,43547	0,0616248	-0,365861	0,28561	0,6279808
CG14634-RB	CG43867	0,648694	12,4558	0,0511311	20,0036	1,69121	0,945145	-0,0276	0,879281	0,6279808
CG14635-RA	CG14634	0	0,00849891	0	8,62893	0	0,320796	-0,041811	0,731452	0,6279808
CG14636-RA	CG14635	0	9,29946	0,299796	0,739442	6,33662	0	0,412365	0,057541	0,6279808
CG14636-RB	CG14636	0,0467077	0,0425446	0,0448422	0,0520668	0,0307176	0,0531518	0,41222	0,05759	0,6279808
CG14637-RA	CG14636	54,1725	65,9695	56,4065	45,1898	0,0705249	43,3211	-0,153164	0,544431	0,6279808
CG14639-RA	abs	4,72443	4,60427	8,43709	8,19922	5,17171	5,63338	0,45722	0,110779	0,6279808
CG1463-RA	TwdlF	548,409	175,823	188,338	8,74636	0,032261	79,8797	0,576402	0,013258	0,6279808
CG14640-RA	ey	0,0236944	0,0180541	0,557327	0,0249635	0,0338132	0,0254837	0,758599	4,86E-05	0,6279808
CG14641-RA	ey	1,87052	9,08919	1,398	2,58277	1,94656	1,11559	-0,0602	0,776736	0,6279808
CG14642-RA	ey	1,33457	17,5418	0,0213544	0,0233532	0,0316321	0,906119	0,097326	0,748307	0,6279808
CG14642-RB	ey	0,0232935	0,0195813	2,0015	2,98682	0,80887	0,672537	0,074455	0,809015	0,6279808
CG14643-RA	TwdlV	144,257	0,233885	141,114	137,501	37,9598	0	0,740456	0,000313	0,6279808
CG14644-RA	CG14641	27,7824	26,7329	39,554	37,9291	24,8894	30,832	0,353094	0,307018	0,6279808
CG14645-RA	CG14642	0,0467628	0,0425948	0,0448951	54,8742	103,43	0,0239417	0,134046	0,65419	0,6279808
CG14646-RA	CG14642	0,0469957	0,0428069	0,0451186	0,0544325	172,363	54,6339	0,232866	0,282697	0,6279808
CG14647-RB	TwdlG	137,922	465,108	590,838	5,33374	0,0333458	99,8394	-0,138027	0,544666	0,6279808
CG14648-RA	CG14644	0,350789	0,34857	0,459243	0,650287	1,20653	0,0558899	-0,226417	0,270603	0,6279808
CG14648-RB	CG14645	0,165604	0,150843	4593,43	0,335157	0,453973	0,342141	-0,215781	0,297735	0,6279808
CG1464-RA	CG14646	11,449	0,0341064	0,0417988	13,1176	6,90874	0,0198742	0,0685	0,799571	0,6279808
CG1464-RB	CG14647	11,8336	0,013223	0,0180235	0,0190906	0,0202726	0,0218568	0,069431	0,79809	0,6279808
CG1464-RC	lost	44,7148	0,0170323	70,2753	113,819	14,0307	17,1218	0,07056	0,795918	0,6279808
CG1464-RD	lost	61,2204	0,0172174	33,8731	0,0281165	0,0260242	15,2419	0,065337	0,809493	0,6279808
CG14650-RA	CG14650	12,8173	14,8356	14,3199	0	0	0,0811865	0,16625	0,516768	0,6279808
CG14651-RB	CG14651	0,0290656	0,026475	0,0558095	0,0221819	0,0300455	0,0506697	0,025015	0,909043	0,13772387
CG14654-RA	CG44098	0,983272	22,0489	1,6992	0,289404	0	1,61279	-0,393437	0,271116	0,6279808
CG14655-RA	CG14655	12,5227	1,61214	10,5582	6,71464	6,72355	7,5186	0,448516	0,130111	0,6279808
CG14656-RA	ctrip	2,79144	8,6154	10,714	55,254	0,0608937	7,80146	-0,411989	0,136161	0,6279808
CG14657-RB	CG14657	9,45187	10,8327	9,82756	8,17671	24,0639	14,0483	0,382763	0,127585	0,13772387
CG14658-RA	CG14658	0	0,165955	1,3562	26,2724	4,69046	0,0422644	-0,037875	0,76416	0,6279808
CG14659-RA	CG14659	0	0	22,5286	0,0141383	0,0560789	4,21477	NA	NA	0,6279808
CG14660-RA	laf	0	28,7838	22,5175	0,0854049	0,0191504	0,0157178	-0,024364	0,884084	0,6279808
CG14661-RA	CG14661	0,943072	0,775885	4,38099	3,36581	43,0508	12,5885	-0,562072	0,115421	0,6279808
CG14662-RA	CG14662	0,528607	1,37189	49,8276	0,508251	0	52,0925	0,316026	0,349499	0,6279808
CG14666-RA	Tim17a2	0,0361268	0,0329068	0,0346839	0,0405108	0,269675	0,041355	-0,523072	0,10405	0,6279808
CG14667-RA	CG14667	0,0540866	16,9361	0,0564049	5,10773	0,0397993	3,94895	-0,220499	0,388141	0,6279808
CG14667-RB	CG14667	8,3758	10,6909	32,4425	21,7572	18,0886	13,5813	-0,220499	0,388141	0,6279808
CG14668-RA	CG14668	0,0946646	0,0107887	0,0400063	4,61138	2,75313	1,61711	-0,017073	0,951559	0,6279808
CG14669-RA	CG14669	2,88984	3,47709	0,0113713	3,36953	7,97504	2,24731	0,11164	0,634192	0,6279808
CG14670-RA	Syx16	10,6916	48,9333	0,0202028	0,140611	0,033123	0	-0,780755	0,000607	0,6279808
CG14671-RA	Hcs	4,58537	1,19976	0,0134097	32,6652	0,0428982	0,0146817	-0,38698	0,232658	0,6279808
CG14672-RA	CG14671	25,202	17,3617	0,249174	0,0366366	8,37669	0,302341	0,265723	0,221772	0,6279808
CG14673-RA	Spec2	16,1691	7,93942	23,429	10,2718	6,30986	17,9613	0,217484	0,520387	0,6279808
CG14674-RA	CG14673	0,520283	0,789849	0,915754	1,475	1,20653	0,564513	-0,95459	0,00288	0,6279808
CG14675-RA	plx	1,65782	1,9415	2,69746	2,53161	0,0191053	1,92537	-0,456707	0,19398	0,6279808
CG14676-RA	glob3	0,0591019	5,25852	0,238843	0,461442	2,36987	49,415	-0,108193	0,626382	0,6279808
CG1467-RA	CG14676	0,103351	5,58609	7,59239	0,031686	11,6337	0,0428296	-0,511169	0,043346	0,6279808
CG14680-RA	CG1468	19,9576	13,6982	7,03851	0,129909	0,546584	0,495886	0,002419	0,99367	0,6279808
CG14680-RB	Cyp12e1	4,76189	2,70334	39,0165	5,70179	18,907	20,1692	0,002419	0,99367	0,6279808
CG14681-RA	Cyp12e1	0,04558	0,0415174	13,2963	7,14081	2,75822	11,0452	-0,369364	0,074264	0,6279808
CG14681-RB	Skeletor	0,0469733	29,0285	0,0450972	1,69514	17,4237	0,33509	0,230834	0,226975	0,6279808
CG14683-RA	Skeletor	28,6893	2,00385	28,1841	0,0676265	0	1,02998	-0,346345	0,190673	0,6279808
CG14683-RB	CG14683	0,0437299	0,968401	0	0	0,0592429	0,044649	-0,346345	0,190673	0,6279808
CG14684-RA	CG14683	16,9363	4,81492	1,33901	1,23281	1,38739	1,02992	0,01071	0,97493	0,6279808
CG14685-RA	CG14684	1,28895	5,82718	10,6167	0	0,974594	0	-0,022156	0,930972	0,13772387
CG14685-RB	Cap-H2	3,13381	0,0172999	2,81718	0,0503559	0,0682073	0,0514052	-0,023693	0,926154	0,13772387
CG14685-RC	Cap-H2	0,0192081	2,82562	0,0184409	26,7133	26,9639	29,4702	-0,010871	0,965656	0,13772387
CG14685-RD	Cap-H2	4,26899	3,40894	4,02157	0,0225077	0,0304868	0,0229767	0,182922	0,450181	0,6279808
CG14686-RA	Cap-H2	0,0206552	0,0188142	0,0198302	17,5462	15,7801	10,3531	0,108669	0,672444	0,6279808
CG14687-RA	Desi	16,768	0,018887	22,8245	0,0216926	20,5233	16,5408	0,091927	0,697086	0,6279808
CG14688-RB	CG14687	47,4003	47,605	61,1316	49,4271	0,477176	0,4384	0,384167	0,056739	0,6279808
CG14689-RA	CG14688	68,6715	79,742	107,045	90,2885	57,2194	68,6395	0,018376	0,923792	0,6279808
CG1468-RA	CG14689	0	4,15121	6,81931	4,3722	0	0,246378	-0,407508	0,233093	0,6279808
CG14690-RA	Fer2LCH	0,0479621	0,0545167	0,0460465	0,0526493	0,154809	0	-0,070526	0,562659	0,6279808
CG14691-RA	Fer2LCH	0,0475479	0	0,0456489	0,0510187	0	0	0,101091	0,718164	0,6279808
CG14691-RB	Fer2LCH	328,584	0,051121	480,944	0,053635	0	0,0764649	0,101091	0,718164	0,6279808
CG14692-RB	tomboy20	0	0	0	29,4155	5,17578	5,1074	0,536626	0,028457	0,6279808
CG14693-RB	CG14691	0,0407959	0,0398322	0,0419833	3,76316	0,0275676	0,0207766	-0,543153	0,121483	0,6279808
CG14693-RC	CG14691	0,239453	17,7878	31,3599	3,84874	1,916	2,58381	-0,52519	0,133255	0,6279808
CG14693-RD	CG14692	0,903005	65,4442	0,986384	60,1891	0,777553	51,6453	-0,119297	0,635071	0,6279808
CG14694-RA	CG14693	1,23403	71,0933	1,19573	5,96751	0,104323	0,0786243	-1,076282	0,001854	0,6279808
CG14695-RA	CG14693	0,0392219	0,0977579	1,4737	0,150259	13,3112	0,15339	-1,230008	0,000381	0,6279808

gene_id	Symbol	W1_FPKM	W2_FPKM	W3_FPKM	MCM51_FPKM	MCM52_FPKM	MCM53_FPKM	FC	p-value	p-adj
CG14696-RA	CG14693	0,0624536	164,702	0,0376554	34,5255	14,6323	49,4499	-0,150042	0,622095	0,6279808
CG1469-RA	CG14694	0,350225	0,0740603	1,2065	117,951	24,1195	149,927	-0,020479	0,921161	0,6279808
CG1469-RB	CG14695	0,949366	18,7222	34,6444	52,5383	11,5765	50,1611	-0,020848	0,919752	0,6279808
CG1469-RC	Dana\GF16171	15,5331	9,46163	22,5076	0,0715032	2,29476	68,7131	-0,020397	0,921478	0,6279808
CG14701-RA	Gycbeta100B	3,00058	4,49784	4,01489	0,0217427	3,47027	0,0221958	-0,32095	0,344102	0,6279808
CG14704-RA	CG14701	11,8987	8,64748	17,0137	10,1806	6,79744	58,9139	-0,378402	0,263986	0,6279808
CG14704-RB	PGRP-LB	2,89628	1,41626	3,80503	4,15582	0,051102	0,0719778	-0,375648	0,267474	0,6279808
CG14704-RC	PGRP-LB	0,0628296	2,06026	4,25257	4,99912	5,26542	4,8303	-0,378972	0,267495	0,6279808
CG14708-RA	PGRP-LB	9,94141	5,37349	21,5272	5,95407	4,56486	18,7914	-0,041811	0,731452	0,6279808
CG14709-RA	CG14708	0	0	5,1974	6,68895	7,21338	0,242821	0,099504	0,635012	0,6279808
CG1470-RA	Mrp4	17,0195	17,6738	18,6922	22,3093	12,33	11,2456	0,043883	0,855841	0,6279808
CG14710-RB	CDase	9,49781	8,75518	3,70802	1,47939	65,6492	0,86576	0,540877	0,034094	0,6279808
CG14711-RA	CDase	2,97518	2,6411	0,067726	0,535478	1,0182	0,0475194	-0,363053	0,1415	0,6279808
CG14712-RA	CDase	0,0244756	0,022294	3336,6	0,0482539	0,318933	0,352082	0,591276	0,011112	0,6279808
CG14715-RA	CDase	0,0242956	0,0221301	0,0839479	1,66372	0,0653602	5,67337	0,073479	0,795335	0,6279808
CG14716-RA	CDase	0,0231304	0,0210688	0,0874184	0,0836698	4,07151	0	0,108837	0,670297	0,6279808
CG14717-RA	CG14710	6,07516	0,0338951	0,100269	4,20339	45,514	66,5134	-0,060347	0,691408	0,6279808
CG14718-RA	CG14711	6,28185	0,0889453	0,0407516	13,4795	2,22486	2,6636	-0,118639	0,637618	0,6279808
CG14719-RA	CG14712	3,97779	3,45565	13,4269	0,0175734	0,169189	0,0179395	-0,054384	0,807802	0,6279808
CG1471-RA	CG14715	40,7011	38,3677	72,9377	3,33865	15,2088	0,114151	-0,090145	0,679687	0,6279808
CG1471-RB	Ho	16,9219	0,039527	9,39682	11,1829	0,0647438	15,0916	-0,051816	0,812646	0,6279808
CG1471-RC	CG14717	0	26,5484	0	8,8007	0,065697	96,8732	-0,090435	0,678746	0,6279808
CG1471-RD	Dsim\GD18777	0,0940805	18,393	2,61024	1,04749	0,0649795	0,0495132	-0,090435	0,678746	0,6279808
CG1471-RE	l-t	0	13,0113	0,0722515	22,3169	1,46849	0,0489725	-0,088404	0,68584	0,6279808
CG14720-RA	Sec24AB	18,8699	0,553664	1,61338	0,0936599	7,93801	8,21293	0,214332	0,529474	0,6279808
CG14721-RA	CG14720	1,60601	24,4932	2,20267	0,0477988	14,8289	0,0905296	-0,070616	0,799096	0,6279808
CG14722-RA	CG14721	9,74667	1,18494	6,5767	0,0134513	0,0634099	0,0137316	0,063852	0,771729	0,6279808
CG14723-RA	CG14722	17,1157	7,86965	0	0,0145023	0,184585	2,56127	0,887967	0,009247	0,6279808
CG14723-RB	HisCl1	0,0387163	0,0352655	2,11119	0,0452081	2,0289	0,0197169	0,887967	0,009247	0,6279808
CG14723-RC	HisCl1	0,0389904	0,0355152	0,781428	6,83658	0,869713	1,6387	0,746007	0,033807	0,6279808
CG14724-RA	HisCl1	0,0398048	0,0362569	0,0148298	0,0102771	0,0216181	0,0162927	-0,549089	0,06943	0,6279808
CG14724-RB	Dpse\GA13206	0,0963671	0,0877778	0,0925181	0,122813	0,166351	0,125372	-0,549033	0,069468	0,6279808
CG14728-RA	Dpse\GA13206	690,104	582,992	872,005	827,47	1675,35	1476,43	-0,168956	0,585559	0,6279808
CG1472-RA	sad	0,689455	0,0373442	0,0420203	0,0450535	17,2355	14,47	0,201474	0,357426	0,6279808
CG14731-RA	CG14731	0,079995	17,8893	0	26,3163	2,02399	2,66775	0,001213	0,995045	0,6279808
CG14734-RA	Tk	2,40125	7,62789	7,34241	0	0	0,144237	0,20455	0,533633	0,6279808
CG14735-RA	Dsim\GD18799	0,0415746	5,62515	0,278232	11,8814	3,9227	0,116295	0,100134	0,47135	0,6279808
CG14736-RA	CG14736	0,032523	0,399926	1,95296	3,19369	0	0	1,515257	1,35E-05	0,6279808
CG14736-RB	CG14736	0,0835275	0,030433	0,031224	596,101	4,40055	4,03892	1,515257	1,35E-05	0,6279808
CG14736-RC	CG14736	0,284713	0,0305102	0,0962297	9,56475	6,13281	5,70371	1,515257	1,35E-05	0,6279808
CG14739-RA	CG14739	0	0,100088	0,059472	3,04807	6,95563	0	-0,060347	0,691408	0,6279808
CG14740-RA	Es2	6,43546	6,87651	4,12036	7,70207	2,03488	2,04318	-0,119955	0,564466	0,6279808
CG14741-RB	CG14740	0	0,0564248	0	9,00303	0	0	-0,449964	0,050249	0,6279808
CG14743-RA	ATP8B	1,95643	1,97279	0,176868	6,58606	2,65842	1,8831	0,01649	0,931484	0,6279808
CG14744-RB	CG14743	0	0	0	4,14901	0,494012	0,0652413	NA	NA	0,6279808
CG14745-RA	CG14744	0	0	0,0906758	12,2621	0	0,737155	0,428313	NA	0,6279808
CG14746-RA	PGRP-SC2	1,60725	0,813331	13,316	41,7131	2,8884	4,1345	0,124627	0,471185	0,6279808
CG14749-RA	PGRP-SC1a	0,118416	0,107861	1,53476	2,81917	0,376563	4,28632	0,028651	0,915068	0,6279808
CG1474-RA	Gle1	4,6625	4,12968	38,6481	5,28538	5,29746	44,9797	0,208837	0,351635	0,6279808
CG14750-RA	RpL13A	1367,74	9,47114	16,6257	16,4356	4,39437	0,0770041	-0,150203	0,587065	0,6279808
CG14752-RA	Vps25	40,3044	28,4606	8,03258	36,8661	52,0717	4,57078	0,348043	0,17924	0,6279808
CG14755-RA	CG14752	176,692	27,8176	55,4141	1,15626	0,0142243	7,37068	-0,443294	0,198871	0,6279808
CG14756-RA	pdm3	0,734177	0,0200044	47,6825	0	0	1,3654	-0,051565	0,86892	0,6279808
CG14757-RA	CG14756	240,1	0	0	0,265083	0	0	-0,190319	0,586919	0,6279808
CG14759-RA	CG14757	2,22321	1,92028	384,981	458,611	13,0061	771,99	NA	NA	0,6279808
CG1475-RB	CG14759	0	0	0,797947	0,831268	0,350532	0	-0,336207	0,233425	0,6279808
CG14760-RA	CG14760	0,602579	0	0	0	0,425026	0	0,114132	0,748136	0,6279808
CG14762-RA	CG14762	14,2699	15,3359	0,0473173	17,4083	10,0142	8,16196	0,32479	0,189823	0,6279808
CG14763-RA	CG14763	0,761243	0,0470697	0,0188706	3,3269	0	0	-0,073939	0,833754	0,6279808
CG14764-RA	CG14764	6,88542	19,4297	0,0369319	0,0420088	8,40137	0,104286	0,044494	0,838807	0,13772387
CG14767-RA	CG14767	0,0310555	0,0282875	0,0298152	0,0333035	0,0451098	0,0339975	0,267701	0,260121	0,13772387
CG14767-RB	CG14767	0,0317418	0,0289126	0,030474	0,0340962	0,0461836	0,0348067	0,268304	0,258884	0,13772387
CG14767-RC	CG14767	77,8671	101,01	82,0059	105,483	47,7857	54,0546	0,267701	0,260121	0,13772387
CG14770-RA	CG14770	33,6176	32,469	36,7961	7,37297	5,3347	27,0646	0,841785	0,000663	0,13772387
CG14773-RA	CG14773	0	0,449153	68,23	2,14718	0,022917	33,0297	0,01562	0,897983	0,13772387
CG14777-RB	CG14777	9,69886	0,0148841	3,26555	0,0569248	0,077105	0,0178302	-0,738846	0,030462	0,6279808
CG14777-RC	CG14777	0,0886129	8,61625	15,581	71,7327	34,9607	20,5516	-0,746655	0,028139	0,6279808
CG14778-RA	CG14778	0,0944388	21,4813	0,0850737	46,7989	0,0731851	6,07742	-0,210973	0,525038	0,6279808
CG14779-RA	pck	0,0493963	0,0449936	0,0474234	9,76677	26,9961	0,0565953	0,318149	0,14367	0,6279808
CG14779-RC	pck	0,0505682	0,046061	0,0485485	6,76563	0,149619	0,058111	0,318602	0,142979	0,6279808
CG14779-RD	pck	59,101	67,7035	71,619	0,0203274	0,16212	51,4471	0,318149	0,14367	0,13772387
CG14780-RA	Cp36	0,193204	0,10559	0,0285923	0,0318391	0	0	-0,042983	0,882281	0,6279808
CG14781-RA	CG14780	4,16419	2,95653	6,34956	0,0540308	6,17007	6,26984	-0,496346	0,14178	0,6279808
CG14782-RA	mei-38	1,19261	0,0153472	4,563	0,0169191	33,6263	6,86367	-0,023996	0,908558	0,6279808
CG14785-RA	rush	35,8915	47,3492	38,1717	0,0174662	8,13519	34,6615	0,06008	0,856684	0,6279808
CG14786-RA	CG14785	1,31149	2,74054	0,0514213	9,92592	1,07054	1,25156	-0,214317	0,323068	0,6279808
CG14787-RA	Lrpprc2	5,70682	7,62519	0	7,37533	0,00479623	6,6537	-0,402661	0,217593	0,6279808
CG14788-RA	CG14787	6,2162	6,9204	0,0230707	4,95876	0	13,2958	-0,065876	0,819765	0,6279808
CG14789-RA	Ns3	10,9097	3,35823	19,9801	0,00354094	4,56222	0,0270517	-0,518964	0,054082	0,6279808
CG1478-RA	O-fut2	3,4634	7,68622	5,20978	0	2,41317	73,2303	0,043757	0,864065	0,6279808
CG14791-RB	Rab27	0,0482775	1,04009	3,06952	3,21195	1,8576	3,13236	-0,365317	0,189642	0,13772387
CG14791-RC	Rab27	2,54542	11,2551	0,0463493	0,0554401	0,0750939	0,020751	-0,34658	0,211679	0,6279808
CG14792-RA	sta	1198,96	28,8086	48,8094	33,1201	45,4391	51,2157	-0,492054	0,063215	0,6279808
CG14792-RB	sta	0,0558702	0,00743808	0,00783977	1,25801	0,0112371	0,00846894	-0,492002	0,06327	0,6279808
CG14792-RD	sta	0,0638002	4,3292	3,98225	5,58847	2,10629	2,69554	-0,492064	0,063214	0,6279808
CG14795-RB	CG14795	25,7042	19,7756	34,8554	35,5732	72,7725	0,601212	-0,573245	0,075273	0,6279808
CG14796-RA	Mur2B	0,472146	0,653472	0,547478	0,91547	0,0272811	0,498737	-0,259666	0,357389	0,6279808
CG14797-RB	CG14797	0,122893	0,11194	0,913348	5,13564	0,33263	0,17313	-0,041764	0,90644	0,6279808
CG14798-RA	CG14798	0,0793544	0,0722814	0,0151666	0,0225926	0,169573	0,519088	0,385326	0,1605	0,6279808
CG14799-RA	prage	0	0	0,447526	0,723225	0	0,445857	-0,023025	0,940823	0,13772387
CG14801-RA	bnk	0	0,17229	0,0605313	0,192498	0,157461	0,19651	-0,206648	0,434926	0,6279808
CG14801-RB	prage	6,75599	10,2711	0,0212827	0,0232708	0,0315204	0,0237557	-0,142654	0,578223	0,6279808
CG14801-RC	prage	0,0229059	0,0208643	0,0224661	0,0246368	0,0333707	0,0251502	-0,206471	0,435645	0,6279808
CG14801-RD	prage	0,0221681	0,0201923	0,017846	0,0193485	0,0262076	0,0197517	-0,177065	0,501721	0,6279808
CG14802-RA	prage	0,0234007	0,021315	1,0001	1,05946	0,406381	0,544992	0,053363	0,831868	0,6279808
CG14802-RC	MED18	0,0766542	0,0698219	0,0735926	0,0999084	0,135327	0,10199	0,053363	0,831868	0,6279808
CG14803-RA	MED18	21,0277	24,1392	26,3501	21,2556	19,9161	28,1859	0,180466	0,545381	0,6279808
CG14804-RA	CG14803	0,0194876	0,0177506	3,07767	2,70891	0,0275336	0,020751	0,147021	0,485599	0,6279808
CG14805-RA	Vps26	14,1517	38,0125	19,056	71,2633	9,80345	11,8389	-0,304845	0,221949	0,6279808
CG14806-RA	CG14805	14,9891	10,5065	107,699	183,005	272,223	286,67	-0,213744	0,451202	0,6279808
CG14808-RA	CG14806	30,5525	1566,84	1,26342	2215,65	2,86641	2,01618	-0,447378	0,09368	0,6279808
CG14809-RA	Scgdelta	5,49948	0	0	0,169596	0,0236205	0	0,01562	0,897983	0,6279808
CG1480-RA	CG14809	0,363054	5,85043	7,74244	16,8788	10,3831	7,42311	-0,20035	0,494853	0,6279808
CG14810-RA	CG14810	0,137658	0,334369	0,352427	0	0	0,194962	0,146871	0,608523	0,6279808

gene_id	Symbol	W1_FPKM	W2_FPKM	W3_FPKM	MCM51_FPKM	MCM52_FPKM	MCM53_FPKM	FC	p-value	p-adj
CG14811-RA	CG14811	0,152249	0,369809	0,389781	0,11469	0,367266	0,484711	0,012155	0,966513	0,6279808
CG14812-RA	CG14812	39,6832	39,4088	45,3831	8,96287	2,37691	48,3714	-0,052627	0,791993	0,6279808
CG14813-RA	deltaCOP	34,3016	36,4309	44,3473	48,0098	20,3195	43,5371	-0,268021	0,174723	0,6279808
CG14814-RA	CG14814	0,0185885	0,0169317	7,19543	12,4618	7,67943	5,96268	-0,000417	0,998964	0,6279808
CG14814-RB	CG14814	0,568204	0,526186	0,021991	0,0240874	0,0326266	0,0245894	-0,000417	0,998964	0,6279808
CG14815-RA	Pex5	0,0312612	0,0277741	0,0300126	0,0335408	0,0454313	0,0342398	0,039582	0,866375	0,6279808
CG14815-RB	Pex5	17,6328	28,548	21,8475	22,2066	19,9901	21,4918	0,04886	0,835611	0,6279808
CG14816-RA	Pgam5	14,6664	14,2029	20,4539	20,5421	37,2205	22,9174	-0,341602	0,164624	0,6279808
CG14817-RA	CG14817	43,7734	13,1391	58,6464	16,9362	97,7131	93,9112	-0,315528	0,253462	0,6279808
CG14818-RA	CG14818	153,904	187,134	60,9558	45,6784	58,1937	63,1939	-0,309857	0,30194	0,6279808
CG14820-RA	CG14820	4,88347	3,69639	10,4554	70,5026	4704,89	4,6209	0,194855	0,558145	0,6279808
CG14821-RA	tow	2,05335	2,68861	2,20828	3,70998	0,0579354	2,07625	0,11895	0,628984	0,6279808
CG14821-RC	tow	9,43832	13,6517	11,1793	14,1892	38,2489	6,49197	0,214965	0,391519	0,6279808
CG14823-RA	CG14823	0,106288	0,0968144	0,269901	0,32462	0,188882	0,706857	-0,392017	0,269891	0,6279808
CG14823-RB	CG14823	0,129391	0,117858	8,18681	11,0322	0,246876	6,23976	-0,213762	0,526026	0,6279808
CG14823-RC	CG14823	0,404545	0,0736976	0,015391	1,66339	1,14503	0,941509	-0,256291	0,442383	0,6279808
CG14823-RD	CG14823	0,281129	0,341429	0	0	0,717119	0	-0,431892	0,226414	0,6279808
CG14825-RA	BBS1	0,265805	0,356049	227,437	167,479	361,198	0,310412	-0,470232	0,184689	0,6279808
CG14826-RA	CG14826	0,330179	0,410362	0,0576348	22,1932	0,417955	0,314996	-0,565187	0,053357	0,6279808
CG14826-RB	CG14826	1,53006	1400,05	2,6441	1887,49	1642,63	1433,93	-0,564094	0,053285	0,6279808
CG14827-RA	mei-P22	0,729754	0,755353	0,477687	0,45251	0,450006	0,339152	0,622731	0,08139	0,6279808
CG14829-RB	CG14829	0,650747	1,5795	672,823	7,44689	3,12313	3,0653	-0,784338	0,027126	0,6279808
CG14830-RA	Map205	10,3712	9,89045	10,3696	16,6981	6,7103	5,9227	0,217152	0,335334	0,6279808
CG14834-RA	Map205	0,0141228	0,012864	0,0135587	0,0145472	0,0197042	0,0148503	0,435559	0,173516	0,13772387
CG14835-RA	CG14830	50,8476	7,24911	10,1387	10,5456	43,0561	7,50443	-0,040143	0,812688	0,6279808
CG14837-RC	CG14834	1,54901	1,11612	2,44158	1,38345	1,14175	63,5076	0,088002	0,780399	0,6279808
CG14838-RA	CG14835	0	0	0,240656	0	0	0	-0,153575	0,665812	0,6279808
CG14839-RA	CG14837	0,593323	0,790887	0,805811	0,648529	0,0193455	0,623708	0,50539	0,144496	0,6279808
CG1483-RA	CG14838	0,184678	0,309875	0,149307	0,54341	0,152156	0	0,009408	0,971939	0,6279808
CG1483-RB	CG14839	0,0670141	0,0685495	0,216754	0,236542	0,0905179	0,24147	0,009396	0,971979	0,6279808
CG14840-RA	fliI	7,23181	7,16086	8,2319	11,0077	111,682	6,28089	-0,013096	0,914398	0,6279808
CG14841-RA	CG14840	0	0	0	4,75083	0	0	0,014979	0,954083	0,6279808
CG1484-RA	CG14841	0,069117	1,05511	0	4,73013	0,0933585	0,00619294	-0,11208	0,594768	0,6279808
CG14850-RA	CG14850	0	8,38149	14,0633	0,38232	34,7944	12,1413	-0,003144	0,989422	0,6279808
CG14851-RA	CG14851	0,791759	1,26708	3,04054	9,59183	2,8629	5,92077	0,007937	0,980896	0,6279808
CG14852-RA	CG14852	0,503435	6,12156	0,062975	5,67373	10,0245	1,48693	-0,406768	0,227075	0,6279808
CG14853-RA	CG14853	2,58445	1,93036	2,6177	12,5607	7,61711	6,8463	0,331183	0,160054	0,6279808
CG14853-RB	CG14853	3,12527	3,79131	2,64585	4,16257	2,08783	2,23958	0,268246	0,256456	0,6279808
CG14854-RB	CG14854	1,93756	21,3125	34,1444	1,06386	32,8881	44,2655	-0,919417	0,005143	0,6279808
CG14855-RA	CG14855	1,94736	1,80437	2,7399	4,28411	1568,5	17,0155	-0,398093	0,150417	0,6279808
CG14856-RA	CG14856	4,18558	3,39058	4,79667	5,43088	6,75346	6,21717	-0,588986	0,027703	0,6279808
CG14857-RA	CG14857	0,0321225	5,22177	15,0882	0,0355303	10,0255	10,3276	0,355454	0,119449	0,6279808
CG14860-RB	CG1486	14,9473	15,4136	0,0517147	20,0455	5,51331	12,016	0,14111	0,644792	0,6279808
CG14861-RA	CG1486	0,0181344	0,0165181	115,419	0,0188557	6,306	0,0192486	0,01562	0,897983	0,6279808
CG14864-RA	CG14860	2,1555	2,67733	0,0308395	3,71439	0,048126	0,0362707	-0,200377	0,545888	0,6279808
CG14864-RB	CG14861	0	0	0,0355388	0	5,77597	45,0004	-0,201079	0,543653	0,6279808
CG14865-RA	CG14864	0,401027	49,0752	0,0385009	99,5717	0,0595592	0,0448874	-0,177986	0,551894	0,6279808
CG14866-RA	CG14864	0,446275	0,025604	0,759527	0,0299301	0,771169	0,992519	0,165293	0,606019	0,6279808
CG14866-RB	l(3)neo43	45,6238	0,0744266	13,6533	10,4608	8,93095	10,2363	0,187483	0,561368	0,6279808
CG14867-RA	CG14866	1,45016	2,87077	2,43178	10,62	3,73099	6,97762	-0,204675	0,504565	0,6279808
CG14868-RA	CG14866	6,05945	10,4437	6,1202	3,54927	2,6193	1,63215	0,081139	0,722704	0,6279808
CG14869-RA	Rbp	2,72998	3,72998	0,0303719	37,9288	7,4613	4,28211	0,323805	0,154437	0,6279808
CG14869-RB	CG14868	5,65097	7,57162	6,94361	20,7774	3,87706	5,40146	0,323805	0,154437	0,6279808
CG1486-RA	AdamTS-A	9,68491	0,0150894	0,00917291	0,0171614	16,017	10,228	-0,02071	0,919878	0,6279808
CG1486-RB	AdamTS-A	0,00955452	57,0756	2,36574	68,4913	3,60945	2,71428	-0,02071	0,919878	0,6279808
CG14870-RA	krz	11,4423	12,0204	15,4637	14,9466	10,4058	9,19968	-0,208302	0,531748	0,6279808
CG14871-RB	B9d1	1,27502	0,990584	1,3321	4,66131	7,12324	22,1566	0,560638	0,114368	0,13772387
CG14872-RA	Trissin	1,01932	0,00870292	36,7776	6,873	0,01319	0,00994081	0,706876	0,03	0,6279808
CG14872-RB	CG44013	18,1647	13,3712	45,1187	896,834	0,0244153	0,0184009	0,615019	0,058771	0,13772387
CG14876-RA	CG44013	0,0751145	0,0684195	0,0721144	0,0502407	0,0496078	0,0691015	0,165099	0,522203	0,6279808
CG14876-RB	CG43317	0,191181	0,0497545	1,3738	0,169928	2,46973	10,0351	0,207879	0,362549	0,6279808
CG14877-RA	CG43317	0,244524	0,222729	33,7921	0,0762635	10,5727	0,288921	0,038997	0,908768	0,6279808
CG14879-RA	CG14877	0,475055	0,0344776	0,0261415	0,041272	0,107205	4,51356	-0,762709	0,004445	0,6279808
CG14879-RB	CG14879	4,24588	2,97625	3,27877	5,87183	182,067	180,513	-0,759516	0,00462	0,6279808
CG1487-RA	CG14879	0,0389445	1,56083	5,02882	4,0723	129,96	106,383	0,062077	0,75572	0,6279808
CG14880-RA	Cyp311a1	0,03306	10,6954	47,8451	0,00801259	2,28805	0,00817657	0,620832	0,006176	0,6279808
CG14880-RB	Cyp311a1	0,61318	0	0,0595277	0,00801259	0,010861	21,2221	0,620115	0,00621	0,6279808
CG14881-RA	CG14880	5,65471	4,62578	6,4796	0,0276099	2,85994	8,23266	0,341619	0,215686	0,6279808
CG14881-RB	CG14880	8,59826	8,28791	10,6739	14,0109	11,3803	2,63838	0,35251	0,199641	0,6279808
CG14882-RA	CG14881	3,01804	35,0327	66,7235	23,2844	6,41437	0,637935	0,41177	0,158277	0,6279808
CG14883-RA	CG14881	0,0702188	2,79355	3,32557	2,72668	1,1002	0,15697	0,372136	0,094146	0,13772387
CG14884-RA	CG14882	4,48826	14,5398	6,74725	2,88804	1,04522	15,9361	-0,089115	0,759659	0,6279808
CG14885-RB	CG14883	20,9478	8,30072	10,3076	27,6811	0,208276	12,1182	-0,66947	0,013042	0,6279808
CG14885-RC	CSN5	11,4256	21,6092	20,2163	22,9794	18,8509	0,518107	-0,691162	0,009834	0,6279808
CG14886-RA	Gyc89Da	0,846055	4,05964	1,33873	0,153765	0	0	-0,34329	0,113941	0,6279808
CG14887-RA	Gyc89Da	0,563721	1,41285	1,17955	14,2691	94,5749	88,5271	-0,97064	0,002217	0,6279808
CG14887-RB	Gyc89Db	5,46925	0,513476	7,14763	15,8289	1,66913	1,71225	-0,97064	0,002217	0,6279808
CG1488-RA	Dhfr	0,104716	5,15019	0,100534	10,4061	7,81415	11,2847	0,26195	0,437328	0,6279808
CG1488-RB	Dhfr	6,7628	11,1125	11,1759	0,557446	0,207449	66,1224	0,26195	0,437328	0,6279808
CG14891-RA	Rpt6	42,4457	33,0368	65,2714	61,28	64,8188	0,0426125	0,0799	0,654847	0,6279808
CG14891-RB	CG14891	0,0414355	29,5081	31,2779	0,0455833	65,5822	2,74131	0,0799	0,654847	0,6279808
CG14892-RA	CG14891	0,13145	0,0377423	0,0397805	0,1147	1,54185	3,31376	0,729279	0,009336	0,6279808
CG14893-RA	CG14892	3,2317	4,84571	3,86635	11,2745	4,62733	9,54563	-0,032129	0,924984	0,6279808
CG14894-RA	CG14893	0,165916	0,113346	76,9516	0,291225	11,6276	0,0338095	-0,424012	0,150572	0,6279808
CG14895-RA	CG14894	20,2401	3,1908	29,5816	3,21052	11,4301	1,09738	-0,276971	0,292917	0,6279808
CG14895-RB	Pak3	11,1679	6,10208	2,34303	13,9468	15,5543	21,2247	-0,265413	0,31375	0,13772387
CG14898-RA	Pak3	1,92928	12,4934	20,1676	3,86468	0	0	-0,088844	0,750747	0,13772387
CG14899-RA	Sdhaf3	13,2095	13,4577	14,0551	16,8145	1,49684	0,11688	0,204837	0,290557	0,6279808
CG1489-RA	Der-2	14,2463	15,3533	17,0512	1,36481	13,1098	11,3833	-0,450657	0,088895	0,6279808
CG14900-RA	Usp7	12,8653	0,431044	13,9579	0,00800966	0,010861	38,6411	0,135295	0,695498	0,6279808
CG14901-RA	Cad89D	3,30769	0,0953827	3,18026	15,0483	0,100243	0	-0,104208	0,770322	0,6279808
CG14902-RA	Gr89a	0,242703	0,221071	0,436893	2,26538	13,3127	7,0388	-0,136867	0,632229	0,6279808
CG14903-RA	Decay	12,7157	4,6369	26,7035	7,06369	1,61553	2,42045	-0,413649	0,170052	0,6279808
CG14904-RA	CG14903	36,5253	0,119734	0,1262	56,6095	3,92059	7,19208	0,346256	0,17699	0,6279808
CG14905-RA	Scp2	17,7521	13,3618	18,7449	3,57562	8,71956	6,32799	-0,256571	0,35711	0,6279808
CG14906-RA	CG14905	1,30107	1,7565	1,94058	2,41136	11,8432	0,048339	-0,062755	0,837077	0,6279808
CG14907-RA	CG14906	0,0611814	0,0557282	0,0587378	0,0750885	0	0,0234085	-0,403433	0,131849	0,6279808
CG14907-RB	CG14907	4,49312	4,60254	7,31201	6,16838	14,1324	0,0231666	-0,277638	0,26047	0,6279808
CG14909-RA	CG14907	2,91075	4,53869	7,19938	10,7982	0	0,0233354	0,136154	0,571543	0,6279808
CG1490-RB	VhaM9.7-d	0	0,563544	0	0,0148924	27,1356	0	0,272876	0,228897	0,6279808
CG14910-RA	CG42747	5,50632	4,29903	4,21653	10,4775	7,62122	2,62291	-0,19168	0,501309	0,6279808
CG14911-RA	CG42747	8,08186	9,06033	6,7359	12,674	3,30365	6,23026	0,124003	0,655056	0,6279808
CG14913-RA	Dpse\GA13348	0,174701	0,382247	0,0559078	7,94804	0,325003	0,327651	-0,021134	0,932873	0,6279808
CG14913-RB	Dpse\GA13348	0,0456745	0,15913	0,131551	11,9744	0	0	-0,023784	0,928011	0,6279808

gene_id	Symbol	W1_FPKM	W2_FPKM	W3_FPKM	MCM51_FPKM	MCM52_FPKM	MCM53_FPKM	FC	p-value	p-adj
CG14914-RA	CG14914	0	0,0801772	0	0	0	0	-0,013096	0,914398	0,6279808
CG14914-RB	CG14914	0	0	0	0	0	0,583526	-0,041811	0,731452	0,13772387
CG14915-RA	CG14915	0	20,1025	0	0	0	0	-0,070526	0,562659	0,13772387
CG14916-RA	Gr32a	0,0495816	6,7738	7,36713	0,222141	4,03015	4,25204	0,223965	0,490456	0,6279808
CG14919-RA	AstC	7,27216	0	0,0870587	5,2276	1,8775	0,022007	-0,362417	0,218364	0,6279808
CG14920-RB	CG1492	0,0295553	0,026921	22,8484	19,9919	13,9886	0,601418	-0,341045	0,222156	0,6279808
CG14921-RA	AstCC	0,151134	5,8301	1,74632	0,0159717	13,2032	2,42103	-0,265108	0,380706	0,6279808
CG14925-RA	CG14921	2,04026	0,862046	3,36772	0,123912	1,1868	1,75324	0,049617	0,76224	0,6279808
CG14926-RA	Osi21	0	0,177228	0,121063	8,32381	8,62329	8,9698	-0,10739	0,599076	0,6279808
CG14928-RA	CG14926	0	6,74794	5,109	89,9854	2,9323	4,07221	0,724167	0,035358	0,6279808
CG1492-RA	spz4	20,5295	1602,97	0,012986	0,0282491	0	0,0536606	0,162151	0,475732	0,6279808
CG14930-RA	CG14930	0	0	0	0,34246	0	0	NA	NA	0,6279808
CG14931-RB	CG14931	0,408334	0,202876	0,605857	0,807056	2,31254	2,47279	0,294108	0,378158	0,6279808
CG14932-RB	CG14932	0,0745215	0,706689	0,595882	3,45878	1,05657	0,980615	-0,188927	0,595585	0,6279808
CG14932-RC	CG14932	0,853424	0,16252	0,0856482	0,0893421	0,156164	36,1526	-0,188927	0,595585	0,6279808
CG14933-RA	CG14933	61,1005	45,091	56,1859	35,6673	66,1303	51,5328	0,060791	0,837782	0,6279808
CG14934-RA	Mal-B1	2,24018	1,4918	2,964	0,260836	0,544824	0,65347	1,538369	7,35E-06	0,6279808
CG14935-RA	Mal-B2	0,0351717	0,0320368	0,0337669	0,0380982	0,0516043	0,0388921	-0,29881	0,271137	0,6279808
CG14935-RB	Mal-B2	1,72709	2,69683	2,521	3,20576	3,00513	2,15762	-0,305936	0,260749	0,6279808
CG14936-RA	Tsp33B	0,0892114	37,1948	43,7294	0,840772	3617,25	16,8446	0,188401	0,387173	0,6279808
CG14937-RA	CG14937	1,28343	1,47072	2,2656	36,4451	48,0663	0	-0,685816	0,013646	0,6279808
CG14938-RA	crol	0,0096621	13,518	0,00927619	21,1775	15,7463	6,45615	0,34674	0,183896	0,13772387
CG14938-RB	crol	0,00937025	21,1888	0,008996	35,9967	0,0133419	15,2074	0,354284	0,174054	0,6279808
CG14938-RC	crol	8,78113	0,0560569	8,68184	2,13302	0,0129302	16,0717	0,366191	0,161428	0,6279808
CG14938-RD	crol	34,5442	0,0191819	23,3065	5,65229	5,16892	8,74065	0,35155	0,178237	0,6279808
CG14939-RA	CycY	19,607	20,5729	23,4714	22,7932	13,8875	0	0,141156	0,451221	0,6279808
CG14941-RA	CG1494	0,0771096	10,9516	0,10095	6,07571	4,63584	0,0812674	0,115862	0,646318	0,13772387
CG14945-RA	esc	2,52812	3,27973	3,05232	3,23737	2,28665	2,89325	-0,427471	0,066615	0,13772387
CG14945-RB	CG14945	2,00051	0,888361	12,3777	125,875	101,757	108,413	-0,450181	0,055673	0,6279808
CG14946-RB	CG14945	4,22336	77,335	110,338	0,0413757	0,0560437	0,0422379	0,203708	0,420772	0,6279808
CG14947-RA	firl	11,953	0,0138689	12,4897	7,88662	10,1648	2,54095	0,526424	0,136983	0,6279808
CG14948-RA	CG14947	0,894016	5,17027	1,44352	3,56255	3,41747	5,2406	0,364169	0,111967	0,6279808
CG14949-RA	dpr18	5,70964	5,30383	40,2642	4,68026	3,89361	3,05366	0,345764	0,195516	0,6279808
CG1494-RA	CG14949	19,7595	16,7254	0,012333	20,8181	28,5019	0,0134677	-0,189678	0,595898	0,6279808
CG14950-RB	CaMKI	76,1223	86,6837	0,028971	22,3042	0,0246718	0,0185941	-0,16332	0,581903	0,6279808
CG14952-RA	CaMKI	0,0295553	0,026921	24,1356	10,8786	13,2708	38,9255	0,028577	0,882746	0,6279808
CG14955-RA	CaMKI	0,0203644	0,0185493	0,0159563	0,057321	0,0776416	0,0322377	-0,62934	0,078603	0,6279808
CG14955-RB	CaMKI	30,7937	40,4132	0,0156841	22,3897	27,0988	6,2528	-0,702548	0,049652	0,6279808
CG14956-RA	CaMKI	18,3968	0,0242329	62,1452	20,0507	20,5691	17,1488	-0,279417	0,177923	0,13772387
CG14957-RA	CaMKI	0,0301762	0,0274866	40,6179	70,3269	0	9,71124	0,053159	0,881925	0,6279808
CG14958-RA	CaMKI	24,1283	22,6489	18,1879	0,0315797	145,34	0,0329645	-0,017083	0,960583	0,6279808
CG14959-RA	Fife	2,39532	3,61864	2,52401	22,5001	24,1517	0,0224401	0,47917	0,046906	0,6279808
CG14959-RB	CG14952	0,430182	17,8849	0	0,543949	0,0182013	2,88506	0,47861	0,046894	0,6279808
CG14959-RC	CG45067	0,0227717	0,165936	0,0218622	0,0239387	10,1573	45,4584	0,47917	0,046906	0,6279808
CG1495-RA	CG45067	0,26905	0,169663	0,139087	0,968132	3,94135	5,38947	0,155453	0,542854	0,6279808
CG1495-RB	BtbVII	7,91161	8,11178	0,0199069	3,16149	5,61675	9,6097	0,156888	0,539141	0,6279808
CG1495-RC	CG14957	2,95398	33,988	10,2991	7,99024	0,0308877	1,05885	0,166382	0,514351	0,6279808
CG1495-RD	CG14958	2,44958	0,0200177	0,037918	0,023059	0,0606933	0,0457421	0,19442	0,44902	0,6279808
CG1495-RE	ckd	0,0635548	0,0578901	0,0610164	20,857	8,57109	0,0516023	0,169068	0,50807	0,6279808
CG1495-RG	ckd	19,2545	23,2923	18,7238	0,0663882	0,100223	19,2001	0,15383	0,547417	0,6279808
CG1495-RH	ckd	0,0578766	0,052718	0,055565	418,329	10,1916	16,08	0,196931	0,442973	0,6279808
CG14960-RA	CG14960	30,7532	40,6238	32,2229	0,0739923	16,5528	0,0677716	0,361276	0,224379	0,6279808
CG14961-RA	CG14961	0,36107	0,131555	0,519974	0	0	40,036	-0,659146	0,061779	0,6279808
CG14962-RA	Asciz	0,0328899	16,5144	3,55241	20,7748	270,135	181,834	0,145725	0,558533	0,6279808
CG14963-RA	CG14963	0,0752	1,77865	0,0214628	8,10704	0,0320179	0,0136066	0,057766	0,846821	0,6279808
CG14964-RA	CG14964	4,5886	1,04445	4,21283	7,14349	0,0318012	8,32212	0,372493	0,238572	0,6279808
CG14965-RA	CG14965	1,45431	0,0195368	0,0600347	0,022477	0,0983456	0,0741192	0,298599	0,388444	0,6279808
CG14966-RA	CG14966	14,8397	17,1453	17,9963	40,8958	4071,17	3795,75	-0,372826	0,1457	0,6279808
CG14967-RA	hob	0,00941699	0,00857764	0,00904087	0,00959468	0,0129961	0,00979461	0,246104	0,255617	0,6279808
CG14968-RA	CG14968	0,0730388	0,0620691	1,95032	0,902809	498,467	12,8937	-0,101115	0,700743	0,6279808
CG14968-RB	CG14968	14,3013	0,0665288	10,8688	9,49643	1,79425	9,09612	-0,102605	0,697584	0,6279808
CG14968-RC	CG14968	0,0681427	38,784	11,1497	13,0909	475,519	0,0890309	-0,103366	0,695447	0,6279808
CG14968-RD	CG14968	0,0730388	0,630697	0,0654211	0,0803047	12,8829	42,6998	-0,104983	0,689586	0,6279808
CG14968-RE	CG14968	37,7397	0,250082	0,0701216	0,0872136	10,4817	0	-0,103684	0,693362	0,6279808
CG14969-RA	CG14969	9,08895	0	0,45312	975,021	0,400477	0,0338095	0,240004	0,277536	0,6279808
CG14971-RA	CG14971	8,83423	0,0401511	121,429	7,96706	2,8343	0,0785325	0,253145	0,214222	0,6279808
CG14974-RA	CG14974	0	0	0	0	0,23736	0,239293	-0,014388	0,910154	0,6279808
CG14975-RA	Rdh	0	0	0,6608	23,2288	1736,16	11,0052	0,03968	0,899928	0,6279808
CG14977-RA	CG14977	15,0892	15,0189	31,1343	0	4,54301	0	-0,054855	0,848169	0,6279808
CG14979-RB	Gr63a	0,134849	419,311	0	0,0182651	0	0,0186457	-0,331776	0,342245	0,6279808
CG14980-RB	Ccz1	6,55094	5,98741	9,57346	615,531	6,39284	1158,56	-0,156264	0,511523	0,6279808
CG14981-RA	mge	101,718	75,9309	0,025446	0,0287671	5,15478	36,0913	0,014499	0,958677	0,6279808
CG14981-RB	mge	0,0793544	0,0722814	0,0253443	0,0286421	0,0301266	0,0227052	0,062485	0,82354	0,6279808
CG14982-RB	CG14982	2,29803	31,7836	0	16,2669	0,985017	15,0483	-0,040144	0,872142	0,6279808
CG14983-RA	CG14983	0,162216	0	178,18	112,235	0,0214983	0,0162024	0,01562	0,897983	0,6279808
CG14984-RB	CG14984	9,65026	5,06725	0,0761849	0,0964004	1,72466	1,542	0,159616	0,633894	0,6279808
CG14985-RB	CG14985	6,79571	5,52602	7,34828	5,74503	0,130575	0,0984092	-0,240351	0,387631	0,6279808
CG14987-RB	Gr64d	0	0,0214365	0,0223868	0	0	0,118933	0,015925	0,916254	0,6279808
CG14989-RB	CG14989	91,3909	80,8363	98,0621	136,806	93,7095	83,624	-0,231436	0,223891	0,6279808
CG14990-RA	nyo	10,9614	6,59809	9,71278	13,1483	3,40581	7,96583	0,118946	0,673779	0,6279808
CG14991-RA	nyo	6,69002	10,4047	7,35379	0,456645	0,0250386	1,89913	-0,165423	0,462604	0,6279808
CG14991-RB	CG14990	0,0615805	0,0213225	0,022474	0,024646	8,14723	0,0772695	-0,165178	0,463509	0,6279808
CG14992-RA	Fit1	10,2369	0,0560918	0,059121	0,0756923	0,102526	6,18377	0,191668	0,41858	0,6279808
CG14993-RA	Fit1	21,3056	31,9499	22,7522	22,5344	15,4598	21,7865	-0,219241	0,447216	0,6279808
CG14994-RA	Ack	5,65977	6,35134	5,12943	6,69526	3,80425	3,35977	0,273202	0,22172	0,6279808
CG14994-RB	Faa	28,8441	11,5726	10,4551	9,57213	12,0022	4,40875	0,293431	0,189426	0,6279808
CG14994-RC	Gad1	28,9625	29,9355	26,0122	18,1698	19,0043	14,3424	0,270022	0,22737	0,6279808
CG14995-RA	Gad1	7,63483	7,23679	12,3294	12,5743	11,4118	7,30734	0,137345	0,5744	0,6279808
CG14995-RC	Gad1	0,0199224	0,0181467	0,0191267	0,0208023	0,0281768	0,0212358	0,138781	0,568788	0,6279808
CG14995-RD	CG14995	28,8159	10,0322	12,1526	17,3755	5,27991	16,1322	0,140363	0,560747	0,6279808
CG14995-RE	CG14995	0,023409	7,78672	0,0203149	6,33068	0,0300152	4,88027	0,24045	0,322063	0,6279808
CG14996-RB	CG14995	9,99856	6,23063	11,7846	12,8796	13,5866	5,58111	-0,152978	0,513169	0,6279808
CG14997-RA	CG14995	3,73475	30,7566	19,2111	25,534	22,5322	3,1785	-0,255547	0,340927	0,6279808
CG14997-RB	Chd64	237,02	207,199	282,584	9,47689	6,79098	7,55136	-0,255547	0,340927	0,13772387
CG14998-RA	CG14997	0,0407205	7,82087	76,1522	6,88253	0,0605697	36,8323	-0,133987	0,57383	0,6279808
CG14998-RB	CG14997	7,90438	1,71667	135,457	7,93341	11,3469	0,0689526	-0,133227	0,575738	0,6279808
CG14998-RC	ens	0,0133039	32,4993	7,86841	4,87492	2,15668	0,547332	-0,13267	0,577077	0,6279808
CG14998-RD	ens	19,8245	67,5762	0,0160939	0,193795	0,262496	5,55149	-0,144362	0,54527	0,6279808
CG14998-RE	ens	0,0172357	63,1039	0,0149981	0,0133154	0,697135	40,5181	-0,140553	0,555078	0,6279808
CG14998-RF	ens	6,8575	0,0121181	45,974	34,0711	2,7006	56,8656	-0,157087	0,509616	0,6279808
CG14999-RA	ens	0,0167634	24,005	65,6353	6,86579	0,591589	3,82666	-0,68046	0,00392	0,6279808
CG1499-RA	ens	3,58525	0,0156994	25,2455	15,4891	318,157	0,0185276	0,378885	0,245917	0,13772387
CG1499-RB	RfC4	3,87141	4,5869	6,06422	254,494	280,641	0,550132	0,379659	0,24482	0,6279808
CG15002-RB	fw	7,65423	2,16588	2,41271	6,95427	1,48934	1,66311	-0,102148	0,770634	0,6279808

gene_id	Symbol	W1_FPKM	W2_FPKM	W3_FPKM	MCM51_FPKM	MCM52_FPKM	MCM53_FPKM	FC	p-value	p-adj
CG15003-RA	fw	2,5202	7,88681	29,7968	45,4116	23,7296	29,2602	0,211648	0,467806	0,6279808
CG15004-RA	mas	10,8054	36,9343	8,25405	56,4153	14,2153	6,9422	0,09928	0,698595	0,6279808
CG15005-RA	Teh4	6,96545	10,4773	34,4972	313,505	1,37605	29,9636	0,137319	0,698632	0,6279808
CG15006-RA	Teh2	4,65373	4,10951	6,82685	7,22809	5,36355	4,16687	0,330903	0,324007	0,6279808
CG15007-RA	CG15005	14,7476	1,44093	2,51733	2,06224	0,0235333	1,26004	-0,068162	0,78947	0,6279808
CG15008-RA	Cpr64Aa	0,258109	0,0152693	0,55755	22,1347	9,03907	11,0771	0,663628	0,063449	0,6279808
CG15009-RA	Cpr64Ab	256,341	264,615	1,19747	12,3204	193,377	0,731606	0,377634	0,287654	0,6279808
CG15009-RB	Cpr64Ac	0,640948	0,0142296	0,818976	0,0173741	4,69144	0,0177361	0,320871	0,368085	0,6279808
CG15009-RC	Dsim\ImpL2	0,0697983	0,0912375	0,0120725	21,1163	107,899	1,83478	0,374246	0,292142	0,6279808
CG1500-RA	Dsim\ImpL2	91,8007	28,2512	9,93688	0,290254	76,5028	4,36	0,015174	0,959419	0,6279808
CG1500-RB	Dsim\ImpL2	0,0587514	0,0114539	8,18301	0,012906	4,36109	6,09019	0,041358	0,88926	0,6279808
CG15010-RA	unc	0,187687	0,239342	5,58189	0,329256	5,80989	1,94703	-0,01312	0,955406	0,6279808
CG15010-RB	ago	0,0125747	0	39,7025	0,0826206	2,39218	41,4914	-0,012653	0,957009	0,6279808
CG15010-RC	ago	11,1586	10,8836	20,9	265,231	9,21452	12,4535	-0,013347	0,954625	0,6279808
CG15011-RA	ago	4,3833	0,554203	19,6342	0,0675451	0,174823	29,2542	-0,65955	0,001632	0,6279808
CG15012-RB	CG15011	3,05044	3,25721	67,3127	42,5625	0,0175603	4,20987	-0,496252	0,023732	0,6279808
CG15013-RA	CG15012	32,0814	34,5122	39,2635	52,7776	0,045875	14,4046	0,327904	0,330159	0,6279808
CG15013-RB	dyl	59,8505	85,7395	65,3877	28,2864	16,8993	20,1315	0,32434	0,335583	0,6279808
CG15013-RC	dyl	0,02418	0,0220248	0,0232142	3,45574	0,0345461	19,5456	0,32775	0,330395	0,6279808
CG15014-RA	dyl	40,4323	63,0028	42,1025	13,4047	11,2943	10,3275	-0,132076	0,671814	0,6279808
CG15015-RA	CG15014	7,08395	4,37651	12,4488	8,04316	51,6964	0,0260361	0,017512	0,946168	0,6279808
CG15015-RB	Cip4	10,6744	13,7532	12,5308	5,13661	37,0819	2,48059	0,043893	0,865668	0,6279808
CG15016-RA	Cip4	12,8395	0,154854	0,326433	33,1696	0	14,3481	0,166761	0,571892	0,6279808
CG15019-RA	mRpS6	30,1926	14,5647	53,4205	0,692815	41,4054	41,1826	-0,170887	0,592888	0,6279808
CG15019-RB	CG15019	22,5469	10,3322	32,8043	0,117345	18,988	27,4833	-0,146661	0,64214	0,6279808
CG1501-RA	CG15019	50,2189	29,9645	106,801	83,8735	21,3201	95,0603	-0,099682	0,769891	0,6279808
CG15020-RA	tsg	0	0	0,211174	0,180189	0	0,0596135	-0,227606	0,403863	0,6279808
CG15021-RA	CG15020	4,39233	4,79151	3,38352	5,54911	86,2187	4,28854	-0,186202	0,585825	0,6279808
CG15022-RA	CG15021	51,7274	0,081812	113,755	59,9677	76,6313	0,0396019	0,679701	0,026892	0,6279808
CG15023-RB	CG15022	445,046	0,171228	505,16	525,933	5,86738	370,103	-0,074499	0,77824	0,6279808
CG15024-RB	CG15023	198,833	104,342	167,781	8,38139	331,192	0,0688943	-0,225099	0,477482	0,6279808
CG15025-RA	CG15024	108,583	0	0,171245	0,213085	116,978	302,156	-0,135379	0,496562	0,6279808
CG15027-RA	CG15025	0	0	0	689,143	968,637	864,195	-0,237488	0,496846	0,6279808
CG15028-RB	CG15027	14,8671	10,4191	6,86358	17,0334	34,9671	89,8142	-0,277091	0,349648	0,6279808
CG15028-RC	CG43737	0,590445	0,950312	0,977203	0,203975	3,37461	0	-0,278876	0,3404	0,6279808
CG15029-RA	CG43737	0,99241	0	0	0,191625	0,159467	0	-0,068109	0,807013	0,6279808
CG1502-RA	CG43737	3,02848	0,153068	3,28748	0	0	0	-0,02376	0,883718	0,6279808
CG15030-RA	CG1503	0,160508	0,194936	0,0302416	0	0,0458043	0,0867349	0,01562	0,897983	0,6279808
CG15031-RA	CG15030	0	0,928	0,878081	0	0	0	NA	NA	0,6279808
CG15032-RA	PPYR1	0	0	0,352427	0,0635088	0,259558	0	-0,261886	0,241941	0,6279808
CG15032-RB	gce	2,79565	2,83475	2,05343	5,8613	1,89658	2,07785	-0,329426	0,132828	0,6279808
CG15033-RA	gce	6,40214	7,06557	2,66836	8,08918	1,11367	0,438592	0,024053	0,869957	0,6279808
CG15034-RA	CheA7a	0,185606	0,0113545	0,0119677	0,298134	15,5647	0,515098	0,105944	0,740334	0,6279808
CG15035-RA	CG15034	0,382679	43,01	20,9562	0,313252	2687,27	0	0,044335	0,715924	0,6279808
CG15036-RA	CG15035	0	38,7721	8,35066	0	0,148996	0,112292	0,059792	0,706172	0,6279808
CG1503-RA	CG15036	0	5,63791	0,434499	0,0215296	0,0291619	0,0219782	0,031342	0,919301	0,6279808
CG15040-RA	CG1504	0,672528	0,740207	0,538056	1,26225	1,09676	0,826584	0,256409	0,469767	0,6279808
CG15042-RA	CG15040	0,110359	0,195754	30,0208	0,0268888	0,0421885	29,7958	NA	NA	0,6279808
CG15043-RA	CG15042	0	8,00782	10,3695	26,7546	0,0335211	12,6216	-0,466392	0,182232	0,6279808
CG15044-RA	CG15043	21,4675	28,5593	0,0476013	34,427	0,0754112	24,8497	-0,02303	0,948583	0,6279808
CG15046-RA	CG15044	6,81028	10,4258	9,85891	5,80731	6,71928	15,0308	-0,393042	0,181337	0,6279808
CG15047-RA	CG15046	1,13464	1,62118	1,26019	2,85898	2,40512	3,19568	0,156694	0,605974	0,6279808
CG15048-RB	CG15047	0	0	0	6,13558	0,670771	0	-0,137057	0,625548	0,6279808
CG1504-RA	Bx	0,579613	0,527952	0,556463	0,0364542	2,76556	1,46036	-0,375566	0,27524	0,6279808
CG15056-RA	gd	0	0	0,100428	0	0	0,183944	-0,235869	0,413268	0,6279808
CG15057-RA	CG15056	0,0746902	0,068033	0,119512	0,319315	6,31324	0,0897245	0,01562	0,897983	0,6279808
CG15059-RA	CG15057	0,379746	0	0	0,667457	0,142696	1,78419	NA	NA	0,6279808
CG15059-RB	CG15059	0	0	0	1,35236	0,0361409	0	NA	NA	0,6279808
CG1505-RA	CG15059	0	0	0	0,031265	5,92308	0,0578114	0,0332	0,813053	0,6279808
CG15064-RA	Ac3	5,4725	6,05762	4,77745	6,69812	5,074	3,5963	-0,036789	0,9038	0,6279808
CG15065-RA	Ac3	0,0368795	0,0335924	0,0354065	0,0401162	0,0543376	0,0409521	0,655658	0,053309	0,6279808
CG15066-RA	Him	5,98723	10,283	7,13318	0,0521906	0	0,178496	0,265237	0,449285	0,6279808
CG15067-RA	CG15065	26,8433	0,796118	25,0055	0,592428	15,5419	26,3406	1,151231	0,001267	0,6279808
CG15068-RA	IM23	5,72751	0,636221	0,321322	66,3995	23,6132	2,19225	0,435344	0,208378	0,6279808
CG1506-RA	CG15067	3,4797	1,24524	7,68366	13,8682	88,9158	2,70367	-0,032796	0,891974	0,6279808
CG1506-RC	CG15068	16,4316	9,40414	183,807	0,112535	0,964976	29,5368	-0,675602	0,00863	0,6279808
CG15071-RA	Pur-alpha	7,49557	7,65785	9,3356	46,2567	3,76133	9,91065	0,254623	0,474881	0,13772387
CG15072-RA	Pur-alpha	0,0508797	0,0463448	0,0488476	22,7906	10,8861	0,0585154	-0,230902	0,405285	0,13772387
CG15072-RB	Pur-alpha	23,2853	18,3819	20,0061	0,0322916	12,5047	12,0406	-0,453367	0,120257	0,6279808
CG15072-RC	Pur-alpha	24,06	19,5546	17,473	0,0308262	0,0407627	17,1921	-0,248467	0,380277	0,6279808
CG15073-RA	CG15071	1,38323	1,64762	0,0506009	5,96505	4,54129	8,45568	0,357134	0,137423	0,13772387
CG15077-RA	Sik3	2,76381	3,05179	3,92894	3,25134	2,76427	1,87935	-0,046127	0,895315	0,6279808
CG15078-RA	Sik3	0,0265294	0,0241648	0,0443717	1,79616	0,853217	0,0525292	0,484579	0,076653	0,13772387
CG15078-RB	Sik3	0,428381	0,0205368	3,20522	4,37583	2,1919	2,18522	0,563492	0,046513	0,13772387
CG1507-RA	CG15073	3,38227	3,11101	1,19189	3,22221	0,0321101	0,929723	-0,073357	0,773408	0,6279808
CG1507-RB	Cyp12b2	0,592941	0,0337557	0,0206187	0,0596563	4,57437	0,0764788	-0,073268	0,773841	0,6279808
CG1507-RC	Mctp	0,793119	0,889142	87,403	1,57817	0,977381	0,0608995	-0,074843	0,76936	0,13772387
CG1507-RD	Mctp	5,12018	6,66882	0,0616688	4,02968	1,89594	65,0809	-0,071284	0,779964	0,6279808
CG15080-RA	CG15080	25,7636	6,24597	1,70914	0,0458689	12,7069	847,095	0,529024	0,117852	0,6279808
CG15081-RA	Phb2	0,0460459	0,0458738	0,0442068	91,2894	0,0724131	124,481	-0,097929	0,688545	0,6279808
CG15081-RB	Phb2	0,0503627	0,0412802	0,0483512	0,0498228	0,0804408	0,050861	-0,097804	0,688909	0,6279808
CG15081-RC	Phb2	0,0453195	0,0508474	0,0435095	0,0253476	0,0710866	0,0258758	-0,097804	0,688909	0,6279808
CG15081-RD	Phb2	0,0534162	100,836	0,0512828	14,0947	0,0862725	0,0275165	-0,111048	0,648712	0,6279808
CG15081-RE	Phb2	116,477	0,0394665	137,36	19,5158	133,952	3,77334	-0,279108	0,261246	0,6279808
CG15081-RF	Phb2	0,0433285	0,0218966	0,0415979	5,63241	0,0674853	4,27869	-0,097996	0,688305	0,13772387
CG15082-RA	CG15082	0,630354	0,607944	1,03236	0,787464	2,19484	1,82754	-0,547698	0,124498	0,13772387
CG15083-RA	CG15083	78,5821	79,3367	85,512	0,0542086	9,28079	0,0553382	-0,705973	0,01032	0,13772387
CG15084-RA	CG15084	5,25126	5,21284	6,20507	17,2126	3,54015	5,16112	0,127167	0,63518	0,6279808
CG15085-RA	edl	52,0756	67,2551	1,09614	51,6905	0,0389482	1,12377	0,604012	0,010888	0,6279808
CG15086-RE	CG44433	0,0421004	0,0198199	0,0254698	6,11446	3,94585	4,4514	-0,080649	0,53498	0,6279808
CG15086-RF	CG44433	0,0264939	0,0180487	0,411271	19,696	13,1716	1,21599	0,09244	0,789009	0,6279808
CG15087-RA	Vps51	7,2512	5,84133	0,0450651	0,0281405	0,0381165	0,0534474	0,348272	0,10419	0,6279808
CG15088-RA	List	1,36019	0,0379565	0,244741	0,0604765	2,92367	0,0211158	-0,428157	0,167193	0,6279808
CG15088-RB	List	0,270859	0,464402	0,0845948	13,5675	0,0621298	0,0205544	-0,463441	0,142133	0,6279808
CG15093-RA	CG15093	0,052706	0,0480083	0,0185397	2,35795	1,66237	0,161719	-0,135007	0,685683	0,6279808
CG15093-RB	CG15093	34,8916	28,567	0,0220369	0,0514569	0,0808049	14,6619	-0,134818	0,686093	0,6279808
CG15093-RC	CG15093	7,4833	0,0585091	0,0218171	1,71083	64,6729	0	-0,119129	0,720596	0,6279808
CG15094-RA	MFS15	0,0370242	1,51759	20,1626	5,21812	0,101477	0,0229767	-0,039263	0,861632	0,6279808
CG15094-RB	MFS15	17,2404	10,962	3,4856	1,74044	0,0545702	0,0238822	-0,038765	0,8634	0,6279808
CG15095-RA	MFS14	18,5568	11,8295	12,305	0,0412204	3,87283	4,4098	-0,499915	0,014967	0,6279808
CG15095-RB	MFS14	0,0224315	14,012	22,9945	26,8562	18,4452	16,3954	-0,497875	0,015281	0,6279808
CG15096-RA	CG15096	11,3235	18,2345	26,3646	38,6096	23,4045	25,5749	0,106818	0,60093	0,6279808
CG15096-RB	CG15096	13,3681	0,0204322	0,0215356	0,023562	0,0319149	0,024053	0,166107	0,416999	0,6279808
CG15097-RA	CG15097	0,0240392	16,5569	0,0346186	1,0025	0,0343335	14,2625	0,203352	0,352189	0,6279808

gene_id	Symbol	W1_FPKM	W2_FPKM	W3_FPKM	MCM51_FPKM	MCM52_FPKM	MCM53_FPKM	FC	p-value	p-adj
CG15097-RB	CG15097	13,3748	5,69716	17,4632	7,07367	0,0365105	0,590817	0,202285	0,354478	0,6279808
CG15098-RA	CG15098	30,3573	33,6131	57,1199	35,3877	65,3109	59,4602	-0,335374	0,277596	0,6279808
CG15099-RA	CG15099	6,25002	6,24224	6,1691	6,03209	21,7215	7,22182	0,031976	0,887825	0,6279808
CG15100-RA	MetRS	11,1075	9,56452	13,068	5,81668	7,95591	9,88137	-0,135827	0,551624	0,6279808
CG15101-RA	Jheh1	23,6699	16,0167	7,60757	6,52329	4,20622	6,3566	-0,032718	0,905129	0,6279808
CG15102-RA	Jheh2	0,048419	0,0441034	0,697805	0,123931	0	163,745	0,097561	0,754479	0,6279808
CG15102-RB	Jheh2	6,88173	5,90525	2,03503	1,8032	209,408	4,28984	0,139873	0,651546	0,6279808
CG15104-RA	Topors	5,33532	14,5059	19,8093	31,7236	2,92448	39,8463	0,107907	0,633218	0,6279808
CG15105-RA	tn	0,0142784	0,0130058	0,0137082	9,75792	22,1435	6,72229	-0,167677	0,43672	0,6279808
CG15105-RB	tn	13,0682	14,8134	12,8486	11,6314	0,041813	9,73198	-0,182526	0,395607	0,6279808
CG15105-RC	tn	16,3544	22,6135	15,8568	18,0914	3,82517	13,7089	-0,157855	0,461883	0,6279808
CG15106-RA	Jheh3	34,985	0	13,9993	8,59889	1,4311	3,40288	-0,574665	0,041246	0,6279808
CG15107-RA	CG15107	4,39631	4,51249	7,68549	0,0534609	11,2536	0,0545749	0,428849	0,128873	0,6279808
CG15109-RC	CG15109	0	7,86533	0,0141991	0,0909706	0	0,0155759	-0,159109	0,398509	0,6279808
CG15109-RD	CG15109	0	2,78055	9,38818	0,0492272	3,23538	8,28587	-0,159109	0,398509	0,6279808
CG15109-RE	CG15109	0	0,0136148	0	0,119451	0	0	-0,159109	0,398509	0,6279808
CG15110-RA	Eph	0,0181743	0,0165544	6,92386	0,018899	0,0363469	0,0273933	0,19881	0,428633	0,6279808
CG15111-RA	Eph	26,641	21,9627	0,737721	26,85	0,032168	0,0242437	0,116523	0,542284	0,6279808
CG15111-RB	Eph	7,03606	5,60688	0,708378	8,64727	0,805419	0,726885	0,114279	0,549954	0,6279808
CG15112-RA	Eph	5,79029	4,87408	0,0228053	6,6176	0,0339031	0,0255514	0,155925	0,558511	0,6279808
CG15112-RB	botv	7,82309	8,09979	7,37025	0,028237	4,25957	0,0288254	0,139674	0,600377	0,6279808
CG15112-RC	CG15111	16,1394	15,778	19,0268	14,2352	11,9243	4,06566	0,131529	0,622955	0,6279808
CG15112-RD	CG15111	5,84719	7,8664	9,97162	7,39647	6,2586	4,41139	-0,070749	0,793638	0,6279808
CG15112-RE	ena	6,04171	6,8211	6,00244	0,0202089	6,82893	0,0206301	0,148746	0,576769	0,6279808
CG15112-RF	ena	7,84903	11,5637	7,84	0,0247386	0,0280264	0,0252541	0,14023	0,598739	0,6279808
CG15113-RA	ena	0,019379	0,0176517	0,018605	5,58548	0,0273731	2,6703	-0,109068	0,701301	0,6279808
CG15113-RB	ena	0,0234922	0,0213984	0,022554	0,0167684	0,0335085	0,0171178	-0,109068	0,701301	0,6279808
CG15113-RC	ena	3,08539	4,09986	3,19739	5,21615	4,65367	1,76469	0,077388	0,778364	0,6279808
CG15115-RA	ena	0,0162004	0,0147564	0,0155533	7,46798	0,0227129	5,31188	0,427489	0,083186	0,6279808
CG15116-RA	5-HT1B	1,11458	0,731407	4,88694	1,04985	0,020667	4,87749	NA	NA	0,6279808
CG15117-RA	5-HT1B	0,0245574	0,0223686	3,2893	0,0259261	10,0654	2,16147	0,191002	0,456525	0,6279808
CG15117-RB	5-HT1B	0,0262971	0,335345	0,01435	0,280522	0	0,0157473	0,194441	0,445754	0,6279808
CG15117-RC	CG15115	0,212599	0,34857	0,24493	0	0,020054	0	0,193948	0,446926	0,6279808
CG15118-RA	CG15116	0	0	0	0	0,0208944	3,92772	0,061651	0,794915	0,6279808
CG15118-RB	CG15117	30,1963	47,2727	35,6317	38,0098	1,40684	26,0347	0,061651	0,794915	0,6279808
CG15118-RC	CG15117	0,0289341	0,0263552	0,0277785	0,0308695	3,04296	0,0315128	0,061651	0,794915	0,6279808
CG15118-RD	CG15117	9,94336	12,1226	7,66368	22,4718	0,133982	6,23422	0,063967	0,78718	0,6279808
CG15118-RE	CG15118	10,7941	10,4335	7,38641	12,261	6,60968	5,41609	0,058936	0,804015	0,6279808
CG15119-RA	CG15118	0,0266149	0,0242427	0,0255519	0,0268998	0,0382472	0,0274604	-0,520725	0,067295	0,6279808
CG1511-RA	CG15118	4,36052	6,8089	6,03251	0,0251637	3,02008	0,0256881	0,224753	0,36616	0,6279808
CG1511-RB	CG15118	0,0254268	0,0231605	0,0244113	18,073	0,036436	14,9591	0,224753	0,36616	0,6279808
CG1511-RC	CG15118	0,0238742	0,0217463	0,0229207	10,3277	0,0340844	5,35577	0,22472	0,366189	0,13772387
CG1511-RD	mip40	13,2752	9,52041	0,022191	284,843	0,0300051	22,0299	0,13652	0,587799	0,13772387
CG15120-RA	Cul2	23,9491	20,6841	21,4345	26,6857	19,6222	15,6561	-0,410425	0,121743	0,13772387
CG15121-RA	Cul2	0,0229776	3,47431	0,0220598	6,04798	4,95893	5,06551	-0,013096	0,914398	0,6279808
CG15122-RA	CG15120	0,0650565	184,1	0,0391665	7,77791	1,27133	11,3708	-0,24008	0,270394	0,6279808
CG15124-RA	Ir56b	0	0,059258	67,247	0,0760417	0,102999	84,8677	-0,014388	0,910154	0,6279808
CG15125-RA	Ir56c	0	0,0371598	0	0	0,358417	0	0,483056	0,158685	0,6279808
CG15126-RA	CG15124	0,172073	1,65121	3,3743	0,0544103	3,42126	26,0023	-0,551442	0,123238	0,6279808
CG15127-RA	CG15125	1,47873	0	521,858	3,08428	0,0736991	0,119681	-0,610433	0,083713	0,6279808
CG15128-RA	CG15126	35,6891	14,4893	76,7507	2,03747	2,65938	0	-0,118379	0,63666	0,6279808
CG1512-RA	CG15127	8,38195	4,85855	17,8187	51,084	68,3582	60,7843	-0,235175	0,261256	0,6279808
CG1512-RB	CG15128	0	0,0745251	0,0785498	1,92945	19,0407	0,158449	-0,231592	0,267972	0,6279808
CG15130-RA	CG1513	6,06243	0,066431	0,0700185	2,21066	0,701561	0,741053	-0,233516	0,470848	0,6279808
CG15133-RA	CG15130	3,91029	0,0137768	0,0145209	0,0156159	1,41733	0,0159413	-0,511522	0,081709	0,6279808
CG15134-RA	CG42556	0,415063	6,04613	0,470937	0,0545003	0,492299	0,429745	-0,737895	0,01239	0,6279808
CG15134-RB	tweek	1,12855	0,0140864	22,3663	3,16725	0,0213339	0,0163124	-0,737895	0,01239	0,6279808
CG15136-RA	tweek	0,0154648	8,0239	19,6171	0,0159795	1,11325	11,5629	NA	NA	0,6279808
CG15138-RA	CG15136	0	0	0	0	0	0	0,352872	0,189155	0,6279808
CG15138-RB	beat-IIIc	11,7057	0	0	1,45018	0	5,19257	0,352872	0,189155	0,6279808
CG1513-RA	beat-IIIc	0,041126	9,45769	12,6027	15,9169	6,41	1,3597	-0,302295	0,199296	0,6279808
CG15140-RA	Dsim\GD16870	2,9222	0,308966	0,347361	4,99054	3,95967	3,29022	-0,388497	0,27394	0,13772387
CG15141-RA	CG15140	0,944941	0,41442	0	0	1,81536	0	0,41063	0,041937	0,13772387
CG15142-RA	CG15141	7,50645	6,20568	0,612423	8,6917	0,585708	11,174	0,130417	0,617992	0,6279808
CG15143-RA	CG15142	0,319207	0	0,868297	0,23743	0,3216	0,643036	0,485734	0,148177	0,6279808
CG15144-RA	CG15143	0,385255	19,1219	0,38791	0,430885	10,6478	0	0,036819	0,916096	0,6279808
CG15145-RA	CG15144	0,175675	0,0343138	38,7118	0,131519	0,102783	0,0895207	-0,186594	0,495864	0,6279808
CG15147-RA	CG15145	0,0627859	16,8035	0,0361669	0	1,586	2,8797	0,01562	0,897983	0,6279808
CG15148-RD	CG15147	0,165465	1,60735	2,95536	2,81357	0,202015	0,192389	0,207415	0,51983	0,6279808
CG15149-RA	btv	0,301503	0,260425	0,309423	0,425356	0,124898	0,00700198	-0,360774	0,129806	0,6279808
CG1514-RA	CG42634	0	0	0	0,48787	0,864627	0,947016	-0,534589	0,009174	0,6279808
CG15150-RA	Ykt6	24,9873	31,6148	35,3936	30,7115	3,9915	20,5683	-0,064185	0,82407	0,6279808
CG15151-RA	elfless	0,170007	0,154854	0,124386	0,10258	0,223811	0,168677	-0,037973	0,856945	0,6279808
CG15152-RA	rdo	3,84101	5,66069	6,86574	7,41777	8,16631	0	-0,352358	0,258159	0,6279808
CG15153-RA	CG15152	64,4583	54,025	70,9193	3,397	125,106	105,44	-1,033355	0,001718	0,6279808
CG15154-RA	CG15153	7,57308	7,31032	13,1798	27,5936	0	7,72353	0,370733	0,174941	0,13772387
CG15154-RB	Socs36E	21,4719	4,29452	19,6581	0,0204219	0	11,168	0,370733	0,174941	0,6279808
CG15155-RA	Socs36E	0,0194417	9,81345	0,0186652	17,4251	0	0	-0,112323	0,704481	0,13772387
CG1515-RA	CG15155	0,110372	0,100534	0,611352	4,17821	0,715756	0,145067	0,42671	0,067836	0,6279808
CG15160-RA	PCB	0,0159308	0,0138753	0,0155305	0,112343	0,0220562	0,0168857	0,129632	0,591506	0,6279808
CG15161-RA	PCB	0,0157486	0,0137234	0,0153501	13,8887	0,0226782	7,56894	0,900788	0,004796	0,6279808
CG15162-RA	PCB	0,0161766	6,57503	6,80776	0,016479	0,022405	0,0158773	0,226559	0,315266	0,6279808
CG15167-RA	PCB	0,0159887	4,13137	0,0144646	0,0162835	6,16597	9,89266	0,044335	0,715924	0,6279808
CG15168-RA	PCB	4,41758	7,31978	12,0533	0,0167428	5,24163	10,3467	-0,019079	0,946699	0,6279808
CG15169-RA	PCB	0,0150663	1,73816	11,6355	0,0165411	12,5462	0,0170657	0,188317	0,596875	0,6279808
CG1516-RA	PCB	4,87166	11,7887	6,79237	0,0157315	6,05233	17,8998	-0,562663	0,027092	0,6279808
CG1516-RB	PCB	8,31124	0	0,122465	14,6096	5,08584	0	-0,551742	0,02953	0,6279808
CG1516-RD	PCB	4,83777	0,11619	0,119018	19,2158	1,27779	106,93	-0,562663	0,027092	0,13772387
CG1516-RE	CG15160	5,9432	5,81627	5,43676	5,70893	3,57893	3,22417	-0,551645	0,029589	0,6279808
CG1516-RG	IFT46	1,54479	0,434988	0,354279	3,97562	2,49131	0,527276	-0,551742	0,02953	0,6279808
CG1516-RI	MESR3	38,5834	8,32721	8,31781	8,68541	7,37724	8,45824	-0,552727	0,029322	0,6279808
CG1516-RJ	CG15167	0	0,0990449	1,84611	3,12267	1,06161	1,31966	-0,563694	0,026858	0,6279808
CG1516-RK	CG15168	24,6826	16,6874	0,282192	3,92849	2627,63	0,146545	-0,552823	0,029264	0,6279808
CG1516-RL	CG15169	0,763883	0,37689	1,03895	6,28592	0,194649	0,603797	-0,552823	0,029264	0,6279808
CG15170-RA	na	3,53115	6,48606	3,51322	7,46222	0	2,42543	0,128606	0,671951	0,6279808
CG15171-RA	CG15170	0,156764	0,16064	31,1297	16,4457	852,103	40,4539	0,414365	0,214342	0,6279808
CG15172-RA	robl37BC	0,664556	1,29712	0,521041	0,748505	1,48565	1,77311	-0,370736	0,286267	0,6279808
CG15173-RA	CG15172	0,746237	13,8273	14,0955	16,4873	8,21416	9,70551	0,116601	0,635906	0,13772387
CG15177-RA	Ttc19	11,5004	11,9532	15,6683	21,0233	0,646673	14,9727	0,044335	0,715924	0,6279808
CG15178-RA	CG15177	0	0	0,0935646	0,0159254	0,0246513	0	NA	NA	0,13772387
CG15179-RA	sowi	0	0	0	6,57335	11,752	0	-0,070526	0,562659	0,6279808
CG1517-RD	sunz	0	0,0887706	0	1,04755	2,8907	0,144032	0,012492	0,958587	0,6279808
CG15180-RA	Stt3A	11,9538	13,2007	0,016423	5,78382	0,161271	14,5214	0,009311	0,977509	0,6279808
CG15184-RA	glob2	0,135587	0,205836	0	0,301231	7,91137	0,991648	0,01562	0,897983	0,6279808

gene_id	Symbol	W1_FPKM	W2_FPKM	W3_FPKM	MCM51_FPKM	MCM52_FPKM	MCM53_FPKM	FC	p-value	p-adj
CG15185-RA	CG15184	0	0	0,607466	97,7624	0,0252083	0	0,177527	0,555143	0,6279808
CG15186-RA	CG15185	0,0963205	0,233961	0,246596	21,5476	26,5327	13,0096	0,140312	0,567186	0,6279808
CG15186-RB	CG15186	6,7098	9,33989	0	0	0	0,122074	0,128631	0,59891	0,6279808
CG15186-RC	CG15186	0,0154143	0,137622	0,241756	0	0,799774	0	0,143018	0,56041	0,6279808
CG15188-RA	CG15186	0,904152	92,3468	86,331	0	4,82928	0,411342	0,600013	0,023653	0,6279808
CG15189-RA	Osi20	242,834	37,8878	16,6333	2389,12	84,695	104,245	0,721049	0,003067	0,6279808
CG1518-RA	Osi19	180,196	0,425848	205,416	1,68088	501,496	97,8812	-0,340399	0,09008	0,6279808
CG15191-RA	Prosalpha7	41,1878	37,0311	5,43905	0,0678931	3,51376	5,21324	-0,065903	0,786011	0,6279808
CG15194-RA	Prosalpha7	0,0754576	0,068732	26,3701	33,3992	21,3566	3,65777	-0,054434	0,878223	0,6279808
CG15196-RA	e(y)2	28,892	10,6763	22,1014	9,55705	63,4351	6,27844	0,521366	0,050191	0,6279808
CG15198-RA	CG43155	1,50172	6,33272	10,7165	9,30166	0,0174266	4,53107	NA	NA	0,6279808
CG15199-RA	CG15196	0,479126	0,0804632	0	0	10,7202	0,109728	-0,722147	0,032833	0,6279808
CG1519-RA	CG15198	0	0	1,53183	15,1394	2,36341	0,920225	-0,106919	0,705744	0,6279808
CG1519-RB	CG15199	6,38965	15,3456	999,777	54,3634	0,345299	2,3804	-0,106919	0,705744	0,6279808
CG15200-RA	WASp	11,8614	0,0167744	0,0178394	9,4748	0,026614	0,0200579	-0,014388	0,910154	0,6279808
CG15201-RA	WASp	4,17268	9,40382	0,0176803	0,0265744	0,0263625	0,0198683	0,269799	0,442862	0,6279808
CG15202-RA	WASp	10,4937	11,5482	11,484	18,4819	7,49054	7,91351	-0,639758	0,023395	0,13772387
CG15203-RA	CG15200	0,177942	0,0379565	16,7229	2,33181	0,04199	8,41214	-0,070263	0,816154	0,6279808
CG15207-RA	CG15201	3,55897	14,2683	0,0372573	18,0283	0	0	-0,795434	0,008914	0,6279808
CG15208-RA	CG15202	23,0696	118,657	5,86661	0,0310002	0,0832158	9,3194	0,012987	0,941653	0,6279808
CG15209-RA	CG15203	45,2977	0,0430827	1,10822	43,2249	3,23748	1,11331	-0,36526	0,188994	0,6279808
CG1520-RA	CG43901	0,668398	8,20995	0	1,6058	38,5976	0,342386	0,10103	0,635462	0,6279808
CG1520-RB	CG15208	0,119789	23,1862	0,0227726	24,1413	5,29258	36,3569	0,107072	0,615295	0,6279808
CG1520-RC	CG15209	21,7918	28,6045	34,1468	30,9291	3,75809	43,9246	0,12092	0,572344	0,6279808
CG15210-RA	CG15210	11,0126	10,0311	14,8019	63,5151	0,949167	69,3867	-0,187553	0,4702	0,13772387
CG15211-RA	CG15211	0,0472984	18,1211	2,34537	0,0551616	1,32196	1,79281	0,458275	0,06015	0,13772387
CG15211-RB	CG15211	0,0475024	0,00592931	53,3756	0,0554401	27,6573	38,492	0,473773	0,052341	0,6279808
CG15211-RC	CG15211	0,0509059	0	56,5548	0,0601464	10,4559	52,4575	0,458275	0,06015	0,6279808
CG15211-RD	CG15211	0,049458	0	21,8283	0,0581301	19,2838	14,5025	0,458275	0,06015	0,6279808
CG15212-RA	CG15212	688,34	13,5977	801,787	6,02335	3128,46	4,65377	0,895903	0,000284	0,13772387
CG15213-RA	CG15213	338,071	32,578	557,17	97,8533	13,1958	593,32	0,057365	0,840618	0,6279808
CG15216-RA	CG42748	5,13089	5,63108	4,58956	9,31051	6,66539	3,34947	-0,243003	0,400305	0,6279808
CG15218-RA	CycK	22,934	25,6257	28,7261	30,4149	19,9869	24,0383	0,013602	0,943008	0,6279808
CG15218-RB	CycK	0,0360152	0,0328051	0,0345767	0,0390928	0,0529514	0,0399074	0,013888	0,941723	0,6279808
CG15219-RA	CG15219	0	0,286057	0,180903	3,84601	0	0	0,059792	0,706172	0,6279808
CG15220-RA	cac	2,96552	2,55013	0,00758209	0,395758	4,78186	0,298472	-0,535075	0,044082	0,6279808
CG15221-RB	cac	0,00789751	0,0071936	0,00758209	13,5712	0,999871	3,45569	0,034915	0,920559	0,6279808
CG15224-RB	cac	0,671289	0,0071936	0,00757937	0,717902	0,0482561	0,0363687	0,338513	0,144311	0,6279808
CG15224-RC	cac	0,00789751	0,0071936	0,00757937	4,64178	0,446827	0,48025	0,177863	0,433874	0,6279808
CG15224-RD	cac	0,00789468	0,00719102	0,83722	1,14683	2,09524	1,04588	0,164922	0,473348	0,6279808
CG15224-RE	cac	0,00789468	1,55326	3,02308	0,0180442	0,325115	8,17871	0,0845	0,712232	0,6279808
CG15224-RF	cac	0,00789185	0,00718845	0,00757394	1,6962	0,668163	0,0184202	0,166958	0,46671	0,13772387
CG15224-RG	cac	0,00789185	0,00718845	0,00757394	0,0654327	0	0,107595	0,165299	0,472393	0,6279808
CG15225-RA	cac	0,00788903	0,00718587	0	0	0	0,245026	-0,116527	NA	0,6279808
CG15226-RA	cac	0,00788903	0,00718587	0,0279211	0	0,529755	0,503568	-0,502381	0,096878	0,6279808
CG15227-RA	RPA3	12,8007	33,7494	19,4875	15,9663	2,42027	0,0905897	-0,055549	0,749779	0,6279808
CG1522-RA	CG15221	2,02357	0,020821	44,686	0,0234197	16,4482	0,0239077	0,325632	0,216038	0,13772387
CG1522-RB	CkIIbeta	36,4221	38,9519	34,8537	37,8339	4,36488	19,141	0,325002	0,21626	0,6279808
CG1522-RC	CkIIbeta	0,0813073	0,0740603	0,0780599	0,0993061	14,8087	0,101375	0,322696	0,221823	0,6279808
CG1522-RD	CkIIbeta	0,0725576	0,0660904	0,0696596	0,0865264	0,134511	0,0883295	0,322065	0,222047	0,6279808
CG1522-RE	CkIIbeta	0,0701689	0,0639147	0,0673664	0,0831419	0,117201	0,0848744	0,338182	0,198317	0,6279808
CG1522-RF	CkIIbeta	0,0517971	0,0471803	14,6201	0,0584913	0,112616	0,0597101	0,334682	0,203919	0,6279808
CG1522-RG	CkIIbeta	24,2929	47,4593	22,4432	59,8531	0,0792268	38,2991	0,326663	0,214773	0,6279808
CG1522-RH	CG15225	0,137658	0	0	6,6631	0	0,149805	0,330197	0,208924	0,6279808
CG1522-RI	CG43668	0,143956	0,0983439	0,0863791	6,81431	5,47659	4,42057	0,333698	0,205067	0,6279808
CG1522-RJ	CAH14	0	0	4,78338	5,03421	7,66852	3,40418	0,330166	0,210826	0,6279808
CG15231-RA	CG1523	9,53727	0,0253168	3,92502	14,2289	0,0289163	3,59373	0,738503	0,03725	0,6279808
CG15233-RA	IM4	11,9153	2,3477	9,61431	0,23638	15,4152	0,0297563	0,07559	0,706677	0,6279808
CG15234-RA	CG15233	0	0,196688	0,20731	0,3853	0	0	-0,177229	0,620398	0,6279808
CG15236-RA	CG15234	0,37645	0,277583	0,103261	0,458226	0,447659	0,279979	0,287918	0,25911	0,6279808
CG15236-RB	CG15236	0,955449	3,61461	2,44272	61,2104	47,603	64,1136	0,280139	0,273874	0,6279808
CG15237-RA	CG15236	2,97804	0,0751703	0,0792298	144,019	139,284	108,551	0,227523	0,343403	0,6279808
CG15239-RA	CG15237	0,138912	0,0324513	2,48182	0,201799	122,224	127,513	0,342196	0,298009	0,6279808
CG1523-RA	CG15239	120,951	179,895	138,788	220,928	0,294154	140,118	0,164524	0,427028	0,13772387
CG15240-RA	RpS14a	877,991	995,834	0,10906	3,1067	11,8332	1,02417	0,238263	0,446155	0,6279808
CG15247-RA	RpS14a	0,0826291	0,0752643	64,3849	1,61742	8,97207	12,394	0,119561	0,687928	0,6279808
CG15249-RB	CG42541	1,11867	1,38125	199,928	2,14942	0,61876	0,891249	0,529154	0,067972	0,6279808
CG15249-RC	CG15247	5,62381	9,66259	38,5368	1,00003	0,690197	17,459	0,529154	0,067972	0,6279808
CG1524-RA	CG15249	0,0963671	0,0877778	209,226	0,197564	314,813	313,585	-0,61252	0,050862	0,6279808
CG1524-RB	CG15249	26,3912	38,4622	0,131429	20,6077	48,0963	0,201681	-0,612795	0,050828	0,6279808
CG15250-RA	CG43386	62,9797	82,7428	46,3613	130,459	86,6031	90,253	0,703357	0,005805	0,6279808
CG15251-RA	CG43386	153,205	190,017	160,253	109,768	134,105	145,476	0,342952	0,1869	0,6279808
CG15252-RA	CG15252	1,00074	0,656314	1,09528	0,850685	0,494342	0,0447977	0,553881	0,092684	0,6279808
CG15252-RB	CG15252	0,0395269	0,0360038	0,0379482	0,043278	0,0586203	0,424372	0,540082	0,100889	0,6279808
CG15253-RA	CG15253	1,08392	0,513402	10,4471	6,53256	0,280737	5,88303	-1,039148	0,003494	0,6279808
CG15254-RA	CG15254	0,864251	0,220194	4,49552	3,24224	15,7538	11,0853	-0,364448	0,230113	0,6279808
CG15255-RA	CG15255	2,35589	0,644088	25,0225	2,48339	0	0	-0,338157	0,325833	0,6279808
CG15256-RA	CG15256	0,313832	0,207898	0,818133	0,874336	0	16,4063	-0,403787	0,255819	0,6279808
CG15257-RA	Tim17b2	0,0913913	0	0	0	0	0,459568	-0,092508	0,454136	0,6279808
CG15257-RB	Tim17b2	0,113715	14,1404	11,7833	16,6218	0	0,0446645	-0,120017	0,498709	0,6279808
CG15258-RA	CG15258	0	0,0318672	6,83939	7,31062	0,0267657	0,0201722	NA	NA	0,6279808
CG15259-RB	nht	0,174251	14,5753	0,0182098	0	1,47573	52,6306	-0,015057	0,945026	0,6279808
CG15260-RA	CG15260	0	45,7393	92,2487	0,132575	0	0	0,01562	0,897983	0,13772387
CG15261-RA	UK114	65,5494	0	0	0	166,323	0	-0,447875	0,149505	0,6279808
CG15262-RA	CG15262	0,155595	0,0172768	0	10,7692	48,3704	26,588	-0,374993	0,29302	0,6279808
CG15263-RA	CG15263	0,112757	0	0,105728	0	0	0	0,01562	0,897983	0,6279808
CG15266-RA	Uxt	6,82379	0,0147131	0,0155077	3,10993	16,083	0,0170657	-0,351888	0,26542	0,6279808
CG15267-RA	dao	2,05312	25,622	21,9966	34,6199	1,7185	17,8776	0,298278	0,218444	0,13772387
CG15269-RA	CG15269	1,59196	1,96186	1,38453	2,13822	1,76661	0,805818	0,451294	0,156897	0,6279808
CG15270-RA	RpS14b	298,063	11,1284	4,93351	7,36052	1,79837	3,56128	0,385593	0,183393	0,6279808
CG15270-RB	CG15270	4,90256	7,32014	3,73828	0	0,977047	0	0,39022	0,178527	0,6279808
CG15274-RA	CG15270	0,0128311	0,0116874	0,0123186	4,45855	0,896805	2,6441	-0,155245	0,584501	0,6279808
CG15278-RA	GABA-B-R1	2,28881	7,56326	0	0,666515	2,03774	0,0239673	NA	NA	0,6279808
CG15279-RA	CG15278	0	0	0	0	0	0	0,302308	0,266307	0,6279808
CG15279-RB	CG15279	5,30064	3,50601	7,47244	4,80473	4,02425	0	0,274524	0,309159	0,6279808
CG15279-RC	CG15279	4,88627	3,14957	6,58766	5,23658	5,77333	2,49325	0,256294	0,351215	0,6279808
CG1527-RA	CG15279	5,59611	2,90284	4,03939	4,22998	3,57377	3,04823	-0,68514	0,031386	0,6279808
CG15282-RA	gammaCOP	0,0204211	0,0186009	7,39275	7,38754	0,0289163	0,425965	0,154052	0,532024	0,6279808
CG15283-RA	gammaCOP	30,8403	28,9693	0,00961212	1,20127	29,3214	12,486	0,025944	0,860432	0,6279808
CG15284-RA	CG15282	395,71	449,645	702,336	690,905	558,456	675,947	-0,153603	0,667388	0,6279808
CG15286-RA	Dpse\GA13625	0,113015	0,0130679	0	0,484617	1,29051	0	-0,041051	0,840969	0,6279808
CG15287-RA	Dpse\GA13626	0,465323	38,0539	1,11684	0,0277495	2,4787	9,41382	0,403892	0,178483	0,6279808
CG15288-RB	CG15286	0,053837	14,577	15,7492	16,2913	0,203992	0,883039	0,286038	0,379437	0,6279808
CG1528-RA	ms(2)34Fe	0,0546873	0,0498129	410,698	0,0213448	0,559455	3,35336	-0,290357	0,115692	0,6279808

gene_id	Symbol	W1_FPKM	W2_FPKM	W3_FPKM	MCM51_FPKM	MCM52_FPKM	MCM53_FPKM	FC	p-value	p-adj
CG1528-RB	wb	7,05191	3,01452	4,81291	10,2161	13,116	0,500728	-0,289482	0,11694	0,6279808
CG15293-RA	CG1529	0,0206638	0,018822	4,34013	0,0216143	0,0292767	3,77158	0,961082	0,007226	0,6279808
CG15295-RA	CG1529	4,76896	5,11657	1,52184	5,79925	3,14604	3,73181	-0,1643	0,61001	0,6279808
CG15296-RA	CG15293	1,984	0,576198	4,58247	5,54685	3,70559	0,485264	0,087616	0,61641	0,6279808
CG1529-RA	CG43347	0,85194	0	0,157281	0,580044	0	0,960952	0,075858	0,740544	0,6279808
CG1529-RB	CG15296	0	0,271518	0	0	0	0,218146	0,075858	0,740544	0,13772387
CG15302-RA	Ubr3	5,11306	0,0559007	13,4295	10,2197	0,315309	9,83786	-0,264608	0,37707	0,13772387
CG15304-RA	Or9a	0	0,0239056	0,0251966	0,0278198	0,432861	0,32623	-0,49391	0,092604	0,13772387
CG15306-RA	Neb-cGP	653,135	18,2714	636,414	6,61856	14,4652	14,1717	-0,041811	0,731452	0,6279808
CG15307-RA	CG15306	0	0	0	421,331	413,09	0	-0,771342	0,008252	0,6279808
CG15308-RB	CG43740	3,33185	2,9071	4,24259	5,14983	0,0976826	821,484	-0,903983	0,011069	0,13772387
CG15309-RA	CG15308	1,53037	0,983975	5,70413	11,2291	15,2099	12,6288	-0,116027	0,647033	0,6279808
CG15309-RB	CG15309	0,0358588	0,0326627	0,0344266	0,0389081	0,0527013	0,0397189	-0,118868	0,639148	0,6279808
CG1530-RA	CG15309	7,31237	11,5633	7,90557	12,5887	7,78405	9,36082	0,312736	0,116099	0,6279808
CG15311-RA	Ubr3	5,45844	5,71	0,0589195	0,0710406	0,096225	0,072521	-0,143435	0,647004	0,6279808
CG15312-RA	CG43902	0,720276	0,362024	1,25563	0	0,289449	0,516093	-0,111382	0,704312	0,6279808
CG15312-RB	CG15312	0,979188	2,81881	1,61576	18,7501	0,0783918	0	-0,050745	0,867758	0,6279808
CG15312-RC	CG15312	0,0368179	11,2958	0	0,0649486	78,5694	7,33201	-0,109361	0,708244	0,6279808
CG15313-RA	CG15312	1,02343	33,3871	22,3352	22,0372	34,4744	17,132	0,46851	0,188262	0,13772387
CG15314-RA	Cht6	9,98943	21,3259	522,146	0,29985	0,0648379	0,120342	0,396475	0,26346	0,6279808
CG15317-RA	Cht6	4,5886	11,2327	0,183816	11,3593	0,157691	3,58098	0,051914	0,848032	0,6279808
CG15319-RB	CG15317	8,94529	13,9783	10,9545	11,5391	13,3095	11,4921	-0,278609	0,261008	0,6279808
CG1531-RD	nej	3,01053	1,1539	4,28674	2,90582	1,29993	1,53487	-0,223735	0,434507	0,6279808
CG15321-RA	CG1532	33,1316	23,3237	43,2905	33,3161	40,3671	41,1733	0,196462	0,535732	0,6279808
CG15322-RA	nej	2,37985	3,10283	2187,56	38,8514	0,510246	0,61745	0,01861	0,939792	0,6279808
CG15324-RA	CG15322	0,0745105	0,0381248	0,0715345	0,0175687	4687,93	0,0420005	-0,013096	0,914398	0,13772387
CG15325-RA	Ir7c	0,0527763	59,0114	0	6,52676	0	0	-3,066198	1,68E-22	0,6279808
CG15326-RA	Ir7g	0,117573	0,0338951	0,034284	74,2144	24,7478	0,0190146	0,093526	0,710765	0,6279808
CG15329-RA	Ir7b	0,049458	0	0	0	0	0	-0,287038	0,417736	0,6279808
CG1532-RA	hdm	0,113066	2,75377	3,4024	0,461607	0,335868	0,364962	-0,170174	0,536126	0,6279808
CG15330-RA	CG15330	0	0,253391	2531,24	2251,23	2441,81	2710,57	0,024437	0,899292	0,6279808
CG15332-RA	CG15332	0,0397409	5,32434	0,233632	0,0376197	0,340667	0,27449	-0,317849	0,37447	0,6279808
CG15332-RB	CG15332	0,22597	9,59237	0	11,4205	0,0509561	4,18172	-0,378891	0,28951	0,6279808
CG15333-RA	CG15333	0	0,206945	5,44994	1770,65	0,0417934	9,02365	-1,202001	0,000665	0,6279808
CG15335-RB	cyr	4,08939	5,99169	12,1519	55,8551	1,51797	2,79881	0,342835	0,221909	0,6279808
CG15336-RB	CG15336	0	0	52,8956	13,6549	11,7402	10,3507	-0,070526	0,562659	0,6279808
CG15337-RA	CG15337	0	96,1369	1,59546	0,60073	1,66685	1,61316	-0,013765	0,912179	0,6279808
CG15343-RA	CG15343	1,90406	1,2656	1,97622	2,16962	1,51588	1,18881	-0,433361	0,211826	0,6279808
CG15345-RA	CR15345	3,18096	0,0137234	18,3277	11,072	3,14436	10,2064	0,222535	0,415567	0,13772387
CG15347-RA	CG15347	146,22	142,538	7,73226	148,723	4,22554	5,72386	-0,013355	0,960997	0,6279808
CG15350-RA	Cp7Fb	0	0,0883471	0,0210637	0,0230188	76,1638	0,0644292	-0,222673	0,443132	0,6279808
CG15351-RA	Cp7Fc	0,175113	0,239257	0,0218396	0,0239126	0	0,233887	0,335674	0,310278	0,6279808
CG15352-RA	rdgA	0,794727	1,44778	30,1099	18,9042	0	2,32859	0,109507	0,754283	0,6279808
CG15353-RA	CG15353	0,460138	16,4866	3,09776	2,75821	0	0,0122645	0,192887	0,525025	0,6279808
CG15356-RA	CG15356	3,0758	0,0106834	0,0857638	5,68032	0,151139	0,113907	-0,004163	0,98871	0,6279808
CG15357-RA	CG15357	0,544956	1,42497	0,0281191	0,111582	0,0434623	0,0327558	0,212747	0,304944	0,6279808
CG15358-RA	CG15358	0,514327	0,5236	0,726154	0,845095	1,20403	0,0122088	-0,522884	0,138796	0,6279808
CG15361-RA	Nplp4	3,38851	1,23459	0,0112603	83,0077	20,4444	1,71335	0,292901	0,399112	0,6279808
CG15362-RA	CG15362	7,10283	4,09472	0,0291128	65,1586	0,0451555	0,0340319	-0,259786	0,405503	0,6279808
CG15364-RA	Nost	0,18825	0,0264827	1,52248	0,441416	0,5979	0,450614	0,598693	0,092698	0,6279808
CG15365-RA	CG15365	8,97625	4,10665	8,3219	3,17625	4,96409	4,96027	0,499671	0,100786	0,6279808
CG15366-RA	CG44325	0,279983	187,713	1,56059	25,4198	27,2115	26,6478	0,001231	0,997073	0,6279808
CG15367-RA	Dlip1	1,9552	2,8769	3,36918	4,05658	5,72087	4,05656	-0,456854	0,155534	0,6279808
CG15368-RA	CG15368	0,337811	0,518849	5,65229	1,87483	4,05552	4,36311	-0,497928	0,128966	0,6279808
CG15369-RA	CG15369	65,373	4,57113	97,8546	5,36404	0	3,97276	-0,046437	0,896391	0,6279808
CG15370-RA	CG1537	7,44399	5,48535	7,66862	8,20377	0,0336348	16,214	0,482992	0,117244	0,13772387
CG15371-RA	CG15370	10,9236	14,8231	14,1351	0	4,47527	121,978	0,490032	0,150471	0,6279808
CG15373-RA	Gr8a	0,0436721	7,07549	0,0419278	0,0207761	6,09978	8,68101	-0,147668	0,658594	0,13772387
CG15373-RB	CG15373	0,0197674	0,0175488	0,322624	0,399973	12,2565	14,7747	-0,134886	0,686353	0,6279808
CG15374-RA	CG15373	0,337154	0,114776	0,0184965	0,0200857	4,66079	27,6926	-0,204746	0,246519	0,6279808
CG15375-RA	CG15374	0	0	0	0	2,20974	2,3893	0,027557	0,871332	0,6279808
CG15376-RA	CG15375	0,121911	3,14648	47,6876	34,5892	0,0395874	4,96691	0,099222	0,728597	0,6279808
CG15377-RA	CG43689	5,00078	18,1661	4,27634	0	3,4093	3,78979	-0,070504	0,817097	0,6279808
CG1537-RA	Or22c	0	0,0746731	0,0211198	9,93873	0,0269434	0,0235643	-0,314516	0,273462	0,6279808
CG15382-RA	CG15382	0,0675161	9,77583	10,7043	4,62437	0,0330862	4,1681	0,698691	0,007149	0,6279808
CG15385-RA	CG15385	3,62077	0,0614983	2,28536	34,2105	9,92111	8,22857	0,157503	0,580445	0,13772387
CG15386-RA	CG15386	36,3613	3,21016	4,91088	12,2395	42,0599	5,10159	0,169482	0,540546	0,6279808
CG15387-RA	CG15387	0,0971704	0,0257244	0,0932894	0,0322442	0,186904	0,140862	-0,078672	0,701509	0,6279808
CG15387-RB	CG15387	0,0746057	92,023	0,0716259	0,0334048	0,121179	0,0913277	0,081208	0,753132	0,6279808
CG15390-RA	tmod	22,7456	0	0	0	0,0350756	2,70041	0,171951	0,544071	0,6279808
CG15393-RA	tmod	0,0210321	0,103	0,0704853	0,0637447	0,0355082	0,0486537	0,175102	0,621578	0,6279808
CG15394-RB	tmod	0,0235425	0,0804202	0	0,0861314	46,2729	0,0429663	-0,04294	0,904417	0,6279808
CG15395-RB	tmod	0,0245301	0,0668739	0,0508579	0,233894	0,0387117	9,60084	NA	NA	0,6279808
CG15396-RB	tmod	0,0248157	25,8204	26,7955	0,0954178	3,02235	17,3427	-0,026752	0,917316	0,6279808
CG15396-RC	tmod	57,0984	0,0191575	0	0,121793	0	3,9935	-0,22552	0,398092	0,13772387
CG15398-RA	tmod	17,5239	0,0214441	0,256483	17,8895	16,0862	6,35369	0,002075	0,995274	0,6279808
CG15399-RA	CG15390	3,36626	4,10937	6,86343	5,5188	3,75391	3,90317	-0,106925	0,758004	0,6279808
CG1539-RA	Slh	1,35401	0,82222	1,29993	3,01448	0	12,4825	0,207075	0,329306	0,6279808
CG1539-RB	CG15394	1,40476	0,947819	4,24577	0,146582	0	0	0,220969	0,298707	0,6279808
CG1539-RC	CG15395	0	0	51,6569	0	3,23772	51,0675	0,221585	0,295178	0,6279808
CG1539-RD	Gr23a	0,0623552	0,13519	0,149662	0,197922	0,188577	0	0,216729	0,304296	0,6279808
CG1539-RE	Gr23a	0,184971	0,252727	0,0563883	0,0714257	0,0793339	0	0,217369	0,303113	0,6279808
CG1539-RF	CG15398	0,447972	0,0348705	5,26061	10,0535	6,96544	0,053962	0,216866	0,303987	0,6279808
CG1539-RG	CG15399	0,498515	0,206401	0,348076	0,404476	0,547866	0,511136	0,205381	0,33141	0,6279808
CG15400-RB	G6P	4,51833	3,42967	2,36448	3,23405	0,340361	1,18196	0,307895	0,276423	0,6279808
CG15403-RA	CG15403	0	0,0145918	0	7,99785	0,0191627	0,361395	-0,041811	0,731452	0,6279808
CG15404-RA	CG15404	0	1,48022	0	0	13,605	0	NA	NA	0,6279808
CG15406-RA	CG15406	0,942223	1,13215	2,23261	3,82597	8,06679	530,826	-0,704572	0,031009	0,6279808
CG15408-RA	CG15408	0,518797	0,326391	1,13199	0	0	0,567155	0,106626	0,764751	0,6279808
CG15412-RB	CG15412	1,15455	1,66836	1,25897	0,0165744	0,863987	0,858688	-0,03606	0,909963	0,6279808
CG15414-RA	CG15414	3,61764	5,73932	3,65716	4,12811	3,80492	2,48658	0,289094	0,317633	0,13772387
CG15415-RA	Spindly	1,21244	1,76995	0,413856	0,0130182	5,01133	0,814307	0,208053	0,538731	0,6279808
CG15418-RA	CG15418	0,139204	0,126797	9,58701	0,202419	0,274178	17,2721	0,255041	0,304829	0,6279808
CG15422-RA	CG1542	21,1768	10,1108	0,717936	0,960943	0,366004	0,108084	-0,798706	0,017004	0,6279808
CG15423-RA	CG15422	82,8571	44,9966	118,214	1,25241	515,75	0,300186	-0,533582	0,11268	0,6279808
CG15425-RA	CG15423	38,304	7,8802	0,331346	0,0158336	0,464591	0,301087	0,01562	0,897983	0,6279808
CG15427-RA	CG15425	0,142201	0	0	0	0	0	-1,080189	0,000109	0,6279808
CG15427-RC	tutl	0,0361136	0,0131998	0,0139126	0,0392091	2,73469	0,0400261	-1,07913	3,28E-05	0,6279808
CG15427-RD	tutl	0,0144914	0,0190123	0,020039	0,0149397	4,21184	1,70508	-0,539005	0,053528	0,6279808
CG15427-RE	tutl	0,0208726	12,1951	7,02566	0,0218436	0,0531089	0,0222988	-1,009437	0,000154	0,6279808
CG15429-RA	tutl	8,16035	0,256385	0,168895	22,1352	2,16149	8,70654	0,13384	0,66329	0,6279808
CG1542-RA	CG15429	0,316658	0,0328947	0,0346712	0,28915	0,181768	0,220665	0,014387	0,965676	0,6279808
CG15431-RA	Tbh	0,964363	1,48813	18,4189	100,604	5,94258	5,38116	-0,839846	0,003316	0,6279808
CG15432-RA	CG15431	1,1152	0,88883	1,17361	3,56331	8,58166	1,56884	-0,406433	0,091937	0,6279808

gene_id	Symbol	W1_FPKM	W2_FPKM	W3_FPKM	MCM51_FPKM	MCM52_FPKM	MCM53_FPKM	FC	p-value	p-adj
CG15433-RA	CG15432	22,3678	27,9024	4,48872	0	56,1459	6,82923	-0,16606	0,541746	0,6279808
CG15434-RA	Elp3	11,2459	6,75143	19,1788	5,70702	10,2601	13,4419	-0,097972	0,655605	0,6279808
CG15435-RA	Dpse\GA13724	0	5,05952	9,2007	11,0378	5,01524	54,7293	0,131401	0,621558	0,6279808
CG15436-RA	CG15435	2,85657	3,00484	2,79556	4,19669	1,68161	2,60391	0,460433	0,156625	0,6279808
CG15437-RA	CG15436	1,43021	1,45472	1,39598	1,59652	108,126	109,461	-0,093634	0,666108	0,13772387
CG15438-RA	morgue	6,79235	8,19593	0,424043	1959,65	0,148129	0	0,155678	0,512142	0,6279808
CG15439-RA	MFS18	17,2093	11,8525	11,8555	16,6017	11,2773	12,0522	0,201542	0,413562	0,6279808
CG1543-RB	CG15439	4,2259	0,24139	15,1887	12,4845	13,0321	12,6434	-0,949861	0,000439	0,6279808
CG15440-RA	CG1544	5,75593	10,3199	10,3176	5,0578	3,95775	9,70634	-0,35922	0,155245	0,6279808
CG15441-RA	CG1544	5,53561	1,92084	5,83166	16,6738	14,9715	10,4711	-0,682033	0,038389	0,6279808
CG15441-RB	CG1544	0,0223556	0,0203631	0,0214628	0,0234781	0,0318012	0,0239673	-0,636332	0,051307	0,6279808
CG15442-RA	CG15440	10,556	7,80467	15,5992	16,6039	12,0911	13,9022	-0,619281	0,043332	0,13772387
CG15443-RA	Gs1l	4,42825	4,85339	38,9537	8,74646	62,437	52,9869	0,107232	0,649971	0,6279808
CG15444-RA	Gs1l	18,145	16,1342	2266,04	25,0362	2368,98	2640,78	-0,652549	0,010333	0,6279808
CG15444-RB	mRpL27	1925,19	2232	4,95908	3,59072	2,1322	2,20944	-0,644846	0,009504	0,6279808
CG15444-RC	CG15443	5,59843	6,05892	7,97797	5,91476	5,61904	5,85497	-0,642559	0,009677	0,6279808
CG15444-RD	ine	13,5815	0,0171585	0,205645	0,0196193	0,304423	0,427108	-0,643591	0,009497	0,6279808
CG15445-RA	ine	0,0182194	2,09748	4,1744	3,28541	0,0745918	0,0561625	0,633231	0,005376	0,6279808
CG15445-RB	ine	2,73144	0	19,4803	0	0,484381	0,0562169	0,62131	0,006174	0,6279808
CG15445-RC	ine	0,0193506	0,0264285	0,0174918	0,0307568	0,160567	1,35838	0,62131	0,006174	0,6279808
CG15445-RD	CG15445	4,93111	0	0	0,276481	0,140402	0	0,633231	0,005376	0,6279808
CG15446-RB	CG15445	5,85976	1,24355	2,36838	1,42664	2,78575	0,1916	0,01807	0,946869	0,13772387
CG15449-RA	CG15445	0,0321746	0,00952971	0,0100444	0,883962	0,0144734	0,211356	-0,03318	0,922105	0,6279808
CG1544-RA	CG15445	0,0314997	0,0650898	0,0686049	0,0849644	0,115085	0,197187	-0,732723	0,000654	0,6279808
CG1544-RB	CG15446	0,151899	0,13836	0,0308895	0	0,0468625	0,010908	-0,716501	0,000886	0,6279808
CG1544-RC	CG15449	2,50287	3,03972	0,0198385	4,08387	4,06419	0,0220647	-0,716549	0,000893	0,6279808
CG15450-RA	CG1545	8,8009	9,82646	9,34781	12,8193	17,7347	4,83994	-0,061983	0,681756	0,6279808
CG15452-RA	CG15450	0	0,020233	1,05562	1,27067	0	0,0836265	-0,013096	0,914398	0,6279808
CG15456-RA	CG15452	0	0,078232	0,0824568	0,103873	1,44464	13,3871	0,410321	0,211598	0,6279808
CG15457-RA	CG15456	7,11234	5,64248	10,2424	63,8173	0,0324723	12,974	-0,084631	0,572257	0,6279808
CG15458-RA	Obp19c	0	0,0923106	0	10,9507	3,27007	0,0228985	NA	NA	0,6279808
CG15459-RA	CG15458	0	2,36806	0,0210427	0,0237147	0,030383	0,0244731	0,020837	0,894955	0,13772387
CG1545-RB	CG15459	0,0901855	0	24,9331	0,10509	0,0334583	0	0,320732	0,188202	0,6279808
CG15460-RB	PH4alphaSG2	0,0389904	0,0328947	0,0374332	0	2,01237	92,8391	0,110977	0,707675	0,6279808
CG15461-RA	PH4alphaSG2	1,54865	9,53484	3,43701	0	0	54,6511	-0,120295	0,702196	0,6279808
CG15464-RB	jb	0,214532	0,195411	0	87,5904	0	0,388782	1,030768	2,41E-05	0,6279808
CG15465-RA	CG15461	0	1,11456	0,451829	0,491831	0,121509	0,566488	0,93117	0,000169	0,6279808
CG15468-RA	rg	12,4671	13,5136	5,39481	3,1591	4,30007	3,47422	-0,013096	0,914398	0,6279808
CG1546-RA	CG15465	9,20608	12,6628	0,0746315	0,0514104	0,0290197	0,0524817	-1,009717	0,00144	0,6279808
CG1546-RB	CG15468	0	0,0839811	0	0	0,0540415	0	-1,009717	0,00144	0,6279808
CG15470-RA	CG42594	0,477427	0,521848	0,825045	8,77216	3,64475	0,0479599	-0,617174	0,074233	0,6279808
CG15471-RB	CG15471	0	0	0,0328858	1,14132	0,881319	0,0348702	-0,013765	0,912179	0,6279808
CG15471-RC	CG15471	0	0	0	0,208978	0	0	-0,013765	0,912179	0,6279808
CG15472-RA	CG15472	0	0	0,560512	0	0	0	0,044335	0,715924	0,6279808
CG15473-RA	CG15473	0,785518	1,23459	1,24212	18,3909	1,26496	1,42959	-0,225379	0,425987	0,6279808
CG15475-RA	CG15475	0,385793	0	1,24584	1,71888	0,657684	0,331331	0,260119	0,436022	0,6279808
CG15477-RA	CG15477	0	24,2852	0	9,99901	0,0190401	8,12963	NA	NA	0,6279808
CG15478-RA	CG15478	2,52485	3,1969	2,57873	2,49605	2,11959	2,32235	0,22735	0,38403	0,6279808
CG15479-RA	Mabi	0,197124	0,107732	1,2869	0,0125831	7,7629	1,40192	-0,451837	0,184757	0,6279808
CG15480-RA	cathD	50,5036	54,4308	0,00824854	0,00873719	0,0118346	0,0089157	-0,230855	0,515413	0,6279808
CG15481-RA	CG15480	0,65944	0	7,48769	15,0462	0,0382965	6,63292	-0,302265	0,384361	0,6279808
CG15482-RA	Ski6	6,88395	4,93751	17,3075	0,0125831	0,434455	16,4092	0,451302	0,114468	0,6279808
CG15483-RA	CG15482	0,0668332	0,806657	0,0641639	16,8868	15,3605	14,1881	0,044335	0,715924	0,6279808
CG15484-RA	CG15483	0	0,0709746	0,0748075	0	0	0	0,009541	0,977704	0,6279808
CG15485-RA	CG15484	0,585213	19,4588	19,9676	25,8849	67,4345	63,1938	0,003752	0,990754	0,6279808
CG1548-RA	Nepl7	0,775162	0,596508	1,46274	0,932486	0,0220018	0,832995	-0,051655	0,829674	0,6279808
CG15497-RA	CG15497	6,58227	5,30379	9,07397	6,49982	16,2757	11,6922	-0,348416	0,259662	0,6279808
CG15498-RA	CG15498	0,14503	6,13131	0,0835427	5,70902	2,51636	2,81885	0,011506	0,960404	0,13772387
CG15499-RA	CG15499	0	0	0	0	0	0	NA	NA	0,6279808
CG15503-RA	TTLL12	7,93517	9,43771	0,00824854	0,00873719	0,0118346	0,00889566	NA	NA	0,6279808
CG15504-RA	CheB93a	0	0	2,09929	0	1,10795	0,916445	-0,075072	0,812931	0,13772387
CG15505-RA	dmrt99B	1,44393	1,78636	0,0395573	0,0453013	0,986947	0,441941	0,628301	0,05379	0,13772387
CG15506-RA	Obp99d	10,6934	0,0375305	0,0387124	0,0442368	13,1027	16,2298	0,828561	0,00886	0,6279808
CG15506-RB	CG15506	38,628	18,2132	2,12418	26,5332	0,175856	0,064152	0,902692	0,004531	0,6279808
CG1550-RA	CG15506	0,0551704	4,12444	49,7936	6,82122	48,9122	39,7893	0,051942	0,803116	0,6279808
CG15510-RA	Prtl99C	0,156578	0,0855732	3,91838	0,0201595	3,22909	430,043	0,039235	0,885315	0,6279808
CG15514-RA	CG15514	14,8194	3,52471	2,8318	2,31	10,7378	4,00087	0,199872	0,354688	0,6279808
CG15515-RA	CG15515	47,1512	21,5879	110,21	7,92558	0,0271318	9,09054	-0,827661	0,018893	0,6279808
CG15517-RA	CG15517	0,900405	2,42332	5,7121	0,0794566	0,0221053	0,0553537	-0,595149	0,092377	0,13772387
CG15520-RA	CG1552	1,60524	8,93983	10,2189	8,5269	0,0240146	0,109459	-1,068197	0,001485	0,6279808
CG15522-RA	CG1552	0,0865243	5,56986	3,59428	8,1267	0,0242429	8,6226	-0,144666	0,650558	0,13772387
CG15523-RA	Capa	2,2251	2,52608	0,0151521	13,1514	6,93142	0,0166599	0,012718	0,963928	0,13772387
CG15524-RA	CG15522	1,40269	5,98015	0,0191036	0,0208023	0,0278776	0,021209	-0,150034	0,558001	0,13772387
CG15525-RA	Vps13B	0,893868	0,147365	144,242	8,25961	85,5975	93,9345	-0,4038	0,142098	0,6279808
CG15526-RA	Sas-6	0,0437299	11,6678	7,45461	2,51976	7,06638	6,09478	-0,368178	0,194241	0,6279808
CG15527-RA	CG15525	18,5113	44,4059	10,1997	8,49647	4,66503	6,10705	-0,126217	0,646771	0,13772387
CG15528-RA	CG15526	0,487285	0	0,280694	1,91487	14,5073	1,18049	-0,338329	0,327013	0,6279808
CG15529-RA	RpS28a	0,508276	0,771622	0,487975	9,34575	0	0	-0,452663	0,14751	0,6279808
CG1552-RA	CG15528	6,56623	3,81464	10,0764	7,98198	0	9,24601	-0,644439	0,061197	0,6279808
CG1552-RB	CG15529	3,29413	37,0513	4,79598	15,6808	5,92194	5,12624	-0,650451	0,05844	0,6279808
CG15530-RA	Nop17l	18,3135	0,00782896	0,00822715	0,00871408	0,0118033	0,00896203	-0,01793	0,956938	0,6279808
CG15530-RB	Nop17l	0,0174727	0,00782591	0,00823676	0,00872446	0,0118173	0,00896561	-0,01793	0,956938	0,6279808
CG15531-RA	Nop17l	0,0189347	0,00782591	0,00825176	0,00874066	0,0118393	0,00897279	-0,780518	0,013759	0,13772387
CG15531-RB	CG15530	2,4234	4,77275	1,55631	0,0352641	3,01319	0,0359989	-0,811222	0,010546	0,6279808
CG15532-RB	CG15530	0,0329939	0,0300531	0,0316761	3,94935	0,048152	2,10844	0,53355	0,00942	0,6279808
CG15532-RC	CG15531	0,064172	0,0584523	0,0616089	0,0748326	0,101361	0,076392	0,500586	0,010616	0,6279808
CG15533-RA	CG15531	2,74404	2,03761	5,12571	5,28168	7,61554	6,23476	0,192232	0,533331	0,6279808
CG15534-RA	hdc	10,8242	10,8076	10,5806	10,0745	5,81385	5,79677	0,679346	0,048297	0,13772387
CG15535-RA	hdc	0,0277202	0,0252495	3,48632	0,0294881	0,0399418	0,0301025	-0,280604	0,360901	0,6279808
CG15536-RA	CG15533	1,22918	0,0416804	0,154272	19,0325	16,2766	15,1482	0,243829	0,363749	0,13772387
CG15537-RB	CG15534	0,457589	2,60695	14,3469	9,92073	3,46996	0,025846	-0,276307	0,300222	0,6279808
CG15538-RA	CG15535	20,9135	13,7123	8,05912	0,182874	0,162172	0,210536	-0,244098	0,390182	0,6279808
CG15539-RA	CG15536	5,94333	7,53658	3,43467	3,88457	3,73541	3,92815	-0,131112	0,713979	0,6279808
CG15539-RB	CG15537	2,95537	15,2815	3,38972	0,0241936	0,0417153	0,0314391	-0,131112	0,713979	0,6279808
CG1553-RA	Osi23	0	0,0878208	3,75779	4,05477	19,6882	6,96839	0,101056	0,732806	0,6279808
CG1553-RB	CG15539	0,04223	60,5884	0,355994	46,0905	0,38051	1,03961	0,101324	0,73217	0,6279808
CG1553-RC	CG15539	0,370804	2,49401	0	0,042634	0,0505234	0,0380775	0,099218	0,737475	0,6279808
CG15543-RA	RpII215	10,1986	24,2058	5,26082	0,0887405	4,68897	73,3614	-0,037875	0,76416	0,6279808
CG15544-RA	CG15543	0	40,5579	54,8433	6,60438	151,144	26,4948	0,567889	0,049364	0,6279808
CG15545-RA	CG15544	1,74466	2,12405	1,33181	1,65126	0,891354	0,886956	0,794391	0,017192	0,6279808
CG15546-RA	CG15545	1,13501	1,6765	1,70814	0,904152	0,967216	0,67814	-0,089829	0,724569	0,6279808
CG15547-RA	CG15546	12,3256	11,6419	17,0811	13,0979	16,1469	13,4395	0,327552	0,214758	0,6279808
CG15548-RA	CG15547	0,0517431	0,124868	6,16807	8,05796	2,31084	0	0,044335	0,715924	0,6279808
CG15549-RA	CG15548	0	0	0,0648638	0	2,58744	0	-0,137135	0,476817	0,6279808

gene_id	Symbol	W1_FPKM	W2_FPKM	W3_FPKM	MCM51_FPKM	MCM52_FPKM	MCM53_FPKM	FC	p-value	p-adj
CG1554-RA	CG15549	0,0891311	0	1,05174	0	0	1,60058	0,044859	0,866087	0,6279808
CG15550-RA	cn	1,31878	1,09203	18,1846	4,40122	2,2192	0,0089157	-0,506948	0,156157	0,6279808
CG15551-RA	CG15550	0,864868	0,682744	48,8582	1,46331	75,4412	0,110934	-0,041811	0,731452	0,6279808
CG15551-RB	Ctr1C	0	0	0	0	0,396645	0	-0,041811	0,731452	0,6279808
CG15552-RA	Ctr1C	0	0	0	0	0,0830609	0	0,314323	0,272524	0,6279808
CG15553-RB	Sox100B	1,56272	1,54721	1,36984	6,87434	0,928006	109,752	-0,582026	0,091184	0,6279808
CG15553-RC	CG15553	0,0381794	0,0347764	0	9,62122	0,0294506	0,540299	-0,582026	0,091184	0,6279808
CG15554-RA	CG15553	0,531441	0,345766	5,20011	0,0416635	9,40605	1,77476	-0,462792	0,188679	0,6279808
CG15555-RA	CG15554	9,04414	3,46072	16,4539	0,734433	13,5129	3,3911	-0,190164	0,515085	0,6279808
CG15556-RA	ppk24	0,203254	0,209377	8,47123	0,0950104	68,3265	0,0969902	0,292546	0,300842	0,6279808
CG1555-RA	CG15556	2,38753	19,0115	27,1285	4,96397	25,561	3,0698	-0,66382	0,033124	0,6279808
CG15561-RA	CG15561	7,12465	3,86092	0,227956	0,221503	0,157341	0,402277	0,123556	0,709796	0,6279808
CG15563-RA	CG15563	10,2864	20,2622	7,24853	16,7527	7,1689	2,27648	0,495626	0,132684	0,6279808
CG15564-RA	CG15564	3,44145	6,36739	10,4555	13,9284	5,33746	6,50694	0,019279	0,953449	0,6279808
CG15570-RA	CG15570	0,0648757	0	0,0856412	0,159282	0	0,283544	-1,614201	5,91E-06	0,6279808
CG15571-RA	CG15571	0	0	0	0	0,521892	0	-0,289494	0,157417	0,6279808
CG15572-RA	CG15572	0,0415572	0	0,196602	0,97543	0,230859	0,215382	-0,013096	0,914398	0,6279808
CG15573-RB	Femcoat	0	0,12283	0	0,298968	0	0	0,058036	0,747319	0,6279808
CG15576-RB	CG15576	0	0	0,215771	0,830207	0	0	-0,041811	0,731452	0,6279808
CG15577-RA	CG15577	0	0	0	0,0452859	0,291002	0	-0,013096	0,914398	0,6279808
CG15578-RA	CG15578	0	0	0	0	0	0	NA	NA	0,6279808
CG15579-RA	CG15579	0,757751	0,690212	0,800235	44,457	0,0865659	0,432068	0,353461	0,267515	0,6279808
CG15580-RA	Kmn1	4,00701	0,0623908	10,3069	4,97584	5,49324	0,0722872	-0,053683	0,691569	0,6279808
CG15580-RC	CG15580	0	0	0,0273356	0	0	0,0972589	-0,053683	0,691569	0,6279808
CG15581-RA	CG15580	0	0	0,11534	0	0	0,0309759	0,01562	0,897983	0,6279808
CG15582-RA	Or83c	0	0	0	0	0	0	-0,820018	0,01466	0,13772387
CG15585-RA	Dsim\Obp83cd	57,9356	3,04766	29,7956	1,99343	2,809	141,536	-0,047755	0,849002	0,6279808
CG15589-RA	Osi1	0,106919	0,258056	0,460293	0,200705	3,84308	0,123732	0,373688	0,259506	0,6279808
CG1558-RA	Osi24	11,1801	159,047	0,0215649	127,204	42,0484	57,0548	-0,036001	0,902122	0,6279808
CG15590-RA	Upf1	5,5145	6,67604	6,08805	0,0233284	21,1131	18,0279	0,902598	0,005006	0,6279808
CG15591-RA	Osi5	8,84267	0,0214747	0,0528786	0,0248318	0,0336348	0,0253492	-0,029814	0,932258	0,13772387
CG15592-RA	Osi8	1,20138	1,53202	0,99961	0,139893	5,10888	205,466	0,809985	0,00715	0,6279808
CG15593-RA	Osi9	162,887	1,05947	0,851837	16,9729	0,417345	8,73461	0,490638	0,143789	0,6279808
CG15593-RB	Osi10	0,745102	1,45801	19,8547	0,0431081	118,017	9,62272	0,484442	0,147865	0,6279808
CG15594-RA	Osi10	0,374163	0,526331	0,86133	0,940614	10,6166	0,200117	0,018222	0,948634	0,13772387
CG15594-RB	CG15594	0,88522	1,73614	187,727	1,05688	36,3428	67,3338	-0,031425	0,914419	0,13772387
CG15595-RA	CG15594	2,04988	1,61638	0,741161	4,42325	71,6451	0,731704	0,622154	0,080934	0,6279808
CG15596-RA	Osi13	0,99166	151,835	2,27875	158,986	0,410539	1,74305	-0,020689	0,909957	0,6279808
CG15597-RA	Osi11	0	0,0993176	0	0,805769	0	0,142808	0,044958	0,899959	0,6279808
CG15598-RA	CG15597	3,77759	13,2719	16,2658	9,71835	0,0441066	0,504977	0,391968	0,246806	0,6279808
CG15599-RA	Osi17	9,12064	110,492	8,28838	135,294	121,73	84,0559	0,093996	0,792192	0,6279808
CG1559-RA	CG15599	0,192922	0,0394751	0,447608	0,0479031	0,064885	7,04024	-0,124112	0,621941	0,6279808
CG15601-RA	mys	58,0536	66,2275	0,0793288	79,2072	0,51158	0,103401	0,133413	0,594407	0,6279808
CG15602-RA	CG15601	5,78362	5,01554	7,5683	8,08507	5,75369	4,75357	0,243517	0,349175	0,6279808
CG15605-RA	CG15602	5,23875	5,54147	8,1662	5,59265	1,60922	13,9499	NA	NA	0,6279808
CG15609-RB	CG15605	0	0	0,0107508	0,0201533	0,0141926	0,0106964	-0,082311	0,697463	0,6279808
CG15609-RD	Ehbp1	3,09759	4,455	4,37385	17,0712	6,76521	0,0197343	-0,074454	0,723252	0,6279808
CG15609-RE	Ehbp1	8,61778	9,49912	6,7312	4,85032	0,0261846	2,13708	-0,069068	0,743789	0,13772387
CG15609-RF	Ehbp1	1,85949	2,24667	1,86637	5,28368	4,96647	4,98374	-0,121827	0,563376	0,13772387
CG15609-RG	Ehbp1	4,61239	2,52397	0,0168371	4,01958	6,00266	0,0184109	-0,083011	0,69487	0,13772387
CG15609-RH	Ehbp1	0,0173762	0,0158274	4,304	7,73746	0,0244287	0,0200043	-0,51005	0,01611	0,13772387
CG1560-RA	Ehbp1	0,0188161	0,017139	0,0180646	0,0180351	0,0265428	3,90589	0,098683	0,667941	0,13772387
CG15611-RA	CG1561	0,0316506	0,0288295	0,0303864	0,0339907	1,2348	0,034699	-0,18788	0,562582	0,6279808
CG15611-RB	CG1561	0,0315147	0,0287058	0,030256	0,0338336	0,0460406	0,0345386	-0,182213	0,574361	0,6279808
CG15614-RA	CG15611	0,0420419	0,0382946	0,0125427	2,27052	0,0155178	0,0116951	0,323501	0,300467	0,6279808
CG15615-RA	CG15611	11,8882	8,45982	11,1159	0	1,95966	1,56231	0,946076	0,002456	0,6279808
CG15616-RA	CG15614	1,82565	5,58564	0,528103	21,785	0,190707	615,679	0,392564	0,135367	0,13772387
CG15617-RA	CG15615	180,793	6,06405	8,11617	25,2205	9,86991	0,0174373	1,381942	0,000102	0,6279808
CG15618-RB	Acp53C14b	0,339897	0,704887	3427,61	0,38232	0	0	-0,182622	0,390311	0,13772387
CG1561-RA	CG15617	0,747932	0,634284	0,347789	0,885246	0	0	-0,178537	0,597636	0,6279808
CG1561-RB	THADA	2,84576	2,52464	1,47681	6,48138	2,26851	2,77199	-0,178537	0,597636	0,6279808
CG15623-RA	c-cup	0	0	8,20869	19,1998	0,569498	0,429208	-0,647483	0,020044	0,6279808
CG15624-RA	hoe2	2,45833	2,8229	0,0615492	0,243612	0,101246	0,0817787	-1,142515	2,5E-05	0,6279808
CG15624-RC	hoe2	0,0257539	0,0234584	0,0652864	36,8913	0,108509	21,695	-1,0998	7,07E-05	0,6279808
CG15625-RA	CG15625	0,074916	19,6173	0,950655	0,0194513	10,9635	5,86836	0,057371	0,801682	0,6279808
CG15626-RA	tank	24,7217	35,1361	0,0313629	4,74271	1,99197	0,0359028	0,513905	0,032231	0,6279808
CG15626-RB	tank	0,0326677	0,029756	8,01378	3678,87	5,64183	5,91662	0,514436	0,031665	0,6279808
CG15627-RB	Ir25a	2,66151	3,45611	0,0275972	6,00668	0,0415212	0,0312929	0,145575	0,591761	0,6279808
CG15628-RA	CG15628	3,3533	3,10547	17,0857	38,2894	2,08632	1,69052	0,069188	0,810137	0,6279808
CG15629-RA	CG15629	36,1736	34,4732	2,44962	20,6145	47,1917	40,0573	0,376359	0,075429	0,6279808
CG15630-RA	fipi	8,66885	0,307074	6,9725	5,99359	0,0356643	3,54867	-0,205344	0,364252	0,6279808
CG15631-RA	CG15631	0,0826362	5,01865	0,179425	0	0,0684689	9,7639	-0,237722	0,414576	0,6279808
CG15632-RA	Taf12L	0,449496	2,06247	0,593371	11,3228	0,025665	0,0193427	-0,353184	0,301072	0,6279808
CG15634-RA	Elba3	0,114397	0,0447261	2,8665	0,0550694	0,0760786	7,89048	-0,847456	0,014389	0,6279808
CG15635-RB	CG15635	0	13,6121	0,0682605	20,9958	0	0	-0,013096	0,914398	0,6279808
CG15636-RA	HP6	1,85259	0	0,644518	3,85468	1,7363	4,02057	-0,692547	0,04333	0,6279808
CG15638-RA	CG15638	0,203102	0,0109896	0,0134296	0,0144041	0	0	0,01939	0,899027	0,13772387
CG15639-RA	CG43779	0,859989	2,63254	2,70895	0	0,0170341	0	-0,535801	0,12232	0,6279808
CG15641-RA	CG15641	0	0,376838	0,177561	10,196	0,44353	0,716735	0,124168	0,632448	0,6279808
CG15642-RA	CG15642	0,89777	2,39819	0,616654	1,4139	10,6639	1,04875	0,035755	0,919642	0,6279808
CG15643-RA	CG15643	4,99178	1,18883	3,29153	2,69699	7,33016	40,481	0,061048	0,781328	0,6279808
CG15645-RA	cerv	5,50153	4,46308	4,78663	5,26323	1,81528	9,11841	-0,007372	0,976572	0,6279808
CG15645-RB	cerv	3,88393	2,97038	3,93987	4,33291	17,5001	0,675277	0,032006	0,899255	0,6279808
CG15646-RA	CG15646	0,696664	3,23583	0,123578	0,174157	5,30195	0,286376	-0,889965	0,00669	0,6279808
CG15649-RA	CG15649	25,1754	61,7703	23,1537	51,203	19,0882	25,0241	0,598954	0,075101	0,6279808
CG15650-RA	CG15650	21,5727	33,7351	77,7507	61,4875	85,5914	91,9556	0,378043	0,135165	0,6279808
CG15651-RA	CG15651	4,17502	0,0175334	0,0184803	0,218499	0,187277	0,0217789	-0,212664	0,356511	0,6279808
CG15653-RA	CG15653	11,1415	10,383	12,1185	3,86432	2,94554	4,40884	-0,496224	0,091606	0,6279808
CG15657-RA	CG15657	1,46013	1,11719	0,112145	0,158195	5,7653	0,161492	0,378462	0,229856	0,6279808
CG15658-RA	Lapsyn	23,5292	14,3517	15,6171	26,038	16,2126	13,2377	0,263688	0,18107	0,6279808
CG15661-RA	Ugt49C1	0,0362921	0,0330574	0,0348426	0,0394202	0,0533949	1,77149	-0,326698	0,186687	0,6279808
CG15661-RB	Ugt49C1	5,22348	5,29231	6,12321	6,29487	7,44728	4,66105	-0,327901	0,185205	0,6279808
CG15666-RD	BBS9	0,574279	1,56468	3,27019	0,0142885	0,0193539	0,0145862	-0,206444	0,484027	0,6279808
CG15667-RA	Sara	1,57198	2418,21	2,84531	0,370712	0,0502957	10,7884	0,091434	0,695213	0,6279808
CG15667-RB	Sara	7,02265	0,0554884	4,97872	9018,44	15,5194	9,26288	0,091504	0,694638	0,6279808
CG15669-RA	MESK2	0,0312416	0,028457	0,0299938	0,0344528	0,0371024	0,0351707	0,241805	0,197499	0,6279808
CG15669-RB	MESK2	24,6395	27,3699	26,8287	31,1978	0,0372729	16,1285	0,051499	0,777503	0,6279808
CG15669-RC	MESK2	0,033833	0,0308175	0,0324817	0,0376412	0,0199643	0,0384255	0,167184	0,35846	0,6279808
CG15669-RD	MESK2	18,0587	20,3811	18,7858	14,9014	0,0338645	9,59671	0,243948	0,193227	0,6279808
CG15669-RE	MESK2	0,0136192	0,0124053	0,0130753	0,014012	0,033916	0,014304	0,242206	0,196686	0,6279808
CG15669-RF	MESK2	0,0136954	0,0124747	0,0131484	0,0140929	265,362	3,38423	0,282422	0,13647	0,6279808
CG15669-RI	MESK2	0,0258648	0,0235594	0,0248317	0,0273919	0,0985628	0,0279626	0,052105	0,776865	0,6279808
CG15669-RJ	MESK2	0,0259766	0,0236613	0,0249391	0,0275177	8,50763	0,0280911	0,047894	0,795625	0,6279808
CG15669-RK	MESK2	0,0143032	0,0130283	0,0137319	0,0147391	0	0,0150463	0,169334	0,353136	0,6279808

gene_id	Symbol	W1_FPKM	W2_FPKM	W3_FPKM	MCM51_FPKM	MCM52_FPKM	MCM53_FPKM	FC	p-value	p-adj
CG15671-RA	C901	4,29285	3,61739	3,97615	10,1969	0,0338517	3,77612	0,077815	0,737716	0,6279808
CG15673-RB	cv-2	10,3764	7,07908	9,8813	0,0149464	0,020245	0,0152579	0,028485	0,91793	0,13772387
CG15674-RA	CG15673	7,19948	5,16313	11,5563	15,8449	5,19571	0,34923	-0,161676	0,573746	0,6279808
CG15676-RA	CG15674	6,1977	0,291813	9,22157	6,25572	0,0509852	0,0719008	NA	NA	0,6279808
CG15678-RA	CG15676	0	1,0195	0,12176	0,336862	8,99674	15,2342	-0,733272	0,010778	0,6279808
CG1567-RA	pirk	3,9811	3,17886	7,61936	10,7103	7,57667	19,8971	-0,177928	0,594478	0,6279808
CG15684-RA	CG42668	7,78068	5,88781	464,107	9,86577	1,12227	0,898096	0,020975	0,940778	0,6279808
CG15685-RB	Ir92a	0,164368	2,05181	0,105202	23,8655	8,51791	0	0,264755	0,372908	0,6279808
CG15693-RA	rod	0,010012	6,29921	0,0196055	0,0213482	2,94132	0,368905	-0,256305	0,348395	0,6279808
CG15695-RA	RpS20	1538,95	1865,99	2707,1	2657,79	3358,67	3628,12	-0,53982	0,07268	0,6279808
CG15696-RA	CG15695	4,61586	72,5583	6,03661	107,216	39,9429	39,0925	0,145204	0,679665	0,6279808
CG15697-RA	Dsim\GD19379	0,795358	2,73675	126,905	5,21074	7,39078	6,42319	-0,449448	0,105919	0,6279808
CG15697-RB	RpS30	0,127395	0,11604	0,122307	0,178317	0,241531	0	-0,449467	0,105917	0,6279808
CG15699-RA	RpS30	1926,5	2176,74	3563,62	3826,67	5017,85	15,309	2,067535	2,73E-09	0,6279808
CG1569-RA	Strn-Mlck	7,54291	11,1304	6,80714	9,07175	24,5293	0	-0,495872	0,096154	0,6279808
CG15701-RA	CG15701	0,0200615	5,80761	0,0192602	0,036862	7,2722	0,0376302	-0,264603	0,344662	0,6279808
CG15704-RA	prim	2,44193	2,48596	5,92996	7,85209	1,62649	0,0346275	-0,355666	0,313617	0,6279808
CG15705-RA	CG15705	0,351467	1,1646	0,150245	0,473848	0,0459458	1,73455	-0,091622	0,584546	0,6279808
CG15706-RA	CG15706	9,58475	2,13371	10,2459	162,548	2,7737	1,45115	-0,049483	0,852356	0,6279808
CG15707-RA	krimp	0,596915	0,142961	2,32967	0,247893	0,474448	0	1,235287	0,000557	0,6279808
CG15708-RA	CG15708	0	143,433	0,1008	1,70085	20,2238	2,71864	-0,013096	0,914398	0,6279808
CG15709-RA	CngA	0	1,63449	0,724164	0,301519	60,6371	1,80534	-0,064246	0,835805	0,6279808
CG15710-RA	Spase25	0,0858128	0,117246	0,0113044	0,0755421	29,45	1277,24	NA	NA	0,6279808
CG15711-RA	CG15710	0	0	0	0,162958	0	0	NA	NA	0,6279808
CG15712-RA	CG15711	0	0	0	0	0	0	0,509999	0,117873	0,6279808
CG15715-RA	CG15712	6,35148	10,7776	0,29678	6,47606	0	3,17927	-0,126753	0,616986	0,6279808
CG15717-RA	CG15715	106,52	0,0157241	0,0165732	0,0573709	0,0242627	0,0585664	-0,607086	0,023479	0,6279808
CG15719-RB	CG15717	29,2622	27,618	41,0829	42,0583	13,8602	58,7444	0,335607	0,215997	0,6279808
CG1571-RA	CG15719	0,292371	0,133156	0,374258	0	0	0	0,433353	0,178997	0,6279808
CG15720-RA	CG1572	65,3965	242,923	6,06056	5,37474	5,55282	429,765	-0,015522	0,954846	0,6279808
CG15720-RB	CG1572	271,352	12,8812	0,0711439	26,5632	2,99662	31,8046	-0,084253	0,754184	0,6279808
CG15721-RA	rad	0,875219	2,9055	1,38177	2,49697	0	22,4784	0,01562	0,897983	0,6279808
CG15725-RA	rad	1,24628	1,3464	2,06064	27,5963	0,916729	0,494751	-0,469527	0,155083	0,6279808
CG15727-RA	CG15721	0	0,0560396	0,138387	0,168737	0	0	0,2324	0,386605	0,6279808
CG15728-RA	CG15725	0,354294	5,77943	12,6907	12,9674	2,86633	12,9272	0,212837	0,551141	0,6279808
CG15729-RA	Aven	4,30929	0,0160597	6,1059	0,0701333	0,094996	38,6166	-0,111464	0,754789	0,13772387
CG1572-RA	CG15728	0,0578598	35,0943	0,0555489	8,80849	0,265786	4,14041	-0,240936	0,411473	0,6279808
CG1572-RB	hwt	0,386409	16,0029	0,525548	1,10937	77,385	68,4968	-0,240994	0,411357	0,6279808
CG15730-RA	Mks1	0,17988	51,6533	0,230261	451,203	0,211188	38,042	-0,145728	0,67753	0,13772387
CG15731-RA	CG15731	283,142	0	5,87669	0	148,778	285,62	0,15852	0,555977	0,6279808
CG15732-RA	Ir11a	0	0,075993	0,0480582	0,0895666	0,0753162	0	0,00299	0,988751	0,6279808
CG15734-RA	CG15734	0,155961	0,805709	0,149732	0,0718933	0,0108531	69,371	0,68452	0,042548	0,6279808
CG15734-RB	CG15734	0,974885	0,14206	1,01394	0	0,0108491	0,0733914	0,68452	0,042548	0,6279808
CG15735-RA	Lsm12	36,1344	0,0573932	46,91	17,959	33,3911	0,601693	0,128758	0,582936	0,6279808
CG15736-RA	Chrac-16	8,17484	7,95583	5,81349	9,07699	3,04061	4,97322	-0,268076	0,400184	0,6279808
CG15737-RA	wisp	0,406601	0,335088	27,067	31,8688	91,2998	102,724	0,206279	0,562134	0,6279808
CG15738-RA	sicily	5,29321	19,0394	51,3192	18,7123	23,0396	28,5421	-0,275534	0,355417	0,6279808
CG15739-RA	CG15739	13,7346	0,0347363	0,0213328	24,5329	12,0992	12,1138	-0,102967	0,686912	0,6279808
CG15740-RA	CG15740	77,9283	5,75542	21,1156	15,1378	0,283551	16,88	0,835807	0,007707	0,6279808
CG15741-RA	CG15741	2,10536	3,24535	1,39934	2,18881	0,163925	0,790631	0,577991	0,105568	0,6279808
CG15742-RA	CG15742	0,107967	0,0155883	0,0164301	0,0885641	0,0243953	0,0183858	0,079884	0,644003	0,6279808
CG15742-RB	CG15742	0,0977454	0,107732	0,11355	0,940143	0,145834	0,164252	0,173708	0,412757	0,13772387
CG15742-RC	CG15742	0,0888117	0,0966589	0,101879	20,0228	0,132028	0,142052	0,173708	0,412757	0,6279808
CG15743-RA	CG15743	12,7022	11,1102	14,1725	13,8533	4,79085	9,51742	0,344213	0,212948	0,6279808
CG15744-RA	CG15744	4,24076	3,77177	4,98264	31,637	1,89947	3,30383	-0,025787	0,930623	0,13772387
CG15745-RA	IP3K2	0,0268637	4,10025	2,64103	4,04958	2,87157	0,0291124	0,591021	0,001351	0,13772387
CG15745-RB	IP3K2	20,4282	2,35393	2,68914	5,78759	5,6852	11,8756	0,58902	0,001463	0,6279808
CG15747-RA	CG15747	4,48554	4,07416	0,895052	2,70353	3,8165	5,11295	-0,119242	0,614226	0,6279808
CG15749-RA	dmrt11E	0,553546	0,76958	6,36808	0,335009	0,309669	0,341989	0,893544	0,012444	0,6279808
CG15753-RB	CG1575	5,1208	3,70535	8,72983	9,09362	0	53,5515	-0,233596	0,461077	0,6279808
CG15754-RA	Neto	0,834291	0,937247	1,20145	1,72177	0,443085	1,07092	0,010141	0,937924	0,6279808
CG15756-RA	CG15754	0	0	0	277,805	0	174,967	-0,219287	0,433605	0,6279808
CG15757-RA	CG15756	0,110495	0,100646	2,7708	0,895011	0,0390505	0	0,50872	0,146063	0,6279808
CG1575-RA	Cpr12A	1,74683	0	0,176803	0,229232	0,0379545	0,129104	-0,248288	0,399543	0,6279808
CG15760-RA	CG15760	2,14069	2,78184	2,47993	0,250908	2,30659	0,577693	-0,155558	0,577759	0,13772387
CG15764-RA	CG15764	0	0	0	0,158261	7,90539	30,1758	-0,080649	0,53498	0,6279808
CG15765-RA	CG15765	0,0214555	54,2756	2,20405	4,06428	10,0901	1,5145	-0,221637	0,387116	0,6279808
CG15766-RA	AgmNAT	9,69316	10,632	11,2062	0	0,174654	6,16406	0,093058	0,765821	0,6279808
CG15767-RA	CG15767	0,734687	0,39734	2,58411	5,61529	8,10785	8,95897	0,576138	0,093008	0,6279808
CG15770-RA	mRpL52	31,1151	2,98635	24,7775	18,2775	28,0921	28,2119	-0,36629	0,211221	0,6279808
CG15771-RA	CG42699	1,37328	9,92498	2,05371	3,84161	2,49607	1,87725	0,235601	0,465615	0,6279808
CG15771-RB	CG15771	26,13	2,11687	27,4517	65,1806	2,39235	21,3267	0,274981	0,393051	0,13772387
CG15772-RA	CG15771	5,41855	63,4522	5,93922	0,0315494	17,7557	3,5866	-0,160466	0,583222	0,6279808
CG15773-RA	CG15772	0,840157	1,21543	0,782877	1,86536	0,845027	5,28648	0,41189	0,236991	0,6279808
CG15779-RA	CG15773	0,988609	0,740128	3,14689	12,509	4,92457	2,93106	0,03899	0,897314	0,6279808
CG1577-RA	Gr5a	0,0742426	0,0516769	24,5593	0,402131	0	0,127428	-0,235262	0,47754	0,6279808
CG15784-RA	CG1578	5,17124	6,92112	6,05154	7,4691	34,5723	4,75794	-0,263194	0,445205	0,6279808
CG15785-RA	CG15784	5,58048	4,5427	24,1893	15,8643	0,472379	0,661098	0,319774	0,23999	0,6279808
CG15786-RA	CG42749	28,1146	0,554349	0,703528	0,0200919	33,3733	0,412825	-0,325123	0,283156	0,6279808
CG1578-RA	CG42749	19,9885	19,5444	24,1924	3,99739	0,52559	3,11863	0,052906	0,807544	0,6279808
CG15792-RA	zip	5,14334	2,33275	13,7321	11,3015	0,0423085	0,02973	-0,034044	0,899877	0,6279808
CG15792-RB	zip	26,8774	0,0249564	2,54178	0,23718	0,0210869	7,39801	-0,034277	0,899193	0,6279808
CG15792-RC	zip	0,0102592	11,2656	2,37001	1,08325	0,779215	3,11618	-0,033726	0,900833	0,6279808
CG15792-RD	zip	0,0103621	0,013736	0,0263041	0	4,03905	13,5476	-0,033493	0,901516	0,6279808
CG15793-RA	Dsor1	6,8829	12,5152	9,2748	15,3975	4,36519	5,42277	0,091015	0,68989	0,6279808
CG15797-RA	ric8a	8,3687	0,636064	0,44404	1,69819	4,1624	8,10669	-0,02582	0,898907	0,6279808
CG15800-RA	CG15800	0,417321	0,0198854	32,6894	0,754955	0	41,2297	0,034192	0,855763	0,6279808
CG15803-RA	CG15803	0,809393	0,791927	309,871	9,37353	4,47123	10,4741	-0,415785	0,121239	0,6279808
CG15804-RA	Dhc62B	0,18824	2,49633	0,135541	2,65143	1,6684	0,0937046	0,152502	0,65574	0,6279808
CG15804-RB	Dhc62B	0,0056763	0,143872	0,00544958	0,341158	0,204886	0,00585342	0,163765	0,631444	0,6279808
CG15807-RA	Cyp313a5	0,0659879	0	0	214,477	0	0,0261881	-0,048652	0,86735	0,6279808
CG15811-RA	Rop	35,3753	60,6407	2,98877	56,049	18,5256	16,6555	-0,385742	0,055639	0,6279808
CG15812-RA	CG15812	3,01658	5,17959	5,89206	0,0232134	2,2654	5,90038	0,254687	0,25367	0,6279808
CG15812-RB	CG15812	8,30968	10,6031	9,55974	12,6633	0,0331969	20,7273	0,254687	0,25367	0,6279808
CG15814-RA	CG15814	13,9321	15,2913	19,566	10,1671	0,0419506	14,4166	-0,030657	0,894362	0,6279808
CG15814-RB	CG15814	9,61004	0,0370911	10,2622	2,96312	12,4995	8,13379	-0,028726	0,900618	0,6279808
CG15814-RC	CG15814	2,53907	0,0328051	0,0345767	3,30716	11,9767	3,22313	-0,028759	0,900626	0,6279808
CG15814-RD	CG15814	0,0404711	7,87044	5,38137	139,742	0,0529514	3,37894	-0,012096	0,958369	0,6279808
CG15816-RA	raskol	2,20977	15,1383	4,21361	28,3519	3,13915	4,44903	-0,26723	0,354806	0,6279808
CG15817-RA	Usp1	1,50152	1,25476	6,4799	3,91065	0	2,09414	0,047306	0,808356	0,6279808
CG15817-RB	Usp1	6,69948	6,1544	0,8468	5,78881	2,79191	3,81213	0,045025	0,817276	0,6279808
CG15818-RA	CG15818	6,65513	171,408	5,49829	40,8641	2,42169	8,12672	-0,070546	0,806943	0,6279808
CG15819-RA	Ziz	26,5234	0,0300932	18,9176	20,6868	14,9104	14,8311	0,499481	0,05327	0,6279808
CG15820-RA	CG1582	5,289	0,0157405	0,0165906	0	1,73214	4,03461	-0,68082	0,056839	0,6279808

gene_id	Symbol	W1_FPKM	W2_FPKM	W3_FPKM	MCM51_FPKM	MCM52_FPKM	MCM53_FPKM	FC	p-value	p-adj
CG15822-RB	CG15820	0,558545	0,508762	2,35944	1,6562	3,23552	2,40061	0,218062	0,438497	0,6279808
CG15822-RC	CG15822	9,11166	7,31181	10,464	1,58829	7,63343	0,908378	0,1907	0,503429	0,6279808
CG15824-RA	CG15822	10,4241	7,51299	9,37353	9,3116	11,1298	9,11176	0,315528	0,365979	0,6279808
CG15825-RA	CG15824	0,0607044	40,1808	5,91019	0,0397834	8,46105	0,0405049	-0,559731	0,073589	0,6279808
CG15825-RB	fon	91,7987	0,0392179	143,667	0,10098	65,7421	80,8125	-0,560573	0,073193	0,6279808
CG15828-RA	fon	47,1757	36,0338	69,883	9,97061	200,075	132,402	-0,078038	0,802634	0,6279808
CG15828-RB	Apoltp	2,14196	32,6802	0,0695069	112,785	19,111	16,2305	-0,078373	0,801809	0,6279808
CG15829-RA	Apoltp	6,50133	1,57909	462,269	34,1456	0,0322843	0,0243314	-0,228131	0,513308	0,6279808
CG1582-RA	Acbp6	30,5029	2,52581	0,0100725	39,4723	81,1876	0,0110351	0,228874	0,3814	0,6279808
CG15831-RA	GIIIspla2	5,07503	2,63254	13,2904	4,81458	0,146815	0,613248	-0,263653	0,180107	0,6279808
CG15835-RA	Myo81F	0	0	0	0,0200247	0,0271236	0,020442	0,341203	0,270776	0,6279808
CG1583-RA	Kdm4A	2,48234	1,72556	5,06428	2,68759	5,64825	2,23889	0,012171	0,971715	0,6279808
CG15841-RA	Orc6	3,70044	2,84189	244,278	5,41099	197,996	0,298191	NA	NA	0,6279808
CG15844-RA	Acp33A	0	0	0	0	0	0	0,133933	0,61695	0,13772387
CG15845-RA	CG43324	3,64916	4,00056	3,52844	3,56329	4,70426	2,75053	0,369285	0,089162	0,6279808
CG15845-RB	Adf1	4,99924	7,72741	6,56305	42,5692	5,2051	12,9049	0,360964	0,096287	0,6279808
CG15845-RC	Adf1	12,3534	14,4619	14,6922	0,0528605	6,07059	0,0596043	0,368395	0,089789	0,6279808
CG15848-RA	Adf1	19,1761	16,9112	17,6097	26,9223	8,6929	5,45537	-0,743804	0,02765	0,6279808
CG1584-RA	Scp1	7,37514	5,59309	0,114163	6,4835	0	13,7118	0,158297	0,560009	0,6279808
CG15855-RA	Eip63F-1	22,6407	2,4013	19,7534	0	0	9,47457	0,32273	0,159534	0,6279808
CG15860-RA	pain	17,1554	16,1614	16,7939	18,4794	14,8632	12,7916	0,016158	0,935439	0,6279808
CG15861-RA	CG15861	6,32839	13,4219	6,93933	9,34787	6,4963	43,1193	0,502207	0,10312	0,6279808
CG15862-RA	Pka-R2	6,91678	9,96935	9,19812	26,3764	18,3379	5,54942	-0,297076	0,177029	0,6279808
CG15862-RB	Pka-R2	0,0309682	0,028208	0,0297313	1,55126	0,607637	0,0338947	-0,296249	0,178293	0,6279808
CG15862-RC	Pka-R2	11,8693	10,0991	9,26328	10,9148	0,282868	12,2313	-0,289812	0,188956	0,6279808
CG15862-RD	Pka-R2	5,90637	9,71335	7,19295	6,82574	0,270031	7,2778	-0,391191	0,073871	0,6279808
CG15862-RE	Pka-R2	0,0467407	0,0425747	0,0448739	0,0397772	5830,73	0,0531938	-0,346711	0,128645	0,6279808
CG15863-RA	Pdrg1	0,165604	10,2139	4,37501	3,32416	3,91482	3,11294	-0,066392	0,836657	0,6279808
CG15864-RB	CG15864	0,857015	4,69563	6,94215	9,30866	0,458896	3,91258	0,353656	0,322116	0,6279808
CG15865-RA	CG15865	0,817771	0,744882	1,05297	0,772054	0,776237	0,76491	0,048098	0,866033	0,6279808
CG15871-RA	Crk	54,7524	0,0216368	0,0238783	0,0250299	0,0334583	0,0268252	-0,276618	0,266107	0,6279808
CG15873-RA	Crk	95,2744	0,0226463	0,0225219	0,0262671	0,0339031	0,0252162	NA	NA	0,6279808
CG15874-RA	Crk	0,0677932	2107,47	0,0228053	31,2	0,035579	0,0255514	NA	NA	0,6279808
CG15876-RA	mRpL38	20,9382	23,6355	33,8284	2,7453	33,2266	3,38473	-0,060347	0,691408	0,6279808
CG15877-RA	CG15873	0	3,47303	34,1665	0	0	0	0,064213	0,832529	0,6279808
CG15878-RA	Pgam5-2	0	0	0,0252568	0	0,35275	20,6089	0,60853	0,041898	0,6279808
CG15879-RA	CG15876	0	0,0374838	0	0,237293	0	0,242238	-0,084095	0,793548	0,6279808
CG1587-RA	CG15877	6,26738	4,96007	13,6377	19,7272	6,84391	5,60189	-0,099829	0,691989	0,6279808
CG1587-RB	CG15878	1,64076	1,99269	1,65301	3,01721	0,4206	12,9956	-0,099378	0,69325	0,6279808
CG1587-RC	CG15879	2,37591	1,57924	4,19213	2,18752	3,28021	2,99063	-0,099763	0,692135	0,6279808
CG15880-RA	CG15880	1,13067	2,12537	1,15952	4,34241	0,914097	4,89142	0,598502	0,027507	0,6279808
CG15881-RA	Ccdc58	2,13527	2,18676	2,54608	0,356794	22,7395	34,4366	-0,217684	0,36158	0,6279808
CG15881-RB	Ccdc58	20,0122	0,0097237	0,0100651	0,0526914	1,20399	0,155159	-0,208961	0,362619	0,6279808
CG15882-RA	CG15882	0	259,196	5,27105	94,5452	2,81772	2,56412	0,063548	0,603667	0,6279808
CG15883-RA	Obp18a	38,6117	5,40582	0,915148	3,69718	0,472174	0,0476879	-0,122333	0,68051	0,13772387
CG15884-RA	Cpr97Eb	12,3579	17,4119	20,2855	2,37818	1,16511	0,473226	1,036989	0,000186	0,6279808
CG15887-RA	apn	97,8167	95,2489	22,7872	0,0234697	22,4117	16,7795	0,666523	0,004346	0,6279808
CG15888-RB	CG15888	0	0,0822969	0	0,183519	0	0,112795	-0,013096	0,914398	0,6279808
CG15889-RA	Ravus	5,85213	1,62366	2,48389	9,11557	2,50866	1,27415	0,046273	0,853368	0,6279808
CG15890-RA	CG15890	11,8663	0,0367289	0,0387124	0	0,0599191	0,0451586	-0,273704	0,188169	0,6279808
CG15892-RA	CG15892	0,0589086	1,41008	0,0565557	8,66146	14,6362	7,52833	0,110167	0,711469	0,6279808
CG15892-RB	CG15892	4,98971	0,053658	7,88911	21,3833	17,7013	15,2042	-0,105068	0,699499	0,6279808
CG15894-RA	CG15894	0,0139293	0,0126878	0,294205	0,0143414	6,4845	1,33279	-0,468732	0,116144	0,6279808
CG15894-RB	CG15894	1,56579	2,13606	1,50325	4,43435	35,3965	31,2958	-0,471515	0,113608	0,6279808
CG15896-RA	mldr	2,11443	18,1625	2,31997	5,63993	2,07535	0,0691653	-0,380441	0,170128	0,6279808
CG15897-RA	wuho	10,2507	8,16325	7,81101	2,61606	4,26212	5,4095	-0,268575	0,306821	0,6279808
CG15899-RB	Ca-alpha1T	0,00690833	14,4145	0,0190063	1,32523	0,542506	0,495049	-0,49672	0,045102	0,6279808
CG15899-RC	Ca-alpha1T	0,913315	2,37659	4,14092	2,54176	0,135532	0,236168	-0,517571	0,038986	0,6279808
CG15902-RA	Ugt304A1	2,51421	11,7262	31,3305	3,34894	6,66257	4,56992	-0,923444	0,002957	0,6279808
CG15904-RA	Ir56d	0	412,519	0,0444027	0,76438	1,57493	0,394639	-0,041811	0,731452	0,6279808
CG15905-RA	CG15905	1,81278	1,70138	1,79327	24,6638	995,763	0,921157	-0,223335	0,509093	0,6279808
CG15908-RA	CG15908	30,5861	29,6466	35,5434	0,0991567	64,044	54,7107	-0,428519	0,122047	0,6279808
CG15908-RB	CG15908	0,0473435	0,0431238	0,0454526	10,1833	0,0715998	0,053962	-0,218343	0,388088	0,6279808
CG15909-RA	CG15909	0,592749	0,568333	0,868587	0,923153	55,8492	0,536253	0,010528	0,976149	0,6279808
CG15912-RA	REG	54,3535	46,4146	78,1821	68,8902	3,60432	0,0459609	-0,044548	0,881694	0,6279808
CG15914-RA	CG15912	2,49826	3,71971	0,213537	1,74042	4,6015	0,204443	0,190877	0,510463	0,13772387
CG15916-RA	CG15914	2,18505	1,69838	2,71313	2,27187	1,84835	0,0124437	-0,152124	0,571173	0,13772387
CG15917-RA	CG15916	10,5846	0,0432788	14,5931	11,5065	14,5071	2,45194	0,682383	0,031584	0,6279808
CG15918-RA	Gbp1	4,85636	0,0440711	0	0,0114464	12,695	63,1397	-0,617156	0,078658	0,6279808
CG15919-RA	Cda9	3,4529	0,0353276	4,19772	0,0104781	17,0817	16,9654	-0,168366	0,475588	0,6279808
CG1591-RA	CG15919	0,297639	0	0,0230539	0,749146	0,906527	130,656	-0,384415	0,139301	0,6279808
CG15920-RA	resilin	0,189389	0,172509	1,10565	0,0253184	0,0342939	0,025846	-0,669895	0,058274	0,6279808
CG15920-RB	resilin	0,024013	0,0218727	0,266677	69,2488	2,18642	0	-0,648518	0,067163	0,6279808
CG15922-RA	CG15922	35,0079	0,0372518	27,1714	31,8708	4,68911	0,0175236	-0,373575	0,215574	0,6279808
CG15923-RB	CG15923	2,57378	3,32591	0,0148854	0,0160222	0,0242759	0,0163561	0,785989	0,00136	0,13772387
CG15925-RA	Parp16	0	0	40,1512	0	0	0	-0,070526	0,562659	0,6279808
CG15927-RA	CG15927	10,491	6,81058	8,2913	3,45872	3,96318	3,53079	1,291417	1,07E-05	0,6279808
CG15929-RA	lin-52	17,7039	20,0622	0,0112244	0,0119748	0,833374	29,6419	-0,189302	0,414729	0,6279808
CG15930-RA	CG15930	0,35989	0,546355	0,875308	38,2497	6,7298	5,5445	0,106255	0,766173	0,6279808
CG1594-RA	hop	5,38412	6,26186	5,43384	8,04639	0,029407	117,005	0,137611	0,598176	0,6279808
CG1597-RB	GCS1	6,35182	64,3122	7,94917	92,5742	103,846	3,63447	-0,279577	0,201921	0,6279808
CG1598-RA	CG1598	0,0547136	77,3422	400,184	16,7871	532,45	0,0326287	0,01709	0,942908	0,6279808
CG1599-RA	Vamp7	20,3495	25,0189	0,0724438	0,0906948	0,122847	0,0925847	-0,110772	0,556865	0,6279808
CG1600-RA	Drat	0,0365127	17,4404	31,2228	38,0126	38,3401	0,0369684	-0,010108	0,969934	0,6279808
CG1600-RB	Drat	185,47	1,42402	0,0350544	2,05852	0,797582	0,366835	-0,006572	0,980434	0,6279808
CG1600-RC	Drat	0,0335637	0,0272255	184,575	0,0319627	0,0432936	37,2686	-0,001501	0,995516	0,6279808
CG1602-RA	CG1602	3,2373	0,0135048	3,93197	3,96799	1,86076	2,61018	0,455563	0,067987	0,6279808
CG1603-RA	CG1603	4,85303	25,6913	26,9341	3,73522	3,0089	0,0153484	-0,208021	0,322337	0,6279808
CG1605-RA	az2	3,10145	4,22997	3,31196	4,56546	3,18416	2,27767	0,040844	0,876	0,6279808
CG1607-RA	CG1607	0,0213976	0,0194904	3,52312	1083,18	1115,55	1,73637	0,032913	0,880807	0,6279808
CG1607-RB	CG1607	32,8372	32,623	25,1434	0,0413238	0,0559734	0	0,030693	0,888731	0,6279808
CG1609-RA	Gcn2	0,0115135	0,0104873	31,109	0,0119013	0,0161204	0,02726	0,275616	0,210507	0,6279808
CG1615-RA	Ork1	0,587546	1,76381	1,51613	0,017932	0,0299748	0,13814	-0,384111	0,106679	0,6279808
CG1615-RB	Ork1	0,921202	0,0654675	5,60197	2,43925	1,33874	0,742016	-0,378402	0,111239	0,6279808
CG1616-RA	dpa	5,4847	6,69336	9,33094	13,0827	5,53173	8,05735	-0,348554	0,175658	0,6279808
CG1618-RA	comt	11,5034	11,7421	13,749	14,6072	11,201	8,29901	0,001334	0,994964	0,6279808
CG1619-RB	Dsim\Hmr	1,6597	1,60868	3,02339	2,54682	0,914557	1,48163	0,207043	0,484896	0,6279808
CG1620-RA	CG1620	5,14504	6,73232	6,31861	6,43615	3,74075	4,2276	0,220462	0,308895	0,6279808
CG1621-RA	Coop	4,68054	7,09965	6,44897	6,26311	3,86942	4,27131	0,295563	0,223663	0,6279808
CG1622-RA	CG1622	12,739	1,96413	1,40478	7,47977	1,27343	0,959734	0,01132	0,959855	0,6279808
CG1623-RB	hebe	58,6213	55,5315	71,7792	11,8816	26,8331	64,0884	-0,335437	0,221222	0,6279808
CG1623-RD	hebe	0,0369001	0,0336112	21,9112	11,7745	31,07	21,8565	-0,335634	0,220056	0,6279808
CG1623-RE	hebe	0,0375289	0,0341839	0,0360299	37,5576	46,7858	0,0417399	-0,336074	0,220529	0,13772387
CG1623-RF	hebe	0,0346733	9,50644	0,0332885	10,5859	0,0508111	0,0382943	-0,335951	0,220516	0,6279808

gene_id	Symbol	W1_FPKM	W2_FPKM	W3_FPKM	MCM51_FPKM	MCM52_FPKM	MCM53_FPKM	FC	p-value	p-adj
CG1625-RA	Dvir\GJ20828	0,695807	0,86445	1,07467	0,941572	0,756359	0,879176	0,061269	0,84376	0,6279808
CG1625-RB	Dvir\GJ20828	0,018784	0,0584523	0,0616089	0,495089	0,101361	0,076392	0,070514	0,820677	0,6279808
CG1628-RB	CG1628	11,0535	7,23941	19,8774	3,22783	2,3218	1,60675	-0,567901	0,058162	0,6279808
CG1629-RA	yellow-h	50,3247	0,021368	24,7513	0,0247015	0,0355932	15,9218	0,017901	0,953188	0,6279808
CG1631-RA	CG1631	0	0	245,254	0,380846	0,830941	0,0589255	NA	NA	0,6279808
CG1632-RA	CG1632	9,1268	0	5,30905	0,0179908	9,96319	9,60426	0,148288	0,643452	0,6279808
CG1633-RA	Jafrac1	88,5898	7,09706	179,227	6,132	163,264	152,502	-0,285153	0,392182	0,6279808
CG1633-RB	Jafrac1	0,0688528	0,0217856	0,0661028	3,4214	0,110115	0,0829895	-0,290449	0,383491	0,6279808
CG1634-RA	Nrg	72,8331	73,689	69,8265	13,1272	15,4666	12,4893	0,07839	0,733525	0,6279808
CG1634-RB	Nrg	0,015087	0,0137422	0,0144844	0,0591211	13,0508	14,0722	0,047257	0,839654	0,6279808
CG1634-RC	Nrg	22,1908	23,4212	29,7547	9,78497	0	5,0613	0,076913	0,73878	0,6279808
CG1635-RA	CG1635	12,1281	11,5713	20,8114	0,0184854	0,70105	0,0217931	0,119846	0,635532	0,6279808
CG1636-RA	CG1636	5,18533	6,75096	2,0793	4,01084	3,50108	4,68671	-0,042903	0,854957	0,6279808
CG1637-RA	CG1637	20,8204	28,5906	0,0197274	31,4541	86,5888	21,5987	-0,444956	0,0907	0,6279808
CG1637-RB	CG1637	9,13334	0,0385152	0,0177066	19,3459	19,125	11,3272	-0,672736	0,007006	0,6279808
CG1637-RC	CG1637	0,041882	0,038149	0,0181473	15,3454	0,0631407	10,4777	-0,033847	0,879393	0,6279808
CG1638-RB	CG1638	0,610255	0,287515	0,606084	21,0819	12,6975	12,7134	-0,1659	0,642462	0,6279808
CG1639-RA	l(1)10Bb	38,6698	31,0735	40,4659	45,8938	0,0247814	331,5	-0,343609	0,163962	0,6279808
CG1640-RA	CG1640	0,0271396	0,0247206	0,0260556	0,02883	1,76411	0,0294308	-0,448387	0,119353	0,6279808
CG1640-RB	CG1640	29,2356	26,7083	53,5429	34,3637	1,86104	47,1151	-0,446634	0,121068	0,6279808
CG1640-RC	CG1640	0,0269294	0,0245292	0,0258539	0,0285924	1,36591	0,0291882	-0,447788	0,119973	0,6279808
CG1640-RD	CG1640	0,0264233	0,0240682	0,0253679	5,04866	125,04	0,0286048	-0,447642	0,120028	0,6279808
CG1640-RE	CG1640	0,0257038	0,0234128	0,0246772	0,0272109	0	0,0277779	-0,446732	0,121054	0,6279808
CG1640-RF	CG1640	0,0242066	0,022049	0,0232397	0,0255343	0	0,0260663	-0,448108	0,119531	0,6279808
CG1641-RA	sisA	0,602254	0,313471	2,1063	4,4117	0,0251444	0,0432982	-0,675431	0,059065	0,6279808
CG1643-RA	Atg5	13,902	13,8062	4,37078	7,08376	0	0,0779862	0,181406	0,360934	0,6279808
CG1644-RA	Cyp6t1	2,55016	51,684	1,88191	55,3078	0,397413	30,9294	1,346958	6,23E-05	0,6279808
CG1646-RA	CG1646	0,0430742	14,0871	7,95326	0,049486	5,50952	5,69935	-0,213329	0,339276	0,6279808
CG1646-RB	CG1646	7,6562	5,48364	6,66062	17,1963	3,74676	6,48136	-0,212606	0,341054	0,6279808
CG1646-RC	CG1646	4,49011	9,8646	9,56738	0,0213482	7,60889	4,52542	-0,213329	0,339276	0,6279808
CG1646-RD	CG1646	0,0277941	0,039235	7,6483	0,0195901	10,6367	7,80001	-0,207367	0,353594	0,6279808
CG1646-RE	CG1646	0,0185815	5,81738	0,0413538	0,0196485	0,0670291	0,0505172	-0,213329	0,339276	0,6279808
CG1646-RH	CG1646	0,0184158	4,24544	12,1886	0,0194628	10,0847	8,80621	-0,617916	0,010862	0,6279808
CG1647-RA	CG1647	2,98565	31,2198	0,0423853	0,0488984	0,0662333	1,71576	0,464654	0,043942	0,6279808
CG1648-RA	CG1648	24,9296	48,721	0,493254	41,3864	0,616433	2,24685	-0,122063	0,636005	0,6279808
CG1648-RB	CG1648	140,015	111,346	0,315749	156,468	0,238938	1,12471	-0,118886	0,643487	0,6279808
CG1650-RA	unpg	2,10045	2,93244	2,09194	3,52125	1,55241	2,55481	-0,075338	0,797908	0,13772387
CG1651-RA	Ank	0,0130233	0,0118625	0,0119564	183,279	331,756	276,268	0,082846	0,69809	0,6279808
CG1651-RB	Ank	0,0124538	0,0113438	0,0121978	0,0133803	0,0181237	0,0136591	0,082749	0,698436	0,6279808
CG1651-RC	Ank	0,0127053	0,0115728	52,4663	0,0127783	0,0173083	0,0130446	0,082798	0,698266	0,6279808
CG1651-RD	Ank	62,3656	57,8356	4,19913	0,0130439	0,0176681	0,0133157	0,082846	0,69809	0,6279808
CG1652-RA	Dsim\GD10684	0,424423	0,447634	7,45362	0,842397	4,38257	3,44685	-0,499495	0,13528	0,6279808
CG1655-RA	sofe	0,72206	0,899307	1,11764	5,4241	49,7208	49,544	-0,115531	0,717014	0,6279808
CG1656-RA	lectin-46Ca	0,269087	0,21787	5,08515	0,600746	3,03586	23,7886	-0,305861	0,390259	0,6279808
CG1657-RA	Gapvd1	6,19318	8,26048	6,22135	10,5264	0,0244957	0,0423176	-0,215108	0,353018	0,6279808
CG1659-RA	unc-119	11,6452	12,9119	13,1732	3,76862	10,4004	2,36846	0,005184	0,979315	0,6279808
CG1660-RA	Tim9a	51,0845	51,5792	72,3725	71,9507	88,5458	86,1072	-0,019786	0,938003	0,6279808
CG1662-RA	CG1662	6,96232	7,1375	13,8006	0,061724	9,89225	10,0605	-0,03209	0,914858	0,6279808
CG1663-RA	CG1663	2,63963	2,97313	3,46068	1,24242	1,4	52,1383	0,403958	0,149161	0,6279808
CG1664-RA	sbr	18,0134	21,4585	16,7722	23,4291	0	14,1516	-0,004768	0,982375	0,6279808
CG1665-RA	Marc	3,8359	0,0537538	77,0725	50,1125	55,2802	62,7558	0,194511	0,448951	0,6279808
CG1666-RA	Hlc	31,1148	0,42015	2,91516	0	0	0	0,030006	0,89884	0,6279808
CG1667-RA	Sting	7,46835	5,55361	0,0829598	5,16025	41,8299	0,665159	0,43981	0,082324	0,6279808
CG1668-RA	Obp19d	0	0	0	0,022431	2,50973	26,8481	-0,074864	0,550506	0,6279808
CG1668-RB	Obp19d	0	0	0	24,2708	0,0325789	48,7551	-0,074864	0,550506	0,6279808
CG1669-RA	kappaB-Ras	5,52241	5,97026	12,3246	5,7345	0,0377777	9,82629	-0,343012	0,24737	0,6279808
CG1669-RB	kappaB-Ras	3,82577	2,95523	0,0360095	4,87385	9,18066	0,041714	-0,343012	0,24737	0,6279808
CG16700-RA	Obp19b	0	0	0	238,271	0,0334207	20,7793	-0,513312	0,116298	0,6279808
CG16704-RA	CG16700	9,90323	13,8902	12,6378	7,45199	4236,77	0,195306	0,183397	0,607667	0,6279808
CG16705-RA	CG16704	3,7132	30,1861	105,239	287,531	603,825	518,146	-0,434826	0,178984	0,6279808
CG16707-RA	SPE	9,33795	0,206826	0,217996	0,393173	2,07421	0,401365	0,481668	0,062474	0,6279808
CG16707-RB	vsg	0,0297148	0,590707	37,9836	63,6417	33,5897	48,0283	0,482167	0,062261	0,6279808
CG16707-RC	vsg	0,029755	0,0549815	77,8885	80,7897	0,336197	0,416634	0,482167	0,062261	0,6279808
CG16707-RD	vsg	65,1278	182,799	12,5174	0	9,74637	7,90716	0,481668	0,062474	0,6279808
CG16708-RA	vsg	64,4656	0,0409432	0,0119003	0	0,0172248	0,0129817	-0,162907	0,506117	0,6279808
CG16708-RB	Cerk	9,51768	0,0492658	8,93861	6,06794	10490,9	1,82635	-0,109616	0,65602	0,6279808
CG1670-RA	Cerk	7,19403	8,53751	9,7064	77,062	0,130384	135,664	NA	NA	0,6279808
CG16710-RA	CG1671	8,10443	0,0145636	0,0151196	4920,84	0,0223209	0,0170917	-0,151654	0,654289	0,6279808
CG16711-RA	CG16710	0,909877	0,410506	0,49924	1,05354	0,0269353	0,488405	0,214689	0,416827	0,6279808
CG16711-RB	CG16711	4,09139	58,0391	0,0130914	0,724042	8,33328	2,07835	0,217264	0,411085	0,6279808
CG16712-RA	CG16711	0,0136361	0,0286784	25,1289	48,3952	0,687194	0,588462	-1,007439	0,003805	0,6279808
CG16713-RA	IM33	621,515	0	693,424	5,01393	8,10753	9,45923	-0,839685	0,015988	0,6279808
CG16716-RB	CG16713	140,003	0,693792	15,4479	1,5151	13,6732	1,15625	-0,078931	0,799805	0,6279808
CG16717-RA	TTLL6A	0,0588702	0,0464879	0	0,0575212	0,0679994	0,0512485	0,286804	0,294153	0,6279808
CG16718-RA	CG16717	3,86188	3,08869	0,157803	0,385754	2,51271	2,90095	-0,026577	0,915337	0,6279808
CG16718-RB	subdued	5,49546	0,0999776	0,00549966	7,45387	2,73551	0,144032	-0,001907	0,993867	0,6279808
CG16719-RA	subdued	0,0164586	2,82589	0,00579508	0,02393	0	3,94121	-0,908672	0,009548	0,6279808
CG1671-RA	CG16719	0,275827	22,6332	0,647314	119,572	4,41311	1,06523	-0,2184	0,417974	0,6279808
CG16720-RA	5-HT1A	3,32932	3,31763	3,18995	2,78732	5,80395	1,80455	0,316412	0,168132	0,6279808
CG16720-RB	5-HT1A	2,76328	3,10837	2,55409	2,84523	9,5759	1,39434	0,343766	0,125676	0,6279808
CG16721-RA	CG16721	63,5395	75,4911	626,342	62,7377	561,953	551,414	0,063351	0,776026	0,6279808
CG16723-RA	CG16723	0,396138	0,0213377	0,699782	0,0291231	2,12806	0,02973	0,042998	0,899656	0,6279808
CG16724-RA	tra	0,0613518	17,9885	13,7171	32,3337	18,6099	3,73086	0,457011	0,103153	0,6279808
CG16724-RB	tra	5,59428	0,0587949	15,6977	19,8746	3,3501	4,53578	0,444142	0,116663	0,6279808
CG16725-RA	Smn	13,5663	30,7609	41,6371	5,5544	0,0345194	0,0254644	-0,07428	0,800694	0,6279808
CG16727-RA	CG16727	0,751717	0,718951	2,09291	1,28676	0,865389	0,210483	0,207008	0,545347	0,13772387
CG16728-RA	Git	12,2172	12,0807	15,4144	17,8506	8,93937	11,4336	0,001009	0,99623	0,13772387
CG16732-RA	CG1673	10,8379	16,4373	12,8962	31,0269	15,912	29,7725	0,314056	0,286978	0,6279808
CG16733-RA	Ugt303B1	1,82132	1,71686	5,50124	1,1432	5,229	4,13004	0,119722	0,720452	0,6279808
CG16734-RA	St2	1,80703	1,39752	1,34206	62,6536	0	1,37689	0,255634	0,431583	0,13772387
CG16735-RA	CG16734	1,27269	28,3364	1,49615	0	0	0,159768	0,01562	0,897983	0,6279808
CG16736-RA	CR16735	0	0	0	19,9325	3,70319	0	0,491173	0,129718	0,6279808
CG16738-RA	CG16736	0,237683	31,9972	40,2617	0,417453	1,05339	34,1133	0,182265	0,483623	0,6279808
CG16739-RA	slp1	7,5457	2,46579	1,49859	0,0198917	1,30137	0,0203062	0,311308	0,345111	0,6279808
CG1673-RA	CG16739	0,144692	0,0342228	0	0,148356	2,6927	0	-0,540761	0,038841	0,6279808
CG16740-RA	CG1674	60,4909	58,1273	66,3804	0,0300941	5,28908	0,0307212	-0,029653	0,881565	0,6279808
CG16741-RA	CG1674	0,0470067	0,042817	0,0451293	0,0504329	0,0683117	0,0514838	-0,041811	0,731452	0,6279808
CG16742-RA	CG1674	130,626	135,168	128,093	78,9539	61,2484	57,796	0,326563	0,169111	0,6279808
CG16742-RB	CG1674	0,0282536	0,0257354	0,0271252	25,9435	37,4032	0,024148	0,588833	0,025191	0,6279808
CG16743-RA	Rh2	0,0725046	0,449153	0	0	7,92127	7,62738	-0,228589	0,501919	0,6279808
CG16747-RA	CG16741	0	0,10445	0,0360709	0,132398	0	0,491022	-0,248261	0,280034	0,6279808
CG16747-RB	FAM21	0,0147667	0,0309813	69,6721	0,057321	0,0452929	8,67715	-0,238199	0,301312	0,6279808
CG16747-RC	FAM21	0,0431398	11,1491	0,0299277	2,2818	0,0776416	0,0375043	-0,238922	0,299285	0,6279808
CG16749-RA	CG16743	26,7512	14,5727	61,0642	30,9217	55,6896	50,0166	-0,942837	0,007847	0,6279808

gene_id	Symbol	W1_FPKM	W2_FPKM	W3_FPKM	MCM51_FPKM	MCM52_FPKM	MCM53_FPKM	FC	p-value	p-adj
CG1674-RA	Oda	0,039933	0,0363737	0,0383381	0,0437667	0,0592823	0,0446787	0,130575	0,481376	0,6279808
CG1674-RB	Oda	0,0351343	0,0320027	0,033731	0,0380542	0,0515447	0,0388472	0,348603	0,058213	0,6279808
CG1674-RC	Oda	512,905	548,555	497,634	584,649	609,524	588,587	0,379065	0,040224	0,6279808
CG1674-RD	CG16749	11,3731	8,29146	0	0	11,1052	6,99534	0,092836	0,624853	0,6279808
CG16752-RA	Ntmt	4,81026	4,17773	8,52029	6,1546	9,05102	10,3022	0,774737	0,004048	0,6279808
CG16753-RA	SPR	5,67125	0	4,71945	0,651065	38,2755	0,513344	-0,753585	0,013608	0,6279808
CG16755-RA	CG16753	10,8496	8,22198	33,7248	31,3125	1,36276	22,0157	-0,256154	0,468649	0,6279808
CG16756-RA	Or85f	0,42033	0,280768	0,403542	0,639486	0,685475	0,32623	-0,448384	0,200325	0,6279808
CG16757-RA	CG16756	4,71061	2,95714	5,86699	2,53465	0	0,0191554	0,03258	0,885313	0,6279808
CG16758-RB	Spn	7,09642	7,82585	7,58184	10,3795	5,6315	4,79637	-0,420722	0,07577	0,6279808
CG16758-RD	CG16758	0,0320187	0,0291648	0,0307398	1,08535	0,0466178	0,035134	-0,415461	0,079603	0,6279808
CG16758-RE	CG16758	0,035531	0,0323641	0,0341119	1,51993	0,0521775	0,0393241	-0,324509	0,159829	0,6279808
CG16758-RF	CG16758	27,1336	48,4897	55,0432	5,6539	53,6025	61,3514	-0,319433	0,167141	0,6279808
CG1675-RA	CG16758	0,0306949	0,027959	0,0294689	2,20666	13,1035	0,0335733	-0,417789	0,186662	0,6279808
CG16761-RA	cactin	5,24793	6,53797	24,3976	1,89697	13,3781	1,22067	0,473064	0,14117	0,6279808
CG16762-RA	Cyp4d20	2,12017	1,17088	2,75673	0,0162114	1,52611	1,15017	0,538616	0,090139	0,6279808
CG16765-RB	CG16762	0,345495	0,118013	1,53409	0,0168887	0,237993	0,179366	0,187324	0,439675	0,6279808
CG16765-RC	ps	49,0351	3,61156	52,2315	49,6482	17,3741	0,0177361	0,287848	0,205745	0,6279808
CG16765-RD	ps	26,4126	2,23321	27,1156	0,0190465	11,7357	2,49612	0,290426	0,202148	0,6279808
CG16765-RF	ps	36,5522	0,356133	42,402	51,3595	22,3288	21,684	0,489868	0,026049	0,13772387
CG16765-RG	ps	0,0203519	53,2709	0,019539	0,0212724	0,0288136	27,9199	0,471909	0,032252	0,6279808
CG16765-RH	ps	0,0349115	28,0279	0,0335171	0,0377923	0,0511899	20,0391	0,491944	0,025344	0,6279808
CG16765-RI	ps	0,0263811	43,6071	0,0253274	0,0279733	0,03789	4,47066	0,389535	0,082889	0,6279808
CG16765-RJ	ps	0,0257539	0,0185379	0,0247252	0,0272672	0,0369335	5,13336	0,297307	0,192047	0,6279808
CG16765-RK	ps	0,025108	0,0317998	0,0241052	0,0265422	0,0359516	3,08643	0,474153	0,031587	0,6279808
CG16766-RA	ps	83,5887	0,0240297	70,0804	87,0448	44,7147	35,2136	-0,556214	0,113686	0,6279808
CG1676-RA	TyrRII	0,225131	1,52972	22,4455	1,01941	27,3065	28,2169	-0,08286	0,758681	0,6279808
CG16771-RA	Dsim\GD16158	19,4653	19,5058	0,0208491	0,0171749	4,61443	11,9564	-0,232603	0,304832	0,13772387
CG16772-RA	Alp12	8,19574	21,3917	1,66283	37,4909	0,0443257	0,0334065	0,263676	0,454934	0,13772387
CG16775-RB	CG16772	0,723459	0,358253	0,236	1,06704	0,614377	1,60905	-1,181197	0,000837	0,6279808
CG16777-RA	CG16775	0,706946	0,386361	2,80534	1,67889	0,0458986	0,0345919	0,355705	0,164525	0,6279808
CG16778-RA	ps	10,7941	0,0234584	15,6914	11,0092	9,9417	0,0606797	0,335363	0,20314	0,6279808
CG16778-RB	lov	0,0150801	0,0129946	8,59492	2,59638	0,0197086	0,0318863	0,333908	0,204931	0,6279808
CG16778-RC	lov	0,0142661	10,2564	5,54511	0,0146997	0,601615	0,0158924	0,33488	0,203631	0,6279808
CG16778-RD	lov	10,0751	0,0128668	28,7595	9,23642	0,0144617	0,015006	0,332192	0,206897	0,6279808
CG16779-RA	lov	0,0141258	0,212423	0,00984941	0,0145504	20,5028	4,98579	0,147784	0,614796	0,6279808
CG1677-RA	CG16779	1,26748	1,59505	1,15281	1,78292	11,8803	0,626388	-0,140703	0,543293	0,6279808
CG16781-RA	whe	0,270803	0	0	6,24273	1,48038	5,52974	0,162674	0,632453	0,6279808
CG16782-RA	CG16781	0,174113	0,178418	0,313423	19,9741	0,173978	0,216619	-0,284286	0,330306	0,6279808
CG16783-RA	CG16782	1,98891	0,161502	0,113483	0,2004	0,219276	0,110222	-0,221349	0,428045	0,6279808
CG16784-RB	fzr2	0,0428878	0,0390652	0,0411748	23,3522	0,064139	0,048339	0,25515	0,329755	0,6279808
CG16784-RC	pr	34,1744	21,564	32,8467	0,0936599	27,9885	25,2606	0,236811	0,365075	0,6279808
CG16785-RA	pr	0,0633719	0,0577235	11,6206	19,114	0,0998865	0,0752805	0,306422	0,129873	0,6279808
CG16785-RB	fz3	10,1694	9,20831	11,5056	0,174538	4,11433	7,9427	0,339489	0,09101	0,6279808
CG16786-RA	fz3	0,03008	0,027399	0,0288786	0,175001	0,0634549	0,0478235	0,517771	0,052469	0,6279808
CG16786-RB	CG16786	39,9546	42,0023	38,6646	1,73812	13,0223	20,5654	0,530326	0,046714	0,6279808
CG16787-RA	CG16786	6,57221	8,3041	5,59191	9,25403	4,56999	2,82883	-0,46856	0,151548	0,6279808
CG16788-RA	CG16787	14,6702	1,78438	6,56163	13,1031	30,2698	5,73943	-0,100523	0,70457	0,6279808
CG16789-RA	RnpS1	20,2451	18,2196	5,60069	46,5185	16,3334	29,8974	-0,377797	0,167081	0,6279808
CG1678-RA	CG16789	0,91366	2,13015	6,97785	8,26879	0,113475	45,9544	0,01562	0,897983	0,6279808
CG16790-RA	CG16790	2,63623	1,14292	3,87805	16,8352	20,8919	9,351	0,119856	0,710097	0,6279808
CG16791-RA	CG16791	14,7505	17,8697	0,017826	0,0193258	9,59826	0,0197285	-0,066028	0,812455	0,6279808
CG16792-RA	SmF	38,4413	17,8636	62,2511	79,6316	702,553	76,7467	-0,091502	0,733203	0,6279808
CG16793-RA	brv2	0,0698181	2,61204	0,035171	0,0286296	0	0,0751202	0,122419	0,590927	0,6279808
CG16798-RA	CG16798	10,7712	11,6463	10,2525	15,0036	3,31323	7,15518	0,275208	0,375208	0,6279808
CG16799-RA	Dpse\GA24823	6,44901	5,9705	13,7343	4,3345	4,90434	6,02835	-0,072533	0,795307	0,6279808
CG16800-RB	CG16800	0,19457	0	0,112079	0	0	0,251383	0,04326	0,815378	0,6279808
CG16801-RC	Hr51	2,24786	124,234	19,9298	29,9048	20,2121	20,4324	0,310208	0,257559	0,6279808
CG16807-RA	roq	16,7818	0,0145039	16,1498	0,0164708	217,363	167,799	0,078766	0,757666	0,6279808
CG16812-RA	GstT4	143,802	226,162	152,828	0	0,171302	0	-0,129079	0,598075	0,6279808
CG16813-RA	CG16812	9,99435	9,03551	15,8773	0,0125831	0	0	-0,00944	0,945067	0,6279808
CG16815-RB	CG16813	0	0	0,176377	0,0125759	0	0	0,052121	0,801675	0,6279808
CG16817-RA	CG16815	0,183714	4,15715	0,285751	0,0125831	1,9647	1,54201	-0,435657	0,099786	0,6279808
CG1681-RA	p23	161,174	333,959	188,44	568,8	227,873	250,642	0,023913	0,927233	0,6279808
CG16820-RA	CG16820	22,5344	42,725	0,273434	12,6742	0,0170439	0,0342398	0,61093	0,001704	0,6279808
CG16824-RA	CG16824	0,847127	1,69757	89,8042	0	4,4276	101,311	0,341279	0,130358	0,6279808
CG16825-RA	CG16825	0,0541606	0,511017	0,908205	5,08784	0,0170439	0,842747	-0,350026	0,231807	0,6279808
CG16826-RA	CG16826	0	0	0	0	14,3481	0	NA	NA	0,6279808
CG16827-RA	ItgaPS4	0,0197359	0,00865536	0,0189477	0,0209478	0,0131164	0,0213843	0,010234	0,970269	0,6279808
CG16833-RD	Ant2	0,0472308	2,01151	79,7637	0,0550694	3,11524	13,0252	0,001512	0,99416	0,13772387
CG16833-RE	Ant2	0,0493472	0,0292641	6,99041	0,0579767	220,866	203,757	0,022363	0,913609	0,6279808
CG16833-RF	TTLL4A	0,0165784	0,0151007	0,0159162	0,0171749	0,0232634	0,0175327	0,022363	0,913609	0,6279808
CG16833-RG	TTLL4A	0,0165617	0,0150856	0,0159003	0,017157	0,0232392	0,0175145	0,00134	0,994814	0,6279808
CG16833-RH	TTLL4A	8,40435	8,41423	11,1302	7,75408	4,84968	5,40237	0,001512	0,99416	0,6279808
CG16834-RB	TTLL4A	2,49659	0,0187166	0,0197274	2,38174	1,67591	2,91098	-0,027275	0,934575	0,6279808
CG16836-RA	TTLL4A	0,0168146	0,0153159	0,016143	2,47481	1,62127	0,0177924	0,175537	0,623221	0,6279808
CG16837-RA	lectin-33A	0	15,6536	97,0569	0,517508	0,524023	0,890741	NA	NA	0,6279808
CG16838-RC	CG16836	2,21731	1,90173	5,21544	0,0229706	41,66	29,9831	0,136632	0,597043	0,6279808
CG16838-RD	CG16837	0	0,0772941	0	0,101422	0	1,29547	0,154798	0,55061	0,6279808
CG1683-RA	elg1	2,33279	1,20133	0,379861	0	1,36125	1,29523	0,46205	0,082865	0,6279808
CG1683-RB	elg1	0,717061	2,5112	0	0,360037	1,41597	0,719802	-0,326694	0,276378	0,6279808
CG16840-RA	Art8	10,1772	0	18,7456	9,37286	2,9539	1,38269	-0,015221	0,962098	0,6279808
CG16844-RA	IM3	104,012	1,71673	22,7976	4,8363	46,7515	14,5146	0,997761	0,004683	0,6279808
CG16848-RA	CG16848	0	0,718044	2,48238	1,06378	2,28259	0,0384036	-0,064379	0,736278	0,6279808
CG16849-RA	CG42809	0	0,23238	7,10401	6,43012	6,47384	4,7537	-0,166395	0,305573	0,6279808
CG16850-RA	peng	14,576	9,49832	28,276	22,5333	22,2995	22,245	-1,070327	0,001933	0,6279808
CG16852-RA	CG42784	0,289807	8,93406	0,885282	2,62218	1,78818	1,04396	-0,070526	0,562659	0,6279808
CG16853-RA	CG16852	0	0,0127415	3,09289	0,0143915	0,174481	0,542454	-0,364695	0,283792	0,6279808
CG16854-RA	CG16853	0,239856	0,33933	1836,76	1897,87	9,59944	12,5045	0,036211	0,893839	0,6279808
CG16857-RA	CG16854	1,63034	1,79185	11,4008	9,13427	0,0213952	8,89446	-0,438433	0,079964	0,6279808
CG16858-RA	bdl	5,55494	8,07137	7,37529	8,79035	9,72425	8,05907	-0,036184	0,850547	0,6279808
CG1685-RA	vkg	138,996	169,804	154,167	189,64	121,018	118,794	-0,330231	0,258466	0,6279808
CG16863-RA	CG16863	0,711741	0,0809319	0,0853025	0,110832	1225,59	34,3069	0,350552	0,245786	0,6279808
CG16863-RB	CG16863	0,188039	0,107925	0,113754	0,161293	4,29013	0,113141	0,321513	0,292086	0,13772387
CG16865-RA	CG16865	7,11309	10,3751	9,99361	9,5805	0,0163178	0,164654	-0,195954	0,477679	0,13772387
CG16868-RA	Dsim\GD25286	3,8936	3,02178	4,75868	29,5208	2,8907	2,51603	0,085681	0,710313	0,6279808
CG16869-RA	Ance-2	0,989699	1,14735	0,0150229	0,754576	0,824067	1,83998	0,671572	0,031282	0,6279808
CG16870-RA	Acyp	0	0	19,2068	0	0	13,2714	NA	NA	0,13772387
CG16873-RA	NimB5	12,933	13,0922	6,42142	8,4192	13,176	9,36321	-0,553745	0,054153	0,6279808
CG16874-RA	Vm32E	0,378009	12,1206	5,61779	5,56878	0,260616	2,67959	0,255777	0,203296	0,13772387
CG16876-RA	NimC4	30,8698	24,3332	29,3643	59,7895	25,3096	44,122	-0,509054	0,047998	0,6279808
CG16879-RA	CG46301	2,10017	2,44347	1,81296	3,87657	0,0701369	3,03438	-0,851781	0,001607	0,6279808
CG16880-RB	CG1688	2,12318	2,45405	2,30862	2,35598	0,0902871	1,44587	-0,03779	0,907599	0,6279808
CG16884-RA	NimC3	6,59983	1,11456	0,0122425	0,0130932	24,7903	0,336992	-0,099643	0,72016	0,6279808

gene_id	Symbol	W1_FPKM	W2_FPKM	W3_FPKM	MCM51_FPKM	MCM52_FPKM	MCM53_FPKM	FC	p-value	p-adj
CG16885-RA	Vajk3	236,44	207,795	95,1033	259,085	15,3674	12,8968	-0,057449	0,836488	0,6279808
CG16885-RB	Vajk2	264,704	237,077	0,0590659	266,385	0	0,0350984	-0,121072	0,673748	0,6279808
CG16886-RA	Vajk2	0,112629	0,10259	0,720151	0,150602	0,258687	0,194962	0,49341	0,063119	0,6279808
CG16888-RA	Vajk1	109,167	112,927	31,028	108,383	7,06624	11,7691	-0,242489	0,479773	0,6279808
CG16889-RA	CG16888	1,42565	18,4244	1,60675	0,0235452	8,27166	1,11158	-0,662299	0,05066	0,6279808
CG1688-RA	Adat1	0,0224163	1,46796	3,83043	0,0120471	0,595302	64,4605	0,206059	0,357436	0,6279808
CG16890-RA	lz	0,119049	0,019716	0,0429594	0,50643	31,554	0,0314098	-0,134547	0,608126	0,13772387
CG16890-RB	lz	0,483623	0,651966	0,0432818	7,29823	64,9216	5,68274	-0,291462	0,321397	0,6279808
CG16892-RA	CG16890	0,0888512	0,264704	0,0328234	0,0369447	0,044636	4,91972	-0,164459	0,482782	0,6279808
CG16894-RA	CG16890	0,118486	24,273	35,8836	30,5593	0,102705	0,0122981	0,136973	0,455979	0,6279808
CG16896-RA	Aladin	4,83584	4,2909	9,206	8,66266	0,295263	4,35579	0,283531	0,299911	0,6279808
CG16898-RA	CG16894	0,195106	0,148097	0	0,121494	6,50711	6,17398	0,138206	0,665225	0,13772387
CG16899-RB	CG16896	4,03486	123,93	5,58044	172,794	205,072	2,63196	-0,591099	0,060183	0,6279808
CG1689-RA	CG16898	4,28737	5,19166	14,358	6,10353	3,34101	5,59422	0,157508	0,642564	0,6279808
CG1689-RB	FoxP	0,825433	1,98615	1,01039	1,8115	1,46045	2,4186	0,125061	0,712601	0,6279808
CG16901-RA	CG1690	0	0	0	0,181842	0,0418522	0	-0,104308	0,650717	0,13772387
CG16901-RB	sqd	30,2295	33,4544	50,8805	45,0215	20,2214	23,7896	0,150152	0,516055	0,6279808
CG16901-RC	sqd	19,9509	17,9604	19,3078	23,1716	7,34984	14,3744	0,150737	0,515364	0,6279808
CG16901-RD	sqd	0,0344025	0,0313362	0,0330285	0,037195	0,0503808	0,03797	-0,02982	0,902802	0,6279808
CG16902-RC	sqd	26,6649	24,728	38,7963	46,8595	27,0494	25,3795	0,067071	0,849777	0,6279808
CG16903-RA	Hr4	19,3893	29,2956	68,9236	90,684	36,3675	55,3816	-0,333573	0,105602	0,6279808
CG16904-RA	CG16903	22,5427	22,9207	28,8508	32,1808	27,9225	30,1887	-0,460232	0,196382	0,6279808
CG16905-RA	CG16904	0,634703	2,40479	2,53466	25,694	2,63906	13,3675	-0,411992	0,225687	0,6279808
CG16908-RA	eloF	1,87474	0,741054	2,33901	0	4,15253	3,17521	-0,157318	0,575035	0,6279808
CG1690-RA	CG16908	0,825185	0,694037	0,948703	15,8888	102,328	11,2456	-0,095685	0,563966	0,6279808
CG16910-RA	Imp	0,0237199	0,0216058	0,0165602	0,0249919	43,7179	0,0217437	-0,072168	0,720659	0,13772387
CG16912-RA	Imp	0,020377	0,0185608	55,4052	0,0212999	4,86816	0,0219352	-0,031074	0,894437	0,6279808
CG16914-RA	Imp	0,0205481	0,0187166	0,0226344	0,0214874	0,196362	7,72797	-0,400049	0,214776	0,13772387
CG16916-RA	Imp	10,6141	0,0167993	96,527	13,4927	11,1972	0,0200999	0,064883	0,777357	0,6279808
CG1691-RA	Imp	0,0189022	0,0172174	28,4121	0,0196896	4,24335	0,0180988	0,39674	0,078968	0,6279808
CG1691-RB	Imp	0,017093	0,0155695	12,9093	0,0177294	0,145236	0,0182709	0,296432	0,199483	0,6279808
CG1691-RC	Imp	0,0172492	0,0157117	0,0402092	0,0178979	8,02115	22,5968	0,396439	0,079209	0,6279808
CG1691-RD	Imp	43,4986	56,2272	34,4637	40,6995	1,30495	0,0253492	0,499994	0,027824	0,6279808
CG1691-RE	Imp	0,0235761	0,0214747	48,6866	0,0248318	84,9945	41,7021	0,498546	0,027976	0,6279808
CG1691-RF	key	22,684	2,24996	24,7029	7,95036	0	26,7717	0,525771	0,01995	0,6279808
CG1691-RG	TyrRS-m	8,77801	9,16966	12,5238	27,0607	0,0214776	9,81916	0,50221	0,027116	0,6279808
CG1691-RH	Lcp9	0,355247	0,539305	0,341058	0,131122	4,2982	3,00776	0,502372	0,02708	0,6279808
CG1691-RI	Rpt3	33,0157	40,2254	0,0488727	51,2166	0,0270824	0,0204109	0,392897	0,083591	0,6279808
CG16922-RA	mal	3,58951	3,91785	4,71369	4,62491	0,0321217	3,7183	-0,274333	0,411015	0,6279808
CG16926-RA	Myo10A	2,52797	2,03605	3,12573	1,072	0,565206	1,82871	-0,538352	0,048107	0,6279808
CG16928-RA	CG16926	40,4009	0,0421277	106,396	0,203304	0,275377	0,986022	2,586884	3,57E-30	0,6279808
CG1692-RA	mre11	10,8539	0,0485505	0,0146584	4,95635	0,0213594	3,64836	-0,051707	0,803572	0,6279808
CG16931-RA	tty	6,3636	7,16904	14,6692	11,5433	0,032168	5,19685	-0,013096	0,914398	0,6279808
CG16932-RA	tty	0,0226003	0,0205859	0,0174101	0,0237488	0,0828297	0,0242437	-0,115351	0,605033	0,13772387
CG16932-RB	Eig71Ea	0	0,556346	12,5517	41,5854	0	17,0557	-0,115489	0,6044	0,6279808
CG16932-RC	Eps-15	5,33431	6,83612	27,3753	6,37461	25,8024	0,033078	-0,115921	0,599896	0,6279808
CG16935-RA	Eps-15	3,58418	3,46047	41,4027	0	17,6698	0,0343446	0,012286	0,970061	0,6279808
CG16936-RA	Eps-15	5,33276	6,10454	0	0	9,21206	3,44574	-0,371954	0,184853	0,6279808
CG1693-RA	CG16935	11,9219	8,10718	30,9486	11,4582	18,2684	6,26446	-0,27933	0,254764	0,6279808
CG1693-RB	GstE12	135,933	132,136	238,123	4,67466	235,646	23,9564	-0,41766	0,099331	0,6279808
CG16940-RA	Dis3l2	8,76113	0,108444	41,3435	0,186916	3,3671	4,03588	0,239719	0,35499	0,6279808
CG16940-RB	Dis3l2	0,0186129	0,156871	0,068285	0,280963	0,0266617	0,0200939	0,241458	0,325527	0,6279808
CG16940-RC	Dis3l2	3,17371	19,6661	0,0260663	21,0123	2,90643	2,32967	0,239719	0,35499	0,6279808
CG16941-RA	Sf3a1	7,56945	0,0402136	37,438	23,9115	5,60871	23,7418	-0,050328	0,831949	0,6279808
CG16944-RA	sesB	586,473	1,36529	206,576	586,123	62,2032	3,36233	-0,468533	0,134314	0,6279808
CG16944-RB	sesB	0,0388072	3,15305	0,0278884	0,0424144	46,0658	74,533	-0,468529	0,134316	0,6279808
CG16944-RC	sesB	135,105	1,04956	21,6925	214,12	0,0257465	0,0194041	-0,468569	0,134262	0,6279808
CG16944-RD	sesB	0,0380037	0,0338951	0,0454093	0,0414538	0,0264327	0,0199213	-0,468576	0,134255	0,6279808
CG16947-RA	Rchy1	15,0567	5,31186	9,34663	21,9059	11,0943	5,69064	0,129243	0,660263	0,6279808
CG16952-RA	CG1695	1,40407	1,07292	1,65558	1,91388	0,0311464	1,37292	0,046763	0,821894	0,6279808
CG16952-RB	CG16952	14,2752	13,9173	6,89084	14,0325	7,70157	5,38511	0,052788	0,799884	0,6279808
CG16952-RC	CG16952	0,0173853	0,0158358	13,4684	3,46514	1,92605	13,177	0,040723	0,845031	0,6279808
CG16953-RA	CG16952	2,72509	2,65573	3,10452	4,58269	1,76971	10,0548	0,228976	0,315821	0,6279808
CG16954-RA	CG16953	18,752	21,9805	17,5767	0,0296473	0,204459	0,0279859	0,029507	0,886693	0,6279808
CG16954-RB	Hsp60D	0	25,1489	0	0,380846	0	0,0766643	0,029507	0,886693	0,6279808
CG16956-RA	Hsp60D	0	0	26,6728	0	0,0509561	0	0,048251	0,77366	0,6279808
CG16957-RA	CG16956	0	0,0752331	0	0	0	2,83342	NA	NA	0,6279808
CG16959-RA	CG16957	0	0	0	0	0	0,133924	0,105409	0,706915	0,6279808
CG16959-RB	CG16959	11,6072	21,2778	10,6469	14,6662	10,8758	11,8776	0,10547	0,707035	0,6279808
CG1695-RB	CG16959	0,0294499	0,026825	0,0282737	0,0314591	0,0426115	0,0321146	-0,460917	0,079425	0,6279808
CG16960-RA	Dd	13,3912	67,1284	0,0543743	0,0647571	0,0565407	0,960957	0,05799	0,71375	0,6279808
CG16961-RA	Or33a	0	0	0	0,169817	0	0	-0,070526	0,562659	0,6279808
CG16963-RA	Or33b	0	0	0,0836897	0,105763	0,654814	0,22824	0,580588	0,093156	0,6279808
CG16964-RA	Crys	0,618495	0,203438	4,12356	0,246158	0	1,88191	-0,041811	0,731452	0,6279808
CG16965-RA	CG16964	0	0	0	0,287231	0,226395	0,0100552	-1,310159	0,000223	0,6279808
CG16969-RA	CG16965	0,446243	0,246785	0,979246	4,70701	0,920482	0,00974495	-0,418314	0,069769	0,6279808
CG1696-RA	dgt2	9,21989	4,73946	2,45199	0,0220483	9,34283	1,4853	0,301322	0,146248	0,6279808
CG16970-RA	rho-4	5,00187	6,25465	2,29438	4,03907	6,58406	2,34504	-0,643962	0,048461	0,6279808
CG16971-RB	rho-4	0,0286208	0,0260698	11,4883	5,81932	17,179	5,27863	-0,847093	0,001322	0,6279808
CG16971-RC	rho-4	10,1342	9,00541	0,245879	12,2737	0,1202	5,13659	-0,856063	0,00107	0,6279808
CG16971-RD	CG43778	0,792165	0,650818	0,909648	1,43271	0,0170439	2,21063	-0,849412	0,001254	0,6279808
CG16972-RA	CG33229	66,991	57,2918	0,0697108	140,624	161,777	98,5696	0,140829	0,59906	0,6279808
CG16973-RA	CG33229	0,14009	0,127603	0,0992751	0,204302	0,276729	0,208559	0,027921	0,920599	0,6279808
CG16973-RB	CG33229	0,12428	41,6587	0,114301	119,956	129,681	86,6423	0,041793	0,881297	0,6279808
CG16973-RC	CG16972	2,25078	2,11747	0,0255828	0	63,8588	11,0079	0,04638	0,867901	0,6279808
CG16973-RD	msn	3,27878	4,7324	5,42647	0,0146572	4,04175	0,0172406	0,046719	0,866915	0,6279808
CG16973-RE	msn	0,0156813	0,0142836	0,015055	0,0156419	0,0219585	0,0165492	0,049928	0,857671	0,6279808
CG16974-RA	msn	6,59093	10,2144	6,2677	25,4303	4,4785	6,85766	-0,10834	0,648455	0,6279808
CG16975-RA	msn	7,9662	11,4063	7,15732	0,0156233	3,06178	5,69929	0,01498	0,94648	0,6279808
CG16975-RB	msn	6,89725	10,1406	7,38021	0,0344168	7,00974	5,097	-0,023831	0,916754	0,6279808
CG16975-RC	CG16974	27,1535	0,024272	50,7971	0,921595	22,7068	0,0128453	-0,024829	0,913289	0,6279808
CG16976-RB	Sfmbt	6,33635	27,5693	28,912	19,7449	10,5795	13,4789	-0,346794	0,250457	0,6279808
CG16978-RA	Sfmbt	1,46019	33,2003	13,328	0,0784873	26,5891	0,0128453	-1,438092	4,93E-05	0,6279808
CG16979-RA	Sfmbt	0,0139764	262,182	2,25365	1,03713	0,0454313	0,012838	0,251049	0,230288	0,6279808
CG1697-RA	Fife	1,08846	0,570146	47,8565	1,98971	0,0178697	5,72676	0,140815	0,535289	0,6279808
CG1697-RB	CG16978	0,286788	0,0111753	11,0102	7,3973	0	13,8282	0,140383	0,536433	0,6279808
CG1697-RC	CG16979	14,1591	6,1349	6,25561	7,9248	36,1654	7,42328	0,189739	0,395302	0,6279808
CG16982-RA	CG1698	7,6361	10,422	8,39188	8,36318	21,2298	4,73712	0,070976	0,77026	0,6279808
CG16982-RB	Roc1a	0,0551244	0,0502111	0,0529227	0,0627826	0,0850395	0,0640909	0,070921	0,77046	0,13772387
CG16983-RA	Roc1a	68,5852	65,0099	109,553	82,6861	60,5272	86,1495	-0,081426	0,636699	0,13772387
CG16983-RB	SkpA	0,0611435	0,0556937	4,59945	0,0707356	0,0103341	4,31914	-0,081745	0,635672	0,6279808
CG16983-RC	SkpA	0,0450824	0,0410642	0,00716746	0,0500499	0,0102569	0,00773022	-0,085013	0,622926	0,6279808
CG16983-RD	SkpA	71,4683	81,2143	0,00712559	271,981	0,010196	0,0076843	-0,088691	0,607724	0,6279808
CG16983-RE	SkpA	47,7642	56,7734	5,23744	126,628	5,43505	2,45615	-0,079313	0,645359	0,13772387

gene_id	Symbol	W1_FPKM	W2_FPKM	W3_FPKM	MCM51_FPKM	MCM52_FPKM	MCM53_FPKM	FC	p-value	p-adj
CG16983-RF	SkpA	35,7078	0,0613104	22,4547	0,0791472	9,66786	24,1552	-0,079097	0,64622	0,13772387
CG16983-RG	SkpA	0,0452988	0,0412612	0,0587014	0,0503174	0,0958118	0,0722096	-0,079088	0,646472	0,6279808
CG16983-RH	SkpA	0,076862	0,0700113	0,0432818	0,0927372	0,0677928	0,0510928	-0,084534	0,624637	0,13772387
CG16984-RA	SkpA	120,444	142,606	112,534	0,101603	146,061	71,3815	-0,088563	0,745058	0,6279808
CG16985-RA	CG16984	2,79933	2,4967	3,66735	3,68127	2,65044	3,96119	-0,12842	0,678622	0,6279808
CG16986-RA	CG16985	7,48759	5,45617	14,2173	10,2087	11,7228	12,0701	-0,224844	0,446048	0,6279808
CG16987-RA	CG16986	17,5896	0,0130142	25,6244	0,0147227	0,019942	36,5022	0,108578	0,689803	0,6279808
CG16987-RB	daw	6,46108	5,19	7,25639	5,42016	0,0462439	0,0389371	0,126513	0,641318	0,6279808
CG16988-RA	daw	1,86276	0,774346	0	3,65447	3,82427	2,76832	0,221323	0,389578	0,6279808
CG16989-RA	Roc1b	8,60608	0,255895	0	0,115292	0,0165324	0	-0,106274	0,63249	0,6279808
CG1698-RA	CG16989	8,84724	12,1162	9,53334	0,164715	9,10246	8,28958	0,366764	0,107238	0,6279808
CG16991-RA	Tsp66A	0	0	0	0	0	6,75062	NA	NA	0,6279808
CG16991-RC	Tsp66A	0	3,59811	0	0,358491	4,01014	167,222	NA	NA	0,6279808
CG16992-RA	mthl6	0	0	0	3,13729	103,217	0	-0,013096	0,914398	0,6279808
CG16993-RA	in	1,04626	1,30669	1,49117	0,0417691	0,0565766	0,0426395	-0,570842	0,074024	0,6279808
CG16995-RA	CG16995	0,37458	0,067956	0,431543	65,2733	0	0	0,240792	0,286947	0,6279808
CG16996-RA	Phae1	2,41741	0,846902	33,991	2,20552	19,1476	98,327	-0,61675	0,082753	0,6279808
CG16997-RA	Phae2	2,53211	1,61083	6,04018	4,53846	6,54285	26,3755	-1,327919	0,000164	0,6279808
CG16998-RA	CG16998	1,4882	10,0469	1,30629	2,41626	0	0	-0,111769	0,742401	0,6279808
CG17002-RB	CG17002	5,20117	0,0346763	0,0228464	0,0415322	0,0273145	13,0304	-0,133954	0,537792	0,6279808
CG17003-RA	CG17003	0	0,0798936	0,0842081	0	1,47191	0	0,044335	0,715924	0,6279808
CG17005-RB	CG17005	0,0859468	0,0407307	70,9319	0,0495978	0,0671805	0,0506313	-1,322709	0,000169	0,6279808
CG17009-RA	aust	0	0,277357	0,292335	0,354959	3,60175	8,8324	-0,044602	0,859921	0,6279808
CG17010-RA	CG1701	0	9,52093	0	0	58,4869	0	0,244383	0,406722	0,6279808
CG17010-RB	CG17010	0,182951	0,166644	7,24653	4,45172	9,52977	6,85026	0,169038	0,531726	0,6279808
CG17011-RA	CG17010	0,0727974	0,062629	6,35436	6,14726	16,5033	8,26077	0,087982	0,76297	0,6279808
CG17012-RA	lectin-30A	0,147491	0,26869	0,3776	0,210507	8,62691	0,346149	NA	NA	0,6279808
CG17018-RA	Send1	0	0	0	1,4174	2,79987	0	0,267846	0,349435	0,6279808
CG17018-RC	Marf1	0,893407	0,912845	1,13369	0,0157315	0,0213084	0,870276	0,267846	0,349435	0,6279808
CG17018-RD	Marf1	0,015233	0,0138753	0,526487	0,015462	0,0209434	0,0160593	0,267846	0,349435	0,6279808
CG17019-RA	Marf1	0,0149809	0,0136457	0,0143826	39,3226	39,6612	0,0157842	-0,205021	0,330687	0,6279808
CG1701-RB	CG17019	7,89361	45,7902	0,0353277	44,4803	0,0582759	0,0364081	NA	NA	0,6279808
CG17023-RA	GstT3	1,4938	0,0680328	0,0285366	4,64275	0,0441721	0,0914518	-0,152858	0,566664	0,6279808
CG17023-RB	GstT3	8,05045	8,62261	7,82407	5,40282	3,85192	10,2135	-0,144221	0,590333	0,6279808
CG17023-RC	Dbp80	0,100317	16,9831	0,0963106	0,129321	0,0308663	0,132016	-0,366786	0,176964	0,6279808
CG17024-RA	Dbp80	0,0509189	0,28763	0,0488852	0,0573709	0	0,0585664	-0,490593	0,144535	0,6279808
CG17025-RA	Dbp80	64,455	0,091376	42,4558	83,1103	0,175166	48,2097	-0,380132	0,245593	0,6279808
CG17026-RA	CR17024	4,42174	2,22913	2,6874	4,47235	0	28,1461	-0,422634	0,237128	0,6279808
CG17027-RA	asRNA:CR17025	0,118486	6,77197	34,1347	12,4949	15,2255	0	-0,221749	0,494112	0,6279808
CG17028-RA	CG17026	0,966022	1,19669	2,44844	1,15204	3,48716	2,20671	-0,263276	0,440746	0,6279808
CG17029-RA	CG17027	6,68902	16,7333	10,3867	4,87613	9,35531	9,35362	-0,3134	0,352709	0,6279808
CG1702-RA	CG17028	1,23651	28,7951	2,78232	1,64797	2,88161	2,29612	0,241012	0,341506	0,6279808
CG1702-RB	CG17029	30,0435	4,52105	58,7296	24,948	62,667	55,2076	0,238295	0,349645	0,6279808
CG17030-RA	CG1703	25,1728	5,30234	6,8434	6,999	26,2312	21,777	-0,041811	0,731452	0,13772387
CG17031-RA	CG17030	0	60,6549	163,708	0	0	9,64435	NA	NA	0,13772387
CG17032-RA	Ref2	0	0	0	0	1,19684	0	-0,062501	0,832872	0,13772387
CG17033-RA	CG17032	27,5768	0,0280372	50,3941	0,0329867	0,0446807	0,0336741	-0,322859	0,204136	0,6279808
CG17035-RA	elgi	15,0117	13,9539	14,2743	15,4119	29,2443	17,713	0,138061	0,526018	0,13772387
CG17035-RC	GXIVsPLA2	9,26156	8,57213	8,46141	8,92112	7,11952	8,22714	0,138061	0,526018	0,6279808
CG17036-RA	GXIVsPLA2	0,0741036	2,09246	1,67188	3,36053	2,00337	0,0905897	-0,69021	0,052551	0,6279808
CG1703-RA	CG17036	1,79244	0,917286	2,34015	2,62169	0,0615725	4,81923	0,026802	0,916633	0,6279808
CG17044-RA	yellow-e2	2,19221	25,967	4,8816	6,58806	2,05852	5,32136	-0,815412	0,003909	0,6279808
CG17045-RA	yellow-e3	2,93058	1,52862	19,421	54,3069	59,6389	64,7277	-0,398395	0,236469	0,13772387
CG17046-RA	klar	5,2099	0,651745	43,1726	9,6142	10,1895	8,29541	0,161991	0,509841	0,6279808
CG17046-RB	klar	46,0245	1,03545	4,86714	15,4944	9,17646	10,5679	0,176583	0,468861	0,13772387
CG17046-RC	klar	4,20588	2,76367	0,00934451	4,6298	2,32978	11,7441	0,371904	0,12129	0,13772387
CG17046-RD	klar	0,00973326	7,97839	420,597	9,60036	2,45546	2,89666	-0,329095	0,20273	0,13772387
CG17047-RA	CG17047	11,7158	12,8244	13,4437	0	4,34717	0	0,456742	0,132242	0,13772387
CG17048-RA	CG17048	0	0,240746	6,71844	0	0	3,48925	0,01562	0,897983	0,6279808
CG17049-RA	CG43691	0	0,562296	1,03167	0,569637	0	6,38368	NA	NA	0,13772387
CG17050-RA	Met	4,79211	15,9861	427,796	27,3272	0,234245	5,23355	NA	NA	0,6279808
CG17051-RA	CG43691	0	0	0	9,39839	15,7482	0	-0,128663	0,656324	0,6279808
CG17052-RA	dod	16,429	12,3109	27,2464	19,709	13,2605	25,077	0,584856	0,020606	0,6279808
CG17058-RA	obst-A	285,846	0,0199645	0,0218924	0,0240523	3,27397	1,49455	-0,133118	0,479374	0,6279808
CG17058-RB	Peritrophin-A	0,0511951	0,046632	75,2751	0	0	0	-0,134025	0,47626	0,6279808
CG17059-RA	Peritrophin-A	68,6968	60,2798	0	0	0	0	0,00165	0,995046	0,6279808
CG1705-RA	CG17059	77,1457	60,7336	77,8542	89,463	117,017	126,179	-0,236919	0,28903	0,6279808
CG17060-RA	Rab10	49,9609	0,0191677	40,6903	28,2865	6,41455	0,0235066	-0,096536	0,591348	0,6279808
CG17061-RA	mthl10	8,6051	10,6274	10,5314	10,0586	7,45334	4,9039	-0,063935	0,766067	0,6279808
CG17061-RB	mthl10	5,75968	5,67605	6,54875	4,03015	5,22147	0,0411777	0,010846	0,958945	0,6279808
CG17063-RA	Inx6	0	0	0,0491503	0	0	0	NA	NA	0,6279808
CG17064-RA	mars	0,0207091	44,5153	74,0295	63,4461	0,0391018	11,5764	-0,06018	0,84582	0,6279808
CG17065-RA	CG17065	19,4009	14,6917	0	0,0230268	47,417	0,0234738	-0,217099	0,387524	0,6279808
CG17068-RA	CG17068	0,0210433	19,7141	0,0210707	0,0229947	24,8551	0,0242088	0,030256	0,888211	0,6279808
CG17075-RA	Glo1	21,0976	17,1728	57,8775	27,9486	0	51,2681	-0,067056	0,834614	0,6279808
CG17077-RB	Dsim\GD22974	1,04558	1,35611	45,9052	2,20466	0,247689	0	-0,101364	0,640611	0,6279808
CG17077-RC	pnt	7,70542	8,7814	8,73129	8,61267	4,69813	6,37081	0,114977	0,593361	0,13772387
CG17077-RD	pnt	1,8016	1,69922	0,024534	2,15066	0,0366304	0,0276069	-0,043866	0,845545	0,13772387
CG17078-RA	pnt	2,99612	3,3898	3,50221	5,68541	4,17305	3,59835	0,074079	0,78355	0,6279808
CG1707-RA	CG17078	9,08634	9,71658	10,1715	10,021	0,00503799	4,07167	-0,315427	0,35432	0,6279808
CG17081-RA	cos	7,18638	7,54854	6,5936	7,10531	4,56494	4,53591	0,168069	0,522263	0,6279808
CG17082-RA	Cep135	2,83519	2,67184	4,1908	4,18225	1,8264	2,1862	0,012045	0,957124	0,6279808
CG17082-RB	conu	12,2663	23,2266	9,33261	25,5831	12,6845	17,3891	0,009819	0,965042	0,6279808
CG17082-RC	conu	36,3995	20,8191	18,624	9599,61	0,0419506	0,0222619	0,017647	0,937146	0,6279808
CG17082-RD	conu	0,0307091	23,1422	1202,21	0,0218075	0,0466665	0,0217508	0,017516	0,937609	0,6279808
CG17082-RE	conu	0,0290231	0,027972	0,0722788	0,0213068	6,20147	29,0999	0,011695	0,958348	0,6279808
CG17082-RF	conu	0,0320497	0,0264362	2354,97	42,5899	8,21921	0,0290495	0,014789	0,947401	0,6279808
CG17083-RA	conu	8,24914	0,0291931	0,0618492	7,08919	39,2414	24,5792	-0,964158	0,006691	0,6279808
CG17084-RA	CG17083	0,352898	0,183682	0,0232625	0,099721	0,0838549	0,168569	-0,25164	0,215283	0,6279808
CG17085-RA	mthl9	15,9301	15,5406	14,5054	11,702	6,73764	6,72108	0,073866	0,83015	0,6279808
CG17086-RA	SmydA-3	0,466144	0,58382	0,0136365	0,0148087	0,0308131	0,0151173	0,788501	0,00253	0,13772387
CG1708-RA	SmydA-3	3,24832	0	3,52284	0,0439711	9637,63	8,88434	0,257307	0,216525	0,13772387
CG17090-RA	Vha100-1	0,0227612	0,630837	6,93149	0,680779	0,724896	11,1824	-0,126508	0,70806	0,6279808
CG17090-RB	Vha100-1	0,0212531	1,58385	0,1152	8,3597	8,81108	5,85425	-0,130149	0,699908	0,13772387
CG17097-RC	Vha100-1	0,0217141	2,50492	36,1756	1,47528	3,2982	0,167525	NA	NA	0,6279808
CG17098-RA	Vha100-1	0,02165	0,0207325	0,714156	24,1406	15,586	21,9477	-0,041811	0,731452	0,6279808
CG1709-RA	Vha100-1	0,0213561	0,0193588	0,760136	0,952416	0,570856	4,87358	-0,184975	0,321168	0,6279808
CG1709-RB	Vha100-1	10,7147	0,0197787	10,8368	3,43675	2,90983	2,8815	-0,175856	0,342509	0,6279808
CG1709-RC	Vha100-1	13,0413	0,0197203	5,36661	3,96992	4,67046	1,01126	-0,185758	0,318937	0,6279808
CG1709-RD	Vha100-1	5,63982	0,0194526	4,22732	0,0511772	6,43757	3,89237	-0,176312	0,34125	0,6279808
CG1709-RE	Hipk	5,5419	7,28988	2,32676	3,90662	7,71459	3,55974	-0,186206	0,317766	0,6279808
CG1709-RF	Hipk	6,54682	17,1378	20,1877	4,47434	1,9731	33,7153	-0,184526	0,322347	0,6279808
CG1709-RG	Dsim\GD23720	0	0	0	0,125905	49,1066	41,65	-0,175084	0,344055	0,13772387
CG1709-RH	CG17098	0	0,571753	0	0	0,0341104	0,0257077	-0,174013	0,348897	0,6279808

gene_id	Symbol	W1_FPKM	W2_FPKM	W3_FPKM	MCM51_FPKM	MCM52_FPKM	MCM53_FPKM	FC	p-value	p-adj
CG17100-RA	Hcf	0,00921603	0,0083946	0,00884794	0,00938558	2,31219	2,06546	0,111857	0,656236	0,6279808
CG17104-RA	Hcf	18,9429	21,5388	15,4371	23,7235	9,7006	23,8417	0,574342	0,003046	0,6279808
CG17105-RA	Hcf	0,00919167	0,00837241	0,00882455	0,00936025	0,0126785	4,95122	-0,041811	0,731452	0,6279808
CG17107-RA	Hcf	2,71709	0,00807475	2,56601	6,80321	2,13109	3991,39	-0,592535	0,08112	0,6279808
CG17108-RA	cwo	20,5735	28,1844	17,2668	26,2938	15,0135	14,5088	-0,111614	0,692634	0,6279808
CG17109-RA	CG17104	26,2667	19,4128	11,7067	24,4376	37,7842	41,4619	-0,469295	0,182626	0,6279808
CG1710-RA	CG17105	0	0	0	0	1,19286	13,0197	0,006044	0,981061	0,6279808
CG1710-RB	CG17107	11,5976	0,332522	0	40,3333	16,4106	15,7247	0,009101	0,971457	0,6279808
CG1710-RC	CG17108	0	0,154854	0,217622	0,221028	0,138945	0,225634	0,009101	0,971457	0,6279808
CG1710-RD	CG17109	45,6983	18,4924	96,0511	38,1783	20,8107	1,33034	0,00901	0,971739	0,6279808
CG17110-RA	CG17110	0,689281	0,974242	1,02685	1,81387	16,3743	0,0527351	-0,329775	0,3218	0,6279808
CG17111-RA	CG17111	4,03845	5,58297	4,63333	0,444373	15,0396	2,73673	0,565322	0,024563	0,6279808
CG17116-RA	Lip2	1,51623	33,9572	48,4232	28,1982	0	69,9948	-0,254356	0,445122	0,6279808
CG17117-RA	hth	0,0207351	18,3281	0	16,5116	0,0293827	0,0221446	0,460565	0,036104	0,6279808
CG17117-RB	hth	16,8386	11,9085	0	11,6075	0,0222986	0,0168056	0,460592	0,036075	0,6279808
CG17117-RC	hth	24,2297	11,0807	0	9,3179	19,7472	18,4459	0,460674	0,036074	0,6279808
CG17117-RD	hth	0,015904	4,14786	0	5,34896	0,0222819	0,016793	0,419069	0,068969	0,6279808
CG17117-RE	hth	44,1837	43,0945	0	0,800214	27,4186	31,5172	0,430538	0,038713	0,6279808
CG17117-RF	hth	64,8858	0,793274	0	51,0269	26,4347	39,6136	0,537366	0,012856	0,6279808
CG17118-RA	CG17118	0,0707702	9,49913	18,9563	42,9323	20,3682	10,374	0,502505	0,103969	0,6279808
CG17119-RA	CG17119	0,0428878	9,99767	18,1437	0,0473523	0,064139	9,50553	-0,168683	0,60886	0,6279808
CG17119-RB	CG17119	10,2278	0,0390652	8,52854	6,57661	16,4304	4,91711	-0,169009	0,608267	0,6279808
CG17121-RA	Gr43a	0,0452058	0,0431238	0,0434003	0,0502024	0,0679994	0,0512485	0,247464	0,261284	0,6279808
CG17122-RA	Gr43a	0,0473435	0	0,0454526	0,0528605	0,0715998	0,053962	-0,334768	0,276366	0,6279808
CG17124-RA	CG17121	27,0417	7,03816	6,2947	2,93458	6,9262	4,86847	0,271655	0,210682	0,6279808
CG17124-RB	CG17122	0,609306	5,93896	257,751	4,95417	4,47113	4,5292	0,460653	0,023028	0,6279808
CG17127-RA	CG17124	78,8048	343,951	24,6431	12,3434	14,6152	16,0115	0,428121	0,059282	0,6279808
CG17127-RB	CG17124	0,0555881	4,94333	9,27089	66,6288	3,93069	6,35884	0,42828	0,059189	0,6279808
CG17129-RA	CG17127	0,175889	0,160212	553,914	0,106939	0,161904	0	0,426342	0,139915	0,6279808
CG17129-RB	CG17127	418,285	417,448	1,45574	0,483532	0,0406513	0	0,426342	0,139915	0,6279808
CG17129-RC	CG17129	0,299302	0,00790713	1,57996	9,50481	0,0433567	0,0326762	0,459661	0,112263	0,13772387
CG1712-RA	CG17129	1,25931	0,00886573	0,0294963	10,5174	1,09494	0,593082	0,368451	0,147822	0,13772387
CG1712-RB	CG17129	0,0307234	178,156	128,197	0,0320092	0,0445914	0,527235	0,368451	0,147822	0,6279808
CG17131-RA	tyn	25,1875	23,0861	16,1637	7,77704	3,26319	6,78263	-0,05043	0,879392	0,6279808
CG17131-RB	tyn	13,3863	14,7493	16,8413	3,24782	13,5417	15,2697	-0,050456	0,879328	0,6279808
CG17134-RA	CG17134	11,2838	1,9361	9,24672	10,9667	1,6958	0	-0,136684	0,674601	0,6279808
CG17136-RA	Rbp1	0,112437	0,0827114	37,0507	14,6594	52,096	0,743374	-0,218831	0,443172	0,6279808
CG17136-RB	Rbp1	22,7466	18,1093	12,5301	1,16359	1,98102	2,58628	-0,316865	0,297294	0,6279808
CG17136-RC	Rbp1	10,5493	32,9223	54,0043	0,0429116	3,16781	1,89178	-0,220095	0,440408	0,13772387
CG17136-RD	Rbp1	30,6511	12,8503	10,7507	0,0622795	0,0581241	0,275849	-0,168473	0,522892	0,6279808
CG17137-RA	Porin2	0,845631	0,903063	0,168864	0,0621314	0,310001	0,342357	1,120825	0,001428	0,6279808
CG17140-RA	CG17140	0,0546231	0,124386	0,0524415	4,96269	2,26583	1,87025	0,204949	0,447993	0,6279808
CG17140-RB	CG17140	0,084544	0,0256695	0,202918	3,52619	2,97271	2,81791	0,12831	0,586281	0,6279808
CG17141-RA	CG17141	5,168	6,86616	3,11768	16,6223	1,04755	0,660698	-0,255713	0,356727	0,6279808
CG17142-RA	pyx	0,0271507	0,066139	34,4228	0,0931304	0,0390676	0,0294437	-0,126329	0,620997	0,6279808
CG17142-RB	pyx	0,0169527	0,0941886	76,4746	0,150947	1,87641	1,60484	0,038637	0,877202	0,6279808
CG17143-RA	thoc7	22,1561	0,0529811	45,0158	0,0705841	14,3258	19,21	0,353745	0,185897	0,6279808
CG17143-RB	thoc7	0,0711258	0,0526566	0,134494	0,0700587	0,122847	0,0925847	0,353745	0,185897	0,6279808
CG17145-RA	CG17145	8,25949	1,6906	27,042	6,10192	7,35575	6,62346	0,025788	0,942533	0,6279808
CG17146-RA	Adk1	30,511	5,21881	0,0401075	37,1336	116,137	94,55	-0,801226	0,016958	0,6279808
CG17146-RB	Adk1	7,60181	74,7985	7,38695	10,4261	20,7079	16,0038	-0,803828	0,016478	0,6279808
CG17147-RA	CG17147	3,57343	17,3391	7,60992	3,51838	14,6776	12,1789	-0,337934	0,333046	0,6279808
CG17148-RA	Est-P	0,0417761	4,01063	659,445	0,045997	5,89096	53,9421	0,342774	0,330446	0,6279808
CG17149-RA	Su(var)3-3	1,86677	0,0489453	4,03469	0,0196251	1,36162	0,020034	0,423605	0,125059	0,6279808
CG17149-RB	Su(var)3-3	2,44958	57,7427	0,687433	46,603	0,332653	35,0296	0,423605	0,125059	0,6279808
CG17150-RD	Chchd3	73,932	0,0471312	0,131612	92,4931	2,41151	9,45307	-0,264216	0,448555	0,6279808
CG17150-RE	Dnah3	0,0398287	0	0,038238	0,0397177	0,0537979	0,0405453	0,01562	0,897983	0,6279808
CG17152-RA	Dnah3	0,0467483	0	0,0661407	0,0848552	0,0602516	0,0421293	0,11473	0,590073	0,6279808
CG17153-RA	Ir68b	0,0519465	0,0473164	0,0831195	0,0933478	0,0171553	0	-0,168754	0,371541	0,6279808
CG17154-RA	ssp	14,8659	8,2202	12,5458	10,9783	0,0528573	9,46461	0,015925	0,916254	0,6279808
CG17158-RA	CG17154	0	0,209953	0,221291	0	0,0550074	0,443458	0,088381	0,66917	0,6279808
CG17159-RA	cpb	33,0379	31,9083	40,4973	14,921	22,566	30,0726	0,373592	0,132034	0,6279808
CG1715-RA	CG17159	18,9311	27,7251	14,9116	14,3741	8,15687	0	-0,086351	0,738302	0,6279808
CG17161-RA	Set2	3,92903	4,82624	4,65002	6,43033	1,60446	2,71022	-0,324822	0,13714	0,6279808
CG17161-RB	grp	7,33617	5,16984	10,7386	13,2811	8,54935	9,36277	-0,298064	0,165031	0,6279808
CG17161-RC	grp	0,0287453	0,0261832	0,0275972	0,0306541	0,0415212	0,0312929	-0,297601	0,16515	0,6279808
CG17161-RD	grp	2,07498	2,33215	4,43855	5,88898	6,31802	3,70335	-0,203902	0,333934	0,6279808
CG17162-RB	grp	7,44642	6,51967	5,70336	0,0453637	5,70321	5,30449	0,366161	0,139454	0,6279808
CG17162-RC	CG17162	0,0173019	0,0157598	4,37696	4,92994	3,81696	0	0,367509	0,13862	0,6279808
CG17162-RD	CG17162	0,0183036	0,0166722	0,0175725	0,0193371	0,0261923	0	0,372709	0,133046	0,6279808
CG17162-RE	CG17162	5,39451	0,0165166	0,0174086	6,49403	3,31225	0	0,3671	0,138911	0,6279808
CG17163-RA	CG17162	0,0189112	0,0172257	0,0181559	0,0200186	0,0271153	0	0,400049	0,084972	0,6279808
CG17166-RA	CG17163	6,88262	0,169698	0,178862	3,00066	6,66245	2,06938	-0,473866	0,069427	0,6279808
CG17167-RA	mRpL39	24,7712	30,8041	37,8789	37,6568	48,0773	44,5692	-0,310927	0,166531	0,13772387
CG17167-RB	CG17167	4,44673	2,55309	0,0404657	4,6018	3,76421	5,21609	-0,311907	0,164856	0,13772387
CG17168-RA	CG17167	2,9561	5,5391	9,7912	5,01548	13,2742	0,327223	0,153449	0,511903	0,13772387
CG17169-RA	CG17168	17,8067	6,5221	6,98858	0,320543	3,90652	6,11573	0,470009	0,175402	0,6279808
CG1716-RA	CG17169	1,88021	2,93593	28,7981	26,1267	0,434178	11,9977	0,200019	0,485084	0,6279808
CG17170-RB	su(f)	0,0243224	0,0221545	0,0233509	0,0256635	0,0347614	0,0859049	-0,051197	0,822874	0,6279808
CG17170-RE	su(f)	9,92049	13,9813	12,1116	13,4486	6,21972	0	-0,047059	0,838301	0,6279808
CG17172-RA	ATbp	3,80303	4,37355	0,185126	0,340323	0,460969	2,49292	0,340792	0,155903	0,6279808
CG17173-RA	CG17173	0,0431117	6,3158	0,039998	6,76984	0,0645098	0,0486185	0,11643	0,74382	0,6279808
CG17174-RA	ACXB	0	0,0435923	0	0,11501	0	0,0392667	NA	NA	0,6279808
CG17176-RA	ACXA	0	0	10,1199	0	0	6,08966	0,01562	0,897983	0,6279808
CG17177-RA	CG17177	0,0495321	0,106714	0,0713307	0,135855	0,0567562	8,45025	0,066302	0,844662	0,6279808
CG17178-RA	ACXE	0	11,5685	0,01808	0	0	0	NA	NA	0,6279808
CG17180-RA	ABCA	3,46079	0,0155816	16,5536	4,17113	0,12286	3,30759	-0,213082	0,429897	0,6279808
CG17181-RA	BORCS6	4,76594	4,09997	5,50765	5,22463	0,449777	6,13443	0,042416	0,897784	0,6279808
CG17183-RA	Kah	4,21313	4,42391	3,71902	8,10234	1,53709	2,47577	-0,153145	0,595348	0,6279808
CG17184-RA	MED30	5,07233	2,07412	1,66888	38,1804	1,69269	1,90184	0,146315	0,437986	0,6279808
CG17184-RB	Arfip	10,9585	11,1453	12,4249	0,607257	2,4604	2,86949	0,146918	0,436134	0,13772387
CG17186-RA	Arfip	0,0311287	0,0283542	0,0298854	12,5743	8,54521	3,00654	0,219132	0,44961	0,13772387
CG17187-RA	Dpse\GA14373	2,64772	6,98668	10,1128	3,90243	1,51352	11,9154	-0,217456	0,420161	0,6279808
CG17189-RA	CG17187	2,50449	7,30855	10,7357	28,6562	2,90747	8,37312	NA	NA	0,6279808
CG1718-RA	CG17189	0	0	0,328121	6,90241	8,77731	0,280698	-0,326166	0,183918	0,6279808
CG17190-RA	CG17190	4,09989	3,44207	31,0899	45,7566	3,08179	27,8131	0,365162	0,197387	0,6279808
CG17191-RA	CG17191	0,0909301	0,0828254	2,41525	0,456275	0,0415792	1,1431	-0,091662	0,767437	0,6279808
CG17192-RA	CG17192	0	0	0,15004	0	0,0454082	0,118074	-0,138638	0,481815	0,6279808
CG17193-RA	CG17193	3,96624	2,62115	45,5174	0,0691036	0,0936012	0,0705435	0,069585	0,799641	0,6279808
CG17195-RA	CG17195	0	0	0	0	0	0	NA	NA	0,6279808
CG17196-RA	CG17196	0	0	0	0	0	0	-0,041811	0,731452	0,6279808
CG17197-RB	CG17197	0	0,147878	0	0	0,211725	0	NA	NA	0,6279808
CG17198-RA	CG17198	0	0	0,0779322	0	0	0,0426536	-0,041811	0,731452	0,6279808
CG17199-RA	CG17199	0	1,44658	0	0,0711819	0,0964163	0	-0,074864	0,550506	0,6279808

gene_id	Symbol	W1_FPKM	W2_FPKM	W3_FPKM	MCM51_FPKM	MCM52_FPKM	MCM53_FPKM	FC	p-value	p-adj
CG17200-RA	Ugt35E1	0,338504	0,876502	9,80692	1,65983	0,314087	0,438965	-0,870953	0,01442	0,6279808
CG17202-RA	CG17202	15,7384	653,999	16,3631	3,35155	2,34954	16,8935	-0,05292	0,838906	0,6279808
CG17207-RA	CG17207	0,155757	7,55056	0,149536	0,319031	330,314	36,801	-0,153029	0,588656	0,6279808
CG17208-RA	Gfrl	0,294982	0,0162945	0,0330974	0,0185897	0,0251799	0,0189771	-0,200281	0,574477	0,6279808
CG17209-RB	RpIIIC160	2,63362	1,91496	3,06764	17,2832	8,55012	2,0793	0,056693	0,824512	0,6279808
CG17210-RA	Pglym78	0,0459924	3,00064	15,1901	5,45958	6,34698	2,47264	0,540925	0,129468	0,6279808
CG17211-RA	Pglym78	0,0499565	0,0179197	3,4388	0,0327084	0,0443037	5,06793	-0,189474	0,570432	0,6279808
CG17212-RA	Pglym78	119,311	1,14725	5,77943	0,0336608	0,0455937	0,0333899	-0,759146	0,02521	0,6279808
CG17212-RB	scpr-B	0,077584	4,92027	0,0744853	5,99484	2,77777	8,4309	-0,874648	0,011624	0,6279808
CG17213-RA	CG17211	2,66579	2,98341	8,219	11,3054	1,10786	7,38712	-0,472321	0,186929	0,6279808
CG17216-RA	rho-6	0,114981	0	67,0435	0,0546813	0,119305	0,031856	0,219072	0,496596	0,6279808
CG17217-RA	rho-6	0,357811	2,49909	0,355388	62,8395	0,318161	49,5208	0,408859	0,249591	0,6279808
CG17218-RA	Gr33a	0,300766	0,231811	0,0260878	73,215	36,1254	43,6606	-0,275094	0,265584	0,6279808
CG17218-RB	KP78b	7,98998	3,73679	9,31462	9,24947	1,9236	2,94971	-0,275094	0,265584	0,13772387
CG17219-RA	CG17217	0,136803	33,2485	0,131339	0,234891	5,16888	0,239785	-0,130508	0,673522	0,13772387
CG1721-RA	crok	0,0680725	0,0620052	0,0653537	0,080207	0,108641	0,0818783	-0,232752	0,418246	0,6279808
CG1721-RB	crok	64,2716	48,9605	56,411	67,1464	68,7135	76,5752	-0,232656	0,418466	0,13772387
CG1721-RC	CG17219	4,41387	3,29822	50,5538	2,78557	5,2849	44,2173	-0,232313	0,419254	0,6279808
CG17221-RA	CG1722	0,337984	0,820958	5,91103	0,0611512	10,7832	0,0624254	-0,430743	0,151247	0,6279808
CG17221-RB	CG17221	5,25088	5,28257	2649,34	2,88324	0,751703	0,695652	-0,430133	0,151652	0,6279808
CG17223-RA	CG17221	0,0487764	0,0444289	0,102538	19,2279	25,9227	18,7616	0,078431	0,744996	0,6279808
CG17224-RA	alpha4GT1	5,18112	4,64728	6,83476	5,70467	0,0271071	1,652	-0,18858	0,487159	0,6279808
CG17224-RB	CG17224	0,0509189	0,0463805	0,0488852	0,0573709	4,45795	7,0747	-0,187777	0,4889	0,6279808
CG17226-RA	CG17224	21,806	16,6939	23,5769	18,9847	0,0229229	0,0208604	0,065796	0,591492	0,6279808
CG17227-RA	Or59c	0	0,0730419	0	0	0,300741	0	0,284555	0,189084	0,6279808
CG17228-RE	DNAlig3	3,41546	3,6355	3,88209	0,0139202	0,247537	2,36182	0,1367	0,627109	0,13772387
CG17228-RF	pros	0,00922246	0,00840046	55,315	45,9453	1,31821	2,23259	0,137757	0,624647	0,6279808
CG17228-RG	pros	5,64175	3,7427	4,82817	9,16746	0,243697	1,84085	0,137134	0,626072	0,6279808
CG1722-RA	pros	0,785843	2,58755	7,09637	0,00939227	39,1455	47,8814	0,060735	0,863036	0,6279808
CG17230-RA	CG17230	4,06905	3,44678	0,030401	4,71546	13,59	0,0347169	0,131808	0,535742	0,6279808
CG17230-RB	CG17230	0,0348562	0,0317495	0,0334641	0,0377274	0,0460644	0,0385135	0,151747	0,561008	0,13772387
CG17233-RA	CG17233	3,1028	0,0724555	158,732	0,346192	3,73343	23,0945	-0,061288	0,805714	0,13772387
CG17233-RB	CG17233	5,38529	0,0930716	14,9631	0,887044	1,44068	0,0395089	-0,069057	0,784417	0,13772387
CG17233-RC	CG17233	0,0120775	97,823	9,60541	31,3919	0,130958	18,6564	-0,090019	0,718509	0,6279808
CG17234-RA	CG17234	0	0	0	0	0	0	NA	NA	0,13772387
CG17237-RA	CG17237	0	0	0	0	0	0	NA	NA	0,6279808
CG17239-RA	CG17239	0	0,565856	0	0	0	17,2793	-0,041811	0,731452	0,6279808
CG17240-RA	CG1724	0,11688	0	0	0	0	0	NA	NA	0,6279808
CG17241-RA	Ser12	0	0	1,03017	7,41749	0	0	-0,132075	0,667564	0,6279808
CG17242-RA	Or94a	0,0851493	1,04976	10,5239	5,89077	13,3058	10,6206	NA	NA	0,6279808
CG17244-RA	CG17242	0	0	0	0,302375	22,0856	0	-0,105123	0,75449	0,6279808
CG17245-RA	Gbp3	13,9919	7,46274	29,7775	16,482	23,9263	24,7143	0,091495	0,731531	0,6279808
CG17246-RA	PlexB	26,1343	21,8253	19,7089	26,8619	12,8002	11,8017	-0,058719	0,765729	0,6279808
CG17246-RB	Dpse\GA14410	0,0247599	0,022553	8,79525	13,9752	8,51467	7,75438	-0,058066	0,768265	0,6279808
CG17246-RC	Dpse\GA14410	0,0254758	0,0232052	0,0244583	0,0269548	0,0365105	0,0275165	-0,058584	0,766287	0,6279808
CG17248-RA	Dpse\GA14410	59,1466	79,3771	70,3797	71,8307	55,7027	53,3716	0,228687	0,323499	0,6279808
CG17248-RB	nSyb	0,0409476	0,727085	0,0393122	0,829344	0,0609419	0,043354	0,228195	0,324616	0,6279808
CG17248-RC	nSyb	0,0388529	4,65642	0,0373011	5,64067	0,0575246	20,2486	0,297702	0,204655	0,6279808
CG17248-RD	nSyb	33,3586	0,0222036	37,3874	0,0257237	25,3743	0,0447381	0,228118	0,32479	0,6279808
CG17248-RE	nSyb	0,0399813	5,67564	0,0383845	11,1195	0,0593611	0,0764788	0,228623	0,323729	0,6279808
CG17249-RA	nSyb	0,0642344	0,0202398	0,0616688	0,0233284	0,101477	5,68992	0,107999	0,674051	0,6279808
CG1724-RA	CG17249	6,33257	0,0585091	41,2695	7,85962	21,9275	6,28914	0,01562	0,897983	0,6279808
CG17250-RA	dlg1	0,025591	14,9141	7,29184	0,0276912	125,775	6,54323	-0,331276	0,28627	0,6279808
CG17252-RA	dlg1	0,0308478	14,069	7,62469	0,0339732	0,0555553	3,0214	0,140312	0,552342	0,6279808
CG17255-RA	dlg1	0,0310215	0,02331	3,95971	0,0341847	0,0747167	4,24541	0,061117	0,834127	0,6279808
CG17255-RB	dlg1	11,2793	0,0280983	0,0251866	0,0133262	0,0750939	1,08627	0,06141	0,8332	0,6279808
CG17256-RA	dlg1	5,00207	0,0282566	0,0149064	0,0128656	0,0814687	0,0278931	-0,275627	0,437597	0,6279808
CG17257-RA	dlg1	0,0127102	14,7463	0,0136316	5,65923	0,0787376	6,71643	-0,202459	0,329805	0,6279808
CG17257-RB	dlg1	0,0262344	0,0114191	0,0133028	0,027808	0,0745918	35,9754	-0,202459	0,329805	0,6279808
CG17258-RA	dlg1	0,0155266	0,0115773	7,99274	10,7089	0,0785297	0,0477544	0,239118	0,421705	0,13772387
CG17259-RA	dlg1	0,0141987	0,0238962	0,0131867	0,0146279	1108,79	104,665	-0,286857	0,22493	0,6279808
CG1725-RA	dlg1	0,0138563	0,0141427	52,5843	0,0142637	0,0574506	44,2547	0,041408	0,862683	0,6279808
CG1725-RB	dlg1	8,53802	8,32247	102,117	17,2578	350,028	102,939	0,155046	0,495786	0,6279808
CG1725-RC	dlg1	5,34302	0,0126212	0,0361326	5,85817	0,0561494	0,0418698	1,091331	4,47E-08	0,6279808
CG1725-RD	Or42a	0,135291	0	0,0779322	548,234	3,11357	0,159569	0,04128	0,86319	0,6279808
CG1725-RE	BCL7-like	11,4837	410,134	12,9166	0,641389	6,42159	0,288685	0,044904	0,851106	0,6279808
CG1725-RF	nocte	9,57613	0,0254847	0,00782912	14,4322	0,0112215	0,889824	-0,329743	0,114043	0,6279808
CG1725-RG	nocte	1,3904	0,0256367	22,8529	0,0297813	1811,28	10,3062	0,041338	0,862923	0,6279808
CG1725-RH	Nek2	0,395348	0,288088	1,48028	1,30541	0,747131	0,769451	0,153861	0,500179	0,6279808
CG1725-RI	GABPI	6,74079	6,87997	9,12927	45,6948	6,88492	2,84361	0,94077	8,07E-06	0,13772387
CG1725-RJ	GABPI	0,0418554	0,0381248	1,4985	0,807109	0,062434	6,77075	0,932921	1E-05	0,13772387
CG1725-RK	CG17258	0,0235509	0,0214517	0,143956	5,21857	0,0335968	3,87192	0,063654	0,789534	0,13772387
CG1725-RL	SerRS	31,2591	0,0972839	0,0226102	16,8215	4,65147	14,1864	0,155015	0,496253	0,13772387
CG17260-RA	CG17260	1,40739	0,13658	8,49523	0,0460935	4,03742	0,0253206	-0,10198	0,741937	0,13772387
CG17261-RA	CG17261	1,20536	0,424239	1,52543	3414,21	4342,09	3856,37	-0,4561	0,183029	0,6279808
CG17262-RA	cnir	9,61864	2337,17	0,0401837	0,0248037	6,20787	2,82178	-0,416949	0,101515	0,13772387
CG17264-RA	CG17264	0,714289	0,426271	0,780348	10,9677	0,0272312	9,30864	-0,315993	0,250163	0,6279808
CG17265-RA	CG17265	17,1167	25,1066	14,1376	16,6605	8,12877	1,27847	0,568729	0,037463	0,6279808
CG17266-RA	CG17266	7,31785	3,18577	5,05042	5,71165	3,80329	5,30901	-0,434182	0,197771	0,6279808
CG17267-RA	CG17267	0,0777667	0,314085	0,66899	0,924069	0,127406	1,98474	-0,601821	0,056864	0,6279808
CG17267-RB	CG17267	0,113989	5,30667	5,77969	4,06749	2,66881	2,41117	-0,514761	0,106232	0,6279808
CG17268-RA	Prosalpha4T1	0,104771	0,190866	0	32,8507	0,180057	0,135702	-0,137449	0,60964	0,6279808
CG17269-RA	Fancd2	0,172409	1,58125	5,05203	2,51259	0,355347	0,304234	-0,33644	0,339538	0,6279808
CG17270-RA	CG17270	5,85022	2,4222	4,68977	6,76874	3,05199	3,2649	0,647069	0,005817	0,6279808
CG17271-RA	CG17271	25,012	22,1301	30,5022	0,0363135	15,2517	21,5386	0,384919	0,119888	0,6279808
CG17271-RB	CG17271	29,3347	28,0322	30,6567	0,0355687	11,0251	19,0417	0,379486	0,087947	0,6279808
CG17272-RA	CG17272	0,107674	0,0980765	0,103373	0,141849	0,192135	0,144805	-0,144733	0,566288	0,6279808
CG17273-RA	AdSS	75,6878	0,780195	0,646115	0,471694	0,854652	0,644117	-0,525398	0,059013	0,6279808
CG17278-RA	CG17278	11,6754	0	14,0971	0,364966	0	0,552026	0,465178	0,099652	0,6279808
CG17279-RA	CG17279	0,157324	0,431023	0,4543	0,839903	7,66115	0,182032	-0,281899	0,392754	0,6279808
CG17280-RA	Tim8	59,9805	0,0154496	103,908	90,826	0,0583902	26,5252	-0,400128	0,212439	0,6279808
CG17282-RA	Dpse\GA14437	274,572	272,263	10,2634	403,019	0,0346804	943,089	0,1576	0,608575	0,6279808
CG17283-RA	Bdbt	5,45875	6,13532	9,48812	4,66545	7,47483	0,0195849	0,05818	0,868056	0,6279808
CG17284-RA	CG17283	0,259955	0,00745559	0	0	0,0391876	0,0197982	-0,692476	0,040584	0,6279808
CG17284-RB	Obp93a	0,80471	0,559699	0,0177016	1,50027	0,0409309	80,2168	-0,700388	0,040121	0,6279808
CG17285-RA	Obp93a	0,122893	26,0986	0,0177016	7,55635	0,686986	5,68325	-0,552024	0,122959	0,6279808
CG17285-RB	Fbp1	0,0193506	1,42416	3,80683	3,4064	10,8437	9,31725	-0,552024	0,122959	0,6279808
CG17286-RA	Fbp1	0,216664	0,0179358	4,11756	14,3916	6,15452	4,24998	-0,280748	0,357144	0,6279808
CG17287-RA	spd-2	1,50678	10,1426	0,06197	37,9074	3,73987	34,9278	-0,129344	0,62591	0,6279808
CG1728-RA	CG17287	0,0974571	0	0,249506	0,121344	1101,26	0,330408	-0,259117	0,355359	0,6279808
CG17290-RA	CG17290	821,994	598,96	1450,41	182,628	11485,7	1150,3	0,274369	0,385029	0,6279808
CG17291-RA	Pp2A-29B	26,434	40,4861	34,5102	38,8964	11,6203	43,8082	-0,186802	0,371884	0,6279808
CG17291-RB	Pp2A-29B	30,6919	0,0373211	41,3778	0,0450227	45,9735	0,0459609	-0,186802	0,371884	0,6279808
CG17292-RA	CG17292	45,8005	13,5969	63,8464	9,6511	0,0609835	14,2128	0,111626	0,662655	0,6279808

gene_id	Symbol	W1_FPKM	W2_FPKM	W3_FPKM	MCM51_FPKM	MCM52_FPKM	MCM53_FPKM	FC	p-value	p-adj
CG17292-RB	CG17292	0,040973	8,65623	0,0393365	0,114292	39,0515	31,1354	0,115242	0,652311	0,6279808
CG17293-RA	Wdr82	13,3203	14,5954	22,9061	19,934	14,3285	19,691	-0,087941	0,728597	0,6279808
CG17294-RA	CG17294	5,5946	6,99562	6,80134	9,77338	5,99746	5,62988	-0,085923	0,716821	0,6279808
CG17295-RA	Rcd4	0,0848214	0	0	0	0	0	0,270086	0,278431	0,6279808
CG17298-RA	CG17298	0,528607	132,949	176,815	155,85	0,724811	0,730715	0,279343	0,351461	0,6279808
CG17299-RA	SNF4Agamma	0,0185676	0,0169126	0,0187147	0,0203337	0,0261769	0,0207574	0,062544	0,811333	0,6279808
CG17299-RB	SNF4Agamma	0,0194933	0,0177559	6,57475	16,1178	0,0275421	5,97433	0,06807	0,79503	0,6279808
CG17299-RC	SNF4Agamma	4,99397	7,9461	0,0256345	0,0283342	7,06427	0,0289246	-0,000576	0,998311	0,6279808
CG17299-RE	SNF4Agamma	0,0267009	0,024321	17,3373	0,0331194	0,0383788	7,21708	-0,003986	0,988293	0,6279808
CG17299-RF	SNF4Agamma	18,1358	28,2827	0,0201664	0,0219894	11,8193	0,0224476	0,024399	0,925186	0,6279808
CG17299-RG	SNF4Agamma	0,0210054	0,0191331	0,019497	0,0212244	0,0297848	0,0216667	0,0925	0,725279	0,6279808
CG17299-RI	SNF4Agamma	0,0203081	0,018498	0,0176279	0,0191017	0,0287486	0,0194997	0,105245	0,68767	0,6279808
CG17299-RJ	SNF4Agamma	0,0183612	0,0167247	12,9898	44,4869	0,0258734	12,992	-0,00501	0,985293	0,6279808
CG17299-RK	SNF4Agamma	14,3248	22,3596	0,0243737	0,026856	17,8526	0,0274156	-0,003107	0,990875	0,6279808
CG17299-RL	SNF4Agamma	0,0253877	0,0231249	8,76856	20,8653	0,0363766	6,13216	0,093376	0,722816	0,6279808
CG17299-RM	SNF4Agamma	7,05539	12,633	10,852	0,034525	0,0345194	10,7255	0,085882	0,743969	0,6279808
CG17299-RN	SNF4Agamma	9,20009	15,9412	3,69849	2,19108	13,3256	3,84616	0,084912	0,746692	0,6279808
CG17300-RA	CG17300	0	0	0	0,133954	0	0,203208	-0,041811	0,731452	0,6279808
CG17301-RA	Prosbeta4R1	0	0	0,206231	19,7463	7,86122	0	0,044335	0,715924	0,6279808
CG17302-RA	Prosbeta4R2	0	0	0,126907	0,192623	141,019	0	0,01562	0,897983	0,6279808
CG17304-RA	btsz	0,309344	0,169063	0,178193	2,61033	0,87012	0,655775	-0,215121	0,499232	0,6279808
CG17319-RA	2mit	2,60077	7,56891	2,62502	4,3615	2,33009	1,95447	0,059784	0,839676	0,6279808
CG17320-RA	Gat	36,1693	26,6656	48,1078	36,5768	6,58618	26,2238	0,01934	0,934526	0,6279808
CG17321-RA	Gat	39,4215	32,1699	16,6988	30,7978	20,2474	26,6245	0,597398	0,003076	0,6279808
CG17322-RA	ScpX	27,123	352,779	7,46586	1,67714	508,73	0,0396253	0,384533	0,214344	0,6279808
CG17322-RB	CG17321	13,9929	19,7228	58,4263	19,0475	70,7297	6,20951	0,387757	0,209523	0,6279808
CG17322-RC	Ugt36E1	0,0347768	3,62449	0,104394	35,626	53,6918	30,2588	0,387757	0,209523	0,6279808
CG17322-RD	Ugt36E1	0,0346673	21,507	0,0333878	12,5562	0,0525771	1,71363	0,38037	0,220997	0,6279808
CG17323-RA	Ugt36E1	0,0349423	1,14829	0,0332827	722,482	0,0523919	461,869	-0,083408	0,707302	0,6279808
CG17324-RA	Ugt36E1	5,62193	15,5329	0,0335467	78,1245	0,0528573	0,116876	-0,41627	0,244811	0,6279808
CG17325-RA	Ugt36D1	23,8295	21,0234	0,0885989	29,1937	7,46129	7,27428	-0,695272	0,010768	0,6279808
CG17325-RB	Ugt36F1	0,157324	0,26869	29,632	0,422648	1,03585	1,24485	-0,69589	0,010709	0,6279808
CG17327-RA	CG17325	191,594	24,8617	35,784	0,182677	0,0207921	0,0156702	-0,173842	0,537311	0,6279808
CG17327-RB	CG17325	29,1323	0	0,0142822	26,0114	5,60796	4,39589	-0,16421	0,558531	0,6279808
CG17328-RA	CG17327	32,1848	14,686	44,9331	0,13244	5,38928	5,75685	-0,041143	0,875044	0,6279808
CG17329-RA	CG17327	0,102179	0,043435	0,0980978	1,92872	4,08138	4,95653	-1,712141	6,08E-07	0,6279808
CG1732-RA	CG17328	0,0470737	0,042878	4,9487	0,0525234	0,0711433	0,0536179	-0,100492	0,687085	0,6279808
CG1732-RB	CG17329	0,546492	0,407277	0,810846	5,47742	2,55592	0,289145	-0,100492	0,687085	0,6279808
CG17330-RA	jhamt	2,25787	0,665378	4,04848	3,05527	3,68145	4,81313	-0,447422	0,206976	0,6279808
CG17331-RA	Prosbeta4	44,5885	41,4266	65,8422	49,9456	68,3719	64,0944	-0,159484	0,56413	0,6279808
CG17332-RA	VhaSFD	104,536	133,945	154,755	133,346	126,185	104,312	-0,010954	0,960079	0,6279808
CG17332-RB	VhaSFD	0,0288961	0,0263206	0,027742	0,0308262	0,0417543	0,0314686	-0,009552	0,96518	0,6279808
CG17332-RD	VhaSFD	0,027353	0,024915	0,0262606	0,0290717	0,0393778	0,0296775	-0,011012	0,959863	0,6279808
CG17333-RA	CG17333	15,2552	10,4833	35,6182	25,1717	0,606743	0,812827	-0,680383	0,044492	0,6279808
CG17334-RA	lin-28	53,5789	100,799	0,158723	521,244	0,00984181	0,2685	0,497555	0,085788	0,6279808
CG17336-RA	Lcch3	9,25711	7,22744	6,866	5,21423	27,9677	4,22366	0,246889	0,208954	0,6279808
CG17336-RB	Lcch3	0,0248717	0,0226549	0,0238783	0,970626	3,25698	0,0541793	0,260304	0,184686	0,6279808
CG17336-RC	Lcch3	4,00352	5,34462	9,2735	6,84402	0,0350343	467,26	0,258532	0,186471	0,6279808
CG17337-RA	Dvir\GJ14978	71,3244	41,5276	3,26412	61,5712	97,694	0,0210959	-0,065498	0,826757	0,6279808
CG17341-RA	CG46301	4,55397	6,68739	5,568	6,321	4,13847	2,73037	-0,246367	0,374303	0,6279808
CG17342-RA	Lk6	8,50004	3,12605	2,95381	1937,15	2,29628	1856,98	-0,056517	0,852198	0,6279808
CG17342-RB	Lk6	38,2665	0,0410362	0,0432523	0,0500119	20,2389	15,4696	-0,091661	0,760644	0,6279808
CG17343-RA	CG17343	17,102	12,7454	23,6333	0,0177629	0,152129	62,307	0,103942	0,703451	0,6279808
CG17344-RA	CG17344	0,123429	40,3805	49,9806	69,6363	17,2712	33,5029	0,121201	0,525951	0,13772387
CG17347-RA	DCTN6-p27	44,4358	0,0478556	0,05044	54,0139	29,1925	71,1667	-0,547556	0,046013	0,13772387
CG17348-RA	drl	33,3049	3,44132	30,888	9,45198	7,31959	0,00970698	0,427716	0,162709	0,6279808
CG17349-RA	CG17349	0	2,46666	0,00961212	6,42691	0,0138362	4,73151	0,093085	0,581996	0,6279808
CG17350-RA	CG17350	0,0510633	0,066139	0,0490238	0,570493	0,0817673	0,0616248	0,884662	0,001784	0,6279808
CG17352-RA	Culd	0,708774	1,18677	0,0200137	2,31426	0,0300354	0,0222692	-0,458253	0,110363	0,6279808
CG17352-RB	Culd	0,340305	0,339033	0,89846	0,0222794	0,0297154	0,732889	-0,458253	0,110363	0,6279808
CG17352-RC	Culd	0,0211736	0,0192864	0,0203279	0,0221744	0,19255	0,0226365	-0,460678	0,108424	0,6279808
CG17352-RD	Culd	0,744034	0,59181	1,37833	2,32006	3,85551	1,17071	-0,458253	0,110363	0,6279808
CG17352-RE	Culd	0,107849	0,147355	0,103542	0,142155	9,91302	0,145117	-0,59283	0,09753	0,6279808
CG17358-RA	Taf12	0,0580801	0,0393976	15,8162	18,5611	6,44766	17,6647	0,291428	0,172695	0,6279808
CG17358-RB	Taf12	23,6787	57,8675	11,752	15,6654	2,87193	6,10899	0,294731	0,168847	0,6279808
CG17358-RD	Taf12	10,7782	32,9213	0,0557603	0	0	0,106038	0,251217	0,231844	0,6279808
CG17359-RA	CG17359	1,85141	1,45538	26,0006	0,645628	2,68646	0,0309189	0,72681	0,014101	0,6279808
CG17360-RA	Prosalpha3T	0	0	0,601325	3,25363	0	0,0425317	-0,031957	0,900767	0,6279808
CG17361-RA	prd1	3,67934	20,5006	16,6079	44,2272	22,8715	22,1966	0,510282	0,091918	0,6279808
CG17362-RA	CG17361	2,79822	3,25065	2,72706	3,86438	0,995601	3,84513	-0,096882	0,751615	0,6279808
CG17364-RA	CG17362	8,12547	0,693866	406,694	1,18193	9,29888	0,0335733	-0,874136	0,009038	0,6279808
CG17364-RB	CG17364	0,825365	0,395013	0,270712	50,1421	1,96908	31,4678	-0,827745	0,018667	0,6279808
CG17367-RA	CG17364	0,025634	0,0233492	34,5625	32,0474	0,445184	1,28999	-0,044945	0,863244	0,6279808
CG17367-RB	Lnk	0,0203959	1,05871	1,02114	0,0213206	0,837142	0,837515	-0,032995	0,8999	0,6279808
CG17367-RC	Lnk	8,12786	2,19075	0,0195813	14,5357	0,0288788	0,743548	-0,047697	0,855041	0,6279808
CG17369-RA	Lnk	8,27144	0	8,13528	11,4043	2,88457	0,0217648	-0,043153	0,833707	0,6279808
CG17369-RB	Vha55	218,385	31,0719	49,5761	0,157817	0,213763	43,2143	-0,043116	0,83385	0,6279808
CG1736-RA	Vha55	0,0247321	0,106211	0,111947	2,50622	2,46829	0,161105	NA	NA	0,6279808
CG17370-RA	CG1737	13,6596	14,6574	5,72242	22,8534	36,7315	1,09597	-0,098703	0,665197	0,6279808
CG17370-RB	SppL	9,82724	0,888361	0,235825	23,4012	3,71385	0,344665	-0,090867	0,689729	0,6279808
CG17370-RC	SppL	0,0322846	7,52796	10,6549	0,0347251	1,38546	0,0517548	-0,072285	0,754244	0,6279808
CG17374-RA	SppL	5,92001	1,32181	1,76672	0,0453637	0,271234	0	-0,328781	0,256281	0,6279808
CG17375-RA	FASN3	7,46891	3,56379	6,04464	6,14058	7,39524	5,99285	0,095338	0,712765	0,6279808
CG17376-RA	CG17375	0,167705	0,0916545	0,193209	0	0	3,28893	0,223924	0,234435	0,6279808
CG17376-RB	CG17376	0,160507	0,657906	5,69234	0	0	0	0,337397	0,179289	0,6279808
CG17377-RA	CG17376	0,738427	0,149469	0,385242	2,95728	0	0	-0,039118	0,754991	0,6279808
CG17377-RB	CG17377	0	2,23298	0,0744853	0	0,186234	0,14751	-0,039118	0,754991	0,6279808
CG17377-RC	CG17377	0	13,712	9,12951	11,7872	0,103901	8,47081	-0,104244	0,537109	0,6279808
CG17378-RA	CG17377	0	8,06104	8,75055	10,5896	0,0986195	5,57416	0,070557	0,813209	0,13772387
CG17378-RB	CG17378	1,0032	1,33179	0,850423	0,975144	39,2183	2,25624	-0,079959	0,790404	0,6279808
CG1737-RA	CG17378	0,0333099	0,030341	0,0319795	0,0359177	59,7004	0,386383	-0,027743	0,909865	0,6279808
CG17380-RB	Dsim\GD17014	15,3761	9,23617	14,0838	13,3683	8,90622	19,4891	0,01562	0,897983	0,13772387
CG17381-RB	CG17380	0,0647803	0,243717	0	2,81607	0,860162	0,068427	0,012227	0,950341	0,6279808
CG17382-RB	Ir94g	0,0605091	0,0895632	0,0944	0,148645	0,154686	0	-0,027338	0,908483	0,6279808
CG17383-RA	Ir94h	0,0983272	0,0918597	1,51392	0,0645997	0,0875007	0	0,223709	0,300676	0,6279808
CG17383-RB	jigr1	0,0430274	16,8578	10,6162	0,165968	1,12402	0,771278	0,250991	0,246072	0,6279808
CG17383-RC	jigr1	0,0413577	14,7466	22,7026	3,12325	0,199045	0,347486	0,22181	0,306032	0,6279808
CG17383-RD	jigr1	0,036736	0,0391924	0,0413089	0,047523	0,0643702	0,0485133	0,221479	0,300758	0,6279808
CG17383-RE	jigr1	9,06654	0,0376714	0,0397059	0,0454889	0,0616151	0,0464368	0,222687	0,299577	0,6279808
CG17383-RF	jigr1	0,0385581	0,0334617	0,0352687	0,039946	3,5062	0,0407783	0,216768	0,314397	0,6279808
CG17385-RA	jigr1	20,8672	12,3206	14,2295	10,7081	5,98984	5,46221	-0,518891	0,03875	0,6279808
CG17386-RA	CG17385	47,2478	52,025	2,32204	0,419655	5,93489	7,17131	-0,944169	0,000589	0,6279808
CG17387-RA	Achl	2,01343	2,71058	61,9589	4,97661	3,79264	0,4284	0,162052	0,636749	0,13772387
CG17388-RA	CG17387	0,529779	2,8644	0,422046	10,4399	2,69472	0,402322	-0,042742	0,807942	0,6279808

gene_id	Symbol	W1_FPKM	W2_FPKM	W3_FPKM	MCM51_FPKM	MCM52_FPKM	MCM53_FPKM	FC	p-value	p-adj
CG17389-RA	Oaz	0,226287	0	0	0,442173	4,32018	88,0235	-0,020689	0,909957	0,6279808
CG1738-RA	Oaz	0	10,5335	0	2,41109	0,82161	173,097	-0,305127	0,312204	0,6279808
CG17390-RB	Oaz	1,22332	1,45772	1,07793	4282,65	1,16129	0,0156666	-0,045037	0,885371	0,6279808
CG17397-RA	MED21	50,4319	17,0126	16,6844	45,3546	9,85668	52,3068	-0,172327	0,506418	0,6279808
CG17397-RB	MED21	22,1395	39,5599	34,3105	40,6311	68,4524	21,6885	-0,172676	0,50547	0,6279808
CG17404-RA	Ntf-2	0,13862	0,126265	0,133084	0,168297	0,272504	14,8014	-0,405407	0,21403	0,6279808
CG1740-RA	Ntf-2	38,4499	35,3741	70,311	43,297	48,6808	47,5194	-0,100617	0,691981	0,6279808
CG1740-RB	CG17404	0	137,354	10,3524	0,0772457	1,01317	1,34189	-0,232681	0,378481	0,6279808
CG17420-RA	Mgstl	6,35499	5,73291	0,11733	0,201183	0,227959	0,171804	-0,620686	0,012594	0,6279808
CG17420-RB	Mgstl	41,1578	33,7502	84,6282	60,0814	50,5851	59,5288	-0,619894	0,012704	0,6279808
CG17420-RC	RpL15	0,0717695	17,6555	4,85348	5,29459	4,50873	4,68856	-0,619894	0,012704	0,6279808
CG17420-RD	RpL15	0,0675852	0,0396572	0,0312189	8,95968	7,1071	7,75785	-0,620033	0,012688	0,6279808
CG17429-RA	RpL15	4582,46	29,5797	0,777605	10,2742	17,3813	11,6932	NA	NA	0,6279808
CG1742-RA	RpL15	0,0792592	9,43665	4,69209	20,0468	16,5451	0,0189932	0,190765	0,517971	0,6279808
CG1742-RB	CG17429	0	0	0	0,0900159	0	0,0918917	0,207489	0,482854	0,6279808
CG17436-RA	Gs2	26,7171	3,59482	4,3377	11,0406	6,48432	0,034897	-0,232067	0,292192	0,13772387
CG17437-RA	Gs2	59,771	7,74834	6,32761	2,9795	5,12337	0,0136039	0,002811	0,991354	0,6279808
CG1743-RA	Gs2	0,0376357	56,8795	39,4562	1,58374	57,3742	3,06096	-0,54524	0,070806	0,13772387
CG1743-RB	vtd	46,9268	39,3497	45,6029	52,1765	50,3975	40,7526	-0,540932	0,073622	0,13772387
CG1743-RC	wds	12,8592	9,29029	18,2208	17,9196	8,81784	12,4019	-0,545117	0,070896	0,13772387
CG17440-RA	chp	0,142463	0,185379	13,8533	5,77519	7,06368	8,57495	-0,215811	0,499198	0,6279808
CG17446-RA	CG17440	0,664336	7,59885	6,33731	7,29169	3,11909	0,021209	0,193199	0,43462	0,6279808
CG1744-RA	Cfp1	4,95901	8,9658	9,26649	11,7452	6,47926	56,6746	0,087834	0,801266	0,6279808
CG17450-RA	HP5	6,49532	1,36787	2,6432	3,65563	0,0463034	0,954959	0,320908	0,368022	0,13772387
CG17450-RB	HP5	0,0393855	0,756783	1,2434	1,03856	0,0180504	3,29253	0,188812	0,586801	0,13772387
CG17453-RA	CG17450	0,577361	0,473311	0,739069	0,52082	0,915967	0,659986	-0,087767	0,76157	0,13772387
CG17454-RA	CG17450	1,1085	1,37832	1,75681	1,38157	2,10337	1,98467	-0,123573	0,614326	0,6279808
CG1745-RA	Cyp317a1	6,21716	4,52268	8,65788	6,21451	22,9098	5,35983	0,17533	0,568674	0,6279808
CG1745-RB	CG17454	110,101	96,929	80,3798	123,397	121,844	97,561	0,296869	0,253822	0,6279808
CG17461-RA	Dpse\GA14517	945,612	924,349	0,214193	0,449525	0	0,202328	0,212687	0,456268	0,6279808
CG17462-RA	Dpse\GA14517	0,0378947	0,0345172	1,96391	5,03216	1,67959	1,2675	0,258538	0,461635	0,6279808
CG17469-RA	Dpse\GA14517	0,0474115	0,0431857	1,5879	4,68412	1,37412	1,97757	0,18075	0,460646	0,6279808
CG17469-RB	Kif3C	0,905977	23,7211	45,5813	0,0401651	0,0316321	268,356	0,050743	0,836757	0,6279808
CG1746-RA	Trf4-2	0,404981	3,38635	0,213316	12,119	0,0381981	4,56379	-0,086736	0,719717	0,6279808
CG1746-RB	Mitf	0,023228	17,915	2,88788	7,50307	10,3614	3,033	-0,086768	0,719617	0,6279808
CG1746-RC	Mitf	22,7617	1,56039	15,2817	1,99905	9,42251	10,7875	-0,086859	0,71929	0,6279808
CG17470-RA	Sk1	0,967656	0,0342812	2,44019	39,0054	0,0198136	0,0282682	-0,420018	0,225587	0,6279808
CG17471-RA	Sk1	6,7888	30,9875	5,58656	0,0467796	0,0193203	0,0346811	-0,140164	0,59164	0,6279808
CG17471-RB	CG17470	0,175361	0,163907	11,5902	11,9702	0,0455472	11,2552	-0,140427	0,59093	0,6279808
CG17472-RA	PIP4K	0,0460565	52,4856	0,0190692	30,8746	35,3878	29,8007	0,01562	0,897983	0,6279808
CG17475-RA	PIP4K	64,2784	23,3472	37,8901	15,0708	0,0381654	29,1818	-1,03556	0,003714	0,6279808
CG17477-RA	CG17472	0,184914	0,0285424	0,0300838	0,0336264	0	0,0343271	-0,399998	0,236165	0,6279808
CG1747-RA	CG17475	13,4124	3,62317	3,21585	7,76371	1,45207	46,0969	0,184375	0,444887	0,6279808
CG1747-RB	CG17477	0,31058	0,256933	1,11816	6,72897	52,179	1,25222	0,18896	0,432521	0,6279808
CG17484-RA	p120ctn	122,652	103,167	15,8359	31,4702	0,0323898	10,1694	-0,155194	0,553024	0,6279808
CG17484-RB	p120ctn	0,0227482	0,0207206	6,80772	22,3251	0,283347	12,0303	-0,156053	0,55094	0,6279808
CG17486-RB	CG17486	31,0795	28,1426	0	0,0239126	123,878	19,143	0,177957	0,384111	0,6279808
CG17489-RA	RpL5	474,276	461,155	0,117548	0,168728	0,228543	0,172244	-0,821074	0,001492	0,6279808
CG17489-RB	RpL5	0,0752857	0,0685754	23,2279	29,7865	33,5403	25,5134	-0,821072	0,001492	0,6279808
CG17489-RC	RpL5	2349,69	2892,72	38,1148	37,9084	20,8027	23,4729	-0,821102	0,001491	0,13772387
CG17489-RD	RpL5	0,0644223	0,0586802	84,8831	83,4488	0	0,546342	-0,811089	0,001671	0,6279808
CG17489-RE	RpL5	0,0741036	0,0674987	0,0218396	0,0464183	83,2027	49,6471	-0,811087	0,001671	0,6279808
CG17490-RA	Uba5	32,7183	1,62179	0,0245689	30,0827	0,0370101	4,837	0,299321	0,201711	0,6279808
CG17490-RB	CG17490	0,0510764	0,0465239	0,0490364	0,0575714	54,4211	0,0587711	0,152506	0,507427	0,6279808
CG17490-RC	CG17490	4,31385	5,30736	5,75216	7,49143	0,0779808	3,25616	0,232972	0,350598	0,6279808
CG17490-RD	CG17490	0,0518377	0,0472174	0,0497673	0,0585433	3,63227	0,0597632	0,278597	0,26509	0,6279808
CG17492-RA	CG17490	6,08378	5,37445	4,54936	3,97907	0,0792972	3,92358	0,086068	0,732208	0,6279808
CG17493-RA	mib2	13,1016	16,0212	15,2512	11,7653	7,82946	10,3581	0,135104	0,593107	0,6279808
CG17494-RA	CG17493	73,2043	72,4891	47,7789	70,4526	3,86938	46,0454	0,035594	0,851077	0,6279808
CG17494-RB	Dpse\GA29082	4,04725	0,0177663	5,46793	11,7959	4,47487	4,04805	0,034197	0,856898	0,6279808
CG17494-RC	Dpse\GA29082	0,0198924	0,0181194	0,0190979	5,06396	0,0281324	0,0212023	0,035592	0,851136	0,6279808
CG17498-RA	Dpse\GA29082	23,7958	23,8653	22,8357	0,0207695	16,7544	17,5067	0,112935	0,688831	0,6279808
CG1749-RA	mad2	0,0872095	3,73351	0,112278	7,24847	0,0231428	0,0239077	-0,112704	0,583459	0,6279808
CG17508-RA	CG1750	6,8219	12,3719	6,07864	0,134319	0,0971157	9,57353	-0,10931	0,693103	0,6279808
CG17509-RA	CG1750	0,064172	24,5568	0,108998	4,84263	1,62105	0,0636365	-0,007509	0,971981	0,6279808
CG1750-RA	CG17508	102,918	91,2247	60,883	94,8239	0,212239	0,159956	-0,056202	0,848452	0,6279808
CG1750-RB	pds5	5,31031	0,106776	0,0594719	0,158959	0,215311	0,162271	0,026853	0,927271	0,6279808
CG17510-RA	Spase25	72,0461	9,20713	0,0296157	93,9302	6,56608	0,0149327	-0,319971	0,254413	0,6279808
CG17510-RC	Fis1	134,078	115,494	84,8349	171,858	11,7497	12,7134	-0,322525	0,254	0,6279808
CG17510-RD	Fis1	0,103459	0,0942374	0,0993266	0,134604	12,6735	28,3025	-0,292141	0,297457	0,6279808
CG17510-RE	Fis1	32,1339	26,5199	18,078	47,2756	32,1393	10,5278	-0,296277	0,294552	0,6279808
CG17510-RF	Fis1	36,0233	41,9304	24,089	0,186916	62,8255	16,5039	-0,296455	0,29426	0,6279808
CG17514-RA	Fis1	0,11599	0,105652	0,111357	0,156691	14,7946	79,2338	0,082898	0,694018	0,6279808
CG17514-RB	CG17514	26,1064	20,6975	23,2046	0	18,5881	15,7675	-0,048349	0,867902	0,6279808
CG17514-RC	CG17514	0,153426	0,139751	0,147298	24,5657	0,317031	0,238933	-0,068352	0,812105	0,6279808
CG17515-RA	CG17514	0,156701	0,142734	0,150442	0,234056	0,327501	0,246824	0,042837	0,850761	0,6279808
CG17515-RC	Rab21	0,0645481	0,0587949	0,06197	0,075346	0,102057	0,0769161	0,041605	0,854788	0,13772387
CG1751-RA	Rab21	9,95407	12,4781	12,9943	12,6803	11,7467	10,9249	0,060111	0,829324	0,6279808
CG17520-RA	CkIIalpha	0,0421491	3,43102	73,3104	20,5854	19,394	21,0806	-0,127987	0,605784	0,6279808
CG17520-RB	CkIIalpha	89,1908	0,0383924	48,4605	0,046451	0,0629181	0,0474189	-0,12793	0,605952	0,6279808
CG17520-RC	CkIIalpha	56,5295	84,2259	3,26994	83,4235	85,9483	70,9604	-0,126343	0,610011	0,6279808
CG17521-RC	RpL10	0,0895739	12,2427	2,86117	3,74594	4290,73	2902,67	-0,147323	0,542113	0,6279808
CG17521-RD	RpL10	3021,29	421,592	282,445	516,116	0,363516	504,641	-0,14733	0,542097	0,6279808
CG17522-RA	GstE10	12,9776	38,4858	16,5467	17,8922	20,028	13,2029	-0,364179	0,281605	0,6279808
CG17523-RA	GstE2	13,0744	27,174	73,4271	15,1912	24,9597	20,7019	-0,302243	0,295509	0,6279808
CG17524-RA	GstE3	39,7508	16,3286	17,6371	9,09481	143,013	133,365	-0,45939	0,154567	0,6279808
CG17525-RA	GstE4	9,33359	6,6574	16,4993	100,115	39,6426	30,6343	-0,131972	0,688167	0,6279808
CG17527-RA	GstE5	7,21007	6,29754	120,176	158,093	116,434	111,522	-1,053768	0,001843	0,6279808
CG17528-RA	CG17528	0,023509	0,768405	0,0278639	7,9903	0,0295383	0,0351707	-0,172377	0,417469	0,6279808
CG17528-RB	CG17528	0,0231954	6,77644	0,0307696	98,9295	0,0288602	8,02295	-0,172633	0,416896	0,6279808
CG17528-RC	CG17528	10,0881	0,0214136	7,01159	0,0247572	36,3645	0,025273	-0,165766	0,435839	0,6279808
CG17528-RD	CG17528	25,4624	0,0211279	3,88204	0,0244087	21,3786	0,0249173	-0,172633	0,416896	0,6279808
CG17530-RA	Cbs	22,112	17,8296	40,9073	29,4881	27,9852	34,2928	-0,418806	0,078352	0,6279808
CG17531-RA	Cbs	0,034048	4,82256	0,0326881	0,0367797	0,0498184	0,0375462	-1,244281	2,81E-06	0,6279808
CG17533-RA	GstE6	117,19	13,9673	171,102	215,564	29,8498	243,596	-0,778825	0,002993	0,6279808
CG17534-RA	GstE7	39,7857	145,231	14,9893	23,9584	169,477	25,2903	-0,81132	0,018194	0,6279808
CG1753-RA	GstE8	10,8268	61,1843	44,675	148,794	220,759	118,061	-0,206224	0,440577	0,6279808
CG1753-RB	GstE9	11,293	11,8549	37,0625	33,1933	63,1346	55,8104	-0,203365	0,446196	0,6279808
CG17540-RA	Dpse\GA29081	42,9254	36,2171	40,5505	46,7938	45,5165	44,2037	-0,149593	0,499256	0,6279808
CG17540-RB	Dpse\GA29081	11,1684	7,68478	11,3258	11,5604	10,5276	7,45947	-0,149068	0,500974	0,6279808
CG17540-RC	Dpse\GA29081	0,0468733	0,0426955	0,0450012	0,0522734	0,0708046	0,0533626	-0,151704	0,493615	0,6279808
CG17544-RA	CG17544	3,52216	4,06595	4,74986	5,91967	0,125923	6,96105	-0,398047	0,062458	0,6279808
CG17544-RB	CG17544	1,69549	6,32211	0,0252367	2,6795	14,51	0,0284475	-0,390883	0,066731	0,6279808
CG17544-RC	CG17544	1,11111	1,1998	2,36205	0,136949	26,4198	0,0286413	-0,398047	0,062458	0,6279808

gene_id	Symbol	W1_FPKM	W2_FPKM	W3_FPKM	MCM51_FPKM	MCM52_FPKM	MCM53_FPKM	FC	p-value	p-adj
CG17549-RA	CG17549	8,68317	0,0440066	22,1563	7,19746	0,0394649	12,893	-0,381757	0,24177	0,6279808
CG17549-RB	CG17549	11,5271	6,60069	21,7348	15,4809	1,88706	21,2875	-0,379292	0,245734	0,6279808
CG17549-RC	CG17549	31,3414	12,4189	59,6226	8,67411	4,81328	49,1622	-0,379292	0,245734	0,6279808
CG17556-RA	CG17556	29,5771	0,134872	0,0423853	3,96942	12,3759	0,0499174	0,504156	0,037835	0,6279808
CG17559-RA	dnt	6,7609	0,161937	97,8177	0,108313	105,613	0,242347	0,08833	0,771388	0,6279808
CG17560-RA	CG43155	0,708654	0,0358751	0,0378125	0,0431081	3,54131	0,0440064	-0,33224	0,2843	0,6279808
CG17562-RA	CG17560	1,85012	1,18367	0,159289	43,1587	3,28311	3,84227	-0,009115	0,975672	0,6279808
CG17564-RB	CG17562	2,29211	1,81783	3,39569	3,52009	5,44986	0,0540921	-0,180254	0,522277	0,6279808
CG17565-RA	CG17564	3,5637	44,0311	0,253973	45,1443	2,65592	3,27428	-0,086736	0,722984	0,6279808
CG17566-RA	CG17565	8,08331	12,4376	11,8596	0,0754323	10,6245	12,7063	-0,008318	0,980702	0,6279808
CG17567-RA	gammaTub37C	0,239563	5,58275	0,825836	0	3,90869	0,0104278	0,242108	0,322225	0,6279808
CG17567-RB	CG46059	0	0,43529	0,173008	7,38268	0	80,9647	0,242108	0,322225	0,6279808
CG17567-RC	CG46059	0	0,168274	2,88588	0,0662114	0	76,6825	0,242108	0,322225	0,6279808
CG17567-RD	CG46059	0	0,164144	3,48406	0,439441	0	0,0104278	0,242108	0,322225	0,6279808
CG17568-RA	CG46059	0	7,92672	6,704	3,5581	0,234011	0,0104278	0,402109	0,09354	0,6279808
CG17569-RA	CG17568	5,10211	3,76808	0,171142	0	5,13044	0,0104375	0,183239	0,422644	0,6279808
CG17569-RB	gry	0,0141743	0,0129109	0,0136082	0,014602	0,0197785	0,0149062	0,183239	0,422644	0,6279808
CG1756-RA	gry	0,0143404	0,0130622	0,0137677	14,0561	0,020018	0,0150868	-0,168866	0,607408	0,6279808
CG17570-RA	CG17570	7,66807	0	0	13,2425	0	25,1973	-0,808097	0,020099	0,6279808
CG17571-RA	CG17571	9,07913	4,60687	0	0	33,353	0	-0,109988	0,710929	0,6279808
CG17572-RA	CG17572	1,49703	1,34009	2,94882	2,2899	53,8551	22,4393	0,384546	0,232398	0,6279808
CG17574-RA	CG17574	0,057225	0,0521245	0,0549394	0,0365148	0,0494595	0,0372757	-0,156813	0,500102	0,6279808
CG17574-RB	CG17574	0,057225	0,0521245	0,0549394	937,109	1142,97	1235,64	-0,069898	0,774566	0,6279808
CG17574-RC	CG17574	14,4287	14,0596	26,2552	0,0948736	0,128507	0,0968505	-0,17737	0,477206	0,6279808
CG17574-RD	CG17574	20,0254	16,9105	24,9474	0,0981231	0,132908	0,100168	-0,077691	0,748261	0,6279808
CG17575-RA	Dsim\GD25810	0,282376	1,62353	0	0,0246737	0	0,293633	0,163497	0,319224	0,6279808
CG17577-RA	Cyp9h1	1,59142	0	5,9032	0	0,304361	2,626	-0,789899	0,019258	0,6279808
CG17579-RA	sca	0,0234339	0,0213452	0	6,7197	0	0,105016	-0,244065	0,436367	0,6279808
CG17579-RB	sca	3,48961	3,90281	0,0316078	0,0655299	0,0887607	0,0668954	0,009116	0,973271	0,6279808
CG17580-RA	CG17580	0	0	0,022498	2,17103	3,15061	0,0251879	-0,041811	0,731452	0,6279808
CG17584-RA	Or49b	0	0	0,313354	0	5,67753	3,84806	-0,013096	0,914398	0,6279808
CG17592-RA	cad	12,1263	14,8534	3,93624	8,93786	2,35991	2,9852	0,193449	0,423077	0,6279808
CG17592-RB	cad	0,0303983	0,0276889	8,90039	4,59946	0,0359227	7,59348	0,182036	0,449423	0,6279808
CG17593-RA	Usf	0,898015	0,784359	4,38902	3,27535	0,0351863	3,49949	-0,257825	0,383484	0,6279808
CG17594-RA	Usf	3,59198	2,71944	3,18259	0,20027	2,42567	0,320206	0,188559	0,411326	0,6279808
CG17594-RB	CG17593	51,5785	0,936563	1,32485	1,40592	0,125199	0,420775	-0,067001	0,793718	0,6279808
CG17594-RC	scro	0,036312	0,0330755	0,0348617	4,89997	0,0534269	0,0402657	0,185581	0,419828	0,6279808
CG17594-RD	scro	0,0653138	0,0594923	0,0627051	10,1362	0,103477	0,0779862	0,174737	0,474737	0,6279808
CG17596-RA	scro	3,52181	3,31789	3,3425	0	3,45674	2,42216	-0,156732	0,533218	0,6279808
CG17597-RA	scro	8,98254	10,1491	8,12836	0,471613	5,8082	5,74291	-0,548765	0,096908	0,6279808
CG17598-RA	S6kII	3,4495	4,1734	3,99429	6,5845	2,81162	0,0296644	-0,006398	0,978273	0,6279808
CG17599-RA	CG17597	27,618	0,0135504	7,88579	0,0153503	0,0555207	61,4528	0,291227	0,343497	0,6279808
CG1759-RA	CG17598	13,8497	16,01	14,5658	21,6455	9,56034	0,0263007	0,364942	0,076737	0,6279808
CG1759-RB	Cluap1	1,78359	2,75808	0,09912	2,9484	0,968076	1,46945	0,346334	0,090609	0,6279808
CG17600-RA	CG17600	9,00288	10,8425	10,7056	17,7907	6,03785	0,030848	0,219001	0,398119	0,6279808
CG17600-RB	CG17600	17,2297	22,5855	18,9848	15,2197	7,03038	0,0277779	0,461678	0,067794	0,6279808
CG17601-RA	CG17601	0	0,0940414	2,44907	0	0	0	0,044335	0,715924	0,13772387
CG17603-RA	Taf1	2,91586	3,07869	1,52063	1,28374	1,77253	0,917573	-0,173532	0,5154	0,13772387
CG17603-RB	Taf1	0,640477	0,627949	0,763685	3,37023	3,56417	1,79588	-0,176135	0,508896	0,13772387
CG17603-RC	Taf1	1,54935	10,2473	6,73332	7,99951	0,512808	4,30885	-0,176877	0,506199	0,13772387
CG17604-RA	c(3)G	0,0251941	0,0229486	0,959514	0,0230752	135,881	4,72698	0,369183	0,130045	0,6279808
CG17604-RB	c(3)G	0,0257639	0,0234675	1,24386	0,0190135	0,780857	0,880578	0,373921	0,127217	0,6279808
CG17604-RC	c(3)G	2,79488	2,48629	155,708	0,0289313	0,542439	27,4613	0,369183	0,130045	0,6279808
CG17608-RA	Agpat2	6,96793	6,79022	0,231184	121,688	2,73373	2,2102	-0,546967	0,058045	0,6279808
CG17608-RB	Agpat2	8,74595	7,29455	4,57453	38,3136	12,1619	12,2912	-0,539585	0,060462	0,6279808
CG17610-RA	grk	15,4613	0,149811	0,226983	3,3901	0,0282303	0,105784	0,608173	0,013704	0,6279808
CG17611-RA	eIF6	16,6444	7,78274	9,87581	20,0718	7,27247	10,5997	-0,300219	0,377447	0,6279808
CG17612-RA	CG17612	3,97902	0,0286511	6,97583	0	2,52115	20,1451	0,460698	0,056632	0,6279808
CG17612-RB	CG17612	0,03138	0,0285831	0,0301267	22,2135	0,0424292	0,0339118	0,460698	0,056632	0,6279808
CG17618-RB	CG45049	13,2219	1,04153	10,2708	0,811618	0,584203	1,49441	0,294375	0,141288	0,6279808
CG17622-RB	Itgbn	13,1482	4,50655	10,6769	9,60173	81,4443	65,3638	-0,301035	0,391039	0,13772387
CG17623-RA	wake	0,224557	4,37082	4,6968	5,81292	0,092726	3,05161	-0,18038	0,589538	0,6279808
CG17625-RA	wake	0,351467	0,0184867	0,019171	0,0212108	9,49701	0,0216527	-0,197151	0,497477	0,6279808
CG17626-RA	CG17625	0,176046	11,2378	0,281691	0,441602	29,525	0	-0,039573	0,866045	0,6279808
CG17626-RB	CG17626	0,400728	0,12167	0,115761	0,190703	0,162866	0,372652	0,039214	0,901637	0,13772387
CG17629-RA	CG17626	0,0982295	0,357897	0,377225	0,543706	0,32867	0,248052	0,01562	0,897983	0,6279808
CG1762-RA	kl-3	0	0	0	0,00807	0	0,0204358	0,434532	0,097599	0,6279808
CG17631-RA	nod	1,07061	0,997283	1,95404	2,34631	2,79907	1,68881	NA	NA	0,6279808
CG17632-RA	CG17631	0	0	0	0	0,0209581	3,82504	0,071065	0,818502	0,6279808
CG17632-RB	bw	7,4619	12,6612	6,40604	15,4681	51,5821	6,68669	-0,311532	0,339667	0,6279808
CG17633-RA	bw	0,0549411	0,0500441	0,0527467	0,0625443	22,3924	0,0638475	-0,210227	0,533418	0,6279808
CG17636-RA	CG17633	11,2167	3,80907	1,5641	8,0577	0,082295	2,74926	-0,510638	0,139731	0,6279808
CG17637-RA	CG17636	7,70575	3,96722	13,42	6,12963	17,4614	15,3347	0,065382	0,683415	0,6279808
CG17639-RA	CG17637	0,130843	0,0993176	0,114301	0,121298	8,49763	0,0640606	-0,117382	0,700551	0,6279808
CG17639-RB	GstD11	1,22028	1,1324	0,0578978	16,9087	17,0552	0,121084	-0,047718	0,878208	0,6279808
CG1763-RA	GstD11	1,58616	13,2173	392,107	19,3911	1,53162	0,0710652	-0,414033	0,16171	0,6279808
CG17642-RA	CG1764	19,7105	25,5995	18,1152	0,0180105	15,4529	16,3075	-0,286913	0,342162	0,6279808
CG17645-RA	mRpL48	21,4348	4,2934	40,4225	6,14219	38,2746	44,7357	-0,404378	0,138108	0,6279808
CG17646-RA	Pglym87	0	0	0	0,315872	0,685617	7,84383	0,035731	0,892511	0,6279808
CG17646-RB	CG17646	7,62931	0,0761854	0,135549	0,100596	0,136258	0	0,065217	0,807203	0,6279808
CG17648-RA	CG17646	10,6179	0	0	0	3,15188	0	0,01562	0,897983	0,6279808
CG1764-RA	CG17648	0,197045	0	0	0	0	0	0,231699	0,324683	0,13772387
CG17650-RA	EcR	7,08266	3,02296	1,88921	3,87347	3,59191	2,81118	-0,382888	0,252587	0,6279808
CG17652-RA	EcR	5,33846	4,43729	4,24237	0,014111	3,004	2,99509	-0,475377	0,124751	0,6279808
CG17654-RA	EcR	0,0138446	0,0717645	2,60517	3,81097	0,204303	1,60042	-0,525481	0,048923	0,6279808
CG17654-RB	EcR	0,0137125	10,5238	0,0131648	0,567691	1,3855	0,0144051	-0,525417	0,048895	0,6279808
CG17654-RC	EcR	3,69775	6,85882	3,77321	9,28816	3,55946	1,25294	-0,525765	0,048741	0,6279808
CG17654-RD	CG17650	3,43747	3,59495	18,3213	0	0	0	-0,525705	0,048762	0,6279808
CG17654-RE	CG17652	12,6758	9,17127	33,8573	16,0643	25,9461	33,1735	-0,52069	0,051163	0,6279808
CG17657-RA	Eno	179,325	0,0266784	0	0,349842	0	0	-0,153575	0,481421	0,6279808
CG17658-RA	Eno	0,0306901	0,0256149	0,142235	0	0	0,216276	0,059948	0,852445	0,6279808
CG1765-RA	Eno	0,029289	0,0276212	1,24604	1,41084	0,570579	0,654023	0,479682	0,088661	0,6279808
CG1765-RB	Eno	0,0281214	0,0813695	4,62951	247,908	1,84992	59,787	0,422596	0,137876	0,6279808
CG1765-RC	Eno	0,0303239	0,0106362	3,0562	0,0337816	8,23729	9,48259	0,461861	0,10414	0,6279808
CG1765-RD	frtz	3,6372	30,6694	3,21578	27,4719	3,56006	3,65159	0,475833	0,091467	0,6279808
CG1765-RE	CG17658	0,0364523	2,76745	7,32407	6,24304	5,29827	0,0404351	0,475578	0,091669	0,6279808
CG17660-RA	CG17660	12,7318	14,4642	13,2486	42,4746	8,66372	9,63188	0,188995	0,354513	0,6279808
CG17662-RA	Eglp3	1,24143	0,401243	0,101445	1,27016	0,0426522	3,72066	-0,610777	0,087284	0,6279808
CG17664-RA	Eglp2	0,0732818	0,0667501	0,0703549	0,0875613	0,118602	0,0893859	-0,084798	0,789971	0,6279808
CG17664-RB	Eglp2	11,5308	14,6173	22,5989	10,655	21,3175	20,31	-0,084798	0,789971	0,6279808
CG17665-RA	IntS3	12,2105	7,26801	311,288	575,736	9,63753	8,86595	0,188867	0,366883	0,6279808
CG17665-RB	IntS3	3,66459	6,26697	12,9632	16,0799	0,0346804	1,84309	0,255241	0,210257	0,13772387
CG17666-RA	CG17666	0,095359	0,0760021	0,057219	0,0586541	0	0,0613254	0,204886	0,49634	0,6279808
CG17666-RB	CG17666	0,309193	0,125171	0,131931	0,106864	0	0,270545	0,17087	0,531862	0,13772387

gene_id	Symbol	W1_FPKM	W2_FPKM	W3_FPKM	MCM51_FPKM	MCM52_FPKM	MCM53_FPKM	FC	p-value	p-adj
CG17667-RA	CG42709	15,7998	4,37977	5,77037	0,0277028	2,28537	2,81333	0,25406	0,309775	0,6279808
CG17667-RB	CG42709	0,026141	17,2343	13,2535	6,23719	11,1901	10,1561	0,240816	0,337349	0,6279808
CG17669-RA	CG17669	0,958472	30,4493	24,2621	21,4689	6,88672	18,1037	-1,13079	6,82E-05	0,6279808
CG17672-RA	CG17672	37,0333	49,5453	41,0051	60,7385	18,4971	29,4463	0,25637	0,365806	0,6279808
CG17673-RA	SP	0	0,185091	0,154724	0,342948	0,135056	0,215725	NA	NA	0,13772387
CG17678-RA	cta	18,7139	16,9352	0,0470366	0,0549317	14,1312	5,41758	-0,005534	0,979165	0,6279808
CG17680-RA	dia	2,255	2,582	11,9893	7,65044	3,5514	9,03962	0,069199	0,784992	0,6279808
CG17681-RA	dia	11,5013	11,2832	2,01371	8,35992	6,25727	0,033225	-0,306107	0,392364	0,6279808
CG17683-RA	EMRE	40,6584	36,7758	54,2575	42,2892	4,29235	49,0896	0,189758	0,332986	0,6279808
CG17683-RB	CG17681	0,299664	0,409432	0,176606	6,38674	0,192483	0,81452	0,202073	0,304622	0,6279808
CG17683-RC	CG17683	0,0364523	0,0332032	0,0349964	0,0396098	0,0536517	0,0404351	0,189758	0,332986	0,6279808
CG17683-RD	CG17683	0,0366952	0,0334245	0,0352296	0,0398976	0,0540415	0,040729	0,189899	0,332778	0,6279808
CG17683-RE	CG17683	7,74836	3,86771	8,95454	4,41831	6,19077	4,93307	0,189899	0,332778	0,13772387
CG17684-RC	CG17683	0,0365531	4,04535	0,0350932	10,7651	5,7734	4,70411	0,820004	0,004892	0,6279808
CG17686-RB	CG17683	28,8114	23,1361	32,4549	22,6375	13,8609	18,6332	-0,162926	0,465801	0,6279808
CG17686-RC	CG17684	1,37798	1,50007	2,67816	0,881612	0	0,813488	-0,059019	0,788438	0,6279808
CG17686-RD	DIP1	9,50549	7,94805	11,9793	2,54543	8,05172	1,11058	-0,064809	0,768132	0,6279808
CG17686-RE	DIP1	4,63271	1,44585	1,34312	5,43122	0,824801	8,28242	-0,16301	0,465758	0,6279808
CG17686-RF	DIP1	12,5944	9,78383	5,93108	13,982	7,22903	7,15393	-0,155921	0,48537	0,6279808
CG17687-RA	DIP1	15,9212	4,96821	12,0279	21,2751	10,376	12,4405	-0,195648	0,554699	0,6279808
CG17689-RA	DIP1	5,1962	13,2423	0,0167837	12,9403	10,6624	11,2392	-0,013209	0,961345	0,6279808
CG17689-RB	CG17687	0,217217	2,11181	0	36,6998	0	0	0,021607	0,937061	0,6279808
CG1768-RA	Spt20	0,692811	18,4647	13,2929	17,5494	14,1199	12,5654	-0,076547	0,708192	0,6279808
CG1768-RB	Spt20	1,55001	1,41445	1,06652	11,3702	0,232994	8,68422	-0,056478	0,782448	0,6279808
CG17691-RA	CG17691	0,054578	0,0497134	0,0501873	54,5878	0,0840781	0,0633664	-0,263551	0,351384	0,6279808
CG17691-RB	CG17691	15,9905	24,9069	18,74	28,6187	18,0388	30,956	-0,476367	0,115213	0,6279808
CG17691-RC	CG17691	0,152717	0,139105	0,146617	17,6523	0,314794	0,237248	-0,263551	0,351384	0,6279808
CG17691-RD	CG17691	54,9218	41,185	95,4471	80,4571	94,2778	75,9067	-0,221479	0,430424	0,6279808
CG17697-RA	fz	0,0178022	0,0162155	0,0170912	0,0184958	0,0250527	0,0188812	0,359799	0,146574	0,6279808
CG17697-RB	fz	18,0881	20,9569	15,992	20,3117	9,08607	10,2534	0,110515	0,68457	0,6279808
CG17698-RA	CG17698	19,7278	0	1059,12	26,7724	29,0552	11,4686	-0,54902	0,047149	0,6279808
CG17698-RB	CG17698	8,65428	3,41347	18,3803	16,5465	28,4203	2,19857	-0,60432	0,032528	0,6279808
CG17698-RC	CG17698	14,323	24,418	7,42996	8,36212	15,266	0	-0,598574	0,034734	0,6279808
CG17704-RE	HDAC4	8,10036	0,0244693	0,0257908	1,74468	0,038628	7,05991	0,103165	0,682572	0,6279808
CG17704-RF	HDAC4	3,93152	21,8434	23,0057	15,0998	11,4051	2,22108	0,103165	0,682572	0,6279808
CG17706-RA	HDAC4	2,86215	8,83198	8,76478	8,27788	7,34305	4,96609	0,020909	0,891561	0,6279808
CG17707-RA	Nipped-B	21,712	20,3709	0,247929	0,0346703	133,493	0,0244109	0,211479	0,488077	0,6279808
CG1770-RA	Nipped-B	9,18237	9,80631	6,1824	40,7218	0,0335337	0,0353928	-0,269497	0,192993	0,6279808
CG1770-RB	CR40282	0	0	78,2674	4,53666	5,34837	78,7128	-0,252493	0,224462	0,6279808
CG1770-RC	CG17707	3,7858	4,3904	3,47062	6,23318	1,87155	2,11446	-0,262239	0,20746	0,13772387
CG17712-RA	mew	25,6369	14,8026	21,0884	0,107666	12,3445	15,059	-0,063543	0,760577	0,6279808
CG17715-RA	mew	0,0173534	32,0235	21,2346	0,0974729	15,4217	16,1673	-0,190331	0,436337	0,6279808
CG17715-RB	CG17712	13,0997	15,0677	2,62792	4,26284	0,222885	0	-0,19072	0,435391	0,6279808
CG17715-RC	CG17715	0,0408969	0,0372518	0,0388071	5,4057	2,78749	12,7076	-0,183725	0,454539	0,6279808
CG17715-RD	CG17715	0,0442077	0,0402674	0,045616	0,0530734	0,0608588	0,0244909	-0,19072	0,435391	0,6279808
CG17715-RE	CG17715	0,0489934	0,0446266	30,1328	39,6362	0,0663317	3,68167	-0,190331	0,436337	0,6279808
CG17715-RF	CG17715	0,0404216	0,0368188	9,42169	15,2496	0,0744053	14,4512	-0,183725	0,454539	0,6279808
CG17716-RA	CG17715	0,0475138	0,0432788	2,3488	4,53882	0,0600804	0,0458669	0,451813	0,097244	0,6279808
CG17717-RA	CG17715	40,7717	40,1795	16,5373	22,3349	0,0718882	0,0499916	0,531301	0,136284	0,6279808
CG17717-RB	CG17716	8,13721	26,0497	7,28392	0,0519781	0,0704047	3,69628	0,531301	0,136284	0,6279808
CG1771-RA	CG17717	0,0635548	0,0578901	0,0610164	0,320337	0,100223	0,0755341	-0,077841	0,757773	0,6279808
CG1771-RB	CG17717	0,711419	0,382915	0,589868	0,0755188	0,364486	0,333027	0,212456	0,399041	0,6279808
CG17721-RA	dap	18,9026	14,0543	9,49479	0,0286545	0,0243686	3,18316	0,068495	0,834813	0,6279808
CG17723-RA	dap	16,4946	0,281772	19,4528	36,3182	0	21,4846	-0,13181	0,553378	0,6279808
CG17723-RB	CG17721	2,96782	2,7543	6,66624	4,58827	64,6272	0,410228	-0,132906	0,550071	0,6279808
CG17723-RC	ZnT63C	0,0346733	0,68706	0	7,87728	5,94247	12,7488	-0,135294	0,542908	0,6279808
CG17723-RD	ZnT63C	30,7791	0,0315829	0,0332885	0,0375126	0,0508111	1056,03	-0,13783	0,535811	0,6279808
CG17723-RE	ZnT63C	0,0308958	41,0376	52,6421	70,4692	45,2365	0	-0,125737	0,573397	0,6279808
CG17724-RA	ZnT63C	0,0276391	0,028142	0,0296618	0,0331194	0,0448604	0	0,446849	0,156715	0,6279808
CG17725-RA	ZnT63C	15,004	0,0251756	0,0265352	0,029396	0,039817	0,510374	-0,356572	0,140465	0,6279808
CG17725-RB	CG17724	37,3458	0,0581137	1,66858	19,1603	0,154963	14,9803	-0,356846	0,140164	0,6279808
CG17726-RA	Pepck1	0,025417	0,0231516	0,0244019	2,88446	0,0364211	0,0274491	-0,153328	0,557252	0,6279808
CG1772-RA	Pepck1	94,9224	140,392	192,863	31,4233	158,087	137,727	0,239012	0,345716	0,6279808
CG1772-RB	CG17726	4,26371	4,79624	7,49342	5,90586	3,75864	6,62135	0,239012	0,345716	0,6279808
CG17732-RA	CG1773	1,45319	1,51277	3,50212	2,24738	0,141551	2,64069	0,02507	0,944058	0,6279808
CG17734-RA	CG17732	0,168015	0	0,337274	0,0105994	111,818	0,137272	-0,29059	0,389438	0,6279808
CG17734-RB	CG17734	40,0348	17,4762	84,7595	47,2301	82,2798	78,0575	-0,291567	0,38673	0,6279808
CG17735-RB	CG17734	7,39082	3,96938	8,03278	5,85014	10,0685	0,0842366	0,090142	0,716235	0,6279808
CG17736-RA	ctrip	29,8531	0,0626681	6,22098	0,0795293	0,0203419	1,03174	-0,012356	0,972411	0,6279808
CG17737-RA	schuy	0,0341831	0,0311363	10,2884	0,0340878	0,0461721	10,9838	-0,370432	0,128814	0,6279808
CG17738-RA	eIF1	286,238	3,0038	14,6225	4,80148	31,6903	2,20578	-0,148617	0,645997	0,6279808
CG17739-RA	CG17738	48,7129	0,0178719	13,6519	231,92	0,030147	0,0260361	-0,755048	0,015624	0,6279808
CG1773-RA	CG17739	0,96759	5,35075	0,0210148	0	2,2967	0	0,158662	0,653631	0,6279808
CG17742-RA	CG1774	4,93769	0,00911962	36,6006	48,4861	0,510824	3,64224	-0,183774	0,583321	0,6279808
CG17743-RA	Dscam2	1,79945	2,6225	17,7012	5,67058	2,28215	3,00101	-0,355589	0,187959	0,6279808
CG17743-RB	pho	39,2417	38,4881	26,151	81,5994	0,0544039	20,9574	-0,351254	0,193341	0,6279808
CG17744-RA	pho	9,57757	6,54293	6,07143	54,8601	8,33599	36,0341	NA	NA	0,6279808
CG17746-RA	CG17744	0	0	0,93213	0,470972	0	0,686969	0,101186	0,665604	0,6279808
CG17746-RB	CG17746	4,79876	6,76408	29,1806	20,4615	5,49073	0,0755341	0,100867	0,668269	0,6279808
CG1774-RA	CG17746	15,0737	269,306	350,213	44,6508	3,47893	13,1404	-0,576575	0,041252	0,6279808
CG17751-RB	Med	0,0187254	0,0172405	0,0181715	0,0200369	0,0271401	0,0626876	-0,081494	0,791026	0,6279808
CG17752-RA	Med	0,0176171	0,0207683	0,0218899	0,0244449	0,0331107	3,33807	-0,321902	0,354223	0,6279808
CG17753-RA	CG17751	0,15855	0,157832	0,0659426	1,14279	0,190591	0,652209	0,004045	0,986661	0,6279808
CG17754-RA	CG17752	0,154023	1,55171	0,369678	0,788786	0,898817	0,183762	-0,276649	0,271673	0,6279808
CG17754-RC	Ccs	5,40322	0,0497134	21,9404	0,0570491	0	0,0582378	-0,277162	0,270931	0,6279808
CG17754-RD	CG17754	9,50632	13,7684	0,0276936	14,7547	0,0273229	2,99475	-0,144753	0,568928	0,6279808
CG17759-RA	CG17754	2,6538	0,0262746	3,59562	0,0307686	6,80068	0,0231666	-0,030864	0,890087	0,6279808
CG17759-RB	CG17754	1,80901	4,89181	8,46491	5,57536	0,0416763	0,340346	-0,019431	0,931284	0,6279808
CG17759-RC	Galphaq	0,024797	0,0294646	0,0238424	12,4417	48,5861	0,045649	-0,020912	0,925934	0,6279808
CG17759-RD	Galphaq	5,60279	1,05714	28,191	0,027855	5,32903	0,0267825	-0,335046	0,167486	0,6279808
CG17759-RE	Galphaq	5,89171	0,0225869	1,67134	0,0447172	0,03548	29,635	-0,019431	0,931284	0,6279808
CG17759-RF	Galphaq	0,0424197	0,0174774	0,0173958	0,0262358	0,0270906	5,07362	-0,335046	0,167486	0,6279808
CG17759-RG	Galphaq	12,6899	8,63846	3,69464	23,8624	8,13627	38,6015	-0,030864	0,890087	0,6279808
CG17759-RH	Galphaq	0,0262762	0,0386388	12,8453	0,0188395	0,0633648	1,11656	-0,019218	0,932048	0,6279808
CG1775-RA	Galphaq	0,0610808	10,4227	47,871	0,0196485	10,9341	1,76301	0,122259	0,607406	0,6279808
CG1775-RB	Galphaq	0,0248343	5,40912	0,0310558	20,3208	0,0377298	1,56348	0,131633	0,579256	0,6279808
CG17760-RB	CG17760	3,67574	1,73385	1,97452	2,2206	3,45755	2,11946	0,322877	0,293521	0,6279808
CG17762-RA	Tomosyn	5,7297	5,21901	4,91439	3,81475	3,99023	2,34241	-0,075343	0,718053	0,6279808
CG17762-RB	Tomosyn	4,81028	4,1701	5,60865	6,7555	4,52166	4,95251	-0,074041	0,722629	0,6279808
CG17762-RC	Tomosyn	1,93443	1,72677	2,37718	2,76919	1,71651	1,68138	-0,091139	0,65576	0,6279808
CG17762-RD	Tomosyn	0,0147601	0,0134445	0,0141705	0,0152262	1,98122	1,36857	-0,085433	0,679422	0,6279808
CG17762-RF	Tomosyn	0,01231	0,0112128	0,0118183	0,0126265	0,0171027	0,0128896	-0,089592	0,660842	0,6279808
CG17762-RG	Tomosyn	0,01231	0,0112128	0,0118183	0,0126265	0,0171027	0,0128896	-0,083804	0,684809	0,6279808
CG17764-RA	CG17764	0,0856645	2,05378	28,6087	60,058	0	71,2276	0,260234	0,444999	0,6279808

gene_id	Symbol	W1_FPKM	W2_FPKM	W3_FPKM	MCM51_FPKM	MCM52_FPKM	MCM53_FPKM	FC	p-value	p-adj
CG17765-RA	CG17765	61,1152	0,0923575	81,7566	21,3426	0,0418522	0,0315423	0,140285	0,511429	0,6279808
CG17766-RA	Dsim\GD24728	5,40753	5,86788	2,591	8,30537	0,0657938	0,0394394	-0,146096	0,508924	0,6279808
CG17767-RB	Tim9b	0,140986	0,12842	0,135355	18,5356	16,1149	25,9117	-0,640561	0,028247	0,6279808
CG17768-RA	CG17768	28,3549	20,8997	25,539	27,668	0,152129	34,2001	-0,415949	0,120312	0,6279808
CG17769-RA	Acam	0	0	0	0,215557	0	0	NA	NA	0,6279808
CG17770-RA	CG17770	0	0	0	0	0	0	NA	NA	0,6279808
CG17776-RA	CG17776	39,0727	0	0,467593	21,0057	135,522	0,493225	-0,261799	0,396874	0,6279808
CG17777-RB	CG17777	0,487046	0,674736	0,369811	192,35	0,236319	3,23094	0,365256	0,208497	0,6279808
CG17778-RA	CG17778	6,46587	5,83578	7,85165	6,07797	7,96881	5,20744	0,038253	0,88956	0,13772387
CG17780-RA	CG17780	4,46361	7,83762	3,12365	8,77159	0,992223	2,36734	0,304705	0,389298	0,6279808
CG17780-RB	CG17780	0,834993	0,0380285	0,0400822	0,0459649	0,487108	0,0469227	0,305311	0,387258	0,6279808
CG17781-RA	CG17781	2,59898	2,9692	1,75508	5,51785	1,17581	2,29088	-0,105116	0,752806	0,6279808
CG17781-RB	CG17781	0,032332	0,0294502	0,0310406	0,0347801	0,289718	0,0355049	0,078612	0,818899	0,6279808
CG17782-RA	CG17782	1,57959	11,311	8,19085	1,84958	0,0202129	12,9395	0,043696	0,890501	0,6279808
CG17784-RA	CG17784	1,11052	0,368274	0,388162	2,0646	1,81938	1,97227	-0,340598	0,341218	0,6279808
CG17785-RA	Golgin84	11,7016	1,29695	12,9584	11,9372	2,30714	0,919743	0,132246	0,494723	0,6279808
CG17786-RA	CG17786	18,8431	0,0730712	4,16226	1,31617	0,29764	0,0094175	0,563676	0,081277	0,6279808
CG17795-RB	mthl2	0,0324697	12,0186	0,0173815	22,6481	0,0115363	0,0192156	0,071523	0,840951	0,6279808
CG17797-RA	Acp29AB	0	1,8755	45,7356	64,6791	0,296919	63,8699	NA	NA	0,6279808
CG17799-RA	lectin-29Ca	0	2,24099	42,0662	0	98,8656	0,103855	-0,041811	0,731452	0,6279808
CG17800-RAA	Idgf4	0,0433095	0,863603	1,75046	1,97264	1,42539	0,0488657	0,120616	0,684762	0,6279808
CG17800-RAB	Idgf4	344,495	0,0394493	0,316773	0,553163	0,216412	319,913	0,117366	0,693227	0,6279808
CG17800-RAC	Dscam1	0,0085917	0,00781372	0,00824532	0,00873371	0,0118299	0,00895845	0,118317	0,690766	0,6279808
CG17800-RAD	Dscam1	0,0085917	0,00782591	0,0123583	0,00872677	0,0118205	0,0089692	0,120156	0,685784	0,6279808
CG17800-RAE	Dscam1	0,0085917	0,00781473	0,00822928	0,00871638	0,0118064	0,00896203	0,120178	0,685743	0,6279808
CG17800-RAF	Dscam1	0,0085917	0,00782286	0,00829709	0,00878963	0,0119056	0,00897279	0,117029	0,694119	0,6279808
CG17800-RAG	Dscam1	0,00857942	0,00782286	0,00829709	0,00878963	0,0119056	0,0089513	0,117813	0,692081	0,6279808
CG17800-RAH	Dscam1	0,00857942	0,00782591	0,00829383	0,00878612	0,0119009	9,76524	0,11998	0,686054	0,6279808
CG17800-RAI	Dscam1	0,00856941	0,00782591	0,0124261	0,00877559	0,0118866	0,0237055	0,11875	0,689222	0,6279808
CG17800-RAJ	Dscam1	0,00857942	0,0117251	0,00828517	0,00877676	0,0118882	0,0228363	0,118169	0,69132	0,6279808
CG17800-RAK	Dscam1	0,00857831	0,00788125	0,00829383	0,00878612	0,0119009	2,28643	0,116797	0,69452	0,6279808
CG17800-RAL	Dscam1	0,00858835	0,00786272	0,0124456	0,00878963	0,0119056	6,9398	0,120298	0,685486	0,6279808
CG17800-RAM	Dscam1	0,00858835	0,00787197	0,00829383	0,00878612	0,0119009	55,5786	0,121144	0,683196	0,6279808
CG17800-RAN	Dscam1	0,00865245	0,00786888	0,00829709	0,00878963	0,0119056	17,9209	0,117528	0,692913	0,6279808
CG17800-RAO	Dscam1	0,00864226	0,011808	0,0397059	20,8099	5,11626	4,43327	0,11829	0,691072	0,6279808
CG17800-RAP	Dscam1	0,00863887	0,00786888	0,0387124	17,7224	0,0616151	0,0464368	0,121356	0,682805	0,6279808
CG17800-RAQ	Dscam1	0,00864226	0,00785964	20,1804	4,61336	0,0599191	0,0451586	0,119879	0,686508	0,6279808
CG17800-RAR	Dscam1	0,00862985	0,00786272	11,2764	1,93915	0,0670291	1,35774	0,117858	0,691803	0,6279808
CG17800-RAS	Dscam1	0,00862985	0,0078658	0,0365209	0,0429396	6,13801	0,0438343	0,11851	0,690333	0,6279808
CG17800-RAT	Dscam1	0,00863887	0,00787197	45,8844	12,9	0,0919616	17,0125	0,120735	0,684291	0,6279808
CG17800-RAU	Dscam1	0,0086321	0,00785348	0,0692792	0,085962	8,67917	0,0877533	0,119422	0,687893	0,13772387
CG17800-RAV	Dscam1	0,00864226	13,3524	384,002	0,049486	4,8392	3,3536	0,117299	0,693284	0,13772387
CG17800-RAW	Dscam1	0,0129634	188,03	0,042776	5,10029	2,19295	4,00044	0,116783	0,694496	0,6279808
CG17800-RAX	Dscam1	0,00863887	0,0159153	0,0181785	245,645	0,0581619	6,12258	0,119741	0,686733	0,6279808
CG17800-RAY	Dscam1	0,00863887	13,789	0,00897309	0,0514569	14,3861	1,86435	0,120355	0,685113	0,6279808
CG17800-RAZ	Dscam1	0,0086321	3,41894	2,06763	0,0181394	2,61756	0,0181526	0,117205	0,693554	0,6279808
CG17800-RB	Dscam1	0,00862197	1,99481	2,42249	4,29551	2,33145	1,57229	0,120342	0,685202	0,6279808
CG17800-RBA	-	0,00857831	0,00782286	0,00829709	0,00878963	0,0119056	0,0089692	0,117205	0,693554	0,6279808
CG17800-RBB	-	0,00864226	0,0156139	0,0164571	0,0095212	0,0240859	1,74393	0,120182	0,685479	0,6279808
CG17800-RBC	-	0,0085917	0,00781473	0,0082776	0,00876858	0,0118771	0,0089692	0,114505	0,700565	0,6279808
CG17800-RBD	-	4,52145	20,3501	0,0167748	11,4768	1,37921	8,45598	0,115578	0,697894	0,6279808
CG17800-RBE	-	0,00859505	0,00781676	0,00824854	0,00873719	1,22728	0,00895964	0,117906	0,691713	0,6279808
CG17800-RBF	-	0,0085917	0,00782896	0,00828733	0,00877909	0,0118913	0,00895845	0,116741	0,694783	0,6279808
CG17800-RBG	-	4,49091	0,00781676	0,00824854	0,00873719	0,0118346	0,00895964	0,11746	0,693012	0,6279808
CG17800-RBH	-	0,00863887	0,0118033	9,96463	11,6452	16,7493	19,8127	0,116381	0,695688	0,6279808
CG17800-RC	-	0,0085917	0,00781473	6,45859	0,0088002	0,0119199	0,00897279	0,121122	0,683237	0,6279808
CG17800-RD	-	0,00864226	0,00786888	19,8257	67,9117	20,2621	16,7733	0,120843	0,683922	0,13772387
CG17800-RE	-	0,0085917	0,00785348	0,00829709	0,00878963	0,0119056	17,6744	0,119945	0,686378	0,6279808
CG17800-RF	-	0,0085917	0,00786888	0,00829383	0,00878612	0,0119009	1,10473	0,116986	0,6942	0,6279808
CG17800-RG	-	0,00858835	0,00781372	0,00829058	0,0087826	0,0118961	0,0089692	0,116267	0,695862	0,6279808
CG17800-RH	-	0,0085917	0,00787197	0,00829383	0,00878612	0,0119009	30,8613	0,118982	0,688735	0,6279808
CG17800-RI	-	0,00857831	0,0078658	0,00829383	0,00878612	0,0119009	10,6771	0,120127	0,685871	0,6279808
CG17800-RJ	-	0,00864226	0,00786272	2,52828	5,13201	51,0437	2,87994	0,119743	0,686894	0,6279808
CG17800-RK	-	0,00863887	0,00851334	9,17049	16,9156	0,116436	0,0210365	0,118058	0,691754	0,6279808
CG17800-RL	-	0,00859505	0,00780764	0,00828517	0,00877676	0,0118882	0,0223506	0,117849	0,691763	0,6279808
CG17800-RM	-	0,00858835	0,00787197	0,00828409	0,00877559	0,0118866	2,80789	0,120634	0,684781	0,6279808
CG17800-RN	-	0,00864226	0,011808	82,8859	0,0442368	1,97924	69,2118	0,120421	0,685467	0,6279808
CG17800-RO	-	0,00863887	0,0405843	0,0184588	0,0678931	1,66461	0,0693079	0,117915	0,691634	0,6279808
CG17800-RP	-	0,00857163	0,00787197	0,00829058	0,0087826	0,0118961	7,59572	0,118058	0,691754	0,6279808
CG17800-RQ	-	0,0128825	0,0078658	0,00828733	0,00877909	1,26572	0,775183	0,118149	0,691371	0,6279808
CG17800-RR	-	0,00862197	0,00786066	15,6942	9,81845	11,4242	8,51555	0,1202	0,685396	0,6279808
CG17800-RS	-	0,00862872	0,00786888	39,5734	5,33257	12,9908	15,333	0,120496	0,685219	0,6279808
CG17800-RT	-	0,00858165	0,00782286	0,00829709	0,00878963	0,0119056	0,00896203	0,117015	0,694081	0,6279808
CG17800-RU	-	0,00858165	0,00780561	0,00829058	0,0087826	0,0118961	0,00897279	0,118534	0,690526	0,6279808
CG17800-RV	-	0,00863549	0,00786888	10,9017	0,0261836	7,39947	0,0267292	0,119686	0,686906	0,6279808
CG17800-RW	-	0,0129532	0,017984	0,0189552	19,7816	0,0279125	1,79026	0,11923	0,688137	0,6279808
CG17800-RX	-	0,0128725	0,00787197	0,00828733	0,00877909	1,37369	0,163454	0,11665	0,694935	0,6279808
CG17800-RY	-	0,00858165	0,00781372	0,00829383	0,00878612	0,0119009	0,00896561	0,117559	0,692957	0,6279808
CG17800-RZ	-	0,0086321	0,00786066	0,0237977	24,5933	0,0354659	12,7605	0,120716	0,68453	0,6279808
CG17801-RA	-	0,00863549	0,00785964	0,0082776	0,00876858	0,0118771	3,36312	0,273196	0,41335	0,6279808
CG17802-RA	-	0,00857831	0,00782591	0,00829709	0,00878963	0,0119056	0,0089692	-0,030399	0,921836	0,6279808
CG17803-RA	CG17801	0,376144	0,00565255	1,60552	0,0213828	2,31175	3,11349	-0,647087	0,021211	0,6279808
CG17806-RA	CG17802	1,10839	0,867904	1,61813	0,0207761	20,399	0,00640707	0,305131	0,26044	0,6279808
CG17807-RA	CG17803	1,29272	0,998637	10,2789	10,5805	0,759373	20,7905	0,3476	0,145728	0,6279808
CG1780-RA	CG17806	2,41945	2,03281	48,8656	37,7964	4,14987	0,815475	0,265655	0,180909	0,6279808
CG1780-RB	CG17807	3,39504	21,5306	22,7745	20,9593	0,0252297	17,7491	0,265704	0,180809	0,6279808
CG17814-RA	Dpse\GA25453	14,6003	0,0589486	1,08731	1,174	1,72608	1,14821	-0,311174	0,330901	0,6279808
CG17816-RD	CG17816	1,89724	1,74896	1,97508	3,93245	3,59361	2,45772	-0,756641	0,001738	0,6279808
CG17816-RE	CG17816	1,69389	1,72613	2,29706	3,42898	1,84453	1,69018	-0,73638	0,002468	0,6279808
CG17816-RG	CG17816	0,0237114	0,021598	0,0227644	0,0249824	1,3886	0,025503	-0,705442	0,003442	0,6279808
CG17818-RA	rdgBbeta	12,0073	15,2858	17,4176	17,3879	4,77417	3395,42	-0,072288	0,738785	0,6279808
CG17819-RA	CG17819	0	0,560396	0,143522	0,0591211	1,12465	9,16143	0,01562	0,897983	0,6279808
CG17820-RA	Uba1	17,9979	18,2984	23,3745	28,0716	11,6696	17,4867	-1,793085	4,28E-07	0,6279808
CG17821-RA	fit	0,492185	0	0,354395	0,127076	3,31577	0,060353	0,228109	0,301848	0,6279808
CG17824-RA	CG17821	0,0918571	29,5781	16,8743	17,4881	0	14,7084	-0,382126	0,209323	0,6279808
CG17826-RA	CG43896	0,629294	0,560744	0,369618	1,11066	1,0634	0,820377	-0,510624	0,15322	0,6279808
CG17828-RA	CG17826	1,67532	0,286602	4,98432	7,43938	28,7374	3,26696	0,113533	0,610819	0,6279808
CG17828-RB	Vsp37A	0,0515948	0,0469961	0,0495341	2,07492	1,32452	9,70147	0,147976	0,521451	0,6279808
CG17828-RC	Vsp37A	4,73954	7,68539	0,0500026	18,4035	48,391	0,0864007	0,113533	0,610819	0,6279808
CG17828-RD	Vsp37A	4,94397	1,94354	11,5665	5,25921	2,08465	3,71045	0,11418	0,608354	0,6279808
CG17829-RA	Vsp37A	0,0712279	0,0648793	0,068383	43,9145	29,6132	3,77582	0,19307	0,355353	0,6279808
CG17829-RB	CG17829	1,68756	1,52342	3,35603	5,00696	1,67798	0,0329161	0,19307	0,355353	0,6279808
CG17829-RC	CG17829	0,0309392	1,40908	0,0297035	0,0331694	0,0449281	2,11808	0,19307	0,355353	0,6279808
CG1782-RA	CG17829	6,46867	7,09603	7,31503	5,82938	4,72171	4,81091	-0,004435	0,985091	0,6279808

gene_id	Symbol	W1_FPKM	W2_FPKM	W3_FPKM	MCM51_FPKM	MCM52_FPKM	MCM53_FPKM	FC	p-value	p-adj
CG17834-RA	Slip1	34,1156	33,4393	28,9482	35,6199	17,8789	20,2653	-0,295053	0,238998	0,6279808
CG17834-RB	CG17834	5,34321	5,48156	5,60251	4,24352	0,053684	4,33215	-0,438825	0,07021	0,6279808
CG17834-RC	CG17834	2,07966	0,0300682	4,69041	3,91144	6,81047	1,83355	-0,289164	0,242534	0,6279808
CG17834-RD	CG17834	4,59617	0,0513682	5,90151	5,34692	8,65238	3,55904	-0,438825	0,07021	0,6279808
CG17835-RA	CG17834	8,65254	11,4662	7,58587	13,0185	13,0803	12,4133	-0,13519	0,578285	0,6279808
CG17835-RB	inv	0,0197026	0,0179465	0,0189156	0,00717968	0,0278515	5,59393	-0,134422	0,580292	0,6279808
CG17835-RC	inv	0,0200189	0,0182346	0,0192194	12,4528	0,0283199	0,0259557	-0,135357	0,577369	0,6279808
CG17835-RD	inv	0,0306237	0,0278942	0,0294006	0,0205621	0,044436	0,0209906	-0,13519	0,578285	0,6279808
CG17836-RA	inv	6,34292	8,30611	6,49078	0,0209079	5,2696	0,0213436	-0,480056	0,012459	0,6279808
CG17836-RB	Xrp1	0,018784	8,73759	0,0180031	8,77167	2,71905	2,34016	-0,452175	0,018284	0,6279808
CG17836-RC	Xrp1	0,0326677	10,1968	3,8011	13,2955	4,31489	4,68435	-0,434852	0,025196	0,6279808
CG17836-RD	Xrp1	42,0746	6,45738	10,4437	8,79671	4,65299	5,68899	-0,405865	0,037501	0,6279808
CG17836-RE	Xrp1	0,0388757	6,63209	1,78851	5,39235	1,2704	1,87197	-0,419889	0,031383	0,6279808
CG17838-RA	Xrp1	89,359	89,7359	10,2432	148,937	2,46771	6,00057	0,436027	0,212927	0,6279808
CG17838-RB	Syp	0,894693	1,10501	0,334848	0,600608	0,322096	0,422053	0,229424	0,516892	0,6279808
CG17838-RC	Syp	0,029164	0,0265646	0,0279991	0,0311321	0,0421686	0,0317808	0,230931	0,514868	0,6279808
CG17838-RD	Syp	0,388123	0,707059	0,0248414	0,802931	0,0371179	0,176598	-0,064429	0,856285	0,6279808
CG17838-RE	Syp	0,202512	0,0194171	0,286519	0,45836	0,0302491	0,0227976	0,12197	0,729096	0,6279808
CG17838-RF	Syp	0,0292933	0,0266823	0,0281233	0,0312799	0,0423688	0,0319317	0,136057	0,698772	0,6279808
CG17838-RG	Syp	0,0213653	0,019461	0,184608	0,0223853	0,0303211	0,0228518	0,210657	0,552985	0,6279808
CG17838-RH	Syp	0,032961	0,0450347	0,0316445	0,0355111	0,0481001	0,0362511	0,436027	0,212927	0,6279808
CG17839-RB	Syp	0,0398127	0,0362642	0,0382226	0,0436218	0,201695	0,0445308	-0,46484	0,069432	0,6279808
CG17839-RC	CG17839	0,0303425	21,0746	6,89496	1,36572	1,44825	1,42758	-0,086052	0,735312	0,6279808
CG1783-RA	CG17839	5,7504	0,523673	27,7407	1,01054	1,9892	9,63454	0,229061	0,291289	0,6279808
CG17840-RA	FIG4	4,36028	0,0165955	0,235707	0,0189479	0,340467	0,445811	0,057854	0,78774	0,6279808
CG17841-RA	CG17841	49,8657	0,0206779	53,9806	60,4023	64,6979	58,4295	-0,294091	0,196443	0,6279808
CG17843-RA	Dsim\GD20911	23,255	14,9797	0,0273591	0	33,3958	0	-0,03935	0,902174	0,13772387
CG17853-RA	CG1785	15,4763	4,59356	27,045	31,2441	10,874	15,9696	0,185286	0,397749	0,6279808
CG17855-RA	Or43b	0,0570274	5,77885	38,0702	40,883	26,2506	0,212812	-0,153494	0,62339	0,6279808
CG17856-RA	CG17855	4,13444	0,521095	6,78391	0	6,97614	0,242821	NA	NA	0,13772387
CG1785-RA	UQCR-14L	0	0	0	0,114591	0,19877	0	-0,032158	0,913126	0,6279808
CG17866-RB	Cyp318a1	0,420642	0,0301133	12,2525	0,00801551	0,0973799	0,00817955	-0,041811	0,731452	0,6279808
CG17867-RA	kl-2	0	0	0	0,602897	0,816627	0,0195451	-0,323118	0,280018	0,6279808
CG17868-RB	Or10a	0,135291	0,0739392	0,0779322	0,257321	0,0460169	0,310946	0,328353	0,330946	0,6279808
CG1786-RA	Or35a	0,134301	0	0,567321	0,256569	0,855502	9,31562	0,039129	0,911345	0,6279808
CG17870-RA	Hexo2	14,0974	0	15,5186	21,9467	13,9092	18,7475	-0,167413	0,438812	0,6279808
CG17870-RB	14-3-3zeta	89,0253	0,00717902	234,035	0,0239387	25,2945	0,00820149	-0,044041	0,83574	0,6279808
CG17870-RC	14-3-3zeta	144,092	0,00700603	36,3927	0,0799151	13,1156	0,00816862	-0,041239	0,846279	0,6279808
CG17870-RD	14-3-3zeta	160,791	0,0174117	23,0859	385,072	0,0213952	0,00796832	-0,040113	0,850459	0,6279808
CG17870-RE	14-3-3zeta	103,122	0,0184188	0,0146822	177,93	3,75446	0,0203369	-0,040297	0,849757	0,6279808
CG17870-RF	14-3-3zeta	0,0678628	0,00980928	3,67565	0,0238089	365,024	0,0215694	-0,165685	0,443891	0,6279808
CG17870-RG	14-3-3zeta	230,457	0,0259126	139,385	0,0238089	1,00511	9,70774	-0,043084	0,839265	0,6279808
CG17870-RH	14-3-3zeta	0,0387845	234,077	136,647	0,02314	8,76921	0,0309474	-0,043351	0,838264	0,6279808
CG17870-RI	14-3-3zeta	0,0226546	0,019392	72,2982	27,3485	0,177606	98,7591	-0,354168	0,117803	0,6279808
CG17870-RJ	14-3-3zeta	0,0226546	270,721	0,0218622	738,63	11,5925	0,0227667	-0,164772	0,446579	0,6279808
CG17871-RB	14-3-3zeta	0,0220498	364,967	0,0651523	1849,14	10,5041	0,133855	0,073265	0,817645	0,6279808
CG17876-RA	Or71a	0,174343	0,132336	1,08724	0,0300255	14,3055	0	-0,564077	0,114984	0,6279808
CG17878-RA	Amy-d	3,52595	1,05818	1,96072	0,0205557	0,0146757	0,0110605	-0,466874	0,192044	0,6279808
CG1787-RA	Gpa2	1,0744	0	0	4,24108	0	3,3165	-0,224264	0,318468	0,6279808
CG17883-RA	CG17883	81,0246	78,251	2833,67	12,7473	11,7911	3,988	-0,047756	0,858895	0,6279808
CG17883-RB	CG17883	0,0421223	0,0383679	26,9713	0,71809	15,3415	142,063	-0,047756	0,858895	0,6279808
CG17885-RA	Or1a	0,84066	20,6049	25,6617	0,084637	0,0371642	39,8551	0,510921	0,145718	0,6279808
CG17888-RA	Pdp1	0,0180997	19,0995	0,0286957	0,0146376	0,0432936	13,2058	0,306843	0,09529	0,6279808
CG17888-RB	Pdp1	0,0216099	0,0196838	30,8819	0,0218726	6,57197	0,0301295	0,33905	0,081031	0,6279808
CG17888-RC	Pdp1	0,0142078	0,0129415	0,0266354	0,0215719	0,0399775	0,0276182	0,268108	0,134652	0,6279808
CG17888-RD	Pdp1	0,0208991	0,0190363	0,0245435	70,2501	0,0366454	5,34948	0,250382	0,168273	0,6279808
CG17888-RE	Pdp1	0,0206251	0,0187867	11,9726	18,4496	5,75365	0,0258063	0,306654	0,095559	0,6279808
CG17888-RF	Pdp1	109,204	98,4423	0,0230205	18,2851	0,0342413	11,1791	0,24937	0,169367	0,6279808
CG17888-RG	Pdp1	45,157	30,6043	54,8394	1,04467	0,0254891	46,6603	0,338896	0,081228	0,6279808
CG17888-RH	Pdp1	0,0237711	0,0216524	60,3716	0,0319627	0,0306859	0,0192101	0,249804	0,169062	0,6279808
CG17894-RA	CG1789	14,9294	11,5095	31,5639	10,4449	4,34738	36,4953	0,347976	0,101179	0,6279808
CG17894-RB	cnc	53,0047	61,7472	0,0181317	32,0188	27,1215	0,0200819	0,32333	0,120904	0,6279808
CG17894-RC	cnc	0,0187787	0,0171049	0,0180031	0,0195552	0,0264876	0,0199331	0,285783	0,170345	0,6279808
CG17894-RD	cnc	0,018886	0,0172027	0,0169587	0,019672	0,0266458	0,0187288	0,348067	0,101201	0,6279808
CG17894-RE	cnc	0,018752	0,0170806	11,6415	0,0195262	0,0264484	7,7968	0,348102	0,101033	0,6279808
CG17894-RF	cnc	0,0176642	0,0160898	0,00953984	7,51433	7,74121	0,0103476	0,345658	0,103662	0,6279808
CG17894-RG	cnc	10,1742	10,008	0,118994	9,28609	4,63346	0,269994	0,342564	0,106557	0,6279808
CG17896-RA	cnc	0,00993672	0,00905105	448,49	5,17371	3,2021	501,373	-0,773149	0,020504	0,6279808
CG17896-RB	CG17896	0,0319258	0,0290803	0,0306507	0,0343093	0,0464721	0,0350242	-0,770263	0,021053	0,6279808
CG1789-RA	CG17896	55,1077	35,5118	100,141	65,014	162,596	130,205	-0,535948	0,092048	0,13772387
CG17903-RA	Dpse\GA14714	463,977	271,238	892,069	435,56	1119,08	1047	-0,397236	0,243441	0,6279808
CG17904-RA	CG17904	14,0102	14,5755	11,3195	19,08	17,8356	22,6983	-0,1804	0,497777	0,13772387
CG17905-RA	ChLD3	12,1336	15,1033	25,5896	18,5363	1,39675	18,3611	0,412943	0,150218	0,6279808
CG17906-RA	CG17906	0,0798018	0,072689	0,0426992	0,49814	0,0667784	3,66709	0,544055	0,096663	0,6279808
CG17907-RA	Ace	3,16816	1,70958	1,72597	0,169672	6,37538	1,27837	-0,135813	0,556182	0,6279808
CG17907-RB	Ace	2,60286	4,74172	0	3,88985	3,18112	0	-0,133124	0,567351	0,6279808
CG17911-RA	CG1791	0,0579782	0,0528105	0,0556625	0,0665223	0,0901048	0,0679084	-0,014277	0,953602	0,6279808
CG17912-RA	CG1791	0,498943	0,50794	0,479016	0,561172	0,250204	0,347862	0,197714	0,397551	0,6279808
CG17912-RB	Or85c	0,169425	0,15393	0,216878	18,8398	0	0,104284	-0,01319	0,955966	0,6279808
CG17914-RA	BuGZ	20,4201	17,288	3,75333	2,72405	4,27367	72,9265	-0,577071	0,010198	0,6279808
CG17916-RA	BuGZ	2,31169	2,66302	1,96889	19,7664	63,0011	1,19584	-0,041811	0,731452	0,6279808
CG17917-RA	yellow-b	27,9797	32,9774	36,4981	6,19076	50,4692	42,601	-0,466775	0,191646	0,6279808
CG17919-RA	Or92a	0	0	0	0,106703	0,0358218	6,70308	-0,458022	0,099901	0,6279808
CG1791-RA	CG17917	0,675304	0,201697	0,648332	159,907	0,975034	1,05949	0,184539	0,603926	0,6279808
CG1791-RB	CG17919	48,4053	2202,47	80,2423	78,3419	0,339857	0,159013	0,184539	0,603926	0,6279808
CG17921-RA	CG1792	3,69054	3,91419	1980,77	4,03737	1,59613	0,786261	-0,012809	0,959625	0,6279808
CG17921-RB	HmgZ	92,3946	1,17289	2,09927	0	0,896773	0,0261677	-0,012847	0,959613	0,6279808
CG17922-RA	HmgZ	290,517	87,7317	0,0388309	0	1,8686	118,201	0,090778	0,681438	0,6279808
CG17923-RB	CngB	3,30612	0,0939432	31,1313	0,134057	0,181581	0,13685	NA	NA	0,6279808
CG17924-RA	JYalpha	0	1,37412	0,0997434	27,2417	0	0	-0,073061	0,747049	0,6279808
CG17925-RA	Acp95EF	0,0802214	0,0342748	0,0140357	0,00922527	7,05752	4,93857	NA	NA	0,6279808
CG17927-RA	CG17925	0,115437	0,042059	0,177322	0,362228	0,347541	0	-0,419685	0,051914	0,6279808
CG17927-RB	Mhc	111,989	4,33053	0,00962086	8,90855	0,0138491	0	-0,417317	0,053357	0,6279808
CG17927-RC	Mhc	0,010012	6,68953	73,1819	12,5744	0,0138362	0	-0,419588	0,052001	0,6279808
CG17927-RD	Mhc	0,010012	19,4818	212,106	41,6018	321,701	0	-0,42133	0,050949	0,6279808
CG17927-RE	Mhc	0,0150317	0,046512	71,6199	3,43724	87,5223	0,0419614	-0,414979	0,054861	0,6279808
CG17927-RF	Mhc	0,010012	70,2048	0,00961212	5,15892	89,3264	0,10949	-0,419695	0,051942	0,6279808
CG17927-RG	Mhc	204,615	14,1224	0,00961212	3,66065	0,0138362	1,26596	-0,417938	0,052986	0,6279808
CG17927-RH	Mhc	81,8481	3,72004	0,00961212	0,339232	0,0138362	4,92921	-0,416736	0,053753	0,6279808
CG17927-RI	Mhc	78,7043	2,19085	0,00896168	0,0603669	0,0128798	2,18684	-0,418391	0,052754	0,6279808
CG17927-RJ	Mhc	0,010012	1,68679	0,00896168	67,5547	0,0128798	4,34604	-0,416271	0,054033	0,6279808
CG17927-RK	Mhc	0,010012	0	0,00896168	86,6504	0,0128798	16,7416	-0,412666	0,056385	0,6279808
CG17927-RL	Mhc	0,00933451	4,02255	7,4078	1,93895	0	13,1371	-0,41264	0,056389	0,6279808
CG17927-RM	Mhc	0,00933451	6,25201	0,085341	9,86509	2,6905	0	-0,415606	0,054461	0,6279808

gene_id	Symbol	W1_FPKM	W2_FPKM	W3_FPKM	MCM51_FPKM	MCM52_FPKM	MCM53_FPKM	FC	p-value	p-adj
CG17928-RA	Mhc	0,00933451	9,27379	9,82173	0,0540751	20,3781	5,21208	-0,911692	0,004298	0,6279808
CG17929-RB	CG17928	4,63263	3,26195	0,920225	6,79969	10,9814	101,84	-0,107027	0,625527	0,6279808
CG1792-RA	CG17929	0	12,334	13,316	10,8256	3,76789	0,031691	-0,129942	0,628822	0,6279808
CG17930-RA	MED26	10,4846	13,1592	16,827	26,4662	7,2526	11,4509	-0,530673	0,137693	0,6279808
CG17931-RA	MED26	38,548	31,429	19,4489	17,9685	16,3158	17,1697	0,145364	0,520981	0,6279808
CG17932-RA	CG17930	5,05662	8,87268	14,1994	11,6885	6,18509	4,4916	-0,017303	0,95368	0,6279808
CG17932-RB	CG17931	102,734	94,862	120,478	1,76207	5,22466	0,081383	-0,017303	0,95368	0,6279808
CG17934-RA	Ugt37D1	0,0348195	0,031716	0,0334288	7,736	0,534426	0,973015	NA	NA	0,6279808
CG17935-RA	Ugt37D1	35,4191	27,0054	59,5965	21,5926	4,96036	0,436883	0,01562	0,897983	0,6279808
CG1793-RA	Mst84Db	0	52,2881	0,0259561	0,0223625	0,169559	0,3563	0,108611	0,641589	0,6279808
CG1793-RB	Mst84Dd	0	2,60065	10,2204	0	0	0	0,109561	0,638455	0,6279808
CG17941-RA	Mmp2	10,7787	0,422555	10,3667	0,579412	0,0176996	0,0133395	0,183293	0,521377	0,6279808
CG17943-RA	Mmp2	0,0127273	13,0291	0,012219	16,9477	0,631748	0,106681	0,457811	0,091357	0,6279808
CG17944-RA	ds	3,4652	0,539789	4,03388	39,0199	1,32966	1,9562	NA	NA	0,6279808
CG17944-RB	comm	30,8091	45,0231	31,851	37,8206	2,56357	22,0607	NA	NA	0,6279808
CG17945-RA	CG17944	0	0	0	0	0	0	0,066601	0,72269	0,6279808
CG17945-RB	CG17944	0	0	0	0	0	0	0,066601	0,72269	0,6279808
CG17946-RA	Mst84Dc	0,212689	0,193732	0,256151	1,023	0,0302902	0,0228285	NA	NA	0,13772387
CG17947-RA	Mst84Dc	0,538659	0,490648	0,50114	0,0678232	0,46805	0,161442	0,118795	0,575629	0,6279808
CG17949-RA	Mst84Da	0,184055	0,369393	0	43,8162	0	0,0371316	-0,003225	0,990727	0,6279808
CG1794-RA	alpha-Cat	73,2006	57,9727	59,5159	70,2799	47,3764	47,9961	0,117668	0,659584	0,6279808
CG1794-RB	His2B:CG17949	2,92607	1,67185	2,8084	0,593672	2,82019	3,34319	0,11567	0,666271	0,6279808
CG17950-RA	Ogg1	5,06514	16,2682	5,91353	10,3207	0	0,060353	-0,141019	0,61097	0,6279808
CG17950-RB	HmgD	238,174	0	0	1,59492	0,0601209	31,7031	-0,141006	0,611054	0,6279808
CG17952-RA	HmgD	0,045361	0	1,27854	27,4446	24,1427	0,721187	0,112029	0,693028	0,13772387
CG17952-RB	LBR	0,0232853	231,817	38,0942	0	2,49197	323,963	0,108346	0,701412	0,6279808
CG17952-RC	LBR	8,06302	0,0368413	0,38681	0	0	0	0,108762	0,700655	0,13772387
CG17956-RA	LBR	2,15016	30,7286	129,374	0,295377	0	0	-0,112975	0,686029	0,6279808
CG17957-RA	Mst87F	0,208222	0,632211	36,2	0,00954741	0	15,742	0,021803	0,937064	0,6279808
CG17958-RA	Sry-alpha	1,69425	0,0781979	127,981	4,14821	129,352	0,0395554	-0,456089	0,034706	0,6279808
CG17959-RA	Sry-delta	12,1809	0,0398322	0,0282485	9,49439	1,53893	1,33827	-0,055549	0,749779	0,6279808
CG1795-RA	CG17959	0	25,4736	0	26,5243	0,21749	0,238123	0,128362	0,639446	0,6279808
CG17962-RA	Tango4	12,821	9,80294	17,6538	57,8822	17,1211	0,331273	-0,021317	0,913975	0,6279808
CG1796-RA	Z600	0,145863	2,14125	52,2229	4,42933	34,8815	1,96128	-0,377112	0,103945	0,6279808
CG17970-RB	Cyp4ac2	0,32864	0,228913	0	0,298659	0,0670795	3,65157	0,070447	0,843841	0,6279808
CG17974-RA	CG17974	0,385469	0,0119449	7,25634	0,0125328	0,0169758	0,012794	0,78038	0,025124	0,6279808
CG17975-RA	sut2	1,85162	0,94752	2,41683	10,472	0,0959327	0,0723007	-0,541815	0,099875	0,6279808
CG17976-RA	sut3	0	0	17,1896	2,04162	3,29999	16,5811	-0,23366	0,334494	0,6279808
CG17977-RA	CG17977	7,31254	7,18788	6,66692	22,1935	23,8858	19,976	0,018381	0,941944	0,6279808
CG17982-RA	CG17982	12,2525	2,55081	18,6949	2,77931	3,3996	11,9144	0,130274	0,604926	0,6279808
CG17985-RA	CG17985	13,0485	0,0152564	13,386	13,4957	9,70951	1,25725	0,14688	0,517344	0,6279808
CG17988-RB	Ance-3	0,584645	0,422009	5,2193	0,539981	1,42585	5,97257	0,012368	0,969046	0,13772387
CG17991-RA	ras	0,0208266	0,0189703	0,0199948	0,021793	175,734	0,0222471	0,313935	0,379689	0,6279808
CG17994-RA	ras	0,0214625	0,0195495	0,0206053	0,0224923	13,4613	0,022961	0,12155	0,709792	0,6279808
CG17996-RA	ras	21,4019	18,2751	30,8141	29,3339	0,0295188	23,0596	-0,451857	0,14248	0,6279808
CG17998-RA	CG17991	0,0617532	0,0562491	0,0592867	0,075954	0,33996	0,0775367	0,112071	0,632271	0,6279808
CG17999-RA	CG17994	0,721352	28,8351	8,17676	9,83403	7,54166	7,36322	0,378538	0,142135	0,6279808
CG1799-RA	CG17996	59,9309	38,7772	46,1092	63,2331	123,785	106,092	-0,182637	0,383618	0,6279808
CG1799-RB	Gprk2	22,1677	24,55	3,64217	28,8785	15,0472	0,0262597	-0,183074	0,38892	0,13772387
CG1799-RC	CG17999	3,95427	5,67287	0,181041	9,25167	5,42677	4,70062	-0,182204	0,38419	0,13772387
CG18000-RA	pasha	2,40128	1,93366	0,0702769	2,76755	2,28259	1,2023	-0,304174	0,133806	0,6279808
CG18000-RB	sw	11,8897	261,671	0,686545	0	94,0536	160,781	-0,304174	0,133806	0,6279808
CG18000-RC	sw	0,0234339	0	0	0	0,149328	0	-0,304174	0,133806	0,6279808
CG18000-RD	sw	0,0232361	13,0239	0	0	0,0781858	0,280332	-0,3078	0,128828	0,6279808
CG18000-RE	sw	0,0234589	0,0213452	1,89957	0	67,7143	0	-0,3078	0,128828	0,13772387
CG18000-RF	sw	0,0232117	0,0211651	12,4659	0	0	0	-0,3078	0,128828	0,13772387
CG18000-RG	sw	0,0234339	0,021368	0,022498	9,04113	0	0	-0,30501	0,131923	0,13772387
CG18000-RH	sw	0,0232607	0,0211428	0,0223081	0,0246737	1,43104	7,91483	-0,30501	0,131923	0,13772387
CG18000-RI	sw	0,0345625	0,0213452	0,0225219	0,0244539	0,0990001	0,0251879	-0,301339	0,137089	0,13772387
CG18000-RJ	sw	0,0228742	0,0211874	0,0222846	0,0247015	0,405318	0,0249635	-0,3078	0,128828	0,6279808
CG18000-RK	sw	0,0228031	0,0314819	0,022498	0,0244268	31,066	0,0252162	-0,3078	0,128828	0,6279808
CG18000-RL	sw	0,0225695	0,0208354	0,0223316	0,0246737	19,9968	0,0249358	-0,309073	0,128016	0,6279808
CG18000-RM	sw	0,0219181	0,0207707	0,0331821	4,52672	9,32031	0,0251879	-0,309073	0,128016	0,6279808
CG18001-RA	sw	0,0219472	0,0205578	0,0219606	2,35114	1,95366	2,39889	-0,599318	0,052387	0,6279808
CG18003-RB	RpL38	5849,52	8,46019	0,0711439	30,5755	0,0237273	0,0178823	-0,594304	0,056111	0,6279808
CG18004-RA	CG18003	9,19988	11,466	1,09683	10,2516	0,0275336	0,020751	-0,310947	0,327313	0,6279808
CG18004-RB	CG18004	0,0592785	0,053995	0	0,0775634	22,505	23,5209	-0,310947	0,327313	0,6279808
CG18004-RC	CG18004	7,29525	6,73774	8,79303	23,4205	0,0459695	0,0346454	-0,312303	0,325006	0,6279808
CG18005-RA	CG18004	0,066164	0,0602667	16,0635	0	0,032168	0,0242437	0,189622	0,361057	0,6279808
CG18009-RE	beag	10,961	12,9971	0,0161143	14,7462	8,49458	10,2643	-0,001626	0,995175	0,6279808
CG1800-RA	Trf2	11,9457	14,2544	14,2381	22,5232	8,89958	0,251915	0,205423	0,453209	0,6279808
CG18011-RA	CG1801	0,0125867	0,0114648	0,012084	0,0129186	0,0174983	62,0951	0,03463	0,874819	0,6279808
CG18012-RA	CG18011	1,7644	1,69743	1,71296	2,76522	4,64391	1,333	0,006762	0,973485	0,13772387
CG18013-RA	Alg1	19,6718	26,6545	25,7039	0,00627628	0,0289632	0,0218284	-0,266433	0,401222	0,6279808
CG1801-RB	Psf2	1,50357	4,60787	5,57345	8,19131	8,84626	4,33361	-0,232344	0,378373	0,6279808
CG18023-RA	Eip78C	0,0200981	0,0561092	0,108998	14,8305	4,93369	7,70877	0,337311	0,314084	0,6279808
CG18023-RC	Eip78C	14,194	0,0368113	0,100534	0,338922	0,330765	17,5877	0,41386	0,208271	0,6279808
CG18024-RA	SoxN	18,1031	0,0980236	0,172195	0,137557	18,015	9,75906	0,417934	0,166513	0,6279808
CG18028-RA	lt	6,57919	5,42107	10,4269	14,4741	43,2815	0,0560763	0,017755	0,931277	0,6279808
CG18028-RB	lt	20,4239	18,843	14,2864	15,6701	10,4889	0,0452802	0,017755	0,931277	0,6279808
CG18028-RC	lt	0,0228189	0,020785	0,0219075	0,023991	11,1703	6,93223	0,017755	0,931277	0,6279808
CG18030-RA	regucalcin	15,0163	20,0986	0,588936	3,81576	0	0,330037	-0,039699	NA	0,6279808
CG18031-RA	regucalcin	0,0630093	0,0564777	0,0317395	0,00801844	17,4757	0,00818254	0,219552	0,476306	0,6279808
CG18039-RA	regucalcin	26,262	12,8997	0,703157	0,00801844	13,1563	0,00818254	0,179561	0,463312	0,6279808
CG1803-RA	regucalcin	0,0620042	57,9397	0	0,00801844	61,0002	0,00817955	-0,651139	0,035454	0,6279808
CG1803-RB	Jon99Fi	6,26278	1,66651	31,6478	71,3675	0,642977	1,23956	-0,650798	0,035659	0,6279808
CG1803-RC	FarO	4,05836	14,1081	12,5823	11,3915	2,70295	2,84728	-0,649497	0,036062	0,6279808
CG1803-RD	GluRIID	7,01575	9,26126	6,52246	2,37962	2,52026	5,35184	-0,650798	0,035659	0,6279808
CG18041-RA	kek6	1,24994	1,0906	4,00935	2,552	2,56967	4,0004	0,144276	0,497295	0,6279808
CG18048-RA	dgt1	10,4245	9,4954	12,9256	11,8066	7,1936	14,812	0,467361	0,052312	0,6279808
CG1804-RA	CG18048	5,76928	167,649	7,27963	226,164	3,47699	5,00959	-0,445523	0,14999	0,6279808
CG18063-RC	CG1806	29,8563	30,9859	0,0283749	0,0315797	0,0427749	0,0322377	0,007621	0,975757	0,6279808
CG18063-RD	CG18063	0,107732	0,09813	0	1,40424	0	0,0866194	0,007621	0,975757	0,6279808
CG18064-RA	CG18063	0,107469	0,0978906	0	0,38232	0	0,0834587	NA	NA	0,6279808
CG18066-RA	Met75Cb	1,63702	0	36,2762	0,0152051	0,0205954	0	-0,396889	0,190644	0,6279808
CG18067-RA	Cpr57A	54,5681	11,1034	23,9096	22,2869	2,47635	0,0741606	0,785323	0,027674	0,6279808
CG18069-RA	CG18067	9,63782	3,32318	0,0125229	0,0443858	0,0707485	0,0533203	-0,010751	0,968611	0,6279808
CG18069-RB	CaMKII	52,747	47,2082	26,7026	26,7818	5,66304	4,20704	-0,012731	0,962781	0,6279808
CG18069-RC	CaMKII	40,9044	30,8901	30,6136	20,6996	0,0438466	24,5324	-0,010044	0,970716	0,6279808
CG18069-RD	CaMKII	0,0216311	0,0197031	0,0207672	0,0226781	0,0353116	21,8396	-0,010911	0,968143	0,6279808
CG18069-RE	CaMKII	0,0213377	0,0194359	0,0204855	0,0223549	1,26386	0,0231507	-0,010944	0,968063	0,6279808
CG18069-RG	CaMKII	0,0228346	0,0207994	0,0219226	0,0240084	0,0224331	0,0228208	-0,010944	0,968063	0,6279808
CG18069-RH	CaMKII	46,3131	42,8645	15,3439	0,0249446	0,0198886	0,0245087	-0,170567	0,504235	0,6279808
CG18069-RI	CaMKII	0,0222428	0,0202602	0,0213544	0,0233532	0,976746	18,963	-0,011322	0,966841	0,6279808

gene_id	Symbol	W1_FPKM	W2_FPKM	W3_FPKM	MCM51_FPKM	MCM52_FPKM	MCM53_FPKM	FC	p-value	p-adj
CG18069-RJ	CaMKII	0,0292803	0,0266705	17,0914	30,6166	1,7333	0,0238399	-0,170855	0,504149	0,6279808
CG1806-RA	CaMKII	0,0370865	0,033781	36,4598	68,927	0,817748	0,0319165	-0,121566	0,562153	0,6279808
CG18076-RA	shot	13,747	14,6658	11,9316	0,0199399	0,0270087	0,0203554	-0,190215	0,403212	0,6279808
CG18076-RB	shot	0,00237754	0,00216563	0,00228258	14,5427	6,22485	7,29008	-0,194321	0,393238	0,6279808
CG18076-RC	shot	18,6973	14,1206	13,9431	0,0210414	0,0285007	0,0214799	-0,194912	0,391715	0,6279808
CG18076-RE	shot	0,00368569	0,00335718	0,00353849	0,022302	0,0302082	0,0227667	-0,190802	0,401585	0,6279808
CG18076-RG	shot	0,00369002	0,00336112	0,00354264	0,0224158	2,78768	0,0228829	-0,190327	0,402488	0,6279808
CG18076-RH	shot	0,00367646	0,00334878	0,00352963	0,0191684	0,0259637	0,0195678	-0,203806	0,37095	0,6279808
CG18076-RI	shot	0,00373528	0,00340235	0,00358609	0,0191851	0,0259864	0,0195849	-0,193796	0,39443	0,6279808
CG18076-RJ	shot	0,00373212	0,00339947	0,00358305	17,2598	6,75912	9,06818	-0,194785	0,392097	0,6279808
CG18076-RK	shot	7,48991	7,53026	6,65625	21,5809	8,92314	20,5581	-0,18978	0,403749	0,6279808
CG18076-RL	shot	0,0037258	0,00339372	0,00357699	0,00238371	0,756679	0,00243338	-0,189681	0,404448	0,6279808
CG18076-RM	shot	0,00372265	0,00339085	0,00357397	10,6655	0	5,63092	-0,19067	0,402077	0,6279808
CG18081-RA	CG18081	91,4707	78,3588	103,297	5,00873	149,336	3,92174	-0,483393	0,050394	0,6279808
CG18085-RA	sev	1,30899	0,0432685	62,6632	2,33731	21,1791	70,1505	-0,252174	0,290725	0,6279808
CG18087-RA	Sgs7	3,80811	2,34371	4,74294	4,49608	12,0785	9,7639	-0,382665	0,280469	0,6279808
CG18088-RA	CG18088	1,53291	0,771281	2,62101	140,832	35,3382	27,1147	-0,471546	0,173862	0,6279808
CG18090-RA	CG18809	23,227	18,5548	0,836013	0,0204156	0,027653	0,020841	-0,400824	0,230675	0,6279808
CG18091-RA	Dsk	2,32067	1,22986	3,40274	3,25485	4,05292	3,58774	0,01424	0,954827	0,6279808
CG18094-RA	CG18091	0	13,485	0	11,3423	1,72739	0	-0,131935	0,588436	0,6279808
CG18095-RA	CG18094	3,04832	21,7475	8,42103	0,480231	16,9743	0,0504414	-0,445629	0,152304	0,6279808
CG18096-RA	CG18095	1,28381	0,050662	0,434428	0,291473	0,737723	0,367872	-1,464493	4,09E-05	0,6279808
CG1809-RA	Tep1	0,0626312	0,0427866	0,112743	0,621007	0	0,366672	-0,802892	0,0113	0,6279808
CG18102-RA	mRNA-cap	16,1502	0,0468498	6,65791	0,0107167	8,5336	5,62379	0,116477	0,577868	0,6279808
CG18102-RB	Dsim\shi	0,0161252	0,014688	6,58725	0,0166877	0,0226036	0,0170354	0,110751	0,595572	0,6279808
CG18102-RC	Dsim\shi	0,0210656	0,019188	0,0202243	0,0220556	0,0298745	0,0225152	0,119951	0,565819	0,6279808
CG18102-RD	Dsim\shi	7,77851	7,15832	0,0154135	0,016612	0,022501	4,14614	-0,164447	0,441044	0,6279808
CG18102-RE	Dsim\shi	0,0209454	0,0190785	0,0201089	0,0219235	0,0296956	0,0223804	0,116313	0,578268	0,6279808
CG18102-RF	Dsim\shi	0,014964	0,0136302	0,0143663	0,0154439	0,0209188	0,0157657	-0,166936	0,432468	0,6279808
CG18102-RG	Dsim\shi	6,5088	0,0141327	8,30448	16,1235	12,242	6,6611	-0,166773	0,433283	0,6279808
CG18102-RH	Dsim\shi	0,0150595	0,0137172	0,014458	0,0155459	0,021057	0,0158699	0,108055	0,604771	0,6279808
CG18102-RI	Dsim\shi	23,9309	33,6595	17,9853	20,7233	12,7875	14,1217	0,108055	0,604771	0,13772387
CG18103-RC	Dsim\shi	9,94598	15,577	13,3425	19,0733	12,6581	10,4083	-0,293948	0,407822	0,6279808
CG18104-RA	Piezo	0,326032	22,4122	25,6617	5,06712	2,926	0,379304	-0,558243	0,08218	0,6279808
CG18105-RA	arg	25,057	20,769	81,2403	27,5642	69,7641	47,561	-0,342146	0,265972	0,6279808
CG18106-RA	ETH	19,0433	3,68698	6,26828	17,148	5,97408	0,318769	0,813584	0,017654	0,6279808
CG18107-RA	IM2	40,3992	13,1708	36,2616	12,2782	23,3975	7,88085	0,677571	0,055537	0,6279808
CG18108-RA	CG18107	2,96202	24,5872	3,17184	16,4347	6,12257	32,0311	1,164612	0,000817	0,6279808
CG18109-RA	IM1	69,4874	15,7826	57,3172	119,052	8,49612	7,82162	NA	NA	0,6279808
CG1810-RA	CG18109	0	0	0	0	0	12,2874	-0,055979	0,782694	0,6279808
CG18110-RA	ppk30	0,408766	0,613254	0,461694	0,0338163	0,0458043	0,234744	-0,124976	0,726603	0,6279808
CG18111-RA	Obp99a	121,393	96,6152	248,061	432,908	0,0613609	0,0462453	-1,197812	0,000117	0,6279808
CG18112-RA	Vps16B	2,75005	0,061248	0,0985043	7,21569	0,0282661	4,42044	0,038101	0,8814	0,6279808
CG18124-RA	CG1812	1,61203	0,956794	0,208568	2,23392	1,57535	1,42682	-0,10168	0,714259	0,6279808
CG18125-RA	CG1812	2,96468	3,5072	1,29802	2,11468	1,09996	2,01204	0,125818	0,636824	0,6279808
CG18128-RA	mTTF	12,0404	0,0490384	0	0	344,501	0,358165	-0,376878	0,276879	0,6279808
CG1812-RA	Send2	0,22943	0,250777	15,9672	0,190983	37,6912	2,33592	0,222335	0,351395	0,6279808
CG1812-RB	CG18128	0,173884	3,1499	0	3,98587	41,4221	1,26907	0,265833	0,273762	0,6279808
CG18130-RA	CG18130	0,102355	0,357389	0,556846	0,516542	0,0499299	0,428569	-0,457369	0,160214	0,6279808
CG18130-RB	CG18130	0,288961	0,171084	0,180323	0,368119	0,584286	0,354732	-0,409	0,222387	0,6279808
CG18131-RA	CG18131	0,0418025	0,0411203	0,0401329	0,050126	0,0623468	6,27091	-0,498226	0,16392	0,6279808
CG18131-RB	CG18131	0,045144	0,237943	0,043341	0,305669	0,067896	0,0469883	-0,498226	0,16392	0,6279808
CG18131-RC	CG18131	0,0435378	0,0427865	0,0417988	0,496111	0,0652169	0,0511705	-0,498226	0,16392	0,6279808
CG18131-RD	CG18131	0,258353	0,0325567	0,293132	0,0387707	0,400699	0,0491514	-0,498226	0,16392	0,6279808
CG18131-RE	CG18131	0,0357424	0,0347564	0,0343149	0,0416372	0,0525152	0,444856	-0,498226	0,16392	0,6279808
CG18131-RF	CG18131	0,0381573	31,908	0,0366333	31,5844	0,0563978	0,0395786	-0,498226	0,16392	0,6279808
CG18132-RA	CG18132	0	8,81011	24,5823	0,328969	6,5431	10,5031	NA	NA	0,6279808
CG18135-RA	CG18135	13,6697	1,48609	14,8887	23,1789	0	0,0548866	-0,48278	0,081077	0,6279808
CG18135-RB	CG18135	0,0210031	11,5818	2,03624	17,8024	8,51095	9,40189	-0,455709	0,097333	0,6279808
CG18135-RC	CG18135	33,2524	22,8747	16,0839	0	12,6312	12,4813	-0,460045	0,096368	0,6279808
CG18135-RD	CG18135	29,4267	18,0559	18,469	0,0237232	1,48038	1,36156	-0,459915	0,096346	0,6279808
CG18136-RA	SmydA-2	5,57974	5,13257	5,8679	4,58311	4,87436	4,92463	0,123804	0,625496	0,6279808
CG18139-RA	Hmx	0,221978	43,4207	85,2773	53,9975	4,65839	66,853	0,14395	0,662798	0,13772387
CG18140-RA	CG1814	4,76136	7,9882	7,24749	0,0446868	0,0605286	5,90384	0,176169	0,589854	0,6279808
CG18143-RA	CG1814	8,78	10,3694	7,97323	12,6208	8,30141	6,99494	-0,692558	0,032267	0,6279808
CG18144-RA	CG1814	8,42948	10,9037	7,2244	15,9936	9,40558	7,96309	-0,064019	0,822371	0,6279808
CG18145-RA	Cht10	9,07707	7,76873	8,00096	14,2587	0,0324959	31,6199	0,517633	0,067384	0,6279808
CG18146-RA	DhpD	18,5785	11,644	26,5092	18,6656	7,35912	39,697	-0,651689	0,0659	0,6279808
CG18146-RB	Hand	46,8195	18,4234	9,47616	58,0989	0,03076	5,12198	-0,651689	0,0659	0,6279808
CG1814-RA	Dsim\GD22299	3,92686	1776,4	0,0204767	4,1408	0,0308237	0,0232306	0,097562	0,649731	0,6279808
CG1814-RB	NimC2	0,135982	0,154827	0,0301697	0,0337297	0	0,0344326	0,097562	0,649731	0,6279808
CG1814-RC	NimC2	0,0314248	0,0286239	25,2456	0,419494	23,8791	33,6309	0,117207	0,58904	0,6279808
CG18155-RB	CG1815	0,00942102	0,00858131	0,00904474	0,00959887	0,015939	0,0120126	0,268267	0,364557	0,6279808
CG18156-RA	CG1815	5,55863	7,21246	6,24058	6,8922	24,0518	0,0543544	-1,794737	2,85E-11	0,6279808
CG18157-RA	CG1815	0,0114941	0,0104696	0,011035	1,35279	0,0721206	4,77124	0,01562	0,897983	0,6279808
CG1815-RA	CG18155	1,24704	1,12033	6,97587	33,6632	0,424302	0,100317	0,241111	0,352788	0,6279808
CG1815-RB	Mis12	1,7451	1,40893	20,0249	92,7244	45,6755	13,4123	0,243255	0,349903	0,6279808
CG1815-RC	betaNACtes2	0,0556194	0	4,01362	7,00598	5,32801	0,0615684	0,243255	0,349903	0,6279808
CG18160-RA	CG18160	0	0	0	0,0708513	0	0	NA	NA	0,6279808
CG18166-RA	CR18166	0,971337	0,680585	2,33133	2,92844	2,10759	3,23805	0,354318	0,267584	0,6279808
CG18166-RB	CR18166	1,18496	0,925153	1,57815	0,820921	0,416383	0,535614	0,193048	0,5459	0,6279808
CG18171-RA	Ptp10D	0,0091244	0,00831114	0,00877572	11,6128	3,87937	0,00950131	0,165755	0,543104	0,6279808
CG18173-RA	Ptp10D	0,00914081	0,00832608	7,76372	12,0227	9,67016	4,54765	-0,198426	0,52991	0,13772387
CG18174-RA	Ptp10D	9,62791	11,1554	4,00791	4,98447	0,0125838	3,15432	0,036338	0,868534	0,6279808
CG18176-RA	Ptp10D	4,90219	5,65954	12,0467	17,5378	0,0126069	6,63585	-0,020556	0,936332	0,6279808
CG18177-RA	CG18171	1,72658	28,3679	2,84755	265,005	0,0420098	0,0316611	0,235276	0,281602	0,6279808
CG18177-RB	PIG-Wb	12,735	7,79667	15,5335	7,43899	16,5166	3,57563	0,235276	0,281602	0,6279808
CG18177-RC	Rpn11	33,4636	34,1154	139,184	39,3519	33,0854	37,2117	-0,095388	0,689985	0,6279808
CG18178-RA	defl	1,37104	2,60172	3,01644	19,4296	1,72605	1,37033	0,004059	0,98984	0,6279808
CG18179-RA	Naa60	0,0512348	0,0466682	0,778259	0,659101	0,0430223	0,0324242	-0,295625	0,312931	0,6279808
CG1817-RA	Naa60	7,75543	12,451	0,028528	0,0317624	0,0430846	0,0324712	0,269102	0,333412	0,6279808
CG1817-RB	Naa60	7,96794	5,81181	0,0285665	0,0318084	27,0549	24,328	0,080587	0,779303	0,6279808
CG1817-RD	CG18178	15,8613	1966	0,0491884	0,0577733	0,0782543	0,0589772	0,080554	0,779594	0,6279808
CG1817-RE	CG18179	0,529212	0,0986664	6,0969	5,70946	0,0084298	0,66165	0,284775	0,304291	0,6279808
CG18180-RA	Updo	19,635	24,9392	41,1813	34,5041	23,9148	36,25	-0,186234	0,506195	0,6279808
CG18186-RA	Updo	0,0543387	0,0494955	0,0521684	0,0650127	0,08806	0,0663674	-0,583101	0,092158	0,6279808
CG18188-RA	CG18180	1,69237	0,20659	30,906	0	4,64053	0,39804	-0,514075	0,142418	0,6279808
CG1818-RA	Oxp	2,07297	0	109,148	0,038657	129,923	140,677	-0,144868	0,573919	0,6279808
CG1818-RB	Damm	12,6908	4,26719	10,8413	6,63815	62,398	18,325	-0,13917	0,588157	0,6279808
CG18190-RB	CG18190	4,19775	3,91464	35,3702	23,8654	0,0734259	26,1277	-0,017462	0,947887	0,13772387
CG18193-RA	Dsim\CoVIIa	0,282859	0,309177	0,162937	0	0,350901	0,26446	0,164696	0,5132	0,6279808
CG18208-RA	Octalpha2R	0,799759	1,07732	0,886774	0,397499	1,13879	0	0,346759	0,235519	0,6279808
CG18210-RA	RpL31	0,12756	6,38111	2,03068	1,59023	1,48042	1,62337	0,38124	0,132268	0,6279808
CG18211-RA	RpL31	0,12397	0,0145109	24,106	15,463	18,1192	12,2365	-0,04808	0,875831	0,6279808

gene_id	Symbol	W1_FPKM	W2_FPKM	W3_FPKM	MCM51_FPKM	MCM52_FPKM	MCM53_FPKM	FC	p-value	p-adj
CG18212-RA	RpL31	2481,62	0,0143449	15,2498	0,208835	0,151526	0,0168224	-0,050969	0,861448	0,6279808
CG18212-RB	Cngl	1,71207	0,0953323	30,1672	0,0462229	0,0475365	21,9093	-0,051114	0,86107	0,6279808
CG18212-RD	betaTry	4,67831	4,1039	45,5505	68,6728	118,564	104,106	-0,051035	0,861262	0,6279808
CG18212-RE	alt	18,3881	18,7531	3,58823	2,26835	36,3642	1,00039	-0,050282	0,863309	0,6279808
CG18212-RF	alt	0,0198984	0,0181248	0,686453	2,42238	3,72589	3,06908	-0,051114	0,86107	0,6279808
CG18212-RG	alt	4,05531	3,91373	22,772	2,19802	0,0276873	11,4993	-0,051656	0,859686	0,6279808
CG18213-RA	alt	0,0200981	0,0183067	0,0191036	0,2501	11,375	38,2839	-0,476946	0,121034	0,6279808
CG18214-RA	alt	0,02044	0,0186182	3,42985	1,18812	2,05599	0,190863	0,0815	0,734115	0,6279808
CG18214-RB	alt	0,0204527	0,0186297	0,0192954	2,257	1,70305	1,95659	0,189684	0,41171	0,6279808
CG18214-RC	CG18213	1,03519	0	2,22029	28,0867	5,09838	0,197187	0,081535	0,733857	0,6279808
CG18214-RD	trio	20,6892	23,2334	15,9323	9,84338	9,05189	9,11397	0,176004	0,447976	0,6279808
CG18214-RE	trio	0,0158266	0,0144159	0,0151944	0,0165869	0,022467	0,0169325	0,227187	0,329614	0,6279808
CG18214-RF	trio	0,0134629	0,0122629	0,0129252	0,0140031	0,0189672	0,0142948	0,126643	0,591535	0,6279808
CG18217-RA	trio	0,00694512	0,0063261	0,00666773	0,00707636	0,00958497	0,00722382	-0,453298	0,079648	0,6279808
CG1821-RA	trio	3,13626	3,81981	2,86354	8,84437	2,16967	2,92335	-0,303539	0,240621	0,6279808
CG1821-RB	trio	10,6333	16,3729	11,1727	20,1489	13,7692	14,4724	-0,303586	0,240563	0,6279808
CG1821-RC	CR18217	3,21498	3,1873	3,53266	4,84311	88,7781	129,353	-0,303547	0,240534	0,13772387
CG18223-RA	bif	11,1061	0,0338507	10,1087	4,31813	3,81914	9,97221	0,236001	0,398801	0,13772387
CG18223-RB	bif	10,5335	20,7685	12,9757	23,9969	2,73031	14,8369	0,045133	0,818583	0,6279808
CG18228-RA	CG18223	0,0680493	0,061984	0,0653314	0,0857383	6,46922	22,3765	-0,541623	0,112968	0,6279808
CG1822-RB	CG18223	0,0664747	0,0605498	0,067438	0,0832469	3,95098	1,12828	0,164845	0,385564	0,6279808
CG1822-RC	CR18228	2,41505	1,45187	13,3055	2,14709	4,9147	4,32537	0,153235	0,415615	0,6279808
CG18231-RB	CG18231	0	0,644976	14,1341	0,165578	0,089939	10,9779	0,01562	0,897983	0,6279808
CG18233-RA	CG18233	0,061638	0,0583202	0,0830325	0	0	0,106937	0,150972	0,527286	0,6279808
CG18234-RC	CG18234	0	0,0935735	0	6,54218	0	0	-0,0276	0,879281	0,6279808
CG18241-RA	CG1824	22,3953	19,7688	28,3084	10,1539	5,30728	9,42261	-0,337669	0,264811	0,6279808
CG18243-RB	Toll-4	0,0489016	0	0	0	0,153162	0	-0,396941	0,219132	0,6279808
CG18247-RA	Ptp52F	1,48218	11,7398	0,0206187	15,3177	2,09985	0,374952	0,042088	0,861645	0,6279808
CG18249-RA	Shark	7,85925	0,0218093	0,0229871	0,0242827	5,38756	0,0257668	-0,428468	0,122119	0,6279808
CG1824-RA	CG18249	4,33014	6,84964	5,47416	11,5336	4,46869	7,31774	-0,641078	0,017636	0,6279808
CG18250-RA	Map60	16,2528	15,58	28,0219	0,0154511	0	0,0157731	0,353407	0,270123	0,6279808
CG18250-RB	Dg	49,0353	0,0287698	12,8268	27,7501	0,00658943	21,7328	0,354233	0,268945	0,6279808
CG18250-RC	Dg	0,0116646	0,0408876	0,0430956	0,0279733	2,64361	0,0121955	0,341748	0,285825	0,6279808
CG18255-RA	Dg	5,8249	0,017993	0,0189647	0,0529454	0,011855	4,09178	0,446411	0,047676	0,6279808
CG18255-RB	Strn-Mlck	0,0197536	0,00439521	0,00463256	0,013337	56,1939	0,0214047	0,556632	0,010875	0,6279808
CG18255-RC	Strn-Mlck	21,8912	5,62193	3,54538	48,6715	12,2021	13,34	0,304576	0,172879	0,6279808
CG18255-RD	Strn-Mlck	0,0126003	0,00783916	0,00826251	139,433	0,03789	0,0133421	0,612033	0,011708	0,6279808
CG18255-RE	Strn-Mlck	0,00482529	0,00272637	0,00287361	278,577	529,725	0,00496619	-0,437494	0,093437	0,6279808
CG18255-RF	Strn-Mlck	6,3908	0,571029	0,662054	5,58634	2,77357	3,32415	0,441647	0,047152	0,6279808
CG18255-RG	Strn-Mlck	0,00860624	70,3534	31,2459	20,4165	14,9672	0,00893465	0,591476	0,007312	0,6279808
CG18258-RA	Strn-Mlck	0,00299315	0,0106249	0,0111987	0,0633769	0,0161817	0,00306773	0,005662	0,980467	0,6279808
CG18259-RA	CG18258	0,0671503	0,17295	0,107447	0,309331	0	0,127618	-0,029796	0,881242	0,6279808
CG1825-RA	CG18259	9,93417	11,4963	11,9765	1249,27	0,167286	9,94255	0,031969	0,904628	0,6279808
CG18262-RA	BTBD9	4,32903	4,68946	5,70804	4,77977	0,030466	2,97311	-0,068241	0,785534	0,6279808
CG18265-RA	CG18262	4,12389	5,61314	12,1515	0,286054	24,1583	0,292015	-0,116286	0,715864	0,6279808
CG18266-RA	CG18265	2,55768	3,29924	542,531	0,0374913	3,49542	5,11504	-0,421979	0,191483	0,6279808
CG18268-RA	CG18266	0,0915686	69,2795	0	98,4841	3,05572	0	0,125051	0,647591	0,6279808
CG18269-RA	CG18268	0,0645481	0,0647397	0,06197	0,381313	0,51649	0,389258	-0,041811	0,731452	0,6279808
CG1826-RA	CG18269	0	1,57257	1,67598	9,49409	2,0036	16,9913	0,336589	0,109749	0,6279808
CG18271-RA	CG1827	3,53977	4,91215	3,78613	4,81817	2,6278	5,78922	-0,496687	0,096651	0,6279808
CG18273-RA	CG1827	1,15222	1,04952	1,99115	3,52015	1,41138	1,14261	-0,216405	0,436952	0,6279808
CG18278-RA	slx1	1,67868	1,59413	2,0917	27,5564	1,32621	48,6126	2,046513	1,06E-08	0,6279808
CG18279-RA	CG18273	5,81131	3,63351	13,3486	7,9691	5,79999	6,23276	0,893543	0,004321	0,6279808
CG18279-RB	CG18278	4,45806	4,34568	0,0913195	0,120787	0,9509	0,0370294	0,893543	0,004321	0,6279808
CG1827-RB	IMPPP	6,56655	13,9874	4,39264	0,0427723	27,4388	20,0663	-0,212446	0,429894	0,6279808
CG1827-RC	IMPPP	43,0945	44,3131	1092,45	715,005	0,0579354	0,0816794	-0,214604	0,425853	0,6279808
CG18281-RA	dre4	3,02915	3,4723	4,58148	111,051	2,75378	54,5457	0,17635	0,425276	0,6279808
CG18284-RA	dre4	0,830084	1,40536	1,89704	0,0173695	0,744113	2,57348	0,01562	0,897983	0,6279808
CG18285-RA	CG18281	0,178583	0,0903701	0,0229455	0,0675802	0	0,0574904	0,007295	0,979095	0,6279808
CG18285-RB	CG18284	0,0683073	0,0414793	0,0474969	0	21,3948	0,0268071	0,040932	0,883354	0,6279808
CG18287-RA	igl	13,5986	0,637427	14,5132	0,033678	0,148129	7,23088	0,297322	0,314698	0,6279808
CG1828-RA	igl	87,0543	13,6105	70,8775	8,0462	82,2986	74,7818	-0,112611	0,675333	0,6279808
CG1828-RB	ppk19	1,53751	0,0405934	0,0427855	0,0494118	21,2641	23,1576	-0,116352	0,666107	0,6279808
CG18290-RA	Cyp6v1	3,22542	2,0593	5,10794	1,06654	0,309105	1,08877	0,446182	0,069314	0,6279808
CG18290-RB	Act87E	548,196	525,175	639,501	343,484	0,0619572	447,701	0,446415	0,069225	0,6279808
CG18292-RA	Act87E	0,0415658	0,037861	0,0399057	0,0457416	306,835	0,0466947	0,16868	0,45037	0,13772387
CG18294-RA	CDK2AP1	14,9692	4,10567	0,0473762	3,01451	0,377625	0,0316462	3,036076	1,34E-28	0,6279808
CG18296-RA	CG18294	81,4454	0,0504637	0,0473526	22,9	13,7208	55,6591	-0,402461	0,116373	0,13772387
CG1829-RA	axo	1,10937	0,931012	6,91382	6,32872	0	0,0257274	-0,612574	0,060252	0,6279808
CG18301-RA	PhKgamma	0,037163	0,0244065	0,0356787	0,0092903	0,0566484	0,0426936	0,133091	0,613465	0,6279808
CG18302-RA	PhKgamma	16,2266	0,105282	18,4517	0,00930736	12,0302	14,7617	-0,131419	0,365453	0,6279808
CG18304-RA	PhKgamma	0,0267948	11,6798	0,0257246	10,4839	0,03943	0,0297169	-0,199924	0,318912	0,6279808
CG1830-RA	CG18301	0,130173	40,118	30,2721	23,4622	0	20,1513	0,00435	0,981594	0,6279808
CG1830-RB	CG18302	0	0	0	0,0251829	0	0,0990624	-0,186759	0,386606	0,6279808
CG1830-RC	CG18304	6,53498	12,3821	6,61708	3,19548	4,66082	22,8024	-0,22864	0,283418	0,6279808
CG18313-RA	betaNACtes4	0,184776	0	0,106438	0,00886651	2,70472	0,145906	-0,091622	0,584546	0,6279808
CG18314-RA	DopEcR	14,5104	0,0252813	0,0266466	0,0295277	9,48663	0,030143	-0,019241	0,934736	0,6279808
CG18314-RB	DopEcR	0,0276854	25,1481	16,941	18,0025	0,0398882	8,86141	0,418007	0,053332	0,6279808
CG18314-RC	DopEcR	0,0277552	10,7949	12,3558	12,6177	0,0399955	6,85959	-0,024389	0,917248	0,6279808
CG18315-RA	Aprt	0,0835696	2,59158	4,66259	3,98666	3,51243	9,08314	0,144984	0,547068	0,6279808
CG18315-RB	Aprt	41,9327	0,076121	11,4091	7,42237	2022,86	3,86111	0,144984	0,547068	0,6279808
CG18316-RA	udd	26,3119	20,1486	32,421	0,0524398	0,0678444	0,0511316	0,122531	0,564977	0,6279808
CG18317-RA	Rim2	13,9518	20,9599	12,7874	27,0928	14,8772	12,2541	0,004718	0,987923	0,6279808
CG18319-RA	ben	61,8497	60,7935	69,0094	7,41705	0	46,5324	0,305297	0,113497	0,6279808
CG18321-RA	Clamp	38,1784	40,7857	40,0589	45,9486	26,9711	27,9925	0,202074	0,40854	0,6279808
CG18324-RA	Clamp	0,0253877	0,0231249	0,0243737	0,026856	0,0363766	0,0274156	0,009933	0,968964	0,6279808
CG18324-RB	miple2	104,045	94,4014	118,018	136,236	0,052826	11,7994	0,017497	0,945738	0,6279808
CG18327-RA	CG18324	18,0872	20,1092	4,2453	17,3063	99,3344	21,4649	-0,090341	0,780657	0,6279808
CG1832-RA	CG18324	3,29681	4,17078	114,528	3,99154	22,5202	2,41179	0,138249	0,467768	0,6279808
CG1832-RB	CG18327	2,0937	1,58385	2,72553	13,4477	3,08639	18,9756	0,138616	0,466578	0,6279808
CG18330-RA	Pcyt2	2,32253	1,58664	3,69769	0,0262462	18,0742	0,019074	-0,564902	0,046449	0,6279808
CG18331-RA	Muc68Ca	0,0526753	0,0639737	0,1652	1,1432	0	1132,74	-0,209638	0,557708	0,6279808
CG18332-RA	CSN3	5,21134	16,2439	20,4635	17,0461	0	0	0,158716	0,530464	0,6279808
CG18335-RA	CG18335	0,0512614	0,0466924	0,049214	10,0128	2,59545	10,8122	0,283529	0,346976	0,13772387
CG18336-RA	CG18336	0,0889312	0,103472	0,105728	45,4921	0,938946	2,59243	0,008249	0,973112	0,6279808
CG18340-RA	Ucp4B	0,830947	2,01836	46,9873	17,962	5,91196	5,37544	0,000213	0,999494	0,6279808
CG18340-RB	Ucp4B	0,775643	1,50517	0,0300696	4,87585	0,273623	1,81714	0,038992	0,907994	0,6279808
CG18341-RA	Prosbeta2R1	0	1,29653	0	2,4356	0	7,43422	-0,041811	0,731452	0,6279808
CG18343-RA	CG18343	5,07741	5,2584	21,1679	2,08536	37,1527	4,32054	1,080717	0,002172	0,6279808
CG18345-RA	trpl	0,116184	0,455876	0,0171605	0,176618	11,7985	0,018961	-0,187941	0,543005	0,6279808
CG18345-RB	trpl	0,546046	0,508944	0,329172	0,562852	30,7132	0,197754	-0,183465	0,551189	0,6279808
CG18345-RC	trpl	0,0173352	0,0157901	0,332856	0,361645	0,0562202	0,383756	-0,125002	0,695606	0,6279808
CG18347-RA	GC1	13,4556	4,91517	21,153	0,0738265	0,362822	0,0753649	-0,317745	0,253211	0,6279808
CG18348-RA	Cpr67Fb	39,0367	15,989	0,0353868	59,5772	20,2032	0,0409272	-0,714089	0,026896	0,13772387

gene_id	Symbol	W1_FPKM	W2_FPKM	W3_FPKM	MCM51_FPKM	MCM52_FPKM	MCM53_FPKM	FC	p-value	p-adj
CG18349-RA	Cpr67Fa2	82,6565	42,8971	25,0626	0,0185688	0,0543046	22,0057	-1,309979	0,000154	0,13772387
CG18350-RA	CG1835	0,833301	0,775763	1,02207	0,521431	1,10004	0,662149	0,223487	0,38815	0,6279808
CG18350-RB	CG1835	0,645855	0,0983972	0,777833	1,45679	1,39996	1,02579	-0,216711	0,382797	0,6279808
CG18350-RC	Sxl	27,3358	0,0209368	0,0220675	0	0,103477	0	-0,141503	0,559353	0,6279808
CG18350-RD	Sxl	0,0124656	0,0189643	0,0199885	8,12374	0,133107	15,4343	0,161931	0,530451	0,6279808
CG18350-RE	Sxl	30,7404	24,7772	13,1306	38,7976	0	0	0,222945	0,389554	0,6279808
CG18350-RF	Sxl	0,0229856	0,0187516	0,0197643	2,49705	7,90387	8,82873	0,154244	0,550869	0,6279808
CG18350-RG	Sxl	0,0235592	0,0413463	0,0435791	1,9648	13,131	60,2782	0,162274	0,529417	0,6279808
CG18350-RI	Sxl	0,02082	0,0320027	0,033731	0	89,1973	4,68127	-0,13634	0,576263	0,6279808
CG18350-RJ	Sxl	24,8246	0,0334245	0,0352296	36,797	0	0	-0,211924	0,389443	0,6279808
CG18350-RK	Sxl	0,0205865	0,0344579	0,0363187	0	0,0386867	0,866809	-0,215719	0,384791	0,6279808
CG18350-RL	Sxl	0,0453921	0,0306033	0,032256	0,180456	0	0	-0,21244	0,388547	0,6279808
CG18350-RM	Sxl	0,0351343	0,0289822	0,0305474	1,47525	1,65825	0,0920585	0,060835	0,827185	0,6279808
CG18350-RN	Sxl	0,0366952	0	0	0,0141992	0,0192329	14,4625	0,240052	0,353305	0,6279808
CG18350-RO	Sxl	0,0378297	0	0,367395	41,2254	24,3438	16,0144	0,106474	0,703261	0,6279808
CG18356-RA	Sxl	0,0335979	0,0819979	0,178193	16,7016	0,0173251	0,0130573	-0,255532	0,318844	0,6279808
CG1835-RA	Sxl	0,0318182	0,290475	0,064512	0,0258447	0,0350068	14,7782	-0,199543	0,572955	0,6279808
CG1835-RB	CG42663	10,0504	9,73891	8,31453	27,1632	19,657	20,0853	-0,336311	0,346079	0,6279808
CG18361-RA	Rad23	0,0453921	0,0413463	0,0435791	3084,22	15,0072	0,0254163	-0,110555	0,644852	0,6279808
CG18361-RB	Rad23	60,2433	57,0087	79,5372	0,0867543	5,87735	0,0255612	-0,110555	0,644852	0,6279808
CG18362-RA	dsh	8,35215	9,40727	1,77756	6,0074	2,92525	0,0189504	0,075128	0,673706	0,6279808
CG18362-RB	dsh	0,0242332	0,0220733	0,0171513	3,27355	0,774595	0,0186768	0,068529	0,700919	0,6279808
CG18362-RC	Mondo	0,0202687	22,0019	24,1312	32,0088	1,33088	20,4709	0,073637	0,68003	0,6279808
CG18362-RD	Mondo	0,0182178	0,0142218	0,0149898	6,5046	0,0291905	0,016693	0,132976	0,454517	0,6279808
CG18362-RE	Mondo	25,7396	0,0564777	0,0566906	0,0718933	0,0260622	0,0733914	0,117769	0,507527	0,6279808
CG18363-RA	Mondo	17,241	0,0550821	0,055355	0,069836	9,00119	0,0712912	0,015925	0,916254	0,6279808
CG18367-RA	Mondo	0,0156134	0,0550821	0,055355	0,069836	19,7752	0,0712912	0,195901	0,579708	0,6279808
CG18368-RA	38322	0	69,6484	0,060416	0,0720117	0,25102	0,0735122	1,071906	0,001008	0,6279808
CG18369-RA	CG18367	7,26366	6,72296	1,3923	9,15958	0,0475365	2,41879	0,054636	0,748216	0,6279808
CG1836-RA	CG18368	0,940254	0,0429086	0,78765	0	0	0,287878	-0,053781	0,783937	0,6279808
CG1836-RB	S-Lap5	0,0796479	0,0283497	11,1998	1,97366	0,347784	0	-0,054208	0,782362	0,6279808
CG18371-RA	prtp	70,7608	41,9113	98,1957	0,0237831	79,8347	4,43778	0,002089	0,994601	0,6279808
CG18372-RA	CG18371	0	0,861565	0,194591	0,74328	33,1621	0,758769	-0,106516	0,74816	0,6279808
CG18374-RA	AttB	2,55426	0,225982	6,84797	2,83027	0,377233	4,64808	-0,332333	0,223852	0,6279808
CG18374-RB	Gk1	8,71788	0,0254309	10,7147	0,0196837	0,05893	12,2779	-0,329147	0,224051	0,6279808
CG18375-RA	Gk1	11,1031	2,85722	23,2405	6,33217	28,2913	14,8015	0,173477	0,526733	0,6279808
CG18375-RB	ASPP	5,92258	0,731238	1,66003	2,86777	2,71239	3,78413	0,191783	0,481812	0,6279808
CG18377-RA	ASPP	2,83764	4,69381	4,39511	0,265974	2,50327	1,16048	0,59475	0,072492	0,6279808
CG18377-RC	Cyp49a1	9,51712	18,1872	7,24448	8,61031	0,0322493	0,024305	0,684733	0,038454	0,6279808
CG18377-RD	Cyp49a1	2,01015	0,0267297	1,76083	2,33351	0,0322493	0,024305	0,611341	0,06436	0,6279808
CG1837-RA	Cyp49a1	5,09013	15,35	5,46662	10,6593	0,0313433	0,0236222	-0,307762	0,125111	0,6279808
CG18389-RA	myo	0,0198208	0,0215825	0,0190291	3,36132	0,0280264	0,0211224	0,685985	0,031785	0,6279808
CG18389-RB	myo	13,5018	1,99495	8,21147	13,5775	14,5289	8,63174	0,230854	0,513082	0,6279808
CG1838-RA	myo	19,6476	1,21561	14,0624	13,3844	12,2346	12,7153	-0,168437	0,536396	0,6279808
CG1838-RB	myo	0,0214974	1,70799	0,0206388	0,0225308	7,33988	0,0230002	-0,162515	0,549758	0,6279808
CG1838-RC	Eip93F	0,0170313	0,121036	0,0474234	0,0306256	0,0427339	0,0312638	-0,168129	0,537594	0,6279808
CG1838-RD	Eip93F	0,488672	3,67508	0,124712	0,0312354	5,86068	0,0318863	-0,15475	0,568684	0,6279808
CG18396-RA	Fbxl4	3,41654	4,60579	3,60809	7,0237	2,83193	3,38899	-0,041811	0,731452	0,6279808
CG18397-RA	Mst98Cb	0,0620431	0	0	0	0	0	0,415681	0,11347	0,6279808
CG18398-RA	ssp3	2,97848	2,22241	3,03533	10,4991	7,55242	8,90153	0,208012	0,350778	0,6279808
CG1839-RA	Tango6	3,43687	2,87757	10,2166	0,477099	0,0336475	8,18841	-0,062729	0,792673	0,6279808
CG18402-RA	CG1840	27,9623	5,30898	10,9344	10,6604	0,41543	9,0595	0,079861	0,775384	0,6279808
CG18402-RB	InR	0,00851639	0,00775732	0,00817625	3,80404	1,52864	1,53201	0,083988	0,764253	0,6279808
CG18402-RC	InR	0,00858946	0,00782388	0,0082464	0,00865911	0,0117288	0,00883955	0,038482	0,891118	0,6279808
CG18402-RD	InR	3,45485	4,85463	4,26778	1,84684	0,0118314	0,00891688	0,03851	0,891141	0,6279808
CG18404-RA	InR	3,01824	4,26541	2,399	5,26603	2,78083	2,0693	-0,039698	0,794165	0,6279808
CG18405-RA	CG18404	0	0	10,5855	7,18886	12,6221	9,49643	-0,351597	0,267859	0,6279808
CG18405-RB	Sema1a	0,0158734	0,0144586	0,0152395	0,0164174	0,0222375	0,0167595	-0,475093	0,136434	0,6279808
CG18405-RC	Sema1a	0,0150184	0,0136798	0,0144185	0,015502	0,46032	0,0158251	-0,303318	0,356048	0,6279808
CG18408-RA	Sema1a	3,43787	3,89857	3,02904	9,46199	2,30675	2,38887	0,074193	0,758145	0,6279808
CG18408-RB	CAP	0,0253877	117,95	0,0243737	0,0213898	0,0363766	31,0311	0,112029	0,643665	0,6279808
CG18408-RC	CAP	0,0240043	0,0618141	0,0230456	0,0281285	0,0342808	143,99	0,170779	0,487633	0,6279808
CG18408-RD	CAP	0,020459	165,368	0,0196419	0,0267144	0,0289726	72,9928	0,236492	0,345169	0,6279808
CG18408-RE	CAP	0,0265187	0,0353276	0,0254596	0,0235368	0,0381003	16,5897	0,236442	0,345363	0,6279808
CG18408-RF	CAP	0,0252616	0,0206354	0,0242526	11,2202	0,0361849	0,0161247	0,170814	0,487642	0,6279808
CG18408-RG	CAP	0,0224087	0,0206354	0,0215137	0,00803408	0,0318807	4,05889	0,190672	0,440805	0,6279808
CG18408-RH	CAP	0,0101328	0,0200845	9,41191	0,00800188	8,4122	1,88325	0,190568	0,440994	0,6279808
CG18408-RI	CAP	0,00791265	8,78961	0,00759661	0,00780566	0,0108822	13,209	0,191255	0,43782	0,6279808
CG18408-RJ	CAP	0,0078815	0,0355922	0,00756671	0,0199218	0,0108386	71,2056	0,190405	0,441338	0,6279808
CG18408-RK	CAP	0,00769159	0,0231249	0,00738438	0,0211291	0,0105728	0,045189	0,172799	0,482881	0,6279808
CG18408-RL	CAP	0,0191155	0,0218648	0,018352	16,2224	0,0269842	0,0274156	0,07478	0,756237	0,6279808
CG18408-RM	CAP	0,0202211	0,0186355	0,0194135	0,0303156	14,621	0,0258361	0,074129	0,758345	0,6279808
CG18408-RN	CAP	0,0107691	0,0241551	8,84503	106,84	9,67646	0,0218355	0,224256	0,369969	0,6279808
CG18408-RO	CAP	0,0284483	0,02301	0,027312	0,022302	0,0410627	0,0287147	0,18599	0,451227	0,6279808
CG18408-RP	CAP	190,507	0,0204114	101,307	132,151	83,1546	0,0272711	-0,545735	0,010845	0,6279808
CG1840-RA	CAP	0,0212896	0,00922964	0,0204393	174,38	0,0302082	0,0240272	0,111956	0,664813	0,6279808
CG18410-RA	Tango10	1,05188	0,0187867	1,30689	64,8414	2,178	25,8054	-1,033215	0,000265	0,13772387
CG18412-RA	Tango10	6,64187	8,29057	6,70938	0,0240347	2,48685	0,0245355	0,404631	0,160116	0,6279808
CG18417-RA	Ude	50,4159	0,0233492	4,25283	0,0146572	0,0318579	0,0149626	-0,622147	0,0431	0,6279808
CG18418-RA	ph-p	8,82663	9,36129	7,96568	10,9201	11,1387	12,5236	NA	NA	0,6279808
CG1841-RA	CG18417	3,1113	28,9139	4,08299	3,95301	5,58285	5,41498	0,190946	0,409845	0,6279808
CG1841-RB	CG18418	0	0	0	0	1,19427	1,61783	0,183543	0,428438	0,6279808
CG18420-RA	Dhc98D	0,0396013	0,190163	0,0620321	8,80696	6,51083	21,3011	NA	NA	0,6279808
CG18426-RA	CG18420	0	0	0	0	0,254444	0	0,051672	0,815741	0,6279808
CG18428-RA	ytr	31,552	0,209553	0,299751	48,532	0,159602	0,254932	0,065379	0,749901	0,6279808
CG1842-RA	CG18428	23,8249	22,2359	28,7888	1,41921	7,53994	27,3553	-0,343291	0,311692	0,6279808
CG18432-RA	Cc2d2a	0,309647	0,41694	0,0107036	0,0114048	0,326177	0,0116425	-0,587058	0,090735	0,6279808
CG18437-RB	unc80	2,86544	7,98581	2,69529	8,36004	2,90365	2,35456	-0,100995	0,698081	0,6279808
CG18444-RA	SelG	69,3203	87,1082	2,01996	22,7316	9,6411	0,671147	-0,155206	0,632274	0,6279808
CG18445-RA	alphaTry	35,5206	1,71284	66,2131	12,7114	8,77082	19,7472	-0,178433	0,350277	0,6279808
CG18446-RA	oys	17,7139	18,2537	24,5113	18,7664	0,15217	8,35538	-0,771471	0,005594	0,6279808
CG18449-RA	CG18446	2,43915	2,46386	3,99295	6,46561	3,23815	5,85687	0,044335	0,715924	0,6279808
CG1844-RA	CG18449	0	0	0,0784461	0	0	0	-0,225339	0,434555	0,6279808
CG18455-RA	Br140	2,80058	5,26978	2,90722	12,2039	4,10346	6,50512	0,559497	0,038891	0,6279808
CG18455-RB	Optix	7,25719	20,0676	14,9202	8,08827	2,56013	4,25109	0,601863	0,02723	0,13772387
CG1845-RA	Optix	0,0492858	0,00764985	8,8104	0,0553005	1,12089	1,76929	0,016459	0,948984	0,6279808
CG18466-RA	Nmdmc	22,3901	26,1865	5,61855	21,5772	3,23657	4,05378	0,087153	0,733639	0,6279808
CG18466-RB	Nmdmc	7,36823	15,0758	0,017142	9,8899	0,0235644	0,0177596	0,087431	0,733133	0,6279808
CG18467-RA	CG18467	0,170739	107,872	17,0328	18,6264	0,0919616	0,382421	-0,015337	0,965318	0,6279808
CG18468-RA	Dpse\Lhr	0,0590138	0,019188	92,7703	309,627	1384,26	0,0693079	0,109238	0,694008	0,6279808
CG18469-RA	CG18469	0	0	0	0	0	0,28338	NA	NA	0,6279808
CG18471-RA	CG1847	0,0381353	2,53121	129,293	75,8928	56,9647	85,0217	-0,510115	0,043121	0,6279808
CG18472-RA	CG1847	15,1348	95,5829	0,0202049	7,78803	0,0298445	0,0224926	-0,163166	0,634532	0,13772387
CG18473-RA	Dsim\GD11211	2,28589	0,23268	19,1369	0	3,21426	1,9756	0,10331	0,666628	0,6279808

gene_id	Symbol	W1_FPKM	W2_FPKM	W3_FPKM	MCM51_FPKM	MCM52_FPKM	MCM53_FPKM	FC	p-value	p-adj
CG18476-RA	Spag1	0,793092	1,17648	0,0123787	385,402	0,017938	0,0135191	0,059121	0,795007	0,6279808
CG18477-RA	CG18473	11,2541	13,8801	19,4732	13,9092	11,9394	13,3788	NA	NA	0,6279808
CG18478-RA	CG18476	3,07524	3,34062	3,72879	16,5081	25,7568	14,2428	NA	NA	0,6279808
CG1847-RA	CG18477	0,118416	89,2196	1,87082	136,62	5,08084	0,928739	-0,292967	0,275384	0,6279808
CG1847-RB	CG18478	0,074662	0,114932	2,88456	0,175936	0,253589	2,92747	-0,295046	0,27168	0,6279808
CG18480-RA	LIMK1	6,14476	7,60596	3,52229	28,0994	2,5104	2,88486	0,627364	0,002396	0,6279808
CG18482-RA	LIMK1	0,0120716	1,98471	1,74421	0,0275637	0,0167622	1,37134	-0,070526	0,562659	0,6279808
CG1848-RA	LIMK1	1,0486	0,0107926	0,0113754	36,1862	1,82694	0,0123933	0,194973	0,490708	0,6279808
CG1848-RC	CG18480	9,42295	9,25239	12,2696	7,28153	4,99333	0,0981947	0,193288	0,494394	0,6279808
CG1848-RD	CG18482	0	12,3892	0,0335881	0,125757	0,0429602	0,0323774	0,231301	0,414626	0,6279808
CG18490-RB	run	4,33481	0,145848	4,85316	6,4037	2,82805	3,61867	-0,755835	0,00029	0,6279808
CG18490-RC	CG42671	1,02002	0,626053	0,899813	13,9871	0,0990001	0,0172495	-0,756065	0,000284	0,6279808
CG18492-RA	CG42671	3,25788	0,0148657	0,0156686	0,0168974	2,6933	5,55876	0,057573	0,756358	0,6279808
CG18493-RA	Tak1	15,4086	16,6613	17,3647	18,8243	0	0	-0,861454	0,010334	0,6279808
CG18495-RA	CG18493	29,7848	20,0109	40,6215	29,0279	90,4985	67,9785	0,337987	0,320803	0,6279808
CG18495-RB	Prosalpha1	23,1833	15,3053	0,0082389	0,00872677	0,0118205	0,00889801	0,337987	0,320803	0,6279808
CG18497-RA	Prosalpha1	0,0721614	0,0657295	0,00824854	0,00873719	0,0118346	0,00898358	0,361794	0,214661	0,6279808
CG18497-RB	spen	2,17271	20,1429	16,5922	0,00371943	0,0407813	0,0290746	0,361999	0,214367	0,6279808
CG18497-RC	spen	4,28202	1,39622	22,0807	0	0,0407813	0,0312638	0,358818	0,219019	0,6279808
CG1849-RA	spen	10,738	0,00336902	22,5408	21,3618	0,0385779	0,0296122	0,112282	0,652006	0,6279808
CG18505-RA	CG1850	0	11,7343	0,11753	8,04757	0	0	-0,058918	0,821424	0,6279808
CG18507-RA	Acyp2	28,8647	31,8038	0,195631	0,0456781	4,30708	401,111	-0,540502	0,032106	0,6279808
CG18507-RB	CG18507	8,86975	10,2511	0	2,14844	7,1696	0	-0,460374	0,070897	0,6279808
CG18508-RA	CG18507	9,13044	0,066431	3,82427	0,507802	4,43039	0	0,109439	0,692393	0,13772387
CG1850-RA	CG18508	19,6196	14,3649	25,2515	0,0246922	0,0395173	0,0297826	0,294079	0,197693	0,6279808
CG18516-RA	Ady43A	11,8695	27,2311	20,0125	22,6206	12,3873	10,1315	0,025032	0,930023	0,13772387
CG18518-RA	AOX4	0,0438053	0	0	0,0276971	1,5517	1,57552	NA	NA	0,6279808
CG18519-RA	CG18518	0	0	1,70878	16,2156	13,8437	0	0,735971	0,038193	0,6279808
CG18519-RB	AOX2	0,208405	0,20796	0,280564	0,0189971	0	0,12747	0,741585	0,036708	0,6279808
CG1851-RA	AOX2	0,0182648	5,94193	5,59916	3,36971	0,0425912	0,0320993	0,538895	0,011661	0,6279808
CG18522-RA	AOX1	17,129	13,8782	29,3756	28,4519	4,91089	7,0666	-0,911895	0,00274	0,6279808
CG18525-RA	Spn88Ea	23,1614	0,0850092	10,4476	23,8055	8,54254	0,13306	0,437595	0,030642	0,6279808
CG18525-RB	Spn88Ea	78,1591	10,9472	18,0232	0,130344	13,4383	27,9711	0,437595	0,030642	0,6279808
CG18528-RA	CG18528	4,37852	4,00639	0,684324	0,019457	0,0386949	5,29755	-0,243146	0,327301	0,6279808
CG18530-RA	CG18530	0,621226	51,4525	6,62702	0,328658	0,34089	0,256915	0,051196	0,886048	0,6279808
CG18531-RB	Gr2a	0,267802	0,0487866	0,725964	0,0607565	0,082295	0,0620225	0,036914	0,917439	0,13772387
CG18531-RC	Gr2a	0,0560122	0,586728	1,87014	0,531766	0,37413	0,542847	0,036914	0,917439	0,6279808
CG18536-RA	CG18536	0,314313	0,715746	2,80402	8,356	0,506932	0,268744	0,309137	0,353527	0,6279808
CG18536-RB	CG18536	0,0899397	0,0819233	1,00587	2,49347	0,15243	0,11488	0,294585	0,378717	0,6279808
CG18537-RA	CG18537	0,174804	0	0	0	0	0	0,168649	0,4427	0,6279808
CG18538-RA	CG18538	0,180535	0	0	0,410874	0	0	-0,033614	0,827297	0,6279808
CG18539-RA	CG18539	0	0	0,167822	0	0,367266	0	0,01562	0,897983	0,6279808
CG18540-RA	Or43a	0,107826	0	0,0621117	0	0	0	0,024129	0,896871	0,6279808
CG18542-RA	CG18540	0,192081	0,265373	0,170529	0	0,622319	0	-0,546664	0,102145	0,13772387
CG18543-RA	CG18542	0,054533	8,92372	12,8147	1,21862	0,171794	26,2504	0,534299	0,135306	0,6279808
CG18545-RA	mtrm	0,819123	0,525043	2,50485	43,7608	0,0235209	5,33663	0,093692	0,724964	0,6279808
CG18547-RA	CG18545	0,250287	10,3628	0,672814	7,31457	0,0170146	1,90152	-0,875723	0,012644	0,6279808
CG18548-RA	CG18547	7,51152	0,032645	0,0608992	18,0534	6,51661	1,47071	-0,196499	0,536055	0,6279808
CG18549-RA	GstD10	20,2885	20,0622	53,3566	32,9358	33,7567	40,0911	0,276986	0,237862	0,6279808
CG1854-RA	CG18549	20,7658	22,8056	20,8039	0	2,36006	0	0,067432	0,675386	0,6279808
CG18550-RA	yellow-f	0,0476852	30,4619	0,0457806	0,450806	0,0503524	47,4922	-0,1366	0,648534	0,13772387
CG18557-RA	CG18557	0,0943941	33,8584	32,9119	0,189189	5,29684	10,7437	-0,06597	0,818699	0,13772387
CG18558-RB	CG18558	0,0375502	6,77258	0,18229	7,34468	0	0	0,87166	0,013359	0,6279808
CG18559-RA	Cyp309a2	1,67146	1,31533	7,4777	1,40135	1,50432	1,57603	-0,909717	0,010595	0,6279808
CG18561-RA	ttk	6,88713	0,0294214	0,01647	7,5403	9,78656	0,0892672	NA	NA	0,6279808
CG18563-RA	ttk	0,0172537	18,2692	4,5312	10,6412	2,71535	6,27455	-0,004347	0,98913	0,6279808
CG18568-RA	ttk	32,2671	0,0344382	2,38318	20,4045	16,5965	2,8981	0,075669	0,824064	0,6279808
CG1856-RA	ttk	10,0693	19,6068	0,0762461	163,468	7,4403	11,31	0,06072	0,850162	0,6279808
CG1856-RB	ttk	0,0159501	4,54866	426,912	0,0694678	41,1236	4,2108	0,04249	0,895174	0,6279808
CG1856-RC	ttk	0,0171551	4,40389	16,2805	0,989215	7,33825	78,6141	0,295062	0,357828	0,6279808
CG1856-RD	CG18561	0	0	0	0,0944655	0,0296956	0,096434	0,303578	0,343438	0,6279808
CG1856-RE	CG18563	1,73715	1,4358	13,9041	1,96956	10,6741	5,86117	0,048757	0,880027	0,6279808
CG1856-RF	CG18568	0,58853	0,194936	0,333878	0,644899	0,124735	0,0977192	0,297669	0,353927	0,6279808
CG18572-RA	nec	13,7573	11,9927	19,078	0,300746	0,276588	0,185655	-0,336193	0,161449	0,6279808
CG18572-RB	r	0,0147854	0,0134676	0,0141949	0,0154439	0,0209188	0,0157657	-0,73726	0,009139	0,6279808
CG18577-RA	r	4,77135	3,84602	6,16628	8,67941	4,33009	4,57076	-0,323868	0,356397	0,6279808
CG18578-RA	CG18577	11,8821	5,0993	9,28495	19,2907	21,9238	14,0685	-0,10771	0,703901	0,6279808
CG1857-RA	Ugt302C1	25,6189	20,9974	5,62048	4,71054	10,4562	8,78731	-0,216787	0,356221	0,13772387
CG18581-RA	CG18581	60,5696	0	0,0131621	0	0,0191094	84,0848	0,074096	0,806571	0,6279808
CG18582-RA	mbt	10,7648	13,015	13,5028	15,9025	5,54818	7,97461	0,230555	0,379057	0,6279808
CG18584-RA	koi	13,1321	12,6362	13,6481	26,1511	21,9439	15,7703	-0,722142	0,001075	0,6279808
CG18585-RA	CG18585	21,9048	14,6716	0,00973156	15,3064	2,08208	0,0287392	-0,238121	0,468475	0,6279808
CG18586-RA	CG18586	0,0974571	0	8,03772	0,271322	0,144485	16,306	-0,278769	0,323616	0,6279808
CG18591-RA	Spn43Ad	9,85828	7,36033	15,6043	0,305555	0,161533	0,224143	0,014678	0,953901	0,13772387
CG18593-RA	SmE	50,2926	41,5457	77,8192	391,133	594,968	81,8761	-0,021768	0,932335	0,13772387
CG18594-RA	viaf	64,062	59,3873	2,60756	0,0315042	0,103118	0,0777159	-0,298486	0,392627	0,6279808
CG18596-RA	Pebp1	161,046	43,5166	2,64648	162,288	3,18615	2,28403	0,009767	0,966607	0,6279808
CG18598-RA	CG18596	3,54204	24,3921	4,07895	5,42267	0,452364	3,00287	-0,081207	0,797663	0,6279808
CG18599-RA	CG18598	2,46416	3,65774	3,90532	5,51664	3,00678	3,43605	0,333253	0,284338	0,6279808
CG1859-RA	CG18599	1,16142	1,09045	2,45336	1,07988	6,06466	5,35937	-0,685713	0,033509	0,6279808
CG18600-RA	CG18600	15,4611	119,26	24,5533	246,839	22,8369	22,8479	-0,338528	0,296072	0,6279808
CG18604-RA	Sik3	11,0525	12,498	2,98616	11,3787	6,45272	1,74149	0,262428	0,295832	0,6279808
CG18605-RA	CG18605	0,488366	0,0419418	0,46886	0,0636929	3,30626	0,0650202	-0,257946	0,464083	0,6279808
CG18606-RA	AANATL6	0,419529	1,14641	33,2137	4,15469	0,0302082	12,0347	-0,055857	0,869031	0,6279808
CG18607-RA	AANATL4	2,39831	48,2236	8,08232	19,8555	0,0301368	24,9378	-0,636035	0,072595	0,6279808
CG18608-RA	prod	12,1794	0,444837	6,0127	0,0524816	0,476752	0,0535751	-0,010419	0,963194	0,6279808
CG18609-RA	CG18609	13,0883	0,22312	0,0355454	0,0402879	17,4027	0,0411274	-0,073798	0,784859	0,13772387
CG18616-RA	Not10	5,80418	0,385811	87,3798	0	0	0,378864	-0,219152	0,327803	0,13772387
CG18617-RA	Vha100-2	0,0219618	24,4037	0	0,0212518	4,26571	0,0159754	-0,358796	0,093494	0,13772387
CG18617-RB	Vha100-2	69,5962	2,46793	4,20824	0,150602	3,49413	0,0257767	-0,362986	0,09056	0,13772387
CG18619-RA	REPTOR-BP	0,115787	0,105467	0,111162	233,253	0	0,158076	-0,093801	0,760634	0,13772387
CG18619-RB	REPTOR-BP	0,114981	0,104733	0,110388	0,155949	0	240	-0,093906	0,760365	0,6279808
CG18619-RC	REPTOR-BP	211,555	280,221	112,141	2218,69	71,4965	0,159199	-0,093906	0,760365	0,6279808
CG18619-RD	REPTOR-BP	0,115584	0,105282	0,110968	1,85465	7,61539	3,50064	-0,093801	0,760634	0,13772387
CG18622-RA	Ephrin	18,5793	14,0588	2,36536	6,25683	50,3852	0,0307212	0,09937	0,654514	0,6279808
CG18624-RA	Ephrin	0,0282536	0,0257354	11,6567	22,7795	0,0427749	0,0304293	-0,252294	0,418983	0,6279808
CG18624-RB	Ephrin	0,0280022	0,0255063	20,0773	0,0300941	9,62672	0,0290746	-0,250841	0,421596	0,6279808
CG18627-RA	Ephrin	0,026831	0,0244395	0,0271252	0,0298082	26,7675	3,04027	-0,400376	0,136883	0,6279808
CG18628-RA	CG18622	14,1908	0,541245	7,19156	2,09891	4,70939	0,0375462	-0,111163	0,755608	0,6279808
CG1862-RA	Dpse\GA15019	0,1422	150,443	0,13652	14,1106	0,282868	0,213186	-0,07763	0,740572	0,13772387
CG1862-RB	Dpse\GA15019	150,619	1,18013	322,749	2,18881	340,47	398,944	-0,076535	0,744118	0,6279808
CG1862-RC	betaggt-II	14,8566	21,9503	20,1792	42,1222	10,8427	5,02794	-0,076535	0,744118	0,6279808
CG1862-RD	CG18628	1,15005	150,714	52,5453	99,3541	12,4405	16,3456	-0,076535	0,744118	0,6279808
CG18631-RA	Cc2d2a	0,0346491	0,0315608	0	0	42,5567	0,0130168	-0,100453	0,746985	0,6279808

gene_id	Symbol	W1_FPKM	W2_FPKM	W3_FPKM	MCM51_FPKM	MCM52_FPKM	MCM53_FPKM	FC	p-value	p-adj
CG18635-RA	CG18635	4,76477	6,00353	6,23211	7,35915	2,46756	6,63683	-0,248697	0,252609	0,6279808
CG18636-RA	CG18636	0,188788	0,343923	0,181248	8,81462	13,069	9,35705	0,214784	0,459438	0,6279808
CG18641-RA	Hr38	0,399471	0,254201	1,45819	0,0153076	1,45389	0,0156265	0,306656	0,299886	0,6279808
CG18642-RA	Hr38	0,0148363	1,5964	3,11408	7,48089	4,14765	5,25238	0,293094	0,210379	0,13772387
CG18643-RA	CG18641	8,93003	1,49544	1,55461	16,3109	0	7,90179	-0,251509	0,42981	0,6279808
CG18646-RA	Bem46	12,0851	0,00815019	13,0958	0,0204346	2,01254	0,0210234	-0,469461	0,068869	0,6279808
CG18647-RA	Dtd	9,08186	6,57883	8,23854	2,96944	16,2741	13,0002	0,470067	0,04371	0,6279808
CG18649-RA	rad	2,12948	53,1905	51,7314	4,6299	3,77265	2,32235	-0,493663	0,136163	0,6279808
CG1864-RB	bin	3,99088	4,5346	47,5496	0,113115	26,9192	53,628	0,110144	0,758036	0,6279808
CG1864-RC	CG18649	475,376	11,0769	0	9,80107	0	0,369067	-0,055102	0,856545	0,6279808
CG18657-RA	Spn43Ab	23,9595	14,5092	51,2295	0,148073	0,200566	0	0,156182	0,558836	0,6279808
CG18659-RA	Spn43Ab	0,0502987	0,0458156	0,0482898	0,0769664	0,0647207	0	-0,01644	0,938048	0,6279808
CG18659-RB	NetA	15,877	0	18,2328	0,1159	9,85394	10,7555	0,088588	0,678815	0,6279808
CG1865-RA	CG18659	11,6972	6,40503	6,49231	12,6732	0,0449508	7,02291	-0,807546	0,018241	0,6279808
CG1865-RB	CG18659	5,22971	41,7483	9,12921	0,033747	57,9387	0,0344502	-0,807723	0,018207	0,6279808
CG18660-RA	Moca-cyp	12,1715	15,3626	48,9618	21,4095	55,461	14,0948	-0,322804	0,229609	0,6279808
CG18660-RB	Moca-cyp	0,0180848	0,0164729	0,0094743	0,0188019	0,0136333	0,0191937	-0,323043	0,229358	0,6279808
CG18660-RC	Moca-cyp	2,47143	4,39956	16,4778	4,30042	11,1215	2,35832	-0,31244	0,244231	0,6279808
CG18661-RA	Nckx30C	0,0187627	0,0170903	0	17,7701	6,61665	0,0748205	-0,819123	0,021051	0,6279808
CG18662-RA	Nckx30C	1,08001	2,43436	63,0651	0,533938	2,46184	30,6416	-0,358453	0,258642	0,6279808
CG18666-RA	Nckx30C	3,10289	3,02331	0,0514213	0,231415	3,86481	0,207607	0,176265	0,620599	0,6279808
CG18669-RB	CG18661	0,077645	0,0707244	0,0745438	0,101603	0	0	-0,013097	0,965329	0,6279808
CG1866-RA	CG18662	0,157324	0,238835	23,2596	13,6679	11,7153	14,4716	-0,149108	0,477931	0,6279808
CG1866-RB	Dsim\GD23756	0,516218	0,0416035	1,239	0,397321	0	0	-0,153742	0,464077	0,6279808
CG1866-RC	CG42811	1,09488	0,790101	0,4383	2,96669	1,12474	1,7854	-0,124806	0,554623	0,6279808
CG18672-RA	Or98b	0,0858872	0	0	0	0	0	0,362945	0,22663	0,6279808
CG18673-RB	CAH16	0,233319	3,06875	56,9896	12,6618	9,81661	3,04776	0,099631	0,472914	0,6279808
CG18675-RA	CAH5	0	0,050492	0,0752808	40,4086	30,9909	58,7144	0,061164	0,81325	0,6279808
CG18676-RA	CG18675	0,0416707	0,0379565	7,403	6,07999	2,09083	4,1359	-0,648555	0,069429	0,6279808
CG1867-RA	Teh3	0,294325	0,268091	0,0673664	0,0831419	4,57515	0,0848744	0,01562	0,897983	0,6279808
CG18681-RA	Smyd4-1	0,0292803	0,0266705	2,47375	0,031265	0,0423487	0,0319165	0,130195	0,653365	0,6279808
CG18682-RA	Smyd4-1	0,0281094	0,025604	0,0269867	0,0299301	0,0405405	0,0305537	0,707623	0,047924	0,6279808
CG1868-RA	epsilonTry	6,33821	1,45459	200,614	37,3819	12,1751	22,5764	0,278629	0,219151	0,6279808
CG1868-RB	CG45073	0,436144	0,595906	0,690896	0,413087	0,164299	0,165638	0,275015	0,223426	0,6279808
CG1869-RA	Cht7	86,3368	3,4219	79,3803	7,58612	0,0228642	0,0172318	0,413123	0,079866	0,6279808
CG1871-RA	e(r)	12,7744	16,9655	18,3963	44,0315	24,1389	33,4192	-0,159342	0,409348	0,6279808
CG1871-RB	e(r)	26,9143	25,1	33,7336	35,0473	23,2434	15,0582	-0,160737	0,405954	0,6279808
CG18729-RA	Zwilch	0,343989	0,408691	44,8189	20,0642	22,3147	27,9686	-0,143521	0,682124	0,6279808
CG18730-RA	eEF1alpha2	0,025885	0,0235779	0,0248512	0,0274146	0,0373196	2,20686	-0,212262	0,513997	0,6279808
CG18731-RA	eEF1alpha2	0,0259155	0,0236056	0,0248804	0,0274489	3,23925	1,30817	-0,059999	0,784424	0,6279808
CG18734-RA	eEF1alpha2	4,13516	0,0236892	4,29459	5,14463	4,07058	5,04894	0,19823	0,289779	0,6279808
CG18734-RB	eEF1alpha2	0,026038	4,38769	0,0374971	0,0275868	0,0242495	0,0182759	0,197632	0,291453	0,6279808
CG18734-RC	Amy-p	4,56908	0	6,15438	0	0,0272978	0,0205733	0,197632	0,291453	0,6279808
CG18734-RD	CG18731	37,9223	36,0919	36,0388	54,9271	49,9147	18,8284	0,19823	0,289779	0,6279808
CG18734-RE	Fur2	0,0120562	0,0109816	0,0115746	0,0122783	0,929518	0,0126164	0,197632	0,291453	0,6279808
CG18734-RF	Fur2	0,0120364	0,0109636	2,57691	0,0122577	2,1892	1,56593	0,197632	0,291453	0,6279808
CG18734-RG	Fur2	14,443	13,1293	12,1808	0,0121717	1,64033	7,82899	0,235154	0,2113	0,6279808
CG18735-RA	Fur2	0,0119797	0,0109119	0,0115012	0,0121515	0,0167402	0,0125341	0,241626	0,459864	0,6279808
CG1873-RA	Fur2	0,0119602	0,0108941	0,0114825	0,0368208	1,68464	0,0125132	0,058395	0,786818	0,6279808
CG1873-RB	Fur2	0,0118785	0,0108197	0,011404	0,0440005	7,42667	0,0124253	0,059705	0,781927	0,6279808
CG1873-RC	Fur2	0,0118593	0,0108022	0,0113856	46,51	0,016631	0,0124047	0,059705	0,781927	0,6279808
CG1873-RD	CG18735	0,271545	0,0736976	337,015	325,489	0	0	0,058395	0,786818	0,6279808
CG18740-RA	mor	12,873	11,9632	10,0878	12,3364	3,59634	9,73651	0,021179	0,938195	0,6279808
CG18741-RA	Dop1R2	2,12817	1,74342	1,82472	0,0101473	0,0137446	1,25639	0,225037	0,340932	0,6279808
CG18741-RB	Dop1R2	0,0139205	0,0126797	0,0133645	0,0157015	0,0212678	0,0146306	0,054679	0,822053	0,6279808
CG18743-RA	Hsp70Ab	170,478	0,0332032	162,899	0	0	0,0810079	-0,458032	0,144265	0,6279808
CG18744-RB	CG18744	1,67906	0,0334803	0,0352883	0,0379447	6,72531	0,0408031	-0,16521	0,599088	0,6279808
CG18745-RA	CG18745	5,01149	2,22647	17,6634	4,83949	57,9973	9,25505	-0,468346	0,18453	0,6279808
CG18746-RA	CG18746	4,53855	0,430017	5,96765	0,0399702	2,5499	3,79453	-0,970127	0,005823	0,6279808
CG18747-RB	CG18747	7,28044	1,84127	57,4808	30,7669	20,721	60,2495	-0,454745	0,20016	0,6279808
CG18748-RB	CG18748	1,21457	1,52118	5,66019	4,67682	0,0541399	10,0824	-0,617994	0,07685	0,6279808
CG18748-RC	CG18748	2,22421	0,0413463	10,6941	2,74704	0,0513963	7,75975	-0,61935	0,076326	0,6279808
CG18749-RB	CG18749	0,858854	17,1462	5,55948	8,0402	2,01769	4,18736	0,308677	0,30152	0,6279808
CG18754-RA	CG18754	0,589344	5,72634	15,4046	9,9425	0	0	-0,222334	0,534421	0,6279808
CG18764-RA	CG18764	3,70634	0,0386636	13,0699	50,1532	8,38052	7,69664	0,283698	0,251052	0,6279808
CG18765-RB	CG18765	11,3053	9,11348	16,6764	0,0886816	19,8602	35,6538	0,012428	0,961966	0,6279808
CG18766-RA	CG18766	4,79515	3,4942	5,52436	7,08057	3,40045	6,63293	0,180615	0,410709	0,6279808
CG18766-RB	CG18766	16,0998	16,8913	21,7523	19,7214	1,77589	11,7266	0,203136	0,358317	0,6279808
CG18767-RA	mRpL36	33,6626	28,1966	0,0207468	54,0101	102,085	12,7185	-0,534097	0,09621	0,6279808
CG18769-RA	MCU	0,0198188	0,0180523	0,0190272	0,0210414	0,0285007	0,0214799	0,306417	0,142177	0,6279808
CG18769-RB	MCU	1,19008	0,0136354	1,35813	1,33699	1,05334	0,81232	0,293914	0,159162	0,6279808
CG18769-RC	MCU	0,0185138	0,0168637	0,0177744	2,07509	2,00688	0,0199805	0,308051	0,140043	0,6279808
CG18769-RD	MCU	2,53919	0,0172602	3,96594	0,0200613	0,0271731	1,74808	0,305634	0,143292	0,13772387
CG18769-RE	MCU	4,9988	5,03782	5,87345	4,57917	3,79799	4,4709	0,318462	0,132221	0,6279808
CG18769-RF	MCU	1,51656	4,16336	0,0202221	0,022454	0,0304141	0,0229219	0,358087	0,090094	0,13772387
CG18773-RA	Cul1	0,0199988	0,0354733	5,68313	3,78568	0,0576733	2,76575	-0,465051	0,076344	0,6279808
CG18777-RA	Cul1	24,846	1,05329	0,037633	2,11765	2,85898	0,0437773	-0,929412	0,008537	0,6279808
CG18778-RA	Cul1	22,0624	6,8129	0,0399225	0,047523	7,58845	6,06629	-0,259752	0,460587	0,6279808
CG18779-RA	Cul1	0,0201083	0,037877	0,0414349	0,0495978	0,0287764	0,537652	-0,327318	0,315048	0,6279808
CG1877-RA	Lcp65Ab2	43,2155	13,3352	55,8454	2106,71	510,58	1378,88	-0,044482	0,807753	0,6279808
CG1877-RB	Cpr65Ax2	564,219	1,45914	3362,15	3,98996	0	3,67047	-0,046448	0,799488	0,6279808
CG1877-RC	Cpr65Au	4,21143	1,25664	2,92785	3,61789	6,81837	4,98296	-0,046557	0,799021	0,6279808
CG1877-RD	Lcp65Ag3	2759,98	1802,92	4588,56	2356,64	5752,62	4415,6	-0,045157	0,804856	0,6279808
CG18780-RA	CecB	0,488245	0,370606	0,718831	1,05282	0	0,654565	-0,046656	0,879226	0,6279808
CG18783-RA	MED20	3,44411	4,65802	7,68735	4,87413	3,3756	4,79978	0,187851	0,550398	0,6279808
CG18783-RB	Kr-h1	0,0126712	0,0115418	0	0,0130079	4,17997	0,0132789	0,176247	0,579717	0,6279808
CG18783-RC	Kr-h1	6,66439	8,48683	0,0121651	19,9675	0	6,07274	0,193966	0,540976	0,6279808
CG18787-RA	Kr-h1	18,1815	20,5212	6,3056	19,9906	0,992575	8,57635	-0,148552	0,648254	0,6279808
CG18788-RB	CG18787	5,1517	1,82482	1,99716	31,1112	1,14615	1,28867	0,844758	0,00194	0,6279808
CG18789-RA	CG18788	0,0376143	0,0342617	0,036112	0,0409896	0,0555207	0,0418438	0,072161	0,833956	0,6279808
CG1878-RA	CG18789	3,88979	12,1427	10,2791	7,65133	0,0544039	3,78577	0,342208	0,170505	0,6279808
CG18801-RA	Ku80	0,961387	1,28988	1,85349	0	192,702	0	-0,468642	0,087641	0,6279808
CG18802-RA	alpha-Man-IIa	5,20608	4,59152	4,98228	5,50362	0,064885	0,0489013	0,29984	0,252425	0,6279808
CG18803-RA	Psn	10,3895	16,5219	5,73146	0,0414538	6,22569	11,5806	0,025584	0,895794	0,6279808
CG18803-RB	Psn	0,0339025	3,01457	0,0353277	0,0411946	12,7887	92,4292	0,036372	0,853373	0,6279808
CG18803-RC	Psn	9,8404	3,86913	0,0434895	0,0416635	14,0104	23,9243	0,028547	0,883657	0,6279808
CG18806-RA	OS9	0,75712	0,313471	1,6008	2,7502	0,121759	0	0,256972	0,409272	0,6279808
CG18808-RA	CG18808	0,0520693	0,150969	0,0499896	0,0617819	3,50657	0,0630693	0,326421	0,36034	0,6279808
CG18809-RA	CG18809	0,0472082	191,542	74,7712	0,0521493	111,137	0,125606	-0,333915	0,118755	0,6279808
CG18809-RB	CG18809	0,0467738	60,1865	399,67	78,2925	0	452,559	-0,333915	0,118755	0,6279808
CG18810-RA	CG18810	0,179945	11,4824	0,276413	0,368446	0,712577	0,227141	0,23419	0,445854	0,6279808
CG18811-RA	Capr	14,4514	1,9284	0,29923	7,86683	1,98109	1,76148	0,011913	0,961951	0,6279808
CG18812-RA	Gdap2	19,3104	22,8764	3,00905	24,9374	12,5656	0,00890862	0,578351	0,002072	0,6279808
CG18812-RB	Gdap2	24,1583	29,0702	25,3415	0,0402879	7,6417	0,00891925	0,605043	0,001308	0,6279808

gene_id	Symbol	W1_FPKM	W2_FPKM	W3_FPKM	MCM51_FPKM	MCM52_FPKM	MCM53_FPKM	FC	p-value	p-adj
CG18812-RC	Gdap2	14,6413	16,8919	22,6602	24,8554	13,8452	0,00891925	0,49454	0,009358	0,6279808
CG18814-RA	CG18814	5,89138	104,204	186,211	133,255	8,28585	11,1884	2,531382	1,86E-19	0,6279808
CG18815-RA	CG18815	0,0570767	19,0713	6,53547	10,5525	9,41561	12,6829	-0,220308	0,405291	0,6279808
CG18815-RB	CG18815	23,2859	20,8195	0,054797	15,2111	0,0884966	5,84572	-0,219877	0,406569	0,6279808
CG18815-RC	CG18815	23,7302	11,8605	38,0837	7,09753	48,2193	0,0666964	-0,218621	0,408815	0,6279808
CG18816-RA	Tsp42Eb	0,0441192	0,0401868	0,042357	0,0488622	0,0661841	0,0498804	-0,639893	0,053727	0,6279808
CG18816-RB	Tsp42Eb	37,744	30,9232	54,8052	44,1392	87,4654	65,6528	-0,639893	0,053727	0,6279808
CG18817-RA	Tsp42Ea	143,253	155,991	108,216	144,016	196,951	163,079	-0,252522	0,330173	0,6279808
CG18817-RB	Tsp42Ea	0,0592078	0,0539305	0,056843	0,0721294	0,0976997	0,0736324	-0,252522	0,330173	0,6279808
CG18817-RC	Tsp42Ea	143,302	155,303	115,461	144,279	208,23	172,589	-0,252005	0,331471	0,6279808
CG18823-RA	CG1882	0,0413577	5,274	0,00824854	0,00873719	0,0118346	0,00891925	-0,013096	0,914398	0,6279808
CG1882-RA	CG1882	0,0403229	0,0367289	0,0082389	0,00872677	0,0118205	0,0089157	-0,264933	0,255201	0,6279808
CG1882-RB	CG1882	10,597	13,3296	0,00824854	0,00873719	0,0118346	0,00890508	-0,240177	0,303968	0,6279808
CG1882-RC	CG1882	13,8388	20,0861	0,00824854	0,00873719	0,0118346	0,0089157	-0,250324	0,283437	0,6279808
CG1882-RD	CG18823	0	0,341322	1,5712	17,8626	0	0,855476	-0,25473	0,273132	0,6279808
CG1883-RA	RpS7	0,0705435	0,0796467	2,14133	3,13815	31,1899	33,1575	-0,282757	0,284951	0,6279808
CG1883-RB	RpS7	1922,31	0,0829394	27,3479	7,65799	0,374019	3,3596	-0,362313	0,180603	0,6279808
CG1883-RC	RpS7	0,0874403	2090,37	57,438	20,2735	77,5864	79,7657	-0,28282	0,284788	0,6279808
CG1883-RD	RpS7	0,0910552	0,0277399	8,22329	54,63	1,99008	1,9809	-0,282757	0,284951	0,6279808
CG18853-RA	Uros1	4,34937	3,83053	5,25414	4,22727	6,18817	8,56098	0,797063	0,00874	0,6279808
CG18854-RA	CG18853	7,39699	9,29429	0,00824532	0,00873371	0,0118299	0,0089157	-0,612646	0,008736	0,6279808
CG18854-RB	hpRNA:CR18854	90,7088	83,59	0,0251168	8,72797	7,65968	6,73005	-0,612646	0,008736	0,6279808
CG18854-RC	hpRNA:CR18854	0,0262344	0,0238962	31,8201	3,4883	0,0312774	0,0235725	-0,612532	0,008759	0,6279808
CG18858-RA	hpRNA:CR18854	0,0261617	0,0238299	0,128241	0,0224082	0,030352	0,0228751	0,052825	0,829417	0,6279808
CG18859-RB	CG18858	8,52184	0,0421277	11,6034	0	0,0364807	110,408	0,01562	0,897983	0,6279808
CG1885-RA	Or19a	0,141916	16,5666	0,0362358	8,69859	34,5302	0	-0,421329	0,174202	0,6279808
CG18869-RA	ATP7	7,10081	16,6664	0,0366122	15,8174	0,0563622	0,418884	0,45418	0,04394	0,13772387
CG1886-RB	Dsim\GD13807	5,0749	44,489	7,01402	39,2247	3,56057	0,0253016	-0,169496	0,547282	0,6279808
CG18870-RA	dsb	2,3895	2,48581	2,22162	45,907	10,5959	4,1586	0,219562	0,378242	0,6279808
CG1887-RD	CG18870	4,64673	14,7745	3,89163	4,88458	0	5,32595	-0,253535	0,387789	0,6279808
CG1888-RA	CG1888	13,0785	1,36443	17,1242	20,5758	10,1907	1,06767	0,231532	0,382074	0,6279808
CG1889-RA	CG1889	0,0538077	0,0490118	0,0516586	0,0642519	0,0870296	0,0655908	0,759981	0,003863	0,6279808
CG1889-RB	CG1889	0,053633	0,0488527	0,0514909	0,0640023	0,0866915	0,0653359	0,654211	0,029946	0,6279808
CG1889-RC	CG1889	2,51688	2,67464	2,1255	1,52394	1,66714	1,21907	0,654211	0,029946	0,6279808
CG1890-RA	CG1890	11,6689	8,61682	17,2423	22,0798	0,0371797	0,0280209	-0,29137	0,28952	0,6279808
CG1891-RA	sax	16,6537	18,6602	0,00825176	13,4382	0,0118393	0,00890626	0,020982	0,929356	0,6279808
CG1891-RB	sax	0,0247877	0,0225784	0,00825176	0,00874066	0,0118393	0,0089228	0,02254	0,924149	0,6279808
CG1893-RA	scramb2	30,7905	0,0359823	2479,21	2500,76	0,0324251	0,0244375	0,361503	0,057366	0,6279808
CG1894-RA	CG1894	0,130482	0,0740418	0,150324	8,8684	0,0612646	8,12486	0,157473	0,590553	0,6279808
CG1895-RA	Cyp28c1	0,282934	0,654201	0,501477	0,694471	1,04905	0,383487	0,246166	0,490063	0,6279808
CG1896-RA	CG1896	0,0794181	0,617716	0,0165646	0,10467	3,14024	6,24423	-0,279911	0,33388	0,6279808
CG1897-RA	Dr	12,5932	15,8477	37,641	82,4828	38,7813	46,6457	0,580376	0,034449	0,6279808
CG1898-RA	HBS1	8,41263	8,19598	10,7427	216,425	7,39828	1,36275	-0,170173	0,41083	0,6279808
CG1900-RA	Rab40	9,41688	0,0054302	0,00572345	0,00602602	0,00816229	0,00595446	0,153787	0,513998	0,6279808
CG1900-RB	Rab40	0,042176	0,00510545	0,00538117	0,00566103	0,0076679	4,05186	0,153787	0,513998	0,6279808
CG1901-RA	mav	30,5928	31,3968	30,4223	23,93	0,0255988	0,0192928	0,432083	0,036484	0,6279808
CG1901-RB	mav	20,4428	22,9186	21,4311	26,3091	18,4255	17,0751	0,432083	0,036484	0,6279808
CG1901-RC	mav	0,0253683	0,0231072	0,024355	0,0268341	0,0230889	0,0174012	0,478454	0,022552	0,6279808
CG1902-RA	CG1902	48,6158	44,9142	41,6339	0,041506	17,2049	0,0423709	0,226671	0,240233	0,6279808
CG1902-RC	CG1902	0,0380475	0,0346563	9,78947	0,0308985	0,0476634	0,0315423	0,227397	0,237831	0,6279808
CG1903-RA	sno	6,02241	7,18912	6,89337	9,90011	0,0146204	0,736981	-0,488194	0,114164	0,6279808
CG1903-RB	sno	0,0105659	0,00962419	0,0101439	0,0107939	0,961432	24,1482	0,008356	0,972477	0,6279808
CG1903-RC	sno	0,0097261	0,0088592	0,00933763	0,00991669	0,0609835	24,2486	0,002922	0,990353	0,6279808
CG1903-RD	sno	0,040973	0,0373211	0,0393365	0,0450227	79,776	80,9775	-0,003873	0,98712	0,6279808
CG1906-RA	alph	0,041203	0,0442656	1,22073	14,0573	4,6699	3,96373	-0,099725	0,655564	0,6279808
CG1906-RB	alph	38,3681	0,604552	0,927932	2,58897	4,86709	2,37869	0,159554	0,448921	0,6279808
CG1906-RC	alph	0,0403229	8,97424	8,04452	2,9455	11,122	4,79577	-0,082972	0,708949	0,6279808
CG1906-RD	alph	0,0460245	1,52067	7,6464	6,12176	500,532	1,10969	-0,082972	0,708949	0,6279808
CG1906-RE	alph	0,0485971	5,3424	13,4907	2,74563	1,47241	7,94464	-0,138766	0,537595	0,6279808
CG1907-RA	CG1907	24,6886	22,67	36,2825	0,0110584	0,0149787	35,53	-0,335302	0,233698	0,6279808
CG1908-RA	Lint-1	8,86371	0,109165	0	0,254926	1,99218	0,229598	-0,131226	0,519616	0,6279808
CG1909-RA	CG1909	3,85755	1,8851	0,0268838	4,49409	20,1896	6,46659	-0,103457	0,66375	0,13772387
CG1909-RB	CG1909	8,91836	9,92721	0,0257594	141,866	0,0437392	6,40552	-0,101276	0,670477	0,6279808
CG1910-RA	CG1910	0,0323004	16,0374	4,75029	3,53091	0,0940946	0,106851	-0,040546	0,87563	0,6279808
CG1910-RB	CG1910	25,5614	0,0157158	0,0310103	8,54046	0,637099	473,614	-0,008203	0,974725	0,6279808
CG1910-RC	CG1910	0,037808	38,6987	27,608	9,00517	0,141776	0,0354674	-0,004496	0,986199	0,6279808
CG1910-RD	CG1910	16,5028	11,3449	0,036298	20,4351	423,084	21,2327	-0,00598	0,981644	0,6279808
CG1911-RA	Cap-D2	1,8363	1,57424	3,8148	3,90874	0,0693737	0,0522842	0,024031	0,939064	0,6279808
CG1912-RA	Gycalpha99B	8,58959	0,0367289	0,0466561	20,2626	5,00409	5,6457	0,270209	0,191778	0,6279808
CG1913-RA	alphaTub84B	690,356	0,297048	0,0367394	0,116135	0	0	-0,131453	0,525446	0,6279808
CG1915-RA	sls	14,9601	21,4256	0,0550347	10,4923	7,8182	0,0670287	0,280471	0,233036	0,6279808
CG1915-RC	sls	12,1635	10,9706	0,304009	15,1279	5,12217	0,494311	0,456769	0,067504	0,6279808
CG1916-RA	Wnt2	4,76608	0,925922	6,03485	0,0107376	0,0133779	0,0100824	0,13446	0,587958	0,13772387
CG1919-RA	Cpr62Bc	217,852	287,599	312,285	36,6053	1,3846	9,50222	0,550211	0,006342	0,6279808
CG1921-RB	sty	62,5613	70,547	0,0760024	0,00494923	4,01981	0,0981221	0,264174	0,265937	0,6279808
CG1921-RC	sty	16,6138	19,4298	2035	23,1185	0,00670376	2004,4	0,605839	0,020414	0,6279808
CG1922-RA	onecut	12,3134	11,5174	8,08971	13,4535	0,0417543	144,359	0,080169	0,75877	0,6279808
CG1924-RA	CG1924	0	0	0,00758209	0,446134	6,26687	2,31392	-0,115958	0,399535	0,6279808
CG1925-RA	mus205	0,00934639	11,2184	0,00823569	0,00872331	0,0118158	0,00897279	0,692872	0,018702	0,6279808
CG1927-RA	CG1927	68,6683	79,6925	107,979	104,317	103,52	105,994	-0,300347	0,203698	0,6279808
CG1934-RA	ImpE2	142,79	128,033	146,946	259,262	11,4309	145,747	-0,058647	0,853609	0,6279808
CG1935-RA	JTBR	22,5877	9,14374	52,1884	0,0832469	4,96659	46,309	0,129342	0,605548	0,6279808
CG1937-RA	sip3	14,944	10,9516	10,1748	0,0164996	4,26253	81,05	0,204884	0,433642	0,6279808
CG1938-RA	Dlic	6,67097	0,250916	0,0287217	0,0614363	20,5351	0,0565953	-0,274438	0,204305	0,6279808
CG1938-RB	Dlic	0,0299166	6,75304	0,0282108	4,2681	4,96733	0,0613998	-0,247419	0,260341	0,6279808
CG1938-RC	Dlic	0,0293844	0,0272501	3,83759	0	6,1754	0,0593414	-0,276226	0,201359	0,13772387
CG1938-RD	Dlic	4,84998	0,0267654	1,49292	2,64127	182,234	0,0562169	-0,275094	0,20316	0,6279808
CG1939-RA	Dpck	39,631	35,8995	0,0131894	0,0141748	0,0191998	0,0151173	-0,388008	0,163499	0,6279808
CG1941-RA	CG1941	18,068	30,248	4,16592	15,7726	5,85188	4,39171	-0,079409	0,782234	0,6279808
CG1942-RA	Dgat2	2,91818	14,2285	23,1452	2,09625	7,69227	8,57163	-0,416475	0,200305	0,6279808
CG1943-RA	CG1943	49,1361	75,7336	84,751	67,5419	25,2614	0,0144329	0,893457	4,82E-05	0,6279808
CG1943-RB	CG1943	0,0707702	77,3124	0,0679436	0,0839897	0,113765	0,0144701	0,895175	4,62E-05	0,6279808
CG1943-RC	CG1943	46,3694	0,0644624	40,1408	22,7864	22,5927	0,0143131	0,895075	4,62E-05	0,6279808
CG1944-RA	Cyp4p2	0,731675	15,4128	16,2685	4,97855	10,5929	9,63913	-0,480542	0,179367	0,6279808
CG1945-RA	faf	0,00767016	0,25165	0,00736381	0,00778353	1787,8	66,272	0,039429	0,859379	0,6279808
CG1945-RC	faf	4,91365	2,06324	5,23336	6,89145	0,118445	0,0204544	-0,115068	0,615874	0,6279808
CG1946-RA	CG1946	0,696146	6,05815	1,05821	10,832	1,3792	0,0201541	-0,765428	0,023704	0,6279808
CG1950-RA	Uch-L5R	0	0,543012	15,8491	0,912734	0,605176	0,00817657	-0,041811	0,731452	0,6279808
CG1951-RA	CG1951	6,00504	31,4563	5,29457	30,2375	0,0691051	17,9082	0,131276	0,564155	0,6279808
CG1954-RA	Pkc98E	18,5822	0,0806789	0,026399	0,0299437	0,461198	11,0318	0,304158	0,114972	0,6279808
CG1956-RA	Rap1	79,04	84,1006	82,4192	0,102712	3,63749	1,28707	0,337302	0,080394	0,6279808
CG1957-RA	Cpsf100	0,0274972	9,56001	0,340144	7,97581	1,41083	1,09912	-0,018686	0,940108	0,6279808
CG1957-RB	Cpsf100	4,00641	4,02168	20,77	0,232363	0,950646	1,28608	-0,00686	0,978236	0,6279808
CG1958-RA	CG1958	0,202687	0,276932	0,421615	1,17804	11,6284	0,68168	-0,752368	0,035103	0,6279808

gene_id	Symbol	W1_FPKM	W2_FPKM	W3_FPKM	MCM51_FPKM	MCM52_FPKM	MCM53_FPKM	FC	p-value	p-adj
CG1960-RA	mu2	0,0163608	0,0149026	1,72781	2,01592	0,0229464	0,0172938	0,058727	0,821975	0,6279808
CG1960-RB	mu2	0,692709	0,895093	0,0154661	4,37924	0,800019	1,12741	0,058727	0,821975	0,6279808
CG1962-RA	Cen	9,90763	14,0256	6,73427	7,13207	6,56372	3,94074	0,416342	0,046545	0,6279808
CG1962-RB	Cen	3,76741	0,0215825	0,0235766	0,0259261	1,40578	0,0264663	0,416788	0,046092	0,6279808
CG1962-RC	Cen	0,0250889	0,0228527	11,8719	15,3463	4,84866	6,57436	0,388231	0,065514	0,6279808
CG1963-RA	Pcd	21,3133	55,0731	36,9103	5,00557	16,6046	43,645	-0,357595	0,242453	0,6279808
CG1964-RA	Kul	2,25552	2,12622	0,0529669	43,3102	8,79492	2,01314	-0,528316	0,040491	0,6279808
CG1965-RA	CG1965	8,20647	9,15545	0,0299938	8,99298	0,0466665	0,0351707	0,282221	0,220558	0,6279808
CG1966-RA	Acf	1,7894	1,38573	3,95702	16,7593	0,0558333	9,17449	-1,111353	0,000254	0,6279808
CG1967-RA	p24-1	54,5844	0,0202398	2,41551	62,6699	46,4756	26,042	0,038968	0,871535	0,6279808
CG1968-RA	CG1968	6,77686	0,0151501	5,39994	0,0349832	4,4183	6,31736	-0,051542	0,811464	0,6279808
CG1968-RB	CG1968	0,032507	0,905702	0,0148194	12,4564	2,05694	1,90194	-0,051873	0,809562	0,6279808
CG1969-RA	Gnpnat	8,34138	1,95837	43,8376	4,0977	12,952	3,38121	-0,292596	0,286357	0,6279808
CG1969-RB	Gnpnat	33,6752	6,19447	0,70187	12,7175	22,0477	22,6011	-0,291089	0,289443	0,6279808
CG1970-RA	Dpse\GA25154	129,421	107,007	174,201	8,2815	2,91887	12,3421	-0,138996	0,565204	0,6279808
CG1971-RA	CG1971	0,270885	242,711	1,22674	23,8027	0,0470602	0,0186768	-0,13304	0,705951	0,6279808
CG1971-RB	CG1971	0,0690687	8,64588	10,3768	8,88932	20,3452	10,0471	-0,132309	0,705566	0,6279808
CG1972-RA	IntS11	10,7577	25,3448	3,46436	30,9595	27,6907	39,5467	-0,192684	0,443093	0,6279808
CG1973-RA	yata	9,99663	14,1251	8,12593	12,5533	5,62146	5,79001	0,310298	0,265332	0,6279808
CG1975-RA	Drep2	7,95846	4,86567	8,2175	8,28122	13,5439	5,6314	0,23152	0,208628	0,6279808
CG1975-RB	Drep2	0,0138883	0,0140601	0,0312086	0,0142978	5,27315	0,0145957	0,26413	0,162983	0,6279808
CG1976-RA	RhoGAP100F	6,86018	24,4119	5,38071	10,2535	4,57596	0,00794573	0,159794	0,551428	0,6279808
CG1977-RA	alpha-Spec	51,2674	45,7856	0,0160898	2,98233	21,5207	51,6432	-0,31397	0,221422	0,6279808
CG1978-RA	Or45a	0,348685	0,317607	0,167379	0,433328	5,84693	4,95307	0,150118	0,670941	0,6279808
CG1979-RA	CG1979	0,376198	0,395386	0,611216	18,8008	0,0384284	1,20644	-1,381998	2,7E-06	0,6279808
CG1980-RA	dj	0	0,0551831	0	0	0	0	0,129354	0,390779	0,6279808
CG1980-RB	dj	0	0,252963	28,7477	0	0	57,1583	0,129354	0,390779	0,6279808
CG1981-RA	Thd1	17,5605	20,4892	0,0168416	0,0212861	7,83905	8,33357	0,285025	0,31419	0,6279808
CG1982-RA	Sodh-1	10,6397	0,41111	0,317762	0,177781	0,0380678	0	-1,071897	0,002316	0,6279808
CG1983-RA	CG1983	9,58783	12,9512	15,8734	12,8798	12,8297	13,1074	-0,03059	0,899154	0,6279808
CG1984-RA	djl	0	7,3065	0	1,20523	0	0	0,460854	0,126257	0,6279808
CG1986-RA	CG1986	16,5879	30,156	19,0412	187,216	14,8903	19,7358	0,531127	0,0677	0,6279808
CG1987-RA	Rbp1-like	35,6696	23,8738	54,867	40,4966	33,1582	41,4993	0,049321	0,856195	0,6279808
CG1988-RA	CG1988	0,0320497	0,0291931	7,27813	0,0344528	5,33349	5,40316	-0,136871	0,673315	0,6279808
CG1988-RB	CG1988	0,0343846	0,0313199	22,2505	0,037174	14,3452	15,3648	-0,136871	0,673315	0,6279808
CG1989-RA	Yippee	0,0543089	0,0494683	0,0521397	37,9485	0,462406	0	-0,127637	0,538318	0,6279808
CG1989-RB	Yippee	24,1627	31,6362	28,1065	7,26795	0,0836056	12,7761	-0,128075	0,536999	0,6279808
CG1994-RA	l(1)G0020	6,29858	8,99765	11,2302	0,343968	18,9496	3,74466	-0,094523	0,736154	0,6279808
CG1998-RA	CG1998	1,45673	1,73517	2,09782	2,45601	0,19276	2,08129	-0,235074	0,367809	0,6279808
CG1999-RA	CG1999	0,0900216	24,6779	21,9641	0	85,1873	0	-0,040292	0,831087	0,6279808
CG2003-RA	CG2003	5,32339	7,18358	0,018837	0	0	0	0,144118	0,575202	0,6279808
CG2003-RB	CG2003	13,9694	8,89169	0,0269752	0	0	0	0,143367	0,5773	0,6279808
CG2004-RA	CG2004	9,2893	19,7671	17,3155	15,5706	5,60761	7,68783	-0,395425	0,155142	0,6279808
CG2006-RA	CG2006	1,74483	0,0292926	0	0	68,5352	0,372317	-0,031523	0,881983	0,6279808
CG2009-RA	bip2	46,5605	42,857	53,747	80,7138	0,0233242	0,0175786	0,471135	0,038839	0,6279808
CG2010-RA	CG2010	0,0295113	0,026881	7,26829	6,24512	0,72201	2,19535	0,312767	0,136479	0,6279808
CG2010-RB	CG2010	16,493	18,4122	4,89886	2,6564	38,1731	5,89694	0,337342	0,109087	0,6279808
CG2013-RA	Ubc6	69,2898	68,0671	0,697805	0,376873	17,9402	0,354646	0,275708	0,142265	0,6279808
CG2014-RA	Dpse\GA15185	0,155108	0,0500025	0,0232995	5,19654	7,6607	6,1366	-0,026156	0,875963	0,6279808
CG2016-RB	CG2016	43,5844	0,0243999	70,8655	2,08795	71,0414	45,4632	0,193739	0,481187	0,6279808
CG2017-RA	CG2017	0,0315147	0,0287058	0,030256	0,0338336	0,0458278	0,0309759	0,024076	0,917583	0,6279808
CG2017-RB	CG2017	0,0284728	0,025935	0,0273356	0,0303436	0,0411005	2,80942	0,025432	0,913074	0,6279808
CG2017-RC	CG2017	3,08727	0,0278426	3,62426	5,63212	0,0443477	14,9159	0,022885	0,921612	0,6279808
CG2017-RD	CG2017	19,0121	28,0521	17,2839	21,4313	16,1901	4,55515	0,02988	0,897726	0,6279808
CG2017-RE	CG2017	3,27782	7,7788	5,41889	9,44932	7,39243	3249,51	0,027928	0,90433	0,6279808
CG2019-RA	disp	3,33962	5,2946	9,73015	5,90033	0	0	0,342611	0,167655	0,6279808
CG2021-RA	CG2021	18,7747	0,00517036	26,5133	0,00573394	15,6182	6,95532	0,043612	0,884807	0,6279808
CG2022-RA	plh	0,219765	0,266902	67,1881	45,5667	0,46743	0,232336	0,106036	0,743733	0,6279808
CG2023-RA	CG2023	8,97818	8,17795	2,0165	14,479	4,87472	3,74588	-0,072105	0,769447	0,6279808
CG2025-RA	Nrd1	6,71045	53,4475	76,8826	0,591616	5,14436	3,0603	0,248731	0,345593	0,6279808
CG2028-RA	CkIalpha	0,0300937	0,0274115	0,0288918	2,30091	1,70109	2,01228	0,073727	0,694022	0,6279808
CG2028-RB	CkIalpha	45,7618	40,4077	48,7732	4,56825	2,65945	4,02323	0,01926	0,91654	0,6279808
CG2028-RC	CkIalpha	95,3221	93,2814	89,1845	2,31863	1,15526	1,216	0,050588	0,785118	0,6279808
CG2028-RD	CkIalpha	0,0319258	0,0290803	0,0306507	6,50561	5,02345	3,81992	0,042641	0,818357	0,6279808
CG2031-RA	Hpr1	6,49082	6,00613	9,26991	0,0719718	0	0	0,283518	0,220726	0,6279808
CG2033-RA	RpS15Aa	2025,9	2245,18	2885,11	3282,12	3765,45	3431,96	-0,278506	0,241081	0,6279808
CG2033-RC	RpS15Aa	0,0967906	0,0881635	0,0929247	0,123503	0,167286	0,126077	-0,278506	0,241081	0,6279808
CG2033-RD	RpS15Aa	0,106631	0,0971269	0,102372	0,14004	0,189685	0,142958	-0,278506	0,241081	0,6279808
CG2033-RE	RpS15Aa	0,104275	0,094981	0,10011	0,135994	0,184204	0,138827	-0,278506	0,241081	0,6279808
CG2034-RA	CG2034	10,1307	8,27117	15,8938	0	0,108973	47,4831	0,149002	0,587637	0,6279808
CG2038-RA	CSN7	20,7354	1,10168	35,8433	3,52482	7,03874	19,4061	0,322508	0,188944	0,6279808
CG2040-RA	hig	4,11632	2,01109	0,0159683	7,88415	0,633803	0,459748	-0,374327	0,066458	0,6279808
CG2040-RB	hig	0,0154359	5,77459	7,47481	0,0159485	0,308675	0,232636	-0,374656	0,066658	0,13772387
CG2040-RC	hig	8,43395	1,96904	20,9122	11,199	4,97012	3,41459	-0,373021	0,067865	0,13772387
CG2040-RD	hig	0,0166326	7,57168	6,07005	0,0172332	0,0216024	0,0162809	-0,3727	0,067669	0,6279808
CG2041-RA	lgs	12,2251	12,7752	13,3645	17,2699	0,119716	0,0095553	0,126699	0,601468	0,6279808
CG2043-RA	Lcp3	4,5833	20,4187	7,94488	2,76788	2,48606	53,9043	0,685903	0,053944	0,6279808
CG2044-RA	Lcp4	27,0528	1,25913	4,01931	9,44242	5,42656	5,6962	2,208977	7,22E-19	0,13772387
CG2045-RA	Ser7	0,578395	9,89367	14,9178	0,0497851	18,6068	20,0311	-0,040048	0,905689	0,6279808
CG2046-RA	CG2046	14,5903	11,492	2,86072	4,45933	8,13097	11,445	0,214417	0,359435	0,6279808
CG2047-RA	ftz	0,150343	0,0338189	0,631481	0,508319	0,0860651	0,139762	0,154401	0,638459	0,6279808
CG2048-RA	dco	0,0207807	1,83315	3,57627	1,87148	0,0564335	13,5326	0,164592	0,465703	0,6279808
CG2048-RB	dco	14,185	0,0189285	5,01762	5,38766	1,1203	2,61964	0,162654	0,471694	0,6279808
CG2048-RC	dco	23,4177	19,128	11,7025	0,0372581	1,99659	0,0380344	0,162989	0,47056	0,6279808
CG2049-RB	Pkn	12,618	0,0114062	6,44307	0,849496	0,0174062	0,0131184	-0,108372	0,649648	0,6279808
CG2049-RC	Pkn	0,0124468	9,33467	1,81037	6,56945	4,87743	6,11378	-0,119012	0,616813	0,6279808
CG2049-RD	Pkn	0,0142846	0,0116761	1,85826	2,66719	0,0178304	0,0134381	-0,121545	0,60852	0,6279808
CG2049-RE	Pkn	0,0125223	15,1503	0,0120221	5,03107	11,0464	12,4402	0,127423	0,597742	0,6279808
CG2049-RF	Pkn	8,29677	1,28056	7,06392	0,0128506	3,36947	2,53944	-0,110821	0,641475	0,6279808
CG2049-RG	Pkn	0,0128186	1,82999	0,0123067	13,8055	1,6498	1,50468	-0,128211	0,586037	0,6279808
CG2050-RA	mod	110,978	77,2239	0	0	0	0	0,056796	0,841678	0,6279808
CG2051-RA	Hat1	5,8012	6,11848	0,115409	0,0399944	0,222837	0,167943	0,091053	0,740455	0,6279808
CG2051-RB	Hat1	0,0367769	0,0334989	29,0988	0,164515	3,96221	2,45304	0,093156	0,734709	0,6279808
CG2051-RC	Hat1	0,12021	0,109496	8,79806	27,8203	2,29449	19,6971	-0,093537	0,763043	0,6279808
CG2052-RA	dati	0,0172087	0,0156749	1,37128	0,0178543	3730,04	0,00958116	0,347758	0,14946	0,6279808
CG2052-RB	dati	4,99454	6,03472	3,4344	4,84872	0,117509	10,7642	0,349382	0,147741	0,6279808
CG2053-RA	CG2053	0,251558	0,203677	0,134173	0,318592	0	0,165922	0,145401	0,63065	0,6279808
CG2054-RA	Cht2	28,9006	30,0013	35,4386	2,46592	3,32044	3,81873	-0,282447	0,172255	0,6279808
CG2056-RA	spirit	0,0439139	3,57998	0,04216	0,0486099	1,74059	2,60978	-0,685493	0,013796	0,6279808
CG2056-RB	spirit	3,5654	0,039605	4,36223	5,2898	6,42591	2,85999	-0,681994	0,014633	0,6279808
CG2059-RA	CG2059	10,1929	10,94	13,6865	0,0227721	0,841198	0,0206617	0,000603	0,998057	0,6279808
CG2060-RA	Cyp4e2	12,3177	9,21805	16,5241	3,61816	8,65056	9,90914	0,240768	0,322091	0,6279808
CG2061-RA	CG2061	0,0321276	0,0449488	0,00624952	1,84076	0,0467888	0,00672545	0,462057	0,074955	0,6279808
CG2061-RB	CG2061	0,0340656	507,565	0	0,868891	1,14436	0,420233	0,494765	0,057257	0,6279808

gene_id	Symbol	W1_FPKM	W2_FPKM	W3_FPKM	MCM51_FPKM	MCM52_FPKM	MCM53_FPKM	FC	p-value	p-adj
CG2061-RC	CG2061	3,1435	0,0353483	0	44,4029	1,32778	0,32088	0,462057	0,074955	0,6279808
CG2062-RA	Cyp4e1	8,89731	3,65401	43,6904	15,0475	46,5015	39,556	0,430004	0,104065	0,6279808
CG2063-RA	CG2063	23,0574	0,0325743	0,0343334	26,6883	21,3509	0,0396019	0,042008	0,847642	0,6279808
CG2064-RA	CG2064	5,74117	23,0874	25,5849	34,3285	67,7567	68,6378	-1,083994	0,001633	0,6279808
CG2065-RA	CG2065	15,8637	11,9129	33,5271	23,3446	28,8668	24,5935	-0,337627	0,266217	0,6279808
CG2069-RA	Oseg4	3,25043	12,826	9,46868	33,4322	0,00776666	37,358	-0,326626	0,25503	0,6279808
CG2070-RA	CG2070	2,15131	1,53965	3,06857	3,11778	0,0367813	0,063546	-0,701132	0,036472	0,6279808
CG2071-RA	Ser6	13,2666	1,14193	0,741084	16,7847	7,76108	49,1513	-0,666144	0,062062	0,6279808
CG2072-RA	Mad1	8,40957	6,89487	7,43794	21,399	5,09788	6,52097	0,031479	0,89786	0,6279808
CG2075-RA	aly	0,0437203	0,0165955	0,0174918	5,63367	0,025665	0,0193427	0,139669	0,390249	0,6279808
CG2076-RA	CG2076	24,6278	0,0430211	10,3173	0,0345431	36,922	8,82897	-0,18821	0,417186	0,6279808
CG2078-RA	Myd88	14,2531	9,43486	10,428	10,6035	0,0193664	13,635	-0,179496	0,333557	0,6279808
CG2079-RA	Dok	5,3171	7,04072	1,26283	8,18893	0	0,0938768	0,091488	0,684654	0,6279808
CG2081-RB	Vago	0,0951186	0,0463686	2,14601	5,96434	5,90457	3,71277	-0,750854	0,027016	0,6279808
CG2081-RC	Vago	8,35581	0,0450498	7,82026	1,42787	17,2121	20,4725	-0,750854	0,027016	0,6279808
CG2082-RA	CG2082	0,0289553	0,0263744	65,1728	0,072367	0,106059	10,631	-0,313596	0,203581	0,6279808
CG2082-RB	CG2082	6,43088	0,026386	0,0892635	62,6881	0,163009	8,66558	-0,278297	0,260658	0,6279808
CG2082-RC	CG2082	0,0352593	0,0321166	2716,2	44,1781	0,0980216	0,0323463	0,090294	0,680043	0,6279808
CG2082-RD	CG2082	0,0632911	0,0576499	0	0	53,2616	0,0323618	-0,593328	0,061539	0,6279808
CG2082-RE	CG2082	0,0881012	0,0802487	0,0374038	0,0441184	0,158945	0,0402416	0,204363	0,334048	0,6279808
CG2083-RB	Tet	5,5916	7,91446	4,91214	11,4779	0,0205385	3,27088	0,016123	0,958832	0,6279808
CG2086-RA	drpr	13,2441	5,36937	10,3448	2,38568	1,89784	33,7213	-0,038145	0,883308	0,6279808
CG2086-RB	drpr	8,40254	1,55655	9,92062	28,1543	0,115555	3,12985	-0,103204	0,691395	0,6279808
CG2087-RA	PEK	8,10983	0,172454	10,6937	0,252173	0	0,257428	-0,227209	0,429995	0,6279808
CG2091-RA	CG2091	6,29058	4,89523	13,3388	8,8284	5,81392	7,60397	0,096135	0,748211	0,6279808
CG2092-RA	scra	0,0298895	0,589179	0,0286957	1,14617	0,0537486	0,936296	-0,264658	0,353651	0,6279808
CG2092-RB	scra	3,30405	2,91929	2,91208	3,49849	181,345	2,66385	-0,26451	0,353824	0,6279808
CG2092-RC	scra	0,0157749	0,0158817	2,45347	0,0180995	36,0629	0,0184767	-0,555633	0,072718	0,6279808
CG2093-RA	Vps13	11,6651	260,846	0,0907536	0,0318699	165,565	1,0742	-0,279762	0,149136	0,6279808
CG2095-RA	Sec8	12,1708	14,5566	6,29952	2333,07	4,5123	2,75603	0,004114	0,984151	0,6279808
CG2096-RA	flw	0,0262449	32,7453	39,3334	36,5254	0,037682	0,0283995	0,074367	0,710179	0,6279808
CG2096-RB	flw	37,6126	1,30484	0,322834	2,19094	31,1096	29,9082	0,076349	0,702589	0,6279808
CG2097-RA	Sym	2,45499	1638,87	12,604	16,7021	9,71306	10,2189	0,220065	0,442016	0,6279808
CG2098-RA	FeCH	22,705	0,109496	0,115409	0,164515	0,222837	20,8643	-0,114926	0,652112	0,6279808
CG2098-RB	FeCH	0,0476852	0,0170564	0,0179775	0,0194973	0,0264093	0,0543984	-0,1111	0,662573	0,6279808
CG2098-RC	FeCH	0,0500321	0,0160469	0,0169135	0,0182955	0,0247814	11,4763	-0,114926	0,652112	0,6279808
CG2099-RA	RpL35A	802,531	0,0258792	0,01229	0,0302739	0,0215919	0,0134193	-0,321857	0,245675	0,6279808
CG2100-RA	CG2100	5,94237	10,6582	10,7286	13,0532	6,17179	11,066	0,106759	0,691773	0,6279808
CG2101-RA	mRpS35	28,415	9,02942	42,1216	0,0151631	0,0182274	0,0137176	-0,568346	0,077011	0,6279808
CG2102-RA	cas	10,2643	3,68761	3,23701	6,66508	7,52801	3,74756	-0,516019	0,054864	0,6279808
CG2103-RA	Pgant6	23,6052	0,235103	24,7325	0,0256735	2,02482	11,3066	0,190436	0,477484	0,6279808
CG2103-RB	Pgant6	0,0243314	0,712514	0,0233595	1,78543	7,19044	0,0154791	0,18955	0,478848	0,6279808
CG2104-RA	CG2104	0	0,214952	0,22656	2,27177	13,5142	15,7348	-0,143232	0,431375	0,6279808
CG2105-RB	Corin	0,369981	0,374449	0,526228	0,89543	0,665274	0,0405082	-0,57194	0,060965	0,6279808
CG2107-RA	CPT2	6,82962	5,92649	2,84692	0,117173	1,54132	0	-0,207571	0,423884	0,6279808
CG2108-RA	Rab23	13,923	16,1856	0,0131593	20,5489	10,4659	8,62316	0,29258	0,323545	0,6279808
CG2109-RA	mRpL44	33,4108	30,7909	0,0317025	38,7137	47,3603	0,0374	-0,174446	0,520316	0,6279808
CG2110-RA	Cyp4ad1	2,52771	30,4422	47,0719	11,1897	53,9484	4,64569	-0,61531	0,061194	0,6279808
CG2111-RA	CG2111	0,0354484	0	0	0,635615	0	0,0389035	0,05708	0,775751	0,6279808
CG2113-RA	CG2113	0	0,0980236	0	0	0	0	0,01562	0,897983	0,6279808
CG2114-RA	FMRFaR	3,25545	2,94571	11,3733	0	0	0,119614	0,615981	0,008159	0,6279808
CG2116-RA	CG2116	7,97296	10,1219	0	47,6332	0,0419112	8,24373	-0,013118	0,949867	0,6279808
CG2118-RA	Mccc1	0,0283142	6,10542	2,18825	0,0347435	4,30034	0,0249543	-0,120102	0,666877	0,6279808
CG2118-RB	Mccc1	13,6414	0,00698651	13,9027	47,9953	0,0335085	0,167943	-0,120919	0,664684	0,6279808
CG2120-RA	CG2120	1,85102	2,72831	39,79	0,030942	43,4856	3,38748	-0,063973	0,839888	0,6279808
CG2121-RA	CG2121	5,92866	3,77854	0,0337489	0,0380762	9,12356	3,91664	-0,371646	0,195323	0,6279808
CG2121-RB	CG2121	0,0351529	2,04926	6,61042	23,4067	0,0515745	2,68257	-0,369764	0,197187	0,13772387
CG2124-RA	Dpse\GA15256	2,55085	2,88808	2,81517	2,08639	0,775379	0,0398248	-0,38706	0,145647	0,6279808
CG2125-RA	ci	27,7781	30,5138	20,5134	29,6727	13,3771	12,6847	0,424537	0,101808	0,6279808
CG2126-RA	CG2126	9,30245	12,6557	17,3022	14,227	3,6401	11,3136	0,237803	0,263891	0,6279808
CG2127-RA	CG2127	0,0418202	0	0	0,0230429	2,64648	0	-0,013096	0,914398	0,6279808
CG2127-RB	CG2127	0,100501	0	0	58,1595	15,6655	0	-0,013096	0,914398	0,6279808
CG2128-RA	HDAC3	6,23696	23,5999	68,0651	33,0138	26,1624	61,329	0,087634	0,712408	0,6279808
CG2129-RA	CG2129	2,16473	14,722	2,22672	12,4572	8,83638	4,5384	0,328025	0,201255	0,6279808
CG2135-RA	betaGlu	4,94792	0,0156261	18,6218	42,4692	0,0105428	11,8884	-0,051977	0,847362	0,6279808
CG2137-RA	Gpo2	0,0277746	10,1765	0,0167394	35,4172	0,455959	10,2992	0,004401	0,984677	0,6279808
CG2139-RA	aralar1	10,4741	10,7497	10,3507	6,65735	6,29291	0,0263729	-0,33164	0,261617	0,6279808
CG2139-RB	aralar1	0,0230016	0,0209514	3,8125	6,00546	6,04615	0,0261677	-0,326693	0,269666	0,6279808
CG2139-RC	aralar1	4,69455	0,0218727	0,023498	0,0258346	0,0349931	0,0248437	-0,325359	0,27016	0,6279808
CG2139-RD	aralar1	0,0288709	0,0262976	0,0233252	0,0256336	0,0347209	25,0341	-0,545842	0,063697	0,6279808
CG2140-RA	Cyt-b5	366,227	9,19455	0,0203606	62,8268	1,89322	11,3928	-0,340889	0,155206	0,6279808
CG2140-RB	Cyt-b5	86,4469	12,1233	4,67563	189,959	2,30946	0,032534	-0,35052	0,143246	0,6279808
CG2144-RA	PIG-G	3,33767	2,60887	4,2577	0,0104731	0,0141859	2,5087	0,037871	0,880556	0,6279808
CG2145-RA	CG2145	33,7326	27,0766	0,0164103	42,4394	0,0624777	0,0255127	-0,325048	0,192372	0,6279808
CG2146-RA	didum	0,0146121	3,36025	4,0488	4,55858	1,67293	0,0106913	0,197067	0,332187	0,6279808
CG2146-RB	didum	4,05208	0,0133097	0,0140285	4,19374	2,19739	26,9062	-0,380422	0,084887	0,6279808
CG2146-RC	didum	0,0102592	4,08955	3,8853	5,15311	1,87314	0,0166764	0,198046	0,330044	0,6279808
CG2147-RA	CG2147	10,0128	11,2498	13,6783	10,3234	9,18313	13,0825	0,165169	0,536499	0,6279808
CG2150-RA	CG2150	170,923	0,0548312	197,858	0,0177964	7,82304	116,522	0,98513	0,000956	0,6279808
CG2151-RA	Trxr-1	15,7948	0,0271151	0,011091	0,0118286	0,021067	0,0158773	-0,662158	0,01153	0,6279808
CG2151-RB	Trxr-1	0,0297684	18,1593	0,0144646	0,0155533	0,0175603	0,0132345	-0,6628	0,011353	0,6279808
CG2151-RC	Trxr-1	19,9895	5,87021	0,0121255	0,0129644	6,41171	6,16064	-0,657662	0,01207	0,6279808
CG2152-RA	Pcmt	19,1327	0,682247	0,0124029	1,11642	0,0194806	0,0135464	-0,222771	0,38871	0,6279808
CG2155-RA	v	23,3104	1,46244	1,095	3,31445	41,8937	0,866771	-0,34068	0,236633	0,6279808
CG2157-RA	CG2157	67,2259	86,5339	0,0195631	62,4167	1,75534	84,361	0,088145	0,73293	0,6279808
CG2158-RA	Nup50	9,13377	8,52668	16,855	13,2876	6,36801	8,29504	0,269648	0,301419	0,6279808
CG2160-RA	Socs44A	15,4817	15,5625	17,6675	20,4374	12,9537	14,8187	0,044547	0,816361	0,6279808
CG2161-RA	Rga	15,8487	536,88	0,068383	443,636	406,165	0,0864007	0,052705	0,818744	0,6279808
CG2161-RB	Rga	10,3832	0,0270785	11,8896	0,0317777	0,0430431	9,86361	0,052048	0,820908	0,6279808
CG2162-RA	CG2162	7,49831	1663,13	28,7958	23,4415	30,1903	34,6639	0,146337	0,500792	0,6279808
CG2162-RB	CG2162	31,9526	0,0497134	0,0538513	0,0675451	0,0481001	0,0689526	0,149879	0,490327	0,6279808
CG2163-RA	Pabp2	0,0472308	0,0361987	24,3988	15,7137	4,90602	6,27343	0,125019	0,608934	0,6279808
CG2163-RB	Pabp2	74,2697	8,94922	2,2739	0,0480778	0,0589689	17,2749	-0,009087	0,96999	0,6279808
CG2168-RA	RpS3A	1990,36	0,0276	0,0287738	0,032056	9,28979	8,48047	-0,472287	0,075534	0,6279808
CG2168-RB	RpS3A	0,0727173	0,0189943	0,0290905	0,0324347	13,1984	0,0327239	-0,472284	0,075534	0,6279808
CG2168-RD	RpS3A	0,0738551	12,0465	0,02002	0,0218219	0,04342	0,0331105	-0,472297	0,075524	0,6279808
CG2171-RA	Tpi	0,0557288	0,0161459	29,641	44,6979	50,4633	46,2807	-0,535515	0,075315	0,13772387
CG2171-RB	Tpi	153,853	7,05854	9,17084	2,69611	28,2381	17,6404	-0,535454	0,075343	0,6279808
CG2171-RC	Tpi	0,0498434	0,0507616	0,053503	0,0635701	0,086106	0,0648947	-0,535877	0,075172	0,6279808
CG2173-RA	Rs1	13,5322	1,85819	90,6504	77,3603	1,79914	67,5234	-0,139295	0,631955	0,6279808
CG2174-RC	Myo10A	13,9173	114,603	1,75748	3,53992	13,8185	2,10625	0,032212	0,917441	0,6279808
CG2174-RD	Myo10A	0,00650951	0,0519246	39,1901	0,0405108	0,0548721	34,5202	0,031769	0,918586	0,6279808
CG2175-RA	dec-1	0,0117038	0,0326509	0,225754	0,0361068	0,0556497	0,0400499	-0,405114	0,222907	0,6279808
CG2175-RB	dec-1	0,0627302	0,0325274	0,0112364	0,8243	0,052352	60,262	-0,281084	0,3858	0,6279808

gene_id	Symbol	W1_FPKM	W2_FPKM	W3_FPKM	MCM51_FPKM	MCM52_FPKM	MCM53_FPKM	FC	p-value	p-adj
CG2175-RC	dec-1	0,0357102	414,65	0,0344142	0,0392324	0,0489069	0,0701853	-0,281084	0,3858	0,6279808
CG2177-RA	Zip102B	44,6212	0,0205088	0,0216163	0,0883834	12,4211	0,0263833	0,044061	0,841215	0,6279808
CG2177-RB	Zip102B	6,79927	0,0215285	0,0226911	0,0236551	0,0320409	32,1099	0,043227	0,844091	0,6279808
CG2177-RC	Zip102B	0,0239347	0,0216446	0,0228135	0,0248975	0,0337238	0,0270191	0,044061	0,841215	0,13772387
CG2179-RA	Xe7	16,4396	18,3757	13,395	0,0784723	186,61	5,49734	-0,24925	0,21268	0,13772387
CG2179-RB	Xe7	8,53617	7,29395	7,25217	6,26421	12,6902	0,0143989	-0,22165	0,263433	0,6279808
CG2182-RA	CG2182	20,2413	22,5327	11,1447	65,5426	0,102174	0,172608	0,060246	0,779143	0,6279808
CG2182-RB	CG2182	12,4207	16,2641	0,0210987	13,3641	10,4154	134,126	0,0896	0,673945	0,6279808
CG2183-RA	Gasz	2,5068	28,4028	2,28333	0,0435354	3,64722	1,65924	0,031963	0,91649	0,6279808
CG2184-RA	Mlc2	1671,1	0,0642559	2332,73	1890,25	3,53608	2,45215	-0,346231	0,202897	0,6279808
CG2184-RB	Mlc2	0,0304544	2270,66	0,029238	0,0326112	8,00399	6,88183	-0,346228	0,202902	0,6279808
CG2185-RA	elm	75,7191	6,15618	0,0232568	18,9466	0	8,39502	-0,742048	0,01881	0,6279808
CG2186-RA	CG2186	4,72289	5,5871	6,10271	10,8084	4,47002	4,79862	-0,3175	0,207965	0,6279808
CG2187-RA	CG2187	1,52266	146,149	6,40824	1,60049	0,0241054	0,0181673	-0,712372	0,040748	0,6279808
CG2189-RA	Dfd	5,38584	6,25571	1,75601	2,7202	2,93312	1,29743	0,272293	0,286189	0,6279808
CG2191-RA	Smvt	8,70713	4,46122	7,33494	9,41728	4,00674	2000,16	-0,36945	0,263632	0,6279808
CG2194-RB	su(r)	26,2927	0,0181248	0,067702	17,6766	119,282	0	-0,82841	0,010303	0,6279808
CG2194-RC	su(r)	0,0198984	17,4395	2,00165	2,64374	0,962176	1,18884	-0,830617	0,010108	0,6279808
CG2196-RA	salt	3,16717	1,31517	7,87443	3,5278	8,92034	16,3144	-0,846556	0,014167	0,6279808
CG2198-RA	Ama	0,0371282	9,35127	0,0356453	0,0404114	3,01954	0,0412535	-0,432675	0,161489	0,6279808
CG2198-RB	Ama	7,88507	17,2058	8,97201	22,1882	3,01558	9,26979	-0,435147	0,158035	0,6279808
CG2198-RC	Ama	27,4896	0,102707	48,0896	78,9871	0,0224953	41,3748	-0,432675	0,161489	0,6279808
CG2199-RA	CG2199	0,023652	0,032823	103,906	0	0	0	0,276364	0,288592	0,6279808
CG2199-RB	CG2199	19,0588	29,8211	182,987	0,0249163	8,5943	0,0254355	0,277096	0,287374	0,6279808
CG2200-RA	CG2200	18,539	17,9847	2,94981	2,97101	2,68562	1,90237	-0,273836	0,324449	0,6279808
CG2201-RA	CG2201	0,028302	0,0257795	0,0271716	0,0301492	0,0408373	0,0307774	0,22985	0,30098	0,6279808
CG2201-RB	CG2201	8,3437	10,2943	8,36542	7,19602	6,28036	5,22471	0,223164	0,319066	0,6279808
CG2201-RC	CG2201	0,0334618	0,0304793	0,0321253	11,9455	0,0488905	3,56573	0,233302	0,297193	0,6279808
CG2201-RD	CG2201	23,8736	32,5929	20,6875	21,3362	12,8653	16,2011	0,222535	0,320344	0,6279808
CG2201-RE	CG2201	7,16629	8,7086	10,9523	9,05258	9,6135	6,04105	0,230363	0,298407	0,6279808
CG2202-RA	CG2202	3,44037	3,02927	3,66328	5,53074	2,03029	4,29081	-0,440179	0,044512	0,6279808
CG2204-RA	Galphao	0,0287829	0,0262174	0,0276333	9,18355	0,0424494	0,0319924	0,353661	0,108981	0,6279808
CG2204-RB	Galphao	0,0286084	0,0260585	0,0274658	0,0304981	3,00388	4,34688	0,042715	0,855795	0,6279808
CG2204-RC	Galphao	0,0302314	0,0275369	0,029024	0,032355	125,587	116,37	0,0407	0,863611	0,6279808
CG2204-RD	Galphao	0,0300527	0,0273741	0,0288524	0,0321498	147,757	159,565	0,046645	0,842567	0,6279808
CG2204-RE	Galphao	0,0209454	0,0190785	0,0201089	0,0219235	0,0324251	0,0244375	0,044739	0,848914	0,6279808
CG2204-RF	Galphao	0,0304263	0,0277144	0,0292111	0,032579	0,0324251	0,0244375	0,043699	0,852437	0,6279808
CG2204-RG	Galphao	0,0289595	0,0263783	0,0278028	0,0308985	0,108246	0,0815804	0,048521	0,836535	0,6279808
CG2204-RH	Galphao	0,0188806	0,0171978	0,0181265	11,6036	393,168	371,185	0,059887	0,797976	0,6279808
CG2204-RI	Galphao	48,0508	53,7408	53,7709	40,0841	174,013	179,848	0,361999	0,100042	0,6279808
CG2206-RA	l(1)G0193	10,2119	1930,11	17,6829	15,0482	0,0368421	14,2882	0,045002	0,83652	0,6279808
CG2206-RB	l(1)G0193	15,4171	340,686	1,00209	756,955	13,3435	1,80269	0,043648	0,842496	0,6279808
CG2207-RA	Df31	37,5511	0,827766	0,0363604	0,0412979	0,0559383	0,0421585	-0,254017	0,305995	0,6279808
CG2207-RB	Df31	65,9342	0,0151807	159,486	193,008	160,083	154,159	-0,253065	0,307779	0,6279808
CG2207-RF	Df31	174,082	1,00031	291,486	233,772	137,543	244,433	-0,250044	0,311584	0,6279808
CG2209-RA	CG2209	0	0	0	0	16,9971	0,225963	NA	NA	0,6279808
CG2210-RA	awd	183,536	0,0723395	20,535	264,469	0,0223489	0,0168435	-0,403553	0,214894	0,6279808
CG2211-RA	CG2211	0,0360348	0,0215439	0,0345956	0,0478803	0	3,5248	-0,584091	0,008339	0,6279808
CG2212-RA	sws	18,1783	20,8547	13,2525	15,2476	6,07279	10,3321	0,002608	0,99027	0,6279808
CG2212-RB	sws	5,55968	6,8251	13,2447	11,2218	121,236	112,225	0,306047	0,153345	0,6279808
CG2213-RA	msd5	2,06573	1,88161	4,91839	3,5569	3,23553	2,97613	-0,016292	0,960555	0,6279808
CG2216-RA	Fer1HCH	146,84	0,105739	169,547	0,368643	0,137924	3867,98	-0,39035	0,099595	0,6279808
CG2216-RB	Fer1HCH	0,0451338	4,95928	0,0433311	5,7282	0	0,110903	-0,371222	0,119149	0,6279808
CG2216-RC	Fer1HCH	100,528	3,1142	86,1283	323,655	0,113331	0,116674	-0,383474	0,106637	0,6279808
CG2216-RD	Fer1HCH	0,0454442	0	91,1195	0,0522319	3368,34	0,152615	-0,376249	0,113712	0,6279808
CG2216-RE	Fer1HCH	0,0442275	0,0593165	0,042461	115,311	0,147152	3,44348	-0,371222	0,119149	0,6279808
CG2217-RA	CG2217	2,59121	2,01667	2,51873	1,00003	8,40109	0,263353	-0,509024	0,052844	0,6279808
CG2218-RA	CG2218	12,7019	0,327694	4,60518	4,37235	2,67849	3,55923	0,170715	0,468321	0,6279808
CG2219-RA	Arl4	86,0413	0,0272996	57,6342	97,3797	101,059	70,9584	-0,150066	0,568867	0,6279808
CG2221-RA	l(1)G0289	23,7925	29,6457	26,1444	46,5628	89,5548	0,288921	-0,133902	0,591046	0,6279808
CG2222-RA	Psf3	39,5267	29,6602	4,38503	19,8918	20,865	19,3996	0,357193	0,192508	0,6279808
CG2224-RA	CG2224	3,14005	3,47432	0,0277178	0,0307974	2,02514	4,53016	-0,13361	0,579903	0,6279808
CG2225-RA	CG2225	3,63598	29,4608	2,45882	2,92431	1,16953	0,985095	0,071509	0,782134	0,6279808
CG2225-RB	CG2225	2,42625	46,9421	0,676573	1,91209	1,10047	1,37357	0,074915	0,772209	0,6279808
CG2225-RC	CG2225	1,33397	154,897	0,584314	2,53099	1,4893	0,895939	0,185228	0,480843	0,6279808
CG2225-RE	CG2225	0,0166662	3,61409	0,60802	0,0172694	0,0233915	0,438924	0,07414	0,774494	0,6279808
CG2225-RF	CG2225	0,0172943	1,61394	1,56074	1,97129	0,668156	0,665917	0,122293	0,639247	0,6279808
CG2227-RA	Gip	43,025	5,58875	72,0479	44,168	0,512804	0,0371316	-0,497799	0,125885	0,6279808
CG2229-RA	Jon99Fii	11,6392	2,76568	0,0222066	0,0243365	0,032964	3,42131	-0,015198	0,957012	0,6279808
CG2233-RA	CG2233	46,3923	39,0681	11,7451	0,101291	13,1253	13,0712	-1,078755	2,96E-05	0,6279808
CG2238-RA	eEF2	0,0241094	7,03477	18,2823	14,6731	16,7071	19,4848	-0,272396	0,152406	0,6279808
CG2238-RB	eEF2	909,549	51,3575	39,9873	46,5382	27,6485	30,4242	-0,272375	0,152444	0,6279808
CG2238-RC	eEF2	0,0235592	0,0219605	0,0231465	0,0254259	0,0344395	0,0259557	-0,272368	0,152455	0,6279808
CG2239-RA	jdp	0,0344923	0,031418	0,0331147	0,0373003	9,56859	5,55855	0,612618	0,005515	0,6279808
CG2239-RB	jdp	23,8014	34,2357	32,5015	25,8587	0,101458	7,07835	0,612364	0,005552	0,6279808
CG2239-RC	jdp	0,107849	0,0982366	0,103542	0,142155	0,0939797	0,0854133	0,278994	0,280706	0,6279808
CG2241-RA	Rpt6R	0,278605	3,30424	0,557245	0,0531161	3,68301	0,0658373	-0,07297	0,833515	0,6279808
CG2244-RA	MTA1-like	0,014714	0,0134026	0,0141264	0,0151771	0,0205575	0,0154934	0,094736	0,697999	0,6279808
CG2244-RB	MTA1-like	13,0138	13,6083	10,9214	15,7713	7,57056	10,4691	0,096424	0,692827	0,6279808
CG2244-RC	MTA1-like	5,14606	5,3591	7,10297	10,0813	3,12468	2,1331	0,105185	0,666782	0,6279808
CG2245-RA	nero	8,43457	0,0145284	23,3838	0,0179028	8,80086	0,0420793	-0,03373	0,919304	0,6279808
CG2246-RA	CG2246	0,0431398	0,0392947	0,0414168	0,0476605	0	19,5328	0,076954	0,734123	0,6279808
CG2246-RB	CG2246	0,0385357	0,0351009	0,0369965	0,0420893	0,116665	0,0224776	0,079068	0,727539	0,6279808
CG2246-RC	CG2246	0,0434233	5,3001	14,0075	11,6174	0	0,0253111	0,083737	0,710887	0,6279808
CG2246-RD	CG2246	27,4083	21,6906	24,5238	17,3779	0	0,0264351	0,083537	0,711735	0,6279808
CG2246-RE	CG2246	5,38661	0,0356836	0,0376107	0,0428558	12,1238	0,0267612	0,082132	0,715735	0,6279808
CG2246-RF	CG2246	0,0449291	6,95718	8,88574	6,78324	7,63385	44,0749	0,081484	0,718739	0,6279808
CG2247-RA	CG2247	3,63575	2,87174	3,95426	0,0708116	0,0458278	3,0139	-0,146333	0,536305	0,6279808
CG2248-RA	Rac1	86,3196	32,1232	68,3079	91,4255	30,0193	62,5887	0,24187	0,19771	0,6279808
CG2249-RA	Dpse\GA15322	308,407	257,664	1,5049	0,0262881	0,077105	0,058111	-0,574125	0,072984	0,6279808
CG2249-RB	Dpse\GA15322	0,0563307	0,0513099	0,0240777	0,0265102	0,085775	0,0646452	-0,582075	0,069811	0,6279808
CG2252-RA	fs(1)h	7,56271	0,0107252	47,6002	14,3167	1,14175	1,90827	-0,041025	0,876728	0,6279808
CG2252-RB	fs(1)h	0,0117747	5,32713	56,2138	0,0120625	1,07015	1,55662	-0,026285	0,921609	0,13772387
CG2252-RC	fs(1)h	2,41577	0,0100316	776,914	4,29612	39,5806	0,0117216	-0,040876	0,877214	0,6279808
CG2252-RD	fs(1)h	2,84693	0,0102225	2,12099	3,14892	1157,6	37,8311	-0,038773	0,883425	0,6279808
CG2252-RE	fs(1)h	0,0112228	31,8748	2138,4	0,0114823	0,820106	3203,1	-0,090244	0,734599	0,6279808
CG2253-RA	Upf2	2,69464	2,45447	0,0107873	4,76248	4,66764	34,8421	-0,217382	0,372573	0,6279808
CG2254-RA	Ldsdh1	34,5932	9,41305	4,19129	33,4547	1,08584	0,0123139	0,137568	0,553514	0,6279808
CG2256-RB	CG2256	1,70713	2,43917	0,0105734	2,74564	0,0163387	6,04535	-0,306266	0,323502	0,6279808
CG2257-RA	Dsim\GD16131	95,0595	3,13519	5,03895	23,7237	8,3111	4,58865	0,345896	0,110321	0,6279808
CG2257-RB	Dsim\GD16131	0,0289976	0	29,0159	5,53655	3,83354	3,83622	0,35286	0,105672	0,6279808
CG2257-RC	Dsim\GD16131	27,718	0	7,6412	7,64042	0,586272	6,19862	0,352871	0,105701	0,6279808
CG2258-RA	CG2258	0,758788	1,08909	1,3797	1,3009	0,0268059	0,466799	-0,071765	0,802681	0,6279808
CG2258-RB	CG2258	0,312662	0,0183738	0,0193661	0,0210751	16,001	0,0215142	-0,004802	0,986783	0,6279808

gene_id	Symbol	W1_FPKM	W2_FPKM	W3_FPKM	MCM51_FPKM	MCM52_FPKM	MCM53_FPKM	FC	p-value	p-adj
CG2258-RC	CG2258	0,0189946	0,0173016	0,501488	0,487182	6,45953	0,501111	-0,066688	0,817135	0,6279808
CG2259-RA	Gclc	10,2093	7,13016	14,7126	6,07653	7,74592	6,45321	-0,411027	0,166052	0,6279808
CG2259-RB	Gclc	4,34082	2,24918	4,66604	11,0049	8,72779	10,4945	-0,40956	0,16724	0,6279808
CG2260-RA	CG2260	8,94646	5,47954	15,6645	5,52045	14,7011	0,0315868	-0,102654	0,727855	0,6279808
CG2261-RA	CstF50	0,0476508	0,0434037	7,92562	4,46087	13,3484	19,0407	0,06579	0,833414	0,6279808
CG2261-RB	CstF50	3,59467	2,95814	40,1737	22,2835	0,0371333	0,0279859	0,06579	0,833414	0,6279808
CG2262-RA	Smox	11,7289	14,648	12,4277	5,4904	2,05894	30,2653	-0,102966	0,622892	0,6279808
CG2263-RA	alpha-PheRS	18,9565	13,4455	30,7301	1,58096	1,38766	14,8513	-0,354606	0,211994	0,6279808
CG2264-RA	magu	2,81156	0	0,0208285	0,0227486	0,0359082	0,0270626	-0,07916	0,706434	0,6279808
CG2264-RB	magu	0,0250794	0	3,29655	4,78564	0,0322843	0,0243314	-0,073173	0,727564	0,6279808
CG2264-RC	magu	0,0226779	1,43913	1,57264	2,55746	0,0308131	0,0232226	-0,080715	0,701117	0,6279808
CG2264-RD	magu	0,021695	0,022844	0	1,91633	2,46766	1,58202	-0,073679	0,726474	0,6279808
CG2264-RF	magu	1,98418	0,0206566	0	0	1,16153	1,21415	-0,054504	0,796632	0,6279808
CG2267-RA	CG2267	0,114252	0,0413937	0,0957681	0,185066	4,28844	0,0520818	0,092736	0,598737	0,6279808
CG2269-RA	CG2269	0,0189619	2,38351	0,0182045	6,72605	0,0655041	0,0493678	0,00677	0,975644	0,6279808
CG2269-RB	CG2269	14,3852	12,5681	12,2719	5,59224	45,8869	43,5319	-0,011616	0,958482	0,6279808
CG2269-RC	CG2269	2,7253	2,34493	5,6505	4,84928	110,684	101,802	-0,002919	0,989488	0,6279808
CG2272-RA	slpr	4,49435	4,63334	4,68574	6,5324	0,137199	3,42685	0,086227	0,740876	0,6279808
CG2275-RA	Jra	0,042221	0,0384578	0,0405347	0,0483601	0,0267576	1136,28	-0,201868	0,342033	0,6279808
CG2275-RB	Jra	36,394	38,9664	50,7369	49,5175	7,43669	0,0693079	-0,201579	0,342459	0,6279808
CG2277-RA	CG2277	13,2738	12,1047	17,4799	19,1239	14,6824	15,5014	-0,219034	0,315319	0,6279808
CG2278-RA	CG2278	1,8244	38,6985	0,131648	0,170894	2798,41	0	-0,165471	0,600695	0,6279808
CG2286-RA	Dpse\GA15341	14,124	2,26529	0,0555489	8,2682	8,57302	5,10941	-0,38865	0,056707	0,6279808
CG2286-RB	Dpse\GA15341	49,8048	8,9327	3,62842	15,6095	3,19169	4,01604	-0,388552	0,056867	0,6279808
CG2291-RA	CG2291	0	0,321752	0	0	0	0	NA	NA	0,6279808
CG2292-RA	PIG-N	1,18544	0,0197613	0	0	1,61143	0	-0,089441	0,707523	0,6279808
CG2297-RA	Obp44a	185,256	14,1568	1,98181	14,6141	1,10376	1,76055	-1,072962	0,001427	0,6279808
CG2302-RB	nAChRalpha3	1,06499	1,20458	10,0147	18,4932	4,58139	4,7428	0,529656	0,063954	0,6279808
CG2304-RA	Trc8	6,47724	4,07973	4,63754	0,0361343	0,0438251	0,0112888	0,080257	0,71637	0,6279808
CG2304-RB	Trc8	0,0171596	3,7356	0,0095499	0,0196602	10,7997	0,0103587	0,225343	0,300642	0,6279808
CG2304-RC	Trc8	0,0334957	0,0305102	0,0144207	0	2,40329	0,0160287	0,290095	0,172004	0,6279808
CG2304-RD	Trc8	0,0188752	0,0171929	0,0302416	685,388	1,99659	0,0345209	0,22509	0,301314	0,6279808
CG2310-RA	CG2310	0,0548954	54,2145	0,0380392	42,544	31,4653	0,063787	-0,027314	0,883991	0,6279808
CG2310-RB	CG2310	30,3403	0,218067	10,053	4,33	4,93147	33,468	-0,028028	0,880802	0,6279808
CG2316-RA	ABCD	39,489	0,0662359	0,0238693	52,6609	20,9008	0,0268145	-0,042882	0,869688	0,6279808
CG2316-RB	ABCD	0,0245301	0,0672724	22,0574	113,217	0,100223	44,2751	-0,043766	0,866989	0,6279808
CG2316-RC	ABCD	25,2117	34,5718	83,714	0,0798183	153,056	75,312	-0,043195	0,868755	0,6279808
CG2316-RD	ABCD	0,0248717	39,8845	0,0650855	33,531	0,108114	0,0814815	-0,04292	0,869512	0,6279808
CG2316-RE	ABCD	0,0234589	35,1393	9,71042	6,73414	6,88622	5,96321	-0,044712	0,864214	0,6279808
CG2316-RF	ABCD	0,023754	108,073	31,4491	49,2921	47,7848	62,6061	-0,008554	0,9735	0,6279808
CG2316-RG	ABCD	0,0248623	0,0617507	0,0572018	0,0726863	0,07103	0,074201	-0,042795	0,869933	0,6279808
CG2321-RA	CG2321	0,0314997	0,0286921	0,0181213	0,014332	0,0194128	0,0200699	0,995129	0,000248	0,6279808
CG2328-RA	eve	3,24078	0,0409524	2,72242	0,335157	16,6533	11,4019	0,444116	0,134528	0,6279808
CG2330-RA	Neurochondrin	73,6191	0,0140404	0,0147986	0	1,4352	4,40804	0,434817	0,128702	0,6279808
CG2331-RA	TER94	26,2996	17,7331	29,9673	54,3387	9,09907	24,1211	-0,070665	0,746894	0,6279808
CG2331-RB	TER94	46,9696	45,0541	31,1388	23,4454	3,04647	0,0229845	0,174751	0,424014	0,6279808
CG2331-RC	TER94	0,0214834	0,0195686	0,0206254	0,0225154	5,37288	0,0243314	-0,06665	0,761216	0,6279808
CG2331-RD	TER94	0,0226779	7,54999	5,97645	8,52937	9,52061	4,96635	-0,069599	0,750385	0,6279808
CG2336-RA	CG2336	0,0770714	0,070202	0	19,2925	3,72441	27,3461	0,044335	0,715924	0,6279808
CG2341-RA	Ccp84Ad	218,821	248,921	321,149	243,779	224,171	238,233	0,452874	0,037389	0,6279808
CG2342-RA	Ccp84Ag	215,198	206,846	393,216	153,407	185,729	231,095	0,530233	0,036452	0,6279808
CG2345-RA	Edg84A	0,159091	2,37371	3,61386	0,742098	1,1581	0,516985	0,237138	0,483635	0,6279808
CG2346-RA	FMRFa	1,40245	0,11292	3914,24	0,0169234	0,453973	70,5758	0,091695	0,75436	0,6279808
CG2358-RA	Dsim\GD19887	79,2756	89,1548	114,09	125,627	75,6977	107,556	0,012595	0,953655	0,6279808
CG2360-RA	Ccp84Aa	111,778	99,1076	157,599	0,332533	0	84,9017	0,94916	0,00052	0,6279808
CG2368-RA	psq	0,0120981	0,0110198	0,0116149	9,46837	3,42826	3,3669	0,116274	0,577055	0,6279808
CG2368-RB	psq	0,0124726	0,0113609	0,0119745	33,6977	0,207448	0,0137823	0,116274	0,577055	0,6279808
CG2368-RC	psq	4,43558	4,3157	10,4767	0,249062	20,3036	0,0142013	0,120198	0,565668	0,6279808
CG2368-RD	psq	0,0131372	0,0119663	0,0126125	6,51219	0,269231	2,60166	0,178483	0,407798	0,6279808
CG2368-RE	psq	0,0135244	0,012319	0,0129843	0,253608	431,688	0,0147106	0,174678	0,416768	0,6279808
CG2368-RF	psq	0,0144218	0,0131364	0,0138458	0,0124031	4,86004	0,0156229	0,174628	0,416624	0,6279808
CG2368-RG	psq	0,0139942	0,0127469	0,0134352	6,18648	11,3494	0,0143773	0,175946	0,41385	0,6279808
CG2368-RH	psq	7,33489	3,32368	5,95254	5,31709	18,2567	0,0155005	0,176303	0,414113	0,6279808
CG2368-RI	psq	0,0136869	0,012467	0,0131403	0,013501	0,0267496	1,96355	0,11596	0,578359	0,6279808
CG2368-RJ	psq	3,99664	8,78259	0,0141327	0,0139114	3,36947	2,19613	0,178088	0,409195	0,6279808
CG2368-RK	psq	4,20796	2,95534	3,39378	0,0148655	0,0168001	10,8585	0,174234	0,418039	0,6279808
CG2371-RA	Vlet	7,76011	2,15601	5,95438	5,46796	7,41837	7,94976	-0,147293	0,540649	0,6279808
CG2371-RB	Vlet	0,0590665	6,75214	2,2683	1,84013	2,4972	3,08594	-0,149223	0,535382	0,6279808
CG2371-RC	Vlet	4,55509	2,17754	5,52073	6,45718	4,32518	3,44035	-0,149223	0,535382	0,6279808
CG2374-RA	lbm	56,1806	25,7851	64,6127	91,2642	63,849	72,0932	-0,124369	0,556616	0,6279808
CG2380-RB	Nfl	12,2962	9,62089	17,5213	45,1251	81,8584	62,6372	0,48194	0,04907	0,6279808
CG2381-RA	Syt7	0,0225156	42,9304	25,7392	0,0258447	0,0350068	29,7532	-0,130391	0,65959	0,13772387
CG2381-RB	Syt7	0,0236351	0,0228094	0,0240412	39,0336	50,1163	5,73422	-0,13158	0,656772	0,6279808
CG2381-RE	Syt7	0,0237626	35,8118	20,0421	0,0264676	0,0358505	0,0257569	-0,130391	0,65959	0,6279808
CG2381-RF	Syt7	0,0244846	6,22544	36,0945	47,0088	0,0241838	0,417231	-0,118939	0,687853	0,6279808
CG2381-RG	Syt7	47,449	0,0218014	4,45739	7,84318	2,93688	2,39376	-0,13158	0,656772	0,6279808
CG2381-RH	Syt7	0,0250414	30,5006	0,0229788	0,0252312	6,94807	8,27365	-0,130717	0,658711	0,6279808
CG2397-RA	Cyp6a13	7,55612	0,677749	7,79455	2,02628	15,0896	5,63849	-0,593079	0,050278	0,6279808
CG2411-RA	ptc	7,43727	9,55263	43,4852	16,5238	42,103	4,71158	0,011782	0,971898	0,6279808
CG2412-RA	Rad51D	0,0728777	1,10027	4,25275	5,61734	2,67619	0,867896	0,113399	0,690462	0,6279808
CG2444-RA	CG2444	20,1117	10,1098	30,3497	7,66284	52,475	35,9846	-0,564168	0,084978	0,6279808
CG2446-RA	Amun	32,817	0,802487	3,29078	1,08265	2,46104	0,221649	0,0381	0,889458	0,6279808
CG2446-RB	Amun	0,0228583	0,419466	0,486331	0,309618	0,356704	0,0231109	0,003697	0,989291	0,6279808
CG2446-RC	Amun	0,0226313	0,0196709	0,0207332	0,0226391	0,0306649	0,0484783	0,00344	0,990039	0,6279808
CG2446-RD	Amun	0,0223028	0,0391669	0,041282	0,0474888	0,0643239	0,062775	0,003697	0,989291	0,6279808
CG2446-RE	Amun	0,0227874	0,0739592	0,0779533	0,0614936	0,0832935	102,905	0,014284	0,958504	0,6279808
CG2448-RA	FucT6	12,7358	11,2903	0,00875997	16,807	15,8431	0,00948389	-0,47189	0,030274	0,6279808
CG2453-RA	Coq5	8,59156	7,98024	22,287	9,10955	4,84219	7,82368	-0,021942	0,939682	0,6279808
CG2457-RB	inaF-D	0,598666	0,0206142	0,0219454	0,0239561	3,74662	0,0244553	0,562306	0,097831	0,6279808
CG2467-RA	pot	0,0178262	0,0162374	0,0171142	0,0185218	0,0250879	25,1212	0,099926	0,622232	0,6279808
CG2467-RB	pot	20,2392	21,9471	20,0661	24,2301	16,3996	18,0419	0,099926	0,622232	0,6279808
CG2469-RA	Ctr9	0,0177448	0,0161632	0,0170361	0,0184337	0,0249686	0,0188178	0,291298	0,202808	0,6279808
CG2469-RB	Ctr9	19,7739	24,3175	18,5671	21,3458	12,5618	12,9866	0,272703	0,229621	0,6279808
CG2471-RA	Sclp	15,9072	3,73982	20,8877	73,6563	28,126	0,0189077	-0,470438	0,077723	0,6279808
CG2478-RA	brun	7,1586	14,0655	6,97105	16,8891	13,2485	14,5219	-0,006876	0,976901	0,6279808
CG2488-RA	phr6-4	0,0313354	0	0	0	0	0	-0,740795	0,03132	0,13772387
CG2488-RB	phr6-4	0,450444	1,2459	0,156505	0,324567	0,181031	0	-0,740795	0,03132	0,6279808
CG2493-RA	CG2493	8,47983	0	14,3564	29,8565	0,0697532	22,0546	-1,109009	8,47E-06	0,6279808
CG2503-RA	atms	8,08256	9,31296	12,1224	15,8408	6,27489	9,43898	-0,123083	0,57128	0,6279808
CG2505-RA	alpha-Est2	10,9061	7,98705	10,3412	6,9779	15,1425	12,1815	-0,02527	0,92905	0,6279808
CG2507-RA	sas	0,0111546	2,71789	2,14181	1,21064	0,218815	2,96653	0,39245	0,181305	0,6279808
CG2507-RB	sas	22,9762	24,1335	16,9944	0,161546	0	1,41748	0,369051	0,207957	0,6279808
CG2508-RA	Cdc23	1,58802	7,2423	2,14029	0,0327084	59,8992	0,0333899	-0,021261	0,939276	0,6279808
CG2512-RA	alphaTub84D	15,3315	9,86414	1,29837	35,463	20,4234	10,7499	-0,088214	0,75913	0,6279808

gene_id	Symbol	W1_FPKM	W2_FPKM	W3_FPKM	MCM51_FPKM	MCM52_FPKM	MCM53_FPKM	FC	p-value	p-adj
CG2512-RB	alphaTub84D	11,8116	11,5388	0,0624788	7,65558	8,71784	19,475	-0,056972	0,841663	0,6279808
CG2519-RA	CG2519	15,4405	2,23621	0,201259	26,1705	0,0193287	0,371298	0,017744	0,938259	0,6279808
CG2519-RB	CG2519	4,17414	22,6985	9,77569	0,0169626	0,020045	6,24972	0,019644	0,931625	0,6279808
CG2520-RA	lap	0,0382678	26,3522	0,160194	7,95782	38,0863	0	0,490362	0,043424	0,6279808
CG2520-RB	lap	25,0931	5,71435	0,193317	0,853364	4,90133	0,0426395	-0,270378	0,279227	0,6279808
CG2522-RA	Gtp-bp	43,7426	49,7395	0,0474826	90,9661	0,0294021	1,35299	-0,219607	0,232086	0,6279808
CG2525-RA	Hus1-like	6,72748	2,9825	2,97395	0	1,07917	2,38066	0,117598	0,61974	0,6279808
CG2528-RB	CG2528	0	0,0400176	0,0421787	0	0	0,0630401	0,044335	0,715924	0,6279808
CG2530-RA	corto	34,5573	46,3962	33,2495	37,2406	0,197565	12,9593	0,400645	0,097957	0,6279808
CG2533-RA	CG2533	0,341477	0,544322	33,4976	0,787262	526,486	0,176541	0,008698	0,979428	0,6279808
CG2540-RA	CG2540	8,40966	7,9088	11,901	4,05589	3,73828	10,8273	-0,302406	0,304283	0,6279808
CG2543-RA	Fpgs	8,40271	25,6063	8,80048	6,40942	14,7363	0,0715947	0,089113	0,726579	0,6279808
CG2555-RA	Cpr11B	3,31187	347,778	11,7102	18,8687	26,5385	22,4291	-0,171902	0,629231	0,6279808
CG2556-RA	Dsim\GD17083	19,34	0,645274	0,967193	41,0961	1,34682	6,62194	-0,305065	0,258697	0,6279808
CG2559-RA	Lsp1alpha	0	6,3419	43,1834	0,557415	0,0419309	0	0,01562	0,897983	0,6279808
CG2560-RA	Cpr11A	83,2925	0,735776	24,807	7,32507	9,65456	0,501981	0,355751	0,26651	0,6279808
CG2574-RA	CG2574	0	0	0	0	0	0	NA	NA	0,6279808
CG2577-RA	CG2577	0	0	0	0	0	0	NA	NA	0,6279808
CG2595-RA	RacGAP84C	0	0	0	12,3158	2,69332	2,55517	0,012562	0,923862	0,6279808
CG2595-RB	RacGAP84C	0	0,16765	0	8,11442	0,0185224	4,35389	0,012562	0,923862	0,6279808
CG2604-RA	Dsim\GD19638	11,0426	11,9266	18,4582	7,37783	0,0562202	6,17506	0,247149	0,322115	0,6279808
CG2604-RB	Dsim\GD19638	0,0380475	0,0346563	0,0365279	1,46284	0,0581999	2,45252	0,245839	0,325286	0,6279808
CG2604-RC	Dsim\GD19638	0,0392685	0,0357685	0,0377001	1,90093	5,91072	0,0494041	0,245611	0,325625	0,6279808
CG2608-RA	CG2608	11,8723	0,191677	0,404056	1,23538	0,439629	0,219769	-0,002149	0,992199	0,6279808
CG2611-RA	CG2611	11,6099	0	12,2444	0	0	0	0,156837	0,493894	0,6279808
CG2614-RA	CG2614	6,75022	4,36439	0,362841	0,531947	0,0388296	0,0292643	0,07555	0,706144	0,6279808
CG2615-RA	IKKepsilon	2,95408	5,51445	7,55554	6,64189	4,6352	4,48368	0,469655	0,032636	0,6279808
CG2615-RB	IKKepsilon	4,30983	0,0223686	3,43189	4,52455	2,1913	2,88779	0,469655	0,032636	0,6279808
CG2616-RA	CG2616	0	2,27615	9,18524	2,47973	1,27977	1,31072	-0,060279	0,67377	0,6279808
CG2617-RA	CG2617	2,06651	0,0245893	6,97845	0,402645	4,35348	0,266628	-0,422532	0,161112	0,6279808
CG2621-RA	sgg	0,0219036	0	0,0219606	4,42799	0	0,468432	0,014316	0,960163	0,6279808
CG2621-RB	sgg	0,0235257	0,303972	0,0199633	1,12522	0,171302	0,344359	0,112355	0,691466	0,6279808
CG2621-RC	sgg	0,0208463	0,289025	0,020904	0,180518	0,27279	0	0,104682	0,710556	0,6279808
CG2621-RD	sgg	0,0228742	0,110232	19,6546	0,506175	0	0,00536936	0,114362	0,698815	0,6279808
CG2621-RE	sgg	0,0207938	0,0949913	0,0206657	0,11524	0	3,955	0,104375	0,711402	0,6279808
CG2621-RF	sgg	0,0217737	0	0,0205386	2,36515	48,9403	0,0052908	0,102929	0,715172	0,6279808
CG2621-RG	sgg	16,7813	0,335768	0,0234113	1,21866	0,00712437	0,00523643	0,123179	0,66382	0,6279808
CG2621-RH	sgg	0,0215254	0,126019	11,6087	10,5492	0,0069399	22,7545	0,104488	0,711058	0,6279808
CG2621-RI	sgg	0,021393	0	0,0221755	0,651096	0,00702014	5,7705	0,104341	0,711264	0,6279808
CG2621-RJ	sgg	0,0243852	8,18138	18,2749	10,4447	0,006948	0,0068494	0,0213	0,940442	0,6279808
CG2621-RK	sgg	12,522	0	0,235921	0,980459	29,8569	53,8026	0,125214	0,659263	0,6279808
CG2621-RL	sgg	0,0230981	0,0199513	0,0615492	0,0229786	7,43351	0,196808	0,034639	0,904674	0,6279808
CG2621-RM	sgg	16,565	0,0214288	93,8535	0,0247758	0,00908818	0,0620523	0,03767	0,896141	0,6279808
CG2637-RA	Fs(2)Ket	3,80503	2,94101	4,68048	7,06863	2,35774	25,3834	-0,446748	0,057559	0,6279808
CG2641-RA	CG2641	3,03853	2,49652	3,72181	7,45146	16,8124	2,97116	-0,899308	0,001851	0,6279808
CG2647-RA	per	0,569242	1,44916	0,525487	1,72715	1,73651	0,348665	0,277424	0,434728	0,6279808
CG2650-RA	Dsim\GD24605	1,23806	1,15904	2,93851	1,27089	0,275705	2,38569	-0,477795	0,134878	0,6279808
CG2652-RA	CG2652	0,279391	0,286299	0,201174	1,01104	0,290034	0,292397	-0,307884	0,363799	0,6279808
CG2655-RA	HLH3B	1,97788	3,91457	2,3443	4,40698	5,08018	4,14832	-0,559379	0,0653	0,6279808
CG2656-RA	CG2656	7,84897	7,05965	15,1025	7,86595	6,06589	6,8724	-0,13835	0,637325	0,6279808
CG2657-RB	Ir21a	1,26717	1,15423	2,25754	1,10478	1,12258	0,762175	0,525005	0,085569	0,6279808
CG2658-RA	Spg7	0,0217737	2,03288	0,020904	2,64836	18,2639	9,43188	-0,101408	0,635555	0,6279808
CG2658-RB	Spg7	8,12735	9,15017	10,6982	2,17801	4,56196	3,87576	-0,101408	0,635555	0,6279808
CG2662-RA	CG2662	1,22363	1,29728	1,3866	3,18211	3,41775	1,30874	-0,2326	0,420911	0,6279808
CG2663-RA	CG2663	66,1475	44,5883	24,1397	65,257	29,0791	45,8076	0,471595	0,080587	0,6279808
CG2663-RB	CG2663	0,0416707	0,0379565	0,0117439	0,0458689	0,0621298	0,0468247	0,471081	0,080947	0,13772387
CG2665-RA	EbpII	0	22,0905	0,476968	0,0105807	0	0	NA	NA	0,6279808
CG2666-RA	kkv	24,1955	12,0736	2,96188	21,2756	1,48739	6,94875	0,227911	0,46326	0,6279808
CG2666-RB	kkv	0,0122325	29,897	0,0503733	36,0121	0	11,7877	0,219957	0,478281	0,6279808
CG2666-RC	kkv	0,0118444	0,0111422	93,6307	0,0125448	7,71767	0,0128062	0,227911	0,46326	0,6279808
CG2668-RA	Ebp	0,125715	0,068706	0,0203424	7,1591	0	34,4212	-0,10991	0,724656	0,6279808
CG2669-RA	hd	1,61328	2,06354	2,63633	53,21	0,107723	10,6873	0,08162	0,763652	0,6279808
CG2670-RA	Taf7	0,0299031	2,23523	0,0198944	0,0319782	0,0433146	0,0326445	0,460485	0,047566	0,13772387
CG2670-RB	Taf7	0,0335979	0,0163476	0,0177313	0,0362536	0,0491057	0,037009	0,460485	0,047566	0,6279808
CG2671-RA	l(2)gl	25,9197	29,2058	0,0118492	41,6748	0,0171487	16,8034	0,401596	0,108328	0,13772387
CG2671-RB	l(2)gl	0,0123422	0,0112421	0,0122686	0,0126605	0,0177736	0,0129243	0,399762	0,110317	0,13772387
CG2671-RC	l(2)gl	0,012779	0,01164	0,0122567	0,0131219	0,017756	0,0133953	0,401751	0,108147	0,6279808
CG2671-RD	l(2)gl	0,0127666	0,0116287	16,28	0,0131088	12,4256	0,013382	0,399569	0,110571	0,6279808
CG2671-RE	l(2)gl	17,3912	18,6613	42,8681	25,79	23,576	12,5588	0,399569	0,110571	0,6279808
CG2671-RF	l(2)gl	70,9495	82,9893	9,55468	44,3423	3,0085	30,9272	0,399569	0,110571	0,6279808
CG2674-RA	Sam-S	0,0283142	0,0257905	0,0268838	151,807	5,13803	0,0262187	-0,492065	0,052201	0,6279808
CG2674-RB	Sam-S	97,0642	90,3065	157,831	82,1714	80,853	0,0304293	-0,496314	0,051094	0,6279808
CG2674-RC	Sam-S	0,0280736	0,0255714	9,09261	45,4096	31,3293	128,308	-0,493812	0,052436	0,6279808
CG2674-RD	Sam-S	0,0282657	0,0257464	0,135067	0,0248505	0,0336602	0,209861	-0,492721	0,051968	0,6279808
CG2674-RE	Sam-S	0,0282657	0,0257464	31,3202	0,0256835	0,0347885	24,5035	-0,492065	0,052201	0,6279808
CG2674-RF	Sam-S	0,026831	0,0244395	121,574	135,468	4,49562	4,51719	-0,493372	0,051657	0,6279808
CG2674-RG	Sam-S	0,0287203	0,0261604	232,533	360,106	44,1467	49,3946	-0,472983	0,061605	0,13772387
CG2674-RH	Sam-S	0,0272965	0,0248636	0,0271833	0,030163	7,9967	12,0009	-0,493808	0,051532	0,6279808
CG2674-RI	Sam-S	42,9584	26,8597	124,741	117,375	12,9448	11,9536	-0,494617	0,051005	0,6279808
CG2674-RJ	Sam-S	0,0310848	0,0283141	0,0269523	0,0298893	4,82005	137,509	-0,494428	0,052149	0,6279808
CG2675-RA	Ugalt	15,0234	12,4974	10,4914	0,0228353	6,86483	3,32296	0,240343	0,301526	0,6279808
CG2675-RB	Ugalt	3,47303	3,10322	5,58895	12,8055	0,0309305	0,393161	0,23817	0,307521	0,6279808
CG2677-RA	eIF2Bbeta	12,7365	1,28275	20,8903	0,502955	2,22816	5,53266	-0,292206	0,213861	0,6279808
CG2678-RA	CG2678	1,91869	0,214952	0,258926	0,348342	1,91417	0,0983093	0,255197	0,374235	0,6279808
CG2679-RB	gol	2,20458	35,6357	0,0144778	22,5098	0,0143317	0,453413	-0,227207	0,314357	0,6279808
CG2679-RC	gol	0,0273984	0,00934476	0,0136963	2960,68	2,79	1,6204	0,059581	0,793756	0,6279808
CG2679-RD	gol	10,9785	0,00943855	8,49173	0,111582	0,19401	0	-0,230666	0,309307	0,6279808
CG2680-RB	CG2680	2,25323	14,7334	4,94839	21,0222	7,31904	6,92605	-0,267319	0,435297	0,6279808
CG2681-RA	CG2681	3,14027	1,38405	6,18252	11,0216	7,56345	20,6899	-0,412238	0,24313	0,6279808
CG2682-RA	d4	21,6597	25,2705	20,5446	20,2284	12,0781	18,5488	0,189853	0,367982	0,6279808
CG2682-RB	d4	0,0388986	0,0354316	0,037345	0,0425239	50,9123	49,3393	0,00988	0,960305	0,6279808
CG2682-RC	d4	15,9747	17,4361	21,9257	27,0937	0,0381491	0,0287515	0,189853	0,367982	0,6279808
CG2684-RA	lds	2,21311	1,81172	22,9318	3,65699	0,770079	1,63668	0,170821	0,551647	0,6279808
CG2685-RA	CG2685	6,66836	8,642	8,59006	11,9208	4,32809	0,0233111	0,060777	0,814374	0,6279808
CG2691-RA	CG2691	7,92203	5,89906	13,6108	0,0832469	7,10151	7,17163	0,00514	0,984501	0,6279808
CG2692-RA	gsb-n	3,07605	3,46194	1080,52	3,45342	1,68914	25,8243	0,065781	0,819285	0,6279808
CG2694-RA	CG2694	0,0214951	0,0191311	1,55516	0,0223853	0,0303211	0,0228518	-0,054324	0,835394	0,6279808
CG2694-RB	CG2694	0,0210031	1,29931	10,1947	1,65121	0,0352697	0,0265814	-0,054324	0,835394	0,6279808
CG2694-RC	CG2694	1,22094	9,38003	7,04504	12,0862	9,43724	11,9322	-0,026721	0,917751	0,6279808
CG2698-RA	CG2698	0,021324	110,357	20,4443	2,06708	1,91988	1,88888	-0,031968	0,885111	0,6279808
CG2698-RB	CG2698	19,5059	0,0194233	1,13861	0,0223398	0,0302594	0,0228053	-0,031934	0,885196	0,6279808
CG2699-RA	Pi3K21B	6,16616	29,4573	5,32124	8,08711	5,66905	5,37541	-0,154501	0,483491	0,6279808
CG2701-RA	CG2701	7,33969	21,4727	31,2576	31,7648	29,4461	32,7626	-0,290992	0,172142	0,6279808
CG2702-RA	Pbp95	8,40483	9,10626	153,241	0,0560532	0,0759244	4,09552	0,757262	0,002429	0,6279808

gene_id	Symbol	W1_FPKM	W2_FPKM	W3_FPKM	MCM51_FPKM	MCM52_FPKM	MCM53_FPKM	FC	p-value	p-adj
CG2706-RA	fs(1)Yb	0,396116	0,326447	0,416513	6,1567	0,602325	9,64	-0,376257	0,240287	0,13772387
CG2707-RA	fs(1)Ya	0,118045	0,0195792	0,0201643	0,0229466	0,0310813	0,0234248	-0,366718	0,305018	0,6279808
CG2708-RA	unc-45	16,7995	19,5666	19,4874	1,58953	0,869912	1,15753	-0,222145	0,424451	0,6279808
CG2709-RA	vilya	0,367599	0,161286	0,283326	0,308229	0,269338	0,616762	-0,029215	0,930601	0,6279808
CG2711-RA	dwg	5,81481	0,209272	0,926407	0,285262	0,818355	0,247719	-0,266579	0,203966	0,6279808
CG2712-RA	CG2712	2,09093	10,8163	3,79179	2,76999	3,08759	0	0,02416	0,917881	0,6279808
CG2713-RA	ttm50	23,8042	6,78851	0,0206366	8,52592	6,43791	7,42079	-0,358896	0,116525	0,6279808
CG2714-RA	dwg	3,87883	0,00467916	5,53485	6,54799	0,0245025	2,63122	0,162146	0,484078	0,6279808
CG2714-RB	dwg	0,731917	0,00463163	0,0167306	0,0180896	24,1228	3,25019	0,160186	0,489383	0,6279808
CG2715-RA	Syx4	15,2345	0,00474782	15,9644	11,1677	2,40349	18,1099	-0,366537	0,237886	0,6279808
CG2718-RB	Gs1	0,0280022	8,52642	0,0271368	0,0301079	0,00463555	594,304	0,237851	0,238479	0,6279808
CG2718-RC	Gs1	167,161	0,0255063	0,0271368	0,0301079	3,1245	12,1423	0,23744	0,239298	0,6279808
CG2720-RA	Stip1	35,5158	22,0459	41,4242	58,9899	32,3313	33,3733	-0,091631	0,657937	0,6279808
CG2723-RA	ImpE3	0,694447	0,484507	0,893676	38,0334	5,12044	15,2253	-0,812135	0,007007	0,6279808
CG2727-RA	emp	83,6217	66,3337	28,0544	81,3275	0,041025	34,8808	0,587942	0,016127	0,6279808
CG2727-RB	emp	0,0299981	20,7802	20,6927	0,0320872	38,8846	6,359	0,593116	0,015264	0,6279808
CG2727-RC	emp	0,0284238	0,0258904	0,0647314	0,0302878	558,646	0,0309189	0,593347	0,015277	0,6279808
CG2736-RA	CG2736	20,3354	24,93	1,40977	26,0766	17,7384	19,2834	0,125012	0,517574	0,6279808
CG2746-RA	RpL19	1894,57	19,1205	3,6699	0,0279971	0,0422684	0,0108012	-0,280285	0,299371	0,6279808
CG2746-RB	RpL19	0,0893317	0,0266469	0,0280611	3,98407	25,5739	1,73278	-0,280285	0,299371	0,6279808
CG2747-RA	CG2747	0,00976924	1,5083	1,0739	1,11376	5,98285	0,730814	-0,056643	0,793921	0,6279808
CG2747-RB	CG2747	9,13451	7,46473	7,23901	6,23072	1,13024	5,2464	-0,058039	0,788482	0,6279808
CG2750-RA	CG2750	0,0290827	1,25799	0,00758209	0,0356264	0,0108491	0,0920996	0,331486	0,224013	0,6279808
CG2759-RA	w	0,0421402	22,9573	0,107886	0,38695	0,100323	0,0184665	-0,173961	0,55281	0,6279808
CG2762-RA	ush	9,25789	11,1228	0,0248123	10,8418	4,22114	5,26956	0,418834	0,10067	0,6279808
CG2765-RA	CG2765	0,032961	0,0300232	0,0316445	0,0355111	0,0481001	0,0362511	0,143557	0,465118	0,6279808
CG2765-RC	CG2765	35,4352	41,3556	28,2717	20,1437	19,9075	22,4958	0,173622	0,383496	0,6279808
CG2765-RE	CG2765	33,1886	33,1924	35,2369	51,1891	29,7309	28,6953	0,172044	0,388108	0,6279808
CG2767-RA	CG2767	0,0498811	0,0454351	0,0478888	11,1717	10,5158	18,3982	0,424727	0,085581	0,6279808
CG2767-RB	CG2767	70,5865	53,3254	75,6676	12,6459	11,1116	9,6738	0,424727	0,085581	0,6279808
CG2772-RA	CG2772	1,48485	0,856587	4,13408	3,62008	2,73299	10,3832	-1,265997	0,00039	0,6279808
CG2774-RA	Snx1	20,1164	22,2616	13,3641	23,5793	19,1522	3,68877	-0,154382	0,479254	0,6279808
CG2781-RA	ELOVL	127,183	26,4351	6,45531	26,1528	19,3974	16,9335	0,873409	0,000513	0,6279808
CG2788-RA	Ugt36A1	8,46593	6,02234	767,165	7,73831	5,77786	14,9308	0,183652	0,42919	0,6279808
CG2789-RA	Tspo	31,8415	83,6662	0,185329	2,84899	0,278689	0,968562	-0,719651	0,006067	0,6279808
CG2790-RA	CG2790	7,37338	6,55711	203,861	8,09042	43,0411	6,79219	0,249281	0,29093	0,6279808
CG2791-RA	CD98hc	83,6619	92,4931	99,3275	123,622	87,1209	81,9048	-0,166991	0,346898	0,6279808
CG2794-RA	CG2794	6,3099	7,42916	12,0721	13,2022	18,8188	7,04626	-0,1088	0,631583	0,6279808
CG2803-RA	Tina-1	0,048419	4,18441	16,3381	0,0542086	0,0734259	0,0553382	0,223036	0,376812	0,6279808
CG2803-RB	Tina-1	172,273	0,039605	12,4067	279,244	174,486	167,847	0,22024	0,384769	0,6279808
CG2807-RA	Sf3b1	7,90548	6,00086	18,4861	4,03802	6,63058	0,372317	0,103397	0,711343	0,6279808
CG2811-RA	CG2811	31,3993	31,1135	48,1553	0,0454262	9,06544	50,0334	0,014746	0,959232	0,6279808
CG2812-RA	CG2812	7,05276	9,95515	8,18772	12,4381	5,92509	8,1327	-0,05035	0,841829	0,6279808
CG2813-RA	cold	78,9306	0,109165	0,14063	23,4659	92,9527	0,411406	-0,285679	0,231459	0,6279808
CG2816-RB	CG2816	5,07061	1,79181	1,16214	4,19363	4,26486	4,22038	-0,112257	0,738089	0,6279808
CG2818-RA	CG2818	4,69099	3,31725	0,923657	8,05955	8,60095	1,03469	-0,034018	0,868956	0,6279808
CG2818-RB	CG2818	9,97292	8,56658	5,22143	8,23309	120,409	7,11972	0,033286	0,873077	0,6279808
CG2819-RA	Pph13	0,474986	0,43265	0,219563	0,346723	0	18,9307	0,063727	0,858612	0,6279808
CG2822-RA	Shaw	0,0348012	6,18722	0,0334112	0,0376627	0	1,84388	-0,271299	0,291241	0,6279808
CG2822-RB	Shaw	1,42371	0,0132579	2,19834	3,94423	1,32338	2,96312	-0,035308	0,88443	0,6279808
CG2822-RC	Shaw	5,35637	0,0132989	5,12908	5,8382	0,0510144	2915,66	-0,00558	0,981742	0,6279808
CG2826-RA	lectin-21Ca	0	0	0	0,733324	0	0,703108	-0,041811	0,731452	0,6279808
CG2827-RA	Taldo	67,0851	61,3762	119,265	106,728	0,919099	3,54523	-0,938295	0,00361	0,6279808
CG2830-RA	Hsp60B	0,0460352	0,0419321	2,79236	0	1,38672	13,8	0,0677	0,712886	0,6279808
CG2835-RA	Galphas	73,5562	0,0335737	0,035132	18,1207	13,5685	57,4826	0,236787	0,229099	0,6279808
CG2835-RB	Galphas	0,036859	0,033332	37,3119	12,505	2,13155	0,0409272	0,236782	0,229078	0,6279808
CG2835-RC	Galphas	0,0365936	4,31665	7,9296	4,00777	23,8893	0,040606	0,23693	0,230039	0,6279808
CG2837-RA	snsl	30,8552	6,48276	0,0661718	0,0747477	0,110251	0,0717947	0,415949	0,113471	0,6279808
CG2837-RB	snsl	0,0641097	0,0458738	0,0642235	0,0801094	6,70549	6,12555	0,412794	0,116199	0,6279808
CG2837-RC	snsl	0,0680025	28,196	2,95297	38,9916	1,53108	0,0278354	0,41334	0,115808	0,6279808
CG2837-RD	snsl	30,6025	94,8076	0,630494	5,51908	0,0804408	0,060625	0,418948	0,109998	0,6279808
CG2839-RA	CG2839	0,0798983	2,09097	0,651854	1,52339	27,6604	77,1473	0,042922	0,889766	0,6279808
CG2841-RA	CG2841	0,0151492	0,013799	0,0145442	1,18519	0,021187	0,0159678	-0,136987	0,51942	0,6279808
CG2841-RB	CG2841	0,0136841	0,0124644	0,0131375	0,202609	0,758492	0,0143743	-0,135339	0,524544	0,6279808
CG2841-RC	CG2841	6,6902	5,67148	8,2842	0,0156419	6,09845	6,24885	-0,137218	0,519428	0,6279808
CG2843-RA	Cwc25	0,0382827	10,6149	13,3967	0,0432496	0,0585819	0,0441508	0,162532	0,517393	0,6279808
CG2845-RA	Raf	5,14608	1,20101	6,14174	6,20608	4,23807	4,43624	-0,145576	0,468392	0,6279808
CG2845-RB	Raf	0,0189022	5,08381	0,0181473	0,0196896	0,0266697	0,0200999	-0,145576	0,468392	0,6279808
CG2845-RC	Raf	1,27572	0,0172174	1,44146	2,46358	1,85069	1,22947	-0,098966	0,610108	0,6279808
CG2846-RA	CG2846	25,4478	20,1627	31,7714	21,5902	0,222625	0,0397423	-0,464126	0,137996	0,6279808
CG2848-RA	Tnpo-SR	3,87568	4,35608	9,91765	5,55683	13,7746	15,2905	0,424974	0,112543	0,6279808
CG2849-RA	Rala	26,3555	23,3596	21,6406	4,68713	0,0932398	21,0461	0,050407	0,80604	0,13772387
CG2849-RB	Rala	30,3374	31,8401	30,0215	1,15976	0,359056	0,0309759	0,054648	0,791939	0,6279808
CG2849-RC	Rala	0,0284728	0,025935	0,0273356	0,615756	0,552367	3,99968	0,051242	0,803044	0,6279808
CG2851-RA	Gsc	1,25605	16,4345	0	96,0763	0,54413	0	0,468692	0,098791	0,6279808
CG2851-RB	Gsc	1,01846	0,364031	1,31551	0,197469	0,784965	0	0,49808	0,082121	0,6279808
CG2852-RA	CG2852	335,549	329,661	482,141	0,0919606	15,313	18,631	-0,136345	0,564739	0,6279808
CG2854-RA	CG2854	0,321733	43,5172	55,6335	0,577401	2,14272	0,246982	0,049468	0,889938	0,6279808
CG2855-RA	aph-1	21,2195	20,9195	28,7344	27,6901	16,4397	24,0895	0,199335	0,375956	0,6279808
CG2857-RA	Tpc2	0,0566364	72,4288	37,1849	992,93	0	9,35868	0,602849	0,036305	0,6279808
CG2859-RA	Taf10	25,1253	21,4818	44,0999	0,0523149	0,0708608	37,0728	0,030638	0,912613	0,6279808
CG2861-RA	CG2861	0,135	11,6041	0,0450012	9,04239	1,41488	7,80528	0,36286	0,303125	0,6279808
CG2861-RB	CG2861	0,0352279	2,58117	256,065	2,214	0,215957	1,90783	0,189275	0,345732	0,6279808
CG2862-RA	CG2862	44,674	37,216	47,4697	42,849	295,507	38,7594	-0,231941	0,454647	0,6279808
CG2862-RB	CG2862	16,0039	11,109	55,5777	22,6496	203,425	81,1809	-0,232196	0,45421	0,6279808
CG2863-RA	Nle	10,3213	0,423849	0,546013	1,03073	0,660823	20,2009	-0,477069	0,11036	0,6279808
CG2864-RA	Parg	2,482	0,87772	1,51984	2,85158	1,65888	1,89354	0,206144	0,422525	0,6279808
CG2865-RA	CG2865	34,1485	0,366321	0,405409	0,24194	0	0	0,439013	0,108236	0,6279808
CG2867-RA	Prat	21,5054	0,414612	0,0160329	25,5702	18,3351	20,5419	-0,096225	0,685535	0,6279808
CG2871-RA	CG2871	0	0,0996465	0,105028	0	0	0	NA	NA	0,6279808
CG2872-RB	AstA-R1	2,20312	1,86052	1,52426	1,28384	1,78074	1,24751	0,186802	0,524821	0,6279808
CG2875-RA	CG2875	8,5257	6,56546	16,2745	9,75499	7,70484	8,60399	0,148515	0,600449	0,6279808
CG2875-RB	CG2875	0,0597911	0,0363079	0,057403	0,0436797	0,0591644	0,0445898	0,148515	0,600449	0,6279808
CG2885-RA	RabX2	0	0	0	1,45506	0	0	NA	NA	0,6279808
CG2887-RA	CG2887	0,133649	6,82427	0,205297	1019,53	4,26747	0,535532	-0,277979	0,390357	0,6279808
CG2889-RA	CG2889	6,01795	484,329	6,31742	0,25508	0,129534	5,08096	0,170677	0,4264	0,6279808
CG2890-RA	PPP4R2r	10,3336	12,3263	13,3212	4,42003	8,02896	0,0304018	-0,046868	0,870222	0,6279808
CG2890-RB	PPP4R2r	0,0279785	0,00742798	0,026861	15,49	0,0403389	3,45794	-0,049429	0,863106	0,6279808
CG2890-RC	PPP4R2r	2,32199	1698,7	7,17413	3,545	0,0405958	1555,22	-0,046951	0,869986	0,6279808
CG2893-RA	zyd	5,67402	5,16829	3,55246	3,88602	2,40399	10,5976	0,261329	0,199664	0,13772387
CG2893-RB	zyd	17,6057	22,6004	0,0316709	4,65057	0,0495694	0,0369887	0,268262	0,188847	0,6279808
CG2893-RC	zyd	11,6604	7,52833	17,8978	15,0446	13,9351	0,347414	0,265863	0,192865	0,6279808
CG2893-RD	zyd	0,0335808	0,0305877	13,5163	16,4233	11,2945	5,5608	0,26208	0,198738	0,6279808
CG2898-RA	CG2898	0	0,25294	0,0861913	0,109634	0,396096	0,180278	-0,251122	0,364265	0,6279808

gene_id	Symbol	W1_FPKM	W2_FPKM	W3_FPKM	MCM51_FPKM	MCM52_FPKM	MCM53_FPKM	FC	p-value	p-adj
CG2899-RA	ksr	6,71898	10,4526	0,0132252	13,5681	0,0496245	0,0144732	-2,185593	1,35E-23	0,6279808
CG2901-RA	CG2901	0,0508276	0	0	0	0,0158007	1,5243	-0,203338	0,319858	0,6279808
CG2902-RA	Nmdar1	7,91616	1,20577	0,0137856	90,0547	0,0177102	0,0151071	-0,118022	0,647662	0,6279808
CG2903-RA	Hrs	4,57448	4,39076	3,76791	5,41427	2,59662	3,22349	-0,023605	0,918156	0,6279808
CG2903-RB	Hrs	0,0793228	0,0164729	0,0173625	0,0188019	0,0254673	0,0191937	-0,063979	0,779115	0,6279808
CG2903-RC	Hrs	0,0243553	0,306033	0,39424	0,724062	0,422643	0,394309	-0,063979	0,779115	0,6279808
CG2903-RD	Hrs	7,22109	4,81836	0,0247735	5,35392	4,32781	5,06257	-0,622021	0,024829	0,6279808
CG2904-RA	ec	3,33464	6,35431	1,83492	0,0147391	0,0201855	3,24124	0,095368	0,716425	0,6279808
CG2904-RB	ec	4,30393	3,97427	3,54445	2,38499	0,0199643	3,25184	0,098941	0,707519	0,6279808
CG2904-RC	ec	5,20277	3,53562	4,93106	0,0116653	1,81729	0,124407	0,094629	0,718066	0,6279808
CG2906-RC	CG2906	0,0415833	0,567797	0,718152	0,834043	0,602661	0,0485133	-0,025779	0,909214	0,6279808
CG2906-RD	CG2906	5,6322	0,364767	0,133084	0,279114	0,250381	5,43355	-0,025779	0,909214	0,6279808
CG2909-RA	Dsim\GD24700	0,0337007	30,007	0,0323547	0,0363737	7,90357	12,25	-0,278636	0,341666	0,6279808
CG2910-RA	nito	8,63482	2,66956	2,65386	0,022725	0,0307812	0,0231986	0,046815	0,854036	0,6279808
CG2910-RB	nito	0,0216737	4,09731	9,20574	16,549	7,78353	7,04652	-0,177388	0,46643	0,6279808
CG2911-RA	Dsim\GD19598	0,0712279	24,2769	6,85264	27,2765	18,6494	4,09193	0,126717	0,636109	0,6279808
CG2911-RB	Dsim\GD19598	6,82318	9,39231	19,1363	14,4895	0,0342019	13,2332	0,076571	0,760703	0,6279808
CG2913-RA	yin	0,0261825	0,0238488	0,0251367	0,0277495	4,15294	4,80847	-0,104513	0,63076	0,6279808
CG2913-RB	yin	5,2421	4,93044	4,35161	5,58653	1,13418	0,026404	-0,114086	0,601393	0,6279808
CG2913-RC	yin	0,820843	0,903913	0,940965	1,73358	17,0814	17433,9	-0,114086	0,601393	0,6279808
CG2914-RA	Ets21C	1,94865	0,0380766	0,0401329	105,641	0,0623468	0,0162887	-0,0075	0,979951	0,6279808
CG2914-RB	Ets21C	1,03889	6,1231	10,3	0,0460291	11,4446	0,671848	0,17195	0,569022	0,6279808
CG2915-RA	CG2915	18,6831	0,30517	0,0983516	0,285028	0	29,2018	-0,463506	0,045344	0,6279808
CG2915-RB	CG2915	3,13517	0,214441	0,226021	0,230235	0	4,33084	-0,465095	0,044969	0,6279808
CG2916-RA	38596	1,83039	0,018316	0,0193052	0,0652703	30,2877	0,0218143	-0,259386	0,45433	0,6279808
CG2916-RB	38596	0,0391986	0,0519445	0,0547497	9,71696	0,0569012	0,0666303	-0,259386	0,45433	0,6279808
CG2917-RA	Orc4	1,85259	21,4877	4,99576	10,7192	2,76768	22,427	-0,354654	0,252527	0,13772387
CG2918-RA	CG2918	43,8581	0,39402	0,674859	3,39932	0,1497	0,318851	0,101798	0,693716	0,6279808
CG2919-RA	asl	2,49736	3,31737	481,009	15,8593	1,53816	380,303	0,200019	0,503472	0,6279808
CG2921-RA	CG2921	6,43702	23,2828	29,3988	5832,52	3,53071	8,50508	-0,297963	0,19212	0,6279808
CG2922-RA	kra	64,5936	41,6191	104,493	53,5474	45,3534	59,5092	0,138362	0,634124	0,6279808
CG2922-RB	kra	0,039598	0,0360686	0,0380164	0,0433635	0,0587361	0,0442671	0,125012	0,668987	0,6279808
CG2922-RC	kra	30,0106	21,078	0,0321742	33,0615	0,048971	0,0369075	0,123672	0,672032	0,6279808
CG2922-RD	kra	0,036859	0,0335737	0,0353868	0,0400918	0,0543046	0,0409272	0,124115	0,670864	0,13772387
CG2922-RE	kra	0,0347463	0,0316493	0,0333585	0,0375982	0,050927	0,0383817	0,129739	0,658085	0,6279808
CG2922-RF	kra	0,032523	0,0296242	0,031224	0,0350018	0,0474102	0,0357312	0,126171	0,6662	0,6279808
CG2922-RG	kra	156,227	78,761	275,534	114,349	144,773	188,383	0,129402	0,659042	0,6279808
CG2924-RA	CG2924	20,175	20,9799	26,7994	24,7036	10,2607	11,3105	0,308897	0,271793	0,13772387
CG2924-RC	CG2924	0,03019	0,0274991	0,0289842	7,39187	0,0437606	0,0329806	0,308171	0,272819	0,6279808
CG2925-RA	noi	11,2549	10,0418	16,5399	9,73189	4,63247	4,00438	-0,11582	0,634172	0,6279808
CG2926-RA	CG2926	6,38671	6,15681	7,33666	17,548	8,82425	12,6436	0,189715	0,512297	0,6279808
CG2928-RA	Reg-5	36,3138	0	3,8734	3,02521	0	6,02337	0,236772	0,423147	0,6279808
CG2929-RA	Pi4KIIalpha	1,1214	0,025029	12,1102	0,0227407	8,84986	8,72009	-0,232715	0,264345	0,6279808
CG2929-RB	Pi4KIIalpha	5,62363	0,445002	10,6042	51,4248	5,54901	6,29318	-0,141404	0,505257	0,6279808
CG2929-RC	Pi4KIIalpha	0,0216879	0,305515	1529,76	23,0676	47,805	48,7621	-0,232715	0,264345	0,6279808
CG2930-RA	CG2930	31,2191	34,6463	31,5823	22,7385	34,6647	26,8463	-0,018164	0,935472	0,6279808
CG2930-RB	CG2930	18,0392	13,8942	38,3788	30,0834	0,0370408	20,7266	-0,018075	0,935791	0,6279808
CG2930-RD	CG2930	0,0258243	0,0235226	0,0247929	0,0273464	1,00009	0,0279162	-0,323242	0,179152	0,6279808
CG2931-RA	CG2931	12,2814	0,0117674	0,0250375	2,25477	0,0373978	0,0282089	0,087747	0,718577	0,6279808
CG2932-RA	Klf15	0,652517	0,969741	0	0,0798674	0,0636359	0	-0,18947	0,59633	0,6279808
CG2934-RA	VhaAC39-1	115,926	141,175	1,74224	149,419	0,463189	0,864133	-0,028629	0,904052	0,6279808
CG2937-RA	mRpS2	25,2628	25,5717	47,851	4,07791	2,46346	17,2593	-0,251943	0,376732	0,6279808
CG2938-RB	CG2938	3,86583	4,90268	5,90021	1,25255	0,454375	0,202811	0,286954	0,186734	0,6279808
CG2939-RA	slp2	7,76952	8,03337	6,37056	36,2378	3,85495	19,9475	0,099207	0,714883	0,6279808
CG2941-RA	Glos	1,08658	1,0254	1,29693	4,18008	3,55101	0,41727	0,192749	0,533233	0,6279808
CG2943-RA	EMC1	20,6057	45,6265	3,72947	36,5683	17,1458	17,6915	-0,178412	0,414187	0,6279808
CG2944-RA	gus	30,9621	23,31	30,9586	95,4602	3,85898	4,30569	-0,089927	0,668501	0,6279808
CG2944-RB	gus	0,0350411	0,0319178	0,0336415	2213,91	0,00786938	0,00593084	-0,085233	0,683318	0,6279808
CG2944-RC	gus	0,0366748	0,033406	0,03521	0,0904458	19,1009	22,9625	-0,089862	0,66901	0,6279808
CG2944-RD	gus	0,037338	0,03401	0,0358467	3119,71	0,0575989	0,04341	-0,090099	0,668337	0,6279808
CG2944-RE	gus	0,0374226	0,0340871	0,0359279	0,0751741	9,05667	11,8579	-0,089862	0,66901	0,6279808
CG2944-RF	gus	76,5245	72,4618	64,0571	0,0887405	2,0506	3,1175	-0,090099	0,668337	0,6279808
CG2945-RA	cin	11,5395	8,4762	17,8153	0,029136	0,0394649	0,0297431	0,209139	0,340899	0,6279808
CG2945-RB	cin	0,0317265	0,0288987	0,0304594	13,7353	9,74755	10,9442	-0,182156	0,456013	0,6279808
CG2947-RA	Dsim\GD16651	0,032523	0,0130935	10,2357	15,815	12,0471	14,8097	0,768151	0,006484	0,6279808
CG2947-RB	Dsim\GD16651	25,917	7,55087	3,9795	4,73492	0	0,0151241	0,768151	0,006484	0,6279808
CG2948-RA	rev7	2,37962	1,76426	3,29404	3,42005	2,65786	3,84193	-0,296703	0,374605	0,6279808
CG2950-RA	mxt	0,0291126	0	1,79707	0,031088	1,12074	11,6872	-0,293239	0,194706	0,6279808
CG2950-RB	mxt	6,4804	0	0,143848	33,6898	0,026822	0,0198566	-0,293818	0,193538	0,6279808
CG2950-RC	mxt	12,854	0,113731	17,7039	0	5,14038	1,34541	-0,294201	0,193063	0,6279808
CG2952-RA	PPO3	0,134361	60,1555	0,171993	0,155217	40,7705	0,0611208	0,000644	0,998457	0,6279808
CG2955-RA	CG2955	0	4,51833	2,78913	9,37902	14,1405	4,18978	-0,032096	0,86244	0,6279808
CG2956-RA	twi	0,484743	9,03309	3,95901	4,92651	7,43176	15,529	0,328047	0,29314	0,6279808
CG2956-RB	twi	2,26081	23,3834	8,42791	5,77592	14,7574	43,1905	0,322674	0,3018	0,6279808
CG2957-RA	mRpS9	43,8932	13,9397	67,0702	53,6587	49,6622	0,0189344	-0,062465	0,786157	0,6279808
CG2958-RA	lectin-24Db	0,255668	0,23288	0,981825	0,782627	11,5829	21,4813	0,167394	0,639086	0,6279808
CG2960-RA	RpL40	1137,41	1,10394	2,07216	0	2,23141	29,6066	-0,275776	0,325739	0,6279808
CG2961-RA	Ipod	3,49464	5,66647	1,35936	0,0135537	1,7178	0,0138361	-0,589697	0,049968	0,6279808
CG2962-RA	CG2962	1013,63	4,27179	9,49503	2,85725	2,83805	3,70166	0,49315	0,09628	0,6279808
CG2964-RA	CG2964	0,0352091	0,0320709	10,9658	0,0381423	0	5,15687	0,363305	0,30866	0,6279808
CG2968-RA	Dpse\GA22488	197,63	174,296	378,037	308,399	579,334	516,066	-0,545743	0,079062	0,6279808
CG2969-RA	Atet	0,0174819	0,0159237	0,0167837	0,0181494	1,32602	0,0185276	-0,056511	0,757784	0,6279808
CG2969-RB	Atet	0,0160586	0,0146273	0,0154172	0,0166161	0,0501543	0,0169624	-0,045582	0,802916	0,6279808
CG2969-RC	Atet	10,3168	9,04748	8,29532	13,3325	0	9,51895	0,003051	0,986655	0,6279808
CG2969-RD	Atet	12,2654	11,9212	14,8573	11,5664	21,8013	8,84216	-0,041242	0,823269	0,6279808
CG2970-RA	Stoml2	19,8569	19,4852	32,35	2,16737	32,8514	27,3027	-0,246223	0,379165	0,6279808
CG2972-RA	CG2972	12,4028	0,0879919	0,844478	0,0282977	1,23594	0,757858	-0,244894	0,384788	0,6279808
CG2973-RA	Cpr23B	26,9152	0	0,0360504	22,5881	0,195075	0,11905	0,644523	0,005292	0,6279808
CG2974-RB	Naxe	3,71925	9,08581	0,441842	15,4749	0,162565	0,0516023	0,156877	0,618065	0,6279808
CG2975-RA	CG2975	5,594	0	0,0728043	0,053635	3,88874	5,28691	-0,537173	0,034438	0,6279808
CG2976-RA	Fnta	10,5175	9,63417	38,6393	0,0306541	19,1237	25,4868	-1,273159	7,3E-05	0,6279808
CG2977-RA	Inx7	0,0403475	7,11138	84,7807	0,0442665	9,59273	14,2315	-0,257619	0,285489	0,6279808
CG2977-RB	Inx7	38,6187	36,6369	6,40792	51,2051	30,2671	40,2494	-0,207888	0,381017	0,6279808
CG2979-RA	Yp2	0,276557	0,213152	0,0415797	21,647	3,9808	5,16568	0,219904	0,5099	0,6279808
CG2980-RA	thoc5	5,39262	10,5479	23,4843	7,08186	4,75436	23,7916	-0,113331	0,632126	0,6279808
CG2981-RA	TpnC41C	14,4391	6,13642	0,0222689	15,7738	9,72137	10,8924	0,0378	0,913054	0,6279808
CG2982-RA	NO66	0,0301624	0,027474	0,0289577	1,65066	95,2331	4,00527	0,058953	0,807436	0,6279808
CG2982-RB	NO66	9,27308	8,60141	14,3334	6,23363	3,22333	0,463821	0,058953	0,807436	0,6279808
CG2983-RA	CG2983	0	0,069074	358,066	0,0409133	0,0554173	1,87993	0,250774	0,411007	0,6279808
CG2984-RA	Pp2C1	10,1369	13,2754	12,1356	0,0290333	6,69545	12,8154	-0,03447	0,899024	0,6279808
CG2985-RA	Yp1	0,0631298	0,287515	0,101014	0,296123	293,544	0,118846	-0,074648	0,809731	0,6279808
CG2986-RA	RpS21	0,149945	5,98658	20,6973	5,26521	6,35319	1,41429	-0,580783	0,029763	0,6279808
CG2986-RB	RpS21	2180,84	3,31254	1,92221	5,51685	0,0276787	4,74267	-0,57817	0,030591	0,6279808
CG2986-RE	RpS21	0,106803	15,5732	5,91431	3,08758	7,71211	13,7431	-0,580808	0,029759	0,6279808

gene_id	Symbol	W1_FPKM	W2_FPKM	W3_FPKM	MCM51_FPKM	MCM52_FPKM	MCM53_FPKM	FC	p-value	p-adj
CG2987-RA	alpha-Catr	5,72368	5,67548	2,79569	0,0473523	6,25035	4,48829	-0,017156	0,943103	0,6279808
CG2988-RA	ems	9,46038	51,7305	7,50686	1,08888	0,0483607	0,0364475	0,481668	0,10576	0,6279808
CG2989-RA	Cht6	20,7936	0,786092	59,4404	0,0578748	3,44372	47,892	0,430619	0,19367	0,6279808
CG2989-RB	Cht6	3,29341	0,0335363	79,4997	63,1296	0,727264	0,0288873	0,528594	0,108643	0,6279808
CG2990-RB	CG2990	0,375778	0,258801	2,75333	0,0478683	3,01622	0,311607	-0,033984	0,913698	0,6279808
CG2990-RC	CG2990	0,375466	0,250244	6,79323	397,431	0,0991292	0,310819	-0,033984	0,913698	0,6279808
CG2991-RA	CG2991	7,9613	7,25171	7,70774	18,5857	31,7883	6,11016	0,236634	0,2903	0,6279808
CG2991-RB	CG2991	0,0217022	0,0197678	0,0208354	8,19171	8,77111	0,0232306	0,238281	0,287312	0,6279808
CG2991-RC	CG2991	27,851	30,2876	21,9563	5,03895	3,6508	16,3929	0,254778	0,257196	0,6279808
CG2993-RA	CG2993	1,86877	0,187107	0,197212	0,0197783	0,360445	2,54638	-0,474181	0,08392	0,6279808
CG2995-RA	Dsim\GD16494	5,95808	6,60381	5,78821	49,2618	3,99025	9,33917	0,216242	0,388846	0,6279808
CG2998-RA	RpS28b	1914,6	2,96078	0,0217348	1,58541	2,36627	3,10403	-0,627094	0,017115	0,6279808
CG2999-RA	unc-13	3,59261	5,45814	3,30514	3,75397	11,5771	17,2783	0,310463	0,180873	0,6279808
CG2999-RB	unc-13	6,70873	6,56451	6,5091	6,11379	0,0186775	0,0140765	0,370336	0,113097	0,6279808
CG2999-RC	unc-13	3,6888	2,89153	4,00254	5,86183	12,1061	4,88884	0,313017	0,17807	0,6279808
CG30000-RA	fzr	9,18067	13,3812	10,1322	8,27037	4,06389	8,11872	-0,449596	0,120049	0,13772387
CG30001-RA	fzr	11,9326	11,0076	7,85036	5,52517	8,2622	3,65511	0,015269	0,962073	0,6279808
CG30001-RB	GstT1	19,6758	13,723	22,5781	20,5963	30,5729	28,5745	0,043299	0,901736	0,6279808
CG30002-RA	CG30001	0,827367	0,563368	0,97419	1,05693	0,464619	0,391463	-0,038632	0,913063	0,6279808
CG30005-RA	CG30001	0,064172	0,436299	0,0180338	0,0748326	0,0264955	0,280967	-0,134958	0,63331	0,6279808
CG30007-RA	CG30002	0,423563	0,0115929	0,32919	0,0130672	0,22479	45,3937	0,264858	0,34544	0,6279808
CG30007-RB	GstT2	0,0847487	4,40011	0,0813638	5,77102	0,294456	0,287974	0,209738	0,462576	0,6279808
CG30008-RA	tea	1,2025	1,31123	1,41534	0,114491	0,0706656	0,0634261	-0,467283	0,155676	0,6279808
CG3000-RA	tea	0,666386	0,46825	0,456979	22,7675	2,16527	1,74396	0,009121	0,972725	0,6279808
CG3000-RB	CG30008	1,32295	19,5378	19,0338	13,1595	0,126863	0,0956116	0,018778	0,943545	0,6279808
CG30010-RA	Hex-A	39,0818	30,9769	6,04181	4,56969	1,27964	3,57579	0,088748	0,766789	0,6279808
CG30011-RA	CG30010	4,50517	4,40285	8,74058	6,60755	6,98728	5,35197	0,062876	0,763191	0,6279808
CG30011-RC	gem	7,21005	6,86943	5,24929	3,63445	4,66889	4,18912	0,063064	0,762487	0,6279808
CG30015-RA	gem	22,0005	25,3509	25,5437	9,54235	16,9486	16,6751	-0,180731	0,40752	0,6279808
CG30015-RB	CG30015	10,8703	11,7553	338,2	0	0,0305808	0,0230475	-0,200788	0,359012	0,6279808
CG30015-RC	CG30015	0,0107779	0,00981728	40,684	0,029594	24,7765	26,7527	-0,200767	0,359019	0,6279808
CG30015-RD	CG30015	0,0105306	0,00959198	0,0569109	98,37	0,0437606	0,0329806	-0,137704	0,52986	0,6279808
CG30016-RA	CG30015	2,5116	2,18249	15,9297	8,50677	0,0437606	0,0329806	-0,944563	0,004737	0,6279808
CG30017-RA	CG30016	6,08594	91,2756	94,8498	102,834	0,0235893	0,0177783	0,034	0,851122	0,6279808
CG30018-RC	CG30017	0	0,133303	0,0635214	0,222968	0,0368573	0,0277779	1,642205	3,27E-06	0,6279808
CG3001-RB	mthl13	1,19703	5,59305	9,93102	7,47829	0,0207294	5,39656	-0,034418	0,888677	0,6279808
CG30020-RA	Gga	3,98519	3,0424	55,2051	3,61766	3,08555	35,4663	0,099674	0,677412	0,6279808
CG30021-RA	CG30020	5,76366	7,00207	7,6217	8,78405	0,0399775	0,0301295	0,282897	0,207313	0,6279808
CG30021-RB	metro	11,61	0,211181	2,04348	0,0225771	1,66731	11,3523	0,28468	0,20449	0,6279808
CG30021-RC	metro	0,0249563	277,74	17,2152	60,1029	14,4165	0	0,280004	0,212326	0,6279808
CG30022-RB	metro	0,0239001	0,287445	0,30208	0,0323074	0,248992	2,7704	-0,18247	0,523423	0,6279808
CG30023-RA	CG30022	10,3792	12,9048	0,0641639	12,1853	0,037698	0,0801227	0,387213	0,1435	0,6279808
CG30025-RA	sprt	10,4783	6,93507	13,2932	0,0353587	0,106311	8,31259	0,01562	0,897983	0,6279808
CG30026-RA	CG30025	5,63639	2,25238	45,5796	7,16264	24,0152	15,7258	0,131561	0,616881	0,6279808
CG30028-RA	CG30026	5,09264	4,56076	5,5877	5,54707	2,32596	4,0669	0,01562	0,897983	0,6279808
CG3002-RB	gammaTry	5,62869	3,39	45,1889	15,7159	12,8513	15,101	0,281574	0,232848	0,6279808
CG30030-RA	CG42797	1,37697	1,36592	17,164	14,7407	3,84979	13,289	0,22722	0,516618	0,6279808
CG30031-RA	Gr47b	0,238829	0,362569	48,2705	0,474031	25,024	34,5315	0,07663	0,535079	0,6279808
CG30033-RB	CG30031	5,74385	1,10145	45,3179	16,3562	12,7004	16,0742	-0,19304	0,522201	0,6279808
CG30034-RA	CG30033	0,069286	0,0631105	0,0665187	2504,89	10,0887	4,06117	-0,018276	0,937726	0,6279808
CG30035-RA	CG30034	0,0938578	0,0854922	0	7,85058	0	0,190416	-0,415577	0,062121	0,6279808
CG30035-RB	Tret1-1	7,36036	6,37985	151,293	0,0444157	1,32548	10,3856	-0,224308	0,340331	0,6279808
CG30036-RA	Tret1-1	26,5066	26,2168	0,0537751	8,12028	0,627753	5,62164	0,819948	0,015935	0,6279808
CG30037-RA	CG30036	0,0504909	45,2221	38,7669	59,2296	6,98327	4,53421	0,044335	0,715924	0,6279808
CG30038-RA	CG30037	0	0,0676253	0,0712773	0	12,3693	0	0,364743	0,268186	0,6279808
CG30039-RA	MCPH1	7,11075	12,9901	7,13185	13,026	0	5,57035	-0,013096	0,914398	0,6279808
CG3003-RB	CG30039	0,135959	45,1677	0,0313474	0,0718149	0,024422	0,0733114	-0,524685	0,070226	0,6279808
CG30040-RA	Lst8	15,0114	14,1321	1,53023	39,7591	9,64948	1,42381	0,332432	0,131691	0,6279808
CG30042-RA	jeb	4,72497	1,88991	5,10948	5,13644	3,34602	5,67355	-0,223356	0,531169	0,6279808
CG30043-RA	Cpr49Ab	0,720058	2,46986	4,52141	1,91967	1,76049	0,102069	-0,234657	0,5016	0,6279808
CG30044-RA	CG30043	1,85401	0,0448929	2,73203	0,411644	0,201572	1,9484	-0,38895	0,182028	0,6279808
CG30044-RB	s-cup	1,44544	0,0237079	3,72607	0,0275753	0,0474606	0,639749	-0,382315	0,189048	0,6279808
CG30045-RB	s-cup	0,0260278	0,931849	1,84565	0,760793	3,92795	4357,36	0,173497	0,609985	0,6279808
CG30046-RB	Cpr49Aa	2,78289	0,308649	0,133833	3,89094	34,4245	3,2592	-0,017136	0,954626	0,6279808
CG30047-RA	CG30046	1,92575	0,185761	0,021297	6,15381	3,2362	0	0,011515	0,973803	0,6279808
CG30048-RA	CG30047	0,362917	0,0683937	5,15938	0,391214	0,0174504	6,60159	0,01562	0,897983	0,6279808
CG30048-RB	CG30048	0	0	0	0	466,776	23,1893	NA	NA	0,6279808
CG30049-RA	CG30048	0	0	0	1,00092	0,0749049	1365,09	-0,865528	0,009496	0,6279808
CG3004-RA	CG30049	0,424925	486,188	0,804575	0,150184	1,02411	0,56508	0,243352	0,256258	0,6279808
CG30050-RB	CG30050	1,57082	1,01505	0,389183	1,63391	1,18429	0	0,095234	0,788011	0,6279808
CG30051-RC	Tmem18	5,04226	2188,74	0,0977954	3912,08	0,588283	0,0239247	-0,200051	0,484726	0,6279808
CG30052-RA	Obp49a	4,5886	4,37865	11,6636	6,85337	13,1622	11,7192	-0,337259	0,298384	0,6279808
CG30053-RA	CG30053	0,819393	0,925487	1,10133	8,44649	11,621	9,22152	-0,22156	0,513261	0,6279808
CG30054-RD	CG30054	4,26895	3,49397	2,46501	4,69392	0,504411	0,966952	0,105361	0,732053	0,6279808
CG30055-RA	lncRNA:CR30055	15,485	16,4709	16,7793	13,4635	14,6861	10,7117	0,259473	0,277202	0,6279808
CG30056-RA	CG30056	1,0879	2,45127	1,09942	4,53069	0,16212	1,58348	-0,042568	0,905326	0,6279808
CG30058-RA	CG30058	0,0638002	60,6061	17,2413	14,3406	17,6527	0,0758749	0,075365	0,83119	0,6279808
CG30059-RA	CG30059	3,86715	3,72748	0,0392878	44,2235	27,3359	1,32655	1,113396	0,000424	0,6279808
CG30060-RA	Fmo-1	30,1591	79,9353	0,0353868	6,35366	23,7579	8,50629	-0,066873	0,810942	0,6279808
CG30062-RA	CG30060	0	6,51773	0,019495	7,83825	0,021457	1542,41	0,05248	0,878825	0,6279808
CG30065-RA	CG30062	0,318694	0,290289	25,3527	1,38478	75,0176	0,0146657	-0,19057	0,470533	0,6279808
CG30067-RA	CG30065	0	4,44041	0,0249457	0,0281526	3,67408	0,123304	-0,566757	0,052113	0,6279808
CG30067-RB	Obp50a	0	12,3053	0,214918	3,25034	0	0	-0,566757	0,052113	0,6279808
CG30069-RA	Obp50a	0	0,0287744	114,344	2,8716	55,4912	2,35483	0,298843	0,212709	0,13772387
CG3006-RA	CG30069	15,7787	19,0799	13,5639	21,3638	0,00507091	10,0666	0,532421	0,047389	0,6279808
CG30071-RA	CG30071	0	0	18,2836	0,220363	0,00507524	0	-0,041811	0,731452	0,6279808
CG30072-RA	Obp50c	0,167894	1,97462	1,91476	2,59304	2,62583	2,60234	-0,485403	0,160874	0,6279808
CG30073-RA	Obp50b	0,0702935	12,29	0,789922	0	10,8134	7,28411	-0,843187	0,007039	0,6279808
CG30074-RA	Obp50d	0,696202	0,345899	0,0674859	0,498267	3,74514	0,50865	0,236396	0,479942	0,6279808
CG30075-RA	CG30075	0	0	3,64949	12,3755	7,77897	7,27294	NA	NA	0,6279808
CG30076-RA	CG30076	0,549384	0,50765	0,634389	124,335	0,0870296	0,497771	-0,054619	0,878609	0,6279808
CG30077-RA	Blos1	19,0427	4,89107	28,4144	5,24456	6864,7	7,00997	-0,157644	0,562719	0,6279808
CG30080-RA	CG3008	13,4364	0	0,607653	0,0310733	16,7641	0,19402	-0,181243	0,52335	0,13772387
CG30081-RA	CG30080	5,99079	413,119	447,866	2,29558	0,0695358	755,052	-0,009238	0,978969	0,6279808
CG30083-RB	Ir51b	0,313069	0,0116852	13,4648	18,4318	0,38293	7,92724	0,044335	0,715924	0,13772387
CG30084-RA	CG30083	0	4,59579	4,75322	0,0493748	0,0668785	1,72446	-0,416851	0,107692	0,6279808
CG30084-RC	Zasp52	0,0192491	50,283	0,0184803	16,8615	14,6181	19,2957	-0,432689	0,097629	0,6279808
CG30084-RE	Zasp52	58,0741	0,00817677	58,2566	15,8236	0,0152734	0,011511	-0,44792	0,090207	0,13772387
CG30084-RF	Zasp52	0,00897689	14,5274	0,00861835	0,0482892	2,62148	3,54312	-0,433957	0,096767	0,6279808
CG30084-RG	Zasp52	11,2354	14,5295	12,4573	0	0,014618	0,011017	-0,446415	0,090136	0,6279808
CG30084-RH	Zasp52	13,1823	0,039762	18,1927	0,0340434	3,35007	2,51463	-0,545797	0,029754	0,6279808
CG30085-RA	Zasp52	0,0436528	0,563544	0,0419093	43,21	0,0144477	0,0108886	-0,019686	0,94606	0,6279808
CG30087-RA	Rif1	4,352	4,98942	6,78697	8,90961	2,54401	4,177	-0,074864	0,550506	0,6279808
CG30088-RB	CG30087	0	19,1145	10,3953	0,152876	3,35213	0	-0,041811	0,731452	0,13772387

gene_id	Symbol	W1_FPKM	W2_FPKM	W3_FPKM	MCM51_FPKM	MCM52_FPKM	MCM53_FPKM	FC	p-value	p-adj
CG30089-RA	CG30088	0	0,162666	0,0182202	0,220349	2,97989	2,70117	0,2754	0,325499	0,6279808
CG3008-RA	CG30089	14,6559	22,1509	13,154	20,5749	1026,87	9,75906	-0,252136	0,238698	0,13772387
CG30090-RA	CG3009	4,3574	2,96121	4,45876	1,53088	3,68865	2,68826	0,163601	0,609686	0,6279808
CG30091-RA	CG3009	0,0501841	0,0457112	0,0481798	0,0284075	0,0764446	0,0576133	-1,314197	9,38E-05	0,6279808
CG30092-RB	CG3009	0,0330434	4,81572	0,0317237	33,0194	3,51595	4,23288	-0,668696	0,00516	0,6279808
CG30092-RC	CG30090	2,59055	0	14,913	2,17677	0,0176996	0,0133395	-0,329443	0,152078	0,6279808
CG30092-RD	CG30091	0,814978	0,323583	1,16361	2,52756	8,23406	10,2262	-0,391649	0,058004	0,6279808
CG30093-RA	jbug	27,1891	29,2667	25,2791	43,7412	0	0,12812	-0,415561	0,236603	0,6279808
CG30094-RA	jbug	27,784	25,6335	25,5235	0,0388164	0	0	0,530155	0,064634	0,6279808
CG30095-RA	jbug	0,0129138	0,0117628	0,0123981	0,0132645	0	0	-0,037346	0,866315	0,6279808
CG30096-RB	COX6AL	0,313453	8,31473	5,06623	0,20232	4,73444	1,12954	0,064557	0,804846	0,6279808
CG30098-RA	CG30094	10,7577	8,7772	0,0313165	15,35	0,0475618	0,0358454	0,093957	0,582148	0,13772387
CG30099-RA	CG30095	0,143435	0,130651	0,137706	59,5551	0	0,332686	-0,62138	0,082496	0,13772387
CG3009-RA	CG30096	7,47798	3,46116	5,67871	22,4576	2,15648	2,66495	-0,074879	0,795732	0,6279808
CG3009-RB	CG30098	0	0,18599	0,196035	0	0	0	-0,104603	0,718638	0,6279808
CG3009-RC	CG30099	0,3031	0	0,436492	1,47473	0,0417543	0,483722	-0,466189	0,125369	0,6279808
CG30100-RA	CG30100	12,3179	10,1369	49,6786	195,644	6,62002	14,8177	-0,640307	0,036708	0,6279808
CG30101-RA	Vajk4	304,781	0,0537538	3,92971	157,016	245,842	0,340979	0,547442	0,038483	0,6279808
CG30103-RA	CG30103	0,0506717	0	0,0184642	0,0206912	0,0258434	0,0204668	0,01562	0,897983	0,6279808
CG30104-RA	NT5E-2	1,82619	1,37211	3,37483	0,0200491	0	1,6761	0,166516	0,622573	0,6279808
CG30104-RB	NT5E-2	0,0331761	0,0175181	23,9818	2,93875	20,1524	14,2648	0,163821	0,628014	0,6279808
CG30105-RA	CG30105	2,88558	0,0374527	0,0394753	14,4391	2011,87	94,8924	-0,29278	0,370149	0,6279808
CG30106-RA	CCHa1-R	0,969113	0,184013	0,223789	6,09205	2,75387	0,139912	0,088108	0,776615	0,6279808
CG30108-RB	CG30108	18,5423	0,939815	0,0332652	0,20622	10,5114	15,3665	-0,748406	0,013056	0,6279808
CG30109-RA	CG30109	15,7147	3,18634	0,121526	0,0591211	5,81702	0,0394625	-0,010536	0,967811	0,6279808
CG30110-RA	Shmt	41,0771	32,7342	64,5688	50,3114	57,9008	57,0303	0,01562	0,897983	0,6279808
CG30114-RA	Shmt	15,0353	12,7048	21,4972	21,6927	23,5551	23,8413	NA	NA	0,6279808
CG30115-RE	CG30110	0,0519465	1,42848	0	0	0	0	0,187181	0,479114	0,6279808
CG30116-RA	CG30114	0	0	0	0,145289	0,118918	0,0896241	-0,005096	0,981566	0,6279808
CG30116-RB	GEFmeso	13,727	35,4543	4,28985	37,6234	0,0919616	31,3439	-0,095746	0,66124	0,6279808
CG30116-RC	CG30116	0,0103573	1,11322	0,00994359	10,7858	7,02499	0,010796	-0,10973	0,613626	0,13772387
CG30116-RD	CG30116	2,13176	1,39998	2,28413	0,0118223	1,66538	1,75172	-0,164164	0,475417	0,6279808
CG30118-RA	CG30116	0,0116133	0,0105782	0,0111495	0,0118074	0,0143248	0,0121405	0,450988	0,0246	0,6279808
CG30118-RB	CG30116	2,63661	3,22928	2,60758	0,0118138	2,47148	1,83607	0,525753	0,010193	0,6279808
CG30118-RC	Ttd14	18,5848	17,3673	35,0343	15,5906	1,78883	14,8013	0,52832	0,009654	0,13772387
CG3011-RA	Ttd14	5,05726	4,1148	0,0272768	2,9402	10,2978	0,0309047	-0,435831	0,091368	0,6279808
CG3011-RB	Ttd14	3,95931	4,36217	0,0279498	3,74617	10,2528	4,04936	-0,423852	0,101696	0,6279808
CG30120-RB	CG30120	0,0268856	0,040869	35,2717	26,8359	43,6177	25,065	-0,339199	0,112359	0,6279808
CG30121-RA	lncRNA:CR30121	0,0734285	11,2714	0,146981	31,1045	0,0387541	0,0243843	0,001611	0,995273	0,6279808
CG30122-RB	CG30122	18,426	0,0241325	0,0270097	9,45851	11,3272	12,0246	-0,094537	0,721257	0,6279808
CG30125-RA	Ir56a	0,109397	0	0	0,061408	15,1799	0	-0,026072	0,90826	0,13772387
CG30126-RA	5-HT1B	0,446459	0	0	2,3886	4,8117	0	-0,247776	0,318239	0,6279808
CG30127-RA	Rgk1	0,864434	2,27534	20,6458	4,20495	0,0298545	5,87176	-0,523871	0,070288	0,6279808
CG30128-RA	Obp56c	1,35111	0,104915	0,0257385	5,11398	0,0385445	1,82817	-0,408772	0,245145	0,6279808
CG30128-RB	Obp56c	0,806269	0	9,2626	0,0448084	4,68645	2,08181	-0,396146	0,259761	0,6279808
CG30129-RA	Obp56b	7,02256	7,2689	40,1382	0,0193883	0	2,07079	-0,030822	0,925607	0,6279808
CG30141-RA	CG3014	4,06817	0,051237	4,09835	1,89256	1,57583	0,0624556	0,089672	0,732111	0,6279808
CG30142-RA	CG3014	0,0189837	9,26205	0,0182255	2,87434	3,62861	2,34789	-0,176601	0,613916	0,6279808
CG30145-RA	Obp57a	0	2,07011	0,0159522	0,0183108	2,87339	0,0136814	-0,10111	0,776782	0,13772387
CG30147-RA	Obp57b	0,731464	8,03861	0,016927	3,35448	4,12989	51,155	0,273735	0,281019	0,13772387
CG30147-RB	Obp57e	1,68807	0,0577605	97,4876	0,0862998	0	0,0233597	0,274028	0,280413	0,6279808
CG30148-RA	Hil	16,9531	23,4041	15,8169	0,0367387	1,09384	1,32799	0,726427	0,042373	0,6279808
CG30149-RB	Hil	5,17418	3,50987	3,68988	13,4811	1,43847	1,06208	0,148616	0,558091	0,6279808
CG3014-RA	CG30148	1,82418	74,1501	0,0449693	47,8681	56,9215	8,4734	0,458716	0,113313	0,6279808
CG3014-RB	rig	1,58467	0,0289962	2,40535	11,936	1,09539	0,0349151	0,458573	0,110427	0,6279808
CG30150-RB	Obp57d	0,0468401	0,0368413	0,0388309	0,0172152	2,00201	53,8872	-0,387829	0,197579	0,6279808
CG30151-RA	CG30151	1,95959	0,0160597	61,7448	0	2,33211	1,41759	-0,151921	0,656812	0,6279808
CG30152-RA	Dpse\GA15681	59,8834	0,252649	61,6765	1,91403	6,43082	0,309508	0,522811	0,031255	0,6279808
CG30154-RA	CG30154	17,9903	0,498439	4,04916	1,94173	0,0181532	1,75762	-0,099945	0,74813	0,6279808
CG30156-RA	CG30156	0,881012	4,26191	1,29099	16,1237	1,45679	0	-0,392691	0,202636	0,6279808
CG30157-RA	CG30157	2,76488	6,88175	0,0440941	7,6119	0,0692123	0,0521626	-0,274147	0,40312	0,6279808
CG30158-RA	CG30158	2,8481	3,599	21,8293	2,82363	2,87328	0,0256001	0,110852	0,647865	0,6279808
CG30159-RA	CG30159	0,0439139	0,0399998	0,04216	0,0486099	0,0658424	0,0496228	-0,114829	0,598216	0,6279808
CG30159-RB	CG30159	0,0533012	32,6016	38,1746	30,7459	0,0818423	0,0616813	0,025133	0,90817	0,13772387
CG30160-RA	Usp30	8,61166	0,0195432	4,75198	2434,58	10,1456	9,88521	-0,639893	0,053727	0,6279808
CG30161-RA	CG30160	26,8156	20,512	41,2956	37,1624	77,057	57,0303	-0,696192	0,044837	0,6279808
CG30163-RA	CG30161	0,238829	0,326312	82,2379	49,053	0,115532	0,0870717	-0,914549	0,003989	0,6279808
CG30169-RA	Cpr60D	21,6272	13,5963	629,437	0,717007	0,243508	0,183522	0,161509	0,513345	0,13772387
CG3016-RA	Brca2	1,77188	0,00939288	0	2,05348	5,09656	0	-0,281393	0,141413	0,6279808
CG30170-RA	Alas	33,3676	13,8157	12,5063	77,4181	248,416	15,7896	0,031574	0,925788	0,6279808
CG30172-RA	bgcn	0,24649	36,7022	44,0112	0,322799	22,7873	31,6355	-0,164768	0,577649	0,6279808
CG30173-RA	CG30172	153,314	139,996	109,991	0,0169757	0,0464479	243,472	-0,491938	0,061427	0,6279808
CG30176-RA	HSPC300	78,4537	58,943	220,756	10,9058	11,4127	207,366	-0,103066	0,698105	0,6279808
CG30177-RA	Pym	6,76842	11,2493	43,846	8,75945	33,2794	37,946	0,01562	0,897983	0,6279808
CG30178-RA	CG30177	0	0	15,3319	0	0	0	-0,624946	0,080673	0,6279808
CG3017-RA	CG30178	0,415572	30,7577	0,448845	1,73401	1,12191	1,66667	0,414572	0,142316	0,6279808
CG30181-RA	lwr	62,6688	68,7143	94,7604	80,2569	2,07628	68,5413	0,367839	0,284492	0,13772387
CG30182-RA	lwr	0,0535171	0,0487471	0,0513796	0,0607005	0,992445	0,0619654	0,265373	0,455742	0,13772387
CG30183-RA	ppk3	0,780747	1,36617	1,02572	1,07285	0,830469	0,594522	-0,026986	0,928969	0,6279808
CG30183-RB	CG42560	0,0837816	0,0207277	0,0255794	0,103033	0,0391876	0,0798868	-0,076394	0,799761	0,6279808
CG30184-RA	CG30183	1,85698	0,0562491	0,0592867	0,0370903	32,7972	0,0378632	0,539134	0,114966	0,6279808
CG30185-RA	CG30183	0,445546	133,675	157,087	14,313	0,100789	12,2683	-0,32836	0,300643	0,6279808
CG30186-RA	CG30184	0,393309	5,74473	5,68405	7,21536	0,293189	27,1979	-0,070526	0,562659	0,6279808
CG30187-RA	AIMP3	23,6881	25,573	32,9439	27,0589	27,9061	27,5114	0,062331	0,823807	0,6279808
CG30189-RA	Gr59c	0	4,01243	0	2,12419	0	0	-0,142667	0,572154	0,6279808
CG3018-RA	CG30187	2,26828	2,4928	2,24869	2,32778	2,65188	2,27355	0,136196	0,52177	0,6279808
CG3018-RB	Gr59a	0,089777	0,031418	0,034352	0	0,0505234	0	0,136196	0,52177	0,6279808
CG30190-RA	su(w[a])	3,18319	2,81141	2,47268	5,01257	3,50732	2,59786	0,321953	0,138031	0,6279808
CG30190-RB	su(w[a])	0,0164997	0,0150291	0,0158407	2,16419	0,0231488	3,11699	0,32621	0,126491	0,6279808
CG30191-RA	su(w[a])	10,5661	11,2013	11,8768	13,5766	8,34139	8,41586	0,044335	0,715924	0,13772387
CG30192-RA	ReepA	17,8948	4,64554	0,0604736	13,9718	6,28737	6,61126	NA	NA	0,6279808
CG30193-RA	ReepA	42,9969	0,0815904	14,3769	7,96325	95,0161	0,0498434	-0,176437	0,366831	0,6279808
CG30193-RB	Gr59b	0,0900216	10,4194	0,0331147	0	0,0711433	0,180278	-0,186859	0,338013	0,6279808
CG30193-RC	CG30192	0	0,0850092	0,0282989	476,796	0	0	-0,176992	0,369289	0,6279808
CG30193-RD	ReepA	0,0425747	0	8,95451	15,0185	0,047085	8,02345	-0,107872	0,582342	0,6279808
CG30193-RE	ReepA	0,0444357	6,18616	17,6396	9,14025	1,16149	1,29796	-0,186859	0,338013	0,6279808
CG30193-RF	ReepA	0,0479158	3,12404	42,3565	18,0622	0,820799	1,20567	-0,186859	0,338013	0,6279808
CG30193-RG	ReepA	0,0444058	0	0,0507629	47,1187	9,884	5,62359	0,147846	0,467367	0,6279808
CG30194-RB	ReepA	0,0453818	155,341	0	0,0598732	5,07952	0,0354861	0,233418	0,239097	0,6279808
CG30194-RD	ReepA	16,331	0,0294358	0	0	7,05065	1,99581	0,214715	0,276728	0,6279808
CG30194-RF	ReepA	6,95314	3,17956	0,200073	0	0,0665877	0,158452	-0,13384	0,637523	0,6279808
CG30195-RA	CG44253	11,7019	10,6706	0,1888	0,488813	9,01249	9,85556	-0,773912	0,014348	0,6279808
CG30196-RA	CG44253	0,196654	0,179126	0,0258749	0,0286172	0,662101	0,200193	-0,551755	0,087847	0,6279808
CG30197-RA	CG44253	0,0269514	4,50478	20,1036	15,2891	2,17134	0,0292135	-1,145643	0,000492	0,6279808

gene_id	Symbol	W1_FPKM	W2_FPKM	W3_FPKM	MCM51_FPKM	MCM52_FPKM	MCM53_FPKM	FC	p-value	p-adj
CG3019-RA	CG30195	29,5845	19,0835	62,2431	529,91	1218,42	1016,14	-0,220223	0,258069	0,6279808
CG3019-RB	CG30196	22,0693	8,86003	11,199	50,4836	148,979	68,3779	-0,260162	0,185523	0,6279808
CG3019-RC	CG30197	52,5854	1,80684	62,1152	5,37539	11,1337	2,57804	-0,277413	0,155034	0,6279808
CG30203-RB	CG30203	1,64157	0,0625494	1,7012	0	2,15799	1,7156	-0,597409	0,05922	0,6279808
CG30217-RA	CG3021	6,71543	0,917957	9,15316	90,4079	0,0443037	0,0333899	0,250344	0,310959	0,6279808
CG30217-RB	CG43326	18,8319	10,8461	9,85625	10,5297	64,9914	17,7353	0,257642	0,304584	0,6279808
CG3021-RA	CG43326	5,26672	3,26214	3,8681	3,3561	17,939	90,156	-0,274948	0,333569	0,6279808
CG30222-RE	GABA-B-R3	0,628693	2,20177	46,9112	1,43581	2,53311	0,33515	0,01562	0,897983	0,6279808
CG30222-RF	GABA-B-R3	1,92096	3,37238	0,399872	1,40827	4,1599	0,776362	0,01562	0,897983	0,6279808
CG3022-RA	CG30222	0,0413318	0	0	3,89048	4,4743	2,16591	0,43765	0,082662	0,6279808
CG3022-RB	CG30222	0,107359	0	0	0	8,88105	0	0,515586	0,04132	0,6279808
CG3024-RA	Torsin	12,2323	13,1896	20,5445	17,4729	21,5009	20,5528	-0,357113	0,195133	0,6279808
CG30259-RA	mof	2,96761	3,48112	9,68394	12,6031	2,82149	9,10849	-0,324705	0,361724	0,6279808
CG30259-RB	CG30259	0,177836	0,0929568	0,235144	0,399777	0,113475	6,59914	0,040365	0,880829	0,6279808
CG3025-RA	CG30259	0,111993	0,504667	0,118205	0,170034	0,0140452	0,177594	0,159467	0,550029	0,6279808
CG30263-RB	mus81	0,0311727	0,174622	6,52581	0,00950472	0	4,6389	-0,067964	0,835425	0,6279808
CG30265-RA	stum	0,192914	0,0587375	0,159389	8,94665	0,0406513	31,215	-0,115206	0,733413	0,6279808
CG30268-RB	CG30265	2,35853	0,471547	3,42323	5,59393	23,4437	19,8609	-0,09751	0,554745	0,6279808
CG30269-RA	CG30268	0,0175687	16,1526	0,042774	0,0518224	5883,88	6072,47	-0,156241	0,506591	0,6279808
CG3026-RA	CG30269	34,9381	16,3289	13,8968	2213,56	7,91043	2707,83	0,57563	0,025422	0,13772387
CG30270-RC	pyd3	36,3953	30,9575	5,1015	9,0163	11,9761	13,0338	NA	NA	0,6279808
CG30270-RD	CG30270	0	0	0	0	0,0232816	0,0175465	NA	NA	0,6279808
CG30271-RB	CG30270	0	0	0	0	0	0	-0,645741	0,05955	0,6279808
CG30271-RC	CG30271	0,392789	0,0298149	0,220423	0,427285	0,661282	0,314011	-0,613669	0,074469	0,6279808
CG30272-RA	CG30271	0,0353221	0,482607	0,831281	1,84692	1,09802	0,380806	-1,220222	0,000382	0,6279808
CG30273-RA	MFS1	4,75446	1,85313	0,345676	0,389528	0,172498	0,926509	-0,66888	0,001861	0,6279808
CG30274-RA	CG30273	9,55685	7,59751	56,9637	465,66	55,7158	64,0932	-0,564315	0,078241	0,6279808
CG30275-RF	CG30274	0,939245	0,898306	1,82601	0,726383	0,0653124	4,59445	-0,013096	0,914398	0,6279808
CG30275-RG	CG30275	0,0161371	0	0,0152933	0,0167004	0	0,0165188	-0,013096	0,914398	0,6279808
CG30275-RH	CG30275	0,0164422	0	0,0155788	0,0170284	0	0,016185	-0,013096	0,914398	0,13772387
CG30275-RI	CG30275	0,0161056	0	0,0152639	0,0166666	0	0,0163621	-0,013096	0,914398	0,6279808
CG30275-RJ	CG30275	0,0162842	0	0,015431	0,0168585	0	0,0165922	-0,013096	0,914398	0,6279808
CG30277-RA	CG30275	0,0247743	2,0128	0,015648	0,0245217	0	0,0361833	-0,363082	0,310127	0,6279808
CG30278-RA	Oatp58Da	0,489178	0,0526106	0,0175304	10,8731	0,0257242	0	-0,052197	0,843825	0,6279808
CG30279-RA	CG30278	0,0710493	0,053231	0,0169406	0,0231238	0,0248227	0	-0,241703	0,268755	0,6279808
CG3027-RA	CG11170	0	0,508823	0	9,19453	17,2886	14,4434	-0,295222	0,34444	0,6279808
CG30280-RA	Ipp	17,9873	0,320743	1,77198	111,256	15,5402	20,489	0,09719	0,785934	0,6279808
CG30281-RA	CG30280	0,586655	0,0301888	6,04381	0,0818001	4,44519	6,08181	-0,450494	0,200083	0,6279808
CG30283-RA	CG30281	0,0655515	0,0178913	15,5277	0,018326	4,35711	5,80705	0,524179	0,052189	0,13772387
CG30284-RA	CG30283	7,13365	0	6,42785	16,2883	19,9698	8898,35	0,221605	0,516438	0,13772387
CG30284-RB	CG30284	0,451396	148,635	0	15,9235	11141,2	21,1008	0,22435	0,513014	0,6279808
CG30285-RA	CG30284	0,559631	7,82756	16,1358	0,527834	0,0954021	0,157706	-1,414557	9,75E-06	0,6279808
CG30286-RA	CG30285	2,33844	0,0454695	0,047925	0,0561009	0,075989	0,0572699	0,084347	0,713134	0,6279808
CG30287-RA	CG30286	0	9,76267	0,129993	0	7,84312	5,30024	-0,080745	0,73969	0,6279808
CG30288-RC	CG30287	0,115923	8,35074	0,185488	5,27508	8,03121	0,509838	-0,092733	0,775087	0,6279808
CG30289-RA	CG30288	2,10135	0,29903	0,487094	287,35	0	194,056	-0,593302	0,097379	0,6279808
CG3028-RA	CG30289	0,32829	2,02039	1,79327	0,0600368	0	0,0612879	0,015872	0,958211	0,6279808
CG30290-RA	or	14,9256	11,1339	20,9132	0,0400918	48,7294	11,6986	-0,115831	0,682518	0,6279808
CG30291-RA	Ppcdc	19,7834	0,0492122	220,704	0,0645667	0,087456	0,0659121	-0,022629	0,92994	0,6279808
CG30293-RA	CG30291	20,1787	16,9593	32,5248	23,5463	18,3623	9,46417	0,093957	0,582148	0,6279808
CG30295-RA	Cht12	0,0406621	0,540348	0,30667	0,0463204	8,4767	30,4148	0,347361	0,243777	0,6279808
CG30295-RB	Ipk1	0,0274212	8,96296	0,0263259	1,44215	0,0394824	1,65542	0,350886	0,211419	0,6279808
CG30296-RC	Ipk1	1,33465	0	1,31866	0	0,698663	6,89247	-0,290224	0,234227	0,6279808
CG30296-RD	RIC-3	0,0283142	4,0749	3,81925	0,0285924	1,52332	0,0301295	-0,284602	0,243349	0,6279808
CG30296-RE	RIC-3	4,49445	3,09566	0,0266354	5,96568	193,697	0,0291882	-0,283839	0,243851	0,6279808
CG30296-RF	RIC-3	0,0269294	0,0245292	0,0258539	19,3353	26,3358	7,21988	-0,127838	0,605221	0,6279808
CG30296-RG	RIC-3	3,35256	0,0258792	7,84208	1,27917	0,254836	22,4847	-0,2892	0,235809	0,6279808
CG3029-RA	RIC-3	28,3745	19,2943	23,7742	15,6408	0,0549235	23,3801	0,162313	0,478008	0,6279808
CG30321-RA	CG3032	0,926081	1,58164	0,245667	0,0982694	4,19943	3,30598	0,043378	0,833002	0,6279808
CG30323-RB	CG30321	0	0	0,356386	0,366852	2,4653	1,858	0,093152	0,732893	0,13772387
CG30324-RA	CG30323	0,179359	10,4229	6,28338	0,0640645	22,243	5,03472	0,264047	0,452094	0,13772387
CG30325-RA	CG30324	0,460281	0,920283	29,0667	33,7947	0,0497628	0,0509581	0,01562	0,897983	0,6279808
CG30329-RA	CG30325	0	10,7737	0	1,51653	22,1036	0	0,022862	0,858938	0,6279808
CG3032-RA	Vha100-3	0,0608434	0,0646393	0	0,039338	0,161797	0,350489	0,243749	0,465333	0,6279808
CG30330-RA	GAA1	6,9053	216,53	73,7226	10,0761	0,0499858	2,26644	-0,020163	0,931827	0,6279808
CG30334-RA	Gr59d	0,253488	0	0,0811212	0	0	0,377692	-0,635893	0,040582	0,6279808
CG30338-RA	CG30334	4,559	0,0215131	36,6798	0,0386797	5,688	0,761135	-0,27473	0,363952	0,6279808
CG30339-RA	CG30338	0,0655515	0,0597088	0,0629333	44,4146	13,036	38,2802	-0,333631	0,350069	0,6279808
CG3033-RA	CG30339	8,67202	2,98548	21,1747	0	0,382605	15,5604	-0,3531	0,088057	0,6279808
CG30340-RA	MED22	14,1122	0,0485113	0,0315815	0,0603669	14,3973	1,04655	-0,450241	0,200189	0,6279808
CG30340-RB	CG30340	0,0289595	0,356107	0,0278028	0,98434	1,65556	0,0680458	-0,346576	0,330087	0,6279808
CG30342-RA	CG30340	0,0554793	0,0505344	0,0532635	5,86082	5,34863	19,1161	-0,109567	0,679428	0,13772387
CG30343-RA	Prp38	7,78178	0,0254309	0,030184	0,0219381	7,49549	0,0223953	0,394758	0,141787	0,13772387
CG30344-RA	CG30343	32,8796	33,5544	20,166	24,681	16,2159	21,5383	-0,076101	0,725798	0,6279808
CG30345-RA	CG30344	17,4367	21,0374	20,3526	2,26388	60,8209	64,2197	-0,494416	0,120753	0,6279808
CG30349-RA	CG30345	1,69083	1,03351	2,5631	24,6598	13,9571	20,0785	-0,044417	0,877018	0,6279808
CG3034-RA	CG30349	14,4172	10,6792	28,1556	0,809652	13,9554	0,57614	0,046716	0,872873	0,6279808
CG30350-RA	cm	15,7183	10,876	5,85656	13,6745	60,845	77,4767	-0,011246	0,943435	0,6279808
CG30354-RA	CG30350	0	0,0132404	0,0693046	0,0222643	0,0301571	0,0227282	0,521056	0,139902	0,13772387
CG30355-RA	UQCR-11L	0,731853	0,00381732	11,9611	0,00421909	2,48826	0,004307	-0,301203	0,399997	0,13772387
CG30356-RA	CG30355	0,789752	0,00382752	0,046147	0,00423046	1,53645	0,00431862	0,860987	0,00139	0,13772387
CG30357-RA	CG30356	0,121687	6,29077	0,116827	0,0227015	0,0307494	0,0231746	-0,383188	0,262343	0,13772387
CG30359-RA	CG30357	0,538662	1,02219	0,581791	1,90731	0,843632	0,831346	-0,250336	0,396642	0,13772387
CG3035-RA	Mal-A5	6,94335	5,15163	10,241	65,714	3,43274	14,6253	0,293784	0,221173	0,6279808
CG30360-RC	CG3036	12,6193	8,92428	3,15663	3,10599	0,042089	4,72171	-0,458009	0,147668	0,6279808
CG30361-RC	Mal-A6	2,60086	0,480211	27,2885	13,3308	29,3551	9,8337	0,086302	0,736201	0,6279808
CG30361-RD	mtt	2,89783	3,71855	0,394762	25,8625	37,5384	1,76388	0,000847	0,997361	0,6279808
CG30361-RE	mtt	0,74113	0,842233	0,00987548	8,13989	2,74543	0,39199	0,005208	0,983773	0,6279808
CG30361-RF	mtt	0,0120937	0,0110158	3,72107	168,618	0,0196955	0,3592	0,052407	0,837998	0,6279808
CG30362-RA	mtt	0,0102863	0,00936949	5,57168	2,30746	0,683594	0,0107203	0,013049	0,916306	0,6279808
CG30363-RA	boly	0	0	0	0,10867	28,0413	0	0,063548	0,603667	0,6279808
CG30364-RA	cola	0	0	0,206769	0,0490996	0	0	NA	NA	0,6279808
CG30365-RA	hubl	0	0	0,741951	16,4478	0,0312118	0	0,059792	0,706172	0,6279808
CG30366-RA	spaw	0	0,140113	0,028971	0	0	0,243839	0,044335	0,715924	0,13772387
CG30369-RA	swif	0	0	0,142619	0,0622548	0	0	NA	NA	0,13772387
CG3036-RA	CG30369	0	246,625	0	617,495	0	0,427415	-0,135317	0,560754	0,6279808
CG30371-RA	CG30371	15,8307	45,003	0	0,0920234	0	0,0235231	-0,41675	0,167023	0,6279808
CG30372-RB	Asap	9,46919	13,3446	0,0140513	14,2711	6,50441	6,77914	0,101858	0,70164	0,6279808
CG30373-RA	CG30373	28,8705	0,0430211	23,0367	5,55685	16,3416	0,0490797	-0,531961	0,089949	0,6279808
CG30374-RA	CR30374	0,186655	0	0,3584	0	0	0	0,130803	0,471701	0,6279808
CG30375-RA	CG30375	0,303607	0,45253	0,317979	0	0	0,507265	-0,076847	0,828226	0,13772387
CG30376-RA	CG30376	0	0	393,866	0	0	347,236	NA	NA	0,6279808
CG30377-RA	CG30377	9,64658	0,00781372	0,00824532	0,00873371	0,0118299	0,0089692	0,566291	0,022018	0,6279808
CG30378-RA	CG30378	0,235424	31,7441	29,0841	0,021369	6,70413	21,5162	0,061982	0,793734	0,6279808

gene_id	Symbol	W1_FPKM	W2_FPKM	W3_FPKM	MCM51_FPKM	MCM52_FPKM	MCM53_FPKM	FC	p-value	p-adj
CG30379-RA	CG30379	0,20023	0,182383	0,192233	26,4885	0,0128965	21,9922	0,219719	0,39684	0,6279808
CG30380-RA	Dsim\GD16488	12,6148	7,89518	11,1398	16,6872	8,76414	3,47988	0,256092	0,439178	0,6279808
CG30381-RA	Dsim\GD16488	3,10714	5,02935	5,90562	0,0462553	4,37751	13,541	0,320066	0,285074	0,6279808
CG30381-RB	CG30380	6,39687	4,38717	7,87541	5,94453	414,934	0,12824	0,317162	0,274959	0,6279808
CG30382-RA	PIG-X	3,48197	0,0610616	1,86642	0,125623	0,0245699	0	0,337987	0,320803	0,6279808
CG30383-RA	PIG-X	0,0670366	20,1575	28,0848	1,43209	0,0267176	7,99296	-0,260897	0,384141	0,13772387
CG30384-RA	CG30382	21,4039	13,1127	0,00823569	0,00872331	0,0118158	0,00891925	0,616056	0,084034	0,13772387
CG30385-RA	CG30383	2,12045	2,39874	0,00824854	0,00873719	0,0118346	0,00890508	0,760795	0,033455	0,6279808
CG30386-RA	CG30384	0,219894	0,654297	0,520742	161,534	85,4592	136,901	-0,172788	0,629081	0,13772387
CG30387-RA	CG30385	0,482447	0,474601	0,537286	23,559	3,01159	5,99239	-0,324401	0,106019	0,13772387
CG30387-RB	CG42672	0,985342	11,5645	0,0137577	4,6078	3,4225	2,41429	-0,323493	0,10736	0,6279808
CG30387-RC	CG42672	0,0132258	0,0125024	0,01259	4,48341	5,93735	0,0141297	-0,343557	0,086615	0,6279808
CG30387-RD	CG42672	1,72744	0,0110232	0,0131484	11,4431	8,41793	9,51742	-0,53576	0,010624	0,6279808
CG30387-RE	CG42672	0,0137258	0,0122355	0,185037	13,224	3,13768	0,0143192	-0,32422	0,105989	0,6279808
CG30387-RF	CG42672	0,0121018	1,0004	0,0126975	0,0138413	0,0187481	0,0137569	-0,554857	0,008067	0,6279808
CG30387-RG	CG42672	0,0134328	0,0130528	2,17003	10,4644	5,73529	0,0143866	-0,531316	0,011405	0,6279808
CG30387-RH	CG42672	1,60292	3,48617	0,0131776	4,20347	3,99353	8,19637	-0,535986	0,010651	0,6279808
CG30388-RA	CG42672	0,0143301	3,73108	0,0116185	0,0134761	0,0182535	3,26846	0,061706	0,799878	0,6279808
CG30389-RA	CG42672	3,97813	0,686337	0,0128963	0,0140929	0,0190889	7,13971	0,124575	0,616439	0,6279808
CG30389-RB	Magi	5,9272	6,62687	23,0405	9,36048	0,0234591	24,3138	0,22418	0,369254	0,6279808
CG30389-RC	CG30389	8,70436	9,79274	9,23262	11,1793	2,75801	4,75074	0,12389	0,61785	0,6279808
CG3038-RA	CG30389	0,0167126	0,0152229	3,23308	23,3274	8,79906	1,04217	-0,345047	0,157659	0,6279808
CG3038-RB	CG30389	3,66194	4,15428	0,0159804	202,446	28,5237	2,65406	-0,336556	0,170166	0,6279808
CG30390-RA	CG3909	19,1869	32,5177	26,3741	31,382	18,5127	11,0484	0,146475	0,547703	0,6279808
CG30391-RA	CG3909	39,6486	35,96	34,5534	75,0096	73,6634	27,7891	0,287021	0,173711	0,13772387
CG30392-RA	Sgf29	7,12977	0,119734	18,5701	12,0682	0,0816177	0,0615121	-0,202712	0,435738	0,6279808
CG30393-RA	CG30391	0,109542	0,0240778	0,0510488	5,73802	0,0379706	0,0286169	0,186763	0,446138	0,6279808
CG30394-RA	CG30392	37,4507	0,190995	0,491196	4,22565	0,256576	0,158717	0,127697	0,587954	0,6279808
CG30394-RB	CG30393	0	14,0168	0,105167	0,0280328	7,01439	8,60534	0,129234	0,584488	0,6279808
CG30395-RB	CG30394	0,0200961	4,61283	0,0192934	0,977023	1,49247	1,37267	0,73733	0,017628	0,6279808
CG30396-RA	CG30394	0,0207005	0,0188554	0,0198737	0,0213551	0,0201354	0,0218001	0,056958	0,701834	0,6279808
CG30398-RA	CG30395	0,139892	0,247342	0	14,5551	0,0682595	5434,89	-0,550606	0,122211	0,6279808
CG3039-RA	Gr58a	0	14,0975	18,5665	0	0,570867	0	0,109757	0,714506	0,6279808
CG3039-RB	CG30398	0,622666	0,0413179	0,100729	38,5079	0,149998	0,0453107	0,144093	0,631031	0,6279808
CG30401-RA	CG3040	20,1814	4,661	5,26974	0,0165411	0,0168572	0,0126306	0,319871	0,370664	0,6279808
CG30403-RB	dany	0,957622	0,123841	0,536301	0,11524	8,97257	7,77934	0,608662	0,012641	0,6279808
CG30404-RA	CG30403	5,07534	6,46486	10,6303	264,294	292,562	7,07969	-0,002139	0,991522	0,13772387
CG30404-RB	Tango11	0,0404463	0,366991	418,899	1,38454	0	0,0383417	-0,005363	0,978735	0,13772387
CG30409-RA	Tango11	28,7547	162,378	0	0,0443858	0	0	-0,020883	0,945934	0,13772387
CG3040-RA	CR30409	0,0941252	0	6,67054	7,15588	0,127138	7,59721	0,07791	0,730791	0,13772387
CG30410-RA	Orc2	1,20809	1,45193	26,7624	56,3655	44,0547	33,218	-0,450707	0,103553	0,13772387
CG30411-RA	Rpi	7,74888	6,04396	0,0903658	2,14468	14,2364	1,25586	0,318075	0,263814	0,13772387
CG30412-RA	CG30411	52,442	15,7204	2,2165	14,4236	0	46,4294	NA	NA	0,13772387
CG30412-RB	CG30412	0	0	0	0,0601115	0	0,0380956	NA	NA	0,6279808
CG30413-RA	CG30412	0	0,031666	0,0556268	16,399	5,20615	6,3791	-0,031505	0,898572	0,6279808
CG30414-RA	CG30413	1037,61	1120,55	1,7058	0,0152756	2278,13	2170,76	0,017222	0,955083	0,6279808
CG30415-RA	CG30414	1,0082	1,10672	1,46431	1931,74	0,230018	0,0321453	-0,108009	0,7113	0,13772387
CG30415-RB	roh	0,10838	0,0791927	0,139116	0,143081	17,8405	0	-0,108009	0,7113	0,6279808
CG30416-RA	roh	337,478	0,09872	5,03187	359,869	0,17796	0	NA	NA	0,13772387
CG30417-RA	CG30416	0	0,17079	0,324024	0	0	0	0,012562	0,923862	0,6279808
CG30418-RA	tbrd-3	0	0	0	0	0	0	0,352225	0,144204	0,6279808
CG30419-RA	nord	3,38851	3,83667	3,34353	0,235225	0	0,300888	-0,206401	0,465592	0,13772387
CG3041-RA	CG30419	2,09169	2,66247	0,0532336	0,237514	0	0,0645212	-0,493923	0,066485	0,6279808
CG30420-RA	Atf-2	3,25626	1,84073	5,043	1,71448	0,0290575	2,86412	-0,047481	0,821195	0,6279808
CG30420-RB	Atf-2	1,3951	1,64665	1,23105	3,13946	3,60728	1,03166	-0,285427	0,235858	0,6279808
CG30420-RC	Atf-2	1,80777	3,55697	2,22973	5,6841	2,13406	1,8079	0,440634	0,069076	0,6279808
CG30421-RA	Dpse\GA15834	9,54543	23,6538	0,0330572	56,5155	0,0406883	0,0306652	-0,044983	0,864257	0,6279808
CG30423-RC	CG30423	118,43	14,2435	122,009	16,5151	27,9852	195,249	-0,354544	0,148314	0,6279808
CG30424-RB	CG30424	6,82981	2,70767	7,9621	5,75235	12,0599	10,1821	-0,38739	0,212909	0,6279808
CG30424-RC	CG30424	0,0434805	0,0441034	0,0417438	0,839983	0,0651217	1,06951	-0,390591	0,212586	0,6279808
CG30424-RD	CG30424	1,79922	308,812	2,89145	2,08347	4,20553	0,0967113	-0,409739	0,186726	0,6279808
CG30425-RA	RpL41	18016,4	0	0,3304	0,0647571	1,39462	51,2039	-0,566971	0,054226	0,6279808
CG30427-RA	CG30427	36,4156	28,7246	23,4803	20,877	4,09937	18,3795	0,08335	0,714918	0,6279808
CG30427-RB	CG30427	63,9339	58,0617	56,1265	29,298	2,41923	46,3502	0,318075	0,154014	0,6279808
CG30427-RC	CG30427	0,0424197	0,0386388	0,0407255	28,7562	16,5913	0,0477556	0,306391	0,19772	0,6279808
CG30427-RD	CG30427	0,041306	0,0376243	0,0396562	23,0323	31,2926	0,0463728	0,088966	0,69696	0,6279808
CG30428-RA	CG30428	9,97807	0	6,64904	7,73216	0,588405	0,714318	-0,044189	0,864128	0,6279808
CG30429-RA	CG30429	0,0933275	0	18,0748	0	7,97638	1,25222	0,139669	0,390249	0,6279808
CG30430-RA	CG30430	0,388682	4,63254	0,00994827	0,444158	0,0141859	0,0148536	-0,043124	0,847628	0,6279808
CG30431-RA	CG30431	2,59193	1,33289	28,0218	30,3276	6,77206	0,186322	0,043348	0,880046	0,6279808
CG30432-RA	CG30432	0,222029	0	0,0182098	0,0197605	0,623136	16,0356	-0,026405	0,904113	0,6279808
CG30438-RA	Ugt50B3	0,0284728	0,025935	0,0273356	0,0303436	103,446	82,6675	0,681624	0,015818	0,6279808
CG30438-RB	Ugt50B3	0,0277785	0,0253026	0,026669	0,0295542	208,554	171,179	0,6807	0,015849	0,6279808
CG30438-RC	Ugt50B3	10,292	8,85005	6,27476	10,6592	0,182323	0,137409	0,64393	0,023353	0,6279808
CG30438-RD	Ugt50B3	5,21806	7,75203	5,64467	3,26491	75,2392	32,6207	0,681474	0,015851	0,6279808
CG30440-RA	Cht11	3,37645	9,94173	0,426323	9,1944	0	0	0,146595	0,594125	0,6279808
CG30441-RB	CG30440	13,7924	11,1036	13,7141	19,104	0,0263858	13869,8	-0,069953	0,811395	0,6279808
CG30443-RA	IFT20	7,13245	7,67795	0,177859	13,6056	3,16191	0,019886	0,012592	0,951843	0,13772387
CG30445-RA	Opbp	5,839	6,37209	7,55797	12,9273	5,27184	6,07257	-0,241538	0,391532	0,6279808
CG30446-RA	Tdc1	10,9064	10,0663	14,0306	9,78542	11,8799	12,3718	-0,398173	0,097778	0,6279808
CG30447-RA	Tdc2	3,16934	3,8783	3,56525	4,82561	5,5592	4,09097	-0,013096	0,914398	0,6279808
CG30447-RB	CG30447	0	35,3144	28,0948	35,6273	19,2683	24,1794	-0,013096	0,914398	0,6279808
CG30448-RA	CG30447	0	0,0190477	0,0133842	0,0143539	0,0194424	0,014653	-0,095866	0,662639	0,6279808
CG3044-RA	Obp56i	0	0,0126851	0,0133701	0,0143382	0,0194212	0,014637	0,010028	0,969027	0,6279808
CG30450-RA	CG3045	0,039933	0,0364398	6,30283	15,1303	0,029181	3,91019	0,01562	0,897983	0,6279808
CG30456-RB	Obp56f	0	0,0354362	0,129112	0,0274718	0,0697532	0,0525702	-0,651126	0,025172	0,6279808
CG30456-RC	CG30456	4,3057	1,14874	3,44546	0,0463204	0,0278428	0,020984	-0,554678	0,05264	0,6279808
CG30457-RA	CG30456	2,09837	2,25543	0,018556	9,23858	0,0181828	0,0137037	0,056973	0,868953	0,6279808
CG30458-RA	CG30457	432,743	186,43	1122,88	473,995	812,641	736,765	0,635746	0,034796	0,6279808
CG30459-RA	CG30458	260,352	181,359	491,793	631,525	0,204459	234,979	0,061348	0,833629	0,6279808
CG3045-RA	CG43328	0,166159	2,20554	56,2618	9,68286	0,0350068	2,22293	0,540028	0,028839	0,6279808
CG30460-RA	CG30460	0,0130645	0,0119	0	1,87781	4,13037	0,0472856	0,528249	0,050065	0,6279808
CG30460-RB	CG30460	14,6195	18,0686	6,49909	4,19384	15,3768	14,4682	0,534316	0,047686	0,6279808
CG30460-RC	CG30460	0,011198	0,0101999	1,01278	8,06965	0,0574136	14,2322	0,512299	0,058786	0,6279808
CG30460-RD	CG30460	2,59359	2,91593	0,0372354	22,9579	53,0473	4,11537	0,576609	0,03382	0,13772387
CG30460-RE	CG30460	0,0102639	0,00934911	66,3962	0,0423871	41,982	7,72717	0,518648	0,053205	0,13772387
CG30460-RF	CG30460	0,0106049	0,00965972	22,0178	63,8727	0,164362	2,34096	0,512291	0,058768	0,6279808
CG30460-RG	CG30460	0,0111886	0,0101913	36,0103	57,351	219,448	18,7447	0,518027	0,05632	0,6279808
CG30461-RB	CG30461	0,0833588	0,0711424	0,0800294	0,0778861	3,00834	0,0795091	0,456484	0,128805	0,6279808
CG30461-RC	CG30461	0,093902	0,0795066	0,0901515	0,0870432	36,6124	0,0888569	0,456484	0,128805	0,6279808
CG30461-RD	CG30461	0,0858498	0,0731306	0,0824209	0,0800628	0,105497	0,0817311	0,456484	0,128805	0,13772387
CG30461-RE	CG30461	0,0297371	0,0270866	0,0292515	0,0326274	0,108446	0,0302721	0,413841	0,164424	0,6279808
CG30463-RA	Pgant9	4,01645	0,102765	95,2999	1,70281	4,99593	1,16109	0,271966	0,241065	0,6279808
CG30463-RB	Pgant9	9,1033	4153,46	0,285751	10,0915	15,7153	0,00909902	0,271553	0,242055	0,6279808

gene_id	Symbol	W1_FPKM	W2_FPKM	W3_FPKM	MCM51_FPKM	MCM52_FPKM	MCM53_FPKM	FC	p-value	p-adj
CG30463-RC	Pgant9	6,89161	16,2491	4,85211	4,15296	137,761	0,00930675	0,271435	0,242274	0,6279808
CG30463-RD	Pgant9	15,3296	0,0176052	29,2917	17,9953	30,5706	7,31811	0,272084	0,240846	0,6279808
CG30463-RE	Pgant9	0,0576745	5,9373	108,494	138,787	69,0332	0,184024	0,137437	0,542543	0,6279808
CG30464-RB	Ir52d	0	0,0835923	4,97969	1,29552	1,42337	3,07821	-0,041811	0,731452	0,6279808
CG30466-RA	CG30466	2,17465	1,94273	1,74652	2,16566	1,3326	1,17995	0,307547	0,291591	0,6279808
CG30467-RA	CG30467	4,19891	3,0953	6,71242	5,09077	3,31306	9,10026	0,179052	0,503506	0,6279808
CG30468-RA	Ir52c	0,0917721	0,0840124	3,47528	0	4,97131	2,96186	0,018935	0,921072	0,6279808
CG30469-RB	Ir52b	0,0553399	0	10,1678	0,0160809	4,78768	4,27795	0,211974	0,255772	0,6279808
CG30470-RA	Sgs1	0,167238	0,190415	54,7034	15,8626	37,869	0,0763053	-0,260652	0,44773	0,6279808
CG30471-RA	Stacl	0,45886	0,396323	0,0235503	0,436487	0,0350756	0,0264351	0,255879	0,464785	0,6279808
CG30472-RA	CG30471	0,450108	5,27296	0,0466561	0	0,0217816	0,0555668	NA	NA	0,6279808
CG30473-RA	CG30472	0	3,21601	1,42356	0	0,0185841	0,476942	-0,203552	0,55278	0,6279808
CG30475-RA	Obp51a	1,6799	0,595064	0,896	1,04608	18,5196	17,2125	-0,958669	0,001636	0,6279808
CG30476-RA	aPKC	1,3587	0,0296096	1,69348	19,9999	10,464	0,0292771	-0,240427	0,354938	0,6279808
CG30479-RA	ave	26,5647	19,6887	5,89209	39,8991	42,1907	0	-0,564363	0,0967	0,13772387
CG3047-RA	CG30479	26,1274	12,9341	7,11871	6,16695	11,4725	8,12873	0,032119	0,927656	0,6279808
CG30480-RA	Traf4	0,0293323	0,0267179	0,0281607	0,0332196	0,110186	2,65719	-0,830452	0,011454	0,6279808
CG30482-RA	Traf4	19,837	26,7072	19,921	0,0337643	0,100848	2,71662	-0,283037	0,390192	0,6279808
CG30483-RA	Traf4	0,0309827	0,0282212	0,0297453	0,033678	2,18883	0,0319772	0,290374	0,243087	0,6279808
CG30484-RA	CG30480	4,35972	2,2344	0,265982	0,173351	0,0348157	23,659	-0,120081	0,64047	0,6279808
CG30485-RA	CG30482	0,0707451	0,128879	0,0679195	0,337878	0,0685215	0,224547	-0,227786	0,496746	0,6279808
CG30486-RA	Prosap	9,64072	33,6933	7,85816	5,30054	2,83927	191,29	-0,050333	0,715224	0,6279808
CG30486-RB	CG30484	0,0751717	0,0342358	69,5579	0,166338	0,0549907	14,8301	-0,050333	0,715224	0,6279808
CG30487-RA	CG30485	0,0212463	0,0193526	8,62339	0,0226625	5,55038	4,92797	-0,247198	0,485752	0,6279808
CG30488-RA	CG30486	0,212964	0	0,0751916	19,0781	10,9859	22,6223	0,359166	0,22764	0,6279808
CG30489-RA	CG30486	0,0836755	0,264266	0,0772989	26,9761	22,2444	18,1335	-0,558579	0,084239	0,6279808
CG3048-RA	CG30487	1,41313	75,3961	105,898	5,87913	0,0542056	0,0408526	0,07777	0,790179	0,6279808
CG3048-RC	antr	0,217593	0	946,384	0,0655299	5,49775	5,63423	0,10909	0,708592	0,6279808
CG3048-RD	Cyp12d1-p	1,24472	1,05691	1,19189	0,747333	0	0	0,128039	0,660418	0,13772387
CG30490-RA	CG30490	0,0907636	0	0,0871385	51,3579	0	0	-0,007158	0,964229	0,13772387
CG30491-RA	CG30491	0,0506457	6,0124	11,9612	0,0570234	0,0772385	47,8725	-0,014353	0,95448	0,13772387
CG30492-RA	CG45050	4,33762	7,23712	0,01646	8,6721	0,0244421	0,0193594	-0,285615	0,178221	0,6279808
CG30492-RB	CG45050	0,0167493	0,0201517	0,0160803	0,0176062	3,13162	5,47483	-0,285925	0,17773	0,13772387
CG30492-RC	CG45050	0,0186655	0,0194484	0,01792	0,0197427	0,0267416	10,7538	-0,284321	0,166216	0,6279808
CG30492-RD	CG45050	0,0179701	0,0190544	0,0172523	0,0189642	3,21363	0,0582116	-0,118676	0,536167	0,6279808
CG30492-RE	CG45050	6,96476	2,10539	6,58732	8,42378	5,83351	14,1538	-0,152331	0,423718	0,6279808
CG30492-RF	CG45050	12,1073	0,113559	14,3458	8,57841	10,7337	0,53666	-0,24672	0,22975	0,6279808
CG30493-RB	Coq9	8,75474	7,4484	11,1454	7,97533	7,42655	0,00891925	0,071001	0,788091	0,6279808
CG30494-RA	CG43340	0,448873	0,0170018	0,637622	0,664549	0,865372	21,7572	-0,630474	0,052875	0,6279808
CG30495-RA	CG30495	3,17482	2,91444	0,196247	0,412589	13,4411	170,827	-0,209296	0,430899	0,6279808
CG30496-RA	CG30496	2,24221	0,246153	3,4283	4,57287	1,45226	0,00891925	-0,098314	0,743359	0,6279808
CG30497-RA	CG46385	10,0413	9,81577	0,0148541	9,01862	12,1384	0,00891925	0,431181	0,057291	0,6279808
CG30497-RB	CG46385	43,5009	56,3265	47,9391	42,7946	13,1569	0,00890626	0,485018	0,036666	0,6279808
CG30497-RC	CG46385	0,0103265	0,0094061	5,59153	5,40931	3,4873	0,00890626	0,460128	0,045446	0,6279808
CG30498-RA	boca	21,9627	2,10035	0,0322232	3,9486	2,36001	40,3892	-0,449617	0,078681	0,6279808
CG30499-RA	Rpe	18,1085	15,7564	33,0649	22,5868	34,5919	176,625	-0,491034	0,115062	0,6279808
CG30502-RA	Cyp6d5	57,3276	35,2159	80,6797	61,6645	0,0222875	0,0167972	0,319182	0,251352	0,6279808
CG30503-RA	fa2h	18,8023	35,9082	23,1737	30,3146	14,7152	20,8898	-0,571859	0,090382	0,6279808
CG3050-RA	CG30503	0,0956694	0,0871423	0,0918484	0,121679	1,4766	15,0453	0,056227	0,831252	0,6279808
CG3051-RA	AMPKalpha	0,0240305	0,0218886	12,5253	0,0253379	11,5829	13,5939	-0,266739	0,145619	0,6279808
CG3051-RB	AMPKalpha	30,4427	34,0811	7,407	46,5519	15,9922	0,733108	-0,26811	0,143603	0,6279808
CG3051-RC	AMPKalpha	0,0250699	0,0228354	1,00517	0,0264995	5,64892	0,00361473	-0,266615	0,145836	0,6279808
CG3052-RA	HLH4C	2,38968	0,384136	0,3696	2,1961	0,0241903	0,574092	0,043041	0,881023	0,6279808
CG3054-RA	l(2)k05819	2,38895	5,4923	37,4333	0	0,045617	0,174664	0,038283	0,874919	0,6279808
CG3054-RB	l(2)k05819	4,77938	15,3466	24,7276	4,74807	0,0245834	0,0830428	0,082558	0,735928	0,13772387
CG3056-RA	ssx	6,00259	12,9768	8,36609	18,9434	4,65284	6,11061	0,255834	0,390632	0,6279808
CG3056-RB	ssx	13,2545	17,7599	13,9645	14,6224	5,05967	7,99009	0,251242	0,399714	0,6279808
CG3057-RA	colt	12,5778	6,38362	23,7498	355,139	38,7137	12,9029	-0,292649	0,320444	0,13772387
CG3057-RB	colt	0,0467077	0,0425446	0,0448422	29,387	28,5152	0,0531518	-0,292602	0,320895	0,13772387
CG3057-RC	colt	10,4367	0,0427258	0,0450332	11,3071	7,79687	16,3644	-0,293222	0,320014	0,6279808
CG3057-RD	colt	10,7233	20,6347	38,7484	0,0520668	0,0705249	30,5672	-0,294005	0,318787	0,6279808
CG3058-RA	Dim1	19,0983	0	0	5,7086	0,0225067	0,0760053	0,023936	0,93426	0,6279808
CG3059-RA	NTPase	20,2529	7,08936	0,0298292	0,0345612	2,83919	24,0764	0,042861	0,825131	0,13772387
CG3059-RB	NTPase	7,92288	30,0834	43,7011	6,77433	0,0617857	9,35369	0,036911	0,849311	0,6279808
CG3059-RC	NTPase	33,8492	0,0292783	0,0308594	6,08892	4,63611	9,74902	0,04167	0,829893	0,6279808
CG3059-RD	NTPase	0,0321433	5,90914	8,00775	5,30415	6,79184	35,1683	0,043631	0,822651	0,6279808
CG3060-RA	mr	2,7004	67,2673	4,32918	47,3398	0,0538784	2,80372	0,05459	0,826167	0,6279808
CG3061-RA	CG3061	37,5794	52,4738	42,3072	58,5407	32,7727	8,87282	0,00245	0,99092	0,6279808
CG3062-RA	CG3062	0,31498	2,40915	1,93785	6,45001	111,686	2,35643	-0,329278	0,3541	0,6279808
CG3065-RA	CG3065	0,0352655	0,0321223	2,69163	0,0382087	2,0558	1,727	0,249952	0,287252	0,6279808
CG3065-RB	CG3065	7,40986	7,69466	12,8645	8,11513	5,12874	7,27062	0,249952	0,287252	0,6279808
CG3065-RC	CG3065	5,12878	5,27229	0,035171	6,85396	0,0539435	2,40942	0,249952	0,287252	0,6279808
CG3066-RA	Sp7	2,22934	0,0319178	0,0336415	1,3765	2,46905	0,0387353	-0,315214	0,377929	0,6279808
CG3066-RB	Sp7	0,0367564	0,372427	2,30171	7,86045	3,46341	1,92956	-0,284197	0,426657	0,6279808
CG3066-RC	Sp7	0,0350411	5,61033	11,9961	2,17074	10,1735	3,75506	-0,315214	0,377929	0,13772387
CG3066-RD	Sp7	0,892079	3,99779	5,67227	2,52372	14,4031	4,58302	-0,314932	0,378376	0,6279808
CG3068-RA	aurA	1,70125	1,76006	2,70201	3,4383	1,64062	2,10697	0,202354	0,389453	0,6279808
CG3069-RA	Taf10b	19,1568	16,9373	26,6119	337,104	59,5027	30,0004	-0,22414	0,393175	0,6279808
CG3071-RA	Dsim\GD16604	16,5535	11,8988	21,1677	4,27632	19,8543	18,4519	-0,249028	0,340911	0,6279808
CG3073-RA	temp	6,83805	5,34469	8,71928	17,1368	2,90942	2,25646	-0,07621	0,749551	0,13772387
CG3074-RA	Swim	44,3089	0	0,711441	0,0132379	0,248578	0,0135137	-0,624616	0,055507	0,6279808
CG3074-RB	Swim	12,0806	6,45369	0,536034	0,0437667	0	0,0446787	-0,628163	0,054003	0,13772387
CG3075-RA	Nf-YC	5,54345	7,81875	6,94508	5,94677	3,42099	4,04706	0,292554	0,263063	0,6279808
CG3077-RA	CG3077	9,85961	21,8796	4,85915	12,0649	5,35359	6,9414	0,697333	0,02139	0,6279808
CG3078-RA	CG3078	3,02758	0,658619	2,95672	16,0114	2,78424	1,97424	0,067365	0,815636	0,6279808
CG3081-RA	CG3081	0,905149	0,378034	0,0165257	0,921411	0,485651	4,49527	0,045052	0,887305	0,6279808
CG3082-RA	l(2)k09913	30,7045	0,0817751	3,12145	45,3326	0	34,7282	0,001694	0,993616	0,6279808
CG3082-RB	l(2)k09913	0,0357811	0	4,49861	0,0388164	0,319974	0,0396253	0,000888	0,996653	0,6279808
CG3082-RC	l(2)k09913	0,0344923	31,5143	297,962	0,0373003	38,1268	0,0380775	0,001694	0,993616	0,6279808
CG3082-RD	l(2)k09913	13,6984	0,0325919	40,7116	0,0525234	0,0525771	10,8913	0,051879	0,811709	0,6279808
CG3083-RA	Prx6005	0,0911808	0,0830538	11,8178	0,114491	13,5271	12,4804	-0,236171	0,436796	0,6279808
CG3085-RA	CG3085	2,45382	2,79388	17,3091	2,55121	2,00751	1,55121	0,405024	0,104962	0,6279808
CG3086-RB	MAPk-Ak2	0,0341536	0,0311095	0,0327895	0,0369033	0,492868	2,00562	-0,201837	0,34584	0,6279808
CG3086-RC	MAPk-Ak2	7,77494	10,2566	9,26616	11,4335	2,0884	0,296443	-0,201082	0,347725	0,6279808
CG3086-RD	MAPk-Ak2	39,7134	52,591	41,2556	63,5156	0	0,0376723	-0,201801	0,345885	0,6279808
CG3088-RA	CG3088	0	5,39407	0,35035	1,13265	0,115858	0	0,01562	0,897983	0,6279808
CG3090-RA	Sox14	5,8296	13,6436	8,16547	14,5565	3,44673	6,65823	0,233294	0,43362	0,6279808
CG3090-RB	Sox14	9,17091	7,54834	2,01826	10,8637	1,50574	0,937144	0,272099	0,359705	0,6279808
CG3091-RA	CG3091	0,776627	0,283979	0,0544209	0,805878	6,32253	3,28334	-0,208581	0,552634	0,6279808
CG3091-RB	CG3091	1,30375	4,88836	2,397	0,8708	1,40727	0,843216	-0,208581	0,552634	0,6279808
CG3092-RA	CG3092	0,0760659	0,184763	0,134495	0,236779	0,354424	0,244542	-0,058458	0,85049	0,6279808
CG3093-RA	dor	3,74215	5,31627	5,08283	0	0	0	-0,040615	0,863554	0,6279808
CG3095-RA	hfw	7,53129	8,73636	8,18783	9,59587	3,32121	3,74111	0,458148	0,098084	0,6279808
CG3096-RA	Brd	4,71085	3,55699	0,411559	73,5616	30,3705	0,313642	0,550035	0,121527	0,6279808

gene_id	Symbol	W1_FPKM	W2_FPKM	W3_FPKM	MCM51_FPKM	MCM52_FPKM	MCM53_FPKM	FC	p-value	p-adj
CG3097-RA	CG3097	30,5568	0,126376	0,149851	0,290268	0,126663	0,127695	0,22659	0,335768	0,6279808
CG3099-RB	CG42797	5,12918	27,5576	33,0795	38,2157	22,4552	34,0427	-0,009483	0,971988	0,6279808
CG31000-RA	b6	14,6492	18,8048	0,0161513	0,026856	0,818396	8,2131	0,248054	0,27	0,6279808
CG31000-RB	heph	0,01921	0,0174978	0	0,0210683	0,0285372	0,0215074	0,191784	0,411793	0,6279808
CG31000-RC	heph	0,01921	0,0174978	0	0,0182247	2,49047	1,93958	0,356869	0,117634	0,6279808
CG31000-RD	heph	9,54211	12,5077	0,291887	0,0243186	0,0329397	0,0248253	0,262323	0,246413	0,6279808
CG31000-RE	heph	5,69952	6,72182	0,0184427	0,0230914	0,0312774	0,0235725	0,244352	0,273139	0,6279808
CG31000-RF	heph	3,20533	5,40582	0,0184427	0,0277261	0,0375552	0,0283039	0,258469	0,248651	0,6279808
CG31000-RG	heph	4,13551	0,0194171	9,88737	0,0225462	0,0305389	0,023016	0,262495	0,245864	0,6279808
CG31000-RH	heph	0,0195858	0,0178401	5,54125	0,0204727	0,0277303	0,0208993	0,267325	0,24082	0,6279808
CG31000-RI	heph	0,0191932	0,0174825	3,5514	0,0299165	0,0405221	0,0305399	0,255946	0,25524	0,6279808
CG31000-RJ	heph	0,022531	0,0205228	0,0204657	0,0348353	0,0471846	0,0355612	0,255794	0,254657	0,6279808
CG31000-RK	heph	0,0236859	0,0215748	0,0188035	0,0431646	3,31257	6,1718	0,26516	0,241142	0,6279808
CG31000-RL	heph	0,021824	0,0198788	0,0184266	17,3387	12,9584	13,0948	0,256928	0,253401	0,6279808
CG31000-RM	heph	0,0222503	0,0202671	0,0216311	0	0	0	0,17272	0,452797	0,6279808
CG31000-RN	heph	0,02194	0,0199844	0,0227399	0,0976153	0,21298	0,0996494	0,255488	0,256385	0,6279808
CG31000-RO	heph	0,0201656	0,0183682	0,0209524	0,133494	0,127565	0,0433686	0,267325	0,24082	0,6279808
CG31000-RP	heph	0,0175516	0,0159872	0,0213616	0,196106	0,265627	0,200193	0,184873	0,425901	0,6279808
CG31000-RQ	heph	0,0231142	0,021054	0,0210637	1,53924	1,16218	2,19324	0,267983	0,265448	0,6279808
CG31000-RR	heph	0,0220057	0,0200443	0,0193601	0	0	0	0,265148	0,244741	0,6279808
CG31000-RS	heph	0,0261617	0,0238299	3,81665	0,105763	0,230756	0,107967	0,349419	0,131036	0,6279808
CG31000-RT	heph	0,0215114	0,0195941	0,022191	0	0	0	0,358485	0,117484	0,6279808
CG31000-RU	heph	0,0196207	0,0178719	0,0211268	0,467215	0	0	0,355309	0,123725	0,6279808
CG31000-RV	heph	0,0280974	0,0255931	0,0251168	0,589082	1,51724	1,01393	0,181783	0,429282	0,6279808
CG31002-RA	heph	0,0323795	0,0294935	0,0206522	0	0	0	-0,122182	0,653324	0,6279808
CG31003-RA	Ugt35D1	36,3477	40,99	0,0455178	0,0459329	0,0622164	0,04689	0,082183	0,806626	0,6279808
CG31004-RA	gskt	0,297472	0,220153	37,5376	60,2546	20,9437	44,1981	-0,042786	0,852692	0,6279808
CG31004-RB	mesh	0	0	0	122,732	60,9324	58,4402	-0,042786	0,852692	0,6279808
CG31005-RA	mesh	0	0	0	0,0145407	0,0196955	0,0148437	0,816075	0,006012	0,6279808
CG31006-RA	qless	120,363	163,933	90,8604	100,365	6,77828	47,7305	0,121719	0,635135	0,6279808
CG31006-RB	stops	1,13706	1,63203	1,32321	16,2852	0,0504663	9,33415	0,088979	0,73344	0,6279808
CG31007-RA	stops	2,87962	2,27128	2,88817	35,1031	2,06685	17,6483	-0,0276	0,879281	0,6279808
CG31008-RA	CG31007	0	89,7366	121,52	65,4112	112,071	0,994074	NA	NA	0,6279808
CG31009-RA	CG31008	0	19,9195	0,467306	61,2081	4,82648	1,214	-0,267219	0,252221	0,6279808
CG3100-RA	Cad99C	8,67875	9,61827	8,72313	16,0464	7,07879	9,25498	0,457875	0,077668	0,6279808
CG31010-RA	CG31010	0	0,0226038	0,0938874	0,0247944	5,16632	0,349877	NA	NA	0,6279808
CG31012-RA	cindr	50,086	77,9345	48,4495	65,1239	32,4019	36,9189	-0,057856	0,823959	0,6279808
CG31012-RB	cindr	0,024048	0,0219046	0,0230875	0,0253574	0,0343467	0,0258858	0,147337	0,581173	0,6279808
CG31012-RC	cindr	0,0292933	0,0266823	0,0281233	0,0312799	7,52473	0,0319317	0,225634	0,37166	0,6279808
CG31012-RD	cindr	0,0360545	0,0328409	0,0346144	0,0391392	0,0530143	0,0399548	0,124944	0,633259	0,6279808
CG31013-RA	CG31013	0,16837	0,0402855	0	0,146517	1,02818	0,0547526	-0,083309	0,809969	0,6279808
CG31014-RA	PH4alphaSG1	12,2968	0,27815	0,0405433	0,0369762	39,9085	5,57153	0,351043	0,242821	0,6279808
CG31015-RA	PH4alphaPV	4,35647	0,04331	5,45185	0	0,0441721	422,979	0,264242	0,34554	0,6279808
CG31016-RA	CG31016	0	0,041111	0,0584851	0,120655	12,7119	186,068	-0,07431	0,727365	0,6279808
CG31017-RA	PH4alphaNE3	0,121836	153,836	0,0625198	0	3,81042	0,0537464	-0,238065	0,382346	0,13772387
CG31019-RA	CG31019	0,734177	0,911916	1,0733	1,40359	0,0335842	0,0291755	-0,504317	0,096213	0,6279808
CG31020-RA	spdo	2,08527	0,0355152	0,564161	0,114292	0,057748	0	-0,382344	0,196659	0,6279808
CG31021-RA	CG31021	0	230,832	0,0548445	2,88244	11,4445	0,0533203	-0,154664	0,495209	0,6279808
CG31022-RA	PH4alphaEFB	38,2158	0,674025	0,815782	0,108639	0	0	0,125647	0,692134	0,6279808
CG31025-RA	Ppi1	0,0185624	0,492837	0,720873	1,02734	0,863367	0,0197227	-0,120277	0,714129	0,6279808
CG31025-RB	Ppi1	0,0756522	0,0169079	0,017821	0,0193201	0,0261692	0,101568	-0,120277	0,714129	0,6279808
CG31028-RC	CG31028	1,67509	166,484	259,757	194,905	345,988	346,046	0,034367	0,89469	0,6279808
CG31028-RD	CG31028	4,86993	0,0454009	0,0478527	0,0560055	0,0758598	0,0571726	0,054123	0,835872	0,6279808
CG31029-RA	CG31029	0,806073	0,0516819	0,118025	0,140816	0,0514471	0,73249	-0,381159	0,21026	0,6279808
CG31030-RA	CG31030	36,0469	31,4852	0,0170178	9,50807	0,0249407	0,0187968	-0,695176	0,014701	0,6279808
CG31030-RB	CG31030	0,0419884	6,76954	5,80162	0,0224846	3,78749	4,66152	-0,695176	0,014701	0,6279808
CG31031-RA	sima	0,781543	0,0195432	1,32331	30,327	1,3746	0,890686	-0,757575	0,033247	0,6279808
CG31033-RA	Atg16	0,0409223	0,0176104	2,04297	5,04961	2,32886	0,0458982	-0,02072	0,920903	0,6279808
CG31033-RB	Atg16	2,01643	20,3725	0,039508	0,0452391	0,0612766	0,0461818	-0,022192	0,915374	0,6279808
CG31033-RC	Atg16	5,04793	44,6657	4,34027	0,0445057	4,73412	5,93375	-0,06012	0,776128	0,6279808
CG31033-RD	Atg16	4,071	0,0182015	7,73394	7,21771	4,21566	5,05136	-0,353898	0,154002	0,6279808
CG31033-RE	Atg16	0,0444157	0,338126	0,0426417	0,0492272	0,0666786	0,050253	-0,019133	0,926725	0,6279808
CG31034-RA	Jon99Cii	10,6107	1718,58	9,1426	0,0200308	14,9978	9,97621	-0,766365	0,027027	0,6279808
CG31036-RA	CG31036	2,35648	3,05845	0,0189326	5,59658	0,0281768	0,522488	-0,101509	0,66878	0,6279808
CG31036-RB	CG31036	0,838109	1,05081	0,06144	0,0205814	0,0281413	170,984	-0,105571	0,655083	0,6279808
CG31037-RA	ca	4,51022	76,3533	0,340082	15,0073	17,7828	20,1002	-0,068941	0,772805	0,6279808
CG31038-RA	CG31038	0,0157824	0,520552	37,9287	7,26814	31,0069	1,35925	0,078835	0,747934	0,6279808
CG31038-RB	CG31038	0,0192155	2,6286	7,3987	1,59032	4,73973	0,224689	-0,220902	0,393956	0,6279808
CG31038-RC	CG31038	12,1192	14,3167	1,34667	0,397879	1,49887	30,4443	0,257623	0,268322	0,6279808
CG31038-RD	CG31038	0,0199224	0,0617297	0,368893	77,4942	0,0601209	5,63904	-0,249476	0,334864	0,6279808
CG31038-RE	CG31038	0,0198984	8,29457	1,09451	2,26252	0,594242	92,0774	0,08227	0,737112	0,6279808
CG31038-RF	CG31038	10,2761	0,0143757	2,6716	1,1223	2,2335	0,55638	0,079079	0,747131	0,6279808
CG31039-RA	Jon99Ci	14,1435	1,63116	0,0191267	12,894	2,9641	0,0212358	-0,152303	0,634027	0,6279808
CG31040-RA	CG3104	3,06957	0,0436872	2,99302	12,5857	2,98574	6,93512	0,096122	0,641155	0,6279808
CG31041-RA	CG3104	1,33326	3,94689	1,89716	0,149916	4,40952	0,0371013	0,145998	0,677418	0,6279808
CG31043-RA	Cog7	6,67803	11,1037	0,0650634	17,2931	10,1245	12,4611	-0,011012	0,968082	0,6279808
CG31043-RB	CG31041	4,74023	2,6168	70,6324	16,3531	0,390287	0,0442962	0,116342	0,666435	0,6279808
CG31043-RC	gukh	5,49434	6,68021	0,037323	12,9596	77,3205	89,4876	0,078329	0,773174	0,6279808
CG31043-RD	gukh	5,81397	12,1893	88,8365	3,66968	7,61335	15,3949	-0,019904	0,942323	0,6279808
CG31044-RA	gukh	6,85688	2,05956	19,5982	63,8475	11,601	5,37892	0,083294	0,782537	0,6279808
CG31045-RA	gukh	1,43155	32,1481	14,1238	27,5355	5,35982	256,414	-0,150696	0,593507	0,6279808
CG31045-RB	lncRNA:CR31044	0,611814	5,72681	0,0479612	12,3621	54,9527	44,6452	0,121329	0,667652	0,6279808
CG31045-RC	Mhcl	0,0167252	16,7597	0,0108223	15,8619	0,082295	0,0620225	-0,08809	0,743796	0,6279808
CG31045-RD	Mhcl	0,0102132	1,87293	0,0096516	2,928	4,24089	4,59481	-0,149745	0,557761	0,6279808
CG31045-RF	Mhcl	14,648	0,0487866	30,2274	0,017333	5,59649	6,49212	-0,161494	0,569684	0,6279808
CG31045-RG	Mhcl	13,7703	2,307	2,27415	0,010425	4,13011	5,16742	-0,16802	0,551104	0,6279808
CG31048-RB	Mhcl	0,0112725	34,6907	0,0816432	15,6604	0	0	0,011123	0,969857	0,13772387
CG31048-RC	Mhcl	0,0100531	0,0152345	46,6855	24,6201	7,36449	12,5912	-0,144224	0,628528	0,6279808
CG3104-RA	spg	0,905725	1,3	0,58497	1,96628	0,888527	0,61672	-0,687416	0,002687	0,13772387
CG3104-RB	spg	2,82189	4,26533	2,72391	5,00902	2,00711	1,91186	-0,676573	0,003357	0,6279808
CG31050-RA	Pask	1,72094	2,47805	4,03416	2,1283	3,33964	4,32641	-0,035069	0,864238	0,6279808
CG31051-RA	CG31050	0	0,43996	0,278232	0,503212	1,43854	1,15625	0,599612	0,008836	0,6279808
CG31052-RA	CG31051	9,69377	11,749	12,5354	32,8793	6,76519	1396,69	-0,486592	0,14244	0,6279808
CG31053-RA	Dpse\GA15967	7,16026	0,0783678	0,788035	0,0233947	15,2084	1,63442	0,18042	0,609893	0,6279808
CG31054-RA	nenya	1,08783	0,330292	1,50856	0,655774	4,73811	0,435814	-0,260573	0,46247	0,6279808
CG31055-RA	CR31054	0,887776	3,95416	10,2078	4,1136	0,0313213	2,40924	-0,424077	0,22866	0,6279808
CG31055-RB	CG31055	0,0338735	0,0307283	0,0323877	0,0364139	0,0493228	0,0371727	-0,424077	0,22866	0,13772387
CG31055-RC	CG31055	0,0329117	3,38059	2,80577	5,68401	2,50623	3,05169	-0,424077	0,22866	0,6279808
CG31056-RA	CG31055	0,0337351	22,4705	24,6687	0	0	19,4851	0,128512	0,630806	0,6279808
CG31057-RA	Acp98AB	1,27069	0	0,609969	8,07259	0	52,031	0,320005	0,150686	0,6279808
CG31058-RA	tau	26,5124	34,0689	26,1381	27,0998	21,5116	18,5991	-0,401738	0,234325	0,6279808
CG31059-RA	tau	0,493841	0,854666	1,01935	1,25735	1,6559	1,07327	NA	NA	0,6279808
CG3105-RA	Gr98b	0	1,24806	1,10499	0	0	1,07975	0,339467	0,221118	0,6279808
CG31060-RA	CG3106	37,3149	29,8344	3,0016	4,1091	1,55659	2,80328	0,044335	0,715924	0,6279808

gene_id	Symbol	W1_FPKM	W2_FPKM	W3_FPKM	MCM51_FPKM	MCM52_FPKM	MCM53_FPKM	FC	p-value	p-adj
CG31061-RA	Gr98c	0,0807774	0	0	0	1,27568	0	-0,040292	0,831087	0,6279808
CG31062-RA	Gr98d	0,079995	0	0	0	0	0	0,030863	0,915776	0,6279808
CG31062-RB	side	4,09591	0,134046	2,44193	69,8534	1,60273	54,9864	0,028241	0,922437	0,6279808
CG31063-RA	side	2,28167	5,35152	3,23365	0,35382	2,68247	0,235141	-0,390923	0,19145	0,6279808
CG31064-RA	CG42813	16,6446	15,1992	27,7586	3,79098	2,23383	7,24365	0,042379	0,855566	0,6279808
CG31064-RB	CG31064	4,17666	4,63711	4,2425	1,81821	0,690793	1,16479	-0,194767	0,368207	0,6279808
CG31064-RE	CG31064	3,84813	2,23931	1,91524	4,09858	0,10288	2,71806	0,002501	0,991253	0,6279808
CG31065-RB	CG31064	1,68209	0,416696	2,66193	0,949285	5,80035	0,558418	-0,140548	0,658352	0,6279808
CG31068-RA	ppk31	0,521926	7,89369	10,1335	20,2112	30,5376	0,433707	-0,334119	0,288785	0,6279808
CG31069-RA	CG31068	0,629022	0,171798	0,603899	0,187628	7,75796	0,256214	-0,095398	0,772109	0,6279808
CG3106-RA	spn-D	0,161318	0,0475407	2,03313	10,6857	0,575467	0,264635	-0,348502	0,29113	0,6279808
CG31072-RA	CG3107	0,0208397	0,0189823	0,0200074	0,0175173	33,6966	22,5373	0,306229	0,173069	0,6279808
CG31072-RB	CG3107	0,0203833	0,0185665	0,0195692	26,828	0,0513963	0,0387353	0,306229	0,173069	0,6279808
CG31072-RC	CG3107	45,997	30,3477	39,0868	0,0379447	0,0540088	0,0407043	0,306656	0,172589	0,6279808
CG31075-RA	Lerp	0,0188322	2,59205	25,4001	4,59275	1,21566	2,26943	-0,138515	0,670353	0,6279808
CG31076-RA	Lerp	0,870522	29,4606	0,0116107	0,0201718	2,21357	14,8584	-0,027776	0,924081	0,6279808
CG31077-RA	Lerp	5,1127	0,0110158	2,37752	2,89787	0,0273229	0,0126568	NA	NA	0,6279808
CG31079-RA	CG31075	0,0418025	177,935	27,3907	11,535	0,0461358	19,1393	-0,070526	0,562659	0,6279808
CG3107-RA	CG31076	21,5064	28,0975	16,219	43,2406	93,2411	19,5941	-0,027166	0,908978	0,6279808
CG3107-RB	CG31077	0	0	0	0	51,306	0	-0,027063	0,909339	0,6279808
CG3107-RC	CG31079	0	18,3146	0,0429303	53,7863	0,0671805	0,0506313	-0,026952	0,909676	0,6279808
CG31080-RA	CG3108	59,3001	1678,04	7,97922	5,06444	9,07147	0,13163	-0,127402	0,626881	0,6279808
CG31081-RA	TwdlH	16,3212	17,3552	20,812	15,0582	13,2402	1,07955	0,35261	0,310881	0,6279808
CG31082-RA	TwdlR	1,36962	1,27797	0,83105	1,10965	0,618383	17,5754	-0,246984	0,268044	0,6279808
CG31084-RA	scrib	17,466	20,4319	15,9631	22,9211	4,26727	3,91881	0,960705	0,001665	0,6279808
CG31086-RA	-	0,151318	30,2869	0,145274	29,3976	33,6075	13,0774	-0,466745	0,144994	0,6279808
CG31087-RA	CG31086	163,968	0,0277016	0,100481	11,0137	645,339	393,166	-0,448688	0,166419	0,6279808
CG31088-RA	CG31087	7,49927	6,56012	12,9069	7,45306	0,465195	12,194	-0,01548	0,9633	0,6279808
CG31089-RA	CG31088	0,35976	6,71962	0,0348086	8,86284	3,77184	3,41096	-0,048684	0,890705	0,6279808
CG3108-RA	CG31089	0,156578	0,213933	0,400864	2,90077	1,30428	0,0354487	0,238269	0,475303	0,6279808
CG31091-RA	mRpL16	37,1727	31,3245	20,749	17,5468	17,8528	20,0532	0,092087	0,794135	0,6279808
CG31092-RA	CG31091	0,230283	0,111871	4,49536	0,230793	0,183168	11,339	0,325697	0,173577	0,6279808
CG31092-RB	LpR2	9,16991	10,4738	0,0182569	2,15646	1,57981	1,8534	0,343799	0,14937	0,6279808
CG31092-RC	LpR2	2,72561	0,0173215	0,0176622	0,0275407	0,037304	0,0281146	0,321592	0,179456	0,6279808
CG31092-RD	LpR2	4,19852	0,0167572	0,0167793	3,05305	3,11086	1,71391	0,546878	0,021364	0,6279808
CG31092-RE	LpR2	0,0147206	0,0159195	0,0162506	0,0151946	0,0205812	0,0155112	0,319279	0,181686	0,6279808
CG31092-RF	LpR2	0,0147304	0,0154179	1,51466	1,67449	0,0200811	0,0151343	0,341366	0,151397	0,6279808
CG31092-RG	LpR2	0,0143841	0,0149693	0,166647	0,0148354	0,0200946	0,0151445	0,323357	0,175776	0,6279808
CG31093-RA	LpR2	0,0143935	8,04843	9,82294	9,85107	1786,31	8,35512	0,01562	0,897983	0,6279808
CG31094-RA	CG31093	0	0	0	0	0	0	-0,068724	0,74483	0,6279808
CG31094-RB	LpR1	10,7813	2,43712	4,31323	0,0198139	9,55182	0,0202268	-0,066908	0,749623	0,6279808
CG31094-RC	LpR1	0,0190165	4,45182	0,0249587	0,0191405	0,0268381	0,0195394	-0,072001	0,733175	0,6279808
CG31094-RD	LpR1	0,018397	0,0134085	2,31069	0,0181444	0,025926	0,0185225	-0,070871	0,737543	0,6279808
CG31094-RE	LpR1	0,0174773	0,0134175	0,0141421	0,01755	0,0245767	0,0179157	-0,061114	0,769609	0,6279808
CG31094-RF	LpR1	0,0169266	0,0131021	0,0138096	1,71849	0,0237715	1,26125	-0,070182	0,737899	0,6279808
CG31096-RA	LpR1	0,0164341	0,0131106	0,0138186	1516,28	1,21016	2091,06	0,514751	0,106247	0,6279808
CG31097-RB	Lgr3	0,536899	0,621494	0,644319	0	0	0,0673761	0,403491	0,243631	0,6279808
CG31098-RA	CG31097	1,00521	1,94382	10,8763	0,778535	4,21519	0,826787	-0,026345	0,919075	0,6279808
CG31099-RA	CG31098	35,9603	6,07053	67,6773	11,1527	6,0847	5,02824	-0,074596	0,705461	0,6279808
CG3109-RA	CG31099	0,0705939	1062,27	1,06745	0,132504	0,909997	0,478857	-0,273794	0,342195	0,6279808
CG31100-RA	CG31100	15,808	11,8086	1,94742	2,60117	0,0902871	0,0138276	0,106141	0,624844	0,6279808
CG31102-RA	CG31102	3,83544	2,098	2,3376	4,96622	5,39176	5,58904	0,112384	0,670021	0,13772387
CG31103-RB	CG31103	4,16445	3,24885	3,14434	5,8015	0,159057	2,15687	-0,799495	0,020054	0,6279808
CG31104-RA	CG31104	1,76904	1,13231	11,2791	15,2209	9,8033	10,2055	0,121521	0,733949	0,13772387
CG31105-RB	ppk22	0,0966587	0,0636444	0,320507	0	0,0198532	0,174883	0,264038	0,443316	0,6279808
CG31106-RA	CG31106	4,91425	1,98417	8,22177	2,63937	10,3647	16,1626	-0,19371	0,569777	0,6279808
CG31108-RA	Dsim\GD18234	6,34398	4,16798	8,77681	5,07894	1,7592	4,95921	0,264546	0,312838	0,6279808
CG31109-RA	CG31109	11,9293	119,707	109,083	0,0272446	0,036903	128,535	0,276775	0,20756	0,6279808
CG31111-RA	CG31111	5,05612	10,7049	10,9322	5,51288	0,0339418	0,0454946	-0,19009	0,470267	0,6279808
CG31115-RB	CG31115	0,268328	0,244412	0,354216	0,458871	2,42353	0,343919	-0,294577	0,399912	0,6279808
CG31116-RA	ClC-a	2,19902	0,0169031	0,03717	0,0159601	0,057303	0,043187	-0,147396	0,58583	0,6279808
CG31116-RC	ClC-a	0,016957	0,563765	0,0374332	0,0174062	0,057748	0,0435224	-0,157375	0,560314	0,6279808
CG31116-RD	ClC-a	0,0154467	0,0140699	0,0382149	2,84153	0,0612346	0,0461501	-0,137852	0,609479	0,6279808
CG31116-RE	ClC-a	0,0167933	0,0152965	3,89493	0,0423055	2,64381	3,03101	-0,162112	0,550691	0,6279808
CG31116-RF	ClC-a	3,43612	3,35159	0,00966926	0,042634	0,0139204	0,0104912	-0,147812	0,583385	0,6279808
CG31118-RA	RabX4	25,6044	37,5852	29,1743	0,0144167	19,789	0,0149626	0,22759	0,34725	0,6279808
CG31119-RA	HDAC11	0,263134	17,5936	0,421041	21,117	0,0198532	0,0254451	-0,564088	0,114346	0,6279808
CG31120-RA	sud1	4,76119	7,3856	8,95642	8,54828	10,6696	9,70284	-0,07909	0,75671	0,6279808
CG31120-RB	sud1	4,07627	3,50636	10,084	0,798012	1,15938	5,33008	-0,154832	0,544662	0,6279808
CG31121-RA	CG31121	0,0213792	0,0194736	0,0205253	0,0224005	3,31516	2,00986	0,066671	0,812125	0,6279808
CG31121-RB	CG31121	14,2491	18,7373	14,2752	24,3526	6,9699	0,0287884	0,012868	0,963732	0,6279808
CG31122-RA	CG31122	7,87802	10,3262	287,849	0,421458	0,0833713	0,0628336	0,16075	0,535926	0,6279808
CG31125-RA	CG31125	1,31994	1,43846	1,96873	51,5738	7,87302	0,00779291	-0,055432	0,849839	0,13772387
CG31126-RA	CG31126	28,4079	0,0191149	49,1764	1,58781	0,0253013	0,0224252	-0,109012	0,693381	0,13772387
CG31127-RA	Wsck	4,71645	42,9453	5,11869	1,35671	0,025702	37,405	-0,182738	0,408113	0,6279808
CG31128-RA	CG31128	0	0	0	10,9953	0	2,15575	-0,041811	0,731452	0,6279808
CG31131-RB	CG31131	0,242926	0,12293	0,647843	3,18717	1,46056	2,1804	NA	NA	0,6279808
CG31132-RA	BRWD3	5,27417	3,73442	6,41555	0,0150764	0,558198	4,52039	-0,075552	0,72942	0,6279808
CG31133-RA	Slimp	3,79702	4,56953	0,163835	8,73267	4,03058	5,52526	0,035683	0,892924	0,6279808
CG31136-RA	Syx1A	32,7306	33,3125	6,23643	19,947	3,59294	22,8162	0,190209	0,351632	0,6279808
CG31137-RA	twin	0,0267622	0,0170227	0,0333527	0,0264676	0,0336729	0,0579109	-0,03256	0,88542	0,6279808
CG31137-RB	twin	0,0255218	7,72747	19,1085	0,0200247	12,8327	0,0432982	-0,026043	0,908263	0,6279808
CG31137-RC	twin	0,0234229	9,68238	0,0235649	70,6202	0,0358505	0,0997222	-0,033012	0,883662	0,6279808
CG31137-RD	twin	0,0231601	0,0213352	0,0181611	0,0429396	0,0271236	8,4992	0,067135	0,76846	0,6279808
CG31137-RE	twin	15,4316	0,0316438	76,5419	0,0488984	57,8392	2,78019	-0,038454	0,864659	0,6279808
CG31137-RF	twin	0,0245453	0,0222583	0,0365209	0,0496351	0,0581619	0,199866	-0,03256	0,88542	0,6279808
CG31139-RA	CG31139	0,0520693	18,294	15,655	0,0617819	19,3406	19,3936	0,407647	0,155897	0,6279808
CG31140-RA	ewg	0,0138912	0,0126531	0,0133364	0,0143009	0,0193707	0,0145989	0,518853	0,030672	0,6279808
CG31140-RB	ewg	14,3873	10,6315	9,65024	0,023991	5,81133	3,73172	0,560957	0,017356	0,6279808
CG31140-RC	ewg	0,0225233	0,0205158	0,0216237	0,0236636	0,0320524	0,0241567	0,576604	0,01416	0,6279808
CG31140-RD	ewg	0,0222503	0,0202671	0,0213616	5,9241	0,0316433	0,0238483	-0,298504	0,277242	0,6279808
CG31140-RE	ewg	0,0196791	6,10349	5,59236	12,2531	2,81657	6,03834	-0,590041	0,03082	0,6279808
CG31141-RA	ewg	0,0194589	0,0177245	0,0186817	0,0202961	0,0274912	0,020719	0,013766	0,948533	0,13772387
CG31142-RA	ewg	0,0192547	0,0263078	0,0277285	0,0200735	0,0271897	0,0204918	0,034427	0,89215	0,6279808
CG31145-RA	ewg	13,2212	10,5045	9,55367	10,5684	6,85184	7,43848	0,087413	0,735662	0,6279808
CG31145-RB	CG31140	0,0373169	0,0320027	0,033731	4,0153	5,71681	3,07741	0,089038	0,731096	0,6279808
CG31145-RC	CG31140	0,0121337	1,82731	0,0279129	24,2646	9,36759	1,38966	0,082154	0,75009	0,6279808
CG31145-RD	CG31140	0,0124891	3,78643	4,91189	0,0406357	2,01474	0,0236888	-0,003225	0,990238	0,6279808
CG31145-RE	CG31140	31,5901	1,10504	3,54909	0,0124406	1,41046	80,138	0,101581	0,693789	0,6279808
CG31146-RD	CG31140	4,18688	2,17493	0,0212258	5,59573	63,4007	0,0388472	0,204664	0,414749	0,6279808
CG31147-RA	CG31141	0,221236	0	0,1062	0,229563	0,193038	0	0,08244	0,767225	0,6279808
CG31148-RA	CG31142	15,0616	31,487	22,6379	9,74034	20,5579	22,0731	-0,225635	0,527992	0,6279808
CG31149-RA	CG31145	8,78152	9,04333	7,00238	14,1228	0,648044	4,71005	0,115687	0,611676	0,13772387
CG3114-RA	CG31145	8,0594	0,0235594	0,0248317	0,0273919	7,94742	0,0279626	0,1864	0,406376	0,6279808

gene_id	Symbol	W1_FPKM	W2_FPKM	W3_FPKM	MCM51_FPKM	MCM52_FPKM	MCM53_FPKM	FC	p-value	p-adj
CG3114-RB	CG31145	0,0258648	0,0172817	0,018215	0,0197664	5,34339	0,0201783	0,03782	0,856699	0,6279808
CG3114-RC	CG31145	0,0189728	0,0173815	0,0183202	0,0198857	0,0371024	0,0203001	0,163355	0,461643	0,6279808
CG3114-RD	CG31145	0,0190824	0,685886	1,29524	1,21557	0,0267737	1,19283	0,164037	0,45965	0,6279808
CG3114-RE	Nlg1	2,10593	1,44345	45,1179	0	0,163304	0	0,16281	0,462979	0,6279808
CG3114-RF	mthl11	0,257606	0,0977688	0,103049	1,52258	0,100168	0,274263	0,213709	0,337285	0,6279808
CG3114-RG	Gba1a	1,07181	2,82799	0,0889467	6,6914	88,0101	2,84915	0,21295	0,338844	0,6279808
CG3114-RH	CG42613	11,2612	12,0479	12,2151	8,1921	6,16538	7,0316	0,214013	0,336439	0,6279808
CG31150-RA	cv-d	9,76786	11,995	0,0211127	0,378591	24,0244	26,9984	-0,009423	0,969023	0,6279808
CG31151-RA	wge	5,79425	8,32022	0,0126705	0,0135649	1,02517	0,0138475	0,050706	0,86283	0,6279808
CG31151-RB	wge	0,0131976	0,0120213	3,66053	6,55587	4,14468	2,78136	0,065066	0,825045	0,6279808
CG31151-RC	wge	3,28469	4,82227	0,898076	1,30845	0,0183737	1,98239	0,04696	0,872854	0,6279808
CG31152-RA	rumi	1,55508	12,7074	15,7119	2,73101	8,27027	10,4508	0,103097	0,760735	0,6279808
CG31155-RA	Dpse\GA16051	14,3321	28,4613	90,8784	0,375383	31,3102	16,939	-0,085992	0,716846	0,6279808
CG31156-RA	CG31156	4,15316	0,0541407	0,0570645	0,0684574	40,6662	0,0698839	0,243651	0,319554	0,6279808
CG31156-RB	CG31156	0,0199685	0,153407	0,215588	0,0687431	0,366425	0,27616	0,239492	0,326059	0,6279808
CG31157-RB	CG31157	0,499818	10,7752	0,311906	14,2815	14,9998	3,26904	-1,827969	1,6E-08	0,6279808
CG31158-RC	Efa6	1,78484	1,54632	0,0111632	0,729226	0	5,27849	0,104383	0,645543	0,6279808
CG31158-RD	Efa6	6,95711	7,19183	6,39093	0,415542	1,18015	0,449557	0,097109	0,66781	0,6279808
CG31159-RA	mRRF2	3,01696	2,15242	3,46719	2,86288	0,0287301	2,76035	0,119532	0,650306	0,6279808
CG31161-RA	CG31161	0,317673	0,144679	0,438415	0,735766	2,35778	0,525065	0,453518	0,195727	0,6279808
CG31163-RB	SKIP	0,0132878	0,0927849	0,539005	0,554766	32,4797	3,44037	-0,082643	0,767666	0,6279808
CG31163-RD	SKIP	11,8846	0,118013	0,0127571	58,8674	22,945	38,0246	0,186123	0,511869	0,6279808
CG31164-RA	Ir94a	0	1,04976	0	0	0,0420692	0	-0,041811	0,731452	0,6279808
CG31169-RA	CG43342	0,471257	3,50292	0,0151004	0,377824	28,3231	0,0168224	0,404042	0,211802	0,6279808
CG31169-RB	CG43342	0,0157286	0,377222	0,934962	0,511059	0,0318921	0,763318	0,289866	0,36685	0,6279808
CG31173-RA	CG3117	0	0,0342033	536,867	12,8743	6,52809	5,15539	-0,266463	0,374069	0,13772387
CG31174-RA	Gr93c	0,13835	0,269801	0,0656243	0,465677	0,135843	0	-0,03424	0,916731	0,6279808
CG31176-RA	CG31174	0,0989651	0,0476284	0,0950124	23,4862	8,21954	0,129724	0,550245	0,014104	0,6279808
CG31178-RA	CG31176	17,7664	2,832	3,74968	14,6793	0,24756	5,58666	-0,792939	0,024395	0,6279808
CG31178-RB	Dsim\GD20903	0,0584397	0,282654	0,0561056	0,0719718	21,8515	0,0734715	-0,792939	0,024395	0,6279808
CG31179-RA	Dsim\GD20903	0,372374	79,6856	0,148959	3,79991	0,0503953	42,0556	-0,101943	0,770613	0,6279808
CG3117-RB	CG31179	0	0,267892	0,0941199	0,385973	0	0	-0,030953	0,86593	0,6279808
CG31183-RA	CG31183	1,31976	3,36105	1,40079	13,1726	0,0946936	0,0807964	-0,125434	0,682305	0,6279808
CG31184-RA	LSm3	21,3794	12,6984	148,664	7,36025	3,80443	36,3356	-0,510839	0,076429	0,6279808
CG31189-RA	CG31189	0	0	0,275812	7,83849	0,732459	0,172911	-0,167316	0,299882	0,6279808
CG31190-RA	CG3119	7,92793	7,77679	9,2841	7,47869	0,159088	21,6742	-0,702185	0,004132	0,13772387
CG31190-RB	CG3119	0,108916	0,0992082	0,104566	0,144019	14,5767	0	-0,701152	0,004067	0,6279808
CG31190-RC	Dscam3	0,376686	0,257299	22,9703	1,50845	0,718155	0,4947	-0,703123	0,004119	0,6279808
CG31190-RD	Dscam3	0,701146	0,439545	2,53708	0,0102947	1,13799	0,423259	-0,828238	0,006873	0,6279808
CG31191-RA	Dscam3	0,0102671	0,0166047	0,0914511	1,3404	0,0141972	0,0193538	0,504505	0,042188	0,6279808
CG31191-RB	Dscam3	0,0182295	18,0657	21,5355	0,0189588	0,0256798	15,0948	0,521737	0,035168	0,6279808
CG31193-RA	CG31191	3,80883	5,21169	3,97221	2,76489	1,91448	1,41139	-0,072587	0,832403	0,6279808
CG31195-RA	CG31191	0,0150904	0,0137454	0,0144877	1,3248	0,354828	0,769363	-0,346716	0,206445	0,6279808
CG31196-RA	TotX	0,76705	0,399247	0,473409	0,531721	0,891562	1,14679	-0,040894	0,828385	0,6279808
CG31196-RB	CG31195	16,8256	15,1995	14,976	35,6556	13,264	13,0503	-0,040894	0,828385	0,6279808
CG31196-RC	14-3-3epsilon	0,04625	0,0421277	38,7023	44,6933	0,0697532	0,0525702	-0,040929	0,828231	0,6279808
CG31196-RD	14-3-3epsilon	59,9039	56,8654	0,0444027	6,66499	53,2211	46,9149	-0,040894	0,828385	0,6279808
CG31198-RA	14-3-3epsilon	0,046445	0,0423053	97,9361	1,89858	0,0700818	26,2947	-0,397592	0,250074	0,6279808
CG31199-RA	14-3-3epsilon	164,362	166,394	0,04459	0,0370694	164,261	164,666	-0,223002	0,356027	0,6279808
CG3119-RA	CG31198	38,9278	20,1582	8,2638	7,44598	4,71537	3,64254	-0,161499	0,557713	0,13772387
CG3119-RB	CG31199	0	0,0891212	0,093934	0,286428	0,266059	0,124484	-0,468487	0,124014	0,6279808
CG31200-RA	CG31200	0,180535	0,164444	0,103995	21,4541	0	0,305118	-0,445805	0,178552	0,6279808
CG31201-RB	GluRIIE	8,18961	5,85557	5,79929	0,0162795	0,732079	3,79649	-0,104509	0,672	0,6279808
CG31202-RA	alpha-Man-Ic	0,242678	0,978642	12,424	8,27077	202,217	7,23557	0,115999	0,74264	0,6279808
CG31204-RA	CG31204	0,0417233	0,0380045	0,0400569	0,0529454	0,0717149	18,5937	0,429801	0,225307	0,6279808
CG31205-RB	CG31205	0,108085	0,0984512	0,103768	12,8874	15,4032	3,89881	-0,37551	0,1891	0,6279808
CG31206-RA	CG31206	0	0,0200221	0,0351723	24,3386	54,6225	46,7888	0,01562	0,897983	0,6279808
CG31207-RA	CG31207	0,196889	0,107604	0,113415	0,795615	0,337637	9,15066	0,145626	0,375579	0,6279808
CG31208-RA	Gr92a	0	0,0444509	0	0	0,0732155	0	NA	NA	0,6279808
CG31210-RA	CG3121	0,11577	0,0428475	0,111146	6,2166	1,84876	2,9834	-0,091622	0,584546	0,6279808
CG31211-RA	CG31210	0,172772	0,0959912	0,0162881	0	0	0,234402	-0,039213	0,838345	0,6279808
CG31211-RB	CG31211	19,5778	21,2908	19,8447	23,6074	15,35	16,2915	-0,039213	0,838345	0,6279808
CG31211-RC	CG31211	0,0222803	0,0202944	0,0213904	0,0233947	0,0316883	0,0238822	-0,040205	0,834157	0,6279808
CG31212-RA	CG31211	0,0225003	0,0204948	0,0216016	0,0236381	0,0320179	0,0241307	0,050184	0,86794	0,6279808
CG31213-RA	Ino80	2,16342	2,02805	3,38499	3,96414	1,12201	1,81387	0,023845	0,88794	0,6279808
CG31216-RA	CG31213	0	4,18452	4,95932	11,3229	0	8,06465	-0,162195	0,522635	0,6279808
CG31217-RA	Naam	4,1892	0,125099	0,0124394	0,0811235	7,95513	10,5325	-0,68496	0,003029	0,6279808
CG31219-RA	Dvir\GJ23926	7,16236	7,88156	10,1356	13,9756	0,0475365	12,9408	0,218536	0,537531	0,6279808
CG31219-RB	CG31219	0,485554	2,98266	0,00968993	32,8103	22,4441	21,4442	0,38305	0,217293	0,6279808
CG3121-RA	CG31219	0,0722402	27,5144	19,6093	4,73882	4,64522	0,00590792	0,049552	0,888247	0,6279808
CG31220-RA	CG31220	0,109761	0,0208546	0,0219809	12,4192	0,0332588	0,0250658	0,005662	0,980467	0,6279808
CG31221-RA	CG31221	42,8819	0	0,683341	0,0159177	26,798	0,0162494	0,473215	0,041742	0,6279808
CG31221-RB	CG31221	0,0154071	0	0,88735	0	0,0215606	0,0813344	0,473215	0,041742	0,6279808
CG31222-RA	CG42613	2,05321	1,94307	2,304	4,40646	3,74497	2,08427	-0,517362	0,073852	0,6279808
CG31223-RA	CG31223	3,15684	1,63604	4,78127	3,59256	3,49552	3,84348	-0,152918	0,632538	0,6279808
CG31224-RA	CG31224	10,1816	2,58754	0,858543	0,106027	3,94588	0	0,159934	0,505896	0,13772387
CG31225-RB	Ir94f	0,0591019	0,0622618	0,0518274	0	0,198106	1,77556	-0,009391	0,971314	0,6279808
CG31226-RA	CG31226	0,192266	0,0872268	0,0919374	11,0864	201,441	12,4237	0,021239	0,919006	0,6279808
CG31226-RB	CG31226	0,780419	8,55761	15,5273	9,79157	15,4134	8,60282	0,021239	0,919006	0,13772387
CG31229-RA	CG31229	25,4495	3,36996	6,75673	1,86992	0,0396226	0,61846	-0,205559	0,496777	0,6279808
CG31230-RA	CG3123	0	8,97597	4,89918	0,0894845	9,44464	0	0,284789	0,379187	0,6279808
CG31231-RA	CG31230	1,52207	73,9292	0,601704	5,29361	0	0	0,106419	0,694855	0,6279808
CG31232-RA	CG31231	0,0750862	0,0218172	16,1921	0,0252505	2,17302	11,9388	0,110197	0,615209	0,6279808
CG31232-RB	koko	0,0359564	0,0200044	0,0210847	6,86076	0,0399775	0,0252351	0,276842	0,267851	0,6279808
CG31232-RC	koko	15,5438	92,6821	105,435	0,0183516	14,6408	0,444296	0,288165	0,246427	0,6279808
CG31232-RD	koko	0,0554482	0,0327515	0,0345203	31,9087	0,034775	0,0238992	0,28728	0,247658	0,6279808
CG31232-RE	koko	0,0594385	20,5108	20,0297	0,0295145	5,7172	0,0252635	0,290156	0,244873	0,6279808
CG31232-RF	koko	5,04019	0,0505061	0,0532336	31,9817	2,13969	0,0137372	-0,19062	0,436686	0,6279808
CG31233-RA	koko	5,34096	0,0541407	0,0570645	0,0256735	0,0204071	0,0204109	-0,62141	0,068026	0,6279808
CG31235-RA	CG31233	49,4003	20,5114	96,8764	61,1896	11,2249	88,328	0,017315	0,936057	0,6279808
CG3123-RA	naz	11,1821	12,7896	4,79949	0,0661217	0,966631	8,80819	-0,092203	0,455035	0,6279808
CG31240-RA	CG3124	0	0	0	2,39949	0	0	0,382947	0,123198	0,6279808
CG31241-RB	repo	9,97975	7,60367	8,06723	8,50835	2,34589	2,68849	0,080379	0,725925	0,6279808
CG31243-RA	Tgs1	0,0335979	0,0306033	0,032256	3,56279	2,36877	2,29496	0,469581	0,071119	0,6279808
CG31243-RE	cpo	0,0268419	0,0244495	0,0257698	0,0284934	0,0385945	0,0290872	0,469663	0,071031	0,6279808
CG31243-RG	cpo	5,62987	6,18465	6,82365	5,74857	2,77948	2,94666	0,321109	0,229814	0,6279808
CG31243-RH	cpo	0,0131346	0,0119639	0,01261	0,0134982	0,0182834	0,0137795	0,220541	0,435405	0,6279808
CG31243-RJ	cpo	0,0131398	0,0119687	0,012615	0,0135037	0,0182909	0,0137851	0,489747	0,058452	0,6279808
CG31244-RA	cpo	0,0223405	0,0203493	0,0214482	0,0234613	0,0317785	0,0239502	0,204702	0,340425	0,6279808
CG31245-RA	CG31244	0,144649	0,131757	0,0833233	0,1052	0	0	0,170827	0,409946	0,6279808
CG31246-RA	CG31245	0,171181	0	0,361557	0	0,17557	0	-0,047725	0,892877	0,6279808
CG31247-RA	tinc	0,385764	0,627465	0,317449	1,91291	0,0289444	0,0218143	0,574916	0,08019	0,6279808
CG31247-RB	tinc	0,0193054	0,0175847	0,0185343	0,0201287	0,0272644	0,0205481	0,575315	0,079924	0,6279808
CG31247-RC	tinc	5,59389	6,74688	3,926	0,0112722	0,0152682	1,45539	-0,102736	0,761857	0,6279808

gene_id	Symbol	W1_FPKM	W2_FPKM	W3_FPKM	MCM51_FPKM	MCM52_FPKM	MCM53_FPKM	FC	p-value	p-adj
CG31247-RD	tinc	0,0101085	0,00920752	0,00970476	0,0103157	1,09339	0,0105306	0,593285	0,069973	0,6279808
CG31248-RA	tinc	11,9488	19,946	8,64216	19,6457	3,41834	5,55594	0,385648	0,19616	0,6279808
CG31249-RA	CG31248	7,86458	4,50021	9,87368	5,16067	0	0	0,163655	0,481876	0,6279808
CG3124-RA	CG31249	9,13585	8,1075	12,6347	11,087	7,06965	9,09446	NA	NA	0,6279808
CG31251-RA	IntS6	6,17129	11,1603	17,6659	0,439187	0,824791	3,90535	0,270064	0,369952	0,6279808
CG31253-RA	IntS6	1,28021	0,712018	6,71753	8,88565	2,94292	0,774563	NA	NA	0,13772387
CG31256-RA	CG31251	1,84102	2,3477	40,6305	2,40849	2,12816	2,01372	-0,030585	0,881783	0,13772387
CG31258-RA	CG31253	0,242926	0,12293	1,40024	6,71436	2,60624	0,214138	0,161595	0,610257	0,13772387
CG31259-RA	Brf	5,97353	5,90719	17,5221	15,186	0	0,0945365	0,150437	0,621673	0,6279808
CG3125-RA	Cenp-C	1,29713	1,15569	0,250244	0,312262	114,78	101,741	0,108899	0,665168	0,6279808
CG3125-RB	CG31259	19,7642	2,43111	3,73972	2,94195	2,61967	2,82769	0,114887	0,646894	0,6279808
CG31262-RB	CG31262	0,365488	3,42932	0,0300696	0	0	0,0343096	-0,54681	0,122262	0,6279808
CG31262-RD	CG31262	0,0313205	174,088	0	7,16858	0,288482	5,89067	-0,54681	0,122262	0,6279808
CG31265-RA	CG31265	5,41424	3,12004	20,1988	6,2898	2,18632	20,2989	-0,724153	0,042449	0,6279808
CG31266-RA	CG31266	0,47847	0,333277	1,4051	0,0299709	0	0,0305954	-0,947899	0,00797	0,13772387
CG31266-RB	CG31266	6,14885	4,16726	18,8694	10,7063	0	32,2596	-0,926147	0,009585	0,6279808
CG31267-RA	CG31267	4,95031	2,02582	6,81142	5,0067	5,99248	0	-0,394494	0,270205	0,6279808
CG31268-RA	CG31268	0,379746	7,73003	0,231002	1,36769	0,0328065	0,231629	0,02767	0,936954	0,6279808
CG31269-RB	CG31269	1,1283	0,3271	16,3719	1,18821	16,5965	2,83553	-0,408112	0,248552	0,6279808
CG31272-RA	Pgk	90,6948	0,0431238	130,533	119,042	183,251	175,766	-0,784819	0,004434	0,6279808
CG31274-RA	CG31272	4,09041	23,8134	24,7784	0,0215366	0,0291715	0,0219854	0,635876	0,040595	0,6279808
CG31274-RB	CG31274	0,0441486	12,8056	16,8072	12,7763	5,68522	14,0229	0,635876	0,040595	0,6279808
CG31275-RA	CG31274	8,4095	8,27374	10,4647	13,4653	5,66039	7,69602	0,076465	0,555158	0,6279808
CG31275-RB	CG31275	0,360631	0	22,681	81,2614	8,0869	4,57118	0,076465	0,555158	0,6279808
CG31278-RA	CG31275	0,109821	0,210811	0,0207808	0,035095	0,0192163	0,0766531	0,014345	0,957108	0,6279808
CG31279-RA	CG31278	4,22896	3,15145	0,13026	9,46929	0	0,0690357	0,001371	0,996243	0,6279808
CG3127-RA	CG31279	0,15695	21,1179	0	0,793617	3,23261	3,45612	-0,591478	0,039949	0,13772387
CG31280-RA	Gr94a	0,244726	0,0330211	0,0411748	4,52791	0,0126749	0,048339	-0,310052	0,384224	0,13772387
CG31281-RA	Tpl94D	0	0	0	0,178779	0	0	-0,034896	0,785626	0,6279808
CG31284-RA	wtrw	0,0178503	0,0272378	0,0171374	0,0185479	0,0251232	2,7143	-0,431769	0,072771	0,13772387
CG31284-RB	wtrw	2,27932	5,55449	3,31772	3,6321	3,83976	0,55216	-0,430177	0,073817	0,13772387
CG31284-RC	wtrw	0,529444	0,0317607	0,0172304	0,0186529	0,0252654	43,0392	-0,430177	0,073817	0,13772387
CG31286-RB	CG31286	0,0478233	0,0435607	0,0355853	0	0,115061	0	-0,6858	0,049693	0,6279808
CG31286-RC	CG31286	0,0370657	0,033762	20,3978	0,57229	0	0,475697	-0,708802	0,042995	0,6279808
CG31287-RA	CG31287	0,0850398	0,0774601	0,0160572	0,11469	38,5757	1,53564	0,157958	0,601555	0,6279808
CG31288-RA	CG31288	3,02308	46,7146	4,46706	44,9376	48,5153	6,22395	0,134646	0,704646	0,6279808
CG31289-RA	Dph5	19,9391	13,1123	36,2271	20,6446	2,09029	37,2259	-0,436486	0,181557	0,6279808
CG31291-RA	Rab18	90,7258	86,6397	0,053503	0,0635701	142,724	130,978	-0,107895	0,595379	0,6279808
CG31291-RB	CG45105	4,32979	6,10862	1,27328	2,15534	0	10,892	-0,114032	0,572589	0,6279808
CG31291-RC	CG45105	0,0197674	0,0180055	7,0006	12,5806	5,70751	3,96709	-0,109069	0,590362	0,6279808
CG31292-RA	CG45105	1,18512	0,0172718	0,0646214	0,0791472	3,53538	7,79514	-0,211017	0,531829	0,13772387
CG31293-RA	CR31292	3,26667	2,16401	3,92025	9,43286	0,0279474	0,078901	0,674155	0,015004	0,6279808
CG31294-RA	rec	2,48407	0	10,2807	0,0609814	0,0855284	0,0201662	-0,041811	0,731452	0,6279808
CG31296-RA	CG31294	0	0	385,384	202,598	0,115982	1,86368	-0,043909	0,891658	0,6279808
CG31298-RA	CG31296	0,195955	0,107093	0,301005	0,0810954	0	0	0,369769	0,141982	0,6279808
CG31299-RC	beat-Vb	5,42792	5,75167	5,15902	0,235641	4,23018	3,18812	-0,182286	0,477814	0,6279808
CG31299-RD	cu	11,0147	11,5687	0,0296341	13,6175	0,0448153	0,0337755	-0,191928	0,452379	0,6279808
CG31299-RE	cu	0,0308669	0,0281157	23,9661	0,0330861	25,08	24,7205	-0,204628	0,425878	0,6279808
CG3129-RA	cu	28,411	22,9625	262,459	21,5754	288,928	279,973	-0,428052	0,115339	0,6279808
CG31300-RA	CG31300	6,95536	6,43695	0,0414168	66,7622	4,83088	3,54826	-0,54564	0,02348	0,6279808
CG31301-RA	CG31301	18,7178	2,14611	3,08303	3,06271	1,82101	2,75949	0,03938	0,88107	0,13772387
CG31302-RA	Rbp	1,16343	4,54869	0,0659653	8,00323	0,0365853	0,0268359	-0,067063	0,796102	0,13772387
CG31302-RB	Rbp	0,0134968	0,0118113	1095,46	0,0133208	4,21464	0,0275729	-0,067063	0,796102	0,6279808
CG31302-RC	Rbp	1,9691	21,9943	0,04343	11,9205	0,0232453	6,0701	-0,066356	0,798308	0,6279808
CG31303-RA	CG42542	3,28419	2,81287	4,70599	0,0441775	3,03424	0,0450981	-0,639083	0,044501	0,6279808
CG31304-RA	CG42788	8,10725	267,036	23,2318	7,86723	0,0815432	5,33855	0,24536	0,302099	0,6279808
CG31313-RA	Duox	7,63273	0,0859807	0,241664	0,18785	2,63605	0,14702	-0,552263	0,015886	0,6279808
CG31315-RA	CG31313	20,1579	0,335114	26,6414	2,8711	0,0102299	0,124204	NA	NA	0,6279808
CG31317-RA	CG31315	0	0	0,0247349	2,67082	3,16309	0,790115	0,348268	0,242369	0,6279808
CG31317-RB	stumps	2,27938	0,013017	5,11398	8,75734	0,0233242	2,33323	0,366031	0,217297	0,6279808
CG31317-RC	stumps	4,27967	5,18501	12,1213	12,9104	5,79886	7,36106	0,335819	0,25944	0,6279808
CG3131-RB	stumps	13,5532	20,7776	0,0184463	0,0203588	0,0275761	2456,01	0,061231	0,829279	0,6279808
CG31320-RA	Ect3	29,1027	1,81012	4,23309	0	2,85837	6,21335	0,427508	0,125583	0,13772387
CG31321-RB	HEATR2	1,30824	0,099372	0,104738	1,49266	0,624854	0	-0,266985	0,447251	0,6279808
CG31322-RA	CG31321	5,13723	0,0174319	0,0183733	0,0874451	6,55891	6,55489	0,025967	0,918226	0,6279808
CG31323-RA	MetRS-m	4,20515	4,54146	6,71163	0,123006	4,2843	5,11271	-0,247739	0,31406	0,6279808
CG31324-RA	CG31323	6,16622	5,22247	14,7375	0	7,52222	0,0332414	0,178629	0,424792	0,6279808
CG31326-RA	CG31324	9,44537	144,361	0,0159082	6,96551	0,0441066	8,76274	0,29868	0,3669	0,6279808
CG31327-RA	CG31326	0,281413	0,141366	2,4585	0	1,18277	0,0432011	0,01562	0,897983	0,6279808
CG3132-RA	CG31327	0	0,416804	1,93298	0,0192468	30,5554	35,0587	1,814274	3,24E-11	0,6279808
CG31332-RA	Unc-115b	0,0230578	57,6514	7,39589	0,533673	7,44949	4,63235	-1,33119	4,64E-09	0,6279808
CG31332-RB	Unc-115b	0,0226081	0,0496724	0,0523549	0,056389	6,3812	6,83378	-1,33119	4,64E-09	0,6279808
CG31332-RC	Unc-115b	35,8247	0,0210026	0,0221368	31,5771	0,858963	87,887	-1,33119	4,64E-09	0,6279808
CG31332-RD	Unc-115b	12,3303	0,020593	15,5191	13,8529	0,586993	0,81296	-1,33119	4,64E-09	0,6279808
CG31335-RA	Gr93d	0,230631	0,151222	0,0796945	0,161546	0,135021	0,174268	0,056504	0,861754	0,6279808
CG31336-RB	Gr93b	0,222478	0,126655	0,0830325	0,453784	92,516	0,106937	0,325774	0,25673	0,6279808
CG31337-RA	CG31337	34,0766	0,107925	0	0,161293	19,2482	17,4674	1,043032	0,000206	0,6279808
CG31342-RB	ord	0,0746339	0,215275	0,0786407	0	0,248395	2,25337	0,414794	0,114836	0,6279808
CG31342-RC	CG31342	0,0166243	0,240987	1,59603	11,3114	0,362153	0,126672	0,415047	0,114677	0,13772387
CG31343-RA	CG31342	12,3636	11,8985	8,71982	0,053288	5,59523	12,9123	-0,456994	0,125974	0,13772387
CG31344-RA	CG31343	23,3656	14,8409	37,546	13,2411	38,4608	31,8454	0,576382	0,04465	0,6279808
CG31345-RA	CG31344	19,9888	95,041	22,4399	22,0398	4,93804	6,6591	-0,256779	0,391167	0,6279808
CG31347-RA	CG31345	18,9631	17,6184	0	21,2525	0	5,2956	-0,901722	0,010242	0,6279808
CG31349-RA	CG31347	1,12812	0,0225277	0,0237443	15,9477	33,9544	0,357342	0,269255	0,366705	0,13772387
CG31349-RB	pyd	5,69961	6,82226	0,0532635	11,5557	0,49182	41,73	0,274	0,351852	0,6279808
CG31349-RC	pyd	0,0132425	0,0120622	0,053669	0,0129874	4,37921	32,0102	0,280243	0,341678	0,6279808
CG31349-RE	pyd	0,0131791	0,0120045	59,2669	0,0136125	11,4174	1,23363	-0,237668	0,42461	0,6279808
CG31349-RF	pyd	20,9974	25,6483	40,9808	0,0135454	0,0566484	280,658	-0,162254	0,583538	0,6279808
CG31349-RG	pyd	6,88936	8,96472	5,7025	30,6267	98,7198	0,063486	0,28966	0,323106	0,6279808
CG31349-RH	pyd	0,0160547	0,0146237	0,0252971	17,7957	0,0459458	16,9185	-0,306578	0,308078	0,6279808
CG31349-RI	pyd	0,0175051	0,0159448	25,5705	0,016612	0,0362878	5,03195	0,269954	0,358972	0,6279808
CG3134-RA	pyd	0,0154107	0,0140371	0,0502669	0,0181744	4,70617	0,0285199	-0,240346	0,501129	0,6279808
CG31352-RA	shf	3,6098	3,20461	4,36282	3,05701	13,8389	4,00848	-0,206596	0,337913	0,6279808
CG31353-RA	Unc-115a	43,6902	4,21797	3,98154	5,35001	2,91761	2,01493	NA	NA	0,6279808
CG31357-RA	CG31353	0	0,0155133	0,0163511	0,0313245	0,0239245	0,0319772	0,024752	0,905411	0,6279808
CG31358-RA	CG31357	17,3926	19,2633	0,0138277	2,51108	13,839	0,0149626	1,354851	7,75E-05	0,6279808
CG31359-RA	CG31358	0,270486	9,0136	0,0798282	1,59815	286,263	7,89799	-1,18568	0,000468	0,6279808
CG3135-RA	Hsp70Bb	90,9404	113,474	92,6211	2,25492	1,91966	2,91934	-0,209751	0,47585	0,6279808
CG31360-RA	Atf6	0,0268092	0,0244197	0,0257385	0,0398734	0,0550753	0,0415081	0,139722	0,620901	0,13772387
CG31361-RA	Atf6	40,5362	33,9273	30,1275	0,0406608	0,0552116	0,0416108	0,03792	0,85194	0,6279808
CG31361-RB	Atf6	0,0168963	0,0153903	0,0162215	0,0407614	67,8086	63,7679	0,115021	0,572849	0,6279808
CG31361-RC	CG31360	9,58803	7,10862	9,90082	7,86059	9,62922	11,3047	0,11518	0,572151	0,6279808
CG31362-RA	dpr17	4,71741	4,35833	5,09281	5,14507	1,867	3,57474	-0,172005	NA	0,6279808
CG31363-RA	dpr17	0,0276044	0,025144	0,0265019	0,0293567	3,9433	0,0299684	0,216585	0,427895	0,6279808

gene_id	Symbol	W1_FPKM	W2_FPKM	W3_FPKM	MCM51_FPKM	MCM52_FPKM	MCM53_FPKM	FC	p-value	p-adj
CG31363-RB	dpr17	0,0264975	0,0241357	0,0254391	0,0281045	0,0397638	0,0286902	0,30575	0,262201	0,6279808
CG31363-RC	Jon99Ciii	9,91508	0,0947245	4,24314	68,8186	8,1063	2,5158	0,217898	0,422783	0,6279808
CG31363-RD	Jupiter	0,0432528	4,96285	4,67732	66,005	0,12012	2,93891	0,216651	0,427677	0,6279808
CG31363-RE	Jupiter	55,8274	0,078232	0,0125675	41,3651	5,09171	4,10261	0,216494	0,425541	0,6279808
CG31363-RH	Jupiter	32,1567	0,0714125	0,0154023	184,715	11,9718	12,6931	0,215781	0,429361	0,6279808
CG31365-RA	Jupiter	109,116	1,76357	0,0162714	0,0485025	0,671768	0,747198	0,312199	0,15703	0,6279808
CG31366-RA	Jupiter	0,0438265	0,114249	17,7387	0,0479728	13,2802	16,0553	-0,51987	0,107165	0,6279808
CG31367-RA	Jupiter	0,0433948	2,45553	0,0157229	4,06334	1,71647	2,43011	0,271027	0,334509	0,6279808
CG31368-RA	CG31365	7,27374	0,578716	8,51435	5,42678	2,78656	10,9112	-0,091298	0,744973	0,6279808
CG31368-RB	Hsp70Aa	150,115	0,0577789	5,73347	0,379638	3,20271	0,273445	-0,091298	0,744973	0,13772387
CG31369-RA	CG42724	15,1356	19,6758	290,043	23,0038	7,33679	9,75529	0,396728	0,119371	0,6279808
CG3136-RA	CG31368	3,21063	3,08417	0,0139217	0,0149498	0,121674	0,0917012	-0,130421	0,64097	0,6279808
CG3136-RB	CG31368	0,0143612	0,0132346	3,9616	5,91122	1,93824	3,05239	-0,129579	0,642837	0,6279808
CG3136-RC	CG45263	4,97415	3,84697	8,15304	8,25365	5,09596	0,691115	-0,260805	0,359866	0,6279808
CG31370-RB	CG31370	4,09175	0	0,0677744	4,923	0,111422	0	-0,080694	0,762115	0,6279808
CG31371-RA	CG31371	0,696452	4,45169	7,47194	12,3264	4,07945	0,411404	-0,544414	0,107487	0,6279808
CG31373-RA	CG31373	6,48878	0	0,0571846	5,28116	15,0525	12,1121	-0,464632	0,129932	0,6279808
CG31374-RA	sals	7,83023	9,07381	9,70594	0,0324666	0	7,54362	-0,140793	0,518626	0,6279808
CG31374-RB	sals	0,0193261	0,0176035	3,60879	4,85334	4,0576	0,0209123	-0,279507	0,185532	0,6279808
CG31374-RC	sals	9,9794	10,6565	10,9895	14,4494	3,99455	15,6128	-0,279058	0,186254	0,6279808
CG31380-RA	CG31380	0,0811743	0	0	0,156312	0	0,149858	-0,020285	0,93504	0,13772387
CG31381-RA	CG31381	1,79439	1,11528	2,79689	2,55724	0,424377	3,75123	-0,125218	0,686542	0,6279808
CG31386-RA	lncRNA:CR31386	0,869106	0,802487	0,662943	0,763134	0,649008	0,614109	0,090024	0,765854	0,6279808
CG31388-RA	CG31388	3,95339	0,068706	4,86715	0,0333879	0,0452241	0,0957476	0,2366	0,37316	0,6279808
CG31389-RA	CG31389	0,172372	0,0149173	0,0416616	0,046814	0,0229701	0,0477895	-0,011826	0,942873	0,6279808
CG31390-RA	Syt1	0,018913	0,0172273	0,0181576	0,0197014	0,0266856	0,0201119	-0,095377	0,680712	0,6279808
CG31391-RA	Syt1	32,8099	38,1295	20,3162	15,7362	19,9697	18,609	-0,499563	0,158238	0,13772387
CG31392-RA	Syt1	8,46853	0,0153354	0,0161636	19,6206	4,1467	0,017816	0,092538	0,722197	0,6279808
CG3139-RA	Syt1	0,0168103	7,19665	8,69888	0,0174246	7,64975	5,19464	0,19285	0,449497	0,6279808
CG3139-RB	MED7	12,0847	0,0187575	0,0197704	2,39491	2,36081	1,34656	0,19285	0,449497	0,6279808
CG3139-RC	CG31391	0,154744	0,158571	0,167134	0,238506	0,143237	0,0534899	0,208795	0,414269	0,6279808
CG3139-RD	CG42726	1,98961	12,9111	15,5746	23,3764	25,5599	11,9711	0,235837	0,363431	0,6279808
CG31404-RA	Adk2	93,3922	0,0180812	153,335	112,005	0,029048	0,0180503	-0,070526	0,562659	0,6279808
CG31405-RA	CG31404	0	0	0	0,233948	0	0	0,344889	0,233502	0,6279808
CG31406-RA	Gr85a	0,13835	2558,19	2448,51	7583,99	0	0,10176	-0,037166	0,868499	0,6279808
CG31406-RB	CG31406	0,104716	5,29959	0,109815	0,153765	2,78355	0,15697	-0,303693	0,337308	0,6279808
CG31407-RA	CG31406	0,187573	0,312566	0,28813	0,454889	0,141443	0,397217	-0,013096	0,914398	0,6279808
CG3140-RA	CG31407	0	10,2935	6,08604	5,00796	0,00629123	14,0814	-0,488676	0,107566	0,6279808
CG31410-RB	Npc2e	12,8372	12,1679	23,8357	0,552409	26,5237	13,2539	0,324155	0,289929	0,6279808
CG31413-RA	Qsox4	0,0555882	5,79726	5,06255	2,70823	9,65139	4,01887	0,063688	0,754971	0,6279808
CG31414-RB	Gba1b	4,97765	9,21615	10,8519	121,984	3,44966	8,30644	0,248103	0,419359	0,6279808
CG31415-RA	CG31415	0,839945	17,0402	13,773	0,0284934	9,69293	0,057564	0,023706	0,947182	0,6279808
CG31418-RA	CG31418	0	0,0378948	0	0,607533	5,68707	0,0746786	NA	NA	0,6279808
CG31419-RA	CG31419	0	0	218,885	15,2037	0,180379	10,137	NA	NA	0,6279808
CG31421-RA	Takl1	4,31689	2,31836	7,04321	2,91453	8,88378	7,78661	-0,430502	0,217364	0,6279808
CG31423-RA	Ir94c	0,0308478	0,0280983	35,5097	0,0339732	2,66256	22,1925	-0,293515	0,410294	0,13772387
CG31424-RA	Ir94b	0	0	0	0,109532	2,93251	1,48567	-0,037875	0,76416	0,6279808
CG31426-RA	eIF2D	0,036859	18,4416	0,0293191	0,0233037	0,031565	0,0343622	-0,182731	0,391601	0,13772387
CG31427-RB	CR31427	0	0,175187	0,0615492	6,98803	2,28708	0,0685311	0,106881	0,661299	0,6279808
CG31427-RC	CR31427	0	0,307479	2,36731	3,20442	2,23243	0,20889	0,074371	0,764354	0,6279808
CG31431-RA	foxo	0,0159078	4,44593	11,226	0,149184	3,80688	57,9436	-0,055469	0,802266	0,6279808
CG31436-RA	foxo	0,0184948	24,4736	0,0152725	3,65298	0	58,7912	-0,235755	0,403911	0,6279808
CG31437-RA	foxo	45,9528	0,026829	0,0177561	17,0961	0	2,56426	-0,270412	0,397548	0,6279808
CG31438-RA	foxo	0,0171151	0,01449	37,7549	10,0657	3,41974	16,3227	0,119425	0,594695	0,6279808
CG31439-RA	foxo	0,0159078	0,0168464	0,0164316	0	11,7711	0	-0,051824	0,882958	0,6279808
CG3143-RA	CG31431	22,972	21,5142	54,9152	0	30,9046	24,2519	0,331066	0,170896	0,6279808
CG3143-RB	CG31436	4,43576	4,08456	6,74859	0,592747	18,4948	49,4746	0,328045	0,17545	0,6279808
CG3143-RC	CG31437	0,45541	0,305256	0,0297313	0,0188503	0,0255329	0,0192431	0,331161	0,171302	0,6279808
CG3143-RD	CheB93b	0,161951	0,147516	0	0,143081	1,46269	0,15402	0,331161	0,171302	0,6279808
CG3143-RE	Muc96D	0,387066	10,7012	9,49331	6,37012	3,27889	1,58505	0,331066	0,170896	0,6279808
CG31441-RA	CG31441	2,94692	2,83202	3,29643	3,02271	0,300028	5,37457	0,110814	0,674683	0,6279808
CG31445-RA	CG31445	0,544462	0,0532309	0,336633	0,0747477	158,68	185,131	-0,132673	0,690511	0,6279808
CG31446-RA	CG31446	31,9747	21,0301	24,9741	18,629	12,8485	15,5333	-0,823641	0,012926	0,6279808
CG31447-RA	MESK4	8,21133	19,9504	0,304253	12,3654	21,4406	8,13118	0,502672	0,142121	0,6279808
CG31449-RA	Hsp70Ba	72,4966	90,6445	71,0108	298,916	138,757	58,4988	2,41395	1,28E-11	0,6279808
CG31450-RA	mRpS18A	52,1682	11,6813	14,1296	5,9101	0,618161	15,0783	-0,484563	0,088672	0,6279808
CG31451-RA	lncRNA:CR31451	122,631	138,724	112,84	35,3732	31,6673	27,9218	0,929397	4,33E-05	0,6279808
CG31453-RA	pch2	4,38235	0	0,366687	84,0237	2,79499	18,9445	-0,190714	0,48708	0,6279808
CG31454-RA	CG31454	2,2943	0,725934	5,05454	85,4913	8,20266	7,79343	-0,519114	0,14631	0,6279808
CG31457-RB	CG31457	4,53052	4,78275	0,0088221	0,206658	5,9664	0,0366262	-0,193929	0,38257	0,6279808
CG31459-RA	CG31459	0	0	0	0	0	0,12357	-0,041811	0,731452	0,6279808
CG31460-RA	CG31460	13,9846	38,78	74,8271	1,84795	0,422961	0,855554	0,3145	0,217546	0,6279808
CG31461-RA	CG31461	0	1,82117	0,0241879	0,0272785	1,46753	1,29087	NA	NA	0,6279808
CG31462-RA	CG45263	0,585668	5,62357	4,56284	14,5603	0,540175	0,513471	-0,575639	0,070973	0,6279808
CG31463-RA	CG31463	13,5931	0,029364	30,4762	0,0346703	13,8327	3,65973	0,143499	0,592082	0,6279808
CG31464-RA	CG31464	2,01979	12,3096	2,44698	17,3927	5,80704	11,5216	-0,638648	0,050657	0,13772387
CG31465-RA	CG31465	0,0577788	8,38898	0,0665654	12,6996	0,459955	5,76611	0,171365	0,43651	0,6279808
CG31467-RA	CG31467	1,69375	0,0427865	1,42051	0	0,0709735	19,3727	0,400751	0,179794	0,6279808
CG31468-RA	Dsim\GD18362	0,209101	2,08306	0	1,62576	0,49306	15,8658	-0,014388	0,910154	0,6279808
CG31469-RA	CG31469	0,562566	1,02485	0,687396	1,69491	1,65583	1,0356	-0,251505	0,478779	0,6279808
CG31472-RA	sgll	0,0562507	7,06219	0,054004	0,0642519	0,0870296	0,0655908	0,249395	0,40574	0,6279808
CG31472-RB	sgll	10,2522	0,0172916	16,7353	7,15307	11,8275	9,01942	0,249395	0,40574	0,6279808
CG31473-RA	CG31473	0,0710747	0,0587949	0,0682359	0,0844201	0,114348	0,0861793	0,479493	0,157372	0,6279808
CG31475-RA	CG31475	10,6193	12,3933	12,0714	13,431	0	0,637125	0,163191	0,434635	0,6279808
CG31477-RA	ATPsynepsilonL	5,35971	3,30473	7,04557	9,21272	0,346577	0,261201	-0,509367	0,115805	0,6279808
CG31477-RB	ATPsynepsilonL	0,162481	0,147999	0,155991	0,255869	7,44583	10,6734	-0,509367	0,115805	0,6279808
CG31478-RA	mRpL9	37,9355	32,6262	66,3855	43,5729	74,2769	73,5694	-0,338222	0,273042	0,6279808
CG31481-RA	pb	1,71136	0,461873	0,0173151	0,0187484	0,981892	0,0191391	0,176653	0,549758	0,6279808
CG31481-RB	pb	0,0180354	0,0164279	0,0173151	0,0187484	0,0253948	0,0191391	0,176653	0,549758	0,13772387
CG31481-RC	pb	0,0180354	2,11099	0,0172445	0,0186687	0,0253948	0,0190577	0,176653	0,549758	0,6279808
CG31481-RD	pb	0,0179619	0,0163609	4,99721	6,58105	0,0252869	3,43878	0,176653	0,549758	0,6279808
CG31482-RA	CG31482	0,302176	8,95187	6,03661	0	0,0215711	45,7909	0,219637	0,244398	0,6279808
CG31483-RA	Pif2	0,111741	0	0	0,0403372	0,054637	0,0545749	0,01562	0,897983	0,6279808
CG31493-RA	CG3149	6,78332	15,3917	6,04455	4,46213	10,7597	13,4527	0,01649	0,931484	0,6279808
CG31495-RA	CG31493	0	0,121835	0	0,183535	0	6,1148	0,507679	0,150906	0,6279808
CG31496-RA	CG31495	0,70252	0,604353	0	0,721451	14,1036	0	0,427473	0,19406	0,6279808
CG31498-RA	CG31496	6,07735	0	0	0	0	0	0,421595	0,171614	0,6279808
CG3149-RA	CG31498	0,237683	9,44486	3,15443	12,0875	0,0662333	23,8141	-0,325176	0,197785	0,13772387
CG31501-RA	nxf4	0	0	0	0	0	0	NA	NA	0,13772387
CG31507-RA	TotZ	0	0,138466	0	19,5187	0,189413	0,0274491	0,01562	0,897983	0,6279808
CG31508-RA	TotC	1,53522	0,196789	0,0244019	0,293778	0,421704	0,639219	0,377032	0,146747	0,6279808
CG31509-RA	TotA	1,47087	0	1,19487	0	0	0	0,480663	0,15138	0,13772387
CG31510-RA	Rbp9	0,0189873	6,89373	0,018229	9,21804	0,0777093	8,61836	0,173092	0,53675	0,6279808
CG31515-RA	Rbp9	0,0189058	8,50118	0,0181507	1,71167	23,471	0,819267	0,01562	0,897983	0,6279808

gene_id	Symbol	W1_FPKM	W2_FPKM	W3_FPKM	MCM51_FPKM	MCM52_FPKM	MCM53_FPKM	FC	p-value	p-adj
CG31516-RA	Rbp9	0,0194227	0,71279	0,0186469	0,0201041	28,8734	9,35977	0,321042	0,323907	0,6279808
CG31517-RA	Rbp9	0,0193374	0,00755574	0,0185651	0,0200125	13,7393	7,7579	-0,047753	0,886079	0,6279808
CG31519-RA	Rbp9	1,75126	2,74842	1,75022	0,0205942	0,0451326	1,61423	0,563379	0,082401	0,6279808
CG3151-RB	Rbp9	5,85813	0,0103401	6,73969	0,0204981	37,0076	3,54821	-0,3094	0,109665	0,6279808
CG3151-RC	Rbp9	0,019281	0,009898	0,0277663	1,7269	0,0468134	0,020523	-0,316186	0,099851	0,6279808
CG3151-RE	CG31510	6,55386	7,03332	6,30789	9,80336	0,0974863	3,10616	-0,3094	0,109665	0,6279808
CG3151-RF	CG31515	0	0,201968	0	0,0688513	0,0932596	0	-0,316186	0,099851	0,6279808
CG3151-RG	CG31516	1,55914	3,55942	1,74319	2,34335	49,7458	1,65048	-0,241529	0,202587	0,6279808
CG3151-RH	CG31517	0,202936	0,184848	0,207989	2,80922	0,0487038	0,0367061	-0,297748	0,127083	0,6279808
CG3151-RI	Or82a	0,0855905	129,849	0,520423	0,166618	0,090931	393,844	-0,297748	0,127083	0,6279808
CG31522-RA	Trap1	23,0519	20,1715	31,3472	6,14586	1,58033	2,02395	0,744379	0,005555	0,6279808
CG31522-RB	CG31522	108,221	11,0646	113,687	7,78885	5,15106	12,8411	0,761864	0,00436	0,6279808
CG31522-RC	CG31522	33,8873	0,0109616	0,0805374	0,0123358	0,0167089	68,5388	0,02981	0,914249	0,6279808
CG31522-RD	CG31522	133,982	4,04508	2,44452	11,4464	3,75471	3,01291	0,758548	0,004573	0,6279808
CG31523-RA	CG31522	0,0838879	0,328888	45,6771	0,285228	0,386344	37,5984	0,527218	0,009084	0,6279808
CG31523-RB	CG31523	85,3311	26,7286	20,8938	0,0245359	0	3,52501	0,535148	0,007615	0,6279808
CG31523-RC	CG31523	20,3334	38,2601	61,6524	0,0438541	14,6964	12,2403	0,532066	0,00788	0,6279808
CG31523-RD	CG31523	0,027353	86,0321	76,8367	86,7051	5,09429	7,1524	0,536483	0,00748	0,6279808
CG31524-RA	CG31523	0,0233099	17,2525	16,3903	119,559	15,5997	18,4654	NA	NA	0,6279808
CG31524-RB	CG31524	0	0	0	14,2705	13,8715	15,9788	NA	NA	0,6279808
CG31525-RA	CG31524	0	0	0,540216	0,0392091	0,19255	0,145117	-0,391306	0,25555	0,6279808
CG31526-RA	CG31525	0,224748	26,8739	11,4221	0	0	0,0871846	-0,070526	0,562659	0,6279808
CG31526-RB	lncRNA:CR31526	0	0	0	1,96978	0,033234	0,0162181	-0,070526	0,562659	0,6279808
CG31528-RA	lncRNA:CR31526	0	0	0	14,1355	2,38302	0,229087	-0,497784	0,163188	0,13772387
CG3152-RA	CG31528	0,213665	0,013145	0,0170958	0,0182449	0,0201491	30,6722	-0,109222	0,614415	0,6279808
CG31530-RA	Npc2b	53,9372	64,042	0,604911	0,749092	0,831565	0	-0,159166	0,54299	0,6279808
CG31531-RA	Npc2b	39,3558	36,9343	38,2744	0,988905	0,546588	0,502715	-0,069249	0,799002	0,6279808
CG31531-RB	CG44098	3,4629	0,687904	3,92661	1,34724	0	2,81987	-0,084094	0,756984	0,13772387
CG31531-RC	smash	8,61396	11,7447	5,39499	199,844	0,0221602	6,45733	-0,073319	0,787095	0,6279808
CG31533-RA	smash	0,0120342	9,75387	0,0115536	84,5442	28,6821	6,27777	-0,041811	0,731452	0,13772387
CG31534-RA	smash	3,33919	2,65076	4,25262	13,8791	7,92236	0,0169838	0,130276	0,622546	0,6279808
CG31534-RB	CG31533	0	0,034911	0	0,0177533	50,5725	46,1494	0,130418	0,621965	0,6279808
CG31534-RC	smash	9,90195	106,747	10,1485	13,5617	186,032	4,80015	0,128738	0,626315	0,6279808
CG31534-RD	smash	10,8816	62,3142	6,76398	14,6019	6,30227	0,0125928	0,127287	0,63023	0,6279808
CG31536-RC	smash	0,0160782	131,071	0,015436	0,0166371	5,80039	3,59246	-0,383535	0,171313	0,6279808
CG31536-RE	smash	0,0158202	0,0764109	0,0151884	0,0163604	0,0225351	0	0,091874	0,740879	0,6279808
CG31538-RA	Cdep	19,042	46,4483	16,9579	65,4231	21,7633	0,0290244	-0,41838	0,232757	0,6279808
CG3153-RA	Cdep	0,0267875	0,526632	0,0257176	30,6655	0,191383	0,599864	-0,119574	0,628024	0,6279808
CG3153-RB	CG31538	0,305341	0,0115996	4,02438	13,9779	1,21392	3,01626	-0,11839	0,630805	0,6279808
CG31542-RA	CG31542	0,197595	0,0719934	65,3537	0,0614363	6,38894	60,1717	-0,153222	0,551449	0,6279808
CG31543-RA	Hph	9,862	16,096	7,33498	29,7909	40,9957	9,6683	0,225107	0,525881	0,6279808
CG31543-RB	Hph	41,5717	110,461	15,4353	116,032	37,2033	13,4396	0,252181	0,476608	0,6279808
CG31543-RC	Hph	24,4424	56,7177	8,95881	0,0419286	0,088147	8,77106	0,216412	0,53898	0,13772387
CG31544-RA	CR46300	0	0	0	0	0	0,0739241	NA	NA	0,13772387
CG31546-RA	CG31546	0,0697983	17,2215	53,6799	37,3667	0,11191	0,0843423	-0,089247	0,79752	0,6279808
CG31547-RA	NKCC	6,29455	9,04387	0,0123981	10,4044	12,599	0,0135409	-0,940978	0,001814	0,6279808
CG31547-RB	NKCC	2,04891	12,8875	0,012226	10,2966	0,022976	8,21843	-0,943406	0,001803	0,6279808
CG31548-RA	CG31548	28,7074	13,8289	62,1581	38,1562	52,9867	56,63	-0,281179	0,332338	0,6279808
CG31549-RA	CG31549	22,3956	23,9947	28,5466	18,8405	27,9387	22,4224	0,027041	0,920458	0,6279808
CG31550-RA	CG31550	7,31081	4,78308	10,6135	9,02873	0,052826	12,3425	0,212921	0,360545	0,13772387
CG31550-RB	CG31550	7,07243	7,34414	0,0157268	3,01898	58,1847	0,0173161	0,076602	0,740778	0,13772387
CG31550-RC	CG31550	8,79457	4,84287	0,0148124	8,92392	0,0528573	0,016273	0,077453	0,736467	0,13772387
CG31551-RA	CG31551	0,0632003	0,0359795	0,0834295	0,101759	0	0,0253541	0,09403	0,745081	0,6279808
CG31554-RA	CG42675	0	0,226606	0,0281858	7,36922	0	0	-0,124117	0,60393	0,6279808
CG31555-RA	CG11000	6,13241	6,14148	11,0823	15,5172	2,92956	12,558	0,474841	0,046156	0,6279808
CG31556-RA	CG46026	1,49071	11,4848	0,876594	13,0228	1,89926	4,64087	0,648803	0,067933	0,6279808
CG31557-RA	Obp83ef	8,49188	12,701	1,13931	17,1029	1,09661	0,104364	-0,242078	0,359571	0,6279808
CG31558-RA	Obp83g	126,48	47,2355	3,53549	49,7273	9,72584	20,7609	0,469238	0,056267	0,6279808
CG31559-RA	CG31559	29,4792	0,0423947	0,0209109	0,0518619	0,0702473	0,0529426	0,319547	0,324703	0,6279808
CG31560-RA	Dsim\GD16508	9,48058	8,38621	1,34078	1,67055	1,75144	1,76208	-0,195261	0,303032	0,6279808
CG31561-RA	CG31560	0	1,10325	0	9,17249	3,79559	0	0,689132	0,013502	0,6279808
CG31562-RA	Osi16	7,69701	8,08958	10,7244	0,850459	0,0852431	0,233399	0,01562	0,897983	0,13772387
CG31563-RA	CR31562	0	2,66076	0,0222611	10,6148	198,061	4,84189	-0,272567	0,434849	0,6279808
CG3156-RA	CG31563	1,51899	0,0501693	236,181	0,0627229	0,0849585	0,0640299	-0,111421	0,622984	0,6279808
CG3157-RA	gammaTub23C	3,49457	4,77464	4,3189	10,8809	8,25384	0,00860545	0,048029	0,858712	0,6279808
CG3158-RA	spn-E	0,531612	0,671465	0,0256034	0,920204	0,267752	0	-0,545827	0,045718	0,6279808
CG3159-RA	Eaat2	2,99203	3,2875	40,3947	28,4422	0,209409	1,54534	0,103595	0,69286	0,6279808
CG3159-RB	Eaat2	3,84972	4,43039	0,796964	0,372416	20,3891	0,227549	0,074356	0,773523	0,6279808
CG31600-RA	PGAP1	2,47216	0,180334	0,142555	0,188045	0,118211	0,143506	0,01562	0,897983	0,6279808
CG31601-RA	CG31600	0	40,1195	18,943	0	0,0173689	0	0,056609	0,851887	0,6279808
CG31605-RA	CG31601	0,0916194	0,0834533	0,281472	0	1,64398	0	-0,025738	0,90763	0,6279808
CG31605-RB	Bsg	69,4811	0,0219926	5,83073	3,76404	26,2357	36,6912	-0,026947	0,903382	0,6279808
CG31605-RC	Bsg	78,5931	0,0197873	12,4951	0,0266281	51,8327	0,0232547	-0,008684	0,968635	0,6279808
CG31605-RD	Bsg	0,0219327	0,0254524	0,0799286	0,0263406	8,46182	0,0303607	-0,009383	0,966133	0,6279808
CG31605-RE	Bsg	0,0235677	5,22782	58,6615	5,15154	23,4925	0,013859	-0,027636	0,900976	0,13772387
CG31605-RF	Bsg	38,1224	6,77424	48,6667	0,0135761	10,2566	8,14714	-0,009623	0,965253	0,13772387
CG31605-RG	Bsg	0,0238226	0,0758333	68,2696	19,9649	122,347	14,0845	0,012044	0,954282	0,6279808
CG31605-RH	Bsg	34,1767	21,8675	0,0210567	26,3989	0,138691	0,0182214	-0,009709	0,964962	0,13772387
CG31605-RI	Bsg	0,0217236	9,44675	0,0226264	0,0178495	8,17014	0,0194097	-0,026389	0,90541	0,13772387
CG31606-RA	Bsg	0,027943	27,3268	51,9331	0,0190135	4,24912	0,0209448	-0,011134	0,96862	0,6279808
CG31606-RB	CG31606	0,488846	6,5334	8,90341	23,7126	10,8036	8,32948	0,05269	0,859551	0,6279808
CG31607-RA	CG31606	0,167846	33,7373	2,04748	0,56519	0,227959	1,89449	0,12232	0,567045	0,6279808
CG31609-RB	CG43394	18,5707	2,20292	0,0440635	0,645738	1,30059	0,0521221	NA	NA	0,6279808
CG3160-RA	CG31609	0	0,0531369	0,0180543	0,0311248	28,6349	1,78627	-0,202134	0,417761	0,6279808
CG31611-RA	Vha16-1	0,0225926	0,0418349	14,7827	0,0510978	0,0328427	0,0529843	NA	NA	0,6279808
CG31612-RA	Vha16-1	556,732	0,0457807	2,47448	0,0565341	11,8242	3,66053	-0,068135	0,828899	0,6279808
CG31613-RA	Vha16-1	0,0237968	5,23633	0,0216902	8,79753	3,32242	1,85515	NA	NA	0,13772387
CG31617-RA	Vha16-1	124,429	0,0352036	647,076	6,59441	7,45601	8,4201	NA	NA	0,6279808
CG31618-RA	His4:CG31611	1,20451	1,02883	0,881067	3,45957	1,92576	0,812947	NA	NA	0,6279808
CG31619-RA	CG31612	0,609224	0,509438	0,469831	0,0128231	0,503069	0,379143	0,195793	0,358175	0,6279808
CG31619-RB	His3:CG31613	0,847127	4,89439	2,60489	2,24231	1,0125	3,56687	-0,331443	0,125184	0,6279808
CG31619-RC	His1:CG31617	0,846183	0,435648	2,26055	0,941435	2,60377	0,639559	0,189882	0,372641	0,6279808
CG3161-RA	His2A:CG31618	0,812884	1,05776	2,17402	7,09818	4,96179	1,7599	-0,099387	0,627099	0,6279808
CG3161-RB	nolo	0,0187148	0,0170468	9,16261	0,815779	0	0,0198919	-0,099464	0,62682	0,6279808
CG3161-RC	nolo	4,64235	0,0113824	5,21472	0	0	1,165	-0,099832	0,625538	0,6279808
CG3161-RD	nolo	6,37474	10,8734	9,20028	0,0194858	0	5,63306	-0,099553	0,626518	0,6279808
CG31620-RA	LS2	0	0	0,056	0	861,362	0,161311	-0,113341	0,728831	0,6279808
CG31622-RA	Gr39b	0,238111	0,216888	0,142876	0,821466	9,73333	0,453007	-0,63791	0,072495	0,6279808
CG31622-RB	Gr39a	0,059049	0,0566371	0,0596957	0,0721294	3,85008	0,0736324	-0,667165	0,059821	0,13772387
CG31622-RC	Gr39a	0,0576578	0,00624816	1,0005	8,11657	0,0973799	0,0070928	-0,728029	0,041941	0,6279808
CG31622-RD	Gr39a	0,0576578	649,028	0,00658559	607,24	0,0945932	486,645	-0,76125	0,033035	0,6279808
CG31623-RA	Gr39a	0,0621792	0,0455727	637,949	0,0562446	0,0945932	0,0574166	-0,134717	0,638011	0,6279808
CG31624-RA	dtr	0,672956	0,587434	0,538398	0,17543	0,317858	0,239557	-0,013952	0,911565	0,6279808
CG31626-RA	Dsim\GD21644	0,233649	0,212824	0,13459	0,163253	7,1975	8,57933	0,290081	0,162291	0,6279808

gene_id	Symbol	W1_FPKM	W2_FPKM	W3_FPKM	MCM51_FPKM	MCM52_FPKM	MCM53_FPKM	FC	p-value	p-adj
CG31626-RB	CG31626	511,731	0,016594	0,0174902	0,0192411	358,825	0,0196421	0,290113	0,162338	0,6279808
CG31627-RB	CG31626	0,0500321	23,1444	22,5681	12,1301	0,0761836	12,7177	0,25622	0,451278	0,6279808
CG31628-RA	CG31627	1,77333	2,21222	1,55446	3,17368	1,55568	0,845632	-0,502743	0,012223	0,6279808
CG31628-RB	Gart	8,10299	10,162	20,5736	16,996	8,38338	11,6894	-0,836102	0,000156	0,6279808
CG3162-RA	Gart	0,0427214	0,0389136	13,6597	0,047149	11,3415	17,9065	-0,03026	0,851331	0,13772387
CG31630-RA	CG3163	14,4992	99,5392	22,678	6,31724	181,517	277,14	-0,512284	0,132468	0,6279808
CG31632-RA	milt	2,38026	0,0281157	20,6395	6,76857	14,0837	13,2466	0,45202	0,082851	0,6279808
CG31633-RA	sens-2	9,94765	0	0,139116	0,272153	2,45975	0	-0,18836	0,463355	0,6279808
CG31634-RA	CG31633	6,54785	0,0308701	0,0325372	0,0365959	0,0495694	0,0373585	0,225167	0,384931	0,6279808
CG31635-RA	Oatp26F	4,06763	4,26338	3,73131	4,98214	1,29638	556,82	-0,453075	0,026402	0,6279808
CG31635-RB	CG31635	4,9973	51,7719	32,1639	38,6242	32,0824	27,9283	-0,415937	0,04479	0,6279808
CG31636-RA	CG31635	0,0097837	6,07987	1,61622	9,69608	7,75699	5,28538	0,237647	0,447232	0,13772387
CG31637-RA	CG31636	1,47303	12,4352	4,18842	21,1096	10,1685	5,20206	0,178191	0,437576	0,6279808
CG31638-RA	CG31637	17,7223	21,7858	15,2608	1,83355	1,51602	1,45389	0,169408	0,451818	0,13772387
CG31638-RB	CG31638	4,93429	4,70156	2,36219	4,10631	0,463737	13,2112	0,213082	0,361066	0,6279808
CG31639-RA	CG31638	5,29347	6,10484	6,38329	7,46747	2,17383	2,42445	-0,093098	0,64001	0,6279808
CG31639-RB	CG31639	0	14,7918	0,370492	0	0	0,355002	-0,093098	0,64001	0,6279808
CG3163-RA	CG31639	0	0,0300082	0,120449	0	0	0,178175	0,212492	0,354529	0,6279808
CG31641-RA	CG3164	86,8404	0,0221708	92,26	11,3085	0,0403754	8,76092	0,464231	0,042688	0,6279808
CG31641-RB	CG3164	25,0296	15,7884	32,6286	0,205577	116,012	76,438	0,45902	0,046442	0,6279808
CG31641-RC	CG3164	0,0235929	1,75956	0,0226506	26,4425	0,278455	23,4613	0,44632	0,056268	0,13772387
CG31642-RA	CG3164	0,0243403	1,59307	0,0233682	0,0298082	19,5307	0,0253684	-0,37564	0,277275	0,6279808
CG31643-RA	stai	19,2316	0	0,0316288	0,035492	3,07446	0,0362316	-0,012916	0,952282	0,6279808
CG31644-RA	stai	0,0329446	0	32,5156	33,4889	13,1859	17,9533	-0,073806	0,645207	0,6279808
CG31646-RA	stai	32,6915	27,4772	0,146168	0,498574	0,164787	0,0928433	0,022942	0,923009	0,6279808
CG31647-RA	CG31642	0,152249	22,4424	0	0	0	0	-0,029574	0,84188	0,6279808
CG31647-RB	CG31643	3,14019	0,0142498	5,76743	0,0244812	6,33862	4,99273	-0,006415	0,962915	0,6279808
CG31648-RA	CG31644	0	4,96175	8,68784	21,5308	0	0,447388	-0,440102	0,178139	0,6279808
CG3164-RA	DIP-theta	3,59831	0	3,72963	0,108197	0,0454544	0	-0,101261	0,607859	0,6279808
CG3164-RB	lncRNA:CR31647	0	19,4018	0,695208	7,8719	0,0319035	11,3534	-0,124758	0,529102	0,6279808
CG3164-RC	lncRNA:CR31647	0	7,38662	5,29546	65,676	13,0427	0,194821	-0,101211	0,607843	0,6279808
CG3164-RD	CG31648	7,05703	18,7778	22,8064	25,0601	0,0660372	17,5685	-0,12469	0,529114	0,13772387
CG31650-RA	CG3165	6,48736	16,6243	22,656	12,5105	3,56948	12,3282	-0,113386	0,577914	0,13772387
CG31650-RB	CG31650	31,2033	24,4524	21,4226	37,4719	23,0376	18,909	-0,119604	0,556594	0,6279808
CG31650-RC	CG31650	0,0415397	0,0378372	0,0362565	0,0457098	7,54579	0,0420268	-0,114697	0,573183	0,13772387
CG31651-RA	CG31650	0,0412287	0,0375539	0,0241787	0,0453325	0,0614031	0,0271829	0,350355	0,160653	0,6279808
CG31651-RB	Pgant5	5,83921	69,3877	32,9701	108,435	4,86854	11,4146	0,344842	0,165468	0,6279808
CG31658-RA	Pgant5	22,8601	7,15666	16,1813	8,94409	9,56434	14,3736	0,187861	0,597707	0,6279808
CG31659-RA	Nnf1b	1,02068	0,216791	2,68188	4,43606	0,934479	6,7716	0,613029	0,068027	0,6279808
CG3165-RA	CG31659	0,455696	1,08958	0,71093	0,69832	0	0,430504	0,33959	0,144314	0,13772387
CG31660-RB	aop	12,9084	32,4526	11,4355	5,35634	86,7843	3,12554	-0,564336	0,028663	0,6279808
CG31660-RC	aop	12,2539	2,65528	9,21032	0,0794334	2,07169	45,3244	-0,562098	0,029948	0,13772387
CG31661-RA	smog	0,778336	0,779079	0	34,8346	5,30572	1,53644	0,069916	0,632492	0,13772387
CG31662-RA	smog	1,4807	2,01884	0	7,07922	39,5418	8,08174	-0,064503	0,680926	0,13772387
CG31663-RA	CG31661	0	0,127298	0,428968	1,09377	0	0,0956805	-0,138996	0,526371	0,6279808
CG31664-RA	Gr22a	0	13,3774	0	0,314314	9,03191	3,91604	-0,228136	0,514172	0,6279808
CG31665-RA	CG31663	4,79729	162,689	5,9787	0,0119596	5,33037	4,58092	0,4778	0,042205	0,6279808
CG31665-RB	CG31664	3,05527	1,55762	0	8,40292	6,13069	5,17562	0,4778	0,042205	0,6279808
CG31666-RA	wry	0,0116769	4,57582	0,0112106	5,55903	0,0161994	5,93106	0,974711	3,38E-07	0,6279808
CG31666-RB	wry	13,0088	0,0828254	9,96612	0,179383	7,23221	1,59312	0,982529	2,72E-07	0,6279808
CG31666-RC	Dpse\chinmo	100,363	103,372	92,9004	36,9059	28,8316	34,7801	0,972033	3,63E-07	0,6279808
CG31666-RD	Dpse\chinmo	0,0132266	0,0120477	0,0126984	13,923	4,69612	0,013879	1,136909	3,49E-09	0,6279808
CG31668-RB	Dpse\chinmo	0,0132187	0,0120405	0,0126907	0,0135873	0,018404	0,0138704	-0,355172	0,264114	0,6279808
CG3166-RA	Dpse\chinmo	0,013594	0,0123823	0,013051	0,0139852	0,0189431	0,0142766	0,403725	0,117481	0,6279808
CG3166-RB	CG31668	1,30132	0,0198591	0,0209316	0,022867	11,7477	12,1888	0,372382	0,144455	0,13772387
CG31670-RA	MAN1	0,0303146	0,0276127	0,0291039	0,0214455	2,08254	0,035006	0,440237	0,142952	0,6279808
CG31671-RA	MAN1	7,56732	6,85164	11,7659	0,0207238	281,614	20,1429	-0,161199	0,547991	0,6279808
CG31672-RA	erm	1,182	0,372288	0,0294644	0,0333372	0,0457574	0,0344855	0,024658	0,944257	0,6279808
CG31673-RA	tho2	5,83023	8,53078	9,59826	12,1648	6,23102	8,05139	-0,261326	0,355204	0,6279808
CG31674-RA	Kebab	0,354749	0,365277	105,37	4,97599	0,0467643	131,902	-0,48063	0,173642	0,6279808
CG31675-RA	CG31673	10,1788	10,093	0,112877	12,9064	28,1248	28,5654	-0,38448	0,166353	0,6279808
CG31676-RA	CG31674	3,5444	1,05838	8,89208	3,70041	0,03548	0,0267399	1,737125	1,44E-12	0,6279808
CG31677-RA	CG31675	16,0445	228,185	161,872	77,2291	0,0793678	32,7929	0,01562	0,897983	0,6279808
CG31678-RB	CG31676	39,8206	49,7948	43,3844	12,0979	0,179329	16,231	0,104034	0,690887	0,6279808
CG31678-RC	CG31677	0	0	0	0	0	1,20477	0,132804	0,611122	0,6279808
CG31679-RA	CG31678	8,38037	9,73745	0,0267929	0,175751	0,562953	0,71502	-0,041811	0,731452	0,6279808
CG3167-RA	CG31678	0,013382	1,51756	7,1605	4,10389	4,82112	3,39438	0,078074	0,742093	0,6279808
CG3167-RB	Tengl3	0	0	0	0	0,146871	0,110691	0,078866	0,739476	0,6279808
CG31680-RA	CG3168	12,1961	6,62196	0,0199132	4,45106	0,0331846	5,97786	-0,204746	0,246519	0,6279808
CG31681-RA	CG3168	6,04091	4,83326	26,3139	0,0252505	0,0342019	14,5909	NA	NA	0,6279808
CG31682-RA	CG3168	7,64486	8,03802	5,3508	12,7911	10,5426	2,8246	-0,080649	0,53498	0,6279808
CG31683-RA	CG31680	0	0,337028	1,08113	1,58566	10,3554	1,22025	0,101052	0,691662	0,6279808
CG31686-RA	CG31681	0	0	0	0	0	0	-0,582413	0,099553	0,6279808
CG31687-RA	Tengl1	0	0	0,0994307	0	0	0	-0,111079	0,658839	0,6279808
CG31688-RB	CG31683	8,19471	0,0227205	5,64108	54,6756	21,2255	66,5073	-0,751811	0,002772	0,6279808
CG31688-RC	CG4267	0,790652	0	0	0,181513	0	16,1054	-0,753618	0,002715	0,6279808
CG31689-RA	CG31687	1,86407	16,5758	0,019533	0,0514972	4,28696	0,0525702	0,107599	0,610952	0,6279808
CG31689-RB	CG31688	5,19092	7,83309	6,3067	2,90712	6,4129	5,7418	0,111466	0,598876	0,6279808
CG31689-RC	CG31688	0,0203456	4,005	0,0129728	8,19006	2,29972	0,0217087	0,10774	0,610975	0,6279808
CG31689-RD	CG31689	3,36744	94,7495	0,0761546	0,104504	0,141551	2,8426	0,10833	0,608759	0,6279808
CG3168-RA	CG31689	0,0246398	5,35242	0,0233825	0,0262462	0,0355507	0,0265604	-0,149444	0,570336	0,6279808
CG3168-RB	CG31689	0,0250509	0,0722527	10,4836	10,7125	3,91253	0,02703	-0,147394	0,575312	0,6279808
CG3168-RC	CG31689	16,4299	0,0221845	0,0681789	0,0528605	0,0715998	14,0935	-0,120012	0,64521	0,6279808
CG31690-RA	Spt3	6,51034	32,9281	33,0687	0,141293	3,8105	1,93974	0,009177	0,969516	0,6279808
CG31690-RB	CG31690	3,66609	0,0228181	0,0240503	0,0264782	0,035865	0,0267931	0,009177	0,969516	0,13772387
CG31691-RA	CG31690	0,0180848	18,8734	20,7434	14,7491	13,7039	5,78575	-1,794434	4,96E-08	0,13772387
CG31692-RA	TotF	1,51484	0,985587	6,86233	8,14659	3,23226	3,2293	-0,417881	0,140394	0,6279808
CG31692-RB	fbp	90,2032	0,0521245	5,76358	0,0817163	5,21609	0,292249	-0,419645	0,138236	0,6279808
CG31693-RA	fbp	0,057225	0,393376	7,84802	4,23662	0,343841	5,33014	-0,041811	0,731452	0,6279808
CG31693-RB	CG31693	0	0	0,506684	0	0	0	-0,041811	0,731452	0,6279808
CG31694-RA	CG31693	0	0	0	0	0,0836453	0	-0,052682	0,772752	0,6279808
CG31695-RA	CG31694	58,4356	56,0357	62,4501	67,7032	47,3917	54,1161	0,067432	0,675386	0,6279808
CG31697-RA	scw	0,11699	0	0,0673904	10,6093	0	0	0,531935	0,117878	0,6279808
CG31698-RA	CG31697	1,80207	0,134947	14,16	151,236	0,0163268	20,6773	-0,489218	0,114602	0,6279808
CG3169-RA	CG31698	0,202273	0	0,194194	261,848	0,750522	1,15032	0,212668	0,356916	0,6279808
CG31700-RA	CR31700	0	0	0	0	0	0	NA	NA	0,6279808
CG31702-RA	CG31702	0	0	0	0	0,908841	0	NA	NA	0,6279808
CG31703-RA	CG31703	0	0	0,088847	1,18498	0,0932343	1,56693	NA	NA	0,6279808
CG31704-RA	CG31704	0,513012	1,12149	2,75812	6,50632	10,1826	5,24557	-0,695711	0,051587	0,6279808
CG31705-RA	CG31705	14,7408	13,746	0,0237354	71,1246	0,0353675	0,0266551	-1,416932	4,57E-08	0,13772387
CG31705-RB	CG31705	4,93936	3,95612	0	24,3965	0	0	-1,415599	4,68E-08	0,13772387
CG31705-RC	CG31705	0,0247228	0,0225192	0	0,026111	0	0	-1,418187	4,47E-08	0,6279808
CG31706-RA	CG31706	8,0927	0	0,0732526	32,3788	0,0738512	0,0556587	-0,67123	0,022061	0,6279808
CG31708-RA	DIP-zeta	0,761618	0	0,0469485	234,187	0,309669	0	0,091117	0,720386	0,6279808

gene_id	Symbol	W1_FPKM	W2_FPKM	W3_FPKM	MCM51_FPKM	MCM52_FPKM	MCM53_FPKM	FC	p-value	p-adj
CG31708-RB	DIP-zeta	3,30801	9,08549	0	0	204,061	0,785599	0,081691	0,75008	0,6279808
CG31709-RA	CG31709	0,0713818	0,764935	3,77469	1,23617	0,0173926	0,0131081	0,180377	0,483747	0,6279808
CG31710-RA	Tre1	21,6189	2,09652	3,00088	0,0648846	0	2,31787	0,881785	0,000846	0,6279808
CG31712-RA	CG31710	34,8	0,0289822	31,4012	11,6875	0,306631	631,374	0,084483	0,67625	0,6279808
CG31713-RA	CG31712	17,6406	9,24292	234,603	217,798	66,5606	11,212	-0,277305	0,354745	0,6279808
CG31714-RB	Apf	11,6223	129,023	94,6532	65,2978	4,31901	5,49696	-0,370049	0,143181	0,6279808
CG31715-RA	CG44153	1,06282	1,48303	8,38938	6,74451	8,33319	8,0995	-0,274648	0,368016	0,6279808
CG31716-RA	CG31715	74,4067	49,4278	29,8112	20,2156	18,3064	55,7002	-0,015955	0,93768	0,6279808
CG31716-RB	Cnot4	0,0192267	0,017513	0,0184588	0,020043	0,0271483	0,0204606	-0,015523	0,939277	0,6279808
CG31716-RC	Cnot4	0,0188322	0,0171537	0,01808	0,0196134	0,0265665	0,0200221	-0,015955	0,93768	0,6279808
CG31716-RD	Cnot4	5,03482	5,02444	4,81596	11,2785	5,48054	5,53526	-0,015846	0,938048	0,6279808
CG31716-RE	Cnot4	0,0188429	0,0171634	0,0180903	0,0196251	0,0265823	0,020034	-0,015547	0,93923	0,6279808
CG31716-RG	Cnot4	3,74256	7,76242	8,66512	5,24478	3,12318	3,95181	-0,015661	0,938714	0,6279808
CG31717-RA	Cnot4	21,1484	16,6798	18,2208	24,7051	13,0114	13,8113	-0,279676	0,29245	0,6279808
CG31718-RA	CG31717	13,2402	10,7666	18,4857	19,1016	29,6906	0,0480284	-0,03418	0,907132	0,6279808
CG31719-RA	Ir31a	0,122363	0,0928804	0,0978963	0,150238	35,8692	0,0711785	0,108743	0,6584	0,13772387
CG31719-RB	RluA-1	26,1752	23,5036	13,152	19,1517	20,0715	12,9215	0,108514	0,65901	0,6279808
CG31719-RC	RluA-1	0,0209787	0,0191088	0,039582	0,0219601	0,0297451	0,0224177	0,10841	0,659345	0,6279808
CG3171-RA	RluA-1	0,0218096	0,0198657	5,5156	0,0228749	0,0309842	6,16054	0,314106	0,146432	0,13772387
CG31720-RA	twf	13,4633	7,73585	16,124	5,2723	0	2,88816	-0,116594	0,696952	0,6279808
CG31720-RB	mthl15	0,0391754	0,0356836	0,0376107	0,0428558	0,0580484	0,0437488	-0,067434	0,820781	0,6279808
CG31721-RA	mthl15	4,66811	2,51059	4,52755	3,54485	4,54951	3,37249	0,086578	0,76539	0,6279808
CG31728-RB	Trim9	13,7153	0,0961958	0,0506954	134,343	9,0815	9,63433	-0,481721	0,144218	0,6279808
CG31729-RA	l(2)k05911	1,24706	18,2512	0	0,0125831	0,0170439	10,4296	-0,152559	0,416146	0,13772387
CG31729-RB	CG31729	0,0132319	0,0120526	27,0743	0,0136013	14,6646	17,53	-0,155511	0,408563	0,6279808
CG3172-RA	CG31729	23,3659	22,8742	7,00369	28,411	91,5902	5,98805	-0,038589	0,87167	0,6279808
CG31730-RA	IntS1	3,62167	4,34311	3,50242	76,0792	17,4255	1,35258	0,074904	0,676357	0,6279808
CG31731-RA	CG31730	0	0,0608763	27,721	0,0125759	0,0170439	26,2243	0,100869	0,703019	0,6279808
CG31731-RB	Eato	11,5539	1,58017	3,39433	1,6404	35,9051	6,19407	0,093237	0,722972	0,6279808
CG31732-RB	Eato	2,64506	7,30131	6,3319	0,796773	0,150122	3,14939	-0,1387	0,558557	0,6279808
CG31732-RC	yuri	0,0329282	0,0896967	0,025949	11,838	0	0,305977	0,006344	0,979251	0,6279808
CG31732-RD	yuri	0,0270286	0	14,2019	0,0287045	0	0	0,006344	0,979251	0,6279808
CG31732-RE	yuri	0,0296748	19,4613	0	3,10521	0	0	0,078999	0,750189	0,6279808
CG31732-RF	yuri	0,0198446	0,0299932	2,23486	91,9728	0,127865	0,0538245	0,031975	0,896586	0,6279808
CG31732-RG	yuri	8,08995	0,0246195	72,3108	63,7837	4,3402	6,44554	0,076445	0,757398	0,6279808
CG31732-RH	yuri	5,73615	0,0270298	44,4376	0,039116	0,0480483	0,0362121	0,173712	0,491731	0,6279808
CG31733-RA	yuri	0,0270286	0,0180758	0,0345956	0,0378792	0,0388804	0,0293026	NA	NA	0,6279808
CG31733-RB	ms(2)35Ci	0	0	0	0,289249	7,43816	141,158	NA	NA	0,6279808
CG31735-RA	ms(2)35Ci	0	0	0	0	0,289256	0,397857	NA	NA	0,6279808
CG31735-RB	CG31735	0	2,24996	2,70504	0	3,94423	0	NA	NA	0,6279808
CG31736-RA	CG31735	0	3,6339	0	4,08991	16,1722	9,91941	NA	NA	0,13772387
CG31739-RA	CG31736	0	0	10,8939	0	0	0	0,00743	0,979537	0,13772387
CG3173-RA	AspRS-m	8,68747	0,460846	11,5704	0,450151	0,0510435	34,0254	0,07885	0,778317	0,6279808
CG31740-RA	Fmo-2	31,6329	18,4121	35,6495	40,0069	23,194	2,14423	-0,84063	0,006761	0,13772387
CG31741-RA	CG31740	0	0	0,0816082	0,283023	1,68238	0,114458	-0,633848	0,021675	0,6279808
CG31742-RA	CG31741	0	0	0	1,43001	3,75986	3,02176	-0,070526	0,562659	0,6279808
CG31744-RA	Prosbeta5R2	0	0	0	0	0	0	0,097512	0,666804	0,13772387
CG31747-RA	Gr36b	0,0836404	0	0,160599	0,255823	0,0295579	0	-0,062968	0,745795	0,13772387
CG31748-RA	Gr36a	0,0842805	0	0,0809143	0,163537	0,297225	0,103641	-0,201609	0,250533	0,13772387
CG3174-RA	Gr36c	0	0,0732196	0	0,162039	3,12857	0,0979088	0,176425	0,384425	0,6279808
CG31750-RA	CG31750	0,0803843	0	0,0771737	0,10184	0	0	-0,073338	0,779129	0,6279808
CG31751-RA	CG31751	7,48511	10,3586	22,1508	15,7049	7,64132	26,8313	0,384263	0,066768	0,6279808
CG31751-RB	CG31751	42,948	0,0477671	73,4295	61,1442	0,0843962	13,5573	0,392292	0,061149	0,6279808
CG31752-RA	CG31752	0,0922848	58,3868	1,3216	0,0388164	57,8553	0,0394857	-0,03823	0,82214	0,6279808
CG31753-RA	ham	10,6353	15,1687	33,9408	2295,61	22,8772	24,9179	0,350482	0,186837	0,6279808
CG31755-RA	SoYb	0,266077	2,34918	0,610242	0,380535	0,289973	0,257232	0,098617	0,770211	0,6279808
CG31759-RC	CG31759	0,263563	1,47127	2,46638	0,0866487	1,02714	300,054	0,461798	0,097986	0,6279808
CG31759-RD	CG31759	1,646	2,11061	1,4113	3,00444	454,798	2,16171	0,46575	0,095081	0,6279808
CG31760-RA	CG3176	1,38815	1,82077	2,93195	2,84234	8,30168	14,1252	-0,31748	0,190541	0,6279808
CG31761-RA	CG31760	2,63694	0,134746	2,6121	0,0288679	74,8521	2,24595	0,390821	0,110124	0,6279808
CG31761-RC	bru2	10,5201	13,9214	0,0698898	0,167473	38,5784	0,0413042	0,397761	0,104796	0,6279808
CG31761-RD	bru2	0,0170709	0,0155494	5,48219	13,0713	0,0570467	50,4806	0,400538	0,102815	0,6279808
CG31761-RE	bru2	10,2759	13,0506	0	0	0,0548048	0	0,386157	0,113581	0,6279808
CG31762-RA	bru2	3,27278	0,0154061	14,8498	12,0496	68,4052	13,5496	0,116967	0,576062	0,6279808
CG31762-RB	bru1	4,61153	0,0231962	0,0244489	0,0265529	0,0364956	0,0271062	0,137074	0,51524	0,6279808
CG31762-RC	bru1	0,025466	0,0228788	0,0241143	2,34201	1,1723	1,9495	0,116967	0,576062	0,6279808
CG31762-RD	bru1	0,0251175	2,38568	3,53023	0,0295145	1,75504	0,0301295	0,19762	0,323755	0,6279808
CG31763-RA	bru1	2,72234	0,0252707	0,0266354	1,71402	0,0399775	1,18362	0,077097	0,782202	0,6279808
CG31764-RA	bru2	6,36286	6,76169	25,3101	19,459	16,5928	16,9166	-0,38205	0,199585	0,6279808
CG31764-RB	vir-1	20,5101	15,7198	29,1284	24,3801	4,53124	4,01854	-0,381768	0,199978	0,6279808
CG31764-RC	vir-1	3,83654	5,63699	7,10748	0,0421162	0,0239823	0,0180745	-0,568671	0,066109	0,6279808
CG31764-RD	vir-1	4,3303	0,033857	8,29685	7,67971	5,04483	6,30181	-0,037483	0,893083	0,6279808
CG31769-RA	vir-1	22,5166	15,4976	42,4568	39,9277	2,29324	3,22339	0,427978	0,227113	0,6279808
CG3176-RA	CG31769	4,35665	1,48813	11,4442	83,2108	5,70125	0,0373585	0,116396	0,599897	0,6279808
CG31770-RB	He	5,92236	4,35172	43,0786	18,1393	0,356686	21,6963	-0,213562	0,502334	0,6279808
CG31771-RA	CG31771	0,45401	46,2952	51,4757	0,747776	0,0751268	35,1797	0,09162	0,797899	0,6279808
CG31772-RA	CG43707	1,35506	18,6822	0,0140171	0,0150557	3762,09	0,0384475	-0,541395	0,01393	0,6279808
CG31773-RA	CG31773	0	0	0,261413	98,6814	233,717	201,386	-0,18879	0,333289	0,6279808
CG31774-RA	fred	0,644243	0,321355	0,331115	0,135887	0,464739	0	-0,527473	0,074017	0,6279808
CG31774-RB	fred	0,0153285	0,321131	0,0380773	196,139	0,0582952	378,166	-0,527473	0,074017	0,6279808
CG31775-RA	CG31775	801,513	0	0	0	0	0	-0,455941	0,131594	0,6279808
CG31776-RA	CG31776	0,218238	0,198787	5,39462	0,377153	0,202257	0,0920545	-0,130292	0,709829	0,6279808
CG31777-RA	CG31777	145,094	133,713	156,63	0,329402	0,852306	0	-0,238629	0,400035	0,13772387
CG31778-RA	CG31778	8,34016	1,09309	7,85384	6,89736	2,06921	3,28703	-0,17909	0,579175	0,6279808
CG31779-RB	Acp24A4	0	0,338126	0,649207	2,17377	1,53417	8,65452	-0,304237	0,245186	0,6279808
CG31780-RB	Rrp1	0,917146	0,587038	0,0237977	18,4607	4,0094	0,0267292	NA	NA	0,6279808
CG31781-RB	Rrp1	3,36174	3,62097	6,92126	8,131	1,00164	6,50761	-0,22377	0,27358	0,6279808
CG31782-RA	CG31780	0,118416	0,107861	0,10752	0,0690676	0	0	0,192906	0,331745	0,6279808
CG31782-RB	lncRNA:CR31781	36,7897	36,0703	39,6206	10,3886	33,0319	9,57117	0,196279	0,324683	0,6279808
CG31783-RA	CR43671	0,021012	0,0191392	0,0201728	0,0219967	0,0297947	0,0224551	-2,089095	2,9E-15	0,6279808
CG31784-RA	CR43671	1,69047	2,17383	0,0173577	2,63204	0,02546	0,0191882	-0,318935	0,326485	0,6279808
CG31784-RB	ninaD	1,34067	1,16884	1,69165	0	0,430706	6,06897	-0,318935	0,326485	0,6279808
CG31785-RB	CG31784	0	0,0851517	0,02002	0,0217355	1,65217	1,24518	-0,036187	0,91727	0,13772387
CG31786-RB	CG31784	0	0,0850714	4,07017	0,358888	0	0,0963256	-0,184247	0,565808	0,13772387
CG31787-RA	CG31785	0,346855	0,0380285	0,444002	0,00684623	0,517129	5,44993	NA	NA	0,13772387
CG31788-RA	CG42750	0,648177	1,14608	0,951735	0,381784	1,43718	0	-0,041811	0,731452	0,13772387
CG31789-RA	CG31787	0	0	0	0,00685692	2,19008	0,0232146	0,146049	0,664236	0,13772387
CG3178-RA	CG31788	0	0	0	0,00685692	8,66816	0,0206999	-0,518317	0,058616	0,6279808
CG3178-RB	CG31789	29,2894	9,66456	19,4681	1,21071	26,4972	21,2813	-0,520384	0,057816	0,6279808
CG31790-RA	CG43338	3,97411	8,55068	7,45858	79,2304	28,9664	2,77159	0,486504	0,026481	0,6279808
CG31791-RA	Mst36Fb	0,260483	50,1697	35,4031	4,87195	2,04995	29,5664	-0,414811	0,214821	0,6279808
CG31792-RA	CG31792	0,968512	0,395463	2,00394	0,0208814	1,85496	0,0213165	-0,377863	0,266399	0,6279808
CG31793-RA	CG31793	1,65152	101,887	53,9711	51,5774	70,0187	66,5131	-0,51232	0,077403	0,6279808
CG31794-RA	Pax	0,0298176	14,6616	14,2243	119,186	15,5181	30,5473	-0,744751	0,011864	0,13772387

gene_id	Symbol	W1_FPKM	W2_FPKM	W3_FPKM	MCM51_FPKM	MCM52_FPKM	MCM53_FPKM	FC	p-value	p-adj
CG31794-RB	Pax	0,0323426	4,97636	46,62	113,883	31,9269	27,5862	-0,74161	0,012317	0,6279808
CG31794-RC	Pax	0,0256108	3,82242	19,9783	0,0102149	40,5611	21,6804	-0,73914	0,012457	0,13772387
CG31794-RD	Pax	0,027036	32,0214	0,0286267	0,0102149	4,68989	8,11474	-0,863732	0,006889	0,6279808
CG31794-RF	Pax	0,0307091	0,0347163	50,7525	0,00950884	8,34541	7,94528	-0,735799	0,012661	0,6279808
CG31794-RG	Pax	256,661	9,03326	0,0245879	0,00950884	5,02756	23,013	-0,7343	0,012807	0,6279808
CG31795-RA	IA-2	26,1907	26,5506	26,5529	19,5495	14,9615	24,5982	-0,353945	0,137512	0,13772387
CG31795-RB	IA-2	7,19849	9,77048	9,56721	13,1503	10,4773	9,36383	0,378474	0,077268	0,6279808
CG31797-RA	CG31797	0,0633639	0	0,0608331	0,0129925	11,3703	0,00970698	0,021313	0,920621	0,6279808
CG31798-RA	CG31798	0,103513	2,78002	0,198758	3,11806	62,9141	0,186024	-0,335245	0,310998	0,6279808
CG31800-RA	RpII140	11,1819	0,0155897	12,0178	36,8651	13,8856	0	-0,17795	0,486806	0,6279808
CG31801-RA	CG31800	30,2106	23,4641	38,4039	43,7249	45,6499	36,8224	-0,178791	0,592526	0,13772387
CG31802-RA	Mst36Fa	0,817644	3,84488	5,02614	4,80626	1,89523	2,2137	NA	NA	0,6279808
CG31803-RA	CG31802	0	0	0	0,0226158	0	9,90885	0,280521	0,316491	0,6279808
CG31804-RA	CG31803	0,177815	0,194359	0,0228876	0,219136	11,518	1,62224	NA	NA	0,6279808
CG31805-RB	CG31804	0	3,9708	0,0394834	0,0452081	0,0612346	1,69692	-0,213434	0,377132	0,6279808
CG31806-RA	CG31805	0	0,476268	0	109,175	0,11891	0,055636	0,105981	0,668718	0,6279808
CG31807-RA	CG31806	0	0,950658	0,621929	3,4683	3,97113	0,0222766	0,173791	0,62649	0,6279808
CG31809-RB	CG31807	0,649622	0,887582	1,381	0,704733	1,19611	0,719418	-1,001673	0,003094	0,6279808
CG3180-RA	CG31809	4,25999	12,5303	6,81513	39,0032	21,6383	14,1554	-0,14919	0,545426	0,6279808
CG31810-RA	Ts	4,14702	0,00837125	6,71858	8,7315	8,96604	0,0204295	-0,844473	0,016281	0,6279808
CG31811-RA	CG31810	8,95747	4,07954	9,93992	11,1663	0,202214	0,152401	0,127467	0,609175	0,6279808
CG31811-RB	CenG1A	2,30252	2,70729	2,75959	2,36178	6,57181	6,97601	0,137572	0,579416	0,6279808
CG31811-RC	CenG1A	4,77759	3,188	3,09254	8,76234	40,7692	24,5073	0,194207	0,437311	0,6279808
CG31812-RB	CenG1A	16,2519	21,6371	11,5041	4,40905	5,75905	5,39334	-0,244007	0,413502	0,6279808
CG31813-RA	CG31812	0,101864	3,38664	4,303	0,13191	0,178673	0,134658	0,263036	0,303923	0,6279808
CG31814-RA	nur	1605,17	1978,1	2425,15	2386,44	4448,94	4636,06	-0,222574	0,418015	0,6279808
CG31815-RA	DIP-kappa	3,29431	5,40017	1,53136	0,463456	0	14,9323	-0,734862	0,039529	0,6279808
CG31816-RA	CG31815	0,198277	0,338634	0,380716	1,09031	0,539081	0,601269	-0,09221	0,750585	0,6279808
CG31817-RA	CG31816	0,324538	0,354734	0,0121255	0	0,046838	1,30157	-0,283607	0,428021	0,6279808
CG31819-RA	CG31817	0,135065	1,41062	0,092622	0,332058	0,0765215	0,0691057	0,044335	0,715924	0,13772387
CG3181-RA	CG31819	0,0585433	0,0533253	0	0	0	0	-0,460513	0,070067	0,6279808
CG31820-RA	sei	1,30061	2,50223	0,0230344	0,026194	0,0349794	0,0263626	0,025482	0,880577	0,6279808
CG31821-RA	sei	1,19963	0,0218542	0,0305621	2,35102	0,0463274	0,0349151	-0,512909	0,149012	0,6279808
CG31822-RA	CG31820	0	0,195835	0	0,132503	0	0	NA	NA	0,6279808
CG31823-RA	CG31821	4,96598	0	0	0	0,195865	0	-0,243644	0,416154	0,6279808
CG31824-RA	CR31822	0	0	0	2,70029	1,43275	0	NA	NA	0,6279808
CG31826-RA	CG31823	2,16928	0	0	3,104	0	3,30477	0,059686	0,865382	0,6279808
CG31827-RA	CR31824	0	0	0	0	0	1,52494	NA	NA	0,6279808
CG31828-RA	CG31826	1,33439	1,52578	0	0	0,238306	0	NA	NA	0,6279808
CG31829-RA	CG31827	0,074662	0	0,113686	0,230315	0	0	-0,062722	0,796749	0,13772387
CG3182-RA	CG31828	0	0,0477923	0,0839555	0,0197605	5,72956	6,3985	0,470819	0,051824	0,6279808
CG3182-RB	CG42615	0,217117	0,0186876	14,8372	0	0	2,0097	0,470819	0,051824	0,6279808
CG31832-RA	geminin	22,5003	39,2163	5,26235	38,8049	27,1387	27,2751	-0,469472	0,135028	0,6279808
CG31835-RA	CG31832	13,9753	15,2777	38,9722	23,0577	0,0494595	17,5913	NA	NA	0,6279808
CG31839-RA	CG31835	0,0809092	0	0	236,847	426,504	242,468	-0,36461	0,158952	0,6279808
CG3183-RA	NimB2	56,477	72,9589	83,7364	15,4679	25,7358	41,8198	-0,148974	0,632615	0,6279808
CG31840-RA	CG44303	2,8631	3,29824	4,47345	59,0637	5,19364	4,85985	0,025482	0,880577	0,6279808
CG31842-RA	lncRNA:CR31840	0	0,15512	0,163497	35,283	101,769	0	0,090433	0,725896	0,6279808
CG31845-RA	mRpS23	22,3377	21,7966	36,8292	0,0403125	0,0546036	2404,75	-0,640491	0,056672	0,13772387
CG31846-RA	asRNA:CR31845	0,0810082	0,0737879	0,0777727	0,0988592	3,79179	0,294994	-0,269308	0,451567	0,13772387
CG31847-RA	CG42784	0,302954	0,575947	0,260238	0,264339	0,512805	0,296056	0,28846	0,236635	0,6279808
CG31848-RA	CG42784	8,3302	1086,49	112,73	0	0	0	-0,013096	0,914398	0,6279808
CG31849-RA	CG31848	0	0,0161459	39,5244	53,937	2,89648	4,656	0,036612	0,903535	0,6279808
CG3184-RA	CG31849	1,05264	38,097	1,37153	0,0335408	13,3136	0,0128453	-0,38544	0,171026	0,6279808
CG31851-RA	Naa20B	0	0,0277741	2,32509	14,5887	0,0170439	18,3266	0,020909	0,891561	0,6279808
CG31852-RA	Tap42	4,20393	3,17601	21,2503	20,0006	19,699	18,3272	-0,165445	0,570065	0,6279808
CG31855-RA	CG31855	0,0606942	0,0552845	10,7675	10,6449	11,3364	4,2735	-0,153717	0,531688	0,6279808
CG31855-RB	CG31855	23,0407	25,0027	0,0170178	20,4268	0,0249407	7,5724	-0,153717	0,531688	0,6279808
CG31856-RA	CG31856	0,402456	0,0111753	0,963965	0	0,0569962	1,70669	0,422284	0,225828	0,6279808
CG31858-RA	t-cup	0	0	0	0	0	0	NA	NA	0,6279808
CG31860-RA	eEF5	408,115	517,842	0,0881472	343,955	408,926	375,535	0,337467	0,290184	0,6279808
CG31862-RA	eEF5	865,771	857,468	1807,86	1708,85	1800,95	1713,2	0,008953	0,966822	0,6279808
CG31864-RA	ZnT33D	0,213678	0,12461	0,0946818	0,0312058	39,0228	0,0558207	-0,268461	0,29526	0,6279808
CG31865-RA	CG31862	0,300892	0	0,288874	0,285228	0	0,469017	0,780972	0,008256	0,6279808
CG31866-RA	Qtzl	10,7825	35,5813	6,06238	3,19274	82,9058	0,117694	0,182683	0,551539	0,6279808
CG31867-RA	Ada1-1	7,54458	2,00916	4,00261	14,0671	3,76903	4,17733	NA	NA	0,6279808
CG31868-RA	Ada1-2	7,83122	10,8858	13,0035	2,87549	0,0965372	3880,11	0,093094	0,71208	0,6279808
CG31869-RA	CG31867	0	0,0170855	0,239825	0,019532	16,7281	0,019939	0,141702	0,529822	0,6279808
CG31869-RB	Dpse\GA25710	8,85277	2,24966	7,63498	3,30198	3,83545	2,83586	0,14047	0,533369	0,13772387
CG31869-RC	CG31869	2,4071	2,28205	1,72615	0,0180499	0,0244488	0,0184261	0,105139	0,644243	0,6279808
CG3186-RA	CG31869	4,24926	5,66378	4,73809	0	0	0	-0,369702	0,112375	0,13772387
CG3186-RB	CG31869	0,0173899	0,0158399	0,0166953	0	0	0	-0,369702	0,112375	0,6279808
CG31870-RA	Sirt4	7,047	8,21729	2,27174	2,28686	17,4178	11,4693	NA	NA	0,6279808
CG31870-RC	Sirt4	1,45202	3,47794	0,0573741	0,0688868	0,0220671	0,0623098	NA	NA	0,6279808
CG31871-RA	Sirt4	0,059761	0,0544345	11,7023	15,6198	0,086106	0,0703223	0,246173	0,320775	0,6279808
CG31872-RA	CG31870	0	0	0	0	0	0	0,062102	0,696631	0,6279808
CG31873-RA	CG31870	0	0	0	7785,91	0,0200585	8215,9	-0,196931	0,485888	0,6279808
CG31874-RA	CG31871	18,5058	22,8285	18,2163	19,6584	10,4555	0	-0,036994	0,916329	0,6279808
CG31875-RA	CG31872	0,0296837	0,0180253	0,662892	0,0501176	0	0	0,29941	0,329469	0,6279808
CG31876-RA	Mulk	16,328	0,0827114	28,0765	0,113897	0,154274	0,11627	0,020837	0,894955	0,6279808
CG3187-RA	Fum4	0,281266	0,177367	2707,23	0,373107	2670,73	3035,99	-0,028398	0,921599	0,6279808
CG3187-RB	CG31875	2,57905	0,235629	5,01477	3,36042	2,52306	2,24219	-0,017756	0,950756	0,6279808
CG3187-RC	Cpr30F	0,224748	0,611651	0,215771	39,3722	57,1959	22,6169	-0,027141	0,925102	0,6279808
CG31882-RA	CG31882	0	0,214034	0,225593	0,318082	0	0,201584	0,108168	0,712742	0,6279808
CG31883-RA	CG31883	0,234728	0,240532	0,563382	5,50832	1,19756	0,578344	-0,318309	0,371569	0,6279808
CG31884-RA	Trx-2	0,0723985	0,0659456	2,83087	431,132	0,656073	1,34755	-0,523034	0,069655	0,6279808
CG31884-RB	Trx-2	354,594	303,359	6,09156	1,06701	3,28611	3,60795	-0,523084	0,069633	0,13772387
CG31886-RA	CG31886	3,90202	0,0278813	9,03489	1,90941	8,11438	5,49995	-0,225689	0,384169	0,6279808
CG31886-RB	CG31886	7,29681	5,21437	0,029387	8,0109	3,08988	3,17711	-0,229462	0,385009	0,6279808
CG31893-RA	Dpit47	11,114	9,52669	13,7701	14,5593	10,6476	14,31	-0,176246	0,572523	0,13772387
CG31894-RA	Dpse\GA16549	8,55238	0,153799	0	0,101735	89,7854	2,29612	-0,017198	0,932762	0,6279808
CG31897-RA	CG31894	0,17419	0,158664	0	13,5936	0	0,275283	0,463541	0,19526	0,13772387
CG31898-RA	CG31897	0,298102	0,104189	0,109815	11,0733	1,21939	7,12515	-0,271956	0,447331	0,6279808
CG3189-RA	CG31898	0,0647168	2,72476	3,05719	7,67802	1,96268	3,58625	-0,237675	0,305895	0,13772387
CG31900-RA	CG43394	5,86858	0,0418058	5,61815	17,503	16,0662	3,71174	-0,293935	0,25117	0,6279808
CG31901-RA	Mur29B	10,5361	0,0166506	6,02147	6,31825	0,0308557	6,97051	0,543659	0,079963	0,13772387
CG31902-RB	Spn28Da	0,130071	0	181,267	3,64853	1,9901	141,649	0,022507	0,938425	0,13772387
CG31904-RB	CG31904	0,103577	0,197267	0,12656	0,0258245	0,0349794	0	-0,053841	0,879858	0,6279808
CG31904-RD	CG31904	1,00186	1,18473	1,38371	6,0662	0,598494	7,06993	-0,031446	0,929549	0,6279808
CG31907-RA	CG31907	0,269697	0,245659	0,345234	5,23895	3,98758	0,171518	-0,006616	0,985052	0,6279808
CG31908-RA	CG31908	15,3224	19,387	0,0410151	14,9311	14,9493	13,6738	-0,160421	0,381855	0,13772387
CG31908-RB	CG31908	12,0672	10,4466	1,09981	22,3754	8,2746	10,1967	-0,149587	0,413619	0,6279808
CG31909-RB	Dsim\GD23456	0,591526	0,119734	0,5048	0,186387	1,09299	0,520386	0,016275	0,953881	0,6279808
CG31909-RC	Dsim\GD23456	0,113597	0,620833	0,10906	0,769352	0,20635	0,155518	0,013106	0,963602	0,13772387

gene_id	Symbol	W1_FPKM	W2_FPKM	W3_FPKM	MCM51_FPKM	MCM52_FPKM	MCM53_FPKM	FC	p-value	p-adj
CG31910-RA	CG3191	5,81617	3,66218	41,5609	3,41264	8,0318	6,73938	0,028938	0,920194	0,6279808
CG31911-RA	CG31910	0,220498	1,88331	3,22915	2,66109	0,314794	0,0448575	0,104519	0,624303	0,6279808
CG31913-RA	Ent2	41,651	44,0325	368,569	59,32	29,0555	35,0985	0,058036	0,747319	0,6279808
CG31915-RA	CG31913	0	157,341	133,402	0	86,31	0,0258261	0,540638	0,038756	0,6279808
CG31918-RA	CG31915	22,6354	0	0	0	0,342495	0	-0,165678	0,521758	0,6279808
CG31919-RB	Nepl3	0,0298356	0,0271763	0,0286439	0,0319008	0,0432098	0,0325655	0,564729	0,017276	0,6279808
CG31919-RD	CG44000	46,4568	46,3403	0,0233166	40,5469	35,2534	0,0261576	0,567859	0,017156	0,6279808
CG3191-RA	CG44000	41,7262	68,4362	17,1872	21,5329	0,0480224	11,2769	0,001161	0,996593	0,6279808
CG31920-RB	Dpse\GA16565	72,5933	0,0365727	3,03977	18,7084	3,2202	9,92722	-0,473437	0,144603	0,6279808
CG31921-RA	Dpse\GA16565	0,0499818	0,0390905	150,997	95,0567	0,0293924	5,64789	0,121372	0,648587	0,6279808
CG31922-RA	CG43755	0,0398607	1,74259	0,0382687	0	2,82723	0,0445898	-0,068667	0,803313	0,6279808
CG31924-RA	CG31921	0,042266	8,96873	0,0676298	9,35718	0,0591644	0,0470927	-0,020689	0,909957	0,13772387
CG31924-RB	CG31922	12,7389	7,80195	8,34195	9,60446	8,54221	15,2839	-0,020689	0,909957	0,6279808
CG31926-RA	CG31924	0,0859615	0,0782997	0	0,227628	0	0,232372	-0,101172	0,771816	0,6279808
CG31928-RA	CG31924	0,233099	0,212322	10,272	28,1037	17,3706	25,7179	0,272445	NA	0,6279808
CG31929-RA	CG31926	0,160769	0	0	21,481	0	0	0,01562	0,897983	0,6279808
CG3192-RA	CG31928	0,289115	2,74774	0	0,348441	0,20926	0	-0,310463	0,308086	0,6279808
CG3192-RB	Gr22c	0	0,0771622	5,38573	18,8509	7,26137	0,104284	-0,255965	0,402451	0,6279808
CG31931-RA	crn	11,022	12,0527	7,49615	43,4072	3,30069	33,5567	0,01562	0,897983	0,6279808
CG31932-RA	Gr22b	0	0	0	0	0	0	NA	NA	0,13772387
CG31933-RA	Gr22f	0	0	0	0	0	15,4746	NA	NA	0,6279808
CG31935-RA	CG31933	0	11,2984	0	3,57744	0	10,0014	-0,053428	0,819852	0,6279808
CG31936-RA	CG31935	3,8986	4,54885	4,65882	6,70197	3,30772	3,43968	-0,039493	0,874043	0,6279808
CG31937-RA	Gr22e	0	0,0777603	10,1844	0,102155	12,4423	5,97275	-0,446694	0,107306	0,6279808
CG31938-RA	CG31937	18,2339	16,5286	19,2468	29,9078	32,3031	21,685	-0,170386	0,58576	0,6279808
CG3193-RA	Rrp40	0,0893317	13,4471	57,6536	0,0320872	57,5634	2,36541	-0,045683	0,834482	0,6279808
CG31941-RA	Hsepi	2,04469	1,84844	2,33202	3,26499	1,73611	1,84729	NA	NA	0,6279808
CG31941-RB	Obp22a	0	0,00971585	0	1,35582	143,508	6,64966	NA	NA	0,13772387
CG31948-RA	Obp22a	0	8,28254	0	0	2,53878	0,0111264	0,17407	0,547319	0,13772387
CG31948-RB	CG31948	0,266993	0,216174	0,257106	0,117132	1,35946	5,33999	0,225462	0,465952	0,6279808
CG31949-RA	CG31948	0,0535605	0,0487866	0,827436	0,97323	6,03644	0,301923	NA	NA	0,13772387
CG3194-RA	CG31949	0	0,0885095	0	0,0434779	0	0	-0,136463	0,587352	0,6279808
CG31950-RA	RpL12	1380,57	1672,37	2535,84	2379,38	2881,18	3288,74	-0,247645	0,476703	0,6279808
CG31952-RA	RpL12	0,0977933	0,0890769	0,0938874	0,125145	0,16951	0,127753	-0,130501	0,470226	0,6279808
CG31953-RA	RpL12	0,112246	0,102242	0,107763	0,149917	0,203064	0,153041	0,172315	0,517863	0,13772387
CG31954-RA	Sbat	14,2623	6,82442	23,0522	24,4104	9,2092	48,4143	-0,635626	0,043676	0,6279808
CG31955-RA	CG31952	0,138042	2,54726	0,145054	4,02148	2,20683	3,11986	-0,891537	0,002677	0,6279808
CG31956-RA	CR31953	0,469734	146,745	2,25486	1,25937	0,83706	1,37751	0,303647	0,257248	0,6279808
CG31956-RB	CG31954	3,88884	5,54844	7,30184	6,45898	11,1065	11,2964	0,304916	0,255033	0,6279808
CG31957-RA	CG31955	1,76539	5,3206	2,46968	213,282	0	81,0702	-0,455852	0,143054	0,6279808
CG31958-RA	Pgant4	0,0280259	0,025528	0,0269066	47,4601	0,040412	0,0304569	0,01562	0,897983	0,6279808
CG31959-RB	Pgant4	10,0614	17,6976	10,4611	140,371	8,5379	8,19825	-0,111536	0,753359	0,6279808
CG3195-RA	CG31957	21,3918	13,937	7,35864	19,9947	4,27993	34,475	-0,371347	0,175587	0,6279808
CG3195-RB	CR31958	0,139401	0,190464	0	1,53588	3,12814	0,997381	-0,371337	0,175606	0,6279808
CG3195-RC	CG43707	0,53633	0,749074	0,514909	1,32164	4,86085	8,21939	-0,371091	0,175846	0,6279808
CG31960-RA	CG31960	0,86942	0,580747	0,612109	0,303346	3,88618	0	-0,281434	0,430591	0,6279808
CG31961-RA	TBCC	2,01752	2,1123	0,0445274	0,0516586	0,300319	0,478191	0,187177	0,378317	0,6279808
CG31961-RB	TBCC	11,1445	12,8534	18,3524	16,7742	0,069972	0,0527351	0,187681	0,370815	0,6279808
CG31962-RA	Sr-CIII	0,205844	2,34479	3,40222	0,023403	0,801348	0,0238907	-0,387195	0,254004	0,6279808
CG31973-RA	Cda5	30,4799	34,852	0,00887517	16,804	1,81326	12,0043	0,633229	0,012802	0,6279808
CG31973-RB	Cda5	6,90341	8,10482	3,6113	5,01154	2,75201	2,40727	0,590624	0,020713	0,6279808
CG31973-RD	Cda5	0,0092444	0,00842044	14,4545	3,7622	9,68732	1,50625	0,559649	0,033177	0,6279808
CG31973-RE	Cda5	4,81557	7,1119	11,5471	10,1315	6,7091	2,5119	0,642045	0,011077	0,6279808
CG31974-RA	CG31974	4,39618	5,8245	9,77497	4,42958	3,84327	5,03501	0,412189	0,147654	0,6279808
CG31975-RA	CG31975	1,74027	0,891652	5,14282	2,05155	4,17125	2,74268	-0,093952	0,791571	0,13772387
CG31976-RA	ovm	0,468993	0,142397	0,250145	0,723181	0,979553	0,376631	-0,25862	0,425517	0,13772387
CG31988-RA	CG3198	8,52135	26,6593	24,9452	24,8686	5,38879	7,90736	0,131926	0,692112	0,6279808
CG31989-RA	CG31988	0,282376	0,285786	0,451829	0,312813	0,422028	0,316422	-0,139023	0,542284	0,6279808
CG3198-RA	Cap-D3	1,46331	1,66969	1,28547	16,8967	0,0574506	18,1504	0,09684	0,675538	0,6279808
CG31991-RA	CG3199	0	0	0	0,0164543	0,0321101	0,0242001	-0,284334	0,130982	0,13772387
CG31991-RB	mdy	10,9313	10,548	3,41447	25,7275	0,0790864	6,75132	-0,283237	0,132629	0,6279808
CG31991-RC	mdy	10,335	11,0072	4,17437	0,0305546	53,3935	45,378	-0,286515	0,128608	0,6279808
CG31991-RD	mdy	0,028658	0,0261037	15,6151	0,030884	27,2046	3,678	-0,282502	0,136149	0,6279808
CG31992-RA	mdy	0,0289468	0,0263667	57,2515	3,56121	17,2331	16,1123	-0,029109	0,876159	0,6279808
CG31992-RB	gw	0,0142569	0,0129861	0,0136874	0,0146899	0,0198975	0,014996	-0,027712	0,882227	0,6279808
CG31992-RC	gw	0,0133253	0,0121376	0,0127931	0,0137003	0,0185571	0,0139857	-0,029198	0,875765	0,6279808
CG31992-RD	gw	0,0129875	0,0118299	0,0124687	0,0133424	0,0180724	0,0136204	-0,0297	0,873637	0,6279808
CG31992-RE	gw	0,0135244	0,012319	0,0129843	0,0139114	0,0188431	0,0142013	-0,026534	0,887157	0,6279808
CG31992-RF	gw	0,0142877	0,0130142	0,013717	0,0147227	0,019942	0,0150295	-0,026906	0,885552	0,6279808
CG31992-RG	gw	0,0139146	6,05202	0,0133589	15,5895	9,09902	7,57361	-0,032408	0,862456	0,6279808
CG31992-RH	gw	49,7222	37,8009	51,9593	45,1078	27,5866	33,2156	-0,027111	0,884782	0,6279808
CG31997-RA	gw	0,0143249	0,0130481	0,0137527	0,0147623	0,0199956	0,0150699	-0,112836	0,705789	0,6279808
CG31998-RA	CG31997	249,422	176,899	190,072	66,8961	44,205	38,6956	-0,06111	0,82989	0,6279808
CG31999-RA	CG31998	19,5263	11,2	32,0538	24,6767	15,3561	22,5446	0,27452	0,19735	0,6279808
CG3199-RA	CG31999	32,2815	0,0226549	14,6012	0,0262776	27,9769	6,05833	NA	NA	0,6279808
CG32000-RA	Reg-2	8,75202	11,1469	7,13981	28,5222	33,3298	4,55851	0,051082	0,834378	0,6279808
CG32000-RB	anne	0,0299709	34,0607	9,1004	12,806	0,0295579	11,974	0,051398	0,833357	0,13772387
CG32000-RC	anne	0,0303007	0,0277399	32,8777	30,8441	19,8856	6,18505	-0,515626	0,052098	0,6279808
CG32000-RD	anne	0,0208529	0,0128202	0,043857	0,0326112	0,0194848	20,164	-0,481665	0,064084	0,13772387
CG32000-RE	anne	19,6347	78,1558	0,0135125	0,014496	35,326	0,0332908	-0,529663	0,0471	0,13772387
CG32000-RF	anne	0,0139705	22,7548	16,5381	23,5252	0,0441721	0,014798	-0,557247	0,044522	0,13772387
CG32000-RG	anne	51,8615	16,3258	13,687	28,2753	0,0196349	9,26485	0,037538	0,877469	0,6279808
CG32000-RH	anne	0,0304544	30,1407	29,1339	21,9859	51,626	19,3093	0,020826	0,931637	0,6279808
CG32006-RA	anne	0,0140747	8,37888	30,2862	9,76256	26,0582	28,7646	-0,284871	0,362966	0,6279808
CG3200-RA	CG32006	7,9666	7,70117	0,0125031	6,91749	10,3784	6,82398	-1,042916	0,000348	0,6279808
CG32016-RA	Mlc-c	147,955	0,577438	17,2639	1,35545	11,9437	12,8052	0,305523	0,191276	0,6279808
CG32016-RB	4E-T	0,0322688	0,0104297	0,03098	0,0347068	0,0470106	0,0354301	0,304352	0,192813	0,13772387
CG32016-RC	4E-T	4,75702	65,0131	6,91249	0,0191851	2,37673	0,0195849	0,306351	0,189629	0,13772387
CG32016-RD	4E-T	0,0309392	0,00955391	0,0297035	0,0331694	0,0449281	0,0338606	0,397893	0,083859	0,6279808
CG32016-RE	4E-T	25,7576	0,0293927	21,4306	21,2538	12,4839	15,804	0,403244	0,080342	0,6279808
CG32016-RF	4E-T	15,1736	0,0167947	13,8449	22,2583	7,83795	9,85362	0,401359	0,082293	0,13772387
CG32017-RA	4E-T	0,0188537	0,0281816	0,0181007	6,29804	2,65222	0,020046	0,455076	0,101341	0,6279808
CG32018-RA	CG32017	24,1576	23,3166	14,0271	7,07751	0,0423487	57,9431	0,017203	0,945242	0,6279808
CG32018-RB	Zyx	57,6234	49,2818	27,6165	37,2259	44,3268	24,9377	0,016158	0,94858	0,6279808
CG32018-RC	Zyx	0,0353221	0,0321738	0,0339113	0,0382753	0,0518441	0,0390728	0,01624	0,948331	0,6279808
CG32018-RD	Zyx	0,0341889	0,0311416	8,50127	14,5202	0,0500419	6,95326	0,017191	0,945293	0,6279808
CG32018-RE	Zyx	0,0354547	11,5292	9,30957	47,7329	35,0147	17,2096	0,016397	0,947829	0,6279808
CG32018-RF	Zyx	38,9189	35,3444	23,1534	0,0422242	0,0571929	10,7613	0,017444	0,944489	0,6279808
CG32018-RG	Zyx	0,0338735	0,0308543	0,0325205	0,0365756	0,0495419	0,0373377	0,049225	0,843905	0,6279808
CG32019-RA	Zyx	0,03283	0,0299038	0,0315187	0,0353587	0,0478936	0,0360955	0,404342	0,040744	0,6279808
CG32019-RC	bt	0	0,0840983	47,4236	49,1048	29,2241	0	0,403814	0,041221	0,6279808
CG32019-RD	bt	0	616,378	0,00231018	0,00241268	0,003268	0	0,404343	0,04074	0,6279808
CG32019-RE	bt	0	0	7,16943	0,00241613	0,00327267	0	0,404327	0,040745	0,13772387
CG3201-RA	bt	0	0	0,002316	0,0024188	4,15612	0	0,071585	0,729958	0,6279808

gene_id	Symbol	W1_FPKM	W2_FPKM	W3_FPKM	MCM51_FPKM	MCM52_FPKM	MCM53_FPKM	FC	p-value	p-adj
CG32021-RA	CR43361	6,51183	6,1931	6,61949	4,80426	19,0766	5,6962	0,26502	0,346479	0,6279808
CG32022-RA	CG32022	7,22045	0	51,3073	0,316259	0,256703	0,573315	0,068719	0,791074	0,6279808
CG32023-RA	CG32023	0	4,2107	24,9837	0,704855	0,0215398	1,57	0,01562	0,897983	0,6279808
CG32024-RA	CG32024	0,137658	0,23238	2,06524	1,39549	540,424	0,17562	0,019597	0,944686	0,6279808
CG32025-RA	Fhos	0,0109821	0,0100033	0,0105435	0,0112299	25,5696	161,121	0,333838	0,174938	0,6279808
CG32025-RB	Fhos	2,4022	2,17327	1,77004	2,18812	0,229036	0,0667626	0,32847	0,183973	0,6279808
CG32026-RA	CG32026	0,110959	0,329345	19,518	17,7439	0,0885845	13,7133	-0,115258	0,745188	0,6279808
CG32027-RA	lncRNA:CR32027	0,0930646	0,03506	0,0369534	0,0536787	0,266006	0,0280677	0,102824	0,723237	0,6279808
CG32029-RA	Cpr66D	73,2486	4,87237	2,57334	9,13826	0	0	0,599706	0,01299	0,6279808
CG32030-RA	RpL17	1057,83	1335,03	1,48923	1,38571	12,768	1,22355	0,526131	0,039825	0,6279808
CG32030-RB	RpL17	0,0653138	0,0594923	16,5164	1988,11	0,546647	0	0,180629	0,477871	0,6279808
CG32031-RA	RpL17	0,0763293	0,069526	0,110006	0,0763943	0	15,108	-0,50669	0,061756	0,6279808
CG32031-RB	RpL17	0,0806129	0,0734278	44,1833	0,0919606	0,139524	0,155102	-0,506205	0,062379	0,6279808
CG32031-RC	Fhos	0,0153927	0,0140208	0,0147779	0,0159023	0,0695358	0,0162337	-0,509719	0,058249	0,6279808
CG32031-RD	Fhos	18,8166	21,4983	15,4779	17,1936	0,0588911	10,4123	-0,506288	0,06231	0,13772387
CG32031-RE	Argk	27,9043	25,1692	53,472	46,9859	62,4561	50,0372	-0,506839	0,061665	0,6279808
CG32031-RF	Argk	139,294	142,01	6,43802	313,261	2,6767	3,0228	-0,507367	0,061507	0,6279808
CG32032-RA	Argk	0,0421491	0,0383924	0,0284725	11,2348	0,0440631	0,0332086	-0,288937	0,274139	0,6279808
CG32033-RA	Argk	0,0308381	0,0280895	1,59493	0,0325307	0,985267	0,834064	-0,283291	0,414624	0,6279808
CG32036-RC	Argk	0,036332	0,0330937	6,50402	3,08853	5,00249	5,68849	0,343543	0,144986	0,6279808
CG32037-RA	Argk	0,0277668	0,025292	227,034	5,08691	535,583	431,377	0,886946	0,000187	0,6279808
CG32038-RA	CG32032	35,5008	37,5925	43,3278	15,0663	2,71843	6,59028	-0,063349	0,793754	0,6279808
CG32039-RA	CG32033	0,248033	7,4199	13,1041	17,8485	10,7472	14,0701	-0,021787	0,948078	0,6279808
CG3203-RA	CG32036	23,5443	4,9293	6,4585	8,57681	1,727	27,0098	-0,626068	0,025898	0,6279808
CG3203-RB	CG32037	15,2466	17,8221	15,2938	15,8861	1,96352	2,86238	-0,626215	0,025887	0,6279808
CG3203-RC	ghi	29,053	28,5738	46,0873	1,29134	24,2249	37,627	-0,626143	0,025895	0,6279808
CG3203-RD	CG32039	10,0069	7,41024	14,1908	11,3287	13,8878	12,3692	-0,626241	0,025868	0,6279808
CG32040-RA	Rap2l	0,0287328	0,0261718	0,0275852	0,0306399	0,0675875	0,050938	-0,048644	0,852961	0,6279808
CG32042-RC	Rap2l	126,3	106,524	82,423	133,631	3,95798	5,50739	0,372623	0,159428	0,6279808
CG32042-RD	CG32040	3,82286	4,53635	81,7806	0,0460935	145,391	80,6575	0,445885	0,098856	0,6279808
CG32042-RE	PGRP-LA	0,0400783	0,0365061	0,0384776	5,48504	0,0595195	0,0448575	0,445885	0,098856	0,6279808
CG32042-RF	PGRP-LA	0,0403722	0,0367738	0,0387597	0,0417427	0,0599996	0,0452193	0,372623	0,159428	0,6279808
CG32043-RA	PGRP-LA	0,042447	0,0386636	0,0407516	0,0397532	0,0634099	0,0477895	0,289474	0,234038	0,6279808
CG32043-RB	PGRP-LA	6,12104	9,59136	9,20524	5,73644	4,07239	5,51482	0,292045	0,231939	0,6279808
CG32043-RC	CG44838	0,0172177	15,6509	13,4474	9,78194	0,0815432	0,0614559	0,333821	0,168214	0,6279808
CG32043-RD	CG44838	3,15407	0,0156831	0,01653	0,017864	3,3133	4,00207	0,033158	0,894641	0,6279808
CG32045-RB	CG44838	2,24668	5,26493	5,10315	8,1495	11,2314	10,682	-0,284544	0,168367	0,6279808
CG32045-RD	CG44838	14,2149	0,012145	0,0128009	0,0137088	0,0241969	0,0182362	-0,281733	0,17334	0,6279808
CG32048-RA	fry	2,56631	0,0124207	1,01034	2,17593	0,0187166	0	0,160527	0,462525	0,6279808
CG32048-RB	fry	2,568	1,42665	3,60581	23,5754	2,12135	8,5768	0,233873	0,273313	0,6279808
CG3204-RA	CG42673	8,82945	11,423	1,89306	1,6658	1,39703	1,09711	-0,186702	0,467393	0,6279808
CG3204-RB	CG42673	2,02326	2,20213	0,154573	11,4403	6,69314	6,17309	-0,180702	0,481632	0,6279808
CG32050-RA	CG43897	0,314647	26,0633	49,4936	40,6282	30,5362	35,7915	-0,622796	0,074603	0,6279808
CG32052-RB	CG32052	1,55384	0,01071	0,0151702	0,0163401	0,0221327	0,0166806	0,290518	0,342046	0,6279808
CG32053-RA	CG32053	5,09109	5,86078	8,06999	3,87971	6,30254	5,50525	-0,108579	0,677723	0,6279808
CG32054-RA	CG32054	5,45052	5,76109	10,3942	1,2325	38,4546	0,0202207	0,264126	0,382577	0,6279808
CG32055-RA	CG32055	0,596947	0	0,553343	40,9746	0,153567	0,103641	-0,001502	0,996636	0,6279808
CG32056-RA	scramb1	18,4718	0,230305	0,242743	69,2315	0,221512	0	0,238136	0,335925	0,6279808
CG32056-RB	scramb1	0,0413577	0	0,153674	0,0284688	0,189838	0	0,235302	0,35162	0,6279808
CG32056-RC	scramb1	9,37003	0	0,0211691	0,0239126	0,0639784	0,048218	0,235302	0,35162	0,6279808
CG32057-RA	dpr10	0,019011	0,0173165	0,0182517	1,22267	4,63479	0,063486	0,579725	0,00916	0,6279808
CG32057-RB	dpr10	11,1898	10,7188	9,01137	5,93463	1,3213	24,8609	0,580277	0,009125	0,6279808
CG32057-RC	dpr10	0,0194761	0,0177402	0,0186982	0,0203149	0,398224	0	0,577652	0,009424	0,6279808
CG32058-RA	Ir67c	0	0	42,666	1,18359	8,81902	0	-0,231974	0,424134	0,6279808
CG32060-RA	Or2a	0,724971	1,15562	0,795446	6,6822	0,732999	4,56357	-0,056025	0,813123	0,6279808
CG32061-RA	CG32060	0,0777363	0,15735	0,0231212	0,456221	0,062173	0	NA	NA	0,6279808
CG32062-RB	CG32061	0	1,30955	0,287695	0,11915	0	1,67466	0,580424	0,007211	0,6279808
CG32062-RD	Rbfox1	0,0178938	0,0162989	40,315	0,018595	0	0,0221592	0,579293	0,007275	0,6279808
CG32062-RE	Rbfox1	6,14189	5,93979	4,02187	8,46922	0	69,0508	0,583459	0,006995	0,6279808
CG32062-RF	Rbfox1	24,2592	24,3078	0,0175062	16,8588	6,93645	43,4761	0,58976	0,006204	0,6279808
CG32062-RG	Rbfox1	0,0197438	0,017984	5,26979	0,0206071	3,08292	41,2029	0,630193	0,002845	0,6279808
CG32063-RA	Rbfox1	13,3823	16,5015	0,0171791	17,4775	0,0256872	0,817596	0,044335	0,715924	0,13772387
CG32063-RB	S-Lap3	0	0,0243999	11,1732	0	0	0	0,044335	0,715924	0,6279808
CG32064-RA	S-Lap3	0	0,0791002	0	0,0374486	0	38,3164	NA	NA	0,6279808
CG32065-RA	Dsim\GD14244	0	0	13,1228	0	0	0	0,01562	0,897983	0,6279808
CG32066-RB	CG32065	0,0922848	9,93724	0,293689	0,462175	0	7,43182	0,021591	0,923912	0,6279808
CG32066-RC	CG32066	0,0305388	0,0278169	0,0293191	49,4444	24,4489	4,39553	0,009915	0,965018	0,13772387
CG32067-RA	CG32066	32,8845	28,6235	33,5519	34,1054	0,0271401	0,0193594	0,06079	0,838514	0,6279808
CG32067-RB	simj	6,19745	5,84372	28,8636	0,0327084	7,60577	0,0333899	0,073084	0,806248	0,6279808
CG32067-RC	simj	0,0182345	0,0166093	0,0189552	29,3764	125,831	26,9308	0,06079	0,838514	0,6279808
CG32068-RB	simj	2,98782	7,4908	16,2819	0,0948736	5,967	0,694585	-0,232961	0,416338	0,6279808
CG32069-RA	Adi1	31,5952	0,0241067	0	0	0,123016	4,09578	0,938049	0,001882	0,6279808
CG3206-RA	CG32069	30,2361	334,822	26,2145	3,77125	29,4247	0,0287761	0,19123	0,571118	0,6279808
CG32071-RA	CG32071	0,802246	57,6196	0,770204	6,49313	0,0161905	0,0122022	-0,014095	0,966355	0,6279808
CG32072-RA	Elo68alpha	1,92197	0,8979	1,77285	0,0119531	0,534391	231,689	-0,386819	0,263484	0,6279808
CG32073-RA	CG32073	14,5167	0,705891	7,20176	21,9905	121,252	4,79577	-0,534273	0,119547	0,6279808
CG32074-RA	CG32074	10,0205	4,76037	0,0194254	5,35776	4,14431	14,6774	-0,308507	0,387761	0,6279808
CG32075-RA	CG32075	6,13005	17,7507	9,26678	5,27644	5,23307	6,09538	0,04037	0,895341	0,6279808
CG32076-RA	Alg10	3,66125	41,5477	0,0244207	0,0269108	0,0364509	4,84047	0,002693	0,991468	0,6279808
CG32077-RA	nol	1,15517	3,34369	0,0187092	25,8582	81,6881	46,6857	0,595558	0,08981	0,6279808
CG32079-RB	CG32079	0	27,0934	0,115424	25,8714	3,68636	16,315	0,058036	0,747319	0,6279808
CG32081-RA	RhoGAP5A	1,32247	0,0325567	3,56874	2,10595	1,69098	1,4706	-0,013096	0,914398	0,6279808
CG32082-RA	RhoGAP5A	2,61485	5,59558	2,85431	4,18219	1,81335	2,90983	0,05772	0,847623	0,6279808
CG32082-RB	CG32081	0	7,68454	0	0,135897	14,9257	0	0,117641	0,686096	0,6279808
CG32082-RC	IRSp53	9,66338	14,2901	8,58329	2,70082	4,48016	8,41291	0,151925	0,611546	0,6279808
CG32085-RA	IRSp53	4,07238	6,43876	3,74312	9,45853	1,79525	3,73505	-0,149756	0,544859	0,6279808
CG32086-RA	IRSp53	4,07623	6,36499	3,61475	4,77514	8,87198	0,0168477	0,029665	0,925205	0,6279808
CG32087-RA	CG32085	5,87365	17,9866	27,0594	0,065335	16,2752	42,5685	0,583916	0,102504	0,6279808
CG32088-RA	CG32086	0,127314	0,231933	0,101857	0,23039	3,29192	24,7164	0,139164	0,695045	0,6279808
CG32089-RA	CG32087	0,0449802	0,0409711	0,650634	0,0520257	0,13837	0,0457895	0,103009	0,606988	0,6279808
CG3208-RA	sunn	0,181278	0,350881	0,217547	7,10223	0,155774	0,0358073	0,174501	0,494734	0,6279808
CG3208-RB	Vha16-2	0,207786	1,83878	137,767	0,0218871	106,633	0	0,174501	0,494734	0,6279808
CG32090-RA	Gpat4	18,2617	12,1458	21,0172	17,8463	18,5598	21,0437	0,069658	0,720558	0,6279808
CG32091-RB	Gpat4	23,5811	21,5246	39,0898	26,6199	36,7814	27,2737	0,205993	0,282388	0,6279808
CG32095-RA	Vha16-3	0,34631	0,189266	0,130995	158,106	0	0	-0,271369	0,340671	0,6279808
CG32096-RA	CG32091	7,0175	7,80233	8,52312	124,212	5,50997	0,63078	0,205765	0,451203	0,6279808
CG32096-RB	CG32095	0,0468401	80,7621	87,3281	98,974	65,708	0,921721	0,211144	0,438703	0,6279808
CG32096-RC	rols	0,0106032	0,00965817	0,0101797	0,010833	0	0,0110587	0,202383	0,458441	0,6279808
CG32096-RD	rols	1,81929	0,0104551	1,82376	3,68576	55,2893	0,0119954	0,198647	0,467001	0,6279808
CG32096-RE	rols	0,011117	0,0101262	0,010673	0,0113714	0,0146733	0,0116083	0,196445	0,471575	0,6279808
CG32099-RA	rols	10,215	19,1038	9,7065	13,9722	1,28954	8,63151	-0,288374	0,237171	0,6279808
CG3209-RA	rols	0,0185202	0,0112463	0,0177805	0,0126653	0,0154026	0,0129293	-0,229738	0,389261	0,6279808
CG3209-RB	CG32099	4,82647	3,72641	11,6437	9,74103	38,9203	3,5049	-0,226318	0,395446	0,6279808
CG32100-RA	Drp1	12,0897	17,2292	12,9662	0	0	1,37114	0,035012	0,874556	0,6279808

gene_id	Symbol	W1_FPKM	W2_FPKM	W3_FPKM	MCM51_FPKM	MCM52_FPKM	MCM53_FPKM	FC	p-value	p-adj
CG32102-RA	CG32100	7,73456	8,66498	8,5364	11,7315	3,90877	2,58593	0,118333	0,660666	0,6279808
CG32103-RA	CG32102	0,3031	0,441736	1,54498	19,9245	25,4065	26,6151	-0,399249	0,049355	0,6279808
CG32103-RB	SCaMC	13,4615	25,4184	2,24497	0,0264889	0,816419	14,6006	-0,397534	0,050309	0,6279808
CG32103-RC	SCaMC	0,0250604	2,8245	0,0250474	8,69212	0,0374449	0,309956	-0,388771	0,061495	0,6279808
CG32104-RB	SCaMC	2,75245	0,0237641	16,0756	32,0231	0,886158	1,77789	0,033914	0,901502	0,6279808
CG32105-RB	CG32104	1,30808	8,88641	8,81969	1,869	14,7111	14,2445	-0,039251	0,852869	0,6279808
CG32106-RA	Lmx1a	3,67366	4,33643	4,39067	37,9076	0,0207342	37,6998	-0,007158	0,964229	0,6279808
CG32107-RA	CG32106	0,0279588	0	0,0268421	0,0476307	0	0	0,118576	0,735464	0,6279808
CG32108-RA	CG32107	0,234605	9,61306	0,211986	25,352	0,390836	1,83387	0,506756	0,085777	0,6279808
CG32109-RA	CG42588	5,31244	2,18067	3,93288	21,891	0,664037	0,544532	-0,241398	0,416812	0,6279808
CG3210-RA	CG32109	16,2123	0,0209587	14,2186	5,89842	0,0179886	16,1676	0,09639	0,712472	0,6279808
CG32110-RA	CG32110	0,205844	0,257808	0,197622	0,196913	0,0218403	0	0,085098	0,782992	0,13772387
CG32111-RA	lncRNA:CR32111	1,39454	1,73215	0,772877	10,5895	3,3796	0,666095	0,498012	0,130731	0,6279808
CG32112-RA	CG32112	2,91731	12,7476	7,5765	0,0394202	4,37504	5,55337	0,137228	0,584228	0,6279808
CG32112-RB	CG32112	0,889156	15,9247	0,0348426	1,68402	27,6929	0,0402416	0,1444	0,563537	0,6279808
CG32112-RC	CG32112	3,82619	4,41178	2,07786	17,2902	7,64666	1,7658	0,140333	0,573833	0,6279808
CG32113-RB	Vps13D	1,51986	1,04661	0,0279745	1,87669	0,0421288	1,0758	0,073955	0,756652	0,6279808
CG32115-RA	CG32115	6,59905	0,088928	0,0562383	13,1479	0,0578603	4,47306	0,182041	0,58933	0,13772387
CG32117-RA	CG32117	0,139794	0,0355782	0,13421	1,85747	24,8857	8,972	0,020837	0,894955	0,13772387
CG32118-RA	CG32118	0	0	0	21,9833	26,9732	99,2264	NA	NA	0,13772387
CG32119-RA	CG32119	0,0554949	0,185345	0,195354	0,226758	0,0845433	0,102635	0,085864	0,783539	0,13772387
CG32120-RA	Dsim\Sr-CIV	0,811743	0,0619203	0,0391585	0,896979	0,09681	0	-0,1053	0,719146	0,6279808
CG32121-RB	sens	1,01137	71,4026	276,723	26,8434	13,3024	14,658	0,066391	0,816563	0,6279808
CG3212-RA	CG32121	1,6799	2,58119	0,139116	2,03901	0	0,112543	0,274821	0,415634	0,6279808
CG32130-RA	CG3213	0,0407876	0,193023	0,0290639	22,6151	1,05153	0,627741	-0,509784	0,030517	0,6279808
CG32130-RB	stv	0,0200372	0,0182512	12,5212	0,0252408	11,1297	7,14916	-0,498834	0,034185	0,6279808
CG32130-RC	stv	8,55791	11,9077	14,9093	20,3529	4,02078	17,9079	-0,498793	0,034173	0,6279808
CG32130-RD	stv	8,39215	12,3388	16,6597	13,7754	0,933212	8,28546	-0,492596	0,036558	0,6279808
CG32130-RE	stv	12,9655	12,2274	0,0259597	0,0209278	9,45898	0,0293154	-0,509744	0,030505	0,6279808
CG32132-RA	stv	6,01632	8,14007	0,0225513	23,4513	10,2791	0,0257668	0,045653	0,863071	0,6279808
CG32133-RA	dysc	4,84177	7,13417	6,56243	9,25471	2,72395	2,12685	0,037756	0,911204	0,6279808
CG32134-RA	Ptip	1,35319	1,66092	1,45578	3,04364	6,62321	6,37114	-0,072899	0,780052	0,6279808
CG32134-RB	btl	2,03446	0,0176259	0,0185777	0,020178	2,01248	0,0217719	-0,125271	0,660272	0,6279808
CG32135-RA	btl	0,0196908	0,260146	0,11346	0,460778	0,0273312	0,614863	-0,444107	0,204303	0,6279808
CG32136-RA	Nxf3	0,393309	0,411991	6,99449	12,0909	49,4539	59,668	0,435262	0,094783	0,6279808
CG32136-RB	Tsp68C	5,68268	5,29185	4,81566	5,27401	0,098781	0,0744473	0,452209	0,082606	0,13772387
CG32137-RA	Tsp68C	0,0627699	0,0571751	1,35591	0,0729277	7,05435	5,00855	0,307698	0,184791	0,6279808
CG32137-RB	CG32137	17,3702	21,4777	14,175	12,5644	0,0320065	0,024122	0,276006	0,232319	0,6279808
CG32138-RA	CG32137	4,48337	6,79044	6,56656	8,5533	0,638722	0,781359	0,342977	0,255972	0,13772387
CG32138-RB	Frl	3,65389	13,3089	12,6015	18,1218	1,19985	3,64943	0,341924	0,257369	0,6279808
CG32138-RC	Frl	5,34457	6,13406	0,0195873	52,435	1,19892	3,65911	0,342733	0,256254	0,6279808
CG32139-RA	Frl	8,97484	4,40242	14,0276	26,5203	3,84911	0,0174418	0,21818	0,455397	0,6279808
CG3213-RA	Sox21b	5,22173	7,22463	6,88513	7,79372	4,10316	1,655	0,096932	0,776311	0,6279808
CG32141-RA	Dpse\GA16708	0,110371	0,100534	0,105963	84,7484	37,9603	0	0,144837	0,574797	0,6279808
CG32146-RA	Dpse\GA16708	64,6341	60,9238	114,493	0	0,0366832	0	0,548922	0,060893	0,6279808
CG32146-RB	saturn	0,138234	0,209855	0,265426	0,309427	0	0	0,546013	0,062399	0,6279808
CG32147-RA	dlp	25,5584	34,5664	425,091	415,155	67,3511	105,113	-0,04337	0,847891	0,13772387
CG32148-RA	dlp	0,0156664	0,0142701	0,0371264	0,0422512	0,106438	0,0802183	-0,041811	0,731452	0,6279808
CG32149-RA	CG32147	28,1518	25,5741	37,3476	4,35033	32,6349	30,7415	0,036441	0,879534	0,6279808
CG32149-RB	CG32148	0	0	0,147356	0	6,36661	0	0,031045	0,897738	0,6279808
CG32149-RC	RhoGAP71E	4,13568	0	0	0,361405	0	0	-0,001885	0,993826	0,6279808
CG32149-RD	RhoGAP71E	0,0230819	0,421212	34,8003	0	0	19,0771	-0,008061	0,973704	0,6279808
CG3214-RA	RhoGAP71E	4,62737	0,361698	8,16385	0	0	34,251	-0,378652	0,214502	0,6279808
CG3214-RB	RhoGAP71E	3,31195	4,46652	25,6187	0	0	3,66956	-0,378652	0,214502	0,6279808
CG32150-RA	Gpdh2	0	0,0746113	0,0786407	0	119,418	719,783	0,521052	0,017936	0,6279808
CG32150-RB	meru	0,0172627	6,05039	7,3957	114,928	3,94164	137,845	0,495345	0,026676	0,6279808
CG32152-RA	meru	7,05759	93,8344	100,945	133,673	157,847	4,94421	-0,490756	0,163931	0,6279808
CG32154-RA	CG32152	0,438003	10,8446	0	1,81678	0,693946	7,31116	0,368969	0,278571	0,6279808
CG32155-RA	l(3)72Dr	0,350113	1,01586	0,366687	0	1,00945	0,429272	0,094932	0,756823	0,6279808
CG32156-RA	l(3)72Dp	1,37767	0,550092	2,39337	0,31237	0	4,84081	0,145572	0,584927	0,6279808
CG32156-RC	Mbs	0,0134913	0,0122888	2,72649	6,31969	0,0187955	0,0141654	0,142618	0,59259	0,6279808
CG32156-RE	Mbs	7,1684	9,51097	3,40648	0,0382531	6,00711	5,75752	0,184368	0,407546	0,6279808
CG32156-RF	Mbs	0,016086	0,0146522	0,0154435	13,0396	3,16897	2,97826	0,380846	0,156189	0,6279808
CG32156-RG	Mbs	6,32056	7,88381	3,72439	22,4077	4,7756	4,27726	0,056574	0,829725	0,6279808
CG32156-RH	Mbs	0,0353032	0,0321566	7,27009	5,39283	0,051814	0,0390502	0,045039	0,864257	0,6279808
CG32158-RA	Mbs	33,1128	37,5323	16,9404	2,98255	15,3633	14,0351	-0,879485	0,002257	0,6279808
CG32158-RB	CG42514	0,02654	0,0241745	0,024822	17,1789	0,037087	0,027951	-0,495417	0,047681	0,13772387
CG32158-RC	CG42514	0,0258546	0,0235502	0,0305425	0,0281526	0,0476125	0,0358837	-1,108149	0,000131	0,6279808
CG32158-RD	CG42514	2,02013	0,0289776	11,9999	0,0273805	17,2073	17,6993	-1,044116	0,000319	0,6279808
CG32158-RE	CG42514	9,39059	6,22619	6,0537	0,0351512	14,1916	12,4262	-1,106934	0,00013	0,6279808
CG32158-RF	CG42514	0,0311385	0,0283631	0,0128527	9,05977	0,0186463	0,0140529	-0,87802	0,002222	0,13772387
CG32158-RG	CG42514	0,0133874	0,0121942	0,0126	17,3372	0,0182684	0,0137682	-0,483054	0,053505	0,6279808
CG32159-RB	CG42514	0,787451	1,01015	3,74114	0,0137661	3,85945	3,97488	0,423287	0,222778	0,6279808
CG3215-RA	dsx-c73A	24,5349	29,7745	15,1103	3,91928	7,63069	8,70434	0,044335	0,715924	0,6279808
CG32160-RA	CG3216	0,173472	0,783718	1,24038	0,0200674	0,0271814	0,261883	0,123396	0,7112	0,6279808
CG32161-RA	CG3216	0,0192491	4,87737	0,838371	1,05282	0,9239	0,781698	-0,120185	0,499323	0,6279808
CG32163-RA	lncRNA:CR32160	0,0926731	0,401243	0,163115	0,467539	0	0,0839258	-0,021763	0,933616	0,6279808
CG32164-RA	CG32161	0	6,16802	36,1152	7,97356	0,38065	5,71685	-0,613829	0,03456	0,13772387
CG32165-RA	CG32163	9,86635	6,1196	221,616	9,35288	42,6186	47,8657	0,374875	0,254274	0,13772387
CG32167-RA	Arts	2,88462	19,6316	301,74	17,5894	2,98288	3,57437	-0,213386	0,532957	0,6279808
CG32169-RA	Apl	2,194	2,6885	3,52995	3,34157	47,545	0,0260159	0,39741	0,088651	0,6279808
CG32169-RB	CG32167	0,467518	0,236582	0,548589	1,03787	0,308811	0,501482	0,066444	0,799286	0,6279808
CG32169-RC	Rbp6	16,1503	0,109231	0	0	0	0,0341702	0,481173	0,029474	0,6279808
CG32169-RD	Rbp6	0,0312022	0	0	0	8,71828	0,0549764	0,058815	0,799117	0,6279808
CG3216-RC	Rbp6	0,0481368	0,123841	0,0959419	0,181749	0,045339	0,0292262	-0,360021	0,312642	0,6279808
CG3216-RD	Rbp6	0,0269624	18,423	17,0616	5,60684	0,0729458	0	-0,339391	0,341096	0,6279808
CG32170-RA	CkIIalpha-i3	12,4347	10,1019	1,94739	7,15552	9,89729	3,45502	-0,033476	0,893225	0,6279808
CG32171-RA	CkIIalpha-i3	0,0493226	2,44849	8,65779	2,68261	7,46391	0,0842366	0,259938	0,269798	0,6279808
CG32171-RB	Dpse\GA16730	5,18526	5,14714	40,7114	15,4071	25,6635	31,6246	0,167554	0,464543	0,6279808
CG32171-RC	Dpse\GA28580	0,0221905	0,0202126	3,47611	11,1992	3,29967	4,56139	0,187988	0,421269	0,6279808
CG32171-RD	Dpse\GA28580	0,0230578	0,0210026	0,0213042	0,0232955	0,0315538	0,0237809	0,170419	0,456364	0,6279808
CG32171-RE	Dpse\GA28580	0,0234922	15,6743	0,0221368	0,0242559	0,0328548	0,0247613	0,20034	0,353288	0,6279808
CG32171-RF	Dpse\GA28580	0,0235173	0,0214212	10,7921	17,925	0,0335085	9,57754	0,187594	0,422273	0,6279808
CG32171-RG	Dpse\GA28580	0,0320187	0,0291648	0,022578	0,0247665	12,0419	0,0252825	0,167186	0,465551	0,6279808
CG32171-RH	Dpse\GA28580	52,7115	45,1985	0,0307398	0,0344168	0,0466178	0,035134	0,170491	0,456214	0,6279808
CG32172-RB	Dpse\GA28580	0,0506846	0,046167	42,4783	40,6483	46,9245	44,3552	0,279759	0,228563	0,6279808
CG32172-RC	Dpse\GA28580	63,6919	53,4447	0,0486602	26,6491	0,0773054	0,058262	0,197633	0,388566	0,6279808
CG32174-RB	lncRNA:noe	9,01983	8,72343	0,0746606	8,12916	7,78342	0,096021	-0,400893	0,104212	0,6279808
CG32174-RC	lncRNA:noe	0,0777667	0,0708352	16,5075	0,094061	0,127406	24,1008	-0,172642	0,359417	0,6279808
CG32176-RA	Coq4	5,22199	8,79983	6,82461	12,2157	21,0944	23,4362	0,52951	0,044674	0,6279808
CG32177-RA	Coq4	0,0774629	20,2471	24,7885	28,7251	1550,68	1,74474	0,20213	0,283916	0,6279808
CG32178-RA	CG32176	8,86844	9,84358	10,0787	829,109	0,584024	0,0382725	-0,46389	0,187485	0,6279808
CG32178-RB	Ndfip	28,7023	30,5703	35,549	35,1408	63,9186	7,38797	-0,364781	0,29181	0,6279808
CG32178-RC	Adgf-A2	0,0375289	0,0341839	0,0360299	13,0641	0,0553829	16,1824	-0,364781	0,29181	0,6279808

gene_id	Symbol	W1_FPKM	W2_FPKM	W3_FPKM	MCM51_FPKM	MCM52_FPKM	MCM53_FPKM	FC	p-value	p-adj
CG32179-RA	Adgf-A2	0,0383344	0,0349177	0,0368034	1,74059	0,0566844	24,6213	0,197234	0,361819	0,6279808
CG3217-RA	Adgf-A2	0,0382015	0,0347965	0,0366757	0,0750817	0,0564692	0,076308	0,154121	0,561377	0,6279808
CG3217-RB	Krn	13,5503	18,7346	15,459	16,6974	6,56678	4,33113	0,153252	0,563352	0,6279808
CG32180-RA	fs(1)K10	5,09838	5,03339	7,10098	0,888286	2,63544	0,751897	0,315307	0,210066	0,6279808
CG32180-RB	Eip74EF	11,4727	0,0154774	0,0163132	5,09418	3,71919	0,0179875	0,374375	0,139265	0,13772387
CG32180-RD	Eip74EF	0,0132002	5,68563	7,33346	13,9865	0,0183775	6,45597	0,374375	0,139265	0,6279808
CG32181-RA	Eip74EF	14,7152	3,63066	0,0146618	0,0157729	14,1127	2,99345	-0,252406	0,477592	0,6279808
CG32182-RA	CG32181	0,443462	0,403936	0,3406	0,0194543	0,207967	0,0408288	-0,061002	0,859307	0,6279808
CG32183-RA	CG32182	2,17631	4,46622	2,06421	0,0184802	1,42899	0,0188653	0,399479	0,085399	0,6279808
CG32183-RB	Ccn	0,0270951	0,0294801	0,484587	0,0416899	5,45703	1,04858	0,373765	0,108452	0,13772387
CG32185-RA	Ccn	4,87087	12,1732	7,65052	6,18144	1,1316	5,13983	-0,299524	0,239225	0,6279808
CG32186-RA	edin	0	0	196,226	13,8249	0,914695	13,7288	-0,009858	0,973812	0,6279808
CG32187-RA	CG42816	0,524412	0,150843	4,54445	26,1706	0,814466	2,06161	-0,015671	0,958477	0,6279808
CG32188-RA	CG32187	1,72548	50,8817	0,0260129	5,41365	6,60234	0,0293794	0,076501	0,820814	0,6279808
CG32189-RA	CG32188	4,88297	2,30412	27,2292	0,793308	1,24112	20,0814	-0,053751	0,880621	0,6279808
CG3218-RA	CG32189	7,66542	41,4918	85,5242	23,6991	12,1582	22,3699	0,130355	0,57796	0,6279808
CG32190-RA	Klp59C	0	0,0769648	0	11,5362	15,6259	1,17273	-0,185873	0,393258	0,6279808
CG32191-RA	NUCB1	40,6275	3,36926	2,65538	6,88139	4,54421	2,24809	0,098289	0,704042	0,6279808
CG32192-RB	CG32191	6,99208	2,19574	10,3591	1,85557	2,41845	7,04684	-0,054713	0,832105	0,6279808
CG32192-RC	CG32192	0,0700945	0	0	65,8506	4,46301	0,0580028	-0,054713	0,832105	0,6279808
CG32193-RA	CG32192	0,283081	0	15,9969	23,3628	3,46427	9,36814	-0,230566	0,51285	0,6279808
CG32195-RA	CG32193	0,0618301	0	0,135887	2,80025	0,0412907	21,9574	-1,095974	0,000309	0,6279808
CG32196-RB	CG32195	7,38995	1,40603	0,0453227	0,0526914	1,21877	0,0537894	-0,276367	0,383457	0,6279808
CG32197-RA	CG32196	10,6237	6,81771	3,44677	28,0112	5,69876	0,015522	NA	NA	0,6279808
CG32198-RB	Met75Ca	0	49,773	1,21994	0,0716587	0,0970622	19,1832	-0,326432	0,21586	0,6279808
CG32199-RA	CG32198	0,884227	0,0260472	0,0274539	0,0830371	0,112474	46,0519	0,589341	0,047383	0,6279808
CG3219-RA	CG32199	0,214913	0,113132	0,131068	0,357612	0,599625	0,995847	NA	NA	0,6279808
CG32201-RB	CG32201	0,708089	0,142369	106,965	104,354	117,429	105,192	1,82294	3,48E-07	0,6279808
CG32202-RA	CG32202	19,9052	1,42059	21,9371	1,28274	1,59644	2,17291	-0,117186	0,655013	0,6279808
CG32203-RB	Dsim\GD12299	0	0,0437825	0,33213	3046,23	85,9559	0,0289994	0,01562	0,897983	0,6279808
CG32204-RB	CG32204	4,99103	5,51173	4,06021	3,88037	31,3239	2,88522	0,288056	0,268077	0,6279808
CG32206-RB	CG32206	3,28466	3,19523	2,95665	6,5552	1,95343	1,36648	0,574104	0,014807	0,6279808
CG32207-RA	hpRNA:CR32207	4,76333	14,6771	0,0531889	0	0	0	-0,42144	0,141863	0,13772387
CG32208-RB	825-Oak	39,372	38,4197	0,0361738	24,61	26,0884	31,3269	1,008347	0,003034	0,6279808
CG32209-RB	serp	220,852	273,475	236,706	11,2629	123,252	152,797	0,261723	0,28508	0,6279808
CG32210-RA	dgt3	0,535252	0,487544	4,45364	5,47015	5,20661	4,08924	-0,065065	0,789049	0,6279808
CG32211-RA	Ltn1	7,20142	0,0425446	3,73713	15,5602	0,0753475	38,5159	0,031972	0,894722	0,6279808
CG32212-RA	Taf6	12,0386	3,96806	0,103711	6,04082	1,9476	1,99159	0,498278	0,163004	0,6279808
CG32213-RB	CG32212	3,2448	0,0451285	0,0462142	11,7886	0,0556245	10,0294	-0,230882	0,49286	0,6279808
CG32214-RB	CG32213	42,9709	38,4263	0,0475656	47,504	46,5981	0	0,777246	0,00543	0,6279808
CG32217-RA	CG32214	59,2989	64,4733	26,8323	102,061	102,945	136,915	0,301214	0,305201	0,6279808
CG32219-RA	Dsim\GD12242	43,5998	0,0649494	42,4653	13,1116	25,5529	22,8325	-0,8113	0,017534	0,6279808
CG3221-RB	CG32219	0,0474115	0,0431857	0,0455178	0,0529454	0,0717149	0,0540487	-0,256855	0,457065	0,6279808
CG32220-RA	CG3222	0,121642	0,17728	4,28897	0	0,952436	7,56396	0,094588	0,743613	0,6279808
CG32221-RA	Csas	12,9557	10,6354	1,16517	10,3036	14,1622	3,02738	-0,159183	0,536273	0,6279808
CG32223-RA	CG32221	3,95819	10,5596	5,44235	756,878	372,653	9,18405	-0,282579	0,202986	0,6279808
CG32225-RA	CG42674	4,29596	1,71623	400,844	51,8137	99,8289	76,7878	-0,229833	0,400247	0,6279808
CG32226-RA	CG32225	19,6407	2,66053	6,0872	4,25944	7,57614	10,5921	-0,138017	0,520113	0,6279808
CG32227-RA	Pex23	5,89725	6,59153	0,0191613	6,97327	4,80423	2,62058	0,220307	0,43726	0,6279808
CG32228-RA	gogo	4,8549	0,0181796	0,0515885	0,0208418	0,0282303	0,0212761	0,041871	0,87684	0,6279808
CG3222-RA	CG32228	0,0721352	0,0728061	0,0346271	0,0609814	0,0525928	0,0396371	0,11367	0,698216	0,6279808
CG32230-RA	CG3223	8,82566	8,24462	12,5905	67,6775	29,1694	0,0572212	-0,630734	0,032948	0,6279808
CG32230-RB	Dpse\GA16771	0,167421	0,152499	0	0,268375	0	22,608	-0,630734	0,032948	0,6279808
CG32232-RA	Dpse\GA16771	1273,57	934,174	0,160734	1974,74	4,03266	12,1691	NA	NA	0,6279808
CG32233-RA	CG32232	0	0	0	0,168515	148,773	150,385	-0,040292	0,831087	0,6279808
CG32234-RA	CG32233	0	0,148243	0	0	0	180,074	0,242165	0,382004	0,6279808
CG32235-RA	axo	3,21342	4,17708	40,916	0	14,9948	11,6933	0,17287	0,319146	0,6279808
CG32236-RA	CG32235	0	0,145848	0	0	0	0	NA	NA	0,6279808
CG32237-RA	CG32236	0	0	3,85887	8,29199	0,3813	0,0238568	-0,205083	0,55661	0,6279808
CG32238-RA	CG32237	30,525	0,235103	78,6875	42,5725	71,6093	46,7353	-0,137007	0,695297	0,6279808
CG32239-RA	TTLL1B	0,325337	0,34079	0,0213688	0,0252022	0	0,0260663	0,228427	0,401746	0,6279808
CG3223-RA	RhoGEF64C	7,30255	0,73528	6,20901	9,42585	0	4,77108	-0,171672	0,4695	0,6279808
CG32240-RA	CG3224	22,7111	0,205649	0,216754	0,142848	0	52,1236	-0,009012	0,962385	0,6279808
CG32241-RA	CG32240	0	14,3408	9,34308	81,0838	6,76643	29,7634	-0,34277	0,336228	0,6279808
CG32242-RA	CG32241	1,6981	0,295701	8,03149	4,15898	0	6,40549	0,229497	0,490384	0,6279808
CG32243-RA	NT1	8,66353	9,53466	14,9632	14,3071	131,724	5,40928	0,919956	0,000144	0,6279808
CG32244-RB	CG32243	11,714	11,4439	0,183343	11,7353	3,83153	0	0,216629	0,454569	0,6279808
CG32244-RC	NT1	12,5518	15,5672	11,0449	232,315	3,91295	5,38178	0,215451	0,452368	0,6279808
CG32245-RA	NT1	5,83398	614,811	8,19703	6,106	5,74674	4,1279	-0,169192	0,551711	0,6279808
CG32245-RB	CG42540	16,0362	0	0	0	2,32958	2,09319	-0,857762	0,003694	0,6279808
CG32245-RC	CG42540	0,0173306	2,56609	0,6272	1,73221	15,1626	7,9385	-0,163195	0,566099	0,6279808
CG32246-RA	CG42540	0,0302453	1,10512	1,96703	0	2,744	2,95236	-0,057792	0,851547	0,6279808
CG32248-RA	CG32246	0,0601784	5,85701	0,706477	1,0582	0,199694	0,572569	0,152236	0,670426	0,6279808
CG32249-RA	CG32248	6,08318	0,0462736	0	179,197	0	3,55002	0,007616	0,973438	0,6279808
CG3224-RA	CG32249	0,0594564	0,716506	11,5797	0,121797	397,423	0	-0,367999	0,255715	0,6279808
CG32250-RA	CG3225	0,0287453	0,0261832	9,25608	16,8002	13,8178	10,7909	-0,327401	0,2846	0,6279808
CG32251-RA	PMP34	11,2938	7,51264	17,8613	11,9316	12,1905	30,1803	0,040006	0,89891	0,13772387
CG32252-RA	Claspin	2,31677	2,47204	3,15631	4,55664	0,0494869	52,8725	0,236835	0,454777	0,6279808
CG32255-RA	asRNA:CR32252	0,0701689	302,343	12,5592	9,60432	26,5156	11,0037	-0,27413	0,314897	0,6279808
CG32256-RA	Gr64f	0	0	0	0,297366	0,110869	0,0835572	0,099631	0,472914	0,6279808
CG32257-RA	Gr64c	0	0,0712547	0,0751028	0	0,52155	0	-0,283295	0,200749	0,6279808
CG32258-RA	Gr64b	0	0,109749	0,0731963	0,22561	0	0	0,091159	0,66576	0,6279808
CG32258-RB	Gr64e	0	0,0234584	0,111711	0	0	0,0250565	0,059792	0,706172	0,6279808
CG32259-RB	Gr64e	0	10,7552	9,23803	9,65648	0	0,0456651	-0,746223	0,028926	0,6279808
CG3225-RA	CG32259	0,423563	0,666401	59,6342	1,57717	1,5533	1,35915	0,757278	0,006031	0,13772387
CG32260-RA	CG3226	13,8216	18,472	24,2708	26,2066	2,44768	2,78434	0,146212	0,643279	0,6279808
CG32261-RA	CG32260	0,815976	7,77409	0,887837	1,19057	0,628718	53,7019	-0,553902	0,072173	0,13772387
CG32262-RA	Gr64a	0	0	0,115676	0	0,12198	0,18605	-0,324678	0,260808	0,6279808
CG32263-RA	CG32262	7,52884	6,97353	11,2844	2,3335	1,13909	0,0236305	-0,041811	0,731452	0,6279808
CG32264-RA	CG32263	0	0	2,34951	8,70002	12,9828	0,0247796	0,047076	0,83799	0,13772387
CG32264-RB	CG32264	2,44543	0,184244	0,0165602	0,427753	134,519	29,1768	0,031734	0,885818	0,6279808
CG32264-RD	CG32264	10,2693	10,9276	5,23891	8,83899	0	7,31473	0,02337	0,921225	0,6279808
CG32264-RE	CG32264	0,0172492	0,0296242	0,0168864	0,0350018	0,0474102	0,0357312	0,022423	0,924328	0,6279808
CG32264-RF	CG32264	3,71137	12,2535	0	10,8974	6,34066	6,55736	0,022423	0,924328	0,6279808
CG32266-RA	CG32264	0,017589	0,0141427	425,315	27,5398	23,0038	136,405	-0,941662	0,003814	0,6279808
CG32267-RA	CG32266	256,482	0,0157117	0,258926	3,01897	0,0126659	2,63758	-0,107557	0,702134	0,6279808
CG32268-RA	CG32267	90,4092	17,2121	0,0623354	7,76959	4,75292	0,0774474	-0,029268	0,887213	0,6279808
CG32269-RA	Drsl6	0	25,5535	0,0334994	12,2519	0,0511606	7,02949	-0,071244	0,763969	0,6279808
CG3226-RA	CG32269	8,62423	11,1336	9,13577	10,9121	7,37168	10,3882	-0,138465	0,562194	0,6279808
CG32271-RA	insv	2,47735	1,20452	16,2348	1,93758	3,89078	4,80826	-0,117353	0,727807	0,6279808
CG32274-RA	CG32271	0,221138	0,362569	0,849221	0,181513	1,00733	0,49424	0,025482	0,880577	0,6279808
CG32276-RA	Drsl1	0	8,29716	8,53289	4,6202	0	0,0300085	0,394087	0,04158	0,6279808
CG32276-RB	CG32276	0,0764767	0,0696603	0,0734222	0,0996064	0,134917	0,101682	0,394176	0,041515	0,6279808
CG32277-RA	CG32276	543,151	583,67	563,863	559,67	395,787	482,054	-0,218981	0,397754	0,13772387

gene_id	Symbol	W1_FPKM	W2_FPKM	W3_FPKM	MCM51_FPKM	MCM52_FPKM	MCM53_FPKM	FC	p-value	p-adj
CG32278-RA	CG32277	0,116467	0	0,18636	0,245492	0,33252	0,15558	-0,095574	0,705214	0,6279808
CG32279-RA	CG32278	39,9881	1,34535	0,0223553	0,0133289	0,0303314	4,13083	0,115085	0,742062	0,6279808
CG3227-RA	Drsl2	0,692412	11,9083	2,94392	3,38584	3,66002	10,573	-0,217909	0,50006	0,6279808
CG32280-RA	kz	2,84297	38,4457	4,94291	7,36919	0,082295	12,5281	-0,000836	0,997446	0,6279808
CG32280-RB	CG32280	0,0625323	0,0228267	0,0457476	9,10877	0,0721206	0,0543544	-0,001189	0,996367	0,6279808
CG32280-RC	CG32280	101,397	0,023268	0,0315763	10,5502	0,0494047	0,0372344	-0,004358	0,986682	0,6279808
CG32281-RA	CG32280	0,0476508	23,0317	11,3366	24,0014	37,7143	19,9748	0,108763	0,707355	0,6279808
CG32282-RA	CG32281	3,72349	5,11548	0,0216458	47,0117	0,0312336	3,91421	0,323034	0,327136	0,6279808
CG32283-RA	Drsl4	1,28303	0,681724	51,2151	32,2337	0,108773	1,63599	0,01562	0,897983	0,6279808
CG32284-RA	Drsl3	0	0,0665288	0	0	1,22286	2,45825	-1,020068	0,004356	0,6279808
CG3228-RA	CG32284	4,29995	8,78773	9,9765	15,1595	4,72626	6,10347	-0,066151	0,809767	0,13772387
CG32295-RA	CG32295	0,0857386	0,156193	0,0823142	0,103655	0	0,105815	0,111988	0,623449	0,6279808
CG32296-RA	Mrtf	4,21122	4,4704	3,38134	0,0287045	0,0388804	0,5777	0,10609	0,725368	0,6279808
CG32296-RB	Mrtf	0,0119149	26,5409	2,81305	48,5546	13,4384	2,62383	0,107937	0,72113	0,13772387
CG32296-RC	Mrtf	0,713567	0,0122813	0,537632	6,66872	46,0512	0,0124645	0,106306	0,724879	0,13772387
CG32298-RB	CG32298	0,0573243	0,20886	0,110968	0,17958	0,1711	8,0472	-0,013205	0,964073	0,6279808
CG32299-RA	CG32299	0,316656	0,115373	6,9116	0,114594	0,155219	6,88523	0,020596	0,932431	0,6279808
CG32300-RB	oxt	6,33804	6,09888	6,9528	0	0,687943	0,0650202	0,235541	0,250014	0,6279808
CG32301-RA	CG32301	0,0306759	0	0	2,06286	6,69062	0,0523171	-0,141385	0,507414	0,6279808
CG32302-RA	CG32302	43,7238	31,8494	0,424577	0,957358	0,0100445	0,00721916	-0,462073	0,164264	0,13772387
CG32304-RA	obst-I	0,450517	7,30482	43,7003	21,5548	1,39771	0,0459609	0,121023	0,731428	0,6279808
CG32305-RA	CG32305	0	17,4789	10,1445	8,8861	10,8414	35,612	-0,055469	0,774038	0,6279808
CG32306-RA	Svil	0,0092444	0,00842044	0,00887517	0,00941509	0,0127528	0,00961127	0,641065	0,043419	0,6279808
CG32306-RB	Svil	4,55942	8,81565	4,55187	12,9573	4,3364	4,65906	0,301114	0,341227	0,6279808
CG32306-RC	Svil	7,95578	18,0352	6,59151	9,62337	7,45377	0,0357502	0,666536	0,035913	0,6279808
CG32306-RD	Svil	19,1633	35,9601	16,3556	16,3805	0,0520558	10,0129	0,310225	0,327394	0,6279808
CG32313-RA	snama	6,86089	6,45565	8,78517	4,5388	3,88613	5,29163	0,162108	0,650238	0,6279808
CG32315-RA	CG32313	0,495795	30,4046	0,0264467	71,6378	0,0559032	0,0299018	0,062211	0,794707	0,6279808
CG32318-RA	dlt	0,0167592	19,1351	30,6381	0,0215507	67,3279	43,1557	0,007017	0,978812	0,6279808
CG32319-RA	CG32318	1,13464	3,70606	7,81241	3,94298	6,92125	10,8852	0,044335	0,715924	0,6279808
CG3231-RA	Naa30B	0,124986	0	0,119994	34,6785	0,0263003	0,347027	0,1375	0,575226	0,6279808
CG32320-RC	CG32320	0,0382384	0,0580502	0,0367111	18,4421	8,75658	0	0,16849	0,409914	0,6279808
CG32333-RA	CG32333	1,88701	1,89134	1,79144	2,821	4,21501	1,8219	-0,409364	0,081123	0,6279808
CG32333-RB	CG32333	0,0635548	0,0578901	0,0610164	0,0739923	2,45968	0,0755341	-0,585662	0,06409	0,6279808
CG32334-RB	CG32334	0	0	0	0	0	0	NA	NA	0,6279808
CG32335-RB	CG32335	6,94074	3,9222	3,62454	4,34605	9,18159	10,1301	-0,150852	0,574952	0,6279808
CG32343-RB	tim	0,0126809	1,30414	0,612985	2,50252	4,21139	0,0138418	0,058683	0,790648	0,13772387
CG32343-RC	tim	1,06774	0,012319	0,0129843	0,898635	64,1693	0,0231427	0,057702	0,793885	0,6279808
CG32344-RA	tim	0,457905	0,0122115	0,830178	0,0139114	0,0176332	0	-0,155645	0,578208	0,6279808
CG32346-RA	tim	0,0135244	0,0120165	0,0126654	0,0137863	1,02364	0	0,254863	0,334177	0,13772387
CG32346-RB	tim	0,75076	0,0196967	0,0207604	0,0135593	0,980977	3,83684	0,254863	0,334177	0,6279808
CG32346-RC	tim	0,0131923	3,2781	6,38075	0,0226703	0,0188431	0,836698	0,02601	0,92505	0,6279808
CG3234-RA	tim	0,021624	1,14246	2,16748	44,9731	0,0186736	62,9407	-0,111471	0,688309	0,6279808
CG3234-RB	Atac3	6,51241	7,94823	6,88369	2,80601	5,01339	6,27339	-0,111047	0,690143	0,6279808
CG3234-RC	Atac3	0,0306379	0,0279072	0,0294143	8,14357	0,0444582	0,0335064	-0,372478	0,234376	0,6279808
CG3234-RE	CG32344	10,3581	7,24384	18,4631	2,95099	10,3725	12,3467	-0,115937	0,676879	0,6279808
CG3234-RF	E(bx)	17,1498	17,8489	13,4919	22,6323	9,55188	8,39051	-0,115937	0,676879	0,6279808
CG3234-RG	E(bx)	0,00757693	0,00690159	0,0072743	0,00768728	0,0104125	0,00784746	-0,111471	0,688309	0,6279808
CG3234-RH	E(bx)	2,92711	2,63138	2,72047	0,00863868	0,0117011	1,27831	-0,111471	0,688309	0,6279808
CG32350-RA	Vps11	15,1739	9,82247	23,6283	4,54874	22,9342	2,81331	-0,415118	0,148151	0,6279808
CG32351-RA	S-Lap2	0,0315097	2,14841	2,96719	3,26382	0,0471099	0,0355049	0,026697	0,938084	0,6279808
CG32352-RA	CG43078	0,820116	0	1,23353	63,4069	34,489	1,28846	-0,934331	5,09E-05	0,6279808
CG32352-RB	CG43078	0,0115666	0	0,0111046	0,0428558	0,0875418	1,8582	-0,952162	3,76E-05	0,6279808
CG32352-RC	CG43078	3,35107	0	5,7175	2,11056	3,24286	0,0131491	-0,936486	4,87E-05	0,13772387
CG32352-RD	CG43078	2,01517	0	0,0120541	0,0118435	0,0160421	6,72177	-0,95056	4,21E-05	0,6279808
CG32353-RA	CG43078	0,273606	0,199376	0,262678	0,0128807	2,31507	0,733048	-1,276987	0,000171	0,6279808
CG32354-RA	CG32354	15,6289	14,5796	16,6905	0,0924769	13,7785	2,87552	0,396206	0,133815	0,6279808
CG32355-RA	CG43078	20,5269	15,5212	16,9011	22,3283	29,3569	28,0059	-0,726989	0,007039	0,6279808
CG32356-RA	ImpE1	0,0766836	0,0698488	0,0736209	0,0382753	0,12526	0,0944039	0,083632	0,808754	0,13772387
CG32356-RB	ImpE1	20,7628	22,009	21,4836	3,71752	4,62339	11,2894	-0,213048	0,502065	0,6279808
CG32364-RA	tut	3,05112	3,38712	65,9362	170,468	54,9302	50,5337	0,419737	0,198496	0,6279808
CG32365-RA	CG32365	5,99502	6,55811	7,2328	19,3774	10,7802	11,2144	0,140909	0,483315	0,6279808
CG32368-RA	CG32368	5,20407	2,6574	8,17562	5,17651	0,00660797	0,00498017	-0,148093	0,674033	0,6279808
CG32369-RA	CG32369	0,0224697	0,0204669	0,0215722	1,71203	0,943833	1,532	0,24226	0,462068	0,6279808
CG32369-RB	CG32369	24,2609	47,6805	16,3701	0,0360158	0,812403	0,0367663	0,24038	0,471098	0,6279808
CG32371-RA	CG32371	0	0	0	0,0465166	0,063007	0,0474859	-0,041811	0,731452	0,6279808
CG32372-RA	ltl	8,97135	11,9542	7,46521	10,7029	4,75934	4,71443	0,360143	0,191197	0,6279808
CG32373-RA	CG32373	0,0349299	24,5681	3,34344	0,150358	0,141272	0,0586557	0,151201	0,657199	0,6279808
CG32373-RB	CG32373	1,1187	13,4838	0,00453974	51,0826	68,8295	85,1943	0,198415	0,55468	0,6279808
CG32374-RA	CG32374	0	0	0	0	1,06078	1,10959	-0,013096	0,914398	0,6279808
CG32376-RA	CG32376	0	0	0	0,141698	0	0	NA	NA	0,13772387
CG32377-RA	Ank2	0,113205	1,73036	3,14385	2,63646	2,77007	2,32828	-0,032798	0,920639	0,6279808
CG32379-RB	CG32379	0,15999	0,072865	0,2304	0,205462	0,587969	0,443129	-0,560763	0,095737	0,6279808
CG32380-RA	Pif1	3,09568	47,0072	39,3157	39,3242	4,56957	38,803	-0,182647	0,367623	0,6279808
CG32380-RB	SMSr	4,16673	20,9917	4,99169	27,4324	11,7178	0,0493678	-0,066446	0,753427	0,6279808
CG32381-RB	SMSr	3,87981	82,664	5,01139	122,5	72,7606	2653,05	0,357739	0,198227	0,6279808
CG32382-RB	unc-13-4A	2,68801	4,22588	2,77296	3,22095	2,11598	1,39729	0,01562	0,897983	0,6279808
CG32383-RB	sphinx2	0	26,5185	53,6259	0	0	3,87386	NA	NA	0,6279808
CG32388-RA	sphinx1	0	0	0	0	29,7752	30,4691	0,14017	0,581117	0,6279808
CG3238-RA	CG32388	0,202894	5,27727	72,5761	21,0399	14,677	22,5995	0,125678	0,652	0,6279808
CG32391-RA	frma	0,176328	0	12,8255	3,87307	1,08984	0,294684	-0,251755	0,335372	0,13772387
CG32392-RA	frma	0,0380256	13,3463	3,80453	0,0382087	0,0194043	0,186322	-0,24694	0,405292	0,6279808
CG32392-RB	CG32391	0,137658	0	2,7708	0	0,258687	0,194962	-0,410508	0,150054	0,6279808
CG32393-RA	CG32392	0,377936	1,30443	26,3764	0,0116323	20,6916	3,01615	-0,191425	0,570022	0,6279808
CG32393-RB	CG32392	0,848364	2,43915	27,3339	6,69325	0,0427135	0,0119063	-0,173288	0,606137	0,6279808
CG32394-RA	dikar	0,931764	0,0389136	0,0416069	1,65808	0,774225	0,594352	0,137861	0,637747	0,6279808
CG32395-RB	dikar	0,0427214	3,34248	0,799795	4,95468	0,0263236	0,0481315	0,013766	0,948533	0,6279808
CG32396-RA	dikar	2,9029	0,73029	3,46549	0,0479031	0,064885	1,82026	-0,041565	0,87689	0,6279808
CG32397-RA	CG32395	0,148019	0	0,0852645	4,8561	9,21631	3647,8	0,071955	0,765265	0,6279808
CG32398-RA	CG32396	0,120211	0,0547482	0	0,147226	0	0	NA	NA	0,6279808
CG3239-RA	jv	3,18847	3,97021	3,21309	4,73753	2,44096	2,4363	-0,468882	0,141058	0,6279808
CG3239-RB	CG32398	0	0	0,286267	0	0	0	-0,498981	0,116998	0,6279808
CG32400-RA	Lcp65Ab1	37,443	11,1775	50,1453	169,348	4954,51	3786,94	-0,465051	0,076344	0,13772387
CG32401-RA	Or65a	0,13173	0,122033	0,20235	0	0	0	0,246847	0,368952	0,13772387
CG32402-RA	Or65b	0	0	0,0779322	0,151406	0	0,0979088	0,01562	0,897983	0,6279808
CG32403-RA	Or65c	0	0,143987	0,0771737	0,206658	0	4,49065	-0,033745	0,890246	0,6279808
CG32404-RA	Cpr65Aw	0,466638	2,76247	4139,91	150,064	5039,75	8,97199	-0,431914	0,117349	0,13772387
CG32405-RA	Cpr65Av	256,418	209,902	260,625	133,136	392,948	326,728	-0,041583	0,896963	0,6279808
CG32406-RA	PVRAP	9,1131	13,4186	0,19824	0	0,176729	0	0,437021	0,050261	0,6279808
CG32407-RA	CG32407	9,54909	66,075	9,51552	0,839857	1,36847	10,9677	0,084504	0,730257	0,6279808
CG32409-RA	CG32409	20,4701	133,538	14,6729	19,6967	0,167865	30,8347	-0,194555	0,547182	0,6279808
CG32412-RA	msl-2	0,0174174	0,015865	36,8907	0	15,8263	0	-0,425242	0,137617	0,6279808
CG32413-RB	msl-2	4,74721	4,60359	0	0	0,349071	0,195719	-0,013765	0,912179	0,6279808
CG32417-RA	QC	34,0885	10,1937	38,7871	40,6426	7,66794	62,6707	0,095363	0,759745	0,6279808

gene_id	Symbol	W1_FPKM	W2_FPKM	W3_FPKM	MCM51_FPKM	MCM52_FPKM	MCM53_FPKM	FC	p-value	p-adj
CG32418-RA	CG32413	0	8,63746	61,8829	31,33	32,743	34,3206	0,178854	0,573306	0,6279808
CG3241-RA	Myt1	1,54972	1,11686	1,63497	19,0374	0,191311	14,5863	-0,395554	0,054073	0,6279808
CG3241-RB	vito	11,2416	6,42033	23,1121	25,6254	0,356261	1,26554	-0,396288	0,053862	0,6279808
CG32423-RA	sob	8,50306	12,4015	7,79901	12,7579	0,0183661	5,36629	0,749893	0,000393	0,6279808
CG32423-RB	shep	39,6578	34,2371	52,8823	32,1591	17,9246	19,732	0,744718	0,000442	0,6279808
CG32423-RC	shep	0,0214346	0,0195242	0,0205785	6,43596	0,0304244	0,0229297	0,724628	0,000616	0,6279808
CG32423-RD	shep	12,2827	10,2993	10,7926	5,00584	5,03804	4,77186	0,763837	0,00018	0,6279808
CG32425-RA	shep	0,0220719	0,0201046	0,0211903	0,0231644	0,0313764	0,0236471	-0,423179	0,06779	0,6279808
CG32425-RB	CG32425	3,12542	5,895	14,5436	0,0348537	5,68082	0,03558	-0,601518	0,044782	0,6279808
CG32425-RC	CG32425	4,80239	3,27258	0,030309	10,5599	6,1935	7,38614	-0,206604	0,303204	0,6279808
CG32425-RE	CG32425	2,60018	2,39785	9,91103	6,49181	0,0341235	4,89918	-0,424034	0,067255	0,6279808
CG32425-RF	CG32425	2,60438	3,42409	3,9073	5,44583	0,0915379	12,333	-0,643042	0,026511	0,6279808
CG32428-RA	CG32425	0,0239001	0,0217699	8,77483	0,0251926	0	0,0257175	0,217226	0,271929	0,6279808
CG32428-RB	CG32428	22,1384	19,1317	27,9013	6,96577	9,5241	9,1904	0,216201	0,274066	0,6279808
CG3242-RA	CG32428	3,29261	8,9141	0,0585389	3,98191	0,0700818	2,82508	0,179327	0,52183	0,6279808
CG32432-RA	CG32432	1,66653	1,77551	3,29944	1,83017	10,5122	1,00664	0,088201	0,737445	0,6279808
CG32433-RA	Gr77a	0,685231	0,825318	0,775339	1,44866	0,0524535	26,096	0,217787	0,513288	0,6279808
CG32434-RA	siz	4,24811	15,7537	3,22772	8,62694	0,0290954	0,0212291	0,223597	0,413398	0,6279808
CG32434-RB	siz	5,94986	0	3,23207	9,30716	0,0433989	0,0214936	0,222485	0,415162	0,6279808
CG32434-RC	siz	19,5986	0,259425	18,0433	21,7332	0,0335968	0,0233354	0,232112	0,395351	0,6279808
CG32435-RA	chb	0,0124164	0,0113097	0,0119205	0,0127388	0,0172548	0,0130042	-0,081952	0,727942	0,6279808
CG32435-RB	chb	3,28255	2,45568	3,77957	7,16249	4,05764	3,45078	-0,057738	0,80716	0,6279808
CG32435-RC	chb	10,4427	11,3312	8,29077	12,1084	6,72117	5,52071	-0,058199	0,805612	0,6279808
CG32436-RA	CG32436	0,232087	0	2,96359	0	4,68774	0	0,305503	0,370758	0,6279808
CG32437-RA	CG32437	0	0	0	0	0	0	NA	NA	0,13772387
CG32438-RA	SMC5	1,04206	64,7701	0,0211057	55,3935	1,13896	0,0191445	0,243864	0,41263	0,6279808
CG32438-RB	SMC5	0,952413	0,0183402	0,0173198	0,0210347	0,0792391	0,632202	0,243864	0,41263	0,6279808
CG32438-RD	SMC5	1,22789	0,018498	1,48779	0,0212244	1,42066	19,2789	0,248074	0,402768	0,6279808
CG32440-RA	Clect27	933,175	0,10134	106,044	55,912	22,399	30,1943	NA	NA	0,13772387
CG32441-RC	CG32440	0	8,55913	8,47548	6,88994	4,85651	5,00875	-0,042697	0,850202	0,13772387
CG32441-RD	CG32441	0,0778583	46,9309	2,38132	0,0941955	740,475	2,38199	-0,039549	0,860653	0,6279808
CG32442-RA	CG32441	45,9617	3,27633	0,0747486	56,8013	0,0277908	2,83352	-0,16869	0,494975	0,6279808
CG32442-RB	Arv1	3,9134	3,98811	4,91548	0,0920891	0,124735	17,513	-0,16869	0,494975	0,6279808
CG32442-RC	Arv1	1,94865	1,46173	5,55379	0,0931304	9,5552	0,0328518	-0,16869	0,494975	0,6279808
CG32443-RA	Arv1	2,19824	2,28333	0,295743	0,575804	0,389755	8,80725	0,30751	0,176816	0,6279808
CG32444-RA	Pc	8,0687	7,18423	6,9021	2,93537	2,21091	1,53467	-0,615707	0,068514	0,6279808
CG32445-RA	CG32444	9,9577	2,92964	0,0715991	0,189047	0,191427	0,0895652	-0,321217	0,175319	0,13772387
CG32446-RA	CG32445	0	0	19,9178	17,8093	28,0053	37,0614	0,693193	0,008958	0,13772387
CG32447-RA	Atox1	49,9429	37,5111	4,81624	15,3297	28,9115	32,8406	0,679449	0,02421	0,6279808
CG32447-RB	CG32447	6,79172	11,1664	5,96621	7,2934	0,0467888	2,92171	0,684577	0,023205	0,6279808
CG32448-RB	CG32447	0,0215817	0,0196581	0,0207197	0,0226236	3,67168	0,023095	-0,312834	0,304285	0,6279808
CG3244-RA	CG32448	0,319719	69,5182	0,306949	5,04494	1,3177	2,16265	-0,128931	0,618618	0,6279808
CG32450-RA	PpN58A	0,0838526	34,8468	0,100121	3,67513	3,42215	0,0996494	0,061137	0,771245	0,6279808
CG32451-RA	CG32450	0	0,178913	0	0	0	0,875141	-0,137777	0,543717	0,6279808
CG32451-RB	SPoCk	1,32294	3,46293	0,0136228	0,0144928	0	5,61302	-0,144497	0,525382	0,6279808
CG32451-RC	SPoCk	0,0141895	0,0129248	6,07373	2,01945	0,112357	0,0150328	-0,138014	0,542991	0,6279808
CG32451-RD	SPoCk	8,64465	5,59808	0,01372	0,012078	1,35018	0,74166	-0,136151	0,547529	0,6279808
CG32452-RA	SPoCk	0,0142908	0,013017	0,0135096	82,2266	91,3561	0,0147948	0,409913	0,191392	0,13772387
CG32452-RB	CG32452	0,0338041	0,0307911	0,730215	0,434095	1,00618	1,16187	0,409913	0,191392	0,6279808
CG32453-RA	CG32452	1,99783	1,75535	3,30989	2,38881	2,68414	90,3027	-0,581548	0,083108	0,6279808
CG32454-RA	CG32453	303,66	272,226	10,121	0,0402633	128,468	0,0411023	-0,431177	0,228042	0,13772387
CG32458-RA	CG32454	0,240568	5,93642	0,0344953	0,0438541	0,0573399	9,09709	-0,257607	0,364126	0,6279808
CG32459-RA	nrm	1,84839	1,9666	2,13245	0,0478335	0,0647908	0,0488303	-0,353464	0,307038	0,6279808
CG3245-RA	CG32459	0,189601	0,0818493	0,0850359	8,51508	0,0358938	1,32909	0,163937	0,420083	0,6279808
CG32461-RA	CG3246	26,8715	26,034	6,87121	9,77644	0,024489	7,27233	0,212247	0,360597	0,13772387
CG32462-RA	CG32461	0,11212	0,170211	20,8924	11,5054	11,773	6,27045	-2,079137	6,26E-09	0,6279808
CG32463-RA	CG32462	0	0,172702	3,02131	0,014726	12,6835	0,0149228	0,01562	0,897983	0,6279808
CG32464-RA	Tengl2	0	2,59307	10,9324	0,116345	11,9105	0	0,223571	0,355361	0,6279808
CG32464-RB	mtd	0,0349238	0,0237359	40,12	0,0159408	0,0224444	29,1525	-0,043309	0,859888	0,6279808
CG32464-RC	mtd	87,2092	1,63907	17,3274	0,0143821	0,0395699	12,6352	0,224903	0,349881	0,6279808
CG32464-RD	mtd	0,0349423	5,64061	0,0406993	0,0141778	0,45981	0,0477217	-0,92324	0,000396	0,6279808
CG32464-RF	mtd	21,0268	0,0197549	59,4501	0,0132645	0,368298	50,8481	0,025338	0,917754	0,6279808
CG32464-RG	mtd	0,0330214	11,0243	4,25841	0,013075	7,7836	5,0233	-0,004607	0,985026	0,6279808
CG32464-RH	mtd	11,9955	4,65262	6,90047	0,0131454	5,1192	32,2763	0,225389	0,351588	0,6279808
CG32464-RI	mtd	0,0163811	4,91441	9,19184	18,7913	4,82561	8,37079	0,227334	0,347614	0,13772387
CG32464-RJ	mtd	0,0154287	6,73482	2,54563	0,0127413	5,29873	5,95697	-0,042562	0,862251	0,6279808
CG32464-RK	mtd	0,0139676	0,031811	8,84872	0,0142699	29,7666	5,68419	-0,003702	0,987956	0,6279808
CG32464-RM	mtd	0,0137754	124,621	7,12526	0,0147987	14,4481	14,1751	0,008697	0,971687	0,6279808
CG32464-RN	mtd	0,0129138	0,0318279	9,60913	0,0165702	5,9868	4,14443	-0,042736	0,861701	0,6279808
CG32464-RO	mtd	0,0127347	0,0263629	1,22746	0,0292135	27,7858	0,0309047	-0,003743	0,987835	0,6279808
CG32464-RP	mtd	0,0128013	0,0300782	0,0276573	0,687874	0,0410061	0,608032	-0,042258	0,863287	0,6279808
CG32464-RQ	mtd	0,0129189	22,5154	6,48613	0,532974	0,831412	8,68392	0,224395	0,353639	0,6279808
CG32464-RR	mtd	0,0124187	0,014921	0,444481	28,1714	9,31397	3,8613	0,00269	0,991247	0,6279808
CG32464-RS	mtd	0,0138621	0,0140535	3,44886	43,1114	4,90756	33,3273	-0,000541	0,998238	0,6279808
CG32464-RT	mtd	0,0143591	0,0127226	33,9266	15,3131	76,5955	78,592	-0,00235	0,99234	0,6279808
CG32464-RU	mtd	0,0160158	0,0125476	55,1005	0,0582328	9,6411	6,04455	-0,042417	0,862719	0,6279808
CG32468-RA	mtd	0,0274782	0,0117628	12,2988	11,6288	8,68102	1,26482	-0,041811	0,731452	0,6279808
CG3246-RA	CG32468	0	0,012579	4,33938	0	0	0	-0,288861	0,201371	0,6279808
CG32473-RA	CG32473	4,44374	0,0210688	17,7728	7,44336	18,7228	13,5628	-0,242472	0,362559	0,6279808
CG32473-RB	CG32473	2,74115	0,410266	0,0176622	6,28372	0,025926	0,0195394	-0,206644	0,415988	0,6279808
CG32473-RC	CG32473	16,0689	2,3693	18,866	14,7277	9,70385	13,6857	-0,215364	0,38907	0,6279808
CG32474-RA	dysf	0,0200068	17,5585	8,77431	1,26951	0,0243288	7,90001	0,294877	0,381541	0,6279808
CG32474-RB	dysf	1,55438	8,31207	0,0802997	0,0142452	7,0839	0,0175236	0,523239	0,108202	0,6279808
CG32474-RC	dysf	0,0138388	0	17,4606	11,7491	3,09741	0,0183356	0,515738	0,112361	0,6279808
CG32475-RA	mthl8	8,80863	9,95322	4,57096	0,0358039	0,0484967	0	4,751357	3,7E-46	0,6279808
CG32476-RA	mthl14	12,3407	12,0658	15,4969	11,6906	13,5769	10,3435	0,131428	0,582079	0,6279808
CG32479-RA	Usp10	5,53578	5,69101	6,77185	8,6873	3,13757	4,66614	0,022466	0,92982	0,6279808
CG32483-RA	Cog3	5,28919	5,83202	18,1382	31,9443	12,0053	20,6038	-0,440581	0,218337	0,6279808
CG32484-RA	CG32483	5,23101	7,17215	14,6818	13,5016	11,0865	160,92	0,102683	0,646219	0,13772387
CG32485-RA	Sk2	4,60375	8,34378	23,2883	20,5859	26,6933	26,5962	-0,202153	0,379967	0,6279808
CG32486-RD	CG32485	14,9694	32,7198	20,5187	20,7355	0,0386949	4,12483	0,104058	0,673975	0,6279808
CG32487-RA	CG32486	61,5262	74,0727	0,0523981	0,0620729	0,243883	0,198115	-0,041811	0,731452	0,6279808
CG32488-RA	CG32487	0	0	0	0	8,61848	0	-0,02376	0,883718	0,6279808
CG3248-RA	CG32488	0	0	0,151618	8,88808	0	8,43495	0,201154	0,436448	0,13772387
CG32490-RA	spoon	18,4003	0,019833	16,2928	0,113554	0,988842	2,1346	0,148146	0,569145	0,6279808
CG32490-RC	spoon	0,0217737	6,03929	0,033857	13,7762	0,901956	13,0966	0,149003	0,566859	0,6279808
CG32490-RE	spoon	9,72037	7,34643	0,237294	0,0228353	1,08186	0,0233111	-0,349749	0,150471	0,6279808
CG32490-RG	cpx	17,8593	35,5651	0,0179521	0,0194685	11,8916	18,685	0,148268	0,56885	0,6279808
CG32490-RH	cpx	0,0179035	63,7076	0,0175887	0,0196896	17,8605	22,6078	0,147749	0,570049	0,6279808
CG32490-RI	cpx	0,0187733	0,0653726	0,0179521	0,0192131	7,2575	0,0612321	0,148243	0,568906	0,6279808
CG32490-RJ	cpx	0,017807	0,0615612	0,0181473	0,307131	88,0419	0,0491514	0,160147	0,540381	0,6279808
CG32490-RK	cpx	0,018351	5240,43	0,0177264	15,0721	17,7129	75,3164	0,155009	0,55236	0,6279808
CG32490-RL	cpx	0,0175702	0,0721947	0,174436	0,0214106	40,768	17,0891	0,156865	0,548235	0,6279808
CG32490-RM	cpx	0,0186989	11,3257	0,0174581	26,1896	24,6744	24,0402	0,148789	0,567396	0,6279808

gene_id	Symbol	W1_FPKM	W2_FPKM	W3_FPKM	MCM51_FPKM	MCM52_FPKM	MCM53_FPKM	FC	p-value	p-adj
CG32490-RN	cpx	0,0183205	14,5352	0,0196602	27,0942	15,4005	0,0416365	0,148699	0,567703	0,6279808
CG32490-RO	cpx	0,0186989	17,0205	43,1343	56,5257	0,0252012	14,34	0,148616	0,567999	0,6279808
CG32490-RP	cpx	0,0189022	0,0163077	30,5097	0,804003	0,0264798	0,487651	0,149088	0,566907	0,13772387
CG32490-RQ	cpx	0,0184638	0,0171	29,8039	0,0431081	0,0250597	0,0440064	0,148591	0,567815	0,6279808
CG32490-RR	cpx	0,181693	0,0162198	0,0359483	9,17297	0,0258584	6,49792	0,148746	0,567668	0,6279808
CG32491-RA	cpx	0,0181843	0,0167154	14,603	0,0149668	0,0247128	0,0152787	-0,034875	0,889472	0,6279808
CG32491-RAA	cpx	0,0204781	0,0160042	0,435904	0,0148756	0,0263703	0,0151855	-0,042196	0,866384	0,6279808
CG32491-RAB	cpx	58,5772	0,0170323	0,0378125	7,60417	0,0258135	5,7218	-0,048391	0,845929	0,6279808
CG32491-RAC	mod(mdg4)	0,0416182	38,5156	0,0399559	47,9582	0,0620434	48,8167	-0,042445	0,866052	0,6279808
CG32491-RB	mod(mdg4)	0,034637	23,3282	0,0332536	0,0458052	0,0507533	20,2039	-0,02299	0,92726	0,6279808
CG32491-RC	mod(mdg4)	0,0303564	9,5717	0,029144	0,0374699	0,0440196	14,9559	-0,050799	0,837238	0,6279808
CG32491-RD	mod(mdg4)	0,0389675	30,6775	0,0374111	0,0324986	0,0577106	0,0467596	-0,036044	0,885388	0,6279808
CG32491-RE	mod(mdg4)	0,042176	59,5855	0,0404915	0,041272	0,0629625	0,0331758	-0,034062	0,89041	0,6279808
CG32491-RF	mod(mdg4)	0,0392685	7,51344	0,0377001	0,0464837	0,0581999	0,0434942	-0,031697	0,89964	0,6279808
CG32491-RG	mod(mdg4)	0,0307234	0,0293783	0,0294963	0,0429676	0,0445914	0,0421321	-0,017613	0,943924	0,6279808
CG32491-RH	mod(mdg4)	0,0308525	0,0379087	0,0296203	0,0329208	0,0447929	0,0474524	-0,001545	0,995079	0,13772387
CG32491-RI	mod(mdg4)	0,0543784	0,0315498	0,0522065	0,0330695	0,0533311	0,0438629	-0,02682	0,915169	0,6279808
CG32491-RJ	mod(mdg4)	0,0407959	0,0276507	0,0391665	0,0393731	0,0606933	0,0336068	-0,040353	0,871797	0,13772387
CG32491-RK	mod(mdg4)	0,037808	0,0354943	0,036298	0,0448084	24,5659	0,0337586	-0,03653	0,882143	0,6279808
CG32491-RL	mod(mdg4)	41,3909	0,0344776	0,0303573	0,0412204	21,0441	0,0401935	-0,039713	0,872954	0,6279808
CG32491-RM	mod(mdg4)	0,041126	0,0384169	0,0394834	71,3131	0,0612346	0,0457421	-0,037911	0,879474	0,6279808
CG32491-RN	mod(mdg4)	0,0331928	0,0357685	0,0318671	0,0452081	0,0484658	0,0420793	-0,033735	0,892161	0,6279808
CG32491-RO	mod(mdg4)	0,0296615	0,027985	0,0284768	0,0357811	0,0429395	36,935	-0,032275	0,896661	0,6279808
CG32491-RP	mod(mdg4)	0,0370657	0,0495316	0,0355853	0,043392	0,054637	0,0365267	-0,027641	0,910773	0,6279808
CG32491-RQ	mod(mdg4)	0,0356652	0,0371598	0,0342408	0,0403372	0,0523919	0,0323618	-0,019397	0,938195	0,6279808
CG32491-RR	mod(mdg4)	0,0321589	107,518	0,0308745	0,0452702	0,046838	0,0411777	-0,038227	0,878764	0,6279808
CG32491-RS	mod(mdg4)	38,5154	0,0374604	100,096	0,0345794	19,1999	0,0394857	-0,025716	0,918078	0,6279808
CG32494-RA	-	0,0411773	0,0344382	0,0395326	0,0386797	0,0613187	0,0442962	0,013049	0,916306	0,6279808
CG32495-RA	-	0,0396217	0,0281026	0,0380392	0,0317012	0,0587748	0,0461501	-0,157095	0,499537	0,6279808
CG32495-RB	-	0,0378513	56,2654	0,0363395	0,0426064	0,0559032	0,0382507	-0,157095	0,499537	0,6279808
CG32495-RC	CG32494	0	0	0,147665	0,113408	0	3,05892	-0,102257	0,641251	0,6279808
CG32496-RA	Gss2	5,25427	4,67063	5,85476	14,3348	32,7078	0,0525954	NA	NA	0,6279808
CG32498-RA	Gss2	8,29839	8,1387	15,1392	20,4861	0,441453	0,242295	0,167473	0,362829	0,6279808
CG32498-RB	Gss2	18,2848	18,5229	25,9872	16,699	0,0272063	0,0205043	0,143166	0,43888	0,6279808
CG32498-RC	CR32496	0,212326	0,309442	0,203846	28,191	30,1592	28,8399	0,304128	0,09599	0,6279808
CG32498-RD	dnc	4,57436	6,33514	4,88927	5,53081	3,9442	2,71859	0,303278	0,097101	0,6279808
CG32498-RE	dnc	0,0162882	0,0212548	0,0156377	0,0168628	0,0228407	0,0172142	0,187643	0,306025	0,6279808
CG32498-RF	dnc	0,0175843	3,84922	4,78603	0,01826	0,0247333	2,53112	0,185692	0,311783	0,13772387
CG32498-RG	dnc	0,0174036	0,0255931	0,0167085	0,0180648	0,0244689	0,0184412	0,184051	0,315641	0,6279808
CG32498-RI	dnc	7,57385	0,0195941	8,24198	5,32747	4,20342	2,30259	0,303546	0,096701	0,6279808
CG32498-RL	dnc	0,0233346	0,0234219	0,0224026	0,0245633	0,0332712	0,0250752	0,210914	0,248023	0,6279808
CG32498-RM	dnc	0,0235425	0,0250707	4,74646	6,79333	3,7707	3,03653	0,169612	0,357331	0,6279808
CG32498-RN	dnc	0,0280974	6,57554	0,0269752	0,0299165	0,0405221	0,0305399	0,184056	0,314607	0,6279808
CG32498-RO	dnc	0,0215114	84,5733	0,0206522	0,0225462	0,0305389	0,023016	0,128232	0,493118	0,6279808
CG32499-RA	dnc	0,0257138	0	0,0246868	0,0272221	0,0368725	0,0277894	0,449402	0,084746	0,13772387
CG3249-RA	dnc	0,027524	0	0,0264246	0,0292654	4,40058	0,0298752	-0,117052	0,604638	0,6279808
CG3249-RB	dnc	7,49782	0	0,0297453	6,07668	0,0449961	6,00855	-0,103176	0,646795	0,6279808
CG3249-RC	Cda4	119,786	0,156899	120,88	0,0650747	0	0,125257	-0,103672	0,645328	0,6279808
CG32500-RA	Os-C	0,141591	0,115743	0,731963	1,34198	0,307516	0,211844	NA	NA	0,6279808
CG32500-RB	Os-C	0,794778	0,979447	0,673266	0,118187	0,0293634	0,930592	-0,024626	0,92837	0,6279808
CG32505-RA	CG32500	10,1896	0,0195115	15,4784	11,6263	14,5741	16,6765	-0,003125	0,98861	0,6279808
CG32505-RE	CG32500	1,24383	0,0187458	1,16273	0,911641	0,0477399	0,0359797	-0,002389	0,991297	0,6279808
CG32506-RB	Pp4-19C	17,3468	18,0283	2,04364	0,0326112	1,43201	0,0332908	-0,768327	0,004283	0,13772387
CG3250-RB	Pp4-19C	0,0297237	0,0270744	13,1663	9,09592	7,97345	6,50787	0,350019	0,326545	0,6279808
CG3250-RC	CG32506	1,02091	7,04913	0,0381995	0,043593	0,0311899	0,0445013	0,378422	0,289602	0,6279808
CG32511-RA	CG3251	0,249186	0,226976	0,0835519	0,509741	8,44361	3,21016	NA	NA	0,6279808
CG32512-RA	CR32511	0	0	0	0	1,04517	0	-0,323774	0,206974	0,6279808
CG32513-RA	CG32512	3,08274	2,57301	4,73631	5,01005	4,16953	3,87125	0,383779	0,108751	0,6279808
CG32513-RB	bves	0,0171063	0,124739	9,83281	0,0345975	0,121427	0,0181135	0,37609	0,116895	0,6279808
CG3251-RA	bves	12,3097	3,42436	0,0986064	0,0338163	0,409384	6,13033	-0,469327	0,18901	0,6279808
CG32521-RA	NAAT1	3,38749	2,81935	3,77883	4,31758	0,24243	0,863956	-0,497964	0,026552	0,6279808
CG32521-RB	CG32521	41,7436	45,2395	0	0,147404	0,0877139	2,20378	-0,497964	0,026552	0,6279808
CG32521-RC	CG32521	0,0382457	0,0348368	1,05458	1,108	0	0,0131878	-0,760553	0,002379	0,6279808
CG32523-RA	CG32521	0,0566364	0,0515883	5,76698	2,15879	0,627953	65,7495	0,103115	0,628485	0,6279808
CG32528-RA	CG32523	0,227065	0,172355	14,0719	13,8565	0	8,11466	-0,404005	0,126942	0,6279808
CG32529-RA	parvin	75,2591	70,927	81,1219	0,123042	192,095	1,33527	0,101875	0,756226	0,6279808
CG32529-RC	Hers	1,89335	0,0407216	6,47587	1,08235	0,0570467	1,86895	0,139125	0,672197	0,6279808
CG32529-RD	Hers	1,36147	0,129267	0,0160478	4,80231	0,0478679	0,047054	0,128419	0,69639	0,6279808
CG3252-RA	Hers	9,15962	0	1,33522	5,58408	0,0557287	0,0429938	-0,057175	0,844107	0,6279808
CG32531-RA	CG3253	3,3168	2,98168	4,16252	8,13297	3,86098	3,35117	-0,747419	0,018992	0,6279808
CG32532-RA	mRpS14	66,1613	46,2674	3,89644	112,387	220,598	3,93	0,502893	0,106966	0,6279808
CG32533-RA	CG32532	1,48678	2,14368	1,23373	0,0236976	14,5715	11,5595	-0,424539	0,054802	0,6279808
CG32534-RA	CG32533	2,90681	3,75309	4,59145	5,13335	3,40168	0,370939	-0,288603	0,380152	0,6279808
CG32536-RA	CoRest	2,48456	0,0352655	5,94719	0,90141	0,0558682	0,0421057	-0,327897	0,153004	0,6279808
CG32537-RA	CG32536	4,21325	5,99876	6,71543	0,0362935	0,0491598	7,49903	-0,155219	0,45289	0,6279808
CG32538-RA	CG32537	5,62455	5,60157	5,65199	5,93287	10,9495	5,73514	0,673807	0,012656	0,6279808
CG32538-RB	nAChRalpha7	0,754999	0,892554	0,879067	0,6575	0,605881	0,543539	0,639631	0,020914	0,6279808
CG32538-RC	nAChRalpha7	1,43601	0,889455	2,3713	0,870573	0,724892	0,653921	0,628941	0,019927	0,6279808
CG3253-RA	nAChRalpha7	0,790486	1,16405	1,10042	0,638498	1,02502	0,661645	0,14612	0,57404	0,6279808
CG32540-RB	Pgant2	43,8921	0	0,0167218	0,0180797	5,69253	0,0184564	-0,120376	0,640816	0,6279808
CG32541-RA	CCKLR-17D3	2,4608	3,34706	2,95315	0,656686	0,487219	0,528449	0,179746	0,519995	0,6279808
CG32544-RA	CG43759	7,52837	9,85745	1,43244	2,85937	0,0920565	0,0583631	-0,920879	0,002265	0,6279808
CG32547-RC	CG32544	5,05987	5,37702	14,2244	15,3434	3,58171	2,95827	0,136242	0,587508	0,6279808
CG32548-RB	CG32547	1,49664	2,17022	1,71763	2,00258	1,28369	1,30163	0,381809	0,12401	0,6279808
CG32548-RC	CG32548	0,0495816	0	38,2222	0,401427	14,0597	14,9573	0,480069	0,059305	0,6279808
CG32549-RA	CG32548	30,7283	0	0,259632	0	0	0,0319014	-0,107159	0,582688	0,6279808
CG32549-RB	CG32549	25,1908	0,689933	0,0232483	14,8589	4,23117	0	-0,107352	0,582043	0,6279808
CG32549-RC	CG32549	10,2637	31,3398	0,022562	9,15048	0,0382145	0,835129	-0,111618	0,566809	0,6279808
CG32549-RD	CG32549	0,0292673	11,1824	0,867916	0	0	0,394904	-0,111038	0,569187	0,6279808
CG32549-RE	CG32549	4,16839	0,0266587	0,547595	0,967589	20,8205	61,0018	-0,153875	0,430263	0,6279808
CG32549-RF	CG32549	0,0242154	0,0231516	9,48925	0,166546	13,9994	5,70865	-0,107627	0,581147	0,6279808
CG3254-RA	CG32549	0,0235006	0,0220571	0,577476	1,43055	0,0423286	7,03371	0,721738	0,001919	0,6279808
CG32551-RB	CG43841	0	12,1505	62,7024	7,8369	0,391713	0,0260764	-0,099373	0,647901	0,6279808
CG32554-RA	CG32554	3,61764	5,31195	7,10334	5,88968	7,57406	5,37662	-0,064682	0,842011	0,6279808
CG32555-RA	RhoGAPp190	0,00905314	0,00824623	0,00869156	0,00921624	1,76061	0,00940828	0,443179	0,077626	0,13772387
CG32555-RB	RhoGAPp190	0,00877074	0,007989	0,00842044	3,60397	1,35122	0,00910888	0,443138	0,078022	0,13772387
CG32555-RC	RhoGAPp190	19,3068	22,2143	21,3043	18,7348	5,51471	10,6145	0,440716	0,079713	0,13772387
CG32556-RA	chas	13,3914	5,93308	5,81957	19,5	10,2235	3,14923	0,067028	0,762041	0,13772387
CG32556-RB	chas	5,2375	2,20952	8,69946	10,2237	3,09686	5,14216	0,116574	0,602478	0,13772387
CG32557-RA	Mco4	2,44207	2,52782	6,28022	81,1619	1,67199	4,34044	-0,597414	0,03784	0,6279808
CG32561-RA	xmas	0,388506	0,517206	0,631212	1,29966	0,303428	0,464336	-0,03708	0,914111	0,6279808
CG32562-RA	xmas	1,20995	0,916037	1,4558	2,18232	0,702965	0,805743	-0,045301	0,884044	0,6279808
CG32563-RA	CG32563	0,193204	0,0611651	0	0,318296	0,431134	0,523392	-0,08332	0,769915	0,6279808
CG32564-RA	CG32564	62,5304	63,5209	6,88181	8,00279	0,527703	17,7202	-0,488829	0,094529	0,6279808

gene_id	Symbol	W1_FPKM	W2_FPKM	W3_FPKM	MCM51_FPKM	MCM52_FPKM	MCM53_FPKM	FC	p-value	p-adj
CG32568-RA	CG32568	2,23014	18,0296	5,61632	4,73901	0,494012	4,02349	1,623978	1,86E-07	0,6279808
CG32569-RB	TwdlZ	5,01049	0,0711703	24,5774	415,127	1,92965	385,597	0,267172	0,356238	0,6279808
CG32570-RA	CG3257	0,01829	0,0207135	5,52743	0,0239039	0,0257688	0,024402	0,605727	0,010072	0,6279808
CG32571-RA	CG3257	7,19151	8,22493	14,44	9,25544	5,06221	10,4727	0,124748	0,599061	0,6279808
CG32572-RA	TwdlY	431,145	0,0997012	8,88389	64,4773	28,2805	62,9897	-0,445787	0,184936	0,6279808
CG32573-RA	TwdlX	61,8549	2782,62	0,0345328	20,6722	8,732	12,6858	0,192496	0,434098	0,6279808
CG32574-RA	CG32572	0,0297952	0,0271395	0,021953	0,0318545	0,0431471	2,96303	-0,511724	0,134378	0,6279808
CG32575-RA	CG32573	0,864635	0,92085	1,08552	0,904867	0,920806	0,694102	0,227951	0,313841	0,6279808
CG32575-RB	Twdlalpha	10,7373	61,5452	0,0130216	0,996689	0,51038	0,663235	0,220866	0,32958	0,6279808
CG32575-RC	hang	1,02861	0,75421	1,25387	3,50045	0,0164593	0,0088605	0,216935	0,339793	0,6279808
CG32576-RA	hang	4,43966	4,33813	5,12776	13,3434	8,54778	2,01304	-0,211654	0,486076	0,6279808
CG32576-RB	hang	2,07741	2,49352	2,72138	41,7348	44,7437	2,69565	-0,146132	0,593053	0,6279808
CG32577-RA	CG32576	0,0409984	0,0256695	65,0519	0,0314741	28,175	46,0127	0,133746	0,619043	0,13772387
CG32578-RA	CG32576	0,102922	0,0373442	0,0393609	0,0304698	0,0610252	0,0459923	-0,011683	0,968885	0,6279808
CG32579-RA	disco-r	10,1498	10,9734	0,0787448	12,6622	0,197121	4,97412	-0,330388	0,180035	0,6279808
CG3257-RA	PIG-Q	2,46184	2,39939	2,31625	4,03975	12,6387	1,75871	0,275553	0,243646	0,6279808
CG3257-RB	CG32579	7,22545	18,908	0,0429207	20,5559	8,63543	8,30483	0,267881	0,256929	0,6279808
CG32580-RA	ase	3,09769	33,4237	7,9891	18,6647	0,0557635	5,18183	0,621673	0,078476	0,6279808
CG32581-RA	Muc14A	0,0434424	0,0389521	0,0280221	0,0578836	12,1786	0,162339	0,026449	0,900539	0,13772387
CG32581-RB	CG32581	1,75571	0,0438144	2,6092	2,61667	0	2,24242	0,026449	0,900539	0,6279808
CG32582-RA	CG32581	11,3141	15,645	16,0967	18,0108	0	14,4312	-0,10142	0,773341	0,6279808
CG32582-RB	lncRNA:CR32582	0,102975	0,562781	0	1,16672	0,0337238	0,0254163	-0,10142	0,773341	0,6279808
CG32588-RA	lncRNA:CR32582	1,41224	1,46178	24,488	15,447	0,352977	0	-0,070526	0,562659	0,6279808
CG3258-RA	CG32588	0	4,46697	0	0	0	0,315974	-0,14531	0,585868	0,6279808
CG32590-RA	IFT54	1,86972	1,32839	2,01045	2,01926	1,86487	1,62791	-0,056883	0,808892	0,6279808
CG32591-RA	CG32590	11,0397	14,4551	58,1583	58,3586	106,76	0,023482	-0,340531	0,286206	0,6279808
CG32592-RA	CG32591	0,1102	27,7798	0,640188	0,164259	0,326024	0,167682	-0,289623	0,299294	0,6279808
CG32593-RA	hiw	1,29364	23,864	41,0292	8,50586	9,57187	11,7686	-0,034282	0,891418	0,6279808
CG32593-RB	Flo2	8,74248	10,7691	0,234084	23,4855	11,366	9,718	-0,034699	0,890118	0,6279808
CG32593-RC	Flo2	11,5599	0,0215054	4,73669	20,3623	9,93932	8,61725	-0,137006	0,575379	0,6279808
CG32593-RD	Flo2	0,0236098	0,0212098	86,8941	0,0248693	0,0336856	0,0253875	0,004695	0,985374	0,6279808
CG32593-RE	Flo2	0,0232853	0,0236149	0,140347	0,0245085	0,0331969	0,0250192	-0,035818	0,886594	0,6279808
CG32593-RF	Flo2	0,0259256	0,0223852	12,3004	0,0274603	0,0371952	0,0280326	-0,036234	0,885297	0,6279808
CG32593-RG	Flo2	0,0245757	27,0438	16,0765	0,0259465	5,12706	0,0264872	0,01127	0,964861	0,6279808
CG32593-RH	Flo2	20,3711	0,0253345	0,0197889	0,0302739	8,47952	12,6477	0,013044	0,959351	0,6279808
CG32594-RB	Flo2	0,0278136	0,902242	6,6404	0,029594	0,0400853	0,0302107	0,168922	0,546886	0,6279808
CG32594-RC	be	7,72934	0,0180163	0,0189893	2,93455	1,00703	1,2322	0,148119	0,59352	0,6279808
CG32595-RA	be	8,59575	35,0661	65,4901	12,9361	5,78553	6,7196	0,19268	0,590275	0,6279808
CG32597-RA	hog	2,05569	0,0410362	0,0432523	16,0101	0,0677414	0,051054	0,118101	0,71951	0,6279808
CG32597-RB	l(1)G0469	10,0947	4,2631	13,0657	24,2665	0,0120097	5,84228	0,147884	0,656636	0,6279808
CG32598-RA	l(1)G0469	8,53131	0,177921	8,32877	5,00514	0,639585	1,69925	0,026052	0,900741	0,6279808
CG32599-RA	betaNACtes6	0,175734	0,256113	0,05109	0	5,47946	0,0116204	-0,894189	0,010114	0,6279808
CG3259-RA	CG32599	1,21208	1,45104	1,86188	73,8086	0,0785989	0,0687412	-0,127408	0,637907	0,6279808
CG32600-RA	Zfrp8	8,33517	0,0166598	0,0230372	0,0190245	6,41806	0,0194209	0,441868	0,061436	0,6279808
CG32601-RA	dpr8	3,94889	4,78179	4,19259	0,290543	6,90541	1,95736	0,025944	0,860432	0,6279808
CG32602-RA	betaNACtes3	0,184364	0,26869	0,0354	0,0113832	11,6551	0,137403	0,149421	0,641774	0,6279808
CG32603-RA	Muc12Ea	0,117872	22,3869	6,88297	12,1829	7,86193	5,07784	-0,056031	0,858716	0,6279808
CG32604-RA	CG32603	5,35015	0	12,0141	237,927	7,44685	0	-0,157677	0,463168	0,6279808
CG32604-RB	l(1)G0007	3,92538	0,0101364	0,0106838	0,067338	0	0,200438	0,031323	0,884684	0,6279808
CG32606-RA	l(1)G0007	6,57726	0,00793947	0,00836823	20,8362	3,04405	0,223085	NA	NA	0,6279808
CG3260-RA	mamo	0	5,49463	4,96856	0	7,55669	5,96482	0,108027	0,665188	0,6279808
CG32613-RA	CG32613	0	8,86139	7,21696	0	0,0295873	3,731	0,01562	0,897983	0,6279808
CG32614-RA	CG32614	0	0	0	0	0	0	NA	NA	0,6279808
CG32616-RA	Ste12DOR	0,192081	0	0	5,41437	0	0	NA	NA	0,6279808
CG32625-RA	CG3262	0,075717	1053,04	1305,08	1649,48	1062,4	1038,5	0,413528	0,191244	0,6279808
CG32626-RA	CG3262	53,0663	0,0214594	0,0226183	0,0248131	0,0336095	0,0253301	-0,14612	0,531813	0,6279808
CG32626-RB	CG32625	4,23563	1,9519	6,01836	3,18506	4,50114	2,86977	-0,110687	0,622246	0,6279808
CG32626-RC	AMPdeam	0,0208726	0,0746937	0,0177561	0,219207	11,6176	0,0225872	-0,115315	0,612356	0,6279808
CG32626-RD	AMPdeam	2,88331	0,0202104	0,0976061	52,6799	0	0,0226709	-0,135109	0,556267	0,6279808
CG32627-RA	AMPdeam	6,91694	0,0516415	0,0213808	0,0218436	0	0	-0,033627	0,912685	0,6279808
CG32627-RB	AMPdeam	6,12673	0	0	6,31181	0	0	0,001427	0,996332	0,6279808
CG32627-RC	Nna1	0,0178455	0,0162549	0,291256	0,0185426	0,386123	0,018929	-0,032044	0,915047	0,6279808
CG32627-RD	Nna1	0,0147009	0,0133906	0,0141138	0,0151631	0,0205385	0,0154791	-0,041071	0,893467	0,6279808
CG32628-RA	Nna1	0,0165493	0,0150743	0,0158883	0,0171436	0,0232211	0,0175008	0,175481	0,546516	0,6279808
CG32628-RB	Nna1	0,758428	0,444593	0,583951	0,581821	0,382335	0,593945	0,041763	0,881682	0,6279808
CG3262-RC	Nna1	0,0238742	0,511037	0,0229207	0,403925	0,720462	0,357559	0,255358	0,186819	0,6279808
CG3262-RD	Nna1	1,26073	0,734415	1,09778	0,685438	0,196462	0,818212	0,255358	0,186819	0,6279808
CG32631-RA	CG32631	0	0,069768	0	0,740333	0,0368573	0,313026	-0,013096	0,914398	0,6279808
CG32632-RB	Tpst	3,8448	4,79716	4,07574	9,71867	1,86099	3,24117	0,016547	0,957625	0,6279808
CG32632-RC	Tpst	5,01377	5,21294	5,01311	7,98581	78,4155	64,8677	-0,018425	0,952517	0,6279808
CG32633-RA	CG32633	38,8614	30,1977	62,6664	31,4148	2,48275	3,02983	-0,41428	0,205655	0,6279808
CG32635-RA	Neto	0,442078	0,37583	0,518737	0,203714	54,9805	0	-0,288839	0,346488	0,6279808
CG32638-RB	CG32638	16,6095	15,6638	14,6782	20,0406	14,4935	17,7483	0,443447	0,028987	0,6279808
CG32639-RA	CG32639	1,92529	0,130714	0,0918484	18,423	0,119961	0,124214	0,787881	0,02768	0,6279808
CG32640-RA	Alp8	0,115517	3,01743	0,0122465	0,73714	0,0179308	3,19674	0,69393	0,017318	0,6279808
CG32641-RA	CG32640	13,4682	12,2677	17,4308	24,938	19,041	0,703655	0,69393	0,017318	0,6279808
CG32642-RC	CG32641	13,4986	12,2954	17,4702	36,1912	19,1202	0,0101233	-0,025091	0,901538	0,6279808
CG32643-RA	CG32642	0	0	0,201867	0	0	0,985364	-0,041811	0,731452	0,6279808
CG32644-RC	CG32643	0	0	0	0	15,07	0,0956805	0,01562	0,897983	0,6279808
CG32645-RB	Mur11Da	0	0,0787781	0	0	1,64971	0,106937	0,637404	0,048949	0,6279808
CG32647-RA	CG32645	26,8907	36,2465	22,0978	30,9653	6,38926	10,1941	-0,575802	0,041407	0,6279808
CG32647-RB	CG32647	2,13301	1,67234	0,048425	0,0343093	0,540678	0,676679	-0,558349	0,046333	0,6279808
CG32649-RA	CG32647	1,6287	0,204438	0,215478	1,03593	0,528365	0,0328678	-0,540537	0,061277	0,6279808
CG3264-RA	Coq8	10,0262	9,74946	13,64	10,6509	17,7555	71,8885	-0,01668	0,949466	0,6279808
CG32650-RA	Eb1	0,0352843	0,0325039	8,24287	0,0382309	0,273339	0,206004	-1,566923	1,17E-05	0,6279808
CG32651-RA	Eb1	0,0356268	58,438	8,34695	37,8744	29,9352	24,4635	-0,041811	0,731452	0,6279808
CG32652-RA	Eb1	10,9373	0,0333504	28,0554	0,0387024	0,0154053	0,0116103	0,341953	0,314872	0,6279808
CG32654-RD	Eb1	47,2669	107,775	160,945	47,5366	0,0142812	0,0107632	-0,401132	0,153496	0,6279808
CG32654-RE	Eb1	0,0549208	0,12653	0,133363	0,0398012	67,6094	53,2303	0,091275	0,743137	0,6279808
CG32655-RA	CG32650	0,176516	0,0803916	0,169466	63,4201	54,4061	43,5288	0,35441	0,30532	0,13772387
CG32656-RA	CG32651	0	0	0	0,0597379	0,335104	0	-0,602681	0,077094	0,6279808
CG3265-RA	lncRNA:CR32652	0,351467	0,533568	0,412414	0,409669	0	0,252555	0,007908	0,97102	0,6279808
CG3265-RB	Sec16	0,00966728	0,00880563	0,00928117	2,39237	0,0134565	0,0101417	0,007812	0,971425	0,6279808
CG3265-RD	Sec16	9,62006	10,4201	8,00729	14,2942	5,46072	5,85139	0,00818	0,970007	0,6279808
CG3265-RE	CG32655	0,731958	4,20665	1,70064	2,26173	0,0629625	1,11912	0,007812	0,971426	0,6279808
CG3265-RF	Muc11A	2,44358	0,266312	1,79685	14,7065	0,0108571	0,352476	0,040057	0,852362	0,6279808
CG32662-RA	rudhira	36,0135	0,0191697	35,3669	5,98549	2,87846	22,3754	0,499173	0,062258	0,6279808
CG32663-RA	rudhira	12,0628	109,645	10,101	3,70152	15,5099	12,3076	-0,395874	0,146813	0,6279808
CG32664-RA	Gr10a	0,134629	0,220733	0,387755	0,877431	0,0375078	0,0990624	0,094254	0,782839	0,6279808
CG32666-RA	Drak	15,1274	11,5155	9,12081	13,7953	9,42202	8,86096	0,131375	0,571705	0,6279808
CG32666-RB	Drak	9,19605	0,012511	10,9576	13,0562	8,43194	7,74372	0,131438	0,57093	0,6279808
CG32667-RA	ssp7	48,0955	29,3671	0,0453444	6,33061	4,03038	244,002	-0,697482	0,037471	0,6279808
CG32668-RA	CG32668	0,994197	4,95171	0,0400063	0,195467	5,86724	0,0591848	0,033991	0,9004	0,6279808
CG32669-RA	CG32669	2,54009	1,58305	3,36918	2,41137	0,0242891	5,10541	-0,65885	0,047406	0,6279808

gene_id	Symbol	W1_FPKM	W2_FPKM	W3_FPKM	MCM51_FPKM	MCM52_FPKM	MCM53_FPKM	FC	p-value	p-adj
CG32670-RA	Mccc2	15,3363	12,3499	19,4733	15,7778	15,3187	15,8715	-0,045962	0,77089	0,6279808
CG32671-RA	Rab9Fb	0,187716	49,701	0,784919	0,00658817	0,00892371	0,970642	NA	NA	0,6279808
CG32672-RA	Rab9Fa	0	1,09955	0,0308444	24,7631	6,51732	0,0352629	0,356531	0,087425	0,6279808
CG32673-RA	Atg8a	238,625	331,026	319,392	313,974	20,843	215,508	NA	NA	0,6279808
CG32675-RA	Rab9E	0	0,376593	0,396931	0,146097	0,341939	0,388986	-0,09737	0,627786	0,6279808
CG32675-RB	Tango5	0,0384908	0,03506	3,0117	0,0420356	3,02475	4,32678	-0,052479	0,794405	0,6279808
CG32675-RC	Tango5	10,6339	9,35145	12,737	20,342	3,21907	12,8413	-0,100576	0,615789	0,6279808
CG32676-RA	Tango5	6,31132	8,41863	8,73711	4,75869	9,80887	0,0538755	0,282879	0,337558	0,13772387
CG32677-RA	stx	25,8925	37,8424	20,4496	36,3408	4,37099	14,834	-0,029468	0,919909	0,6279808
CG32678-RA	ssp7	3,46451	0	0,0340326	0,0381094	2,39491	2,11761	NA	NA	0,6279808
CG32679-RA	Rab9D	0	0	0	0,682621	0,303945	0,420233	-0,244707	0,48039	0,6279808
CG3267-RA	CG32679	0,475055	0,354038	0,248772	0,799858	0,0492684	90,8388	-0,114891	0,630391	0,6279808
CG32681-RA	phtf	11,1041	10,0698	10,7265	11,3166	6,06382	7,34433	-0,31488	0,369511	0,13772387
CG32683-RA	CG32681	0,168076	0,121736	0,119268	0,294348	0,182213	0,234924	-0,306858	0,184266	0,13772387
CG32685-RC	CG32683	1,43895	0	1,73918	0,188008	1,63471	1,32083	0,08796	0,718745	0,13772387
CG32686-RA	ZAP3	10,2179	1,38796	0,0256034	86,036	0,591273	0,772479	-0,496531	0,155707	0,13772387
CG32686-RB	CG32686	0,0227013	0,021841	0,0871783	0,180173	0,171788	0,131276	-0,485501	0,172812	0,6279808
CG32686-RC	CG32686	0,0239782	0,10179	0,0230205	0,0252796	0,0342413	0,0258063	-0,483497	0,171363	0,6279808
CG32687-RA	CG32686	0,0745006	0,245325	0,0625844	0,0833631	0,120524	0,0752635	0,285889	0,230814	0,6279808
CG32688-RA	CG32687	17,0187	8,29925	10,7381	13,0676	5,07155	6,52937	-0,18459	0,49412	0,6279808
CG32688-RB	Hk	0,822011	0,662682	5,02966	0,0117422	3,69139	0,0119869	-0,249985	0,367033	0,6279808
CG3268-RA	Hk	2,30263	1,40972	0,0110121	7,88863	0,0183586	5,54197	0,248047	0,236292	0,6279808
CG32690-RA	Rab2	78,7585	82,2768	0,114095	0	6,11157	0	0,143499	0,604688	0,6279808
CG32691-RA	lncRNA:CR32690	0,298087	0	0,150229	0,367075	0,189809	0,264396	NA	NA	0,6279808
CG32693-RA	CG32691	0	0,320141	0,787336	0	0	0	-0,013952	0,911565	0,6279808
CG32694-RA	Gr9a	0	5,63833	4,77002	7,79604	0,0383293	3,88854	0,525025	0,030509	0,6279808
CG32694-RB	CG32694	0,0513144	0,0467407	13,4967	1,9115	5,5597	7,65113	-0,166547	0,41018	0,6279808
CG32694-RC	CG32694	44,361	0,0795414	2,04482	0,544408	1,92395	1,9354	0,524721	0,030472	0,6279808
CG32694-RD	CG32694	86,4636	134,67	11,1768	1,64803	6,28703	0,0590808	-0,182319	0,37739	0,6279808
CG32695-RA	CG32694	0,0266686	0,0242916	0,0492648	0	15,2917	65,6666	-0,505053	0,07779	0,13772387
CG32697-RA	CG32695	6,3862	7,83355	10,3003	25,2904	5012,77	4884,19	0,048482	0,80291	0,6279808
CG32697-RB	Ptpmeg2	0,0226391	0,0200845	11,708	7,4455	5,24178	3,78211	0,04907	0,800795	0,6279808
CG32697-RC	Ptpmeg2	3,71539	14,1321	1,15164	11,6503	0,0317785	7,61144	0,051226	0,792385	0,6279808
CG32697-RD	Ptpmeg2	9,52676	0,0203493	4,04521	0,0234613	11,839	0,0239502	0,048112	0,802467	0,6279808
CG32697-RE	Ptpmeg2	0,0223405	0,0247105	87,3588	8,56515	12,5021	10,8255	0,046908	0,806644	0,6279808
CG32697-RF	Ptpmeg2	6,03608	26,9449	2,67612	17,0953	79,7337	8,77803	0,05121	0,792636	0,6279808
CG32698-RA	Ptpmeg2	15,5197	2058,19	2,80604	3654,22	50,945	58,1029	0,443282	0,177873	0,13772387
CG32699-RA	CARPB	0,792911	0,126442	0,3776	0,050549	0,0674343	0,0508226	-0,225679	0,38979	0,13772387
CG32699-RB	LPCAT	10,9705	8,88444	10,9632	13,0843	6,7673	7,58378	0,072343	0,742841	0,13772387
CG3269-RA	LPCAT	0,031114	0,0283408	0,0298713	0,033371	0,0452012	0,0340663	0,280124	0,145616	0,6279808
CG32700-RA	CG3270	5,46423	0,0418252	7,53835	7,58408	7,66885	6,69056	0,484238	0,039658	0,6279808
CG32700-RB	Dsim\GD16059	0,0366342	0,0333689	0,035171	0,0398252	0,0539435	0,0406551	0,484238	0,039658	0,13772387
CG32701-RA	Dsim\GD16059	5,6542	5,70205	6,53872	5,33946	3,47285	3,90894	-0,175684	0,449427	0,6279808
CG32702-RB	l(1)G0320	168,073	201,892	191,104	324,865	135,251	172,128	-0,554588	0,009215	0,6279808
CG32703-RA	Cubn	1,68843	1,78189	1,64335	3,1987	1,95583	1,78363	0,203798	0,520736	0,6279808
CG32704-RA	Erk7	0,453444	1,04733	0,981234	0,570605	6,63344	6,73761	0,100134	0,47135	0,6279808
CG32706-RA	Ir8a	0	0	42,1847	8,82882	5,33413	6,98456	0,274045	0,443024	0,6279808
CG32707-RA	CG32706	1,58582	12,7155	21,2573	1,94829	2,12945	1,56934	-0,128448	0,652287	0,6279808
CG32708-RA	APC4	1,28523	0,98704	1,70568	22,7648	19,1988	24,2089	-0,514152	0,050321	0,6279808
CG3270-RA	Dsim\GD16928	16,8292	0,255027	3,23304	246,077	312,05	296,366	-0,089477	0,709822	0,6279808
CG32710-RA	PGAP3	0,051716	0,0471065	9,40187	7,76105	12,1957	14,8792	0,01562	0,897983	0,6279808
CG32711-RA	PGAP3	4,42424	3,71046	1,68811	0,0583877	0,0790864	8,35577	0,082013	0,773372	0,6279808
CG32712-RB	CG32710	0	7,54865	0	104,127	17,2206	0	-0,029407	0,93298	0,13772387
CG32713-RA	lawc	28,7152	4,66596	18,4382	88,3704	26,4152	23,081	-0,172994	0,564388	0,6279808
CG32714-RC	Dsim\GD24571	0,493104	0,299435	0,789015	1,55915	0,720219	7,60274	-0,070184	0,767385	0,6279808
CG32714-RD	CG32713	0,297639	0,0269331	0,628653	0,451623	3,91875	0,455209	-0,241959	0,309279	0,6279808
CG32714-RE	CG33181	0,02194	0,0199844	0	0,152955	0,0311791	0,0234984	-0,249195	0,295675	0,6279808
CG32714-RF	CG33181	0,0227482	0,0207206	0,0581989	0,0844256	0,0323898	0,0244109	-0,070683	0,765865	0,6279808
CG32714-RG	CG33181	0,0297818	0,0271273	0,504356	0,229113	0,0431263	0,0325026	-0,002926	0,990268	0,6279808
CG32717-RA	CG33181	11,361	11,6068	0,185488	0,15147	10,3652	11,5487	0,091402	0,747363	0,6279808
CG32717-RB	CG33181	0,0320808	0,0292214	0,659134	0,602511	3,69249	0,0352075	0,032324	0,911485	0,6279808
CG32717-RD	sdt	1,6175	1,58973	12,422	8,03548	8,90765	7,63262	0,088081	0,758013	0,6279808
CG32717-RE	sdt	3,99096	3,44251	39,3691	47,0092	21,0195	26,1282	0,048274	0,867201	0,6279808
CG32717-RF	sdt	0,0115524	11,7006	8,77388	12,139	3,55453	3,50229	0,046334	0,873233	0,6279808
CG32717-RG	sdt	0,0150663	57,3264	21,558	17,6341	4,4198	5,32835	0,036415	0,899232	0,6279808
CG32717-RH	sdt	0,01263	5,12641	1,19098	1,16271	0,041531	0,571744	0,062985	0,827031	0,6279808
CG32718-RB	sdt	12,0622	7,766	8,11321	0,0306614	0,617079	0	0,01562	0,897983	0,6279808
CG32719-RA	sdt	4,3858	39,8525	2,14592	3,21354	3,26456	2,63935	0,763618	0,028121	0,6279808
CG3271-RA	CG32718	0,102922	0,0750829	5,81278	0,0883734	3,93852	0	-0,057305	0,832474	0,13772387
CG3271-RB	CG32719	0,180387	0	0	0,0412462	0,0558682	0,0421057	-0,057305	0,832474	0,6279808
CG32720-RA	CG32720	0	0,624375	0,112569	0,0398976	0,0540415	0,040729	NA	NA	0,13772387
CG32721-RA	NELF-B	6,08236	6,15887	246,388	9,09096	7,0613	6,43815	0,088336	0,731637	0,6279808
CG32726-RA	CG32726	1,60096	0,68706	37,0325	0,0504329	0,0683117	0,0514838	-0,610727	0,087938	0,6279808
CG32727-RA	CG32727	0	0,0612066	0	0,0241758	0,0327463	0,0246796	NA	NA	0,6279808
CG32732-RA	sced	4,23872	3,92481	54,179	3,93707	2,45392	42,549	0,251033	0,235302	0,6279808
CG32733-RA	Setd3	16,6482	4,28149	24,5694	12,778	1,17116	1,97924	-0,087973	0,472205	0,6279808
CG32736-RA	CR32733	0	0	0,0116533	12,447	2,8582	0,0122645	-0,458878	0,104625	0,13772387
CG3273-RA	CG32736	28,4067	32,9367	0	0,374224	1,07613	1,19355	0,520217	0,021316	0,6279808
CG32741-RA	Bap170	3,23975	3,63634	4,24464	30,4886	19,4874	24,7101	0,694028	0,001095	0,6279808
CG32742-RA	CG32741	8,70058	0,0455268	9,34206	6,68764	7,7936	4,388	0,07965	0,79043	0,6279808
CG32743-RA	Cdc7	3,7315	5,72447	4,29476	7,52605	20,9609	19,6513	-0,129962	0,620643	0,6279808
CG32744-RA	nonC	2,92473	6,07813	5,35823	7,70147	0	0,953632	0,260245	0,311029	0,6279808
CG3274-RA	Ubi-p5E	169,904	142,459	234,099	0	0,255986	46,3958	0,113642	0,711456	0,6279808
CG32750-RA	CG32750	1,13489	0,0961445	2,46161	1,10105	1,54057	0,947512	0,194684	0,566236	0,6279808
CG32751-RA	CG32751	0,168884	0,804018	0,19234	0,920849	18,2943	0,674654	-0,244235	0,461803	0,6279808
CG32754-RA	vanin-like	0,372009	0,145222	2,39801	1,08893	7,24497	0,426831	0,139839	0,689682	0,6279808
CG32755-RA	CG32755	0,615805	0,224367	23,2229	4,2959	37,6116	1,9622	-0,404093	0,24632	0,6279808
CG32756-RA	CG32756	5,64148	5,15471	6,43169	6,28713	5,64763	4,45112	-0,05236	0,81561	0,6279808
CG32758-RA	Snx27	7,84088	5,20898	1,25814	31,002	23,6905	1,1091	0,144651	0,601302	0,6279808
CG32762-RA	CG32762	0	0,239469	0,210334	0	1,2899	0,0390049	-0,161466	0,528006	0,6279808
CG32763-RA	l(1)G0045	30,8278	9,42641	11,8105	6,84455	7,17349	8,23306	0,661454	0,014535	0,13772387
CG32767-RA	CG32767	0,00882581	7,26979	0,0084733	8,63991	0,0121636	0,00916723	0,193515	0,471885	0,13772387
CG32767-RB	CG32767	5,61283	0,0201923	6,03413	0,404398	3,19633	3,57341	0,178907	0,506814	0,6279808
CG32770-RA	CG3277	5,52026	6,93287	2,53944	0,543093	6,46024	13,3576	0,008676	0,975719	0,6279808
CG32771-RA	CG42594	2,19704	0,0810702	0,136717	0,0522734	7,50329	0,03359	-0,360846	0,287114	0,6279808
CG32772-RA	CG42594	1,0455	1,95118	1,97746	0,0469809	6,69906	186,887	0,31137	0,168326	0,6279808
CG32772-RB	CG32772	6,7744	7,53813	0,0333234	0,0375554	100,82	0,0383379	0,34282	0,13309	0,6279808
CG32773-RA	CG32772	7,75398	9,51304	0,0352296	0,0398976	0,050869	0,040729	0,231475	0,46116	0,6279808
CG32774-RA	lncRNA:CR32773	0,786618	5,75932	0,60416	10,8751	0,0437177	10,1375	0,000633	0,998469	0,6279808
CG32775-RA	Muc4B	0,113767	0,124352	0	0,170438	0	0,393329	0,150882	0,464914	0,6279808
CG32776-RA	GlcAT-I	10,4116	10,2853	12,9429	41,6262	7,56036	8,87638	0,228865	0,404807	0,6279808
CG32776-RB	CG42541	0,877936	0,856133	0,0198322	0,0216072	145,513	0,577399	0,206742	0,460129	0,6279808
CG32776-RC	CG42541	0,020638	0,0187985	0,960963	1,03721	0,51103	3,34908	0,228865	0,404807	0,6279808
CG32776-RD	CG42541	0,454226	0,858128	0,0161513	0,0174385	0,0292384	0,497851	0,206742	0,460129	0,6279808

gene_id	Symbol	W1_FPKM	W2_FPKM	W3_FPKM	MCM51_FPKM	MCM52_FPKM	MCM53_FPKM	FC	p-value	p-adj
CG32778-RA	CG42541	0,0168103	0,015312	0,621349	0,861973	0,610124	72,624	-0,272537	0,394867	0,6279808
CG32779-RA	CG43689	0,779966	0,0240201	0,949987	2,42547	26,5527	0,804593	-0,057736	0,681327	0,6279808
CG3277-RC	fd3F	0,303607	0,45253	0,158989	0,0303436	0,0411005	0,673519	0,066038	0,791466	0,6279808
CG32783-RA	Tif-IA	42,4023	28,7842	48,4974	1,05758	0,563387	33,1076	0,042907	0,894852	0,6279808
CG32786-RA	CG32783	0,610457	0,645014	0,785341	1,17467	18090,6	0,233255	0,06222	0,728618	0,6279808
CG32788-RB	CG32786	0,671503	0,567168	0,785341	1,09458	36,4439	0,134425	-0,037875	0,76416	0,6279808
CG32789-RA	Crg-1	6,22225	6,01289	4,03303	40,175	24,3658	0,368578	0,398524	0,18784	0,6279808
CG3278-RA	HIP	15,5388	14,9295	0,0138006	0,0350018	0,0474102	0,0357312	-0,038434	0,876075	0,6279808
CG32790-RA	Vti1a	9,05136	19,9996	21,525	0,217934	77,9537	0,222475	-0,262311	0,431731	0,13772387
CG32791-RA	CG43689	0,61638	8,03751	0,852136	8,1891	1,10704	0,783239	-0,421948	0,212674	0,6279808
CG32792-RE	DIP-alpha	0,220928	3,64956	0,313984	0,639134	0,336903	0,229714	0,612952	0,030239	0,6279808
CG32792-RF	ppk8	0,0366139	0,0333504	0,0351515	0,0398012	0,0539109	0,0406305	0,617158	0,028744	0,6279808
CG32793-RA	ppk8	0,0349115	0,0317998	0,0335171	0,0377923	0,0511899	0,0385798	-0,747633	0,033808	0,6279808
CG32795-RA	CG32793	0,203076	0,0189405	0,0600727	0,021757	0	0,117641	-0,141777	0,598852	0,6279808
CG32795-RB	CG32795	29,1106	24,4556	46,0385	25,4896	0,0294701	0,0230318	-0,140895	0,601461	0,13772387
CG32795-RC	CG32795	0,0368795	0,0335924	0,0354065	5,72747	0,0309305	0,0228829	-0,142203	0,597836	0,6279808
CG32796-RA	CG32795	0,0356652	0,0324864	0,0342408	0,0386797	12,2852	0,0262699	0,356972	0,254993	0,6279808
CG32796-RB	boi	2,58084	3,52981	2,49292	6,37983	1,14312	2,35948	0,254979	0,420259	0,6279808
CG32796-RD	boi	0,0146121	0,0133097	0,0140285	0,0152544	0,0206622	0,0155723	0,254984	0,419467	0,6279808
CG32797-RA	boi	4,94718	7,80564	4,04941	4,52756	1,86596	2,54721	0,01562	0,897983	0,6279808
CG3279-RA	CG32797	0	0	0,156248	7,8793	1,15706	1,23667	-0,07522	0,739251	0,6279808
CG32801-RA	Tmc	1,54943	1,89695	1,87143	1,67494	2,04062	1,75691	-0,443261	0,184632	0,6279808
CG32803-RA	CG32801	1,10819	1,32486	4,52993	3,76608	1,90431	5,32752	0,021324	0,942176	0,6279808
CG32803-RB	CG32803	0,0890908	0,811501	4,78945	1,53779	4,31619	2,90246	0,013014	0,964366	0,6279808
CG32806-RA	CG32803	5,63894	4,49898	8,04653	8,30965	3,39933	7,11163	-0,013096	0,914398	0,6279808
CG32806-RB	CG32806	0	0	0	0,68786	0,258687	0	-0,013096	0,914398	0,6279808
CG32808-RA	CG32806	0	0	0	0,69577	0	0	-0,613667	0,06506	0,6279808
CG32809-RB	CG32808	2,78214	0,0177506	0,0906669	18,5944	4,16205	0,122184	-0,177777	0,585235	0,6279808
CG32809-RD	CG32809	2,37658	3,14696	0,0154661	0,0174385	0,0245699	1,39096	-0,053048	0,863917	0,6279808
CG3280-RB	CG32809	0,024878	0,0226606	0,0206926	7,64146	0,0225807	0,0274156	0,187175	0,401594	0,6279808
CG32810-RB	CG3281	0,0364523	8,11469	0	1,99338	5,12443	3,01276	0,133521	0,559569	0,13772387
CG32812-RA	inc	20,8028	19,3443	24,8509	27,3604	0,723669	15,6792	-0,216823	0,535436	0,6279808
CG32813-RA	CG32812	0,877114	0,932092	0,982428	1,2217	1,5444	1,24715	0,350508	0,252375	0,13772387
CG32813-RB	CG32813	9,27862	4,40661	6,8657	4,43102	0,0275336	4,77484	0,352707	0,249572	0,6279808
CG32813-RC	CG32813	0,0163406	0,0439745	0,0156879	10,1809	0,0236581	2,62628	0,35313	0,249299	0,6279808
CG32813-RD	CG32813	5,73698	2,76994	4,2217	0,11046	0,0343203	0,0551567	0,330473	0,282174	0,13772387
CG32813-RE	CG32813	10,1277	2,49897	9,35725	0,11969	34,3875	3,24857	0,328142	0,28539	0,6279808
CG32813-RG	CG32813	0,0194876	3,34966	0,0187092	3,24326	0,0358938	6,49101	0,350388	0,252243	0,6279808
CG32815-RA	CG32813	0,0168489	0,989246	0,016176	4,02295	80,2613	0,0172716	-0,343127	0,218767	0,13772387
CG32816-RA	CG32815	1,75365	6,15509	1,87068	6,07717	0	0,0361731	0,583868	0,019094	0,6279808
CG32816-RB	CG32816	25,0569	10,2091	3,00952	6,28358	4,11902	3,04984	0,584299	0,018919	0,6279808
CG32816-RC	CG32816	9,26209	6,31122	29,8239	9,51067	5,38415	32,3656	0,620338	0,012519	0,6279808
CG32816-RD	CG32816	0,0426387	0,0383679	46,2338	93,16	33,4087	2,6931	0,6201	0,012714	0,6279808
CG32817-RA	CG32816	0,0421223	17,4535	0,0364649	0,0207761	0,0281413	0,917102	0,500889	0,157186	0,6279808
CG32817-RB	CG32817	0,116948	0,106525	8,92379	11,4145	9,95479	9,63004	0,500889	0,157186	0,6279808
CG32819-RA	CG32817	2,1088	1,92085	0,112278	0,18176	0,246195	0,185547	NA	NA	0,6279808
CG32819-RB	CG32819	0,860064	0,83041	1,20554	0,976253	1,12181	0,832873	NA	NA	0,6279808
CG3281-RA	CG32819	0,761803	0,896291	1,4018	0,801549	1,83479	1,55069	-0,143814	0,533053	0,6279808
CG32820-RA	CG32820	1,18591	0,758063	1,75958	1,18194	1,22586	1,48536	NA	NA	0,6279808
CG32820-RB	CG32820	0,616475	15,1334	1,09493	0,645621	1,84362	15,7039	NA	NA	0,6279808
CG32821-RA	CR32821	8,30954	6,65699	3,83759	2,44203	0	3,94136	NA	NA	0,6279808
CG32823-RB	Sdic3	6,95245	7,32541	6,89991	7,42275	3,169	5,49232	-0,041811	0,731452	0,6279808
CG32825-RA	Or19b	0,170299	0,0517066	1,35559	0,0421162	2,02592	0,0179348	0,327339	0,135276	0,6279808
CG32829-RA	CG32829	0,374899	53,2172	0,494897	1,24268	0,429129	0,535548	-0,250057	0,452313	0,6279808
CG32830-RA	Dpse\GA17170	90,9497	0,0321394	0,0351515	118,789	0,0539109	0,0406305	-0,088093	0,786143	0,6279808
CG32832-RA	ab	0,158455	0,144332	0,0255622	0,0140718	10,299	20,3651	0,01562	0,897983	0,6279808
CG32833-RA	CG32832	0	0,187302	18,4887	0,380075	0,0418326	0,0315276	NA	NA	0,6279808
CG32834-RA	CG32833	0	6,12369	0,0426323	0,0560055	0	0	0,044335	0,715924	0,6279808
CG32835-RA	CG32834	0	10,7065	0,046002	0,0512967	11,2618	0	-0,070526	0,562659	0,6279808
CG3283-RA	lncRNA:CR32835	0	0	0,0408742	0	0	0	-0,093003	0,712075	0,6279808
CG32843-RA	RpII15	16,424	1,50947	27,7992	6,12356	19,0302	6,10489	0,644254	0,012609	0,6279808
CG32845-RA	Dh31-R	6,29005	10,4456	21,3966	5,65733	4,06267	0,174969	-0,427472	0,139974	0,6279808
CG32846-RA	CG32845	1,99195	1,39969	1,74847	3,33204	76,6289	53,4033	-0,496144	0,164023	0,6279808
CG32847-RB	CG32846	0,4064	15,1531	10,993	14,7697	3,45019	19,3018	-0,373574	0,296604	0,6279808
CG32848-RA	CG32847	0,734177	1,21589	0,512621	2,13479	1,87512	1,72461	0,545736	0,017948	0,6279808
CG32849-RA	VAChT	18,0707	20,0023	14,4112	14,1988	6,5799	5,39303	0,780958	0,026108	0,6279808
CG3284-RA	Hex-t2	0,43471	0,137831	0,715453	0,10599	0,207003	0,15601	-0,014043	0,958249	0,6279808
CG32850-RA	CG3285	4,80776	0,08525	5,78701	0	0,993824	3,47518	-0,389588	0,202169	0,6279808
CG32853-RA	CG32850	35,4328	0,0223023	0,0235067	0,0250394	0,033916	35,265	-0,041811	0,731452	0,6279808
CG32854-RA	mthl12	0	0	0	18,2301	0,996767	0	-0,35924	0,250548	0,13772387
CG32855-RA	mRpS21	102,495	61,1845	21,3633	103,148	10,0039	14,6191	0,802859	0,013975	0,6279808
CG32856-RA	CG32855	28,5199	0,00930287	0,120449	6,66559	17,061	4,62299	-0,011786	0,963517	0,6279808
CG32857-RA	CG32856	25,4161	18,0731	26,8501	35,2544	0,997692	5,32846	NA	NA	0,13772387
CG32858-RA	CG32857	11,1615	15,1338	16,4409	12,17	13,7928	15,7558	-0,010168	0,965724	0,13772387
CG32858-RB	sn	17,5264	19,6524	0,274618	12,2923	2,07619	0,0771162	-0,022543	0,923669	0,6279808
CG32858-RC	sn	12,9786	13,2177	3,13578	25,8185	0,063523	2,66232	0,027804	0,90716	0,6279808
CG32859-RA	sn	6,55394	0,0236427	9,17524	12,9672	3,8451	2,16005	0,206435	0,563694	0,6279808
CG3285-RA	eIF4E7	0,553709	0,821914	0,307946	2,85051	0,640337	0,349705	0,403081	0,13199	0,6279808
CG3287-RB	koi	86,0036	99,5947	110,542	1,10322	2,76881	0,606961	-0,460916	0,031777	0,6279808
CG3287-RC	koi	0,0243672	0,0221954	0,023394	2,10521	0,0201855	1,72537	-0,462334	0,031364	0,6279808
CG32885-RA	CG3288	0,533653	0,927985	0,512338	33,0774	2,00347	1,94898	-0,608984	0,055208	0,6279808
CG32885-RC	pgc	0,0969326	0,0882929	12,8911	0,123735	0,1676	0,126314	-0,401078	0,176686	0,6279808
CG3288-RA	pgc	1,69095	1,64833	10,0724	3,16471	0,0925337	0,069739	-1,059617	0,002239	0,6279808
CG3289-RA	Ptpa	5,51827	0	0,143739	0,346462	3,66454	0,301617	0,134716	0,602422	0,6279808
CG32904-RA	Alp2	0,0562508	9,20977	2,16008	0,0633769	2,8294	2,57627	-0,183246	0,554035	0,6279808
CG3290-RA	seq	0,0184209	2,0641	0,0185669	14,1641	0,574962	2,90579	-0,013096	0,914398	0,6279808
CG3291-RA	pcm	5,03654	4,03463	5,11491	26,382	15,7723	18,0319	-0,06162	0,788488	0,6279808
CG3292-RA	Prx5	0,233759	0,278641	14,6844	1,83736	1,86437	1,96541	0,719627	0,018752	0,13772387
CG32937-RA	FoxP	2,51088	0,733065	2,47403	3,45731	2,62837	0,899737	-0,216041	0,446459	0,6279808
CG32939-RA	CG32939	2,51088	2,76858	4,60974	7,9471	5,04734	4,43585	-0,546664	0,102145	0,6279808
CG32944-RE	CG3294	2,43182	2,09086	2,55289	2,11615	0,0265744	2,73865	-0,124467	0,551282	0,6279808
CG32944-RF	CG3294	0,796003	1,11866	0,938887	7,57596	0,0273312	0,0205985	-0,074777	0,727679	0,6279808
CG32945-RA	CG32944	0,0393855	4,76489	0,0176181	0,0194685	6,20105	30,418	NA	NA	0,13772387
CG3294-RA	CG32944	6,6563	0,153537	0,0168685	0,0190575	0,563432	9,14638	-0,878558	5,3E-05	0,13772387
CG3294-RB	CG32945	0	0	0	1,59538	1,75682	9,00865	-0,864785	7,98E-05	0,6279808
CG3295-RA	CG3295	24,3944	5,89611	28,5816	34,1463	23,4564	25,089	0,013838	0,946829	0,6279808
CG32970-RA	mnd	14,2694	11,8775	16,6216	20,4442	0,0412907	5,85017	-0,174658	0,603862	0,6279808
CG32970-RB	mnd	16,8637	16,0595	68,195	45,8658	13,7132	10,6892	-0,167746	0,618199	0,6279808
CG32971-RA	mnd	19,1947	14,3274	3,16375	2,62591	14,2538	17,0404	-0,65654	0,053724	0,6279808
CG32971-RB	wb	0,745477	2,22007	0,466039	2,07628	0,0203419	0,153741	-0,666727	0,04937	0,6279808
CG32971-RC	wb	0,281351	3,78001	0,465196	0,0335408	2,343	280,203	-0,666727	0,04937	0,6279808
CG32972-RA	CG32971	1,0105	3,36623	0,0808453	2,13249	0,020009	0,507469	0,258668	0,457955	0,6279808
CG32972-RB	CG32971	0,0913068	203,003	0,08766	0,11469	4,59416	61,7918	0,495075	0,152259	0,6279808
CG32975-RA	CG32971	1,118	0,0181521	1,63336	0,124202	0,020027	310,191	0,118226	0,624013	0,6279808

gene_id	Symbol	W1_FPKM	W2_FPKM	W3_FPKM	MCM51_FPKM	MCM52_FPKM	MCM53_FPKM	FC	p-value	p-adj
CG32975-RB	CG43333	7,374	5,79083	449,332	14,3167	12,6636	10,3873	0,203046	0,388228	0,6279808
CG32975-RC	CG43333	43,516	55,6606	0,108131	52,163	0	0,132682	0,204038	0,38539	0,6279808
CG3297-RA	nAChRalpha5	0,0338908	0,0308701	4,37209	9,3546	4,03205	0,0254355	0,331068	0,093701	0,6279808
CG3297-RC	nAChRalpha5	0,023652	0,0215439	0,0325372	0,0365959	0,0495694	3,0786	0,332662	0,091986	0,6279808
CG3297-RD	nAChRalpha5	5,2682	5,72469	0,0227073	0,0249163	0,0337493	3,82616	0,343796	0,078102	0,6279808
CG32982-RA	RNaseZ	3,42069	0,40326	0,796945	4,64012	3,95997	2,99626	0,194166	0,422758	0,6279808
CG32982-RB	CG32982	11,198	14,5793	9,72629	17,1144	0,0812462	0	0,249756	0,299388	0,6279808
CG32982-RC	CG32982	3,72028	6,521	4,18264	6,67626	7,09702	9,3588	0,242508	0,31689	0,6279808
CG32983-RA	CG32982	12,636	17,6887	12,4249	10,3461	8,31772	2,7868	-0,041811	0,731452	0,6279808
CG32984-RA	CG32983	0	25,9802	45,6805	23,6721	33,8291	0,0209188	-0,291864	0,414659	0,13772387
CG32985-RA	CG32984	0,316242	0,813331	11,6586	1,06879	5,39784	7,23272	-0,00097	0,997303	0,13772387
CG32986-RA	CG32985	0,0875873	4,7048	0,184996	4,03367	0,964798	0,980475	0,044335	0,715924	0,13772387
CG32987-RA	CG32986	0,113532	0	0,1239	0	0,6342	0	0,01562	0,897983	0,13772387
CG32988-RA	CG32987	0	0	0,120909	0	2,61352	0	0,044335	0,715924	0,13772387
CG3298-RB	CG32988	0	0,117552	0	14,4763	0	0	-0,229982	0,377567	0,13772387
CG3299-RA	Vinc	38,0045	10,2085	17,1093	4,87666	36,028	2,29138	-0,095573	0,608389	0,13772387
CG33002-RA	mRpL27	31,5326	24,32	57,4278	42,6804	0,664872	0,648399	-0,495599	0,141816	0,6279808
CG33003-RA	CG33003	3,1012	2,26981	31,0334	1,77379	8,82275	0,0344678	0,260398	0,447306	0,6279808
CG33003-RB	CG33003	1,21622	6,40019	1,11222	0	0,0449961	0,0343798	0,260786	0,446587	0,6279808
CG33007-RA	Ack-like	2,98747	3,62341	2,82214	5,9156	2,31896	2,02756	-0,081041	0,786736	0,13772387
CG33012-RA	CG3301	2,73829	2,2362	6,43633	15,3356	3,69895	5,45009	-0,040936	0,906065	0,13772387
CG33012-RB	CG3301	3,32224	2,61634	12,0605	32,309	10,8371	5,77998	-0,044113	0,898915	0,13772387
CG33013-RC	CG33012	0,0256042	2409,41	714,22	0	5,16708	0,0348067	1,244274	0,00047	0,6279808
CG33017-RB	CG33012	0,546677	0,0860214	0,0473173	0,701194	0,84217	29,7749	0,01562	0,897983	0,6279808
CG3301-RA	CR33013	0,803354	0,518323	0,948017	0,0268998	3,09322	0,996029	-0,256086	0,455742	0,6279808
CG3301-RB	CG33017	0	0,0310773	0,0327556	0,0339207	0	1,78768	-0,203181	0,547764	0,6279808
CG3302-RA	Crz	4,43965	15,7817	8,41774	23,2663	9,54276	9,94425	-0,188543	0,572714	0,6279808
CG3303-RA	EndoU	10,3628	10,8971	0,0182045	0,0197545	7,85834	0	-0,053489	0,802004	0,6279808
CG33048-RA	Mocs1	5,35419	1,58534	13,0136	6,36382	13,4868	11,5367	-0,343084	0,155902	0,6279808
CG33048-RC	Mocs1	1,62979	0,0256695	0,0308444	0,0345431	4,49815	0,0352629	-0,346374	0,15094	0,6279808
CG33051-RA	Lamp1	251,85	248,077	209,183	282,805	191,386	179,946	-0,467314	0,07639	0,6279808
CG33052-RA	CG33051	13,2983	10,4096	5,87872	21,2815	19,9726	4,20484	-0,100545	0,650049	0,6279808
CG33054-RA	Gorab	8,49206	0,0705586	15,5431	18,6279	20,1766	22,4703	0,261461	0,401512	0,13772387
CG33054-RB	CG33054	0,113532	13,6807	0,0407168	0,045997	27,3502	0,177236	0,380185	0,215125	0,6279808
CG33056-RA	CG33054	0,104716	0,0404207	0,0417347	0,173618	28,1747	0,15697	-0,607366	0,003055	0,6279808
CG33056-RB	CG33056	13,7504	11,087	0,0394017	0,153765	6,20032	0,0547081	-0,579034	0,005036	0,6279808
CG33056-RC	CG33056	0,044376	0,0395964	0,0709584	0,0535913	12,2641	0,0522842	-0,61009	0,002371	0,13772387
CG33056-RD	CG33056	9,47879	0,0373829	0,141917	0,051217	55,8731	26,9103	-0,612843	0,002305	0,6279808
CG33056-RE	CG33056	0,043471	0,0673227	0,0323877	35,5649	0,0720042	0,051054	-0,602971	0,002704	0,6279808
CG33057-RA	CG33056	0,0410409	0,134645	29,969	0,0500119	29,5539	0,0477895	0,248171	0,304721	0,6279808
CG3305-RA	CG33057	7,77066	10,3903	6,24462	0,0328061	1,56966	0,719085	0,101314	0,630693	0,6279808
CG33060-RA	CG3306	0	0	9,0899	0,38938	7,18107	0	0,066194	0,852398	0,6279808
CG33061-RB	CG33060	0,485852	0,183495	0,777412	777,466	14,928	30,5666	-0,024024	0,944889	0,6279808
CG3306-RA	CG33061	1,24525	0,36879	0,971353	31,5172	40,5576	12,2299	0,044335	0,715924	0,6279808
CG3307-RA	PR-Set7	3,75877	0,0266469	2,94817	0,00606652	8,06583	0,0892672	0,015323	0,952591	0,6279808
CG3307-RB	PR-Set7	2,26941	49,3786	4,11751	0,0840969	26,2149	2,99573	0,012314	0,961755	0,6279808
CG3307-RC	PR-Set7	0,0188483	17,3957	0,0180955	19,6893	6,96895	0,606171	0,018987	0,941006	0,6279808
CG33080-RA	CG3308	23,7825	2,20365	27,7131	5,95351	141,203	21,7235	-0,131668	0,654342	0,13772387
CG33080-RB	CG33080	7,17988	6,84873	17,2369	17,2849	5,38132	5,38324	-0,090202	0,757526	0,6279808
CG33082-RA	CG33080	0,0210791	0,0192003	8,99484	8,60655	4,06049	2,17639	-0,456906	0,153392	0,6279808
CG33083-RA	CG33082	0,0305388	6,40709	0,0293191	6,19116	3,0119	9,55332	0,236119	0,488872	0,13772387
CG33087-RC	Gr97a	0,207786	0,26024	0,423909	0,148858	2,99238	0,029125	-0,143788	0,615859	0,6279808
CG3308-RA	LRP1	2,70754	4,57394	2,25853	10,6003	131,842	1,81104	0,038574	0,837676	0,6279808
CG33090-RB	CG3309	2,51303	3,77	3,24904	9,94041	2,50208	7,1395	0,068196	0,732676	0,6279808
CG33092-RA	CG33090	15,249	10,7622	0,13216	10,5324	357,496	0,11708	0,202008	0,499924	0,6279808
CG33092-RB	P5CDh2	0,0284973	0	0,0275373	0	0,0750939	0	0,202008	0,499924	0,6279808
CG33092-RC	P5CDh2	0,0286829	0	0,0275732	27,0055	0,28331	23,9527	0,202008	0,499924	0,6279808
CG33092-RD	P5CDh2	0,0287203	0,0259573	0,0280859	0,0176629	0,0411384	0,0180309	0,202008	0,499924	0,13772387
CG33092-RE	P5CDh2	0,0292544	0,0261264	0,0281607	0,34173	0,0414248	0,239591	0,202008	0,499924	0,6279808
CG33092-RG	P5CDh2	0,0293323	0,0261604	0,0283495	0	0,0414826	0	0,202008	0,499924	0,6279808
CG33093-RA	P5CDh2	0,221467	0,0266469	9,09443	0,0303715	0,0423085	0,0310044	0,656207	0,028217	0,6279808
CG33094-RB	CG33093	5,63681	33,7846	60,7071	78,3487	3,72131	44,2529	0,102611	0,575813	0,6279808
CG33095-RA	Synd	47,5154	53,8483	52,4913	53,801	151,291	122,166	0,32902	0,215193	0,6279808
CG33095-RB	CG33095	17,1794	19,9495	20,6751	27,2452	3,76222	12,5918	0,311783	0,234375	0,6279808
CG33096-RC	CG33095	18,0607	20,8855	16,0426	19,9141	3,80711	8,56692	-0,009277	0,971132	0,6279808
CG33097-RA	CG33096	8,9231	9,16933	14,1733	17,8799	4,64803	9,63569	-0,354139	0,218324	0,6279808
CG33097-RB	CG42724	5,26911	4,35026	6,36693	13,0321	3,81461	10,8477	-0,374403	0,20015	0,6279808
CG33098-RB	CG42724	0,02309	0,599411	0,0221678	0,0242917	0,0329032	3,02561	0,020596	0,932431	0,6279808
CG33098-RC	CG33098	0,194646	0,0709187	0,0747486	0,21591	0,0192453	0,263441	0,020596	0,932431	0,6279808
CG33098-RD	CG33098	0,066164	0,0602667	0,0635214	0,647074	0,067896	0,0791796	0,034125	0,854068	0,6279808
CG33099-RA	CG33098	0,077645	0,226359	0,238583	3,52471	41,5725	0,10372	-0,737156	0,004807	0,6279808
CG3309-RA	CG33099	22,7052	0,0310453	4,78031	40,437	31,3352	18,9096	-0,162551	0,545446	0,6279808
CG33100-RA	eIF4EHP	4,22748	2,72365	2,68524	5,13624	24,9215	4,01223	0,217562	0,406052	0,6279808
CG33100-RB	eIF4EHP	18,6039	16,1786	6,89305	3,08196	0,174882	12,9565	0,107518	0,617098	0,6279808
CG33101-RA	Nsf2	16,0637	8,31147	0,0318032	26,8831	3,15191	3,63912	-0,12687	0,5567	0,6279808
CG33102-RA	Hex-t1	0,44858	356,804	0,344531	15,2203	0	0,171113	0,459795	0,178973	0,6279808
CG33103-RA	Ppn	0,00901978	0	4,68745	2,31927	6,70939	6,07702	0,250162	0,344133	0,13772387
CG33103-RB	Ppn	69,5597	0,0268849	1,47375	0	0,0124365	0,00720447	0,251951	0,341365	0,6279808
CG33103-RC	Ppn	0,00709375	34,855	3,93971	0	33,8633	0,986916	0,302659	0,248704	0,6279808
CG33103-RD	Ppn	0,00696586	23,7215	0	0	8,03202	4,30743	0,252594	0,339041	0,13772387
CG33104-RA	eca	60,5805	68,9087	88,0154	108,422	117,982	121,012	-0,066114	0,762153	0,13772387
CG33105-RA	p24-2	26,709	30,4196	53,9977	97,8742	118,93	112,123	-1,497167	8,45E-07	0,13772387
CG33106-RA	mask	0,00516433	11,947	14,1569	10,5403	0,00705789	0,0153905	0,137608	0,593259	0,6279808
CG33106-RB	mask	11,8652	0,174848	0,0614301	6,19523	6,46798	0,0152337	-0,052507	0,846305	0,6279808
CG33107-RA	CG33107	0,0569129	0,0518402	0,0546398	17,3826	4,7745	0,0315868	-0,375501	0,248874	0,6279808
CG33108-RA	CG33108	3,92281	3,70429	53,1236	6,32959	137,72	4,04648	0,192833	0,454608	0,6279808
CG33109-RA	CG33109	0	0	0,212456	0,173389	0,271217	0,139493	0,011195	0,934319	0,6279808
CG33110-RA	CG33110	41,7001	16,8881	43,7745	27,4132	45,9033	32,8629	-0,171227	0,591828	0,6279808
CG33111-RA	CG33111	7,02314	13,6935	7,16236	0,0915772	8,76504	9,96863	0,167942	0,592625	0,6279808
CG33111-RB	CG33111	12,6609	21,3129	11,5481	17,9987	0,0404486	0,0304845	0,181729	0,561689	0,6279808
CG33111-RC	CG33111	0,0280497	0,0255496	0,0269294	0,0300392	3,67879	3,56624	0,173194	0,580186	0,6279808
CG33113-RA	Rtnl1	218,222	255,004	108,886	101,533	88,9923	99,1579	0,203346	0,342316	0,6279808
CG33113-RB	Rtnl1	161,635	175,992	133,457	170,626	80,6419	132,571	0,210651	0,324173	0,13772387
CG33113-RC	Rtnl1	0,0477887	0,0435292	0,04588	0,0534176	0,0723544	0,0545307	0,215919	0,315467	0,6279808
CG33113-RD	Rtnl1	0,0485257	0,0442005	50,7105	63,8278	32,554	51,5054	0,207894	0,33192	0,6279808
CG33113-RE	Rtnl1	0,0459924	0,0418931	47,6879	0,0511772	35,9157	0,0522436	0,210389	0,324681	0,6279808
CG33113-RF	Rtnl1	0,0445355	0,040566	0,0427567	0,0493748	0,0668785	0,0504036	0,164023	0,438378	0,6279808
CG33113-RG	Rtnl1	0,024825	0,0226123	0,0238335	37,4448	14,437	21,0675	0,207649	0,332349	0,6279808
CG33114-RA	Gyc32E	0,115293	0,20659	5,84114	0	0,0264562	0	-0,620836	0,081344	0,6279808
CG33114-RB	Gyc32E	0,0187573	0,12252	8,49762	0,340932	0	0,24853	-0,620836	0,081344	0,6279808
CG33115-RA	NimB4	35,4985	3,20995	15,8592	38,0317	41,7148	21,6122	-0,03148	0,89688	0,6279808
CG33116-RA	CG33116	12,4417	7,24959	14,7677	10,4798	5,4331	2,85313	0,328173	0,1341	0,6279808
CG33117-RA	Victoria	0,592851	0,441826	3,77765	1,78927	0,0449961	2,08483	-1,336073	0,000181	0,6279808
CG33119-RA	NimB1	15,7209	16,2861	16,7405	4,95677	6,71398	8,70215	-0,304091	0,236839	0,6279808

gene_id	Symbol	W1_FPKM	W2_FPKM	W3_FPKM	MCM51_FPKM	MCM52_FPKM	MCM53_FPKM	FC	p-value	p-adj
CG33120-RA	Rnp4F	0,0200859	0,0182956	0,0192836	1,77601	2,45353	0,0214183	-0,056237	0,837184	0,6279808
CG33121-RA	Rnp4F	3,43192	3,55886	5,66713	6,42093	0,00788676	4,55152	0,145626	0,375579	0,6279808
CG33122-RA	Rnp4F	1,3319	0,0186644	0,0196723	0,0214246	0,00842582	0,021871	-0,35301	0,253385	0,6279808
CG33123-RA	Rnp4F	0,0204717	0,018647	0,0196541	0,0214037	0,00814441	0,0218497	-0,144696	0,586733	0,6279808
CG33124-RC	CG33120	1,43454	0,0316771	15,7444	0,0386797	30,5469	0,0398365	0,127997	0,689772	0,6279808
CG33125-RA	Spn28Db	0,158684	133,927	0,149851	0,246956	0,125408	0	-0,013765	0,912179	0,13772387
CG33126-RA	cutlet	0,980912	3,20227	12,4754	2,54113	0,0203931	0,0153694	-1,253582	9,6E-07	0,6279808
CG33127-RA	LeuRS	11,338	12,8676	12,1736	0,118351	8,24573	9,06187	-0,854901	0,015111	0,6279808
CG33128-RA	CG33124	3,8593	3,68605	97,5684	2,90636	2,04748	4,7785	-0,763007	0,031425	0,6279808
CG33129-RA	CG33125	0	0	0,0247028	0	0	0,133194	-0,364682	0,155973	0,6279808
CG33129-RB	NLaz	6,10877	9,64696	0	0,202623	0,192966	0,144931	-0,364841	0,15579	0,6279808
CG33129-RC	CG33127	18,8567	0,0828254	54,1995	17,1826	0,0161437	85,9069	-0,364727	0,156085	0,6279808
CG33129-RE	CG33128	7,11921	0,219659	0,231521	0,100904	0	0,355702	-0,369249	0,146262	0,6279808
CG3312-RA	CG33129	0,0449291	90,0131	0,0424989	0,557691	1,06652	0,0268573	-0,175932	0,492038	0,6279808
CG3312-RB	CG33129	157,168	0,0403214	0,022371	2,31693	0,033826	1,69298	-0,175932	0,492038	0,6279808
CG3312-RC	CG33129	0,0442669	0,0212248	13,8849	17,9999	49,7944	0,0508995	-0,175932	0,492038	0,6279808
CG3312-RD	CG33129	0,0233017	0,392094	0,413269	132,142	96,3253	159,202	-0,175121	0,493257	0,6279808
CG33130-RA	DCAF12	6,17559	0,167185	5,65397	0,113921	5,67942	2,31741	-0,134213	0,609554	0,6279808
CG33130-RB	Patronin	1,65004	0,0101552	5,33254	8,12901	0,0154479	3,21107	-0,137652	0,599369	0,6279808
CG33130-RC	Patronin	4,60661	5,94771	1,57239	3,39169	2,66316	2,82442	-0,1393	0,599225	0,6279808
CG33130-RD	Patronin	0,0107046	0,00975048	0,0101245	0,0107727	2,40855	0,0109972	-0,139639	0,598385	0,6279808
CG33130-RE	Patronin	0,0105457	0,00960576	3,6579	8,83398	0,0145917	3,19334	-0,138292	0,60051	0,6279808
CG33131-RA	Patronin	4,55755	7,64545	0,426531	1,15939	3,01575	0,468755	0,003229	0,987661	0,6279808
CG33133-RA	SCAP	3,72732	3,54457	4,39616	0	2,79664	3,07305	-0,280883	0,180954	0,6279808
CG33134-RA	grau	8,1983	7,91341	10,556	11,4592	9,55475	23,8679	0,076793	0,729999	0,6279808
CG33135-RA	Debcl	9,57003	12,7332	10,6118	7,65574	7,9085	9,9516	0,027543	0,915841	0,6279808
CG33135-RB	KCNQ	3,0583	1,29621	2,61257	1,24523	0,0235271	1,93179	0,031609	0,903885	0,6279808
CG33135-RC	KCNQ	1,18668	1,34409	1,23835	0,938508	0,627946	1,27242	-0,184726	0,517827	0,6279808
CG33135-RD	KCNQ	2,17484	2,37307	3,11021	2,28459	0,0220453	2,93648	0,022222	0,932552	0,6279808
CG33136-RA	KCNQ	0,0315297	0,0287195	0,0302704	0,033851	3,07024	0,0345564	0,452076	0,165472	0,6279808
CG33137-RA	CG33136	0,440506	0,0195622	54,4615	7,00964	0,456374	0,0305815	-0,142792	0,680258	0,6279808
CG33138-RA	CG33137	0,5824	1,35034	1,11828	4,34812	22,677	1,61261	-0,679902	0,001843	0,6279808
CG33139-RA	AGBE	13,4794	16,1075	15,341	0,0474546	1,58801	21,8512	-0,13373	0,498181	0,6279808
CG3313-RA	Impbeta11	5,20374	0	0	0	0,0267817	0,0201843	-0,062886	0,781629	0,6279808
CG33140-RA	RpL7A	1478,89	1784,83	2601,4	2638,98	36,5611	3150,09	0,045919	0,836527	0,6279808
CG33141-RA	RpL7A	0,0635548	0,0578901	0,0610164	0,0739923	2968,28	0,0755341	-0,056825	0,855695	0,6279808
CG33141-RB	RpL7A	0,0654431	0,0596101	0,0628293	0,0765719	0,100223	0,0781674	-0,05701	0,85526	0,6279808
CG33143-RB	CG33140	0,0727388	0,0795066	0	10,1348	4,38521	6,63582	0,841355	0,004002	0,6279808
CG33143-RC	sns	5,87412	4,78897	4,85546	51,1917	27,3045	33,1473	0,789046	0,006902	0,6279808
CG33144-RA	sns	0,00809886	0,0123951	0,00777539	0,193503	0,262101	0,197535	-0,305678	0,136668	0,6279808
CG33145-RA	CG33143	5,00659	4,08007	5,8192	4,09521	0,153742	3,02312	0,131087	0,576821	0,6279808
CG33145-RB	CG33143	4,40167	33,2286	52,7776	30,4891	0,0465691	41,2858	0,131087	0,576821	0,6279808
CG33147-RA	CG33144	9,41746	12,1773	10,8257	16,689	0,0195189	10,1401	0,135807	0,610731	0,6279808
CG3314-RA	GalT1	5,56819	4,23102	5,09257	4,75657	4,15841	3,80802	-0,495586	0,054754	0,6279808
CG3314-RC	GalT1	0,0302038	0,0275117	0,0289974	0,0323233	0,0437821	0,0329968	-0,49557	0,054764	0,6279808
CG3314-RD	Hs3st-A	5,19932	1,36649	0,680108	0,101685	3,81358	0,023295	-0,495586	0,054754	0,6279808
CG33150-RA	TrxT	0,327994	0,0948317	3,12955	0,461623	0,394347	0	0,832761	0,019529	0,6279808
CG33151-RA	Gr59f	2,21876	6,55594	1,74049	1,93294	4,11589	0,87475	0,547513	0,125968	0,6279808
CG33152-RA	Gr59e	1,07373	2,63316	1,16301	3,10173	2,76552	0,6926	0,632463	0,04475	0,6279808
CG33155-RA	hbn	1,65006	2,18769	1,42574	1,55978	0,773648	0,811266	-0,377542	0,158465	0,6279808
CG33155-RC	CG33155	48,8762	47,0771	75,1847	66,3417	0,0196999	91,7697	-0,397717	0,139574	0,6279808
CG33155-RD	CG33155	45,7485	44,0279	54,468	80,9142	105,542	63,9716	-0,378094	0,156039	0,13772387
CG33156-RA	CG33155	0,0764176	0,0696064	0,0733654	0,0920891	73,9382	0,0940081	0,604769	0,028863	0,6279808
CG33156-RB	CG33156	0,0195916	23,0097	12,5824	0,0402141	11,0668	10,7701	0,608196	0,030776	0,6279808
CG33156-RC	CG33156	0,036962	0,0329068	11,3614	0,0365148	11,7838	11,7318	0,604937	0,028418	0,6279808
CG33156-RE	CG33156	0,0338214	43,1285	0,0347409	0,0399702	0,0549735	0,0414314	0,542614	0,054675	0,6279808
CG33156-RF	CG33156	0,0367564	0,026917	0,0188091	0,0435354	0,0276873	0,0208668	0,620251	0,027404	0,6279808
CG33158-RB	CG33156	14,4855	0,0277102	0,0354858	16,5819	0,0544703	0,0410521	-0,182287	0,490311	0,6279808
CG33159-RA	CG33158	3,20312	2,01918	23,9182	19,3729	3,69774	10,3238	-0,007158	0,964229	0,6279808
CG3315-RA	CG33159	0,128054	0	0,12294	0,278567	0	0	-0,15696	0,660207	0,6279808
CG33160-RA	CG33160	48,9362	17,2899	54,7479	28,9568	193,618	202,959	-0,633331	0,063259	0,6279808
CG33162-RA	SrpRbeta	40,436	0,0265411	6,65792	47,0756	27,4808	0	0,391289	0,067503	0,6279808
CG33166-RA	stet	1,73821	1,53431	1,51395	1,19043	24,375	0	0,213929	0,462779	0,6279808
CG33166-RB	stet	1,12662	1,90951	1,24592	2,88408	19,0418	7,42255	0,135193	0,639797	0,6279808
CG33169-RA	CG33169	166,024	0,0107386	120,795	204,692	0,207309	184,367	0,189624	0,436713	0,6279808
CG33169-RC	CG33169	0,113989	151,666	0,109436	0,153051	1520,2	0,15624	0,187325	0,441667	0,6279808
CG33170-RA	CG33170	22,6828	16,9181	28,3796	18,1634	20,005	24,7954	0,02499	0,927814	0,6279808
CG33170-RB	CG33170	37,443	32,8034	73,9072	51,5727	97,5459	106,24	-0,226967	0,428638	0,6279808
CG33171-RC	Mp	8,60838	8,93281	0,102043	0,139447	8,38004	0,142353	-0,129655	0,564713	0,6279808
CG33171-RE	Mp	0,0160313	1,73039	0,124223	0,182263	0,022467	0,731611	-0,129801	0,564272	0,6279808
CG33172-RA	CG33172	1,28082	0,436134	0,184409	0,362786	1,02118	0,859776	0,075674	0,820724	0,6279808
CG33173-RA	CG43672	0,150063	0,189504	40,3591	29,4859	31,2429	35,2863	-2,658424	1,37E-20	0,6279808
CG33174-RA	inaE	3,70188	0,0103384	3,15637	0,312463	2,76081	3,10287	-0,341894	0,150925	0,6279808
CG33174-RD	inaE	0,01135	5,53372	0,0108967	0,151984	0,728267	0,011858	-0,182381	0,439349	0,6279808
CG33174-RE	inaE	4,30355	0,0107291	4,60766	98,8255	3,18338	3,73549	-0,183966	0,434189	0,13772387
CG33174-RF	inaE	0,0117789	0,380392	0,0113085	11,7199	0,0163447	0,0123184	-0,180919	0,442876	0,6279808
CG33177-RA	CG33177	1,24548	1,91068	1,888	3,70017	2,80528	1,52332	-0,022354	0,948963	0,6279808
CG33178-RA	CG33178	40,1858	1,62324	1,31457	52,5441	81,451	92,8647	-0,564685	0,080646	0,6279808
CG33179-RB	beat-IIIb	0,0208529	4,47087	0,0199445	3,2479	0,056378	0	0,366845	0,114833	0,13772387
CG33179-RC	beat-IIIb	4,06457	0,320141	0,129517	0,273394	0,0562787	3,26904	0,366845	0,114833	0,6279808
CG33180-RB	AANAT1	23,6513	19,4297	40,6289	6,58971	0,0915842	36,3687	0,070329	0,755174	0,6279808
CG33182-RA	AANAT1	0,044919	4,35749	7,13718	16,8777	22,5459	0,0530261	0,013066	0,966271	0,6279808
CG33183-RA	Ranbp16	5,74696	5,79344	6,26033	8,14539	4,11617	0,0268252	0,213986	0,539713	0,6279808
CG33183-RB	Kdm4B	0,0353978	0,04797	0,061252	1,36741	0,100675	4,60565	0,232712	0,504204	0,6279808
CG33183-RC	Hr3	14,6481	23,9678	10,6415	34,363	0,0292384	9,56812	0,225297	0,517644	0,6279808
CG33183-RD	Hr3	10,1728	12,7695	5,17287	10,1214	2,52881	3,17537	0,232993	0,503651	0,6279808
CG33189-RA	Hr3	0,0126712	0,0115418	0,0121651	0,0130079	0,0458514	0,0132789	0,211658	0,553915	0,6279808
CG3318-RA	Hr3	0,0157411	0,0143381	0,0151124	0,0162755	39,0451	0,0166146	-0,085387	0,762431	0,6279808
CG3318-RB	CG33189	1,02481	1,60023	0,796474	1,49158	0,607903	3,62555	-0,068033	0,807744	0,6279808
CG33191-RA	Cdk7	2,7769	0,130049	4,01037	3,20163	73,081	0,456524	0,198974	0,577592	0,6279808
CG33192-RA	CG33191	0,0427123	0,0389053	0,0410063	0,0490076	0,066381	0,567149	-1,229527	0,000546	0,6279808
CG33193-RA	MtnD	9,68089	0,0294935	12,2319	0,0348353	0,0471846	0,0355612	0,077649	0,731424	0,6279808
CG33194-RA	sav	5,23626	36,3047	30,251	35,0646	0,0316883	0,0238822	0,292042	0,270809	0,6279808
CG33196-RC	CheA29a	0,144481	0,482545	4,76735	2,33507	2,04632	0,339639	-0,162338	0,605495	0,6279808
CG33197-RA	dpy	1,01009	0,0297266	0	0,118543	3,62914	0,950814	-1,036747	2,35E-06	0,6279808
CG33197-RB	mbl	0,0165784	0,0151007	0,0167438	0,0171749	0,130384	0,0175327	-1,048218	1,25E-06	0,6279808
CG33197-RC	mbl	0,0230578	0,0210026	15,2353	0,0242559	78,9433	18,1356	-1,101859	1,35E-06	0,6279808
CG33197-RD	mbl	0,0174404	0,0158859	15,4244	0,0181045	11,2334	0,0184817	-0,838675	1,95E-05	0,6279808
CG33197-RE	mbl	11,2356	15,5719	0,0162464	19,9634	103,212	11,7792	-0,801535	3,02E-05	0,6279808
CG33197-RF	mbl	14,575	15,7913	81,7728	21,6579	1,92564	15,1786	-0,845311	1,9E-05	0,13772387
CG33198-RA	mbl	4,36595	0,015414	1687,78	13,7949	6,47294	0,0179109	0,023974	0,929224	0,6279808
CG33199-RA	pen-2	31,6074	3,44247	7,19695	8,29602	0,855767	30,9105	-0,406265	0,05254	0,6279808
CG3319-RA	CG33199	32,2299	43,4877	39,7551	112,565	68,7266	55,1944	-0,089874	0,754302	0,6279808
CG33200-RA	Rab1	0,0350969	0	0,0130457	0,0139793	17,6706	0,0388024	0,499033	0,148076	0,6279808

gene_id	Symbol	W1_FPKM	W2_FPKM	W3_FPKM	MCM51_FPKM	MCM52_FPKM	MCM53_FPKM	FC	p-value	p-adj
CG33202-RB	Rab1	147,901	19,0403	2,01475	1,8507	24,7026	135,219	-0,146983	0,557708	0,6279808
CG33203-RB	VepD	3,19503	0,158385	2,69413	4,62141	1,49089	0,378437	0,16953	0,523457	0,6279808
CG33203-RC	dpr11	3,03075	4,26485	3,42317	0,0534609	0,0724131	27,5147	0,16788	0,528068	0,13772387
CG33205-RA	CG33203	3,8959	10,3057	0,0301124	0,0237831	0,0322144	0,0237893	0,362477	0,177521	0,6279808
CG33205-RB	CG33203	0,0196732	2,03197	0,0213113	0,0347801	0,0471099	0,0242787	0,348571	0,190355	0,6279808
CG33205-RC	CG43897	0,0220498	0,464402	0,0253173	3,09737	0,025187	12,8059	0,341284	0,199701	0,6279808
CG33205-RE	CG43897	0,0192211	0,259307	5,29785	0,289604	4,57446	0,0210365	0,377405	0,165452	0,6279808
CG33205-RF	CG43897	0,0240567	0,0796467	1,2045	8,69307	9,95763	7,19761	0,362448	0,178196	0,6279808
CG33205-RG	CG43897	112,251	11,8161	0,0839479	0,026845	0,0279125	0,24869	0,354854	0,187377	0,6279808
CG33205-RH	CG43897	39,0272	0,666622	18,2121	0,0279614	6,43486	16,7427	0,36254	0,178063	0,6279808
CG33205-RI	CG43897	134,101	0,139969	0,147527	6,02079	0	42,9962	0,420406	0,127909	0,6279808
CG33206-RA	CG43897	42,0159	7,28463	8,17678	0,108639	135,321	0,0236222	-0,002129	0,992021	0,6279808
CG33206-RB	CG43897	53,9766	0,023116	0,0243644	15,7738	29,0698	0,0204544	0,002092	0,992177	0,6279808
CG33207-RA	Gmap	7,83785	4,70782	7,47251	36,2147	5,64406	5,42081	0,189376	0,571012	0,6279808
CG33207-RB	Gmap	9,00132	13,8912	10,7529	38,5757	3,02241	2,79982	0,293239	0,375501	0,6279808
CG33208-RA	pxb	0,0294368	20,8577	0	0	1,42735	0	-0,413242	0,15312	0,6279808
CG33208-RB	pxb	12,9045	0,0268131	1,45074	2,42296	0	1,38888	0,13671	0,624916	0,6279808
CG33208-RC	Mical	0,00460919	28,0513	0,00443966	16,9156	0,0211018	4,693	0,116614	0,68013	0,6279808
CG33208-RD	Mical	0,853971	3,86079	0,00441125	8,7394	0,0432307	1,7814	0,116614	0,68013	0,6279808
CG33208-RE	Mical	0,00462436	0,0137172	7,39288	0	7,86851	0,0047109	0,141194	0,613492	0,6279808
CG33208-RF	Mical	0,00459477	0,0137454	1,44475	8,41606	4,11901	0,0258361	0,115865	0,682138	0,6279808
CG33208-RG	Mical	9,29753	0,0271886	0,00439688	0,0217499	7,16266	0,0278815	0,116853	0,679534	0,6279808
CG33208-RH	Mical	1,22671	0,00419837	0,0230456	0,0225462	5,82986	0,180006	0,116614	0,68013	0,6279808
CG33208-RI	Mical	0,0045798	0,87904	0,0247639	0,0216926	0	2,23415	-0,395341	0,170721	0,6279808
CG3320-RA	Mical	0,0240043	0,00421218	0	0,0222418	0,0425506	6,80191	-0,016888	0,934684	0,13772387
CG3320-RB	Mical	0,0257941	0,00418524	4,94479	0,0226781	32,3432	3,04008	-0,016782	0,935091	0,6279808
CG33213-RA	Dpse\GA17372	270,233	10,494	9,13773	362,74	3,47435	5,34161	-0,393536	0,147323	0,6279808
CG33214-RA	Dpse\GA17372	0,109096	1,30359	0,0241052	0,144334	7,09104	0,0270953	-0,034796	0,87977	0,6279808
CG33217-RA	CG33213	0,0543535	0,049509	0,0521826	11,3112	10,8202	14,0468	-0,570253	0,05277	0,6279808
CG33218-RA	Glg1	7,32924	15,6138	8,57304	5,37123	15,0858	3,612	0,233801	0,481993	0,6279808
CG3321-RA	CG33217	23,3926	13,9141	11,6275	16,7915	18,2589	18,6316	-0,333699	0,268475	0,6279808
CG3321-RB	lncRNA:CR33218	0,594791	0	4,35111	0,679798	17,2916	2,93427	-0,333709	0,268484	0,6279808
CG33221-RA	LanB2	80,1243	0,337415	66,3789	0,626061	30,1324	39,9272	NA	NA	0,6279808
CG33222-RA	CR33221	0	0,0645316	0,0680166	16,0327	23,5616	0,135831	0,218774	0,418956	0,6279808
CG33223-RA	CR33222	1,45756	0,129526	1,86579	220,018	1,73713	1,54975	0,034862	0,854192	0,6279808
CG33224-RA	CG33223	0,157925	0	0,0530555	0,0629626	3,06131	0,0996785	-0,136755	0,669147	0,6279808
CG33225-RB	Nost	0,0391213	0,0268729	2,06623	0,0443261	0,06004	0,0452498	-2,097779	1,35E-09	0,6279808
CG33226-RA	CG33225	0,161161	0,322952	0,247558	0	2,21325	0	0,348005	0,250442	0,6279808
CG33228-RA	CG33226	0,20023	0,246666	0,461358	108,862	0,0466665	0,378437	-0,089311	0,761202	0,6279808
CG33229-RA	CG33228	0,0957622	6,50131	5,06045	7,68003	3,66902	157,44	-0,408097	0,148943	0,6279808
CG3322-RA	CG33229	128,369	99,7517	0,165343	96,1615	141,952	121,911	0,263746	0,391329	0,6279808
CG33230-RA	CG3323	0,720376	25,3727	0,164486	0,0206653	0	0,170268	0,073548	0,797718	0,6279808
CG33231-RC	CG33230	6,21466	5,02384	0,638625	5,14054	1,45043	0,0262597	-0,244541	0,383457	0,6279808
CG33232-RA	Svil	4,48049	6,01865	3,82359	7,61036	6,40613	5,26428	0,358584	0,166863	0,6279808
CG33232-RB	Svil	0,0142262	0,0129582	0,013658	0,0385214	0,0198532	0,0149626	0,358488	0,166945	0,6279808
CG33232-RC	Svil	0,0151492	0,013799	0,0145442	67,7065	0,021187	0,0159678	0,358584	0,166863	0,6279808
CG33232-RD	Svil	22,7614	27,6735	16,7904	0,0328879	10,7315	13,6153	0,354813	0,17089	0,6279808
CG33233-RC	Svil	0,0151319	0,0137832	0,0145275	0,468659	0,0211619	0,0159489	-0,749497	0,029525	0,6279808
CG33233-RD	CG33233	0,0705435	0,0642559	0,067726	4,87338	4,81793	1,80221	-0,548906	0,124556	0,6279808
CG33234-RB	CG33233	0,296367	0,0385646	0,28453	14,8046	8,37004	22,9444	-0,328158	0,3583	0,6279808
CG33234-RD	CG33234	0,043168	0,0409246	0,0450012	0,0131612	0,0178269	1,79269	-0,328158	0,3583	0,6279808
CG33235-RA	CG33234	0,202181	1,51633	1,3216	0,0129364	0,0175224	0,0854133	0,046487	0,858374	0,6279808
CG33236-RB	CG33235	0,0347402	0,0379725	0,0297825	0,0410151	38,7466	0,014561	NA	NA	0,6279808
CG33237-RB	Ste:CG33236	0,108856	0,148731	0	0,178725	0	0	NA	NA	0,6279808
CG33238-RB	Ste:CG33237	0,108856	0,099154	0,104509	0,169476	0	0	NA	NA	0,6279808
CG33239-RB	Ste:CG33238	0,108677	0,148486	0	0,178725	0	0	NA	NA	0,6279808
CG3323-RA	Ste:CG33239	0,109036	0,148976	0	0,18006	0	0	0,441346	0,173413	0,6279808
CG33240-RB	Pkg21D	5,13875	5,47871	8,19569	7,25021	4,19564	3,10839	NA	NA	0,6279808
CG33241-RB	Ste:CG33240	0,108856	0,148731	0	0,169895	0	0	NA	NA	0,6279808
CG33242-RB	Ste:CG33241	0,108856	0,148731	0	0,178725	0	0	NA	NA	0,6279808
CG33243-RB	Ste:CG33242	0,164096	0,148731	0	0,178725	0	0	NA	NA	0,6279808
CG33244-RB	Ste:CG33243	0,108856	0,148731	0	0,169895	0	0	NA	NA	0,6279808
CG33245-RB	Ste:CG33244	0,108856	0,14947	0	0,179168	0	0	NA	NA	0,6279808
CG33246-RB	Ste:CG33245	0,163285	0,099154	0,104509	0,178725	0	0	NA	NA	0,6279808
CG33247-RB	Ste:CG33246	0,163285	0,148731	0,104509	0,171938	0	0	NA	NA	0,6279808
CG3324-RA	Ste:CG33247	0,108856	0,099154	0	0,178725	0	0	-0,420217	0,083633	0,6279808
CG33250-RA	spn-B	4,45482	0,0629566	0	0,0199459	0	0,873133	0,017224	0,951721	0,6279808
CG33251-RA	AlkB	6,30942	12,5351	27,0153	10,5297	0,016511	36,1513	-0,429398	0,103828	0,6279808
CG33252-RA	CG33251	0,0712279	0,0648793	0,068383	0,084637	0,0695901	0,0524473	0,203291	0,452998	0,6279808
CG33253-RA	CG33252	0,475746	0,0202398	0,152248	37,5194	29,9023	48,5246	0,00407	0,987124	0,6279808
CG33255-RA	CG33253	13,3221	21,2443	14,5674	9,48407	0,0448604	13,9951	-0,289425	0,363081	0,6279808
CG33256-RA	CR33255	4,55992	6,46535	7,8057	0,076633	0,841938	1,12742	-0,148921	0,658785	0,6279808
CG33257-RA	Lmpt	0,756883	2,11652	2,04635	2,07527	2,6732	2,19625	0,086186	0,807389	0,6279808
CG33258-RA	CG33257	0,7985	0,969772	0,990202	1,75605	49,5877	52,544	0,621589	0,079379	0,6279808
CG33259-RA	CG33258	0,894016	0,444181	1,01436	0,588147	0,353217	0,266206	NA	NA	0,6279808
CG3325-RA	CG33259	0	0	2,21155	0	15,8682	0,014402	0,414659	0,14043	0,6279808
CG33260-RB	Fign	0,533485	6,78098	0,310965	3,83694	0,0224501	4,23071	-0,41563	0,233065	0,6279808
CG33261-RA	CG33260	0,0913068	0,0831686	0,08766	0,0334218	0,04527	0,11708	0,015772	0,946344	0,6279808
CG33261-RB	Trl	8,33021	12,3818	8,91231	0,262502	0	5,9941	0,013617	0,95558	0,6279808
CG33261-RC	Trl	5,10815	0,0158261	0,0166807	7,3577	3,10381	0,0186819	-0,181779	0,447228	0,6279808
CG33261-RD	Trl	16,8072	21,3923	17,6934	7,3592	3,04089	12,4272	0,050136	0,829546	0,6279808
CG33261-RE	Trl	10,742	9,29442	7,40127	12,3902	7,40354	9,83352	-0,183683	0,440112	0,6279808
CG33261-RF	Trl	0,0258142	0,0235134	0,0247832	13,9572	8,46559	0,0279046	-0,185824	0,432112	0,6279808
CG33261-RG	Trl	0,0353915	0,032237	0,0339779	0,027335	0,0370255	0,0404109	-0,172364	0,471063	0,6279808
CG33261-RH	Trl	0,0331042	0,0301536	0,031782	0,039586	6,97291	0,0375043	-0,1806	0,451008	0,6279808
CG33261-RI	Trl	0,0342362	0,0311847	0,0328688	24,6188	0,0497628	0,0389371	-0,032274	0,896165	0,6279808
CG33262-RA	Trl	0,0303937	0,0276847	0,0291798	0,0381423	0,051664	0,0341182	0,01562	0,897983	0,6279808
CG33263-RA	CG33262	0	0	0	0	11,3129	0	0,054636	0,748216	0,6279808
CG33264-RA	CG33263	0,241505	0	1,59651	0,177583	0,278028	0	-0,133566	0,361568	0,6279808
CG33264-RB	Or69a	0	0	0,0440942	0	0,0684606	0	-0,164316	0,304789	0,6279808
CG33265-RA	Or69a	0	0,56887	1,94867	0,115529	0,337178	0,483205	-0,070175	0,843545	0,6279808
CG33267-RA	Muc68E	0,16519	0,307466	0,411164	0,349968	0,282166	0,249665	-0,134642	0,697765	0,6279808
CG33268-RA	CG33267	4,35917	3,86615	2,6432	8,24226	4,90451	5,52942	-2,093484	4,27E-09	0,6279808
CG33269-RA	CG33268	0	0	12,1211	1,78288	4,05738	6,03632	-0,927241	0,008967	0,6279808
CG3326-RA	CG33269	263,066	11,2884	296,038	23,8493	0,364314	3085,52	0,136631	0,69023	0,6279808
CG33270-RA	E23	4,22054	0,753543	0,013934	8,43044	0,020268	0,0152752	-0,879301	0,012636	0,6279808
CG33271-RA	E23	1,50378	0	0,886751	3,65686	0	1,14056	-0,960498	0,006516	0,6279808
CG33272-RA	E23	0,0145137	1,84442	11,0715	1,81554	1,26465	8,57394	-0,881504	0,011734	0,6279808
CG33273-RA	CG33270	346,589	88,7018	383,709	1241,47	0,0988365	0,160502	0,336866	0,259404	0,6279808
CG33275-RA	CG33271	69,788	17,019	83,8696	11,5016	48,1041	31,9641	0,57996	0,048511	0,6279808
CG33275-RB	CG33272	375,791	129,39	5,46373	866,335	4,73154	6,78052	0,579283	0,049374	0,6279808
CG33275-RC	Ilp5	0,509845	11,6773	20,1276	20,8584	0,0443037	55,8843	0,693825	0,018611	0,6279808
CG33276-RB	Pura	0,0103605	0,00943707	0,0150122	1,50764	0,0218938	0,0165005	1,327359	0,000208	0,13772387
CG33278-RA	Pura	4,55124	6,21564	0,0157503	6,73273	0,0230115	0,0173429	0,084543	0,804028	0,6279808

gene_id	Symbol	W1_FPKM	W2_FPKM	W3_FPKM	MCM51_FPKM	MCM52_FPKM	MCM53_FPKM	FC	p-value	p-adj
CG3327-RA	Pura	18,5838	27,2373	0,0162049	13,6066	0,0237021	0,0178633	-0,024751	0,942742	0,13772387
CG3327-RB	Urm1	1,81762	1,46084	3,07946	0,902809	1,96977	3,77604	-0,006019	0,986125	0,6279808
CG3327-RC	CG33278	0,199625	0,399721	0,110363	0,235065	4,69906	0,193847	-0,025718	0,940492	0,6279808
CG33281-RA	CG3328	9,87818	12,4695	56,8064	5,89358	0,0703581	8,45789	-0,293298	0,336225	0,6279808
CG33282-RB	CG33281	3,46231	0,0136364	5,65623	0,0149634	2,32491	1,53754	-1,114819	0,001713	0,6279808
CG33283-RA	CG33282	0,214997	0	7,49564	1,81497	0,156302	4,99167	0,01562	0,897983	0,6279808
CG33284-RA	CR33283	0	0	0,128937	0,683829	0	14,5251	-0,068102	0,725122	0,6279808
CG33285-RA	CG33284	0	3,69898	0	0,675298	0,028519	0	-0,013096	0,914398	0,6279808
CG33286-RB	CG33285	0	23,7986	0,128588	0	0,0281679	25,9596	0,014729	0,952296	0,6279808
CG33287-RA	CG33286	0,0300892	22,5853	17,668	0	1,5866	0	-0,070526	0,562659	0,6279808
CG33287-RB	CG33287	0	0	0,19515	0	0	0,0214252	-0,070526	0,562659	0,6279808
CG33288-RB	CG33287	0	0,0274073	0,0481457	0	0,559364	0	-0,415675	0,135427	0,6279808
CG33289-RA	CG33288	0,0189619	0,0172718	0,0273068	0,0197545	0,0267576	0,0201662	-0,236497	0,50823	0,6279808
CG3328-RA	ppk5	0,385058	0,350737	0,0743979	9,49303	0,126863	0,337991	-0,053694	0,799558	0,6279808
CG33290-RA	Prosbeta2	34,8401	32,3812	16,0056	9,24806	35,8057	1,67426	-0,385477	0,272218	0,6279808
CG33291-RA	CG33290	1,08846	0,583202	0,614698	3,67887	13,4493	0,858393	-0,052324	0,861497	0,6279808
CG33292-RA	CG43980	7,3064	5,064	6,67086	15,1042	5,03923	0,0352629	-0,007156	0,984023	0,6279808
CG33293-RA	CG43980	0,954429	2,43322	0,634368	1,59949	0,51194	3,72661	-0,497178	0,156675	0,6279808
CG33296-RA	CG33293	0,395073	0,404842	0,237058	1,69489	0,0548721	0,785728	-0,0864	0,750365	0,13772387
CG33298-RA	CG33296	0,0631701	0,115079	18,9882	0	11,9903	13,3255	0,337247	0,178156	0,13772387
CG33298-RB	CG33298	12,6228	3,7317	0,343273	4,07567	0	0	0,33664	0,179046	0,13772387
CG33299-RB	CG33298	2,75383	0,281904	0	0	0	0	0,244193	0,445756	0,13772387
CG3329-RA	CG33299	0,340597	0,517066	1,03548	0,0862998	0	0,428235	0,086559	0,74754	0,6279808
CG33300-RA	CG3330	0	0	0	8,99898	0	0	-0,301909	0,39405	0,13772387
CG33301-RA	Muc30E	2,71757	2,79361	2,75812	7,51424	0,8219	2,80686	0,289496	0,416915	0,13772387
CG33302-RA	CG33301	5,10896	0	14,3177	3,14486	5,48042	5,07881	-0,092784	0,724469	0,13772387
CG33303-RA	Cpr31A	95,5058	60,575	7,13756	0,985931	4,10188	5,2031	0,144923	0,522642	0,13772387
CG33304-RA	CG33303	24,905	26,7239	9,54371	9,2392	7,74432	6,02004	-0,108153	0,625089	0,13772387
CG33306-RA	rho-5	6,15322	6,91654	5,73001	8,4417	5,98445	31,9431	-0,329619	0,339896	0,13772387
CG33307-RA	CG33306	0,900663	0,223742	6,60309	6,85506	11,0023	9,62442	-0,785079	0,028267	0,13772387
CG33308-RA	CG33307	4,33659	2,02701	19,1187	5,17565	13,8865	13,7816	NA	NA	0,13772387
CG33309-RA	CG33308	0	0	166,848	276,217	475,078	485,569	NA	NA	0,6279808
CG33309-RB	CG33309	0	0	0	0	0	0	NA	NA	0,6279808
CG3330-RA	CG33309	0	0	0	0	0	0	NA	NA	0,6279808
CG33310-RA	e	6,16317	1,36315	9,21253	102,509	5,44061	7,17137	0,759132	0,033188	0,6279808
CG33317-RA	CG33310	0,291698	21,8742	24,9946	29,8893	0,0435471	0,0299018	0,330521	0,30935	0,6279808
CG3331-RA	CR33317	0,282376	4,19194	0,0512828	0,0152156	0,0288602	0,0217508	-0,499641	0,109415	0,6279808
CG33320-RA	OtopLb	3,9932	1,6104	10,5797	1,91829	2,99274	1,25272	-0,008813	0,974188	0,6279808
CG33321-RA	OtopLb	1,67124	1,24339	8,96972	2,03435	5,00605	0,345739	-0,104244	0,537109	0,6279808
CG33322-RA	CheB38a	0,166858	0,252037	0,26699	0	0	0,411953	0,851431	0,015734	0,6279808
CG33323-RA	CheB38b	0	0	0	0	0	0	0,128586	0,564215	0,13772387
CG33324-RA	CG33322	0,0617532	0,151986	0,0592867	0,406636	0,341939	0	0,366144	0,258699	0,6279808
CG33325-RA	Fer1	6,38337	7,02315	0,234951	7,54137	4,94377	5,22569	-0,195341	0,503836	0,6279808
CG33329-RC	gpp	24,0692	42,514	20,3255	44,8084	12,0145	2,88531	0,150588	0,670624	0,6279808
CG3332-RA	CG33325	0	14,7143	44,2646	19,7225	6,86671	6,74424	-0,56435	0,103164	0,6279808
CG3332-RB	Sp212	1,3168	0,483354	0,420551	1,35378	0,395256	0,864791	-0,56473	0,104449	0,6279808
CG33330-RA	Nop60B	38,4235	0,0222775	96,318	94,5722	0,0349657	0,0263523	0,307581	0,270777	0,6279808
CG33331-RA	Nop60B	0,0268528	27,2343	0,0257803	0,0285058	24,9967	30,6659	-0,008688	0,973748	0,6279808
CG33332-RA	Nop60B	12,3143	0,0260022	0,0234806	0,0258144	0,0412144	0,0310617	0,255713	0,347234	0,6279808
CG33333-RA	Nop60B	39,1269	26,8371	52,1114	25,6068	17,6693	31,7738	-0,041811	0,731452	0,6279808
CG33336-RA	Nop60B	0,0285466	11,5604	0,0274064	0,0304276	48,8257	6501,85	0,312371	0,259339	0,6279808
CG33336-RB	jvl	25,0235	27,0997	19,2082	38,6569	0,215311	2,57843	0,280555	0,309836	0,6279808
CG33337-RA	CG33331	0,0582166	0,0530277	0	0,0706597	0	0	0,270641	0,438706	0,13772387
CG33338-RA	CG33332	0,0509452	0,0464044	0,0750435	0,0602014	0	0	-0,158317	0,651669	0,13772387
CG33339-RA	CG33333	0	0	8,12717	35,7977	0	0,00848924	0,089587	0,798758	0,6279808
CG3333-RA	p53	4,95459	7,31094	0,0348043	7,47465	12,0892	0,0401935	-0,178349	0,50723	0,6279808
CG3333-RB	p53	0,670667	4,21477	0,391585	11,3974	0,0485977	0,362026	-0,140612	0,599352	0,6279808
CG3333-RC	CG33337	0,946157	2,65717	0,982824	2,78372	0,479106	0,383812	-0,140737	0,599045	0,6279808
CG3333-RD	p38c	0,740346	0,561965	1,59925	3,57813	15,74	5,54035	-0,151173	0,573152	0,6279808
CG3333-RE	CG33339	0,463149	0,609365	5,99974	8,70834	1,56797	0,612312	-0,138063	0,606167	0,6279808
CG33340-RA	CG33340	0,43319	2,04445	1,65556	1,38314	5,69341	1,45094	-0,051522	0,883992	0,6279808
CG33341-RA	CG33341	1,68598	0,590064	0,444235	0,0891005	7,14358	0,0909572	0,074288	0,824715	0,6279808
CG33342-RB	CG33342	0	0	0	0,216028	0	0	-0,070526	0,562659	0,6279808
CG33344-RA	CCAP-R	2,03067	18,2933	0	1,87378	9,72628	1,30932	0,359023	0,178457	0,6279808
CG33346-RA	CG33346	0,345044	0,550007	1,5735	1,62122	2,19595	1,13125	-0,461641	0,197112	0,6279808
CG33348-RA	CheB42a	0	0,0216758	0	0,0250775	0,0339676	0,0205859	-0,080649	0,53498	0,6279808
CG33349-RA	ppk25	0,218312	139,238	0,116441	289,31	273,948	1,32667	-0,048931	0,884633	0,6279808
CG33350-RA	CG3335	7,41177	3,89209	13,1427	7,42975	7,70354	7,59975	0,73772	0,021438	0,6279808
CG33351-RA	CheB42c	3,0242	678,095	2,90341	574,874	515,648	8,8894	0,1842	0,445394	0,6279808
CG3335-RA	CheB42b	0,167705	0,0205789	0	0,0237403	0,0321564	0,140165	-0,042898	0,890875	0,6279808
CG3337-RA	CG3337	15,0954	12,9181	23,518	0,0191851	0,0804408	5,38944	-0,308098	0,298923	0,6279808
CG3338-RA	Vps53	5,27047	2,2209	2,77	2,84515	0,0295873	3,41144	-0,177387	0,439076	0,6279808
CG3339-RC	CG3339	0,0212134	0,0407923	0,0407323	0,00459928	60,1616	5,30282	0,600033	0,077032	0,6279808
CG3339-RD	CG3339	0,00456462	0,00415777	0,0043823	0,592902	10,3374	58,3672	0,600033	0,077032	0,6279808
CG3340-RA	Kr	5,3831	4,45432	0,149333	0	0	0	0,039395	0,888738	0,6279808
CG3342-RA	CG3342	4,00419	9,80742	13,0897	36,0862	0,0256872	190,771	0,093743	0,684532	0,13772387
CG3344-RA	hiro	7,12451	109,906	9,91965	1,672	17,0687	13,8105	-0,704286	0,047788	0,6279808
CG33453-RA	CG3345	0,205035	0,286365	13,7518	0,448773	24,5959	0,0307915	-0,135998	0,677909	0,6279808
CG33454-RA	CG33453	0,187716	0,455958	0,18229	0	25,0823	0,315776	-0,24432	0,411048	0,6279808
CG33458-RA	CG33454	0,379746	0,345899	0,360436	0,309331	0	0,10505	0,137758	0,561086	0,6279808
CG33459-RA	CG33458	0	0,320141	0,224953	0,247399	0	2,58254	-0,904453	0,011465	0,6279808
CG3345-RA	CG33459	0,231845	0,0447816	4,52417	1,08469	1,20592	0,0316689	-0,177899	0,615948	0,6279808
CG33460-RA	pon	3,99416	0,255027	0,850893	0,606775	0,237705	0,362935	-0,259983	0,438899	0,6279808
CG33461-RA	CG33460	0,53575	0	0,109626	9,62135	8,06013	4,61748	-0,035298	0,896079	0,6279808
CG33462-RA	CG33461	0,19457	0,040566	0,0427567	0	1,08909	4,54812	0,045145	0,842433	0,6279808
CG33463-RA	CG33462	0,329282	0,104009	0,105377	6,65934	5,40017	0,0504036	-0,202759	0,569008	0,6279808
CG33464-RA	CG33463	0,906104	0,952318	0,736081	1,6571	16,8679	0,0204855	0,063294	0,835717	0,6279808
CG33465-RB	sli	29,4299	37,4756	22,9829	51,206	5,44812	19,7247	-0,103874	0,764078	0,6279808
CG33466-RA	CG33465	0,326859	0,11451	0,362082	0,444605	0,321262	0,286608	-0,112376	0,633756	0,6279808
CG33467-RA	Fs	0,0188698	1,41801	0,0301267	31,1522	0	33,9	-0,045338	0,793699	0,6279808
CG33468-RA	CG33467	0,176673	0	21,5412	3,7621	4,44499	2,4085	-1,019635	0,003392	0,6279808
CG33469-RA	CG33468	2,35531	1,33361	2,69245	153,823	0,730872	7,28394	-1,005737	0,002635	0,6279808
CG3346-RA	CG33469	2,32565	1179,52	47,1263	2,94322	0,421677	9,12308	-0,033498	0,899677	0,13772387
CG33470-RA	CG3347	0	0	0	20,8694	13,0445	7,98291	0,893543	0,004321	0,6279808
CG33470-RB	CG33470	6,56655	13,9874	11,8862	8,3534	8,82982	8,38988	0,893543	0,004321	0,6279808
CG33472-RA	CG33470	43,0219	44,2801	59,533	8,09732	4,95921	5,93045	0,224739	0,331314	0,6279808
CG33472-RB	qvr	5,04538	6,1901	0,0459464	0,0535043	25,2849	4,14995	0,223934	0,333671	0,6279808
CG33473-RB	qvr	3,11799	6,82624	29,6183	13,4815	30,2251	14,1782	0,318327	0,25423	0,6279808
CG33474-RA	luna	10,5772	0,961885	0	11,394	1,9936	6,35047	-0,915752	0,001677	0,6279808
CG33475-RA	CG33474	5,66988	61,6068	6,17619	0,0442665	18,9384	99,6979	-0,219196	0,516838	0,6279808
CG33476-RA	CG33475	0,744098	0,453382	0,357189	0,949917	1,4785	1,51771	0,149381	0,674485	0,13772387
CG33477-RA	CG33476	0,862436	0,966851	0,700607	0,250526	1,43035	0,735661	0,05328	0,797208	0,13772387
CG33478-RA	CG33477	0,2767	0,252037	0	1,21506	0,339338	0	-0,205193	0,366158	0,13772387
CG33478-RB	Or46a	0,0576578	0	0,138387	16,4881	22,4468	16,5612	0,173708	0,412757	0,6279808

gene_id	Symbol	W1_FPKM	W2_FPKM	W3_FPKM	MCM51_FPKM	MCM52_FPKM	MCM53_FPKM	FC	p-value	p-adj
CG3347-RA	Or46a	0,149945	0	0,259121	30,9043	21,8423	25,3034	NA	NA	0,6279808
CG33481-RB	CG3348	0,139498	31,0768	26,8179	0	0,00622975	0,103641	-0,365578	0,242885	0,6279808
CG33481-RC	dpr7	71,4056	51,0733	3,58001	3,37417	0,0407627	0,0197111	-0,309486	0,322864	0,6279808
CG33481-RD	dpr7	0,0595815	0,054271	3,58387	6,71078	81,0142	4,40069	-0,309023	0,323582	0,6279808
CG33482-RA	dpr7	47,6916	30,5288	3299,16	68,23	0,0984541	40,7494	NA	NA	0,13772387
CG33483-RA	CG42685	0	32,7013	0	0	0	0	-0,336532	0,3233	0,13772387
CG33484-RA	CG33483	5,62869	4,17962	2,81941	4,91825	8,49292	7,89518	-0,063027	0,772824	0,6279808
CG33484-RB	zormin	5,47265	2,87143	15,5625	15,8732	7,86229	8,61477	-0,053782	0,805961	0,6279808
CG33484-RC	zormin	0,00731359	0,0454718	3,48816	10,386	1,92465	2,73721	-0,191263	0,348158	0,6279808
CG33484-RD	zormin	0,00697985	0,00673177	4,92414	16,4313	11,2117	20,3033	-0,072094	0,747478	0,6279808
CG33485-RA	zormin	13,2245	4,73982	0,00702149	8,82934	6,2469	7,10096	-0,249524	0,297715	0,6279808
CG33486-RA	dpr9	3,55344	3,6585	0,0434003	5,22644	11,736	0,0512485	-1,045447	0,001148	0,6279808
CG33487-RA	AsnS	5,07034	8,57496	13,6888	38,0408	23,0746	10,0669	NA	NA	0,6279808
CG33488-RA	CR33487	1,25347	7,29713	7,76937	1,31003	6,52655	6,1405	-0,151915	0,508928	0,6279808
CG33489-RA	att-ORFB	0,0514743	0,0396224	0,0494184	0,0191183	7,0283	8,61093	-0,343549	0,336571	0,6279808
CG3348-RA	CG33489	6,19344	1,13171	5,94607	2,57178	4,71594	5,31732	-0,006728	0,980076	0,6279808
CG33490-RA	CG3349	0,443462	0,201968	10,4221	0,4294	0,0439979	7,77029	0,015391	0,96558	0,6279808
CG33491-RA	CG33490	4,7143	1,5176	4,54418	18,5107	0,0272063	0,0205043	0,01562	0,897983	0,6279808
CG33492-RB	CR33491	1,88021	2,93974	3,09972	7,55257	3,169	5,04877	0,236241	0,509185	0,6279808
CG33493-RA	Ir41a	0,602568	4303,78	21,0297	0,0309711	17,9302	6,63205	-0,565545	0,096672	0,13772387
CG33494-RA	CG33493	24,8042	19,0309	0,111163	1,00861	4,37358	0,107191	0,201937	0,475681	0,6279808
CG33495-RA	CG33494	28,6672	29,72	0	27,4956	4,58389	3,6303	-0,037602	0,914347	0,6279808
CG33496-RA	Dup99B	2,02094	16,355	46,2303	42,828	0,0669286	0,0504414	NA	NA	0,6279808
CG33497-RA	CR33496	1,25347	1,46796	1,46128	1,31003	6,9514	3,20922	NA	NA	0,6279808
CG33498-RA	Sdic2	6,562	1,46796	1,46128	8,57328	5,31907	3,20922	NA	NA	0,6279808
CG33499-RA	CR33498	1,80535	1,89111	1,90657	2,06749	3,49648	4,05063	NA	NA	0,6279808
CG3349-RA	Sdic4	7,80517	8,69334	8,45991	8,56577	6,49954	6,47825	0,208942	0,558409	0,6279808
CG33500-RA	bigmax	31,6465	30,3121	46,8125	0,0129953	4,30007	0,00469512	-1,186808	0,000792	0,6279808
CG33502-RA	CG33500	61,6213	23,218	138,991	382,553	17,5936	15,6122	NA	NA	0,6279808
CG33503-RA	CG33502	11,1185	1,22751	16,4387	12,172	13,7269	0,882926	-0,643145	0,050794	0,6279808
CG33505-RA	Cyp12d1-d	0,963529	0,894527	1,6001	367,494	5,65127	1,92431	-0,112415	0,694567	0,6279808
CG33506-RA	U3-55K	7,17362	43,2633	12,2458	8,42622	5,00314	8,14873	0,014508	0,961696	0,6279808
CG33507-RB	CG33506	12,1919	0,182014	20,8948	50,8044	8,2295	16,2085	-0,149539	0,541573	0,6279808
CG33508-RA	dpr2	2,02434	1,8439	494,234	2,77291	2,3547	1,95306	-0,415285	0,216842	0,6279808
CG33509-RA	ppk13	1,02721	0,913895	0,419965	2,04478	0,10862	1,54856	0,046378	0,8949	0,13772387
CG3350-RA	CG33509	0,799495	1,33634	1,99434	1,28693	2,30038	0,131863	-0,38477	0,18756	0,6279808
CG33510-RB	mRpL11	22,6492	0	2,71872	4,08525	0	0,987751	-0,116989	0,729273	0,6279808
CG33511-RA	CG33510	0,412652	0,694459	0,564384	0,332934	0,506804	25,4277	-0,258033	0,459736	0,6279808
CG33512-RA	CG33511	1,07303	0,070476	1,32838	0,688225	1,99881	0,902592	-0,311712	0,226417	0,6279808
CG33513-RA	dpr4	4,26416	17,3621	13,199	14,9774	0,0731251	10,1764	-0,460313	0,129864	0,6279808
CG33513-RB	Nmdar2	0,622614	1,57425	0,783255	1,5531	0,483736	0,0205607	-0,460914	0,126531	0,6279808
CG33513-RC	Nmdar2	0,318725	0,0175949	0,0185451	0,020141	11,8314	0,601202	-0,497405	0,09101	0,6279808
CG33514-RA	Nmdar2	0,400298	0,401081	0,530348	1,40785	1,37648	76,3587	-0,30441	0,361353	0,6279808
CG33516-RA	CG33514	20,0168	2,68615	30,2792	0,0255046	13,76	0	-0,281063	0,360899	0,6279808
CG33517-RA	dpr3	0,804495	1,84012	1,42458	1,85163	0	1,31289	-0,2654	0,404888	0,6279808
CG33517-RB	Dop2R	3,12441	0,029889	0,54499	0	0	0	0,404548	0,083758	0,6279808
CG33517-RC	Dop2R	0,0418554	0,0343791	2,97014	0	0	0,173804	-0,364284	0,229873	0,6279808
CG33517-RD	Dop2R	0,0385581	6,18836	0,0401837	0	0	0	-0,298044	0,332886	0,6279808
CG33517-RE	Dop2R	0,0328137	2,69894	0,0370181	0,462849	0	0	-0,311553	0,311943	0,6279808
CG33519-RB	Dop2R	0,0377432	3,19941	0,0315031	0,0790295	0,626932	26,1011	-0,516718	0,043856	0,6279808
CG33519-RC	Unc-89	7,3031	90,9314	12,9634	8,16451	0,0229937	13,1181	-0,516572	0,043888	0,13772387
CG3351-RA	Unc-89	0,0051854	0,0155293	22,7515	0,00523219	77,0534	13,1448	-0,278204	0,327655	0,6279808
CG33520-RA	ft	3,75979	3,85229	2,88064	6,00221	1,46318	2,40003	-0,292517	0,22327	0,6279808
CG33520-RB	Rpb4	22,6677	21,1134	24,1694	22,7068	30,6426	35,7067	-0,292511	0,222914	0,6279808
CG33520-RC	Rpb4	0,028436	0,0259015	0,0273003	0,0303017	0,0410438	0,0309331	-0,292517	0,22327	0,6279808
CG33520-RE	Rpb4	0,0278958	0,0254094	0,0267816	0,0296873	0,0402117	0,030306	-0,292511	0,222914	0,13772387
CG33520-RF	Rpb4	6,08606	5,21125	6,16525	7,88755	6,31819	6,99358	-0,187263	0,467364	0,6279808
CG33521-RA	Rpb4	0,0429312	0,0260698	0,0274777	0,0305122	0,0413289	0,031148	0,322845	0,140141	0,6279808
CG33521-RB	CG33521	52,8737	39,129	16,4872	0,0281766	5,43816	14,6557	0,320551	0,142773	0,13772387
CG33521-RC	CG33521	56,6827	0,0241939	14,9445	0,0279852	12,5687	0,0216807	0,322156	0,14106	0,6279808
CG33521-RD	CG33521	0,0265614	0,0240393	0,019509	0,0512568	76,7158	0,0216041	0,322446	0,140066	0,6279808
CG33522-RA	CG33521	0,0263916	0,0419515	0,0194432	66,2582	35,7459	0,0211689	0,004837	0,982609	0,6279808
CG33522-RB	scaf6	6,41253	0,0638469	8,15223	9,04554	4,48044	6,12819	-0,149344	0,578817	0,6279808
CG33523-RA	scaf6	0,0700945	15,7337	0,0672949	0,0830371	0,112474	0,0847674	-0,000979	0,995764	0,6279808
CG33523-RB	CG33523	15,4568	16,2233	29,3892	12,6768	5,88518	16,9828	-0,009365	0,960158	0,6279808
CG33523-RC	CG33523	12,2416	0,046061	25,02	0,0304698	0	10,9696	0,083565	0,656334	0,6279808
CG33523-RD	CG33523	17,4397	0	13,2451	40,9003	19,6138	9,4709	0,023657	0,902578	0,6279808
CG33524-RA	CG33523	0,0505682	16,2248	0,027442	1,06964	10,0523	0,058111	-0,058936	0,817721	0,13772387
CG33525-RA	CheB53b	0,161161	0,146796	0,257873	0,385936	0,279771	0,0760614	-0,207394	0,453003	0,6279808
CG33525-RC	CoRest	0,0378297	1,54655	11,5735	4,18575	17,1304	7,89028	-0,206617	0,454409	0,6279808
CG33525-RD	CoRest	0,0355693	0,0344579	0,0363187	0,0423055	6,11375	0,0329806	-0,205942	0,454097	0,6279808
CG33525-RE	CoRest	19,0358	0,0323989	0,0341486	7,43992	0,0437606	0,0193538	0,235307	0,376448	0,6279808
CG33525-RF	CoRest	0,018752	21,3133	16,4298	0,36952	1,68151	5,91306	0,225329	0,39745	0,6279808
CG33526-RA	CoRest	0,0387163	0,0170806	0,0180031	0,104934	4,84955	12,6306	-0,184041	0,345474	0,6279808
CG33526-RB	PNUTS	0,0248904	0,0226719	0,0238963	0,0262986	0,0356216	0,0268466	-0,193519	0,320823	0,13772387
CG33526-RC	PNUTS	21,9601	25,2068	28,429	28,2011	24,0053	25,6164	-0,193519	0,320823	0,6279808
CG33526-RD	PNUTS	14,1897	12,251	15,1433	15,4578	10,7887	12,7631	-0,219962	0,243887	0,6279808
CG33527-RA	PNUTS	0,024649	0,0336781	0,0354968	0,0260285	0,0352558	0,0265709	-0,138805	0,580414	0,6279808
CG33528-RC	SIFa	11,3235	8,59521	11,2461	13,845	0,0912098	6,87918	-0,962013	4,49E-05	0,6279808
CG33528-RD	Vmat	19,6412	5,81616	14,1011	4,51849	14,9358	0,0350242	-0,919572	0,000137	0,6279808
CG33528-RE	Vmat	0,0271731	6,1429	0,0260878	7,58709	0,0464721	0,588776	-0,025653	0,910613	0,6279808
CG3352-RA	Vmat	0,0278723	2,63605	5,25815	0	2,10771	2,99501	0,023801	0,938251	0,6279808
CG33530-RA	Smyd5	7,47983	28,2039	14,175	0,0191851	87,0463	12,5838	0,220803	0,467564	0,13772387
CG33531-RA	Acp53C14c	0,440506	1,04323	0,326321	0,921322	0,774735	0,583887	-0,099396	0,718343	0,6279808
CG33532-RA	Ddr	1,02776	1,18155	0	0	0,0175638	0	0,250348	0,428268	0,6279808
CG33533-RA	lectin-37Da	1,22579	0,0319348	307,294	1,61135	7,26771	6,20368	0,070254	0,842131	0,6279808
CG3353-RA	lectin-37Db	2,40674	39,3889	0,0531889	1,34581	0,0615301	3,13614	-0,311775	0,309242	0,6279808
CG33541-RA	Mst77F	0,242926	0,0829777	0,233224	86,0599	0,0262153	7,91513	NA	NA	0,13772387
CG33542-RB	CG33541	0	0,10259	0,739435	0,0214804	3,14287	0,029862	0,289526	0,418318	0,6279808
CG33543-RC	upd3	0,311959	0,338278	0,812927	0,0300529	0,699204	0,705874	0,361569	0,194936	0,6279808
CG33544-RA	CG33543	2,8993	4,66402	109,498	0	0	6,14393	0,21329	0,546042	0,6279808
CG33545-RA	Vkor	1,36758	44,5737	0,0757605	0,59859	0,277728	0,296398	-1,24621	2,01E-06	0,6279808
CG33546-RB	nab	1,59163	11,5365	1,35003	14,7877	3,34843	2,84389	-0,776126	0,000818	0,13772387
CG33546-RD	gfzf	14,311	19,5008	25,9338	10,4324	1,45091	10,825	-0,928191	4,25E-05	0,13772387
CG33547-RA	gfzf	4,20551	5,98934	6,70165	57,3545	0	40,2498	0,178908	0,499571	0,13772387
CG33548-RA	Rim	2,18777	3,23298	2,27489	3,00492	1,90984	1,23352	0,254427	0,28593	0,13772387
CG33548-RB	SmydA-8	5,52031	1,3947	19,538	27,2184	2,69962	5,12881	0,141836	0,548575	0,6279808
CG3354-RA	SmydA-8	10,633	31,204	3,30889	5,86338	21,3198	12,8383	-0,004493	0,988015	0,6279808
CG33550-RA	CG3355	0,0491026	0,83618	0,0185777	2,57295	0	0,129319	-0,104244	0,537109	0,13772387
CG33552-RA	CG3355	0,550801	0,0625206	0,109828	0,436711	0,804198	0,0877732	0,069658	0,720558	0,6279808
CG33554-RA	CheB53a	0	0	0	0,206548	0	0	0,219182	0,424699	0,6279808
CG33554-RD	CG33552	0,348502	0,190464	0	0	0	0,59857	0,219202	0,423895	0,6279808
CG33555-RB	Nipped-A	8,50495	8,29874	7,47559	11,1283	42,436	46,4838	0,280674	0,200635	0,6279808
CG33555-RC	Nipped-A	0,0057504	0,00523786	0,00552072	0,00580977	12,2285	8,59608	-0,054333	0,806742	0,6279808

gene_id	Symbol	W1_FPKM	W2_FPKM	W3_FPKM	MCM51_FPKM	MCM52_FPKM	MCM53_FPKM	FC	p-value	p-adj
CG33555-RD	btsz	5,62005	0,00823269	6,12648	4,52213	6,1133	6,15632	-0,005717	0,979215	0,6279808
CG33555-RE	btsz	0,00903828	0,00811612	0,00741806	0,00920079	1,98559	1,4217	0,213263	0,331138	0,6279808
CG33555-RF	btsz	0,00891031	0,0202602	0	0,00906784	0,0896979	0	0,215627	0,325828	0,6279808
CG33555-RG	btsz	0,0222428	19,6478	2,25754	0,0233532	1,67209	1,9645	0,281134	0,199356	0,6279808
CG33555-RH	btsz	19,7354	5,37256	7,12072	11,0811	0	5,76256	0,280579	0,200632	0,6279808
CG33556-RA	btsz	7,38329	0,00703798	30,0048	10,6398	6,18203	0	0,17598	0,460861	0,6279808
CG33556-RB	btsz	0,00772667	0	0,248188	3,89464	12,9225	17,6535	0,17556	0,461593	0,6279808
CG33557-RA	form3	7,2228	8,31166	0,0368247	8,41181	0,014642	0,0427479	-0,11183	0,754643	0,6279808
CG33558-RA	form3	0,0104916	0,00955644	62,0236	0,0108099	6,81673	79,9282	0,178178	0,577095	0,6279808
CG33558-RB	CG33557	0,802246	416,017	0,31651	0,297352	0,216361	3,5564	0,274023	0,386354	0,6279808
CG3355-RA	mim	8,13071	0	6,50048	0,0201656	0,271267	2,25509	-0,692444	0,052986	0,6279808
CG3355-RB	mim	3,28404	0,287234	2,22473	2,28339	0,419458	0,204443	-0,689024	0,054218	0,6279808
CG3356-RA	CG3356	9,6622	12,6302	0,465851	3,33896	0,188967	1,86062	0,105756	0,658612	0,6279808
CG3358-RA	CG3358	0,0680025	0,0619414	48,8638	0,0519028	3,62962	27,4396	0,152556	0,522668	0,6279808
CG3358-RC	CG3358	8,53488	9,26283	22,5613	3,67746	0,0899232	0,0247522	0,152556	0,522668	0,6279808
CG3359-RB	mfas	0,0364702	0,0332196	0,0234026	3,32023	0,0329397	5,19801	-0,182027	0,540543	0,6279808
CG3359-RC	mfas	0,0226857	0,0206637	0,0232142	41,7661	0,0348429	177,13	-0,188643	0,527188	0,6279808
CG3359-RD	mfas	14,0302	19,0308	0,0233768	4,24113	0,0345461	0,0404351	-0,192425	0,517769	0,6279808
CG3359-RE	mfas	0,0250414	0,0228094	0,0231888	25,6827	0,0348021	0,0644907	-0,179252	0,546653	0,6279808
CG3359-RF	mfas	0,0248343	0,0226208	0,0218096	35,7626	0,0345061	0,180678	-0,188659	0,525972	0,6279808
CG3359-RG	mfas	0,0212485	0,0193546	0,968205	19,5983	0,0323428	1,39714	-0,178231	0,548995	0,6279808
CG3359-RH	mfas	21,336	33,731	0,0563047	10,9515	3,72001	7,14016	-0,183051	0,538213	0,6279808
CG3359-RI	mfas	0,0248063	0,0225953	10,615	0,0256535	5,95177	1,51273	-0,186485	0,531479	0,6279808
CG3359-RJ	mfas	23,2003	29,8846	10,713	0,0238434	4,5233	4,49241	-0,182732	0,539745	0,6279808
CG3359-RK	mfas	0,0231142	0,021054	2,97542	34,8165	1,41151	5,09116	-0,187321	0,529568	0,6279808
CG3359-RL	mfas	0,0243762	0,0222036	6,99173	0,0264676	3,64175	6,1243	-0,184252	0,536068	0,6279808
CG3359-RM	mfas	0,02418	0,0220248	0,018837	0,0262358	197,8	1,18178	-0,179085	0,547013	0,6279808
CG3359-RN	mfas	0,0243493	0,022179	4,30668	0,0222568	0	3,03777	-0,182197	0,540182	0,6279808
CG3359-RO	mfas	0,0241535	0,0220006	224,498	34,5097	0	192,459	-0,178399	0,548634	0,6279808
CG3359-RP	mfas	0,0227169	0,0206921	0	0,0262045	42,1791	0	-0,182881	0,538574	0,6279808
CG3360-RA	Cyp313a1	0,0655949	21,5179	3,32019	25,5342	35,7742	3,49921	-0,103596	0,715797	0,6279808
CG3361-RA	mrt	24,0239	27,4679	0,188379	32,6297	17,442	2,6713	-0,00502	0,981023	0,6279808
CG33626-RB	cN-IIIB	4,41838	5,03576	3,31981	4,71754	2,78434	0,197374	0,156195	0,648691	0,6279808
CG33627-RA	CG33626	0,917721	1,01542	0,3776	0,0461905	161,007	1,51216	0,06906	0,58405	0,6279808
CG3362-RA	CG33627	0	0,272584	0,172383	0,33508	0,456515	0,810193	0,182705	0,447793	0,6279808
CG33630-RA	ocm	2,50946	2,48377	6,33089	0,0632042	0	2,09845	-0,258262	0,446592	0,6279808
CG33631-RA	CG33630	0,155108	3,71785	5,26248	2,9299	0,0194255	0,42157	-0,057578	0,854146	0,6279808
CG33632-RA	CG33631	0,280935	0,0170903	5,8472	0,0143414	8,78292	0,0281853	0,196567	0,567829	0,6279808
CG33635-RA	CG33632	0,608434	3,93693	5,95815	0,0199158	0,0523919	0,0357692	0,243984	0,35961	0,6279808
CG33639-RA	CG33635	6,60759	179,533	0,0257594	0,0284811	4,16785	37,5369	0,228934	0,446822	0,6279808
CG3363-RA	CG33639	1,61809	1,78184	1,4839	0	17,7708	9,20008	0,11715	0,630848	0,6279808
CG33640-RA	CG33640	0,896378	0,145651	2,87295	0,180823	0,627753	0,473113	0,425124	0,226498	0,6279808
CG33641-RA	CG33641	0,302545	4,19467	0,0279252	25,2878	0,370314	0,279091	-0,010281	0,974335	0,6279808
CG33642-RA	CG33642	0	0,0264944	0,0845948	20,8789	1,45393	0,485842	-0,011569	0,965103	0,6279808
CG33643-RA	CG33643	0,18457	0,214028	2,48007	8,178	0	0,367539	-0,601307	0,049513	0,6279808
CG33644-RA	CG33644	0,207786	3,12438	8,27014	10,5121	0	0,309956	0,182117	0,516307	0,6279808
CG33645-RA	CG33645	0,312859	8,29105	0,0115831	0,0123682	0	0	0,205888	0,499035	0,6279808
CG33647-RA	CG33647	0,749552	0,42015	42,0226	31,7152	29,9345	69,4533	-0,597001	0,091855	0,6279808
CG33648-RA	CG33648	0,311092	0,340037	0	20,0802	39,8639	0,222967	0,253637	0,306385	0,6279808
CG33650-RA	drongo	0,027943	0,0577384	0,0268269	0,0265636	0,0402843	0,0303607	-0,527259	0,037589	0,6279808
CG33651-RA	drongo	20,2078	8,76717	18,1778	0,0333203	11,3948	13,2517	-0,132296	0,677394	0,6279808
CG33653-RA	drongo	23,0614	0,873673	21,3362	0,0242291	7,30908	8,99706	0,100826	0,663316	0,6279808
CG33653-RB	drongo	0,0251271	0,035187	0,0241235	0,0243996	0,0359806	0,0271172	0,100413	0,663862	0,6279808
CG33653-RC	drongo	0,0310701	11,0358	0,0298292	32,6923	0,0451326	0,0340147	0,101963	0,659591	0,13772387
CG33654-RA	DNApol-gamma35	10,6805	9,08127	11,8994	26,8704	27,2303	0,0447381	-0,254899	0,317011	0,6279808
CG33658-RA	CG33651	0,269331	1,73565	0,0568599	76,167	1,04413	0,00538138	-0,061649	0,853507	0,6279808
CG3365-RA	Cadps	0,0112514	21,1844	38,1295	35,0703	0,0379061	0,0287638	0,595389	0,04664	0,6279808
CG3365-RB	Cadps	7,35372	16,5106	16,9323	0,0212381	0,0694277	0,0285683	0,480902	0,10901	0,6279808
CG3365-RC	Cadps	18,6701	0,0185094	0,0253375	0,0211631	81,4078	0,0523249	0,595264	0,046691	0,6279808
CG3365-RD	CG33654	0	9,03593	0,187683	0	0,947268	1,04614	0,170884	0,575983	0,6279808
CG3365-RE	CG33658	0,302545	4,03465	3,38391	2,89929	3,70024	1,54867	0,170835	0,580997	0,6279808
CG33664-RA	CG33664	0	0,154061	0	0	0,255986	0	NA	NA	0,6279808
CG33665-RA	CG33665	0	0,154061	0	0	0	0	NA	NA	0,6279808
CG33666-RA	CG33666	0	0,154061	0	0	0,221356	0	NA	NA	0,6279808
CG33667-RA	CG33667	0	0,139752	0,0981994	2,03162	1,30727	0,227491	-0,041811	0,731452	0,6279808
CG33668-RA	CG33668	0	0,154061	0	19,8064	14,4468	15,7063	NA	NA	0,13772387
CG33669-RB	CG33669	0	15,8612	0	0	0,255986	0	NA	NA	0,6279808
CG33670-RA	stg1	0,530625	0,340708	7,48025	0,27838	0,0431471	0,400372	0,80175	0,019631	0,6279808
CG33672-RA	CG33672	0,0339605	0,0309336	13,922	14,9914	2,97176	0,0200579	0,181469	0,463726	0,6279808
CG33673-RA	CG33673	0	0	262,812	0,0299437	330,847	279,91	0,049617	0,76224	0,6279808
CG33678-RA	solo	0,0984738	0,032823	6,74488	0,0207173	8,27669	9,92608	0,199119	0,556529	0,6279808
CG33679-RA	CG33679	0,506329	13,5119	2,68059	0,0234781	19,6123	17,7413	-0,249145	0,434551	0,6279808
CG33680-RA	CG3368	5,62141	6,95581	7,59361	0	0	0,013266	NA	NA	0,6279808
CG33681-RA	CG33680	0	0	0,289666	0	0	2,95771	-0,188168	0,568164	0,6279808
CG33687-RA	CG33681	0,0556194	4,45431	0,0118492	0,203464	2,92379	0,0129243	0,046767	0,889673	0,6279808
CG33688-RA	CG33687	0,857272	4,8188	1,12442	0	0,411267	0	0,022403	0,887437	0,6279808
CG33689-RA	CG33688	0,187716	0,170984	0,332478	0,331091	0,127683	0,367539	0,409866	0,195921	0,6279808
CG3368-RA	CG33689	0,415572	0,504708	0,494634	0,685833	0,201595	1,18046	0,02364	0,908061	0,6279808
CG33690-RA	CG33690	1,09482	0,293306	8,72158	1,65896	7,06861	0	-0,176126	0,615758	0,6279808
CG33691-RA	CG43736	3,50244	20,6346	2,93033	0,0124712	13,3137	13,5288	0,266075	0,347935	0,6279808
CG33691-RC	CG43736	0,0159887	0,65994	0,0153501	9,3456	0,611338	0,380973	0,26793	0,344064	0,6279808
CG33692-RB	CG43736	6,63476	20,1544	8,96086	0,0328224	20,7757	0,0127046	0,14644	0,560041	0,6279808
CG33692-RC	CG43736	5,86717	6,93967	0,0294143	0,0120141	6,2615	6,28012	0,409529	0,151629	0,6279808
CG33694-RA	cana	6,96266	0,04496	5,85858	8,89377	2,7717	0,0160977	0,032919	0,868612	0,6279808
CG33695-RC	CG33695	0,0533012	0,0429086	6,69257	0,0604222	3,54732	4,88518	0,017949	0,934328	0,6279808
CG33695-RD	CG33695	0,0493594	0,0503792	0,0511724	0,0553935	59,3707	6,70133	0,022115	0,918858	0,6279808
CG33695-RE	CG33695	0,0471073	7,89153	0,047388	0,0525653	3,42296	100,815	0,036401	0,867696	0,6279808
CG33695-RF	CG33695	0,055309	0,0139074	0,0452258	0,0630228	5,12102	0,0660408	0,03261	0,880982	0,6279808
CG33696-RA	CNMaR	0,671007	0,737251	0,948741	6,34761	0	24,5604	0,20526	0,4887	0,6279808
CG33698-RA	CheA86a	0	0	13,5362	7,08669	4,04732	3,88038	NA	NA	0,6279808
CG33700-RB	CG33700	0,297639	0,162666	0,129357	0,0965662	0,1308	0,473113	-0,547069	0,089063	0,6279808
CG33702-RA	CG33702	0,183544	0,167185	0,352427	0	1,05798	1,03982	-0,084927	0,747528	0,6279808
CG33703-RA	CG33703	0,181527	0,275579	0	0	0,627753	0,0985784	-0,152682	0,530446	0,6279808
CG33704-RA	CG33704	0	0	0	0	0	0	NA	NA	0,6279808
CG33710-RA	ebd1	8,61736	0,642234	18,729	0,0329208	27,392	21,7478	0,238046	0,354229	0,6279808
CG33714-RA	CG33710	0,202687	0,307702	0,324319	0,344958	0	0	-0,289313	0,303223	0,6279808
CG33714-RB	SLIRP1	0,0538661	12,0412	53,0529	63,8766	7,5127	0,0661065	-0,289313	0,303223	0,6279808
CG33715-RB	SLIRP1	85,9388	0,049065	0,0367181	0,0417427	67,6671	0	0,069118	0,793088	0,6279808
CG33715-RD	Msp300	0,00260391	0,00237182	0	0,002612	1,10465	1,36774	-0,250183	0,296024	0,6279808
CG33715-RE	Msp300	0,00177016	0,00161238	0	4,1897	1,15338	0,453752	0,150502	0,414416	0,6279808
CG33715-RF	Msp300	0,0258243	52,3377	0	0,0273464	84,4956	57,7255	0,253781	0,316659	0,6279808
CG33715-RG	Msp300	0,00223149	0,00203259	0	0,00223653	44,4762	38,5135	-0,118197	0,536444	0,6279808
CG33717-RA	Msp300	18,8477	15,3754	0,00249991	18,2316	0,045524	0,0343096	0,527761	0,019564	0,6279808
CG33717-RD	PGRP-LD	0,0184897	0,0425647	5,59773	0,0515176	0,0317221	0,0174418	0,586885	0,010679	0,13772387

gene_id	Symbol	W1_FPKM	W2_FPKM	W3_FPKM	MCM51_FPKM	MCM52_FPKM	MCM53_FPKM	FC	p-value	p-adj
CG33718-RC	PGRP-LD	45,8561	70,0347	2,21983	0,0195726	0,0260622	2,93597	0,528073	0,019564	0,6279808
CG33718-RD	Pmi	0,0187947	11,3371	95,6541	3,10644	1,81934	0,593803	0,557118	0,014124	0,6279808
CG33719-RA	Pmi	0,0223028	0	0,0158368	0	12,3877	2,03794	0,281176	0,248091	0,6279808
CG33719-RB	Pif1A	15,0525	15,0642	10,2024	23,2751	15,9367	0,0168435	-0,484037	0,052915	0,6279808
CG3371-RA	Pif1A	0,0159501	0,0145284	0,015313	0,0164996	0,0223489	57,4874	0,065092	0,776815	0,6279808
CG33720-RB	Pif1B	32,8554	40,8233	25,1415	16,3077	8,92169	0,0500288	0,461173	0,058943	0,6279808
CG33721-RA	CG33721	0,302545	0,0802487	0,140971	0,587722	0	0,345326	0,209026	0,448672	0,6279808
CG33722-RB	CG33722	5,10941	1,22949	0,349684	1,31321	7,76195	0,342893	0,324248	0,277419	0,6279808
CG33723-RA	CG33723	0	11,0599	0	0	0,847122	0,296302	-0,101958	0,638778	0,6279808
CG33724-RA	CheA56a	0	0,0937395	16,8939	0,0238866	27,2799	8,64099	0,044335	0,715924	0,6279808
CG33725-RB	CG33725	0,375431	0,455958	0,300364	20,7387	12,8742	16,9264	0,04419	0,886759	0,6279808
CG3373-RA	Hmu	24,9888	1,38641	0,945535	1,55318	0,0123817	0,771944	0,185636	0,530516	0,6279808
CG33748-RA	primo-1	9,95302	5,90338	12,3999	12,9671	32,9281	29,8448	-0,992637	0,002921	0,6279808
CG33748-RB	primo-1	0,0518784	0,543426	0,0498064	0,0585953	0,0793678	0,0598163	-0,99908	0,002822	0,13772387
CG33748-RC	primo-1	0,0526221	0,0479318	0,0505203	0,0595485	0,0806588	0,0607894	-1,034659	0,001761	0,6279808
CG33752-RA	CG33752	0	0,161792	0	0	4624,59	0,611506	-0,202487	0,349275	0,6279808
CG33756-RA	gdl	3,25292	112,029	14,2921	2,54117	0,0265507	0,0758749	0,411099	0,140137	0,6279808
CG33757-RA	CG33757	0	3,9515	0	41,585	2,91801	3,98842	0,01562	0,897983	0,6279808
CG33758-RA	CG33758	0,615232	28,7832	0	22,3599	0	0	-0,154451	0,609415	0,6279808
CG33764-RA	CG3376	17,489	0,0265646	1,42796	18,9683	2,48073	0,0317808	-0,117351	0,707315	0,6279808
CG33766-RA	CG3376	8,65246	5,80858	8,57695	16,2123	10,2448	11,5791	-0,019916	0,944104	0,6279808
CG33767-RA	CG33764	0	33,3766	0,35048	0,0717367	0,633284	0,0732316	-0,124633	0,598712	0,6279808
CG33768-RA	CG33766	0,307616	0,280198	0,29533	0	0	0,276794	0,01562	0,897983	0,6279808
CG33769-RA	CG33767	0	0,159224	0,284215	0	0	0	-0,013096	0,914398	0,6279808
CG3376-RA	CG33768	0	0	0,167822	0,29546	0,367266	0	0,419761	0,148782	0,6279808
CG3376-RB	CG33769	0	0	0	0,28278	0	0	0,424049	0,144384	0,6279808
CG33770-RB	CG33770	0,296039	0,431444	0,176213	0,599023	0	0	0,139907	0,619376	0,6279808
CG33771-RB	CG33771	0	0,16355	0,1888	0,292834	0,400203	0,457144	-0,013096	0,914398	0,13772387
CG33772-RB	CG33772	0	0	0	0	1,27894	3,80894	NA	NA	0,13772387
CG33773-RA	CG33773	0	0,278641	0,352427	41,2818	23,8624	0,0625997	0,035937	0,854488	0,13772387
CG33774-RA	CG33774	84,9742	64,0066	66,7845	165,332	166,881	19,8044	-0,076331	0,7779	0,6279808
CG33775-RA	CG33775	0,180535	0,0288941	0,173325	0,0350391	14,2697	2,06478	0,082162	0,779228	0,13772387
CG33777-RA	CG33777	0,381942	0,173949	2,15834	0,312262	5,49731	0,0247613	0,204925	0,456246	0,13772387
CG33779-RA	CheA84a	0	0	0,292066	8,94593	37,519	29,7387	-0,027527	0,868614	0,6279808
CG33783-RA	CG33783	0	0,379965	0,192233	70,6056	0,559364	0,0199449	-0,251944	0,304382	0,6279808
CG33784-RA	CG33784	0,299257	3,51609	0,632069	1,91219	1,91514	0,521122	-0,474918	0,172961	0,6279808
CG33785-RA	CG33785	0,0554017	6,61915	9,59164	8,84369	2,967	4,09875	-0,683873	0,004307	0,6279808
CG33786-RA	CG33786	9,23213	9,13393	21,5917	4,34443	0	16,6923	-0,684936	0,004271	0,6279808
CG33790-RA	His4r	0,12021	0,109496	0,0381995	0,164515	17,0914	0,167943	0,461488	0,039033	0,6279808
CG33791-RA	His4r	0,117225	0,106776	0,0275134	0,158959	1,82528	0,162271	-0,20313	0,506155	0,6279808
CG33791-RB	His4r	1308,15	1072,59	20,1832	1296,35	0,222837	1732,83	-0,196837	0,51907	0,6279808
CG33791-RC	Kaz1-ORFA	0,0340949	0,0131593	0,0162756	17,141	0,0514259	0,0387577	-0,190115	0,533697	0,6279808
CG33791-RD	CG33791	0,0117768	0,0106261	0,0112	0,355027	0,0163417	0,0123161	-0,20313	0,506155	0,13772387
CG33792-RA	CG33791	0,0120474	0,304301	0,0114548	0,0123496	0,0167276	0,012607	-0,143403	0,685966	0,6279808
CG33795-RA	CG33791	0,0166368	0,0149532	0,583146	0,465477	0,0233486	0,017597	0,013416	0,967336	0,6279808
CG33796-RA	CG33791	0,0172087	0,0154602	0,0162951	0,0178543	0,0241838	0,0182263	-0,058644	0,781673	0,13772387
CG33797-RA	CG33792	0,262206	102,955	0,0232483	0,0255442	0,0345997	65,8323	-0,037875	0,76416	0,6279808
CG33798-RA	CG33795	0,611814	0,278641	0,469902	0,471694	199,229	1,53863	-0,198071	0,48751	0,6279808
CG3379-RA	CG33796	0,183544	0,167185	0	0	44,6531	0,298936	-0,200388	0,487836	0,6279808
CG3379-RB	CR33797	0	4,53496	4,18451	1,49319	3,1064	0	-0,200095	0,48844	0,6279808
CG3379-RC	CG33798	0,307616	0,280198	0,177198	7,29121	1,06746	0,301617	-0,200211	0,488219	0,6279808
CG33800-RA	Oatp58Dc	1,02463	2,87448	2,67059	0,0438541	6,25625	8,74061	0,065796	0,591492	0,6279808
CG33801-RA	Oatp58Dc	4,99818	13,7025	9,64993	1,09922	1,69763	1,1259	0,141975	0,439852	0,6279808
CG33803-RA	CheA46a	0	18,926	0	28,5018	0,530291	0	NA	NA	0,6279808
CG33804-RA	His1:CG33801	0,515068	1,46796	0,881067	3,91383	2,37686	3,08538	NA	NA	0,6279808
CG33806-RA	His3:CG33803	0,635345	0,335114	2,04863	0,626684	0,527066	0,537694	NA	NA	0,6279808
CG33807-RA	His1:CG33804	0,551859	4,27832	2,01331	2,61525	0,0615725	2,14472	NA	NA	0,6279808
CG33808-RA	His3:CG33806	0,847127	0,900225	1,85523	2,28703	99,5693	1,84135	NA	NA	0,6279808
CG33809-RA	His1:CG33807	0,515068	0,301603	1,87202	0,593929	0,558946	0,457331	NA	NA	0,6279808
CG3380-RA	His2A:CG33808	0,929011	0,0331667	0,745518	2,4038	2,533	0,569191	-0,151431	0,580751	0,6279808
CG3380-RB	His3:CG33809	0,847127	0,0376479	2,11828	2,3745	1,6404	1,89616	-0,154302	0,573771	0,6279808
CG33810-RA	His1:CG33810	0,588649	2,92799	0,948841	0,0454576	2,27454	0,0403866	NA	NA	0,13772387
CG33812-RA	His3:CG33812	0,776533	1,02883	1,88596	112,249	2,52819	1,75596	NA	NA	0,6279808
CG33813-RA	His1:CG33813	0,993346	0,46916	2,47248	0,863052	0,931134	0,841026	0,01562	0,897983	0,6279808
CG33814-RA	His2A:CG33814	0,987074	1,00487	2,22976	3,62783	1,86888	2,20988	NA	NA	0,6279808
CG33815-RA	His3:CG33815	0,705939	2,87377	2,40291	2,42778	0,622182	1,75987	NA	NA	0,13772387
CG33816-RA	His1:CG33816	0,588649	0,402137	2,29588	0,646262	1,84277	0,567992	NA	NA	0,6279808
CG33817-RA	His2A:CG33817	0,812884	2,87377	2,17402	0,737529	0,709206	2,15705	NA	NA	0,6279808
CG33818-RA	His3:CG33818	0,635345	0,402137	1,39222	3,86	1,53659	1,75819	NA	NA	0,6279808
CG33819-RA	His1:CG33819	0,588649	0,771622	1,65736	3,92517	2,96059	0,592789	NA	NA	0,6279808
CG33820-RA	Oatp58Db	0,304638	0,298445	0,0786407	0,800907	0,0896379	0	NA	NA	0,6279808
CG33821-RA	His2A:CG33820	0,987074	0,335114	2,11828	2,58088	0,604427	1,74125	NA	NA	0,6279808
CG33822-RA	His3:CG33821	0,564751	0,918878	1,69488	2,44521	1,91554	1,94804	NA	NA	0,6279808
CG33823-RA	His1:CG33822	0,588649	0,835923	2,40184	0,717228	3,06391	0,666326	NA	NA	0,6279808
CG33824-RA	His2A:CG33823	1,16126	0,976308	2,22976	0,721754	1,91288	1,761	NA	NA	0,6279808
CG33825-RA	His3:CG33824	0,847127	0,70732	1,57381	2,35531	3,10485	2,02406	NA	NA	0,6279808
CG33826-RA	His1:CG33825	0,515068	0,335114	2,22523	2,56353	0,691762	0,535416	NA	NA	0,6279808
CG33827-RA	His2A:CG33826	0,870948	2,81955	2,17402	2,47522	1,67661	1,15153	NA	NA	0,6279808
CG33828-RA	His3:CG33827	0,635345	0,835923	1,57381	1,99058	0,526199	1,84259	NA	NA	0,6279808
CG33829-RA	His1:CG33828	0,551859	0,402137	2,29588	2,59448	3,46764	1,78052	NA	NA	0,6279808
CG3382-RA	His2A:CG33829	0,929011	3,03644	1,88596	3,71121	2,10436	1,62312	0,572783	0,107279	0,6279808
CG33830-RA	His3:CG33830	0,635345	0,861448	0,745518	0,871746	0,622235	1,72272	NA	NA	0,6279808
CG33831-RA	His1:CG33831	0,588649	0,46916	2,29588	2,59675	3,11306	0,722278	NA	NA	0,6279808
CG33832-RA	His2A:CG33832	0,929011	3,14488	1,82881	3,98045	2,29662	1,51547	NA	NA	0,6279808
CG33833-RA	His3:CG33833	0,776533	1,25865	0,881067	2,59647	1,90557	2,06393	NA	NA	0,6279808
CG33834-RA	His1:CG33834	0,62544	0,402137	1,82881	2,28113	0,660901	0,736092	NA	NA	0,6279808
CG33835-RA	His2A:CG33835	0,929011	1,16353	2,11828	2,25135	2,62974	1,51669	NA	NA	0,6279808
CG33836-RA	His3:CG33836	0,705939	0,900225	1,98994	2,64745	2,54054	1,73024	NA	NA	0,6279808
CG33837-RA	His1:CG33837	0,551859	0,402137	2,08395	2,4976	3,05987	1,26207	NA	NA	0,6279808
CG33838-RA	His2A:CG33838	0,812884	1,05776	2,06253	2,52975	2,65312	2,32386	NA	NA	0,6279808
CG33839-RA	His3:CG33839	0,847127	0,964527	1,92964	2,91837	0,450188	1,90301	NA	NA	0,6279808
CG33840-RA	His1:CG33840	0,62544	0,402137	1,77166	2,4447	2,60413	0,598284	NA	NA	0,6279808
CG33841-RA	His2A:CG33841	0,987074	1,21642	2,01331	2,46781	1,52583	2,0554	NA	NA	0,13772387
CG33842-RA	His3:CG33842	0,847127	0,964527	1,98994	2,90334	0,51542	0,6716	NA	NA	0,6279808
CG33843-RA	His1:CG33843	0,478278	0,368625	2,08395	2,39115	2,63492	1,75986	NA	NA	0,6279808
CG33844-RA	His2A:CG33844	0,812884	1,02883	1,38693	2,23021	2,62977	1,48161	NA	NA	0,6279808
CG33845-RA	His3:CG33845	0,635345	1,00487	1,8953	1,65433	1,84612	0,524808	NA	NA	0,6279808
CG33846-RA	His1:CG33846	0,551859	0,402137	2,08395	0,621786	0,714334	1,27084	NA	NA	0,6279808
CG33847-RA	His2A:CG33847	0,987074	2,81955	1,88596	3,65503	1,4979	2,0198	NA	NA	0,6279808
CG33848-RA	His3:CG33848	0,776533	0,915384	0,881067	2,39948	0,518646	0,538096	NA	NA	0,6279808
CG33849-RA	His1:CG33849	0,588649	0,335114	2,11927	2,42503	1,91793	1,75733	NA	NA	0,6279808
CG33850-RA	nvy	10,3346	12,9874	9,54236	10,0691	3,30602	9,7929	NA	NA	0,6279808
CG33851-RA	His2A:CG33850	0,870948	0,800961	0,948841	1,77089	1,93863	0,538272	NA	NA	0,6279808
CG33852-RA	His3:CG33851	0,847127	1,02883	1,50753	0,666899	2,80339	1,4887	NA	NA	0,13772387

gene_id	Symbol	W1_FPKM	W2_FPKM	W3_FPKM	MCM51_FPKM	MCM52_FPKM	MCM53_FPKM	FC	p-value	p-adj
CG33853-RA	His1:CG33852	0,588649	0,402137	1,82881	2,39983	0,598326	2,20915	NA	NA	0,6279808
CG33854-RA	His2A:CG33853	0,870948	2,76533	2,04863	2,30887	1,68245	0,910012	NA	NA	0,6279808
CG33855-RA	His3:CG33854	0,847127	0,900225	2,34284	0,884803	2,64669	0,681568	NA	NA	0,6279808
CG33856-RA	His1:CG33855	0,588649	0,402137	2,04863	3,89766	0,653962	1,96309	NA	NA	0,6279808
CG33857-RA	His2A:CG33856	0,754821	3,03644	1,88596	1,99366	1,90388	1,42683	NA	NA	0,6279808
CG33858-RA	His3:CG33857	0,847127	1,1399	2,10255	0,738759	0,714054	1,84551	NA	NA	0,6279808
CG33859-RA	His1:CG33858	0,699021	0,899095	1,94311	1,99603	1,48892	1,12994	NA	NA	0,13772387
CG3385-RA	His2A:CG33859	0,754821	2,92799	1,67232	2,41807	2,29501	2,51147	-0,069567	0,761532	0,13772387
CG33860-RA	CG3386	6,5889	8,95993	8,36702	4,02264	7,06571	8,21192	NA	NA	0,6279808
CG33861-RA	His3:CG33860	0,705939	1,02883	1,80218	2,24863	2,51297	0,766902	NA	NA	0,13772387
CG33862-RA	His1:CG33861	0,62544	0,402137	1,77166	0,718579	0,622465	1,77392	NA	NA	0,13772387
CG33863-RA	His2A:CG33862	0,812884	1,21642	1,86934	2,09863	2,54536	0,524852	NA	NA	0,13772387
CG33864-RA	His3:CG33863	0,635345	0,335114	0,948841	0,59393	2,86306	1,48062	NA	NA	0,6279808
CG33865-RA	His1:CG33864	0,478278	0,835923	2,11927	3,79652	0,508736	1,9083	NA	NA	0,6279808
CG33866-RA	His2A:CG33865	0,812884	2,87377	1,94311	3,91349	2,32011	1,73496	NA	NA	0,13772387
CG33868-RA	His3:CG33866	0,776533	0,900225	0,881067	2,30953	2,60904	1,72135	NA	NA	0,13772387
CG33869-RA	His2B:CG33868	1,4882	1,21642	2,06253	2,11392	1,83	1,21613	NA	NA	0,6279808
CG3386-RA	His4:CG33869	0,690908	0,915384	2,22523	2,54726	1,91785	1,68022	-0,266412	0,207839	0,6279808
CG33870-RA	His2B:CG33870	1,66678	2,98221	2,11828	1,96546	1,8028	1,27427	NA	NA	0,6279808
CG33871-RA	His4:CG33871	1,18887	0,964527	2,18991	2,42629	2,13345	2,14591	NA	NA	0,6279808
CG33872-RA	His2B:CG33872	1,4882	1,31088	0,948841	4,01138	2,54766	1,61331	NA	NA	0,6279808
CG33873-RA	His4:CG33873	0,938578	0,402137	0,881067	2,41825	1,32193	1,01771	NA	NA	0,6279808
CG33874-RA	His2B:CG33874	1,54772	1,05776	1,72807	4,10801	2,61952	0,757258	NA	NA	0,6279808
CG33875-RA	His4:CG33875	1,00115	1,02591	0,745518	2,45083	2,53834	1,59111	NA	NA	0,6279808
CG33876-RA	His2B:CG33876	1,4882	0,951983	1,83956	2,39346	1,67481	1,85475	NA	NA	0,6279808
CG33877-RA	His4:CG33877	0,753717	1,11065	2,17402	3,98345	3,15387	1,5154	NA	NA	0,6279808
CG33878-RA	His2B:CG33878	1,42867	2,92799	1,88596	0,538141	2,29407	1,272	NA	NA	0,6279808
CG33879-RA	His4:CG33879	0,565288	1,02883	2,00679	2,33047	1,52288	1,63177	NA	NA	0,6279808
CG33880-RA	gsb	2,79867	2209,32	14,7744	34,8828	34,0204	2,56571	NA	NA	0,6279808
CG33881-RA	His2B:CG33880	1,4882	1,11065	1,56783	1,84706	2,48714	1,42938	NA	NA	0,6279808
CG33882-RA	His4:CG33881	0,565288	0,858172	0,948841	2,22402	2,35203	1,96017	NA	NA	0,6279808
CG33883-RA	His2B:CG33882	1,60725	2,81955	1,71451	3,8133	1,79747	1,10243	NA	NA	0,13772387
CG33884-RA	His4:CG33883	1,06777	3,03644	2,17402	3,89992	1,52818	1,61216	NA	NA	0,6279808
CG33885-RA	His2B:CG33884	1,4882	1,43029	0,881067	0,66609	2,45722	1,01526	NA	NA	0,6279808
CG33886-RA	His4:CG33885	1,00496	3,03644	1,82881	3,77467	1,885	1,66089	NA	NA	0,6279808
CG33887-RA	His2B:CG33886	1,4882	1,02981	1,01662	0,580765	2,08117	0,625609	NA	NA	0,13772387
CG33888-RA	His4:CG33887	1,13058	1,20144	0,813292	0,631117	0,609621	2,31042	NA	NA	0,13772387
CG33889-RA	His2B:CG33888	1,42867	2,92799	1,90734	4,20273	1,6912	1,17174	NA	NA	0,13772387
CG3388-RA	His4:CG33889	1,2562	1,02883	1,92964	0,735897	2,23304	1,7759	0,141295	0,656484	0,13772387
CG33890-RA	Cad88C	1,13165	1,29859	1221,07	2,61066	2,31507	0,492164	NA	NA	0,13772387
CG33891-RA	His2B:CG33890	1,54772	1,26931	2,11828	2,54651	2,43302	1,21441	NA	NA	0,13772387
CG33892-RA	His4:CG33891	0,882693	0,835923	1,39222	2,20111	2,04022	1,89446	NA	NA	0,13772387
CG33893-RA	His2B:CG33892	1,42867	1,21642	2,06253	2,33796	2,6385	1,20002	NA	NA	0,13772387
CG33894-RA	His4:CG33893	0,756594	0,804018	0,609969	0,688591	2,45808	0,672099	NA	NA	0,13772387
CG33895-RA	His2B:CG33894	1,60725	1,05776	1,82881	4,15612	1,76514	1,65299	NA	NA	0,13772387
CG33896-RA	His4:CG33895	0,819644	1,16353	0,813292	3,87474	1,65816	1,20433	NA	NA	0,13772387
CG33897-RA	His2B:CG33896	1,42867	2,98221	1,88596	1,88485	2,17604	1,50113	NA	NA	0,13772387
CG33898-RA	His4:CG33897	0,756594	2,87377	2,40184	2,13422	2,43709	1,32391	NA	NA	0,13772387
CG33899-RA	His2B:CG33898	1,4882	1,00487	0,813292	2,25003	1,69223	1,54413	NA	NA	0,13772387
CG3389-RA	His4:CG33899	0,630495	1,09117	1,82881	2,60541	2,62482	1,12548	-0,020087	0,952843	0,13772387
CG33900-RA	His2B:CG33900	1,60725	1,11065	0,745518	2,12577	2,36417	1,52901	NA	NA	0,13772387
CG33901-RA	His4:CG33901	1,25144	0,968911	0,881067	2,48219	2,38314	1,26715	NA	NA	0,13772387
CG33902-RA	His2B:CG33902	1,54772	1,09313	1,77166	2,41714	1,88824	1,47569	NA	NA	0,6279808
CG33903-RA	His4:CG33903	1,00115	0,402137	145,793	24,1353	0,6768	0,0464048	NA	NA	0,13772387
CG33904-RA	His2B:CG33904	1,42867	1,0829	1,86225	0,0395622	2,09128	70,8804	NA	NA	0,13772387
CG33905-RA	His4:CG33905	1,06777	108,656	1,92964	3,96166	0,506522	1,3863	NA	NA	0,13772387
CG33906-RA	His2B:CG33906	1,54772	16,0147	1,82881	2,18769	3,94906	1,24647	NA	NA	0,13772387
CG33907-RA	His4:CG33907	1,24671	0,301603	2,36933	2,58596	24,4264	1,23416	NA	NA	0,13772387
CG33908-RA	His2B:CG33908	2,14946	1,07881	0,813292	6,49117	0,0535873	2,41317	NA	NA	0,13772387
CG33909-RA	His4:CG33909	1,78428	0,900225	2,33144	2,16661	3,40565	2,70408	NA	NA	0,13772387
CG33910-RA	His2B:CG33910	2,31548	4,37252	3,03922	6,36575	2,55984	1,61356	NA	NA	0,13772387
CG33911-RA	CG33911	0,0390365	0,0350805	0,036975	0,0420624	0,0226437	0,0429389	-1,093763	8,64E-06	0,13772387
CG33912-RA	CG33912	0,199024	0,181285	0	0,720644	0	0,549958	-0,037875	0,76416	0,13772387
CG33914-RA	CG33914	0,136614	32,5035	0,131158	0,196973	0,266802	42,0591	0,692199	0,05307	0,13772387
CG33919-RA	CG33919	1,4913	1,14939	0,826	3,62919	0,956009	2,3392	0,365469	0,306942	0,13772387
CG33920-RA	CG33920	0,91265	19,7763	0	0,189882	2,83355	0,0298752	-0,388588	0,276946	0,13772387
CG33922-RA	CG33922	0	0,254596	0,177198	0,376335	0	0	0,096037	0,650417	0,13772387
CG33923-RA	CG33923	0,167705	0,182383	6,9148	0,254394	0	0	0,095077	0,702455	0,13772387
CG33924-RA	CheB74a	0,271247	4,18377	0,30208	10,5909	21,6475	20,4499	0,089286	0,678247	0,13772387
CG33926-RA	CG33926	1,73661	0,733041	2,03323	0,275672	1,05527	1,02689	0,553412	0,119533	0,13772387
CG33927-RA	CG33927	0	0,0144102	0,173325	0,845075	0,245327	0,0167013	0,039409	0,884103	0,13772387
CG33928-RA	CG33928	0,36506	0,11201	0,467306	0,290254	0,393151	0	0,104322	0,736603	0,13772387
CG33929-RA	CR33929	0	0	3,44231	0,216028	0	0	-0,041811	0,731452	0,13772387
CG33932-RA	CG33932	21,3869	23,842	27,0705	2932,26	65,0263	99,1999	0,810824	0,012836	0,13772387
CG33933-RB	Indy-2	0	0	2,96454	16,6953	8,36567	7,81943	NA	NA	0,13772387
CG33934-RA	CG33934	0	0	0,0575302	3,06151	1,88311	2,36591	0,01562	0,897983	0,13772387
CG33934-RB	CG33934	0	0	4,26153	20,0353	7,03874	12,4383	0,01562	0,897983	0,13772387
CG33936-RA	CG45050	184,202	216,802	0,0406212	209,483	112,78	113,67	0,450748	0,025465	0,13772387
CG33936-RB	CG45050	0,0279037	0,0254166	0,0486602	0,0304276	0,0412144	0,0310617	0,45255	0,024654	0,13772387
CG33936-RC	CG45050	0,0265721	0,0242037	5,78838	0,0281887	0,0381818	0,0287761	0,484806	0,019722	0,13772387
CG33936-RD	CG45050	0,0285713	0,0260247	9,3835	0,0304558	0,0412525	0,0310904	0,484864	0,019747	0,13772387
CG33937-RA	CG45050	0,0445355	0,040566	0,0129313	0,0493748	0,0668785	0,0504036	-0,274599	0,347678	0,13772387
CG33937-RB	CG45050	0,0463798	0,042246	13,3259	0,0516586	0,069972	0,0527351	-0,255852	0,375927	0,13772387
CG33943-RA	Fatp3	0,029164	34,7392	12,76	5591,94	68,957	78,7639	0,132775	0,667846	0,6279808
CG3394-RA	Fatp3	7,27937	0,0244594	12,3127	0,0355111	0,0386112	0,0290998	-0,630992	0,017816	0,13772387
CG3394-RB	BG642312	3,94755	3,48506	5,07261	4,06473	0,716282	3,71578	-0,634108	0,01727	0,13772387
CG33950-RA	RpS9	1681,09	1,43523	1,97442	2,08815	2,76147	21,1259	-0,238813	0,24304	0,13772387
CG33950-RB	RpS9	0,094304	9,46727	34,1697	5,10923	67,8623	5,89488	-0,410414	0,039594	0,13772387
CG33950-RC	RpS9	0,0755439	3,33746	6,38506	3,20447	15,1231	2,37243	-0,242947	0,236212	0,13772387
CG33950-RD	trol	0,0052124	0,00603696	13,2216	0,00525975	35,7768	0,0234574	-0,243681	0,234779	0,13772387
CG33950-RE	trol	10,902	11,718	0,00493185	6,34665	0,0543376	0,025292	-0,231431	0,256736	0,13772387
CG33950-RF	trol	0,00513703	0,140742	0,00488176	0,0051828	0,0523919	0,0222692	-0,252008	0,217093	0,13772387
CG33950-RG	trol	0,00508485	2,34408	14,7697	0,00512954	0,0311247	0,0245535	-0,243175	0,235746	0,13772387
CG33955-RC	trol	19,9173	5,0254	6,66073	32,2857	0,0335589	0,0222104	0,178557	0,502452	0,13772387
CG33956-RA	trol	5,43914	0,0158734	0,00636298	10,743	0,0295481	0,0233111	0,306698	0,240941	0,6279808
CG33956-RB	trol	0,00994154	0,11486	0	0,00670959	0,0325789	12,618	0,341026	0,189088	0,13772387
CG33956-RD	eys	2,34022	3,16205	2,197	0,0894634	1,74284	1,48882	0,342977	0,188181	0,13772387
CG33956-RF	kay	0,0193336	0,0202602	4,20717	24,1296	0,0316321	0,0584138	0,337875	0,19344	0,13772387
CG33957-RB	kay	12,1413	19,7781	0,180389	23,8067	6,96844	0,0573676	-0,033137	0,899335	0,13772387
CG33957-RC	kay	29,6342	3,93847	423,809	0,0208682	13,7877	0,118351	0,076254	0,768575	0,13772387
CG33958-RA	kay	0,0199826	4,34119	0,0487724	0,697598	434,177	3,11467	-0,065136	0,832369	0,13772387
CG33958-RB	Plp	24,8367	30,0408	27,244	26,1503	0,99343	15,8703	-0,059432	0,867428	0,13772387
CG3395-RA	Plp	2,84067	2,84954	0,0258328	0,0394674	0,630417	2,61587	-0,770955	0,0112	0,13772387
CG3395-RB	CG33958	0,797875	0,934405	0,899754	0,709431	22,069	0,992895	-0,76879	0,011328	0,13772387

gene_id	Symbol	W1_FPKM	W2_FPKM	W3_FPKM	MCM51_FPKM	MCM52_FPKM	MCM53_FPKM	FC	p-value	p-adj
CG3395-RD	CG33958	0,0322059	0,0293354	0,0309196	0,0346339	4,069	0,0353556	-0,770955	0,0112	0,13772387
CG33960-RA	Ocho	6,23096	5,09542	75,7518	11,0337	22,0868	0,0331595	0,155343	0,579761	0,13772387
CG33960-RB	Sema2b	5,42895	7,30015	4,12326	6,75689	2,24987	0,0347707	0,151916	0,587373	0,13772387
CG33962-RA	Sema2b	5,19532	0	4,99555	8,83685	4,16242	0,067607	-0,060347	0,691408	0,13772387
CG33962-RC	Cp7Fa	0	0,0569049	0,0196581	0,0217858	0,685966	0,0740376	-0,060347	0,691408	0,13772387
CG33965-RA	Cp7Fa	0	0,153589	0,0195732	0,0216854	0	0,156142	-0,948186	0,007949	0,13772387
CG33966-RA	CG33965	1,66229	0,599341	9,94121	2,41391	8,97139	8,57514	-0,131699	0,703882	0,13772387
CG33967-RA	CG33966	6,38233	26,4619	9,46965	3,92981	84,6256	12,4379	0,133017	0,624103	0,13772387
CG33968-RA	kibra	12,4633	16,8692	11,0536	18,7131	0,0590469	8,19781	0,316437	0,177992	0,13772387
CG33969-RA	drd	28,0591	3,19433	0,0223553	26,7263	26,4535	17,1079	0,228457	0,287811	0,13772387
CG33969-RB	CG33969	6,23544	7,66331	1,60825	42,6941	48,8518	128,683	0,154507	0,403654	0,6279808
CG3396-RB	CG33969	0,041126	16,6115	22,5143	2,6731	0,0561494	0,0322842	-0,243109	0,374061	0,13772387
CG33970-RA	CG3397	0,413319	181,063	27,3487	0	0	152,698	0,334245	0,156629	0,13772387
CG33970-RB	CG33970	8,16616	9,69507	6,5365	0,0791003	0	7,54218	0,480619	0,041731	0,13772387
CG33971-RA	CG33970	23,0567	30,6936	20,6236	0,10111	0	12,0696	-0,93328	0,006756	0,13772387
CG33976-RA	Ir62a	0,290284	0,694078	0,0257908	6,46484	3,85237	0,0291124	0,147462	0,552036	0,13772387
CG33977-RA	Octbeta2R	3,87596	5,24904	4,78434	5,19809	4,08021	75,0049	-0,377876	0,212673	0,13772387
CG33978-RA	CG33977	39,13	3,59428	5,99208	8,52246	7,80088	7,06099	0,418368	0,131186	0,13772387
CG33979-RA	CG33978	22,7799	6,32448	16,058	20,5536	0,0439329	0,0222766	-0,194924	0,353954	0,13772387
CG33979-RB	capt	0,022094	3,88408	0,0212116	4,93615	3,95483	0,0236721	-0,207445	0,316406	0,13772387
CG3397-RA	capt	39,8382	33,7149	55,026	50,1464	39,0656	46,4823	-0,887492	0,01239	0,13772387
CG33980-RA	Vsx2	0,56044	0,574299	0,443896	2,45861	1,47689	0,374469	0,098864	0,764697	0,6279808
CG33981-RA	CG33981	0,971338	0,00850732	0,00896675	5,48732	2,2396	14,7545	-0,010325	0,964814	0,13772387
CG33981-RB	CG33981	0,0112132	0,0102138	0,0107654	22,5164	13,0036	13,7803	0,07152	0,75948	0,13772387
CG33983-RA	obst-H	20,5162	19,4677	23,9259	29,9718	3,77092	31,3272	-0,450312	0,047719	0,13772387
CG33984-RA	CG42728	23,4565	16,2598	33,3978	0,0196017	8,31482	46,9616	-0,804432	0,016528	0,13772387
CG33985-RA	CG33985	12,6368	0,716506	3,54944	28,3534	7,34629	36,4607	-0,369654	0,236468	0,13772387
CG33986-RA	CG33986	1,45524	0,0581137	0,061252	6,81596	12,9773	9,68263	-0,451501	0,206953	0,13772387
CG33988-RA	Mid1	5,43176	0,0311847	23,6379	0,0381423	0,183071	27,6266	0,13794	0,595088	0,13772387
CG33989-RC	pHCl-1	0,468681	0,0218568	0,201299	1,01962	0,443936	0,51622	-0,514543	0,125904	0,13772387
CG33989-RD	pHCl-1	0,0239956	0,418458	0,610486	0,025299	0,0342676	0,255802	-0,583161	0,071242	0,13772387
CG33989-RE	pHCl-1	0,282711	0,136059	0,0226183	0,782168	1,46229	0,0253301	-0,532067	0,096054	0,13772387
CG33989-RF	pHCl-1	0,308704	0,605324	0,152967	0,0207957	0,0281679	0,112771	-0,530444	0,096488	0,13772387
CG33990-RA	capu	3,13143	0,0168228	0,0177313	0,0192187	0,216834	0,0196192	0,469119	0,160876	0,13772387
CG33991-RA	capu	4,94311	0,0164369	0,0173246	0,0187591	0,077721	0,01915	-0,049957	0,819037	0,13772387
CG33991-RB	capu	0,018469	0,0142128	0,0149803	0,0161281	1,78167	0,0164642	0,316309	0,208311	0,13772387
CG33991-RC	capu	0,0180453	1,10365	0,984286	0,0160534	2,69466	0,016388	0,307404	0,21696	0,13772387
CG33991-RD	capu	0,0156036	0,0164011	0,0172868	0,0187165	0,0260318	0,0191065	0,310989	0,218766	0,13772387
CG33991-RE	capu	0,0155339	0,014186	0,0149521	0,0160966	0,0254093	0,016432	0,323713	0,194968	0,13772387
CG33991-RF	capu	0,018006	0,0134175	0,0141421	2,38813	0,0218456	1,03282	0,324426	0,193804	0,13772387
CG33992-RA	capu	0,0155741	0,0937484	0,757553	0,532469	0,0217445	0,950052	0,022403	0,887437	0,13772387
CG33993-RA	capu	0,0147304	0	0,0494313	8,83658	0,0253516	10,6553	0,524544	0,062917	0,13772387
CG33995-RA	IM14	3,9833	4,06711	3,8242	11,6389	4,90698	0,854637	0,560606	0,017967	0,13772387
CG33995-RB	nuf	0,0250414	0,0228094	0,0240412	0,0264676	0,0358505	0,0270191	0,555539	0,018583	0,6279808
CG33995-RC	nuf	9,96402	10,9586	10,4646	16,2247	5,12372	5,30471	0,560992	0,017939	0,13772387
CG33996-RB	nuf	51,0879	71,2481	36,2763	43,4269	23,7578	34,3677	0,558896	0,00958	0,6279808
CG33998-RA	nuf	10,1506	14,6551	0,0199696	14,4242	7,32589	7,28872	0,305041	0,27408	0,6279808
CG3399-RA	nuf	19,343	14,9889	20,7797	0,0227643	12,5474	6,70032	-0,111392	0,678348	0,6279808
CG3399-RB	nuf	0,027616	0,0251545	0,026513	0,0293698	13,2142	8,15414	-0,087647	0,743718	0,6279808
CG3399-RD	CG43755	0,0909301	0,0641647	0	17,5288	1,94228	0	-0,084357	0,75151	0,13772387
CG3399-RE	CG33993	10,5683	13,4568	10,199	11,4284	3,75604	6,54538	-0,105397	0,693375	0,13772387
CG3399-RF	CG33995	39,2456	45,5142	4,76802	37,8029	31,614	3,18081	-0,118034	0,658441	0,6279808
CG3399-RG	CG33995	0,0255218	0,023247	0,0287478	0,0276099	0,0373978	4,12966	-0,03778	0,884921	0,6279808
CG3399-RH	CG33995	0,028432	0,0258978	0,0275852	0,0310587	0,0420692	0,0312783	-0,087647	0,743718	0,6279808
CG3399-RI	dpr13	9,1868	0,0488924	0,0515327	10,1666	2,87171	0,0123342	-0,106007	0,692521	0,6279808
CG3399-RJ	CG33998	0,45886	0,120391	16,754	0	0,385398	178,438	-0,087294	0,743969	0,6279808
CG34000-RA	Pfrx	15,5063	0,0180758	0,019052	0,0236636	0,0320179	0,0241307	NA	NA	0,6279808
CG34001-RA	Pfrx	0,022554	0,0204948	0,0216016	4,78289	0,0320524	0,0241567	0,027151	0,931467	0,13772387
CG34002-RA	Pfrx	0,0225772	0,0205158	0,0216237	0	0,0299346	2,58996	0,107399	0,450552	0,13772387
CG34003-RA	Pfrx	0,0229696	0,0192248	0,020263	1,83435	0,679351	79,8124	0,441059	0,094367	0,13772387
CG34005-RB	Pfrx	0,0198446	73,9623	98,9524	99,7085	90,8958	0,0553837	-0,516821	0,14873	0,13772387
CG34006-RA	Pfrx	0,0225003	0,0441357	0,0465192	0,0542532	0,0734863	0,640612	0,286752	0,423032	0,6279808
CG34007-RA	Pfrx	0,0225233	0,0192556	0,0202954	3,09915	0	0,257428	-0,011886	0,967705	0,6279808
CG34008-RA	Pfrx	0,021106	8,32542	5,47001	0,0229866	0,34157	0,0225984	0,324175	0,199797	0,6279808
CG3400-RA	CG34000	0	0	0,0273225	28,5311	0	0,0510928	0,015845	0,931432	0,6279808
CG3400-RB	CG34001	0,153665	0,0219365	0,03098	0,0347068	13,1919	0,295638	0,011123	0,952069	0,6279808
CG3400-RD	CG34002	0,634062	0,243178	0,736892	0,270425	0,849984	0,206374	0,014481	0,937364	0,6279808
CG3400-RE	NimB3	124,81	139,783	88,0207	155,431	136,638	152,821	0,009671	0,9581	0,6279808
CG3400-RF	CG34005	7,59233	0,705676	10,5122	0,0367387	1,35534	14,015	0,015977	0,93094	0,6279808
CG3400-RG	lncRNA:CR34006	0,48172	7,40407	0,65518	17,8429	29,1484	27,8185	-0,122036	0,543267	0,6279808
CG3400-RH	CG34007	43,1232	1,29348	22,2585	112,93	6,39721	0	0,014481	0,937364	0,6279808
CG3400-RI	CG34008	0,112821	0,102765	0,108315	4,08425	45,4668	0,0310617	0,015845	0,931432	0,6279808
CG34010-RA	betaTub60D	120,423	136,44	119,111	84,2581	2,48645	0,0362511	-0,478391	0,118849	0,6279808
CG34011-RA	Glyat	7,8549	0,00954281	1,57405	8,21609	26,8087	6,86453	0,011159	0,972445	0,6279808
CG34012-RA	CG34011	0,289807	0,351968	0,0310406	0,0347801	7,95324	0,478211	-0,740682	0,033383	0,6279808
CG34015-RA	CG34012	0,554095	18,8063	17,2512	1,43292	1,53407	1,30937	-0,21202	0,504096	0,6279808
CG34016-RA	CG34015	14,7884	11,3208	23,0336	21,1616	88,7411	34,8505	-0,310858	0,367872	0,6279808
CG34017-RA	CG34016	0,0944388	0,129032	4,72563	6,41982	30,9936	9,57608	-0,033508	0,918876	0,6279808
CG3401-RA	CG42588	2,81966	0,0238111	0,025097	1,37948	1,88579	1,81494	-0,084761	0,788008	0,6279808
CG34021-RA	CG3402	35,6024	3,53311	7,36971	5,6961	5,37258	6,50052	0,043378	0,833002	0,6279808
CG34022-RA	CG34021	0	5,05503	11,4394	6,98906	0,472191	0	-0,933023	0,007602	0,6279808
CG34023-RA	mwh	1,26461	1,53586	1,11293	7,86503	2,25831	3,05671	NA	NA	0,6279808
CG34024-RA	CR46300	0	0,15006	0	0,0136747	174,763	0,0139596	NA	NA	0,6279808
CG34025-RB	lncRNA:CR34024	0	6,1863	1,01405	0	1,3213	0	-0,902883	0,010454	0,6279808
CG34026-RA	CG34025	0,601236	6,68166	3,20443	10,4974	2,1571	4,11671	-0,304107	0,395437	0,6279808
CG34027-RA	CG34026	18,9455	3,06033	24,9984	18,5862	49,8952	51,6921	0,231329	0,335905	0,6279808
CG34028-RA	CG34027	0	0,032997	0,0626021	0,0719718	9,13569	0	-0,220356	0,389604	0,6279808
CG34029-RA	CG34028	0	0	0,166939	0,433191	0,784888	0,274534	0,047993	0,769145	0,6279808
CG3402-RA	CG34029	0	0,0638471	0,4096	0,998322	0	0,287516	0,041564	0,841903	0,6279808
CG34030-RA	Mob4	42,4238	45,5487	52,965	53,9187	29,5603	35,4063	-0,064503	0,680926	0,6279808
CG34031-RA	CG34030	0	2,28294	0,13216	2,66725	56,0525	1,99443	-0,843125	0,013726	0,6279808
CG34032-RA	CG34031	4,97272	4,46745	0,0404657	13,5468	3,43177	13,4743	-0,122106	0,714268	0,6279808
CG34033-RA	CG43894	0	0,55256	0,6272	0,0243906	26,1214	34,5705	-0,099955	0,68967	0,6279808
CG34034-RA	CG34033	0	0,296778	0,375366	0	0,947268	0,713918	-0,767031	0,030798	0,6279808
CG34034-RB	CG34034	0,129645	0,0138817	15,9727	0,182768	9,52329	0,186577	-0,802195	0,012816	0,6279808
CG34035-RA	CG34034	3,28303	15,754	0,124467	8,30968	6,32533	4,90894	-0,729266	0,001837	0,6279808
CG34038-RB	CG42822	16,855	3,14986	29,9008	2,75911	39,1966	0,112573	-0,491908	0,126821	0,6279808
CG34039-RA	CG34038	27,4102	0	0	52,8521	44,0702	0,242122	0,113447	0,676308	0,6279808
CG3403-RA	CG34039	8,84349	6,18077	10,6796	10,3767	7,97717	9,27465	0,191162	0,33195	0,6279808
CG34040-RA	CG34040	0	2,16924	0,0796945	0	20,5144	0	-0,190121	0,33449	0,6279808
CG34041-RA	CG34041	0,13534	0	0	1,49426	0,292571	2660,56	0,142922	0,475949	0,6279808
CG34042-RB	ppk10	0,294577	0,185761	0,696151	0,0643775	0,0871997	94,8979	0,279513	0,433046	0,6279808
CG34043-RB	CG34043	1,67803	1752,4	2367,9	0,15485	11,5143	0,159576	-0,282113	0,428175	0,6279808
CG34044-RA	lncRNA:CR34044	0,271247	0,444728	0,156248	0,883291	0,530468	0,662018	-0,195053	0,561219	0,6279808

gene_id	Symbol	W1_FPKM	W2_FPKM	W3_FPKM	MCM51_FPKM	MCM52_FPKM	MCM53_FPKM	FC	p-value	p-adj
CG34045-RA	CG34045	2,09509	1,25119	9,88112	2,62524	0,0292671	57,1762	-0,62936	0,06746	0,6279808
CG34046-RA	lncRNA:CR34046	0	0,752331	7,05572	3,91874	0,0130017	0,00979889	0,090184	0,772665	0,6279808
CG34047-RA	lncRNA:CR34047	0	0,408506	249,057	0,214621	262,333	0,219093	0,126146	0,614632	0,6279808
CG34048-RA	CG44153	0,190971	0,811764	0,855602	1,76186	11,8722	0,0924521	-0,150356	0,658624	0,6279808
CG34049-RA	CG34049	0	0	0	41,1426	0	0	NA	NA	0,6279808
CG34050-RA	CG34050	0	0,0162769	0	0	480,325	468,76	NA	NA	0,6279808
CG34051-RA	CG34051	0,393309	0,0693393	4,63042	0,45358	0,749149	0,0182313	0,051433	0,87942	0,6279808
CG34052-RA	lncRNA:CR34052	0	37,9571	0	65,7573	23,7532	19,6477	-0,041811	0,731452	0,6279808
CG34053-RB	CG42823	0,208661	0,0169986	0,00785822	11,3896	0,0618713	19,0245	0,042412	0,90209	0,6279808
CG34054-RA	CG34054	13,7998	10,1199	0,0252067	10,4743	28,3661	0,0284115	-0,639061	0,056688	0,6279808
CG34056-RA	CG34056	11,8916	0,335768	13,5101	1,19152	0	0,712427	0,15735	0,515437	0,6279808
CG34057-RA	CG34057	0,679873	16,1642	0,766235	1,45821	4,24355	0,069451	1,20025	0,000609	0,6279808
CG34057-RB	CG34057	2,9958	2,4771	2,48257	17,5387	0,0921515	0,37806	1,20025	0,000609	0,6279808
CG34058-RA	ppk11	0,0639032	0,116415	6,21806	5,5914	0,313453	20,4474	-0,294271	0,297258	0,6279808
CG34059-RA	ppk16	0,124047	0,225982	3,60266	0,019041	45,1178	2,34112	-0,082408	0,811002	0,6279808
CG34063-RA	mt:ND2	47,6249	51,1823	32,7695	34,6142	0,0155937	10,4412	-0,702078	0,038745	0,6279808
CG34067-RA	mt:CoI	6393,16	4158,57	3283,56	5570,34	11285,6	8481,2	-0,797097	0,012263	0,6279808
CG34069-RA	mt:CoII	6850,57	4382,32	3478,93	6041,72	11504,2	11083,5	-0,459503	0,150258	0,6279808
CG34072-RA	CG3407	3,7043	7,93215	6,04645	3,99066	2,57307	2,78989	-0,217808	0,393231	0,6279808
CG34073-RA	mt:ATPase8	0,407876	2291,14	0,391585	1994,61	4317,07	3615,33	-0,713015	0,039395	0,6279808
CG34074-RA	mt:ATPase6	2800,05	0,371522	1182,74	4,70122	6,36783	4,79918	-0,423931	0,204974	0,6279808
CG34076-RA	mt:CoIII	11435,9	2171,84	4710,1	2544,44	7722,11	6282,12	-0,473146	0,178033	0,6279808
CG3407-RA	mt:ND3	518,434	300,847	229,286	399,6	1764,6	1604,52	0,129634	0,583827	0,6279808
CG34083-RA	CG3408	13,907	1482,6	0,0244583	26,8986	0,0365105	0,0275165	-0,868491	0,010476	0,6279808
CG34085-RA	mt:ND5	637,864	40,239	359,138	519,953	1017,29	1113,01	-0,573627	0,086375	0,6279808
CG34086-RA	mt:ND4	753,655	458,051	390,949	491,989	0,95596	975,976	NA	NA	0,6279808
CG34089-RA	mt:ND4L	0,278017	0,253237	0,266913	0,705763	1249,18	0,720469	-0,55295	0,109436	0,6279808
CG3408-RA	mt:ND6	94,2683	285,074	53,5286	79,5801	201,296	225,241	-0,197887	0,444977	0,6279808
CG34090-RA	chk	18,7472	23,1651	16,0599	28,2448	4,75655	11,4856	-0,251516	0,443211	0,6279808
CG34092-RA	mt:Cyt-b	3168,94	8240,02	1433,6	9265,96	19506	16076,5	-1,085541	0,000625	0,6279808
CG34098-RA	mt:ND1	238,162	148,704	164,638	294,521	625,433	614,057	NA	NA	0,6279808
CG34099-RA	Acp54A1	0	4,55755	0	5,40323	3,7609	0	0,192712	0,357855	0,6279808
CG34099-RB	Mkp	13,6234	0,0227462	33,3738	0,0269659	8,82056	0,0275278	0,110139	0,593034	0,6279808
CG3409-RA	Mkp	6,75865	4,62198	19,0871	7,88925	7,87397	3,95119	0,123822	0,64592	0,6279808
CG34100-RA	lectin-24A	0,311312	0,177228	1,38231	1367,05	3,23538	0	0,154105	0,515038	0,6279808
CG34100-RB	mld	1,82594	2,32673	7,44416	7,28412	2,49573	13,0074	0,182676	0,439936	0,13772387
CG34102-RA	mld	8,99786	8,23436	0,0166646	0,0180154	3,35978	6,38873	0,01562	0,897983	0,6279808
CG34103-RA	BG642163	0	4,50564	0	5,53734	0	0	NA	NA	0,6279808
CG34104-RA	BG642167	0	0	1976,78	0	0	0	0,073409	0,809187	0,6279808
CG34104-RB	RhoU	1,39451	0,0235226	1,35121	0,0273464	1,14244	1,35142	0,332912	0,276837	0,6279808
CG34105-RA	RhoU	9,54829	15,8725	7,68333	14,6504	3,91565	5,6879	NA	NA	0,6279808
CG34106-RA	CG34105	7,66703	0,139752	10,905	15,5963	62,8167	7,03342	0,058331	0,833917	0,13772387
CG34107-RA	CG34106	3,77162	0,240746	3,20318	0	0	0,279091	-0,367813	0,216999	0,6279808
CG34108-RA	CG34107	4,85852	4,47465	0,253747	6,90734	7,74685	6,31521	0,081988	0,710633	0,6279808
CG34109-RC	CG42795	0,264304	0,641989	3,57609	0	0,774735	0	-0,211775	0,546517	0,6279808
CG3410-RA	CG34109	0,0646788	0,200307	0,0164571	0,230568	2,56669	0,991983	0,392475	0,221946	0,6279808
CG34110-RC	bs	4,02431	4,32764	3,52761	4,92328	0,136778	2,56383	0,133826	0,705343	0,6279808
CG34111-RA	bs	7,42282	7,94617	9,02181	9,19344	3,03915	5,04455	0,092036	0,716822	0,6279808
CG34112-RA	lobo	0,358104	0,326186	0,354891	0,567412	17,6165	6,03526	-0,356571	0,299932	0,6279808
CG34113-RO	CG34111	0	3,35481	25,7001	0,0162554	0,137517	2,52046	0,157937	0,525875	0,6279808
CG34113-RP	CG34112	3,67088	16,6612	0,0508443	58,863	0,0583902	0,0194546	0,251097	0,303324	0,13772387
CG34114-RB	side-III	1,98166	2,36772	0,894985	1,02889	0,0424696	0,773278	0,518423	0,050921	0,6279808
CG34115-RA	side-III	1,00915	1,18301	0,412414	2,27976	2915,15	0,0345386	0,573185	0,055403	0,13772387
CG34116-RA	side-VI	3,62143	4,53948	0,036782	0,0943538	937,377	23,7977	-0,098265	0,732845	0,6279808
CG34117-RA	CG34115	102,418	24,7342	64,1776	29,738	65,1369	30,3577	-0,499826	0,077782	0,6279808
CG34118-RA	CG34116	0,103893	6,3257	0,0488351	0,0556743	3,50136	23,7155	-0,382868	0,266959	0,6279808
CG34119-RA	CG34117	50,414	41,0233	51,0187	81,0681	116,977	126,012	0,024538	0,943045	0,6279808
CG3411-RA	Gfrl	0,243862	5,92275	5,36782	7,17882	5,26371	3,89586	0,306785	0,231569	0,6279808
CG3411-RB	Gfrl	0,131625	0,0227921	0,024023	3,18497	0	148,216	0,178237	0,471543	0,6279808
CG34120-RB	slmb	23,7839	57,3629	42,9951	34,998	1,80714	11,9042	-0,313904	0,348454	0,6279808
CG34120-RC	CG34120	1,14384	12,4356	0,252267	0,0177437	0,024034	4,0923	-0,413893	0,141971	0,6279808
CG34120-RD	CG34120	0,878826	0,0293068	0,0729159	12,3201	6,45903	0,0353184	-0,413893	0,141971	0,6279808
CG34122-RA	CG34120	0,071459	0,0286921	0,513658	0	0,077496	0,0345209	-1,018229	0,00407	0,6279808
CG34123-RB	plx	0,393309	0,0617508	0	19,8751	5,4055	7,58653	-0,677534	0,00248	0,6279808
CG34123-RC	Trpm	0,00952422	6,22603	1,1887	1,59553	6,87269	0,991288	-0,683918	0,002051	0,6279808
CG34123-RD	Trpm	0,00941699	0,0125319	0,00904087	0,00959468	0,0191792	0,00979461	-0,148624	0,491978	0,6279808
CG34123-RE	Trpm	0,00950231	0,0138817	0,00912278	0,00968351	0,0213186	0,0098853	-0,144983	0,502155	0,13772387
CG34123-RF	Trpm	7,53897	0	5,53706	7,29382	4,69492	5,65231	-0,148624	0,491978	0,6279808
CG34123-RG	Trpm	0,0137582	0	0,0132087	0,0141595	0,0217286	0,0144546	-0,152434	0,481132	0,13772387
CG34124-RA	Trpm	0,0152401	0	0,0146314	0,015739	8,52779	0,016067	0,078197	0,826352	0,13772387
CG34125-RA	CG34124	0,090065	1027,24	0,0515885	37,617	308,252	74,4581	0,079618	0,728905	0,6279808
CG34126-RB	CG34125	0,111114	8,2389	21,5141	0,147898	0,200329	142,123	0,307426	0,271043	0,6279808
CG34127-RA	CG34126	5,01871	20,3256	4,04113	9,43932	5,9968	2,69137	-0,510467	0,059202	0,13772387
CG34129-RA	Nlg3	1,0371	12,7051	15,2105	12,6992	22,707	13,3702	-0,066275	0,671132	0,6279808
CG3412-RA	CG34129	0	7,77345	0,0291976	241,617	0,363442	7,10398	0,050069	0,827556	0,6279808
CG34130-RA	wdp	33,8873	0,0203013	0,0213976	30,9082	0,0304037	21,6307	-0,041811	0,731452	0,6279808
CG34131-RA	wdp	0,0219109	13,8516	20,1914	0,0229866	0,0282303	0,0234656	-0,08831	0,686743	0,13772387
CG34132-RA	wdp	0,0222878	0,0258237	10,3117	0,023403	90,2108	0,0238907	-0,558962	0,051816	0,6279808
CG34133-RA	wdp	9,64534	5274,4	0,093061	26,0292	38,1679	14,3643	-0,197588	0,396233	0,6279808
CG34133-RB	CG34130	0	0	0,0164914	0,142928	6,69463	10,1857	-0,194304	0,40462	0,6279808
CG34134-RA	Muted	0,044376	4,14226	45,0633	2,0842	35,752	37,7404	0,143031	0,571622	0,6279808
CG34135-RA	CG34132	112,711	84,65	149,314	186,965	139,935	386,449	0,365612	0,280662	0,6279808
CG34135-RB	CG34133	4,08095	3,19187	3,37263	7,05925	2,97836	3,03388	0,352349	0,32157	0,6279808
CG34136-RA	CG34133	2,12727	3,32503	2,5488	3,22264	2,72267	1,91631	-0,571111	0,073469	0,6279808
CG34137-RA	CG34134	13,8283	15,8222	11,314	20,3485	14,0988	14,9463	0,871593	0,012293	0,6279808
CG34138-RA	CG34135	0,670592	14,0118	1,54514	1,61251	0,820292	1,13059	0,144711	0,571601	0,13772387
CG34139-RA	CG34135	1,09016	24,8744	1,04661	2,0855	1,42186	1,67047	-0,363664	0,122735	0,13772387
CG3413-RA	CG34136	37,8111	83,5812	47,9819	40,8242	19,6907	63,1953	0,030506	0,86727	0,6279808
CG3413-RB	dunk	1,49438	0,993298	1,97755	1,01454	0,686731	0,43723	0,020621	0,909981	0,6279808
CG3413-RC	CG34138	0,0338908	6,53911	0,00604121	4,096	0	0	0,030537	0,867084	0,6279808
CG3413-RD	Nlg4	1,37352	79,6273	1,69542	2,121	1,98487	1,40637	0,020559	0,910232	0,6279808
CG34141-RA	CG34141	14,7355	1,43301	16,6485	0,0131638	2,3192	1,35076	0,240624	0,255256	0,6279808
CG34143-RC	Ir10a	0,165282	1,20744	185,201	1,16765	1,23541	923,743	0,144729	0,68358	0,6279808
CG34144-RA	CG44422	1,03648	1,10538	8,27312	1,44309	1,11923	0,056311	-0,281715	0,430053	0,6279808
CG34145-RA	RunxA	0,350934	0,54798	2,51558	9,24565	11,7567	4,00676	0,230762	0,518713	0,13772387
CG34147-RA	mRpL34	160,972	95,2953	95,0427	1,24785	11,7408	527,726	-0,410345	0,199563	0,13772387
CG34148-RA	CG34148	5,35036	0,0267179	52,8111	0,030583	0,0424292	0,0312203	0,176706	0,551837	0,6279808
CG34149-RA	CG34149	2,93395	2,91819	1,00368	0,051217	2,53234	2,28223	0,485514	0,090486	0,13772387
CG34149-RB	CG34149	0,0647378	0,0589677	2,89008	39,5152	1,63242	1,52996	0,485514	0,090486	0,6279808
CG34150-RA	Mfe2	36,0848	39,3851	55,1917	42,2662	45,8552	45,3753	-0,221794	0,419205	0,6279808
CG34155-RA	CG34150	9,30526	11,042	7,81018	0,0146572	23,9741	0,0151548	0,423619	0,062267	0,6279808
CG34157-RA	CG34155	4,07455	0,0223437	12,1144	0,0220188	58,9529	1,3906	0,086132	0,767651	0,6279808
CG34157-RB	Dys	0,00572846	0,00521788	0,34962	0,00610248	0,00826585	0,0135874	0,190545	0,515771	0,6279808
CG34157-RC	Dys	0,00603616	0,00549816	0,346775	0,00636541	1,05753	2,56466	0,333662	0,251721	0,6279808

gene_id	Symbol	W1_FPKM	W2_FPKM	W3_FPKM	MCM51_FPKM	MCM52_FPKM	MCM53_FPKM	FC	p-value	p-adj
CG34157-RD	Dys	0,00629254	0,00573168	16,5039	5,99563	1,63988	11,1992	0,419	0,148714	0,6279808
CG34157-RE	Dys	3,44975	4,72604	0,0879032	0,0058966	0,00798698	0,102379	0,463134	0,110136	0,6279808
CG34157-RF	Dys	0,0058352	0,0053151	5,09321	0,01331	0,0180285	0,179595	0,3224	0,268493	0,6279808
CG34157-RG	Dys	0,0129569	0,011802	17,5983	6,78877	2,5699	4,02335	0,322527	0,268261	0,6279808
CG34157-RH	Dys	0,0100931	0,00919345	0,0193779	22,8225	9,34817	0,021528	0,313654	0,282501	0,6279808
CG34159-RA	Dys	27,1646	40,013	9,1358	0,161546	0	7,533	-0,42656	0,076904	0,6279808
CG3415-RA	CG34159	57,2585	46,7228	66,6683	14,3368	9,07137	6,09884	-0,200054	0,390977	0,6279808
CG34160-RA	Rpn8	24,5235	21,4288	0	3,84446	19,8713	2,59319	0,044335	0,715924	0,6279808
CG34161-RA	CG34160	0,222728	6,46344	28,6287	0,189518	0	0	0,099631	0,472914	0,6279808
CG34162-RA	CG34161	0	26,0944	27,7628	0,0950759	0	0	0,025944	0,860432	0,6279808
CG34163-RA	CG34162	0,0750862	15,8525	12,5593	20,4988	27,0682	17,0162	-0,460635	0,170063	0,6279808
CG34164-RB	CG34163	44,669	0,00880091	1,87371	24,937	2,96671	0,0225077	-0,340245	0,126682	0,6279808
CG34165-RA	CG34164	0,0390135	12,9458	0,0309498	13,3653	6,99388	3,99081	-0,860596	0,006597	0,13772387
CG34166-RA	CG34165	71,362	0	68,9346	0	0	0	-1,201907	0,000225	0,6279808
CG34167-RA	CG34166	92,4939	3,68093	0	0	0	0,191764	-0,266598	0,400389	0,6279808
CG34168-RA	CG34167	0,249909	0,500795	0,143956	0	0,921872	0,354453	0,05799	0,71375	0,6279808
CG34169-RA	CG34168	0	0,194568	0,341793	0,0165494	0,0224162	0,0168942	0,06906	0,58405	0,6279808
CG3416-RA	CG34169	0	0,0189943	0,168715	0,0218219	0	0,0980853	-0,009182	0,9676	0,13772387
CG34170-RA	CG34170	0	0	0	0	0	0	NA	NA	0,6279808
CG34171-RA	CG34171	0,115517	0,210442	0,110903	0	0	0	0,188401	0,387173	0,6279808
CG34172-RA	CG34172	6,98453	3,73641	3,406	2,10819	4,2465	8,95067	0,4786	0,180908	0,6279808
CG34173-RA	CG34173	0	0	0	0	0	0	NA	NA	0,6279808
CG34174-RA	CG34174	4,29015	3,883	8,61497	14,3578	0,107593	5,20927	0,101681	0,747795	0,6279808
CG34175-RA	CG34175	0	1,45703	0,177198	6,61595	0,291338	5,49572	-0,190121	0,33449	0,13772387
CG34176-RA	CG34176	26,4802	22,6077	73,1595	47,8659	4,18688	85,8064	-0,268723	0,43395	0,13772387
CG34178-RA	CG34178	0,0742702	0	0,209592	57,6463	0	49,6269	-0,001151	0,995701	0,6279808
CG34178-RB	CG34178	0,218312	0	0,0762765	0,10936	2,53933	0,111639	-0,001151	0,995701	0,6279808
CG34178-RC	CG34178	0,0794498	1314,62	40,3038	56,8081	47,5701	2,90594	-0,001151	0,995701	0,13772387
CG34179-RA	CG34179	0,0330434	0,0414032	0,0436391	0,158576	10,4981	0,0515628	-0,148939	0,633283	0,6279808
CG34179-RB	CG34179	0,117017	74,3707	71,9294	0,0409896	0,0594402	88,7108	-0,147075	0,526058	0,6279808
CG34180-RB	CG34180	0,203518	3,20832	6,59237	7,23876	3,61728	4,81434	-0,056884	0,835639	0,13772387
CG34181-RA	CG34181	0	0	0	0	0	0	0,01562	0,897983	0,6279808
CG34182-RA	RpS28-like	0,429064	69,6475	1,71915	4,52815	2,84652	1,39112	0,267152	0,430929	0,6279808
CG34183-RA	CG34183	0,133576	0,12167	74,518	3,16428	0,258308	0,194676	-0,120397	0,653105	0,6279808
CG34184-RA	CG34184	0	0,133748	25,1321	0,209171	1,74279	0	0,044471	0,836291	0,6279808
CG34185-RA	CG34185	1,99442	1,5785	0	0,0339207	0,0459458	2,82545	0,06276	0,856189	0,13772387
CG34186-RA	Rpb12	121,106	95,3557	91,5248	0,570222	332,579	0,319549	-0,535936	0,039746	0,6279808
CG34187-RA	Trpm	0,388682	2,61015	21,5476	0	18,4295	0	-0,225358	0,495737	0,6279808
CG34188-RA	CG34188	0,149268	0,371522	2,83573	0,0618693	0,0185186	2,08043	0,024053	0,869957	0,6279808
CG34189-RA	CG34189	0,71627	12,2601	0,0179217	0,180268	0,0272146	0,0205733	0,144318	0,669286	0,6279808
CG34190-RA	CG45069	0,0263076	0,0239627	0	0	23,4999	0	0,161547	0,646662	0,6279808
CG34191-RA	CG45069	9,14961	7,39747	0	0	0	0	-0,354694	0,176457	0,6279808
CG34192-RA	CG34190	10,585	0,0107752	155,228	19,8063	1,90341	0,290569	0,35112	0,300508	0,6279808
CG34193-RA	Ufm1	147,044	17,419	24,3609	7642,93	2,85651	15,8482	0,096567	0,482921	0,6279808
CG34194-RA	robls54B	3,21608	3,99468	12,0457	6,63583	0,0237652	1,61838	-0,069847	0,797624	0,6279808
CG34195-RA	CG34193	0	29,5547	0,262497	9,62109	0	0	-0,493907	0,061534	0,6279808
CG34196-RA	CG34194	0,126582	0,1153	0	0	64,4389	9,02395	0,250435	0,442788	0,6279808
CG34197-RA	CG34195	5,17632	0,0381168	5,06507	4,08529	3,13631	23,4788	-0,559323	0,041389	0,6279808
CG34198-RA	CG34196	0,68829	3,41731	2,51104	58,1823	2,52289	1,90141	-0,517638	0,136056	0,6279808
CG34199-RA	hts	1,29505	1,01602	1,12535	2,56764	1,38866	1,1488	0,01694	0,907471	0,6279808
CG3419-RA	CG34198	16,1519	2,1679	2,51973	9,81932	4,23113	3,75917	-0,116397	0,696841	0,6279808
CG3419-RB	CG34199	0	0	0,0492648	0,33693	0	2,96233	-0,111079	0,704769	0,6279808
CG34200-RA	CG3420	41,1242	30,0512	48,3983	46,9262	96,4407	67,0349	-0,394096	0,159063	0,6279808
CG34201-RA	CG34200	209,962	10,2363	11,6528	8,47766	1,16412	738,703	-0,272868	0,433128	0,6279808
CG34202-RA	CG34201	0,357167	90,4373	0,0725821	4,26125	0	0,351034	-0,747003	0,024542	0,6279808
CG34203-RA	CG34202	0,246552	0,542221	0,428627	65,9314	14,0817	0	-0,342051	0,306735	0,6279808
CG34204-RA	CG34203	1,17331	5,21584	3,03166	8,83818	1,70687	4,58691	0,01562	0,897983	0,6279808
CG34205-RA	CG34204	0,393309	1,36259	16,5423	0,0245085	169,981	0,0308339	-0,189783	0,487158	0,6279808
CG34206-RA	CG34205	0	3,45097	7,3343	0,795259	0,2965	0	-0,064052	0,856632	0,6279808
CG34207-RA	CG34206	0,598513	1,51178	3,91253	21,13	2,89726	0	0,842568	0,003444	0,6279808
CG34208-RA	CG34207	0,261512	136,895	17,6734	0,617299	7,03659	28,3724	0,306647	0,391571	0,13772387
CG34209-RA	CG34208	0,173884	13,2778	0	3,32641	1,91122	6,30917	-0,147431	0,666412	0,6279808
CG3420-RA	CG42741	0,491636	0,0413369	2,28949	0,0347618	0,0712	13,2447	-0,54386	0,077643	0,6279808
CG34210-RA	RhoGAP93B	10,9116	15,6668	9,70396	8,28771	2,705	4,76589	-0,070526	0,562659	0,6279808
CG34211-RA	CG34210	0	0	0	2,61964	6,30511	4,50095	0,354941	0,248253	0,6279808
CG34212-RA	CG34211	2,37528	0,0358537	0	8,65679	0	0	0,09339	0,599834	0,13772387
CG34213-RA	CG34212	0,674244	0	14,1687	1,28252	7,04569	0,0439776	-0,082097	0,796014	0,6279808
CG34214-RA	CG34213	3,99434	41,1233	71,6022	18,417	7,7953	30,1815	-0,26432	0,435023	0,6279808
CG34215-RA	CG34214	0,517228	0	0,372427	0,854498	903,242	934,737	-0,302867	0,184891	0,6279808
CG34216-RA	CG34215	28,6179	0	0,1888	0,404657	0,13105	0,279129	-0,426744	0,188269	0,6279808
CG34217-RA	CG34216	0,207786	35,0657	0,518666	0,268694	0,363947	0,00891925	-0,24845	0,450336	0,6279808
CG34218-RA	CG34217	0,628098	0,286057	0,603011	0,305506	17,1079	1,55936	-0,053683	0,691569	0,6279808
CG34219-RA	whip	0	0,0274866	0,1095	0	0	0	0,476665	0,05928	0,6279808
CG3421-RA	CG34219	0,0552474	0,0503232	4,6904	18,2891	20,9139	26,5504	0,541696	0,06977	0,6279808
CG34220-RA	Prosalpha4	20,2164	16,6729	90,5467	0,0299301	49,8229	46,8181	-0,520542	0,136053	0,6279808
CG34221-RB	CG34220	0,295113	0,591381	0,651651	0,0345069	0,0467398	5,19315	0,528226	0,109657	0,6279808
CG34222-RA	CG34221	2,74941	3,07912	3,93775	29,9396	1,03391	2,67118	0,225188	0,392878	0,6279808
CG34223-RA	CG34222	0,124514	0	0,119541	0	0,233934	0	-0,221495	0,484697	0,6279808
CG34224-RA	CG34223	0,231845	10,3361	0,0229455	0,0217499	0	0,0257175	-0,592152	0,042598	0,6279808
CG34225-RA	CG34224	5,49411	4,64885	0,00641553	0,990122	3,8098	0,577655	0,051692	0,753555	0,6279808
CG34226-RA	CG34225	0,314647	51,7935	2,86573	0,0323074	4,90386	0,877348	-0,48124	0,17876	0,6279808
CG34227-RA	CR30029	9,70259	0,0227319	0,222585	0,0356264	596,272	12,4739	0,561125	0,074424	0,6279808
CG34228-RA	CG34227	0,678397	12,4459	0,442799	0,0272109	1,01227	1,0576	0,016239	0,953859	0,6279808
CG34229-RA	CG34228	24,6739	19,732	3,66576	33,1864	0,0327703	1,92629	-0,432987	0,149206	0,6279808
CG3422-RA	CG34229	42,1143	40,2649	7,33263	6,21139	3,93822	1,05764	-1,009853	0,000202	0,6279808
CG34230-RA	SA	6,52725	5,83737	6,74859	9,23256	0,054637	5,29673	0,214999	0,486031	0,6279808
CG34231-RB	CG34230	0	0,296763	1,33645	2,25572	190,101	2,18144	0,238186	0,488062	0,6279808
CG34232-RA	CG34231	0,14882	3,88557	0,142876	5,63571	1,39106	6,95992	-0,19544	0,539544	0,6279808
CG34232-RB	CG34232	43,4412	24,5867	13,6647	13,6775	1,77541	2,23788	-0,219999	0,494008	0,6279808
CG34233-RA	CG34232	0,113402	0,103295	0,016045	8,38972	7,19711	5,14005	-0,005957	0,982394	0,6279808
CG34234-RA	CG42700	0,156578	1,34596	0,150324	1,7907	0	0	-1,041063	0,001131	0,6279808
CG34235-RA	CG34234	0,18825	6,83717	4,13994	5,43923	5,72916	0	-0,188734	0,566753	0,6279808
CG34236-RA	CG34235	0,322007	9,01886	0,958816	13,3109	6,21285	0,700124	-0,105972	0,747266	0,6279808
CG34237-RA	CG34236	0,426296	2,71623	0	8,5597	14,6633	87,5204	-0,03823	0,82214	0,6279808
CG34238-RA	CG42521	0	0	0	0	3,08023	5644,97	NA	NA	0,6279808
CG34239-RA	CG34238	0	0	0,110517	2,32722	0,0378739	0,121962	-0,047945	0,781305	0,6279808
CG3423-RA	CG43693	0	51,5441	0	17,0865	0,0467888	2,62015	0,034984	0,888945	0,6279808
CG34240-RA	path	5,68111	13,0761	39,9971	0,0269548	27,9975	26,2689	-0,184512	0,439188	0,6279808
CG34241-RA	path	35,0671	25,8914	2451,23	44,9156	2957,28	3387,22	0,330263	0,191006	0,6279808
CG34242-RA	CG42671	15,8549	18,4869	12,8426	0,644498	2,16437	8,79151	-0,371128	0,194964	0,13772387
CG34242-RB	CG34241	0,695536	0,633542	0,500817	0	0,717583	0,567656	-0,372205	0,193418	0,13772387
CG34243-RA	CG34242	26,3465	29,0888	25,6227	60,7582	6,6541	27,6987	0,39896	0,258623	0,13772387
CG34244-RA	CG34242	9,72112	0,192493	0,202888	0,401931	4448,51	60,0382	0,139293	0,510772	0,6279808
CG34245-RB	bru3	2,20253	2,00622	1,65487	3,77942	17,2775	11,0418	-0,070526	0,562659	0,6279808

gene_id	Symbol	W1_FPKM	W2_FPKM	W3_FPKM	MCM51_FPKM	MCM52_FPKM	MCM53_FPKM	FC	p-value	p-adj
CG34246-RA	CG34244	0,345585	0,752331	0,675484	0,901061	0,485902	0,675822	-0,120979	0,673609	0,6279808
CG34247-RA	CG34245	0	0	0	0,11469	0,155348	0,267972	0,078037	0,549183	0,6279808
CG34248-RA	Hsc20	3,43198	3,08842	5,87525	0,315528	8,60134	4,83162	-0,392494	0,20997	0,6279808
CG34249-RA	CG34247	0,655515	0	0	32,2531	0	0	-0,565056	0,095862	0,13772387
CG3424-RA	CG34248	18,5598	27,0769	29,2785	229,574	302,455	54,2926	0,144237	0,456	0,13772387
CG3424-RC	ringer	0	0,0689683	67,8307	5,09299	0,119716	34,5153	0,16145	0,404818	0,13772387
CG34250-RA	T3dh	4,94549	3,90098	0,056962	6,07718	13,5424	11,9427	-0,624	0,044908	0,6279808
CG34251-RA	CG34250	135,795	107,248	0,228519	0,479922	760,713	129,409	-0,366758	0,305302	0,6279808
CG34252-RA	CG34251	2,32662	6,56781	6,45605	10,28	9,62245	12,2811	0,737602	0,028756	0,13772387
CG34253-RA	CG44004	0,665957	0,680575	1,45025	0,651832	0,365478	0,348063	0,28053	0,248432	0,6279808
CG34254-RA	CG34253	14,2024	13,962	14,0509	13,9959	9,47045	13,8392	-0,037197	0,893781	0,13772387
CG34255-RA	CG34254	0	0,473164	3,79765	1,35163	10,3985	0,856592	-0,195768	0,466589	0,6279808
CG34256-RA	Blos3	23,1389	48,2432	0,0249195	0,0274947	37,961	114,479	-0,429976	0,225348	0,6279808
CG34257-RB	CG34256	0,543834	21,0124	0,0209801	0,0229067	0,0310273	0,0233841	0,542068	0,09124	0,6279808
CG34258-RA	Ir76a	0,315144	0,430583	30,3708	0,0357229	11,7257	27,101	0,01562	0,897983	0,6279808
CG34259-RB	CG34258	0,194341	0	0	0	0	0	-0,164413	0,421446	0,6279808
CG3425-RA	CG34259	0	0	0	0	0,331091	0,189454	-0,644711	0,045036	0,6279808
CG34260-RA	CG34260	0,0683542	24,0122	0,0656241	28,1871	22,622	58,3622	-0,019922	0,9314	0,6279808
CG34261-RA	CG34261	0,0739104	0,0307283	0,0354263	0,046814	113,984	0,0972707	0,253053	0,399264	0,6279808
CG34261-RB	CG34261	0,147821	18,1944	34,7693	0,0952852	0,0634549	0,275088	0,219664	0,502426	0,13772387
CG34263-RA	CG34263	0,322843	0,294068	0,309949	0,995457	1,34835	1,0162	0,132918	0,671384	0,6279808
CG34264-RB	ddbt	0,323477	0,017247	0,496892	0,0222193	1,94843	0,020136	0,127107	0,664118	0,6279808
CG34264-RC	ddbt	0,123814	0,019846	0,118869	0,0215648	5,81521	0,0233273	0,107216	0,713833	0,6279808
CG34265-RA	CG34265	11,5178	16,3494	0,0436691	0,050549	11,7905	21,9132	-0,442638	0,172761	0,6279808
CG34266-RA	CG34266	0	0	3,55341	43,4914	0	0	NA	NA	0,6279808
CG34267-RA	CG34267	218,852	136,768	242,899	45,0811	3,03072	29,6599	-1,116531	0,000905	0,6279808
CG34268-RA	CG34268	125,353	1,66425	0	10,0646	9,19522	5,75236	-1,15033	0,000554	0,13772387
CG34269-RA	CG34269	0,453264	14,4481	17,3168	0,00992416	3,60118	0,010131	0,332926	0,195061	0,6279808
CG34270-RA	Cpr65Ax1	562,758	279,727	1194,21	1646	649,344	538,351	-0,929412	0,008537	0,6279808
CG34271-RA	Cpr65Ay	11,2374	2,1154	18,7721	27,4064	0	16,438	-0,775309	0,029755	0,6279808
CG34272-RA	CG42673	4,88297	0	0,11534	0	0	0	-0,243775	0,459581	0,6279808
CG34273-RA	CG34273	1,69237	18,0273	6,07811	43,7536	0,195502	0,147342	-0,034893	0,92062	0,6279808
CG34274-RA	CG34274	0	0	22,0946	42,8991	47,5782	34,5596	-0,074864	0,550506	0,6279808
CG34275-RA	CG45105	1,23152	1,33748	45,5999	71,3821	10,4547	13,1175	-0,234674	0,431879	0,13772387
CG34276-RA	CG34276	91,4957	0,0551326	0,0189779	0,0206329	5,77981	6,75376	0,123554	0,695213	0,6279808
CG34277-RA	CG34277	0,187786	0,171049	0,0546083	9,07097	110,747	0,0801227	-0,225121	0,33381	0,6279808
CG34278-RA	CG34278	0	0,106087	0,919374	0,553685	0,0405958	1,1833	-0,170654	0,540002	0,6279808
CG34279-RA	CG34279	0,113793	0,0132833	0,0140006	0,0194284	4,82269	1,28444	-0,291896	0,368742	0,6279808
CG34279-RB	CG34279	0,209986	0,10365	0,109248	143,296	12,8065	0,155878	-0,282164	0,328333	0,6279808
CG34280-RA	pall	11,6398	16,383	15,7515	9,27701	6,83004	8,18048	-0,041811	0,731452	0,13772387
CG34281-RA	CG34280	0,173884	0,191269	0,172383	0,0199822	0,0206192	0,405444	-1,136135	0,001	0,6279808
CG34282-RA	CG34281	16,5704	7,03113	65,2585	64,5078	23,8299	70,7686	-0,262557	0,457711	0,6279808
CG34283-RA	CG34282	11,7567	3,18555	66,1869	129,25	0,824539	13,0374	-0,048535	0,885284	0,6279808
CG34284-RA	CG34283	0,429064	0,521095	0,686545	0,613464	171,996	179,906	-0,000777	0,99798	0,6279808
CG34286-RA	CG34284	76,648	84,6623	111,733	94,3263	0,017152	0,0129268	0,065796	0,591492	0,6279808
CG34287-RA	CG34286	0	0	818,456	0	0	0	NA	NA	0,13772387
CG34288-RA	CG34287	0	3,47917	3,64109	3,99512	0,408756	0	-0,729473	0,024954	0,6279808
CG34289-RA	CG34288	41,2086	29,9315	0,041854	90,1695	2,97579	0,0492233	0,01562	0,897983	0,6279808
CG34289-RB	CG34289	0	0,10449	0	0	0	0	0,01562	0,897983	0,6279808
CG3428-RA	CG34289	0	0,459202	0	0	0	0	0,082097	0,74126	0,6279808
CG34290-RB	swaPsi	0,653247	0,490893	1,86579	1,34981	1,34973	1,66751	-0,061594	0,858446	0,6279808
CG34291-RA	CG34290	18,5368	8,52792	15,3779	13,7155	0,0124957	0,0164924	0,960903	0,001656	0,6279808
CG34292-RA	CG34291	659,67	0,306449	806,177	0,229172	0,574573	0,233947	-0,070526	0,562659	0,6279808
CG34293-RA	CG34292	0	0	0,782509	10,832	3,47888	0	-0,334338	0,28925	0,6279808
CG34294-RA	CG34293	10,0964	31,1539	24,4332	32,6009	0,08392	0,0632472	0,686258	0,005338	0,6279808
CG34295-RA	tau	18,6405	21,1082	15,9462	12,8388	11,4001	11,9321	-0,045338	0,793699	0,6279808
CG34296-RA	CG34295	0,114318	0,00646147	3,63391	35,121	1,44332	27,8818	0,661005	0,064087	0,6279808
CG34297-RA	CG34296	1,93031	229,298	0,712773	205,353	193,427	709,124	-0,141424	0,669388	0,6279808
CG34298-RA	CG34297	1,8825	2,18626	1,3103	62,2281	76,4318	2,59776	0,084284	0,70833	0,6279808
CG34299-RA	CG34298	0,197438	0,0268011	0,100587	2640,06	0,185352	0,0149859	NA	NA	0,6279808
CG3429-RA	CG34299	0	7,05483	0	8,70186	0	3,48934	-0,459419	0,184974	0,6279808
CG34300-RA	CG3430	1,89214	6,38362	7,6863	0,0313245	0,997973	0,0411777	-0,013096	0,914398	0,6279808
CG34301-RA	CG34300	0	0	0	0	0	0	0,255047	0,436873	0,6279808
CG34302-RA	CG34301	61,4484	46,1691	56,2046	9,30435	11,0905	110,862	-0,060279	0,67377	0,6279808
CG34303-RA	CG34302	0	0,105282	3701,09	5946,95	0,474448	2,37327	-0,045962	0,77089	0,6279808
CG34304-RA	CG34303	0,235985	0	0	1,87779	0,0369948	4,87981	0,057152	0,785228	0,6279808
CG34305-RA	CG34304	0	0,0977053	0,205964	0	0,185547	0,139839	-0,373776	0,263897	0,6279808
CG34306-RA	CG34305	163,67	0,243997	191,98	0,0547491	1,77907	0,0555668	-0,044775	0,88086	0,6279808
CG34307-RA	Cdep	1,34354	27,8011	1,12282	0,028432	0,533346	3,09951	NA	NA	0,6279808
CG34308-RA	CG34307	0	0	0	0	0,0717726	0,0540921	-0,041811	0,731452	0,6279808
CG34309-RA	CG34308	0	2,80081	2,77494	3,85578	0	2,43397	0,155203	0,615355	0,13772387
CG3430-RA	CG34409	11,5951	7,9774	5,80285	5,67747	32,3755	12,4159	-0,092069	0,73713	0,6279808
CG34310-RA	Uch-L5	11,2093	7,5489	23,3033	15,819	0,0245361	16,329	-0,218747	0,394533	0,6279808
CG34310-RB	CG34310	68,9692	0,626411	50,8399	0,232405	0,0595195	1,64718	-0,21904	0,394079	0,6279808
CG34313-RA	CG34310	0,135959	0,880376	0,130528	0,0440005	3,91613	0,738401	0,204555	0,349905	0,6279808
CG34314-RA	CG34313	12,0339	10,9479	14,537	46,5987	9,66854	43,4832	-0,117217	0,699	0,6279808
CG34315-RB	Sws1	1,43078	3,3365	1,45688	5,43297	1,87929	1,24043	0,078217	0,757962	0,6279808
CG34316-RA	CG34315	0,887737	0,0181905	13,8306	0,0201656	0,431134	7,39788	0,255295	0,475664	0,6279808
CG34316-RB	CG34316	0,0403475	0,0367513	5,44241	0,0442665	0,0599593	0,045189	0,007251	0,981646	0,6279808
CG34317-RA	CG34316	4,86494	0,0703385	0,0741371	0,0932622	4,51612	0,0952056	-0,070143	0,796539	0,6279808
CG34318-RA	CG34317	22,944	5,22339	6,45293	6,53638	5,84537	31,2807	-0,054432	0,819879	0,6279808
CG34318-RB	Stacl	0,793978	74,0621	0,244741	65,1445	3,70846	0,487975	-0,049553	0,832972	0,6279808
CG34319-RA	Stacl	1,94395	0,0223437	52,5383	0,0258955	24,754	32,9899	-0,083339	0,8043	0,13772387
CG3431-RA	Hk	1,15371	0,955341	0,0126603	9,12936	0,0266617	5,06811	-0,160084	0,602347	0,13772387
CG34320-RA	CR34320	0,855351	3,79668	0,334707	0,350856	0,0195232	1,98582	-0,202296	0,532599	0,13772387
CG34321-RA	inaF-C	0,0429994	0,0311345	0,021412	2,77327	0,0317221	4,53808	0,386944	0,252041	0,6279808
CG34322-RA	inaF-A	0,0541309	88,2485	0,0218773	7,39053	0,0324486	1,58481	0,345859	0,308529	0,6279808
CG34323-RA	CG34323	0,682199	0,778118	0,935646	0,00800966	0,010861	18,7986	-0,32203	0,332987	0,6279808
CG34324-RA	CG34324	0,268601	0,183495	0,193405	0	0,0387285	0,474057	0,305424	0,319112	0,6279808
CG34325-RA	CG34325	19,8561	14,6714	0,0219226	15,3248	20,1662	0,0245087	0,171371	0,549273	0,6279808
CG34326-RA	CG45002	0	0,356837	0	9,49499	4,43442	6,77194	-0,135508	0,451044	0,6279808
CG34327-RA	CG34327	0,103297	0,0940901	0,0991713	99,6894	138,501	123,127	0,320289	0,279154	0,6279808
CG34328-RA	CG34328	0,41092	0,021406	8,33052	0,201472	0,0364211	0,0288007	0,157137	0,619414	0,13772387
CG34329-RA	Diedel3	0,327108	0	0,314044	0	0,0954021	0,0693793	0,090472	0,609827	0,6279808
CG34330-RA	Coprox	25,3735	1,44147	2,26245	108,435	0,0487836	4,44055	-0,345871	0,332503	0,6279808
CG34331-RA	CG34330	0,266435	1,65027	6,92189	0	0,0920565	1856,22	-0,1409	0,692596	0,13772387
CG34332-RA	CG34331	18,5053	6,22935	1,41885	0,0353397	0,062434	4,69212	-0,060398	0,703481	0,6279808
CG34333-RA	CG34332	0,397091	0,0351214	0	0	0	0	-0,142915	0,678685	0,6279808
CG34334-RA	Vml	0,209101	0,138519	0,146	0,233846	0,232552	0,141583	-0,226842	0,426672	0,6279808
CG34335-RA	CG34334	0	0,881251	0,111684	0,132596	6,30188	5,83303	-0,408706	0,175091	0,6279808
CG34336-RA	lncRNA:CR34335	5323,85	7,78331	6,2555	0,0688367	3,64336	23,8601	-0,166395	0,305573	0,6279808
CG34337-RA	yin	0	0	0	14500,6	0,0375868	0,0283277	-1,05414	0,001327	0,6279808
CG34338-RA	CG34337	6,72995	6,13011	5,58009	0,0248131	17,8642	0,0253301	0,044335	0,715924	0,6279808
CG34339-RA	CG34338	0	6,45249	0,18264	343,041	0	0	0,734382	0,02943	0,6279808

gene_id	Symbol	W1_FPKM	W2_FPKM	W3_FPKM	MCM51_FPKM	MCM52_FPKM	MCM53_FPKM	FC	p-value	p-adj
CG34339-RB	mgl	4,13639	0,0137517	4,65395	683,099	527,676	495,558	0,722479	0,032665	0,6279808
CG3433-RA	mgl	0,0150973	5,77832	0,0144943	10,9719	465,704	524,155	0,249568	0,459015	0,6279808
CG34340-RD	CG3434	11,0662	3,98121	3,37342	0,0491905	8,14148	9,73468	0,293108	0,338718	0,13772387
CG34341-RB	Drgx	1,70168	14,4982	2,08559	2,68175	1,15888	0,999608	0,041158	0,866064	0,13772387
CG34341-RC	Pde11	0,970022	4,91793	4,35965	6,91581	3,06968	3,87934	0,042477	0,861464	0,13772387
CG34342-RB	Pde11	4,3375	1,12062	0,164914	2,13899	0,969354	0,683681	-0,396286	0,185022	0,6279808
CG34343-RB	CG34342	0,933803	0,850572	1,02202	10,7677	231,992	235,051	0,182509	0,528646	0,6279808
CG34343-RC	sick	1,29151	1,848	8,91168	15,692	0,0513371	16,8826	-0,184716	0,558941	0,6279808
CG34343-RD	sick	0,0172132	0,015679	3,12917	0,0896163	7,99337	21,1704	0,185641	0,522138	0,6279808
CG34344-RA	sick	10,9069	14,5164	0,0258644	0,037901	6,60421	0,0271391	0,062412	0,767907	0,6279808
CG34344-RB	rdgA	1,40308	68,1361	0,240291	2,66065	1,58687	1,49212	-0,27709	0,243625	0,6279808
CG34344-RC	rdgA	2,73204	0,0259462	11,4978	3,1135	1,82841	1,83946	-0,386403	0,112378	0,6279808
CG34345-RA	rdgA	0,021012	0,0248841	1,84484	0,0219967	0,0297947	0,0224551	0,563216	0,057137	0,13772387
CG34346-RC	CG34345	0,0385357	0,0351009	0,0369965	0,0420893	2,17841	2,77157	0,201886	0,268669	0,6279808
CG34346-RD	mamo	0	0,0190123	0	0	0	0,0222988	0,08712	0,708887	0,6279808
CG34347-RB	mamo	0	2,46217	0,020039	0	44,0724	7,50486	0,039042	0,903217	0,6279808
CG34347-RC	CG34347	1,95545	2,80263	1428,15	0,0228353	3,5574	0,0233111	0,060276	0,851789	0,6279808
CG34348-RA	CG34347	2,90419	5,42117	0,0363812	30,8728	24,7032	25,9301	0,036568	0,861849	0,6279808
CG34349-RA	CG34348	5,23212	4,80505	5,69426	9,0562	0,0346266	6,98175	-0,200162	0,414226	0,6279808
CG34349-RB	stac	6,95914	26,9043	14,1343	8,54748	0,105933	4,02013	-0,198202	0,425419	0,6279808
CG3434-RA	stac	4,83653	23,6345	37,4203	2,88816	50,0953	0,0798375	-0,025415	0,914776	0,13772387
CG34350-RA	Np	7,00527	433,349	62,3923	0,0516992	39,2363	4,17555	0,113599	0,744989	0,6279808
CG34351-RC	CG34351	4,9772	1,55192	4,21798	1,8892	35,4703	24,9302	0,258065	0,278211	0,6279808
CG34352-RA	mgl	4,23528	6,17376	3,18065	661,469	0,0677928	0,0510928	-0,071801	0,835665	0,13772387
CG34353-RA	CG34353	3,04819	0,0194673	0,0205186	5,06136	3,31926	3,32588	-0,020754	0,943262	0,6279808
CG34354-RA	CG34354	2,39817	0	0,309826	1512,87	2,28708	0	0,205419	0,483224	0,6279808
CG34355-RB	CG34355	0,0760657	0,0692859	0,0730276	0,0298623	0,124042	0,0934854	0,248625	0,423855	0,6279808
CG34355-RC	CG34355	10,8286	17,8748	8,79111	14,3117	6,63503	6,0732	0,00161	0,995758	0,6279808
CG34356-RC	bma	1,06093	1,46465	1,25728	0,0624258	11,4742	8,40639	0,135729	0,625982	0,13772387
CG34357-RA	CG34357	0,882612	1,21649	1,1038	0,298533	41,4433	0	-0,403303	0,141459	0,6279808
CG34357-RB	CG34357	0,0295553	0,026921	0,0283749	0	10,5455	0,0682881	-0,864927	0,00839	0,6279808
CG34358-RA	shakB	0,0177496	0	0,351126	0	0,0354659	0,0267292	0,313983	0,164124	0,6279808
CG34358-RC	shakB	2,43991	0,0161676	0,0170407	0,0184389	0,77668	0,79086	0,379502	0,090242	0,6279808
CG34358-RD	shakB	5,29697	2,68934	2,36215	3,65671	0	0,0780622	0,373845	0,091476	0,13772387
CG34358-RE	shakB	0,0247877	7,07237	5,21416	4,65373	0	0	0,311451	0,168815	0,13772387
CG34358-RF	shakB	1,08843	0,0225784	0,0237977	0,0261836	0,239086	0	0,381124	0,088766	0,6279808
CG34359-RA	IP3K2	13,7523	49,596	13,8032	87,1243	4,65637	9,81809	0,274293	0,199639	0,13772387
CG34359-RB	IP3K2	6,79101	0,0627159	6,60383	0,0812955	5,58463	3,82196	0,268426	0,210137	0,13772387
CG34359-RC	IP3K2	0,0239174	16,449	0,0229621	11,4216	1,54825	2,58993	0,371075	0,101532	0,6279808
CG34360-RA	CG3436	0,140686	0,128147	0,0275732	0,0306256	11,1559	9,61203	0,087037	0,771588	0,6279808
CG34360-RB	CG3436	19,982	14,869	0,0262063	0,0290077	28,4991	0,0437204	-0,042429	0,889853	0,6279808
CG34360-RC	Glut4EF	0,0195742	0	0,431543	1,2873	2,59007	0,76998	0,257341	0,374953	0,13772387
CG34360-RD	Glut4EF	0,0139293	0,307074	0,0187924	0,185103	0,0373978	0,233915	0,265764	0,357003	0,6279808
CG34360-RE	Glut4EF	41,7918	0,436134	0,0133729	0,962122	0,0264641	12,9723	0,093658	0,750283	0,6279808
CG34361-RC	Glut4EF	0,0260586	0,0126878	0,30208	6,43577	2,5006	0,0208474	-0,101985	0,679205	0,13772387
CG34361-RE	Glut4EF	0,0187627	68,4737	0,0250178	5,86341	0,62697	0,0146402	-0,166647	0,459301	0,6279808
CG34361-RF	Dgk	2,78435	0,0537538	0,0566568	2,86655	2,42682	0,0204606	-0,159928	0,485644	0,6279808
CG34361-RG	Dgk	0,0197438	19,1681	14,2682	9,00509	3,72621	0,0185174	-0,150096	0,533564	0,13772387
CG34362-RA	Dgk	0,0171418	3,19708	2,92846	3,16239	0,094895	0,020136	0,609145	0,012826	0,13772387
CG34362-RB	Dgk	1,95196	1,29217	3,49567	1,26658	0	0,0097196	0,616757	0,017291	0,6279808
CG34363-RD	trv	0,0247413	0,0225361	15,5186	13,6486	0,0353956	5,59196	-0,419002	0,093951	0,13772387
CG34363-RE	trv	12,8506	15,1407	100,405	91,532	7,21331	1,284	-0,431014	0,086444	0,6279808
CG34364-RC	SLO2	0,274427	0	0,356777	0	0,561217	0	-0,088055	0,798726	0,13772387
CG34365-RC	SLO2	0,292915	0	0,286424	0	0,234137	0	-0,230627	0,373677	0,6279808
CG34365-RD	clos	0,0149707	0,0136364	0,0143728	0,0818001	2,27108	0,0835046	0,133798	0,598066	0,6279808
CG34365-RE	Stacl	0,981865	0	5,18721	1,70758	0,0737292	3,96158	0,204113	0,446403	0,6279808
CG34365-RF	Stacl	0,0155595	0	0	0,0136718	4,80412	0	-0,230627	0,373677	0,6279808
CG34366-RB	Stacl	0,0485971	0,808293	0,143306	3,04825	0	0	0,040196	0,907663	0,13772387
CG34367-RC	Stacl	6,3152	0,0121132	0,0128112	0,908945	0	0,0140061	0,042775	0,878676	0,13772387
CG34368-RC	Shawl	0,444247	0,147516	0,280594	0	0,365425	0	0,532668	0,072968	0,6279808
CG34369-RA	CG34367	1,5695	5,13432	5,74886	7,19571	0,0737901	0,619365	0,116243	0,744982	0,6279808
CG3436-RA	Fili	25,9506	0	0	13,7403	0	0	-0,215541	0,397469	0,6279808
CG3436-RB	ppk9	0,293671	0,692937	0,299563	1,59592	1,01995	1,13761	-0,519173	0,057364	0,6279808
CG34370-RG	CG3437	5,91992	8,32283	15,3759	13,6369	2,78976	7,34423	0,509317	0,010572	0,6279808
CG34371-RA	CG34370	5,72275	6,93606	9,12367	9,01448	8,47294	12,1203	0,303137	0,312433	0,6279808
CG34372-RC	side-V	11,9606	0	0,286612	0	446,497	0	0,241526	0,369675	0,6279808
CG34373-RD	CG43795	2,92008	3,59038	0	2,71793	0	1,69941	-0,49831	0,008759	0,6279808
CG34373-RE	Ect4	0,0132055	0,388117	0,0228217	0,401087	0,124214	0,449374	-0,479894	0,012772	0,6279808
CG34373-RF	Ect4	2,32237	0,0120285	0,0126781	58,0619	7,92139	0,0138561	-0,443716	0,01937	0,6279808
CG34373-RG	Ect4	9,34756	0,0118508	0,0124908	0,0149532	37,1274	0,0136453	-0,49579	0,009095	0,6279808
CG34373-RH	Ect4	0,0135466	9,50345	11,739	0,0127118	0,0202542	10,8908	-0,491666	0,010333	0,6279808
CG34374-RD	Ect4	5,20501	0,0123392	0,0130056	7,53931	0,0172182	0,0142253	0,22359	0,414351	0,6279808
CG34374-RE	RapGAP1	25,0387	5,57531	0,0332536	0,654659	1,18943	8,91572	0,224701	0,416055	0,6279808
CG34374-RF	RapGAP1	8,88196	0,183869	0,639321	0,0374699	0,157807	1,02134	0,231154	0,401302	0,6279808
CG34374-RG	RapGAP1	0,0158658	1,0475	11,3037	0,571051	0,0507533	0,0382507	0,235471	0,392945	0,6279808
CG34375-RA	RapGAP1	5,49814	3,33713	1,64222	2,65082	0,641555	0,773152	0,008942	0,969498	0,6279808
CG34376-RA	CG34375	16,3628	9,53543	18,1656	0,45358	0,377806	3,75686	-0,018294	0,943222	0,6279808
CG34376-RB	CG34376	0,026665	106,584	25,1347	43,1269	25,7956	25,4082	0,178322	0,47074	0,6279808
CG34377-RA	CG34376	28,4999	2,38267	0,0256	39,5835	19,9692	30,0179	-0,510597	0,105756	0,6279808
CG34378-RA	CG34377	0,146835	0,440927	0,290462	1,8208	0	1,70443	0,396684	0,187352	0,13772387
CG34378-RB	Pvf3	3,31066	2,99839	0,0251667	14,9858	0,0491598	14,2237	0,155609	0,636398	0,6279808
CG34378-RD	Pvf3	10,6952	0,0275495	14,7874	11,0737	13,8583	3,83812	0,49271	0,087918	0,6279808
CG34379-RD	Pvf3	0,0533443	0,0150103	4,86185	21,5939	15,5462	8,95119	0,238951	0,364676	0,6279808
CG34379-RF	Shrm	4,58113	6,62094	4,09315	9,32312	0,020667	2,10411	0,344978	0,179514	0,6279808
CG34379-RG	Shrm	9,8359	11,48	8,40788	11,8321	3,05746	0,0156302	0,335091	0,191228	0,6279808
CG3437-RA	Shrm	12,6343	21,6522	12,378	10,9662	9,44703	7,22229	-0,45737	0,052853	0,6279808
CG34380-RC	smal	16,7277	39,0493	16,6363	31,6256	7,02638	9,2658	0,220437	0,485591	0,13772387
CG34381-RA	TrissinR	0,596338	0,0213075	0,0224581	0,0246276	0,0024006	0,00180924	-0,214931	0,49249	0,6279808
CG34381-RB	TrissinR	0,175443	0	0	0	0,0370408	0,0279162	-0,214293	0,493431	0,6279808
CG34382-RC	CG34382	12,934	12,593	28,1165	18,4353	0,0463755	0,123757	0,596328	0,028847	0,6279808
CG34383-RB	CG34383	2,99186	6,59532	2,60431	17,7513	0,0381491	0,578141	0,389981	0,22661	0,6279808
CG34383-RC	CG34383	6,25379	9,40054	0,00630793	20,7694	1,11316	0,00974636	0,369957	0,250844	0,6279808
CG34383-RD	CG34383	0,00657036	0,00598473	0,00899727	0,0357036	7,57668	2,18839	0,388166	0,228898	0,13772387
CG34383-RE	CG34383	0,00937158	0,00853628	3,00368	0,0359569	106,231	0	0,444007	0,168094	0,6279808
CG34383-RF	CG34383	4,28129	5,95983	0	50,7442	16,8437	28,8273	0,388294	0,228828	0,6279808
CG34384-RA	CG34384	1,79696	2,54809	2,19038	4,59605	2,83227	0,866688	0,13149	0,62506	0,6279808
CG34385-RB	dpr12	2,3112	3,324	2,77945	4,78996	0,181183	2,20411	-0,349179	0,236659	0,6279808
CG34386-RC	CG34386	1,41143	30,2601	2,89414	1,38302	55,2264	25,6994	-0,3885	0,258732	0,6279808
CG34387-RC	futsch	1,14169	1,42265	32,7662	10,2951	6,02827	5,57596	-0,079943	0,783377	0,6279808
CG34387-RD	futsch	0,00352261	0,00320863	0,0240686	12,5358	4,61737	0,0258659	-0,138112	0,640607	0,6279808
CG34388-RC	natalisin	0,748971	0,864138	1,50203	1,0817	0,102311	1,1463	-0,172784	0,580792	0,6279808
CG34389-RA	cv-c	26,4921	39,0561	14,0016	21,9005	1,26141	11,701	0,465268	0,115497	0,6279808
CG34389-RC	cv-c	0,00744629	0,00678259	1,69428	0,00755247	4,64295	0,00770985	0,370696	0,200744	0,6279808
CG34389-RD	cv-c	8,80814	11,1605	6,29128	13,6137	2,22502	6,04802	0,370588	0,201454	0,6279808

gene_id	Symbol	W1_FPKM	W2_FPKM	W3_FPKM	MCM51_FPKM	MCM52_FPKM	MCM53_FPKM	FC	p-value	p-adj
CG34390-RA	Rgk2	1,44259	1,7254	1,48852	0,0116839	0,910738	0,906263	0,256597	0,313553	0,6279808
CG34391-RB	DIP-delta	1,46387	1,22672	78,5592	57,812	1,06579	2,6513	0,239838	0,495623	0,6279808
CG34391-RC	DIP-delta	0,0642344	0,0585091	0,0539122	9,30517	0,949223	0,982643	0,677949	0,034319	0,6279808
CG34392-RC	Epac	3,69058	18,4825	0,0338751	35,558	4,81595	0,0390275	0,095793	0,697458	0,13772387
CG34392-RD	Epac	8,89114	4,98946	21,6339	8,2827	8,11572	15,6272	0,077963	0,753028	0,6279808
CG34393-RA	CG34393	2,85087	2,69101	17,8451	2,87144	3,22228	2,2784	-0,109554	0,665719	0,6279808
CG34394-RA	GramD1B	11,2471	13,5704	8,48407	4,11278	14,4505	4,5032	0,432462	0,052188	0,6279808
CG34394-RC	GramD1B	2,02876	2,50015	2,14414	2,65125	5,78604	1,12232	0,42903	0,054007	0,6279808
CG34394-RD	GramD1B	3,65165	2,6992	3,72422	7,87456	1,2729	3,3369	0,42577	0,055532	0,6279808
CG34395-RB	nub	0,0157111	0,0143108	0,0150836	0,0162434	7,3856	0,0165819	0,116255	0,601273	0,6279808
CG34395-RD	nub	12,276	15,6808	12,0623	16,219	1,86952	9,75021	0,090454	0,688307	0,6279808
CG34396-RC	CG34396	9,76096	9,27628	0,0212685	5,14179	3,78526	0,0237389	0,296462	0,174306	0,6279808
CG34396-RD	CG34396	4,05509	3,86955	11,8176	0,0259977	0,214277	11,3898	0,228787	0,280557	0,6279808
CG34397-RB	Rgk3	8,70201	20,5713	33,5858	7,16465	11,0672	13,248	0,181183	0,541983	0,6279808
CG34397-RC	Rgk3	0,0531726	0,0266705	6,75426	18,8473	0,0423487	0,0319165	0,263474	0,368389	0,6279808
CG34398-RA	CG34398	1,1307	63,1527	0,973795	63,0002	0,0277562	141,715	-0,228705	0,315746	0,6279808
CG34398-RC	CG34398	0,0116893	7,41898	0,0112225	0,0787687	144,304	0,0213707	0,001344	0,995116	0,6279808
CG34398-RD	CG34398	8,16772	1,10565	6,91512	0,0204918	0,0283559	14,839	-0,011678	0,957983	0,6279808
CG34399-RC	Nox	5,19015	19,1985	3,24832	2,58195	2,5451	1,44833	0,396129	0,246255	0,6279808
CG34400-RC	Pcp	0,687336	0,584335	10,5121	1,52492	17,0015	0,00584986	-0,051773	0,841712	0,6279808
CG34400-RD	dysc	1,22836	0,0204878	1,94746	3,4852	2,63761	5,29894	-0,047022	0,860574	0,6279808
CG34400-RE	dysc	0,0224926	0,727402	0,0215943	0,0236296	0,0251161	2,35269	-0,047022	0,860574	0,6279808
CG34400-RF	dysc	1,12799	0,798444	0,776268	0,0208484	2,07016	0,018929	0,035085	0,894848	0,6279808
CG34401-RA	dysc	0,0243493	104,221	0,0233768	0,0256936	7,46903	9,48861	-0,180617	0,501802	0,6279808
CG34402-RB	CG34401	5,32423	6,61664	4,64102	10,4781	5,91749	23,8879	NA	NA	0,6279808
CG34402-RC	CG34402	0,0995119	0,0906423	0,0955373	0	0	0,101302	-0,326885	0,33821	0,6279808
CG34402-RD	CG34402	0,0312711	0,0284839	0,0300221	0	0	0,0352075	-0,326885	0,33821	0,6279808
CG34403-RA	CG34402	0,0336607	0,0306605	0,0323162	0,438083	0,656916	0,603581	-0,106398	0,67715	0,6279808
CG34403-RB	pan	2,23639	7,1297	3,29138	7,76888	3,69186	2,90866	-0,127652	0,616597	0,6279808
CG34403-RC	pan	0,0234755	0,0213832	0,0225379	0,02472	0,0334834	0,0252351	-0,131765	0,602426	0,6279808
CG34403-RD	pan	0,0240567	0,0219125	0,0230959	0,0253671	0,03436	0,0258957	-0,103559	0,68846	0,6279808
CG34403-RE	pan	1,34414	1,41537	1,18978	0,0198677	0,0269109	1,07884	-0,118632	0,644634	0,6279808
CG34403-RF	pan	0,0225156	0,0205088	0,0216163	0,0236551	0,0320409	0,024148	-0,100385	0,696821	0,6279808
CG34403-RG	pan	0,0226081	0,020593	0,0217051	0,0237574	0,0321796	0,0242525	-0,118632	0,644634	0,6279808
CG34403-RH	pan	1,72336	1,5791	3,08255	4,19332	5,22401	2,94479	-0,159562	0,53737	0,6279808
CG34403-RI	pan	4,41602	1,67692	2,22957	5,70936	0,0343732	0,0259057	-0,096724	0,669276	0,6279808
CG34403-RJ	pan	0,0237199	0,0216058	0,0227726	0,0249919	0,0338517	0,0255127	-0,024744	0,925842	0,6279808
CG34404-RC	pan	1,28272	0,0156831	1,08272	0,017864	0,0241969	0,0182362	0,311703	0,18866	0,6279808
CG34404-RD	CG34404	0,0248811	0,98829	2,33374	0,0262881	12,6061	0,0768282	0,360792	0,136475	0,6279808
CG34404-RE	CG34404	0,025525	0,0122938	0,0301697	0,0270101	0,108509	12,2771	0,360792	0,136475	0,6279808
CG34405-RC	CG34404	7,92527	3,50679	4,67564	9,35244	0,0356074	0,0817787	-0,049506	0,841856	0,6279808
CG34406-RA	NaCP60E	1,78673	14018,6	0,388706	0	0	0,0480627	0,359947	0,20545	0,6279808
CG34407-RC	CR44003	1,46106	0,307074	2,16132	1,85578	605,452	0,936016	-0,059513	0,834318	0,6279808
CG34407-RD	Not1	13,3314	20,7998	12,9435	30,7619	5,82952	9,00799	-0,059987	0,833016	0,6279808
CG34407-RE	Not1	2,35646	0,00742707	2,81031	0,00828355	0,0112201	1,28523	-0,063353	0,82373	0,6279808
CG34408-RA	Not1	0,00814377	0,00741791	0,00781851	0,00827315	5,74875	0,00844554	0,039941	0,849524	0,6279808
CG34408-RB	CG34408	0,013187	0,0120117	90,6342	0,515511	0,0159049	31,4277	0,030412	0,884651	0,6279808
CG34408-RC	CG34408	5,48022	5,37352	0,78918	2,69501	4,75205	0,692392	0,092829	0,661741	0,6279808
CG34408-RD	CG34408	0,0114702	0,0104478	3,02003	67,5094	43,7036	2,34573	0,046572	0,825431	0,6279808
CG34409-RA	CG34408	9,69201	9,23505	40,3689	0	131,142	221,156	-0,2713	0,408244	0,6279808
CG3440-RA	CG34409	0,621591	41,0623	27,7149	82,8169	0,0763792	0,381491	-0,626555	0,076701	0,6279808
CG34410-RB	Nplp1	2,00215	0,0813695	0,0272885	3,73863	0,0379222	3,44003	0,442797	0,06486	0,6279808
CG34410-RC	Nplp1	12,681	1,94819	3,05258	20,6509	2,2898	20,4673	0,364711	0,135156	0,6279808
CG34411-RB	Nplp1	0,0292544	0,0266233	39,1001	0,0312354	8,15383	0,0106913	-0,314907	0,252767	0,6279808
CG34412-RB	Rab26	3,08732	3,47882	3,3984	9,04238	9,22957	9,61405	-0,169877	0,493718	0,6279808
CG34412-RC	Rab26	2,32779	1,57984	2,97976	8,30806	4,14554	4,70748	0,025042	0,914287	0,6279808
CG34412-RE	Lgr4	0,755153	0,667615	0,810285	24,8348	0,79337	3,88418	0,031721	0,891607	0,6279808
CG34412-RF	Tlk	0,0143747	3,17743	0,0138791	5,41394	1,27331	6,22419	-0,065162	0,78987	0,6279808
CG34412-RG	Tlk	8,09115	0,013168	0,0137319	7,14528	2,25958	0,0126093	0,004126	0,98572	0,6279808
CG34412-RH	Tlk	2,57258	0,0130283	0,00968106	0,0911043	3,4412	0,015213	0,073236	0,750216	0,6279808
CG34412-RI	Tlk	0,0144565	0,00918503	0,0109418	0,0148154	0	0,0150463	-0,112529	0,646237	0,6279808
CG34413-RB	Tlk	0,0143032	0,0103812	0,031224	7,70678	0,0200675	1,59719	-0,016661	0,949127	0,6279808
CG34413-RC	Tlk	0,0100838	0,0296242	31,4877	6,18732	4,31075	0,0119084	-0,017175	0,947557	0,6279808
CG34413-RD	Tlk	0,011397	21,3687	23,0329	0,0149025	1,41256	1,96975	-0,018415	0,943741	0,6279808
CG34413-RE	NKAIN	16,4619	24,5074	15,5439	28,0594	13,5166	12,8063	0,130548	0,597022	0,6279808
CG34414-RA	NKAIN	0,0231385	0,0210761	0,0222143	0,0243455	0,0329762	0,0248528	0,084931	0,762444	0,6279808
CG34414-RG	NKAIN	0,0211736	0,0192864	0,0203279	0,0221744	0,0300354	0,0226365	0,087795	0,754028	0,6279808
CG34414-RH	NKAIN	28,4862	42,5362	28,3207	41,6752	22,1148	23,7989	-0,30735	0,236869	0,6279808
CG34414-RI	spri	22,6342	47,7935	22,9497	24,0166	13,627	22,3852	0,299921	0,305713	0,6279808
CG34415-RC	spri	5,66603	7,39458	5,44506	10,4075	3,82008	3,79182	0,355324	0,182848	0,6279808
CG34415-RD	spri	0,0107973	0,00983493	0,0103661	3,32981	2,71499	2,72128	0,41416	0,122206	0,6279808
CG34416-RE	spri	0,021617	0,0196902	0,0207536	0,0226625	0,0306965	0,0231347	0,726152	0,001956	0,6279808
CG34416-RF	mute	4,18563	2,75256	10,4151	9,21182	694,051	3,74382	0,478265	0,074067	0,6279808
CG34416-RG	mute	0,490926	7,66956	679,706	0,150947	11,6691	8,0933	0,697256	0,005689	0,6279808
CG34416-RH	Ank2	24,9946	0,00440744	0,00816783	11,1426	5,70733	5,96529	0,52748	0,069544	0,6279808
CG34416-RI	Ank2	0,0074738	0,00680714	13,0821	0,990256	1,22257	0,669488	0,514208	0,075915	0,6279808
CG34416-RJ	Ank2	0,00483871	0,00430714	15,2728	52,0418	84,9706	56,586	0,476842	0,074653	0,6279808
CG34416-RK	Ank2	0,00747324	0,00437762	0,0915229	0,0236042	0,0319721	0,0240961	0,495766	0,063759	0,6279808
CG34416-RL	Ank2	0,0047286	0,0042359	80,7161	38,7755	17,0751	11,0376	0,478262	0,07408	0,6279808
CG34416-RM	Ank2	0,00480598	0,00774933	1,66522	0,659175	0,350706	0,327194	0,333003	0,140388	0,6279808
CG34416-RN	Ank2	0,00465039	17,5876	5,52002	7,22456	4,89746	4,7295	0,674737	0,001667	0,6279808
CG34417-RC	Ank2	0,00850762	28,6987	11,2556	5,32661	7,24145	11,8693	-0,454597	0,068546	0,6279808
CG34417-RD	Ank2	18,7537	0,0944315	0,549786	11,3015	7,71044	7,59617	0,314502	0,116283	0,6279808
CG34417-RF	Ank2	20,2536	39,5552	60,7867	13,8453	9,93933	12,9989	0,310391	0,119889	0,6279808
CG34417-RG	CG34417	0,0207655	0,0189147	0,0199361	4,28023	0,0299547	0,0225757	0,421095	0,095629	0,6279808
CG34417-RH	CG34417	6,23508	8,30357	5,53723	0,0221149	4,81275	4,67866	-0,015099	0,951928	0,6279808
CG34418-RA	CG34417	0,00586785	0,00534484	3,05053	10,9033	0,00803227	0,0060536	0,514039	0,01612	0,6279808
CG34418-RB	CG34417	0,00579173	0,0052755	0,0055604	0,00593003	0,00792669	0,00597403	0,493835	0,021041	0,6279808
CG34418-RC	CG34417	60,4731	78,3893	48,0047	0,00585209	33,157	39,8411	0,514039	0,01612	0,6279808
CG34418-RD	sif	0,0961803	18,0822	0,0923389	12,0606	8,76011	7,31196	-0,334897	0,204262	0,6279808
CG34418-RE	sif	0,00779043	0,00763239	0,00747928	0,00851696	35,732	0,00869444	-0,340082	0,198141	0,6279808
CG34418-RF	sif	17,2394	0,00610617	15,3843	0,00678769	15,9878	0,00692913	-0,293764	0,256559	0,6279808
CG34418-RG	sif	0,00837924	0,0065104	0,00804457	1,59058	11,4862	0,00739537	0,515284	0,015692	0,6279808
CG34418-RH	sif	0,00670367	0,00652947	0,00643593	0,00726598	0,743386	0,00741738	0,150518	0,576835	0,6279808
CG34419-RC	sif	0,00714746	0,00695021	0,00686199	1,8845	0,0297055	1,51207	0,071245	0,799362	0,6279808
CG3441-RA	sif	0,00716839	0,0279201	0,00688209	31,3153	13,2898	0,0335231	-0,438722	0,0805	0,6279808
CG3441-RB	sif	0,0076303	144,476	0,00732555	10,6254	0,0210174	111,254	-0,449522	0,072207	0,6279808
CG3441-RC	Hmx	2,04165	3,53096	0,213112	0,441416	37,5682	18,0439	-0,448958	0,072438	0,6279808
CG34420-RC	CG44837	5,28325	6,04752	0,481363	0,103548	0	0,413844	0,113917	0,593021	0,6279808
CG34421-RB	Snoo	1,5571	0,00888886	0,0100582	793,715	364,787	229,37	-0,004239	0,987206	0,6279808
CG34421-RC	Snoo	0,0112489	0,0438783	1,86579	0,10846	0,14691	0,11072	0,005971	0,982011	0,6279808
CG34421-RD	Snoo	0,0104766	0,0364619	0,0093689	0,0514569	0,0696987	11,1218	-0,004849	0,985362	0,6279808
CG34421-RE	Snoo	3,74618	5,8503	0,0462479	13,4737	11,3599	9,33045	-0,225471	0,435082	0,6279808
CG34421-RF	Snoo	0,00975866	0,151986	0,038431	0,011618	0,0157366	0,0118601	0,071908	0,7879	0,6279808

gene_id	Symbol	W1_FPKM	W2_FPKM	W3_FPKM	MCM51_FPKM	MCM52_FPKM	MCM53_FPKM	FC	p-value	p-adj
CG34422-RA	htk	0,0339431	0,0309177	0,0325874	3,08215	0,211606	6,18252	-0,049622	0,817676	0,6279808
CG34422-RB	htk	8,60312	9,21162	9,0478	4,20928	5,31773	5,99487	-0,510083	0,066478	0,6279808
CG34423-RA	CG34423	0,253488	7,81328	0,0582166	10,5977	15,9782	13,4778	-1,207025	0,000716	0,6279808
CG34424-RA	Mthfs	5,76189	7,44924	9,01142	8832,11	0,0206909	0,0155939	-0,664536	0,019003	0,6279808
CG34425-RA	CG34425	0,0999333	0,0910261	0	3,14026	0,0393778	0,0226441	0,110321	0,4419	0,6279808
CG34426-RA	CG34426	11,4437	23,3117	69,9145	0,760793	0,389304	0,314044	-0,366208	0,296364	0,6279808
CG34427-RA	CG34427	0,807878	0,250777	1,18383	0,307633	7,37414	112,643	0,470771	0,188131	0,6279808
CG34428-RA	CG34428	0,238369	0,347397	40,7912	0	0	0	0,348002	0,192853	0,6279808
CG34429-RA	CG34429	0,220253	15,8025	24,8776	0	0	0	0,243786	0,307775	0,6279808
CG34430-RA	pcx	3,44214	1,65796	2,9471	7,53227	9,92549	7,72754	-0,02015	0,954966	0,6279808
CG34431-RA	CG34430	0,249343	0,0350396	14,2872	0,021245	5,06691	0,234705	0,139766	0,695754	0,6279808
CG34432-RA	CG34431	0,261839	0,0182162	0,0192	39,0236	0,088409	0,0216877	NA	NA	0,6279808
CG34433-RA	CG34432	0	0,0436872	0,161645	0,136209	2730,89	0,054223	-0,041811	0,731452	0,6279808
CG34434-RB	CG34433	0	467,874	0	0	0,147032	0,201988	-0,143921	0,681585	0,6279808
CG34435-RA	CG34434	0,293063	0,195757	0,243843	14,4332	8,18467	1,20007	NA	NA	0,13772387
CG34436-RA	CG34435	0	0	0	0,0168112	0,0227708	0	0,087229	0,775527	0,6279808
CG34437-RA	CG34436	0,322715	0,220463	0	0,345431	0	0,516723	-0,261837	0,423777	0,6279808
CG34438-RF	CG34437	8,41034	0	0	0	13,8025	12,0598	0,151579	0,561947	0,6279808
CG34439-RA	Cap-G	4,5474	4,70847	391,462	6,57167	453,292	407,345	-0,354314	0,240492	0,6279808
CG34439-RB	ND-MWFE	127,994	0,744291	0,0871781	1,61514	0,0282482	0,0153414	-0,368973	0,220263	0,6279808
CG3443-RB	ND-MWFE	0,0908049	0,175984	8,82034	1,00498	7,3919	302,339	0,177885	0,539342	0,6279808
CG34440-RA	lmgA	11,7461	0,0306969	0,0323547	13,0254	73,5118	7,59185	0,227524	0,303087	0,6279808
CG34440-RB	lmgA	0,0337007	33,6443	33,2641	0,0363737	23,7943	0,0371316	0,004483	0,985697	0,6279808
CG34442-RA	CG34442	1,32649	0,943954	0,281941	0,556363	0,323999	1,5597	0,203208	0,569277	0,6279808
CG34443-RA	CG34443	2,2943	2,67496	1,71451	0,415819	0,312247	0,464713	0,128944	0,703374	0,6279808
CG34444-RA	CG34444	1,00453	0,254883	0	0	0	0,697717	0,559135	0,116769	0,6279808
CG34445-RA	CG34445	37,9746	37,1265	0,5376	0,666955	0,651251	0,402812	-0,786208	0,011291	0,6279808
CG34446-RA	CG34446	88,4564	70,3794	3539,29	63,7603	127,068	128,743	-0,532969	0,095607	0,6279808
CG34447-RA	CG34447	1,68676	0,348907	9,63367	0,799826	0	0,637318	0,545453	0,098506	0,6279808
CG34448-RA	CG34448	0,542652	16,6993	0,056962	0,0564624	1,35326	1,21862	-0,578784	0,104342	0,6279808
CG34449-RA	Zdhhc8	5,36944	9,6476	5,711	4,44471	0,0283559	2,251	-0,076274	0,758398	0,6279808
CG34449-RB	Zdhhc8	1,47318	0,0182568	0,0192427	0,0155864	5,88244	2,08402	-0,077324	0,755389	0,6279808
CG34449-RC	Zdhhc8	6,34023	7,14237	6,62472	0,0496351	0,497057	0,0159111	0,035993	0,875976	0,6279808
CG34450-RA	phol	5,36919	16,183	20,4499	2,24762	11,5378	3,83259	-0,716766	0,042858	0,6279808
CG34451-RA	CG34450	0,126485	0,138254	0,194293	0,0500499	0,0672311	0,0506694	-0,262545	0,462469	0,6279808
CG34452-RB	CG34451	1,50331	0,732428	3,79278	27,8814	14,3241	3,06182	0,673683	0,011819	0,6279808
CG34453-RB	CG34452	4,37715	38,7565	3,58338	54,1384	2,8129	2,7782	-0,327119	0,326011	0,6279808
CG34454-RA	CG34453	1,35901	1,32325	1,16976	2,68502	3,0417	2,01887	0,202462	0,550044	0,6279808
CG34455-RC	CG34454	2,51592	2,23136	5,4665	5,21907	1,79481	2,58799	-0,309056	0,229966	0,6279808
CG34456-RA	Pdxk	22,7162	22,2018	0,0367181	4,75299	0,0565407	0,0426125	-0,965283	0,006534	0,6279808
CG34457-RA	CG34456	0,105384	1,28229	10,3784	3,81965	1,47762	1,92085	-0,040646	0,905157	0,6279808
CG34458-RA	CG34457	0,468798	0,606809	0,0408041	119,045	0	0	0,07663	0,535079	0,6279808
CG34459-RA	CG34458	0	0	75,6459	0	0,0611507	0,0460868	-0,355085	0,261197	0,6279808
CG3445-RA	CG34459	4,24283	276,399	4,34003	0,0383868	0,0519951	0,0174781	0,099878	0,752368	0,6279808
CG34460-RA	Dpse\GA17457	78,1023	79,703	146,316	89,2993	125,195	139,79	-0,262665	0,45495	0,6279808
CG34461-RA	CG34460	1,20707	2,24492	0,0158328	7,05797	1,94232	16,1849	0,300791	0,134273	0,6279808
CG34462-RA	CG34461	60,1222	67,2542	136,146	88,2156	0,292791	0,220665	0,126216	0,655257	0,6279808
CG3446-RA	CG34462	0,281473	0	0,506684	0,134195	10,2409	193,117	-0,171746	0,559714	0,6279808
CG3448-RB	CG3448	0,068567	7,07172	6,26874	42,2599	13,5525	0,0825819	0,494566	0,027197	0,6279808
CG3450-RA	ubl	22,8057	0,305972	46,6963	2,0734	27,8276	38,4085	-0,200353	0,49269	0,6279808
CG3454-RA	Hdc	0,806787	0,927783	1,04566	1,54147	0,63394	0,74461	-0,087376	0,761735	0,6279808
CG3455-RA	Rpt4	20,8759	8,64055	27,2373	0,0677535	0,0194255	2,69438	0,086054	0,738272	0,6279808
CG3456-RA	Mct1	14,5107	8,59218	6,71311	8,69514	9,04681	12,6549	0,28892	0,336818	0,6279808
CG3457-RA	CG3457	1,29343	1,98602	70,8017	0,0197842	2,02507	0,34911	0,276276	0,437842	0,6279808
CG3458-RA	Top3beta	6,941	10,3072	8,84623	13,2472	6,49262	8,02043	-0,170738	0,470035	0,6279808
CG3460-RA	Nmd3	15,6768	12,8796	33,8456	14,8591	10,086	24,9233	-0,176049	0,554414	0,6279808
CG3461-RA	pn	4,02278	4,03065	4,85523	0,0197842	4,03671	3,10861	0,211351	0,425457	0,6279808
CG3466-RA	Cyp4d2	2,82945	105,034	170,132	3,52328	6,77852	500,182	-0,866497	0,000221	0,6279808
CG3469-RA	betaggt-I	15,3717	12,7864	2,19487	0,0593875	0,0294118	5,44086	-0,015121	0,948601	0,6279808
CG3473-RA	CG3473	0,151319	0,137832	0,29055	0	18,5453	0	0,008457	0,975068	0,6279808
CG3474-RA	Dsim\GD22005	13,2252	13,7111	18,8283	13,291	963,35	826,355	2,807268	2,06E-21	0,6279808
CG3476-RA	MME1	7,38231	8,99985	13,0431	12,6059	0,00776193	15,5474	-0,521896	0,057756	0,6279808
CG3477-RA	Pxd	7,61991	0,622619	9,04542	12,0737	28,2184	5,5798	-0,017703	0,940542	0,6279808
CG3478-RA	ppk	3,14263	0,0130566	0,0720325	36,0513	0	0,0746125	-0,256565	0,3393	0,6279808
CG3479-RA	osp	0,0112899	0,0102836	3,20833	0,0115528	2,92854	0,0117935	0,452595	0,128782	0,6279808
CG3479-RB	osp	13,9262	17,7423	7,58527	12,1972	2,3074	8,92402	0,449069	0,131111	0,6279808
CG3479-RC	osp	24,9801	33,1852	14,0405	27,5122	9,87737	9,94403	0,449129	0,131063	0,6279808
CG3480-RA	mip130	6,66743	6,55183	5,72177	12,0881	1,85386	0,0495862	-0,044034	0,863802	0,6279808
CG3481-RC	Adh	0,0554482	0,0505061	0,0532336	0,0632042	0,0856105	0,0645212	-0,622361	0,063073	0,6279808
CG3481-RE	Adh	55,7088	62,2465	0,0623354	164,043	0,102762	122,577	-0,622227	0,063152	0,6279808
CG3481-RF	Adh	0,0326515	0,0297413	0,0313474	0,0351512	0,0476125	0,0358837	-0,615223	0,06468	0,6279808
CG3481-RH	Adh	225,82	140,327	773,53	362,701	834,301	727,159	-0,615094	0,06476	0,6279808
CG3481-RI	Adh	0,0335637	0,0305722	0,0322232	0,0362138	0,0490518	0,0369684	-0,622926	0,062817	0,6279808
CG3483-RA	CG3483	0	0	19,7241	7,26223	0	0,0284716	NA	NA	0,6279808
CG3488-RA	Hydr2	17,6843	22,9016	2,88048	26,2964	0	0	0,145886	0,537145	0,6279808
CG3491-RA	CG3491	0,0288491	0	6,44683	1530,19	1523,78	5,98924	-0,017422	0,917755	0,6279808
CG3492-RA	CG3492	0	0,0867741	0	0,110481	0	0	-0,041811	0,731452	0,6279808
CG3493-RA	Golgin245	11,4753	11,8289	1012,32	1,36463	0,326377	10469,5	-0,078695	0,750214	0,6279808
CG3494-RA	CG3494	0	0,565753	0	0,11938	0	0	0,01562	0,897983	0,6279808
CG3494-RB	CG3494	0	0	0	0,0216783	0	11,8666	0,01562	0,897983	0,6279808
CG3495-RA	Gmer	3,37036	2,75728	6,14193	3,56579	3,44589	4,91134	0,01063	0,972592	0,6279808
CG3496-RA	vir	2,52396	2,52837	3,18777	0,0314891	7,50163	8,71339	-0,200759	0,477109	0,6279808
CG3497-RA	Su(H)	3,56985	3,04716	0,0288524	4,19657	1,937	1,3924	0,042766	0,877184	0,6279808
CG3499-RB	YME1L	15,8962	15,7004	21,1722	21,7878	14,8262	16,5132	-0,132922	0,488414	0,6279808
CG3500-RA	CG3500	3,23084	0,840845	14,429	0,0700587	5,08198	0,0715186	-0,258805	0,37316	0,6279808
CG3501-RA	EMC8-9	30,8077	29,1931	44,6159	49,4348	35,0108	8,22422	-0,168021	0,474566	0,6279808
CG3502-RB	CG3502	0,763964	0,0552338	0	0,13191	1,39658	0,134658	-0,67738	0,01553	0,6279808
CG3504-RA	inaD	0,0294762	0,026849	0	0,291843	0	0	-0,290483	0,277866	0,6279808
CG3505-RA	CG3505	21,257	11,5815	4,98221	2206,8	6,89508	30,7247	-0,270548	0,244677	0,6279808
CG3506-RA	vas	2,00975	0,164804	0	0,0586044	22,5033	0,0399311	-0,136824	0,598574	0,6279808
CG3508-RA	Hexim	6,0069	7,47375	6,21984	9,52757	2,9486	4,59576	0,121362	0,576984	0,6279808
CG3508-RB	Hexim	3,66378	3,31782	3,37931	2,91849	1,83814	2,58977	0,122254	0,574069	0,6279808
CG3509-RA	BigH1	0,924857	22,6542	7,72628	17,2275	7,01162	6,08449	-0,041571	0,907317	0,6279808
CG3510-RA	CycB	0,0264975	1,28387	8,09875	2,16716	4,27025	8,89033	0,008063	0,98071	0,6279808
CG3510-RB	CycB	0,0258648	1,91879	0,041854	0,0540308	2,29429	1,14969	0,018632	0,9554	0,6279808
CG3510-RC	CycB	13,7802	0,0235594	4,37749	3,0295	13,3423	0,474658	0,018498	0,955724	0,6279808
CG3510-RD	CycB	0,0447331	8,16854	0,0254391	0,0482186	0,0731851	0,0551567	0,014726	0,965051	0,6279808
CG3511-RA	CG3511	3,37871	3,57888	4,47668	3,79186	2,7104	10,0851	0,141494	0,528171	0,13772387
CG3513-RA	CG3513	0	0	0,593977	6,28363	5,7714	5,64435	-0,725064	0,023148	0,6279808
CG3515-RA	CG3515	0	0	18,5173	0,0743967	20,2066	0	-0,013096	0,914398	0,6279808
CG3517-RA	CG3517	0	0,0531057	0,152218	0,0660008	0	0	-0,013096	0,914398	0,13772387
CG3520-RA	CG3520	0,512857	0,663046	0,770327	0,131384	0	0,134121	0,543307	0,066959	0,13772387
CG3522-RA	Start1	8,06384	8,06234	10,7937	9,11135	18,5625	118,51	0,137544	0,51817	0,6279808
CG3522-RB	Start1	0,0288331	0,0262632	0,0276815	1,7478	129,398	0,0687412	0,138307	0,515784	0,6279808

gene_id	Symbol	W1_FPKM	W2_FPKM	W3_FPKM	MCM51_FPKM	MCM52_FPKM	MCM53_FPKM	FC	p-value	p-adj
CG3523-RA	FASN1	7,76281	5,13001	14,509	0,287082	899,39	682,631	-1,133372	0,000211	0,6279808
CG3523-RB	FASN1	0,00761799	0,00693899	0,00731372	0,140338	0,190089	0,143262	-1,132798	0,00021	0,13772387
CG3524-RA	FASN2	0,365637	2,29567	0,397002	0,00772966	0,0104699	0,00789073	-0,971335	0,000137	0,6279808
CG3525-RA	eas	47,0046	35,1703	53,5284	0,0450535	32,9055	38,3437	0,090199	0,681732	0,6279808
CG3525-RB	eas	0,0294631	39,2448	0,0282863	0,149917	7,8496	0,0321299	0,089218	0,684978	0,13772387
CG3525-RC	eas	0,0285836	0,026837	0,027442	422,865	0,0412716	0,0311048	0,081473	0,710122	0,6279808
CG3525-RD	eas	0,0295157	0,0260359	0,0283368	60,6747	0,0427135	0,0321915	0,090955	0,679204	0,6279808
CG3525-RE	eas	0,0281813	0,0268849	0,0270558	41,9415	0,0406513	0,0306372	0,087727	0,689782	0,6279808
CG3526-RA	CG3526	0,327648	0,186528	0,0225861	0	0,0329154	0,0409521	0,219637	0,244398	0,6279808
CG3526-RC	CG3526	0,0641097	0,0583956	0,0200137	35,9393	11,0935	0,0394857	0,189942	0,384034	0,6279808
CG3527-RA	CG3527	12,2941	4,05565	4,27467	5,56978	0	128,137	0,008303	0,978788	0,6279808
CG3528-RA	CG3528	0	0	0	0	0	0	0,01562	0,897983	0,6279808
CG3529-RB	CG3529	15,2124	0,0624556	0,0658284	5,06827	1,13995	14,202	-0,013187	0,944142	0,6279808
CG3530-RA	CG3530	0,0211127	0,0192309	10,8538	22,3239	9,06903	13,2935	-0,018666	0,922902	0,6279808
CG3530-RB	CG3530	0,0220645	0,0200979	0	0,0360158	14,1789	0,0225681	-0,029018	0,880176	0,6279808
CG3530-RC	CG3530	9,83233	11,8577	8,64211	0,035283	7,76035	0,0236388	-0,018134	0,924041	0,6279808
CG3532-RB	GCC185	6,4089	0	7,04558	8,62752	0	5,66993	0,222871	0,391252	0,6279808
CG3533-RA	uzip	39,0048	28,2329	62,6073	4,91726	1,29415	2,78719	0,02704	0,909116	0,6279808
CG3534-RA	CG3534	12,7365	12,9802	8,42876	9,10718	20,9812	7,85713	-0,001438	0,995108	0,6279808
CG3539-RC	Slh	0,0299302	16,4162	0,0621523	0,0320092	29,4638	1,31035	-0,05776	0,78039	0,6279808
CG3539-RD	Slh	0,0300117	0,035811	2,1436	0,0321028	0,0582759	0,0172805	-0,05776	0,78039	0,6279808
CG3539-RE	Slh	16,0613	0,0235318	0,0156957	19,2545	0,0370562	0,0166682	-0,05776	0,78039	0,6279808
CG3539-RF	Slh	0,0461495	16,178	0,0151593	0,0329702	11,6906	1,60859	-0,05776	0,78039	0,6279808
CG3540-RA	Cyp4d14	3,79699	1,6353	4,63392	91,7937	12,8097	7,9776	-0,093822	0,779163	0,6279808
CG3541-RB	pio	0,0614849	0,0560047	0,0590292	270,383	0,0964329	0,0313951	-0,184416	0,345144	0,6279808
CG3541-RC	pio	26,8476	28,352	30,8518	0,0933944	29,9388	15,4934	-0,444645	0,049101	0,6279808
CG3542-RA	CG3542	0,0231466	1,71651	3,96194	1,94991	2,90188	6,65014	-0,013887	0,959055	0,6279808
CG3542-RB	CG3542	5,35009	7,61854	7,22698	6,45039	16,487	5,71435	-0,013887	0,959055	0,6279808
CG3544-RA	CG3544	0,0361861	0,0329608	7,1178	0,0405857	8,14757	6,68954	0,135211	0,679409	0,6279808
CG3546-RA	CG3546	0,256639	0	0,708615	4,0187	0	2,70201	-0,466184	0,160494	0,6279808
CG3548-RA	CG3548	17,0819	17,1864	17,5381	8,91085	9,56694	11,7149	0,277069	0,19371	0,6279808
CG3552-RA	CG3552	2,74245	0,0447483	0,0471649	3,43815	10,0665	12,3528	0,183651	0,480763	0,6279808
CG3552-RC	CG3552	0,0491271	0,0464044	0,0489104	0,0602014	3,01286	0,0502155	0,183651	0,480763	0,6279808
CG3552-RD	CG3552	0,0509452	20,0622	26,7358	26,6166	0,0781173	0,0588739	0,105735	0,657879	0,13772387
CG3556-RA	CG3556	3,54221	7,81238	4,68255	5,54247	3,32971	2,6078	0,006999	0,977446	0,6279808
CG3557-RA	CG3557	0,202894	0,154008	0,162325	3,67712	7,26779	160,319	0,173904	0,528929	0,6279808
CG3558-RA	CG3558	8,99459	1,40205	9,72862	6,12952	0,027895	3,34422	-0,055761	0,790261	0,6279808
CG3558-RB	CG3558	1,71418	2,94131	2,47247	22,9026	0,0277648	0,0585664	-0,055498	0,79115	0,6279808
CG3558-RC	CG3558	4,56015	8,20793	3,75819	1,24643	1,35778	26,3484	-0,055963	0,789569	0,6279808
CG3560-RA	Dpse\GA17519	312,759	0,0937484	0,0988112	0,0315343	0,203064	0,153041	-0,216106	0,482987	0,6279808
CG3561-RA	Dbp21E2	4,77535	1,33418	2,93631	7,0977	2,36156	2,70065	-0,244085	0,344909	0,6279808
CG3563-RA	jvl	0,910253	0,887305	1,4335	9,36216	1,89664	0,0183107	-0,069885	0,78527	0,6279808
CG3563-RB	jvl	1,06304	1,40127	1,53503	0,023279	0,0349383	4,00632	-0,039197	0,880264	0,6279808
CG3563-RC	jvl	6,21652	5,97232	4,76073	5,03161	3,91202	0,0221776	0,013372	0,958792	0,6279808
CG3564-RA	CHOp24	104,909	102,959	98,5966	142,447	131,87	17,0861	-0,335867	0,116043	0,6279808
CG3565-RA	CG3565	0,220498	0,100422	0	0	0	0,144859	0,223154	0,32357	0,6279808
CG3566-RB	CG3566	0,0437685	0,0398673	0,0288655	0,0321655	0,0435684	0,0328357	-0,51254	0,106357	0,6279808
CG3566-RC	CG3566	59,4826	50,1921	0,040883	289,964	332,079	0,0479599	-0,480731	0,122934	0,6279808
CG3567-RA	Cyp6u1	12,5195	0,0286921	234,415	0,0338163	1,10111	4,29911	-0,019674	0,928387	0,6279808
CG3568-RA	CG3568	2,04178	1,73581	2,98702	2,1842	0,48273	1,95487	0,152566	0,585137	0,6279808
CG3569-RA	Or59b	0,0828019	0,0754217	0	0	0	0	0,044335	0,715924	0,6279808
CG3570-RA	Samtor	5,74334	7,14464	12,0889	6,09526	5,27582	9,94223	-0,000108	0,999693	0,6279808
CG3571-RA	KLHL18	1,90051	3,18583	9,98494	0	0	0	-0,259262	0,219786	0,6279808
CG3571-RB	KLHL18	7,11203	5,57592	4,01718	0	0	0	-0,261487	0,21714	0,6279808
CG3572-RB	vimar	13,1332	5,36315	10,2022	0	1,5618	4,844	0,064545	0,792709	0,6279808
CG3572-RC	vimar	0,0193393	0,407354	0,0185669	0,08524	0	2,22189	0,064545	0,792709	0,6279808
CG3573-RA	Ocrl	6,91974	7,7986	3,21303	4,49814	4,47479	2,28944	-0,307732	0,123917	0,6279808
CG3576-RA	schlank	20,7344	11,1248	0,0420203	0,0484312	0,0656004	0,0494404	-0,347773	0,203689	0,6279808
CG3576-RB	schlank	30,9443	27,2792	76,306	67,723	151,882	142,501	-0,36536	0,182766	0,6279808
CG3578-RA	bi	6,02983	0	0	1,0019	7,44662	0	0,259315	0,391942	0,6279808
CG3581-RA	CG3581	0	0	0	0,125028	0	0	-0,041811	0,731452	0,6279808
CG3582-RA	U2af38	18,5012	19,1181	5,67107	2,60997	0,0404853	0,0307352	-0,324834	0,190669	0,6279808
CG3584-RB	qkr58E-3	38,917	44,9404	17,6266	20,1357	75,9142	12,1196	0,336922	0,096699	0,6279808
CG3585-RA	Rbcn-3A	5,78316	5,99358	17,285	23,2737	9,48808	12,4052	-0,299424	0,213877	0,6279808
CG3587-RA	CG3587	4,25213	1,7264	0,0172805	0,0430518	0,0257316	0,0193929	-0,190756	0,516047	0,13772387
CG3588-RA	CG3588	9,15255	12,4864	10,599	15,4701	5,01108	6,71722	-0,532715	0,024957	0,6279808
CG3588-RB	CG3588	0,029876	0,0272132	7,18359	7,57702	19,7307	4,07275	-0,544767	0,022191	0,6279808
CG3588-RC	CG3588	53,3068	53,9726	0,0187424	0,0203651	4,22485	0,0207895	-0,534666	0,025033	0,6279808
CG3588-RD	CG3588	0,0234506	0,0213604	11,8038	19,2348	0,0275846	24,6261	-0,535187	0,024977	0,13772387
CG3589-RA	CG3589	4,12272	0	4,72762	19,9807	41,8219	4,44344	-0,111848	0,644522	0,13772387
CG3590-RA	AdSL	9,90425	0,902797	17,2499	15,7855	56,4215	20,5935	-0,473704	0,094263	0,6279808
CG3592-RA	Dsim\GD16624	0,132152	0,0208354	0	0,0240523	0	0	0,024053	0,869957	0,13772387
CG3593-RA	r-l	0,0332429	0,0302799	0,0177016	0,984888	16,0125	0,0184513	0,001169	0,996228	0,13772387
CG3594-RA	CG3594	15,7324	10,6401	30,4723	5,19323	6,80282	22,1721	0,075298	0,802736	0,6279808
CG3595-RA	sqh	109,286	125,89	5,27872	10,9246	5,27151	4,86542	0,188228	0,312375	0,6279808
CG3597-RA	CG3597	0,243922	0,567797	0,75458	0,898743	0,351286	0,6687	0,042235	0,906039	0,6279808
CG3598-RA	CG3598	0,201697	0,0294103	0,159389	0,0225616	0,227093	0,171151	-0,053523	0,780504	0,6279808
CG3599-RA	Btnd	0,695745	0,144853	2,02295	0,900946	2,29619	0,748405	0,042848	0,902471	0,6279808
CG3600-RA	Hr4	11,7912	18,3736	8,72724	16,1165	0,0523305	16,1538	0,273027	0,438109	0,6279808
CG3600-RB	Hr4	0,0438847	0,0399733	0,042132	0,048574	12,9067	2,85276	0,554602	0,114446	0,6279808
CG3600-RC	Hr4	0,0356268	0,0324513	0,0342038	0,0386343	2,79644	10,1221	0,246289	0,483983	0,6279808
CG3603-RA	CG3603	14,3711	0,0210393	18,7461	0,0243006	21,8287	20,6995	-0,221617	0,494196	0,6279808
CG3604-RA	CG3604	31,9908	2,32501	0	4,40416	0,0245699	0,0185174	-0,698699	0,044906	0,6279808
CG3605-RA	Sf3b2	15,3832	30,6438	8,67815	9,87415	38,6729	2,30937	0,095968	0,69924	0,6279808
CG3606-RB	caz	45,8072	278,874	587,868	0,0300119	651,6	694,766	0,056158	0,828152	0,6279808
CG3608-RA	Adck	16,3762	0,116715	7,78647	9,98352	6,17598	7,01245	0,120595	0,586053	0,6279808
CG3609-RA	CG3609	72,3255	64,1341	1,10325	92,1654	0,17285	0,13027	-0,586894	0,065871	0,6279808
CG3610-RA	CG3610	0	197,799	277,527	0,0662219	163,507	170,492	-0,074864	0,550506	0,6279808
CG3611-RA	CG3611	0	61,9513	0	0	4,52062	1,5283	-0,041811	0,731452	0,6279808
CG3612-RA	Dpse\GA17558	420,384	433,719	592,389	548,356	448,631	467,545	-0,124701	0,531876	0,6279808
CG3613-RA	qkr58E-1	14,1707	15,7411	1,68715	42,5676	0,0220181	31,1334	0,145749	0,503358	0,6279808
CG3615-RA	Atg9	15,5864	6,5323	2,52765	7,42406	1,30681	5,19951	-0,16507	0,415583	0,6279808
CG3616-RA	Cyp9c1	13,6899	16,361	33,6291	19,0095	17,3113	67,5184	-0,170934	0,475497	0,13772387
CG3618-RA	CG3618	9,1985	16,6556	4,99815	34,7396	13,1397	9,6618	0,169584	0,489665	0,6279808
CG3619-RA	Dl	12,3149	12,8658	9,35634	0,0317012	4,86922	0,0293409	0,585548	0,033711	0,6279808
CG3619-RB	Dl	0,0189401	0,017252	0,0181837	0,0467476	2,1827	20,0856	0,099243	0,734456	0,6279808
CG3620-RA	norpA	1,2121	9,40375	1,43749	2,32824	5,47926	6,30441	0,174289	0,43266	0,6279808
CG3620-RB	norpA	0,01426	0,792326	0,0136904	0,0146931	1,53581	1,15233	0,145928	0,513214	0,6279808
CG3620-RC	norpA	2,15413	0,0129889	2,67636	1,34757	2,38588	0,0149993	0,173745	0,433098	0,6279808
CG3620-RD	norpA	0,0158392	2,11362	0,0152066	0,0163807	0,88961	1,06374	0,145445	0,513737	0,6279808
CG3621-RA	Dpse\GA17565	171,167	0,942345	1,95391	0,778266	1,46624	5,86241	-0,775137	0,017933	0,6279808
CG3622-RA	stl	0,285551	0,662834	1,1687	0,703132	0,0518441	0,318364	0,198851	0,513936	0,6279808
CG3622-RC	stl	1,18471	1,52463	10,7426	16,3089	2,23629	2,91087	0,129985	0,67638	0,6279808
CG3624-RA	babos	109,019	125,452	42,7191	12,8711	0,0274912	117,175	0,061963	0,752025	0,6279808

gene_id	Symbol	W1_FPKM	W2_FPKM	W3_FPKM	MCM51_FPKM	MCM52_FPKM	MCM53_FPKM	FC	p-value	p-adj
CG3625-RA	CG3625	26,8469	9,48984	36,1129	45,1438	25,1426	41,2481	-0,147463	0,506666	0,6279808
CG3625-RB	CG3625	32,5846	5,1989	41,9894	30,718	36,5319	40,9267	-0,169559	0,432927	0,13772387
CG3625-RC	CG3625	21,0426	5,03107	20,3618	20,0276	27,7517	17,4934	-0,152248	0,491447	0,6279808
CG3626-RA	CG3626	5,47576	1,07476	5,25705	0,0322758	0	0,0329483	0,182752	0,413562	0,6279808
CG3629-RA	Dll	0,030273	0,0275747	0,0290639	0,0324028	0,0438897	0,133855	0,234758	0,371464	0,13772387
CG3629-RB	Dll	0,0313502	0,028556	0,0300981	0,0336436	0,0455705	0,0501033	0,185221	0,503622	0,6279808
CG3629-RC	Dll	5,16081	5,76	4,91706	6,95092	2,82077	270,997	0,185221	0,503622	0,6279808
CG3630-RA	CG3630	7,5875	13,2202	12,3151	8,2373	0,064139	7,47701	0,071353	0,816037	0,6279808
CG3631-RC	CG3631	0,0248157	1,88742	2,72791	0,0257941	0,0315315	0,0263316	0,201743	0,419102	0,6279808
CG3631-RD	CG3631	2,88384	0,022261	0,0234632	6,53647	19,8204	5,27932	0,201743	0,419102	0,6279808
CG3632-RE	CG3632	0,0161292	10,8118	0,0233395	12,6298	7,83785	7,30275	-0,02376	0,91022	0,6279808
CG3632-RF	CG3632	0,0161726	0,0148474	279,394	0,0168757	0,03548	9,14736	-0,021621	0,918299	0,6279808
CG3632-RG	CG3632	11,612	5,1226	7,20478	6,79319	482,068	25,6576	-0,026139	0,90141	0,6279808
CG3632-RH	CG3632	0,0163003	6,0233	8,35856	8,92867	9,17404	0,0267399	-0,026139	0,90141	0,6279808
CG3633-RA	mRpS29	24,1511	0,211181	7,64355	0,268622	0,836136	0,0161985	-0,165981	0,480886	0,6279808
CG3634-RA	CG3634	7,03057	8,98768	7,91588	13,7204	6,16828	7,06519	-0,078261	0,754317	0,6279808
CG3635-RB	CG3635	1,79294	0,827455	2,34101	2,74039	3,10433	3,32979	-0,835676	0,009616	0,6279808
CG3637-RA	Cortactin	24,6125	2,68918	2,69558	7,9313	13,8268	0,0145389	0,205968	0,415145	0,6279808
CG3638-RA	CG3638	4,69673	7,38549	5,82828	13,9177	0,0135526	0,010214	0,213588	0,447913	0,6279808
CG3638-RB	CG3638	5,74286	5,77562	0,00941944	0,0100055	0,0138362	2,12928	0,212429	0,450148	0,6279808
CG3638-RC	CG3638	5,99961	7,94036	3,00859	0,0102149	21,2559	16,7791	0,214338	0,446446	0,6279808
CG3638-RD	CG3638	7,91449	5,60401	14,1527	18,3596	3,85435	6,48212	0,218405	0,437724	0,6279808
CG3639-RA	Pex12	5,09982	3,60176	0,0347409	5,82486	0,0488777	3,19382	-0,420155	0,186434	0,6279808
CG3640-RA	CG3640	0,630354	20,475	0,114645	20,0964	85,6121	19,1763	0,856813	0,016553	0,6279808
CG3642-RA	Clp	3,87122	1,78091	7,79579	3,77614	5,48351	0,787914	-0,096034	0,765306	0,6279808
CG3644-RA	bic	360,717	424,029	16,892	13,0499	0,907349	10,2845	-0,411943	0,083681	0,6279808
CG3644-RB	bic	0,0367974	0,0335176	37,7217	509,686	0,048757	34,1109	-0,411925	0,083697	0,6279808
CG3645-RA	CG3645	8,57447	9,73106	6,74256	11,3814	0,0580107	0,896795	0,127755	0,584737	0,6279808
CG3645-RB	CG3645	0,0391522	0,0356625	19,5943	0,0428279	0,946324	0,00379694	0,127755	0,584737	0,6279808
CG3647-RA	stc	12,4775	1,38884	1,32729	1,33106	0,999703	0,746762	-0,073038	0,741819	0,6279808
CG3647-RB	stc	0,0161528	4,36937	8,10797	9,24156	12,3404	12,9473	-0,073038	0,741819	0,6279808
CG3649-RA	CG3649	0,0464669	0,0423252	0,044611	27,5114	13,4783	20,1014	0,198619	0,578701	0,6279808
CG3650-RA	CG3650	0	0	0,121063	67,458	23,0063	4,03787	0,01562	0,897983	0,6279808
CG3651-RA	CG3651	7,79343	8,95015	8,26518	13,2948	5,75899	6,90161	-0,0529	0,826715	0,6279808
CG3652-RA	CG3652	19,515	17,3753	5,00336	36,6237	0,231754	1,60701	0,10115	0,684809	0,6279808
CG3653-RA	kirre	6,0604	6,38956	80,5606	0,0319472	0,0498184	54,5471	0,205533	0,336546	0,6279808
CG3653-RB	kirre	0,0195221	0,0177821	0,0225139	88,844	61,5618	5,39212	0,255769	0,228419	0,6279808
CG3654-RD	Jarid2	7,88222	7,08294	8,17536	2077,37	1,54351	1,20361	0,34335	0,185808	0,6279808
CG3655-RA	CG3655	0,0139971	0	0,0134381	0,0144135	8,11561	0,0147139	0,124655	0,619793	0,6279808
CG3655-RB	CG3655	11,7652	0,779113	12,3857	17,4199	1,96977	8,16599	0,131486	0,591749	0,6279808
CG3656-RA	Cyp4d1	10,5694	17,1706	38,7854	0,0473523	7,23982	0,0201965	-0,705546	0,021606	0,6279808
CG3656-RB	Cyp4d1	0,0428878	0,0390652	38,9987	26,9419	59,2918	9,25483	-0,596769	0,0452	0,6279808
CG3658-RA	CDC45L	3,55871	10,6438	5,62644	15,423	33,5368	28,5529	0,003681	0,987994	0,6279808
CG3661-RA	RpL23	5080,02	4912,54	0	0,333324	0	0	-0,572432	0,027822	0,13772387
CG3662-RA	CG3662	0,0231277	138,694	0,022204	131,887	7,0708	79,6432	0,340038	0,100976	0,6279808
CG3662-RB	CG3662	109,589	0,0420394	121,52	0,0513767	0,0260622	0,0524473	0,337879	0,104562	0,6279808
CG3663-RA	CG3663	19,3834	12,8691	0,0650634	19,3019	0,433929	0,872304	-0,527162	0,112031	0,6279808
CG3664-RA	Rab5	0,0374368	0,0341	0,0359415	36,4019	0,0571197	0,0430488	0,008978	0,960949	0,6279808
CG3664-RB	Rab5	0,0372259	0,0339079	0,035739	86,5453	0,0567564	0,0427751	0,009495	0,958685	0,6279808
CG3664-RC	Rab5	0,032029	0,0291743	0,0307498	0,0370694	0,0479708	0,0361537	0,007719	0,966381	0,6279808
CG3664-RD	Rab5	0,0375645	0,0342163	21,4582	9,78037	0,0573399	29,9813	0,009277	0,959612	0,6279808
CG3664-RE	Rab5	114,637	126,423	87,3941	0,018408	99,1508	78,2728	0,009397	0,959106	0,6279808
CG3664-RF	Rab5	0,0333717	0,0303972	0,0320388	0,0430237	0,0502108	0,0378419	0,010292	0,95524	0,6279808
CG3665-RA	Fas2	23,5883	31,0893	19,8049	0,0639096	11,6074	13,2386	0,03402	0,908994	0,13772387
CG3665-RB	Fas2	5,65832	0,0212098	9,1433	1,12894	6,71847	5,4019	-0,037593	0,898376	0,6279808
CG3665-RC	Fas2	12,1739	16,2117	10,0003	1,29963	4,60699	6,76975	-0,014059	0,96262	0,6279808
CG3666-RA	Tsf3	5,63481	0,0182734	5,75964	3,52564	0,0283829	2,91567	-0,186747	0,409966	0,6279808
CG3668-RA	fd59A	0,940035	1,1754	1,22246	1,66638	0,627368	0,858717	0,010712	0,970568	0,6279808
CG3669-RB	CAH6	6,42154	2,96408	0,088698	0,226378	0,225219	0,195227	0,894858	0,001856	0,6279808
CG3671-RA	Mvl	3,62714	8,93846	11,1377	8,96468	6,03828	11,3029	-0,071709	0,720283	0,6279808
CG3671-RB	Mvl	10,0042	0,0286103	0,0301553	0,0337124	4,05846	42,8099	-0,03447	0,86389	0,6279808
CG3671-RC	Mvl	0,0314098	31,5492	27,739	35,9104	0,0456637	18,9199	-0,329677	0,139695	0,6279808
CG3672-RA	Cpr67B	39,2042	12,7113	4,45793	8,84617	3,37119	3,87292	-0,523911	0,139731	0,6279808
CG3675-RA	Art2	0,0928032	0,309949	0,148494	0,114194	0,333282	0,187776	0,089339	0,765849	0,6279808
CG3678-RA	CG3678	25,9211	0,0172174	33,456	0,0196896	1,84396	26,1528	0,2242	0,346806	0,13772387
CG3679-RA	CG3679	0,0243105	0,0221437	0,0462367	0,026194	9,50997	16,6007	-0,410507	0,187147	0,6279808
CG3680-RA	HIPP1	8,00714	12,9066	0,0927438	17,9443	7,4034	9,58746	-0,150614	0,596293	0,6279808
CG3682-RA	PIP5K59B	0,0198804	0,0181085	0,0190864	0,0207564	3,01588	45,6236	0,194599	0,396653	0,6279808
CG3682-RD	PIP5K59B	0,0159964	0,0145706	0,0153575	0,0165494	0,0224162	0,0152061	0,191722	0,407521	0,6279808
CG3682-RE	PIP5K59B	8,292	11,4716	8,33544	10,1962	3,445	1,14477	0,191639	0,404005	0,13772387
CG3683-RA	Dpse\GA17610	62,7089	0,0980765	0,103373	0,141849	154,147	3,20893	-0,392371	0,221557	0,6279808
CG3683-RB	Dpse\GA17610	0,106288	1,13227	0,71605	1,23337	0,188882	7,1832	-0,392371	0,221557	0,6279808
CG3683-RC	Dpse\GA17610	0,107674	0,335319	1,26818	0,0478683	0,192135	22,2999	-0,392371	0,221557	0,6279808
CG3687-RA	CG3687	0	0,138254	0,14572	0	1,45679	0	0,019745	0,891952	0,6279808
CG3688-RA	Rnmt	8,50611	7,49364	0,102649	8,41067	4,36737	29,8286	0,451371	0,05957	0,6279808
CG3689-RB	Cpsf5	0,0300072	0,0273326	0,0288087	1,44836	0,044636	0,84263	-0,133807	0,531776	0,6279808
CG3689-RC	Cpsf5	15,8319	16,2462	25,1493	54,6521	16,739	42,7646	-0,105615	0,632944	0,6279808
CG3690-RA	CG3690	0,381836	0,281245	1,23941	0,652226	0	70,271	-0,04677	0,89603	0,6279808
CG3691-RA	Pof	0,0344325	0,0256914	22,4053	0,0300392	114,269	6,62346	-0,01342	0,959645	0,6279808
CG3691-RB	Pof	0,0342362	0,0313635	27,903	0,0383868	11,2456	138,535	-0,01342	0,959645	0,6279808
CG3692-RA	CalpC	12,4159	15,9862	50,2767	27,7177	8,49374	27,8032	-0,10824	0,702219	0,13772387
CG3694-RA	Ggamma30A	54,8317	0	0,0495084	0,601253	0,282852	5,23148	-0,409713	0,104578	0,13772387
CG3694-RB	Ggamma30A	0,0447163	3,89659	7,24278	7,56156	4,38805	0,493794	-0,411441	0,104574	0,6279808
CG3694-RC	Ggamma30A	0,0515546	54,827	0	72,6401	94,5152	80,3796	-0,410159	0,104133	0,6279808
CG3695-RA	MED23	0,0142836	0,0130105	0,0137131	0,0148958	0,567617	0,0211889	-0,173294	0,577867	0,6279808
CG3695-RB	MED23	1,37714	1,08191	1,83446	2,74656	0,334542	0,0168942	-0,25823	0,333245	0,6279808
CG3696-RA	kis	19,6519	0	0,00343837	30,8464	0,0414826	74,5004	0,3304	0,246428	0,6279808
CG3696-RB	kis	1,17291	6,42795	0,00355096	4,22124	0,0392912	0,0340319	0,386937	0,175519	0,6279808
CG3696-RC	kis	0,00369869	43,7556	0,068457	4,03994	111,871	18,7503	0,327945	0,250014	0,6279808
CG3697-RA	mei-9	0,980327	0,943022	1,24903	0,0245085	0,490547	0,759863	0,196104	0,50293	0,6279808
CG3698-RA	CG3698	0	0,793624	0	2,37857	85,5418	5,11977	-0,070526	0,562659	0,6279808
CG3699-RA	CG3699	101,02	90,8223	1,16713	0,314248	0,66336	14,6691	-0,911125	0,005357	0,6279808
CG3700-RA	CG3700	6,60956	4,36618	9,00385	10,8391	8,07453	5,90045	-0,288341	0,267753	0,6279808
CG3701-RA	CG45063	0,0640891	3,48804	3,11625	6,10806	0,732808	2,56261	0,292903	0,399436	0,6279808
CG3702-RA	CG3702	34,2931	26,6126	43,822	40,9809	25,7241	32,2136	-0,023728	0,915302	0,6279808
CG3703-RA	CG3703	7,05354	0,118013	0	0,625342	0	0,0354487	0,199565	0,394921	0,6279808
CG3704-RA	CG3704	8,7839	10,0311	9,84588	11,5187	7,93463	10,0105	0,193064	0,43087	0,6279808
CG3705-RA	aay	33,7404	42,91	47,1893	41,7839	35,0756	29,4389	-0,144848	0,475978	0,6279808
CG3706-RA	CG3706	0,815986	0,0148989	1,60323	0,822521	0,977437	23,2161	-0,161481	0,643426	0,6279808
CG3707-RA	wapl	2,14364	4,43186	1,77481	37,7729	30,4806	46,8826	0,158958	0,561663	0,6279808
CG3707-RB	wapl	5,86841	2,71838	6,69158	4,85717	0,0267978	41,7133	0,212642	0,435424	0,6279808
CG3708-RA	CG3708	0,0328954	0,71527	1,44472	1,46852	0,0479966	1,58674	0,207422	0,515017	0,6279808
CG3709-RA	CG3709	2,61382	0,02149	5,05575	1,74947	3,59769	3,72579	-0,18066	0,58663	0,6279808
CG3710-RA	TfIIS	17,817	15,3103	20,418	20,5737	0,0318012	21,0462	-0,081482	0,735523	0,6279808

gene_id	Symbol	W1_FPKM	W2_FPKM	W3_FPKM	MCM51_FPKM	MCM52_FPKM	MCM53_FPKM	FC	p-value	p-adj
CG3711-RA	Lztr1	0,817838	0,535836	0,296896	0,736515	3,66105	0,836042	0,04782	0,840989	0,6279808
CG3711-RB	Lztr1	4,29488	2,04105	1209,82	3,76663	0,0817673	3,35886	0,04782	0,840989	0,6279808
CG3712-RA	mRpL33	23,7169	20,754	30,8235	29,8544	0	74,1573	-0,49937	0,081326	0,6279808
CG3713-RA	CG3713	0,374721	14,4372	0,184053	0,0334387	0,0128742	0,309007	0,23963	0,402025	0,6279808
CG3714-RA	Naprt	20,5509	20,1129	20,2366	16,4295	20,5654	19,5087	-0,151478	0,500611	0,6279808
CG3714-RB	Naprt	0,0232607	0,0211874	0,0223316	0,0244812	0,0331599	0,0249913	-0,163157	0,465704	0,6279808
CG3714-RC	Naprt	0,0229776	0,0209296	0,0220598	0,0241669	0,0327343	0,0246705	-0,164025	0,46201	0,6279808
CG3714-RD	Naprt	0,0237626	0,0216446	0,0228135	0,0250394	0,033916	0,0255612	-0,162868	0,464838	0,6279808
CG3714-RE	Naprt	8,56599	7,36841	9,20932	10,3985	6,9869	6,72413	-0,161964	0,468476	0,6279808
CG3714-RF	Naprt	0,024127	3,36241	0,0231634	4,41236	4,96334	3,54526	-0,163157	0,465704	0,6279808
CG3715-RA	Shc	13,9666	20,9611	17,2118	23,5333	11,7552	12,503	0,053953	0,822076	0,6279808
CG3717-RA	bcn92	30,2667	0,0318334	0,0411748	17,351	2,30869	152,545	-0,065007	0,787991	0,6279808
CG3719-RA	CG3719	57,2371	72,8694	0,00338191	79,9401	0,792812	0	-0,122017	0,58033	0,6279808
CG3722-RA	shg	12,9918	14,5797	11,975	4,52256	27,4142	8,43707	-0,08367	0,794665	0,6279808
CG3723-RB	Dhc93AB	0,13008	0,315218	0,190862	0,540757	0	0,223553	-0,530976	0,095527	0,6279808
CG3724-RA	Pgd	22,0472	0,0331484	17,4564	7,06214	2,10922	0,0201965	-0,673866	0,035811	0,6279808
CG3725-RA	SERCA	0,0163933	0,0184019	0,0157385	162,885	0,0280705	0,0204233	-0,250003	0,209407	0,6279808
CG3725-RB	SERCA	109,663	0,0187225	122,898	156,07	31,9933	0,0218924	-0,243144	0,223334	0,6279808
CG3725-RC	SERCA	0,0170489	12,9558	0,016368	17,8227	0,0291144	0,0211556	-0,245541	0,218234	0,6279808
CG3725-RD	SERCA	0,0191932	7,16958	0,0184266	167,761	15,7994	28,3827	-0,24321	0,223227	0,6279808
CG3725-RE	SERCA	0,0205098	0,00472322	0,0196907	2,12263	11,2129	0,0219423	-0,243018	0,223616	0,6279808
CG3725-RF	SERCA	0,0198505	0,0365505	0,0190577	8,86092	10,5864	10,4112	-0,24569	0,217838	0,6279808
CG3725-RG	SERCA	0,0202026	29,9895	0,0193957	0,0342914	0,00708703	0,00534122	-0,243074	0,223433	0,6279808
CG3725-RH	SERCA	0,0205545	86,5531	0,0197335	31,3929	223,399	178,542	-0,081046	0,687102	0,6279808
CG3726-RA	CG3726	5,19004	4,62332	6,91439	6,30878	5,5901	5,00086	-0,471818	0,082533	0,6279808
CG3727-RA	dock	6,81228	23,1907	0,0177512	3,82861	2,14272	0,0196421	0,240121	0,381655	0,13772387
CG3727-RB	dock	0,0184897	5,48417	12,2977	14,3806	0,0336221	8,19775	0,241566	0,3796	0,6279808
CG3727-RC	dock	18,7379	4,08959	3,90226	0,0192411	75,5175	3,2447	0,243448	0,376331	0,6279808
CG3730-RA	csul	0,0341183	3,64877	5,14976	10,3029	0	0,718276	0,457872	0,075535	0,6279808
CG3730-RB	csul	3,47285	0,0184508	0	0,0308262	0,0499299	0,0915032	0,457872	0,075535	0,6279808
CG3731-RA	Dpse\GA17647	0,0370173	0,0337179	2,74749	41,1527	2,81446	3,04696	-0,527322	0,057063	0,6279808
CG3731-RB	Dpse\GA17647	174,717	204,504	3,97205	217,38	0,241863	2,8598	-0,527322	0,057063	0,6279808
CG3732-RA	CG3732	15,1572	5,2507	2,45425	2,47402	41,0813	1,20599	0,239383	0,282421	0,6279808
CG3733-RA	Chd1	11,3016	0,0175624	12,8881	17,6887	2,29919	9,16039	0,002487	0,991071	0,6279808
CG3734-RA	CG3734	31,8233	26,5529	35,897	21,376	40,4155	28,584	-0,086917	0,769229	0,6279808
CG3735-RA	CG3735	6,06819	4,93988	11,3561	7,16722	4,7397	5,49136	0,226921	0,406871	0,6279808
CG3736-RA	okr	0,0233594	6,48974	1,31194	9,54907	1,66881	0,0209253	0,251248	0,284479	0,6279808
CG3738-RA	Cks30A	2,94827	0,0469595	0,0429303	0,0581814	0,0788071	0,0593938	-0,209314	0,475939	0,6279808
CG3739-RA	CG3739	0	0	0	0,0635807	0	0,0649056	-0,070526	0,562659	0,6279808
CG3740-RA	CG3740	19,9306	21,8523	20,1704	7,95555	3,49447	3,98839	-0,242086	0,333208	0,6279808
CG3743-RA	MTF-1	0,0207351	0,018887	0,0199069	30,5565	0,109574	1,03613	0,196558	0,363062	0,6279808
CG3743-RB	MTF-1	1,4964	3,5585	3,54819	15,3508	13,366	0,0221446	0,202186	0,350113	0,6279808
CG3743-RC	MTF-1	0,0174496	0,0158943	0,0167527	11,1752	5,21661	2,30221	0,178426	0,409613	0,6279808
CG3743-RD	MTF-1	1,62055	1,88146	0,0161225	21,0481	21,3411	0,0184919	0,181825	0,402134	0,6279808
CG3743-RE	MTF-1	8,57129	9,25554	8,26338	0,0808963	0,0293827	1,39233	0,242074	0,264397	0,6279808
CG3744-RA	CG3744	2,43701	4,81346	2,42998	5,37711	5,25382	7,99389	0,128723	0,669098	0,6279808
CG3744-RB	CG3744	2,83878	3,63383	2,74153	4,26052	1,56864	1,53602	0,137923	0,648966	0,6279808
CG3744-RC	CG3744	0,0179229	0,0163254	0,0172071	0,0186265	1,88466	1,89488	0,137858	0,648957	0,6279808
CG3746-RA	CG3746	46,2065	0,29146	52,8032	30,7764	68,3572	127,588	-0,653338	0,047422	0,6279808
CG3747-RA	Eaat1	14,7899	0,0238772	0,0251667	0,0277846	0,0376344	0,0283635	-0,310716	0,298175	0,6279808
CG3747-RB	Eaat1	14,6909	0	0,115761	0	0	0	-0,293631	0,32524	0,6279808
CG3747-RC	Eaat1	0,0262136	0,853244	0,276714	0,629704	10,0825	0,227459	-0,291723	0,329077	0,6279808
CG3748-RA	CG3748	0	0	0	0,469833	0	0	NA	NA	0,6279808
CG3748-RB	CG3748	0	0,362056	0	0	0	0	NA	NA	0,6279808
CG3751-RA	RpS24	1245,07	99,2804	24,4964	11,6022	0,180115	2,66042	-0,229833	0,372746	0,6279808
CG3752-RA	Aldh	62,8962	6,72782	7,59957	2,33969	3,8664	4,92127	-0,469798	0,151343	0,6279808
CG3753-RA	Marcal1	0,976965	0,68935	1,54852	0,0205685	0,0271483	15,2796	0,268827	0,408713	0,6279808
CG3756-RA	CG3756	10,5698	0,0602667	85,5139	6,77392	12,3551	15,3833	-0,240932	0,432006	0,6279808
CG3757-RA	y	24,5596	0,393805	0,0387124	0,0206071	0,0279125	60,931	-0,00642	0,981907	0,6279808
CG3758-RA	esg	17,8119	0,0369292	0	25,0222	3,65335	5,5172	0,371722	0,184012	0,6279808
CG3759-RA	Mco1	12,4167	2,24545	2,67638	2,4741	0,187365	4,6225	0,282213	0,340628	0,6279808
CG3760-RA	CG3760	12,3478	11,1101	23,8539	55,6704	0,0633648	4,8084	-0,294817	0,341611	0,6279808
CG3760-RB	CG3760	34,599	22,0012	57,6872	0,0467808	0,0615301	30,7835	-0,257668	0,401086	0,6279808
CG3762-RA	Vha68-2	30,5272	5,91207	10,409	7,43667	0	0,0128453	-0,150011	0,494905	0,6279808
CG3762-RB	Vha68-2	199,222	5,3538	6,81718	1,30878	0	0,0128453	-0,150032	0,494844	0,6279808
CG3762-RC	Vha68-2	0,0266471	1,88841	1,46799	10,3402	2,63596	0,0128453	-0,150111	0,494614	0,6279808
CG3763-RA	Fbp2	0,324255	6,25689	18,6652	18,8469	41,2191	19,1806	0,358839	0,286006	0,13772387
CG3764-RA	CG3764	8,66156	9,46628	7,35445	11,3499	2,62283	5,68249	0,021512	0,927968	0,6279808
CG3766-RA	scat	6,49424	1,43301	141,279	8,57152	163,203	137,531	0,160036	0,486542	0,6279808
CG3767-RA	Jhl-26	36,615	49,0356	24,017	0,661961	0,89663	0,675755	-1,996281	4,41E-16	0,6279808
CG3769-RA	CG3769	1,25347	22,2373	2,19695	68,7705	1,78776	7,02608	-0,108203	0,720331	0,6279808
CG3770-RA	CG3770	11,0319	16,1769	0,0464851	17,7436	42,7534	11,5195	0,173151	0,458859	0,6279808
CG3771-RA	a6	5,47657	5,51794	0,0167748	2,19335	0,0221272	0,0178019	0,47062	0,064231	0,6279808
CG3772-RA	cry	0,639788	0,485636	0,477738	0,649154	0,331123	8,36736	-0,030749	0,929521	0,6279808
CG3773-RA	Gdn1	5,26653	4,5993	6,77632	24,919	4,02522	5,3124	0,354393	0,135156	0,6279808
CG3774-RA	Efr	27,24	21,2482	35,2136	26,6131	51,0461	35,0796	-0,469102	0,12173	0,6279808
CG3774-RB	Efr	0,0352091	0,0320709	0,0338029	0,0381423	0,051664	0,0389371	-0,468544	0,121942	0,6279808
CG3775-RA	Nep6	1,56888	2,93776	0,00501529	0,681983	9,36517	0,738437	-0,37981	0,232785	0,6279808
CG3776-RA	CG3776	0,0674244	714,922	0,0543743	44,8393	33,3321	746,887	0,091415	0,677533	0,6279808
CG3777-RA	Dsim\GD16500	108,162	0,0181248	0,0409357	2,27584	0,114641	0,0480284	0,576907	0,030979	0,6279808
CG3777-RB	Dsim\GD16500	0,0198984	0,017984	0,0404399	48,072	70,7878	0,0473855	0,576969	0,03096	0,6279808
CG3777-RC	Dsim\GD16500	0,0197438	1,72275	39,7336	47,5489	0,0561141	22,3357	0,576987	0,030971	0,6279808
CG3779-RA	numb	11,2029	45,6719	7,18429	8,7917	5,13304	5,0929	-0,16706	0,495857	0,6279808
CG3779-RB	numb	10,9076	12,6586	11,1664	25,7616	10,9159	12,7157	-0,104327	0,674017	0,6279808
CG3780-RA	Spx	8,51493	9,49333	13,141	14,6192	13,2018	12,9754	0,063962	0,790848	0,6279808
CG3781-RA	CG3781	10,0501	11,6137	0,0400316	0,0459009	0,062173	0,0468574	-0,175173	0,428383	0,6279808
CG3782-RA	mRpL28	46,1949	8,01411	14,2896	9,93694	0,10506	0,0791796	-0,117884	0,64443	0,6279808
CG3788-RA	CG3788	0,0528747	0,03878	8,57783	2,60313	0,0661351	0,0523656	0,640228	0,023702	0,6279808
CG3790-RA	Balat	0,135471	0,16967	0,471468	0,282632	0,382827	0,153915	0,065955	0,851664	0,6279808
CG3792-RA	CG3792	27,7834	10,3731	41,4987	0,0370694	0,0502108	38,9394	-0,178423	0,546081	0,6279808
CG3793-RA	CG3793	12,0462	4,40787	13,2344	6,09311	0	4,32852	-0,148147	0,478843	0,6279808
CG3795-RA	CG3795	0	0,847341	0	20,5787	25,8446	27,3195	NA	NA	0,6279808
CG3796-RA	ac	0,324255	0,188344	8,20907	1,9597	1,74768	0,042291	-0,001443	0,996632	0,6279808
CG3797-RA	Ugt316A1	4,3995	3,8879	6,04378	5,15309	6,75303	4,89574	-0,103806	0,65743	0,6279808
CG3798-RA	Nmda1	0,0390596	0,0355782	0,0374995	0,0427169	14,4547	16,7378	0,122622	0,507677	0,6279808
CG3798-RB	Nmda1	0,0377648	0,0343988	0,0362565	0,0411689	20,9705	22,324	0,122198	0,509572	0,6279808
CG3798-RC	Nmda1	0,0393152	0,035811	0,037745	0,0430237	8,09236	0,043607	0,114079	0,537683	0,6279808
CG3798-RD	Nmda1	0,0315749	9,43349	0,0303138	6,90324	0,0181458	0,0420268	0,118807	0,521176	0,6279808
CG3798-RE	Nmda1	10,6665	0,0359179	11,9441	15,1477	30,2353	0,0439202	0,115424	0,533075	0,6279808
CG3798-RF	Nmda1	44,4161	49,223	46,5312	41,8721	4,93449	0,0346097	0,116248	0,53298	0,6279808
CG3799-RA	Exn	27,1351	0,529811	0,0183043	0,0182146	0,0246718	0,310565	0,33212	0,230015	0,6279808
CG3799-RB	Exn	0,0190658	0,0228527	0,0168416	0,795547	7,67755	17,8531	0,332517	0,229481	0,6279808
CG3799-RC	Exn	0,0175423	8,44132	0,0240869	0,0339382	20,8827	0,0202817	0,332517	0,229481	0,6279808
CG3800-RA	CNBP	202,823	19,6214	0,0310255	4,25737	13,4872	9,01256	0,341453	0,194711	0,6279808

gene_id	Symbol	W1_FPKM	W2_FPKM	W3_FPKM	MCM51_FPKM	MCM52_FPKM	MCM53_FPKM	FC	p-value	p-adj
CG3801-RA	Acp76A	0	0,283655	0	0,102473	0	0	0,109454	0,496421	0,6279808
CG3803-RA	CG3803	13,6919	10,6239	20,4478	13,9935	2,54082	2,01435	0,017001	0,948898	0,6279808
CG3806-RA	eIF2Bepsilon	7,18859	8,72206	7,25145	9,11609	7,2176	6,63022	-0,048716	0,847276	0,6279808
CG3806-RB	eIF2Bepsilon	0,0293453	4,15647	2249,39	6,23881	5,12977	9,68037	-0,027309	0,913572	0,6279808
CG3808-RA	CG3808	0,0267658	1,989	5,96251	0,0223853	6,27097	0,0242175	-0,090154	0,726029	0,6279808
CG3809-RA	CG3809	0	0	0	0	58,0088	0	NA	NA	0,6279808
CG3810-RA	Edem1	2,96245	2,60633	9,13397	2,98409	4,55664	6,12677	-0,12772	0,598403	0,6279808
CG3810-RB	Edem1	0,0179994	0,0163951	5,34724	0,0189971	4,9512	4,09112	-0,128963	0,592099	0,6279808
CG3810-RC	Edem1	3,48631	3,99749	6,56242	6,98029	4,31306	5,69369	-0,057989	0,809805	0,6279808
CG3811-RA	Oatp30B	0,0125128	0,0113975	0,012013	9,9741	0,526648	0,447626	0,212057	0,375828	0,6279808
CG3811-RB	Oatp30B	4,5585	4,59747	3,84845	12,7759	0,231798	0,944618	0,212072	0,375834	0,6279808
CG3811-RC	Oatp30B	0,0120981	0,0110198	4,2859	0,85618	3,40938	0,0247159	0,220028	0,357777	0,6279808
CG3811-RD	Oatp30B	22,9332	25,9112	13,296	0,084855	0,0911652	2,18304	0,240216	0,318394	0,6279808
CG3812-RA	Agpat1	3,82206	2,77159	0,0338932	0,298407	3,86101	0,50443	-0,580482	0,016625	0,6279808
CG3812-RB	Agpat1	0,0353032	0,0321566	53,8955	45,2186	0	6,10032	-0,577889	0,016862	0,6279808
CG3814-RA	Lfg	51,2551	60,8567	73,6978	60,7757	56,4376	65,7714	0,019499	0,931456	0,6279808
CG3814-RB	Lfg	11,2532	14,0648	26,4213	21,4377	15,711	14,4996	0,022163	0,922331	0,6279808
CG3815-RA	CG3815	5,25255	6,67572	5,4352	8,15277	4,93813	5,22212	-0,034494	0,894791	0,6279808
CG3817-RA	CG3817	14,8616	11,7121	0,00867729	15,3642	0,0124625	0,00939252	0,266173	0,37523	0,6279808
CG3818-RA	Cpr30B	0,429064	3,18244	0,0116469	3,16222	0,0168476	1,99794	0,351286	0,18021	0,6279808
CG3819-RA	CG3819	0,6712	0,271722	11,0979	5,38761	1,43545	4,17152	0,029443	NA	0,6279808
CG3820-RA	Nup214	4,22513	5,00141	6,15307	6,91398	2,48564	3,37152	0,156291	0,555627	0,6279808
CG3821-RA	AspRS	47,406	73,119	60,3515	72,5829	40,7634	47,0659	0,101215	0,656337	0,6279808
CG3822-RA	KaiR1D	5,97612	0	0	0	0	0	-0,039386	0,890762	0,6279808
CG3823-RA	CG3823	25,7889	6,67443	15,0027	12,7433	2,48303	22,2743	-0,164645	0,564067	0,6279808
CG3825-RA	PPP1R15	52,7423	60,0563	58,0654	64,2641	41,1514	42,1843	0,161585	0,381802	0,6279808
CG3827-RA	sc	0,436524	0,549648	0,0329941	1,8616	2,69288	0,0451586	-0,238733	0,504203	0,6279808
CG3829-RA	CG3829	17,196	0,0614148	742,335	21,9279	17,5806	44,0028	-0,233354	0,225303	0,6279808
CG3830-RA	vg	2,79645	4,19539	2,49358	4,26181	1,00555	1,49299	0,354195	0,27716	0,6279808
CG3831-RA	CG3831	6,41633	0,0573751	22,1618	0,0525653	0	12,172	-0,051496	0,83708	0,6279808
CG3832-RA	Phm	26,5675	0,118633	18,573	12,8266	10,4587	11,3623	0,027492	0,891545	0,6279808
CG3832-RB	Phm	0,0470402	6,43752	13,6613	20,8107	19,7665	15,8628	0,004614	0,981869	0,6279808
CG3835-RA	D2hgdh	21,774	16,0788	33,2649	26,0392	0,12387	30,5212	-0,210904	0,398236	0,6279808
CG3835-RB	D2hgdh	0,036392	31,4686	0,0349385	0,0395385	0,0476889	0,0403624	-0,207911	0,404766	0,6279808
CG3835-RC	D2hgdh	0,0349484	23,388	0,0335526	0,0378357	7,39229	0,0386241	-0,209584	0,401442	0,6279808
CG3836-RA	stwl	2,09837	3,79991	3,10469	3,1898	0,417498	1,25698	0,202858	0,398833	0,6279808
CG3837-RA	Sdr	2,70903	0	294,65	0	23,1958	274,247	-0,415832	0,057977	0,6279808
CG3838-RA	brwl	16,3138	14,8864	16,6894	10,7185	12,5449	12,736	0,22786	0,349321	0,6279808
CG3838-RB	brwl	0,0216998	0,0197657	0,0208331	0,0231807	0,0313984	0,0236638	0,252531	0,296246	0,6279808
CG3839-RA	l(1)sc	0,935321	8,86181	2,99691	0,574957	0,778783	0,0455254	-0,143075	0,687444	0,6279808
CG3841-RA	CG3841	1,775	0,0614775	0,0647975	0,0128406	5,47046	5,03708	-0,164612	0,642695	0,6279808
CG3842-RA	CG3842	9,61438	19,5859	34,6172	38,5421	9,36835	15,8991	0,033238	0,896472	0,6279808
CG3842-RB	CG3842	20,7769	30,1355	28,5288	36,6794	16,1287	19,4157	0,035296	0,890317	0,6279808
CG3843-RA	RpL10Aa	0	2,20783	0,0489104	3,80961	5,21753	0,0614559	NA	NA	0,6279808
CG3845-RA	NAT1	10,457	9,38437	0,0125179	16,8562	2,6552	4,40654	0,154414	0,59363	0,6279808
CG3845-RB	NAT1	47,8679	5,76565	61,5869	58,7157	5,13379	1,06091	0,154327	0,59385	0,6279808
CG3847-RA	CG3847	20,444	19,2114	10,3902	24,2536	15,4534	12,1931	0,08045	0,746964	0,13772387
CG3848-RC	trr	0,00816592	0,0508904	0,0855325	0,0637546	0,150629	1,39567	-0,006134	0,980938	0,13772387
CG3848-RD	trr	3,57593	0,0581137	11,6178	0,0743262	6,49459	6,62517	-0,006134	0,980938	0,6279808
CG3849-RA	Lasp	28,268	41,8013	21,9341	34,2681	15,9824	17,9356	-0,072347	0,805082	0,6279808
CG3849-RB	Lasp	0,0248717	0,0226549	0,0238783	0,0262776	0,0355932	0,0268252	0,294418	0,288631	0,6279808
CG3850-RA	sug	28,3112	8,11373	502,036	9,18436	0,0557635	6,98185	0,317914	0,216621	0,6279808
CG3851-RA	odd	8,65111	9,29407	8,46818	8,75149	0,0307071	6,32	0,426842	0,065921	0,6279808
CG3853-RA	Glut3	0,096433	0	0,031301	0	0	10,731	0,046581	0,885198	0,6279808
CG3856-RA	Oamb	2,89558	1,02096	2,4258	1,73887	1,90659	1,64281	0,104932	0,653519	0,6279808
CG3856-RB	Oamb	0,0164832	0,0179465	0,74377	1,51604	0,783188	0,517558	-0,398308	0,155988	0,6279808
CG3856-RC	Oamb	0,620631	86,7231	0,633674	0,603847	0,947809	0,579557	0,146987	0,527688	0,6279808
CG3857-RA	CG3857	2,08811	3,33803	3,87866	0,0453013	2,96292	4,06592	-0,121799	0,652084	0,6279808
CG3858-RA	gcm2	1,1751	1,49086	5,82107	1,99399	8,62535	8,18125	0,096535	0,772214	0,6279808
CG3858-RB	gcm2	0,0230176	0,020966	2,61232	0,0242113	0,0160594	0,0121034	0,115371	0,727998	0,6279808
CG3860-RA	CG3860	17,4306	13,8111	23,1475	9,658	14,9185	16,0179	0,092936	0,714414	0,6279808
CG3861-RA	kdn	0,0269404	0,0245392	0,0175062	157,997	0,0387453	0,0193594	-0,10779	0,620221	0,6279808
CG3861-RB	kdn	121,823	120,713	9,58935	0,0189642	135,624	3,39038	-0,095391	0,659993	0,6279808
CG3862-RA	CG3862	0,0461531	7,59126	0,0443097	6,73278	5,06133	2,24691	0,142302	0,546851	0,6279808
CG3868-RA	CG3868	6,4396	2,58982	25,1328	3,01955	1,65908	4,88249	0,883639	0,012326	0,6279808
CG3869-RA	Marf	0,0182345	21,4101	4,69854	0,0286048	0,0275081	0,0292008	0,013054	0,944835	0,6279808
CG3869-RB	Marf	0,0194703	10,8634	27,2193	156,646	22,1689	132,447	0,017298	0,926976	0,6279808
CG3869-RC	Marf	27,4831	9,918	0,0258644	27,0265	8,38127	10,6368	0,013054	0,944835	0,6279808
CG3870-RA	RabX1	12,1632	14,4457	19,0259	22,9778	44,0978	9,62091	-0,593604	0,0091	0,6279808
CG3871-RA	Six4	0,553573	7,53818	1,03982	8,28433	27,7071	33,551	-0,020723	0,941305	0,6279808
CG3871-RB	Six4	0,887586	10,3348	1,13618	12,6859	3,67609	4,4147	0,03446	0,905094	0,6279808
CG3874-RA	frc	22,7809	159,285	156,466	162,39	0,783336	221,312	-0,084041	0,656737	0,6279808
CG3875-RA	nsr	0,293315	48,7224	59,5769	2,33917	3,88809	3,54319	-0,16056	0,651847	0,6279808
CG3876-RA	PGAP2	3,79843	4,24825	4,83798	1,21552	2,82228	9,76264	0,389413	0,118278	0,6279808
CG3879-RA	Mdr49	2,2702	10,2851	18,5596	0,29546	553,407	581,561	-0,371294	0,25228	0,6279808
CG3880-RA	Dpse\GA17747	7,61431	15,9659	0,0270789	12,6689	37,1986	23,8739	0,447954	0,151661	0,6279808
CG3881-RA	GlcAT-S	9,22412	10,2116	6,88994	6,5606	3,76223	5,04014	0,449739	0,048077	0,6279808
CG3881-RB	GlcAT-S	4,53859	4,04391	4,73739	5,61976	4,25715	3,24771	0,357063	0,102141	0,6279808
CG3883-RA	Saf6	2,29036	3,13695	6,77134	3,81626	6,59645	6,84122	-0,352559	0,17197	0,6279808
CG3884-RA	CG44250	11,7229	1,2753	10,994	10,4978	4,50787	18,9383	-0,528609	0,084209	0,6279808
CG3884-RB	CG44250	48,9369	1,10963	6,38602	9,19525	1,5444	111,291	-0,621743	0,069252	0,6279808
CG3885-RA	Sec3	5,40544	6,08443	6,88201	8,14621	4,6195	4,97859	-0,049224	0,811524	0,6279808
CG3886-RA	Psc	3,98829	3,44266	4,35113	0,871635	1,871	2,85724	0,117695	0,670112	0,6279808
CG3887-RA	SelT	93,7998	76,7139	83,5419	106,715	145,835	127,65	-0,452126	0,089035	0,6279808
CG3889-RA	CSN1b	9,5815	16,4241	27,0835	19,6791	9,16986	14,1561	0,260949	0,322746	0,6279808
CG3891-RA	Nf-YA	10,6876	12,3088	12,3831	4,50656	40,8595	12,1179	-0,211636	0,274271	0,13772387
CG3893-RA	arx	3,85902	5,3802	5,59514	3,86062	3,61084	4,30226	0,360409	0,196991	0,6279808
CG3894-RB	CG3894	0,0937247	0,0853709	0,0899813	179,804	0,177606	0	-0,282285	0,33768	0,6279808
CG3894-RC	CG3894	0,0427676	10,7712	0,0410594	2,56341	0,0664799	5,27711	0,092828	0,677582	0,6279808
CG3895-RA	ph-d	2,61512	30,5659	3,5052	5,25842	13,597	1,50322	-0,09963	0,761064	0,6279808
CG3895-RB	ph-d	1,11006	0,0253417	0,0135761	2,57027	10,4317	1,29407	-0,100839	0,757876	0,6279808
CG3897-RA	blot	7,05604	2,60883	9,94273	6,68193	7,39618	7,41177	0,144436	0,487811	0,6279808
CG3897-RB	blot	11,5377	13,8313	0,0160288	5,57921	3,4023	0,0176616	0,080198	0,698552	0,6279808
CG3897-RC	blot	0,0166957	0,0152076	2,79425	0,0173011	0,0234345	3,95892	0,135581	0,515143	0,6279808
CG3897-RD	blot	3,18768	2,87831	9,62918	7,42893	4,32407	7,38396	0,083811	0,685648	0,6279808
CG3902-RA	CG3902	44,2734	0,141283	85,5314	29,3124	0,155215	0,0547972	0,1079	0,739729	0,6279808
CG3903-RA	Gli	0,0167083	0,0908496	13,2225	0,0171838	0,410611	0,148468	0,0293	0,911441	0,6279808
CG3903-RB	Gli	11,2378	0,101506	0,0153575	0,102923	0,0232756	0,0238907	0,054599	0,836718	0,6279808
CG3903-RC	Gli	7,72857	3,96457	0	0,184629	0,0255402	0	0,028672	0,913371	0,13772387
CG3903-RD	Gli	11,0332	17,9334	7,7887	12,31	0,243773	0	0,031041	0,906208	0,6279808
CG3903-RE	Gli	0,0159964	4,44731	0,0159242	0,0173148	0,0234529	0,0176756	0,027773	0,916039	0,6279808
CG3905-RA	Su(z)2	1,20983	1,57721	3,31517	79,7694	1,74265	1,43961	0,309678	0,198357	0,6279808
CG3905-RB	Su(z)2	4,36792	0,236066	31,2529	3,95691	7,54623	4,28906	0,300679	0,211965	0,6279808
CG3906-RA	CG3906	0	0	0,0306458	0,133244	0,047791	7,51783	-0,041811	0,731452	0,13772387

gene_id	Symbol	W1_FPKM	W2_FPKM	W3_FPKM	MCM51_FPKM	MCM52_FPKM	MCM53_FPKM	FC	p-value	p-adj
CG3907-RA	CG3907	0,040127	0,0174825	8,1905	32,5936	0,0239501	0,0173295	0,161304	0,393922	0,6279808
CG3907-RB	CG3907	32,4818	0,0186818	0,0049783	0,0440888	0,0270989	79,1037	0,16103	0,394833	0,6279808
CG3909-RA	CG3909	6,26632	4,12677	14,9093	12,0262	1,1142	9,81208	-0,216071	0,503624	0,6279808
CG3910-RA	mtTFB2	2,74771	2,16512	4,98282	3,15154	55,3898	56,4693	0,01793	0,951744	0,6279808
CG3911-RA	Bet3	17,1932	16,0001	28,2092	0,0216926	2,41044	23,2423	-0,166404	0,479761	0,6279808
CG3915-RB	Drl-2	3,05789	4,9853	1,96888	75,9812	0	1,52934	0,476275	0,109505	0,6279808
CG3916-RA	CG3916	0	0,0632654	10,5841	0	0	20,6356	-0,944303	0,005695	0,6279808
CG3917-RA	Grip84	2,53731	2,42465	2,10973	1,56986	0,0299849	5,18713	0,090049	0,696624	0,6279808
CG3917-RB	Grip84	0,591593	0,0199579	0,0210357	0,0221371	5,66401	3,1042	0,104546	0,65412	0,6279808
CG3917-RC	Grip84	0,598423	1,09017	1,71331	8,69213	0,576893	0	0,108139	0,641393	0,6279808
CG3918-RA	CG3918	8,9191	0,0166093	21,0226	0,0203086	168,647	19,0902	-0,050947	0,811928	0,6279808
CG3919-RB	CG3919	1,38265	1,19312	2,30552	1,72622	1,01953	1,67694	0,238464	0,453786	0,6279808
CG3920-RA	Reph	9,25019	6,64611	11,5083	2,70224	4,88865	4,61921	0,783395	0,01807	0,6279808
CG3920-RB	Reph	1,8884	17,4376	7,24359	9,83869	2,71677	2,94673	0,735599	0,025393	0,13772387
CG3921-RA	bark	7,43601	2,91345	4,46183	9,96938	3,99933	0,780654	-0,087422	0,772127	0,6279808
CG3922-RB	RpS17	1328,73	15,6434	14,6431	23,7133	9,72098	14,3851	-0,347439	0,234344	0,6279808
CG3923-RA	ebo	8,39173	3,53641	9,28005	6,02171	0,230312	0,173577	0,197591	0,438394	0,6279808
CG3924-RA	Chi	0,0319104	0,0290662	0,0306359	29,2651	1,35842	1,7564	0,07746	0,706079	0,6279808
CG3924-RB	Chi	21,3085	27,3971	27,5138	38,8404	8,67562	10,3649	0,077762	0,704483	0,6279808
CG3925-RA	ohgt	11,9998	3,18688	14,2037	7,68209	19,6749	16,3765	-0,158324	0,41665	0,6279808
CG3926-RA	Spat	12,2076	4,21833	183,742	13,0024	4,11683	4,34032	-1,044776	0,001145	0,6279808
CG3927-RA	CG3927	0,0925433	0,16859	0,0592314	0,24672	3,51951	0,0752124	-0,07023	0,76277	0,6279808
CG3929-RA	dx	5,82311	5,50083	5,20898	8,36158	2,50098	4,5946	0,036047	0,900013	0,6279808
CG3931-RA	Rrp4	12,8497	0,806069	17,5427	0,222984	0,249526	0,0580335	-0,842119	0,00079	0,6279808
CG3934-RA	Npc2c	2,24239	13,2288	6,56708	0,364717	0,0276616	0,743205	0,281398	0,417525	0,6279808
CG3935-RA	al	17,5823	33,2495	16,4157	0,0118798	5,27976	8,83153	0,489045	0,082572	0,6279808
CG3936-RA	N	7,80797	0,0310133	6,50307	0,0367797	0,0432726	17,2157	0,046527	0,875338	0,6279808
CG3937-RA	cher	128,037	0,495096	0,0179166	0,174852	21,119	0,178496	0,134364	0,602541	0,6279808
CG3937-RB	cher	0,0191709	0,0806067	121,418	1,87912	0,0717726	0,290832	0,22224	0,398738	0,6279808
CG3937-RF	cher	0,0186304	7,83215	0,0184052	0,661386	3148,29	2,62421	0,225599	0,390386	0,6279808
CG3937-RG	cher	0,00880346	35,2683	0,0178863	39,154	0,0448604	1,92864	0,132405	0,607312	0,6279808
CG3937-RH	cher	0,00818514	0,162228	0,00845185	9,20876	0,064139	5,62826	0,225387	0,390833	0,6279808
CG3938-RA	CycE	2,60775	2,90607	0,0171977	1,40507	0,609653	0,0190039	-0,300125	0,259849	0,6279808
CG3938-RB	CycE	2,91268	2,30993	1,51347	8,33115	2,61146	0,0194659	-0,338825	0,208008	0,6279808
CG3938-RC	CycE	0,0179132	0,0163166	0,0183786	0,018616	0,0252155	1,94086	-0,299432	0,262386	0,6279808
CG3938-RD	CycE	0,0183306	0,0166968	159,692	1,73275	0,877982	191,75	-0,298391	0,262357	0,6279808
CG3938-RE	CycE	0,0191432	0,0261554	0,121139	0,0199519	0,027025	0,179602	-0,301327	0,256801	0,6279808
CG3939-RA	CG3939	39,552	8,385	49,8411	12,1785	0,0334458	0,0252067	-0,226309	0,438091	0,13772387
CG3940-RA	CAH7	7,89527	4,34445	11,3248	7,02182	13,6813	10,311	-0,171463	0,572471	0,6279808
CG3941-RA	pita	0,0271359	0,0252707	0,021953	0,0295145	0,0325671	0,0301295	0,030239	0,899652	0,6279808
CG3941-RB	pita	8,52592	12,644	0,0218471	16,2264	0,0324015	0,0295342	0,063091	0,794604	0,6279808
CG3942-RA	CG3942	0	8,88314	0,0633523	3,99985	8,81922	88,5586	NA	NA	0,6279808
CG3943-RA	kraken	30,2869	0,0168417	9,37629	2,4722	2,87502	7,58725	-0,345243	0,176797	0,6279808
CG3944-RA	Dpse\GA17794	96,6864	103,434	0,0398555	1,32935	0,0825997	0	-0,003119	0,990811	0,6279808
CG3945-RA	Rad9	0,0384908	0,0236427	20,9505	12,2499	0,0372418	33,7294	-0,286095	0,292369	0,6279808
CG3945-RB	Rad9	0,0479969	6,63768	0,446738	0,572957	12,8463	0,0429115	-0,437218	0,182613	0,6279808
CG3947-RA	Pex16	16,984	0,0410922	13,124	0,167441	28,157	22,5204	-0,2221	0,43985	0,6279808
CG3948-RA	zetaCOP	0,103136	0,0939432	10,2448	3,31257	3,07934	2,76864	-0,235496	0,330724	0,6279808
CG3948-RB	zetaCOP	97,4019	87,3562	10,596	0,132843	0,108664	0,0818959	-0,266554	0,275863	0,6279808
CG3948-RC	zetaCOP	0,0676544	0,0616243	35,2103	11,3799	8,29346	6,18037	-0,235496	0,330724	0,6279808
CG3949-RA	hoip	92,1005	5,84606	24,983	0,0238348	0,0463034	0,034897	-0,353778	0,31518	0,6279808
CG3953-RA	Invadolysin	17,2893	17,1433	15,4739	0,0319162	0,0062507	5,89202	0,505154	0,038523	0,6279808
CG3954-RA	csw	3,75509	10,8446	9,83325	9,64634	12,2744	12,8526	-0,022782	0,920269	0,6279808
CG3954-RB	csw	6,45252	4,28885	23,2325	7,27422	27,9849	24,3076	-0,020495	0,926794	0,6279808
CG3954-RC	csw	6,34895	7,11542	0,0470909	4,88076	0,0384284	0,0359412	-0,009869	0,965402	0,6279808
CG3955-RA	CG3955	10,2213	229,185	5,48779	340,765	2,33477	3,64293	-0,005875	0,980187	0,6279808
CG3956-RA	sna	10,6974	0	0	0	4,49457	8,06876	0,280932	0,346966	0,6279808
CG3957-RA	wmd	23,2994	0,0390652	0,0411748	0,0473523	0,047804	0,048339	-0,037034	0,870597	0,6279808
CG3957-RB	wmd	0,0428878	0	0,0844979	24,4102	0,45929	0,346149	-0,036144	0,87383	0,6279808
CG3959-RA	pelo	12,7319	0,0206566	13,9829	5,35001	82,1614	0,0880981	-0,155594	0,453259	0,6279808
CG3961-RA	CG3961	4,18622	0,0437189	0,0460799	10,9691	117,501	10,874	0,247643	0,30565	0,6279808
CG3961-RC	CG3961	15,1029	5,8286	7,73183	12,0396	35,0649	0	0,247643	0,30565	0,6279808
CG3962-RA	Keap1	0,0230256	9,53852	0,0203999	9,01493	1,21358	0,0227206	-0,10713	0,7157	0,6279808
CG3962-RB	Keap1	0,0212485	5,7901	8,02599	5,33135	0,0733358	3,70272	-0,111053	0,705629	0,6279808
CG3962-RC	Keap1	5,6892	0,016185	21,8877	25,7219	11,8548	11,8605	-0,103937	0,723838	0,6279808
CG3964-RA	TTLL4B	0,0191543	0,01679	18,8335	0,019964	14,8176	0,230376	-0,33508	0,284409	0,6279808
CG3964-RB	TTLL4B	0,754723	6,37793	0,0176967	1,89509	0,0259788	0,726203	-0,33508	0,284409	0,6279808
CG3966-RA	ninaA	1,23892	32,8702	0,0423854	1,48422	0,0417707	1,2651	0,078301	0,819731	0,6279808
CG3967-RA	Tat	0,0308381	0,0280895	0,0296064	35,9513	0,0447704	25,8375	0,108098	0,551606	0,6279808
CG3967-RB	Tat	29,8322	30,026	33,5002	51,884	23,7639	46,6134	0,108012	0,557428	0,6279808
CG3967-RC	Tat	0,0309103	0,0281552	0,0296757	0,0500119	0,044883	0,051054	0,094838	0,598039	0,6279808
CG3967-RD	Tat	0,0263495	0,024001	0,0252971	0,0587521	0,0378418	0,0599763	0,108105	0,551715	0,6279808
CG3967-RE	Tat	27,1965	28,4238	27,5049	0,0536787	19,7784	0,0547972	0,108098	0,551606	0,6279808
CG3969-RA	Ack-like	0,015191	0,013837	0,0145843	0,0156865	0,0212475	0,0160134	-0,014123	0,965292	0,6279808
CG3969-RB	Ack-like	8,65878	11,192	7,27013	18,0965	3,68058	5,89006	-0,014123	0,965292	0,6279808
CG3971-RA	Baldspot	5,66831	0,0248226	7,05047	7,18818	7,97596	7,53923	0,334238	0,068419	0,6279808
CG3971-RB	Baldspot	37,9326	40,1257	1,21527	8,48331	1,30054	2,79049	0,333993	0,068574	0,6279808
CG3972-RA	Cyp4g1	33,4152	43,0937	4,10118	11,9963	14,7356	0,0276069	-0,210088	0,294138	0,6279808
CG3973-RA	pigs	12,7855	16,7585	48,4323	30,0301	42,9682	2,98413	1,398686	5,75E-08	0,6279808
CG3975-RA	Pol32	4,08734	0,0355572	0,0374774	0,0426892	1,90562	0,0435788	0,688321	0,000753	0,6279808
CG3977-RA	Ctr1A	30,7071	9,50245	13,5897	13,3249	6,85637	19,7271	0,340156	0,120452	0,6279808
CG3978-RA	pnr	4,08304	1,94342	1,9155	25,5632	28,3169	17,2677	0,064616	0,798612	0,6279808
CG3978-RB	pnr	1,33782	36,8932	20,0532	43,5319	0,0371488	24,261	0,055348	0,827476	0,6279808
CG3979-RA	Indy	38,5678	0,111871	0,117912	73,8537	4,13714	6,38485	-0,002226	0,991637	0,6279808
CG3979-RB	Indy	0,0259562	47,7211	80,4241	27,3016	7,90576	5,79	-0,003454	0,986999	0,6279808
CG3979-RC	Indy	12,6276	20,2876	22,393	0,0420356	51,366	35,7984	-0,001654	0,993785	0,6279808
CG3980-RB	Cep97	0,0224544	0,020453	0,0215576	0,0235873	102,777	0,0240788	0,228682	0,33039	0,6279808
CG3980-RC	Cep97	6,25425	7,21525	6,47907	7,92476	0,0319492	3,89805	0,228682	0,33039	0,6279808
CG3981-RA	Unc-76	10,9497	10,2626	11,7082	28,5968	1,96299	15,3727	0,12148	0,624961	0,6279808
CG3981-RB	Unc-76	24,0283	35,1236	18,9	18,9537	0,0197304	10,2205	0,121203	0,625478	0,6279808
CG3982-RA	CG3982	0,635782	0,496383	0,959182	25,858	0,586333	38,0499	-0,128204	0,70606	0,6279808
CG3983-RA	Ns1	19,8853	33,9771	46,9681	6,71133	60,063	51,9267	-0,332981	0,269598	0,6279808
CG3984-RA	CG3984	15,127	12,3244	21,9906	20,7398	0,096225	11,3965	0,557174	0,038044	0,6279808
CG3985-RC	Syn	26,4087	0,0111567	0,0126301	11,8064	0,0203512	1,25824	0,34108	0,208261	0,6279808
CG3985-RF	Syn	0,0122484	37,2334	7,20794	0,0143539	0,019726	5,60873	0,34108	0,208261	0,6279808
CG3986-RA	Cht4	0,561106	0,776864	2,06859	29,0586	0,0399775	0,147703	-0,331265	0,344543	0,6279808
CG3987-RA	CG3987	30,0571	26,4524	41,7998	26,1342	0,0679994	42,1486	-0,295625	0,32537	0,6279808
CG3988-RA	gammaSnap1	30,5818	28,047	36,9233	36,0169	31,3698	29,8346	-0,024545	0,905016	0,6279808
CG3989-RA	Paics	31,301	34,9375	3,40221	0	4,21527	0	-0,593655	0,010459	0,6279808
CG3991-RA	TppII	7,42218	0,0827114	0,0138297	0,113897	0,154274	9,54738	-0,056526	0,798267	0,13772387
CG3991-RD	TppII	0,014405	9,67697	213,319	16,0791	6,01571	6,49766	-0,264003	0,26877	0,6279808
CG3992-RA	srp	13,4882	19,4984	11,7969	0,0274261	0,0395523	0,0279976	0,27841	0,321093	0,6279808
CG3992-RB	srp	4,74819	7,73254	3,07538	0,0292006	0,0345328	0,029809	0,271504	0,333607	0,6279808
CG3992-RD	srp	0,0149911	0,0136549	0,0143924	0,0254947	0	0,026026	0,273007	0,303575	0,6279808

gene_id	Symbol	W1_FPKM	W2_FPKM	W3_FPKM	MCM51_FPKM	MCM52_FPKM	MCM53_FPKM	FC	p-value	p-adj
CG3994-RA	ZnT35C	0,0385132	2,06536	1,77617	21,1777	0,0578228	1,30242	-0,112239	0,678247	0,6279808
CG3994-RB	ZnT35C	13,4597	4,31498	4,52338	0,0167173	9,62986	2,2375	-0,058159	0,828637	0,6279808
CG3995-RA	CG3995	8,91483	8,42099	12,5644	11,2663	4,42761	17,9994	0,324853	0,186477	0,6279808
CG3996-RA	CG42795	0,900746	1,18082	0,929204	1,91345	1,20426	2,20007	-0,43094	0,101553	0,6279808
CG3997-RA	RpL39	5619,54	5372,11	4678,74	3,13255	2,0658	21355,1	-0,660122	0,010783	0,6279808
CG3998-RA	zf30C	13,6052	14,9154	17,1037	20,7469	0,116894	0,0199449	0,171231	0,492956	0,6279808
CG3999-RA	CG3999	12,1898	0,100534	0,121371	11,7494	11,1521	11,2533	-0,212692	0,3159	0,6279808
CG40000-RA	CG4000	15,1341	12,2836	21,747	0,368961	0,49976	5,56274	NA	NA	0,6279808
CG40001-RA	CG40000	0	0	0	0,119369	0	0	NA	NA	0,6279808
CG40001-RB	CG40001	0,245991	0,112033	0,236166	0,0394438	396,863	0,567151	-1,310007	0,000242	0,6279808
CG40002-RA	CG40001	0,119703	0,109033	0,127212	0,0763943	0,401077	0,121856	-0,451234	0,156567	0,6279808
CG40005-RA	Dpse\GA17837	144,349	77,4112	83,3608	116,152	199,654	198,282	-0,041811	0,731452	0,6279808
CG40005-RB	CR40005	11,2156	10,5642	0,0392635	0,0449306	9,48518	9,75702	NA	NA	0,6279808
CG40006-RA	CR40005	2,68519	0,108705	0,042442	0,0489711	3,73215	3,47316	-0,047923	0,865741	0,6279808
CG4000-RA	CG40006	12,6156	7,28512	15,4551	22,3176	9,83576	9,13655	0,052009	0,853819	0,6279808
CG4001-RA	Pfk	4,23028	0,018705	21,7297	30,6437	0,328049	16,7114	-0,361688	0,079068	0,6279808
CG4001-RB	Pfk	10,0524	13,9536	7,28334	0,0620729	16,1615	7,66064	-0,383369	0,062842	0,6279808
CG4001-RC	Pfk	18,5584	23,0815	0,0197151	0,45086	8,91255	0,208863	-0,384727	0,061218	0,6279808
CG4003-RA	pont	9,06963	0,115152	20,0327	21,7421	7,94934	12,1291	0,065732	0,83304	0,6279808
CG40040-RA	CG4004	3,65151	2,72021	5,09969	3,814	0,00714033	2,25386	0,059792	0,706172	0,6279808
CG40040-RB	CG4004	3,45148	1,91944	2,04635	2,61924	0,580768	2,42994	0,056255	0,829893	0,6279808
CG40041-RA	CG40040	0,14147	0,0515441	0,0543277	24,0606	23,2683	18,5773	-0,334474	0,197197	0,6279808
CG40041-RC	CG40040	0,0697003	0,168181	0,177263	16,3849	11,8523	10,2797	-0,383952	0,16066	0,6279808
CG40042-RA	Gpb5	6,03439	8,70691	9,17711	8,66841	12,2372	0	-0,165559	0,56443	0,6279808
CG40042-RD	Gpb5	4,13246	2,27043	2,01519	8,82086	10,7536	0	-0,165559	0,56443	0,6279808
CG40045-RA	Tim23	69,8751	56,5289	130,143	87,7595	0	0,583887	-0,186946	0,48277	0,6279808
CG40049-RA	Tim23	0,0720826	0,0656578	0,0692036	0,08585	102,356	106,431	-0,416876	0,177138	0,6279808
CG40049-RC	CG40045	440,866	3276,45	4402,9	3948,16	538,332	4542,99	-0,417049	0,177022	0,6279808
CG40049-RD	mRpS5	0,0482775	0,0439745	0,0463493	0,0540308	0	0	-0,234387	0,45858	0,6279808
CG4004-RA	mRpS5	339,95	294,29	183,161	245,323	0	0,0551567	0,295516	0,293491	0,6279808
CG4004-RB	mRpS5	0,177782	0,161936	0,170681	0,296105	0	311,272	0,260366	0,361348	0,6279808
CG40050-RA	yki	0,0449597	0,0409524	0,043164	0,0498983	15,1113	18,2133	0,440214	0,169205	0,6279808
CG40053-RA	yki	5,04139	6,42886	5,20304	8,97265	0,0415019	0,0312783	-0,070526	0,562659	0,6279808
CG4005-RD	yki	22,3588	32,3755	26,6536	31,3514	134,326	108,301	0,254664	0,294594	0,6279808
CG4005-RE	nvd	1,42963	1,26152	1,37253	0,747025	0,0471597	0,301312	0,251115	0,299702	0,6279808
CG4005-RF	lncRNA:CR40053	0	3,6411	8,13195	0,0853135	0,151653	0,273968	0,251424	0,299022	0,6279808
CG40064-RA	Akt1	9,27161	5,37379	5,664	4,10186	6,99814	0,011775	NA	NA	0,6279808
CG40068-RA	Akt1	12,5331	9,94585	8,38785	0,76355	0,0141208	0,0104716	NA	NA	0,13772387
CG4006-RA	ARY	0	0	0	0	0	0	0,000619	0,997858	0,6279808
CG4006-RB	CR40068	1,75564	1,88991	55,97	4,69787	2,59288	3,81561	0,000976	0,996625	0,6279808
CG4007-RA	Nrk	8,20252	13,0686	0,0191729	1,61329	0,647021	0,11627	-0,116654	0,672686	0,6279808
CG40080-RA	und	44,026	55,3837	6,72117	5,62501	0,0264641	5,68984	-0,47566	0,078877	0,6279808
CG40084-RA	Haspin	38,8682	39,7562	24,8201	16,4424	33,3845	20,6898	-0,587771	0,039606	0,6279808
CG40084-RB	uex	34,9116	0,06057	0,063841	0,108197	0	0,318325	-0,587771	0,039606	0,6279808
CG40085-RA	uex	10,916	0,059258	0,0624582	0,309441	0	0,10973	-0,013096	0,914398	0,6279808
CG40088-RA	CG40085	0	0	0,283368	0	0	2,32773	0,044335	0,715924	0,6279808
CG4008-RA	CG40088	0	0,206118	0	0	0	0	-0,09318	0,638881	0,6279808
CG40091-RC	CG4009	0,0882424	7,55256	0,41319	2,41476	0,030147	1,40394	0,258572	0,256801	0,6279808
CG40092-RA	CG40091	0,188131	0,289916	0,143263	0	0,135639	0	NA	NA	0,6279808
CG40096-RA	CG40092	0	2,09881	0	0	0	0	0,192804	0,274841	0,6279808
CG4009-RA	CG40096	0,0710493	0	0,289445	0	0	0	0,022862	0,858938	0,6279808
CG40100-RA	CR40100	1,04882	0,477671	0,704853	1,85184	0	59665,2	-0,074797	0,817515	0,6279808
CG40103-RA	CG40103	0	0	0	0	0	0	NA	NA	0,6279808
CG40108-RA	CG40108	0	0,153276	0,323108	0,150957	0	0	NA	NA	0,6279808
CG40113-RA	CG40113	0	0,226378	0,0883521	0	0	0	-0,013096	0,914398	0,6279808
CG40113-RB	CG40113	0	0,0658734	0,0694309	0	0,585225	0	NA	NA	0,6279808
CG40113-RC	CG40113	0	0,123841	0,146617	0	1,91059	0	-0,060347	0,691408	0,6279808
CG40116-RA	CG40116	0	0,496588	0	0	0	0	NA	NA	0,6279808
CG40119-RA	CG40119	0	0	0	0	0	0	NA	NA	0,6279808
CG40120-RA	gek	6,2027	5,59093	6,22818	3,86758	1,30678	4,23956	0,094281	0,621462	0,6279808
CG40120-RB	CG40120	0,0876723	0,079858	0	0,108998	0	0	0,146626	0,476487	0,6279808
CG40121-RA	CG40120	0,32514	0,39488	0	0,391692	0	0	0,01562	0,897983	0,6279808
CG40122-RA	CG40121	0	0,278641	0,293689	0,305054	0,413198	0,311411	-0,074864	0,550506	0,6279808
CG40124-RA	CG40122	0	0	0	0,220363	0,298483	0	-1,036536	0,001804	0,6279808
CG40127-RA	CG40124	0	0,226719	0,095585	0,795633	0,87743	1,03439	-0,501954	0,114034	0,6279808
CG40129-RA	RNASEK	550,1	445,134	6,22096	0,0256037	1117,39	1060,42	0,036475	0,885922	0,6279808
CG4012-RA	Gprk1	52,535	54,2277	39,2606	61,5477	42,212	30,1504	0,007984	0,973525	0,6279808
CG40137-RA	Smr	8,90584	0,00475833	2,79518	3,66163	54,2011	3,67542	NA	NA	0,6279808
CG40138-RA	Smr	0,00525719	0,389948	3,10029	5,81104	0,0464721	3,77241	-0,7136	0,043353	0,6279808
CG4013-RA	Smr	0,00522394	17,2522	15,8097	1,10573	13,5155	13,5625	0,173175	0,441074	0,6279808
CG4013-RB	CG40137	0,549103	0,500161	0,790757	6,093	2,23723	2,11629	0,184624	0,408707	0,6279808
CG4013-RC	CG40138	0,644357	0	0,0836897	0,0236721	0,032064	0,0241653	0,228663	0,279921	0,6279808
CG40143-RA	CG40143	0,192827	0,17564	0,0322396	0,0362337	0,0490787	17,9122	-0,282219	0,3775	0,6279808
CG40153-RA	Fcp3C	0,034048	34,4401	0,0326881	36,839	111,459	84,8051	NA	NA	0,6279808
CG40155-RA	CG40153	0	0	211,586	130,922	102,992	112,813	NA	NA	0,6279808
CG40158-RA	Myo81F	0	0	0	0	0	0	0,044335	0,715924	0,6279808
CG40159-RA	CG40158	0	0	0	0	0	0	NA	NA	0,6279808
CG4015-RA	CG40159	0	0	0	0	0	0	-0,144849	0,613958	0,6279808
CG40160-RA	Spt-I	0,0438265	0,0399202	0,0420761	18,476	0,065697	0,0495132	0,255953	0,28374	0,6279808
CG40160-RB	Spt-I	0,0429715	0,0391414	0,0412552	1,97323	0,0642775	0,0484434	0,255953	0,28374	0,6279808
CG40160-RC	Spt-I	14,024	12,6305	21,4385	23,7288	22,7511	21,4996	0,25492	0,285479	0,6279808
CG40162-RA	CG40160	36,1577	9,41422	1,36655	0,136422	0	20,8461	NA	NA	0,6279808
CG40164-RA	CG40160	0,0487404	53,1694	35,9662	0,252351	0	0	NA	NA	0,6279808
CG40169-RA	CG40160	27,6836	0,585472	0,0467937	33,2673	32,5851	0	NA	NA	0,6279808
CG40169-RB	CG40162	0	0	0	0	0	0,912989	NA	NA	0,6279808
CG4016-RA	CG40164	0	0	0	0	34,9546	0	-0,408991	0,093419	0,6279808
CG4016-RB	CG40169	0	0	0	6,41946	0	19,0149	-0,409298	0,093336	0,6279808
CG4016-RC	CG40169	0	0	0	0	0	0	-0,408257	0,094163	0,6279808
CG40172-RB	CG4017	3,98417	4,00127	4,37427	0,0195378	6,81007	2,84484	NA	NA	0,6279808
CG40174-RA	lncRNA:CR40172	0	777,236	0	0	0	0	NA	NA	0,6279808
CG40178-RA	CG40174	0	8,79639	11,121	0	42,2576	0	-0,794025	0,011184	0,6279808
CG40178-RB	CG40178	14,6667	21,0043	5,35349	3,75621	2,84596	1,90964	-0,793885	0,011193	0,6279808
CG4017-RA	CG40178	56,5761	5,97128	30,1377	0	0	25,1957	-0,557146	0,029362	0,6279808
CG40181-RA	CG40181	0,890511	1,21671	0,59846	1,26121	1,70832	0,962493	0,403856	0,251174	0,6279808
CG40182-RA	CR40182	0	0	0,0310863	0	0	0	NA	NA	0,6279808
CG40188-RA	Pzl	0	0	0	0	0	0	NA	NA	0,6279808
CG40188-RB	Pzl	0	0	0	0	0	0	NA	NA	0,6279808
CG40189-RA	CG40189	0,135401	0,0616665	0,129993	0,208978	0,170942	0,0799805	-0,036477	0,908512	0,6279808
CG40191-RA	Eglp4	76,4223	54,0877	3,54163	22,7459	1,19642	1,45884	0,559141	0,038558	0,6279808
CG40191-RB	Eglp4	0,0614849	0,0560047	94,8236	0,0711942	1,99007	0,0144732	0,576797	0,033939	0,6279808
CG40191-RC	Eglp4	0,0638619	0,0581698	0,0590292	0,0744101	0,0192039	9,21034	0,559239	0,03834	0,6279808
CG40191-RD	Eglp4	55,1344	64,0599	46,995	98,0021	11,565	0,0158999	0,572722	0,034995	0,6279808
CG40195-RA	CG40191	0,0449291	0,0409246	0	0,0498605	0	0	-0,080649	0,53498	0,6279808
CG40196-RA	CG40191	0,0502987	0,0458156	0	16,6163	0	0	-0,247551	0,360813	0,6279808

gene_id	Symbol	W1_FPKM	W2_FPKM	W3_FPKM	MCM51_FPKM	MCM52_FPKM	MCM53_FPKM	FC	p-value	p-adj
CG40196-RB	CG40191	95,1511	74,9285	0,0431347	58,5508	345,828	2,24868	-0,247551	0,360813	0,6279808
CG40196-RC	CG40191	80,6785	69,0582	0,0482898	24,2998	2,63311	0,0259257	-0,247551	0,360813	0,6279808
CG40198-RA	CG40195	0	0	0	0	0,632846	0	-0,741838	0,037716	0,6279808
CG4019-RA	Maf1	90,1708	86,0517	0,0516305	0,0620729	48,4134	44,7542	-0,223485	0,457023	0,6279808
CG4019-RB	Maf1	0,0537784	13,8138	22,4656	26,4645	19,6047	15,627	-0,223053	0,457919	0,6279808
CG4019-RC	Maf1	30,6138	12,1263	1,83875	0,232405	88,0181	67,2208	-0,223592	0,456815	0,6279808
CG4019-RD	CG40198	16,9518	0	33,5536	0,131517	0	37,8434	-0,2234	0,457301	0,6279808
CG40203-RA	CG4020	0,778672	0,58768	1,00388	1,91604	0,613549	1,35062	0,01562	0,897983	0,13772387
CG40204-RA	CG40203	0	0	0	0	0	0	-0,039118	0,754991	0,13772387
CG40207-RA	Myo81F	0	0	0,229843	0,467215	0	0	-0,379229	0,130643	0,6279808
CG4020-RA	CG40207	0	0	0,39648	1,86259	1,15528	0,540535	-0,544791	0,103737	0,6279808
CG40211-RA	CG4021	0	0,138042	0,260699	0	1,55946	0,113047	0,044335	0,715924	0,13772387
CG40211-RB	CG40211	0	0,599467	0	0	0,0766416	0,0577617	0,141483	0,385711	0,13772387
CG40212-RA	CG40211	0	0,0805709	0,212305	0	56,1467	43,1329	0,01562	0,897983	0,6279808
CG40216-RA	CG40212	0	0	0	0	0,0630515	0	NA	NA	0,6279808
CG40218-RA	CG40216	0	0,423184	0,371698	1,62223	29,8488	30,7573	0,278514	0,343801	0,6279808
CG4021-RA	Yeti	486,33	407,774	244,132	0,511317	0	0,0661065	NA	NA	0,6279808
CG40225-RA	CG4022	3,90374	4,51648	127,185	114,139	136,131	80,6018	-0,041811	0,731452	0,6279808
CG40228-RB	CG40225	0	0	0	0,246773	0	0	-0,20179	0,498909	0,6279808
CG40228-RC	Dpse\GA17880	27,3799	26,3978	13,2023	24,1194	23,8136	24,1949	-0,204797	0,490528	0,6279808
CG4022-RA	Dpse\GA17880	184,989	163,482	93,8001	187,94	216,57	249,984	-0,301374	0,258938	0,13772387
CG40239-RA	CG40239	0,404877	0,221274	17,2003	107,32	0,151139	0,113907	0,114498	0,560595	0,13772387
CG40244-RA	CG40244	72,6305	914,826	0,119063	32,7133	35,8069	21,1055	-0,134237	0,681148	0,6279808
CG40245-RA	WDY	0	0,486355	0	0	0	0	NA	NA	0,6279808
CG40249-RA	Pzl	0	0	0	0	0	0	NA	NA	0,6279808
CG4025-RA	CG4025	6,77314	9,04852	7,77605	10,0511	6,54328	6,74827	-0,057163	0,783476	0,6279808
CG40263-RA	IP3K1	0,0331595	0,030204	0,0251866	0,0230914	0,0312774	0,0235725	0,007729	0,97836	0,6279808
CG40263-RB	MFS17	15,6018	0,039553	10,386	0,0480078	0,0650268	0	-0,003107	0,991336	0,6279808
CG40263-RC	MFS17	27,4645	0,0344382	19,3388	0,0412204	0,0558333	9,64895	0,197731	0,471454	0,6279808
CG40263-RD	MFS17	9,00014	0	0,0717068	0	0	16,1823	0,198848	0,477558	0,6279808
CG40263-RE	MFS17	0,0434233	0	0,041689	0	0	0,0914518	0,257514	0,352255	0,6279808
CG40267-RA	MFS17	0,037808	0	0,036298	0	0	0,0490082	NA	NA	0,6279808
CG40267-RB	CG40267	0	0,171634	0	0,260219	0	0	NA	NA	0,6279808
CG40268-RA	CG40267	0	0	0	0	0	0	NA	NA	0,6279808
CG40269-RA	CG40268	0	0	0	0	0	0	NA	NA	0,13772387
CG4026-RA	CG40269	0	0	0	0	0	0	-0,400041	0,082165	0,6279808
CG40270-RA	Act5C	0,041697	0,0379805	3,07217	140,34	2,93057	2,30512	0,076465	0,555158	0,6279808
CG40270-RB	Act5C	764,413	708,673	143,846	7,80316	91,5938	0,0570272	NA	NA	0,6279808
CG40271-RA	Act5C	137,418	139,37	0,0477446	0,0558631	6,95404	18,097	NA	NA	0,13772387
CG40275-RA	Act5C	0,0425838	0,0387882	15,9201	14,3006	0,0756669	108,976	NA	NA	0,13772387
CG40275-RB	CG40270	0,0858128	0	0	0	1,44015	0,250896	NA	NA	0,6279808
CG4027-RA	CG40270	0,366634	0	0,26257	0	282,189	0,0879828	-0,453517	0,085708	0,6279808
CG4027-RB	CG40271	0,168132	0,57503	0	0	1,47516	0	-0,452652	0,08581	0,6279808
CG4027-RC	CG40275	0,211782	0	0,203323	0,211191	0	0,215592	-0,34811	0,172213	0,6279808
CG4027-RD	CG40275	0,0948759	0	0,182173	0,141853	0	0,144809	-0,357744	0,162524	0,6279808
CG40293-RA	jumu	5,92069	0,0683418	5,91388	7,2181	0,334001	3,35613	-0,002208	0,993567	0,13772387
CG40293-RB	Stlk	0,122438	0,111525	56,7297	0	0,0628738	0,0473855	-0,265301	0,350622	0,13772387
CG40298-RA	Stlk	37,4965	32,9646	0,0404399	124,81	20,4179	0,267583	-0,374571	0,247801	0,13772387
CG4029-RA	CG40298	8,56539	5,51709	2,92822	5,90023	13,5904	9,08585	0,263086	0,311031	0,13772387
CG40300-RD	Rbpn-5	7,86075	6,60235	9,24208	8,08775	4,83823	5,82476	0,665689	0,013907	0,13772387
CG40300-RE	37834	0,0246582	0,0224604	7,04853	0,0260388	3,15392	1,4782	0,667338	0,013736	0,13772387
CG40300-RF	37834	4,2639	3,96406	2,2253	3,20605	0,0360242	0,761701	0,659542	0,014771	0,6279808
CG40305-RB	37834	0,024797	0,0225869	2,68077	0,026194	0,03548	0,0267399	1,899944	2,31E-15	0,13772387
CG4030-RA	FucTC	13,2305	11,0295	13,9747	3,11979	3,04385	3,21909	0,236862	0,270961	0,13772387
CG40311-RA	CG40311	0	0	0,118795	0	0	0,0420793	-0,062968	0,745795	0,6279808
CG40311-RB	CG40311	0	0	0,462814	0	0	0,202897	0,364691	0,184144	0,13772387
CG40313-RA	CG40313	0	0	0	0	0,0356501	0	-0,041811	0,731452	0,6279808
CG40313-RB	CG40313	0	0	0	0	0	0	-0,041811	0,731452	0,13772387
CG4032-RA	Abl	6,2514	6,49927	5,73341	140,079	0,102057	0,0640909	0,265007	0,292204	0,13772387
CG4032-RB	Abl	2,54643	2,60659	29,1089	0,0254849	0,0850395	2,15258	0,265367	0,291576	0,6279808
CG40336-RA	Rpl135	6,23032	3,78334	9,85595	7,75877	118,002	5,70428	-0,013096	0,914398	0,13772387
CG40337-RA	CG40336	0	0,442979	0,320888	0	0,227101	0	-0,322142	0,355678	0,13772387
CG40337-RB	CG40337	0,583591	0,0463805	0,560282	0,345646	0,0777093	0,352848	0,009723	0,973074	0,13772387
CG40339-RA	CG40337	0,460993	72,1859	0,126452	1,33598	68,9773	0,522355	NA	NA	0,13772387
CG40339-RB	CG40339	0	0	0	0	0	0	NA	NA	0,6279808
CG4033-RA	CG40339	0	0	0	0	0	0	0,013636	0,9604	0,6279808
CG40341-RA	lncRNA:CR40341	0	8,8613	0	0	0	0	NA	NA	0,6279808
CG40346-RA	CG40346	0	0	0	0	0	0	NA	NA	0,6279808
CG40351-RA	eIF4E1	24,9656	26,4198	28,4709	0,0329537	25,377	0,0336404	0,19606	0,341116	0,6279808
CG40351-RB	eIF4E1	57,0307	76,4744	78,8792	26,1268	33,1277	21,7731	0,197006	0,338306	0,6279808
CG40351-RC	eIF4E1	0,0450517	0,0410362	0,0432523	0,0330529	0,0677414	0,0337417	0,197006	0,338306	0,13772387
CG40354-RA	eIF4E1	0,0520009	0,047366	0,0499239	34,6608	0,0795801	21,2395	-0,151492	0,671826	0,6279808
CG40354-RB	eIF4E1	0,0479969	0,0437189	0,0460799	0,033136	0,0727081	0,0338265	-0,097124	0,784299	0,6279808
CG40359-RA	eIF4E1	27,9475	0,0543362	40,4903	0,0279377	32,5458	0,0285199	-0,013096	0,914398	0,6279808
CG4035-RA	eIF4E1	16,7069	25,4468	26,2245	31,1515	25,9181	22,5072	0,034868	0,86045	0,13772387
CG4035-RB	Set1	0,0121627	358,262	342,813	0,0124712	1061,64	0,012731	0,028935	0,883913	0,13772387
CG4035-RC	Set1	0,0121537	0,0110786	0,0116769	0,0124617	0,0168923	0,0127214	0,067654	0,738308	0,13772387
CG4035-RD	Set1	17,3722	0,0110705	0,0116683	20,1315	0,0168795	10,4226	0,014023	0,94311	0,13772387
CG4035-RE	CR40354	1,85438	2,09681	2,82395	2,18456	3,2429	1,52169	0,034085	0,863308	0,6279808
CG4035-RF	CR40354	2,14508	1,40279	1,37293	0,571687	0,359379	1,10972	0,038527	0,845798	0,6279808
CG4035-RG	CG40359	0,156208	0	0	0,0833003	0	0	0,034789	0,860801	0,6279808
CG40368-RC	CG4036	6,34943	5,91061	13,096	9,16872	4,12662	14,9853	NA	NA	0,6279808
CG4036-RA	CG40368	0	0	0	0,0200247	0,0271236	0,020442	-0,456528	0,151002	0,6279808
CG40376-RA	CG40376	0,686503	0,611651	0,902556	2,77903	3,76421	0	-0,103114	0,756831	0,6279808
CG40378-RA	DIP-lambda	2,5075	0	3,25317	0	0,499833	0	0,040464	0,889631	0,13772387
CG40381-RA	CG4038	55,1199	56,7154	0,0214846	15,4915	1,38552	0,0239929	NA	NA	0,13772387
CG40382-RA	CG40381	0	0,26168	0	12,8571	15,6576	1,13291	0,01588	0,899333	0,13772387
CG40384-RA	CG40382	0,136803	0,350191	0,131339	70,162	119,526	95,1997	-0,071418	0,831791	0,13772387
CG40385-RA	CG40384	0,670335	0	0,275812	8,86778	16,3826	12,2192	0,024053	0,869957	0,6279808
CG4038-RA	CG40385	0,174189	0,0741213	0,20904	0	0	0,177473	-0,136533	0,567599	0,13772387
CG4039-RA	Mcm6	6,60996	6,71261	13,3299	14,0377	15,4229	8,98871	-0,152448	0,597762	0,13772387
CG40409-RA	Nost	5,15405	2,86722	1,344	7,42339	2,15199	1,4755	-0,041811	0,731452	0,6279808
CG4040-RA	CG40409	0	6,90533	0	6,65815	0,121344	0	0,383617	0,194975	0,6279808
CG40410-RA	CG4041	5,24412	6,39169	22,7478	23,4963	0	20,6406	-0,32795	0,283574	0,6279808
CG40410-RB	Alg-2	198,227	0,128879	106,7	180,553	0	0	-0,319343	0,297038	0,13772387
CG40411-RA	Alg-2	0,0260175	0,236955	0,0249784	0,0275637	0	240,849	0,069903	0,745848	0,13772387
CG40411-RB	Parp	2,18796	0	2,17413	0,0176581	0,17814	0	0,069903	0,745848	0,13772387
CG40411-RC	Parp	6,01594	3,48241	3,07521	5,00969	60,8572	2,66686	0,068728	0,749029	0,6279808
CG40413-RA	Parp	9,29521	3,18716	11,5827	13,7998	1,95145	3,4186	0,591852	0,032582	0,6279808
CG4041-RA	CG40413	0,364657	0,66431	0,350093	0	4,57281	0	0,069348	0,787918	0,6279808
CG4042-RA	CG4042	15,5473	6,70648	14,3801	0,831003	0,583327	0,0511316	-0,220191	0,409833	0,6279808
CG40439-RA	Rrp46	5,78064	3,72966	4,40159	0,0506656	8,11929	2,98035	-0,312006	0,32044	0,6279808
CG40439-RB	CG40439	30,6831	32,811	16,5667	21,1692	35,9261	38,5033	-0,315112	0,314817	0,6279808
CG4043-RA	CG40439	198,573	158,95	84,3312	206,069	274,889	278,561	-0,114684	0,734855	0,6279808

gene_id	Symbol	W1_FPKM	W2_FPKM	W3_FPKM	MCM51_FPKM	MCM52_FPKM	MCM53_FPKM	FC	p-value	p-adj
CG40440-RA	Ppr-Y	0,191942	0,0655626	0,115172	0	0,0949863	8,88624	-0,096511	0,632564	0,6279808
CG40441-RA	CR40441	0	0	0	0	0	0,0234878	NA	NA	0,6279808
CG40444-RA	kl-5	0	0	0	0,0142839	0	3,26427	-0,115958	0,399535	0,6279808
CG40445-RA	CG40445	0	0	0	0	0	0,0197401	-0,074864	0,550506	0,6279808
CG40446-RA	ORY	0	0	0,0114949	0	0	10,6267	-0,070526	0,562659	0,6279808
CG40448-RA	Pp1-Y2	0	0	0	0	0,147853	0,0261983	NA	NA	0,6279808
CG40451-RA	CG4045	16,4493	6,29795	0,0256829	0,0914501	1,96473	0,0296644	-0,306537	0,216062	0,6279808
CG40452-RA	Tim17b	199,414	0,2306	236,973	245,082	0,37147	1134,44	0,590094	0,028231	0,6279808
CG40452-RB	Snap25	0,0323637	5,39467	0,0310711	0,0348169	0,969405	0,0355424	0,589805	0,028412	0,13772387
CG4045-RA	Snap25	15,3508	12,5137	13,4128	11,685	0,295101	5,4323	-0,141671	0,54513	0,13772387
CG40461-RA	RpS16	1915,86	2245,73	31,2211	38,7391	77,6478	75,6916	NA	NA	0,6279808
CG40463-RA	CR40461	0,109397	0,398586	0	0,495812	0,0877139	0,138405	NA	NA	0,13772387
CG40467-RA	CG40463	0	0	0	0	0	0	-0,152363	0,647037	0,13772387
CG4046-RA	CG45782	0,344744	0,680369	0,275812	6,01397	2,60924	3,68138	-0,610803	0,030885	0,13772387
CG40470-RA	CG40470	3,91429	0,0815901	0,0859963	0,111962	3569,61	0,114295	-0,121677	0,671919	0,13772387
CG40478-RA	Dyrk3	24,9312	0,018447	0,044217	0,0207368	10,6815	60,0116	-0,037584	0,876041	0,13772387
CG40478-RB	Dyrk3	16,3364	0,0180921	46,6154	0,0115124	21,9982	93,3865	-0,038579	0,872608	0,6279808
CG40478-RC	Dyrk3	0,0203206	0,0102486	0,010802	12,2088	0,0287672	0,0117523	-0,037325	0,87683	0,6279808
CG40478-RD	Dyrk3	0,0202521	7,15889	7,69758	18,8618	0,0286655	3,70017	-0,037325	0,87683	0,13772387
CG40478-RE	Dyrk3	0,0198625	17,6346	15,3051	282,124	0,0280882	10,8192	0,025813	0,914107	0,6279808
CG40485-RA	CG40485	0,898883	0,0132491	0,0139647	0,0658574	0,0892042	2,03309	-1,182882	0,000554	0,6279808
CG40485-RB	CG40485	0,0995616	14,4653	9,62114	9,80868	20,5679	2,11526	-0,973994	0,002963	0,6279808
CG40486-RA	CG40486	0,0574739	1,09887	1,09008	0,862744	2,74441	0,0672297	-1,528992	1,82E-06	0,6279808
CG40486-RB	CG40486	3,21429	0,453438	0,095585	2,30211	1,25846	18,6207	-1,513235	2,26E-06	0,13772387
CG40498-RA	CG4049	2,02963	1,77455	2,25529	8,82919	2,24329	1,55125	0,772183	0,003168	0,13772387
CG40498-RB	CG40498	0,124985	21,9495	0,185488	0,232405	0	0,365989	0,774065	0,003071	0,6279808
CG40498-RC	CG40498	44,032	0,139105	0,0240048	0,699737	0,235167	0	0,768093	0,003275	0,6279808
CG40498-RD	CG40498	20,2732	0,0227749	115,266	0,276283	18,1644	0,490834	0,963445	0,00028	0,6279808
CG4049-RA	CG40498	0,152717	119,735	1,10646	0	17,3267	0,0269758	-0,157157	0,575351	0,6279808
CG40505-RA	Tmtc3	2,28005	1,37567	4,1628	2,80379	0,937058	1,26662	NA	NA	0,6279808
CG40506-RA	Tmtc3	6,64605	7,40398	5,22484	2,48167	4,01548	4,68683	NA	NA	0,6279808
CG4050-RA	CG40505	0,137658	0,376166	0,52864	0	0,115758	0	0,260324	0,242419	0,6279808
CG4050-RB	CG40506	0,510896	0,465359	0,653988	1,67856	2,61955	1,71579	0,261403	0,240773	0,13772387
CG4051-RB	egl	11,172	2,0951	80,902	43,1246	4,07578	33,7119	0,230165	0,383003	0,13772387
CG4052-RA	Cpr5C	2,62206	0	6,11352	7,20669	14,8585	11,2948	-0,249646	0,48545	0,13772387
CG40530-RA	CG4053	1,35401	0,082222	4,80976	3,36609	6,55085	9,90659	NA	NA	0,13772387
CG40534-RA	Mst77Y-16Psi	0	0	0	0	0	0	NA	NA	0,6279808
CG4053-RA	CG40534	0	0	0	1,60276	0	1,63616	-0,820958	0,021172	0,6279808
CG40551-RA	WDY	0,478811	0,127694	0	0,422114	0	0	0,159833	0,32487	0,6279808
CG40577-RA	tamo	3,67893	6,00491	0,0175595	51,1018	7,94164	1,85644	NA	NA	0,6279808
CG4057-RA	tamo	5,13018	3,93317	7,00386	0,0254357	1,67695	4,6847	0,158179	0,447443	0,13772387
CG4057-RB	CG40577	0	0	0	0	0	0	0,191014	0,353746	0,13772387
CG40583-RA	Nep4	3,56551	2,05296	2,50082	6,04191	4,36186	18,288	NA	NA	0,13772387
CG4058-RA	Nep4	9,7874	9,10784	7,80944	5,51567	5,53044	0,141209	0,028495	0,900763	0,13772387
CG4058-RB	WDY	0	0	0	0	0	0	0,051621	0,821172	0,13772387
CG4059-RA	ftz-f1	81,6273	66,3347	46,7046	49,6247	25,8636	33,0633	0,513671	0,071563	0,6279808
CG4059-RB	ftz-f1	0,0171063	109,736	7,30004	1,11813	1,7098	2,29575	0,542607	0,054753	0,6279808
CG40600-RA	TwdlW	149,548	0,0285289	1,03672	0,0336093	7,5121	0,75209	NA	NA	0,6279808
CG40600-RB	CG40600	0,129391	0	0	0	0	0	0,067475	0,590454	0,6279808
CG40601-RA	CG40600	0,173124	0	0,310558	0,630536	0,246876	0	NA	NA	0,6279808
CG40609-RA	CR40601	0	0	0	0	0	0	NA	NA	0,6279808
CG40609-RB	CG40609	0	0,126904	0	0	0	0	NA	NA	0,6279808
CG4060-RA	CG40609	0	0,0876077	0	0	0	0	-0,368864	0,087292	0,6279808
CG4061-RA	Rtca	0,04905	3,91287	14,754	0,0352076	15,4793	20,8974	-0,164626	0,486058	0,6279808
CG40624-RA	ValRS	0,0199705	0,0131211	0,185488	0,0150282	0,0203559	0,999517	NA	NA	0,6279808
CG40625-RA	ValRS	10,6889	109,286	10,5334	220,393	374,843	0,0212895	0,213311	0,401645	0,6279808
CG40626-RA	CG40624	0	0	0	0	0,361319	0	NA	NA	0,13772387
CG40629-RA	JYalpha	0,0641929	0,0292357	0,082172	0	1,10659	1,25929	NA	NA	0,13772387
CG40629-RB	CG40626	0	0	0	0	0	1,82928	0,076465	0,555158	0,6279808
CG4062-RA	lncRNA:CR40629	0,422838	0	0	0	0	0	-0,007779	0,974431	0,6279808
CG4062-RB	lncRNA:CR40629	0,205844	0	0	0	0	0	-0,007779	0,974431	0,6279808
CG40635-RA	ebi	7,21011	7,11016	12,9136	9,4594	11,8197	6,21466	NA	NA	0,13772387
CG4063-RA	CG40635	0	0	0	0,39931	0	0	0,191273	0,434938	0,6279808
CG4064-RA	CG42699	1,96948	2,91514	0,440839	0,363873	14,8852	40,3058	-0,243889	0,413944	0,6279808
CG4065-RA	Naa35	0,0266686	8,72354	0,0218321	8,44377	2,54523	4,30493	-0,132417	0,51369	0,13772387
CG4065-RB	Naa35	7,92431	0,0218568	4,71768	0,025299	3,47334	0,0258261	-0,132417	0,51369	0,13772387
CG40660-RA	CG4066	0,46743	0,391706	0,168715	1,32151	1,05649	0,0267825	NA	NA	0,13772387
CG4066-RA	CG40660	0,173884	0	0	0,46339	0,627665	0	0,473656	0,183602	0,13772387
CG4067-RA	pug	0,0205929	1,24731	0	0	0,116763	0,162673	-0,698687	0,001939	0,6279808
CG4067-RB	pug	0,0214764	0,111479	0,940644	0,720318	1,18057	0,84093	-0,695039	0,002021	0,6279808
CG4067-RC	pug	5,42669	0,0363518	8,38272	18,8159	17,693	8,65883	-0,698687	0,001939	0,6279808
CG4067-RD	pug	15,0554	12,9161	18,1047	9,82515	10,9673	18,6764	-0,696469	0,002012	0,6279808
CG40682-RA	CG44774	0,0221681	1,53512	0,0212827	3,37388	0,0315204	0,0237557	NA	NA	0,6279808
CG4068-RA	CG44774	0,0191155	4,15603	0,018352	4,18888	0,0269842	0,0203369	0,406277	0,188464	0,6279808
CG4068-RB	CG44774	0,0192716	0,0129248	0,0185019	0,0146182	0,0272146	0,0205105	0,490156	0,111939	0,6279808
CG4068-RC	CG44774	0,019797	0,66636	0,0190063	1,10383	0,0279913	0,0210959	0,489252	0,112982	0,6279808
CG4068-RD	CG44774	43,1667	0,00803915	34,0089	0,0089801	18,7444	23,9312	0,489432	0,112727	0,6279808
CG4068-RE	CG40682	2,08771	1,94917	4,50972	0	4,0795	6,20955	0,491734	0,110728	0,6279808
CG4069-RA	CG4069	5,15759	4,097	11,9568	8,5585	0,0707485	7,43228	-0,133193	0,643407	0,6279808
CG40709-RA	Tis11	26,8079	37,5205	26,3595	39,2624	19,3015	15,5816	NA	NA	0,6279808
CG4070-RA	Tis11	18,8212	35,3556	24,325	34,2775	12,3163	14,2529	0,264483	0,333909	0,13772387
CG4070-RB	CG40709	0	0	0,186213	0	0	0	0,280339	0,308778	0,6279808
CG40715-RA	Vps20	29,0997	38,9843	46,0769	38,215	1,59867	49,6492	NA	NA	0,13772387
CG40719-RA	Vps20	0,090556	0,0824847	0,0869392	0,113504	1,19958	0,11587	NA	NA	0,6279808
CG4071-RA	CG40715	0	0	0	0	0	0	-0,10186	0,704294	0,6279808
CG4071-RB	lncRNA:CR40719	0	0	0	0	0	0	-0,10186	0,704294	0,6279808
CG40733-RA	CG4073	0	0,0904244	0	0,0523981	2,19581	2,71876	-0,041811	0,731452	0,13772387
CG4073-RA	RYa	0	0,612585	0,242125	0,367249	0	0	NA	NA	0,13772387
CG40748-RB	CG4074	5,76676	3,25332	7,03663	5,6656	5,30046	6,04992	NA	NA	0,6279808
CG4074-RA	CG40748	0	0	0	0	0	0,579366	0,031632	0,913753	0,13772387
CG40754-RA	CG40754	0	0	0	0	1,48935	0	NA	NA	0,6279808
CG40758-RA	CG40758	0	0	0	0	0	0	NA	NA	0,6279808
CG40769-RA	Nufip	3,84538	2,44415	6,16652	31,7998	2,14471	2,86954	-0,811967	0,019489	0,6279808
CG4076-RA	CG40769	3,10126	0,206118	0	1,32501	0	0,4284	0,71046	0,02012	0,6279808
CG4078-RA	CG4078	3,10793	2,94014	7,67455	3,09537	2,99539	0,621612	-0,114547	0,620771	0,6279808
CG40793-RA	Taf11	10,1864	9,16106	16,0517	15,766	729,926	0,844965	-0,178748	0,425322	0,6279808
CG40794-RA	CG40793	0	0,066431	0,140037	0,184143	0	0,140528	NA	NA	0,6279808
CG40798-RA	CG40794	0	0,0706414	0	0	0,33848	0	NA	NA	0,6279808
CG4079-RA	CG40798	0,393309	0	0	0	0	396,394	-0,405687	0,139489	0,6279808
CG40801-RA	CG4080	6,32616	6,69935	8,97969	12,766	583,116	131,831	NA	NA	0,13772387
CG4080-RA	CR40801	0	0,128238	0	0,317448	0	0	-0,278172	0,229855	0,6279808
CG40813-RA	CG40813	1,69821	0,374994	0	0	1,10659	1,34931	NA	NA	0,6279808
CG40818-RA	CG40818	0	0,374994	0	2,27209	1,91059	0	NA	NA	0,6279808
CG40820-RA	Mcm5	4,08634	34,8034	7,60267	43,4041	2,25001	9,29385	NA	NA	0,6279808

gene_id	Symbol	W1_FPKM	W2_FPKM	W3_FPKM	MCM51_FPKM	MCM52_FPKM	MCM53_FPKM	FC	p-value	p-adj
CG4082-RA	Su(Ste):CR40820	0	0	0	0	0	0	0,452261	0,109117	0,6279808
CG4083-RA	Mo25	26,0934	23,8477	5,17533	29,467	9,64026	0,0769161	0,11371	0,550922	0,13772387
CG40844-RA	Alg3	3,81134	4,91529	6,06834	32,4435	0,95305	4,75348	-0,013096	0,914398	0,6279808
CG40849-RA	CG40844	0,611814	0,891652	3,53167	6,53218	7,25025	4,21087	-0,060979	0,660487	0,13772387
CG4084-RA	CG40849	0	0	0	0	0	0	-0,025856	0,910566	0,13772387
CG40856-RA	CG40856	0	0	0	0	0	0	NA	NA	0,13772387
CG40858-RA	CG40858	0	0	0	0,266308	0	0	NA	NA	0,6279808
CG40862-RA	Su(P)	17,4328	0,0164369	0,0529227	6,83763	39,8794	10,5848	NA	NA	0,6279808
CG40867-RA	CG40862	2,52373	0,626943	0	10,7805	12,1686	0	NA	NA	0,6279808
CG4086-RA	CG40867	0	0	0	0	0	0	-0,634254	0,047584	0,6279808
CG40871-RA	RpLP1	1715,62	4,33844	6,15866	8,15743	4,45879	3,19098	NA	NA	0,6279808
CG40878-RA	CG40871	0	0	0	0	0	0	NA	NA	0,13772387
CG4087-RA	CG40878	0	0	0	0	0	0	-0,410449	0,144589	0,13772387
CG40894-RA	TTLL15	13,2904	0,0920749	0,0599592	14,9791	0,203527	7,72328	NA	NA	0,6279808
CG4089-RA	CG40894	1,49747	2,20194	0,352427	3,29563	5,03526	4,60013	0,292004	0,260547	0,6279808
CG40908-RA	Mur89F	0,0780055	0	3,69574	6,80828	3,37544	6,9764	NA	NA	0,6279808
CG4090-RA	CG40908	0	0	0	0	0	0	-0,808565	0,022531	0,6279808
CG40910-RA	sigmar	12,9544	19,338	12,3615	2,94945	0,10288	7,64543	NA	NA	0,6279808
CG4091-RA	sigmar	15,0019	25,1042	13,3603	14,0562	137,352	7,67401	0,439619	0,119489	0,6279808
CG4091-RB	sigmar	0,0348746	0,0317662	0,0334817	0,0360552	0,208276	0,0385356	0,450696	0,108222	0,6279808
CG4091-RC	CG40910	0	0,216498	0	0	1,00516	0	0,437394	0,121187	0,6279808
CG40923-RA	CG40923	0	0	0	0	0	0,0393913	NA	NA	0,6279808
CG40924-RA	CG40924	0	0	0,18229	7,80469	2,87689	0	NA	NA	0,6279808
CG40928-RA	CG40928	3,05393	0,5115	1,88693	3,82967	2,79526	5,45706	NA	NA	0,6279808
CG40930-RA	PRY	0	0	0	0	0	0	NA	NA	0,6279808
CG40936-RA	kl-3	0	0	0	1,38016	3,45277	4,40427	NA	NA	0,6279808
CG40947-RA	Fum1	51,6047	14,8913	0,0479855	0,058857	28,6502	0,024482	NA	NA	0,6279808
CG40948-RA	Fum1	0,0401514	9,26759	83,4549	75,1398	0,0261923	0,0249081	NA	NA	0,6279808
CG40949-RA	lncRNA:CR40947	0	0	0	0	0	0	NA	NA	0,6279808
CG4094-RA	CR45933	0	0	0	0	0	0	-0,802	0,008176	0,6279808
CG4094-RB	CG40949	0	0,293593	0	0	0	0	-0,797478	0,00863	0,6279808
CG40950-RA	Fum2	0,0429157	5,3207	0,0385477	0,0440299	140,073	0,0249635	NA	NA	0,13772387
CG40955-RA	CG40950	0,0895337	0	0,295817	1,04288	0	0,748865	NA	NA	0,13772387
CG40958-RA	CG40955	0	0	0	0	0	0	NA	NA	0,6279808
CG4095-RA	CG40958	0	0	0	0	0	0	-0,34956	0,250854	0,6279808
CG40968-RA	CG4096	3,58361	4,9705	1,29518	0,693839	8,64206	9,25865	-0,041811	0,731452	0,6279808
CG4096-RA	CR45934	0	0	0	0,444778	0	0,343126	0,050377	0,810294	0,13772387
CG40971-RA	Prosbeta6	30,6036	132,279	95,609	180,326	0,0964329	47,1824	NA	NA	0,13772387
CG40972-RA	CG40971	0	0	0,240291	0,929918	0	0,254791	-0,106269	0,42424	0,13772387
CG40973-RA	CG40972	0	0	0	0	0	0	NA	NA	0,6279808
CG4097-RA	CG40973	0	0	0	0	0	0	-0,117716	0,674506	0,6279808
CG4098-RA	CG4098	5,08971	3,43219	85,5056	0,075346	1,82127	2,06799	0,201057	0,512026	0,13772387
CG40992-RA	Sr-CI	8,87732	0,0468986	0,158989	0	0,0218029	0	NA	NA	0,6279808
CG40995-RA	kl-3	0	0,271422	0,681723	1,42216	0	0,862293	NA	NA	0,6279808
CG4099-RA	CG40995	0	0	0	0	0	0	-0,280997	0,290247	0,13772387
CG41010-RA	Dsim\GD14652	20,4183	12,6349	3,33498	5,96662	13,5866	11,3045	-0,041811	0,731452	0,6279808
CG41012-RA	CG41010	0	0	0,166065	0,711513	0	0,411033	NA	NA	0,6279808
CG41015-RA	CG41012	0,108085	0,472669	0,262136	0,545955	0	0,443925	NA	NA	0,6279808
CG41018-RA	CG41015	0	0	0	0	0	0	-0,041811	0,731452	0,13772387
CG4101-RA	CG41018	0,669688	0,406665	0	0	0,431126	0,803137	0,163674	0,456788	0,13772387
CG41020-RA	WDY	0	0	0,0446738	0,698371	0	0	-0,452352	0,064754	0,13772387
CG41026-RA	CG41026	0	0	0	0	1,28382	0	-0,041811	0,731452	0,13772387
CG41027-RA	CG41027	0	0	0	0	0,546804	0,239629	0,078037	0,549183	0,13772387
CG41038-RA	THG	5,44877	15,4646	11,4365	5,62661	21,3833	0,161881	NA	NA	0,6279808
CG4103-RA	CG41038	0	0	0	0	0	0	0,33447	0,234024	0,6279808
CG41040-RA	Tps1	22,2241	24,6301	2,32648	0,0313245	2,31936	0	NA	NA	0,6279808
CG41041-RA	CG41040	0	0,130274	0	0	0	0	0,044335	0,715924	0,13772387
CG41042-RA	CG41041	0,143021	0	0	0	0	0	0,044335	0,715924	0,13772387
CG41049-RA	CG41042	0	0	0	0,140145	0	0	NA	NA	0,13772387
CG4104-RA	CG41049	0	0,235103	0,2478	0,543949	0	0	-0,867287	0,000266	0,13772387
CG41050-RA	Cyp4e3	0,0626906	0,0951715	104,433	0,421424	17,3305	81,9401	0,126622	0,510433	0,13772387
CG41051-RA	CG41050	0	0	0	0	0	0	NA	NA	0,6279808
CG41053-RA	CG41051	0	0	0	0,0230188	0,0311791	0,0234984	NA	NA	0,13772387
CG41057-RA	CG41053	0,161634	0,235564	0	0	0	0	NA	NA	0,6279808
CG41059-RA	CG41057	0	0	0	0	0	0	0,01562	0,897983	0,6279808
CG4105-RA	CG41059	0,333717	0	0,186214	0,322828	0,130687	0,267308	-0,133786	0,690778	0,6279808
CG41066-RA	CG41066	0,344145	0,0515883	0,530408	0,84706	1,14735	0,864711	NA	NA	0,6279808
CG41069-RA	CG41069	0	0	0	0	0	0	NA	NA	0,13772387
CG41072-RA	Gcn5	9,33016	12,3031	12,8319	14,3621	85,3497	7,95501	-0,041811	0,731452	0,6279808
CG41073-RA	CG41072	0	0	37,0452	0	0	0	NA	NA	0,6279808
CG41074-RA	CG41073	0	0	0	0	0	0	NA	NA	0,6279808
CG41075-RA	CG41074	0	0	0	0	0	0	-0,221932	0,409285	0,6279808
CG4107-RA	CG41075	0,0871714	0	0	0,0200065	0,0270989	0,0204233	0,111345	0,615977	0,6279808
CG41087-RA	Chmp1	66,5836	0	0,521009	0,488674	0,0728267	0,249726	0,001131	0,997188	0,6279808
CG41089-RA	CG45782	0,18457	0	0,767857	0,932879	0,644643	0,485842	NA	NA	0,6279808
CG4108-RA	CG41089	0	0	0,0794947	0	0,585225	0,170723	-0,285543	0,252195	0,6279808
CG41099-RA	Syx8	9,18616	12,8447	11,9911	13,7417	646,453	102,597	0,767749	0,002674	0,6279808
CG41099-RB	CG41099	12,5514	5,64302	11,9592	15,19	9,81863	5,9897	0,604192	0,019459	0,13772387
CG41099-RC	CG41099	28,11	22,2739	18,4969	0,0256336	0,0347209	0,0261677	0,603681	0,019622	0,6279808
CG41099-RD	CG41099	8,6665	6,37989	7,14583	10,3208	9,57966	6,35922	0,602315	0,019898	0,6279808
CG4109-RA	CG41099	0,01829	0,0166598	0,0175595	0,0190245	0,0257688	0,0194209	-0,085789	0,731794	0,6279808
CG41106-RA	CG41106	0	0,0523512	0	0	0	0,13337	0,012562	0,923862	0,13772387
CG41113-RA	RpL35	1419,93	20,0222	59,7903	456,572	56,4804	53,5154	NA	NA	0,6279808
CG4111-RA	RpL35	254,915	9,49039	2593,63	103,755	2666,51	3172,2	-0,264674	0,335384	0,6279808
CG4111-RB	CG41113	0	0	0	0	0	0	-0,263229	0,337853	0,13772387
CG41123-RA	CG41123	0	13,9264	0	11,5174	14,1168	0	0,044335	0,715924	0,13772387
CG41124-RA	CG41124	1,35772	159,378	1,01383	9,07175	285,832	0,0281381	NA	NA	0,13772387
CG41125-RA	CG41125	0	0,0236986	0	0	0,0373352	2,54908	0,044335	0,715924	0,13772387
CG41126-RA	CG41126	0,403722	0	0	0	0	0	NA	NA	0,6279808
CG41127-RA	CG41127	0	0	25,8822	0	0	0	0,01562	0,897983	0,13772387
CG41128-RA	CG41128	418,02	6,63372	258,439	556,584	6,88069	0	-0,300595	0,347551	0,13772387
CG41129-RA	CG41129	0,347311	0,237266	0,708558	0,558739	0,386103	0,180651	0,255505	0,456426	0,13772387
CG41130-RA	Mitf	1,46835	265,851	1,64466	49,2988	2,40096	1,32853	0,071515	0,836048	0,6279808
CG41133-RA	CG41133	0,0426021	0,038805	0,0409006	13,6041	6,7598	6,14572	NA	NA	0,6279808
CG41133-RB	CG41133	0,196654	0,179126	0,1888	6,53133	2,62644	2,03706	-0,140487	0,347789	0,6279808
CG41138-RA	CG41138	0,722398	0	3,65242	1,91175	0	1,82275	-0,094629	0,728782	0,6279808
CG41138-RB	CG41138	0,118912	0	0,693545	0,462396	0	0,419873	-0,256306	0,303853	0,6279808
CG4114-RA	ex	9,26091	0,0144725	11,2717	13,4702	36,1168	44,5432	0,205068	0,461919	0,13772387
CG4115-RA	CG4115	526,583	1,51289	0,0836897	321,968	457,303	17,5447	0,54369	0,012901	0,13772387
CG4116-RA	CG4116	0	26,0211	0,199487	28,2277	0	0	-0,165719	0,385555	0,6279808
CG4118-RA	nxf2	0,673327	64,0264	62,9123	1,4716	99,9895	20,7929	0,236371	0,469926	0,13772387
CG4118-RB	nxf2	0,92342	12,1754	28,3474	0,722602	30,1912	470,776	0,234925	0,473444	0,13772387
CG4119-RA	CG4119	8,51732	3,23558	163,355	4,39861	133,008	141,196	-0,118103	0,599211	0,13772387
CG4120-RA	Cyp12c1	4,77805	2,04589	0,0447473	0,208583	1,87675	0,169485	-0,430828	0,222905	0,6279808

gene_id	Symbol	W1_FPKM	W2_FPKM	W3_FPKM	MCM51_FPKM	MCM52_FPKM	MCM53_FPKM	FC	p-value	p-adj
CG41229-RA	svr	5,37635	6,40247	3,63683	7,12583	0,030466	0,022961	NA	NA	0,13772387
CG4122-RB	svr	0,0113266	0,0103171	0,0108743	0,0115914	0,0157007	0,011833	-0,082585	0,706334	0,6279808
CG4122-RC	svr	0,0114483	0,0104279	0,0109911	0,0117192	0,0158738	0,0119634	0,024566	0,914159	0,6279808
CG4122-RD	svr	16,2962	17,2592	16,2921	21,6343	18,6199	13,8875	0,027303	0,905503	0,13772387
CG4122-RE	svr	0,0113053	0,0102977	0,0108538	0,011569	0,0156703	0,0118101	0,126779	0,571441	0,13772387
CG4122-RF	svr	0,0114265	0,0104081	0,0109702	0,0116964	0,0158428	0,0119401	0,011899	0,956705	0,13772387
CG4122-RG	svr	0,0152366	0,0208178	0,014628	0,0157352	0,0213135	0,0160631	-0,082585	0,706334	0,13772387
CG4122-RH	svr	9,9546	9,21051	8,36867	10,0397	6,53505	6,09844	0,027303	0,905503	0,6279808
CG4122-RI	CG41229	0	0,560396	0	0	0	0	-0,122624	0,593507	0,13772387
CG41232-RA	Mipp1	29,2421	1,03706	3,58477	38,5276	1,02765	53,7248	-0,070526	0,562659	0,13772387
CG41233-RA	Mipp1	57,8951	0,021567	0,0227318	48,6713	0,0337876	32,7283	NA	NA	0,6279808
CG41234-RA	CG41232	0	0	0	0,571969	0,116284	0,0876389	0,044335	0,715924	0,13772387
CG41238-RA	CG41233	0	0,382866	0	0,367249	0	0	NA	NA	0,13772387
CG41239-RA	CG41234	0,190971	0,163107	0	0	0	0	NA	NA	0,6279808
CG4123-RA	Pzl	0	0	0	0	0	0	-0,090119	0,687975	0,6279808
CG4123-RB	CG41239	1,66229	1,51413	0,59846	0	0	0	-0,089843	0,689081	0,13772387
CG41240-RA	CG41240	0,27763	0,252884	0	0	0	0	0,115708	0,573908	0,6279808
CG41242-RA	CG41242	0	0	0,226022	0	0	0,383002	-0,041811	0,731452	0,13772387
CG41243-RA	CR41243	0	0	0,430175	0	0	0	NA	NA	0,6279808
CG41245-RA	CG41245	0	0	0	0	0	0	NA	NA	0,6279808
CG41246-RA	CG41246	0	0	0	0	0	0	NA	NA	0,6279808
CG41247-RA	CG41247	0	0	0	0	0	0	0,01562	0,897983	0,13772387
CG41248-RA	CG41248	0	0	0	0	0	0,317727	NA	NA	0,13772387
CG41249-RA	CG41249	0	0,177019	0	0,0228908	0,0310057	0,0233678	0,01562	0,897983	0,13772387
CG41250-RA	rst	12,8254	14,8196	12,5774	21,8897	3,20392	0,0375462	NA	NA	0,13772387
CG41251-RA	CG41250	0	0	0	0,0233615	0,0316433	0,0238483	-0,159558	0,466079	0,13772387
CG41252-RA	CG41251	0,922848	0,504356	0,885989	4,4041	5,96538	8	0,014671	0,958589	0,13772387
CG41253-RA	CG41252	0,359108	0	0	10,4534	0,035793	0,177236	NA	NA	0,6279808
CG41254-RA	CG41253	1,28719	1,85177	0,146617	4,52815	2,84652	4,6225	NA	NA	0,6279808
CG41255-RA	CG41254	0,14882	0,27111	0,119993	0	0,314794	78,3088	0,130803	0,471701	0,13772387
CG41257-RA	CG41255	0	0	29,4284	0,0264252	3471,5	3779,56	1,346097	0,000151	0,13772387
CG41258-RA	lncRNA:CR41257	3,61112	45,1095	0,0857638	16,8898	0	0,237248	NA	NA	0,13772387
CG4125-RA	CG41258	0	0	0	0	0	0,669778	0,359476	0,206077	0,6279808
CG41262-RA	CG41262	0	0	0	0	0	0	0,469744	0,164257	0,13772387
CG41265-RA	l(2)41Ab	3,37091	1,82373	1,78104	0	0,876884	1,1051	-0,15207	0,623145	0,13772387
CG41265-RB	l(2)41Ab	0,024083	0,0219365	0,0231212	1,77415	0	444,308	-0,269382	0,396043	0,13772387
CG41266-RA	CG41266	0	0	54,7165	0	0,572005	335,051	NA	NA	0,6279808
CG41267-RA	CG41267	0	0	0,379104	0	0,0675363	0,0508995	0,326838	0,290891	0,13772387
CG41268-RA	CG41268	0	0	40,4606	292,9	1,87362	1,9226	-0,013096	0,914398	0,6279808
CG41272-RA	CG41272	0	0	0,320388	0,788598	0,457663	0	0,099493	0,503366	0,13772387
CG41274-RA	CG41274	0	36,7566	0,113686	0,491816	19,45	0,256137	-0,160539	0,571669	0,6279808
CG41276-RA	CG41276	0	0	0	42708,9	3,53943	0,0475194	NA	NA	0,13772387
CG41277-RA	CG41277	0,342954	0	0,878018	1,07877	72,6943	0	-0,041811	0,731452	0,6279808
CG41278-RA	CG41278	0,312662	0,142397	0,550319	1,44714	56219,6	1,43628	NA	NA	0,6279808
CG41279-RA	CG41279	0	0	0	0	0	0	NA	NA	0,13772387
CG41280-RA	nAChRalpha6	0,0220057	0,0200443	0,0211268	0,0227329	0,0307918	0,0232066	NA	NA	0,13772387
CG41281-RA	nAChRalpha6	11,674	10,9642	14,0493	5,71551	3,4354	4,55509	-0,232837	0,469456	0,6279808
CG41283-RA	nAChRalpha6	0,0220057	3,27725	2,66198	0,0357423	0,0484132	0,0364871	0,391874	0,228216	0,6279808
CG41284-RA	nAChRalpha6	0,0213861	0,0194799	0,0205319	122,266	132,426	123,22	-0,42879	0,203437	0,13772387
CG41286-RA	nAChRalpha6	0,0216808	0,0197484	0,0208149	0,027808	0,0376661	0,0283875	NA	NA	0,13772387
CG41287-RA	nAChRalpha6	9,01034	6,71727	9,25246	0,0277261	0,0375552	0,0283039	0,01562	0,897983	0,13772387
CG4128-RA	CG41280	0	0,316771	22,4422	0	0,34063	0	0,600626	0,001219	0,6279808
CG4128-RB	Myo81F	0,30403	0,923106	0	2,86591	1,72115	0	0,595853	0,001279	0,13772387
CG4128-RC	CG41283	0,257105	21,4658	0,205696	1,83769	2,01079	0,055751	0,599842	0,001232	0,13772387
CG4128-RD	CG41284	2,21002	1,91068	30,8948	0,054613	0,0739736	0	0,599571	0,001252	0,6279808
CG4128-RE	CG41286	0	0,188475	0	0	0	0	0,595772	0,001298	0,13772387
CG4128-RF	CG41287	0	0	0	0	0	0	0,632256	0,000988	0,6279808
CG41293-RA	CG41293	0,186303	20,0015	1,80511	9,66457	4,13552	0,0373585	0,065612	0,850548	0,13772387
CG41297-RA	CG41297	85,8486	79,5647	65,8397	0	20,0684	24,3628	4,205315	3,5E-36	0,13772387
CG41298-RA	CG41298	0	0	0,449906	0,024954	0,0338004	0,025474	-0,041811	0,731452	0,13772387
CG41300-RA	CG41300	0	0	0	0,0204346	0,0276787	0,0208604	NA	NA	0,13772387
CG41310-RA	CG41310	0	0	0	0	0	0	0,007789	0,980915	0,6279808
CG41311-RA	CG41311	0	0	0	0,241786	0	0	0,01562	0,897983	0,13772387
CG41312-RA	CG41312	0	0	0	0,55299	0	0	-0,013096	0,914398	0,13772387
CG41314-RA	CG41314	0	0	0	0	0	0	NA	NA	0,13772387
CG41316-RA	CG41316	0	0	0	2,98478	3,08428	2,24226	NA	NA	0,13772387
CG41318-RA	CG41318	0	0	0	0	0	0	-0,074864	0,550506	0,13772387
CG41320-RA	pkaap	17,1218	0,733982	1,49566	3,46103	2,99977	29,0554	-0,391075	0,09121	0,13772387
CG41322-RA	CR41320	2,74228	2,91417	1,06564	4,83553	10,9664	6,22857	-0,094233	0,640845	0,13772387
CG41323-RA	CG41322	1,1152	0,634879	0,736081	2,60675	0,49306	21,9494	-0,013096	0,914398	0,6279808
CG41325-RA	CG41323	0	0	0,104166	35,9646	34,9093	18,3442	-0,041811	0,731452	0,13772387
CG41326-RA	CG41325	0	0	0	0	0	0	0,01562	0,897983	0,13772387
CG41327-RA	CG41326	0	0	0,3304	0	0	0	-0,150262	0,488993	0,13772387
CG41328-RA	CG41327	0,347768	0	0,333878	1,121	0	1,84332	0,117339	0,538151	0,6279808
CG41329-RA	CG41328	0	0,0233855	0,0246484	0	0	0	-0,070526	0,562659	0,6279808
CG4132-RA	CG41329	0	0	0	0,695325	0	0,709814	0,059134	0,765335	0,6279808
CG41332-RA	CG4133	9,62871	22,1292	9,77721	10,966	5,46499	6,69897	0,204702	0,340425	0,13772387
CG41335-RA	CG41332	0,288289	0,262594	0,276775	0,424254	0,601025	0,226926	0,62602	0,049291	0,6279808
CG41336-RA	CG41335	0,0707451	0,371522	0,181119	0,17769	2,42719	1,01335	NA	NA	0,6279808
CG41337-RA	CG41336	1,6315	287,869	0,391585	0	1146,18	274,245	NA	NA	0,6279808
CG41339-RA	CG41337	0	166,119	0,22819	0,742085	288,381	2,94658	0,062601	0,855264	0,6279808
CG4133-RA	CG41339	0,21097	5,35424	0,243053	0,454127	5,44778	4,66342	0,293409	0,193431	0,6279808
CG41343-RA	CG45782	0,931656	0,391669	0,550428	2,90781	0,142342	1,59907	-0,949593	0,00786	0,6279808
CG41344-RA	CG41344	0	0	0	0	0	0,302276	NA	NA	0,6279808
CG41346-RA	CG41346	0	0	0	0	0,161686	0	-0,053683	0,691569	0,6279808
CG41347-RA	CG41347	0,21021	0,0957367	0,168178	0,287588	0	0	0,068231	0,809228	0,13772387
CG41348-RA	CG41348	0	0,374294	0	1,06704	0,0731851	0	0,037163	0,88056	0,13772387
CG4135-RA	beat-IIb	3,80719	0,0907941	0,0948606	1,05152	10,7563	0	-0,713225	0,001444	0,13772387
CG41360-RA	Vsx1	2,14863	2,34047	2,39244	0,0169714	0,0229878	0,246257	NA	NA	0,13772387
CG41363-RA	CG41360	0	0	0	0	0	0	NA	NA	0,13772387
CG41367-RA	CG41363	0,310215	21,1036	0	30,3941	18,8144	67,5863	NA	NA	0,13772387
CG41369-RA	PRY	0	0	0	0	0	0	NA	NA	0,6279808
CG4136-RA	Pzl	0	0	0	0	0	0	0,429456	0,086368	0,6279808
CG41370-RA	CG41370	0	0	0	0	0	0,799269	NA	NA	0,6279808
CG41373-RA	CG41373	23,3035	23,376	12,272	28,4199	27,8317	18,1283	-0,072192	0,612286	0,13772387
CG41374-RA	CG41374	0	0	0	0	0,437656	0	NA	NA	0,13772387
CG41378-RA	CG41378	16,9154	25,0376	0	34,093	14,2954	27,1947	0,191144	0,440001	0,6279808
CG41389-RA	CG41389	0	0,10559	0	0	0	0	0,01562	0,897983	0,6279808
CG41392-RA	Karl	0,0647378	20,7354	17,1328	5,32313	5,05433	10,4884	0,01562	0,897983	0,6279808
CG41395-RA	Karl	25,3417	7,90856	27,5199	16,4517	23,754	19,2277	0,01562	0,897983	0,6279808
CG4139-RA	CG41392	0,311679	0,170339	7,11723	0,28501	0	0,308063	-0,251643	0,352625	0,13772387
CG4139-RB	CG41395	0	0	0,174277	0	0	0	-0,130579	0,635964	0,6279808
CG4140-RA	l(2)35Be	6,56472	4,09791	1474,2	7,70091	9,27834	2110,07	-0,290708	0,35859	0,6279808
CG41418-RA	Pi3K92E	4,60269	11,3493	1,42437	14,7892	8,62998	1,55213	NA	NA	0,6279808

gene_id	Symbol	W1_FPKM	W2_FPKM	W3_FPKM	MCM51_FPKM	MCM52_FPKM	MCM53_FPKM	FC	p-value	p-adj
CG4141-RA	Pi3K92E	7,55985	7,01558	4,70851	4,88718	3,66317	2,54923	-0,017851	0,942661	0,6279808
CG4141-RB	CG41418	0	0	0	0	0	0	-0,018858	0,939443	0,6279808
CG41421-RA	CG41421	112,465	165,513	151,857	784,699	269,512	0	NA	NA	0,6279808
CG41423-RA	CR41423	0	0	0	0	0	0,324063	NA	NA	0,6279808
CG41425-RA	CG41425	0	0	0	0,151992	0,205874	0,155159	NA	NA	0,6279808
CG41427-RA	CG41427	0	0	0,153229	0	0	0	NA	NA	0,6279808
CG41428-RA	CG41428	0	0	0	0,340865	0	0	NA	NA	0,6279808
CG41429-RA	CG41429	0	0	0	0	0	0	NA	NA	0,6279808
CG41430-RA	mbf1	80,1124	101,847	93,7703	131,345	38,1041	75,3039	-0,514499	0,074029	0,6279808
CG41434-RA	mbf1	9,80684	12,7971	13,075	0,0711942	63,8381	12,1974	-0,24581	0,492096	0,13772387
CG4143-RA	-	0,0867138	0,143301	0,654507	0,105088	0	0,107278	0,056642	0,771347	0,6279808
CG4143-RB	CG41434	0,527763	2,71361	1,93427	0	0	0	0,061239	0,753201	0,6279808
CG4144-RA	GNBP2	3,89384	14,2688	3,46779	24,4592	25,2631	27,4456	-0,1326	0,668534	0,6279808
CG4144-RB	GNBP2	0,0476165	2,10007	0,0457147	2,79296	0	3,28699	-0,1326	0,668534	0,13772387
CG4144-RC	GNBP2	0,0480668	0,0433724	0,046147	0,0532019	0	0,0543105	-0,128407	0,678235	0,13772387
CG4144-RD	GNBP2	1,69522	0,0437825	2,56062	0,0537663	3,69933	0,0548866	-0,130762	0,674011	0,13772387
CG41451-RA	Col4a1	304,554	0,0102173	140,964	0,0114763	0,0155448	169,202	NA	NA	0,13772387
CG41452-RA	Col4a1	0,0112899	1124,64	1960,34	2075,48	2631,4	2691,76	-0,278475	0,415489	0,6279808
CG41454-RA	Col4a1	0,011217	0,0936509	0,0987084	0,133513	0,180845	0,136296	-0,322563	0,193042	0,6279808
CG4145-RA	CG41451	0	0	6,38878	5,41381	0,0445469	155,223	-0,115736	0,523156	0,13772387
CG4145-RB	nrm	0,825261	1,00227	0,97717	0,0498605	0,0675363	0,0508995	-0,115726	0,523193	0,13772387
CG4145-RC	CR41454	21,528	14,2005	21,7254	6,94879	30,0592	14,5545	-0,115611	0,523606	0,13772387
CG41473-RA	Hsc70-3	129,436	45,8213	51,4388	0,0416109	12,4447	6,06332	0,01562	0,897983	0,13772387
CG41476-RA	Hsc70-3	0,0210455	1,60422	6,22298	13,9876	15,7491	63,9397	0,149653	0,578833	0,13772387
CG4147-RA	Hsc70-3	0,0222203	6,2078	8,14391	10,7009	17,0874	9,17531	-0,158799	0,581947	0,13772387
CG4147-RB	Hsc70-3	27,3658	9,12068	0,494056	8,00149	10,6657	0,0424779	-0,163493	0,570998	0,6279808
CG4147-RC	CG41473	0	0,0252566	0,1652	0,0302183	0,0409309	0,0249173	-0,15885	0,581861	0,13772387
CG4147-RD	CG41476	0,0983272	0,0229369	0,0266206	0,0272109	0,0368573	4,27489	-0,158967	0,581533	0,6279808
CG41489-RA	wek	4,69979	14,1784	6,95931	0	0	0	NA	NA	0,6279808
CG4148-RA	CG41489	0	0	0	0	0	0	0,037413	0,873496	0,13772387
CG41490-RA	CG41490	0	0,208981	0	25,9038	0	0	NA	NA	0,13772387
CG41491-RA	CG41491	0	0	0	0,529501	0	0	NA	NA	0,13772387
CG41492-RA	CG41492	0	0	0	0	0	0	NA	NA	0,6279808
CG41493-RA	CG41493	0	0	0	0,30788	13,3631	0,320563	NA	NA	0,13772387
CG41494-RA	CG41494	0	0	0	0	0	0	NA	NA	0,6279808
CG41495-RA	CG41495	0	0	0	0	0	0	NA	NA	0,6279808
CG41495-RB	CG41495	0	0	0	0	0	0	NA	NA	0,6279808
CG41496-RA	CG41496	0	0	0	0	0,0368117	0,502223	NA	NA	0,13772387
CG41497-RA	kl-5	0	0	0	0,441405	0	0	NA	NA	0,6279808
CG41498-RA	CG41498	0	0	0	0	0	0	NA	NA	0,13772387
CG41500-RA	CG41500	0,191524	0,468986	0,659084	1,70814	0,732786	1,18998	NA	NA	0,13772387
CG41511-RA	CG4151	0	0	0,127897	22,521	0,0254165	9,96155	0,129093	0,718124	0,6279808
CG41517-RA	CG41511	0,60069	1,0943	1,87427	2,28269	1,77962	1,54327	-0,279862	0,347609	0,13772387
CG41518-RA	CG41517	0	0	0	4,0275	0	6,62265	-0,013096	0,914398	0,6279808
CG41519-RA	Myo81F	0	0,815535	1,6332	0	0	0,513212	NA	NA	0,13772387
CG41519-RB	CR41519	0,298986	0,090779	0,0956814	0,273887	0,375248	0,101455	-0,017422	0,917755	0,13772387
CG4151-RA	CR41519	0,420269	0,127603	0,403483	0,490069	0,32285	0,229415	-0,22465	0,292294	0,6279808
CG41520-RA	Mtr4	4,4693	0,403861	0,744924	1,62937	1,73524	0,783369	0,299273	0,229247	0,6279808
CG41520-RB	CG41520	22,759	0,367738	0,129199	0,0271772	0,430497	11,0855	0,299338	0,228987	0,6279808
CG41521-RA	CG41520	0,0256738	23,8527	19,3214	0	0	0,0277435	NA	NA	0,6279808
CG41522-RA	CG41521	0	0	0	0,18123	0	0	-0,041811	0,731452	0,6279808
CG41524-RA	CG41522	0	0,282566	0	2,73626	0	0	-0,041811	0,731452	0,6279808
CG41526-RA	CG41524	0	0	0	0,423283	0	0	NA	NA	0,13772387
CG41527-RA	CG41526	0	0	1,48217	0,362228	0	0	-0,179302	0,354302	0,6279808
CG41529-RA	Myo81F	0	0	0	1,07353	0	0,680349	0,020837	0,894955	0,6279808
CG41529-RB	CG41529	0	0	0,306128	0,268272	0	0	0,01562	0,897983	0,6279808
CG4152-RA	CG41529	0	0	0,104051	0,0993841	0	0	-0,414962	0,124179	0,13772387
CG41533-RA	eIF2beta	118,105	110,651	192,181	162,957	24,2751	28,8745	NA	NA	0,13772387
CG41534-RA	Su(Ste):CR41533	0,288962	0	0	0,538443	0	0,926774	NA	NA	0,6279808
CG41536-RA	Pp1-Y1	0	0	0,217249	18,875	0,915615	10,5234	NA	NA	0,13772387
CG41537-RA	CG41536	119,476	162,194	162,617	140,774	1633,07	2306,13	NA	NA	0,13772387
CG41537-RB	CG41537	0	0	0	0,184563	0	0	NA	NA	0,13772387
CG41538-RA	CG41537	0	0	0	0	0,585225	0	-0,202384	0,308879	0,6279808
CG4153-RA	CG41538	0	3,45899	6,74472	0	7,18545	0	-0,201577	0,425214	0,13772387
CG41541-RA	Gyc88E	0,0190768	0,0174825	0,0183149	0,0198797	0,0269272	0,0202939	NA	NA	0,13772387
CG41542-RA	Gyc88E	0,0191932	0,952956	0,0184266	0,0200065	0,0270989	0,0204233	0,01562	0,897983	0,13772387
CG41545-RA	Gyc88E	0,916444	3,2171	1,01999	1,8198	1,55987	1,17561	NA	NA	0,13772387
CG41549-RA	CG41541	4,02902	3,09783	2,8366	0	0,41899	4,24215	NA	NA	0,13772387
CG4154-RA	CG41542	0,693104	0,210442	0,887228	0,894318	0	0,81851	-0,899467	0,000873	0,6279808
CG4154-RC	CG41545	0	0	0	0	5,26222	0	-0,899467	0,000873	0,6279808
CG4154-RD	CG41549	0	0	0	0	0	0	-0,719652	0,003653	0,6279808
CG41551-RA	CG41551	0	0	0	0	0,433929	0	NA	NA	0,6279808
CG41552-RA	CG41552	0	0	0	0	0	0	NA	NA	0,13772387
CG41553-RA	CG41553	0	0	0	0	0	0	NA	NA	0,13772387
CG41554-RA	CG41554	0	0	0	0	0	0	NA	NA	0,6279808
CG41555-RA	CG41555	0,234581	0	0	0	0,608496	0	-0,166395	0,305573	0,13772387
CG41556-RA	CG41556	0	0	0	0	0	0	NA	NA	0,13772387
CG41557-RA	CG41557	0	0	0	0	0	0	NA	NA	0,6279808
CG41558-RA	CG41558	0	0	0	0	0,761623	0	NA	NA	0,13772387
CG41559-RA	CG41559	0,426847	0,388802	0,491758	1,06105	0,579935	1,00116	NA	NA	0,13772387
CG41560-RA	CG41560	0,227325	0,138042	0	0,631584	0,42997	0	NA	NA	0,13772387
CG41561-RA	CG41561	0	0	0	0	0	0	NA	NA	0,13772387
CG41562-RA	CG41562	1,64675	0,343269	1,43276	0,0551743	0	0,259312	NA	NA	0,6279808
CG41563-RA	CG41563	0	0	0	0	0	0	NA	NA	0,6279808
CG41564-RA	CG41564	0	0	0	12,3521	0	0	NA	NA	0,13772387
CG41566-RA	CG41566	0	0,0358751	0	0	0	0	NA	NA	0,6279808
CG41568-RA	CG41568	0	0	0,145832	0,437105	0,479798	0	NA	NA	0,6279808
CG41569-RA	CG41569	0,158076	0,863921	0,101175	0,283379	0,245278	0,234372	NA	NA	0,13772387
CG41572-RA	Rpn12	35,55	28,5582	56,0796	45,5811	18,6752	56,0775	NA	NA	0,6279808
CG41573-RA	CG41572	0,244003	0	0,234257	0,208289	0	0	NA	NA	0,6279808
CG41574-RA	CG41573	0	0	0	0	0	0	0,044335	0,715924	0,13772387
CG41576-RA	CG41574	0,455068	0,497409	0,242125	0	0,267007	0	0,065796	0,591492	0,13772387
CG41577-RA	CG41576	0	0,605324	0	0,783	0,4154	0,799316	NA	NA	0,13772387
CG41578-RA	CG41577	0	0	0,253747	0,571969	0	0,454135	NA	NA	0,13772387
CG41579-RA	CG41578	0	0	0	0	0	0	NA	NA	0,13772387
CG4157-RA	CG41579	0	0,247342	0	0	0	0	-0,204983	0,475404	0,13772387
CG41580-RA	wor	3,9687	3,96567	4,83382	0,22407	1,4482	0,0293026	NA	NA	0,13772387
CG41581-RA	CG41580	0	0	0	0	0	0	-0,066275	0,671132	0,6279808
CG41582-RA	CG41581	0	0	1,28588	0,40923	0	0	NA	NA	0,13772387
CG41584-RA	CG41582	0,557359	0,131268	1,45768	3,04096	1,12358	1,91592	NA	NA	0,6279808
CG41586-RA	CG41584	0,14427	0,262823	0,138508	0,197191	0,202624	0,208241	0,01562	0,897983	0,6279808
CG41587-RA	CG41586	1,68193	1,20373	0	0	0	0,984062	NA	NA	0,13772387
CG41588-RA	CG41587	0	0	0	0	1,39898	3,73194	NA	NA	0,13772387
CG41589-RA	CG41588	0	0,292167	1,61475	2,42659	2,65118	0	NA	NA	0,6279808

gene_id	Symbol	W1_FPKM	W2_FPKM	W3_FPKM	MCM51_FPKM	MCM52_FPKM	MCM53_FPKM	FC	p-value	p-adj
CG4158-RA	CG41589	0	0,34723	0	0	0	0	-0,71961	0,004536	0,6279808
CG41592-RA	Pus1	11,5477	26,1336	8,5371	0,0274146	0,0401574	10,3821	NA	NA	0,6279808
CG41595-RA	CG41592	0,168132	0	0	0	0	0	NA	NA	0,13772387
CG41596-RA	CG41595	0	16,906	0	16,8758	22,9677	0	NA	NA	0,13772387
CG41599-RA	CG41596	0	0	0	0	0	0	NA	NA	0,13772387
CG4159-RA	CG41599	0	0	0	0	0	0	-0,422292	0,178594	0,13772387
CG41600-RA	CG41600	0	0,236955	0	0	0	0	0,01562	0,897983	0,13772387
CG41603-RA	CG41603	0	0	0	0	0,316492	0	NA	NA	0,13772387
CG41614-RA	CG4161	0	7,70489	0,248169	0,0214525	0,0280617	0,021149	NA	NA	0,13772387
CG41615-RA	CG41614	0	0	0	0	0	0	NA	NA	0,6279808
CG41616-RA	CG41615	0	0	0	0	0	0	NA	NA	0,6279808
CG4161-RA	CG41616	0,18825	0,428679	0,271097	0,544675	0,747375	0,478777	-0,060226	0,773635	0,6279808
CG41622-RA	lace	20,6487	17,0075	0	22,0381	16,2489	0,721022	NA	NA	0,6279808
CG41623-RA	CG41622	0	0	0	0,508312	0,0747339	2,31943	-0,420061	0,19168	0,6279808
CG41624-RB	UQCR-11	576,421	35,3628	20,7618	523,191	32,1036	1097,25	-1,210206	0,00033	0,6279808
CG4162-RA	spok	2,80842	1,17246	2,58391	4,16449	9,04231	6,2228	0,052917	0,795055	0,6279808
CG4163-RA	Cyp303a1	0,44272	14,5831	25,7737	48,7373	29,9049	15,7798	-0,288429	0,413916	0,6279808
CG4164-RA	shv	36,9536	10,8395	46,9048	41,2082	4,09442	1,6843	-0,395766	0,166809	0,6279808
CG4165-RA	Usp16-45	0,0162402	0,0147927	0,0155915	42,1902	0,907723	46,2778	-0,001753	0,99276	0,6279808
CG4165-RB	Usp16-45	0,0159155	1,75413	0,0152798	0,535092	0,528639	0,0171615	0,000272	0,998881	0,6279808
CG4165-RC	Usp16-45	0,0163892	0,0149284	0,0157346	0	5,93106	2,8219	-0,000378	0,998444	0,13772387
CG4165-RD	Usp16-45	11,8629	10,2856	13,5414	2,45504	2,86253	0,017325	5,94E-05	0,999755	0,6279808
CG4166-RB	not	5,17749	3,96821	6,73778	6,63993	3,79935	2,20082	-0,014067	0,953721	0,6279808
CG4167-RA	Hsp67Ba	1,67763	0,102883	0,108439	0,172256	0,233322	0,175846	-0,531188	0,101879	0,6279808
CG4168-RA	Dpse\GA18003	1,24027	14,2049	15,6501	2,38134	0,0569738	11,6317	0,289252	0,222772	0,6279808
CG4169-RA	Dpse\GA18004	135,551	29,5864	0,0231973	10,6203	14,3387	11,9306	-0,676626	0,024696	0,6279808
CG4170-RA	vig	69,9864	0	0,031613	20,9258	0,0529828	0,0386685	0,102813	0,647989	0,6279808
CG4170-RB	vig	27,1247	1,84379	0,025949	0,0354729	0,0513076	0,0876088	0,102813	0,647989	0,13772387
CG4170-RC	vig	0,0360348	77,1146	0,0284896	0,0287045	0,0539492	0	0,102823	0,647932	0,6279808
CG4170-RD	vig	0,0349854	39,6673	0,019052	0,0317165	0,0290575	0,0218995	0,102606	0,648426	0,6279808
CG4173-RA	37500	45,7562	0,0525646	0,0267477	0,0304276	40,6483	10,2184	-0,096762	0,634735	0,13772387
CG4174-RA	CG4174	0,234341	0,0424546	0	0	0,0685215	0	-0,254158	0,463702	0,13772387
CG4174-RB	CG4174	0,0466088	0,0414602	0,644028	0	0	0,0642443	0,094833	0,785241	0,13772387
CG4174-RC	CG4174	0,0455172	0,0573205	0,225086	0	0	0	-0,314406	0,366145	0,13772387
CG4178-RA	Lsp1beta	0	0,0254524	1,68823	11,0333	1,34915	9,11662	-0,079432	0,728846	0,13772387
CG4180-RA	CIAPIN1	13,5129	0,196386	7,74715	29,8162	2,30483	7,3796	-0,115204	0,587296	0,13772387
CG4181-RA	GstD2	10,9617	0,570875	42,8786	0,0257237	22,824	392,608	-0,35697	0,302672	0,13772387
CG4182-RA	yellow-c	25,2726	2,11755	0,304399	0,0292914	15,6747	0,0328197	-0,028961	0,894733	0,13772387
CG4183-RA	Hsp26	297,767	377,774	217,569	1370,22	6,71175	227,927	-0,766614	0,021254	0,13772387
CG4184-RA	MED15	8,24035	14,4335	10,8871	10,6453	5,71718	6,51771	0,193944	0,491123	0,13772387
CG4185-RA	Dsim\GD23986	15,4177	3,63496	22,1588	20,3748	3,18317	4,32737	-0,13724	0,590143	0,13772387
CG4186-RA	CG4186	25,7627	16,42	53,7521	0,0805994	0,109172	0,0822789	-0,140892	0,657446	0,13772387
CG4190-RA	Hsp67Bc	56,6601	0,0959912	0,04881	8,21928	3,12961	2,0206	-0,823589	0,010566	0,6279808
CG4192-RA	kek3	1,18531	1,20726	1,53109	1,97028	0,442374	1,11159	-0,333393	0,206954	0,6279808
CG4193-RA	dhd	3,03949	2,76858	0,149929	0,735347	38,5275	0,429524	-0,592793	0,058457	0,6279808
CG4194-RA	CG4194	9,37929	1,01756	0,0251235	14,2864	0,0410816	0,0289619	-0,438786	0,104973	0,6279808
CG4195-RA	l(3)73Ah	13,4238	11,821	55,9579	3,59716	22,5507	26,444	0,258074	0,258034	0,13772387
CG4196-RB	CG42542	25,2794	16,3713	28,6988	0,933535	0,775948	0,687232	-0,14131	0,597128	0,13772387
CG4196-RC	CG42542	0,0402738	0,0366841	0,0386652	22,197	3,79669	25,1391	-0,141532	0,596366	0,6279808
CG4198-RA	Dana\GF21335	0,0520556	0,29876	0,486654	16,2714	2,84095	27,8773	0,101529	0,680977	0,6279808
CG4199-RA	CG4199	20,7568	0,024367	4,4923	24,1066	3,32554	5,21473	-0,444083	0,04254	0,6279808
CG4199-RB	CG4199	0,0278214	15,9711	7,78891	0,0303296	28,0382	3,60898	-0,489283	0,026803	0,13772387
CG4199-RC	CG4199	0,0267514	0,0238362	3,44283	18,7391	0,0535551	6,03653	-0,49412	0,024316	0,13772387
CG4199-RD	CG4199	10,9127	15,9042	7,92825	40,4561	0,0512487	2,92177	-0,489749	0,026595	0,6279808
CG4199-RG	CG4199	0,0261687	0,0692062	21,4224	0,0283708	5,12084	16,0991	-0,482383	0,0284	0,13772387
CG4199-RH	CG4199	8,00271	0,0297854	0,0267102	0,0311763	7,8795	0,0309616	-0,483197	0,028172	0,6279808
CG4200-RA	sl	4,69233	5,18393	3,81357	5,53229	2,93881	3,07202	0,126046	0,603709	0,13772387
CG4201-RA	IKKbeta	6,6872	0,0237359	6,62392	0,283592	6,33364	1,18613	-0,010221	0,960019	0,13772387
CG4202-RA	Sas10	11,3819	6,09179	19,4617	11,9679	1,3751	17,8792	-0,3033	0,32881	0,6279808
CG4203-RA	Mau2	4,57716	4,32789	7,34419	6,02813	25,2229	4,17952	0,198806	0,397058	0,13772387
CG4204-RA	EloB	39,6603	52,2268	18,7463	21,965	8,54792	12,3938	-0,518924	0,023852	0,13772387
CG4205-RA	Fdx1	7,52432	10,1151	13,7501	419,838	9,57901	13,6776	0,056574	0,835002	0,13772387
CG4206-RA	Mcm3	3,68716	3,37252	2,43453	2,45768	5,57475	2,20891	-0,711168	0,004432	0,13772387
CG4207-RA	bonsai	37,5825	6,45891	5,19887	5,49539	13,2051	2,44421	0,179057	0,503431	0,13772387
CG4208-RA	XRCC1	1,3476	1,37267	6,61093	5,35256	1,305	2,4269	-0,259893	0,377635	0,6279808
CG4209-RA	CanB	41,9024	46,4972	51,8695	44,3807	31,3025	31,7402	0,363755	0,043599	0,6279808
CG4210-RA	CG4210	0,0933275	8,039	0,0896	16,6518	6,54798	5,30804	-0,350182	0,23677	0,6279808
CG4210-RB	CG4210	13,0924	9,03825	16,0065	7,43914	17,244	13,3208	-0,350182	0,23677	0,6279808
CG4211-RA	nonA	0,0132346	0,012055	0,012706	0,0136041	0,0184268	0,0138876	0,074793	0,748091	0,13772387
CG4211-RB	nonA	17,9281	13,3464	17,5436	15,3227	8,40471	11,8197	-0,104353	0,671428	0,13772387
CG4211-RC	nonA	8,10186	8,94825	11,83	21,6422	7,09657	10,2357	0,068826	0,766486	0,13772387
CG4212-RA	Rab14	33,9407	0,102942	388,927	2,21087	6,96958	1,63989	0,293686	0,112669	0,13772387
CG4212-RB	Rab14	0,0628894	16,4894	42,1282	0,14653	0,0281857	2,32203	0,290384	0,11628	0,13772387
CG4212-RC	Rab14	0,0750292	11,5083	0,0603775	0,0292265	1,5825	20,3724	0,289617	0,117378	0,13772387
CG4213-RA	CG4213	6,66142	4,09477	17,4058	2,71491	16,4992	13,8529	0,290842	0,308368	0,13772387
CG4214-RA	Syx5	24,8076	30,5946	0,0286569	26,9673	0,0432307	20,2938	0,12538	0,510695	0,13772387
CG4214-RB	Syx5	0,0370657	0,033762	55,9192	7,24375	90,3102	3,134	0,125696	0,509558	0,6279808
CG4215-RA	spel1	0,0199284	8,30353	0,0191324	0,0900749	21,6218	1,43349	-0,16896	0,532558	0,13772387
CG4215-RC	spel1	1,50929	0,423849	1,83085	1,55721	0,0990001	5,31949	-0,16896	0,532558	0,13772387
CG4216-RA	term	0	0,134345	0,173443	0,211067	0,0885058	0,15257	0,009189	0,96753	0,13772387
CG4217-RA	TFAM	0,0577082	11,958	7,76543	17,5074	0,0371333	64,5014	-0,240656	0,384979	0,13772387
CG4217-RB	TFAM	33,8142	6,32853	11,7338	11,7191	5,78424	0,154093	-0,240656	0,384979	0,13772387
CG4218-RA	CG4218	0,151689	0,0829015	0,0873785	0	0,0395874	0	0,217865	0,246975	0,13772387
CG42200-RA	elB	0,0274896	0,0250394	0,0263917	6,17646	0	0,0298355	NA	NA	0,13772387
CG4220-RA	elB	28,0412	41,1098	30,296	8,78962	0,295845	19,7203	0,331994	0,187627	0,13772387
CG4220-RB	elB	0,0261825	0,0238488	0,0251367	0,0147855	0	0,0283277	0,224039	0,367839	0,13772387
CG4220-RC	CG42200	0,153665	0,0933124	0,295055	0,204739	0,247067	0,295057	0,301689	0,20762	0,13772387
CG4221-RA	Fbxl7	7,81192	0,0600862	12,116	16,7393	0,0358794	0,0270409	0,282303	0,363635	0,13772387
CG42226-RA	CG42226	0,576853	0	0	0	0	0,50852	0,087499	0,683129	0,13772387
CG42227-RA	CG42227	0,0713562	0	0,0685063	0	0,313145	0	0,024053	0,869957	0,6279808
CG42228-RA	CG42228	0,224544	0	0	0,372395	0	0	0,074368	0,563156	0,13772387
CG42229-RA	CG42229	0	0	0,343521	0	0	0	0,01562	0,897983	0,13772387
CG42230-RC	bbg	19,0387	22,3028	16,0494	18,0995	8,50969	9,93272	0,007595	0,978284	0,13772387
CG42230-RE	bbg	0,0165369	0,0150629	0,0158764	0,0171302	0,023203	0,0174872	0,102478	0,706311	0,13772387
CG42230-RF	bbg	0,0168877	0,0153825	0,0162132	0,017508	0,0237147	0,0178728	0,09977	0,713501	0,13772387
CG42230-RG	bbg	0,0252992	0,0230442	0,0161925	5,34242	0,0236832	0,0178491	0,10065	0,711058	0,13772387
CG42230-RH	bbg	0,0169309	0,0154219	0,0162547	0,0175546	0,0237779	0,0179204	0,09977	0,713501	0,13772387
CG42230-RI	bbg	3,17573	3,66135	3,49663	5,36305	1,86343	2,56046	0,123805	0,64854	0,6279808
CG42230-RJ	bbg	0,00802021	0,00730537	0,00769988	4,3002	0,991651	1,62843	0,040634	0,883064	0,13772387
CG42231-RA	CG42231	18,3094	13,329	17,6467	18,6889	56,4497	77,3102	0,439484	0,101797	0,13772387
CG42232-RB	CG42232	1,69682	1,83313	3,62239	0,025774	24,7113	1,97137	-0,06605	0,831506	0,13772387
CG42233-RB	CG42233	1,24509	1,44439	1,44345	0,053288	5,19371	4,26679	0,215163	0,427973	0,13772387
CG42234-RC	Dbx	1,76938	26,4206	7,60136	79,548	2,3385	0,104852	0,009044	0,972986	0,13772387
CG42235-RA	CG42235	2,20723	0,0265411	3,09613	1,59591	3,45284	2,65134	-0,59473	0,079685	0,13772387

gene_id	Symbol	W1_FPKM	W2_FPKM	W3_FPKM	MCM51_FPKM	MCM52_FPKM	MCM53_FPKM	FC	p-value	p-adj
CG42235-RB	CG42235	1,58099	0,0377345	1,73862	1,70695	0,0421288	0,0317508	-0,870201	0,011315	0,13772387
CG42235-RC	CG42235	1,74281	17,392	1,89086	1,7677	0,0559565	0,0483043	-0,69332	0,043807	0,13772387
CG42235-RD	CG42235	2,26058	82,2103	2,44321	2,28115	5,365	8,4942	-0,770651	0,020005	0,6279808
CG42235-RE	CG42235	0,0291382	12,5753	0,0279745	0,0311027	8,2857	0,291464	-0,617926	0,047704	0,13772387
CG42236-RA	RanBPM	23,577	33,1593	0,0217497	0,0157956	0,0415792	0,0313366	0,185895	0,504219	0,13772387
CG42236-RC	RanBPM	0,015293	0,0139299	0,0217497	5,50601	0,0413098	0,0311336	0,187216	0,501558	0,13772387
CG42236-RD	RanBPM	8,01701	8,81594	0,0211691	3,61094	0,0438251	0,0330292	0,185895	0,504219	0,13772387
CG42237-RA	CG42237	26,3888	42,1188	20,4336	16,0395	52,3482	12,5621	0,422097	0,1331	0,13772387
CG42238-RA	CG42238	0,0283506	0,0258237	0,0272183	0,0302045	0,0409121	0,0308339	-0,114571	0,625078	0,13772387
CG42238-RB	CG42238	9,41468	12,908	9,18683	14,7935	9,12713	9,52677	-0,130432	0,573883	0,13772387
CG42239-RA	CG42239	56,8639	0,0616665	0,0649967	0,0513836	242,356	0,0478916	-0,582608	0,053683	0,13772387
CG42240-RA	CG42240	12,4961	29,0122	18,2301	30,3051	13,4462	12,2414	0,095035	0,749936	0,13772387
CG42241-RA	Pde8	0,775084	58,4083	60,0631	10,3893	69,3649	0,15697	-0,587733	0,088617	0,13772387
CG42242-RD	beat-VII	0,876589	840,505	46,761	56,1016	0,823332	0,663129	-0,351904	0,242148	0,6279808
CG42244-RB	Octbeta3R	3,06012	3,76455	2,65649	0,948387	0,443715	1,35592	0,162407	0,557141	0,13772387
CG42244-RC	Octbeta3R	0,0109222	0,00994872	0,382739	0,049264	0,0667285	0,319592	0,162407	0,557141	0,6279808
CG42244-RD	Octbeta3R	0,0444456	0,0404841	0,0426704	0,0581301	0,0787376	0,0502906	0,194639	0,48532	0,6279808
CG42244-RE	Octbeta3R	0,0515144	0,0469229	0,0494569	0,0397532	0,0538459	0,0593414	0,181451	0,512864	0,6279808
CG42244-RF	Octbeta3R	0,0365733	0,0333135	0,0351126	0,047048	0,0637268	0,0405815	0,346193	0,160423	0,6279808
CG42244-RG	Octbeta3R	0,0426387	0,0388383	0,0409357	0,0421162	0,0570467	0,0480284	0,364465	0,139154	0,6279808
CG42244-RI	Octbeta3R	0,0385581	0,0351214	0,0555272	5,06525	10,2498	0,0429938	0,153076	0,58433	0,6279808
CG42245-RA	CG42245	0,0130774	14,9236	0,0125725	28,4002	6,06916	17,0337	0,12457	0,598215	0,6279808
CG42246-RA	CG42246	7,83079	6,1436	4,8291	0,0217858	0,029509	0,0222398	-0,594873	0,0751	0,6279808
CG42247-RC	DCX-EMAP	0,718645	2,43247	0,868515	0,0324826	0,175688	4,53305	-0,019214	0,945003	0,6279808
CG42247-RD	DCX-EMAP	1,80332	0,027638	2,81927	7,42732	4,40684	55,9659	0,028814	0,91794	0,6279808
CG42248-RD	CG43867	11,4286	0,149058	0,00895789	0,819611	0,0452929	6,24798	0,148321	0,606615	0,6279808
CG42248-RF	CG43867	0,00933055	0,0283943	0	0,82061	5,45987	0,00970278	0,145026	0,615452	0,6279808
CG42249-RB	CG42249	3,39818	4,64322	7,44366	0	0	9,81257	-0,745202	0,037123	0,6279808
CG42249-RC	CG42249	0,0372118	38,5998	0,0357256	5,42558	1,44045	0,041355	-0,842783	0,018175	0,6279808
CG42250-RC	Hmt-1	11,5434	8,54776	0,621929	16,1218	144,996	5,09509	-0,017815	0,924749	0,6279808
CG42250-RD	lqfR	1,54468	1,49837	2,20201	2,77086	2,57661	1,54752	-0,384146	0,050951	0,6279808
CG42251-RC	lqfR	23,571	21,3555	25,3103	24,8117	1,0243	20,4585	-0,345182	0,268765	0,6279808
CG42251-RD	hwt	1,59772	1,60586	0,467315	3,42677	0,678531	0,94355	-0,361848	0,245255	0,6279808
CG42252-RB	hwt	0,0147502	0,698645	160,577	0,647343	0,513281	0,207132	0,188265	0,41645	0,6279808
CG42252-RC	mmd	7,91931	10,4512	8,5677	12,0329	7,07836	0,0200879	0,158719	0,486974	0,6279808
CG42252-RD	mmd	0,0132081	0,0120309	0,0126806	0,0135761	0,0183889	1,20136	0,152118	0,503817	0,6279808
CG42253-RA	mmd	2,02138	0,0172076	2,15829	0,0196778	0,0266538	4,11082	-0,676228	0,002745	0,6279808
CG42253-RB	Ndae1	0,0173443	0,0157984	0,0166515	0,0180006	3,36251	0,0183757	-0,680558	0,002356	0,6279808
CG42253-RC	Ndae1	9,33981	9,35018	15,981	14,4659	0,0363766	15,4003	-0,808144	0,000391	0,6279808
CG42254-RB	Ndae1	3,2541	2,64116	0,0174533	6,71326	0,024382	3,25351	0,177422	0,583624	0,6279808
CG42255-RA	asRNA:CR42254	0,0527341	19,2012	18,3832	24,8053	7,15123	12,1859	-0,781786	0,000757	0,6279808
CG42256-RE	CG42255	0,73012	6,15279	7,86813	6,11235	0,939586	0,887031	-0,334659	0,270734	0,6279808
CG42256-RF	Dscam2	0,00975482	0,390956	0,407386	0,00994663	0,0134728	0,232066	-0,334719	0,247221	0,6279808
CG42256-RG	Dscam2	0,564525	0,243573	0,20443	1,12163	0,901231	0,697164	-0,712251	0,003595	0,6279808
CG42256-RH	Dscam2	0,129082	0,0097981	0,253017	0,413517	0,0148912	0,0112229	-0,69634	0,003032	0,6279808
CG42256-RI	Dscam2	0,0106528	0,00970332	0,0102273	0,0108849	0,0147436	0,0111117	-0,664389	0,007479	0,6279808
CG42256-RJ	Dscam2	0,0107832	0,00982209	0,0103525	0,0110214	0,0149286	0,0112511	-0,690204	0,003252	0,6279808
CG42256-RK	Dscam2	0,277035	0,319634	0,0177313	0,0192187	0,0260318	0,0196192	-0,704645	0,00396	0,6279808
CG42257-RD	Dscam2	0,0290685	0,0176517	0,018605	0,0202089	0,0273731	0,0206301	0,216328	0,300243	0,6279808
CG42257-RE	Snp	0,0419529	0,0242785	8,05516	10,6055	0,0728862	4,72981	0,198258	0,341288	0,6279808
CG42257-RF	Snp	9,75804	0,0421769	0,0255897	0,028944	0,0655522	5,87673	0,20149	0,327912	0,13772387
CG42257-RG	Snp	4,96978	0,039841	0,0444547	0,0538102	3,12004	2,67648	0,159168	0,436874	0,6279808
CG42257-RH	Snp	0,0266543	24,0852	0,0419925	0,0483956	0,020045	0,0151071	0,137672	0,500403	0,6279808
CG42258-RC	Snp	3,84324	0,368113	11,287	12,7575	2,91041	1,5094	-0,070291	0,750503	0,6279808
CG42258-RD	CG42258	7,25258	38,3342	307,389	49,1304	52,1555	0,0812003	-0,132176	0,558969	0,6279808
CG42259-RA	CG42258	37,311	0,259425	0,038823	3,7461	0	4,6469	0,203121	0,439243	0,6279808
CG4225-RA	CG42259	47,2037	38,7095	29,9289	41,6426	30,8039	40,9915	-0,072258	0,701776	0,6279808
CG42260-RA	GluRIIC	6,82784	0,014424	0,0608565	0	0,089721	0,0676192	-0,312223	0,237342	0,6279808
CG42260-RB	CG42260	1,00271	0,0702564	0,0740506	2,96648	31,1531	26,9114	-0,343758	0,197703	0,6279808
CG42260-RC	CG42260	0,993963	1,86792	1,67749	2,83555	29,4975	23,6518	-0,340422	0,241323	0,6279808
CG42261-RA	CG42260	0,323522	0,0151121	0,246887	0,0171883	0,0179668	0,0135409	-0,187808	0,521077	0,6279808
CG42262-RA	fid	1,17086	1,34245	6,56279	1,59948	12,2217	2,9971	-0,213022	0,451838	0,6279808
CG42263-RA	CG45060	10,2584	0,687846	1,19624	80,0893	0,0684689	0,0516023	-0,070569	0,663212	0,6279808
CG42264-RA	CG42263	0,0889711	0	0	0	0	0	-0,275398	0,372138	0,6279808
CG42265-RA	CG42264	0	34,9773	33,9086	38,7537	16,659	25,2072	-0,130412	0,666312	0,6279808
CG42265-RB	OtopLc	2,73681	145,955	1,9468	145,921	2,35057	1,6891	-0,177574	0,558792	0,6279808
CG42266-RA	OtopLc	0,520738	30,9108	0,470957	12,9831	0,531746	0,0159074	-0,219166	0,467213	0,6279808
CG42267-RA	CG42266	0,0846259	0,0571196	46,669	0,244329	0,0825263	54,7535	-0,393344	0,222499	0,6279808
CG42267-RB	RunxB	0,0747745	0,0681098	0,071788	0,089707	1,10807	6,55442	-0,535686	0,131775	0,6279808
CG42268-RD	RunxB	0,709887	0,923734	0,697761	1,34751	6,68071	0,258837	0,086473	0,68693	0,6279808
CG42268-RF	CG42268	16,2402	0,865696	1,62213	2,60346	420,782	448,366	0,092252	0,669547	0,6279808
CG42268-RG	CG42268	0,0123954	17,7773	0,0160126	0,017283	0,0234099	0,0176431	0,091284	0,672767	0,6279808
CG42269-RC	CG42268	0,011758	0,0112906	0,0164486	0,0177724	0,0240729	0,0181428	-0,034852	0,922362	0,6279808
CG42269-RE	CG42269	0,0656382	0,77028	12,85	0,0768397	1,20551	1,12702	0,270707	0,408198	0,6279808
CG4226-RC	CG42269	1,61301	1,18217	0,0630166	0,889998	0,8247	2,29826	-0,485698	0,043906	0,6279808
CG42270-RB	raskol	6,97967	2,25274	2,67798	4,32176	5,3802	39,7451	0,134295	0,609235	0,6279808
CG42270-RC	raskol	0,0122643	8,44656	65,3036	7,08466	4,86474	9,9723	0,131741	0,616061	0,6279808
CG42270-RD	raskol	12,5861	0,0111712	19,0937	4,32682	5,02922	15,9952	0,149888	0,561683	0,6279808
CG42271-RB	CG42271	7,03929	4,20627	6,23884	6,87567	5,21852	33,2229	-0,521487	0,024267	0,6279808
CG42271-RC	CG42271	3,31751	10,1938	3,75287	0,3819	5,69395	5,07245	-0,236372	0,319077	0,6279808
CG42272-RC	CG42272	4,49355	0,532624	0,851751	0,638915	0,603046	0,87711	0,259313	0,278267	0,6279808
CG42272-RE	CG42272	1,77437	0,0171195	0,0951552	13,2644	0	0	0,287453	0,235514	0,6279808
CG42273-RB	mnb	11,0982	0,0144171	0,0151956	0,0163118	0,0220944	0,0166517	0,181234	0,474823	0,6279808
CG42273-RC	mnb	0,0158278	0,0143689	0,0151449	0,0155313	0,0210372	0,0158549	0,128697	0,608017	0,13772387
CG42273-RD	mnb	0,0157749	0,020557	0,0216672	0,540892	0,830388	0,358064	0,137311	0,598555	0,6279808
CG42273-RF	mnb	0,0150457	1,26405	0,631096	0,547704	0,232617	0,282395	0,129889	0,603636	0,6279808
CG42274-RB	RhoGAP18B	7,3933	9,34448	6,70808	17,6662	1,83632	6,8569	-0,104551	0,622732	0,6279808
CG42274-RC	RhoGAP18B	6,25003	6,37643	6,00859	0,837631	23,5543	2,91924	-0,070731	0,736834	0,13772387
CG42274-RD	RhoGAP18B	0,0308958	0,028142	0,0296618	15,7407	16,9745	3,23893	-0,251083	0,211979	0,6279808
CG42274-RF	RhoGAP18B	6,18058	9,7916	8,16622	18,1758	8,12046	6,86778	-0,08605	0,684733	0,6279808
CG42275-RB	alpha-Man-Ia	4,77136	14,2854	0,0180338	8,00979	4,42566	4,6843	0,295608	0,215581	0,13772387
CG42275-RC	alpha-Man-Ia	12,4079	0,0171098	0,0188819	0,019561	0,0264955	0,0199686	0,438369	0,067023	0,13772387
CG42275-RD	alpha-Man-Ia	0,018784	0,0179144	0,019012	3,66973	1,91976	0,0209513	0,298744	0,211527	0,13772387
CG42275-RE	alpha-Man-Ia	3,33362	0,0180379	0,0180594	6,05332	0,028	3,03312	0,295819	0,215644	0,6279808
CG42275-RF	alpha-Man-Ia	0,019803	0,0171341	3,51219	0,0195901	0,0265349	0,0199983	0,298552	0,211772	0,6279808
CG42275-RG	alpha-Man-Ia	0,0188107	3,17314	0,017821	0,02024	1,90055	0,0206617	0,299676	0,210092	0,6279808
CG42275-RH	alpha-Man-Ia	0,0194074	0,0169079	30,1211	0,0193201	0,0261692	0,0197227	0,297823	0,212406	0,6279808
CG42275-RI	alpha-Man-Ia	0,0185624	0,127622	1061,99	0,163035	24,5282	25,0031	0,357841	0,122379	0,6279808
CG42276-RA	Pde9	1,48912	0,526412	10,2611	0,00530549	0,781586	0,0350242	-0,383875	0,107991	0,6279808
CG42276-RB	Pde9	2,93335	3,42601	0,00504722	0,00527154	121,355	54,6611	-0,409302	0,08292	0,6279808
CG42277-RB	rn	0,378503	0,358683	0	19,1772	3,3494	22,3846	0,182356	0,571133	0,6279808
CG42277-RC	rn	0,0213446	0,0537059	0	24,5945	2,02489	7,09047	0,312952	0,336369	0,6279808
CG42277-RD	rn	0,467043	0,0950479	0	7,41826	0,0241969	0,013032	-0,041795	0,903107	0,6279808
CG42277-RF	rn	0,0589611	0,0306969	0	0,017864	2,51657	0,0182362	0,293613	0,37029	0,6279808

gene_id	Symbol	W1_FPKM	W2_FPKM	W3_FPKM	MCM51_FPKM	MCM52_FPKM	MCM53_FPKM	FC	p-value	p-adj
CG42278-RB	corn	4,46573	3,12747	2,69772	491,746	0,923887	1,57924	0,024502	0,936316	0,6279808
CG42278-RC	corn	1,87856	0,0866406	2084,98	2,69412	8,79477	11,5101	0,153415	0,597608	0,6279808
CG42279-RC	Nedd4	0,0169919	0,0205243	0,0144218	0,0155057	0,0238669	0,0158288	-0,167729	0,437306	0,6279808
CG42279-RD	Nedd4	8,26019	0,0138053	0,0145508	0,0156493	4,89652	0,0159754	-0,153107	0,477131	0,6279808
CG42279-RE	Nedd4	0,0152718	17,325	16,216	12,9666	5,83578	10,2694	-0,139899	0,511609	0,6279808
CG42279-RF	Nedd4	0,0150218	0,0146665	0,0154586	0,0166624	0,0210025	0,0170096	-0,155435	0,467054	0,6279808
CG42279-RG	Nedd4	0,0151562	17,4169	6,87055	10,5658	0,0211971	5,70016	-0,187577	0,387929	0,6279808
CG42279-RH	Nedd4	16,5068	0,0120237	0,012673	0,0135677	14,9294	0,0138504	-0,168024	0,43649	0,6279808
CG42279-RI	Nedd4	0,0161017	16,4836	13,5188	11,1679	0,0225693	10,5795	-0,167522	0,437511	0,6279808
CG42280-RE	ome	0,0163973	0,0149842	0,0157424	6,65778	0,0229997	0	0,424004	0,060079	0,6279808
CG42280-RF	ome	34,3917	9,72692	26,91	0,328888	17,7049	0,0572185	0,430801	0,054862	0,6279808
CG42280-RG	ome	0,0164504	5,16994	0,0157934	0,279581	0,023077	0	0,430358	0,054837	0,6279808
CG42281-RD	bun	0,0248437	0,0226293	0,0238514	20,6157	0,0355507	0,0267931	0,080832	0,681907	0,6279808
CG42281-RE	bun	0,025634	0,0233492	0,0246101	0,0271324	0,036751	0,0276978	0,047533	0,804212	0,6279808
CG42281-RF	bun	61,2191	73,7915	77,05	40,6321	47,4993	49,9315	0,043081	0,821693	0,6279808
CG42281-RG	bun	0,0116687	0,0106286	0,0112026	9,20743	0,0161876	0,0122	0,071402	0,718176	0,6279808
CG42281-RH	bun	0,0131791	0,0120045	0,0126528	0,0135454	0,0183473	0,0138276	0,076199	0,696836	0,6279808
CG42281-RI	bun	0,0115605	0,0105301	0,0110988	0,0118371	0,0160335	0,0120838	-0,013333	0,95054	0,6279808
CG42281-RJ	bun	98,5453	109,364	124,942	94,817	116,58	98,6368	0,048306	0,800989	0,6279808
CG42282-RC	NimA	1,01439	2,16069	1,27353	1,97088	0,731452	0,847149	0,205714	0,532674	0,6279808
CG42283-RB	5PtaseI	9,49933	6,16232	5,67324	30,4678	27,5959	0,0255806	0,469106	0,024495	0,6279808
CG42283-RC	5PtaseI	0,0257338	4,88936	15,7662	7,64966	9,43521	7,03302	0,385191	0,090807	0,6279808
CG42283-RF	5PtaseI	5,67146	12,4889	8,21221	15,5208	6,60869	11,2653	0,457032	0,03026	0,6279808
CG42284-RC	CG42284	2,36451	2,28744	5,52645	1,47556	2,90019	1,75105	-0,500769	0,094219	0,6279808
CG42285-RA	CG45263	0,71627	138,054	4,54769	8,64886	2,43182	3,50992	-0,810267	0,019495	0,6279808
CG42286-RA	CG45263	4,30407	6,00113	0,164174	2,29651	4,57123	0,704775	-0,083377	0,758559	0,6279808
CG42287-RA	CG42287	0,191339	11,213	10,8822	131,223	4,13488	2,94179	0,25109	0,4426	0,6279808
CG42288-RA	CG42288	0,310215	0,34857	0,612324	6,19439	3,86819	4,98918	0,025431	0,942638	0,6279808
CG42289-RA	Ir60b	0,0571591	0,0601865	4,9838	0	0,0481001	8,86334	0,113796	0,622308	0,6279808
CG42290-RA	CG4229	159,919	140,961	4,12954	208,889	2,9064	3,05755	NA	NA	0,6279808
CG42291-RA	Ir60c	0	0	13,5579	0,0645355	8263,42	0,0101769	0,01562	0,897983	0,6279808
CG42292-RA	Ir60d	0	0,156193	0,00938599	0	0	0,111814	-1,847349	2,43E-07	0,6279808
CG42293-RA	Ir7f	0,0499818	0,0106606	79,5044	0,0386502	2,17224	0,430994	-0,379713	0,130318	0,6279808
CG42294-RA	Ir7e	0	0,374827	0	4,163	0,0531406	0,0189397	NA	NA	0,6279808
CG42295-RB	Ir7d	0	0,0329127	0,0791377	0,621007	0	0	-0,342193	0,200581	0,6279808
CG42296-RA	lectin-22C	0	2,4705	9,48269	0,167657	1,19622	153,178	0,343678	0,320278	0,6279808
CG42297-RA	CG42296	4,37186	0,670602	0,0102405	8,10649	0,717019	76,1585	0,317463	0,307815	0,6279808
CG42298-RA	Ir94d	0,152894	2,02023	0	5,01198	0,241681	0	-0,188303	0,527754	0,6279808
CG42299-RA	Ir94e	0	0,049172	0,0621929	0,219817	0,0907929	0,149304	-0,014388	0,910154	0,6279808
CG4229-RA	CG42299	0,389254	0,0886399	2,49998	3,39749	0,0613187	2,66818	-0,92284	0,0053	0,6279808
CG42300-RA	CG4230	13,7235	42,0754	0,010839	0,668868	15,2555	0,0117935	-0,153615	0,550854	0,6279808
CG42301-RA	CG42300	0,340597	0,103413	0,326994	0,564611	0,887049	0,715634	0,428455	0,15026	0,6279808
CG42302-RA	CCKLR-17D1	0,745217	0,942771	0,695579	0,114166	0	0	-0,02376	0,883718	0,6279808
CG42303-RA	CG42302	0	0	0,259987	45,5029	0,0530458	26,402	0,211769	0,338219	0,6279808
CG42304-RA	Snup	0,0209987	0,019127	0,0123043	2,18693	1,68818	0,0134354	0,211769	0,338219	0,6279808
CG42306-RA	CG42304	2,19925	2,26202	0,0121001	0,047695	0,57911	0,0132059	0,040073	0,84237	0,6279808
CG42306-RB	CG42306	11,4114	0,0209078	4,20223	0,0854354	23,9274	1,24399	0,037551	0,852336	0,6279808
CG42307-RC	CG42306	0,0281333	0,0206992	6,81404	0,0264201	0,0459106	1,52318	-0,205026	0,49781	0,6279808
CG42308-RA	CG42307	8,92986	1,50555	14,2887	13,4188	19,3467	22,2038	-0,458168	0,105069	0,6279808
CG42309-RB	CG42308	0,114582	0,104369	0,102538	0	1,61671	0,842875	-0,400618	0,130904	0,6279808
CG42309-RE	Mlp60A	3255,23	3497,45	4001,73	4,83183	4,47061	1,95564	-0,398918	0,13595	0,6279808
CG4230-RA	Mlp60A	0,032961	0,0300232	0,0316445	3,55209	1,53624	3,99771	-0,210911	0,360365	0,6279808
CG42310-RB	Or22b	0,43005	0,0380766	0,121433	0,0460291	0,198106	31,5297	0,059978	0,864527	0,6279808
CG42310-RC	prom	0,346249	0,102501	0,249315	0	9,20855	0	-0,421803	0,164473	0,6279808
CG42310-RD	prom	0,0152824	0,0139202	0,014672	0,839232	0	0	0,046939	0,893512	0,6279808
CG42311-RC	prom	0,0207221	0,0185646	0,0198944	0	0	0,265854	-0,018661	0,934226	0,6279808
CG42311-RE	grh	0,0137869	1,60198	0,0113226	9,82686	0,0411574	3,08568	0,06312	0,78103	0,6279808
CG42311-RF	grh	6,25656	2,95229	0,0115137	0,049486	10,1666	1,64843	-0,001404	0,995203	0,6279808
CG42311-RG	grh	0,0117936	6,66953	0,0186707	4,26543	0,0622598	24,1767	0,003729	0,987345	0,6279808
CG42311-RI	grh	0,0119927	27,0315	11,5988	31,5523	1,3951	0	0,002364	0,991973	0,6279808
CG42311-RJ	grh	0,0194474	0,0259685	11,0266	0,137557	0,0867758	0	-0,002662	0,990903	0,6279808
CG42311-RK	grh	11,6296	30,4547	9,11258	8,25314	3,84459	20,0777	-0,045512	0,842545	0,6279808
CG42312-RB	grh	12,104	0,0380285	62,0942	21,3253	2,86227	0,0310187	0,403975	0,205726	0,6279808
CG42312-RC	cno	19,9869	40,0039	15,8469	57,6041	0,117355	11,4582	0,596241	0,057925	0,6279808
CG42312-RD	cno	0,00957251	19,6707	0,00919018	25,9828	0,291173	0,0423709	-0,095712	0,767716	0,6279808
CG42313-RA	con	0,0110133	10,3105	0,0105734	10,9752	0,52935	0,0438629	0,31858	0,187282	0,6279808
CG42314-RC	side-II	4,83696	5,53814	0	0,0178939	0,0242374	3,47397	0,088742	0,684365	0,6279808
CG42314-RD	PMCA	0,0166201	0,0151387	49,3233	45,8544	19,7426	0,088562	-0,16927	0,493692	0,6279808
CG42314-RE	PMCA	0,0163365	0,0148804	6,39109	20,7741	5,29121	0,0902251	0,090531	0,678484	0,6279808
CG42314-RF	PMCA	64,7874	68,8893	18,6342	4,4896	13,7337	8,2799	-0,067296	0,770445	0,6279808
CG42314-RG	PMCA	49,997	30,4059	3,21572	0,0170372	2,82986	11,7677	0,090551	0,678427	0,6279808
CG42314-RH	PMCA	17,4616	16,0377	0,0157934	0,0172197	0,023077	2,03155	0,103213	0,63511	0,6279808
CG42315-RA	PMCA	0,0175423	0,0159787	15,617	0,0169147	7,99195	0,0173922	-0,155554	0,641981	0,6279808
CG42315-RB	Ir93a	0,0282899	0,309177	0,884342	0,558201	0	0	-0,116329	0,731561	0,6279808
CG42315-RC	Ir93a	0,54528	8,21937	6,37882	0,634731	0,935962	0,26446	-0,149134	0,655826	0,6279808
CG42316-RC	Ir93a	0,025417	0,219032	0,23086	0,308615	4,31686	4,31172	-0,343432	0,216002	0,6279808
CG42316-RE	RhoGAP102A	5,53776	3,6416	0,0698129	0,0524398	60,885	0,0535325	-0,345008	0,213252	0,6279808
CG42317-RB	RhoGAP102A	0,0194933	4,74082	0,0709053	100,238	6,75411	79,2982	0,358634	0,162655	0,6279808
CG42317-RC	Csk	0,0130904	1,60312	1,38837	16,9408	1679,37	8,04493	0,313157	0,221556	0,6279808
CG42317-RD	Csk	0,016043	0,0128256	0,0415252	0,0169583	0,0196435	0,0173116	0,357472	0,163757	0,6279808
CG42317-RE	Csk	0,0169483	0,0119236	54,8058	23,7268	0,0182199	8,80414	0,357331	0,16396	0,6279808
CG42317-RF	Csk	23,8661	0,0146131	39,1208	0	0,022484	0,27085	0,357472	0,163757	0,6279808
CG42317-RG	Csk	0,016377	0,0154377	138,527	4,88898	0,0238032	2,2644	0,351091	0,170041	0,6279808
CG42318-RB	Csk	7,38428	34,541	0,0420761	0,136275	10,2234	0,139114	-0,497684	0,020329	0,6279808
CG42318-RC	app	13,2698	16,27	0,0168685	16,713	22,3464	0,0258957	-0,497684	0,020329	0,6279808
CG42318-RD	app	2,05483	0,0135139	0,0230959	0,0153076	0,0496245	0,0256782	0,037198	0,848752	0,6279808
CG42318-RE	app	0,0176736	0,0160984	0,0229124	0,0183567	6,54801	0,91532	0,037198	0,848752	0,6279808
CG42318-RF	app	0,0175702	0,0160042	2,97789	0,0182449	0,741054	0,19581	-0,509894	0,01736	0,6279808
CG42318-RG	app	0,0240567	0,0219125	54,5747	0,0253671	0,259812	7,51941	-0,509894	0,01736	0,6279808
CG42319-RA	app	0,0238656	0,0217384	0,0325706	0,0251541	0,0235395	0,0177408	-0,095597	0,716614	0,6279808
CG42319-RB	CG42319	1,68575	0,278964	1,09689	0,191915	0	0,362161	-0,094064	0,720704	0,6279808
CG42319-RC	CG42319	1,03414	0,032312	0,0136228	0,0139897	0	0,0142813	-0,094782	0,718528	0,6279808
CG42319-RD	CG42319	11,0403	0,0369998	2,12539	0,0445961	0,0604057	2,44639	-0,098818	0,698267	0,6279808
CG42319-RE	CG42319	0,0679326	0,0229748	0,0242156	1,96488	0,0361263	11,5816	-0,326901	0,171125	0,6279808
CG42319-RF	CG42319	0,033615	24,5568	17,7796	14,128	14,7772	0,0436636	-0,356117	0,163267	0,6279808
CG4231-RA	CG42319	3,40876	0,0618777	0,0652193	0,0800122	0,108377	2,42994	-0,021926	0,949548	0,6279808
CG42320-RJ	Doa	0,0305388	0,0294502	9,68815	0,0188449	0,0255256	0,0096485	0,475147	0,029593	0,6279808
CG42320-RK	Doa	0,0313651	46,3934	0,00890883	11,4552	6,17957	0,0192376	0,47444	0,028876	0,6279808
CG42320-RL	Doa	0,0221979	0,0196452	0,0174006	14,7384	10,0163	6,34797	0,432068	0,044885	0,6279808
CG42320-RM	Doa	0,0226313	0,0192864	0,0135761	0,694157	9,49891	11,3639	0,470201	0,031044	0,6279808
CG42320-RN	Doa	0,032332	0,0141194	0,672305	21,6702	0,0330494	0,697734	0,112753	0,612665	0,6279808
CG42320-RP	Doa	41,0218	0,00845236	8,15015	0,0243996	0,0307071	7,60456	0,444683	0,039921	0,6279808
CG42320-RQ	Doa	0,0215676	0,016509	2,0399	9,09745	0,0314205	2,89748	0,470861	0,030701	0,6279808
CG42320-RR	Doa	0,0211736	11,6891	0,0218522	0,023197	0,0313213	0,694964	0,419518	0,055501	0,6279808

gene_id	Symbol	W1_FPKM	W2_FPKM	W3_FPKM	MCM51_FPKM	MCM52_FPKM	MCM53_FPKM	FC	p-value	p-adj
CG42320-RS	Doa	0,0155011	9,9513	0,0204042	0,0231238	0,0308663	0,0249081	0,471869	0,030498	0,6279808
CG42320-RT	Doa	0,00927945	6,24178	5,35763	0,0227879	9,68289	0,0231427	0,475147	0,029593	0,6279808
CG42320-RU	Doa	0,0181245	0,0418931	4,69748	14,5903	13,4054	0,0236804	0,112753	0,612665	0,6279808
CG42320-RV	Doa	9,75724	0,0455038	0,0205032	9,43014	0,0317785	0,0236056	0,109668	0,621528	0,6279808
CG42321-RE	ATP8A	3,72823	0,0236675	0,0142086	0,0152685	9,30277	0,0161713	0,108957	0,607644	0,6279808
CG42321-RG	ATP8A	0,0139528	0,0233916	0,0133336	0,0142978	0,0361555	2,93855	0,099528	0,639744	0,6279808
CG42321-RH	ATP8A	0,0131398	0,0219659	0,0147231	0,0158412	15,8686	2,00796	0,099833	0,644967	0,6279808
CG42321-RI	ATP8A	0,0147997	0,0225333	4,01423	4,60719	0,0293441	0,016586	-0,037528	0,855518	0,6279808
CG42321-RJ	ATP8A	0,0138883	53,0334	3,51533	4,01143	0	0,0140912	0,141208	0,511364	0,6279808
CG42321-RK	ATP8A	0,0153356	0,0188633	0,0150872	0,0162474	5,11638	0,0149393	0,108488	0,615819	0,6279808
CG42321-RL	ATP8A	4,99305	3,42267	0,0128867	0,0138036	77,3588	1,53601	-0,009805	0,962542	0,6279808
CG42321-RM	ATP8A	4,06363	0,0127092	0,0136374	0,0146344	2,67009	0,399793	0,108364	0,609678	0,6279808
CG42321-RN	ATP8A	0,0157149	0,0119687	3,09023	0,0151596	0,0194593	0,538765	0,087812	0,675426	0,6279808
CG42321-RO	ATP8A	0,0134228	0,0134806	0,156248	0,391632	2,62868	0,173804	0,080102	0,702538	0,6279808
CG42321-RP	ATP8A	0,0142048	0,0126504	0,261703	0,244938	0,0206813	4,02049	0,081994	0,701099	0,6279808
CG42321-RQ	ATP8A	3,69647	0,0139687	0,202544	0	0,0193664	85,0838	-0,020343	0,922413	0,6279808
CG42322-RD	CG42322	0,0336492	0,03065	0,0323052	0,0353208	0,0491869	0,0370702	-0,338319	0,143447	0,6279808
CG42322-RE	CG42322	0,0330104	0,0300682	0,031692	0,0193371	0,048178	0,0363098	-0,335564	0,146919	0,6279808
CG42322-RF	CG42322	0,0327974	0,0298741	0,0314875	0,0616086	0,0478422	0,0360568	-0,338319	0,143447	0,6279808
CG42322-RG	CG42322	0,018578	1,96297	0,017836	0,0384989	2,54283	2,23988	-0,338319	0,143447	0,6279808
CG42322-RH	CG42322	0,0542197	0,0493871	0,0520542	12,0165	0,0834492	0,0628924	-0,281837	0,237219	0,6279808
CG42322-RI	CG42322	0,0355119	0,0323467	0,0340935	0,0444757	0,052147	0,0393011	0,142585	0,488285	0,6279808
CG42322-RJ	CG42322	10,3012	10,4579	10,9855	0,121943	6,0243	5,32214	0,145392	0,48049	0,6279808
CG42322-RK	CG42322	0,0405207	0,0369091	0,0389023	0,437977	0,0602426	0,0454025	-0,252004	0,287998	0,6279808
CG42323-RC	CG42323	0,109096	0,099372	0,0962622	0,144334	0,375927	0,102071	0,328621	0,288521	0,6279808
CG42323-RD	CG42323	0,353851	0,515699	0,305746	0,0839897	0,0904262	0,166256	0,069424	0,754445	0,6279808
CG42323-RE	CG42323	0,0525384	0,0478556	0,05044	0,378969	0,0805133	0,0606797	0,264028	0,415554	0,6279808
CG42324-RC	CG42324	0,0657034	84,213	0,0438806	0,0769295	47,8275	3,21534	-0,434325	0,181371	0,6279808
CG42324-RD	CG42324	3,11871	0,0598472	8,70562	5,28073	0,0588523	3,26394	-0,053329	0,855878	0,6279808
CG42325-RB	CG44008	0,0172177	0,0156831	0,01653	0,017864	0,0241969	0,0182362	-0,412127	0,120077	0,6279808
CG42325-RC	CG44008	16,7504	14,1811	16,7672	12,216	19,5487	13,9473	-0,12404	0,629795	0,6279808
CG42325-RD	CG44008	0,0117726	0,0107233	0,0113024	0,0120603	0,0163357	0,0123116	-0,185463	0,47264	0,13772387
CG42325-RE	CG44008	0,0129747	0,0118183	0,0124565	0,0133289	0,0180541	0,0136066	-0,193496	0,45456	0,13772387
CG42325-RF	CG44008	0,0135466	0,0123392	0,0130056	0,0139349	0,018875	0,0142253	-0,137284	0,63311	0,6279808
CG42325-RG	CG44008	0,0173488	0,0158025	0,0166559	0,0180055	0,0243886	0,0183807	-0,209586	0,418413	0,6279808
CG42326-RC	CG42326	3,27262	3,95626	2,59411	4,09896	8,91368	2,76527	-0,150452	0,536999	0,13772387
CG42326-RD	CG42326	4,61707	4,25958	0,474353	6,04129	183,917	5,98866	-0,488812	0,07002	0,6279808
CG42327-RE	CG42327	12,689	15,427	1741,27	10,3942	4,80667	0,0774474	0,319829	0,129276	0,6279808
CG42328-RB	C3G	4,47087	1,76766	2,43684	0,0120979	6,59573	3,58049	-0,007876	0,975105	0,13772387
CG42328-RC	C3G	3,54242	0,00879191	0,00926671	0,0117674	4,47994	4,37658	-0,000664	0,997904	0,6279808
CG42328-RD	C3G	0,00965222	0,00859971	0,00906412	8,31076	3,06557	11,194	0,008265	0,973864	0,13772387
CG42328-RE	C3G	0,00944121	0,0107559	0,0113368	1,05837	3,89762	2,36134	0,012107	0,961702	0,6279808
CG42328-RF	C3G	0,0118084	0,0104696	0,011035	3,73998	2,11336	0,17791	0,004679	0,985173	0,6279808
CG42328-RG	C3G	0,0114941	3,36765	8,56225	8,1547	0,31577	5,93983	0,015753	0,950289	0,6279808
CG42329-RA	CG42329	4,51964	4,34873	4,29329	0,508985	0,190598	2,7071	3,22269	1,31E-23	0,6279808
CG42330-RD	Got2	0,0438556	0	2,32417	1,65011	0,314223	0	-0,769168	0,000835	0,6279808
CG42330-RE	Got2	0,0219984	86,1699	26,8386	0,597414	0,0162732	1,59343	-0,751355	0,001102	0,6279808
CG42330-RH	Got2	65,8952	0	0,320388	0,0981972	5,37688	0,540389	-0,769168	0,000835	0,6279808
CG42331-RB	Dscam4	0,0101974	4,5195	5,36773	1,82703	0,73703	7,05575	0,372844	0,248455	0,6279808
CG42331-RC	Dscam4	0,96093	6,07543	0,680824	2,2319	4,12327	3,91587	0,378792	0,240676	0,6279808
CG42332-RA	Dscam4	0,523931	0,626943	11,0117	0,748355	7,23721	0,584267	-0,145534	0,544716	0,6279808
CG42332-RB	CG42331	0,0135494	13,8642	11,4853	3,1876	28,923	0,86567	-0,136792	0,572465	0,6279808
CG42332-RD	CG42331	10,8292	0,0123417	4,72693	0,0147656	0,823263	6,27703	-0,162763	0,493026	0,6279808
CG42332-RE	Camta	0,0096876	0,336237	0,00930067	0,846529	0,487585	0,567311	-0,153828	0,520533	0,6279808
CG42333-RC	Camta	1,21434	0	0,960101	0,29546	0,0145442	0,0109614	0,20908	0,369883	0,6279808
CG42333-RD	Camta	0,0105121	25,7269	0,0100923	0	1,09629	0,68381	0,227482	0,316314	0,6279808
CG42334-RC	Camta	0,536643	24,2886	0,777768	1,12863	1,26359	0,649894	-0,38759	0,157827	0,6279808
CG42334-RD	Sytbeta	4,94044	533,526	0	34,2042	2,54011	0,0664006	-0,531964	0,056026	0,6279808
CG42335-RC	Sytbeta	4,76358	0,488238	4,98333	19,252	11,905	0	-0,385796	0,163398	0,6279808
CG42336-RB	comm3	8,88559	0,753439	0	5,59857	0,390836	0,761089	-0,061581	0,807501	0,6279808
CG42336-RC	comm3	0,0499188	0	0,21021	0	57,5368	0	-0,086993	0,736515	0,6279808
CG42336-RD	CG42335	3,2048	3,92019	4,06281	41,369	117,94	10,6674	-0,087709	0,734395	0,6279808
CG42336-RE	CG42336	0,113597	30,2811	40,8429	47,5267	3,52286	0,124444	-0,109313	0,693244	0,6279808
CG42337-RB	CG42336	37,9118	37,8733	0,0130833	194,76	16,0417	0,155518	0,166233	0,496437	0,6279808
CG42338-RD	CG42336	54,1498	133,282	2,73974	5,68595	27,0314	47,9213	0,144976	0,625747	0,6279808
CG42338-RE	CG42336	143,382	0	64,4395	0,153597	75,3008	104,868	0,066494	0,824638	0,6279808
CG42338-RF	CG42337	3,65927	4,29733	3,35	4,05775	0,243525	2,46099	0,214874	0,46722	0,6279808
CG42338-RG	Ten-a	0,00596155	0,00560361	0,00590622	0,00622117	0,00842662	0,00686268	0,127455	0,669438	0,6279808
CG42338-RH	Ten-a	0,00560503	0,00525845	0,00554243	0,00583292	0,00790072	0,00672478	0,066311	0,825137	0,6279808
CG42338-RI	Ten-a	0,00615193	9,83702	7,70987	7,59892	2,65076	0,205591	0,233791	0,426888	0,6279808
CG42338-RJ	Ten-a	0,005773	0,00604849	0,00637513	0,0067226	0,0091058	0,0242787	0,214764	0,467514	0,6279808
CG42339-RA	Ten-a	7,91219	0,00592873	0,0062489	5,68421	1,44289	12,8122	-0,452135	0,205922	0,6279808
CG42339-RB	Ten-a	0,00664035	0,545497	0,191652	0,625142	0,27279	2,93343	0,097094	0,672699	0,6279808
CG4233-RA	Ten-a	0,00650887	9,98755	9,32974	10,8739	7,94555	7,49969	-0,47253	0,124159	0,6279808
CG4233-RB	CG42339	5,05309	5,20305	5,82152	5,74046	0,0288508	3,46099	-0,463819	0,130609	0,6279808
CG4233-RC	CG42339	0,109457	0,0997012	0,105085	0,14497	0,0291049	0,14799	-0,475729	0,121025	0,6279808
CG42340-RC	CG42340	1,60479	1,79908	1,61555	3,90234	46,2668	0,962474	0,121366	0,630239	0,6279808
CG42341-RC	Pka-R1	0,0186304	6,44855	0,0178863	0,0210549	7,44621	3,30675	-0,119462	0,653104	0,6279808
CG42341-RD	Pka-R1	0,0185937	0,0169364	0,0178511	0,022859	0	3,46037	-0,119462	0,653104	0,6279808
CG42341-RE	Pka-R1	0,0205417	13,4624	0,0197213	0,0215577	0	10,0468	-0,105621	0,699386	0,6279808
CG42341-RF	Pka-R1	0,0299573	0,0272872	0,0287608	14,5648	6,68279	8,23509	-0,099366	0,716	0,6279808
CG42341-RG	Pka-R1	0,0235509	0,0214517	0,0226102	0,179789	40,7937	0,187708	-0,096541	0,723787	0,6279808
CG42341-RH	Pka-R1	7,21479	0,0167433	11,6032	7,45252	3,90561	0,68937	-0,09869	0,717811	0,6279808
CG42341-RI	Pka-R1	0,0199164	0,0181412	0,0191209	0,006425	3,78159	8,32909	-0,108044	0,688588	0,6279808
CG42341-RJ	Pka-R1	0,0201533	0,018357	0,0193483	11,3659	9,15998	0,0197575	-0,119462	0,653104	0,6279808
CG42341-RK	Pka-R1	28,0287	19,535	10,6611	0,00615492	3,82756	0,021928	-0,453679	0,087546	0,6279808
CG42341-RL	Pka-R1	0,0206122	0,018775	0,0197889	16,8232	3,28616	0,032708	-0,388204	0,143451	0,6279808
CG42341-RO	Pka-R1	11,3734	0,0190785	22,8939	0,169596	0,0262694	0,0253206	-0,09876	0,717676	0,6279808
CG42342-RC	CG42342	0,0184124	0,0167713	0,017677	8,76509	0,0307388	0,04663	0,217597	0,407892	0,6279808
CG42342-RD	CG42342	10,2676	10,8871	9,33638	9,31974	0,0310597	0,0288873	0,206145	0,428202	0,6279808
CG42342-RE	CG42342	14,6031	19,6891	14,2298	10,5252	0,0309627	0,0511316	0,047095	0,858364	0,6279808
CG42342-RF	CG42342	12,3544	13,6731	14,028	16,9409	11,5086	0,023556	0,203725	0,440014	0,6279808
CG42343-RC	DIP-beta	0,034048	0,558239	0,964299	11,0407	12,0906	0,0375462	-0,282351	0,347128	0,6279808
CG42343-RD	DIP-beta	1,19285	1,29735	0,546967	0,0185688	0,0251515	0,952346	-0,437168	0,210516	0,6279808
CG42343-RE	DIP-beta	0,0437106	0,0398146	0,0419647	0,0185688	0,0251515	0,0493678	-0,41733	0,233221	0,6279808
CG42343-RF	DIP-beta	0,0463148	0,0421867	0,044465	0,0181544	0,0245902	0,240236	-0,275987	0,357342	0,6279808
CG42344-RB	brp	0,0195684	0,0178242	1,01043	3,09782	1,6079	1,04652	-0,392246	0,145056	0,6279808
CG42344-RD	brp	1,339	2,08295	0,00993269	0,0105638	0,0143087	0,0107839	-0,383398	0,151012	0,6279808
CG42344-RE	brp	0,0103459	0,00942377	0,00956718	0,0101661	0,01377	0,0103779	-0,381255	0,15393	0,6279808
CG42344-RF	brp	0,00996519	0,00907699	2,26861	4,93774	2,75206	2,09644	-0,36876	0,198346	0,6279808
CG42344-RG	brp	2,76482	2,39821	35,9551	29,3735	64,5658	49,8504	-0,383576	0,150907	0,6279808
CG42345-RA	stw	19,22	15,5911	16,2348	20,7012	11,9191	8,79541	0,470309	0,061555	0,6279808
CG42345-RB	stw	0,0262449	0,0239056	0,0251966	0,0278198	0,037682	0,0283995	0,477259	0,057569	0,6279808
CG42345-RD	stw	0,0262449	0,0239056	0,0251966	0,0278198	0,037682	0,0283995	0,183606	0,505227	0,6279808

gene_id	Symbol	W1_FPKM	W2_FPKM	W3_FPKM	MCM51_FPKM	MCM52_FPKM	MCM53_FPKM	FC	p-value	p-adj
CG42345-RE	stw	27,2184	26,4732	17,9326	20,4936	7,95754	10,8438	0,183483	0,50325	0,6279808
CG42346-RB	CG42346	0,870272	3,96114	0,515529	0,936599	1,16555	0,979355	0,041392	0,893955	0,6279808
CG42347-RB	sqa	10,382	15,8371	13,5757	18,2064	11,7302	14,8375	-0,761154	0,001161	0,6279808
CG42347-RC	sqa	0,0326838	0,0297707	0,0313784	0,0351888	0,0209287	0,035922	-0,405134	0,081256	0,6279808
CG42348-RA	CG42674	6,1308	5,66145	4,20953	5,30286	6,84672	4,47104	-0,105739	0,651257	0,6279808
CG42348-RB	CG42674	1,1497	1,32681	9,28652	0,0106251	0,0143917	1,13171	-0,100527	0,669757	0,6279808
CG42348-RC	CG42674	0,00973326	0,00886573	41,3339	1,54545	0,0134423	0,010131	-0,107877	0,645859	0,6279808
CG42348-RD	CG42674	0,00958501	0,00873069	0	0,00976966	0,0132331	0,00997324	-0,098344	0,675401	0,6279808
CG42349-RB	Pkcdelta	0,0226313	4,10222	0,0217274	2,51532	4,31539	0,00615159	-0,169195	0,449448	0,6279808
CG42349-RC	Pkcdelta	13,183	11,5072	11,6978	11,7917	11,1792	0,00577899	-0,191428	0,4178	0,6279808
CG42349-RD	Pkcdelta	2,42923	2,07222	2,73942	3,3896	2,71225	0,00635081	-0,192268	0,413847	0,6279808
CG42350-RA	moi	4,61527	4,81511	6,31644	0,0362536	0,0491057	0,037009	0,09006	0,692866	0,6279808
CG42351-RB	Jabba	0,962269	0,983391	0,0213904	31,6417	0,719567	3,28991	0,029893	0,924025	0,6279808
CG42351-RC	Jabba	1,79254	1,54035	1147,55	0,0749177	0,851695	1,91327	0,118952	0,689266	0,6279808
CG42351-RD	Jabba	0,0462176	0,0420982	0,0208903	0,673281	2,1941	0,781636	0,067595	0,825055	0,6279808
CG42351-RE	Jabba	1,95619	1,04534	0,0190234	0,0523564	0,0696987	0,103804	0,091755	0,761002	0,6279808
CG42352-RC	Ir40a	0,0434139	0	0,0416799	0,361318	0,203169	0,306406	-1,23019	0,000398	0,6279808
CG42354-RA	CG42354	33,241	47,0202	22,2665	0,0111576	16,1581	16,4938	0,492733	0,067675	0,6279808
CG42354-RB	CG42354	5,6883	6,19997	4,81798	12,8238	5,67033	4,42639	0,336756	0,204786	0,6279808
CG42354-RC	CG42354	0,0109132	0,00994051	0,0104773	0,023197	0,0151131	0,0113901	0,336747	0,204846	0,6279808
CG42355-RB	CG42355	0	0	0	0,0916417	0,477998	0	-0,041811	0,731452	0,6279808
CG42356-RA	CG42356	0,0780424	0	0,111684	0,332911	0,359869	0,250222	-0,099116	0,530826	0,6279808
CG42357-RA	CG42357	0	0	0	0,199495	0	0,235712	-0,386445	0,19701	0,6279808
CG42358-RA	Nsun5	19,1818	0,0678029	27,0136	11,1669	27,3252	5,25458	-0,396714	0,182182	0,6279808
CG42359-RA	CG42359	14,3292	0,025644	0,0270289	0,0197309	0,041618	0,020142	-0,414826	0,212573	0,6279808
CG42360-RA	Caf1-55	12,1247	10,0801	28,7739	18,0266	0,0685742	16,0699	0,016392	0,944194	0,6279808
CG42361-RA	CG42360	7,41077	5,95831	9,55924	0,0852944	0,313202	0,236048	0,101383	0,634288	0,6279808
CG42362-RA	CG42361	15,5392	20,1313	20,3435	0,179776	7,02539	6,59858	-1,922226	1,17E-10	0,6279808
CG42364-RA	CG42362	4,39897	8,3903	27,3185	0	26,0487	0,602792	-0,638941	0,0741	0,6279808
CG42365-RA	CG42364	0,114781	0,04496	8,15813	12,6205	0,209253	7,17912	-0,925552	0,001764	0,6279808
CG42366-RA	CG42365	6,27253	0,301234	0,0270558	18,8893	739,191	0,0674996	-0,268515	0,38979	0,6279808
CG42367-RB	CG42366	0,545181	26,0942	45,4853	0,910575	0,462406	0,92955	-0,172393	0,62309	0,13772387
CG42368-RA	CG42367	0,402902	0	0,515746	1,06345	0,561276	0,697188	0,480646	0,080512	0,6279808
CG42369-RA	DIP-epsilon	3,22579	327,358	5,0247	4,12205	3,37658	2,86645	-0,66205	0,028067	0,6279808
CG4236-RA	CG42369	2,15208	0,0346164	0,034167	0,0385891	210,471	0,0393932	-0,127441	0,668925	0,6279808
CG42370-RA	ArfGAP1	27,8159	12,6223	0,0240594	0,769677	12,6428	0,0282207	-0,001524	0,996593	0,6279808
CG42372-RB	Nepl6	0,637928	0,0324164	925,008	1206,1	43,0031	917,201	-1,386186	7,76E-06	0,6279808
CG42373-RA	CG42372	0,270063	0,245992	8,04181	22,7471	26,6177	34,0836	-0,620296	0,027278	0,6279808
CG42375-RA	Tfb5	21,748	16,5621	32,2128	34,592	39,4699	46,6338	-0,247981	0,375116	0,6279808
CG42376-RA	CG42375	23,4968	21,4803	4,35443	20,9553	4,61769	1,64424	-0,323437	0,244272	0,6279808
CG42376-RB	CG42376	81,8735	74,8389	73,4094	122,803	185,518	154,417	-0,323437	0,244272	0,6279808
CG42377-RA	CG42376	0,130071	0,118477	0,124876	0,21567	0,292126	0,220164	-0,6592	0,008872	0,6279808
CG42378-RA	RhoGEF3	0,456766	0,435679	0,459207	0,794016	0,69107	0,555608	0,297831	0,188115	0,6279808
CG42378-RB	RhoGEF3	0,0192997	0,0193236	0,0183999	8,1813	0,030952	0,0226823	-0,027682	0,896238	0,6279808
CG42378-RC	RhoGEF3	0,0191654	0,0187809	10,6019	0,0328224	0,0302491	0,0220142	-0,014455	0,945019	0,6279808
CG42378-RE	RhoGEF3	13,9877	14,7283	0,0181785	13,3256	0,0300961	5,87292	-0,01466	0,944164	0,6279808
CG42378-RF	RhoGEF3	0,0189347	2,71636	0,0209178	5,56628	0,0292096	2,41865	0,048873	0,817322	0,6279808
CG42378-RG	RhoGEF3	0,021788	2,37592	0,0204657	6,62318	4,56846	1,55824	-0,014226	0,945804	0,6279808
CG42378-RH	RhoGEF3	0,0213171	0,589291	0,0203671	0,789073	2,00402	0,357948	-0,035	0,868675	0,6279808
CG42378-RI	RhoGEF3	0,0212144	0,112779	0,0197951	0,171361	3,54203	0,174931	-0,033036	0,875918	0,6279808
CG4237-RA	RhoGEF3	0,0206186	0,484203	0,867599	0,521071	5,86096	0,397294	-0,106393	0,581693	0,6279808
CG42380-RB	CG4238	1,80836	0,0399467	0,88847	0,0120141	4,77312	0,58722	0,066622	0,744778	0,6279808
CG42382-RA	CG4238	0,762986	0,0200377	10,0219	13,4159	1,8023	0,0352444	0,157058	0,559478	0,6279808
CG42383-RA	CG4238	0,0190879	59,6272	0,042104	0,0108993	1,2032	0,0495497	0,001968	0,994088	0,6279808
CG42384-RB	CG42380	16,7839	2,94925	1,84208	0,0300119	0,0895623	12,6392	0,067121	0,83949	0,6279808
CG42386-RA	CG42382	5,61904	0,896157	1,39934	1,75981	1,30855	5,52444	0,067121	0,83949	0,6279808
CG42388-RE	CG42383	0,0842089	0,0767033	0,215588	0	0,210325	0	0,29466	0,320318	0,6279808
CG42388-RF	tal-1A	64,7082	54,6071	0	0,29889	0,594059	18,2406	0,410981	0,159762	0,6279808
CG42389-RD	tal-3A	24,7617	12,5186	0	10,571	17,9216	0,580183	-0,355262	0,298289	0,6279808
CG42389-RE	Nost	1,92467	0,0273492	2,97868	0,02858	0,0387117	1,02169	-0,153099	0,613342	0,6279808
CG42389-RF	Nost	1,33353	1,80793	317,083	2,90761	1,22101	2,00552	-0,174172	0,568308	0,6279808
CG42389-RG	mtgo	0,0227612	0,0207325	0,0218522	0,0243996	0,0330494	0,0249081	-0,149914	0,621123	0,6279808
CG4238-RA	mtgo	0,0093969	0,00855935	0,00902158	0,00957377	0,0129677	0,00977327	-0,233867	0,341302	0,6279808
CG4238-RB	mtgo	2,71667	3,94303	2,51268	8,09264	2,17516	2,41363	-0,239209	0,324542	0,6279808
CG4238-RC	mtgo	5,75104	8,23001	6,00175	12,257	4,08205	4,03537	-0,232058	0,347835	0,6279808
CG42390-RB	CG4239	0,0340831	10,3137	0,0327218	0,0558158	26,4317	4,18124	-0,203644	0,348459	0,6279808
CG42390-RC	CG4239	0,040127	0,0452643	0,0385243	8,34211	5,00912	0,0569788	-0,071416	0,747632	0,6279808
CG42390-RD	CG4239	32,536	0,0400264	35,2412	10,1792	0,0756028	3,83869	-0,209506	0,333021	0,6279808
CG42391-RA	CG42390	0,0129138	0,0117628	38,817	0,0132645	2,73462	30,1815	-0,107934	0,705662	0,6279808
CG42392-RA	CG42390	3,73671	3,88417	0,0381078	6,49117	0,118211	0,0443839	0,294193	0,408612	0,6279808
CG42393-RA	CG42390	19,1693	21,7762	0,0296157	17,4803	0,0460169	0,0346811	0,056932	0,731892	0,6279808
CG42394-RA	CG42391	0,124671	0,0269814	5,90911	49,0713	7,73332	9,6009	-0,191152	0,480299	0,6279808
CG42395-RA	CG42392	0,128385	0,00815903	0,0185019	13,2532	0,0273229	8,50439	-0,133708	0,656715	0,6279808
CG42396-RA	CG42393	0,144903	29,5706	13,6378	6,20703	12,3563	9,78214	0,086078	0,757791	0,6279808
CG42396-RB	CG42394	101,607	9,49869	109,851	0,141241	6,04581	0,144184	0,086078	0,757791	0,6279808
CG42396-RC	CG42395	0,1318	0,120053	0,210894	0,479746	0,392426	0,183609	0,08673	0,755896	0,6279808
CG42396-RD	wech	0,0157975	5,31307	5,09227	3,22024	1,70068	4,97412	0,095509	0,73209	0,6279808
CG42396-RE	wech	3,95058	3,44287	0,0151666	7,65211	4,96176	2,50525	0,09228	0,741102	0,6279808
CG42398-RA	wech	0,0145809	1,58284	5,0968	2,26125	1,07327	3,1219	NA	NA	0,6279808
CG42399-RA	wech	1,61607	5,2457	0,0139986	7,15612	2,35035	3,05409	0,183726	0,441443	0,6279808
CG4239-RA	wech	3,48965	0,0132813	6,15626	0,0150351	0,0203651	0,0155723	-0,137044	0,489573	0,6279808
CG4239-RB	SteXh:CG42398	1,02068	0	1,39251	0,756489	0,522753	1,11343	-0,13727	0,488682	0,6279808
CG4239-RC	CG42399	1,33634	72,4952	26,4692	20,3626	20,3239	0,965417	-0,136913	0,489538	0,6279808
CG42400-RC	CG42750	2,77934	3,59801	2,6135	7,77548	0,0274658	0	0,225904	0,375929	0,6279808
CG42401-RB	Gfrl	3,12894	3,40459	2,56221	0,652248	2,32736	0	0,259194	0,315734	0,6279808
CG42402-RA	CG42402	3,08578	3,25374	3,10744	3,57554	2,67013	1,75975	0,204057	0,443425	0,6279808
CG42403-RB	Ca-beta	2,79768	3,69877	2,56859	4,39784	2,23734	1,95594	-0,071974	0,813592	0,6279808
CG42403-RC	Ca-beta	0,042176	0,0384169	0,0404915	0,0464837	0,0629625	0,0474524	0,110789	0,724565	0,6279808
CG42403-RF	Ca-beta	0,870042	0,986574	0,0340935	1,14074	1,10167	0,692447	-0,085272	0,775625	0,6279808
CG42403-RG	Ca-beta	0,0389215	0,0354524	0,915492	0,0425514	0,0576361	0,043438	-0,011314	0,967184	0,6279808
CG42404-RC	CG42404	5,99565	7,2587	4,7	7,87363	3,18557	2,95636	0,270629	0,341556	0,6279808
CG4241-RA	DPCoAC	22,4435	11,6247	28,0931	15,4496	0,067896	0,0511705	-0,167593	0,447164	0,6279808
CG4241-RC	DPCoAC	0,0408969	22,6733	0,0392635	8,0807	75,9304	82,3685	-0,225222	0,321532	0,6279808
CG42445-RA	Su(dx)	6,64537	5,36714	4,38249	9,61361	0,0657454	5,43333	0,010057	0,971735	0,6279808
CG42446-RA	Su(dx)	0,0155339	0,0141493	0,0149134	0,0160534	0,0312664	0,016388	0,129007	0,571109	0,6279808
CG42447-RA	Su(dx)	11,377	12,6707	11,0227	9,72128	138,5	5,135	0,582798	0,086053	0,6279808
CG42448-RA	CG42445	6,88148	56,3364	11,4712	0,0139349	0,018875	8,50837	0,373022	0,278045	0,6279808
CG42449-RA	CG42446	0,0646112	0,0588524	0,0620306	227,662	5,01413	0,0910803	-0,09147	0,718767	0,6279808
CG42449-RB	inaF-B	0,0215958	0,020315	0,0217274	127,459	0,0322144	1,17947	-0,09147	0,718767	0,6279808
CG4244-RA	Skadu	0,655515	0,656797	0,219125	0,375791	1,71894	0,3334	0,243474	0,280543	0,6279808
CG4244-RB	CG42449	0,0567336	0,220127	0,0544677	21,7298	1,91264	0,058644	0,232238	0,303481	0,6279808
CG4244-RC	CG42449	0,151042	21,3762	0,145009	4,07768	12,1547	17,676	0,241954	0,283567	0,6279808
CG42450-RA	CG42450	2,05396	1,85574	2,12361	3,1616	2,65577	1,66245	-0,382949	0,128741	0,6279808
CG42456-RA	CG42456	0,0285795	23,2303	56,8366	7,26718	0,0970622	3,86604	-0,169181	0,442162	0,6279808

gene_id	Symbol	W1_FPKM	W2_FPKM	W3_FPKM	MCM51_FPKM	MCM52_FPKM	MCM53_FPKM	FC	p-value	p-adj
CG42457-RA	cpo	10,5402	13,8649	14,1964	19,965	7,19303	7,27079	0,543308	0,038674	0,6279808
CG42457-RB	cpo	15,4189	16,2979	9,39546	7,69672	3,57512	3,74672	0,544045	0,038462	0,6279808
CG42458-RA	CG42458	4,20916	0,12565	0,682116	4,87102	0,598987	3,41438	-0,027307	0,910704	0,6279808
CG42459-RA	Sfp23F	0	4,70003	7,54545	0	6,45289	0	NA	NA	0,6279808
CG42460-RA	CG42460	1,04589	4,7046	0	0	0,400203	0	0,59164	0,049068	0,6279808
CG42461-RA	Sfp24Ba	0	37,4598	0	0	0	0	0,01562	0,897983	0,6279808
CG42462-RA	Sfp24Bb	0	0,569948	0	0	0	0	NA	NA	0,6279808
CG42463-RA	Sfp24Bd	0	172,08	0	0	0	0	NA	NA	0,6279808
CG42464-RA	CG42464	2,0023	0	1,56189	0,629238	0,0249826	0,0188284	-0,313469	0,372591	0,6279808
CG42465-RA	CG42465	0	0	56,7513	0	0,021851	0,0164682	-0,041811	0,731452	0,6279808
CG42466-RA	Sfp24C1	0,276083	0	0,265056	56,3911	2,77894	0,0166023	-0,150077	0,538036	0,6279808
CG42467-RA	CG42467	0	0	0	40,938	0,0232756	0,0175419	0,01562	0,897983	0,6279808
CG42468-RA	Sfp24F	0	0	0,0713038	4,20223	9,29181	1,35053	NA	NA	0,6279808
CG42469-RA	Sfp26Ac	0	0,598613	0,689361	0,605689	0,00353797	0,00266643	NA	NA	0,6279808
CG42470-RA	mRpS10	0,0916448	0,0834764	0,0879845	0,127076	0,172126	0,129724	0,01562	0,897983	0,6279808
CG42471-RA	mRpS10	32,9429	27,7984	60,8811	38,6788	41,6408	64,7333	-0,037875	0,76416	0,6279808
CG42472-RA	CG42470	0	0	0	0	0,313658	0	-0,070526	0,562659	0,6279808
CG42473-RA	CG42471	0	0	0	1,60276	0	0,111056	NA	NA	0,6279808
CG42474-RA	Sfp33A1	0	0	0	0,383451	0	0,236392	-0,037121	0,867429	0,6279808
CG42475-RA	Sfp33A2	0	0	0	0	0	0	NA	NA	0,6279808
CG42476-RA	Sfp33A3	0,235425	0,214441	0	0,728525	0,612613	0	-0,009814	0,969933	0,6279808
CG42477-RA	Sfp35C	0	0	0	1,38874	0	0,0134515	0,645744	0,044648	0,6279808
CG42478-RA	Sfp51E	0,184914	0,336865	0,177528	0,477351	0,864902	0	-0,16196	0,649591	0,6279808
CG42479-RA	Sfp53D	1,19056	0,619203	0	0,19746	0	0	-0,119495	0,614082	0,6279808
CG4247-RA	Sfp60F	1,24203	0,603373	0	0,0104731	3,09452	0	-0,041393	0,892787	0,6279808
CG4247-RB	Sfp65A	0,178906	0	67,4737	0,281171	0,613467	0,28703	-0,041393	0,892787	0,6279808
CG42480-RA	Sfp70A4	0	27,694	26,1249	0	0	17,1316	NA	NA	0,6279808
CG42481-RA	CG42481	0	0	0	0	0	0	0,149605	0,339384	0,6279808
CG42482-RA	Sfp77F	0	0	8,64704	0	3,88966	5,59705	NA	NA	0,13772387
CG42483-RA	Sfp79B	0,674244	0,239469	0,647314	10,0033	0,76598	0,577289	0,171432	0,48692	0,13772387
CG42484-RA	Sfp84E	0,0964611	0,0952817	0,100427	0,263985	0,0260318	0,139403	0,121175	0,631919	0,6279808
CG42485-RA	Sfp87B	0	4,2757	16,7662	0	14,2346	0,026229	NA	NA	0,6279808
CG42487-RA	CG42487	2,93949	1,09745	3,65424	7,3547	4,7967	6,21711	0,358434	0,217782	0,6279808
CG42488-RB	CG42488	0,211782	0,0450273	0,126557	10,2898	15,9213	22,0585	-0,318717	0,295381	0,6279808
CG42489-RA	CG42489	5,64751	9,38806	10,9794	35,4486	51,1912	31,4142	-0,431912	0,176851	0,6279808
CG42490-RA	c(2)M	0,029849	0,0271886	23,2572	0,0319162	13,1586	0,0325813	0,153484	0,551065	0,6279808
CG42492-RA	CR42490	0,241153	0,698219	0,662333	39,2148	0,982107	0,778609	0,324208	0,196098	0,6279808
CG42492-RB	OtopLa	26,9483	34,8824	70,6921	106,222	11,7418	14,3734	-0,357713	0,294198	0,6279808
CG42492-RC	OtopLa	0,0157561	0,0143518	28,4938	35,7003	1,57755	0,0166311	0,084656	0,760004	0,6279808
CG4249-RA	OtopLa	0,0557288	0,0507616	0,0151268	0,0162916	0,0933076	0,0648947	-0,302502	0,271239	0,6279808
CG4250-RA	CG4250	16,6715	7,18875	7,03576	9,16685	15,1555	18,5213	0,290246	0,352169	0,6279808
CG4252-RA	mei-41	0,218678	0,246992	0,323314	0,50401	0,168328	12,4287	-0,173839	0,595418	0,6279808
CG4254-RA	tsr	382,272	241,409	2,24059	545,05	1,35063	1,31612	-0,388823	0,211053	0,6279808
CG4257-RB	Stat92E	18,1795	13,1244	21,9698	0,0580789	12,6398	4,31987	0,24287	0,186347	0,6279808
CG4257-RC	Stat92E	0,0183765	7,28966	0,0176426	6,33122	2,9817	0,0182709	0,235539	0,202741	0,6279808
CG4257-RE	Stat92E	0,0165701	0,0150932	0,0159082	11,4783	0,0232513	25,1417	0,254699	0,166619	0,6279808
CG4257-RF	Stat92E	7,37998	4,89749	5,87799	0,0500499	3,04295	0,0592891	0,254699	0,166619	0,6279808
CG4257-RG	Stat92E	0,0258738	0,0157117	0,0248404	50,7522	0,0242429	0,0458669	0,24287	0,186347	0,6279808
CG4258-RA	dbe	20,9659	12,772	30,385	23,0333	25,3188	0,024734	-0,222437	0,438026	0,6279808
CG4259-RA	CG4259	1,69607	0,772447	3,25665	3,24789	1,86764	6,21454	-1,018178	0,003904	0,6279808
CG4260-RA	AP-2alpha	17,5311	16,4449	12,0724	9,62811	0,150843	7,70341	0,271519	0,259175	0,6279808
CG4260-RB	AP-2alpha	8,53635	8,94344	8,77351	17,2838	0,0624853	6,75379	0,293327	0,221203	0,6279808
CG4261-RA	Hel89B	10,2289	10,4032	0,0235825	0,0264889	0,344596	0,324603	0,177344	0,428575	0,6279808
CG4262-RA	elav	3,3936	4,40072	0,3584	5,15607	2,29523	0,480957	0,073701	0,77015	0,6279808
CG4262-RB	elav	7,35974	4,8998	0,591862	5,95727	0,0788768	0,32623	0,09785	0,698107	0,6279808
CG4262-RC	elav	6,20623	7,26218	13,2155	13,0277	0,0797222	12,3212	0,090223	0,719879	0,6279808
CG4264-RA	Hsc70-4	146,521	190,8	265,423	785,349	433,258	365,104	-0,497402	0,020227	0,6279808
CG4264-RB	Hsc70-4	511,853	769,26	665,972	834,19	410,159	563,912	-0,497068	0,020313	0,6279808
CG4264-RC	Hsc70-4	0,0295818	0,0269451	0,0284003	0,03161	127,831	0,0322687	-0,496825	0,020378	0,6279808
CG4264-RD	Hsc70-4	0,0287078	0,0261491	0,0275612	0,0306114	0,0414633	0,0312493	-0,496917	0,020353	0,6279808
CG4264-RE	Hsc70-4	0,0288709	0,0262976	0,0277178	0,0307974	0,0417153	0,0314391	-0,496817	0,020381	0,6279808
CG4264-RF	Hsc70-4	0,0286208	0,0260698	0,0274777	0,0305122	0,0413289	0,031148	-0,497042	0,020325	0,6279808
CG4265-RA	Uch	5,05219	13,503	0,0648195	16,6329	0,0309735	13,163	0,580349	0,04372	0,6279808
CG4266-RA	CG4266	12,0577	21,712	0,0229705	0,0252215	0,0314982	0,0257471	0,124275	0,603962	0,6279808
CG4266-RB	CG4266	2,60279	136,614	10,954	8,34132	15,4294	8,94673	0,115839	0,628295	0,6279808
CG4267-RA	CG4267	4,07043	0	0	0	0	0	-0,952759	0,006789	0,6279808
CG4268-RA	Pitslre	0,0966022	9,35614	15,1829	2,16055	0,185352	0,139692	-0,085096	0,640696	0,6279808
CG4268-RB	Pitslre	14,8731	213,102	0,106438	18,0382	0,0283379	4,4377	0,311949	0,132966	0,6279808
CG4268-RC	Pitslre	0,110866	0,0325215	16,7549	0,136841	0,2268	0,170931	-0,085096	0,640696	0,6279808
CG4268-RD	Pitslre	14,4164	177,609	26,2754	7,24233	22,5252	20,6773	0,311949	0,132966	0,6279808
CG4269-RA	CG4269	12,8616	0,238203	5,96757	8,05351	0,830383	8,57368	-0,649407	0,050307	0,6279808
CG4270-RA	CG4270	0	0	0	0	0	1,88888	NA	NA	0,6279808
CG4270-RB	CG4270	0	0	0	0	0	0	NA	NA	0,6279808
CG4271-RA	CG4271	0,226598	0	0	0	0	0	0,066786	0,714408	0,6279808
CG4272-RA	Axud1	0,0162242	0,0147781	0,0155762	0,0273577	0,0227476	0,017144	0,274989	0,265749	0,6279808
CG4272-RB	Axud1	0,017721	0,0161415	8,41305	16,5019	0,0249337	0,0187915	0,291305	0,236724	0,6279808
CG4274-RA	fzy	0,0326623	65,2478	101,973	97,9837	3,69163	5,80671	-0,205346	0,442877	0,6279808
CG4276-RA	aru	0,0230336	0,0209806	0,0221137	0,0297409	3,1921	0,0249081	-0,018555	0,929349	0,6279808
CG4276-RC	aru	0,0231872	6,42064	0,0222611	34,8863	0,0330494	20,6021	-0,015198	0,942323	0,6279808
CG4276-RD	aru	22,4207	19,1662	25,1555	18,7205	13,6288	4714,84	-0,020536	0,921846	0,6279808
CG4278-RA	CG4278	12,8195	12,095	19,4213	11,6196	10,6168	14,1152	0,171308	0,511664	0,6279808
CG4279-RA	LSm1	24,0314	0,0217935	150,729	5,94335	39,5199	157,456	0,038759	0,882157	0,6279808
CG4280-RA	crq	0,0242866	11,0693	1,98345	13,2681	4,6359	5,24167	0,317144	0,131782	0,6279808
CG4280-RB	crq	13,4523	0,0221219	0,396607	0,0256236	5,21859	5,27787	0,327819	0,118579	0,6279808
CG4281-RA	CG4281	8,11655	17,5791	0,0236557	0,83326	2,7422	0,140619	0,248047	0,290074	0,6279808
CG4282-RA	CG4282	3,67549	11,5496	4,88179	8,39431	186,176	238,282	0,221876	0,392861	0,6279808
CG4286-RA	CG4286	0	11,0901	8,7574	0,254112	6,14485	7,67955	-0,203847	0,507653	0,6279808
CG4287-RA	CG4287	11,4502	9,14202	18,2439	10,6187	6,43737	2,81135	-0,000592	0,998313	0,6279808
CG4288-RA	MFS9	0,0344743	5,17884	39,6849	180,416	432,797	0	-0,522041	0,128725	0,6279808
CG4288-RB	MFS9	5,62532	0,0314016	0,315555	0,0372792	0,0504948	13,6015	-0,523627	0,127674	0,6279808
CG4289-RA	Pex14	24,2663	3,82442	41,1482	6,07037	1,31705	0,571187	-0,051107	0,844473	0,13772387
CG4290-RA	Sik2	2,96641	1,02119	2,82048	0,50044	10,6363	0,0265604	-0,189706	0,515375	0,6279808
CG4291-RA	CG4291	6,12286	0,0210663	7,96022	0,0248224	4362,11	0,0253397	-0,071056	0,775573	0,6279808
CG4293-RA	CG4293	0,0379819	0,0345965	0,0191036	0,0414277	2,27671	0,021209	-0,01932	0,921813	0,6279808
CG4293-RB	CG4293	4,05245	0,0367289	0,0189552	5,04164	0,670264	0,0210365	-0,017364	0,9298	0,6279808
CG4293-RC	CG4293	4,42762	8,62096	1,55055	7,52171	20,6608	0,0478575	-0,019291	0,921933	0,6279808
CG4293-RD	CG4293	0,0343668	0,0469554	1,97599	0,037153	3,65596	2,17685	-0,02261	0,908801	0,6279808
CG4294-RB	CG4294	6,76033	9,83998	0,0149053	16,3021	14,5683	12,9099	0,029282	0,89343	0,6279808
CG4297-RA	CG4297	3,14386	9,6554	6,60573	4,51617	1,28764	0,0167762	0,166109	0,583502	0,6279808
CG4297-RB	CG4297	0,0158887	4,09773	3,84724	0,0164338	0,683649	6,46426	0,170148	0,573343	0,6279808
CG4299-RA	Set	29,5559	22,5065	52,5252	48,0452	25,7241	36,4814	-0,062016	0,823901	0,13772387
CG4300-RA	Sms	40,8585	31,8006	0,0142437	5,3303	0,03436	0,0187392	0,399057	0,084014	0,6279808
CG4300-RB	Sms	0,0339256	0,0309018	0,0169677	2,1535	0,0340714	0,018625	0,396138	0,084935	0,6279808
CG4301-RA	CG4301	10,3108	5,71064	11,2642	11,7787	8,5636	6,53904	-0,125657	0,629911	0,6279808

gene_id	Symbol	W1_FPKM	W2_FPKM	W3_FPKM	MCM51_FPKM	MCM52_FPKM	MCM53_FPKM	FC	p-value	p-adj
CG4302-RA	Ugt49B1	0,0388986	0,0354316	30,0814	22,7377	37,8622	33,6515	-0,190157	0,483204	0,6279808
CG4303-RA	Bap60	19,0718	6,25778	34,2799	33,7397	16,7883	6,22194	-0,125601	0,621817	0,6279808
CG4306-RA	CG4306	27,031	0	0,714378	21,1847	8,4156	3,92772	-0,103214	0,729188	0,6279808
CG4307-RA	Dpse\GA18097	177,764	34,6528	36,2496	126,021	233,308	158,155	-0,725161	0,028779	0,6279808
CG4307-RB	Dpse\GA18097	0,0817095	158,413	26,0098	18,6574	18,4183	23,553	-0,725107	0,028803	0,6279808
CG4311-RA	Dsim\Hmgs	0,0255811	0,023301	0,0245593	6,02178	0,0374921	0,0282563	0,278032	0,237515	0,6279808
CG4311-RB	Dsim\Hmgs	0,0342895	0,0312333	0,03292	15,1163	0,0517539	0,0390049	0,312033	0,195058	0,6279808
CG4311-RC	Dsim\Hmgs	0,0340247	0,030992	0,0326657	21,6289	0,0513076	0,0386685	0,311753	0,195422	0,6279808
CG4311-RD	Dsim\Hmgs	0,0349608	0,0318447	0,0335644	30,5207	0,0528886	0,0398601	0,313164	0,193819	0,6279808
CG4311-RE	Dsim\Hmgs	120,265	203,407	127,608	13,1059	85,1559	112,773	0,313626	0,193319	0,6279808
CG4312-RA	MtnB	1,86593	0,499888	0	0,0523981	12,2871	0,189971	-0,742411	0,015405	0,6279808
CG4313-RA	CG4313	0,739195	4,63574	5,18736	6,56906	4,66337	2,55634	0,26191	0,301973	0,6279808
CG4313-RB	CG4313	5,41838	8,62718	0,206859	6,79022	0,0352418	10,1991	0,269969	0,289721	0,6279808
CG4314-RA	st	2,71081	2,94619	239,212	0,0134871	74,955	1,46028	-0,564132	0,010985	0,6279808
CG4316-RA	Sb	2,94701	3,38529	3,00561	3,50395	0,078323	0,0106423	0,452803	0,122207	0,6279808
CG4317-RA	Mipp2	15,2671	0,0180325	0,780152	0,484944	0,0547711	1,41868	0,433218	0,043585	0,6279808
CG4318-RA	CG4318	0,0468401	0,0426652	0,0449693	0,0522319	0,0707485	0,0533203	0,398433	0,26331	0,6279808
CG4319-RA	rpr	18,7901	10,451	5,44225	6,26893	4,07696	8,43322	-0,171875	0,388969	0,6279808
CG4320-RA	raptor	2,66337	2,06872	15,3497	1,14815	13,2052	2,48472	-0,445618	0,091364	0,6279808
CG4321-RA	Cyp4d8	0,167175	0,340936	0,215608	0	249,134	0,834551	0,303845	0,392806	0,6279808
CG4322-RA	moody	15,8618	5,8643	16,3176	14,7526	0,677849	4,0028	0,389306	0,045425	0,6279808
CG4323-RA	37591	0	0	0	23,6933	139,717	0,238473	-0,074864	0,550506	0,6279808
CG4323-RB	37591	0	0	12,3291	0,0264464	727,335	2,71929	-0,074864	0,550506	0,6279808
CG4324-RA	CG4324	5,22049	0,520481	6,53185	0,260601	0,138649	0,176186	0,60445	0,040186	0,6279808
CG4325-RA	CG4325	1,0712	1,28384	1,51555	1,62826	1,91487	0,830647	0,177838	0,615934	0,6279808
CG4326-RA	mRpS17	31,6847	18,872	11,3432	21,4642	33,1099	53,7692	-0,128241	0,693952	0,6279808
CG4328-RA	CG4328	3,38053	5,38391	4,49991	4,51116	12,8954	0,0374	0,406263	0,135655	0,13772387
CG4329-RA	CG4329	0,0319877	0,0291366	0,0307101	0,0343809	2,88965	0,0350973	0,22431	0,414376	0,6279808
CG4329-RB	CG4329	0,0179229	0,0163254	0,0172071	0,0186265	35,1126	0,0190146	0,285573	0,325054	0,13772387
CG4330-RA	MFS10	10,2884	11,7142	24,3003	35,6192	10,3738	18,1159	-0,536492	0,010386	0,13772387
CG4332-RA	CG4332	4,92491	3,43122	5,54439	5,53121	2,89667	4,03104	0,134095	0,595858	0,13772387
CG4334-RB	Zip88E	15,925	13,7659	18,429	16,9985	8,55192	10,9895	0,039878	0,867327	0,6279808
CG4335-RA	CG4335	5,1973	17,0231	0,0171745	31,4524	14,8212	18,6926	0,081485	0,766153	0,13772387
CG4336-RA	rux	5,88381	5,17998	8,15413	7,63456	4,82917	6,39984	-0,001294	0,995811	0,6279808
CG4337-RA	mtSSB	29,6636	22,8762	0,00980526	37,586	0,236413	9,62989	-0,093234	0,743275	0,13772387
CG4337-RB	mtSSB	0,12546	0,114278	10,5307	0,174538	1,33356	1,03809	-0,093234	0,743275	0,13772387
CG4338-RA	CG4338	14,707	14,9345	378,626	253,294	16,273	16,7637	-0,101673	0,710615	0,13772387
CG4341-RA	CG4341	9,17124	0,0228875	9,9017	12,2943	7,2947	7,52046	0,042111	0,836916	0,6279808
CG4341-RB	CG4341	0,0158354	0,0283008	0,0152029	0,0163766	0,0221822	0,0167179	0,041608	0,838789	0,6279808
CG4345-RA	grim	10,1101	20,1211	20,0523	16,9234	21,8774	5,58055	0,643007	0,025894	0,6279808
CG4347-RA	UGP	3,59506	3,75218	2,93764	18,0917	25,5643	20,2874	-0,602202	0,034457	0,13772387
CG4347-RC	UGP	16,1682	12,5882	14,205	7,43617	5,05023	3,75516	-0,599699	0,034315	0,13772387
CG4349-RA	Fer3HCH	1,00115	1,07284	0,848085	12,3456	1,69988	1,65453	0,019781	0,955118	0,13772387
CG4351-RA	CG43313	2,59898	2,73849	24,6177	20,0039	2,47217	1,89598	0,133176	0,549683	0,13772387
CG4353-RA	hep	16,1428	20,4283	13,3523	17,7169	8,61209	11,0234	-0,002334	0,992593	0,13772387
CG4353-RB	hep	2,82424	3,40316	3,55427	7,80377	2,49207	2,87977	0,172594	0,498219	0,13772387
CG4353-RC	hep	5,83895	7,08761	6,21145	7,27758	3,45557	4,50766	0,016702	0,948129	0,13772387
CG4354-RA	slbo	74,368	127,926	69,4388	258,054	3,39569	106,082	0,743641	0,022097	0,13772387
CG4356-RA	mAChR-A	2,46074	1,59291	2,42882	3,70736	0	2,31288	0,281544	0,226301	0,6279808
CG4356-RB	mAChR-A	4,67412	5,16757	3,42092	2,62534	0	1,47457	0,281544	0,226301	0,6279808
CG4357-RA	Ncc69	0,0167677	0,122164	0,128762	9,32272	0,759068	0,0270409	-0,597776	0,001044	0,13772387
CG4357-RB	Ncc69	11,5021	0,0152732	0,016098	0,0173787	18,2515	3,80299	-0,597958	0,001046	0,13772387
CG4360-RA	CG4360	18,3583	0,599467	0,46335	0	0	0,394904	-0,148343	0,482986	0,13772387
CG4360-RB	CG4360	0,0250224	20,0268	19,0673	85,7834	0	1,57728	0,11473	0,582293	0,13772387
CG4362-RA	CG4362	0	0,126265	4,34742	0,964229	1,41766	10,6996	0,060066	0,796035	0,6279808
CG4363-RA	CG4363	3,45463	0,08525	0,0898538	7,08375	0,120438	0	-0,316572	0,306986	0,13772387
CG4364-RA	CG4364	59,3687	72,9297	0,601867	99,3424	7,90672	0,299881	-0,251145	0,264865	0,6279808
CG4365-RA	tzn	10,3495	0,0879919	8,84152	86,2391	5,87924	5,99538	-0,314331	0,309408	0,6279808
CG4365-RB	tzn	4,89843	13,7116	16,0634	0,0387252	7,01977	6,04559	-0,308816	0,317569	0,6279808
CG4365-RC	tzn	4,38556	0,100984	16,9178	162,514	17,9892	16,9451	-0,32152	0,2971	0,6279808
CG4367-RA	CG4367	0,890729	0,331911	5,80355	4,6106	6,57579	2,68749	-0,264087	0,373595	0,6279808
CG4370-RA	Irk2	14,9367	5,07518	30,4702	0	0	0,6272	-0,861749	0,014067	0,13772387
CG4370-RB	Irk2	3,03336	0,850152	4,32523	1,17035	1,62033	0	-0,870688	0,013292	0,6279808
CG4371-RA	GstD7	5,82447	3,03162	555,986	19,1775	25,8527	15,1313	-0,28273	0,429471	0,6279808
CG4372-RA	CG4372	0	0,620479	22,365	0,24864	0,156302	16,4336	0,044335	0,715924	0,6279808
CG4373-RA	Cyp6d2	12,7185	22,269	36,4762	19,5465	2,53282	3,81983	1,329268	8,19E-06	0,6279808
CG4374-RA	CG4374	1,5769	0,107668	0,165136	0,107972	5,62738	0,991296	0,130307	0,679125	0,6279808
CG4375-RA	CG4375	0	0	0,120297	0,167942	0	8,53802	-0,145027	0,424098	0,6279808
CG4376-RA	Actn	16,1882	7,75225	13,5563	5,05852	151,607	24,459	-0,18346	0,33675	0,6279808
CG4376-RB	Actn	105,776	36,2278	21,4758	7,20883	0,0267978	0,048339	-0,192533	0,31266	0,6279808
CG4376-RC	Actn	25,398	135,543	138,318	0,99513	7,09772	1,67799	-0,193064	0,311248	0,6279808
CG4377-RA	CG4377	9,61261	1,9442	0,417347	0	0	0,0563507	-0,424552	0,20054	0,6279808
CG4379-RA	Pka-C1	18,1877	10,43	0,0217721	0,0341847	217,806	262,187	-0,318791	0,182061	0,6279808
CG4379-RB	Pka-C1	0,0226779	43,3343	0,0305474	15,0906	12,1905	11,9663	-0,120302	0,5865	0,6279808
CG4379-RC	Pka-C1	0,0318182	22,2957	208,562	34,0724	14,0182	22,1474	-0,119881	0,587646	0,6279808
CG4380-RA	usp	11,6854	0,0172966	0,0182307	202,91	34,2216	3,6057	0,166423	0,461246	0,6279808
CG4381-RA	GstD3	11,8496	12,92	27,0664	0,0256936	21,1341	2,87446	-0,528114	0,064607	0,6279808
CG4382-RB	CG4382	0,28432	2,8486	3,57363	6,31778	10,2057	11,7729	-0,586529	0,101191	0,6279808
CG4385-RA	S	3,94379	4,51513	4,79112	10,3584	0	1,50699	0,021793	0,943244	0,6279808
CG4385-RB	S	2,76665	5,36715	2,48381	4,0734	0	3,18236	-0,019305	0,949496	0,6279808
CG4386-RA	tpr	16,924	0,0756112	14,5279	0	0	0,0514445	0,089678	0,740443	0,6279808
CG4389-RA	Mtpalpha	18,5607	9,42986	29,2257	24,856	25,2013	17,5511	-0,315555	0,284645	0,6279808
CG4389-RB	Mtpalpha	0,0234173	0,0213301	0,022482	0,0246553	0,0333957	0,025169	-0,318926	0,279645	0,6279808
CG4389-RC	Mtpalpha	0,0260483	4,85209	10,7659	4,76564	0,0373822	13,5953	-0,316221	0,284173	0,6279808
CG4390-RA	CG4390	19,228	15,1441	0	10,2066	5,85078	3,98097	-0,512013	0,127961	0,6279808
CG4390-RB	CG4390	0,0599236	0,0545826	0	0	1,18037	0	-0,512756	0,127349	0,6279808
CG4393-RA	CG4393	3,04501	1,31185	1,257	0	0	0	0,00763	0,978529	0,6279808
CG4394-RA	Traf-like	0,0289341	0,0263552	0,0277785	0,0308695	0,041813	0,0315128	0,123869	0,622217	0,6279808
CG4394-RB	Traf-like	43,4881	45,3884	35,6448	24,2419	32,1773	29,5864	0,132892	0,596231	0,6279808
CG4394-RC	Traf-like	0,0300937	0,0274115	0,0288918	0,0321969	11,8357	0,0328678	0,123869	0,622217	0,6279808
CG4395-RA	hec	0,901209	0,489037	0	0	0	0	-0,254977	0,469476	0,6279808
CG4396-RA	fne	45,711	1,70279	2,85881	56,9053	1,66723	1,75661	0,702405	0,004605	0,6279808
CG4396-RB	fne	42,5703	40,8125	51,5353	0	0,101767	0,204576	0,719185	0,003536	0,6279808
CG4398-RA	jtb	9,19997	5,1569	11,2284	0,0669631	12,436	10,7057	-0,169672	0,600461	0,6279808
CG4399-RB	east	3,47981	0,0190062	11,0441	6,91454	0,086356	0,0650831	-0,258245	0,323596	0,6279808
CG4400-RA	Brms1	18,9695	13,7854	20,6304	25,0038	15,0417	18,8267	0,158833	0,541833	0,6279808
CG4402-RA	Loxl2	0,766203	20,4411	0	0,0256336	9,70665	0	-0,245819	0,431074	0,13772387
CG4404-RA	CG4404	0,616451	0,238174	0,510215	10,9826	19,2455	17,0667	0,35936	0,314378	0,13772387
CG4405-RA	jp	6,69611	5,35296	34,0402	9,03061	32,3137	35,8928	0,174312	0,510421	0,13772387
CG4405-RB	jp	3,78045	9,68187	1,00729	7,62197	0,514024	0,0687076	0,177976	0,502092	0,6279808
CG4405-RC	jp	51,765	65,1525	0,0220983	38,0843	0,0327944	2,81832	0,3007	0,259374	0,6279808
CG4406-RA	PIG-K	9,86415	26,8025	12,6521	10,4107	2422,56	2781,86	-0,354154	0,197668	0,6279808
CG4407-RA	CG4407	11,1001	8,6412	2,53377	0,762569	21,4928	0,697783	-0,223918	0,329562	0,6279808
CG4407-RB	CG4407	9,67607	0,00478861	2,21494	76,1773	35,23	1,44902	-0,207506	0,365446	0,6279808

gene_id	Symbol	W1_FPKM	W2_FPKM	W3_FPKM	MCM51_FPKM	MCM52_FPKM	MCM53_FPKM	FC	p-value	p-adj
CG4408-RA	CG4408	2,01301	1,7636	3,34887	2,87867	2,85143	3,03208	-0,353459	0,208723	0,6279808
CG4409-RB	CG4409	57,2923	54,9185	0	0,117432	0,324532	0,156789	-0,491008	0,07869	0,6279808
CG4412-RA	Dpse\GA18167	223,591	229,277	0,0225193	303,763	441,679	0,0257274	-0,11648	0,699215	0,6279808
CG4413-RA	trem	3,76438	3,81897	0,101175	0	0	4,09378	-0,026546	0,912335	0,6279808
CG4414-RA	Ugt317A1	24,6087	29,5608	24,5854	35,4605	12,5205	19,1841	0,213384	0,418424	0,6279808
CG4415-RA	CG4415	1,50935	1,63669	2,55311	3,11656	3,38557	0,0375571	-0,672352	0,018975	0,6279808
CG4420-RA	rngo	10,9042	28,0645	44,5229	4,48024	0,0164866	11,1874	-0,029888	0,88825	0,6279808
CG4421-RA	GstD8	5,43715	3,55372	31,0605	1,70714	13,3928	20,8406	-0,875775	0,008637	0,6279808
CG4422-RA	Gdi	60,6	0,554203	99,4135	83,0333	85,5028	82,9372	-0,337171	0,20982	0,6279808
CG4423-RA	GstD6	0,713783	0,278641	3,8669	0,0238779	19,4196	29,4266	-0,218175	0,531092	0,6279808
CG4424-RA	CG4424	2,04495	2,51583	7,68356	14,6449	1,53316	2,26001	0,120235	0,668782	0,6279808
CG4426-RA	ast	2,74607	2,09181	4,51453	3,48462	3,03675	3,12876	-0,162566	0,557146	0,6279808
CG4427-RA	cbt	0,0258445	0,023541	71,1911	0,0273691	0,0370716	0,0279394	0,42456	0,072692	0,6279808
CG4427-RB	cbt	94,5165	104,203	9,64556	83,1267	51,8856	52,7536	0,444442	0,060433	0,6279808
CG4428-RA	Atg4a	18,7427	22,5121	0,0201985	0,0220261	33,7448	3,02324	0,053651	0,783087	0,6279808
CG4429-RA	eIF4H1	0,0341713	0,0311255	0	0	0,0500138	0,0376934	0,004159	0,984308	0,6279808
CG4429-RB	eIF4H1	0,032507	0,0296096	0	0	0,047385	0,0357122	0,004159	0,984308	0,6279808
CG4429-RC	eIF4H1	106,335	78,3712	0	0	80,8982	92,2222	0,004197	0,984158	0,6279808
CG4432-RA	PGRP-LC	0,0382457	0,0348368	0,0351126	0,0439418	0,0538459	0,0405815	-0,236258	0,253172	0,6279808
CG4432-RB	PGRP-LC	0,0365733	0,0333135	2,72616	0,0442963	3,72539	2,76277	-0,138918	0,495964	0,6279808
CG4432-RC	PGRP-LC	3,67155	3,36077	9,4366	0,046814	7,63217	8,71639	-0,265467	0,206668	0,6279808
CG4432-RD	PGRP-LC	9,98014	7,80723	3,63528	8,84756	0,105557	3,13088	-0,234024	0,258083	0,6279808
CG4432-RE	PGRP-LC	0,0996451	3,14647	40,6927	34,711	33,9835	32,346	-0,109898	0,637626	0,6279808
CG4433-RA	PIG-L	14,762	16,094	2,45179	2,50633	2,40842	0,0374417	0,238309	0,232215	0,6279808
CG4433-RB	PIG-L	0,0339605	0,0309336	5,51843	10,3299	3,73341	5,52748	0,239612	0,23169	0,6279808
CG4434-RA	bb8	0,0918231	0,127947	0,0881556	0,422311	0,0323428	0	-0,213637	0,44609	0,6279808
CG4435-RA	FucTB	2,37302	1,35923	10,8642	16,7912	1,52682	1,81423	0,305243	0,235208	0,6279808
CG4437-RA	PGRP-LF	0,0508406	0,0463091	0,110581	5,41719	12,2563	10,395	-0,596309	0,017828	0,6279808
CG4438-RA	CG4438	0,120576	18,6916	5,15397	4,11805	0,0385945	10,6372	0,076465	0,555158	0,6279808
CG4439-RA	S-Lap8	0,0819393	0,492755	0,125867	70,9618	0,0616224	0,0167972	0,073778	0,789351	0,6279808
CG4440-RA	CG4440	10,3244	0,123333	0,0812459	61,8682	62,4881	0,0576713	-0,250531	0,420879	0,6279808
CG4443-RA	Ubc7	39,9812	9,47586	45,4255	0,0232134	42,6895	5,97524	-0,25552	0,222281	0,6279808
CG4444-RA	px	14,4671	21,6123	14,2459	17,7298	0,0772385	0,970946	0,246984	0,373408	0,6279808
CG4444-RB	px	0,0097261	0,0088592	0,00933763	2,8362	8,01749	0,0582116	0,26593	0,334589	0,6279808
CG4444-RC	px	0,0506457	0,0461316	0,0486229	0,0570234	42,6317	8,43254	-0,022835	0,941576	0,6279808
CG4445-RA	Pgant3	28,5892	0,492091	40,7471	0,980459	45,2051	15,5009	0,440508	0,147841	0,6279808
CG4447-RA	mRRF1	7,11702	78,7941	5,89248	7,79745	0,0775742	0,0584646	0,069575	0,819348	0,6279808
CG4448-RA	wda	4,07283	3,89078	0,322669	6,42526	2,36596	29,7831	0,334232	0,159014	0,6279808
CG4449-RA	Rad60	4,07803	4,62973	4,44474	4,96542	19,3189	3,2368	0,30038	0,245151	0,6279808
CG4451-RA	Hs6st	13,0273	0	0	0,0465245	0	2,32461	0,228013	0,348159	0,6279808
CG4452-RA	CG4452	0,0418554	0,0381248	0,0401837	26,8972	0,062434	0	-0,146739	0,463571	0,6279808
CG4452-RB	CG4452	10,7251	13,9971	20,7263	14,2899	14,9644	0,047054	-0,143651	0,476016	0,6279808
CG4452-RC	CG4452	12,3123	14,355	12,908	0,135157	10,5747	14,2994	-0,145836	0,472199	0,6279808
CG4453-RA	Dsim\Nup153	1,74695	2,24782	4,46975	5,43019	1,98555	1,19234	0,147487	0,52781	0,6279808
CG4453-RB	Dsim\Nup153	3,60055	0,00780865	0,00823035	0,00871754	0,011808	0,00889919	0,147573	0,526331	0,6279808
CG4453-RC	Dsim\Nup153	10,1569	12,2204	9,79568	13,2164	6,23402	8,53543	0,147468	0,52793	0,6279808
CG4454-RA	borr	0,0529595	0,0482392	0,0508443	14,6705	7,63185	6,93216	-0,109468	0,751237	0,13772387
CG4454-RB	borr	5,27172	4,15683	20,5468	0,0599822	0,137622	0,0612321	-0,109468	0,751237	0,13772387
CG4455-RA	CG4455	24,0471	22,7071	31,9868	43,1835	14,654	23,7325	-0,0418	0,875874	0,6279808
CG4456-RA	CG4456	0,11295	0,0324864	0,0342408	0,0386797	0,0523919	0,0394857	-0,846525	0,002773	0,6279808
CG4457-RA	Srp19	47,2641	1449,8	9,25931	2062,18	4,80521	5,22002	0,002936	0,991741	0,6279808
CG4459-RA	CG4459	0,695902	21,4332	0,124467	0,182768	29,1881	0,186577	0,056161	0,85837	0,6279808
CG4460-RA	Hsp22	0,0356652	326,79	133,651	841,138	388,858	191,17	-0,5779	0,079126	0,6279808
CG4460-RB	Hsp22	245,41	1,77121	2,17801	5,96186	1,53726	1,93658	-0,562528	0,089624	0,6279808
CG4461-RA	CG4461	0	0,214696	0	0,773957	0,209666	13,9159	-0,159812	0,510353	0,6279808
CG4462-RA	CG4462	2,30011	1,57132	4,33284	2,22807	3,2791	0,0794139	-0,053122	0,869394	0,6279808
CG4463-RA	Hsp23	295,024	431,007	242,66	1438,29	644,942	251,187	-0,664877	0,044397	0,6279808
CG4464-RA	RpS19a	2284,95	40,5975	11,2259	13,616	8,50508	8,62049	-0,307695	0,224603	0,6279808
CG4464-RB	RpS19a	0,109457	11,9676	66,9943	55,6562	13,7956	12,5324	-0,307742	0,224544	0,6279808
CG4464-RC	RpS19a	0,0781345	18,0874	18,5507	26,1018	72,0202	75,6494	-0,307764	0,22446	0,6279808
CG4465-RA	CG4465	1,03784	1,40225	0,0645996	0,245622	2,1546	1,40624	-0,483026	0,094903	0,6279808
CG4466-RA	Hsp27	156,28	244,791	130,929	736,168	254,868	6,29791	-0,7744	0,017252	0,6279808
CG4467-RA	CG4467	14,989	0	0,0809143	14,8184	3,90042	2,47406	0,296693	0,312076	0,6279808
CG4467-RB	CG4467	0,00983467	14,6441	4,40103	0,0238779	2,9297	4,84323	0,289529	0,323203	0,6279808
CG4468-RA	Xport-A	1,21336	2,11568	1,63085	2,31442	0	2,15071	-0,089515	0,784776	0,6279808
CG4471-RA	Tsp42Ep	0,0741868	1,95966	0,0712238	0,0888602	41,0395	68,8997	-0,370071	0,222502	0,6279808
CG4472-RA	Idgf1	21,606	10,1316	47,3763	45,9032	42,5544	32,1516	-0,323167	0,343425	0,6279808
CG4475-RA	Idgf2	90,6687	77,4732	163,648	15,0909	196,889	163,855	-0,5668	0,050597	0,6279808
CG4476-RB	CG4476	0,21943	0,06246	0,157999	0,046451	0,10408	327,411	-0,687037	0,054014	0,6279808
CG4477-RB	CG4477	0	0	0	367,581	5,37049	32,8404	-0,041811	0,731452	0,6279808
CG4478-RA	ProtB	0	0	0	0	0	0,207812	NA	NA	0,6279808
CG4479-RA	ProtA	0	818,506	2,55229	0,0351137	1,51931	2,98191	0,015925	0,916254	0,6279808
CG4480-RA	CG4480	0	0,131221	0,138307	0	0	0	NA	NA	0,6279808
CG4481-RA	GluRIB	3,40347	3,59908	3,82793	3,49496	2,26761	2,0883	0,38702	0,06922	0,6279808
CG4482-RA	mol	0,0300527	0,345899	0,0324706	0,129892	0,41899	0,0904895	-0,573505	0,025539	0,6279808
CG4482-RB	mol	9,7744	0,0273741	0,0214628	14,9723	23,8605	19,3264	-0,557788	0,029426	0,6279808
CG4482-RC	mol	0,0223556	7,50148	0,0264467	5,39054	16,0054	2,99024	-0,572121	0,026103	0,6279808
CG4482-RD	mol	0,0275469	0,0203631	23,2402	0,0321498	2,24976	0,680403	-0,570076	0,026723	0,6279808
CG4482-RE	mol	12,3336	0,0250916	4,54814	0,0365148	0,0897583	26,6123	-0,556483	0,030044	0,6279808
CG4483-RA	CG4483	0	0	0	47,8628	0,383341	0	NA	NA	0,6279808
CG4484-RA	Slc45-1	37,1274	8,17873	36,8966	0,0330529	35,6105	0,0474189	-0,037078	0,886268	0,6279808
CG4485-RA	Cyp9b1	0,0389215	0,0354524	0,037367	6,46295	3,76895	64,7086	-0,216036	0,426304	0,6279808
CG4486-RA	Cyp9b2	17,9593	14,8566	31,5086	39,0565	0	0,016432	-1,254096	6,62E-05	0,6279808
CG4488-RA	Wee1	5,37118	5,96827	6,60106	8,43788	4,74674	4,96807	-0,04535	0,830579	0,6279808
CG4491-RA	noc	68,8985	85,5381	62,4039	64,7352	0,0375868	40,3414	0,439221	0,050311	0,6279808
CG4494-RA	smt3	192,531	14,5131	26,008	27,2497	28,2703	23,1335	-0,177148	0,492555	0,6279808
CG4495-RA	MICU1	12,5008	44,0973	11,4951	22,9893	3,88871	12,2504	-0,157611	0,48134	0,6279808
CG4495-RB	MICU1	3,4461	0,0151654	15,7833	24,4203	0,0438466	0,0174237	-0,151018	0,498253	0,6279808
CG4496-RA	CG4496	0,0302453	4,39402	16,09	9,75319	16,5839	19,0119	0,118264	0,652796	0,6279808
CG4497-RA	CG4497	3,44059	4,47434	2,1443	6,85286	0,432744	2,66592	-0,071001	0,793796	0,13772387
CG4500-RA	hll	40,378	0,264966	0,309758	0,0127314	0,189812	0,0129967	-0,098784	0,709369	0,6279808
CG4501-RA	bgm	12,7793	5,03788	0,0122307	0,450308	28,1625	0,216989	-0,248892	0,465959	0,6279808
CG4502-RA	CG4502	11,7615	12,531	3,90102	17,0198	7,50543	0,0176108	0,463897	0,031249	0,6279808
CG4502-RB	CG4502	2,90917	0,0306344	0,0290373	0,0170681	0,129399	2,76223	0,463897	0,031249	0,6279808
CG4509-RB	Cad86C	6,01697	1,61214	4,77725	26,3646	41,0861	55,8421	0,521921	0,068166	0,6279808
CG4510-RA	Surf6	21,8916	0,0547813	3,98959	3,79134	0,24756	4,48548	-0,020731	0,946664	0,6279808
CG4511-RA	CG4511	23,7231	8,0596	661,111	14,1828	196,988	159,554	0,270423	0,253701	0,6279808
CG4520-RA	CG4520	0	0	1,45231	0,0856269	0,0360824	0,0874112	NA	NA	0,6279808
CG4521-RA	mthl1	5,63263	8,47575	5,38667	8,65372	2,23546	0,235586	0,309595	0,310986	0,6279808
CG4523-RA	Pink1	3,72809	5,85009	7,42598	6,66415	129,13	0,0250099	-0,107144	0,606031	0,6279808
CG4523-RB	Pink1	0,022811	0,0207778	0,0218999	14,5255	0,0596388	0,0257767	-0,106703	0,607649	0,6279808
CG4523-RC	Pink1	0,0231872	0,0211205	0,0222611	2,60656	0,0641851	10,0326	-0,107144	0,606031	0,6279808
CG4523-RD	Pink1	0,0232361	0,0211651	0,0223081	2,24642	6,67574	150,85	-0,106703	0,607649	0,6279808
CG4523-RE	Pink1	0,023228	0,0211576	0,0223002	0,0239822	0,0324841	0,0600835	-0,107144	0,606031	0,6279808

gene_id	Symbol	W1_FPKM	W2_FPKM	W3_FPKM	MCM51_FPKM	MCM52_FPKM	MCM53_FPKM	FC	p-value	p-adj
CG4523-RF	Pink1	0,0232771	0,0212024	0,0223474	0,0243996	0,0330494	124,175	-0,107144	0,606031	0,6279808
CG4523-RG	Pink1	0,0239521	0,0218172	0,0229954	0,0244539	0,033123	0,0449474	-0,107144	0,606031	0,6279808
CG4523-RH	Pink1	10,958	11,96	12,9286	0,0244449	0,0331107	0,0483738	-0,107144	0,606031	0,6279808
CG4525-RA	Ttc26	1,33938	1,68923	8,21224	1161,89	0,260106	0,0116708	-0,321292	0,217006	0,6279808
CG4527-RA	Slik	0,00977646	1,54017	0,00990014	4,04186	0	0,0108116	-0,483097	0,048274	0,6279808
CG4527-RB	Slik	14,7548	16,2478	4,75152	16,3073	1,18724	5,02047	-0,045789	0,839452	0,6279808
CG4527-RC	Slik	5,28966	0,0545167	0,0130269	0,0105909	0,0135034	3,99358	-0,04622	0,837978	0,6279808
CG4527-RD	Slik	0,010312	0,00890508	0	6,63215	5,88502	0,0142494	-0,047183	0,835004	0,6279808
CG4527-RE	Slik	3,89269	14,5926	0,0914602	8,21636	6,31644	0,405928	-0,003585	0,98754	0,6279808
CG4527-RF	Slik	0,0135689	6,66517	0	0,0139586	0,0142607	0	-0,407059	0,081635	0,6279808
CG4528-RA	snf	29,3316	29,8028	50,275	0,154938	0,767911	45,6063	-0,088379	0,737978	0,6279808
CG4531-RA	aos	17,1728	17,0737	0,02548	36,2385	0,0381328	0,0287392	0,443835	0,067574	0,6279808
CG4532-RA	pod1	0,0121627	0,0110786	0,556463	2,43035	0,0163867	0,01235	0,216667	0,498217	0,6279808
CG4532-RB	pod1	2,7312	3,55035	0,0116769	0,78919	0,015939	0,0120126	0,216183	0,498952	0,6279808
CG4532-RC	pod1	0,0117287	0,0106834	1,79939	6,24127	9,44981	8,01652	0,216667	0,498217	0,6279808
CG4532-RD	pod1	10,0872	13,8801	0,0112603	20,1874	0,0168923	19,4513	0,216857	0,497812	0,6279808
CG4532-RE	pod1	0,0120694	0,0109936	10,2272	15,7845	1,8924	0,012731	0,216832	0,497886	0,6279808
CG4532-RF	pod1	0,0121381	0,0110562	0,0115873	0,885727	0,0162732	1,88479	0,20031	0,531565	0,6279808
CG4533-RA	l(2)efl	90,9712	0,911916	0,775132	1,50224	9,36293	8,34131	-0,143294	0,522796	0,6279808
CG4533-RB	l(2)efl	0,114384	0,0312549	0,0329427	4,19452	0,050239	83,1038	-0,160492	0,477784	0,6279808
CG4535-RA	Fkbp59	24,201	19,0134	13,705	17,5761	32,153	31,9029	-0,367532	0,152059	0,6279808
CG4536-RA	iav	0,411505	0,571165	0,592603	0,715851	4,92553	0,485667	-0,068488	0,814678	0,6279808
CG4537-RB	CG4537	7,61668	7,64036	8,05297	0,190703	15,4668	3,41457	-0,234677	0,375521	0,6279808
CG4538-RA	Dpse\GA18242	0,0184415	0,0167978	40,0425	22,6292	2,10941	27,2461	0,019147	0,932899	0,6279808
CG4538-RB	Dpse\GA18242	33,9139	47,4622	4,81508	4,35572	17,4262	2,74033	0,019045	0,933245	0,6279808
CG4539-RA	Bka	14,2089	9,84581	25,1069	29,5466	15,9389	13,324	0,021868	0,944265	0,6279808
CG4542-RA	xit	0,430626	0,487654	1,16206	6,42803	0,842527	0,707993	0,299048	0,232709	0,6279808
CG4542-RB	xit	3,64142	3,88873	4,54076	0,00983969	1,81543	3,04341	0,214146	0,381539	0,6279808
CG4545-RA	SerT	2,86744	0	2,86264	31,4816	3,36308	26,1193	-0,18103	0,447531	0,6279808
CG4546-RA	CG4546	0,0378513	11,7276	0,0175498	16,1836	3,57379	11,3424	0,07433	0,753936	0,6279808
CG4548-RA	XNP	4,08745	0	4,53819	0	0	0	0,013461	0,956862	0,6279808
CG4548-RB	XNP	4,97879	3,05829	6,89084	0,0883244	0,795237	0,599338	0,02452	0,921364	0,6279808
CG4550-RA	ninaE	0,331	0,282654	27,8434	0,223781	0,348773	0,361763	0,087206	0,807024	0,6279808
CG4551-RA	Dyrk2	14,8069	0,457427	5,25008	4,13309	0,168232	6,00098	0,498532	0,095041	0,6279808
CG4551-RB	Dyrk2	0,0143342	0,708422	12,9093	0,0208089	0,647467	0,0150935	0,498443	0,095129	0,6279808
CG4551-RC	Dyrk2	23,6522	0,77378	0,0137736	3,0231	0,0454313	0,0153309	0,498602	0,094991	0,6279808
CG4551-RD	Dyrk2	0,0143466	0,455598	0,0139831	286,606	5,19171	5,81901	0,508743	0,088583	0,6279808
CG4551-RE	Dyrk2	8,0398	30,9417	4,91592	41,3916	4,78198	3,69442	0,498505	0,095039	0,6279808
CG4552-RA	CG4552	10,7922	11,3621	0,0906669	0,11969	7,99958	6,78094	0,058888	0,769635	0,6279808
CG4553-RA	CG4553	4,43751	5,05438	8,27317	14,3841	11,6259	14,5549	-0,046644	0,884063	0,6279808
CG4554-RA	CG4554	1,96171	0,958018	16,8894	2,24843	32,7552	0,956468	0,033791	0,912153	0,6279808
CG4557-RA	CG4557	5,01997	0,215722	3,13984	3,82704	9,87886	3,39756	-0,063358	0,788618	0,6279808
CG4558-RA	Dsim\GD24760	3,97066	4,5346	6,77818	0,174278	3,22313	2,28918	-0,04349	0,831783	0,6279808
CG4558-RB	Dsim\GD24760	4,62188	4,15578	4,21527	7,50952	0,0133279	0,0100447	-0,063261	0,75686	0,6279808
CG4559-RA	Idgf3	0,0395506	0,0360254	0,0379709	49,9021	0,0586589	0,0442089	-0,446999	0,101803	0,6279808
CG4559-RB	Idgf3	20,369	19,8828	43,054	23,5004	54,2653	47,1162	-0,463498	0,089882	0,6279808
CG4559-RC	Idgf3	18,6079	13,64	26,391	139,089	12,8985	16,6032	-0,463498	0,089882	0,6279808
CG4560-RA	Arpc3A	26,6379	9,15339	14,2563	3,62353	0,975097	0,897319	-0,097964	0,692296	0,6279808
CG4560-RB	Arpc3A	0,180206	4,55279	0,0455505	12,5208	4,71943	0,718435	-0,193607	0,432984	0,6279808
CG4561-RA	TyrRS	37,6962	39,9255	55,6778	0,0443261	0,06004	0,0452498	0,315591	0,14683	0,6279808
CG4562-RA	Dpse\GA18260	21,419	0,0144136	0,31778	4,57297	5,13172	2,75963	-0,309362	0,208323	0,6279808
CG4562-RB	Dpse\GA18260	0,015824	9,26418	11,1578	11,9587	5,97169	6,94463	-0,143622	0,541065	0,6279808
CG4563-RA	CG4563	7,56223	0,83545	0,181248	0	0	10,6173	-0,276786	0,376788	0,6279808
CG4565-RB	CG4565	0,0813073	3,35003	0,0780599	352,186	5,2969	732,48	0,348778	0,25038	0,13772387
CG4567-RA	mEFG1	13,4288	11,7917	16,0912	42,0305	0,0231188	13,9362	-0,228246	0,258895	0,6279808
CG4568-RA	fzo	0,261659	0,150529	79,9637	0,434138	0,221428	55,5289	-0,40555	0,257173	0,6279808
CG4569-RA	Prosalpha4T2	0	0	0	0	0,0293634	0,230431	0,044335	0,715924	0,6279808
CG4570-RA	CG4570	2,33556	76,5895	4,27837	1,61865	9,63978	9,94743	-0,947095	0,000315	0,6279808
CG4572-RA	Dpse\GA18267	0,0360741	0,0344382	0,036298	8,83793	0,0530458	0,0420793	-0,169265	0,497753	0,6279808
CG4572-RB	Dpse\GA18267	0,037808	8,20081	11,7733	1,76192	0,0558333	11,2569	-0,171226	0,492687	0,6279808
CG4572-RC	Dpse\GA18267	9,13911	2,9453	2,92439	0,0412204	11,1642	1,94933	-0,169859	0,495613	0,6279808
CG4573-RA	GluRS-m	0,0403969	0,0367963	0,0387834	62,7096	41,6788	42,4783	0,25839	0,257927	0,6279808
CG4574-RA	Plc21C	3,42648	0,0105209	2,30219	3,24877	5,88164	3,05996	-0,210284	0,296475	0,6279808
CG4574-RB	Plc21C	0,0115504	0,0105671	0,0110891	0,0436797	3,91472	0,012073	-0,217751	0,280341	0,6279808
CG4574-RC	Plc21C	0,0116011	0,0106006	0,0111377	4,22805	4,81587	0,0121274	-0,210043	0,296595	0,6279808
CG4574-RD	Plc21C	0,0116379	3,54293	1,34076	73,1904	7,56247	0,938555	-0,217506	0,280432	0,6279808
CG4574-RF	Plc21C	4,55959	0,0363079	4,07969	30,0079	5,45124	2,70378	-0,208622	0,299926	0,6279808
CG4575-RA	CR4575	0,231845	0,633542	0	6,46529	0,0167591	5,55332	-0,041811	0,731452	0,6279808
CG4576-RA	CG4576	30,3482	0	3,7248	0,13921	0,10469	1,39015	-0,003846	0,983981	0,6279808
CG4577-RA	CG4577	11,7318	12,0485	23,5296	26,8439	11,556	11,789	-0,017617	0,939804	0,6279808
CG4579-RA	Nup154	3,11949	5,65061	6,20735	12,5376	9,49552	1,35877	0,392904	0,148709	0,6279808
CG4579-RB	Nup154	1,33587	0	0,0146573	1,66108	0,0292001	13,1901	0,392904	0,148709	0,6279808
CG4580-RA	Nepl8	0,053287	0	0,0511587	0,159051	0	0	-0,066315	0,709311	0,6279808
CG4581-RA	Thiolase	34,7177	0,0371139	59,405	34,1013	2,10155	0,04568	-0,0413	0,887363	0,6279808
CG4582-RA	CG4582	0,763001	0,0166093	4,72389	1,58412	0,0546705	8,23126	0,197723	0,578325	0,6279808
CG4583-RB	Ire1	6,48953	9,17081	3,10879	6,59328	0,0426319	0,0321299	-0,081946	0,746642	0,6279808
CG4583-RC	Ire1	2,13552	0,0268729	16,5207	0,0315193	6,87171	7,74058	-0,103293	0,682572	0,6279808
CG4584-RA	dUTPase	15,0293	0,866884	45,4922	0,239147	0,242158	0,30221	-0,374858	0,238456	0,6279808
CG4584-RB	dUTPase	6,99781	3,26798	17,8732	42,6133	37,0294	1,68733	-0,377346	0,235557	0,6279808
CG4585-RA	Cpes	21,7902	19,2576	28,7534	25,8227	20,4473	23,2168	-0,0898	0,679627	0,6279808
CG4586-RA	CG4586	3,85269	11,2605	0,516716	0,0124453	0,0444582	0,0335064	-0,805779	0,004147	0,13772387
CG4587-RB	CG4587	0,424905	0	0,10752	0,230315	0	0	-0,360404	0,196167	0,6279808
CG4587-RC	CG4587	1,51778	3,23502	7,41858	8,54063	5,06554	5,65394	-0,360404	0,196167	0,6279808
CG4589-RA	Letm1	0,0180453	0,0164369	0,0173246	0,0187591	0,0254093	0,01915	-0,16387	0,397082	0,6279808
CG4589-RB	Letm1	0,0181793	0,016559	11,1788	13,6543	0,0256061	0,0192983	-0,163248	0,399015	0,6279808
CG4589-RC	Letm1	15,2137	18,2681	9,84303	12,5453	16,2887	15,2023	-0,162609	0,400317	0,6279808
CG4590-RA	Inx2	40,7012	36,6663	1949,89	102,484	9,18317	8,78418	-0,075608	0,78229	0,6279808
CG4590-RB	Inx2	114,152	131,856	0,0627051	166,412	2,58267	3,07279	-0,066101	0,809085	0,6279808
CG4591-RA	Tsp86D	23,8966	25,0144	22,4687	31,0444	118,546	22,4726	-0,150366	0,460709	0,6279808
CG4592-RA	CG4592	7,52679	6,27954	8,40921	4,29268	14,4756	8,02918	0,085188	0,78423	0,6279808
CG4593-RA	CG4593	11,9056	8,89242	3,76268	11,889	0,190089	0,143262	-0,117009	0,674751	0,6279808
CG4594-RA	CG4594	6,80289	7,88651	0,613133	0	0,64501	7,71464	0,328937	0,281653	0,6279808
CG4596-RA	CG4596	8,08464	9,7338	1,67706	30,0536	0	0,0704696	0,277941	0,295953	0,6279808
CG4597-RA	CG4597	1,10709	0,743043	0,72723	1,35572	1,1596	0,596408	0,158113	0,658053	0,6279808
CG4598-RA	CG4598	39,9235	0,666847	10,0624	4,24394	6,77267	0	-0,447633	0,126111	0,6279808
CG4599-RA	Tpr2	59,7473	52,8611	80,8731	72,8039	0,941087	0,877996	-0,031435	0,876625	0,13772387
CG4599-RB	Tpr2	0,0321589	16,5503	0,0308745	0,0345794	65,0266	0,0132345	0,002906	0,988574	0,13772387
CG4600-RA	yip2	56,4497	15,612	18,4646	18,3087	0,0788071	15,3965	-0,245053	0,405263	0,6279808
CG4602-RA	Srp54	24,6162	3,82226	0,757258	0,0226547	3,79989	24,9753	0,137663	0,541644	0,13772387
CG4603-RA	CG4603	17,8748	14,779	26,5983	542,604	3,12956	2709,14	-0,17542	0,499886	0,6279808
CG4604-RA	GLaz	32,4515	48,6122	31,7559	38,0873	21,9343	24,741	0,457302	0,070184	0,6279808
CG4605-RA	Acp32CD	0,19075	11,5399	0,439516	7,23196	6,24626	6,45334	-0,004477	0,989331	0,6279808
CG4606-RA	alpha-Man-IIb	8,83837	1,13534	150,64	0,944038	83,8465	0,0210629	0,288377	0,29636	0,6279808
CG4607-RA	CG4607	0,310986	0,283267	19,2773	0,37054	0,0599593	0,045189	-0,296837	0,401556	0,6279808

gene_id	Symbol	W1_FPKM	W2_FPKM	W3_FPKM	MCM51_FPKM	MCM52_FPKM	MCM53_FPKM	FC	p-value	p-adj
CG4607-RB	CG4607	0,847683	0,504853	43,4928	0,892145	55,8845	53,2655	-0,264899	0,453652	0,6279808
CG4608-RA	bnl	3,62814	3,49817	3,92639	3,63212	0,042693	0,0321761	0,650814	0,016816	0,13772387
CG4608-RC	bnl	4,66555	9,02442	4,88301	4,86673	1,72607	0,0321761	0,684117	0,012247	0,6279808
CG4609-RA	fax	242,53	206,858	0,290063	30,7857	19,354	21,1359	0,606795	0,01256	0,6279808
CG4609-RB	fax	0,0241623	0,0220087	0,0990165	0,134057	0,181581	0,13685	0,602125	0,013776	0,6279808
CG4609-RC	fax	312,884	312,245	135,415	138,589	137,522	150,325	0,606779	0,012561	0,6279808
CG4609-RD	fax	0,0191265	86,3598	0,0649522	0,0796254	0,107853	0,0812846	0,602107	0,013777	0,6279808
CG4610-RA	CG4610	5,50632	17,2015	5913,67	19,3283	0,0463155	2,07452	0,288467	0,160493	0,6279808
CG4611-RA	CG4611	5,37861	4,93948	7,99326	9,58983	645,521	179,682	0,289708	0,246259	0,6279808
CG4612-RA	CG4612	16,8857	0,0123595	20,1517	26,4397	9,86892	0	0,111186	0,665506	0,6279808
CG4613-RA	CG4613	0,748432	0,559988	0,923837	1,6418	0,279107	0,399984	-0,007799	0,982597	0,6279808
CG4615-RA	CG4615	4,29911	4,38828	0,0732807	6,42053	0,817531	1,04778	0,1933	0,438053	0,6279808
CG4616-RA	FLASH	1,00417	10,3773	2,35686	40,9923	24,3404	0,9306	0,166487	0,566969	0,6279808
CG4616-RB	FLASH	0,0193393	0,711435	0,0505606	8,79444	1,5019	0,0205859	0,166487	0,566969	0,6279808
CG4617-RA	CG4617	5,20938	6,43847	0,0773932	6,83088	0,460451	0,98952	-0,047325	0,829097	0,6279808
CG4618-RA	CHMP2B	13,2968	12,1631	21,8896	14,8549	17,8293	18,0134	-0,086771	0,756608	0,6279808
CG4619-RA	CG4619	12,7555	6,64737	39,2115	17,6497	0,0445025	211,87	-0,083886	0,797487	0,6279808
CG4620-RA	unk	21,098	23,955	15,567	19,8143	12,157	5,90018	0,352012	0,158364	0,6279808
CG4620-RB	unk	0,0246951	0,022494	0,0237088	0,02608	0,0353255	5,42212	-0,067328	0,77928	0,6279808
CG4621-RA	YL-1	11,0847	1,7293	17,9357	1,98286	9,06923	13,3462	0,048599	0,848127	0,13772387
CG4622-RA	CG4622	0,034887	17,5729	14,5027	0,0711942	0,0527636	0,0397658	0,191574	0,352422	0,6279808
CG4622-RB	CG4622	0,0251526	0,0229107	0,024148	34,401	0,0368117	0,0277435	-0,759296	0,005371	0,6279808
CG4623-RA	Gdap1	9,72313	14,3777	18,8682	3,84202	16,8851	14,7168	0,072801	0,784475	0,6279808
CG4624-RA	VhaAC39-2	0,241153	0,146439	0,0311117	0	13,5212	13,2962	0,0137	0,960077	0,6279808
CG4625-RA	Dhap-at	5,46439	11,1247	12,1077	3,3648	1,4681	1,5321	0,180187	0,424219	0,6279808
CG4626-RA	fz4	4,4492	3,87171	4,66241	5,349	14,9857	1,31789	0,045734	0,844154	0,6279808
CG4626-RB	fz4	0,864658	1,49977	0,0176622	1,7906	1,85399	0,0175327	0,04533	0,846074	0,6279808
CG4627-RA	CG4627	14,1591	0,0167791	22,2599	0,020855	7,4895	28,0576	-0,466066	0,13983	0,6279808
CG4629-RA	CG4629	0,0213653	0,019461	11,2515	41,0621	9,67439	19,2813	0,006245	0,976126	0,6279808
CG4629-RB	CG4629	0,0210388	0,0191636	11,9757	8,10011	16,2368	0,122184	0,006747	0,974191	0,6279808
CG4629-RC	CG4629	5,07335	6,13532	0,0354198	10,9016	0,16212	0,0228518	0,007271	0,972169	0,6279808
CG4630-RA	CG4630	0,956389	0,0322427	0,0306063	18,9492	12,8878	1,71059	-0,818653	0,007196	0,6279808
CG4631-RA	CG4631	0,0806459	0,0612149	0,116137	0,199482	0	0	0,042735	0,869411	0,6279808
CG4633-RA	AlaRS-m	2,68152	0,0186451	3,66511	24,4256	2,32062	3,08105	-0,178474	0,449592	0,6279808
CG4634-RA	Nurf-38	56,7804	0,0533725	3,78386	2,249	0,673169	0,807408	-0,57473	0,062496	0,6279808
CG4634-RB	Nurf-38	0,0585951	17,1838	3,56641	3,42842	1,33642	1,61246	-0,574904	0,062422	0,6279808
CG4636-RA	SCAR	23,0782	28,0608	22,5103	32,4018	20,7455	20,2391	0,033068	0,882973	0,6279808
CG4637-RA	hh	0,0217665	0,0198264	0,00980981	0,890751	61,6328	0,00955258	0,243143	0,372446	0,6279808
CG4637-RB	hh	4,9343	6,59886	24,1891	7,23515	0,45541	0,0106473	0,267505	0,335883	0,6279808
CG4641-RA	nwk	24,6633	5,90782	0,0473526	8,5778	197,597	4,77366	0,337549	0,111893	0,6279808
CG4643-RA	Fsn	19,4508	20,8465	16,9628	20,724	17,0554	21,4285	-0,06426	0,763449	0,6279808
CG4643-RB	Fsn	0,0492369	0,0448484	0,0472703	0,057824	0,078323	0,0590289	-0,065012	0,760911	0,6279808
CG4644-RA	mtRNApol	5,45108	0,0916545	0	0,0693129	1,67271	0	0,015184	0,939255	0,6279808
CG4645-RA	CG4645	14,9197	28,4972	22,259	32,2713	5,51906	16,9784	-0,266647	0,21579	0,6279808
CG4646-RA	CG4646	18,105	1,1362	9,54949	0,0596024	0,0807318	30,367	-0,421048	0,15657	0,6279808
CG4647-RA	mRpL49	50,9519	54,5245	63,2488	14,7134	3,15786	97,4795	-0,553012	0,046349	0,6279808
CG4649-RA	Sodh-2	19,6277	0,691799	46,2135	2,542	16,9533	0,0829895	-0,57906	0,042603	0,6279808
CG4650-RA	CG4650	1,98511	56,0726	1,02098	0	0	0	-1,429276	3,35E-05	0,6279808
CG4651-RA	RpL13	0,0889709	4,81683	8,54537	0,111018	3,52494	3,42723	-0,324554	0,235692	0,6279808
CG4651-RB	RpL13	1408,7	0,0194275	3,42534	2325,29	3,36722	3,22674	-0,324568	0,235678	0,6279808
CG4653-RA	CG4653	16,6701	7,15944	32,5062	25,647	145,112	99,2901	-1,240504	0,000503	0,6279808
CG4654-RA	Dp	0,026339	0,0239914	6,59992	10,8407	4,35019	0,0285078	0,083433	0,729984	0,6279808
CG4654-RB	Dp	28,5405	30,5112	22,5364	27,35	12,3099	17,2664	0,290889	0,227508	0,6279808
CG4655-RA	Cad86C	1,44213	0,102416	0,107947	2,68984	5,97924	8,15693	0,118751	0,732172	0,6279808
CG4656-RA	Rassf	5,86081	5,95838	20,9121	7,81618	0,0165233	4,11126	0,263826	0,325007	0,6279808
CG4658-RA	CG4658	0,0289849	0,0272748	0,0287478	9,96123	0,0433778	0,0326921	-0,176818	0,402316	0,6279808
CG4658-RB	CG4658	3,77291	27,3516	20,407	16,8693	22,1586	19,3989	-0,176818	0,402316	0,6279808
CG4658-RC	CG4658	16,3473	0,0374604	5,42897	0,0452081	7,42865	5,8795	-0,176818	0,402316	0,6279808
CG4659-RA	Srp54k	40,4505	38,4446	51,9042	9,95471	27,0163	36,5335	0,094687	0,653184	0,6279808
CG4660-RA	CG4660	2,84225	2,63642	3,47976	3,03644	2,98021	2,39253	0,111434	0,677117	0,6279808
CG4661-RA	CG4661	0	0	4,12293	34,8884	3,91434	0,00541605	0,044335	0,715924	0,6279808
CG4662-RA	CG4662	0,0390135	0,0268729	6,20647	2,58835	0,82455	1,52542	-0,017861	0,934024	0,6279808
CG4662-RB	CG4662	19,775	0,026837	0,0283242	0,0314741	2,56951	2,57624	0,280849	0,210399	0,6279808
CG4663-RA	Pex13	17,5357	4,18125	7,7589	23,4256	3,06271	19,5749	-0,092452	0,662805	0,6279808
CG4665-RA	Dhpr	0,0547589	4,0104	4,50235	4,24751	3,63124	2,7156	-0,242875	0,38855	0,6279808
CG4665-RB	Dhpr	4,33233	4,86187	5,63816	0,0768397	6,3527	6,69343	-0,228818	0,423184	0,6279808
CG4665-RC	Dhpr	21,2387	9,62124	9,0589	26,7477	0,0391018	7,2054	-0,235539	0,409174	0,6279808
CG4666-RA	CG4666	89,6744	7,00837	8,95325	92,4432	3,02224	11,5404	0,526704	0,040942	0,6279808
CG4669-RA	CG4669	0	0,0428272	0,04514	0,0835502	0	0,0529499	-0,119955	0,564466	0,6279808
CG4670-RA	Qsox1	21,2765	26,3189	1,75171	1,27399	11,9244	15,6216	0,434767	0,055899	0,6279808
CG4672-RA	TMS1	42,4177	50,6116	49,2076	34,7319	30,829	32,0233	-0,108933	0,559244	0,6279808
CG4673-RA	Npl4	0,0282295	0,0160511	3,04198	0,0300666	7,68299	0,0338947	-0,067336	0,77233	0,6279808
CG4673-RB	Npl4	7,73511	0,0531057	0,016918	12,4409	0,213253	13,8257	-0,066645	0,774485	0,6279808
CG4674-RA	Leash	4,67812	4,39373	1,51659	0	0	7,51353	0,137032	0,547816	0,6279808
CG4676-RA	CG4676	2,55651	3,25413	2,80053	3,69173	3,05046	3,1618	-0,098158	0,717089	0,6279808
CG4677-RA	lmd	2,44868	2,54633	3,87441	1,35408	11,5887	0,776728	0,177169	0,60846	0,6279808
CG4677-RB	lmd	0,0222803	0,0202944	0,0213904	3,11082	3,13678	4,17585	0,175294	0,61252	0,6279808
CG4678-RD	CG4678	1,76017	0,0330574	1,49823	0,0394202	7,65253	1,23001	0,125847	0,660854	0,6279808
CG4678-RE	CG4678	0,0357618	0,0325743	0,0343334	0,0387936	2,54822	0,0396019	0,116491	0,683413	0,6279808
CG4678-RF	CG4678	8,39478	12,1621	7,23033	13,3626	0,0525461	5,40168	-0,022006	0,935242	0,6279808
CG4678-RG	CG4678	0,0340656	0,0465439	1,74972	3,31763	3,6053	1,01718	-0,033944	0,899718	0,6279808
CG4679-RA	CG4679	20,2757	22,664	26,503	25,4203	13,8435	13,6454	0,315499	0,094635	0,6279808
CG4680-RA	Gagr	2,40942	0,039579	4,33028	3,06932	0,0442378	3,64164	-0,419407	0,195055	0,6279808
CG4681-RA	CG4681	1,06428	1,6707	0,869593	0,067338	0,0416569	25,7515	0,171976	0,602208	0,13772387
CG4683-RA	Tengl4	0	51,9851	0,161335	5,44422	2,82754	3,34359	-0,013096	0,914398	0,6279808
CG4684-RA	nwk	5,55934	6,34868	7,18278	9,88773	14,8414	2,37573	-0,745365	0,002984	0,6279808
CG4685-RA	Ssadh	15,0082	11,5557	5,99384	9,90673	0,0407255	3,46638	0,067964	0,796911	0,6279808
CG4685-RB	Ssadh	3,63448	6,06368	8,30306	4,32874	5,88574	6,6575	0,067964	0,796911	0,6279808
CG4685-RC	Ssadh	0,036312	0,0330755	0,610439	0,0394438	0	0,736125	0,067964	0,796911	0,6279808
CG4685-RD	Ssadh	0,0365329	0,0332767	0,104108	3,18886	1,43817	0,195408	0,067964	0,796911	0,6279808
CG4686-RA	Dpse\GA18356	20,4358	2,70488	20,8186	5,48618	3,84286	2,1262	-0,205049	0,404881	0,6279808
CG4688-RA	GstE14	1,01004	0,801299	26,3204	22,3299	2,4497	1,74625	-0,587716	0,082172	0,6279808
CG4690-RA	Tsp5D	0,0371491	41,652	41,662	0,0404362	0,211713	28,6316	-0,313324	0,177333	0,6279808
CG4690-RB	Tsp5D	8,20939	0,0338379	0,0356653	10,975	1,62141	0,0412788	-0,283382	0,224982	0,6279808
CG4691-RA	CG4691	0,0849304	0,0773605	0	0	0	0	0,044335	0,715924	0,13772387
CG4692-RA	Dpse\GA18359	0,128136	0,844367	4,90502	0,382894	24,8929	25,0415	-0,415515	0,20208	0,6279808
CG4692-RB	Dpse\GA18359	363,925	1,95552	22,9503	21,2269	44,9052	41,4142	-0,415515	0,20208	0,6279808
CG4694-RA	her	5,99879	5,95136	7,86235	6,77368	0,0187206	5,06609	0,297492	0,233755	0,6279808
CG4696-RA	Mp20	751,191	560,8	8,53704	8,30895	5,69583	0,0455254	-0,539893	0,092967	0,6279808
CG4696-RB	Mp20	0,0951186	0,0866406	56,9188	44,4457	5,06279	0,0272269	-0,565322	0,078791	0,13772387
CG4697-RA	CSN1a	0	0	0	0	0	0	NA	NA	0,6279808
CG4698-RA	Wnt4	28,6137	34,9111	26,9745	35,5166	12,1792	17,0257	0,36161	0,182212	0,6279808
CG4699-RA	nsl1	5,24148	0,0101792	1408,11	0,0114325	0,0225351	7,65348	0,134996	0,549269	0,6279808
CG4699-RB	nsl1	7,40656	24,2637	0,0296618	32,2356	27,5737	0,0421321	0,134667	0,5501	0,6279808

gene_id	Symbol	W1_FPKM	W2_FPKM	W3_FPKM	MCM51_FPKM	MCM52_FPKM	MCM53_FPKM	FC	p-value	p-adj
CG4699-RC	nsl1	0,0111753	0,164144	0,0411748	18,4311	1,77576	1,17717	0,133793	0,552387	0,6279808
CG4700-RA	Sema2a	12,2886	12,4244	9,44189	0,0264995	4,56577	0,0216807	0,230754	0,423263	0,6279808
CG4700-RB	Sema2a	15,0836	11,8408	14,4628	0,0179614	5,70886	0,723569	0,229798	0,425087	0,6279808
CG4700-RC	Sema2a	0,0195916	0,0178454	0,0188091	152,508	9,03292	0,0205859	0,235625	0,411968	0,6279808
CG4700-RD	Sema2a	30,5553	44,3412	22,0853	0,0182146	5,06382	0,937171	0,230437	0,423296	0,6279808
CG4701-RA	CG4701	0	0	0	0	0	0	NA	NA	0,6279808
CG4702-RA	CG4702	121,955	2,84753	169,262	0	0	103,364	0,20343	0,475221	0,6279808
CG4703-RA	Arc42	10,9189	13,0831	18,8511	9,80025	15,2584	16,4324	-0,142894	0,595706	0,6279808
CG4704-RA	CG4704	0	0,402137	0,0698643	0	0	0	0,044335	0,715924	0,6279808
CG4705-RA	Wdr59	0,0206122	0,018775	0,0197889	0,0215577	1,73295	45,4865	0,008986	0,971347	0,6279808
CG4705-RB	Wdr59	3,27684	4,13276	3,24679	4,81962	13,1461	13,5053	0,008986	0,971347	0,6279808
CG4706-RA	mAcon2	0,0385807	13,6252	0	30,9059	55,6725	42,0943	0,01562	0,897983	0,6279808
CG4707-RA	CG4707	4,4942	5,22006	5,64676	18,9936	17,7912	3,67798	0,214942	0,356269	0,6279808
CG4709-RA	CG4709	5,19168	0,0264014	0,0278272	0,0309275	0,0418915	0,031572	0,067075	0,81005	0,6279808
CG4710-RA	Pino	33,2442	39,3696	40,9611	14,362	0,0303211	0,0224851	0,318858	0,133567	0,6279808
CG4710-RB	Pino	19,9564	23,8884	10,9364	0,0367904	0,0298346	4,46732	0,36858	0,087267	0,6279808
CG4711-RA	squ	0,0569129	0,0518402	8,19597	0,0687431	0,0931129	0,0701755	0,2201	0,480264	0,6279808
CG4712-RA	CG4712	0,0785993	0	0,135828	0,0324028	0,0879391	0,297812	-0,201866	0,447575	0,6279808
CG4712-RB	CG4712	0,030273	12,3447	0,0290639	0	0,0438897	0,101682	-0,201866	0,447575	0,6279808
CG4713-RA	l(2)gd1	6,25174	0,0451624	0,0476013	0,0556743	0,0754112	0,0568344	0,303727	0,160129	0,6279808
CG4714-RA	CG4714	0	0	0,0806058	0,0457895	0	0,0467436	0,011369	0,946739	0,6279808
CG4715-RA	Iris	0,0368933	0,033605	0,020512	0,0223853	0,0498329	0,369406	0,553949	0,080253	0,6279808
CG4716-RA	Dsim\GD10957	0	0	0,108686	0	0,395155	0,199021	-0,040143	0,812688	0,6279808
CG4716-RB	Dsim\GD10957	0	0	0,234757	9,32778	0,134917	0,033078	-0,040143	0,812688	0,6279808
CG4717-RA	kni	9,99853	8,68879	9,87302	0,828819	14,6053	0	0,175061	0,460573	0,6279808
CG4719-RA	Tnks	11,0909	0,0257134	2,10976	8,15938	4,25928	5,46446	0,027932	0,908432	0,6279808
CG4720-RB	Ask1	12,7347	18,1778	0,0123884	16,925	7,11695	8,31753	0,261915	0,334923	0,6279808
CG4720-RC	Ask1	0,0129038	0,0117536	7,30719	0,0132538	0,0179524	0,01353	0,263423	0,33185	0,6279808
CG4721-RA	Nepl15	30,4092	0,0108431	5,62098	0,01043	1,61393	1,21636	-0,623846	0,022616	0,6279808
CG4722-RA	bib	3,13217	3,2912	4,5794	6,76475	2,06876	2,88688	-0,108167	0,710199	0,6279808
CG4723-RA	Nepl14	4,79442	47,0548	0	0,00935759	0	0	-0,232169	0,364139	0,6279808
CG4725-RA	Nepl13	3,77952	0,00930719	0	0,0358785	0,20926	0,0979088	-0,983077	0,005468	0,6279808
CG4726-RA	MFS3	57,3886	13,1883	56,4265	13,7728	2,39248	31,8798	0,512973	0,064668	0,6279808
CG4729-RA	Agpat3	24,174	26,765	25,3693	25,5932	24,689	28,6606	0,01422	0,940283	0,6279808
CG4729-RB	Agpat3	0,0407959	0,0371598	0,0391665	0,0448084	0,0606933	0,0457421	0,010443	0,956124	0,6279808
CG4729-RC	Agpat3	0,0406954	0,0370682	0,03907	0,0446868	0,0605286	0,045618	0,012335	0,948204	0,6279808
CG4729-RD	Agpat3	19,5334	21,7268	31,1327	34,2567	18,4072	19,3988	0,134726	0,469218	0,6279808
CG4730-RA	CG4730	0,826713	0,773945	2,02833	72,7017	114,469	93,3526	0,009569	0,977694	0,6279808
CG4733-RA	CG4733	7,21828	20,4044	3,76002	20,7811	21,8654	20,0531	-0,068084	0,775341	0,6279808
CG4734-RA	CG4734	12,1891	9,01904	0	0	0,173555	0	-0,019945	0,950196	0,6279808
CG4735-RA	shu	0,644738	0,166907	3,09196	0,626468	0,767351	4,0515	0,079443	0,817209	0,6279808
CG4738-RA	Nup160	3,52092	2,84149	16,1579	3,87267	2,23026	4,20257	0,09543	0,732765	0,6279808
CG4739-RA	Ugt302E1	2,21519	1,55655	4,41546	0	0	0	-0,569079	0,079522	0,6279808
CG4740-RA	AttC	0,489651	0,0306188	0,0322724	0,0362736	0,0491328	11,5549	0,535909	0,085614	0,6279808
CG4741-RA	CG4741	3,10103	0,0617297	0,818769	458,338	2,97479	16,8909	-0,138441	0,671018	0,6279808
CG4742-RA	mRpL22	40,9338	22,4344	3191,59	5,48143	1,10301	1,49057	-0,374169	0,178458	0,6279808
CG4743-RA	CG4743	14,4368	24,5727	28,3151	33,7016	15,0834	18,6012	0,489591	0,062418	0,6279808
CG4744-RA	CG4744	0,0140289	6,29857	3,36611	7,95884	10,4995	0,0458982	0,067475	0,590454	0,6279808
CG4746-RA	mab-21	14,8903	21,478	14,3316	29,8394	10,1092	11,8159	0,067117	0,749704	0,6279808
CG4747-RA	Ndf	31,267	24,4948	39,5766	54,9173	24,3295	28,2988	-0,203585	0,406773	0,6279808
CG4749-RA	l(2)10685	3,15714	3,30246	4,67367	4,02393	5,25538	3,19852	0,098418	0,6853	0,6279808
CG4750-RA	loopin-1	0,0317113	0,0288849	0,0304448	0,22074	0	0	-0,202582	0,41303	0,6279808
CG4750-RC	loopin-1	0,0833169	0,0758908	0,0799892	0,0359372	0	0	-0,202582	0,41303	0,6279808
CG4751-RA	CG4751	5,28174	2,91756	19,492	9,43555	26,0879	22,0138	-0,039864	0,885916	0,6279808
CG4752-RA	CG4752	11,3773	5,56479	0,0272183	5,48212	3,69692	0,0669291	-0,166199	0,447101	0,6279808
CG4753-RA	Agpat4	80,8251	1,54504	1,51053	0,694456	4,70379	0,223377	-0,457902	0,104769	0,6279808
CG4753-RB	Agpat4	0,0434519	80,4927	2,10052	0,0326597	0,83419	0,0333403	-0,456828	0,105523	0,6279808
CG4755-RA	RhoGAP92B	16,0071	0,602182	0,985458	0,912197	0,793465	0,5738	-0,076824	0,704309	0,6279808
CG4756-RA	Sap30	20,8736	8,75095	25,7326	0,729992	0,529682	24,947	0,232535	0,320483	0,6279808
CG4757-RA	CG4757	1,43397	1,11871	5,03176	1,03864	2,8028	2,31903	0,483426	0,171515	0,13772387
CG4758-RA	Trp1	72,2368	55,6113	1,3026	1,69251	1,72389	8,80651	0,174333	0,466627	0,6279808
CG4758-RB	Trp1	60,7445	109,753	8,40603	5,92394	1,33545	0,526125	0,172278	0,472562	0,6279808
CG4759-RA	RpL27	1000,39	1274,91	0	0	0	0	-0,19823	0,47518	0,6279808
CG4760-RA	bol	4,28809	23,7787	21,2464	6,79002	0,0629181	2,39412	0,239088	0,350788	0,6279808
CG4760-RB	bol	4,80252	0,0498782	0,0525718	0,0288679	0,0447704	1,2794	0,230562	0,35139	0,6279808
CG4760-RC	bol	9,28296	4,40043	6,49329	0	0,0534589	0	0,23514	0,361458	0,6279808
CG4760-RD	bol	10,0909	19,8916	37,9398	0	0,0400134	34,6285	0,234968	0,337367	0,6279808
CG4760-RE	bol	4,98158	9,77616	14,582	0,468551	0	0,228634	0,179658	0,487391	0,6279808
CG4761-RA	knrl	9,18884	11,496	14,8848	13,6837	1,28332	4,09996	0,626645	0,022954	0,6279808
CG4763-RA	CG4763	0	0	0	0,576808	0	0,115242	NA	NA	0,6279808
CG4764-RA	Dsim\GD23074	22,7684	17,6606	37,1945	19,7815	16,1056	29,8602	0,235746	0,344969	0,6279808
CG4766-RA	CG4766	1,27116	1,96725	1,29148	1,30625	0,56592	0,63843	0,665949	0,032845	0,6279808
CG4767-RA	Tektin-A	0,532134	0	0,525477	0,187219	0	0,303352	0,052573	0,879395	0,6279808
CG4768-RA	CG4768	16,4068	34,2731	0,0750138	27,0333	3,69547	4,72257	-0,031257	0,890975	0,6279808
CG4769-RA	Dpse\GA18418	170,546	3,22632	6,52493	9,99567	2,70924	7,86528	-0,432517	0,14243	0,6279808
CG4770-RA	CG4770	0,0672871	0,245159	2,27509	2,46369	2,0389	2,34117	0,074748	0,791704	0,6279808
CG4771-RA	vret	0,451842	0,292081	0,727657	3,06287	9,52261	8,81624	-0,441632	0,179869	0,6279808
CG4772-RA	Ugt303A1	8,95724	6,33542	11,0498	4,99355	29,0998	25,4861	0,312337	0,326534	0,6279808
CG4774-RA	CLS	2,43099	2,86016	4,93523	0,0570234	0,0772385	0,0582116	-0,018326	0,941342	0,6279808
CG4774-RB	CLS	16,7563	18,188	23,1236	19,8452	23,1159	21,3288	-0,019754	0,936755	0,6279808
CG4774-RC	CLS	0,0476852	0,043435	0,0457806	0,053288	0,0721789	0,0543984	-0,018821	0,939767	0,6279808
CG4775-RA	Tango14	15,5055	97,6138	22,2913	69,3031	17,5506	19,8924	-0,076476	0,78451	0,6279808
CG4778-RA	obst-B	70,9234	82,3425	72,3677	78,8948	33,7528	49,6759	0,590934	0,01102	0,6279808
CG4779-RA	hgo	11,5919	5,91503	5,41534	17,7721	3,06378	3,98825	-0,587258	0,037167	0,6279808
CG4780-RA	Membrin	9,47941	70,5097	111,412	1,43475	0,0868602	1,34444	-0,055887	0,837337	0,6279808
CG4781-RA	CG4781	24,4545	19,3048	34,4517	4,38693	0,775067	3,45697	0,034238	0,896486	0,6279808
CG4783-RA	CG4783	0,257439	8,82911	10,8003	0,356846	0	0	0,230728	0,353055	0,6279808
CG4784-RA	Cpr72Ec	0,184055	111,917	0,0882701	0,0883834	0,444672	0,258261	-0,115124	0,741819	0,13772387
CG4785-RA	IntS14	3,48589	3,44374	6,29373	5,29729	3,77819	4,39007	-0,098375	0,706708	0,6279808
CG4786-RA	Rcd2	13,4741	6,55219	8,28566	11,479	17,8764	7,43259	-0,269538	0,328389	0,6279808
CG4788-RA	CG4788	5,25388	5,36763	0	7,31849	3,62462	5,87021	0,147382	0,583216	0,6279808
CG4789-RA	CG4789	17,0786	5,71301	20,3545	2,83186	5,98949	0	-0,326259	0,183974	0,6279808
CG4790-RA	fs(1)M3	0,0119213	0,0108588	0,0114452	0,0857894	0,0165478	25,1675	0,290242	0,417054	0,6279808
CG4792-RA	Dsim\Dcr-1	1,75939	1,84845	1,99455	17,5801	2,03766	1,2756	-0,01203	0,965642	0,6279808
CG4793-RB	CG4793	0,059978	0,0546321	0,0575825	0,117184	0,17968	0,0582378	0,521012	0,105318	0,6279808
CG4793-RC	CG4793	0,273886	0,359243	0,525893	0,116605	0,108868	0,0452392	1,33191	0,000198	0,6279808
CG4795-RA	Cpn	0	0	0,0226749	2,01163	0	1,38471	0,01562	0,897983	0,6279808
CG4795-RB	Cpn	0	3,05244	0,0580568	0	0	0	0,01562	0,897983	0,6279808
CG4797-RA	CG4797	1,5511	2,85464	0,235387	4,42001	4,20669	53,7329	-0,519325	0,117789	0,6279808
CG4797-RB	CG4797	1,6103	35,6533	1,62301	54,2522	0,380887	0,979157	-0,53075	0,111551	0,6279808
CG4798-RA	l(2)k01209	9,96636	10,8064	0,707557	60,847	0,0232634	15,018	0,225666	0,306231	0,6279808
CG4798-RB	l(2)k01209	0,020419	0,018599	14,2333	0,657575	16,6344	63,4087	0,141578	0,518445	0,6279808
CG4798-RC	l(2)k01209	0,0250953	0,0228585	0,0196035	14,5211	0,0245226	4,0202	0,16341	0,457475	0,6279808
CG4798-RD	l(2)k01209	4,0703	5,24912	0,024093	0,0531161	9,32901	11,1597	0,157135	0,477131	0,6279808

gene_id	Symbol	W1_FPKM	W2_FPKM	W3_FPKM	MCM51_FPKM	MCM52_FPKM	MCM53_FPKM	FC	p-value	p-adj
CG4799-RA	Pen	3,77503	3,06429	41,7785	5,06978	36,4476	0,686852	-0,269114	0,337486	0,6279808
CG4800-RA	Tctp	557,195	690,133	889,99	884,419	1155,1	1212,01	-0,493722	0,066323	0,6279808
CG4802-RA	Mtap	43,8323	10,1972	17,7308	0,0217212	0,285699	7,29946	-0,557057	0,03504	0,6279808
CG4803-RA	Takl2	0,722838	0,542221	3,52287	8,67018	2,34305	3,11552	-0,763017	0,029588	0,6279808
CG4804-RA	Spn31A	5,79935	5,52148	92,0803	1,38369	30,705	1,38641	-0,175811	0,558391	0,6279808
CG4805-RA	ppk28	0,691535	20,1503	0,937182	26,2563	5,93945	2,20485	0,483314	0,146459	0,6279808
CG4805-RB	ppk28	0,0358005	4,48859	0,430647	0,0403125	3,55328	6,77909	0,483314	0,146459	0,6279808
CG4806-RA	CG33228	9,76885	9,13154	20,8176	15,8365	13,3506	14,9269	-0,225944	0,418605	0,13772387
CG4807-RA	ab	0,0136605	0,0124429	26,5689	0,0760874	0	0,0643361	0,398357	0,168622	0,6279808
CG4807-RB	ab	0,0135263	0,0123207	0,0449587	5,26176	2,00845	4,0349	0,388478	0,181474	0,6279808
CG4807-RC	ab	37,7174	37,8232	0,0127748	7,60391	2,79694	1,35132	0,129448	0,675068	0,6279808
CG4807-RD	ab	0,0468291	0,0426552	0,126772	0,0884436	1,96076	0,0145138	0,388604	0,181337	0,6279808
CG4810-RA	eIF3d2	0	0	2,29027	0,0486999	0	0	-0,013096	0,914398	0,6279808
CG4812-RA	Ser8	0	0,0143142	0,0246548	0,0277963	0,020045	4,91035	0,061577	0,773576	0,6279808
CG4813-RA	CG4813	3,98028	4,4461	5,44103	10,0693	0,0852832	2,89269	0,302445	0,179974	0,6279808
CG4815-RA	CG4815	1,20063	0,0589677	0,953937	0,0756054	2,98019	2,11294	-0,710908	0,042191	0,6279808
CG4816-RA	qkr54B	0,0198208	1,19848	21,3518	0	1,53922	12,7685	0,252137	0,282813	0,6279808
CG4816-RB	qkr54B	0,0192323	0,925865	20,0118	1,35266	0,0484394	9,6785	0,252137	0,282813	0,13772387
CG4816-RC	qkr54B	2,71007	0,0302191	0,876624	0,0357617	0,893259	0	0,261525	0,268763	0,6279808
CG4817-RA	Ssrp	6,77159	0,0837474	0,139116	0	0	0,309956	-0,037655	0,887066	0,6279808
CG4818-RA	Cpr72Ea	0,0919423	0,0672724	0,0152872	99,447	0,119236	0,016814	-0,340376	0,232871	0,6279808
CG4820-RA	CG4820	3,21161	3,49418	4,13966	3,65428	3,50657	4,75494	-0,049302	0,850896	0,6279808
CG4820-RB	CG4820	0,0563787	0,0513537	0,054127	0,0679631	0,0920565	0,0693793	-0,049302	0,850896	0,6279808
CG4821-RA	teq	5,33403	7,37764	312,417	21,9626	3,62366	13,1738	0,038935	0,874571	0,13772387
CG4821-RB	teq	0,029657	0,0270137	0,0404657	0,030583	4,93714	4,97996	0,100422	0,669329	0,6279808
CG4821-RC	teq	0,982017	0,00707106	0,0296064	6,13678	6,82503	0,0784409	-0,182236	0,580106	0,6279808
CG4821-RD	teq	6,28579	8,4735	0,0348809	11,6748	0,0749678	34,9865	0,098176	0,68458	0,6279808
CG4822-RA	CG4822	0,0224544	0,020453	0,0215576	0,0235873	0,0319492	0,0240788	0,126599	0,645569	0,6279808
CG4822-RB	CG4822	0,0248623	0,0226463	0,0238693	3,83619	0,035579	0,0268145	0,109876	0,690786	0,6279808
CG4822-RC	CG4822	0,0248904	0,0226719	0,0238963	2,98464	0,0356216	0,0268466	0,123009	0,655115	0,6279808
CG4822-RD	CG4822	0,0253877	0,0231249	0,0243737	0,026856	2,4943	0,0274156	0,118578	0,667596	0,6279808
CG4822-RE	CG4822	0,0244937	0,0223106	0,0235154	0,0258549	0,961273	0,0263936	0,12529	0,649989	0,6279808
CG4822-RF	CG4822	0,0236013	3,20316	0,0226587	0,0248599	0,0336729	0,0253779	0,121907	0,65811	0,13772387
CG4822-RG	CG4822	13,6851	11,5499	11,1162	12,0232	3,42837	10,7078	0,118578	0,667596	0,6279808
CG4822-RH	CG4822	0,0158278	0,0144171	0,0151956	0,0163685	0,0221712	0,0167096	0,118578	0,667596	0,6279808
CG4824-RA	BicC	0,132694	0	0,016041	0	0	0,231344	-0,358688	0,315748	0,13772387
CG4824-RB	BicC	0,0181344	10,3626	4,46849	0,377934	0	5,28882	-0,418068	0,242395	0,6279808
CG4824-RD	BicC	0,0222878	0,0151083	5,18214	0,226622	4,99941	0,0175419	-0,397568	0,266665	0,6279808
CG4825-RA	CG4825	10,8557	30,9562	15,4834	2,67809	2,88614	46,0398	-0,338761	0,222068	0,13772387
CG4827-RA	veil	15,0874	8,39505	0,0190291	10,7634	18,3026	0,0211224	-0,091469	0,777551	0,6279808
CG4829-RA	CG4829	0,0214764	4,98258	11,0921	18,5913	6,19063	8,90661	0,153857	0,505193	0,6279808
CG4829-RB	CG4829	10,4175	27,012	9,56256	9,32734	5,4217	7,55449	0,151056	0,512749	0,6279808
CG4829-RC	CG4829	0,0222203	0,141283	0,0616089	3448,09	1,63451	0,764757	0,154691	0,503389	0,6279808
CG4829-RD	CG4829	10,6684	0,433343	8,50268	0,14497	15,3473	21,1589	0,162832	0,478763	0,6279808
CG4829-RE	CG4829	11,3203	1,58667	50,888	0,0946012	11,1213	13,8446	0,159712	0,487108	0,6279808
CG4830-RA	CG4830	24,4587	0,0623479	4,86228	14,0662	0,313202	0,0776417	-0,354873	0,253723	0,6279808
CG4832-RA	cnn	8,58733	11,2877	4,74977	9,87806	5,30418	0,0162769	-0,031958	0,89287	0,6279808
CG4832-RB	cnn	0,0138244	0,0125922	0,0137976	0,0142299	0,020063	0,759367	-0,050352	0,831474	0,6279808
CG4832-RC	cnn	5,15713	3,74875	1,92607	10,045	0,0215972	5,00524	-0,03042	0,897833	0,6279808
CG4832-RD	cnn	0,0143716	0,0130907	1,32585	0,014812	0,687601	22,6953	-0,029201	0,901904	0,6279808
CG4832-RE	cnn	0,0154323	0,0140568	12,9809	2,60816	3,84709	2,33856	-0,050352	0,831474	0,6279808
CG4835-RA	CG4835	3,21839	2,06284	3,3509	1,73654	7,36567	48,55	0,279135	0,340354	0,6279808
CG4836-RA	CG4836	0,025547	0,0310266	15,6182	0,0824663	11,2585	0,0317187	0,236944	0,507268	0,6279808
CG4836-RB	CG4836	0,0255733	0,0388233	0,0326041	1,88432	0,0496797	0,0345472	0,236944	0,507268	0,6279808
CG4836-RC	CG4836	0,0170709	0,0621975	0,0490533	5,0997	0	0,0459266	0,236944	0,507268	0,6279808
CG4836-RD	CG4836	0,0254486	0,0154536	0,0572879	0	6,45994	0,0179587	0,194743	0,585734	0,6279808
CG4836-RE	CG4836	0,0254748	0,0154694	0,0573619	3,1879	1,97762	0,0179779	0,194743	0,585734	0,6279808
CG4838-RA	beat-Ic	3,15429	2,70106	2,19237	3,52468	4,73957	2,78085	-0,529966	0,062817	0,6279808
CG4839-RA	CG4839	1,4142	0,741661	0,928692	82,5168	0,892869	1,90057	-0,477055	0,071888	0,6279808
CG4839-RB	CG4839	5,33748	4,3427	0,277259	74,2597	0,362296	6,06596	-0,492053	0,063688	0,6279808
CG4840-RA	cbs	7,49002	6,86386	7,05985	0,0449613	0,0609003	4,37276	0,044086	0,8331	0,6279808
CG4841-RA	tweek	10,1074	9,55567	0	0	14,4318	5,74167	0,18472	0,485951	0,6279808
CG4842-RA	CG4842	9,30545	6,06775	13,6431	0,0880292	9,01509	8,22495	0,264733	0,400684	0,6279808
CG4843-RA	Tm2	0,0687096	0,0288157	3,34898	0,0339732	0,0188034	0,0346811	-0,326207	0,216835	0,6279808
CG4843-RB	Tm2	714,404	57,92	0,0425274	41,7914	1,48374	46,8939	-0,326117	0,216925	0,6279808
CG4843-RC	Tm2	0,0452367	0,0533568	0,0562383	0,0337297	0,0602833	0,0344326	-0,509512	0,06468	0,6279808
CG4844-RA	nw	20,4616	13,9592	22,6167	39,4554	3,10331	17,9472	0,086646	0,800316	0,6279808
CG4844-RB	nw	25,178	14,3113	22,3311	23,9376	72,9037	71,2127	0,160892	0,639469	0,6279808
CG4845-RA	psidin	7,08157	6,78447	3,13614	0,0366774	3,90147	11,9902	0,061997	0,811113	0,6279808
CG4846-RA	beat-Ia	8,98153	10,1497	0,0174101	0,0183823	0,0248989	3,87159	0,195518	0,469868	0,6279808
CG4847-RA	Dsim\GD11280	17,5	10,0979	32,9938	11,6361	8,16661	7,68704	0,20077	0,536248	0,6279808
CG4847-RB	Dsim\GD11280	0,0522889	0,0476284	0,0502005	0,0591211	0,0324133	1,87822	0,201146	0,535531	0,6279808
CG4847-RC	Dsim\GD11280	0,0524966	0,0478176	0,0503999	0,0593875	3,51494	4,97986	0,20077	0,536248	0,6279808
CG4847-RD	Dsim\GD11280	0,0456745	0,0416035	0,0438503	0,0507828	19,0321	6,54976	0,199573	0,538715	0,6279808
CG4847-RE	Dsim\GD11280	0,0526221	0,0479318	0,0505203	0,0595485	0,0800799	0,060353	0,20077	0,536248	0,13772387
CG4848-RA	Cog1	9,19101	3,16884	0,13143	0,122512	0,109329	0,177541	0,145004	0,541478	0,6279808
CG4849-RA	CG4849	3,98989	3,73041	6,20181	8,12763	3,74815	4,56308	-0,273061	0,285364	0,6279808
CG4851-RA	Ppt2	8,3432	6,86248	9,42979	0	0	0,172911	-0,091505	0,714883	0,6279808
CG4852-RA	Sras	10,201	21,829	10,6158	0,0378792	12,0562	9,46272	-0,191239	0,41612	0,6279808
CG4853-RA	CG4853	12,0541	13,6814	11,5612	17,2637	0,0804408	0,060625	0,227442	0,297896	0,6279808
CG4853-RB	CG4853	0,0227638	0,0207349	0,0218546	0,02393	0,0687856	0,051841	0,226331	0,300825	0,6279808
CG4854-RA	CG4854	1,72045	1,29776	0,0163049	0	0	1,7352	0,1234	0,689509	0,6279808
CG4857-RB	tyf	5,07413	4,85384	6,02476	8,56019	0,130958	4,34572	-0,218121	0,320822	0,6279808
CG4858-RA	CG4858	30,5063	30,4391	42,3722	45,5068	49,9338	21,3863	-0,333071	0,169072	0,6279808
CG4859-RC	Mmp1	31,4919	0,0872268	7,00827	0,135157	4,46825	0,142353	-0,29327	0,254248	0,6279808
CG4859-RD	Mmp1	0,0282054	27,0267	7,38379	30,9691	7,98849	0,144805	-0,674314	0,006786	0,6279808
CG4860-RA	CG4860	3,54992	205,61	0,0349964	0,354045	2,52745	0	0,457378	0,125561	0,6279808
CG4863-RA	RpL3	0,10444	37,7056	84,2461	69,0941	2,31666	3,40886	-0,390236	0,075051	0,6279808
CG4863-RB	RpL3	0,042447	70,9504	50,3536	36,8778	2,50676	2,62521	-0,390236	0,075051	0,6279808
CG4863-RC	RpL3	0,0372118	0,041594	0,0438402	0,0529454	3,08089	3,36647	-0,750911	0,000661	0,6279808
CG4863-RD	RpL3	0,0976488	0,0374371	0,0394589	0,0451771	10,1001	9,30905	-0,76479	0,000598	0,6279808
CG4863-RE	RpL3	1121,46	216,042	313,022	165,88	0,902535	0,847202	-0,390231	0,075045	0,6279808
CG4865-RA	dgt4	0,0928032	0,0845316	0,0890966	0,129321	12,6431	0,132016	0,031941	0,916401	0,6279808
CG4866-RA	CG4866	32,6777	40,8464	38,1411	31,4211	0,369536	0,278505	-0,605364	0,069113	0,6279808
CG4867-RA	bc10	21,8256	23,1537	41,9459	25,2193	13,7881	46,2294	0,382085	0,066086	0,6279808
CG4869-RA	betaTub97EF	57,7593	56,1346	0,0237531	0,0261317	112,095	0,0266762	-0,582035	0,031896	0,6279808
CG4871-RA	SiaT	0,0414355	61,0555	131,298	79,7861	0,0617429	0,0465332	-0,117721	0,705567	0,13772387
CG4871-RB	SiaT	1,06108	0,0968144	0,102043	0,139447	1,49127	1,57739	-0,117721	0,705567	0,6279808
CG4872-RA	CG4872	4,48786	7,82293	4,25918	5,55345	72,2715	4,51055	0,010642	0,966743	0,6279808
CG4875-RA	Rcp	0,064172	13,7913	0,0206187	0,0225077	0,0304868	0,0229767	0,018255	0,944524	0,6279808
CG4875-RB	Rcp	5,27477	10,5791	9,4138	11,044	6,3598	6,31992	0,018255	0,944524	0,6279808
CG4877-RA	RAF2	1,98345	2,26404	127,516	102,635	1,54752	1,70867	0,278433	0,283785	0,6279808
CG4877-RB	RAF2	1,20911	0,509462	0,0417164	0,0480428	0,023077	0,0173922	0,27772	0,284559	0,6279808
CG4878-RA	eIF3b	0,0254857	0,0232141	0,0244678	0,0269659	0,0365254	0,0275278	-0,132566	0,519948	0,6279808

gene_id	Symbol	W1_FPKM	W2_FPKM	W3_FPKM	MCM51_FPKM	MCM52_FPKM	MCM53_FPKM	FC	p-value	p-adj
CG4878-RB	eIF3b	95,5257	124,072	131,762	181,106	96,07	107,693	-0,132566	0,519948	0,6279808
CG4879-RA	RecQ5	0,0179912	0,0163877	0,0172727	0,0187005	0,0258809	0,0195054	0,297559	0,188091	0,6279808
CG4879-RB	RecQ5	0,0197497	0,0179894	0,0189609	0,0206136	411,709	351,96	0,306829	0,176071	0,6279808
CG4879-RC	RecQ5	0,0275495	0,025094	0,0176328	0,0191072	0,0572296	0,0431317	0,297559	0,188091	0,6279808
CG4880-RA	CG4880	2,09423	5,75458	545,739	0,0139526	11,5234	6,21559	0,29911	0,373903	0,6279808
CG4881-RA	salr	5,07007	6,55379	6,55784	9,72248	0,0818423	0,0288378	0,145938	0,631368	0,6279808
CG4881-RB	salr	0,0152682	48,3564	7,27812	0,0157691	0,0750308	0,0776729	0,14524	0,632919	0,6279808
CG4882-RA	CG4882	23,6969	3,73463	39,3172	0,0334727	7,76632	8,52224	-0,229431	0,402404	0,6279808
CG4884-RA	CG4884	5,71195	2,57451	9,56378	1,57293	5,97632	6,51056	0,230324	0,440679	0,6279808
CG4886-RA	cyp33	0,0501715	0,0456997	2,60782	1,41099	0,0172715	0,803314	0,001306	0,995902	0,13772387
CG4887-RA	CG4887	7,23603	0,0920283	8,39353	15,2503	4,30816	5,69845	0,128518	0,64046	0,6279808
CG4889-RA	wg	6,90011	6,51709	5,40187	8,93366	21,5327	4,47153	0,369089	0,177578	0,6279808
CG4889-RB	wg	1,87869	2,49749	1,41368	0,026856	0,0256061	0,0274156	0,429193	0,111584	0,6279808
CG4891-RA	CG4891	0	0,0145706	0,128386	15,2518	4,29048	8,71088	NA	NA	0,6279808
CG4892-RA	CG4892	0,219036	0,266018	0,210288	25,8305	14,4261	8,29553	0,004271	0,989122	0,6279808
CG4893-RA	ringer	117,023	13,5594	8,28079	22,1448	1,02114	0,844342	-0,229794	0,460805	0,6279808
CG4894-RA	Ca-alpha1D	1,04419	0,00767032	0,00808455	2,35531	1,73874	2,44724	-0,040859	0,858819	0,6279808
CG4894-RB	Ca-alpha1D	4,69839	6,33368	3,22539	30,2832	0,730952	0,00873849	-0,042283	0,85394	0,13772387
CG4894-RC	Ca-alpha1D	0,00837711	0,00763045	0,715785	0,0303296	3,17565	3,39613	-0,043	0,85137	0,6279808
CG4894-RD	Ca-alpha1D	0,00826396	0,00752739	1,23372	0,00856012	0,0115333	0,00869219	-0,041684	0,856126	0,13772387
CG4896-RC	CG4896	0,0205929	2,84461	8,94403	2,40707	2,5077	1,8602	0,223961	0,298043	0,13772387
CG4896-RD	CG4896	3,43851	5,40341	48,2536	2,1924	14,8224	1,89213	0,225269	0,296047	0,6279808
CG4896-RE	CG4896	2,23068	0,0298741	11,9554	0,0353208	55,9748	9,84669	0,202788	0,35213	0,6279808
CG4897-RA	RpL7	1355,53	0,0341645	0,0360095	0,0408625	0,0553486	0,041714	-0,23467	0,343649	0,6279808
CG4898-RA	Tm1	0,0474455	0,0432167	0,0997434	0,0933478	113,691	77,6695	-0,755258	0,012751	0,6279808
CG4898-RB	Tm1	809,036	797,653	0,0282611	48,1788	0,109844	0,0827852	-0,884792	0,005756	0,6279808
CG4898-RC	Tm1	0,0308958	0,028142	10,0742	0,0314441	1163,28	1197,08	-1,006858	0,001905	0,6279808
CG4898-RE	Tm1	0,0428878	0,0390652	56,4369	22,2069	0,0680513	0,0512876	-0,890334	0,005463	0,6279808
CG4898-RF	Tm1	0,0415136	0,0378135	0	0	0	0	-1,007587	0,002108	0,6279808
CG4898-RG	Tm1	0,0266686	0,0242916	0	0,179535	0	0	-0,891474	0,005498	0,6279808
CG4898-RH	Tm1	0,0451132	0,0410922	3,74654	0,144601	0	2,33866	-1,0065	0,001923	0,6279808
CG4898-RI	Tm1	0,0219911	0,020031	0	1,86927	0	0	-1,00755	0,001933	0,6279808
CG4898-RJ	Tm1	0,0182799	0,0166506	0	0	3,16002	0	-0,891382	0,0055	0,6279808
CG4898-RK	Tm1	0,0272291	0,0248021	0	0	0,243181	0	-1,008647	0,002086	0,6279808
CG4898-RL	Tm1	125,371	122,139	0	0	0	0	-0,859001	0,006312	0,6279808
CG4899-RA	Pdh	0,0763293	0	0,0732807	0,243244	0,0223098	2,09864	-0,803677	0,016604	0,6279808
CG4899-RB	Pdh	0,075717	0,0837474	0,0726929	0,819611	7,88217	0,0929686	-0,803677	0,016604	0,6279808
CG4899-RC	Pdh	0,0738551	3,28772	0,0709053	0,252989	1,966	0,0902251	-0,803677	0,016604	0,6279808
CG4899-RD	Pdh	0,0736083	0,069526	0,0706684	1,26885	0,123356	0,0898635	-0,803677	0,016604	0,6279808
CG4900-RA	Irp-1A	8,24239	9,02382	12,4663	4,79451	21,2018	10,2533	-0,108881	0,637834	0,13772387
CG4901-RA	CG4901	9,47531	7,91715	11,0553	9,13816	85,5837	8,03001	0,095049	0,659288	0,6279808
CG4903-RA	MESR4	1,05245	0,00776232	1,06769	2,45085	1,19052	0,48634	0,008488	0,967119	0,6279808
CG4903-RB	MESR4	4,04234	4,71352	4,50468	4,3535	2,46461	3,3867	-0,002165	0,99169	0,6279808
CG4904-RA	Prosalpha6	18,1597	17,1215	16,3942	24,086	22,4153	27,0491	0,023305	0,937801	0,6279808
CG4905-RA	Syn2	0,0203206	0,0185094	0,019509	0,0420624	0,0287672	3,24061	-0,153867	0,536495	0,6279808
CG4905-RB	Syn2	1,81113	1,45767	2,13439	0,046914	2,50015	3,63295	-0,13247	0,593553	0,6279808
CG4905-RC	Syn2	0,0193393	0,0176155	0,0185669	0,0276795	0,0273145	6,82384	-0,129027	0,604684	0,6279808
CG4905-RD	Syn2	0,01829	0,0166598	0,0175595	0,0382087	0,0257688	4,04598	-0,150828	0,54625	0,6279808
CG4905-RE	Syn2	4,45173	5,34557	3,62753	0,0378792	2,71234	19,4476	-0,128608	0,604717	0,6279808
CG4907-RA	CG4907	0,44126	0,659259	0,575711	0,51083	0,731544	0,0131878	-0,456338	0,189038	0,6279808
CG4908-RA	CG4908	13,1011	19,8732	16,7704	0,0718933	10,9221	14,2226	0,129825	0,56919	0,6279808
CG4908-RB	CG4908	0,0400298	3,02156	0,038431	18,6827	1,35699	0,0447977	0,11984	0,597168	0,6279808
CG4909-RA	POSH	7,77118	7,6684	7,29032	7,71159	5,71524	5,94177	0,065813	0,748072	0,6279808
CG4910-RA	CCAP	0,052903	0,0481877	0,0507901	0,0629626	3,16726	0,0642746	0,374331	0,189322	0,6279808
CG4911-RA	CG4911	3,31947	2,32285	7,53637	3,40705	2,50807	3,54192	0,229562	0,463962	0,6279808
CG4912-RA	eEF1delta	62,1539	42,8676	114,433	61,8514	9,50907	77,2654	0,128225	0,671198	0,6279808
CG4912-RB	eEF1delta	70,7038	45,0882	140,976	77,2266	54,7685	90,3705	0,124872	0,679231	0,6279808
CG4913-RA	ear	6,46621	8,7117	8,01443	6,08774	4,00798	4,74901	0,159352	0,562528	0,6279808
CG4914-RA	CG4914	9,91739	0	10,5792	0,0821334	4,14195	0	0,094807	0,748878	0,6279808
CG4916-RA	me31B	51,0562	29,0682	79,9409	0,047048	16,5221	21,6679	0,263248	0,317048	0,6279808
CG4916-RB	me31B	0,0426387	15,0304	15,0643	12,8358	33,4036	42,8844	0,271326	0,299989	0,6279808
CG4917-RA	wfs1	0,0253877	0,0231249	0,0243737	36,1919	0,0235024	41,8812	-0,240115	0,280483	0,6279808
CG4917-RB	wfs1	3,03688	3,01444	4,19894	8,83314	0,0262616	1,97427	-0,240115	0,280483	0,6279808
CG4918-RA	RpLP2	0,112821	3,76567	0,055371	7,08688	0,0237147	0,0391867	-0,791781	0,005655	0,6279808
CG4918-RB	RpLP2	4992	326,051	442,787	26,7808	0,0231909	16,3739	-0,791774	0,005656	0,6279808
CG4919-RA	Gclm	53,0083	2,11691	3,6579	0	9,10669	5,03096	0,508523	0,043245	0,6279808
CG4920-RA	ea	10,1141	13,3048	15,085	5,12042	4,3086	3,62349	0,734143	0,004756	0,6279808
CG4921-RA	Rab4	0,0411175	49,9866	45,0901	69,5198	110,019	0,0869592	0,381154	0,067801	0,6279808
CG4921-RB	Rab4	0,0677007	4,75664	5,01352	0,046914	0,0635453	28,8607	0,315412	0,136274	0,6279808
CG4921-RC	Rab4	42,5424	1,18367	1,09957	0,0851841	0,115382	3,05747	0,098998	0,629671	0,6279808
CG4922-RA	sala	86,5054	0,0835148	6,61647	0,0646927	0	0,0565477	0,048054	0,863694	0,6279808
CG4924-RA	icln	6,30569	43,6119	37,1231	1,16215	0,149966	0,846033	0,085003	0,792597	0,6279808
CG4925-RA	Golgin104	4,61651	4,41345	5,11698	5,42804	636,256	613,229	0,128403	0,544831	0,6279808
CG4926-RA	Ror	9,91429	9,58594	10,02	13,3252	36,4215	6,38569	0,125926	0,586847	0,6279808
CG4927-RC	CG4927	2,23791	2,60584	1,86058	1,32079	1,44346	1,47627	0,5321	0,080565	0,6279808
CG4928-RA	CG4928	18,7891	8,23313	4,26398	23,0561	0	2,67002	-0,071296	0,806181	0,6279808
CG4928-RB	CG4928	11,5654	16,8947	3,1597	1,99821	0	0	-0,096666	0,741088	0,6279808
CG4930-RA	EndoGI	21,6721	25,1143	30,3082	0,00851476	27,7424	28,167	-0,161197	0,46797	0,6279808
CG4931-RA	Sra-1	13,0992	11,6819	14,4815	24,905	1,69565	1,73054	-0,156732	0,467926	0,6279808
CG4933-RA	Tcs3	4,22071	0,656239	7,26589	0	27,5744	6,84552	-0,419394	0,190059	0,6279808
CG4934-RA	brn	3,86276	8,6099	4,3411	26,5492	7,47059	7,85354	-0,18167	0,54775	0,6279808
CG4935-RA	CG4935	1,50036	1,5033	2,85206	5,68016	0,601525	1,04762	-0,084349	0,785202	0,6279808
CG4936-RA	CG4936	7,61411	6,64719	11,1884	0,0312616	0	0	-0,036143	0,876569	0,6279808
CG4937-RB	RhoGAP15B	6,10195	9,35149	0	4,48012	1,61275	0,149685	-0,048465	0,856604	0,6279808
CG4937-RC	RhoGAP15B	2,18553	1,85021	25,7894	1,58365	7,78768	0,0608995	-0,050379	0,85096	0,6279808
CG4938-RA	SerRS-m	4,74126	4,29903	7,86236	5,05169	15,8073	18,2913	0,224835	0,393822	0,6279808
CG4942-RA	CG4942	3,84162	2,49611	4,59794	4,73598	3,29796	3,04817	0,081396	0,776193	0,6279808
CG4943-RA	Smurf	10,3562	4,43302	13,0422	6,63905	1,32698	1867,48	0,226938	0,39649	0,6279808
CG4944-RA	cib	13,5815	14,3375	17,4859	0,0966833	0,175166	19,1855	0,146905	0,594299	0,6279808
CG4944-RB	cib	0,0795454	0,0724555	0,0763683	68,7969	4,38262	0,098698	0,27478	0,319665	0,6279808
CG4944-RC	cib	65,0922	64,3971	56,94	12,8139	4,42389	44,9625	0,146905	0,594299	0,6279808
CG4945-RA	CG4945	0,0256307	0,0233462	6,84618	0,0277378	0,037571	0,0283158	-0,10449	0,678786	0,6279808
CG4945-RB	CG4945	0,0273719	0,0249323	0,024607	0,0297947	0,0403572	0,0304156	0,061205	0,833515	0,6279808
CG4945-RC	CG4945	0,0311092	0,0283364	0,0262787	0,0342914	0,0464479	0,035006	0,050516	0,861698	0,6279808
CG4946-RA	Mob3	10,0203	4,65471	7,13936	8,36841	5,70396	9,84925	0,20967	0,360413	0,6279808
CG4947-RA	Tgt	5,75969	0	7,95529	7,86588	2,96206	7,79412	-0,392227	0,121608	0,6279808
CG4949-RA	CG4949	0,0331429	0	2,89595	2,07468	1,11805	4,90829	-0,084112	0,775771	0,6279808
CG4949-RB	CG4949	1,83385	0,0827114	0,314216	0,113897	0,941838	0,11627	-0,084112	0,775771	0,6279808
CG4950-RB	CG4950	0,170886	819,324	0,145832	1241,3	2088,06	2173,62	0,334831	0,181421	0,6279808
CG4951-RA	CG4951	0,0429715	73,9321	0,0412552	173,312	0,0642775	0,0484434	0,244635	0,367854	0,6279808
CG4951-RB	CG4951	0,0647378	0,0391414	0,0621522	0,0474546	0,102408	0,0771808	0,550095	0,022257	0,6279808
CG4952-RA	dac	0,01263	0,012362	0,0129736	2,69916	2,97295	0,0141893	0,506417	0,042024	0,6279808
CG4952-RB	dac	0,0135884	1,3786	3,96993	0,0138997	0,0175603	0,0141714	0,506741	0,041924	0,6279808
CG4952-RC	dac	0,0135717	6,10522	0,0129577	3,37179	0,018935	0,0141089	0,506417	0,042024	0,6279808

gene_id	Symbol	W1_FPKM	W2_FPKM	W3_FPKM	MCM51_FPKM	MCM52_FPKM	MCM53_FPKM	FC	p-value	p-adj
CG4952-RD	dac	0,0135134	0,0122938	2,98045	0,0138821	3,36962	60,3282	0,506741	0,041924	0,6279808
CG4952-RE	dac	8,16883	0,0122413	0,498521	1,8906	0,0188272	0,0352999	0,506398	0,041966	0,6279808
CG4952-RF	dac	0,0134968	2,64535	1,49157	0,0183362	0,0188272	0,0187183	0,506741	0,041924	0,6279808
CG4952-RG	dac	0,0134392	13,2368	8,91225	21,2978	0,0188034	11,2124	0,506417	0,042024	0,6279808
CG4953-RA	CG4953	6,30105	5,29131	10,763	6,04497	11,0281	10,8091	0,027118	0,907996	0,6279808
CG4954-RA	eIF3c	72,3019	76,2957	0,0481676	2636,41	12,6875	0,210518	-0,138249	0,496771	0,6279808
CG4955-RA	CG4955	0	16,7514	11,7859	18,6252	9,99997	8,88721	-0,066275	0,671132	0,6279808
CG4956-RA	CG4956	0	0	0	0	0	0	NA	NA	0,6279808
CG4957-RA	CG4957	6,93242	15,3471	4,14637	18,8505	24,9028	22,2219	-0,309994	0,317695	0,6279808
CG4960-RA	CG4960	0	0	0	0,157254	0	0	NA	NA	0,6279808
CG4962-RA	CG4962	74,7113	103,688	109,261	167,206	171,189	133,204	-0,51928	0,084165	0,6279808
CG4963-RA	mfrn	10,6676	10,6001	12,7313	11,6184	9,09284	3,8129	0,064291	0,73249	0,6279808
CG4965-RA	twe	0,0284605	0,0259238	4,5904	2,16491	0,0410816	0,0309616	0,522298	0,118347	0,6279808
CG4966-RA	HPS4	7,91294	10,383	6,90627	16,022	10,7473	27,9587	0,1748	0,438933	0,6279808
CG4966-RB	HPS4	0,0193223	0,0577052	0,0185506	14,2651	4,78717	11,5784	0,174269	0,444897	0,6279808
CG4968-RA	CG4968	21,6546	20,7375	33,1002	27,221	18,7974	2,85887	0,131385	0,569485	0,6279808
CG4969-RA	Wnt6	4,29392	3,75015	3,87993	0,549702	2,86968	3,88455	0,276901	0,297801	0,6279808
CG4970-RA	CG4970	0	0	3,83759	0,0545226	0,0122269	0,00921495	-0,02282	0,900541	0,6279808
CG4970-RB	CG4970	0	0	3,05058	0,00902685	0,205667	0,155003	-0,061983	0,681756	0,6279808
CG4971-RA	Wnt10	0,631298	0,632533	0,484867	0,418435	0,537026	0,368905	0,234827	0,504126	0,6279808
CG4972-RA	CG4972	35,1269	31,838	46,4036	30,7654	7,62276	2491,12	-0,04142	0,841778	0,6279808
CG4973-RA	Dpse\GA18564	2,01989	1,54379	0	0,0369687	0	0	-0,113139	0,696216	0,6279808
CG4974-RA	dally	20,115	0,329602	0,644671	0,26662	216,249	0,331511	0,274853	0,248418	0,6279808
CG4975-RA	CG4975	2,32249	1,72336	0,0401753	25,5334	5,42513	5,41506	-0,25084	0,346407	0,6279808
CG4975-RB	CG4975	0,0413233	3,76166	0,0396728	11,2437	14,9186	4,74423	-0,51404	0,018459	0,6279808
CG4975-RC	CG4975	2,08604	0,0223603	4,34787	10,8918	8,73794	0,0481659	-0,25084	0,346407	0,6279808
CG4976-RA	NSD	5,78133	5,99651	6,26435	9,72868	1,04844	4,51896	-0,12759	0,58324	0,6279808
CG4977-RA	Dsim\GD23783	5,58539	0	0,155737	0	0	0	0,245639	0,390007	0,6279808
CG4978-RA	Mcm7	9,35315	8,45838	0,016543	0,456313	470,816	12,7808	-0,252256	0,361139	0,6279808
CG4979-RA	sxe2	1,78696	20,4336	2,74917	0,0102579	1,32149	1,16613	0,635552	0,061734	0,6279808
CG4980-RA	BCAS2	6,72511	4,96598	15,6085	7,69722	3,45871	4,08608	-0,328435	0,305777	0,6279808
CG4982-RA	CG4982	128,222	222,944	149,192	99,4426	324,04	203,436	-0,351598	0,24095	0,6279808
CG4983-RA	CG4983	0	0	0	0	3,84773	2,7189	0,01562	0,897983	0,6279808
CG4984-RA	CG4984	10,7961	8,3142	16,309	0,0422783	0,0572663	0,0231347	-0,556646	0,014055	0,6279808
CG4986-RA	Mst57Dc	0	0	0	0	0	0	-0,041811	0,731452	0,6279808
CG4988-RA	CG4988	0	0,0673729	2925,13	8,21859	0	0	-0,041811	0,731452	0,6279808
CG4991-RA	CG4991	0,0337869	0,0307754	0,0324374	0,0357229	0,196362	0	-0,752008	0,025198	0,6279808
CG4991-RB	CG4991	0,035531	0,0323641	0,0341119	3,13952	0,128138	4288,8	-0,749911	0,025765	0,6279808
CG4991-RC	CG4991	2,82719	1,60745	3,42309	0,763288	0,0483869	0,14799	-0,751344	0,025355	0,6279808
CG4993-RA	PRL-1	43,0128	48,2722	29,5329	0,849393	0,709226	30,5805	0,058283	0,787589	0,6279808
CG4993-RB	PRL-1	36,8922	30,9344	40,6869	18,1693	11,8375	2,85425	0,028354	0,894055	0,13772387
CG4994-RA	Mpcp2	280,142	358,85	22,0143	29,4592	9,67858	11,0399	-0,217668	0,333049	0,6279808
CG4994-RB	Mpcp2	0,038671	0,0352242	0,0150407	0,0161955	0,0219369	0,016533	-0,217825	0,33273	0,6279808
CG4995-RA	CG4995	0,0478579	0,0435923	0,0459464	0,0535043	1,62626	8,18982	-0,476976	0,173685	0,6279808
CG4995-RB	CG4995	0,0483481	0,660582	0,0464171	0,0541196	9,21164	8,22631	-0,475993	0,174369	0,6279808
CG4995-RC	CG4995	4,32049	1,85042	7,60911	3,29795	28,4977	3,5526	-0,460478	0,189224	0,6279808
CG4996-RA	Vps50	7,95654	0,124868	6,95334	0	6,44977	0	0,025988	0,916373	0,6279808
CG4998-RA	CG4998	75,3742	0,230519	0,296434	1,4518	0	0,359948	0,269355	0,408393	0,6279808
CG4998-RB	CG4998	0,0159232	6,14349	0,33132	0,0910709	0,246306	0,185631	0,267987	0,41048	0,6279808
CG4999-RA	Tsp66E	94,272	118,416	49,2905	23,255	0,017956	0,0124992	0,409505	0,096043	0,6279808
CG4999-RB	Tsp66E	30,6164	44,9829	37,5904	60,8682	0,0161029	19,2896	0,42841	0,082618	0,6279808
CG4999-RC	Tsp66E	29,2086	0,0269693	19,2016	38,5397	0,0165847	38,8781	0,408427	0,096741	0,6279808
CG5000-RA	msps	11,1696	12,0316	9,99838	14,1256	26,2964	5,65481	0,201837	0,425329	0,6279808
CG5001-RA	CG5001	35,0599	0,0187575	4,10362	1,89929	0,0300152	0,888763	-0,176392	0,517563	0,6279808
CG5002-RA	CG5002	4,62498	20,5788	8,30758	8,41732	0,0648379	12,4452	-1,119545	0,000437	0,6279808
CG5003-RA	CG5003	5,47345	11,0017	8,66338	9,70468	5,16331	7,04694	0,321746	0,202596	0,6279808
CG5004-RA	CG5004	11,4039	0,262627	0,73184	0,904319	0,0847037	0,0393241	0,003966	0,987257	0,6279808
CG5005-RA	HLH54F	9,8556	10,6976	9,72975	11,3066	0,0639093	12,059	0,009969	0,968843	0,6279808
CG5006-RA	Or33c	0	0,0794017	0	0,103764	0	0,708344	-0,310186	0,141014	0,6279808
CG5008-RA	GNBP3	3,5765	1,95464	7,76865	8,92226	5,11835	6,2786	-0,471598	0,148359	0,6279808
CG5009-RA	CG5009	12,2914	15,7716	16,3102	15,8026	21,1508	13,3461	-0,052064	0,799435	0,6279808
CG5010-RA	Chchd2	101,833	113,32	4,8626	0,0690437	0,201508	0	-0,175388	0,504964	0,6279808
CG5011-RA	CG5011	31,5942	21,6525	0,0314875	8,5347	54,2839	0,0360568	-0,301342	0,35115	0,6279808
CG5012-RA	Dsim\GD14139	73,133	3,42568	2,5144	2,66026	0,0417153	0,022553	-0,076109	0,793779	0,6279808
CG5013-RA	CG5013	5,18974	0,0231072	7,00149	17,7297	1,0575	1,73282	-0,259781	0,375487	0,6279808
CG5014-RA	Vap33	0,0290741	41,7361	0	8,47946	4,66887	8,13128	0,266755	0,163144	0,6279808
CG5014-RB	Vap33	15,3872	0,85339	10,1247	4,58996	5,7584	16,7894	0,265057	0,165208	0,6279808
CG5014-RC	Vap33	12,5765	1,61176	0,0279129	17,9264	0	0,0298355	0,290672	0,130755	0,6279808
CG5014-RD	Vap33	50,5427	6,0983	30,8491	13,2575	7,18457	30,6001	0,269361	0,158429	0,6279808
CG5016-RA	Mst57Db	0	0	9,87233	4,47889	7,10721	4,12717	0,01562	0,897983	0,6279808
CG5017-RA	mil	0,0603433	3,54436	0,0579331	9,11654	1,61767	4,0114	-0,367076	0,273538	0,6279808
CG5018-RA	l(3)72Dn	6,31651	4,20245	12,0027	4,67963	1,26426	5,35309	-0,103077	0,729592	0,6279808
CG5020-RA	CLIP-190	8,52707	20,1692	0,0118573	15,4874	14,1125	14,929	-0,259838	0,207455	0,6279808
CG5020-RB	CLIP-190	5,59993	8,55344	0,0400991	30,0319	30,1024	0,0124047	-0,266592	0,19698	0,6279808
CG5020-RC	CLIP-190	0,0123506	5,487	15,9476	0,380403	0	0,0128086	-0,282783	0,175838	0,6279808
CG5020-RH	CLIP-190	0,0417673	0,0112498	18,7519	9,94376	8,59852	0,242377	-0,26683	0,197955	0,6279808
CG5020-RI	CLIP-190	12,3151	0,0380446	0,0112777	6,24955	4,62585	59,2488	-0,435132	0,113801	0,6279808
CG5020-RJ	CLIP-190	15,8398	14,1988	0,0116312	0,0128007	0,0173386	45,9797	-0,428225	0,1192	0,6279808
CG5020-RK	CLIP-190	0,0117468	10,6816	0,148679	0,0477642	0,0646969	6,90742	-0,584188	0,040005	0,6279808
CG5020-RL	CLIP-190	0,0121151	0,0106998	11,5535	0,0466483	22,1617	0,0297037	-0,284229	0,173327	0,6279808
CG5021-RA	CG5021	10,5707	0,0194046	0,0204525	0,0223171	0,0302286	0,0227821	-0,223401	0,369102	0,6279808
CG5021-RB	CG5021	8,76203	273,498	259,41	187,666	217,383	211,829	-0,223401	0,369102	0,6279808
CG5022-RA	CG5022	5,36527	0,0442981	15,6607	0,0544775	0,438404	5,63533	-0,145764	0,493705	0,6279808
CG5023-RA	CG5023	209,421	0	0,613295	257,978	0,261211	4,28663	-0,643222	0,068354	0,6279808
CG5024-RA	CG5024	0	0,154324	0	0	0	0	-0,041811	0,731452	0,6279808
CG5025-RA	Sps2	10,8124	1,5964	0,0466904	20,4905	4,56072	5,17187	-0,406954	0,122681	0,6279808
CG5025-RB	Sps2	0,0486328	0,503034	0,405447	2,06376	92,0531	15,15	-0,406954	0,122681	0,6279808
CG5026-RA	CG5026	0,0276275	0,0225361	0,0237531	0,0261317	0,0353956	0,0266762	0,234234	0,39042	0,6279808
CG5026-RB	CG5026	3,5466	0,0187283	0,0197397	65,3398	0,0291239	0,0219495	0,4034	0,116107	0,6279808
CG5027-RA	CG5027	11,9136	10,4954	13,3668	7,80194	8,19408	9,72481	0,050275	0,808886	0,6279808
CG5028-RA	CG5028	11,2073	13,1026	15,474	20,6786	14,9118	14,2458	0,122515	0,506435	0,6279808
CG5028-RB	CG5028	0,0436817	0,0397883	0,041937	0,0483247	0,0654561	0,0493316	0,373267	0,036264	0,6279808
CG5028-RC	CG5028	0,0458012	0,0417189	0,0439718	0,0509398	0,0689983	0,0520013	0,325781	0,071964	0,6279808
CG5028-RD	CG5028	56,1321	54,4468	63,6997	46,2187	34,5125	32,3961	-0,04738	0,799631	0,6279808
CG5028-RE	CG5028	0,0471073	0,0429086	0,0452258	0,0525653	0,0712	0,0536606	0,126585	0,491995	0,6279808
CG5029-RA	SamDC	0,108737	0,0990449	46,2355	65,4641	0,194649	105,629	-0,803432	0,020319	0,6279808
CG5029-RB	SamDC	26,033	11,2979	0,104394	0,143705	71,6403	0,146699	-0,977541	0,00499	0,6279808
CG5032-RA	aft	1,83409	6,27947	2,54343	2,47552	0,042089	0,0317208	0,067901	0,762298	0,6279808
CG5033-RA	CG5033	11,5123	2,21423	23,178	16,1631	8,99924	1,24	-0,100116	0,741426	0,6279808
CG5033-RB	CG5033	0,0262136	23,7344	0,0251667	0,0277846	2,19355	0,00971258	-0,100116	0,741426	0,6279808
CG5034-RA	GATAd	14,8143	26,8363	37,7979	44,309	4,956	7,57987	0,359659	0,134442	0,6279808
CG5036-RA	Dhit	24,4474	28,436	30,7013	2,91912	1,43472	3,29875	0,644064	0,000832	0,6279808
CG5037-RA	Dpse\GA18613	36,5285	24,8684	41,3032	39,0177	0,0507244	28,7732	-0,674215	0,028643	0,6279808
CG5038-RA	CG5038	4,20354	44,5776	48,6992	5,1351	31,537	38,1158	0,299231	0,24247	0,6279808

gene_id	Symbol	W1_FPKM	W2_FPKM	W3_FPKM	MCM51_FPKM	MCM52_FPKM	MCM53_FPKM	FC	p-value	p-adj
CG5039-RA	CG5039	16,1794	0,0351214	0,0370181	0,0421162	0,0570467	0,0429938	1,276116	5,63E-08	0,6279808
CG5041-RA	Tfb4	2,73659	2,90812	4,68196	4,87618	2,95507	2,39415	0,166866	0,574476	0,6279808
CG5043-RA	CG5043	0,061485	0,166261	0,0590293	0,0701214	0	0	0,160712	0,423615	0,6279808
CG5044-RA	CG5044	0,0405456	0,0369317	0,0389262	10,7755	5,11358	0,0454331	-0,417747	0,133667	0,6279808
CG5044-RB	CG5044	16,6137	12,0989	27,7081	7,01414	0,0664799	26,6496	-0,417747	0,133667	0,6279808
CG5045-RA	CG5045	13,0218	17,0871	21,1972	0,38313	8,14234	0,0556127	-0,347801	0,172199	0,6279808
CG5047-RA	mTerf3	10,7215	11,2198	0,0708	12,6854	188,982	9,7626	0,138602	0,524195	0,6279808
CG5048-RA	CG5048	6,7257	0,270623	4,69319	0	11,4366	0	-0,603186	0,05271	0,6279808
CG5050-RA	CG5050	1,06317	1,88839	16,8067	1,10849	47,5741	0,0256489	0,528646	0,134679	0,6279808
CG5052-RA	pim	5,63259	4,35047	13,8827	9,627	23,1343	22,9259	0,018082	0,954626	0,6279808
CG5053-RA	RASSF8	10,6496	13,4808	9,5265	0,0183006	0,0247883	0,0186819	0,513086	0,01436	0,6279808
CG5055-RA	baz	6,37433	2,79181	186,213	9,69189	161,816	166,518	0,078252	0,799547	0,6279808
CG5055-RB	baz	2,06158	77,2436	102,739	4,50342	125,495	109,623	0,051895	0,866468	0,6279808
CG5056-RA	CG5056	19,0289	18,3165	23,8729	27,5034	5,10165	4,98528	-0,562084	0,027089	0,6279808
CG5057-RA	MED10	28,9589	31,4213	40,6136	10,8998	41,9095	34,0612	-0,04033	0,855084	0,6279808
CG5059-RA	CG5059	102,001	23,5958	0,0342778	3,83991	3,90379	3,51044	0,241247	0,439064	0,6279808
CG5059-RB	CG5059	0,0357038	0,735613	79,3298	11,0215	11,6186	7,79217	0,281791	0,368931	0,6279808
CG5059-RC	CG5059	89,6091	4,57327	0,0433114	0	0	51,2735	0,277857	0,375603	0,6279808
CG5059-RD	CG5059	0,0451132	0	27,0218	4,9145	4,04569	16,1538	0,265289	0,396374	0,6279808
CG5060-RA	cic	1,49874	1,47706	2,15829	5,38079	6,9747	1,90611	-0,529927	0,081195	0,6279808
CG5060-RB	cic	1,9836	2,93605	8,94023	4,33443	2,69136	1,82386	-0,432289	0,145247	0,6279808
CG5062-RA	CG5062	0,17102	0,113292	16,0986	12,1908	0,0289914	8,01123	0,352875	0,239298	0,6279808
CG5063-RA	Trax	6,79504	3,55725	3,61457	8,93493	44,9318	3,33542	0,311135	0,240311	0,6279808
CG5063-RB	Trax	0,0612949	6,72817	7,37886	0,07526	1,57845	0,0135984	0,311135	0,240311	0,6279808
CG5064-RA	Srp68	36,7758	12,1851	0,0382456	15,5967	25,9123	11,0882	0,086757	0,65293	0,6279808
CG5065-RA	CG5065	20,1585	25,0492	2,27871	3,49686	8,78115	11,73	0,096637	0,759968	0,6279808
CG5067-RA	cic	0,0103573	2,32079	1,00927	3,31299	6,58989	7,05528	0,122954	0,658617	0,6279808
CG5067-RB	cic	12,3787	14,4209	9,95237	16,2299	0	0	0,10914	0,696573	0,6279808
CG5068-RA	CG5068	10,7144	0,609792	48,0447	0,691104	46,8327	0,343042	-0,141974	0,480836	0,6279808
CG5069-RA	croc	2,76408	3,13819	2,98067	0,104422	0	11,4108	0,293267	0,23919	0,6279808
CG5070-RA	CG5070	64,1243	11,1162	0,116469	60,8967	2,20502	2,40181	-0,120438	0,697075	0,6279808
CG5071-RA	CG5071	0,830604	0,113132	69,149	1,06177	5,88495	0,0402657	-0,330997	0,293686	0,6279808
CG5071-RB	CG5071	0,0309682	0,65964	0,516716	0,0332028	5,73441	0,0405326	-0,330997	0,293686	0,6279808
CG5072-RA	Cdk4	12,8701	8,57323	16,0277	9,17871	9,07879	9,98565	0,135054	0,580106	0,6279808
CG5072-RB	Cdk4	5,39984	4,23028	7,30737	6,92607	5,13698	6,33876	0,172951	0,473577	0,13772387
CG5072-RC	Cdk4	0,0338041	1,30862	0,032454	0,0364946	0,0494321	0,037255	0,170516	0,485796	0,13772387
CG5073-RA	Ccm3	70,1966	14,6701	16,6684	0,949735	5,24629	0,568564	0,345089	0,175219	0,6279808
CG5075-RA	Vha68-3	0,255899	0,110411	0,219818	0,199926	0,15867	0,183687	2,671832	2,89E-21	0,13772387
CG5076-RA	Elk	0,789222	4,77749	0,83272	17,9206	0	1,03968	-0,812049	0,001529	0,6279808
CG5077-RA	CG42668	4,65837	2,1887	8,25326	9,44951	10,1994	0,0269975	0,045681	0,818246	0,6279808
CG5077-RD	CG42668	14,3822	0,231784	0	0,503749	0,443329	0,038056	-0,250664	0,198702	0,6279808
CG5077-RE	CG42668	13,7605	6,34619	2,36	8,09855	3,68415	11,1612	0,041183	0,835528	0,6279808
CG5077-RF	CG42668	0,017889	0,102126	6,25107	0,145025	0,196437	370,739	0,045681	0,818246	0,6279808
CG5077-RG	CG42668	0,0323795	0,626943	19,3461	3,33783	3,60811	655,039	0,016507	0,934615	0,6279808
CG5078-RA	CG5078	0	0	0,104681	0,186563	6,51911	0,0883883	-0,069433	0,76063	0,6279808
CG5079-RA	CG5079	0	76,549	0	0,683631	16,1988	6,60595	-0,212738	0,512419	0,6279808
CG5080-RA	CG5080	0,0327974	33,6617	97,4164	48,6122	0	94,4998	-0,003486	0,990893	0,6279808
CG5080-RB	CG5080	114,72	11,883	1,70026	32,1841	2,68735	43,0934	-0,00339	0,991146	0,6279808
CG5081-RA	Syx7	0,0419351	0,0381974	18,9572	25,6585	34,2948	38,0745	0,275735	0,150723	0,6279808
CG5081-RB	Syx7	26,4747	28,1439	0,0215429	0,0461905	0,0625653	0,047153	0,279683	0,146011	0,6279808
CG5083-RA	Rbf2	0,0245635	0,0223742	18,821	22,4482	8,15686	5,98883	0,311636	0,354286	0,6279808
CG5084-RA	CG5084	24,2977	0,0234767	1,81872	0,01419	0,0192205	4,56813	-0,671724	0,058025	0,6279808
CG5085-RA	Sirt2	14,3243	13,6279	16,0858	12,3939	0,523982	0	0,393527	0,060059	0,6279808
CG5087-RA	CG5087	4,75921	0,0346164	3,85328	5,16975	2,52824	2,72432	0,107216	0,632623	0,6279808
CG5089-RA	CG5089	0,475366	0	0,0394343	0,477057	0,126691	0,0954817	0,056951	0,873077	0,6279808
CG5091-RA	gny	0,0259834	76,2464	0,0249457	81,4124	0,0381328	0,0287392	-0,243062	0,348652	0,6279808
CG5092-RA	Tor	4,00825	19,0769	3,88119	57,5284	2,1493	2,6581	-0,074319	0,796123	0,6279808
CG5093-RA	Doc3	2,94593	0,279072	4,1106	0,0284565	6,76809	0,0290495	0,227122	0,389555	0,6279808
CG5094-RA	Sgt	41,1759	17,4991	70,3898	21,5962	0	6,65045	-0,12359	0,651565	0,6279808
CG5096-RA	CG5096	17,4545	2,33125	28,106	3,1557	33,0362	29,3792	-0,091947	0,753576	0,6279808
CG5096-RB	CG5096	32,751	9,64749	46,5773	23,7924	29,9315	18,7387	-0,074777	0,798538	0,13772387
CG5097-RA	MtnC	0	0,133082	0	0,0615227	0,134232	10,9183	-1,033255	0,003886	0,6279808
CG5098-RA	CG5098	3,98828	0,0124198	3,73735	23,8113	4,58116	9,15524	0,370424	0,181383	0,6279808
CG5098-RB	CG5098	0,0137315	0,0125076	0,013183	78,6488	11,8547	80,8417	0,224879	0,406164	0,13772387
CG5099-RA	msi	8,55064	15,1013	0,321741	9,4512	0,0449734	0,243504	0,225124	0,344266	0,13772387
CG5099-RB	msi	8,11378	3,14575	10,0353	9,66679	18,7267	0,0306932	0,220746	0,358177	0,6279808
CG5102-RA	da	22,9545	24,2879	24,8136	32,2126	94,7161	17,1239	0,065852	0,761883	0,6279808
CG5103-RA	CG5103	0	13,5446	0	0,0915232	0,0724719	4,69108	NA	NA	0,6279808
CG5104-RB	CG5104	26,7697	3,17789	29,1193	8,63197	5,26803	14,3339	0,292712	0,159003	0,6279808
CG5105-RA	Plap	2,82442	7,8411	4,08518	12,462	10,067	3,74236	-0,199442	0,379983	0,6279808
CG5106-RA	scpr-C	0,389445	0,325173	0,685466	0,26114	0,682065	0,51699	0,064615	0,843406	0,6279808
CG5107-RA	CG5107	0,153427	26,1901	32,7469	0,587105	0	0,227491	0,01939	0,899027	0,6279808
CG5108-RA	mRpS7	66,3697	22,8587	94,1476	15,8068	15,455	102,334	-0,108144	0,669262	0,6279808
CG5109-RA	Pcl	3,86915	4,41513	4,67004	6,48382	2,2664	77,6444	0,122326	0,648206	0,6279808
CG5110-RA	CG5110	31,7959	23,079	39,1114	8,46565	42,2167	4,05556	-0,056007	0,834195	0,6279808
CG5111-RA	CG5111	0	5,81013	6,4505	11,4543	4,43897	5,62604	0,080748	0,662111	0,6279808
CG5112-RA	CG5112	25,8121	6,22548	27,0161	22,891	5,58822	7,4229	0,292625	0,159334	0,6279808
CG5114-RA	CG5114	12,8186	7,60206	24,1263	14,7627	10,0318	21,9436	-0,402814	0,210796	0,6279808
CG5116-RA	CG5116	10,2512	0,192905	0,203323	7,88351	35,5259	20,4909	0,420016	0,040134	0,6279808
CG5118-RA	Charon	7,72797	3,33662	9,89868	0,145933	19,2107	6,76346	0,121825	0,539823	0,6279808
CG5119-RA	pAbp	0,0214764	2,00109	13,8522	0,0268888	0,0304868	3,37288	0,025464	0,897632	0,6279808
CG5119-RB	pAbp	0,0222803	22,794	4,43463	233,681	0,0316883	42,6456	0,02609	0,895284	0,6279808
CG5119-RC	pAbp	1106,54	20,161	33,1774	5,99864	642,273	9,75822	0,021155	0,915077	0,6279808
CG5119-RD	pAbp	0,0217593	4,62685	5,29735	20,1797	0,0309091	47,7825	0,019446	0,921831	0,6279808
CG5119-RE	pAbp	0,0198148	21,8393	45,1217	30,5833	0,0280176	0,0347707	0,019981	0,919692	0,6279808
CG5119-RF	pAbp	0,019311	0,0288849	0,0304448	2,71827	0,0272728	20,6568	0,025449	0,897693	0,6279808
CG5119-RG	pAbp	0,0229536	22,5227	23,8527	4,26179	345,69	3,96696	0,019864	0,920167	0,6279808
CG5119-RH	pAbp	0,0227247	15,5647	47,4411	30,6137	0,0323545	30,9585	0,026173	0,894946	0,6279808
CG5121-RA	MED28	10,8051	8,546	15,4536	4,76226	2,66515	6,47323	-0,100675	0,705071	0,6279808
CG5122-RA	CG5122	0	0,0111753	0,0397873	17,5498	1,02227	9,36639	-0,039698	0,794165	0,6279808
CG5123-RA	hid	10,2395	0	0,363015	1,75776	0,0713708	0	0,674863	0,028131	0,6279808
CG5124-RA	adp	6,01345	5,81899	6,90855	8,48953	5,98348	5,57848	-0,166764	0,400728	0,6279808
CG5125-RA	ninaC	0,276835	0,25216	0,0966614	3,18619	0,0235582	0,0883318	0,011713	0,973555	0,6279808
CG5125-RB	ninaC	0,100683	0,122278	0,365981	0,376979	0,356523	0,22018	0,063512	0,848502	0,6279808
CG5126-RA	CG5126	6,80872	148,47	76,2337	223,567	45,87	62,9626	1,107309	1,94E-06	0,6279808
CG5127-RA	Vps33B	4,44557	2,81635	4,72859	5,08257	4,54647	3,26628	0,282125	0,21515	0,6279808
CG5130-RA	ZnT77C	4,55303	6,45723	8,04469	7,56786	10,982	5,97251	0,14948	0,504471	0,6279808
CG5130-RB	ZnT77C	10,2613	10,8187	9,79117	7,10501	0,0472096	5,93594	0,140829	0,528414	0,6279808
CG5131-RA	CG5131	16,6148	15,8033	24,5403	6,34095	0,0169952	23,2767	-0,091788	0,746161	0,13772387
CG5133-RA	Doc1	6,14861	143,414	0,0257385	9,65152	0,0479708	0,246559	-0,199015	0,383618	0,6279808
CG5134-RB	CG42518	21,7577	27,1935	32,8478	152,515	24,3036	28,8693	0,039537	0,850856	0,6279808
CG5134-RC	CG42518	0,109639	0,0998666	0,10526	0,0751741	0,196795	0,148317	-0,003941	0,985196	0,6279808
CG5137-RA	Cyp312a1	0,168275	4,36052	2,13651	2,06546	22,7661	0,0546192	0,367959	0,281995	0,6279808
CG5139-RA	CG5139	0	5,28533	0	0	0,0478422	0	0,01562	0,897983	0,6279808

gene_id	Symbol	W1_FPKM	W2_FPKM	W3_FPKM	MCM51_FPKM	MCM52_FPKM	MCM53_FPKM	FC	p-value	p-adj
CG5140-RA	nopo	0,0407708	0,0582825	0,06143	16,8928	53,3848	84,95	-0,18632	0,444083	0,6279808
CG5142-RB	Ttc30	0,742735	0,0111753	0,386381	0	1,96614	0,232005	0,020652	0,94577	0,6279808
CG5144-RA	CG5144	0,30638	31,6901	3,22788	0,452751	0,0561494	6,38207	0,28471	0,420723	0,6279808
CG5146-RA	l(3)psg2	3,58475	2,87674	4,64564	3,88594	1,34653	2,02058	0,41769	0,135783	0,6279808
CG5147-RA	RpIIIC53	9,77239	31,6598	0,713409	10,0043	0,28262	0,342869	0,292739	0,231881	0,6279808
CG5148-RA	cal1	0,018662	0,411802	0,367266	0,480356	0,125437	0,26647	0,66673	0,008198	0,6279808
CG5149-RA	CG5149	0,0337351	0,0307283	0,0323877	0,0364139	0,0493228	0,0371727	-0,076838	0,705805	0,6279808
CG5149-RB	CG5149	25,0882	21,0691	22,1896	28,2405	20,2764	21,1988	-0,07525	0,711921	0,6279808
CG5150-RA	Alp9	0,548499	0,0499611	2,15808	0,468507	0,341854	0,397305	0,407936	0,229554	0,6279808
CG5151-RA	CG5151	6,66434	0	7,31637	3,25082	4,11402	0	0,351688	0,143945	0,13772387
CG5151-RB	CG5151	8,84491	0	0,380461	3,89347	5,79509	274,229	0,370923	0,120319	0,6279808
CG5154-RA	Idgf5	7,91236	8,77301	13,0555	0,822442	1,90253	3,12871	-0,53956	0,045825	0,6279808
CG5155-RA	gudu	0,476509	0,376166	0,325578	0,115853	0,185382	0,20131	0,129479	0,713336	0,6279808
CG5157-RA	CG5157	7,28343	0,776521	2,39435	323,346	0,638913	6,38239	-0,054021	0,859145	0,6279808
CG5160-RA	CG5160	4,46247	10,2683	7,01513	12,4838	6,73078	4,93535	0,034318	0,903075	0,6279808
CG5161-RA	Trs20	36,3715	45,655	23,3173	53,1864	0,396645	27,6143	0,119142	0,684557	0,6279808
CG5162-RA	CG5162	9,32023	0,351968	18,3308	6,71654	0,524923	11,089	-0,324376	0,310214	0,6279808
CG5163-RA	TfIIA-S	84,9624	93,9556	111,634	121,528	92,3535	0,0702371	0,171812	0,374676	0,6279808
CG5164-RA	GstE1	21,9989	8,99051	29,481	0,113699	32,4193	0,308808	-1,319528	2,76E-05	0,6279808
CG5165-RA	Pgm1	54,7042	69,1292	80,6437	83,8365	113,094	0,020567	-0,556946	0,034586	0,6279808
CG5166-RA	Atx2	3,88601	0,0625855	0,0124492	1,58483	2,48409	1,61785	0,597345	0,043016	0,6279808
CG5166-RB	Atx2	0,0129671	866,878	12,1722	0,756427	0,0180431	0,623788	0,575228	0,050614	0,6279808
CG5166-RC	Atx2	14,2085	0,0412048	0,201771	0	3,80403	0,017519	0,597345	0,043016	0,6279808
CG5167-RA	Sccpdh2	11,7767	16,4021	23,0367	0,0385214	11,7941	404,925	-0,195819	0,459994	0,6279808
CG5168-RA	Wdfy2	19,2319	22,6065	27,4545	29,409	6,91236	21,1614	-0,028212	0,886265	0,6279808
CG5169-RA	GckIII	16,7076	12,2502	33,5042	0,0222568	29,6201	28,9579	0,071077	0,74322	0,6279808
CG5170-RA	Dp1	0,0115463	0,0105172	0,0110852	0,790118	0,0160133	0,0120686	0,107932	0,697764	0,6279808
CG5170-RB	Dp1	0,0115322	0,0105044	0,0110716	2,69648	0,0159933	0,0120535	0,107932	0,697764	0,6279808
CG5170-RC	Dp1	0,0115383	0,0105099	0,0110774	0,0118927	0,0160019	0,01206	0,107932	0,697764	0,6279808
CG5170-RD	Dp1	0,0114861	0,0104624	0,0110274	2,83786	0,0159276	0,012004	0,107773	0,698173	0,6279808
CG5170-RE	Dp1	106,083	123,922	100,674	34,1609	51,6881	71,077	0,107948	0,69774	0,6279808
CG5170-RF	Dp1	0,0114147	0,0103973	0,0109588	0,0223322	0,0158259	0,0119274	0,107954	0,697721	0,13772387
CG5171-RA	CG5171	0,0608619	4,66674	0,0584311	9,26061	0,0953003	22,6885	-0,354624	0,223024	0,6279808
CG5171-RB	CG5171	13,2376	5,06193	25,1636	7,73929	32,9236	29,5351	-0,373052	0,199236	0,6279808
CG5171-RC	CG5171	85,3002	6,01389	88,5095	341,501	105,233	111,008	-0,354624	0,223024	0,6279808
CG5172-RC	CG5172	0,969062	0,29423	0,475538	0,13433	30,8294	0,397709	0,23284	0,425814	0,6279808
CG5172-RD	CG5172	0,138912	0,12653	0,533454	0,387538	3,16487	7,79094	0,34459	0,292125	0,6279808
CG5174-RA	CG5174	0,0854059	0,105098	11,6014	27,0169	11,1145	10,611	-0,008195	0,968097	0,6279808
CG5174-RB	CG5174	0,102179	9,73426	20,0947	0,155581	0,142911	1,66282	-0,02211	0,912747	0,6279808
CG5174-RG	CG5174	29,5659	32,1843	16,8776	15,2689	0,179391	13,7142	-0,21291	0,322746	0,6279808
CG5174-RH	CG5174	0,0448681	2,04485	16,4122	37,2531	15,4472	20,9989	-0,009866	0,961726	0,6279808
CG5174-RI	CG5174	18,5797	9,96845	0,882632	7,93847	0,0674343	6,23286	-0,011466	0,955401	0,6279808
CG5174-RJ	CG5174	0,115382	0,694871	31,2872	38,5806	24,7305	50,297	-0,208097	0,330407	0,6279808
CG5174-RK	CG5174	10,5025	14,7119	0,0258118	3,19077	22,2272	0,0291376	-0,190202	0,37768	0,6279808
CG5175-RA	kuk	10,8486	6,02244	27,6728	26,7343	4,26325	11,7377	-0,033817	0,911446	0,6279808
CG5175-RB	kuk	8,80155	6,47126	14,3394	10,1278	6,81677	14,6184	-0,034113	0,910713	0,6279808
CG5177-RA	CG5177	324,345	22,4345	41,8749	75,6158	38,7089	530,878	-0,009868	0,971322	0,6279808
CG5178-RA	Act88F	0,231182	0,287149	4,26538	0,40909	8,20753	0,26367	-0,239806	0,500586	0,6279808
CG5179-RA	Cdk9	7,65349	8,8726	11,6574	11,8544	6,0957	8,20248	0,062953	0,789234	0,6279808
CG5180-RA	CG5180	0,0435569	33,0745	0,0418173	3,07379	25,3668	12,4035	0,192912	0,358626	0,6279808
CG5180-RB	CG5180	8,35192	0,0468863	13,4474	0,0178979	0,0786682	0,0195167	0,181687	0,385199	0,6279808
CG5181-RA	CG5181	11,9149	24,919	15,377	0,13648	6,50336	3,21472	0,352459	0,216153	0,6279808
CG5182-RA	Pk34A	0	0,0111691	0	1,10086	17,2235	0,7537	0,063548	0,603667	0,6279808
CG5183-RA	KdelR	0,0533012	33,8137	0,0511724	5,25923	0,387386	4,38316	-0,045472	0,816672	0,6279808
CG5183-RB	KdelR	85,7023	0,0485505	98,2789	0,285998	38,2265	29,1915	-0,045472	0,816672	0,6279808
CG5183-RC	KdelR	0,0563466	81,8263	0,0540961	0,0604222	0,0818423	3,41321	-0,045472	0,816672	0,6279808
CG5184-RA	Dsim\mRpS11	34,4212	18,8321	0,022106	0,0184595	15,2714	1,04169	-0,283404	0,255599	0,6279808
CG5185-RA	Tom	39,2985	5,54265	18,5074	9,99922	6,19654	8,3489	-0,112989	0,725446	0,6279808
CG5186-RA	slim	14,8915	0,0777936	0,0980978	11,3268	3,15363	0,1352	0,117388	0,578019	0,6279808
CG5186-RB	slim	20,745	0,0930716	30,5974	0,105508	25,9716	24,5986	0,126828	0,548645	0,6279808
CG5187-RA	Doc2	0,0268092	0,0244197	0,710845	94,4993	55,2055	65,5998	-0,157385	0,514178	0,6279808
CG5187-RB	Doc2	3,06447	2,58398	8,25177	111,765	85,4839	73,9294	-0,152504	0,526092	0,6279808
CG5188-RA	CG5188	4,60701	6,95422	9,90632	4,75097	11,4928	7,3368	0,101269	0,741712	0,6279808
CG5189-RA	CG5189	29,8938	29,3236	0,0430761	0,13244	0,0324251	0,0508226	-0,313592	0,206085	0,6279808
CG5190-RA	rswl	9,37688	7,70169	0,0819948	2,87855	3,16535	0,107706	-0,150706	0,595815	0,6279808
CG5191-RA	CG5191	0,0278605	0,0253773	18,7228	47,7429	16,9281	0,0713668	0,482087	0,045385	0,6279808
CG5191-RB	CG5191	0,0285466	14,9643	0,0554033	67,8788	16,2086	52,5725	0,467389	0,053047	0,6279808
CG5191-RC	CG5191	0,025885	0,0235779	51,2145	0,0763943	0,0946936	68,4924	0,447392	0,062556	0,6279808
CG5191-RD	CG5191	15,3562	12,022	54,1171	13,798	43,1096	19,1632	0,482087	0,045385	0,6279808
CG5191-RE	CG5191	14,0694	15,6849	20,097	25,5081	61,4466	19,4243	0,467389	0,053047	0,6279808
CG5192-RB	Rh6	2,77931	2,34784	3,7012	14,1919	1,3264	5,7634	-0,892315	0,002617	0,6279808
CG5193-RA	TfIIB	26,4504	23,4183	29,2375	36,5578	35,469	22,0632	-0,309536	0,180708	0,6279808
CG5194-RA	CG5194	0,704184	5,40337	0,366198	4,41801	38,517	2,78354	0,000453	0,998978	0,6279808
CG5195-RA	atk	1,92846	2,42123	45,1212	27,6915	0,0428365	4,86026	0,218407	0,39483	0,6279808
CG5196-RA	CG5196	12,5397	10,7209	35,0062	0,0992344	0,134414	37,3799	-0,048167	0,808069	0,6279808
CG5196-RB	CG5196	0,035531	2,37722	0,0360095	0,0344889	0,0467154	0,041714	-0,060635	0,760954	0,6279808
CG5198-RA	holn1	7,55231	11,3147	13,7104	9,32567	70,1854	7,36358	0,204547	0,439984	0,6279808
CG5199-RA	CG5199	3,06086	33,9666	5,05761	13,257	40,6514	11,8019	0,233618	0,288721	0,6279808
CG5201-RA	Dad	0,0177687	0,0209733	0,017059	23,0147	0	0,0188442	-0,050653	0,835349	0,6279808
CG5201-RB	Dad	6,92404	0,0193546	4,97706	47,184	4,31914	4,62358	-0,037492	0,877812	0,6279808
CG5201-RC	Dad	4,31176	2,76078	3,75005	0,99563	1,65146	3,51905	-0,050977	0,834411	0,6279808
CG5202-RA	escl	3,78852	3,29399	17,9407	10,5327	0,0298645	0,0727563	0,123786	0,693384	0,6279808
CG5202-RB	escl	0,0369207	0,739859	33,5681	17,9507	10,0069	2,77219	0,123786	0,693384	0,6279808
CG5203-RA	STUB1	33,4516	0,0513244	3,81555	107,908	73,5735	0,0616813	0,038496	0,877312	0,6279808
CG5204-RA	kmg	0,0838526	12,8182	6,37234	5,39388	14,2524	13,8498	-0,203683	0,566074	0,6279808
CG5205-RA	obe	2,11831	0,928467	1,61182	1,43922	0,827678	0	0,088814	0,69642	0,6279808
CG5206-RA	bon	15,2926	15,756	14,3499	17,2469	1,94958	8,13423	0,432006	0,081423	0,6279808
CG5207-RA	scpr-A	0,188573	0,0706689	0,0362082	0,0937932	0,127043	0,0340836	-0,558619	0,034792	0,6279808
CG5208-RA	Patr-1	8,31021	7,55272	9,0586	11,2516	12,3299	7,20296	0,077211	0,732629	0,6279808
CG5210-RA	Idgf6	233,912	0,0176207	1,50222	4,27356	1,65305	0,60458	-0,201097	0,442956	0,6279808
CG5212-RA	Pli	10,0527	1,11456	23,7942	2,02627	6,35599	0,0773584	0,309514	0,249271	0,6279808
CG5212-RB	Pli	24,3037	4,82335	2,46321	7,70666	2,61471	1,97165	0,310265	0,248098	0,6279808
CG5212-RC	Pli	23,6987	3,77258	10,308	20,5018	17,4009	7,40815	0,330485	0,220351	0,6279808
CG5213-RA	CG5213	0	2,17014	8,70635	0,0097379	0,450357	0,253737	0,01562	0,897983	0,6279808
CG5214-RA	CG5214	88,2151	100,24	116,773	92,2507	113,491	104,388	-0,12261	0,618494	0,6279808
CG5215-RA	Zn72D	18,0197	13,5578	3,0135	5,44651	0,0551775	1,83099	-0,054202	0,792157	0,6279808
CG5215-RB	Zn72D	2,60721	4,63227	16,4487	7,9024	161,015	13,5322	-0,048917	0,812765	0,13772387
CG5216-RA	Sirt1	29,9287	0,767056	23,6667	15,488	2,63246	0,0128453	0,120299	0,653188	0,6279808
CG5217-RA	CG5217	0	27,5386	52,5588	31,9023	0	37,922	0,044335	0,715924	0,6279808
CG5219-RA	mRpL15	30,9818	8,99633	77,5063	0,0365553	2,95559	0,0719778	-0,263037	0,290814	0,6279808
CG5220-RA	CG5220	14,1067	10,1253	21,0192	16,6445	10,4732	5,8315	-0,30905	0,275408	0,6279808
CG5222-RA	IntS9	2,76184	2,09639	53,5939	0,0397772	2,12772	0,040606	0,109736	0,66905	0,6279808
CG5224-RA	GstE11	18,9533	23,0847	39,8567	1,75427	43,4664	55,2579	-0,486448	0,107716	0,6279808
CG5225-RA	CG5225	0,222104	43,2622	35,1287	1,48003	1,66488	0	-0,468241	0,145296	0,6279808

gene_id	Symbol	W1_FPKM	W2_FPKM	W3_FPKM	MCM51_FPKM	MCM52_FPKM	MCM53_FPKM	FC	p-value	p-adj
CG5226-RA	CG43066	4,11342	3,99211	4,39576	24,604	5,44915	3,98586	-0,472834	0,029871	0,13772387
CG5227-RA	sdk	7,0433	10,012	63,7832	0,00762942	74,1549	58,3541	0,313986	0,286617	0,6279808
CG5227-RB	sdk	5,37526	0,00680022	41,4869	7,31094	57,6857	38,353	0,313021	0,288092	0,6279808
CG5227-RC	sdk	0,00742203	0,0067605	0,0434895	0,00752745	0,0681552	0,0513659	0,313075	0,288078	0,6279808
CG5227-RD	sdk	0,00740208	6,32767	0,0737921	9,6681	0,125613	0,0946696	0,313431	0,28745	0,6279808
CG5229-RA	chm	4,5273	22,4274	11,7645	4,90191	12,9207	12,9221	0,115186	0,684594	0,6279808
CG5231-RC	Las	12,9509	0,0322543	3,13747	4,43936	3,26946	3,82647	-0,387148	0,125264	0,6279808
CG5232-RA	NANS	3,15704	2,0578	19,2003	0,438015	0,0673082	17,9216	0,128879	0,629725	0,6279808
CG5235-RA	CG5235	2,26036	1,82518	68,3811	5,80877	2,91965	0,0549692	-0,652022	0,016038	0,6279808
CG5237-RA	unc79	3,36658	4,82545	3,17965	20,0483	0,0267256	1,84913	0,29162	0,2719	0,6279808
CG5241-RA	Tasp1	4,53549	3,24535	4,03417	5,12271	49,6635	3,71847	0,367552	0,182854	0,6279808
CG5242-RA	mRpL40	27,8628	28,2834	47,8216	40,8865	1,41536	1,57286	-0,56351	0,06477	0,6279808
CG5245-RA	Dpse\GA26228	0,577803	0,43232	1,06983	20,724	11,1567	0,516642	0,437052	0,21853	0,6279808
CG5246-RA	CG5246	0,669894	0,249621	15,1876	14,008	0,045524	1,24387	0,433043	0,206696	0,6279808
CG5247-RA	Irbp	1,57908	2,80684	2,67877	3,03554	72,5476	2,5763	0,181813	0,530663	0,13772387
CG5248-RA	loco	16,8741	20,773	16,9402	17,7234	0,0419112	3,28485	0,297652	0,232805	0,6279808
CG5248-RB	loco	0,0167422	3,76674	0,0160735	4,21719	23,1979	10,9461	0,288058	0,252865	0,6279808
CG5248-RC	loco	0,0186252	0,0169651	0,0178813	0,0193883	2,17587	0,942868	0,297663	0,23561	0,6279808
CG5248-RD	loco	0,0125867	0,0114648	0,012084	2,83123	4,44144	0,610033	0,274217	0,274227	0,6279808
CG5249-RA	Blimp-1	0,740558	16,0467	0,0244395	15,6893	0,0107111	0,027494	-0,67867	0,056727	0,6279808
CG5250-RA	CG5250	0	0,114206	0,0968008	0,028742	0	0	-0,041811	0,731452	0,6279808
CG5252-RA	Ranbp9	2,03556	1,54511	2,81027	2,95692	0,0127219	0,29085	-0,023033	0,940511	0,6279808
CG5254-RA	CG5254	16,278	16,8512	30,973	36,9626	49,486	49,5614	-0,971307	0,000786	0,6279808
CG5255-RA	CG5255	6,13037	5,28304	5,60358	0	172,423	9,09056	-0,534856	0,084576	0,6279808
CG5258-RA	NHP2	92,4616	32,4562	148,091	15,4533	224,066	0	-0,533064	0,102939	0,6279808
CG5261-RA	muc	21,4676	31,8708	28,6383	191,062	25,0396	33,2443	-0,21476	0,325782	0,6279808
CG5261-RB	muc	33,1132	302,744	491,247	41,3212	0,114765	31,5873	-0,239221	0,275028	0,6279808
CG5262-RA	CG5262	6,79527	0,0308385	6,15084	21,2244	39,546	0,0572212	0,384903	0,134677	0,6279808
CG5263-RA	smg	9,77345	8,37106	17,9187	7,35207	5,7379	6,57284	0,491345	0,103687	0,13772387
CG5263-RB	smg	0,0154071	0,0140338	0,0147917	0,0159177	0,0215606	0,0162494	0,484753	0,107749	0,6279808
CG5263-RC	smg	0,0150492	0,0137078	6,98565	0,0155349	0,0210421	0,0158586	0,491486	0,102364	0,6279808
CG5263-RD	smg	0,0152085	0,013853	0,0146011	1,49878	1,22423	0,0160325	0,312399	0,339205	0,6279808
CG5263-RE	smg	0,0175376	0,0159745	0,0168371	2,12804	0,024665	1,67829	0,491924	0,102032	0,6279808
CG5264-RA	btn	0,0289976	0,026413	0,0278394	2,07981	1,77617	30,6378	-0,397209	0,125943	0,6279808
CG5265-RA	CG5265	0,0488727	0	0	1,21848	0,079078	14,4926	-0,014388	0,910154	0,6279808
CG5266-RA	Prosalpha2	27,5977	0,132764	49,0131	31,9199	82,1089	0,214457	0,00907	0,97384	0,6279808
CG5267-RA	CG5267	0,228478	0,116942	32,6103	2,96119	0,0263236	0,0213165	-0,3584	0,316591	0,6279808
CG5268-RA	blp	15,2403	18,6351	23,8552	20,7625	1,32448	169,752	-0,264319	0,317554	0,6279808
CG5269-RA	vib	35,506	0	0	0	0	0,124331	0,080502	0,766561	0,6279808
CG5270-RA	CG5270	1,48722	1,67639	1,26719	3,30881	1,02863	1,50129	0,430332	0,13816	0,6279808
CG5270-RB	CG5270	5,42083	8,59117	4,91837	5,09098	2,08539	2,91906	0,231553	0,423703	0,6279808
CG5271-RA	RpS27A	4003,28	7,92735	4314,96	10,6379	7,24666	6,45141	-0,580382	0,018819	0,6279808
CG5272-RA	gnu	0,610484	22,45	7,23257	4,16612	4,79187	0,0173922	-0,920858	0,008988	0,6279808
CG5273-RA	CG5273	7,91761	13,3478	5,48648	0,0343093	9,95365	10,6074	0,373967	0,088403	0,6279808
CG5273-RB	CG5273	18,9164	23,6202	28,9212	28,4675	4,60721	10,0274	0,373967	0,088403	0,6279808
CG5273-RC	CG5273	4,96584	4,55168	4,7675	9,49813	5,00443	3,79158	0,37414	0,088264	0,6279808
CG5274-RA	CG5274	0,0498811	0,0346164	0,0325039	7,39466	3,17848	34,6249	-0,001084	0,996079	0,6279808
CG5274-RB	CG5274	6,54836	0,0344185	147,061	7,85716	0,0759244	22,8531	-0,001084	0,996079	0,6279808
CG5276-RA	CG5276	7,62559	6,1818	6,49889	8,29831	4,63003	6,04602	0,130054	0,590614	0,6279808
CG5277-RA	Ip259	0,0780422	7,91426	455,095	13,5277	39,3519	40,9326	-0,015085	0,946056	0,6279808
CG5278-RA	sit	98,8115	42,1484	122,467	21,7355	158,05	129,93	-0,373101	0,218176	0,6279808
CG5279-RA	Rh5	2,32696	7,86969	9,08326	0,00984998	6,12646	5,24443	-0,5996	0,064684	0,6279808
CG5280-RA	CG5280	0,602516	0,036286	0,867676	2,28006	11,9106	0,0445603	0,465641	0,153391	0,6279808
CG5281-RA	CG5281	6,59929	8,43064	8,70662	12,7224	8,13676	8,77206	-0,293032	0,15923	0,6279808
CG5282-RA	CG5282	7,74683	10,4336	0,0366545	10,2858	3,33663	4,19745	0,186257	0,406305	0,6279808
CG5284-RA	ClC-c	9,61684	84,3858	4,04042	7,36284	3,67965	2,5405	-0,179837	0,403352	0,6279808
CG5284-RB	ClC-c	3,4717	45,4981	22,1011	37,4157	11,9922	30,7532	-0,152469	0,479206	0,6279808
CG5285-RA	CG5285	2,82175	3,87246	9,49093	9,99573	42,539	4,03389	0,312705	0,290886	0,6279808
CG5287-RA	CG5287	8,20661	0,03873	12,8133	11,422	0,354619	0,0128453	0,166481	0,423499	0,6279808
CG5288-RA	Galk	22,8015	0,293984	73,1859	0,0414538	3,16301	3,56114	-0,329734	0,246774	0,6279808
CG5288-RB	Galk	0,032879	5,0448	76,6216	44,2776	2,01873	0,235407	-0,328402	0,247408	0,6279808
CG5288-RC	Galk	0,0380037	2,52128	30,2175	0,0436507	0,264276	32,7917	-0,345905	0,224707	0,6279808
CG5289-RA	Rpn6	29,0033	28,099	44,2502	50,2449	25,1839	33,6378	-0,021157	0,927036	0,6279808
CG5290-RA	CG5290	4,72943	5,34096	6,46781	32,6411	5,7203	5,31627	-0,197614	0,390883	0,6279808
CG5292-RA	CG5292	8,44055	4,53005	8,13018	7,29898	0,0262694	0	0,096436	0,712741	0,6279808
CG5295-RA	bmm	18,518	44,4079	29,2094	27,159	32,1044	0,0173339	-0,121973	0,628002	0,6279808
CG5295-RB	bmm	9,42298	0,425231	9,2453	19,1696	7,5588	17,0278	-0,127742	0,609684	0,6279808
CG5300-RA	Klp31E	5,83276	5,12426	5,92287	17,1759	4,52612	3,58079	0,042121	0,834107	0,6279808
CG5302-RA	CG43336	0,0865774	0,0946328	0	0	0	0	0,056249	0,844752	0,6279808
CG5303-RA	mei-S332	0,0400055	25,3026	3,41653	0,0189916	1,80881	0,0236056	-0,40813	0,133878	0,6279808
CG5304-RA	dmGlut	29,5911	21,9526	39,4747	18,5287	24,3675	30,0232	-0,117225	0,705951	0,6279808
CG5308-RA	dpr5	3,5885	6,18077	0	0,0539865	9,30431	4,03241	0,144384	0,524802	0,6279808
CG5308-RB	dpr5	2,41211	5,0562	4,03732	0	384,572	0,0551115	0,272466	0,235464	0,6279808
CG5310-RA	nmdyn-D6	3,04737	3,28351	3,21109	1,82835	0	25,0967	0,0362	0,904649	0,6279808
CG5313-RA	RfC3	2,46375	2,19007	4,33171	4,34223	3,0351	4,12924	-0,218162	0,446341	0,6279808
CG5315-RA	AdipoR	28,8499	7,04584	42,5988	96,9563	39,9046	36,7585	-0,017262	0,930188	0,6279808
CG5315-RB	AdipoR	0,0333099	0,0394234	0,0319795	27,4988	0,0486507	0,0366661	0,048005	0,810009	0,6279808
CG5315-RC	AdipoR	38,6331	13,2722	24,4037	0,0478335	17,9087	21,5888	-0,008762	0,964547	0,6279808
CG5316-RA	CG5316	9,65818	9,66542	7,77027	14,4965	8,73845	9,07827	0,443921	0,155413	0,6279808
CG5316-RB	CG5316	0,0998625	0,0909617	0,095874	0,128564	0,174141	0,131243	0,065765	0,759308	0,6279808
CG5316-RC	CG5316	3,01758	2,92353	3,2526	0,0291618	0,0394998	0,0297695	0,064616	0,763408	0,6279808
CG5317-RA	RpL7-like	65,517	8,92666	2,96584	5,99097	6,77973	8,70193	-0,329161	0,139271	0,13772387
CG5317-RB	RpL7-like	0,0379383	0,0289962	6,21176	0,0346703	4,28337	0,0160516	-0,340545	0,12625	0,6279808
CG5319-RA	lute	36,3183	55,3716	1,43014	0,152696	24,2893	0	0,466416	0,08401	0,6279808
CG5320-RA	Gdh	0,0370657	0,011376	0,0119903	2,13316	0,0326027	0,0130826	-0,01877	0,930576	0,6279808
CG5320-RB	Gdh	107,359	35,261	35,6685	2,50456	2,31781	13,2192	0,063309	0,770865	0,6279808
CG5320-RE	Gdh	38,1574	4,979	0,00930476	74,918	0,0550413	2,82744	0,065677	0,761645	0,6279808
CG5320-RF	Gdh	90,1717	68,5145	74,0619	0,0380542	0,0168508	0,031676	-0,01925	0,928776	0,6279808
CG5321-RB	CG5321	7,2472	13,9948	9,33477	2,7523	7,48831	3,25475	-0,288353	0,224968	0,6279808
CG5322-RA	LManI	3,0224	20,2639	22,5414	21,4526	24,7271	19,3057	-0,501874	0,069487	0,6279808
CG5323-RA	CG5323	36,2464	5,79103	6,6276	0,0225077	2,10146	6,13282	-0,228681	0,427836	0,6279808
CG5325-RA	Pex19	0,0922848	10,4151	2,83967	65,0006	2,81364	1,89089	-0,345925	0,243037	0,6279808
CG5325-RB	Pex19	54,6696	12,604	0,0885989	19,9811	0,173802	0,374789	-0,683336	0,023397	0,6279808
CG5326-RA	CG5326	128,181	121,605	158,798	95,1029	111,725	113,386	0,1879	0,44131	0,6279808
CG5326-RB	CG5326	0,103297	0,0940901	0,0991713	0,13433	0,181951	0,137129	-0,300841	0,252003	0,6279808
CG5327-RA	CG5327	0,0317113	7,30518	5,56785	0,0233947	4,1241	5,89623	-0,097319	0,72822	0,6279808
CG5330-RA	Nap1	77,3991	68,2157	125,452	99,2484	124,895	123,908	-0,377929	0,169232	0,6279808
CG5333-RA	Dsim\trus	6,8045	6,49695	9,55753	4,23204	6,58376	6,73339	0,054179	0,807454	0,6279808
CG5334-RA	CG5334	0	11,453	0	17,2667	11,3984	0	-0,041811	0,731452	0,6279808
CG5335-RA	CG5335	35,4901	10,3039	0,0254358	0,0201348	26,2304	0,0566699	-0,61683	0,049255	0,6279808
CG5336-RA	Ced-12	11,866	0,0840593	1109,47	1,77977	2,3495	3,01075	0,150278	0,473834	0,6279808
CG5337-RA	TBC1D16	3,40068	3,94522	4,04798	32,5739	3,8041	0,173158	-0,11126	0,603881	0,6279808
CG5338-RB	RpS19b	4,81393	27,2002	13,2436	12,4233	2,14891	0,504331	-0,667461	0,023278	0,6279808
CG5339-RA	CG5339	10,2502	101,256	0,0387597	0,0442963	0,0599996	14,2025	-0,28758	0,288398	0,6279808

gene_id	Symbol	W1_FPKM	W2_FPKM	W3_FPKM	MCM51_FPKM	MCM52_FPKM	MCM53_FPKM	FC	p-value	p-adj
CG5341-RA	Sec6	3,88843	23,2004	43,0195	38,8035	3,3259	49,9498	-0,015736	0,943973	0,6279808
CG5342-RA	CG5342	0,0410579	0	0,0394181	0,0719896	21,5293	0,0456234	-0,062968	0,745795	0,6279808
CG5343-RA	Bug22	12,0488	3,44908	21,4161	16,5146	18,4564	13,4412	0,17548	0,493535	0,6279808
CG5344-RA	wkd	19,6048	21,3054	19,8258	22,6747	17,052	15,4823	0,070586	0,719644	0,6279808
CG5344-RB	wkd	0,0285919	0,0260435	0,0274499	0,0312502	0,0423286	0,0319014	0,085566	0,66218	0,6279808
CG5345-RA	Eip55E	20,1214	0	0,292066	0	24,9883	0	-0,115976	0,705746	0,6279808
CG5346-RA	CG5346	20,1765	46,8574	22,0908	14,7856	23,7448	4,08512	0,035724	0,903313	0,6279808
CG5347-RA	CG5347	0	0,0430005	0,0453227	10,3962	0,0713708	0,0537894	0,044335	0,715924	0,6279808
CG5348-RA	CG5348	0,0544581	0,815535	6,19513	1,59793	2,30047	0,0205922	-0,024982	0,935207	0,6279808
CG5352-RA	SmB	36,1802	26,3372	58,5597	57,1072	5,02575	71,9978	-0,591477	0,040293	0,6279808
CG5353-RA	ThrRS	31,2752	0,0247511	0,110388	0,15485	21,8409	22,6446	-0,324436	0,076788	0,6279808
CG5353-RB	ThrRS	0,0271731	20,886	0,601159	0,56446	0,209745	0,158076	-0,323663	0,076803	0,6279808
CG5353-RC	ThrRS	8,16936	54,8981	49,8565	6,74306	1,9354	1,45864	-0,323287	0,077109	0,6279808
CG5354-RA	pie	0,414217	12,2951	21,9802	6,36197	10,2487	11,1647	-0,15012	0,674542	0,6279808
CG5355-RA	CG5355	12,7513	0,31693	0,397673	0,906458	0,0747276	0,403308	0,152123	0,528034	0,6279808
CG5357-RA	CG43795	0,639201	0,0863508	0,104051	0,0947372	0,193804	0,535823	0,104761	0,735467	0,6279808
CG5358-RA	Art4	3,8724	0,0237359	0,480582	0,0204219	13,2537	1,95203	0,043345	0,878395	0,6279808
CG5359-RA	CG5359	5,07351	4,06574	6,36141	5,30018	0,174141	0,131243	0,354143	0,153773	0,6279808
CG5359-RB	CG5359	0,0998625	0,0909617	0,095874	0,128564	2,57472	3,44032	0,064552	0,831438	0,6279808
CG5360-RA	mi	5,15145	4,87999	5,92659	9,14896	0,0847166	4,53901	0,085524	0,760139	0,13772387
CG5361-RA	Alp13	0	0	0	0,0195378	7,56459	4,62183	-0,492534	0,051462	0,6279808
CG5362-RA	Mdh1	60,7139	62,3233	122,745	86,7097	162,964	149,079	-0,597338	0,056506	0,6279808
CG5363-RA	Cdk1	11,5312	9,22526	25,5387	19,9232	10,9401	14,645	0,03123	0,916754	0,6279808
CG5366-RA	Cand1	6,60274	13,7974	7,72011	19,7514	35,0218	7,42194	-0,044507	0,837628	0,6279808
CG5367-RA	CG5367	0,422314	10,339	2,0342	14,3994	1,67348	1,48965	0,132458	0,71069	0,13772387
CG5370-RA	Dcp-1	13,0198	13,2026	0,0220522	16,3066	0,0327223	0,0432982	0,176721	0,454574	0,6279808
CG5371-RA	RnrL	7,74405	9,60739	10,4521	14,3366	0,0724719	0,0552473	-0,032153	0,904436	0,6279808
CG5372-RA	ItgaPS5	0,13202	18,0108	39,4893	24,0002	0,064139	48,398	-0,12872	0,661509	0,6279808
CG5373-RA	Pi3K59F	4,23938	12,4292	0,8496	0,339083	4,85369	4,86567	-0,212236	0,315714	0,6279808
CG5374-RA	CCT1	0,0340831	9,98354	25,8694	65,6836	24,0403	22,7493	-0,584032	0,015657	0,6279808
CG5374-RB	CCT1	55,5664	44,1545	15,9355	5,23079	43,4844	52,9566	-0,584032	0,015657	0,6279808
CG5375-RA	Schip1	4,29233	3,95822	5,80671	4,72693	31,6928	0,038229	-0,16486	0,563589	0,6279808
CG5375-RB	Schip1	1,05587	0,0315332	0,0332361	1,06279	6,88186	31,4612	-0,160693	0,572504	0,6279808
CG5376-RA	CG5376	23,6274	0,323583	0,284215	0,278008	22,1018	0	0,044603	0,87156	0,6279808
CG5377-RA	CG5377	1,82946	1,59288	17,4392	0,0289567	18,8057	0,0295601	-0,567872	0,071168	0,6279808
CG5378-RA	Rpn7	26,6835	8,55393	33,4137	2,72203	11,783	8,78482	0,040295	0,852406	0,6279808
CG5379-RA	CAH8	0,72254	0,15043	93,9685	0,966454	163,342	1,20665	0,105188	0,767203	0,6279808
CG5380-RA	CG5380	4,36411	4,26959	5,98987	4,29812	3,75005	4,7925	0,19845	0,463181	0,6279808
CG5381-RA	CG5381	36,7569	14,3006	13,3839	16,1757	73,1787	65,7492	0,087116	0,699177	0,6279808
CG5382-RB	CG5382	24,7856	34,5175	0,80091	1,27547	2,57058	0,440769	-0,434226	0,081778	0,6279808
CG5383-RA	PSR	11,0314	15,098	20,1533	21,7	17,02	20,4123	-0,411625	0,086496	0,6279808
CG5384-RA	Usp14	18,9117	19,3198	27,726	28,7943	36,9607	0,0546192	-0,22522	0,28421	0,6279808
CG5385-RA	l(2)SH0834	44,6663	39,2321	44,8606	63,0397	2186,04	24,3951	-0,362653	0,098993	0,6279808
CG5386-RA	CG5386	0,215934	15,1163	0,632838	17,0053	3,94557	6,97373	-0,200669	0,531566	0,6279808
CG5387-RA	Cdk5alpha	40,9765	39,7125	40,585	0,0456149	20,829	56,1066	0,036017	0,850371	0,6279808
CG5388-RA	CG5388	0,15319	3,85122	12,8894	25,3084	6,64699	0,293715	0,01562	0,897983	0,6279808
CG5389-RA	Dpse\GA18845	0,0972658	0,0442982	0,093381	0,195626	0,733277	0,843907	-0,076517	0,804322	0,6279808
CG5390-RA	CG5390	57,0826	62,7317	85,8899	63,5778	57,8937	0,0358646	-0,160674	0,535024	0,6279808
CG5391-RA	CG5391	5,47125	0	0	0,206622	0,258487	23,6134	0,491127	0,164192	0,6279808
CG5392-RA	Reck	2,75009	1,79955	19,3449	14,4792	1,45901	45,063	0,282142	0,241372	0,6279808
CG5393-RA	apt	6,5576	1,20354	15,1611	0,0256037	12,5261	0,0261372	0,260764	0,225116	0,6279808
CG5393-RB	apt	0,0225515	5,74772	0,0753406	0,0241581	0,105998	0,0246615	0,299119	0,168882	0,6279808
CG5393-RC	apt	0,0224519	10,0028	709,003	0,0240435	833,708	0,0245445	0,29821	0,169874	0,6279808
CG5393-RD	apt	0,0223456	0,0209223	0,0372573	0,0239213	0,0574506	0,0244197	0,269993	0,208897	0,6279808
CG5393-RE	apt	16,6567	0,0208282	0,0260521	26,2705	0,0399775	16,08	0,299935	0,167882	0,6279808
CG5394-RA	GluProRS	22,535	20,4004	22,4934	26,9921	8,96169	14,4946	0,008368	0,974607	0,6279808
CG5394-RB	GluProRS	3,85734	2,9691	4,35401	10,1618	3,71948	3,97316	0,097293	0,711669	0,6279808
CG5395-RA	nmd	24,8421	0,135793	0,119272	0,0455204	0,209745	12,6814	0,02213	0,920533	0,6279808
CG5397-RA	CG5397	10,3164	15,8537	7,4283	2,74597	0	0,0867479	1,404838	4,73E-06	0,6279808
CG5398-RA	Eglp1	0	0	2,34524	0	3,69205	0,302922	0,021164	0,919479	0,6279808
CG5399-RA	CG5399	185,456	0	0,0524415	0,0473523	0,410283	0	-0,415994	0,212074	0,6279808
CG5400-RA	Eh	2,38046	2,36541	3,11644	0,00831596	0,23487	5,1336	-0,602476	0,057206	0,6279808
CG5402-RA	CG5402	70,5492	92,8816	0,0426323	4,89789	60,9485	7,96415	0,577392	0,012199	0,6279808
CG5403-RA	retn	1,46899	15,6204	0,014924	0,0160652	0,0217604	0,0163999	0,343219	0,17493	0,6279808
CG5403-RB	retn	5,29616	0,0714804	10,027	8,35362	3,44833	4,698	0,343219	0,17493	0,13772387
CG5404-RA	CG5404	2,63774	0	0,0652642	0,0266388	0,0369488	0,0271939	-0,017215	0,955889	0,6279808
CG5405-RA	KrT95D	2,90898	5,03086	2,47452	56,8257	2,26314	4,85684	-0,299671	0,123115	0,6279808
CG5405-RB	KrT95D	0,016892	0,0219525	0,023138	71,4667	6,85533	8,11772	-0,295052	0,130224	0,6279808
CG5405-RC	KrT95D	6,56231	9,38114	7,89231	220,178	10,604	6,81199	-0,304414	0,116858	0,6279808
CG5407-RA	Sur-8	13,1272	0	0,253747	0,397168	19,5553	0	-0,170051	0,445449	0,6279808
CG5407-RB	Sur-8	0,0145831	13,2014	10,9062	0,370712	0	9,94547	-0,169261	0,448014	0,6279808
CG5408-RA	trbl	31,7003	40,3866	0,0362772	42,8007	7,66888	6,32238	-0,235351	0,275044	0,6279808
CG5409-RA	Arp53D	0	0	0,718059	0	0	0	0,01562	0,897983	0,6279808
CG5410-RD	Miro	7,03593	2,71296	4,48922	0,0475573	4,78405	0,0172983	-0,244933	0,246376	0,6279808
CG5410-RE	Miro	6,26139	3,46963	3,94892	0,0403372	3,40965	0,0259457	-0,241627	0,251077	0,6279808
CG5411-RA	Pde8	0,0137754	0,0378372	0,0398806	0,075954	0,0619143	0,0759606	-0,062812	0,78306	0,6279808
CG5411-RB	Pde8	12,301	1,40424	2,09901	174,999	33,8347	99,9987	-0,028996	0,898622	0,6279808
CG5411-RC	Pde8	0,015087	0,922351	0,90411	0,153765	6,41463	60,8223	0,095544	0,672323	0,6279808
CG5411-RD	Pde8	0,0167719	10,2574	10,1979	3,23636	9,29418	0,0775367	-0,032614	0,886423	0,6279808
CG5411-RE	Pde8	5,05083	3,81416	3,94835	1,06284	0,0511313	148,592	0,078169	0,724814	0,6279808
CG5412-RA	CG5412	13,7159	19,2344	60,9098	0,0699101	8,92711	55,0969	-0,245399	0,361631	0,6279808
CG5413-RA	CREG	0,0884943	133,096	0,249573	6,08024	0,0383293	20,7882	0,617104	0,015585	0,6279808
CG5413-RB	CREG	0,0767727	0,0174622	0,0849598	0	0,0678444	0,0198332	0,615202	0,015957	0,6279808
CG5413-RC	CREG	23,3115	0,0169699	14,815	0	0,0312555	69,8601	0,625115	0,013987	0,6279808
CG5414-RA	IleRS-m	3,4484	3,17482	110,993	145,553	3,12136	122,751	0,247783	0,251696	0,6279808
CG5417-RB	Srp14	41,2604	45,2748	53,9071	51,2409	11,4518	0,0302651	0,291803	0,222347	0,6279808
CG5418-RA	CG5418	7,36239	15,4136	0,197599	0,0508612	3,55075	0,0429938	0,137207	0,655761	0,6279808
CG5421-RA	CG5421	0	0,103059	5,53556	7,12259	0,188417	0	0,044335	0,715924	0,6279808
CG5422-RB	Rox8	0,0215605	0,0193029	14,0747	0,0226003	0,0306123	0,0230712	0,11245	0,657052	0,6279808
CG5422-RC	Rox8	17,6517	17,3681	0,0310711	19,9686	7,09018	12,8148	0,111481	0,65999	0,6279808
CG5422-RD	Rox8	10,022	7,46897	9,88342	21,338	9,62306	10,8256	0,113452	0,654858	0,6279808
CG5422-RF	Rox8	0,0209499	0,0194108	12,9407	0,0223247	0,0302389	0,0227898	0,123976	0,624373	0,6279808
CG5423-RA	robo3	6,98445	9,80176	0	0	0,344579	0	0,041528	0,887421	0,6279808
CG5424-RA	f	0,0121851	0,00960269	3,03975	0,0124948	1,88923	2,50754	0,558412	0,080604	0,6279808
CG5424-RB	f	1,17547	4,98424	0,0114638	2,26644	0,0165754	0,0124922	0,368266	0,245458	0,6279808
CG5424-RC	f	3,28721	0,0108764	0,0255108	7,16421	0,0381818	0,0287761	0,377093	0,23328	0,6279808
CG5424-RD	f	0,0119407	5,67576	9,53316	0,0122372	2,41797	4,58446	0,371066	0,241831	0,6279808
CG5424-RF	f	3,30822	14,0361	3,2157	0,0281887	2,34631	2,09611	0,35409	0,26362	0,6279808
CG5424-RG	f	12,7616	3,72679	2,64614	11,251	1,97321	2,06017	0,616835	0,051908	0,6279808
CG5427-RA	Oatp33Ea	11,7576	5,11415	0,0163891	0,0177056	0,117664	0	-0,468521	0,13503	0,6279808
CG5428-RB	St1	26,1562	36,8112	30,9024	27,5809	9,22302	17,0884	0,721858	0,010148	0,6279808
CG5429-RA	Atg6	12,8491	16,1319	0,0203453	17,911	16,1148	15,8518	-0,259796	0,235489	0,6279808
CG5430-RA	a5	0	0	0	0	3,69366	0	NA	NA	0,6279808
CG5431-RA	St3	56,8853	0,015277	0,0161021	0,0173833	0,75224	1,44207	-0,024187	0,918983	0,6279808

gene_id	Symbol	W1_FPKM	W2_FPKM	W3_FPKM	MCM51_FPKM	MCM52_FPKM	MCM53_FPKM	FC	p-value	p-adj
CG5431-RB	St3	0,0617532	7,06146	5,81892	4,95042	0,434194	0,64668	-0,018347	0,939003	0,6279808
CG5432-RA	Ald2	0	0,891191	0	0,114248	9,82683	0	-0,013096	0,914398	0,6279808
CG5433-RA	Klc	54,2473	53,4973	0	56,1251	41,1161	43,2852	0,148359	0,408155	0,6279808
CG5434-RA	Srp72	21,7172	41,543	4,49806	15,9787	2,71426	10,4209	0,064266	0,752243	0,6279808
CG5435-RA	CG5435	0,0753716	0,0686536	0,0723612	0,0905702	3,0805	3,24983	0,0027	0,9924	0,6279808
CG5436-RA	Hsp68	87,3094	121,485	92,6172	10,1454	175,084	71,3006	-0,735539	0,023284	0,6279808
CG5439-RA	CG5439	6,65513	0,0127307	1,83829	0,451623	1,48232	0,0128453	-0,431205	0,073315	0,6279808
CG5440-RA	CG5440	0	0	0	0,337882	0	0	-0,013096	0,914398	0,6279808
CG5441-RA	tx	22,5154	0,226265	23,9291	1079,48	13,7314	1373,53	0,370611	0,143242	0,6279808
CG5442-RA	SC35	25,353	20,4604	0,0668693	0,082414	0,11163	0,0841313	-0,262499	0,26868	0,6279808
CG5442-RB	SC35	40,2858	45,4149	85,3845	80,3002	72,5476	93,3009	-0,273368	0,257429	0,6279808
CG5444-RA	Taf4	0,0193223	0,0176001	0,0185506	0,0201472	0,0272894	0,0190686	0,107526	0,67617	0,6279808
CG5444-RB	Taf4	0,0179717	0,0163698	0,0172539	0,0186793	0,0253013	11,6879	0,107561	0,676174	0,6279808
CG5444-RC	Taf4	17,4258	20,3696	15,8884	21,2794	8,80905	0,0137964	-0,156294	0,555277	0,6279808
CG5444-RD	Taf4	0,0131503	0,0119782	0,0126251	4,12723	1,59381	0,0150463	-0,152145	0,566487	0,6279808
CG5444-RE	Taf4	0,0143032	0,0130283	0,0137319	0,0147391	0,0199643	100,65	0,104702	0,684721	0,6279808
CG5445-RA	CG5445	8,04362	0,0168511	5,7754	0,6918	0,188351	0,477197	-0,033684	0,876165	0,6279808
CG5445-RB	CG5445	17,0361	25,7753	21,5445	1,27358	4,42735	0,360932	-0,019894	0,927161	0,6279808
CG5445-RC	CG5445	0,040973	0,0187108	0,0393365	0,0373214	0,0260775	0,848564	-0,019894	0,927161	0,6279808
CG5445-RD	CG5445	0,0350225	26,4854	0,0336237	6,65947	0,937047	6,83319	-0,033684	0,876165	0,6279808
CG5446-RA	Dsim\GD22122	35,6665	38,4029	56,3113	52,4589	42,9723	52,9155	0,053387	0,824247	0,6279808
CG5447-RA	CG5447	30,0108	32,1397	29,6069	6,99161	0,310415	10,8537	-0,206536	0,328627	0,6279808
CG5450-RA	Cdlc2	0	0,0983972	0,0997434	0	4,83636	0	-0,128494	0,542395	0,6279808
CG5450-RB	Cdlc2	0	0,323776	0,210753	0,142462	0	4,44047	-0,128494	0,542395	0,6279808
CG5451-RA	Smu1	7,34567	5,20229	10,6459	12,9845	10,4318	77,5838	0,015214	0,953149	0,13772387
CG5452-RA	dnk	20,6454	35,2173	46,1816	33,6833	54,7465	59,1762	-0,033827	0,878156	0,6279808
CG5454-RA	snRNP-U1-C	43,9552	25,2106	30,1134	60,001	86,3514	24,7682	-0,280065	0,215927	0,6279808
CG5455-RA	CG5455	0,0232935	0,0217071	0,0223631	2,98503	0,0332093	0,0250285	-0,291415	0,155811	0,6279808
CG5455-RB	CG5455	0,0238312	15,3328	0,0228793	0,0251157	0,0340194	0,0256391	-0,288988	0,15989	0,6279808
CG5455-RC	CG5455	14,9121	6,44572	15,4116	15,9634	16,273	16,832	-0,290308	0,157642	0,6279808
CG5458-RA	CG5458	2,06543	0,0300531	0,0521826	28,1702	0	13,4953	0,243322	0,397518	0,6279808
CG5460-RA	H	0,0172717	0,0157323	7,28219	5,8073	7,73468	8,38766	0,33003	0,209704	0,6279808
CG5460-RB	H	0,0157448	0,0143415	5,94066	2,69043	0,0217022	4,71263	-0,070495	0,804608	0,6279808
CG5460-RC	H	0,0111133	1,45261	0,0165819	10,5743	4,39935	0,0182958	0,33003	0,209704	0,6279808
CG5460-RD	H	7,08036	6,36199	0,015116	7,97783	4,66315	0,0166187	-0,073564	0,795622	0,6279808
CG5462-RA	scrib	4,01715	5,70582	4,53648	5,91101	0,0495694	0,0373585	-0,582867	0,007774	0,6279808
CG5462-RB	scrib	0,0109403	0,0099652	0,0105034	0,0111861	423,114	417,962	-0,583592	0,00769	0,6279808
CG5462-RC	scrib	4,13879	4,91333	2,67768	6,66136	0,0490248	0,0369481	-0,623014	0,004767	0,13772387
CG5462-RD	scrib	0,00930559	0,00847617	0,00893392	0,00947874	0,0568287	0,0428296	-0,558771	0,01019	0,6279808
CG5462-RH	scrib	2,13821	3,04369	3,86083	0,00910544	160,13	176,326	-0,63124	0,004065	0,6279808
CG5462-RI	scrib	3,72551	4,71364	3,62571	15,3067	0,0603241	0,0454638	-0,672981	0,002306	0,6279808
CG5463-RA	GatB	4,43137	3,60144	5,46468	7,24607	2,72299	5,77146	-0,214544	0,319963	0,6279808
CG5465-RA	MED16	1,88823	0,0363737	0,273434	3,01475	1,46776	2,56457	-0,283746	0,313816	0,6279808
CG5466-RA	CG5466	3,33937	3,5121	3,0848	3,09147	1,90954	1,74202	0,455322	0,055516	0,6279808
CG5467-RA	scrib	40,9582	55,5661	28,8029	42,5679	22,6582	23,7577	0,343073	0,208763	0,6279808
CG5468-RA	TwdlM	1476,82	628,657	2099,32	1847,95	2163,38	2229,71	-0,009256	0,972133	0,6279808
CG5469-RC	Gint3	9,57457	0,0175898	1,02153	0,0299573	0,0405773	0,0287269	0,097192	0,634737	0,6279808
CG5471-RB	TwdlJ	11,8136	13,1316	25,7592	11,6957	20,1389	19,628	-0,08943	0,799939	0,6279808
CG5472-RA	Pal2	0,0343132	0,0125476	0,0132252	0,0141778	0,0235458	5,13587	-0,067229	0,788825	0,6279808
CG5472-RB	Pal2	11,5136	14,5541	10,4308	10,7069	0,0335085	1,08584	-0,067229	0,788825	0,6279808
CG5472-RC	Pal2	0,0415397	0,0137422	0,0144844	3,83205	2,22737	2,18844	-0,233429	0,392216	0,6279808
CG5473-RA	Ntan1	6,26281	0,0202944	8,26896	0,33693	9,1846	6,46178	-0,073361	0,721444	0,6279808
CG5473-RB	Ntan1	5,30313	1224,29	2,46931	33,4354	2,48227	2,78359	-0,087131	0,672073	0,6279808
CG5474-RA	SsRbeta	259,543	257,572	4,55932	0	0	1,08603	0,202631	0,326769	0,6279808
CG5475-RA	p38a	16,8295	12,9983	21,6413	44,9339	11,1248	12,9879	0,215407	0,427233	0,6279808
CG5475-RB	p38a	0,0403475	0,0367513	0,038736	0,195803	5,0016	0,045189	0,215838	0,426393	0,6279808
CG5476-RA	TwdlN	987,062	1,43005	3,52427	3,97654	180,09	3,86876	-0,82647	0,00852	0,6279808
CG5478-RA	CG5478	0,0673098	156,079	5,83808	7,30128	7,2678	10,9434	0,830136	0,010044	0,6279808
CG5479-RA	mRpL43	29,0141	29,7846	49,4482	5,26492	61,5529	44,3195	0,075331	0,769899	0,6279808
CG5480-RA	Tb	61,3102	30,0525	8,34276	1,21517	1,8022	0	-0,031996	0,912598	0,6279808
CG5481-RA	robo2	22,4426	0,46512	18,2987	3,28263	0	0,199174	0,407114	0,209452	0,6279808
CG5482-RA	zda	42,553	0,0256258	12,0596	336,969	0	34,2022	0,239521	0,226394	0,6279808
CG5483-RB	Lrrk	1,68386	1,67731	1,64254	0,0179223	0,0220507	1,48854	-0,32781	0,148161	0,6279808
CG5484-RA	Yif1	17,6498	0	4,58529	9,58102	3,64816	6,9051	0,224125	0,27976	0,6279808
CG5484-RB	Yif1	0,0447163	4,68644	0	2,96387	9,3822	104,033	0,223718	0,280428	0,6279808
CG5484-RC	Yif1	3,36429	0,458982	26,1855	6,84914	13,9944	16,8222	0,223586	0,280996	0,6279808
CG5485-RA	Prestin	10,4346	2,85459	15,8236	11,775	187,649	8,80283	0,128625	0,589181	0,6279808
CG5486-RA	Usp47	12,1688	27,8447	0,0130753	0,0328388	0,0248435	0,014304	-0,106001	0,729649	0,6279808
CG5486-RB	Usp47	7,10774	8,73612	7,01954	394,074	0,0364807	6,42512	-0,10172	0,740414	0,6279808
CG5486-RC	Usp47	0,0135912	6,93457	10,4583	9,99522	78,2616	8,54588	-0,102051	0,739431	0,6279808
CG5488-RA	B-H2	3,25139	3,93579	0,0116984	4,19818	0,0169243	0,0127552	0,115956	0,673813	0,6279808
CG5489-RA	Atg7	4,12598	17,3377	22,499	0,0228195	47,2607	0	0,079578	0,722728	0,6279808
CG5489-RB	Atg7	3,00132	11,2102	0,0538919	0,0206847	0,0461358	0,0186242	0,169794	0,44249	0,6279808
CG5490-RA	Tl	29,361	4,37097	919,637	0,0123984	0,0167937	1,97261	0,321322	0,191181	0,6279808
CG5490-RB	Tl	0,0120937	170,407	290,554	293,379	170,743	256,96	0,320206	0,192456	0,6279808
CG5491-RA	IntS12	2,73904	2,70068	0,0317872	2,0647	2,90808	538,754	-0,217302	0,416923	0,13772387
CG5492-RA	Tsp74F	55,7347	82,4659	48,9228	28,5371	27,8761	30,6867	0,818721	0,002422	0,6279808
CG5492-RB	Tsp74F	30,8288	45,9227	16,8291	8,09605	12,8006	12,4762	0,809689	0,002716	0,6279808
CG5493-RA	CG5493	14,3029	0,891652	0,422912	876,302	67,0612	14,6979	-0,119804	0,669835	0,6279808
CG5494-RA	Cpr92F	74,2239	2,95668	66,3981	64,1997	68,0764	51,9302	0,192388	0,431697	0,6279808
CG5495-RA	Txl	32,7796	6,54609	42,4106	2,00533	0,0254964	38,2994	-0,062077	0,808057	0,6279808
CG5497-RA	mRpS28	20,2061	1,85235	32,3512	18,3944	8,3904	2,98876	-0,498816	0,086927	0,6279808
CG5498-RB	CG5498	15,0073	13,1748	17,9474	16,1793	11,3607	11,4184	0,365715	0,095194	0,6279808
CG5499-RA	His2Av	183,488	1223,62	0,171451	1666,62	0	0	-0,037959	0,875945	0,6279808
CG5500-RA	CG5500	15,94	17,6481	19,2311	17,7826	5,73613	0,0647697	-0,188072	0,475444	0,6279808
CG5501-RB	Myo95E	4,98923	24,5967	4,28704	30,2183	0,0175603	3,3829	0,226037	0,283967	0,6279808
CG5501-RD	Myo95E	0,0125915	0,054092	0,0120886	0,0683864	0,0174573	0,013193	0,224812	0,286153	0,6279808
CG5501-RE	Myo95E	0,01263	16,7349	0,0121255	24,2196	0,0218885	0,0132345	0,224812	0,286153	0,6279808
CG5501-RH	Myo95E	0,012558	5,30681	0,0120564	4,74259	22,4503	0,0131569	0,224812	0,286153	0,6279808
CG5501-RI	Myo95E	0,0156331	0,0114692	0,0150087	0,0129237	0,232714	0,0164965	0,020593	0,932254	0,13772387
CG5502-RA	RpL4	796,684	3,23351	2,57504	3,47609	0	104,865	-0,398573	0,080443	0,6279808
CG5504-RA	CG5504	5,84603	1,8668	9,70905	12,0816	1,01584	6,10279	-0,23865	0,41616	0,6279808
CG5504-RB	CG5504	9,9864	7,74476	14,1812	21,9068	7,44701	14,9153	-0,23865	0,41616	0,6279808
CG5504-RC	CG5504	0,0334279	2,72514	3,53021	5,51704	13,0222	3,16237	-0,237748	0,417871	0,6279808
CG5505-RA	scny	4,59435	3,91757	7,89606	14,3144	4,62374	4,33567	0,000753	0,997392	0,6279808
CG5505-RB	scny	0,0161884	0,0147456	0,0155419	9,59865	2,43464	0,0171047	0,017571	0,938715	0,6279808
CG5505-RC	scny	0,0190879	0,0173866	0,0183255	0,0167556	8,88124	0,0203062	0,021157	0,92637	0,6279808
CG5505-RD	scny	0,0183408	0,0167061	0,0176083	0,0198917	4,32141	0,0194771	-0,001825	0,993684	0,6279808
CG5505-RE	scny	11,6967	11,1302	13,909	0,0190796	0,0226955	10,3008	0,020178	0,92961	0,6279808
CG5505-RF	scny	0,0200129	0,0182291	0,0192136	13,9946	1,73721	0,0213368	0,023751	0,917328	0,6279808
CG5506-RA	CG5506	12,2187	1,83838	47,7607	38,0665	0,0308663	121,626	-0,539359	0,076283	0,6279808
CG5507-RA	T48	12,7702	15,011	11,5055	15,2717	0,585538	10,6168	0,106942	0,630288	0,6279808
CG5508-RA	mino	2,76854	1,66189	3,87801	4,03452	7,19144	3,08055	-0,243602	0,308339	0,13772387
CG5508-RB	mino	0,0220351	0,0200711	0,021155	0,0231238	1,04844	0,0236056	-0,226553	0,34205	0,6279808

gene_id	Symbol	W1_FPKM	W2_FPKM	W3_FPKM	MCM51_FPKM	MCM52_FPKM	MCM53_FPKM	FC	p-value	p-adj
CG5508-RC	mino	5,53747	4,53326	6,96391	6,55512	2,65556	4,89964	-0,2256	0,343351	0,6279808
CG5509-RA	CG5509	0,212691	0	0,408391	28,636	10,9088	2,9637	0,06018	0,77461	0,6279808
CG5510-RA	CG5510	12,3861	8,86941	16,8562	14,4203	10,4513	75,7652	0,076841	0,750989	0,6279808
CG5514-RA	BOD1	4,79113	6,08943	6,13028	11,7484	3,64706	2,30709	0,024835	0,926115	0,6279808
CG5514-RB	BOD1	2,31895	3,9547	3,0226	1,91451	1,61762	8,91787	0,019572	0,941722	0,6279808
CG5515-RA	CG5515	32,2766	0,0114387	41,4993	0,0128883	37,2542	40,5617	-0,145598	0,596515	0,6279808
CG5515-RB	CG5515	0,0593851	0,0142397	0,0570132	0,0161597	0,0926298	0,0698114	-0,145598	0,596515	0,6279808
CG5516-RA	CG5516	8,31091	6,22102	11,3365	10,4654	2,84191	1,66409	-0,06036	0,820176	0,6279808
CG5517-RB	Ide	13,6258	10,9893	10,3519	8,45691	8,11733	9,31159	0,061683	0,746063	0,6279808
CG5518-RA	CG46339	21,3189	23,2623	0,543868	14,8284	0,395221	5,32443	0,251311	0,29622	0,6279808
CG5518-RB	CG46339	0,0128937	0,0117445	510,802	2,88739	3,85067	0,0256001	0,250549	0,297947	0,6279808
CG5519-RA	Prp19	12,394	14,9895	0,110774	0,0497851	2,23678	0,158823	-0,210343	0,447244	0,6279808
CG5520-RA	Gp93	88,3819	0,716506	104,191	1,53411	66,1013	80,848	-0,258524	0,305657	0,6279808
CG5521-RA	CG5521	4,41293	5,15977	6,63418	54,2368	3,28479	0,0291755	-0,210951	0,453177	0,13772387
CG5522-RA	CG5522	15,1622	0,0992085	0,00853793	19,5637	0,0123487	0,019839	0,267935	0,362601	0,6279808
CG5522-RB	CG5522	0,019328	7,21906	8,35778	0,0201533	8,92106	2,71657	0,279119	0,344009	0,6279808
CG5522-RC	CG5522	0,0199947	10,6879	0,123258	3,53972	0,244174	20,9528	0,278866	0,344647	0,6279808
CG5522-RD	CG5522	0,0193449	9,26022	1,11745	0,0201718	0,802191	81,4233	0,2692	0,360594	0,6279808
CG5522-RE	CG5522	0,0192716	13,5708	0,570317	0,0200919	1,17083	2,51277	0,277582	0,346616	0,6279808
CG5522-RF	CG5522	0,0186673	0,0525339	0,0871385	0,0194341	0,178336	7,53847	0,280407	0,342044	0,6279808
CG5524-RA	jnj	1,99589	43,8821	0,119092	3,57523	25,5073	22,6232	0,159624	0,495933	0,6279808
CG5525-RA	CCT4	47,5857	0,964765	4,38035	2,04539	9,15223	4,62441	-0,456607	0,029483	0,6279808
CG5526-RA	Dhc36C	0,210311	0,106487	0,1652	0,393097	12,512	37,419	0,176407	0,617588	0,6279808
CG5527-RA	Nep7	7,97079	7,87607	0,647843	1,57293	4,05939	0,214138	0,195051	0,406989	0,6279808
CG5528-RA	Toll-9	0,371213	20,4733	0,0367394	15,0331	10,1192	9,96024	0,054075	0,878545	0,6279808
CG5529-RA	B-H1	2,96447	0,0110991	0,986824	3,96311	0,593569	0,920768	0,164724	0,536615	0,6279808
CG5532-RA	CG5532	29,6164	29,3269	80,902	41,3639	3,91878	66,6301	-0,058505	0,858009	0,6279808
CG5535-RB	CG5535	0,0315599	0,0287469	1,51497	11,0507	6,68274	7,38489	0,00768	0,974085	0,6279808
CG5535-RC	CG5535	10,1646	7,0901	8,67257	97,9669	129,882	115,323	0,009626	0,967671	0,13772387
CG5537-RA	kri	24,8569	17,7529	0,265565	31,3964	0	11,7395	-0,31741	0,244504	0,6279808
CG5538-RA	vrs	0,0746339	0,0679817	0,310496	13,0352	0,118946	0,193158	-0,075058	0,800654	0,6279808
CG5539-RA	CG5539	0,0919423	0,569948	0,0209593	0,11289	0	0	0,187097	0,445427	0,13772387
CG5540-RA	Or98a	0	0,0185265	0,771317	34,1674	23,7679	2,16976	0,044335	0,715924	0,6279808
CG5541-RA	CG5541	30,8598	8,76855	71,35	11,3808	4,28445	4,09212	-0,22847	0,397423	0,6279808
CG5541-RB	CG5541	0,0197792	33,0715	37,4605	29,5613	37,9723	36,5696	-0,225991	0,402901	0,6279808
CG5543-RA	CG5543	3,08766	1,2541	9,49774	11,2261	7,66338	0,392996	0,112884	0,64912	0,6279808
CG5545-RA	Oli	18,6263	0	18,7961	0	0,0806069	0,0957601	0,128246	0,532172	0,6279808
CG5546-RA	MED19	19,2706	19,9999	23,7306	30,0329	13,3344	84,0827	-0,167777	0,391668	0,13772387
CG5547-RA	Pect	0,0297104	0,390399	8,51434	0,0125831	0,0170341	0,0801227	0,146888	0,517091	0,6279808
CG5547-RB	Pect	23,4044	0,110366	29,7904	0,0125831	0,0170439	0,163921	0,028277	0,895236	0,6279808
CG5547-RC	Pect	0,0304919	0,259425	0,029274	11,0959	0,0170439	1,8234	0,025897	0,904052	0,6279808
CG5547-RD	Pect	45,1383	23,2132	24,1913	2,09875	0,0170439	0	0,025829	0,904317	0,6279808
CG5548-RA	Dpse\GA18962	82,3139	69,0916	156,434	0,767361	221,377	233,76	-0,464476	0,15417	0,6279808
CG5549-RA	GlyT	0,32983	6,79596	0,844073	8,15919	0,051102	3,07158	-0,941116	0,005402	0,6279808
CG5550-RA	CG5550	14,3117	0,749209	0,00835244	0,162984	0,0120731	0,0205105	0,268819	0,432081	0,6279808
CG5553-RA	DNApol-alpha60	2,50058	2,81261	1,33886	4,4066	2,3903	13,7202	-0,136851	0,629806	0,6279808
CG5554-RA	CG5554	56,0698	0,316771	67,8516	85,2229	47,0338	61,8017	-0,050362	0,805459	0,6279808
CG5555-RA	CG5555	5,72035	5,74217	4,52422	0,426601	0,166351	4,18228	0,122046	0,566479	0,6279808
CG5556-RA	CG5556	0	0	0	0,44155	0	0	0,01562	0,897983	0,6279808
CG5557-RA	sqz	5,87273	6,92534	5,84413	6,24292	4,81313	3,29132	0,340547	0,203181	0,6279808
CG5559-RA	Sytalpha	2,15678	19,6022	8,74862	21,7781	0	18,6532	-0,125309	0,672725	0,6279808
CG5560-RA	dob	0,5687	0,45326	2,09864	105,223	0,912268	0,783351	0,174339	0,624269	0,6279808
CG5561-RA	CG5561	0,0605645	0,137916	0	0,106359	0,421192	0,108575	-0,095685	0,563966	0,6279808
CG5562-RA	gbb	18,154	16,3013	23,3654	26,6137	15,6473	21,4424	-0,195153	0,371638	0,6279808
CG5565-RA	CG5565	0,109761	0,166629	0	13,2304	0	0	0,084725	0,718334	0,6279808
CG5567-RA	CG5567	63,9262	0,4686	0,385488	36,3693	17,5335	0,983733	-0,249815	0,451556	0,6279808
CG5568-RA	CG5568	0,0864415	0,0787369	36,5795	0,629397	2,9151	0,0196421	-1,317404	0,000231	0,6279808
CG5569-RA	CG5569	10,4104	12,634	19,1219	14,1405	18,6499	10,505	0,019051	0,946196	0,6279808
CG5575-RA	ken	63,0822	0,0277229	47,0771	39,2059	8,73729	6,75194	0,620897	0,049195	0,6279808
CG5576-RA	imd	7,46559	8,26033	11,5206	6,76395	9,71035	10,3739	-0,314797	0,205391	0,6279808
CG5577-RA	CG5577	6,56166	0,0241519	0,0374479	14,5204	3,20825	0,0397076	-0,983398	0,004067	0,6279808
CG5580-RA	sbb	10,2106	11,9067	12,1375	5,87303	5,74576	6,23173	0,133968	0,61982	0,6279808
CG5580-RB	sbb	0,0218963	0,0199447	8,25662	22,5229	0,0311138	0,0234493	-0,089652	0,753218	0,6279808
CG5580-RC	sbb	0,021012	0,0191392	0,0210218	30,442	0,0297947	0,0224551	-0,152761	0,589251	0,6279808
CG5581-RA	Ote	3,11483	18,6388	20,0774	18,8011	19,1277	5,3178	-0,408817	0,170294	0,6279808
CG5582-RA	Cln3	6,85975	3,81518	5,71163	5,4234	0,0389823	2,68796	-0,593194	0,093566	0,6279808
CG5583-RA	Ets98B	3,93197	0,0299783	0,0315972	0,0354538	0,0480224	0,0361926	-0,229446	0,36988	0,6279808
CG5585-RA	Rbbp5	3,15241	72,2878	81,9251	62,6282	3,7007	66,4435	-0,025678	0,929372	0,6279808
CG5586-RC	Tusp	3,93678	5,05927	3,88591	9,7026	3,65818	2,44377	-0,029825	0,898668	0,6279808
CG5588-RA	Mtl	0,0496189	0,0451963	0,0476371	0,0194112	1,36249	0,0568825	0,369077	0,198122	0,6279808
CG5588-RB	Mtl	15,5241	14,6779	17,1048	36,0685	5,17063	9,72269	0,37093	0,198465	0,6279808
CG5588-RC	Mtl	0,0485613	0,044233	0,0466218	0,0201225	0,0736683	0,0555209	0,367747	0,198375	0,6279808
CG5589-RA	CG5589	5,26875	5,73339	48,6222	79,1507	39,3257	43,3613	-0,064276	0,837601	0,6279808
CG5590-RA	CG5590	33,9315	12,3144	55,0563	35,5174	46,5173	0,96513	-0,298549	0,317323	0,6279808
CG5591-RA	Lpt	3,04268	2,66504	3,50125	6,86927	2,38787	2,3954	-0,117289	0,631986	0,6279808
CG5592-RA	CG5592	0	1,57048	2,63352	0,0192411	69,1957	0,110538	-0,041811	0,731452	0,6279808
CG5594-RA	kcc	0,0175749	0,0160085	0,016873	0,0182499	0,0247196	0,0186302	-0,329393	0,130489	0,6279808
CG5594-RB	kcc	11,6024	9,65441	17,1161	22,0876	18,3287	15,9503	-0,317061	0,147706	0,6279808
CG5594-RC	kcc	0,0184124	0,0167713	0,017677	0,0191572	0,0259486	0,0195564	-0,313354	0,152641	0,6279808
CG5594-RD	kcc	25,0903	21,35	25,3029	22,3283	25,1232	22,1084	-0,325405	0,135449	0,6279808
CG5595-RA	Sce	9,88603	24,4168	16,3865	4,81742	9,10945	11,573	0,056349	0,807077	0,6279808
CG5596-RA	Mlc1	0,0776752	0,070752	0,0745729	68,168	105,355	96,3815	-0,208692	0,432598	0,6279808
CG5596-RB	Mlc1	1702,27	1740,25	1966,63	0,0617819	0,083684	0,0630693	-0,207152	0,436	0,6279808
CG5597-RA	CG5597	24,4819	15,8816	10,3971	76,9352	0,045339	0,0341702	-0,884051	0,007963	0,6279808
CG5599-RA	Dsim\GD17193	9,91848	12,1583	7,13984	53,513	132,04	37,9872	-0,114679	0,695874	0,6279808
CG5602-RA	DNAlig1	2,54784	0,0332032	3,56285	1,89409	0,112333	2,7595	0,190308	0,467722	0,6279808
CG5603-RA	CYLD	6,56089	8,34306	6,88273	47,4512	1,82164	5,7119	0,157786	0,416689	0,6279808
CG5603-RB	CYLD	7,21041	2,58729	5,71221	5,59856	7,53795	0,0465654	0,290633	0,139613	0,6279808
CG5603-RC	CYLD	0,0326354	0,0297266	0,0313319	5,58578	0,0475871	23,4364	0,278428	0,151209	0,6279808
CG5603-RD	CYLD	3,61669	5,4057	6,28159	0,0351325	1,58529	31,1559	0,279312	0,14987	0,6279808
CG5603-RE	CYLD	0,0414615	0,037766	0,0398055	5,61124	0,0617857	82,6152	0,27887	0,150537	0,6279808
CG5604-RA	Ufd4	17,8137	17,4883	19,4325	0,241644	0,211735	68,2875	-0,019493	0,934473	0,6279808
CG5605-RA	eRF1	0,0380037	114,656	22,1631	13,1526	21,9027	6,13642	-0,31531	0,095513	0,6279808
CG5605-RB	eRF1	0,0377864	0,0269572	5,84734	9,23289	6,94557	6,13931	-0,314068	0,096099	0,6279808
CG5605-RC	eRF1	0,0381794	1,81741	0,038823	20,4996	15,7597	15,7561	-0,314306	0,095867	0,6279808
CG5605-RE	eRF1	0,0338561	5,89534	0,041166	8,8294	7,80138	6,0368	-0,314985	0,095565	0,6279808
CG5605-RF	eRF1	114,201	7,36559	5,90338	0,0460291	0,0623468	0,0469883	-0,314198	0,095958	0,6279808
CG5605-RG	eRF1	0,029595	23,5592	0,0394834	0,0492272	0,0666786	0,050253	-0,314068	0,096099	0,6279808
CG5608-RA	CG5608	3,51875	2,42731	5,0037	28,7807	0,508028	20,0042	0,252138	0,364901	0,6279808
CG5609-RA	TotB	0	0,331119	1,02955	0,959141	0,237463	0,273497	NA	NA	0,6279808
CG5610-RA	nAChRalpha1	5,51426	6,71438	6,11692	5,97259	2,97674	0,151379	0,607471	0,024317	0,6279808
CG5611-RA	CG5611	13,4367	19,6222	13,2875	20,7232	8,35856	5,27007	0,519934	0,03081	0,6279808
CG5612-RA	CG5612	1,29518	1,3197	2,40259	1,42179	8,25987	4,92256	-0,321421	0,332846	0,6279808
CG5613-RA	CG5613	0,570804	1,05305	0,0197213	0,0504329	0,0683117	0,306234	0,209482	0,484877	0,6279808

gene_id	Symbol	W1_FPKM	W2_FPKM	W3_FPKM	MCM51_FPKM	MCM52_FPKM	MCM53_FPKM	FC	p-value	p-adj
CG5613-RB	CG5613	0,53491	4,0964	25,0191	0,546082	0,646655	0,0514838	0,231989	0,442718	0,6279808
CG5614-RA	CG5614	0	125,116	0,010729	0,0331194	38,1592	0	NA	NA	0,6279808
CG5618-RA	CG5618	7,89309	7,37878	19,1307	22,6208	1,01996	3,90142	-0,396315	0,040984	0,6279808
CG5618-RC	CG5618	1,70292	1,56856	10,1869	3,94189	10,6909	1,41096	-0,382609	0,048785	0,6279808
CG5619-RA	trk	0,0908049	0,801818	0,0871781	0,156319	4,18038	2,55067	0,040319	0,884989	0,6279808
CG5621-RA	Grik	0,539673	0,542424	0,375189	0,12554	0,0262385	0,0197749	1,871746	1,17E-07	0,6279808
CG5621-RB	Grik	0,0215324	0,735496	0,248069	0,0225693	0,0589363	0,149116	1,893207	9,53E-08	0,6279808
CG5623-RA	CG5623	0	0	4,199	0,052988	0,0841574	3,30267	NA	NA	0,6279808
CG5625-RA	Vps35	13,3289	0	0,0629333	9,12453	0,110799	5,8359	0,057055	0,783696	0,6279808
CG5625-RB	Vps35	0,0196421	1,95098	3,95599	4,98994	0,0594006	2,79282	0,04933	0,81243	0,6279808
CG5626-RA	CG5626	2,39034	22,4395	0,0449693	117,955	2,5126	2,21229	0,118016	0,699098	0,6279808
CG5627-RA	Rab3-GEF	3,72451	1,26111	0,0248902	1,01859	1,82253	1,0228	-0,021959	0,921872	0,6279808
CG5627-RB	Rab3-GEF	0,00821669	6,00066	0,0235941	3,33974	2,37809	2,02489	-0,019874	0,929237	0,6279808
CG5629-RA	Ppcs	5,81827	0,0280895	0	0,0424965	0	5,50062	-0,664688	0,003655	0,6279808
CG5629-RB	Ppcs	0,0308381	20,296	479,76	138,292	3,02313	0,0337417	-0,242124	0,234224	0,6279808
CG5630-RA	hdly	14,3831	8,68117	20,0201	15,599	31,2623	27,8481	-0,400307	0,208329	0,6279808
CG5630-RB	hdly	11,7575	7,53426	19,1498	9,028	13,0922	11,2199	-0,351176	0,266486	0,6279808
CG5632-RA	thoc6	4,44755	1,86625	0,066426	6,46149	6,63715	28,5578	0,066805	0,807822	0,6279808
CG5634-RA	dsd	8,1382	9,24119	6,85835	6,18605	2,43566	3,17808	0,137963	0,618259	0,6279808
CG5637-RA	nos	0,0281533	0,0791927	0,222585	0,227158	0	0,339801	-0,286857	0,243011	0,6279808
CG5638-RA	Rh7	0,320677	0,262136	20,2156	0,343404	42,2841	4,11209	-0,248454	0,446713	0,6279808
CG5639-RA	CG5639	13,6198	15,7411	15,5769	0,0557214	4,18303	9,77771	0,560882	0,084471	0,6279808
CG5640-RA	Utx	6,39687	18,3479	3,91974	14,9973	2,43082	2,02666	0,225588	0,49899	0,6279808
CG5640-RB	Utx	15,14	0,0170468	2,15608	20,9694	22,4138	0,0569788	0,23436	0,483779	0,6279808
CG5640-RC	Utx	0,0187148	61,0463	34,135	4,18475	74,2277	0,127211	0,23436	0,483779	0,6279808
CG5641-RA	CG5641	14,6341	12,8938	15,824	0,249075	13,5	0,334457	-0,047203	0,84647	0,6279808
CG5642-RA	eIF3l	58,9378	0,0426652	26,7919	0,0522319	2,66698	2,30723	-0,127419	0,531334	0,6279808
CG5643-RA	wdb	9,05517	13,2944	10,0534	0,0543876	6,50628	3,3684	-0,158334	0,547701	0,6279808
CG5643-RB	wdb	0,0199164	4,59879	0,0191209	19,2907	2,56731	3,46863	-0,159753	0,544513	0,6279808
CG5643-RC	wdb	0,0186462	0,0169842	0,0179014	18,1353	0,0262926	0,0198157	-0,151965	0,564064	0,6279808
CG5643-RE	wdb	11,4503	9,88416	14,6767	0,0219674	8,22944	12,3473	-0,153369	0,560342	0,6279808
CG5643-RG	wdb	0,0192997	3,67412	0,0185289	8,54342	2,62741	0,0205418	-0,160987	0,541524	0,6279808
CG5644-RA	CG5644	0,0398367	36,7478	12,164	20,7387	0,699169	35,0633	0,718899	0,007216	0,6279808
CG5645-RA	CG5645	6,77785	128,969	137,64	0,143392	5,05478	102,34	0,156998	0,60954	0,6279808
CG5646-RA	CG5646	0,314647	0,549321	0,553813	0,467308	0,694448	0,560791	-0,06293	0,860102	0,6279808
CG5648-RA	Prosalpha6T	0	0	0,093198	0,0125831	2,11391	0	0,01562	0,897983	0,6279808
CG5649-RA	kin17	16,9703	1,03059	0,832789	0,668037	0,320178	0,394342	0,236681	0,299869	0,6279808
CG5650-RA	Pp1-87B	107,198	115,432	139,813	8,2519	0,0536517	16,951	0,031115	0,873794	0,6279808
CG5651-RA	pix	90,2121	24,7907	0,0315658	2,81675	0,593936	0,0361537	-0,15844	0,432829	0,6279808
CG5651-RB	pix	40,587	0,0299485	0,0364858	0,85618	0,0385445	0,0423176	-0,158393	0,432974	0,6279808
CG5653-RA	CG5653	0	9,06491	16,5126	8,62574	11,6353	12,4406	-0,041811	0,731452	0,6279808
CG5654-RA	yps	112,763	8,30223	10,0681	7,37567	76,864	7,22164	0,165883	0,4299	0,6279808
CG5655-RA	Rsf1	53,8259	55,6348	73,7994	71,1302	49,1317	50,1005	0,26415	0,21819	0,6279808
CG5656-RA	Alp1	7,87059	4,5376	8,64077	14,8719	3,91178	0	-0,41426	0,196578	0,6279808
CG5657-RA	Scgbeta	10,939	15,0969	19,2049	0,0374274	0,739255	1,87118	-0,233121	0,351222	0,6279808
CG5658-RA	Klp98A	12,2481	13,6404	8,77158	13,2869	6,46742	6,77875	0,249919	0,339831	0,6279808
CG5659-RA	ari-1	7,18478	6,29721	6,15345	0,482291	0	0	-0,173429	0,354327	0,6279808
CG5659-RB	ari-1	0,0252326	0,0229836	1,34448	0	0	0	-0,170699	0,36192	0,6279808
CG5659-RC	ari-1	3,1085	4,48602	4,323	0	0	0,941849	-0,172418	0,357763	0,6279808
CG5660-RA	ValRS-m	1,93168	1,0221	0,822667	0,0307974	1,66222	0,790813	-0,931881	0,000348	0,6279808
CG5661-RA	Sema5c	8,08479	6,27605	9,46372	13,0453	5,09753	5,05099	0,157766	0,510665	0,6279808
CG5661-RB	Sema5c	11,3066	15,8894	8,54958	11,7489	0	15,8381	0,167172	0,486848	0,6279808
CG5662-RA	CG5662	0	0	0	0,21006	0	0,874958	-0,029574	0,84188	0,6279808
CG5663-RA	Dip-C	12,7095	1,40784	2,26972	0,0408625	16,8905	17,3322	-0,268237	0,37579	0,6279808
CG5664-RA	CG5664	2,67115	1,87428	2,83441	2,20315	1,54656	1,77235	0,37174	0,133567	0,6279808
CG5665-RA	CG5665	0,641147	0,0731084	4,69229	0,321569	0,696326	0,613778	0,42605	0,231644	0,6279808
CG5669-RA	Dsim\GD18309	0,0189167	0,0223575	0,040927	0,0567288	0,0662333	9,6394	0,300485	0,280661	0,6279808
CG5670-RA	Atpalpha	0,0184381	0,0339716	186,755	245,477	26,3327	13,4569	-0,125209	0,541431	0,6279808
CG5670-RB	Atpalpha	0,0184381	145,925	81,454	0,0620729	29,58	78,488	-0,137434	0,500306	0,6279808
CG5670-RC	Atpalpha	0,0184381	7,70635	178,207	153,873	0,0244823	0,030848	-0,125563	0,540304	0,6279808
CG5670-RD	Atpalpha	0,0184381	0,0315829	0,0503999	0,0593875	0,0241249	1,38596	0,061607	0,756259	0,6279808
CG5670-RE	Atpalpha	37,5453	28,4737	0,0319152	0,0358395	77,9551	1,2205	-0,137539	0,500065	0,6279808
CG5670-RF	Atpalpha	0,0174128	0,0167947	0,0358062	8,87275	0,0508111	0,0382943	-0,124316	0,544145	0,6279808
CG5670-RG	Atpalpha	0,0174128	0,0167947	180,468	27,0357	25,3241	25,6604	-0,12148	0,55277	0,6279808
CG5670-RH	Atpalpha	0,0171685	0,0167947	13,8346	20,0581	0,0550074	0,0414569	-0,136253	0,503791	0,6279808
CG5670-RI	Atpalpha	139,976	0,0167947	3,04976	8,96129	161,404	166,271	-0,125644	0,539547	0,6279808
CG5671-RA	Pten	0,0375076	0,0288849	0,0304448	0,034061	0,0461358	0,0347707	0,20585	0,356127	0,6279808
CG5671-RB	Pten	0,0317113	3,31506	0,0276212	0,0306827	0,0415599	0,031322	0,188402	0,401401	0,13772387
CG5671-RC	Pten	3,66822	5,80483	7,65487	10,9444	4,77439	4,36481	0,145675	0,532588	0,6279808
CG5671-RD	Pten	7,98788	0,0245092	0,0258328	0,0285676	0,0386949	0,0291628	0,229265	0,309893	0,6279808
CG5671-RE	Pten	0,0269075	8,92982	11,0595	11,8062	7,57607	7,9727	0,245589	0,273618	0,6279808
CG5671-RF	Pten	10,3756	13,0955	12,0948	13,4779	8,46818	5,61225	0,20585	0,356127	0,6279808
CG5674-RA	CG5674	1,87768	2,31027	0,707859	4,81049	2,28869	0,00700198	0,281352	0,231136	0,6279808
CG5674-RB	CG5674	1,53384	0,795587	1,78959	0,0223171	0,729171	2,39538	0,270611	0,250291	0,6279808
CG5674-RD	CG5674	2,92289	2,79385	3,77618	1,5422	0,03436	1,38008	0,359955	0,12814	0,6279808
CG5675-RA	X11L	8,07921	8,07205	0,00904474	5,58511	0,0134789	1,24653	-0,114235	0,678374	0,6279808
CG5676-RA	CG5676	37,3987	35,9602	54,0572	56,145	6,77208	44,0525	-0,094769	0,666618	0,6279808
CG5677-RA	Dpse\GA19051	65,8895	5,40179	0,041462	0,0424144	0,0672311	12,5499	-0,160035	0,476589	0,6279808
CG5680-RB	bsk	66,6157	57,2165	71,2731	71,2181	8,2662	41,5238	0,376587	0,089242	0,6279808
CG5681-RA	CG5681	0	0	0	0,101761	0	0	0,020887	0,887016	0,6279808
CG5682-RA	Edem2	12,8815	11,6437	296,566	0	0,106311	0,0128453	0,025419	0,896583	0,6279808
CG5683-RA	Aef1	14,4056	20,0177	4,33618	26,5579	21,8603	19,6114	0,435568	0,042069	0,6279808
CG5683-RB	Aef1	0,0224392	0,0204391	3,92537	17,6312	6,2998	13,0908	0,438177	0,041174	0,6279808
CG5683-RC	Aef1	9,55781	0,0207778	27,1901	0,0235704	2,50036	0,0240616	0,435568	0,042069	0,13772387
CG5684-RA	Pop2	26,7312	27,9244	32,1134	29,5299	7,35733	18,8938	0,414332	0,057232	0,6279808
CG5684-RB	Pop2	0,0372958	0,0339716	0,0358062	0,0406107	15,7031	0,0414569	0,411879	0,059741	0,6279808
CG5684-RC	Pop2	0,0359564	0,0327515	0,0345203	0,0390233	4,35529	0,0398365	0,412592	0,059158	0,6279808
CG5684-RD	Pop2	0,0459924	0,0418931	0,0441555	0,0511772	13,8575	0,0522436	0,25242	0,242281	0,6279808
CG5685-RA	Calx	0,0123815	0,0112779	0,011887	1,70425	1,93304	3,45163	-0,288249	0,291889	0,6279808
CG5685-RB	Calx	0,0138359	0,0126027	0,0132833	0,0142422	0,0192911	7,82892	-0,278655	0,309466	0,13772387
CG5685-RC	Calx	12,5996	10,8969	9,42863	8,92922	12,5408	0,0344149	-0,276806	0,312408	0,6279808
CG5686-RA	chico	10,9065	11,3002	12,0978	65,0809	69,9401	9,36354	-0,022541	0,901911	0,6279808
CG5687-RA	CG5687	4,12674	0,0427561	2,13359	3,97109	13,7376	53,6053	-0,318968	0,342736	0,6279808
CG5688-RA	Grip163	2,83451	35,8193	2,7213	0,0761295	0,04199	0,599048	0,351689	0,159875	0,6279808
CG5690-RA	Cnb	3,76961	3,62983	4,78232	5,58053	0,0709171	0,375744	0,171918	0,529215	0,6279808
CG5691-RA	mv	0,423021	0,491279	2,70542	13,2212	0,479333	2,81625	-0,33827	0,31074	0,6279808
CG5692-RA	pins	13,6039	15,2221	15,2581	11,8512	3,78713	11,6941	0,201349	0,393751	0,6279808
CG5693-RA	CG5693	0	0,102184	0	0,112122	0	0	NA	NA	0,6279808
CG5694-RA	ova	9,93263	10,6993	8,43405	13,0162	11,4825	6,41822	0,202637	0,312491	0,6279808
CG5694-RB	ova	21,2171	15,0925	20,8892	17,7609	8,9857	13,2459	0,188564	0,348302	0,6279808
CG5695-RA	jar	25,4639	10,1398	12,2681	0,0319162	14,8213	0,0270191	0,10985	0,669123	0,6279808
CG5695-RB	jar	0,0146196	31,4955	7,20127	0,0290717	3,3379	0,020442	0,114242	0,566368	0,6279808
CG5695-RC	jar	0,0144755	0,0133166	0,0286569	0,0276099	2,14106	66,0663	0,110353	0,667264	0,6279808
CG5695-RD	jar	0,0146879	0,0131853	0,0256933	0,0251637	0,0393778	0,0438343	-0,167525	0,526892	0,6279808

gene_id	Symbol	W1_FPKM	W2_FPKM	W3_FPKM	MCM51_FPKM	MCM52_FPKM	MCM53_FPKM	FC	p-value	p-adj
CG5695-RE	jar	0,0156294	0,0133787	0,0245024	0,0248599	0,0373978	0,0499174	0,054578	0,833695	0,6279808
CG5695-RF	jar	0,0186884	0,0142363	0,0224873	28,7821	0,0340844	0,0506694	0,10997	0,668748	0,6279808
CG5697-RA	CG5697	9,5733	11,3421	10,5085	10,1421	0,631434	0,648178	-0,308163	0,294972	0,6279808
CG5700-RB	prc	43,366	2,15375	48,8729	24,4706	1,88443	1,36067	-0,344971	0,162662	0,6279808
CG5701-RA	RhoBTB	6,4991	8,30789	8,18757	7,99148	12,5728	11,7947	0,078378	0,694664	0,6279808
CG5703-RA	Dpse\GA19069	58,7583	54,5422	119,4	70,7601	107,439	2869,36	-0,266978	0,382946	0,6279808
CG5704-RA	CG5704	2,36181	1,60097	4,90409	2,6945	3,27805	3,51831	-0,054057	0,873286	0,13772387
CG5705-RA	mRF1	5,70103	9,18943	7,7165	0,0125831	1,81446	6,9468	-0,008306	0,975801	0,6279808
CG5706-RA	beta-PheRS	8,43341	6,52252	14,3197	10,6664	8,97161	9,86236	-0,116215	0,665922	0,6279808
CG5707-RA	CG5707	8,07152	3,79781	11,5919	0,545585	0,866256	2,18012	-0,347793	0,218227	0,6279808
CG5708-RA	CG5708	24,9056	19,54	32,3807	0,0438833	25,9083	23,4572	0,125706	0,563948	0,6279808
CG5708-RB	CG5708	3,10021	0,0364619	4,22647	23,2853	0,0973799	0,0733914	0,125551	0,564504	0,6279808
CG5709-RA	ari-2	12,447	6,02586	0	0,182595	1,74744	3,77011	0,154737	0,578098	0,6279808
CG5711-RA	Arr1	5,89474	3,86526	4,242	12,3271	4,92411	0,287831	-0,174575	0,527825	0,6279808
CG5712-RA	ACXD	1,72784	0,00635773	2,85466	0,78483	1,33205	1,20975	-0,181348	0,478861	0,6279808
CG5714-RA	ecd	8,12935	9,12001	10,2298	35,7528	33,6074	4,29949	0,212235	0,285037	0,6279808
CG5715-RA	CG5715	0,0345464	10,4549	10,8837	12,4043	0,0506094	0,0256881	0,210346	0,55038	0,6279808
CG5717-RA	yellow-g	0,338184	12,839	24,5534	8,49943	0,473183	0,555537	-0,318066	0,354669	0,6279808
CG5718-RA	Dpse\GA19081	0	0,0837863	0,0441555	0,135021	2,64981	3,83339	-0,006194	0,974196	0,6279808
CG5720-RA	Nab2	8,54478	14,5808	14,0876	0,0373637	5,53934	0,0198625	0,16738	0,472056	0,6279808
CG5721-RA	CG5721	18,6703	0,0639827	0,067438	10,5009	0,101824	0,0767406	-0,233505	0,329152	0,6279808
CG5722-RA	Npc1a	17,4114	13,2186	41,7508	31,9543	9,69174	10,0296	0,046021	0,857134	0,6279808
CG5723-RB	Ten-m	38,1597	47,3889	0,00520329	35,3438	0,0074113	0,0055856	0,397107	0,178652	0,6279808
CG5723-RC	Ten-m	0,00541976	0,00493669	0	0,00547158	0,209666	0,158017	0,395595	0,180232	0,6279808
CG5724-RA	Ugt37A2	4,10641	0,371522	0,0988112	0,119256	0,161534	0,153041	-1,163666	0,001117	0,6279808
CG5725-RA	fbl	0,034631	0,0368338	35,5845	0,0452081	0,0612346	0,0461501	0,007693	0,96646	0,6279808
CG5725-RB	fbl	26,917	0,0390568	9,94307	2,79019	5,44869	0,0423176	0,035525	0,845349	0,6279808
CG5725-RC	fbl	0,0354104	5,32962	5,80934	15,9534	8,5376	0,042053	0,045388	0,803552	0,6279808
CG5725-RD	fbl	0,0404381	0,0374604	9,52709	0,0377274	0,051102	0,0425317	0,04609	0,800662	0,6279808
CG5725-RE	fbl	0,0428786	18,9296	6,22554	8,32538	6,05094	0,037317	0,126494	0,488971	0,6279808
CG5726-RA	CG5726	8,17305	9,11059	10,3341	5,99494	6,08101	6,5551	0,038803	0,834107	0,13772387
CG5727-RA	CG5727	25,9956	22,0156	9,54649	78,5818	7,32957	83,0446	-0,354007	0,191589	0,6279808
CG5728-RA	CG5728	8,53307	0,0271886	13,7116	0,0149227	1,24105	7,50174	0,037374	0,885998	0,6279808
CG5729-RA	Dgp-1	7,02686	4,65796	4,95414	13,3717	0,101017	0,0761326	-0,954091	0,001794	0,6279808
CG5729-RB	Dgp-1	4,28858	8,10943	8,98073	34,5244	0,112758	0,0849816	-0,953793	0,001825	0,6279808
CG5730-RA	AnxB9	135,515	105,616	24,8073	3,02984	3,72772	0,0195849	-0,499929	0,06964	0,6279808
CG5730-RB	AnxB9	56,2481	45,497	62,1054	0,169596	1,28025	0,0195849	-0,457313	0,094021	0,13772387
CG5730-RC	AnxB9	129,27	107,582	2,25333	10,7602	10,7105	26,8898	-0,583787	0,032074	0,6279808
CG5730-RD	AnxB9	0,0524966	0,0478176	1,06186	56,9628	70,4953	20,2805	-0,457347	0,09399	0,6279808
CG5731-RA	CG5731	7,7234	11,1502	23,0793	29,2362	39,3567	14,5126	-0,150993	0,56924	0,6279808
CG5732-RA	Gld2	0,391153	0,0121035	9,25174	0,0136605	0,383357	0,0139451	0,668712	0,035751	0,6279808
CG5733-RA	Dsim\Nup75	2,85475	3,28903	4,97776	0,0832469	2,33297	3,53952	0,001812	0,99469	0,6279808
CG5734-RA	CG5734	10,743	5,41022	12,6298	8,39306	14,8914	34,2682	-0,431312	0,057031	0,13772387
CG5735-RA	orb2	0,0288709	0,0192187	1,15462	4,89256	1,23831	0,0799805	0,079004	0,779257	0,6279808
CG5735-RB	orb2	2,65044	0,632591	0,238126	1,82333	0,0299246	127,507	-0,004994	0,985156	0,6279808
CG5735-RC	orb2	0,550859	0,0251651	0,0265241	0,0220926	0,770384	0,0299951	0,016976	0,95003	0,6279808
CG5735-RD	orb2	0,738474	2,78453	5,14755	0,873648	102,767	2,64688	0,003646	0,989154	0,6279808
CG5737-RA	dmrt93B	0,574279	0,892335	0,810797	1,5894	1,09069	0,876504	-0,123143	0,721429	0,6279808
CG5738-RA	lolal	0,0639856	0,0668985	0,0705113	9,95478	13,183	0	0,373649	0,144764	0,6279808
CG5738-RB	lolal	0,0702435	109,508	128,684	9,72276	6,61728	6,39603	0,374201	0,144304	0,6279808
CG5738-RC	lolal	0,0734447	0,0586802	0,0618492	0	0	0,045711	0,374596	0,143094	0,6279808
CG5738-RD	lolal	100,948	0,0371368	0,0391424	0,0447779	7,31703	27,0321	0,373662	0,14378	0,6279808
CG5738-RE	lolal	0,0644223	19,4302	27,4401	24,7614	0,060652	10,5269	0,370387	0,1481	0,6279808
CG5741-RA	CG43783	0,0164422	68,5798	123,551	0,0170284	1,36838	2,89386	0,360457	0,1586	0,6279808
CG5741-RB	CG43783	4,02423	0	1,74366	4,92467	4,05878	0,0314391	0,360197	0,158555	0,6279808
CG5741-RC	CG43783	4,79717	1,27181	0	4,52698	1,31042	1,62803	0,358091	0,160917	0,6279808
CG5742-RA	CG5742	6,28218	5,54728	6,21568	8,4644	47,7679	4,25639	0,042458	0,855022	0,6279808
CG5744-RB	Frq1	17,4747	19,0551	0,775339	1,68438	4,44625	4,36747	0,305578	0,175939	0,6279808
CG5744-RC	Frq1	4,7275	3,34685	21,7597	1,26675	0,316811	0,027238	0,305252	0,17693	0,6279808
CG5745-RA	CG5745	8,12649	7,25179	5,41749	10,8233	3,75952	3,14113	0,002562	0,990118	0,6279808
CG5746-RA	CG5746	7,09264	0,0114999	1,30291	14,6031	0,120687	0,103243	0,206835	0,473545	0,6279808
CG5746-RB	CG5746	6,6596	9,75726	4,69871	0,961519	0,363523	1,25162	0,204644	0,478593	0,6279808
CG5746-RC	CG5746	6,38471	12,0361	0,0258328	1,24702	12,0687	0,0283158	0,204644	0,478593	0,6279808
CG5746-RD	CG5746	5,976	7,87891	13,828	2,58	0,0204211	0,0291628	0,206835	0,473545	0,6279808
CG5747-RB	mfr	0,0129994	0,0118407	0,0124802	0,013501	54,9625	45,6492	0,211209	0,504905	0,6279808
CG5747-RC	mfr	0,0129408	0,0117874	0,012424	0,0134376	28,928	0,0137823	-0,653952	0,011724	0,6279808
CG5747-RD	mfr	0,0126606	0,0115322	0,012155	0,0131349	0,0591252	0,0137176	0,211209	0,504905	0,6279808
CG5747-RE	mfr	0,0128452	0,0117003	0,0123322	0,0133343	0,0182871	0,0134086	-0,525203	0,061168	0,6279808
CG5747-RF	mfr	0,0127732	0,0116347	0,0122631	0,0132565	0,0182013	0,0136121	0,211209	0,504905	0,6279808
CG5747-RI	mfr	0,0115015	0,0104763	0,0110421	0,0118884	0,0177914	0,0135327	0,211209	0,504905	0,6279808
CG5747-RJ	mfr	0,0118331	0,0107784	0,0113605	0,0122441	0,0180614	0,0121361	0,211209	0,504905	0,6279808
CG5748-RA	Hsf	8,7568	16,4908	4,19785	24,1621	6,49051	3,2227	0,014432	0,967351	0,6279808
CG5748-RB	Hsf	0,0231872	0,0211205	0,0222611	0,0243996	0,0330494	0,0249081	0,014432	0,967351	0,13772387
CG5748-RC	Hsf	22,9984	61,4644	13,5553	67,2994	19,5945	15,6315	0,013043	0,970486	0,6279808
CG5748-RD	Hsf	12,2272	31,4836	9,71763	37,7505	6,96482	4,42648	0,013043	0,970486	0,6279808
CG5751-RE	TrpA1	0,646169	25,665	18,2113	23,6504	0,0792268	0,525308	-0,126618	0,671442	0,6279808
CG5751-RF	TrpA1	0,0172177	0,517978	0,119843	0,324744	3,0591	0,0182362	-0,117795	0,694198	0,6279808
CG5753-RA	stau	16,1212	23,3853	18,6592	0,01419	3,3789	4,19245	0,251024	0,39277	0,6279808
CG5753-RB	stau	3,58144	50,721	16,1189	0,0142947	2,24942	2,00753	0,261177	0,374441	0,6279808
CG5755-RA	Slc25A46b	0	0,0602266	0	0,0202774	31,7506	29,1619	0,01562	0,897983	0,6279808
CG5756-RA	CG5756	14,5481	27,3296	14,7054	22,1001	110,448	13,996	0,421013	0,225381	0,6279808
CG5756-RB	CG5756	21,3827	40,534	20,3938	9,23475	84,1447	2,81459	0,541765	0,113542	0,6279808
CG5757-RA	CG5757	4,32594	0,300332	53,3079	5,11044	4,96541	2,36237	-0,040559	0,895675	0,6279808
CG5758-RA	CG5758	4,934	0,0196516	4,73693	0,00685478	4,55625	0,0208474	0,018295	0,932104	0,6279808
CG5758-RB	CG5758	0,0215746	0,0178295	0,0207129	0,0068505	0,0306333	13,7101	0,014544	0,946008	0,6279808
CG5758-RC	CG5758	0,0195742	17,5767	0,0187924	0,00684836	0,0276616	0	0,021443	0,920524	0,6279808
CG5760-RA	rtet	17,9036	18,4974	19,4224	12,1378	8,3099	5,55061	0,373035	0,125761	0,6279808
CG5762-RA	Dsim\GD18296	1,03355	0,616798	0,44465	0,0161558	2,68817	0,87987	0,019915	0,955623	0,6279808
CG5765-RA	Muc55B	44,1335	0,626943	104,354	1,86259	5,79702	7,62676	-0,436898	0,214354	0,6279808
CG5767-RA	CG5767	8,23987	16,0446	29,8726	0,0303855	33,8626	33,4824	-0,286571	0,352406	0,13772387
CG5768-RA	CG5768	0,330104	0,0751703	1,24199	0	0,0263547	1,01721	0,027501	0,937059	0,6279808
CG5768-RB	CG5768	1,23789	1,0148	3,43934	6,51661	4,51261	0,0132293	0,0312	0,929534	0,6279808
CG5770-RA	CG5770	2,2099	11,3785	25,5641	61,8317	10,7293	32,5201	0,08956	0,795383	0,6279808
CG5771-RA	Rab11	0,0372958	9,74975	0,0167173	0,0375126	0,0259864	16,9045	-0,144906	0,493098	0,6279808
CG5771-RB	Rab11	145,919	2,54473	0,0167173	29,499	0,0259864	3,93628	-0,143507	0,497149	0,6279808
CG5772-RB	Sur	3,79169	4,46905	0,0854174	4,54609	0,150375	0,113332	-0,072021	0,74322	0,6279808
CG5773-RA	CG5773	9,57174	0,0109238	6,08201	4,59013	36,956	7,95352	-0,584807	0,093549	0,6279808
CG5776-RA	CG5776	1,81955	0,0464521	0,0489607	4,12239	5,56288	0,104852	-0,268878	0,363398	0,6279808
CG5778-RA	CG5778	0,108916	0,0992082	2,98013	0,144019	22,849	2,14615	0,328933	0,35668	0,6279808
CG5778-RB	CG5778	3,77466	0,885603	2,1963	2,80503	25,676	0,15697	0,353761	0,321726	0,6279808
CG5779-RA	PPO1	46,4984	12,0902	44,3164	0	1,1962	46,8517	-0,134767	0,630132	0,6279808
CG5780-RB	IFT43	4,14146	4,04583	4,79228	0,102712	0,139124	0,0586685	-0,025729	0,923906	0,6279808
CG5781-RA	CG5781	0,0509975	5,15776	0,039681	22,2594	22,5915	19,9887	-0,354313	0,233531	0,6279808
CG5783-RA	CG5783	7,48814	30,3666	11,1164	2,82968	0	0	-0,322737	0,299153	0,6279808

gene_id	Symbol	W1_FPKM	W2_FPKM	W3_FPKM	MCM51_FPKM	MCM52_FPKM	MCM53_FPKM	FC	p-value	p-adj
CG5784-RA	Mapmodulin	28,6584	0,857765	47,0493	37,9822	1,41872	16,3012	0,335287	0,102703	0,6279808
CG5784-RB	Mapmodulin	0,0285096	0,012558	0,027371	1,99877	34,7274	0,0469227	0,335287	0,102703	0,6279808
CG5784-RC	Mapmodulin	26,9723	6,81308	13,6659	4,96829	0,401468	4,74995	0,128055	0,566216	0,6279808
CG5784-RD	Mapmodulin	0,0417497	0,0107425	0,0400822	8,44801	4,05118	80,8861	0,334883	0,102595	0,6279808
CG5785-RB	thr	1,17835	0,902726	3,73099	2,34816	0,942776	1,3469	0,162245	0,630768	0,6279808
CG5786-RA	ppan	9,58454	5,45968	19,5484	10,8605	10,4264	0,0195849	-0,017145	0,956112	0,6279808
CG5787-RA	CG5787	12,3786	12,7009	17,7488	23,0467	83,8491	10,864	0,017491	0,950918	0,6279808
CG5788-RA	Ubc10	28,2486	1,26549	42,0833	37,3395	37,5903	38,768	-0,145678	0,550121	0,6279808
CG5789-RA	CG5789	5,82944	6,19182	7,47488	9,71853	4,80226	5,93904	-0,179501	0,399461	0,6279808
CG5790-RA	CG5790	0	4,32631	0	4,12883	5,97704	3,69523	0,01562	0,897983	0,6279808
CG5791-RA	CG5791	3,36118	0,746731	5,5094	2,0189	0,0588911	1,58824	0,648697	0,067042	0,6279808
CG5792-RA	Pih1D1	14,3658	10,3986	0,174424	13,5989	10,6945	8,82423	0,74779	0,000648	0,6279808
CG5792-RC	Pih1D1	9,04617	0,0111753	16,3938	11,5126	8,92966	9,46486	0,151749	0,577759	0,13772387
CG5793-RA	CG5793	44,6624	1,28272	0,0177016	1,24308	5,74581	0,018182	-0,23043	0,380692	0,6279808
CG5794-RB	puf	0,624212	0,00685443	0,0072246	39,7898	0,029755	0,0380344	-0,23381	0,409847	0,6279808
CG5794-RD	puf	0,792414	0,0195813	0,0206388	9,01177	3,78139	2,171	-0,235437	0,406439	0,6279808
CG5794-RE	puf	0,00752515	6,34779	5,23435	0,0372581	0,0504663	4,29593	-0,561028	0,073741	0,6279808
CG5796-RA	Ppox	6,70709	4,33758	11,0101	0,00763384	5,69991	4,33194	-0,09068	0,719973	0,6279808
CG5798-RA	Usp8	6,87346	0,0253559	129,944	170,548	0,0259864	5,56511	0,061415	0,766167	0,6279808
CG5799-RA	dve	2,33961	35,5835	30,5272	17,0971	0,0316995	65,3333	0,420389	0,102883	0,6279808
CG5799-RB	dve	11,3645	0,0199579	0,0210357	4,27336	18,3164	19,8576	0,461201	0,073567	0,6279808
CG5800-RA	CG5800	5,77883	33,8549	11,0241	47,4556	2586,52	27,0955	-0,180091	0,528254	0,6279808
CG5802-RA	meigo	0,0346733	0,0156383	0,0332885	1,75065	5,48886	0,060625	0,081885	0,685217	0,6279808
CG5802-RB	meigo	30,2824	128,833	30,6643	13,688	273,701	0,0365863	0,081302	0,687788	0,6279808
CG5803-RA	Fas3	106,557	112,631	72,19	101,187	32,1347	50,2016	0,091694	0,756822	0,6279808
CG5803-RB	Fas3	0,0278136	28,286	17,1832	41,5408	13,9349	14,4376	0,074848	0,80034	0,6279808
CG5803-RC	Fas3	0,0322531	0,0293783	0,0309649	0,0346886	0,0469859	0,0354114	0,296917	0,309442	0,6279808
CG5803-RD	Fas3	38,7464	45,7089	32,0096	63,0036	14,7243	22,5431	0,045218	0,878259	0,6279808
CG5804-RA	Acbp5	80,9133	52,0775	262,484	148,689	0,154541	382,905	-0,320468	0,367602	0,6279808
CG5805-RA	CG5805	17,233	17,4925	24,1783	0,0147656	2,68708	26,5397	-0,56275	0,037234	0,6279808
CG5807-RA	lili	16,8118	0,0203976	14,9471	1,61365	133,439	0,0149626	-0,195671	0,357162	0,13772387
CG5808-RA	CG5808	3,60824	3,68279	0,0246101	0,0146572	3,13546	0,0150733	-0,066608	0,77392	0,6279808
CG5809-RA	CaBP1	81,1068	11,5688	17,8134	15,7715	12,0409	11,6541	0,037805	0,864999	0,6279808
CG5810-RA	CG5810	7,59847	6,87445	0,0811212	0,231069	26,518	0	-1,047065	0,000255	0,6279808
CG5811-RA	RYa-R	0,43471	0,3271	0,0188706	0,291704	0	0	-0,514887	0,137796	0,6279808
CG5812-RA	TwdlT	57,4386	68,6491	0	0,341017	0,176233	0,282886	0,500319	0,103022	0,6279808
CG5813-RA	chif	0,0100961	0,00919626	0,0096929	0,0103028	5,88771	13,05	0,069233	0,797906	0,6279808
CG5813-RB	chif	8,89255	7,17887	10,7034	12,2221	0,692822	0,0105175	0,150172	0,582028	0,6279808
CG5814-RA	CycB3	6,26201	6,47307	13,3655	9,07542	12,1004	11,2638	0,176717	0,588819	0,6279808
CG5815-RA	CG5815	0,0285466	3,14437	3,25045	37,4092	0,0277821	5,37775	0,158615	0,467055	0,6279808
CG5815-RB	CG5815	7,12659	0,0260022	1,47995	0,0366537	0,299324	0	0,158615	0,467055	0,6279808
CG5818-RA	mRpL4	27,9939	29,759	40,3138	34,9086	44,0692	44,218	-0,25	0,336045	0,6279808
CG5819-RA	CG5819	15,4685	15,2088	14,3789	4,32218	19,446	9,11131	0,337663	0,19855	0,6279808
CG5819-RB	CG5819	0,0202273	4,48635	2,76727	10,4181	0,0311355	0,0212761	0,334942	0,202933	0,6279808
CG5820-RA	Gp150	91,0653	6,82122	0,0145664	203,126	3,88587	4,22285	0,447834	0,101101	0,6279808
CG5820-RB	Gp150	0,0155253	84,0091	33,2885	0,0162554	0	0,4358	0,447711	0,101162	0,6279808
CG5820-RC	Gp150	0,0151724	0,0141416	0,251067	0,0158679	0,154143	0,250314	0,447473	0,101388	0,6279808
CG5820-RD	Gp150	87,2494	0,0138201	10,2426	0,0164749	12,5944	8,586	0,448928	0,100163	0,6279808
CG5821-RA	qkr58E-2	18,8326	16,7575	36,0286	25,4472	0,0214931	63,3903	-0,088983	0,686663	0,6279808
CG5823-RA	CG5823	15,659	0,764767	0,0175014	21,8647	14,4755	0,597258	0,022307	0,907928	0,6279808
CG5824-RA	l(3)07882	5,47064	0,0497545	10,5814	26,9558	4,36121	1,42014	-0,004135	0,988921	0,6279808
CG5825-RA	His3.3A	145,553	165,319	267,059	240,597	194,57	276,858	-0,170509	0,510564	0,6279808
CG5825-RB	His3.3A	0,0821839	0,0748588	0,0789015	0,109542	0,148375	0,111824	-0,170509	0,510564	0,6279808
CG5826-RA	Prx3	53,7827	50,0471	1,5272	2,71212	0,0841574	0,450614	0,018908	0,945223	0,6279808
CG5827-RA	RpL37A	875,384	326,494	16,5318	386,465	289,036	5,55297	-0,624476	0,030116	0,6279808
CG5827-RB	RpL37A	0,102815	0,0102836	0,317713	0,0115528	0,0156483	10,9066	-0,624476	0,030116	0,13772387
CG5828-RA	CG5828	24,2139	17,9263	325,546	14,2798	10,2724	274,153	1,283598	1,76E-07	0,6279808
CG5830-RA	CG5830	32,4887	2,78641	3,52427	4,47167	6,4248	18,1618	0,389915	0,053357	0,6279808
CG5834-RA	Hsp70Bbb	87,1534	109,897	86,4391	358,147	166,688	69,7746	-0,423761	0,17522	0,6279808
CG5835-RA	CG5835	16,9634	20,253	18,9581	0,0307256	12,1147	0,0477217	0,04361	0,843864	0,6279808
CG5836-RA	SF1	15,8131	13,885	23,5525	0,0621314	15,7337	0,0634261	-0,288334	0,203205	0,6279808
CG5837-RA	Hem	12,7985	11,0597	2,44691	3,95124	7,98827	1,61761	-0,009207	0,969926	0,6279808
CG5838-RA	Dref	0,0213286	0	0	0,0227564	0	0	0,103543	0,633255	0,6279808
CG5840-RA	P5cr-2	0,0546231	1,93954	0,0524415	64,7527	60,3007	48,0862	-0,316522	0,222939	0,6279808
CG5840-RB	P5cr-2	24,699	0,202193	37,9122	6,38176	1,04162	5,64601	-0,31692	0,222368	0,6279808
CG5841-RA	mib1	10,4952	0,0113954	10,9702	17,5132	5,74782	8,26462	0,194526	0,496197	0,6279808
CG5842-RA	nan	0,278019	0,422065	52,202	0,0170858	0,0265744	0,0209775	-0,03924	0,907297	0,6279808
CG5844-RA	CG5844	12,5696	12,6298	3,89296	15,0951	0,148337	2,7717	-0,168167	0,464008	0,6279808
CG5846-RA	CG5846	0,041126	1457,13	2452,65	2507,14	3408,17	4052,72	-0,539523	0,034406	0,6279808
CG5847-RA	zye	11,0416	0,0347764	0,028413	0,0571718	7,00828	3,99141	0,707835	0,028443	0,6279808
CG5848-RA	cact	0,0298221	0,0297511	13,0762	13,7182	8,39789	10,3681	0,084598	0,661393	0,6279808
CG5848-RB	cact	37,2632	0,027164	0,028631	0,0318853	0,0431889	0,0325497	0,083174	0,667084	0,6279808
CG5848-RC	cact	13,0928	30,9911	49,4117	65,4898	34,3983	25,7951	0,02077	0,916924	0,6279808
CG5848-RD	cact	15,2121	26,8015	14,4487	0,0374699	0,0507533	16,1374	0,084224	0,661651	0,6279808
CG5849-RA	CG5849	7,29832	0,118089	9,54285	8,65542	8,61164	10,2104	0,147149	0,472025	0,6279808
CG5850-RA	CG5850	12,5647	0,0279331	0,0294416	0,0328551	40,1977	0,0335398	0,106722	0,618033	0,6279808
CG5850-RB	CG5850	0,0306664	0,0373829	6,7574	6,54171	0,0634099	4,97582	-0,043794	0,835215	0,6279808
CG5851-RA	sds22	12,5822	12,1639	17,119	329,319	0,149368	245,248	0,439914	0,04716	0,6279808
CG5853-RA	CG5853	18,3911	11,3447	13,3642	14,4483	7,59668	10,5761	-0,336513	0,32857	0,6279808
CG5854-RA	CG5854	0,0423835	0,0339908	0,0358265	0,0310294	1,12299	0,0414825	-0,001139	0,995257	0,6279808
CG5854-RB	CG5854	0,0416095	0,0110522	0,011649	3,80078	0,593872	0,0126998	-0,002652	0,988961	0,6279808
CG5855-RA	cni	31,5745	40,4489	34,7713	102,004	127,786	90,1897	-0,53382	0,045434	0,6279808
CG5855-RB	cni	46,1872	33,6642	22,5421	0,106188	0,143832	13,751	-0,534075	0,045355	0,6279808
CG5857-RA	Ndc1	2,50667	2,49635	4,21949	4,30608	26,2718	5,97137	-0,060884	0,804121	0,6279808
CG5859-RA	IntS8	4,38702	3,24049	10,0262	0,0171213	0,0237084	0,0674996	0,555813	0,031277	0,6279808
CG5860-RA	CG5860	0	0,0080188	22,8403	4,05437	0,0257539	0,0203986	-0,055469	0,774038	0,6279808
CG5861-RA	CG5861	45,9262	45,3094	9,23438	63,0249	8,71186	76,3155	-0,587114	0,026677	0,6279808
CG5862-RA	CG5862	27,9274	5,99202	39,2223	0,0191851	167,862	25,8829	0,342287	0,122676	0,6279808
CG5863-RA	CG5863	1,4183	2,02648	2,02912	8,10157	0,039817	7,48814	-0,414835	0,205314	0,6279808
CG5864-RA	AP-1sigma	26,9278	23,6327	31,1878	26,6503	5,63968	19,9091	0,342694	0,07499	0,6279808
CG5866-RA	CG5866	1,70244	18,979	10,0596	37,2409	1,5994	30,4343	0,019118	0,956302	0,6279808
CG5867-RA	CG5867	0,949366	0,778274	1,51908	0,0125831	6,80143	12,3522	-0,290309	0,404327	0,6279808
CG5869-RA	GMF	97,9823	75,3839	48,0548	95,0942	107,92	110,25	-0,38928	0,182032	0,6279808
CG5870-RA	beta-Spec	75,9679	10,0311	0,0278639	15,0278	23,1838	2,61321	-0,194894	0,451092	0,6279808
CG5871-RA	Oga	6,10631	6,06449	6,54993	0,0191851	0,0485448	29,4577	-0,195249	0,3194	0,6279808
CG5872-RA	CtIP	3,47735	5,3293	11,547	7,52944	0,0564335	0,0385135	-0,394702	0,075182	0,6279808
CG5873-RA	cysu	26,8521	29,1721	0,740711	33,5265	6,79604	11,7959	0,446654	0,160651	0,6279808
CG5874-RA	Nelf-A	9,35412	4,61137	6,18871	0,0180747	10,8165	6,47568	0,164453	0,530431	0,6279808
CG5876-RA	heix	15,9262	45,1978	12,9231	16,8267	38,1799	10,0531	0,175466	0,440547	0,6279808
CG5877-RA	CG5877	2,40117	1,19466	27,106	2,61823	6,93681	14,866	0,049649	0,831762	0,6279808
CG5877-RB	CG5877	1,34444	1,91934	0,0119677	2,06409	0,96385	0,0130573	0,119229	0,612945	0,6279808
CG5878-RB	alpha4GT2	0	0	0	0	0,174594	2,42626	NA	NA	0,6279808
CG5880-RA	CG5880	3,00948	6,83795	6,50477	0,380898	0,0267576	4,4942	0,373264	0,134026	0,6279808
CG5882-RA	CG5882	0,241338	0,0311417	0,0547058	8,65505	0	0,247256	0,147386	0,677545	0,6279808

gene_id	Symbol	W1_FPKM	W2_FPKM	W3_FPKM	MCM51_FPKM	MCM52_FPKM	MCM53_FPKM	FC	p-value	p-adj
CG5883-RA	CG5883	11,7326	3,13693	25,9183	2,23953	4,67839	4,00476	-0,35086	0,326731	0,6279808
CG5884-RA	par-6	14,5778	17,1399	13,8813	11,0162	8,64134	8,24655	0,229712	0,366974	0,6279808
CG5885-RA	CG5885	181,582	38,4068	20,7623	15,8805	0,0573767	61,5456	-0,116558	0,633699	0,6279808
CG5886-RA	CG5886	19,9138	17,4694	26,544	23,6058	5,87118	16,9936	0,257165	0,330035	0,6279808
CG5887-RA	Desat1	12,1648	12,6522	35,84	0,0375768	36,0597	23,8249	-0,069006	0,772241	0,6279808
CG5887-RB	Desat1	16,5049	17,1006	25,6404	0,0371112	32,9292	18,5068	-0,068754	0,773111	0,6279808
CG5887-RC	Desat1	0,034728	0,0316327	0,033341	14,3199	0,050898	0,0383598	-0,069569	0,77	0,6279808
CG5887-RD	Desat1	0,034331	0,0312711	0,0329599	688,478	0,0502673	0,0378845	-0,068696	0,773286	0,6279808
CG5887-RE	Desat1	43,0395	46,1959	20,9807	0,0396098	16,1427	25,9001	-0,067146	0,778251	0,6279808
CG5888-RA	CG5888	24,2828	31,0681	26,345	0,0433065	25,7573	30,1386	-0,390042	0,0775	0,6279808
CG5889-RA	Men-b	14,8795	0,176328	17,8516	0,0552541	0,51453	0,330048	-0,603191	0,047215	0,6279808
CG5890-RA	CG5890	2,71326	3,77983	3,37104	6,14135	9,22739	2,32596	-0,169289	0,592374	0,6279808
CG5892-RA	CG5892	5,01698	12,1396	11,111	0,0180747	12,1395	6,19654	-0,31716	0,375046	0,6279808
CG5893-RA	D	11,6149	18,1589	0,344765	9,25854	4,74573	6,06228	0,137167	0,466841	0,13772387
CG5895-RA	CG5895	2,88426	35,3674	33,9348	33,088	19,4707	4,95434	-0,740366	0,008919	0,6279808
CG5896-RA	grass	0,049249	0,0448594	0,464625	0,0619562	0,074842	11,6125	-0,616827	0,050488	0,6279808
CG5896-RB	grass	6,41124	3,96442	0,0501081	17,9688	13,9198	20,4565	-0,616827	0,050488	0,6279808
CG5897-RA	CG5897	1,67107	0	0	1,30567	0,514971	2,57714	-0,180067	0,598961	0,6279808
CG5899-RA	Etl1	0,0216099	34,2314	14,6497	15,0774	19,1368	0,0231268	-0,173643	0,464869	0,6279808
CG5899-RB	Etl1	0,0216099	0,0196838	14,1442	0,0230349	0,0306859	0,0231268	-0,173643	0,464869	0,6279808
CG5899-RC	Etl1	4,68089	0,0196838	0,0210777	0,0226547	0,0306859	4,31816	-0,173643	0,464869	0,6279808
CG5902-RA	CG5902	66,6779	0,0386058	4,66447	21,7742	0,0515447	0,0495862	-0,125778	0,563287	0,6279808
CG5902-RB	CG5902	0,0351343	0,0379008	20,6673	7,55218	0,0657938	0,0485483	-0,124001	0,56914	0,6279808
CG5903-RA	CG5903	55,7331	43,7133	8,951	7,49989	55,4871	11,674	-0,51527	0,110569	0,6279808
CG5904-RA	mRpS31	32,3642	0,0109437	36,1714	35,02	46,3	44,2914	-0,35777	0,169931	0,6279808
CG5905-RA	Nep1	0,455331	0,437335	2,92785	14,3135	0,0094791	9,07368	-0,441041	0,118408	0,6279808
CG5905-RB	Nep1	2,45045	0,898337	0,486416	5,9463	1,34129	2,57039	-0,486995	0,082571	0,6279808
CG5906-RA	CG5906	0	0,104733	5,24977	0	0	0	0,01562	0,897983	0,6279808
CG5907-RA	Frq2	0,0282174	0,216304	0,512966	0	0,0706925	0,249557	0,267572	0,288029	0,6279808
CG5907-RB	Frq2	0,0274896	1,31082	0,0270904	9,57853	0,481474	17,8747	0,264406	0,294889	0,6279808
CG5907-RC	Frq2	5,66989	0,0250394	1,99257	0,026682	0,0423487	0,0532781	0,265366	0,292142	0,6279808
CG5909-RA	CG5909	0,340597	0,103413	15,8126	0,432875	15,4482	13,5673	-0,034982	0,920343	0,6279808
CG5910-RA	CG5910	16,7458	0,0308807	14,8309	182,28	16,4518	1,06315	-0,098535	0,704356	0,6279808
CG5910-RB	CG5910	5,00749	11,6523	7,21124	0,0316252	4,57696	5,03834	-0,108064	0,678758	0,6279808
CG5911-RA	ETHR	0,879226	1,10736	1,07337	0,0178109	0,737698	0,705857	0,232012	0,37806	0,6279808
CG5911-RB	ETHR	1,46391	2,06999	1,48575	130,463	1,1365	0,953244	0,271214	0,326074	0,6279808
CG5912-RA	arr	6,38807	7,21078	10,2736	9,42216	3,57724	9,97654	0,074118	0,785591	0,6279808
CG5913-RA	CG5913	6,28517	0,0294071	7,71899	1,42954	0,0470354	1,60517	0,188438	0,461411	0,6279808
CG5915-RA	Rab7	143,514	175,272	140,766	0,0254161	0,0344262	1,88463	0,111625	0,595109	0,6279808
CG5916-RA	CG5916	8,08001	5,42597	9,45488	5,89292	13,0725	7,15499	-0,273886	0,303359	0,6279808
CG5919-RA	Idi	20,9035	0,0123773	33,7753	4,20623	0,0514852	19,3298	0,371419	0,115892	0,6279808
CG5920-RA	RpS2	1220,82	6,65395	19,3001	8,44893	7,37366	89,3993	-0,725574	0,01354	0,6279808
CG5921-RA	CG5921	2,24973	2,58727	0,0241327	5,0322	0,0359951	2,00681	-0,252632	0,209417	0,6279808
CG5921-RB	CG5921	2,44667	2,28269	5,38205	2,10664	4,75231	3,27082	-0,258417	0,199904	0,6279808
CG5923-RC	DNApol-alpha73	2,07897	2,48086	3,37298	13,9947	5,34975	2,97935	-0,22006	0,390126	0,6279808
CG5924-RA	mtDNA-helicase	3,26055	3,09767	4,24108	13,9957	3,09905	21,1003	-0,043842	0,858679	0,6279808
CG5925-RA	Desat2	0,0485852	24,7112	0,279868	150,168	15,1041	103,884	-0,489938	0,128466	0,6279808
CG5927-RA	Hesr	0,807594	0,735613	10,9423	19,8702	1,20151	5,38466	0,290871	0,347978	0,13772387
CG5928-RA	CG5928	0,0818106	94,8899	82,0386	0,10006	35,3028	61,1898	0,505877	0,02592	0,6279808
CG5928-RB	CG5928	36,3094	0,0745188	0,0785431	38,3976	1,41209	0,102145	0,505877	0,02592	0,6279808
CG5930-RA	TfIIA-L	10,6661	0,0694458	14,9715	0,0918324	4,72434	0,093746	-0,107583	0,573476	0,6279808
CG5930-RB	TfIIA-L	15,3808	5,82561	17,5378	4,43062	21,7589	4,01466	-0,107515	0,573954	0,6279808
CG5930-RC	TfIIA-L	0,0428322	1,3567	0,0411215	3,00825	0,0640469	0,881318	-0,094817	0,618939	0,6279808
CG5931-RA	l(3)72Ab	5,84642	5,24919	7,57201	25,5808	3,26638	34,4742	-0,068692	0,811691	0,6279808
CG5932-RA	mag	2,12564	1,17407	1,91399	0,0705085	0,867507	0,0408526	-0,458697	0,19649	0,6279808
CG5933-RA	Mettl3	1,69518	1,34697	2,28539	3,29219	30,0388	122,521	-0,228439	0,444271	0,6279808
CG5934-RA	CG5934	28,4084	0,00988175	0,0104154	3,03973	21,5332	29,2882	0,280998	0,199857	0,6279808
CG5935-RA	Dek	0,0354167	0,0230629	0,0340022	18,1635	0,0895623	0,0178871	0,1561	0,546421	0,6279808
CG5935-RB	Dek	19,7122	0,0153785	25,3932	0,017508	353,235	7,75287	0,155599	0,54765	0,6279808
CG5935-RC	Dek	0,026224	13,2622	0,0251766	0,336219	0	0	0,1561	0,546421	0,6279808
CG5935-RD	Dek	2,68388	0,0153825	4,78875	0	0	0	-0,190834	0,478131	0,6279808
CG5937-RA	CG5937	1,32481	1,42169	1,06077	2,1059	0,751202	0,406459	-0,268342	0,367389	0,6279808
CG5937-RB	CG5937	0,0110169	0,010035	0,0105769	0,709795	0,4062	0,589664	-0,263585	0,37641	0,6279808
CG5938-RA	CG5938	0,0444058	0,0404479	11,6528	12,9338	7,97861	18,2322	0,162798	0,466873	0,6279808
CG5938-RB	CG5938	13,2797	18,5673	13,7708	0,0423055	6,47237	0,0507459	0,156657	0,48408	0,6279808
CG5938-RC	CG5938	13,289	15,7791	0,0430177	20,7585	10,0542	0,0806028	0,241162	0,287744	0,6279808
CG5938-RD	CG5938	0,0448073	0,0408136	0,0611734	0,04971	0,0673325	20,5017	0,233491	0,302832	0,6279808
CG5938-RE	CG5938	0,0637183	0,058039	23,7313	0,0789575	0,106948	2,21165	0,174971	0,439524	0,6279808
CG5939-RA	Prm	0,0213033	0,0149767	0,0157855	0	2,41461	0,0173832	0,026055	0,898423	0,6279808
CG5939-RB	Prm	228,839	4,84822	3,34475	3,46989	0	3,10679	-0,043728	0,829989	0,6279808
CG5939-RC	Prm	0,0247413	7,643	4,37449	78,4803	1,2731	2,6494	0,2375	0,263537	0,6279808
CG5939-RD	Prm	0,0205609	5,72895	3,19071	4,64274	0,023065	1,45214	0,183645	0,384354	0,6279808
CG5940-RA	CycA	7,10887	6,83216	3,95773	5,98175	2,16872	1,02354	-0,132294	0,650354	0,6279808
CG5940-RB	CycA	10,6727	6,61967	0,341058	7,45947	0,853844	2,64261	-0,059032	0,839291	0,6279808
CG5941-RA	MCTS1	20,4709	16,4207	27,8232	18,3398	21,2216	23,7268	-0,117847	0,552129	0,6279808
CG5942-RA	brm	0	4,98253	0	0,012315	0,0166807	0,0125716	0,102021	0,724634	0,6279808
CG5942-RB	brm	0	6,23367	0	4,72423	0,0173892	2,56493	0,101923	0,724889	0,6279808
CG5942-RC	brm	0	0,387165	0	13,7644	4,98679	4,54894	0,102021	0,724634	0,6279808
CG5942-RD	brm	0	21,2211	0	0,0128631	0,0174232	0,0131312	0,102021	0,724634	0,6279808
CG5945-RA	CG5945	0,607184	8,44343	0,685803	18,1177	16,9266	1,11717	-0,531701	0,136329	0,6279808
CG5946-RA	CG5946	24,1914	17,3347	46,8174	32,1388	61,5848	55,3932	-0,639703	0,044714	0,6279808
CG5946-RB	CG5946	6,63428	4,81157	10,0467	10,3883	9,5037	9,63772	-0,645548	0,042205	0,6279808
CG5946-RC	CG5946	0,0625719	0,0569948	0,0600727	0,0771999	0,104568	0,0788085	-0,739686	0,020433	0,6279808
CG5946-RD	CG5946	0,056685	0,0516326	0,0544209	0,0648208	0,0878002	0,0661715	-0,747368	0,018675	0,6279808
CG5948-RA	CG5948	1,21911	9,45678	1,6776	8,62408	10,3268	6,1405	0,184618	0,596835	0,6279808
CG5949-RA	DNApol-delta	1,29669	1,06567	48,9504	1,55173	38,0581	45,1044	0,083076	0,757636	0,6279808
CG5952-RA	Fer2	4,91636	0,607117	3,25356	2,93643	0,0156237	19,5805	0,265273	0,265792	0,6279808
CG5953-RA	CG5953	1,98616	0,0115043	0,0130457	0,0129644	0,0248366	0,0142524	-0,495763	0,060657	0,6279808
CG5953-RB	CG5953	7,48906	1,41101	0,0130296	0,0139793	16,2254	2,88176	-0,488375	0,06511	0,6279808
CG5954-RA	l(3)mbt	6,09231	0,0390145	5,72157	0,0472843	2,47197	0,0482696	0,309283	0,123372	0,6279808
CG5954-RB	l(3)mbt	1,1619	6,86419	1,92245	8,52721	1,51171	6,02569	0,306682	0,137452	0,6279808
CG5955-RA	CG5955	2,3527	1,45907	5,74295	2,33663	0,0557984	8,83677	-0,256927	0,434728	0,6279808
CG5958-RA	CG5958	98,6787	109,805	0,0695071	23,892	401,796	0,186369	-0,196727	0,471858	0,6279808
CG5959-RA	Mco3	2,33797	5,54266	2,53871	0	0,0614454	0,600403	0,079632	0,788022	0,6279808
CG5961-RA	CG5961	5,00454	3,63226	10,9493	2,92064	20,6209	3,66405	-1,16162	0,000299	0,6279808
CG5962-RA	Arr2	0,750375	7,98851	1,00857	0,0554401	0,799714	0,979268	0,235794	0,471113	0,6279808
CG5964-RA	CG5964	0,51639	0,610472	0,611795	0,0367183	12,0889	4,40905	0,060141	0,85931	0,6279808
CG5965-RA	woc	8,20256	28,0231	36,7691	32,4859	5,19111	6,62806	0,041674	0,869706	0,6279808
CG5965-RB	woc	1,22048	9,53221	9,44889	11,1649	0,0150215	0,0113211	0,041939	0,86889	0,6279808
CG5966-RA	CG5966	9,726	6,91613	12,1676	12,492	9,81442	11,2955	-0,662996	0,010171	0,6279808
CG5968-RA	CG5968	0	0	0	0,562625	0	0,266556	-0,131419	0,365453	0,6279808
CG5969-RA	CG5969	31,7632	27,1904	36,8858	34,6824	0,0542056	34,8203	-0,103735	0,661786	0,6279808
CG5970-RA	cbc	7,99594	1,2271	5,35841	1,87012	3,94577	6,61031	-0,225169	0,346434	0,6279808
CG5971-RA	Cdc6	4,42271	4,30593	7,70395	7,03674	0,258687	0,116472	0,108601	0,687839	0,6279808

gene_id	Symbol	W1_FPKM	W2_FPKM	W3_FPKM	MCM51_FPKM	MCM52_FPKM	MCM53_FPKM	FC	p-value	p-adj
CG5972-RA	Arpc4	36,1178	0	0	2,24347	1,09389	1,24145	-0,335888	0,250722	0,6279808
CG5973-RA	CG5973	12,5979	32,9624	0,0454852	0,0529029	0,466889	0,333688	0,455003	0,090277	0,6279808
CG5973-RB	CG5973	9,33336	4,01968	55,1345	57,9245	0,165239	0,220304	0,462354	0,087023	0,6279808
CG5973-RC	CG5973	51,3913	0,0376636	89,4283	78,1545	0,357897	93,7401	0,462914	0,086355	0,6279808
CG5974-RA	pll	5,90881	16,8502	7,18556	10,9832	5,8827	12,977	-0,071515	0,724152	0,6279808
CG5976-RA	isoQC	10,1639	9,00551	11,7983	9,19731	0,0681552	8,68348	0,119346	0,607851	0,6279808
CG5976-RB	isoQC	4,50517	2,91907	5,15014	6,4545	126,509	4,22805	0,171962	0,423328	0,6279808
CG5977-RA	spas	0,0241006	6,81538	9,9914	10,4745	5,03354	3,51296	0,208585	0,393484	0,6279808
CG5977-RB	spas	7,7453	10,5799	7,16945	0,048574	125,547	6,72755	-0,036007	0,890021	0,6279808
CG5978-RA	GAPsec	12,9949	0,0169174	0,017831	0,0193315	9,24173	12,1404	0,195795	0,352631	0,6279808
CG5983-RA	ACXC	0	0	0,0551358	12,4291	0	0	-0,153617	0,499267	0,6279808
CG5983-RB	ACXC	0	0,0171537	0	0,0196134	0	0,0200221	-0,153617	0,499267	0,6279808
CG5984-RA	CG5984	3,40096	5,05242	4,47012	6,20746	6,16295	5,79753	-0,29827	0,266388	0,6279808
CG5986-RA	CG5986	15,5988	14,0187	9,31272	22,3146	20,6828	26,1697	-0,239286	0,39148	0,6279808
CG5987-RA	TTLL6B	0,719665	0,558406	1,06197	0,862358	0,396187	0,797314	0,054961	0,867757	0,6279808
CG5988-RB	upd2	0,267153	6,3063	3,65228	5,06066	4,71815	0	-0,396621	0,259549	0,6279808
CG5989-RA	Dvir\GJ12311	10,7801	0,283765	0,448634	0,41214	0,517448	14,4578	-0,188206	0,385606	0,6279808
CG5991-RA	Pisd	0,0323637	0,0294791	28,3766	0,0348169	0,0471597	0,0355424	-0,224978	0,307143	0,6279808
CG5991-RB	Pisd	6,68887	12,1695	11,8918	18,4457	13,0999	9,28413	-0,213381	0,333822	0,6279808
CG5991-RC	Pisd	12,5642	14,9134	0,0204591	15,3931	10,234	11,7595	-0,214554	0,331501	0,6279808
CG5992-RA	Adgf-A	4,70332	12,446	11,0745	0,24313	0,0373509	2,66163	-0,19876	0,450165	0,6279808
CG5992-RB	Adgf-A	16,9517	0,0231843	17,4964	0,0636675	18,8492	0,458853	-0,19876	0,450165	0,6279808
CG5993-RA	upd1	0,412084	0,0266705	1,055	4,5413	3,72687	0,0298355	0,357122	0,271898	0,6279808
CG5993-RB	upd1	0,527045	1,56454	0,534105	0,537686	1,15118	2,73308	0,32959	0,309837	0,6279808
CG5994-RA	Dvir\Nelf-E	0,0565395	4,91825	8,08791	9,21488	26,432	25,3931	-0,780322	0,004088	0,6279808
CG5996-RA	Trpgamma	0,673245	0,644686	0,0165732	0,0179125	0,0242627	0,0182858	0,115488	0,714677	0,6279808
CG5996-RB	Trpgamma	0,0170489	0,0155293	0,531959	0,458014	0,66182	0,321186	0,126942	0,686693	0,6279808
CG5996-RC	Trpgamma	0,0168618	0,0153589	0,0161884	0,0174801	0,0236769	0,172768	0,15307	0,629525	0,6279808
CG5998-RA	Adgf-B	0,0390058	0,334369	14,8539	0,816726	28,6619	0,0281499	0,447584	0,074924	0,6279808
CG5999-RA	Ugt37A3	1,5027	0,717855	0,0997434	0,149917	0,203064	0,155159	-0,086779	0,807893	0,6279808
CG6000-RA	CG6000	0,12259	0,111663	0,117693	1,52773	0,265216	0,199883	-0,745716	0,001563	0,6279808
CG6000-RB	CG6000	0,112374	0,102358	0,107886	33,6375	0,231507	0,174478	-0,741051	0,001625	0,6279808
CG6004-RB	Muc68D	0	31,9144	0,0314718	95,5206	0	9,00338	-0,219028	0,503883	0,6279808
CG6005-RA	CG6005	8,9367	10,1694	10,2325	11,1526	13,427	11,797	-0,260412	0,345729	0,6279808
CG6006-RB	CG6006	5,82297	12,8573	109,482	64,9565	6,20957	11,8021	0,156751	0,449573	0,6279808
CG6006-RC	CG6006	1,90021	4,44852	5,035	3,67568	7,49293	0,820835	0,279679	0,185519	0,13772387
CG6007-RA	GatA	5,68379	0,029756	71,8904	3,2628	0,0264955	0,0199686	0,277894	0,241765	0,6279808
CG6008-RA	NP15.6	93,8895	55,4888	12,8643	10,1418	0,0476379	0,0359028	-0,450792	0,151345	0,6279808
CG6009-RA	P5cr	12,1077	20,5199	0,0313629	0,0195262	15,8655	15,1925	-0,019448	0,949773	0,6279808
CG6011-RA	Prp18	10,5234	6,68707	52,8988	11,9909	15,74	13,6172	-0,230698	0,369908	0,6279808
CG6012-RA	CG6012	2,85117	34,6132	9,40849	11,9843	0,0413864	0,0311913	-0,129858	0,704678	0,6279808
CG6013-RA	CG6013	5,96685	5,50798	10,765	6,23896	7,50293	8,66629	0,030352	0,919758	0,6279808
CG6014-RA	rgn	23,0373	22,7922	34,9295	24,0708	30,2751	24,61	-0,03882	0,869993	0,6279808
CG6015-RA	CG6015	15,2411	14,493	19,8539	19,5696	1,17123	15,4841	-0,017538	0,931002	0,6279808
CG6016-RA	bbc	11,1815	7,65674	8,96563	8,47065	4,14719	4,36716	0,103443	0,563353	0,6279808
CG6016-RB	bbc	24,0812	25,1372	0,0132722	30,193	0,0192744	0,0151207	0,085258	0,634056	0,6279808
CG6017-RA	Hip14	9,98099	10,3205	1,60058	12,8647	0,0275506	0,0207638	0,036564	0,869862	0,6279808
CG6018-RA	gas	7,67891	7,95178	10,3504	8,49804	4,93972	4,5592	-0,03257	0,886368	0,6279808
CG6019-RA	mus308	0,269624	0,225945	0,460762	0,151992	0,205874	0,0266657	-0,51999	0,039607	0,6279808
CG6020-RA	Dpse\GA19302	84,1189	82,4909	158,828	128,305	7,4892	170,361	-0,581567	0,046728	0,6279808
CG6022-RA	Cchl	63,2901	0,033762	60,003	32,674	49,6513	57,5759	-0,471382	0,078325	0,6279808
CG6023-RA	CG6023	0,0503371	0,0458505	0,894042	2,02694	3,80717	0,253559	0,024544	0,933892	0,6279808
CG6023-RB	CG6023	1,6953	1,52182	1,88706	1,61341	0,717267	0,531863	0,019542	0,947315	0,6279808
CG6024-RC	CG6024	16,2309	15,1947	2,86625	19,7515	1,68326	10,1103	-0,278247	0,309293	0,6279808
CG6024-RD	CG6024	4,9004	5,2324	1,72916	3,37201	1,70292	1,46946	0,157688	0,525753	0,6279808
CG6024-RE	CG6024	0,0215254	0,0196068	3,34577	0,0225616	2,41664	0,0230318	0,156384	0,53046	0,6279808
CG6025-RA	Arl1	32,4359	26,6658	8,9963	41,7228	7,02304	7,44558	-0,069768	0,779716	0,6279808
CG6026-RB	CG6026	14,056	0,0354107	11,9188	256,803	70,3511	68,3135	0,244719	0,489296	0,6279808
CG6027-RA	cdi	15,6503	18,9935	199,386	20,91	93,5683	108,557	0,416204	0,094545	0,6279808
CG6028-RA	CG6028	54,996	0,0375071	84,4807	83,6622	119,885	84,6304	-0,367677	0,232936	0,6279808
CG6030-RA	Dpse\GA22433	211,859	0,0877778	28,4719	0,019561	5,39384	2,99003	-0,229249	0,457382	0,6279808
CG6030-RB	Dpse\GA22433	0,0963671	5,18666	6,31923	0,03517	0,0447704	411,444	-0,229232	0,45745	0,6279808
CG6033-RA	drk	0,020306	2,87165	40,8786	37,6552	0,0198224	0,026508	0,442698	0,05771	0,6279808
CG6033-RB	drk	0,0259834	0,247071	8,04226	0,0216641	0,0205338	0,027249	0,443284	0,057499	0,6279808
CG6033-RC	drk	0,0256805	0	7,11672	6,65784	0,329322	24,7052	0,443063	0,057557	0,6279808
CG6033-RD	drk	0,0241153	0,1153	103,38	88,4818	0,33177	0,0221155	0,431928	0,065096	0,13772387
CG6033-RE	drk	0,0247383	0	0,0609578	0,156763	0,230614	10,9116	0,466476	0,046176	0,6279808
CG6033-RF	drk	51,6452	3,1546	10,828	15,8663	12,6185	0,0294695	0,460144	0,0497	0,6279808
CG6034-RA	CG6034	0,390031	0,0177699	13,8906	0,0388971	0	0,0425586	-1,224682	0,000205	0,6279808
CG6036-RA	CG6036	0	0,132569	0,0838372	9,14754	7,91364	0,108198	0,056679	0,777169	0,6279808
CG6038-RA	crim	60,4703	12,5826	8,79746	0,0516182	45,4061	0,0517612	0,07884	0,707029	0,6279808
CG6040-RA	CG6040	15,0435	4,74455	0,0296064	113,377	14,0556	50,5741	0,236902	0,446612	0,6279808
CG6041-RA	CG6041	13,503	7,27927	0,384286	0,81306	0,485568	0,579521	-0,635047	0,060815	0,6279808
CG6042-RA	Cyp12a4	11,0774	210,341	9,98915	0,0330529	6,19928	13,7957	0,02203	0,948893	0,6279808
CG6043-RC	CG44085	0,0185415	1,94867	4,28445	19,0904	4,46938	0,263769	-0,041691	0,857265	0,6279808
CG6043-RD	CG44085	13,1314	3,87523	14,1024	4,70052	4,74563	4,45837	-0,046708	0,840393	0,6279808
CG6043-RF	CG44085	0,0177258	0,0286511	0,0582701	0,0343988	0,094996	0,0715947	-0,045466	0,844676	0,6279808
CG6043-RG	CG44085	25,6727	18,0956	24,3449	0	28,8296	30,5218	-0,046918	0,839656	0,6279808
CG6043-RH	CG44085	0,0170094	0,0168889	179,873	7,17447	151,897	173,861	-0,049479	0,832464	0,6279808
CG6044-RA	CG6044	4,21417	2,44657	9,76493	0,370712	11,3445	2,32171	0,18021	0,534199	0,6279808
CG6044-RD	CG6044	2,86761	4,06318	0,0280225	4,77277	5,54253	1,30244	0,168106	0,567295	0,6279808
CG6045-RA	AOX3	4,15557	0,0158191	0,258438	0,219081	50,1344	35,1167	1,051565	3,49E-05	0,6279808
CG6046-RA	Bin1	33,5978	25,8646	42,0968	56,8087	0,114866	0,178175	-0,212942	0,38536	0,6279808
CG6048-RA	CG6048	4,91474	1,24352	12,7864	14,6503	15,0499	0,0124714	-0,405524	0,257156	0,6279808
CG6049-RA	barc	0,0283709	0,0258422	0,0272378	0,0309857	0,0419703	0,0316313	0,057641	0,782319	0,6279808
CG6049-RB	barc	0,0284197	0,0258867	0,0272846	0,0310441	0,0420494	0,031691	0,057641	0,782319	0,6279808
CG6049-RC	barc	7,78036	6,98267	0,0274618	9,18073	0,0423487	0,0319165	0,057641	0,782319	0,6279808
CG6050-RA	mEFTu1	63,7133	58,6818	0,0133955	0,0143664	0,0381328	0,0155867	-0,217984	0,368194	0,6279808
CG6051-RB	CG6051	16,5587	9,95288	0,588589	0,320905	0,337477	0,0564055	0,004183	0,98869	0,6279808
CG6052-RA	CG6052	0,0323648	0,163847	0,0170774	0,0287797	0	0,0417399	-0,308435	0,296302	0,6279808
CG6053-RA	Dnai2	0,527702	0,021185	0,52472	0,024973	0,833041	0,888343	-0,653645	0,05451	0,6279808
CG6054-RA	Su(fu)	9,1096	36,3989	19,6767	42,8735	49,4198	7,71716	0,272659	0,274368	0,6279808
CG6055-RA	CG6055	61,931	75,2882	84,8438	0,166254	0,0716573	9,31149	-0,013992	0,953603	0,6279808
CG6056-RA	AP-2sigma	54,3612	52,0337	80,4965	61,8163	55,5892	61,4632	0,18664	0,425518	0,6279808
CG6057-RA	SMC1	9,42976	10,3056	29,8516	15,4405	6,01884	6,46685	-0,006097	0,981321	0,6279808
CG6058-RB	Ald1	0,0338908	0,0308701	0,0325372	0,0365959	2,18863	0,0114191	0,228035	0,242825	0,6279808
CG6058-RC	Ald1	524,674	582,022	573,639	475,765	3,08195	2,51647	0,228573	0,241885	0,6279808
CG6058-RD	Ald1	0,0335467	0,0305567	0,0322068	0,0361939	0,012839	0,00967626	0,228012	0,243193	0,6279808
CG6058-RE	Ald1	0,0384236	0,0349989	0,036889	0,0419553	0,0123334	2,61432	0,15689	0,441453	0,6279808
CG6058-RH	Ald1	170,754	297,562	269,526	242,44	9,50877	7,39706	0,158142	0,437933	0,6279808
CG6058-RI	Ald1	0,0405705	0,0369544	0,0389501	0,0445358	19,759	17,2881	0,15726	0,440293	0,6279808
CG6059-RA	CG6059	0	0,347096	0,341451	0,0304276	16,8507	2,06489	0,126054	0,484645	0,6279808
CG6061-RA	mip120	5,90543	5,57025	4,34029	3,72945	0,0292479	1,60054	0,233099	0,257358	0,6279808
CG6064-RA	Crtc	5,7198	0,706414	6,40324	73,3281	20,4802	21,9034	-0,086274	0,725088	0,6279808

gene_id	Symbol	W1_FPKM	W2_FPKM	W3_FPKM	MCM51_FPKM	MCM52_FPKM	MCM53_FPKM	FC	p-value	p-adj
CG6066-RA	CG6066	3,72419	3,12171	9,51991	3,00955	0,771503	0,0310617	0,147636	0,63985	0,6279808
CG6067-RA	CG6067	2,46377	3,29145	4,84336	1,95221	2,68784	10,8661	0,527417	0,080309	0,6279808
CG6070-RA	gb	6,67586	4,89461	8,12078	8,58199	4,45052	4,9824	0,187206	0,490478	0,6279808
CG6071-RA	CG6071	0,560353	0,322911	0,603846	13,5397	0,562509	5,52091	-0,277757	0,404896	0,6279808
CG6072-RA	sra	17,4695	19,8906	21,3594	25,655	1,08913	52,3605	-0,375149	0,096539	0,6279808
CG6073-RA	CG6073	12,2097	0,0156464	255,228	0,0325629	0,179815	16,4038	-0,242802	0,433716	0,6279808
CG6074-RA	CAH9	1,29236	0,181103	6,93542	5,57589	0,0412144	9,44669	-0,14043	0,692089	0,6279808
CG6081-RA	Cyp28d2	8,90729	7,41267	18,6384	5,83909	7,63964	6,13426	0,564115	0,061323	0,6279808
CG6083-RA	CG6083	1,63679	0,0187692	0,0197828	0,823411	0,0453851	0,0219998	-0,256893	0,472857	0,6279808
CG6084-RA	CG6084	0,0507624	0,0275117	0,0289974	7,43405	6,22226	0,0583631	-0,440402	0,138683	0,6279808
CG6084-RB	CG6084	108,915	6,12435	14,2536	11,6879	5,98742	227,859	-0,427602	0,149685	0,6279808
CG6090-RA	RpL34a	74,2168	1,91683	0,765282	273,822	5,29393	0,534808	-0,140398	0,626486	0,6279808
CG6090-RB	RpL34a	173,866	0,0126054	0,0132861	0,827021	0,0232513	0,0145421	-0,140398	0,626486	0,6279808
CG6091-RD	Duba	0,0206058	15,9579	12,8213	0,0215507	1629,21	9,22842	0,031403	0,891732	0,6279808
CG6091-RE	Duba	13,5672	0,028448	0,0299843	17,628	2,90447	0,0342049	0,158935	0,498296	0,6279808
CG6091-RF	Duba	0,0312317	4,55745	2,82855	0,0335067	8,20978	2,90666	0,158935	0,498296	0,6279808
CG6092-RA	Dak1	62,6718	0,0920749	0,0970473	59,8014	0,176902	0,133324	-0,30055	0,307696	0,6279808
CG6092-RB	Dak1	0,0317113	2,5102	7,99882	0,034061	2,92963	2,91791	-0,2995	0,309405	0,6279808
CG6093-RA	abo	2,17771	10,6385	3,43095	15,649	1,06173	1,59525	0,530027	0,077251	0,6279808
CG6094-RA	CG6094	16,2731	14,3747	30,1303	18,14	22,2491	26,0117	-0,036195	0,903677	0,6279808
CG6095-RA	Exo84	0,0304123	4,53251	0	8,96836	0	316,566	0,011065	0,957016	0,6279808
CG6095-RB	Exo84	7,41564	11,921	16,1634	0,0178205	16,9708	0	0,011065	0,957016	0,6279808
CG6096-RA	E(spl)m5-HLH	3,6916	3,09084	19,9188	4,40126	8,9598	13,2485	0,419188	0,200049	0,6279808
CG6097-RA	rt	11,8981	71,0065	0	7,36537	0,0749295	0,0692502	-0,001059	0,997423	0,6279808
CG6098-RA	Lrr47	3,37841	3,68142	3,64145	0,209093	3,18176	2,79389	0,014263	0,952208	0,6279808
CG6099-RA	Dsim\m4	10,4368	17,184	168,037	22,6888	2,21089	9,63595	0,288285	0,364499	0,6279808
CG6103-RD	CrebB	0,057574	36,9543	0,0552745	0,0659893	26,1737	4,72476	-0,440318	0,02781	0,6279808
CG6103-RE	CrebB	4,75396	0,0524424	5,25487	7,05261	0,191767	0,169637	-0,440318	0,02781	0,6279808
CG6103-RF	CrebB	0,121092	5,64101	0,116255	0,166175	0,0893829	0,100317	-0,508236	0,009771	0,6279808
CG6103-RG	CrebB	0,0806129	0,110299	0,0773932	0,0982694	4,05189	0,0831948	-0,511253	0,009222	0,6279808
CG6103-RH	CrebB	2,44938	0,0734278	5,43175	0,0814966	0,225085	0,0753649	-0,433973	0,030393	0,6279808
CG6103-RI	CrebB	0,0634327	3,45658	0,0608992	0,0738265	0,133107	0,109816	-0,443892	0,024304	0,6279808
CG6103-RJ	CrebB	0,0867515	0,0577789	0,0832866	0,107575	0,110388	10,2387	-0,438878	0,026149	0,6279808
CG6103-RK	CrebB	8,50735	0,0790193	7,36795	20,4909	0,0999984	3,90801	-0,552685	0,006325	0,6279808
CG6103-RL	CrebB	0,0534306	7,77618	0,0512965	3,95505	0,14571	0,203246	-0,512391	0,022867	0,6279808
CG6104-RA	E(spl)m2-BFM	14,3897	0,00680868	18,9343	16,171	0,0102699	4,11799	0,67509	0,011249	0,6279808
CG6105-RA	Dpse\GA19355	0,0895337	0,0815535	0,0859577	0,123042	0,166662	0,125606	-0,35023	0,254404	0,6279808
CG6105-RB	Dpse\GA19355	307,188	299,256	502,812	336,754	645,034	651	-0,35023	0,254404	0,6279808
CG6105-RC	Dpse\GA19355	0,05996	0,0546157	0,0575651	0,073252	0,0992203	0,0747784	-0,352754	0,249788	0,6279808
CG6106-RA	CG6106	4,55393	5,82967	4,15259	5,52369	0,983301	5,33954	-0,076518	0,769111	0,6279808
CG6108-RA	CG44085	4,24462	28,672	7,9728	7,8904	9,00308	11,2222	-0,33935	0,117423	0,6279808
CG6113-RA	Lip4	23,6224	0,142285	0,0749844	44,2486	0	0,152387	0,391322	0,117636	0,6279808
CG6114-RA	sff	12,1569	0,640361	7,82826	11,2413	5,83747	112,25	0,476279	0,096052	0,6279808
CG6115-RA	CG6115	88,2886	115,251	0,900453	12,0967	112,002	119,51	0,113066	0,635063	0,6279808
CG6116-RA	Uvrag	10,0876	11,6444	9,7226	13,9251	0,0170439	8,93056	-0,09614	0,645007	0,6279808
CG6117-RA	Pka-C3	19,9766	18,5205	21,2674	30,9526	18,7286	4,39547	0,425075	0,052343	0,6279808
CG6117-RB	Pka-C3	0,0307807	27,6445	0,0295513	19,2008	13,4394	13,9491	0,381858	0,092209	0,6279808
CG6118-RB	CG6118	0,0165659	0	0,0928343	0,332488	0	1,64476	0,302425	0,377258	0,6279808
CG6118-RC	CG6118	39,5016	10,0098	0,0159043	2,76149	6,78621	5,89138	0,300101	0,380952	0,6279808
CG6120-RA	Tsp96F	38,4876	40,3307	24,3423	31,4097	27,34	24,2462	0,201726	0,445941	0,6279808
CG6121-RA	Tip60	6,95684	8,14544	9,34246	9,59398	22,3321	5,70863	0,272311	0,239946	0,6279808
CG6122-RA	piwi	0,0219109	0,0199579	0,0210357	0,0229866	0,0311355	0,0234656	0,550353	0,01654	0,6279808
CG6123-RA	CG6123	0,972822	1,16777	1,02882	2,26219	0,0395874	1,04312	-0,032756	0,910109	0,6279808
CG6124-RA	eater	0,312627	0,0610204	0,362911	0,0205109	5,0697	30,2495	-0,218775	0,507026	0,6279808
CG6125-RA	CG6125	2,34115	0,0619414	0,0652864	0,0138821	0,308646	0,85237	-1,025293	0,000451	0,6279808
CG6125-RB	CG6125	0,832241	0,0226634	0,0238873	4,37615	0,0460169	1,21655	-1,025456	0,00046	0,6279808
CG6126-RA	CG6126	25,8727	16,9975	0,0492266	22,364	49,1955	42,8065	-0,47116	0,148435	0,6279808
CG6127-RA	Ser	6,45644	0,62161	4,73034	0,502912	24,1388	23,6469	-0,493516	0,075096	0,6279808
CG6128-RA	Fuca	3,08009	5,7226	0,0183308	10,4391	0,0269516	0,0203123	-0,33259	0,202332	0,6279808
CG6128-RB	Fuca	0,773282	0,686967	399,628	0,0198977	6,04399	36,1473	-0,445353	0,09367	0,6279808
CG6129-RB	Root	4,37082	4,85429	4,65875	6,29792	6,27544	3,64628	-0,068661	0,73398	0,6279808
CG6130-RA	h-cup	0,0442966	0,0403484	54,6002	82,8052	1,6395	0,0501033	-0,334055	0,300482	0,6279808
CG6131-RA	Cpr97Ea	16,6064	17,8927	0,775611	0,674041	5,41402	0,549134	0,809594	6,65E-05	0,6279808
CG6132-RA	Sgs8	3,03691	2,76623	5,12442	8,09263	1390,63	1067,58	-0,736759	0,029209	0,6279808
CG6133-RA	Nsun2	10,9363	7,40983	20,7404	15,948	11,3683	14,0624	-0,180795	0,53429	0,6279808
CG6134-RA	spz	0,0404216	0,0368188	0,0388071	0,0443559	0,0600804	0,0452802	0,496422	0,022439	0,6279808
CG6134-RB	spz	0,0399089	0,0363518	0,0383149	0,0437376	0,0592429	0,044649	0,495248	0,022414	0,13772387
CG6134-RC	spz	5,85731	4,61381	0,0353671	6,39585	4,07965	3,71005	0,487433	0,024803	0,6279808
CG6134-RD	spz	0,03283	6,54894	7,75361	5,65087	1,63471	3,09691	0,483319	0,0253	0,6279808
CG6134-RE	spz	0,0343668	0,0313036	0,0329941	0,037153	0,050324	0,0379272	0,504434	0,022124	0,6279808
CG6134-RF	spz	6,2864	3,75214	10,2996	3,23426	2,61977	3,36368	0,49604	0,021847	0,6279808
CG6134-RG	spz	0,0353032	0,0321566	0,0338932	0,0382531	3,01794	0,0390502	0,484687	0,025019	0,6279808
CG6134-RH	spz	0,0342066	0,0311578	0,0328404	0,0369654	0,0500699	0,0377357	0,49267	0,022527	0,6279808
CG6134-RI	spz	4,81978	0,0287882	0,0303428	3,15967	0,0459695	0,0346454	0,48347	0,024799	0,6279808
CG6134-RJ	spz	0,035531	0,0323641	0,0341119	0,0385214	0,0521775	0,0393241	0,48723	0,024788	0,6279808
CG6134-RK	spz	0,0370865	0,033781	0,0356053	0,0403619	0,0546705	0,041203	0,483444	0,02604	0,6279808
CG6134-RL	spz	4,3658	3,62758	0,0319957	0,0359372	0,0486772	1,79485	0,495058	0,022392	0,6279808
CG6136-RA	CG6136	0,0316354	0,0558316	10,4453	5,99249	29,1447	7,15842	-0,2059	0,445436	0,6279808
CG6137-RA	aub	0,971093	0,601033	0,0239053	0,049044	20,525	0,050066	-0,218019	0,474429	0,6279808
CG6137-RC	aub	0,391098	0,615322	2,25287	8,5582	0,0332217	0,0250379	-0,213764	0,487319	0,6279808
CG6138-RA	CG6138	0,136898	0,202876	0	121,705	0	0	0,190739	0,361763	0,6279808
CG6140-RA	Fum3	0,102221	0,378531	0	0,0277846	1,6645	0	-1,524014	2,06E-05	0,6279808
CG6141-RA	RpL9	0,0916869	13,2817	13,9674	0	1,35794	1,73854	-0,531877	0,070399	0,6279808
CG6141-RB	RpL9	1504,64	0	0,112079	2,24517	0,369561	0	-0,531877	0,070399	0,6279808
CG6142-RA	CG6142	11,0483	0,373584	0,643459	65,6041	10,2364	0,323341	0,209295	0,475342	0,6279808
CG6143-RA	Pep	41,1061	51,25	52,411	1,90486	24,2272	32,6195	-0,109993	0,681001	0,6279808
CG6143-RB	Pep	21,2206	26,661	34,2902	66,8136	17,0075	23,3764	-0,122108	0,648398	0,6279808
CG6143-RC	Pep	7,53163	0,0221301	13,3303	52,3859	6,55141	14,2556	-0,120444	0,653	0,6279808
CG6144-RA	CG6144	0,0412287	0,480942	20,0597	16,3004	3,40924	7,67495	0,347641	0,170548	0,6279808
CG6144-RB	CG6144	3,88366	0,0375539	0,0201408	0,0453325	4,35054	1,7027	0,349552	0,168389	0,6279808
CG6145-RA	CG6145	0,0361268	0,632673	0,937442	30,3773	0,163606	0,526668	-0,021385	0,918094	0,6279808
CG6145-RB	CG6145	33,6066	0,0263206	0,0462367	13,4246	0,0414795	0,0503557	-0,000579	0,997793	0,6279808
CG6145-RC	CG6145	9,89956	0	0	12,9084	0	0	-0,018156	0,93011	0,6279808
CG6145-RE	CG6145	0,0304217	46,2426	0,0346839	0,0405857	0,0548721	0,041355	-0,000301	0,99885	0,6279808
CG6145-RF	CG6145	0,0361861	7,90413	35,2669	5,70143	27,0081	28,0984	-0,005982	0,977238	0,6279808
CG6146-RA	Top1	23,5705	0,0256222	8,03425	1,88489	2,73067	0,0160939	0,140681	0,546358	0,6279808
CG6146-RB	Top1	0,0135217	2,06101	0,0131258	24,1833	1,25276	2,11284	0,017503	0,939483	0,6279808
CG6146-RC	Top1	0,0284116	0,0142083	0,0149756	0,0139085	0,0174062	3,63836	0,133947	0,569517	0,6279808
CG6147-RA	Tsc1	5,91509	6,01401	7,66684	8,76039	5,73708	5,54927	-0,064539	0,758393	0,6279808
CG6148-RA	Past1	0,0361333	0,0329127	5,64384	0	33,8458	5,09021	-0,232364	0,261465	0,6279808
CG6148-RB	Past1	45,1829	60,2762	10,7918	6,72681	20,7632	21,6308	-0,245375	0,23654	0,6279808
CG6149-RB	CG6149	0,140587	0	1,64993	0,106747	0,19877	0,173469	0,01939	0,899027	0,6279808
CG6151-RA	fwe	86,5107	88,7697	77,9023	76,8429	66,7523	65,476	0,212793	0,296676	0,6279808
CG6151-RB	fwe	0,116399	0,106024	0,11175	0,157439	0,213253	0,16072	-0,373379	0,09488	0,6279808

gene_id	Symbol	W1_FPKM	W2_FPKM	W3_FPKM	MCM51_FPKM	MCM52_FPKM	MCM53_FPKM	FC	p-value	p-adj
CG6151-RC	fwe	0,100317	0,091376	0,0963106	22,0146	0,175166	0,132016	-0,348982	0,106507	0,6279808
CG6153-RA	CG6153	0,078289	50,9029	67,2549	0,745644	0	4,31078	0,15431	0,600267	0,6279808
CG6153-RB	CG6153	3,9764	0,071311	0,0751621	2,22108	0,515258	0,845035	0,15431	0,600267	0,6279808
CG6154-RC	CG6154	4,50399	4,21279	3,49414	2,68857	3,21093	2,57762	-0,422441	0,179507	0,6279808
CG6154-RD	CG6154	0,0442373	0,0402944	0,0424705	0,0490076	0,066381	0,0500288	0,330713	0,21014	0,6279808
CG6155-RA	Roe1	44,1649	0,0184962	0,012615	3,73087	0,0376502	0,0145957	-0,430327	0,120502	0,6279808
CG6157-RA	dah	0,0281294	1,71607	0,0326545	0,155854	0,0318352	0,0313366	0,287444	0,421682	0,6279808
CG6159-RA	Sec10	8,98701	8,18599	6,02472	0,0442665	1,48718	0,883564	-0,00841	0,966654	0,6279808
CG6163-RA	CG6163	0,850482	0	0,706598	0	0,349008	0,613778	0,025292	0,935731	0,6279808
CG6164-RA	Npc2f	2,53783	3,27831	9,72369	11,651	4,70084	8,10567	-0,212919	0,507399	0,6279808
CG6167-RA	PICK1	2,20163	4,39495	5,79036	3,74517	3,90738	1,91436	-0,117369	0,597227	0,6279808
CG6167-RB	PICK1	3,47187	0,0367963	5,64298	5,42295	4,58658	0,38833	-0,107807	0,62725	0,6279808
CG6168-RB	Dsim\GD12810	0	0,377883	0,16661	0,978283	0,101381	0,196636	-0,100412	0,70095	0,6279808
CG6169-RA	DCP2	6,01318	22,7633	16,813	22,0256	11,2277	12,5559	-0,097172	0,74752	0,6279808
CG6169-RB	DCP2	0,0242066	8,38803	5,14067	9,30981	4,06375	4,04235	-0,059987	0,841561	0,6279808
CG6169-RC	DCP2	0,0236774	0,022049	0,0232397	0,0255343	0,0345863	0,0260663	-0,064832	0,82877	0,6279808
CG6169-RD	DCP2	8,34008	0,021567	0,0227318	0,0249446	0,0337876	0,0254644	-0,063092	0,833428	0,6279808
CG6170-RA	HDAC6	1,99609	1,38724	0,0323052	0,030697	0,0626092	2,46551	-0,096801	0,632233	0,6279808
CG6170-RB	HDAC6	0,0155987	0,0309813	0,031394	3,77591	0,540493	0,0166764	-0,069602	0,731114	0,6279808
CG6170-RC	HDAC6	7,33317	0,03065	0,031301	0,016336	0,2417	7,67787	-0,068028	0,73714	0,6279808
CG6171-RA	CG6171	13,8186	0,0286239	0,853409	12,8729	4,95396	5,6958	0,196443	0,384687	0,6279808
CG6171-RB	CG6171	0,0680025	3,07437	0,0129577	0,0801094	0,10194	6,34645	0,196443	0,384687	0,6279808
CG6172-RA	vnd	4,70302	7,34021	0,691225	0,047048	0,0637268	5,83302	0,232911	0,45339	0,6279808
CG6172-RB	vnd	0,753211	1,27126	32,2642	0,0464183	0,0628738	18,7196	0,254781	0,410625	0,6279808
CG6173-RA	Kal1	3,11903	5,71293	43,1585	0,129111	1,55861	2,30294	0,495483	0,103425	0,6279808
CG6174-RA	Arp1	32,5679	0,391457	0,876769	299,439	250,753	2,35979	-0,299322	0,23696	0,6279808
CG6175-RB	CG6175	14,3223	0	0,181041	2,48642	0	0,108082	0,202482	0,477402	0,6279808
CG6176-RA	Grip75	2,35571	0,0288019	0,651748	3,03276	1,56993	0,0462771	0,527556	0,013951	0,6279808
CG6177-RA	ldlCp	6,18426	5,31116	7,1141	0,0490806	2,88982	5,70485	-0,316606	0,160412	0,6279808
CG6178-RA	CG6178	17,9677	6,48833	32,1069	12,5512	0,0175052	23,0409	-0,23511	0,368681	0,6279808
CG6179-RA	CG6179	5,24493	5,29266	9,47849	7,51535	5,30896	6,8058	-0,01261	0,96228	0,6279808
CG6180-RA	CG6180	39,2648	23,863	43,334	77,5187	6,66773	70,6958	-0,540193	0,109552	0,6279808
CG6181-RA	Ge-1	0,0141987	3,21299	0,0136316	0,0146279	2,48266	2,7675	-0,016378	0,951987	0,6279808
CG6181-RB	Ge-1	4,09119	5,5476	4,55929	7,01651	4,63275	4,0187	-0,016378	0,951987	0,6279808
CG6182-RA	TBC1d7	8,66195	5,61857	10,8864	0,170916	9,10918	1,34379	-0,262149	0,355231	0,6279808
CG6184-RA	CG6184	0,020184	0,018385	0,160194	0,0210886	0,0285646	0,00622964	-0,10777	0,634564	0,13772387
CG6184-RC	CG6184	7,31741	9,86529	0,112238	13,34	6,64183	0,00649805	-0,093385	0,684075	0,6279808
CG6184-RD	CG6184	0,0203519	0,0185379	4,45904	0,0212724	0,0288136	2,24133	-0,105467	0,642341	0,6279808
CG6185-RA	Ir68a	0,81228	4,20709	4,87772	8,5201	0,221016	0,422048	0,635814	0,058587	0,6279808
CG6186-RA	Tsf1	0,214104	0,165018	0,869647	0,836092	0,11957	0,0673644	0,085018	0,804361	0,6279808
CG6187-RA	RluA-2	4,05466	3,2829	5,44659	13,507	0,0614031	7,38039	0,350454	0,158144	0,6279808
CG6188-RA	Gnmt	5,59052	3,77489	19,6906	44,5532	16,1766	0,977645	-0,357415	0,317255	0,6279808
CG6189-RA	Mybbp1A	7,27139	5,15052	8,95152	9,87069	6,75118	7,07097	0,024269	0,926926	0,6279808
CG6190-RA	Ube3a	7,6525	7,92876	8,69364	9,88015	6,47638	5,3008	0,198546	0,384831	0,6279808
CG6191-RA	CG6191	10,9075	0,0485505	14,0328	1,60641	6,81897	12,0954	-0,334395	0,086132	0,6279808
CG6192-RA	Reps	4,92448	0,840653	3,39769	5,50252	0,0198136	0,0149327	-0,138297	0,59267	0,6279808
CG6193-RA	Apc2	1,70152	1,81066	3,4242	2,33601	1,39388	9,60941	0,249166	0,304848	0,6279808
CG6194-RA	Atg4b	6,17645	56,163	69,6306	6,34937	4,93122	4,60204	0,043835	0,832035	0,6279808
CG6195-RA	CG6195	10,3534	18,1353	22,9806	4,78221	18,9892	19,2471	-0,672711	0,036668	0,6279808
CG6196-RA	Trs33	13,5278	14,0301	30,2757	17,0611	5,95259	5,49231	-0,106263	0,664114	0,6279808
CG6197-RA	fand	5,32107	20,2602	7,15196	9,20062	13,1972	7,96109	-0,076653	0,734521	0,6279808
CG6198-RA	CHORD	6,45875	0,0115043	12,1719	0,0129644	9,01019	9,69726	-0,167433	0,496011	0,6279808
CG6199-RA	Plod	58,8979	0	52,822	0,0748474	20,4419	26,3042	0,54124	0,107894	0,6279808
CG6199-RB	Plod	50,3957	0,335906	42,1919	0,119583	8,45697	19,0581	0,542275	0,107212	0,6279808
CG6201-RA	CG6201	4,3774	8,01045	11,4382	14,2609	6,00188	6,71102	0,200896	0,450746	0,6279808
CG6202-RA	Surf4	55,8176	3,87432	3,71232	8,31811	11,1781	33,6792	0,311094	0,149594	0,6279808
CG6202-RB	Surf4	51,3335	6,17818	6,9605	0,0445057	32,8704	44,2941	0,310922	0,149844	0,6279808
CG6203-RA	Fmr1	31,8368	1,61434	20,4264	3,86188	0,00631795	3,8272	0,360898	0,18988	0,6279808
CG6203-RB	Fmr1	0,0156405	0,0041716	4,29452	21,8667	0,00631214	0,0158699	-0,285025	0,326735	0,6279808
CG6203-RC	Fmr1	0,0150595	0,0218648	0,014458	7,89837	0,00627134	0,0159036	0,360898	0,18988	0,6279808
CG6203-RD	Fmr1	0,0150904	0,023495	0,0144877	3,80653	6,28989	0,0325813	0,362345	0,188354	0,6279808
CG6203-RE	Fmr1	0,029849	0,118322	0,0286569	0,015579	0,00979107	8,37999	0,378889	0,168885	0,6279808
CG6204-RA	CG6204	3,78696	0,11299	4,0574	2,78399	3,62071	0,175387	0,515079	0,025045	0,6279808
CG6205-RA	por	18,1121	19,4903	14,656	13,1698	9,30409	11,8774	0,453	0,055468	0,6279808
CG6206-RA	LManII	6,74992	3,06979	7,90508	4,25519	2,49082	2,87158	-0,320182	0,234682	0,6279808
CG6206-RB	LManII	8,6648	7,44772	14,3395	0,668037	0,722132	0,749027	-0,31871	0,236902	0,6279808
CG6207-RA	GlcAT-P	0,0197438	0,017984	0,0189552	3,93018	0,0228876	1,82583	0,689538	0,006098	0,6279808
CG6207-RB	GlcAT-P	24,4129	27,9804	16,4582	0,0730895	7,30241	9,27572	0,70139	0,005194	0,6279808
CG6207-RC	GlcAT-P	3,82106	5,33947	3,93603	3,73742	22,7234	2,32788	0,707861	0,004889	0,6279808
CG6208-RA	gammaSnap2	6,35942	4,06665	7,74346	1,85459	0,89942	9,68057	0,078694	0,793999	0,6279808
CG6209-RA	CG6209	0,0471073	20,9424	0,241205	1,19682	0,232074	0	0,344653	0,205333	0,6279808
CG6210-RA	wls	9,34014	0,0177506	0,427325	0,0277495	0,0375868	7,28131	0,022106	0,92821	0,6279808
CG6210-RB	wls	0,0260483	0,0184301	0,684759	0,185922	0,168143	0,0281735	0,023528	0,92365	0,6279808
CG6211-RA	gce	10,5019	0,0113545	10,5115	65,286	13,7768	10,8374	-0,345345	0,113198	0,6279808
CG6213-RA	Vha13	537,564	673,654	0,175628	0,00578732	0,00783896	0,00601947	-0,132149	0,621454	0,6279808
CG6214-RA	MRP	7,16137	4,14045	9,09929	0,0487537	8,83235	3,83237	-0,913254	5,8E-05	0,6279808
CG6214-RB	MRP	0,0122688	6,57108	8,11961	0,0355495	0,048152	1,06008	-0,914006	5,55E-05	0,6279808
CG6214-RC	MRP	0,0122688	0,0111691	0,0117788	43,8959	17,2461	1,40723	-0,914632	5,43E-05	0,6279808
CG6214-RD	MRP	0,0122688	0,0111753	0,0117788	5,91862	3,89255	7,64354	-0,91459	5,24E-05	0,6279808
CG6214-RE	MRP	0,0122688	0,0111753	0,0117788	2,0161	1,85318	0,0362902	-0,913093	5,64E-05	0,6279808
CG6214-RF	MRP	0,012262	4,4813	0,0117788	3,53819	0,0194933	17,3421	-0,915437	5,4E-05	0,6279808
CG6214-RG	MRP	0,0122688	3,82727	0,0117723	10,7001	7,22305	7,91766	-0,915645	5,37E-05	0,6279808
CG6214-RH	MRP	0,0122688	12,2049	0,0117788	3,69429	1,82929	1,56065	-0,916774	5,39E-05	0,6279808
CG6214-RI	MRP	0,0122688	49,9548	0,0117788	1,30878	0,863255	0,868476	-0,912633	5,92E-05	0,6279808
CG6214-RJ	MRP	10,2567	14,3417	0,0117788	45,4505	8,88441	20,811	-0,913966	5,35E-05	0,6279808
CG6214-RK	MRP	0,0122688	0	11,555	15,4974	1,14534	10,0016	-0,914808	5,52E-05	0,6279808
CG6214-RL	MRP	0,0122688	0,96645	0,0117788	3,12212	19,3697	1,46496	-0,915016	5,49E-05	0,6279808
CG6214-RM	MRP	4,95048	1,24031	8,86356	1,40873	1,54097	53,5983	-0,916147	5,51E-05	0,6279808
CG6214-RN	MRP	0,012262	5,56226	6,08965	82,2977	5,94194	211,846	-0,912471	5,76E-05	0,6279808
CG6214-RO	MRP	5,14678	0	0,0117723	241,599	0	0,0288625	-0,915409	5,5E-05	0,6279808
CG6214-RP	MRP	0,0122688	0	0,0117788	0,0282734	8,97779	8,49887	-0,918162	5,27E-05	0,6279808
CG6214-RQ	MRP	6,90735	0,165487	0,0117788	0	5,46022	5,96801	-0,917526	5,38E-05	0,6279808
CG6215-RA	CkIIalpha-i1	2,725	2,59756	3,16373	2,37407	2,36689	2,1209	0,487834	0,055752	0,6279808
CG6216-RA	CG6216	0,0628894	0,057284	0,0603775	21,9946	3,17249	0,0746125	-0,132926	0,649066	0,6279808
CG6216-RB	CG6216	2,41348	3,16565	2,74961	5,22139	9,41985	2,29409	0,012661	0,964069	0,6279808
CG6217-RA	knk	18,8083	2,27866	4,32985	3,64706	8,86149	0,00476159	0,290142	0,311193	0,6279808
CG6218-RA	CG6218	47,8625	57,154	47,3518	46,3784	16,4978	18,359	0,229496	0,259701	0,6279808
CG6219-RA	cav	0,0380402	45,5581	0,0465079	0,0976867	0,0768395	22,4116	0,098921	0,727168	0,6279808
CG6219-RB	cav	0,0426296	0,0346497	0,0361257	24,506	0,0574506	12,9762	0,310204	0,291063	0,6279808
CG6219-RD	cav	0,0431869	0,03883	0,0770174	8,49082	0,132317	13,6359	0,198578	0,496978	0,6279808
CG6219-RE	cav	0,0484427	0,0393376	19,5315	10,1861	4,66315	0,0381423	0,187246	0,51989	0,6279808
CG6220-RA	CG6220	0	2,08753	0,0713307	20,698	32,0934	0	0,01562	0,897983	0,6279808
CG6222-RA	su(sable)	9,76182	11,69	0,0344079	16,0489	0,0526702	0,0396954	-0,052138	0,840644	0,6279808
CG6223-RA	betaCOP	34,7327	0,0486682	39,2312	57,6198	10,1446	31,2577	-0,130719	0,55281	0,6279808

gene_id	Symbol	W1_FPKM	W2_FPKM	W3_FPKM	MCM51_FPKM	MCM52_FPKM	MCM53_FPKM	FC	p-value	p-adj
CG6224-RA	dbo	19,7433	9,99044	6,85937	19,6396	6,44461	4,93744	0,250306	0,28962	0,6279808
CG6225-RA	CG6225	8,84328	10,9535	10,3728	7,38436	0,0323898	0,0244109	-0,048562	0,830709	0,6279808
CG6225-RB	CG6225	0,0227482	0,0207206	0,0218396	7,02075	3,20793	5,99782	-0,048487	0,830624	0,6279808
CG6226-RA	Fkbp39	52,1374	36,0255	111,865	80,0791	62,5902	91,1748	-0,225586	0,460234	0,6279808
CG6227-RA	CG6227	3,64869	3,65933	27,274	6,54609	9,96638	0,691364	-0,027281	0,921214	0,6279808
CG6230-RA	CG6230	11,2174	6,04087	4,14635	0,543949	3,05887	3,40983	0,107321	0,675654	0,6279808
CG6231-RA	CG6231	11,9449	0,0305412	0,0214603	0,0701333	0,0324133	0,0244286	-0,054878	0,836178	0,6279808
CG6231-RB	CG6231	24,019	3,86557	5,72658	0,771579	0,0276359	0,0208281	-0,03496	0,895121	0,6279808
CG6231-RC	CG6231	0,0228953	0,0149916	0,0158013	14,3277	0,0230889	0,0174012	-0,03562	0,893148	0,6279808
CG6231-RD	CG6231	0,0223531	0,0308701	0,0325372	7,98986	0,0495694	0,0373585	-0,036468	0,890248	0,6279808
CG6232-RA	loh	24,0256	2,43657	5,73666	6,48679	4,66306	0,065719	-0,119577	0,621776	0,6279808
CG6233-RA	Ufd1-like	15,6156	16,8123	24,4146	17,8167	15,3756	17,8764	0,067325	0,788367	0,6279808
CG6234-RA	Dtg	27,516	1,99395	25,156	0,0239126	9,6225	8,93771	0,182745	0,575228	0,6279808
CG6235-RA	tws	3,80554	14,0242	0,0282359	0,00466011	0	0,0222031	0,244155	0,246835	0,6279808
CG6235-RB	tws	0,0207873	35,4299	43,6989	0,00462998	3,85464	0,023016	0,261876	0,220179	0,13772387
CG6235-RC	tws	0,0215114	5,09489	6,10842	12,0298	0,0294603	0,0221446	0,262384	0,219271	0,6279808
CG6235-RD	tws	0,0207351	0,0189345	0,019957	1,92268	0,0305389	0,0227052	0,262384	0,219271	0,6279808
CG6235-RE	tws	0,0212349	0,0195941	0,0206522	0,00461474	0,0293827	0,0231507	0,261573	0,220849	0,6279808
CG6235-RF	tws	0,0216311	0,018887	0,0199069	0,0253087	0,0301266	2,00699	0,272777	0,199486	0,6279808
CG6235-RG	tws	5,36248	0,0193422	0,0203867	0,0273124	0,0307176	2,87277	0,262508	0,219403	0,6279808
CG6235-RH	tws	6,01883	0,0197031	0,0207672	0	3,91993	0,0320687	0,261683	0,220599	0,6279808
CG6236-RA	CG6236	0,0277668	0,025292	0,0266578	11,2876	7,14904	0,0301565	-0,309664	0,19864	0,6279808
CG6236-RB	CG6236	3,27196	2,62218	4,62428	6,19223	0,0400134	3,86911	-0,30715	0,20242	0,6279808
CG6238-RA	ssh	2,93627	1,63288	5,47636	20,8423	1,60851	5,35807	-0,138926	0,554115	0,6279808
CG6238-RB	ssh	5,09283	14,1889	2,88947	26,3585	4,75719	2,72236	-0,117229	0,61878	0,6279808
CG6240-RA	Cpr92A	0,919368	0	0	29,1213	0	22,2206	0,217335	0,540752	0,6279808
CG6241-RA	TAF1B	2,36069	20,0659	17,6187	25,4084	1,69601	0,00474145	0,148024	0,592637	0,6279808
CG6244-RA	CG6244	0,398048	1,11456	1,27265	29,0682	3,28197	1,69692	0,490005	0,149302	0,6279808
CG6249-RA	Csl4	9,03728	5,23595	8,92777	5,47259	11,5542	13,9759	-0,120201	0,714251	0,6279808
CG6251-RA	Nup62	9,01035	9,20207	15,9684	12,6216	0,187495	9,93724	0,131125	0,599527	0,6279808
CG6253-RA	RpL14	1736,32	51,5036	3097,15	53,5083	33,7588	6,21487	-0,496813	0,072303	0,6279808
CG6254-RA	CG6254	7,98243	7,97806	9,1218	0,00464467	0,0342808	0,747119	0,235291	0,231521	0,6279808
CG6255-RA	Scsalpha2	0,250287	0,227979	0,019539	0,131519	0	0,0217157	0,35853	0,21855	0,6279808
CG6258-RA	RfC38	2,55338	2,19012	0	7,6308	2,85353	13,2139	-0,019034	0,947897	0,6279808
CG6259-RA	Vps60	39,8617	5,12292	5,94616	10,4178	65,9428	59,438	-0,428381	0,098954	0,6279808
CG6262-RA	CG6262	0	0,0178931	0,0188594	0,0502606	5,19382	0	0,176159	0,371457	0,6279808
CG6262-RB	CG6262	0	0,0611651	0,0681626	0,0259521	0	0	0,176159	0,371457	0,6279808
CG6262-RC	CG6262	0	0,114772	0,15132	0,0346981	0	0	0,100158	0,473131	0,6279808
CG6262-RD	CG6262	0	0,0179948	0,0189665	0,371635	0	0	0,096567	0,482921	0,6279808
CG6262-RE	CG6262	0	0,0978288	0,0687413	6,56719	0	0	0,044335	0,715924	0,6279808
CG6264-RA	Best1	4,90377	2,18184	0,0044251	6,84939	2,53023	0,00475721	-0,487793	0,033983	0,6279808
CG6264-RB	Best1	2,66915	5,53893	0,0044437	5,39987	0,021057	0,00472646	-0,494078	0,031942	0,6279808
CG6265-RA	Nep5	0,245696	0,0179038	0,247593	0,353958	2,06051	15,7415	-0,054699	0,872206	0,6279808
CG6265-RB	Nep5	0,0206315	0,488608	0,191148	72,1605	3,2504	5,50578	-0,066215	0,846261	0,6279808
CG6265-RC	Nep5	0,0189619	0,0172718	52,9685	0,247399	3133,18	0,484367	-0,055039	0,871384	0,6279808
CG6269-RA	unc-4	1,79752	2,57685	2,1699	2,68971	0,934565	1,14908	0,33161	0,253424	0,6279808
CG6271-RA	CG6271	1,08068	2,16938	37,4934	13,2285	10,2644	35,6073	-0,809647	0,021378	0,6279808
CG6272-RA	Irbp18	41,8521	4,36553	4,5167	1,05233	68,5649	4,18902	-0,167568	0,519468	0,6279808
CG6276-RA	CG6276	3,76067	4,1384	4,3069	0,0295409	1,88944	3,79949	0,101814	0,683717	0,6279808
CG6277-RA	CG6277	11,5724	0,856649	6,13618	3,26067	0,0623468	8,59403	-0,338342	0,284231	0,6279808
CG6279-RA	lyd	1,27992	1,32371	2,0864	1,94387	2,18261	162,438	-0,508017	0,076609	0,6279808
CG6280-RA	CG6280	0,744914	0,0329608	0,0324706	0,930597	0,0494595	0,0372757	0,378551	0,222769	0,6279808
CG6281-RA	Timp	18,0998	0,0435607	0,983741	2,52831	1,6396	2,66763	-0,134937	0,632968	0,6279808
CG6281-RB	Timp	0,0478233	0,011983	1,1751	3,73677	16,6925	0	-0,111666	0,692859	0,6279808
CG6282-RA	CG6282	2,04348	0,0174673	0,0184106	0,0199882	2,96493	1,70232	-0,215418	0,390371	0,6279808
CG6282-RB	CG6282	0,0185728	0,0287012	0,0302512	0,0347801	0,0261846	0,0197343	-0,225691	0,366706	0,6279808
CG6282-RC	CG6282	0,498589	13,789	14,6838	13,4276	0,0270742	0,365917	-0,210093	0,400208	0,6279808
CG6283-RA	CG6283	2,79108	0,30937	16,6019	10,2965	49,2803	18,2528	-0,363434	0,264098	0,6279808
CG6284-RA	Sirt6	5,61022	3,18597	4,32221	7,10751	4,63256	6,14881	-0,10913	0,698502	0,6279808
CG6287-RA	Dsim\GD22186	4,49461	0	0,0180082	0	0	0	-0,841505	0,015483	0,6279808
CG6289-RA	Dsim\GD14875	1,39053	0,011001	8,9269	7,69559	1,04331	151,096	1,549865	6,12E-06	0,6279808
CG6290-RA	CG6290	0,270433	3,6555	0,239686	0,478875	0	0,0274491	0,051224	0,82576	0,6279808
CG6291-RA	ApepP	7,62292	0,94461	0,0148471	9,55527	4,58546	1,16481	-0,159171	0,613077	0,6279808
CG6292-RA	CycT	4,24082	4,96918	5,2573	7,64416	3,19522	3,18633	-0,074753	0,784826	0,6279808
CG6292-RB	CycT	3,36783	4,16577	3,10638	7,19241	2,71403	2,34806	-0,094547	0,731609	0,6279808
CG6293-RA	CG6293	6,8033	6,6921	9,20837	7,81937	8,40366	12,2505	-0,13911	0,575535	0,6279808
CG6294-RA	CG6294	0,0390058	5,00962	0,0374479	8,33701	0,0782543	0,0589772	-0,399873	0,098895	0,6279808
CG6295-RA	CG6295	0,514798	0,165499	6,33786	1,38727	660,512	60,2483	0,372923	0,260393	0,6279808
CG6296-RA	CG6296	1,63093	0,7923	5,51754	2,33584	35,5282	3,33364	-0,250746	0,480002	0,6279808
CG6297-RA	JIL-1	0,0116707	0,823844	0,199487	0,906546	9,80411	12,1771	0,253446	0,345925	0,6279808
CG6297-RB	JIL-1	18,1587	0,0106305	0,0112046	1,02708	1,06487	0,642446	0,294732	0,26918	0,6279808
CG6298-RA	Jon74E	1,86655	0,607208	26,2912	7,95208	0	0,160022	0,01631	NA	0,6279808
CG6299-RA	CG6299	0,0411773	4,7259	0,0395326	0,0452702	0,232714	0,175387	-0,413873	0,164229	0,6279808
CG6299-RB	CG6299	58,7735	45,6723	89,2119	56,97	24,3332	26,4063	-0,415762	0,153674	0,6279808
CG6300-RA	CG6300	14,8148	6,63221	2,17342	10,4306	3,7904	4,18135	0,791926	0,000199	0,6279808
CG6302-RA	Pfdn2	92,5545	4,16701	132,591	118,971	14,7398	5,55465	-0,433844	0,125387	0,6279808
CG6303-RA	Bruce	3,72523	4,09096	0,0459132	0,0125615	0,465381	0,0545749	-0,222209	0,410766	0,6279808
CG6304-RA	CG6304	0	0	0	0	0	0	NA	NA	0,6279808
CG6305-RA	Cpr50Cb	60,3743	0,0308069	96,6549	39,8841	59,0585	4,11176	0,425916	0,147334	0,6279808
CG6308-RA	Alg14	6,4814	0,0328171	0,896193	0,12245	122,356	132,635	-0,182681	0,529632	0,6279808
CG6310-RA	CG6310	50,603	3,98316	19,7016	0,0300119	2,30436	0,0306372	0,357584	0,151271	0,6279808
CG6311-RB	Edc3	14,7897	19,4769	12,3017	169,196	9,30369	8,08313	0,407417	0,100926	0,6279808
CG6312-RA	Rfx	1,99604	2,909	2,57683	0	1,9773	2,03241	-0,092293	0,752525	0,6279808
CG6312-RB	Rfx	1,10979	0,888753	0,514855	4,58684	0,0208212	0,292752	-0,087671	0,765782	0,6279808
CG6315-RA	fl(2)d	19,1926	0,696914	14,4808	22,0455	12,5677	12,6801	0,186677	0,410469	0,6279808
CG6315-RB	fl(2)d	17,2503	0	20,7616	24,8278	12,5467	14,9056	0,207706	0,367503	0,6279808
CG6318-RA	Xrcc2	0,131625	0,199822	35,2356	12,4764	71,9219	0,0252635	-0,078128	0,812195	0,6279808
CG6321-RA	CG6321	14,909	2,98361	4,98556	4,98426	0,0406513	2,8254	0,271011	0,374106	0,6279808
CG6322-RA	U4-U6-60K	7,93425	9,7141	16,8924	23,3047	227,626	5,55776	0,419432	0,072204	0,6279808
CG6323-RA	Tsp97E	34,0213	32,3108	33,4847	18,6399	0	569,492	0,021598	0,917771	0,6279808
CG6323-RC	Tsp97E	9,59384	9,85829	9,24344	0,0590152	0,475732	79,3951	-0,227989	0,325007	0,6279808
CG6323-RD	Tsp97E	0,0522063	0,0475531	0,0501212	8,86223	3,56857	28,5604	-0,282111	0,235034	0,6279808
CG6324-RA	CG6324	0,0600472	1,58541	0,907861	1,48048	0,902487	0,984694	0,000945	0,997537	0,6279808
CG6325-RA	CG6325	3,58298	3,08139	3,51845	3,80369	1,57649	2,08419	0,330672	0,192594	0,6279808
CG6327-RA	CG43693	0,0313057	3,06058	0,0300553	13,0374	6,76981	15,1394	-0,395903	0,117034	0,6279808
CG6327-RB	CG43693	1,20903	0,0292641	3,49497	0	2,65596	0	-0,398535	0,115228	0,6279808
CG6327-RC	CG43693	0,0281813	4,072	0,0270558	3,99548	44,5722	16,2914	-0,395751	0,118909	0,6279808
CG6327-RD	CG43693	9,7395	11,3854	14,6129	16,8417	16,398	24,4763	-0,400139	0,115333	0,6279808
CG6329-RA	CG6329	34,4482	0,0575029	0,0624582	0,0734152	0,0792972	0,0597632	0,178809	0,390489	0,6279808
CG6329-RB	CG6329	0,0631297	0,059258	0,0497673	0,0760417	82,9163	83,7889	-0,151906	0,480878	0,6279808
CG6329-RC	CG6329	0,0650565	0,0472174	2,52763	0,0585433	29,8632	2,58813	-0,155753	0,471781	0,6279808
CG6329-RD	CG6329	0,0518377	66,2694	0,0360846	58,9539	3,39529	0	-0,148662	0,491188	0,6279808
CG6330-RA	CG6330	44,3421	2,85037	2,28464	32,8263	0,0386447	0,0289495	-0,611813	0,030907	0,6279808
CG6330-RB	CG6330	32,5659	0,0244793	0,0258013	6,77834	0,0387117	0,0276523	-0,582913	0,038659	0,6279808

gene_id	Symbol	W1_FPKM	W2_FPKM	W3_FPKM	MCM51_FPKM	MCM52_FPKM	MCM53_FPKM	FC	p-value	p-adj
CG6331-RA	Orct	12,7119	0,00470403	10,8391	0,300741	0	7,028	0,137583	0,615791	0,6279808
CG6332-RA	CG6332	0	0,710292	0,306115	0,366636	0,202066	0,292158	-0,110933	0,487557	0,6279808
CG6332-RB	CG6332	0	0	0	7,13551	0	7,69104	-0,110933	0,487557	0,6279808
CG6333-RA	CG6333	0,238026	0,346896	227,262	152,681	4,45883	269,678	0,133767	0,612091	0,6279808
CG6335-RA	HisRS	0,0317876	0,0289543	0,0724928	26,181	0,0650967	0,0668229	-0,42181	0,098966	0,13772387
CG6335-RB	HisRS	19,5626	19,1269	0,030518	0,0312502	2,902	0,0348608	-0,422774	0,098155	0,6279808
CG6337-RA	CG6337	35,7047	28,9057	73,3932	47,7651	27,088	22,0622	-0,525654	0,101608	0,6279808
CG6338-RA	Ets97D	6,2758	4,64981	6,21837	5,91525	9,0094	5,9195	0,192636	0,379517	0,6279808
CG6339-RB	rad50	1,28326	1,59326	0,0594162	1,41366	0,0223153	0,0165941	0,423395	0,095353	0,6279808
CG6340-RA	CG6340	0,124047	0,11299	0,119092	0,171807	0,940895	0,0462135	-0,716101	0,001596	0,6279808
CG6340-RB	CG6340	0,0512348	0,0466682	0,0491884	0,0577733	2,5688	0,0450981	-0,23302	0,207124	0,6279808
CG6340-RC	CG6340	22,7449	26,0343	26,0122	37,5907	0,0139574	104,638	-0,690463	0,001956	0,6279808
CG6341-RA	eEF1beta	361,513	0,02301	3,25151	3,98877	4,806	9,71211	-0,061147	0,82314	0,6279808
CG6342-RA	Irp-1B	30,1495	30,5623	7,47507	3,90598	2,69067	5,67104	0,480473	0,012102	0,6279808
CG6343-RA	Dpse\GA19526	76,3044	75,8629	142,1	105,671	145,537	139,202	-0,419119	0,143296	0,6279808
CG6343-RB	Dpse\GA19526	0,0444157	0,0404569	0,0426417	0,0492272	0,0666786	0,050253	-0,421497	0,140892	0,6279808
CG6345-RA	CG6345	5,06652	4,76763	2,42865	24,1263	6,72612	2,5544	-0,239562	0,330624	0,6279808
CG6347-RA	CG6347	60,7838	38,6198	0,0606083	38,043	0,102999	0,0776262	0,532334	0,014131	0,6279808
CG6348-RA	ro	0,055635	0,0506762	0,0534129	0,0634476	23,7987	0,524339	-0,302698	0,397609	0,6279808
CG6348-RB	ro	0,349094	0,370976	0,474798	0,513636	0,0859402	0,840616	-0,278571	0,43624	0,6279808
CG6349-RA	DNApol-alpha180	2,15356	11,2217	2,38932	4,54922	2,09697	2,00783	0,081781	0,753306	0,6279808
CG6352-RA	OdsH	0,431164	0,613647	0,569172	4,4986	2,40489	59,273	0,383203	0,284097	0,6279808
CG6353-RA	Archease	13,8661	11,285	16,7257	17,4129	11,5789	19,6996	-0,352496	0,150559	0,6279808
CG6354-RA	Rb97D	7,58084	3,99663	4,28999	3,60834	0,0384118	6,47967	0,283793	0,204368	0,6279808
CG6354-RB	Rb97D	0,0237968	0,0243407	0,0256552	0,0285305	0,0366906	0,027494	0,283793	0,204368	0,6279808
CG6354-RC	Rb97D	0,0268747	0,023313	0,024572	4,71053	2,52103	0,0352999	0,281707	0,209029	0,6279808
CG6354-RD	Rb97D	6,31238	6,50275	3,91301	0,0283585	2,79625	0,203271	0,283793	0,204368	0,6279808
CG6354-RE	Rb97D	0,0267225	3,39694	0,0244395	0,0270878	0,046838	2425,94	0,281707	0,209029	0,6279808
CG6354-RF	Rb97D	0,0255943	0,0292926	0,0308745	5,4414	27,3246	3,60968	0,281707	0,209029	0,6279808
CG6354-RG	Rb97D	5,06271	5,81178	3,66999	0,0269328	8,77111	24,7073	0,281031	0,204965	0,6279808
CG6354-RH	Rb97D	0,0254562	32,1458	37,682	0,0345794	0,0799364	7,63267	0,066364	0,776102	0,6279808
CG6354-RI	Rb97D	0,0321589	31,7496	38,3419	45,3217	28,1729	0,0602449	0,281031	0,204965	0,6279808
CG6355-RA	fab1	2,64854	3,37577	5,37497	0,00951433	0,0128872	0,0117114	0,07152	0,75948	0,6279808
CG6355-RB	fab1	2,13555	2,15796	1,02513	0,0114723	0,0155394	0,0283635	-0,010325	0,964814	0,6279808
CG6356-RA	CG6356	14,3506	8,21128	16,8533	17,2425	4,08911	0,0154649	-0,077914	0,791947	0,6279808
CG6357-RA	CG6357	2,72598	1,76145	34,2504	2,32715	0,0994414	0,074945	-0,273064	0,360765	0,13772387
CG6358-RA	Xpac	2,56863	8,35038	4,93208	0,0292265	21,7524	5,48248	-0,376867	0,187895	0,6279808
CG6359-RA	Snx3	54,2816	0,120373	0,596414	0,342787	0,436887	0,106261	0,061069	0,786245	0,6279808
CG6361-RA	Hayan	9,57676	11,1998	9,69359	36,4062	9,43475	7,94957	0,034349	0,871981	0,6279808
CG6361-RB	Hayan	7,07528	0,0451624	23,7401	0,0556743	13,8978	10,0735	-0,055181	0,797758	0,6279808
[truncated: 512,452 more chars]
