# Supplementary material for: Selective role of the DNA helicase Mcm5 in BMP retrograde signaling during Drosophila neuronal differentiation
Source: PLoS Genet. 2022 Jun 23;18(6):e1010255. doi: 10.1371/journal.pgen.1010255 (PMC9258838; doi:10.1371/journal.pgen.1010255)
Supplement: Attachment — Submitted filename: PLOS Genetics review 120521.pdf [file pgen.1010255.s006.pdf]

Summary. This manuscript provides evidence that mutations in *Mcm5, a gene* encoding a subunit of the Mcm2-7 DNA replicative helicase, block the development and differentiation of a particular neuron sub-type (Tv4/FMRFa) in *Drosophila*. The authors further show that the lack of this neuron correlates with the reduced transcription of type I BMP receptor Tkv, a particular gene that is known to be necessary for the development of the Tv4/FMRFa neuron. Moreover, over-expression of this receptor in the *mcm5* mutant rescues the Tv4/FMRFa differentiation defect. Together, these observations provide strong evidence that the differentiation defect of the mcm5 mutant is due to disruption of this particular signaling pathway. These results are consistent with other observations in the liturature which indicate that mutations in DNA replication genes result in central nervous system defects, suggesting perhaps that in addition to its canonical role in unwinding DNA during replication, the Mcm2-7 complex may also have a role in transcriptional regulation.

General concern. As both cited in references by the authors, as well as in other references not cited (e.g., Bik Tye has made similar observations in budding yeast decades ago), it is known that *mcm* mutations can affect gene expression and can cause defects that are at odds with the complex's known role in DNA replication. The observation in this manuscript that *mcm* mutations can cause specific defects in neural development is in itself a noteworthy and interesting result. However, this current manuscript is largely descriptive in nature, cataloging the defect without attempting to provide a mechanistic explanation – e.g., is the observed defect primarily one of decreased transcription, or is the decreased transcription a secondary defect caused in some manner by aberrant DNA replication? As such, this manuscript does not really push the boundaries of knowledge.

Secondary concerns. This manuscript is basically well-written, but there are various short comings

> *Discussion of the mcm5 mutation and its general phenotype.* Although it seems that the mcm5 mutation used is not new to this study and its origin is appropriately cited by the authors, I find it very odd that there is not a clear paragraph early in this paper (introduction) that more generally describes this allele. What is the nature of this mutation (Null, missense, dominant/recessive?). What is the broader phenotype in the entire organism? (Lethality, developmental arrest at some informative point)? It is true that there is some discussion of *mcm5* mutations in the Discussion section, but due to the way the section is written, it is unclear to this reviewer if these comments are of a general nature or specifically refer to the allele used in this study.
>
> *Add an interpretive panel to Fig. 1 to visually reinforce the first paragraph of the Results section.* Most readers of *PLOS Genetics* will likely find that your system is rather complicated. I would suggest that you consider inserting a new Figure 1A panel that provides guidance to your system and shows the relationship among the various developmental stages and micrographs that you discuss inn the text and show in the various figures. Once the reader is properly orientated, many of the presentation issues become much more manageable.

Correlation problems between the figure panels and their citation in the text. It would appear that during the development of this manuscript there were numerous changes to the Figures and Tables that were not propagated through the text of the manuscript.

Is this mcm5 defect related to the entire Mcm2-7 complex or is it unique to mcm5? You try to address this question in Fig. 1, where you show that an *mcm7* mutant also causes a problem with development of the Tv4/FMRFa neuron. However, this issue is never revisited as more information on the *mcm5* defect is obtained. For example, is the *mcm7* defect also suppressed by over expression of Tkv?

Actual expression levels of Tkv in the control and mcm5 mutant. A critical claim in this manuscript is that the *mcm5* allele specifically reduces transcription of the BMP receptor Tkv. However, the actual numbers are not show in the main manuscript but are instead buried in Sup Table 2. Given the importance of this obseration, it needs to be front and center in the paper. I would suggest that addition of either a graph or a table that shows the relative reduction in the TkV transcripts in the *mcm5* mutant, as well as the transcription levels of other genes in this developmental pathway (possibly the same genes that were assayed by immunofluorescence in Fig. 2?). For example, what is the expression level of the *dimm* gene (Fig. 2GH)?

Discussion claim. "Our findings **agree with** studies showing that Mcm5 interacts with the RNA pol II complex or specific transcription factors and can play a role during gene expression (18, 19)". As a gentle reminder, this study doesn't examine interactions between mcm5 and the transcription machinery, and thus there is no data to agree or disagree with previous studies – thus you could just as well state that your data agrees with the notion that the moon is made of blue cheese (or whatever cheese you fancy). I would suggest that you slightly modify this sentence by swapping out "agree with" with "consistent with".

Possibility of obtaining somewhat more mechanistic data. This issue may extend well beyond the interests of your lab, but there may be several additional and possibly useful experiments that could be attempted. 1. Direct interactions between the Mcm2-7 and RNA polymerase – one might be able to address this with a proximity ligation assay (PLA). 2. Mcm defects commonly cause DNA damage through defective DNA replication. There are many available cytological assays to examine DNA damage. Do the Tv4/FMRFa neurons in the *mcm5* mutant have DNA damage? Can you determine if these cells are abnormally stuck in S-phase?

Details:

Figure 1. The possible involvement of *mcm5* in neural differentiation came from a genetic screen conducted by the authors to identify mutations that failed to make a specific type of neuron. This figure is meant to show the relevant results of this screen.

Fig. 1 panels A-D shows the result of the *mcm5* mutant along with several important controls. In A-D, I believe that the take home point is whether or not the indicated cells fluoresce red (wildtype expression of relevant gene) or not (mutant phenotype). In the copy of the figures in my possession, if I squint and magnify the image, I can make out red staining of the SE2 cells in the control and pro-> Mcm5 subpanels. No other red is observable. In contrast, below the micrographs, are "interpretive" panels which are meant to instruct the reader in what they should be observing in the above micrographs. As nearly as I can tell, the micrographs and the interpretive panels are largely unrelated. The apparent lack of experimental robustness undermines the rest of the manuscript. Panels EF is meant to quantify the results show in panel A-D. However, the Y axis is rather cryptically labelled. In F, it is labelled as "SE2 Cells". This makes some sense, as the values in all of the bar graphs have a height of "2" (same as the number of SE2 cells observed in A-D). However in E, the expression of the experiment cells (Tv4 cells) are shown. From panels A-D, the reader has been made to believe that there are 6 experimental cells in the micrographs, however the bar graph in E gives a value in excess of 6, with error bars extending to at least 8. Where do these extra cells come from? I don't believe that this issue is addressed in either the text or legend.

The last panels of Figure 1 (G-J) are further controls; however they are out of order in relation to the main text which presents part of Figure 2 before mentioning Fig. 1 G-J. I'm not sure about the rules at PLOS Genetics, but usually Figures need to be presented in the same order as the text. Panels G-H make some sense, as they are in the same format as panels A-D. Panels I-J are immediately confusing; I believe that they are supposed to represent Tv4 cells in Stage 18 AEL, but they look different that the rest of the cells in this figure. Moreover, for the control cells there appear to be 2 cells show/panel, while in the *mcm5* case only 1 cell is shown. Please explain this in the text. Moreover, both I and J are split into 4 unlabeled subpanels. By staring at these for a while, I believe that the top left penal is the combined image, while the other 3 panels correspond to each individual antibody, however, no information is provided. This comment also applies to Fig. 2

Figure 2. These panels are immunofluorescence experiments to test the presence/absence of various diagnostic proteins in the test neurons in the control and *mcm5* strains. Given that they are all clumped, how do you identify the individual cells (e.g., which is TV1 and which is TV4)? In addition, in the text, it is claimed the presence of pMAD is different in the *mcm5* mutant relative to the control, an important observation that is the connection to the next figure. However to my eyes, I don't see pMAD (green) expression in either wild type or control at least as general cellular staining. Again, if I squint, the control strain seems to have a cell with a few green flecks that I don't see in the mcm5 mutant. Is this what I'm supposed to see? If so, please briefly explain this in the text.

Figure 3. This has many of the same issues as Figure 1, except the data is more robust and one can actually see the immunofluorescence in most cases. However, the ordering of the experimental panels and the interpretive panel do not correspond (e.g., panel D is paired with panel C')

Figure 4. Panel B has 2 parts, the data (coverage white and coverage mcm5) and various genetic maps meant to help interpret the data. What does coverage white mean? I would perhaps suppose that this is really meant to be coverage control, and it so happens that the control has a mutation in the white gene – you might want to reword this and perhaps explain in the figure legend. A problem with this panel is that the actual data is small and difficult to seen, while the genetic maps are large and probably more extensive than is necessary. Please flip the emphasis between the 2 sections of this Figure. Exons 1 and 2 are specifically mentioned in the text; please label them in this figure. Most of the explanation part of this panel (e.g., other genes and exons that are irrelevant to Tkv) are not mentioned in the text. Either they should be trimmed from the figure or discussed in the text. Moreover, as mentioned above, information should be added that actually show the relative expression level of Tkv in the *mcm5* mutant in relation to the other genes in the developmental pathway.
