# Supplementary material for: Selective role of the DNA helicase Mcm5 in BMP retrograde signaling during Drosophila neuronal differentiation
Source: PLoS Genet. 2022 Jun 23;18(6):e1010255. doi: 10.1371/journal.pgen.1010255 (PMC9258838; doi:10.1371/journal.pgen.1010255)
Supplement: Attachment — Submitted filename: review PLoS Genetics 032222.pdf [file pgen.1010255.s008.pdf]

Second review of Rubio-Ferrera et. al March 2022 PLoS Genetics

Larger issues.

Although the authors make a good attempt to repair the various problems raised following the first submission, the fundamental problems remain – this manuscript is built from descriptive and relatively preliminary data. In fairness, this manuscript presents two reasonably strong conclusions: 1) *mcm5* mutants blocks BMP signaling in the Tv4 cells, and 2) ectopic expression of the BMP pathway receptor genes Tkv/Sax largely suppress the *mcm5* Tv4 defect (BTW – does ectopic Tkv/Sax expression suppress the *mcm5* lethality observed in the third larval stage?). Although one might have hoped that the genomic experiment in Fig. 4 would provide a strong correlation between the *mcm5* Tv4 phenotype and reduced gene expression of Tkv or Sax, this is not the case, as 1) the RNA-seq experiment utilizes whole embryos, where as the phenotype being examined is restricted to only a few cells (Tv4). Given that most of the RNA comes from cells that lack the *mcm5* CNS phenotype, data interpretation will likely be clouded; 2) The reduction in Tkv transcript levels looks to be about 50% (Fig. 4B, ~ -0.5-fold reduction). Although the trend is consistent with the possibility that reduced Tkv expression causes the *mcm5* Tv4 phenotype, Fig. 4B shows that the expression of most of the genes involved in Tv4 cell maturation and BMP signaling (which based on cytology don't appear to be seriously altered in the *mcm5* mutant, Fig. 2), vary from the control levels to the same degree as Tkv. These results raise the possibility the relatively small reduction in Tkv transcripts just reflects the background scatter of the assay. Given these issues, the substance of this manuscript is rather thin.

Smaller issues.

Line 139 – "lost or absent" – same thing? Perhaps you mean "absent or reduced?"

Line 145 – " Anti-**c**H2aX ". Don't you mean Anti-<u>gamma</u>H2aX? At least in other organisms, this modification is referred to as gH2AX.

Line 153 – extra word "This revealed that it Mcm5 is expressed"

Fig. 1A. I like the new interpretive panel! However, the legend for FMRFA and SVP are nearly the same color (Red Pink). Differentiate better – maybe make one blue?

The initial screen (Table 1) claims that the *mcm5* mutant causes loss of Tv4 cells, while in Fig 1I and J (and Figure 2) the Tv4 cells are still present in the *mcm5* mutant, but they just do not produce FMRFa. This seems to be a discrepancy. Homozygous mcm5 mutant vs. hemizygous mcm5 mutant? Please clear up this issue.

Although not mentioned in the Results, the *mcm5* mutants appear to increase the number of cells expressing Cas (Fig 2b). Is this significant?

Except for data used in graphs Fig 1GH and 3F, it is not mentioned how many cells/embryos were cytologically examined for each treatment. How representative are the panels shown? Include graph/documentation in supplemental similar to Fig 3F for all treatments?

Fig 4A. X-axis – Log_{10} or Log_2?

Figure 4B. Dimmdac? I believe that s a space is needed. Also, as Materials and Methods indicate that this experiment was done in triplicate, include error bars in this graph?

Fig. 4C. I'm still having difficulty understanding the actual Tkv expression difference between the control and the *mcm5* mutant. As nearly as I can tell from the diagram, the control cells make a significant level of transcripts relatively to the *mcm5* mutant for a sequence on the left end of the graph (Under "Scale [0-325]"). However, the text states that expression of exons 1 and 2 (arrows right end of the graph) have a "striking reduction" in the *mcm5* mutation. (So Tkv is transcribed from right to left?) At least as drawn, it doesn't appear that either the control nor mcm5 mutant produce significant amount of either exon 1 or 2. Please clarify.

Line 470-471 : "…other genes located in the same chromosome region in green (its RNAm in yellow)" I have no idea what this means.
