# Supplementary material for: Selective role of the DNA helicase Mcm5 in BMP retrograde signaling during Drosophila neuronal differentiation
Source: PLoS Genet. 2022 Jun 23;18(6):e1010255. doi: 10.1371/journal.pgen.1010255 (PMC9258838; doi:10.1371/journal.pgen.1010255)
Supplement: Attachment — Submitted filename: Comment s to the Authors.docx [file pgen.1010255.s007.docx]

Madrid, March 10^,^ 2022

**PGENETICS-D-21-01485**

Dear Editor,

We are pleased to hereby re-submit our manuscript PGENETICS-D-21-01485 entitled "*Selective role of the DNA helicase Mcm5 in BMP retrograde signaling during Drosophila neuronal differentiation*". We are very grateful for the insightful and valuable comments from the reviewers and for their apparent enthusiasm over the topic and our results:

Reviewer 1: “*This study is beautifully done. It lends further support for the much less appreciated role of MCM complex in gene expression.*”

Reviewer 2: “*Overall, this is an interesting study.*”

Reviewer 3: “*The observation in this manuscript that mcm mutations can cause specific defects in neural development is in itself a noteworthy and interesting result*”.

The reviewers further made several excellent suggestions for new experiments, and for how the manuscript could be improved. We have made every effort to conduct these new experiments, and to incorporate these changes. We have addressed each reviewer’s comments below. To clarify, the major changes to the manuscript are:

- Analysis of Mcm5 expression using a newly generated antibody.
- Rescue of the Mcm5 mutant phenotype using a *UAS-Mcm5* transgene*.*
- Analysis of mutants for all MCM2-7 complex components.
- DNA damage analysis in *Mcm5* mutants.
- A new cartoon added to Fig 1 to better explain the model used in this study.

In summary, we hope that we have satisfactorily addressed all the reviewer’s comments and look forward to hearing from you. Again, thank you for handling our manuscript.

Sincerely yours,

Ignacio Monedero Cobeta, Ph.D.

Professor Assistant

Department of Physiology

Universidad Autónoma de Madrid

Madrid, SPAIN

Ph: +34-91-497 XXX

Email: [ignacio.monedero@uam.es](mailto:ignacio.monedero@uam.es)

**Reviewer #1**

1) It is unclear exactly where and when Mcm5 acts to ensure the correct Tv4/FMRFa neuron fate. It was mentioned about Mcm5 expression in subsets of CNS cells, which is interesting and should be mapped with respect to the neuronal lineage of interest. Further, one should attempt rescue with Mcm5 transgene (in addition to the Mcm5 misexpression) and (if possible) more targeted Mcm5 knockdown.

**Reply:** Regarding Mcm5 expression, we have analyzed this using a specific antibody against Mcm5 (not yet developed at the time of the initial submission). We have mapped Mcm5 expression in the NB5-6 neuronal lineage, and find expression in both the neuroblast and postmitotic neurons. We have added this information to the manuscript (Fig S2E-F). Regarding transgenic rescue of Mcm5, we have expressed an Mcm5 transgene using the Gal4>UAS system (*Mcm5, pros>UAS-Mcm5*). We observe robust rescue of the phenotype, and have added these results to the manuscript (Fig S1A-B).

2) It was only mentioned near the end of Discussion about the undergrown CNS observed in Drosophila Mcm5 mutants. This info is highly relevant and should be made aware of upon mentioning of Mcm5 mutants. One should also try to reconcile the limited phenotype observed in this study with the undergrown CNS phenotype reported before.

**Reply:** There is a difference in the embryonic phenotype (studied herein) versus larval CNS phenotype (ref 38). In the embryo, we do not observe any apparent differences in size between the wild type and *Mcm5* mutant CNS. We apologize that we did not make this point clear, and have rephrased this section in the Discussion: “*Mcm5* is dynamically expressed in *Drosophila* as well, and *Drosophila* *Mcm5* mutants die at L3 with absent imaginal discs and an undergrown CNS (38). The latter phenotype contrasts with our findings in the embryo, where we did not observe any reduction in growth of the CNS, pointing to different roles for *Mcm5* in the embryonic versus larval CNS.”

**Reviewer #2**

1) Is the novel role the Mcm5 helicase plays in neuronal differentiation specific to Mcm5 (and Mcm7)? To claim that the whole complex Mcm2-7 is involved in neuronal subtype specification, I wonder if knockdown of the other 4 Mcm genes gives rise to the same phenotype in the specification of Tv4 neuron?
**Reply:** This is an interesting issue, and to address it we have now analyzed FMRFa expression in the other four mutants of the MCM2-7 complex. We find loss of FMRFa in the *Mcm3* and *Mcm4* mutants, but no effect in the *Mcm2* and *Mcm6* mutants (new Fig. S1C-J), possibly due to their maternal expression. These results strongly suggest that, rather than a specific function for only Mcm5 and Mcm7 alone, the entire MCM complex is involved in FMRFa neural fate acquisition.

2) Mcm7 phenocopies Mcm5. Is Mcm5 and Mcm7 functionally interchangeable in regulating neuronal differentiation?
**Reply:** Our understanding of the literature is that Mcm5 and Mcm7 are not interchangeable. In line with this notion, we do not find down-regulation of Mcm7 mRNA in the Mcm5 mutant transcriptome analysis, indicating unchanged levels of Mcm7 protein in the Mcm5 mutants. And since there is a mutant phenotype in Mcm5 this would argue that Mcm7 indeed cannot substitute for Mcm5.

3) Is Mcm7 also required for the expression of Tkv or other BMP signaling components? Could pros>saxA or tkvA rescues Mcm7 defect?
**Reply:** We have tried to do the rescue proposed by the reviewer (*Mcm7, pros> UAS-tkvA, UAS-saxA*), but unfortunately, and for reasons that are unclear to us, the VNC of this particular rescue genotype was disorganized. Therefore, we cannot obtain conclusive evidence.

4) As controls, can transgenic pros>Mcm5 expression rescue the Mcm5 defect in Fig1 and Fig3?
**Reply:** We have now performed this rescue: in a Mcm5 mutant background, we have reintroduced the transgene mcm5 through the Gal4>UAS system (*Mcm5*, *pros>UAS-Mcm5*). We have found a robust rescue of the phenotype and have added these results to the manuscript (Fig. S1A-B).

5) Also a bit of a stretch, to relate the study with mammalian CNS development, could human Mcm5 or 7 transgenes rescue the defects in fly brain?
**Reply:** The Mcm5 and Mcm7 genes are highly conserved, and the proteins show 83% and 82% similarity between Drosophila and humans, respectively. Therefore, we would certainly anticipate strong, even complete, rescue. Unfortunately, we cannot test species cross-rescue in a timely manner because the transgenic lines expressing the human genes have not yet been generated.

6) Also, it would be nice of the list and the results of the 35 candidates are revealed (maybe as a supplement table).
**Reply:** This information was available in the original submission as a supplementary table (Table S1), but has now been moved to the main text (Table 1).

**Reviewer #3**

**General concern:**

1) As both cited in references by the authors, as well as in other references not cited (e.g., Bik Tye has made similar observations in budding yeast decades ago), it is known that *mcm* mutations can affect gene expression and can cause defects that are at odds with the complex’s known role in DNA replication. The observation in this manuscript that *mcm* mutations can cause specific defects in neural development is in itself a noteworthy and interesting result. However, this current manuscript is largely descriptive in nature, cataloging the defect without attempting to provide a mechanistic explanation – e.g., is the observed defect primarily one of decreased transcription, or is the decreased transcription a secondary defect caused in some manner by aberrant DNA replication? As such, this manuscript does not really push the boundaries of knowledge.

**Reply:** Our work points to that the MCM2-7 complex phenotype, loss of FMRFa expression, results from a failure in the BMP pathway, evident from 1) loss of pMad in FMRFa cells, 2) RNA seq analysis, which revealed decreased transcription in the tkv receptor, and 3) genetic cross-rescue of Mcm5 by the BMP receptors tkv and sax. Regarding DNA damage, and following the advice of reviewer 3, we have now analyzed the DNA damage by anti-cH2aX antibody staining in *Mcm5* mutants. Indeed, we find that in *Mcm5* mutants there is evidence for increased DNA damage in the CNS (new Fig. S2A-D). However, we do not believe that the DNA damage explains the observed phenotype because: 1) the appearance of DNA damage is stochastic, while our phenotype is highly consistent, 2) we see a lack of FMRFa without observing the DNA damage in Ap cluster cells, and 3) BMP receptor expression can robustly cross-rescue the Mcm5 mutants. In summary, we believe that the loss of FMRFa results from reduced gene expression of the BMP receptor tkv, but also acknowledge that there are signs of DNA damage in the developing CNS. We thank reviewer 3 for the suggestion to probe for DNA damage.

**Secondary concerns:**

1) This manuscript is basically well-written, but there are various short comings

*Discussion of the Mcm5 mutation and its general phenotype.* Although it seems that the *Mcm5* mutation used is not new to this study and its origin is appropriately cited by the authors, I find it very odd that there is not a clear paragraph early in this paper (introduction) that more generally describes this allele. What is the nature of this mutation (Null, missense, dominant/recessive?).

**Reply:** We agree, and we have described the nature of the mutation used (page 12)

What is the broader phenotype in the entire organism? (Lethality, developmental arrest at some informative point)? It is true that there is some discussion of *mcm5* mutations in the Discussion section, but due to the way the section is written, it is unclear to this reviewer if these comments are of a general nature or specifically refer to the allele used in this study.

**Reply:** We agree, and we have now described the broader phenotype in the entire organism (page 12)

*Add an interpretive panel to Fig. 1 to visually reinforce the first paragraph of the Results section.* Most readers of *PLOS Genetics* will likely find that your system is rather complicated. I would suggest that you consider inserting a new Figure 1A panel that provides guidance to your system and shows the relationship among the various developmental stages and micrographs that you discuss inn the text and show in the various figures. Once the reader is properly orientated, many of the presentation issues become much more manageable.

**Reply:** We agree with the reviewer and have inserted a cartoon describing the development of the Drosophila CNS and the Ap cluster neurons (Fig. 1A-C).

*Correlation problems between the figure panels and their citation in the text.* It would appear that during the development of this manuscript there were numerous changes to the Figures and Tables that were not propagated through the text of the manuscript.

**Reply:** We apologize for this and have amended these issues.

*Is this mcm5 defect related to the entire Mcm2-7 complex or is it unique to mcm5?* You try to address this question in Fig. 1, where you show that an *mcm7* mutant also causes a problem with development of the Tv4/FMRFa neuron. However, this issue is never revisited as more information on the *mcm5* defect is obtained. For example, is the *mcm7* defect also suppressed by over expression of Tkv?

**Reply:** We have tried to do the rescue proposed by the reviewer (*Mcm7, pros> UAS-tkvA, UAS-saxA*), but unfortunately, and for reasons that are unclear to us, the VNC of this particular rescue genotype was disorganized. Therefore, we cannot obtain conclusive evidence.

*Actual expression levels of Tkv in the control and mcm5 mutant.* A critical claim in this manuscript is that the *mcm5* allele specifically reduces transcription of the BMP receptor Tkv. However, the actual numbers are not show in the main manuscript but are instead buried in Sup Table 2. Given the importance of this obseration, it needs to be front and center in the paper. I would suggest that addition of either a graph or a table that shows the relative reduction in the TkV transcripts in the *mcm5* mutant, as well as the transcription levels of other genes in this developmental pathway (possibly the same genes that were assayed by immunofluorescence in Fig. 2?). For example, what is the expression level of the *dimm* gene (Fig. 2GH)?

**Reply:** We thank the reviewer for pointing this out, and have now added the data suggested by the reviewer as a fold change graph to Fig. 4B.

*Discussion claim.* “Our findings **agree with** studies showing that Mcm5 interacts with the RNA pol II complex or specific transcription factors and can play a role during gene expression (18, 19)”. As a gentle reminder, this study doesn’t examine interactions between mcm5 and the transcription machinery, and thus there is no data to agree or disagree with previous studies – thus you could just as well state that your data agrees with the notion that the moon is made of blue cheese (or whatever cheese you fancy). I would suggest that you slightly modify this sentence by swapping out “agree with” with “consistent with”.

**Reply:** We have removed that phrase from the text.

*Possibility of obtaining somewhat more mechanistic data.* This issue may extend well beyond the interests of your lab, but there may be several additional and possibly useful experiments that could be attempted. 1. Direct interactions between the Mcm2-7 and RNA polymerase – one might be able to address this with a proximity ligation assay (PLA). 2. Mcm defects commonly cause DNA damage through defective DNA replication. There are many available cytological assays to examine DNA damage. Do the Tv4/FMRFa neurons in the *mcm5* mutant have DNA damage? Can you determine if these cells are abnormally stuck in S-phase?

**Reply:** Regarding question 1, DNA damage, we have analyzed this by staining for cH2aX. Indeed, we observe increased DNA damage in the *Mcm5* mutant. However, we did not find evidence of DNA damage specifically in the Ap cluster cells. Moreover, we find that the cells in the NB5-6 lineage do not appear to be stuck in S phase and that the lineage progresses normally, evident by the expression of a number of markers for the Ap cluster neurons, the four last-born neurons in this lineage. Regarding question 2, the proposed experiment of proximity ligation between MCM2-7 complex members and RNA polymerase, this is a very interesting future avenue of studies. However, we are not able to probe this issue within the time constraints of the current manuscript.

**Details:**

*Figure 1.* The possible involvement of *mcm5* in neural differentiation came from a genetic screen conducted by the authors to identify mutations that failed to make a specific type of neuron. This figure is meant to show the relevant results of this screen.

**Reply:** Indeed, this is a point that the rest of the reviewers have also suggested, so we have added that table to the main text (Table 1).

Fig. 1 panels A-D shows the result of the *mcm5* mutant along with several important controls. In A-D, I believe that the take home point is whether or not the indicated cells fluoresce red (wildtype expression of relevant gene) or not (mutant phenotype). In the copy of the figures in my possession, if I squint and magnify the image, I can make out red staining of the SE2 cells in the control and pro-> Mcm5 subpanels. No other red is observable. In contrast, below the micrographs, are “interpretive” panels which are meant to instruct the reader in what they should be observing in the above micrographs. As nearly as I can tell, the micrographs and the interpretive panels are largely unrelated. The apparent lack of experimental robustness undermines the rest of the manuscript. Panels EF is meant to quantify the results show in panel A-D. However, the Y axis is rather cryptically labelled. In F, it is labelled as “SE2 Cells”. This makes some sense, as the values in all of the bar graphs have a height of “2” (same as the number of SE2 cells observed in A-D). However in E, the expression of the experiment cells (Tv4 cells) are shown. From panels A-D, the reader has been made to believe that there are 6 experimental cells in the micrographs, however the bar graph in E gives a value in excess of 6, with error bars extending to at least 8. Where do these extra cells come from? I don’t believe that this issue is addressed in either the text or legend.

**Reply:** Reviewing the comments of reviewer 3 we have discovered that we made an error in the graph: the control bar (Fig. 1E) was copied with the mutant bar. Reviewer 3 is right, the number of FMRFa neurons in the VNC is typically 6. We are grateful that the reviewer spotted this anomaly and we now provide an updated Figure 1. Regarding the immunostaining signal for the FMRFa cells it is important to underscore that because these images are rather zoomed out and show the entire VNC, containing some 10,000 cells, the signal is not as robust as that observed on a zoomed in image. However, we would still like to stick to showing these zoomed out images because they convey the amazing specificity in the FMRFa expression. Regarding the Y-axes in the graphs they represent the number of cells/VNC indicated on the label.

The last panels of Figure 1 (G-J) are further controls; however they are out of order in relation to the main text which presents part of Figure 2 before mentioning Fig. 1 G-J. I’m not sure about the rules at PLOS Genetics, but usually Figures need to be presented in the same order as the text. Panels G-H make some sense, as they are in the same format as panels A-D. Panels I-J are immediately confusing; I believe that they are supposed to represent Tv4 cells in Stage 18 AEL, but they look different that the rest of the cells in this figure. Moreover, for the control cells there appear to be 2 cells show/panel, while in the *mcm5* case only 1 cell is shown. Please explain this in the text. Moreover, both I and J are split into 4 unlabeled subpanels. By staring at these for a while, I believe that the top left penal is the combined image, while the other 3 panels correspond to each individual antibody, however, no information is provided. This comment also applies to Fig. 2

**Reply:** We have better adapted the order of the figures to their appearance in the text. In addition, following the instructions of the reviewer, we have expanded the figure captions to better guide the readers along.

*Figure 2.* These panels are immunofluorescence experiments to test the presence/absence of various diagnostic proteins in the test neurons in the control and *mcm5* strains. Given that they are all clumped, how do you identify the individual cells (e.g., which is TV1 and which is TV4)? In addition, in the text, it is claimed the presence of pMAD is different in the *mcm5* mutant relative to the control, an important observation that is the connection to the next figure. However to my eyes, I don’t see pMAD (green) expression in either wild type or control at least as general cellular staining. Again, if I squint, the control strain seems to have a cell with a few green flecks that I don’t see in the mcm5 mutant. Is this what I’m supposed to see? If so, please briefly explain this in the text.

**Reply:** We have added a new picture in which the immunostaining of pMad is better.

Figure 3. This has many of the same issues as Figure 1, except the data is more robust and one can actually see the immunofluorescence in most cases. However, the ordering of the experimental panels and the interpretive panel do not correspond (e.g., panel D is paired with panel C’)

**Reply:** We have carefully supervised the order of the figures and have adapted it to their appearance in the text.

Figure 4. Panel B has 2 parts, the data (coverage white and coverage mcm5) and various genetic maps meant to help interpret the data. What does coverage white mean? I would perhaps suppose that this is really meant to be coverage control, and it so happens that the control has a mutation in the white gene – you might want to reword this and perhaps explain in the figure legend. A problem with this panel is that the actual data is small and difficult to seen, while the genetic maps are large and probably more extensive than is necessary. Please flip the emphasis between the 2 sections of this Figure. Exons 1 and 2 are specifically mentioned in the text; please label them in this figure. Most of the explanation part of this panel (e.g., other genes and exons that are irrelevant to Tkv) are not mentioned in the text. Either they should be trimmed from the figure or discussed in the text. Moreover, as mentioned above, information should be added that actually show the relative expression level of Tkv in the *mcm5* mutant in relation to the other genes in the developmental pathway.

**Reply:** As described in Material and Methods, “White” stood for the genetic background of the control. We have changed to “control” to avoid confusion. We agree with the suggestion of the reviewer 3 and we have changed the emphasis on Figure 4 to highlight the read coverage of tkv gene. Also, as outlined above, we have added the information of the expression level in genes of the Tv4 specification pathway as a new panel Fig. 4B.
