# Supplementary material for: Selective role of the DNA helicase Mcm5 in BMP retrograde signaling during Drosophila neuronal differentiation
Source: PLoS Genet. 2022 Jun 23;18(6):e1010255. doi: 10.1371/journal.pgen.1010255 (PMC9258838; doi:10.1371/journal.pgen.1010255)
Supplement: Attachment — Submitted filename: Comment s to the Authors 2.docx [file pgen.1010255.s009.docx]

Madrid, May 6^th^, 2022

**PGENETICS-D-21-01485**

Dear Editors,

We are pleased to hereby re-re-submit our manuscript PGENETICS-D-21-01485 entitled "*Selective role of the DNA helicase Mcm5 in BMP retrograde signaling during Drosophila neuronal differentiation*".

After our revisions Reviewers #1 and #2 are now in favour of publishing our article.

Reviewer #3 outlines some remaining issues that needs addressing before publication, and we have conducted new experiments and amended the manuscript to address these issues. To clarify, the major changes to the manuscript are:

- Rescue of BMP signaling in *Mcm5* mutants with the transgenic expression of the BMP receptors in the Ap4 neuron

We address each reviewer’s comments below.

Sincerely yours,

Ignacio Monedero Cobeta, Ph.D.

Professor Assistant

Department of Physiology

Universidad Autónoma de Madrid

Madrid, SPAIN

Ph: +34-91-497 XXX

Email: [ignacio.monedero@uam.es](mailto:ignacio.monedero@uam.es)

**Reviewer #1**

Well done

Reply: Thank you, we are grateful for your constructive review.

**Reviewer #2**

It would be nice if the protein similarity of Mcm5 and Mcm7 genes between Drosophila and humans are explicitly described

Reply: We have added this information to the manuscript (page 12): “The Mcm5 and Mcm7 genes are highly conserved, and the proteins show 83% and 82% similarity between Drosophila and humans, respectively”. We are grateful for your helpful comments.

**Reviewer #3**

**Major issues**

Although the authors make a good attempt to repair the various problems raised following the first submission, the fundamental problems remain – this manuscript is built from descriptive and relatively preliminary data. In fairness, this manuscript presents two reasonably strong conclusions: 1) mcm5 mutants blocks BMP signaling in the Tv4 cells, and 2) ectopic expression of the BMP pathway receptor genes Tkv/Sax largely suppress the mcm5 Tv4 defect (BTW – does ectopic Tkv/Sax expression suppress the mcm5 lethality observed in the third larval stage?).

Although one might have hoped that the genomic experiment in Fig. 4 would provide a strong correlation between the mcm5 Tv4 phenotype and reduced gene expression of Tkv or Sax, this is not the case, as 1) the RNA-seq experiment utilizes whole embryos, where as the phenotype being examined is restricted to only a few cells (Tv4). Given that most of the RNA comes from cells that lack the mcm5 CNS phenotype, data interpretation will likely be clouded; 2) The reduction in Tkv transcript levels looks to be about 50% (Fig. 4B, ~ -0.5-fold reduction).

Although the trend is consistent with the possibility that reduced Tkv expression causes the mcm5 Tv4 phenotype, Fig. 4B shows that the expression of most of the genes involved in Tv4 cell maturation and BMP signaling (which based on cytology don’t appear to be seriously altered in the mcm5 mutant, Fig. 2), vary from the control levels to the same degree as Tkv. These results raise the possibility the relatively small reduction in Tkv transcripts just reflects the background scatter of the assay. Given these issues, the substance of this manuscript is rather thin.

Reply: As stated by Reviewer 3, one of the clear results of our study is that *Mcm5* mutants display loss of BMP signaling in the FMRFa/Ap4 neurons. Several lines of evidence support this notion. First, a number of previous studies have demonstrated that FMRFa expression in the Ap4 neuron is dependent on the BMP ligand Gbb, provided by the target organ, the dorsal neurohemal organ (DNH). Gbb triggers activation of the hetero-tetrameric Wit/Tkv/Sax receptor complex, and Tkv/Sax kinase activity then phosphorylates Mad. pMad is transported to the nucleus to directly activate the FMRFa gene, in a complex with the co-Smad, Medea. Second, our analysis of the expression of all known FMRFa determinants in *Mcm5* mutants only reveal loss of pMad. Third, the transgenic expression of the BMP receptors, but importantly, not of the ligand Gbb, in *Mcm5* mutants is capable of robustly rescuing FMRFa expression. Fourth, in new experiments we have now found that the transgenic expression of the BMP receptors also produces a robust rescue of pMad in the Ap4 neuron (new data in Fig S3, Page 8). Together, these findings clearly demonstrate that *Mcm5* mutants display BMP signaling defects in the FMRFa/Ap4 neurons.

We agree with the reviewer that given the scarcity of the Ap4 neurons within the entire embryo there are some shortcomings with bulk embryo RNA-seq experiments. Ideally, single cell RNA-seq analysis would have been better, although identifying enough Ap4 neurons from e.g., a scRNA-seq experiment of dissociated CNSs may prove challenging. Nevertheless, the bulk RNA seq analysis lends supports for the genetic analysis, demonstrating a BMP signaling defect in Ap4 neurons.

**Minor issues**

Line 139 – “lost or absent” – same thing? Perhaps you mean “absent or reduced?”

We agree and have amended the text accordingly.

Line 145 – “ Anti-cH2aX “. Don’t you mean Anti-gammaH2aX? At least in other organisms, this modification is referred to as gH2AX.

We apologize for this; it´s a typo error. The proper *Drosophila* notation is H2AvD phosphoS137 and we have been changed the text accordingly.

Line 153 – extra word “This revealed that it Mcm5 is expressed”

We agree and have amended the text accordingly.

Fig. 1A. I like the new interpretive panel! However, the legend for FMRFA and SVP are nearly the same color (Red Pink). Differentiate better – maybe make one blue?

Thank you very much for the positive comments about the new panel. Yes, we agree and have changed the color.

The initial screen (Table 1) claims that the mcm5 mutant causes loss of Tv4 cells, while in Fig 1I and J (and Figure 2) the Tv4 cells are still present in the mcm5 mutant, but they just do not produce FMRFa. This seems to be a discrepancy. Homozygous mcm5 mutant vs. hemizygous mcm5 mutant? Please clear up this issue.

It is a mislabel in Table 1. Table 1 refers to loss of FMRFa in Tv4 cells. To clarify: Tv4 cell is present in *Mcm5* mutant (and we can visualize it by Eya immunostaining (Fig S2 G). We have amended Table 1 accordingly.

Although not mentioned in the Results, the mcm5 mutants appear to increase the number of cells expressing Cas (Fig 2b). Is this significant?

It is not significant. Quantification of Cas positive Ap cluster cells has been added to Fig S3 C.

Except for data used in graphs Fig 1GH and 3F, it is not mentioned how many cells/embryos were cytologically examined for each treatment. How representative are the panels shown? Include graph/documentation in supplemental similar to Fig 3F for all treatments?

We have included the number of embryos examined in figure legends.

Fig 4A. X-axis – Log10 or Log2?

Log2, It has been added to the figure legend and the Graph.

Figure 4B. Dimmdac? I believe that s a space is needed. Also, as Materials and Methods

indicate that this experiment was done in triplicate, include error bars in this graph?

We have amended the space needed. Error bar should not be included in this type of graph since it does not represent the variance of the fold change control-*Mcm5* variation. The fold change is calculated comparing average of expression level of each gene of three control samples compare to average expression level of each gene of three *Mcm5* samples, therefore variance of the fold change could not be calculated in any case since samples are not paired.

Fig. 4C. I’m still having difficulty understanding the actual Tkv expression difference between the control and the mcm5 mutant. As nearly as I can tell from the diagram, the control cells make a significant level of transcripts relatively to the mcm5 mutant for a sequence on the left end of the graph (Under “Scale [0-325]”). However, the text states that expression of exons 1 and 2 (arrows right end of the graph) have a “striking reduction” in the mcm5 mutation. (So Tkv is transcribed from right to left?) At least as drawn, it doesn’t appear that either the control nor mcm5 mutant produce significant amount of either exon 1 or 2. Please clarify.

The *Drosophila* genome section is represented as convention 5´-3´ but *tkv* actually transcribes from the 3´-5´ template. Scale [0-325] adjusted for the maximum amount of reads in this region (left part of the panel), this resolution is automatically provided by TopHat2 (IGV) visualization manager. We have modified the figure to enlarge read coverage representation and make the differences clearer.

Line 470-471 : “…other genes located in the same chromosome region in green (its RNAm in yellow)” I have no idea what this means.

We thought this information could be relevant since *tkv* is not the only gene in the chromosome region depicted, nevertheless since could create confusion we have removed from the figure.
